# PacBio Hi-Fi genome assembly of the Iberian dolphin freshwater mussel *Unio delphinus* Spengler, 1793

**DOI:** 10.1038/s41597-023-02251-7

**Published:** 2023-06-01

**Authors:** André Gomes-dos-Santos, Manuel Lopes-Lima, M. André Machado, Amílcar Teixeira, L. Filipe C. Castro, Elsa Froufe

**Affiliations:** 1 CIIMAR/CIMAR — Interdisciplinary Centre of Marine and Environmental Research, University of Porto, Terminal de Cruzeiros do Porto de Leixões, Avenida General Norton de Matos, S/N, P, 4450-208 Matosinhos, Portugal; 2 Department of Biology, Faculty of Sciences, University of Porto, Rua do Campo Alegre 1021/1055, 4169-007 Porto, Portugal; 3 BIOPOLIS Program in Genomics, Biodiversity and Ecosystems, CIBIO, Centro de Investigação em Biodiversidade e Recursos Genéticos, InBIO Laboratório Associado, Campus de Vairão, Universidade do Porto, 4485-661 Vairão, Portugal; 4 IUCN SSC Mollusc Specialist Group, c/o IUCN, David Attenborough Building, Pembroke St, Cambridge, England; 5 Centro de Investigação de Montanha (CIMO), Instituto Politécnico de Bragança, Bragança, Portugal

**Keywords:** Genome, Conservation genomics

## Abstract

Mussels of order Unionida are a group of strictly freshwater bivalves with nearly 1,000 described species widely dispersed across world freshwater ecosystems. They are highly threatened showing the highest record of extinction events within faunal taxa. Conservation is particularly concerning in species occurring in the Mediterranean biodiversity hotspot that are exposed to multiple anthropogenic threats, possibly acting in synergy. That is the case of the dolphin freshwater mussel *Unio delphinus* Spengler, 1793, endemic to the western Iberian Peninsula with recently strong population declines. To date, only four genome assemblies are available for the order Unionida and only one European species. We present the first genome assembly of *Unio delphinus*. We used the PacBio HiFi to generate a highly contiguous genome assembly. The assembly is 2.5 Gb long, possessing 1254 contigs with a contig N50 length of 10 Mbp. This is the most contiguous freshwater mussel genome assembly to date and is an essential resource for investigating the species’ biology and evolutionary history that ultimately will help to support conservation strategies.

## Background & Summary

The application of genomics approaches to study non-model organisms is deemed a key approach to assess biodiversity and guide conservation^1–4^. Whole genome assemblies provide access to a species’ “entire genetic code”, thus representing the most comprehensive framework to efficiently decipher a species’ biology^5,6^. Genomic resources allow accurate definition of conservation units, identification of genetic elements with conservation relevance, inference of adaptive potential, assessment of population health, as well as provide predictive assessments of the impact of human-mediated threats and climate change^3,5,7,8^. Consequently, assembled genomes and other genomic tools are key resources to study and guide conservative actions and management planning.

**Fig. 1:**
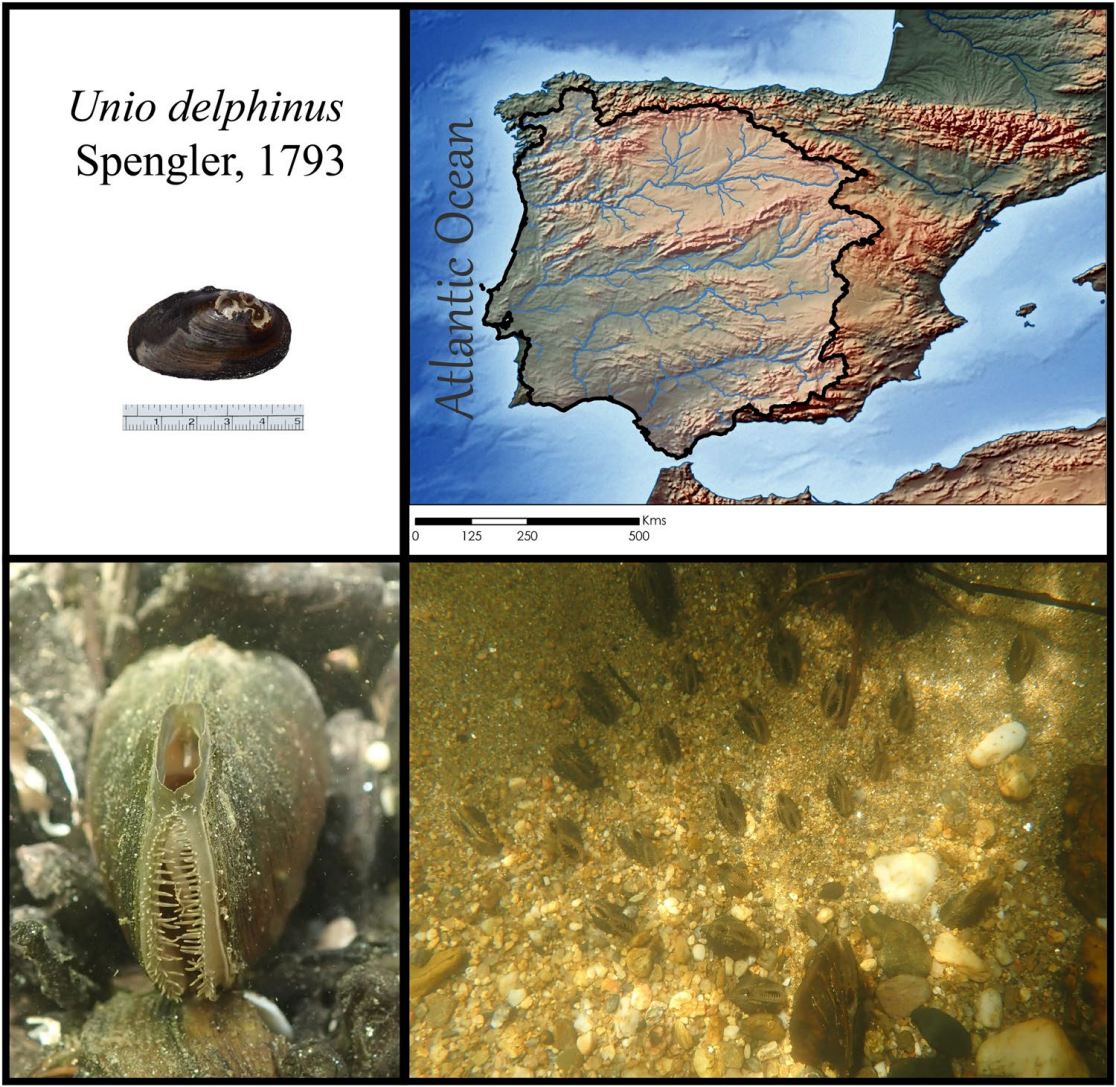
Top left: The *Unio delphinus* specimen used for the whole genome assembly. Top Right: The map of the potential distribution of *Unio delphinus* generated by overlapping points of recent presence records (obtained from^13^) with the Hydrobasins level 5 polygons^72^. Bottom Left: An *Unio delphinus* individual in its natural habitat. Bottom Right: A population of *Unio delphinus* in its natural habitat (Photos by Manuel Lopes-Lima).

Bivalves of the Order Unionida (known as freshwater mussels) are commonly found throughout most of the world’s freshwater ecosystems, where they play key ecological roles (e.g., nutrient and energy cycling and retention)^9–11^ and provide important services (e.g., water clearance, sediment mixing, pearls, and other raw materials)^9,10,12^. Despite their indisputable importance for freshwater ecosystems, freshwater mussels are among the most threatened taxa, with many populations worldwide having well-documented records of continuous declines over the last decades, as well as of many local and global extinctions^13–15^. Threatened species with limited distributions, such as the dolphin freshwater mussel *U. delphinus* Spengler, 1793 (Unionida: Unionidae) only found in the western Iberian Peninsula region (Fig. 1), represent particularly urgent but challenging targets for conservation^16^. The dolphin freshwater mussel, only recently recognised as a valid species^17^, has been strongly affected by a series of human-mediated actions over the last decades, including habitat destruction, dams or barrier construction, pollution, poor river management, water depletion, the introduction of invasive species, among others^16,18^. All these pressures are further augmented by the effects of climate change, especially the increasing interannual variability of water cycles over the last decades, which is particularly evident in the Mediterranean region^19,20^. As a consequence, the area of occurrence of the dolphin freshwater mussel has been reduced by almost one-third from its historical distribution^18^. This concerning trend has triggered an unprecedented effort to understand the threats and promote and implement conservation policies. These are critically dependent on the understanding the multiple aspects of the species’ biology, such as its life history, reproductive demands, ecological requirements, and its abiotic and biotic interactions^13,16,18,21^.

Recent efforts have focused on providing a thorough characterization of the species’ genetic diversity, population structure, and evolutionary history^21–23^. Despite the undeniable achievements of these early molecular studies, the availability of large-scale and more biologically informative genomic resources is almost inexistent, not only for *U. delphinus* but also for all freshwater mussels. In fact, for approximately 1000 known species, only four whole genome assemblies^24–27^ and less than 20 transcriptomes are currently available^28–41^. Recently, the first transcriptome assemblies of five threatened European freshwater mussel species have been published, including the gill transcriptome of the dolphin freshwater mussel^41^. This transcriptome was a fundamental tool to begin studying the evolutionary and adaptive traits of the species. However, single tissue RNA-seq approaches only capture a small fraction of the genetic information. Conversely, whole genome sequence assemblies represent a highly informative and fruitful resource to investigate and decipher multiple aspects of the species’ biology.

Here, we provide the first whole genome assembly of the dolphin freshwater mussel, *U. delphinus*. This is the most contiguous freshwater mussel genome assembly available, and the first Unionidae genome assembly from a European species. This genome provides a unique tool for an in-depth exploration of the many molecular mechanisms governing the biology of this species, which will ultimately guide conservation genomic studies to protect the critically declining population trend.

## Methods

### Animal sampling

One individual of *Unio delphinus* was collected in the Rabaçal River in Portugal (Table 1) and transported alive to the laboratory, where tissues were separated, flash-frozen, and stored at −80 °C. The whole shell and preserved tissues of the individual are deposited at CIIMAR tissue and mussels’ collection, under the voucher code: BIV7592.

**Table 1:** MixS descriptors for the *Unio delphinus* specimen used for whole genome sequencing.

Sample	*Unio delphinus* (BIV6631)
Investigation_type	Eukaryote
Lat_lon	41.564361; −7.258665
Geo_loc_name	Portugal
Collection_date	3/20/2021
Env_package	Water
Collector	Amilcar Teixeira
Sex	Undetermined
Maturity	Mature

### DNA extraction, library construction, and sequencing

For PacBio HiFi sequencing, mantle tissue was sent to Brigham Young University (BYU), where high-molecular-weight DNA extraction and PacBio HiFi library construction and sequencing were performed, following the manufacturer’s recommendations (https://www.pacb.com/wp-content/uploads/Procedure-Checklist-Preparing-HiFi-SMRTbell-Libraries-using-SMRTbell-Express-Template-Prep-Kit-2.0.pdf). Size-selection was conducted on the SageELF system. Sequencing was performed on four single-molecule, real-time (SMRT) cells using Sequel II system v.9.0, with a run time of 30 h, and 2.9 h pre-extension. The circular consensus analysis was performed in SMRT^®^ Link v9.0 (https://www.pacb.com/wp-content/uploads/SMRT_Link_Installation_v90.pdf) under default settings (Table 2).

**Table 2:** General statistics of raw sequencing reads used for the *Unio delphinus* genome assembly.

Sample	Sequencing type	Library type	Platform	Insert size (bp)	Number of reads	Application
PacBio HiFi Cell1	WGS	Long Reads	PacBio Sequel II System	12,044	391,801	Genome Assembly, Assessment
PacBio HiFi Cell2	WGS	Long Reads	PacBio Sequel II System	12,030	345,871	Genome Assembly, Assessment
PacBio HiFi Cell3	WGS	Long Reads	PacBio Sequel II System	12,164	381,954	Genome Assembly, Assessment
PacBio HiFi Cell4	WGS	Long Reads	PacBio Sequel II System	12,095	407,351	Genome Assembly, Assessment
Illumina PE	WGS	Short Reads	HiSeq X Ten	450	949,386,460	Genome size estimation

For short read Illumina sequencing, extracted genomic DNA was sent to Macrogen Inc. where a standard Illumina Truseq Nano DNA library preparation and whole genome sequencing of 150 bp paired-end (PE) reads was achieved using an Illumina HiSeq X machine (Table 2).

### Pre-assembly processing

Illumina PE short read quality was evaluated using FastQC (https://www.bioinformatics.babraham.ac.uk/projects/fastqc/) and after, reads were quality trimmed with Trimmomatic v.0.38^42^, specifying the parameters “LEADING: 5 TRAILING: 5 SLIDINGWINDOW: 5:20 MINLEN: 36”. The quality of the clean reads was re-validated in FastQC. The clean reads were used to estimate genome size, heterozygosity and repetitive content using Jellyfish v.2.2. and GenomeScope v2.0^43^ specifying a k-mer length of 25.

### Mitochondrial genome assembly

PacBio HiFi reads were used to retrieve a whole mitochondrial genome (mtDNA) assembly by applying a pipeline recently developed by our group^44^. Briefly, all Unionida mtDNA assemblies available on NCBI were retrieved (Fasta format; Supplementary_File1) and used as a reference mitogenome database. All the raw PacBio HiFi reads were mapped to the mitogenome database using Minimap2 v.2.17^45^, specifying parameters (-ax asm20). The output sam file was converted to bam and sorted using Samtools v.1.9^46^, with options “view” and “sort”, respectively. Samtools “view” was also used to retrieve only the mapped reads with parameter (-F 0 × 04) and after the bam file was converted to fastq format using the option “bam2fq”. The resulting PacBio HiFi mtDNA reads were corrected using Hifiasm v.0.13-r308^47,48^ with parameters (–write-ec). The corrected reads were assembled using Unicycler v.0.4.8^49^, a software package optimised for circular assemblies, with default parameters. Mitogenome annotation was produced using MitoZ v.3.4^50^ with parameters (--genetic_code 5--clade Mollusca), using the PE reads for coverage plotting.

### Genome assembly

**Fig. 2:**
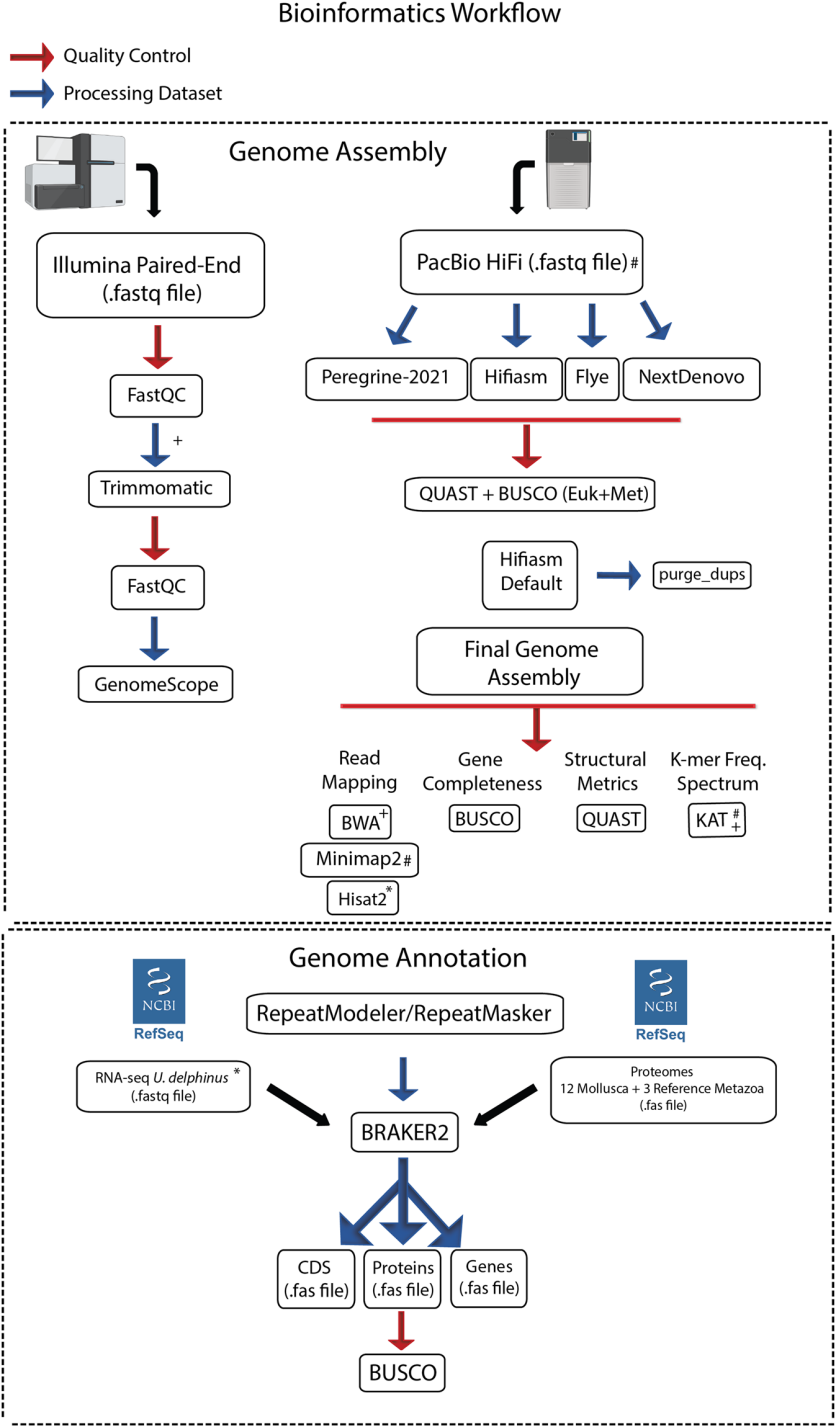
Bioinformatic pipeline applied for the whole genome assembly and annotation. Representative figures created with BioRender.com.

The overall pipeline used to obtain the genome assembly and annotation is provided in Fig. 2.

Firstly, PacBio HiFi reads were assembled using multiple software optimized for PacBio HiFi reads, i.e., Hifiasm 0.16.1-r375^47,48^ with default parameters, Flye v.2.8.3^51^ with parameters (–pacbio-hifi), NextDenovo v.2.4.0 (https://github.com/Nextomics/NextDenovo) with parameters (read_type = hifi) and Peregrine-2021 v0.4.3^52^ with default parameters. After, the overall quality of each assembly was assessed using Benchmarking Universal Single-Copy Orthologs (BUSCO) v.5.2.2^53^ with Eukaryota and Metazoa databases and Quality Assessment Tool for Genome Assemblies (QUAST) v.5.0.2^54^ (Fig. 2). Hifiasm 0.16.1-r375 produced the best results of the tested assemblies and thus was selected for further analyses. Since the genome size was larger than predicted by the GenomeScope report, several new assemblies were produced with this Hifiasm 0.16.1-r375, testing a range of parameters (*l* = 3; *s* = 0.50, 0.45, 0.35), following the authors’ recommendations (https://hifiasm.readthedocs.io/en/latest/faq.html#p-large). Given that reducing the similarity threshold for duplicate haplotigs (i.e., parameter -l and -s) had little impact on the overall statistic, the assembly with default parameters was chosen for further analysis. To separate poorly resolved pseudo-haplotypes, purge_dups v.1.2.5^55^ was applied, first with default parameters and after by manually adjusting the transition between haploid and diploid cut-off (i.e., parameter -m of option calcuts) to 30, 32 and 25 in three independent runs. In all the runs the lower and upper bound for read depth were always maintained, i.e., 5 and 87, respectively. All the cutoff values were determined by inspection of the *k*-mer plot produced by the K-mer Analysis Toolkit (KAT) tool^56^. The influence of purge_dups v.1.2.5 was evaluated using BUSCO v.5.2.2 with Eukaryota and Metazoa databases and QUAST v.5.0.2. Since purge_dups v.1.2.5 did not remove any duplicates (neither with the default nor adjusted cutoffs) the Hifiasm 0.16.1-r375 default assembly was selected as the final assembly. To evaluate the quality of the final assembly, several metrics and software were used. Besides BUSCO v.5.2.2 and QUAST v.5.0.2 metrics, completeness, heterozygosity, and collapsing of repetitive regions were evaluated using a k-mer distribution with KAT^56^. Moreover, read-back mapping was performed for the PE using with Burrows-Wheeler Aligner (BWA) v.0.7.17-r1198^57^, for long reads with Minimap2 v.2.17 and for RNA-seq (SRR19261764^41^) with Hisat2 v.2.2.0^58^. To inspect the genome for possible contamination, we used BlobTools v.1.1.1^59^. Briefly, a blast search of the final genome assembly was conducted against the RefSeq^60^ database, using the BLASTX function from DIAMOND v.2.0.11.149^61^, following authors’ instructions^59^. The blast output, as well as the alignment of PE short reads against the genome performed with BWA v.0.7.17, were used as input to run BlobTools, with contamination screening at Phylum level.

### Masking of repetitive elements, gene models predictions and annotation

Before masking repetitive elements, a *de novo* library of repeats was created for the final genome assembly, with RepeatModeler v.2.0.133^62^. Subsequently, the genome was soft masked combining the *de novo* library with the ‘Bivalvia’ libraries from Dfam_consensus-20170127 and RepBase-20181026, using RepeatMasker v.4.0.734^63^.

The masked assembly was used for gene prediction, performed using BRAKER2 pipeline v2.1.6^64^. First, RNA-seq data from *U. delphinus* was retrieved from GenBank (SRR19261764^41^) (the same individual used for the genome assembly), quality trimmed with Trimmomatic v.0.3839 (parameters described above) and aligned to the masked genome, using Hisat2 v.2.2.0 with the default parameters. Moreover, the complete proteome of 14 mollusc species and three reference Metazoa genomes (*Homo sapiens*, *Ciona intestinalis*, *Strongylocentrotus purpuratus*), were used as supplementary evidence for gene prediction, downloaded from public databases (Table 3). BRAKER2 pipeline was applied, specifying parameters “–etpmode; –softmasking;”. The resulting predictions file (braker.gtf) was filtered to retain only predictions with RNA-Seq and/or protein evidence (using auxiliary scripts selectSupportedSubsets.py) and after converted to.gff3 using the Augustus auxiliary script gtf2gff.pl. Gene predictions were processed using a series of auxiliary scripts from Another Gtf/Gff Analysis Toolkit (AGAT) v.0.8.063^65^. Briefly, gene predictions were clean with agat_convert_sp_gxf2gxf.pl, renamed with agat_sp_manage_functional_annotation.pl, overlapping prediction corrected with agat_sp_fix_overlaping_genes.pl and coding sequence regions (CDS) with <100 amino acid and incomplete gene predictions (i.e., without start and/or stop codons) were corrected and/or removed with agat_sp_add_start_and_stop.pl and agat_sp_filter_incomplete_gene_coding_models.pl, respectively. Finally, the overall statistics of the processed predictions were retrieved using agat_sp_statistics.pl and the predicted genes, protein, CDS and exon sequences were retrieved using agat_sp_extract_sequences.pl. The protein sequences were next used for functional annotation, using InterProScan v.5.44.80^66^, as well as BLASTP searches against the RefSeq database^60^. BLASTP homology searches were obtained using DIAMOND v.2.0.11.149^61^, specifying the parameters “-k 1, -b 20, -e 1e-5,–sensitive,–outfmt 6”. To validate the set of proteins obtained, the BUSCO scores were estimated based on the protein set, using the Eukaryota and Metazoa databases, as described previously.

**Table 3:** List of proteomes used for BRAKER2 gene prediction pipeline.

Phylum	Class	Order	Species	GenBank/RefSeq
**Mollusca**	**Bivalves**			
		**Ostreida**		
			*Crassostrea gigas*	GCF_902806645.1
			*Crassostrea virginica*	GCF_002022765.2
		**Pectinida**		
			*Mizuhopecten yessoensis*	GCF_000457365.1
			*Pecten maximus*	GCF_902652985.1
		**Veneroida**		
			*Dreissena polymorpha*	GCF_020536995.1
			*Mercenaria mercenaria*	GCF_014805675.1
		**Unionida**		
			*Margaritifera margaritifera*	GCA_015947965.1
			*Megalonaias nervosa*	GCA_016617855.1
	**Gastropod**			
			*Biomphalaria glabrata*	GCF_000457365.1
			*Pomacea canaliculata*	GCF_003073045.1
			*Gigantopelta aegis*	GCF_016097555.1
	**Cephalopod**			
			*Octopus bimaculoides*	GCF_001194135.1
			*Octopus sinensis*	GCF_006345805.1
	**Polyplacophora**			
			*Acanthopleura granulata*	GCA_016165875.1
**Chordata**			*Homo sapiens*	GCF_000001405.40
**Chordata**			*Ciona intestinalis*	GCF_000224145.3
**Echinodermata**			*Strongylocentrotus purpuratus*	GCF_000002235.4

## Data Records

The raw reads sequencing outputs were deposited at the NCBI Sequence Read Archive with the accession’s numbers: SRR23060683, SRR23060685, SRR23060678 and SRR23060675 for PacBio CCS HiFi; SRR23060686 for Illumina PE^67^. The Genome assembly is available under accession number JAQISU000000000^68^. BioSample accession number is SAMN32554582 and BioProject PRJNA917855^69^. The remaining information was uploaded to figshare. In detail, the files uploaded to figshare^70^ include the final unmasked and masked genome assemblies (Ude_BIV7592_haploid.fa and Ude_BIV7592_haploid_SM.fa), the two pseudohaplotypes genome assemblies generated by Hifiasm assembler (Ude_BIV7592_pseudohaplotype_1.fas.gz and Ude_BIV7592_pseudohaplotype_2.fas.gz), the annotation file (Ude_BIV7592_annotation_v4.gff3), predicted genes (Ude_BIV7592_genes_v4.fasta), predicted messenger RNA (Ude_BIV7592_mrna_v4.fasta), predicted open reading frames (Ude_BIV7592_cds_v4.fasta), predicted proteins (Ude_BIV7592_proteins_v4.fasta), as well as full table reports for Braker gene predictions and InterProScan functional annotations (Ude_BIV7592_annotation_v4_InterPro_report.txt) and RepeatMasker predictions (Ude_BIV7592_annotation_v4_RepeatMasker.tbl).

## Technical Validation

### Raw datasets and pre-assembly processing quality control

**Fig. 3:**
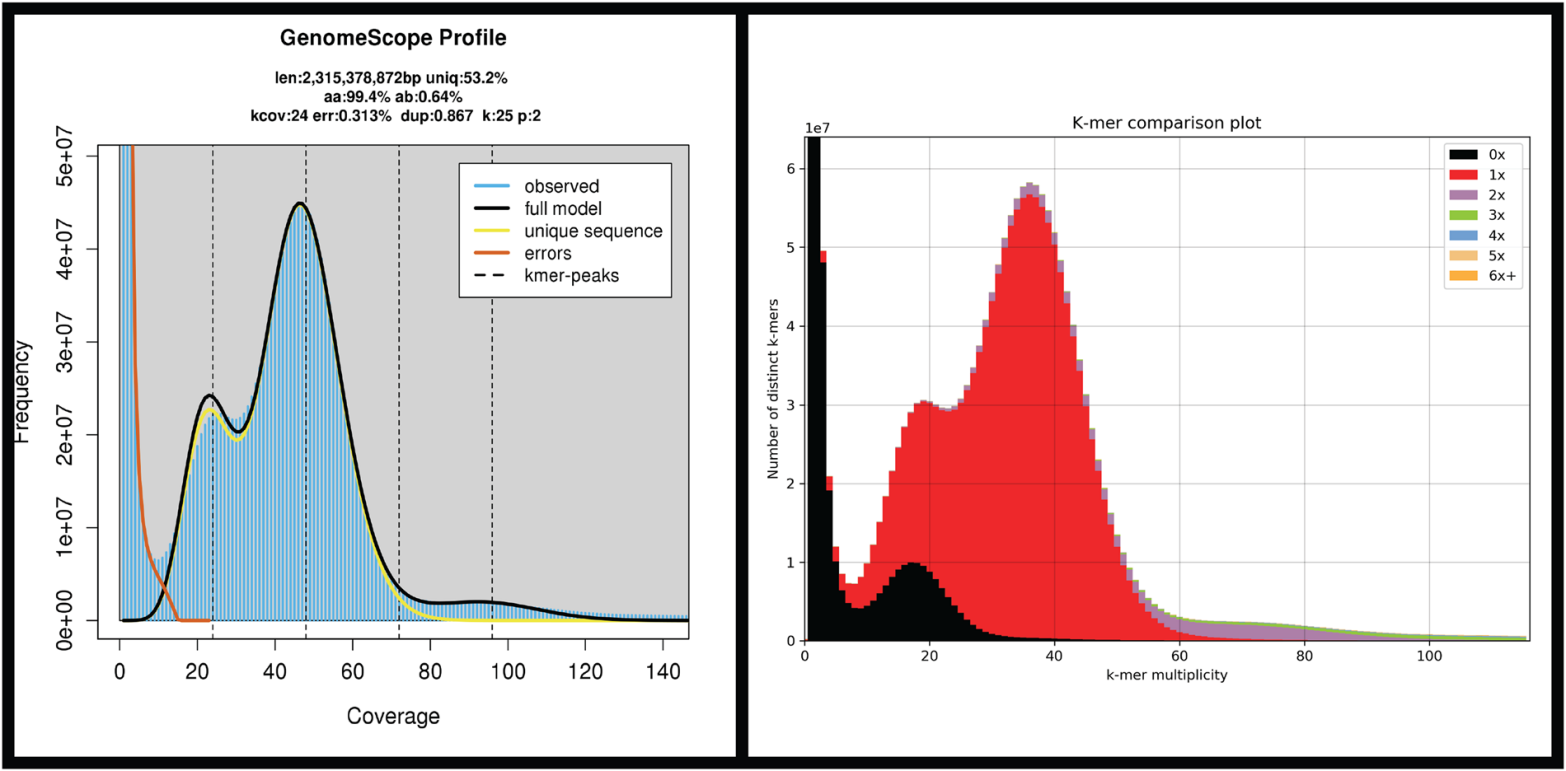
Left: GenomeScope2 k-mer (21) distribution displaying estimation of genome size (len), homozygosity (aa), heterozygosity (ab), mean *k*-mer coverage for heterozygous bases (kcov), read error rate (err), the average rate of read duplications (dup), *k*-mer size used on the run (k:), and ploidy (p:). Right: KAT spectra-cn plot for the *Unio delphinus* genome assembly, to compare the PacBio HiFi *k*-mer content within the genome assembly. Different colours represent the read *k*-mer frequency in the assembly.

Raw sequencing outputs general statistics are provided in Table 2. GenomeScope2 estimated genome size was ~2.31 Gb and heterozygosity levels of ~0.64% (Fig. 3a), both within the values observed for other Unionidae genomes available^24–27^.

### Genome assembly metrics

Hifiasm produced the overall most contiguous and complete (accessed under BUSCO scores) genome assembly of all the tested assemblers (Table 4). Both Flye and Peregrine-2021 were very inefficient in collapsing haplotypes, resulting in unexpectedly large assemblies with high levels of duplicated BUSCO scores (Table 4). Conversely, Hifiasm and NextDenovo efficiently resolve duplicates while ensuring high complete BUSCO scores (Table 4). Additionally, Hifiasm produced a much more contiguous genome assembly, with an almost 5-fold increased N50 length (Table 4). Although the BUSCO scores of the Hifiasm assembly had residual percentages of duplicated sequences, considering the increased genome size compared with GenomeScope estimation, as well as the genome sizes of other Unionidae assemblies (Table 5), we tested several similarity thresholds for duplicates in Hifiasm. The impact of the resulting assemblies on the overall statistics was limited, i.e., -s 0.50-0.35, or had no impact at all, i.e., -l 3 (Table 4). Although two of the assemblies, i.e., -s 0.50 and -s 0.45, show a slight increase in the N50 length (Table 4), given the overall little impact in the final genome size, we opted to use the Hifiasm default assembly as the final assembly. Moreover, purg-dups software did not remove any additional sequences from the Hifiasm default assembly, suggesting that reducing the similarity threshold for duplicate haplotigs (option -s) might be over-purging the assembly.

**Table 4:** *Unio delphinus* genome assemblies tests’ general statistics.

	Hifiasm default p_ctg	Flye	NextDenovo	peregrine-2021	Hifiasm -l 3 p_ctg	Hifiasm -s 0.50 p_ctg	Hifiasm -s 0.45 p_ctg	Hifiasm -s 0.35 p_ctg
Total number of Sequences (> = 1,000 bp)	1,254	33,629	3,428	5,075	1,254	1,244	1,232	1,215
Total number of Sequences (> = 10,000 bp)	1,247	27,176	3,428	5,075	1,247	1,237	1,225	1,209
Total number of Sequences (> = 25,000 bp)	968	15,387	3,301	5,068	968	958	952	936
Total number of Sequences (> = 50,000 bp)	612	9,104	2,887	4,628	612	603	606	589
Total length (> = 1,000 bp)	2,505,989,517	3,518,247,725	2,479,921,507	3,294,016,987	2,505,989,517	2,490,028,688	2,480,905,000	2,476,895,010
Total length (> = 10,000 bp)	2,505,937,610	2,845,972,272	2,479,921,507	3,29,4016,987	2,505,937,610	2,489,976,781	2,480,853,093	2,476,850,017
Total length (> = 25,000 bp)	2,500,313,574	2,651,784,830	2,477,471,122	3,293,869,030	2,500,313,574	2,484,364,781	2,475,348,593	2,471,361,534
Total length (> = 50,000 bp)	2,488,550,340	2,432,987,525	2,461,720,687	3,275,807,993	2,488,550,340	2,472,657,879	2,463,969,155	2,459,930,392
N50 length (bp)	10,919,244	356,382	2,550,545	1,830,736	10,919,244	11,289,431	11,289,431	10,919,244
L50	67	1,955	281	455	67	65	66	63
Largest contig (bp)	43,585,313	5,479,388	1,1041,057	21,870,125	43,585,313	43,585,313	34,144,451	44,270,880
GC content, %	35.07	34.90	35.04	35.01	35.07	35.07	35.07	35.07
Total BUSCO for the genome assembly (%)								
# Euk database	C:98.5% [S:96.1%, D:2.4%], F:1.6%	C:94.5% [S:89.8%, D:4.7%], F:5.5%	C:98.5% [S:96.5%, D:2.0%], F:1.6%	C:98.9% [S:71.8%, D:27.1%], F:1.2%	C:98.5% [S:96.1%, D:2.4%], F:1.6%	C:98.5% [S:96.1%, D:2.4%], F:1.6%	C:98.5% [S:96.1%, D:2.4%], F:1.6%	C:98.5% [S:96.1%, D:2.4%], F:1.6%
# Met database	C:96.5% [S:94.4%, D:2.1%], F:2.3%	C:93.0% [S:88.2%, D:4.8%], F:5.8%	C:96.3% [S:93.9%, D:2.4%], F:2.6%	C:96.5% [S:73.2%, D:23.3%], F:2.5%	C:96.5% [S:94.4%, D:2.1%], F:2.3%	C:96.6% [S:94.7%, D:1.9%], F:2.3%	C:98.5% [S:96.1%, D:2.4%], F:1.6%	C:96.6% [S:94.7%, D:1.9%], F:2.3%

#Euk: From a total of 303 genes of Eukaryota library profile.

#Euk: From a total of 255 genes of Eukaryota library profile.

#Met: From a total of 978 genes of Metazoa library profile.

#Met: From a total of 954 genes of Metazoa library profile.

#, +C: Complete; S: Single; D: Duplicated; F: Fragmented.

**Table 5:** General statistics of the *Unio delphinus* final genome assembly (p_ctg); *Unio delphinus* alternative haplotypes genome assemblies (hap1 and hap2); other published freshwater mussels genome assemblies.

		Hifiasm -l 3 p_ctg	Hifiasm -l 3 hap1	Hifiasm -l 3 hap2	*Megalonaias nervosa*	*Potamilus streckersoni*	*Margaritifera margaritifera*
Total number of Sequences (> = 1,000 bp)		1,254	3,752	3,000	90,895	2,366	105,185
Total number of Sequences (> = 10,000 bp)		1,247	3.743	2,993	54,764	2,162	15,384
Total number of Sequences (> = 25,000 bp)		968	2,774	2,668	29,042	1,831	11,583
Total number of Sequences (> = 50,000 bp)		612	1,938	2,029	12,699	1,641	9,265
Total length (> = 1,000 bp)		2,505,989,517	2,311,195,669	2,291,510,236	2,361,438,834	1,776,751,942	2,472,078,101
Total length (> = 10,000 bp)		2,505,937,610	2,311,130,750	2,291,456,057	2,193,448,794	1,775,453,721	2,293,496,118
Total length (> = 25,000 bp)		2,500,313,574	2,293,207,905	2,285,083,051	1,768,523,103	1,769,874,087	2,236,013,546
Total length (> = 50,000 bp)		2,488,550,340	2,264,885,011	2,262,774,153	1,194,323,847	1,763,052,140	2,152,307,394
N50 length (bp)		10,919,244	4,974,507	4,544,314	50,662	2,051,244	288,726
L50		67	125	121	12,463	245	2,393
Largest contig (bp)		43,585,313	27,621,201	28,529,984	588,638	10,787,299	2,510,869
GC content, %		35.07	35.07	35.04	35.82	33.79	35.42
Clean Paired-End (PE) Reads Alignment Stats							
Percentage of Mapped WGS PE (%)	—	99.81%	—	—	—	—	—
Percentage of Mapped WGS PacBio (%)		99.97%	—	—	—	—	—
Percentage of Mapped RNA-seq PE (%)		96.15%	—	—	—	—	—
Total BUSCO for the genome assembly (%)							
# Euk database	—	C:98.5% [S:96.1%, D:2.4%], F:1.6%	C:94.2% [S:91.8%, D:2.4%], F:3.5%	C:92.9% [S:90.2%, D:2.7%], F:3.1%	C:70.6% [S:70.2%, D:0.4%], F:14.9%	C:98.1% [S:97.3%, D:0.8%], F:0.8%	C: 86.8% [S: 85.8%, D:1.0%], F: 5.9%
# Met database	—	C:96.5% [S:94.4%, D:2.1%], F:2.3%	C:92.1% [S:90.5%, D:1.6%], F:3.5%	C:92.1% [S:90.4%, D:1.7%], F:3.5%	C:71.5% [S:70.1%, D:1.4%], F:14.5%	C:95.0% [S:93.6%, D:1.4%], F:2.3%	C: 84.9% (S: 83.8%, D: 1.1%), F: 4.9%
Masking Repetitive Regions and Gene Prediction							
Percentage masked bases (%)	—	52.83	—	—	25.00	51.03	59.07
Number of mRNA	—	44,382	—	—	49,149	41,065	40,544
Protein coding genes (CDS)	—	44,382	—	—	49,149	41,065	35,119
Functional annotated genes		32,089	—	—	—	—	31,584
Total gene length (bp)	—	869,540,056	—	—	—	—	902,994,752
Total BUSCO for the predicted proteins (%)							
+Euk database	—	C:96.8% [S:88.2%, D:8.6%], F:2.7%	—	—	—	—	C:90.6% [S:81.2%, D:9.4%], F:3.9%
+Met database	—	C:97.3% [S:86.0%, D:11.3%], F:2.3%	—	—	—	—	C:92.6% [S:82.3%, D:10.3%], F:3.2%

#Euk: From a total of 303 genes of Eukaryota library profile.

+Euk: From a total of 255 genes of Eukaryota library profile.

#Met: From a total of 978 genes of Metazoa library profile.

+Met: From a total of 954 genes of Metazoa library profile.

#, +C: Complete; S: Single; D: Duplicated; F: Fragmented.

The final genome assembly has a total length of ~2.5 Gbp, which is relatively larger than the GenomeScope size estimation, i.e., ~2.31 Gbp (Table 5, Fig. 3a). Although unexpected, the fact is that from all the primary assemblies here produced (from different software and Hifiasm parameters), none had a total length close to those estimated from GenomeScope (Tables 4–5). The alternative haplotypes assemblies produced by Hifiasm have a total length similar to the GenomeScope estimations, however, the complete BUSCO scores were reduced for these assemblies with no significate impact on duplicates (Table 5). On the other hand, purge-dups did not report any duplicated sequences in the assembly, which further support that Hifiasm efficiently resolved the haplotype variants. Moreover, the few genome assemblies available for freshwater mussels, show considerable distinct genome sizes (up to 696Mbp difference in size), even within the family Unionidae (Table 5). Consequently, the discrepancies between GenomeScope and the final genome size are likely a consequence of short read-based k-mer frequency spectrum analyses inaccurate estimation of the genome size.

The assembly here presented also shows, the most contiguous freshwater mussel genome assembly available to date, with a contig N50 length of ~ 10 Mbp, which represents a ~5-fold increase in N50 length regarding the only other PacBio-based genome assembly, i.e., from *P. streckersoni*^25^ (Table 5). The levels of completeness reported by BUSCOs scores are also within those observed for other freshwater mussel genome assemblies, with nearly no fragmented nor missing hits for both the eukaryotic and metazoan curated lists of near-universal single-copy orthologous (Table 5). The KAT k-mer analyses revealed a low level of k-mer duplication (blue, green, purple, and orange in Fig. 3b), with a high level of haplotype uniqueness (red in Fig. 3b) and a similar k-mer distribution to GenomeScope2 (performed with Illumina PE reads Fig. 3a,b). Both short-read, RNA-seq and long-read back-mapping percentages resulted in an almost complete mapping (Table 5). Finally, BlobTools Read Coverage Plots (ReadCovPlot) shows a dominance of hits with Mollusca (41.68%), followed by two groups with a similar hit percentage, i.e., undefined (27.41%) and Arthropoda (22.81%) (Fig. S1). The high values of undefined hits are expected given the overwhelming low number of closely related species with annotated genomes available on NCBI. Only 16 bivalves’ genomes have annotations available of NCBI, none of which belong to freshwater mussels or Palaeoheterodonta. In fact, annotations are only available for two higher-level bivalve clades, the vast majority for Pteriomorphia (n = 12) and the remaining for Imparidentia (n = 4). Moreover, this low and biased representation of annotated references most likely also explains the apparent contamination with Arthropoda (Fig. S1), as unspecific hits with unrelated taxa have been observed in other recent freshwater mussel genome assemblies^24^. Nevertheless, to deeply scrutinize for possible contaminations, the percentage of phyla representation was also quantified from the *U. delphinus* predicted proteins, using the RefSeq BLASTP search outputs (Fig. S2, Supplementary File 2). The results show the dominance of hits with Mollusca, with other taxa having residual representation and low percentages of identity, thus unlikely to represent contaminations (Fig. S2, Supplementary File 2).

Overall, these general statistics validate the high completeness, low redundancy, and quality of the final genome assembly.

### Repeat masking, gene models prediction, and annotation

RepeatModeler/RepeatMasker masked 52.83% of the genome, a value within those observed for other Unionida genome assemblies and close to the GenomeScope estimation (Table 6, Fig. 3a). Unlike the results observed in previous freshwater mussel’s genome assemblies^24,25^, most repeats are classified as DNA elements (21.92%, ~ 549 Mgp), rather than unclassified (16.32%, ~ 408 Mgp), with the remaining categories having similar percentages (Table 6). These results might be a consequence of PacBio HiFi reads efficiency in resolving repetitive regions thus facilitating their classification. BRAKER2 gene prediction identified 44,382 CDS, which is close to the predictions of the other freshwater mussel assemblies (Table 5). BUSCO scores for protein predictions showed almost no missing hits for either of the near-universal single-copy orthologous databases used (Table 5). The number of functionally annotated genes was 32,089, which is similar to the number of annotated genes for the *Margaritifera margaritifera* genome assembly (Table 5)^24^. Overall, the numbers of both predicted and annotated genes are within the expected range for bivalves (reviewed in^71^), as well as within the records of other freshwater mussel assemblies (Table 5)^24–27^.

**Table 6:** RepeatMasker report of the content of repetitive elements in the *Unio delphinus* genome assembly.

		Number of elements	Length occupies	Percentage of sequence
SINEs:		286,242	63,776,401 bp	2.54%
	ALUs	0	0 bp	0.00%
	MIRs	12,516	1,728,457 bp	0.07%
LINEs:		405,977	195,956,601 bp	7.82%
	LINE1	6,334	1,682,743 bp	0.07%
	LINE2	236,638	85,131,398 bp	3.40%
	L3/CR1	3,029	1,426,989 bp	0.06%
LTR elements:		166,328	108,444,169 bp	4.33%
	ERVL	5	1,053 bp	0.00%
	ERVL-MaLRs	0	0 bp	0.00%
	ERV_classI	22,367	5,217,054 bp	0.21%
	ERV_classII	965	98,757 bp	0.00%
DNA elements:		1,230,370	549,410,791 bp	21.92%
	hAT-Charlie	23,142	5,053,031 bp	0.20%
	TcMar-Tigger	34,031	12,862,816 bp	0.51%
Unclassified:		1,049,245	408,946,126 bp	16.32%
Total interspersed repeats:			1,326,534,088 bp	52.93%
Small RNA:		3,508	1,074,965 bp	0.04%
Satellites:		21,866	6,300,820 bp	0.25%
Simple repeats:		34,423	7,533,673 bp	0.30%
Low complexity:		180	36,435 bp	0.00%

## Supplementary information

Supplementary Information

## Data Availability

All software with respective versions and parameters used for producing the resources here presented (i.e., transcriptome assembly, pre and post-assembly processing stages, and transcriptome annotation) are listed in the methods section. Software programs with no parameters associated were used with the default settings.

## References

[1] Allendorf FW, Hohenlohe PA, Luikart G (2010). Genomics and the future of conservation genetics. Nature Reviews Genetics 2010 11:10.

[2] Formenti G (2022). The era of reference genomes in conservation genomics. Trends Ecol Evol.

[3] Hohenlohe PA, Funk WC, Rajora OP (2021). Population genomics for wildlife conservation and management. Mol Ecol.

[4] Meek MH, Larson WA (2019). The future is now: Amplicon sequencing and sequence capture usher in the conservation genomics era. Mol Ecol Resour.

[5] Paez S (2022). Reference genomes for conservation. Science (1979).

[6] Stephan, T. *et al*. Darwinian genomics and diversity in the tree of life. *Proc Natl Acad Sci USA* **119** (2022). 10.1073/pnas.2115644119 PMC8795533 35042807

[7] van Oppen MJH, Coleman MA (2022). Advancing the protection of marine life through genomics. PLoS Biol.

[8] Bertorelle G (2022). Genetic load: genomic estimates and applications in non-model animals. Nature Reviews Genetics 2022 23:8.

[9] Vaughn, C. C., Nichols, S. J. & Spooner, D. E. Community and foodweb ecology of freshwater mussels. **27**, 409–423, 10.1899/07-058.1 (2015).

[10] Vaughn CC (2017). Ecosystem services provided by freshwater mussels. Hydrobiologia 2017 810:1.

[11] Lopes-Lima, M. *et al*. Biology and conservation of freshwater bivalves: Past, present and future perspectives. *Hydrobiologia* **735**, 1–13, 10.1007/s10750-014-1902-9 (2014).

[12] Haag, W. R. North American Freshwater Mussels: Natural History, Ecology, and Conservation*.* (Cambridge University Press, 2012).

[13] Lopes-Lima M (2017). Conservation status of freshwater mussels in Europe: state of the art and future challenges. Biological Reviews.

[14] Cuttelod, A., Seddon, M. & Neubert, E. *European red list of non-marine molluscs*. (Publications Office of the European Union Luxembourg, 2011).

[15] Lopes-Lima M (2018). Conservation of freshwater bivalves at the global scale: diversity, threats and research needs. Hydrobiologia.

[16] Lopes-Lima M (2020). Setting the stage for new ecological indicator species: A holistic case study on the Iberian dolphin freshwater mussel Unio delphinus Spengler, 1793. Ecol Indic.

[17] Araujo, R. *et al*. Las náyades de la península Ibérica As náiades da Península Ibérica The naiads of the Iberian Peninsula. **27**, 7–72 (2009).

[18] Araujo R, Feo C, Pou Q, Campos M (2015). Conservation of two endangered European freshwater mussels (Bivalvia: Unionidae): a three-year, semi-natural breeding experiment. Nautilus (Philadelphia).

[19] Robson BJ, Chester ET, Mitchell BD, Matthews TG (2013). Disturbance and the role of refuges in mediterranean climate streams. Hydrobiologia.

[20] Cid N (2017). High Variability Is a Defining Component of Mediterranean-Climate Rivers and Their Biota. Water 2017, Vol. 9, Page 52.

[21] Froufe E (2016). Who lives where? Molecular and morphometric analyses clarify which Unio species (Unionida, Mollusca) inhabit the southwestern Palearctic. Org Divers Evol.

[22] Fonseca MM, Lopes-Lima M, Eackles MS, King TL, Froufe E (2016). The female and male mitochondrial genomes of Unio delphinus and the phylogeny of freshwater mussels (Bivalvia: Unionida). Mitochondrial DNA B Resour.

[23] Araujo R, Buckley D, Nagel KO, García-Jiménez R, Machordom A (2018). Species boundaries, geographic distribution and evolutionary history of the Western palaearctic freshwater mussels Unio (Bivalvia: Unionidae). Zool J Linn Soc.

[24] Gomes-dos-Santos, A. *et al*. The Crown Pearl: a draft genome assembly of the European freshwater pearl mussel *Margaritifera margaritifera* (Linnaeus, 1758). *DNA Research* **28** (2021). 10.1093/dnares/dsab002 PMC8088596 33755103

[25] Smith, C. H. A High-Quality Reference Genome for a Parasitic Bivalve with Doubly Uniparental Inheritance (Bivalvia: Unionida). *Genome Biol Evol* **13** (2021). 10.1093/gbe/evab029 PMC7937423 33570560

[26] Rogers RL (2021). Gene family amplification facilitates adaptation in freshwater unionid bivalve Megalonaias nervosa. Mol Ecol.

[27] Renaut S (2018). Genome Survey of the Freshwater Mussel Venustaconcha ellipsiformis (Bivalvia: Unionida) Using a Hybrid De Novo Assembly Approach. Genome Biol Evol.

[28] Roznere I, Sinn BT, Watters GT (2018). The Amblema plicata Transcriptome as a Resource to Assess Environmental Impacts on Freshwater Mussels. Freshwater Mollusk Biology and Conservation.

[29] Wang, R. *et al*. Rapid development of molecular resources for a freshwater mussel, *Villosa lienosa* (Bivalvia:Unionidae), using an RNA-seq-based approach. **31**, 695–708, 10.1899/11-149.1 (2015).

[30] Luo Y (2014). Transcriptomic Profiling of Differential Responses to Drought in Two Freshwater Mussel Species, the Giant Floater Pyganodon grandis and the Pondhorn Uniomerus tetralasmus. PLoS One.

[31] Patnaik BB (2016). Sequencing, De Novo Assembly, and Annotation of the Transcriptome of the Endangered Freshwater Pearl Bivalve, Cristaria plicata, Provides Novel Insights into Functional Genes and Marker Discovery. PLoS One.

[32] Wang X, Liu Z, Wu W (2017). Transcriptome analysis of the freshwater pearl mussel (Cristaria plicata) mantle unravels genes involved in the formation of shell and pearl. Molecular Genetics and Genomics.

[33] Yang Q (2021). Histopathology, antioxidant responses, transcriptome and gene expression analysis in triangle sail mussel Hyriopsis cumingii after bacterial infection. Dev Comp Immunol.

[34] Bertucci A (2017). Transcriptomic responses of the endangered freshwater mussel Margaritifera margaritifera to trace metal contamination in the Dronne River, France. Environmental Science and Pollution Research.

[35] Robertson LS, Galbraith HS, Iwanowicz D, Blakeslee CJ, Cornman RS (2017). RNA sequencing analysis of transcriptional change in the freshwater mussel Elliptio complanata after environmentally relevant sodium chloride exposure. Environ Toxicol Chem.

[36] Capt C (2018). Deciphering the Link between Doubly Uniparental Inheritance of mtDNA and Sex Determination in Bivalves: Clues from Comparative Transcriptomics. Genome Biol Evol.

[37] Huang D, Shen J, Li J, Bai Z (2019). Integrated transcriptome analysis of immunological responses in the pearl sac of the triangle sail mussel (Hyriopsis cumingii) after mantle implantation. Fish Shellfish Immunol.

[38] Capt C, Renaut S, Stewart DT, Johnson NA, Breton S (2019). Putative Mitochondrial Sex Determination in the Bivalvia: Insights From a Hybrid Transcriptome Assembly in Freshwater Mussels. Front Genet.

[39] Chen X, Bai Z, Li J (2019). The Mantle Exosome and MicroRNAs of Hyriopsis cumingii Involved in Nacre Color Formation. Marine Biotechnology.

[40] Cornman RS, Robertson LS, Galbraith H, Blakeslee C (2014). Transcriptomic Analysis of the Mussel Elliptio complanata Identifies Candidate Stress-Response Genes and an Abundance of Novel or Noncoding Transcripts. PLoS One.

[41] Gomes-dos-Santos A (2022). The gill transcriptome of threatened European freshwater mussels. Sci Data.

[42] Bolger AM, Lohse M, Usadel B (2014). Trimmomatic: a flexible trimmer for Illumina sequence data. Bioinformatics.

[43] Ranallo-Benavidez TR, Jaron KS, Schatz MC (2020). GenomeScope 2.0 and Smudgeplot for reference-free profiling of polyploid genomes. Nat Commun.

[44] Machado AM (2022). A genome assembly of the Atlantic chub mackerel (Scomber colias): a valuable teleost fishing resource. GigaByte.

[45] Li H (2018). Minimap2: pairwise alignment for nucleotide sequences. Bioinformatics.

[46] Danecek P (2021). Twelve years of SAMtools and BCFtools. Gigascience.

[47] Cheng H, Concepcion GT, Feng X, Zhang H, Li H (2021). Haplotype-resolved de novo assembly using phased assembly graphs with hifiasm. Nat Methods.

[48] Cheng H (2022). Haplotype-resolved assembly of diploid genomes without parental data. Nature Biotechnology 2022 40:9.

[49] Wick RR, Judd LM, Gorrie CL, Holt KE (2017). Unicycler: Resolving bacterial genome assemblies from short and long sequencing reads. PLoS Comput Biol.

[50] Meng G, Li Y, Yang C, Liu S (2019). MitoZ: a toolkit for animal mitochondrial genome assembly, annotation and visualization. Nucleic Acids Res.

[51] Kolmogorov M, Yuan J, Lin Y, Pevzner PA (2019). Assembly of long, error-prone reads using repeat graphs. Nature Biotechnology 2019 37:5.

[52] Chin, C.-S. & Khalak, A. Human Genome Assembly in 100 Minutes. *bioRxiv* 705616, 10.1101/705616 (2019).

[53] Manni M, Berkeley MR, Seppey M, Simão FA, Zdobnov EM (2021). BUSCO Update: Novel and Streamlined Workflows along with Broader and Deeper Phylogenetic Coverage for Scoring of Eukaryotic, Prokaryotic, and Viral Genomes. Mol Biol Evol.

[54] Gurevich A, Saveliev V, Vyahhi N, Tesler G (2013). QUAST: quality assessment tool for genome assemblies. Bioinformatics.

[55] Guan D (2020). Identifying and removing haplotypic duplication in primary genome assemblies. Bioinformatics.

[56] Mapleson D, Accinelli GG, Kettleborough G, Wright J, Clavijo BJ (2017). KAT: A K-mer analysis toolkit to quality control NGS datasets and genome assemblies. Bioinformatics.

[57] Li, H. Aligning sequence reads, clone sequences and assembly contigs with BWA-MEM. (2013).

[58] Kim D, Langmead B, Salzberg SL (2015). HISAT: A fast spliced aligner with low memory requirements. Nat Methods.

[59] Laetsch DR, Blaxter ML (2017). BlobTools: interrogation of genome assemblies. F1000Res.

[60] Pruitt KD, Tatusova T, Maglott DR (2007). NCBI reference sequences (RefSeq): a curated non-redundant sequence database of genomes, transcripts and proteins. Nucleic Acids Res.

[61] Buchfink B, Xie C, Huson DH (2015). Fast and sensitive protein alignment using DIAMOND. Nat Methods.

[62] Smit, A. & Hubley, R. RepeatModeler. www.repeatmasker.org (2015).

[63] Smit, A. & Hubley, R. RepeatMasker. www.repeatmasker.org (2015).

[64] Brůna T, Hoff KJ, Lomsadze A, Stanke M, Borodovsky M (2021). BRAKER2: automatic eukaryotic genome annotation with GeneMark-EP+ and AUGUSTUS supported by a protein database. NAR Genom Bioinform.

[65] Dainat J, Hereñú D, Pucholt P (2023). Zenodo.

[66] Quevillon E (2005). InterProScan: Protein domains identifier. Nucleic Acids Res.

[67] (2023). NCBI Sequence Read Archive.

[68] Gomes-dos-Santos A (2023). Genbank.

[69] *NCBI BioProject* https://identifiers.org/ncbi/bioproject:PRJNA917855 (2023).

[70] Gomes-dos-Santos A (2023). figshare.

[71] Gomes-dos-Santos A, Lopes-Lima M, C. Castro LF, Froufe E (2020). Molluscan genomics: the road so far and the way forward. Hydrobiologia.

[72] Lehner B, Grill G (2013). Global river hydrography and network routing: Baseline data and new approaches to study the world’s large river systems. Hydrol Process.

