# Supplementary material for: PacBio Hi-Fi genome assembly of the Iberian dolphin freshwater mussel Unio delphinus Spengler, 1793
Source: Sci Data. 2023 Jun 1;10:340. doi: 10.1038/s41597-023-02251-7 (PMC10235117; doi:10.1038/s41597-023-02251-7)
Supplement: Supplementary file 1 — Supplementary Information [file 41597_2023_2251_MOESM1_ESM.pdf]

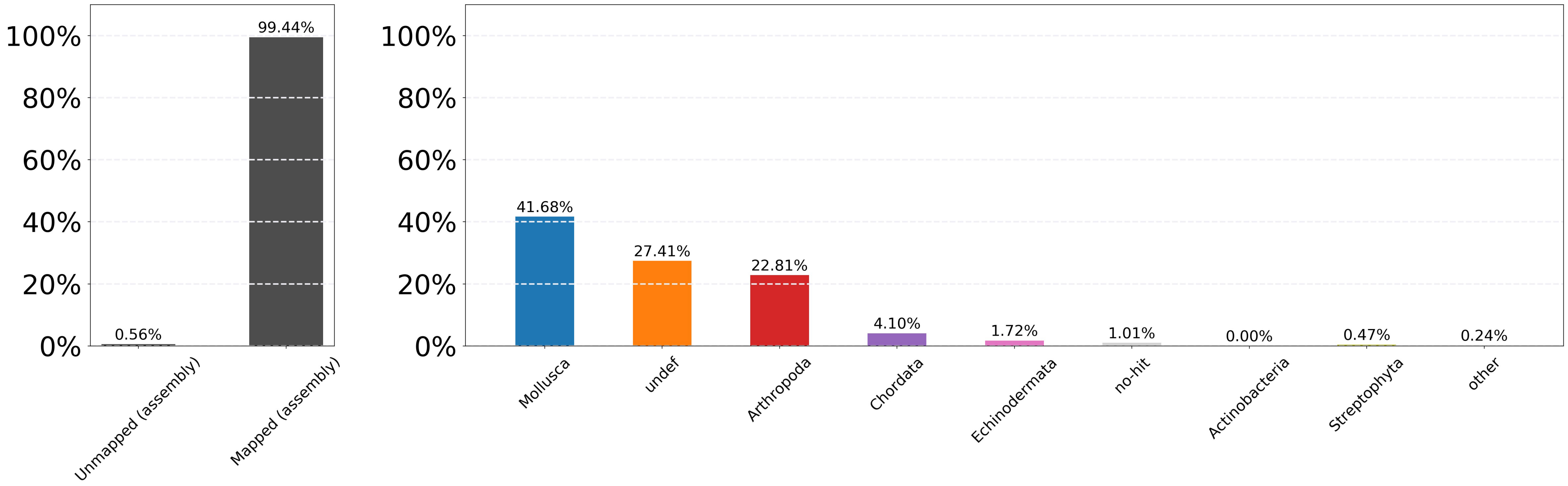

Fig.S1 - Blobtools Read Coverage Plots for the taxonomic level Phylum.

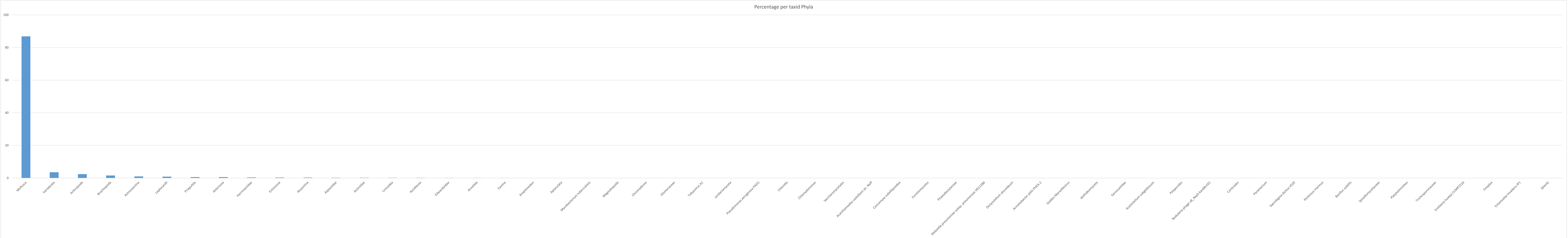

Fig. S2 - Percentage of higher taxonomic level blastp hits of the BRAKER2 predicted protein against RefSeq database.

Supplementary File 1 – List of Unionida whole mitochondrial genome assemblies used in the constructed mtDNA database.

1. Aculamprotula scripta voucher Xiaoping Wu:01-ASF-2017 mitochondrion, complete genome
15,688 bp circular DNA
MF991456.1 GI:1331389993

2. Aculamprotula tientsinensis mitochondrion, complete genome
15,695 bp circular DNA
KR873102.1 GI:954003649

3. Acuticosta chinensis mitochondrion, complete genome
15,653 bp circular DNA
MF687347.1 GI:1307564826

4. Acuticosta chinensis haplotype female mitochondrion, complete genome
15,652 bp circular DNA
MH919390.1 GI:1607271201

5. Alasmidonta heterodon mitochondrion, complete genome
15,909 bp circular DNA
MG905826.1 GI:1354701155

6. Alasmidonta varicosa mitochondrion, complete genome
15,693 bp circular DNA
MG938673.1 GI:1406947749

7. Alasmidonta varicosa voucher NEFC_B19-499 mitochondrion, complete genome
15,700 bp circular DNA
OM736872.1 GI:2325478630

8. Amblema plicata isolate BIV2448 mitochondrion, complete genome
15,946 bp circular DNA
MT648774.1 GI:1877028245

9. Anodonta euscaphys mitochondrion, complete genome
15,741 bp circular DNA
KP187851.1 GI:770593144

10. Anodonta anatina isolate 247 haplotype M mitochondrion, complete genome
16,906 bp circular DNA
KF030962.1 GI:528079477

11. Anodonta anatina isolate 320 haplotype M mitochondrion, complete genome
16,906 bp circular DNA
KF030963.1 GI:528079492

12. Anodonta anatina isolate 106 haplotype F mitochondrion, complete genome
15,653 bp circular DNA
KF030964.1 GI:528079507

13. Anodonta anatina isolate 218 haplotype F mitochondrion, complete genome

15,637 bp circular DNA
KF030965.1 GI:528079522

14. Anodonta anatina isolate 245 haplotype F mitochondrion, complete genome
15,640 bp circular DNA
KF030966.1 GI:528079537

15. Anodonta anatina isolate 146 haplotype F mitochondrion, complete genome
15,645 bp circular DNA
KF030967.1 GI:528079552

16. Anodonta anatina isolate 314 haplotype F mitochondrion, complete genome
15,645 bp circular DNA
KF030968.1 GI:528079567

17. Anodonta anatina isolate AA1 mitochondrion, complete genome
15,649 bp circular DNA
MN594531.1 GI:1958843900

18. Anodonta arcaeformis mitochondrion, complete genome
15,672 bp circular DNA
KF667530.1 GI:585866289

19. Anodonta cygnea mitochondrion, complete genome
15,613 bp circular DNA
MG385135.1 GI:1280962892

20. Anodonta cygnea mitochondrion, complete genome
15,610 bp circular DNA
MF781083.1 GI:1350896077

21. Anodonta cygnea isolate AC70 mitochondrion, complete genome
15,613 bp circular DNA
MN594532.1 GI:1958843914

22. UNVERIFIED: Anodonta cygnea mitochondrion sequence
15,905 bp linear DNA
MT874496.1 GI:2248385936

23. Anodonta exulcerata isolate AC108 mitochondrion, complete genome
15,619 bp circular DNA
MN594533.1 GI:1958843928

24. Anodonta lucida mitochondrion, complete genome
16,285 bp circular DNA
KF667529.1 GI:585866275

25. Anodonta nuttalliana isolate BIV0364 mitochondrion, complete genome
16,015 bp circular DNA
MN594534.1 GI:1958843942

26. UNVERIFIED: Anodontites elongata isolate AnoElo mitochondrion sequence, sequence
15,255 bp linear DNA
MK761136.1 GI:1803023760

27. Anodontites trapesialis isolate H2445 mitochondrion, partial genome
15,117 bp linear DNA
KU873119.1 GI:1150311665

28. Beringiana fukuharai B057 mitochondrial DNA, complete genome
16,014 bp circular DNA
LC592401.2 GI:2038456704

29. Beringiana fukuharai B103 mitochondrial DNA, complete genome
16,200 bp circular DNA
LC592402.2 GI:2038456718

30. Beringiana fukuharai B177 mitochondrial DNA, complete genome
16,092 bp circular DNA
LC592403.2 GI:2038456732

31. Beringiana fukuharai n972 mitochondrial DNA, complete genome
15,989 bp circular DNA
LC592408.2 GI:2038456760

32. Beringiana fukuharai n997 mitochondrial DNA, complete genome
16,158 bp circular DNA
LC592410.2 GI:2038456774

33. UNVERIFIED: Castalia ambigua isolate CasAmb mitochondrion sequence, sequence
17,092 bp linear DNA
MK761137.1 GI:1803023761

34. UNVERIFIED: Chambardia rubens isolate ChaRub mitochondrion sequence, sequence
16,697 bp linear DNA
MK761138.1 GI:1803023762

35. Chamberlainia hainesiana mitochondrion, complete genome
16,746 bp circular DNA
MK994770.1 GI:1695712150

36. Chamberlainia hainesiana mitochondrion, complete genome
17,465 bp circular DNA
MK994771.1 GI:1695712164

37. Contradens contradens isolate Concon_F voucher BIV1811 mitochondrion, complete genome
15,956 bp circular DNA
MW242812.1 GI:2007112088

38. Contradens contradens isolate Concon_M voucher BIV1811 mitochondrion, complete genome

17,057 bp circular DNA
MW242813.1 GI:2007112102

39. Lasmigona compressa isolate H2390 mitochondrion, complete genome
15,903 bp circular DNA
HM856638.1 GI:302702158

40. Cristaria plicata mitochondrion, complete genome
15,712 bp circular DNA
FJ986302.1 GI:229915382

41. Cristaria plicata mitochondrion, complete genome
15,708 bp circular DNA
GU944476.1 GI:291498580

42. Cristaria plicata mitochondrion, complete genome
15,710 bp circular DNA
KM233451.1 GI:677285913

43. Cumberlandia monodonta isolate H3010 mitochondrion, complete genome
16,099 bp circular DNA
KU873123.1 GI:1150311723

44. Cumberlandia monodonta isolate H1526g mitochondrion, complete genome
17,575 bp circular DNA
KU873124.1 GI:1150311738

45. Cuneopsis celtiformis voucher 20-NCU-XPWU-CC01 mitochondrion, complete genome
15,922 bp circular DNA
MW464617.1 GI:2028540050

46. Cuneopsis celtiformis voucher 21-NCU-XPWU-CCM01 mitochondrion, complete genome
16,875 bp circular DNA
MZ571520.1 GI:2296405140

47. Cuneopsis demangei voucher 21-NCU-XPWU-CDF01 mitochondrion, complete genome
15,975 bp circular DNA
MZ571513.1 GI:2296405080

48. Cuneopsis heudei mitochondrion, complete genome
15,982 bp circular DNA
MF687348.1 GI:1331389964

49. Cuneopsis heudei haplotype female mitochondrion, complete genome
15,892 bp circular DNA
MH919389.1 GI:1607271187

50. UNVERIFIED: Cuneopsis heudei mitochondrion sequence
16,744 bp linear DNA
MZ571514.1 GI:2296405155

51. Cuneopsis rufescens voucher 21-NCU-XPWU-CRF01 mitochondrion, complete genome
15,945 bp circular DNA
MZ571512.1 GI:2296405065

52. UNVERIFIED: Diplodon suavidicus isolate DipSua mitochondrion sequence, sequence
18,211 bp linear DNA
MK761139.1 GI:1803023763

53. Echyridella menziesii isolate SB11 mitochondrion, complete genome
16,031 bp circular DNA
KU873121.1 GI:1150311693

54. Echyridella menziesii isolate M9 mitochondrion, complete genome
18,140 bp circular DNA
KU873122.1 GI:1150311708

55. UNVERIFIED: Fossula fossiculifera isolate FosFos mitochondrion sequence, sequence
15,632 bp linear DNA
MK761140.1 GI:1803023764

56. Margaritifera margaritifera mitochondrion, complete genome
16,122 bp circular DNA
MK421956.1 GI:1678501092

57. Margaritifera margaritifera mitochondrion, complete genome
16,122 bp circular DNA
MK421957.1 GI:1678501106

58. Margaritifera margaritifera mitochondrion, complete genome
16,122 bp circular DNA
MK421958.1 GI:1678501120

59. Margaritifera margaritifera mitochondrion, complete genome
17,421 bp circular DNA
MK421959.1 GI:1678501134

60. Margaritifera margaritifera mitochondrion, complete genome
16,124 bp circular DNA
MW556443.1 GI:1989769663

61. Pyganodon grandis haplotype female mitochondrion, complete genome
15,848 bp circular DNA
FJ809754.1 GI:229915704

62. Pyganodon grandis haplotype male mitochondrion, complete genome
17,071 bp circular DNA
FJ809755.1 GI:229915718

63. Pyganodon grandis isolate NEFC_B19-228 mitochondrion, complete genome

15,851 bp circular DNA
OM736870.1 GI:2325478600

64. Gibbosula crassa mitochondrion, complete genome
16,196 bp circular DNA
MH319826.1 GI:1389449463

65. UNVERIFIED: Gibbosula polysticta mitochondrion sequence, sequence
15,714 bp linear DNA
MK728823.1 GI:1893870070

66. UNVERIFIED: Gibbosula rochechouartii mitochondrion sequence, sequence
16,100 bp linear DNA
KX378172.1 GI:1194689569

67. Hyriopsis bialata isolate HyrBia_F voucher BIV1774 mitochondrion, complete genome
15,919 bp circular DNA
MW242816.1 GI:2007112144

68. Hyriopsis bialata isolate HyrBia_M voucher BIV1774 mitochondrion, complete genome
16,761 bp circular DNA
MW242817.1 GI:2007112158

69. Hyriopsis cumingii mitochondrion, complete genome
15,954 bp circular DNA
FJ529186.1 GI:218135570

70. Hyriopsis cumingii mitochondrion, complete genome
15,957 bp circular DNA
HM347668.1 GI:298289276

71. UNVERIFIED: Hyriopsis cumingii mitochondrion sequence
17,100 bp linear DNA
KC150028.1 GI:453040376

72. Hyriopsis cumingii mitochondrion, complete genome
15,958 bp circular DNA
KM393224.1 GI:697994587

73. Hyriopsis cumingii UU-SBD-Unio11 mitochondrial DNA, complete genome
15,961 bp circular DNA
LC498621.1 GI:1817956062

74. Lamellidens marginalis voucher CIFEFGB-LM1 mitochondrion, complete genome
15,732 bp circular DNA
MT230549.1 GI:2026652142

75. UNVERIFIED: Lamproscapha ensiformis isolate LamEns mitochondrion sequence, sequence
15,583 bp linear DNA
MK761141.1 GI:1803023765

76. Lamprotula caveata mitochondrion, complete genome
16,641 bp circular DNA
KX060991.1 GI:1031877807

77. Lamprotula caveata haplotype male mitochondrion, complete genome
17,179 bp circular DNA
KX091842.1 GI:1083262520

78. Lamprotula coreana mitochondrion, complete genome
15,697 bp circular DNA
JX050180.1 GI:390472651

79. Lamprotula cornuumlunae voucher Personal:Xiaoping Wu:FM141126 mitochondrion, complete g
16,635 bp circular DNA
MK728822.1 GI:2034420496

80. Lamprotula gottschei mitochondrion, complete genome
15,915 bp circular DNA
KJ018924.1 GI:589913336

81. Lamprotula gottschei mitochondrion, complete genome
16,851 bp circular DNA
KJ627225.1 GI:647734893

82. Lamprotula leai haplotype F mitochondrion, complete genome
16,530 bp circular DNA
JQ691662.1 GI:379317181

83. Lamprotula leai mitochondrion, complete genome
16,852 bp circular DNA
KC847114.1 GI:511773436

84. Lamprotula scripta haplotype female mitochondrion, complete genome
16,250 bp circular DNA
KU745294.1 GI:1027254042

85. Lamprotula tortuosa mitochondrion, complete genome
15,722 bp circular DNA
KC109779.1 GI:441062040

86. Lamprotula tortuosa mitochondrion, complete genome
16,688 bp circular DNA
KC441487.1 GI:453041969

87. Lampsilis cardium isolate NEFC_B19-224 mitochondrion, complete genome
16,075 bp circular DNA
OM736867.1 GI:2325478555

88. Lampsilis cardium isolate NEFC_B19-225 mitochondrion, complete genome

16,057 bp circular DNA
OM736868.1 GI:2325478570

89. Lampsilis cariosa voucher NEFC_B19-074 mitochondrion, complete genome
15,981 bp circular DNA
OM736866.1 GI:2325478540

90. Lampsilis ornata mitochondrion, complete genome
16,060 bp circular DNA
AY365193.1 GI:39726228

91. Lampsilis powellii isolate H2610 mitochondrion, complete genome
16,043 bp circular DNA
MF326971.1 GI:1314670215

92. Lampsilis powellii isolate H2613 mitochondrion, complete genome
16,990 bp circular DNA
MF326972.1 GI:1314670230

93. Lampsilis siliquoidea isolate H2655 mitochondrion, complete genome
16,094 bp circular DNA
MF326973.1 GI:1314670245

94. Lampsilis siliquoidea isolate H3276 mitochondrion, complete genome
17,008 bp circular DNA
MF326974.1 GI:1314670260

95. Lampsilis siliquoidea isolate NEFC_B19-227 mitochondrion, complete genome
16,093 bp circular DNA
OM736869.1 GI:2325478585

96. Lampsilis siliquoidea isolate NEFC_B19-226 mitochondrion, complete genome
16,089 bp circular DNA
OM736873.1 GI:2325478645

97. Lanceolaria gladiola haplotype female mitochondrion, complete genome
15,732 bp circular DNA
KY067441.1 GI:1186236994

98. Lanceolaria grayana mitochondrion, complete genome
15,736 bp circular DNA
KJ495725.1 GI:630044994

99. Arconaia lanceolata mitochondrion, complete genome
15,782 bp circular DNA
KJ144818.1 GI:597568932

100. Arconaia lanceolata mitochondrion, complete genome
16,789 bp circular DNA
KJ775864.1 GI:669633923

101. Lasmigona complanata isolate NEFC_B19-229 mitochondrion, complete genome
16,006 bp circular DNA
OM736871.1 GI:2325478615

102. Lasmigona subviridis isolate H2958 mitochondrion, partial genome
11,969 bp linear DNA
HM856640.1 GI:302702188

103. Lepidodesma languilati mitochondrion, complete genome
15,754 bp circular DNA
KT381195.1 GI:992321108

104. Toxolasma parvus isolate H1805 mitochondrion, complete genome
15,949 bp circular DNA
HM856639.1 GI:302702173

105. Quadrula quadrula haplotype female mitochondrion, complete genome
16,033 bp circular DNA
FJ809750.1 GI:229915648

106. Quadrula quadrula haplotype male mitochondrion, complete genome
16,970 bp circular DNA
FJ809751.1 GI:229915662

107. Dahurinaia dahurica mitochondrion, complete genome
16,112 bp circular DNA
KF514426.1 GI:555298204

108. Microcondylaea bonellii mitochondrion, complete genome
16,020 bp circular DNA
MK994772.1 GI:1695712178

109. Microcondylaea bonellii mitochondrion, complete genome
16,737 bp circular DNA
MK994773.1 GI:1695712192

110. UNVERIFIED: Monocondylaea parchappii isolate MonPar mitochondrion sequence, sequence
15,528 bp linear DNA
MK761142.1 GI:1803023766

111. Monodontina vondembuschiana mitochondrion, complete genome
16,028 bp circular DNA
MK994774.1 GI:1695712206

112. UNVERIFIED: Monodontina vondembuschiana mitochondrion, sequence
16,364 bp linear DNA
MK994775.1 GI:1695712220

113. Mutela dubia isolate H1495 mitochondrion, complete genome

16,168 bp circular DNA
KU873120.1 GI:1150311679

114. Nodularia breviconcha mitochondrion, complete genome
15,741 bp circular DNA
MT955592.1 GI:1914212499

115. Unio douglasiae haplotype F mitochondrion, complete genome
15,767 bp circular DNA
KM657954.1 GI:731446523

116. Unio douglasiae mitochondrion, complete genome
16,670 bp circular DNA
KP970613.1 GI:893641264

117. Nodularia douglasiae mitochondrion, complete genome
15,761 bp circular DNA
MF314443.1 GI:1502979494

118. Nodularia douglasiae UU-SBD-Unio08 mitochondrial DNA, complete genome
15,779 bp circular DNA
LC496352.1 GI:1782080796

119. Nodularia douglasiae isolate MG mitochondrion, complete genome
15,764 bp circular DNA
MT764726.1 GI:1893869438

120. Nodularia douglasiae mitochondrion, complete genome
15,746 bp circular DNA
MT447062.1 GI:2173806703

121. Nodularia douglasiae biwae B063 mitochondrial DNA, complete genome
15,755 bp circular DNA
LC589171.1 GI:1959241293

122. Nodularia douglasiae biwae n975 mitochondrial DNA, complete genome
15,767 bp circular DNA
LC589172.1 GI:1959241307

123. Unio pictorum isolate 147 mitochondrion, complete genome
15,760 bp circular DNA
HM014130.1 GI:291575410

124. Unio pictorum isolate 109 mitochondrion, complete genome
15,762 bp circular DNA
HM014131.1 GI:291575424

125. Unio pictorum isolate 132 mitochondrion, complete genome
15,761 bp circular DNA
HM014132.1 GI:291575438

126. Unio pictorum isolate 149 mitochondrion, complete genome
15,761 bp circular DNA
HM014133.1 GI:291575452

127. Unio pictorum isolate 310 mitochondrion, complete genome
15,761 bp circular DNA
HM014134.1 GI:291575466

128. Unio pictorum haplogroup M mitochondrion, complete genome
16,632 bp circular DNA
MH349357.1 GI:1452923080

129. Unio pictorum haplogroup M mitochondrion, complete genome
16,632 bp circular DNA
MH349358.1 GI:1452923095

130. Utterbackia imbecillis isolate 28CD10-86 mitochondrion, complete genome
16,103 bp circular DNA
HM856637.1 GI:318088752

131. Parvasolenaia rivularis haplotype female mitochondrion, complete genome
16,613 bp circular DNA
KX966393.1 GI:1079707900

132. Parvasolenaia rivularis voucher Xiaoping Wu:01-SRM-2012 haplotype male mitochondrion, com
17,558 bp circular DNA
KY007142.1 GI:1095467474

133. Utterbackia peninsularis isolate 14GCP2-88 mitochondrion, complete genome
16,803 bp circular DNA
HM856635.1 GI:302702113

134. Utterbackia peninsularis isolate 18HRH6-88 mitochondrion, complete genome
15,593 bp circular DNA
HM856636.1 GI:302702128

135. Physunio superbus isolate PhySup_F voucher PhySup mitochondrion, complete genome
16,057 bp circular DNA
MW242814.1 GI:2007112116

136. Physunio superbus isolate PhySup_M voucher PhySup mitochondrion, complete genome
17,282 bp circular DNA
MW242815.1 GI:2007112130

137. Pilsbryoconcha exilis mitochondrion, complete genome
16,168 bp circular DNA
MK994776.1 GI:1695712232

138. Pilsbryoconcha exilis mitochondrion, complete genome

16,267 bp circular DNA
MK994777.1 GI:1695712246

139. Pleurobema oviforme isolate BIV1946 mitochondrion, complete genome
15,852 bp circular DNA
MT648775.1 GI:1877028259

140. Popenaias popeii isolate BIV2451 mitochondrion, complete genome
15,993 bp circular DNA
MT648776.1 GI:1877028273

141. Potamilus alatus haplotype male mitochondrion, complete genome
16,560 bp circular DNA
KU559010.1 GI:1020159465

142. Potamilus alatus haplotype female mitochondrion, complete genome
16,067 bp circular DNA
KU559011.1 GI:1020159479

143. Leptodea leptodon voucher LEPT20150810V3 mitochondrion, complete genome
16,133 bp circular DNA
KT723012.1 GI:948271079

144. Potamilus streckersoni isolate PohiBra071 voucher UF439535 mitochondrion, complete genom[illegible]
16,293 bp circular DNA
MW413895.1 GI:1958836348

145. Potamilus streckersoni isolate PohiBra085 voucher JBFMC10001.6 mitochondrion, complete ge[illegible]
16,152 bp circular DNA
ON855351.1 GI:2277708024

146. Potamilus streckersoni isolate PohiBra085 voucher JBFMC10001.6 mitochondrion, complete ge[illegible]
16,482 bp circular DNA
ON881148.1 GI:2281572456

147. Potomida littoralis isolate PL702F mitochondrion, complete genome
15,789 bp circular DNA
KT247374.1 GI:959093583

148. Potomida littoralis isolate PL702M mitochondrion, complete genome
16,451 bp circular DNA
KT247375.1 GI:959093597

149. UNVERIFIED: Prisodon obliquus isolate PriObl mitochondrion sequence, sequence
16,691 bp linear DNA
MK761143.1 GI:1803023767

150. Unio japanensis mitochondrial DNA, COIII to CO1 region, haplotype:male type
16,967 bp linear DNA
AB055624.1 GI:15128581

151. Unio japanensis mitochondrial DNA, ND5 to tRNA-Gln region, haplotype:female type
16,826 bp linear DNA
AB055625.1 GI:15128594

152. Pronodularia japanensis UU-SBD-Unio-01 mitochondrial DNA, complete genome
16,803 bp circular DNA
LC505454.1 GI:1817956097

153. Pseudanodonta complanata isolate BIV0186 mitochondrion, complete genome
16,171 bp circular DNA
MN594535.1 GI:1958843956

154. Pseudocuneopsis capitata haplotype female mitochondrion, complete genome
15,845 bp circular DNA
MH919387.1 GI:1607271159

155. Pseudocuneopsis capitata voucher 21-NCU-XPWU-PCM01 mitochondrion, complete genome
16,607 bp circular DNA
MZ571517.1 GI:2296405095

156. Pseudocuneopsis sichuanensis voucher 21-NCU-XPWU-PSF01 mitochondrion, complete genome
15,839 bp circular DNA
MZ571510.1 GI:2296405035

157. Pseudocuneposis sichuanensis voucher 21-NCU-XPWU-PSM01 mitochondrion, complete genom
16,649 bp circular DNA
MZ571518.1 GI:2296405110

158. UNVERIFIED: Pseudunio auricularius isolate PseAur_F mitochondrion sequence, sequence
16,195 bp linear DNA
MK761144.1 GI:1803023768

159. Margaritifera marocana isolate BIV1914F mitochondrion, complete genome
16,001 bp circular DNA
KY131953.1 GI:1178772179

160. Margaritifera marocana isolate BIV1914M mitochondrion, complete genome
17,562 bp circular DNA
KY131954.1 GI:1178772396

161. Ptychorhynchus pfisteri haplotype female mitochondrion, complete genome
16,040 bp circular DNA
KY067440.1 GI:1148297383

162. Rectidens sumatrensis isolate RecSum_F voucher BIV1664 mitochondrion, complete genome
15,983 bp circular DNA
MW242818.1 GI:2007112172

163. Rectidens sumatrensis isolate RecSum_M voucher BIV1665 mitochondrion, complete genome

16,934 bp circular DNA
MW242819.1 GI:2007112186

164. Schistodesmus lampreyanus haplotype female mitochondrion, complete genome
15,855 bp circular DNA
MH919388.1 GI:1607271173

165. UNVERIFIED: Schistodesmus lampreyanus mitochondrion sequence
16,796 bp linear DNA
MZ571515.1 GI:2296405156

166. Schistodesmus spinosus voucher 21-NCU-XPWU-SSF01 mitochondrion, complete genome
15,964 bp circular DNA
MZ571511.1 GI:2296405050

167. UNVERIFIED: Schistodesmus spinosus mitochondrion sequence
16,680 bp linear DNA
MZ571516.1 GI:2296405157

168. Sinanodonta tumens B194 mitochondrial DNA, complete genome
15,807 bp circular DNA
LC592406.2 GI:2038456746

169. Anodonta woodiana isolate AW19 mitochondrion, complete genome
16,242 bp circular DNA
HQ283344.1 GI:310777585

170. Anodonta woodiana isolate AW22 mitochondrion, complete genome
16,243 bp circular DNA
HQ283345.1 GI:310777600

171. Anodonta woodiana isolate AW124 mitochondrion, complete genome
16,242 bp circular DNA
HQ283346.1 GI:310777615

172. Anodonta woodiana isolate AW123 mitochondrion, complete genome
16,243 bp circular DNA
HQ283347.1 GI:310777630

173. Anodonta woodiana isolate AW125 mitochondrion, complete genome
16,243 bp circular DNA
HQ283348.1 GI:310777645

174. Sinanodonta woodiana voucher 2014-phc-si-wo-001 mitochondrion, complete genome
16,256 bp circular DNA
KM272949.1 GI:685165083

175. Sinanodonta woodiana mitochondrion, complete genome
17,738 bp circular DNA
KM434235.1 GI:704002361

176. Sinanodonta woodiana haplogroup M mitochondrion, complete genome
17,615 bp circular DNA
MH349356.1 GI:1452923064

177. Sinanodonta woodiana haplogroup M mitochondrion, complete genome
17,616 bp circular DNA
MH349359.1 GI:1452923110

178. Sinanodonta woodiana isolate SW032 mitochondrion, complete genome
16,243 bp circular DNA
MN594536.1 GI:1958843970

179. Hyriopsis schlegelii sex female mitochondrion, complete genome
15,939 bp circular DNA
HQ641406.1 GI:316891618

180. Hyriopsis schlegelii sex male mitochondrion, complete genome
15,961 bp circular DNA
HQ641407.1 GI:316891632

181. Sinohyriopsis schlegelii mitochondrial DNA, complete genome, isolate: B225
15,951 bp circular DNA
AP018550.1 GI:1605112100

182. Sinohyriopsis schlegelii mitochondrial DNA, complete genome, isolate: B227
15,951 bp circular DNA
AP018551.1 GI:1605112114

183. Sinohyriopsis schlegelii UU-SBD-Unio10 mitochondrial DNA, complete genome
15,954 bp circular DNA
LC498622.1 GI:1817956076

184. Solenaia carinata voucher Xiaoping WU:01-SCF-2012 mitochondrion, complete genome
16,716 bp circular DNA
KC848654.1 GI:512393067

185. Solenaia carinata voucher Xiaoping WU:01-SCM-2012 mitochondrion, complete genome
17,102 bp circular DNA
KC848655.1 GI:512393081

186. Solenaia oleivora haplotype female mitochondrion, complete genome
16,392 bp circular DNA
KF296320.1 GI:544584149

187. Solenaia oleivora voucher Xiaoping Wu:01-SOM-2012 haplotype male mitochondrion, complete
16,765 bp circular DNA
KY007143.1 GI:1095467488

188. Solenaia oleivora mitochondrion, complete genome

16,392 bp circular DNA
MT477834.1 GI:1929135775

189. Cuneopsis pisciculus mitochondrion, complete genome
15,907 bp circular DNA
KP273584.1 GI:748995636

190. Tchangsinaia piscicula voucher 21-NCU-XPWU-APM01 mitochondrion, complete genome
16,652 bp circular DNA
MZ571519.1 GI:2296405125

191. Unio crassus isolate 182 haplogroup F mitochondrion, complete genome
15,781 bp circular DNA
KY290446.1 GI:1143271496

192. Unio crassus isolate 183 haplogroup F mitochondrion, complete genome
15,781 bp circular DNA
KY290447.1 GI:1143271511

193. Unio crassus isolate DR14 haplogroup M mitochondrion, complete genome
16,633 bp circular DNA
KY290448.1 GI:1143271526

194. Unio crassus isolate DR15 haplogroup M mitochondrion, complete genome
16,633 bp circular DNA
KY290449.1 GI:1143271541

195. Unio crassus isolate 176 haplogroup M mitochondrion, complete genome
16,646 bp circular DNA
KY290450.1 GI:1143271556

196. Unio delphinus mitochondrion, complete genome
15,762 bp circular DNA
KT326917.1 GI:1016561854

197. Unio delphinus mitochondrion, complete genome
16,620 bp circular DNA
KT326918.1 GI:1016561869

198. Unio elongatulus isolate UI146F mitochondrion, complete genome
15,763 bp circular DNA
MN594537.1 GI:1958843984

199. Unio mancus isolate UM34F mitochondrion, complete genome
15,756 bp circular DNA
MN594538.1 GI:1958843998

200. Unio tumidus isolate 157 haplogroup M mitochondrion, complete genome
16,607 bp circular DNA
KY021073.1 GI:1143271571

201. Unio tumidus isolate 159 haplogroup M mitochondrion, complete genome
16,607 bp circular DNA
KY021074.1 GI:1143271586

202. Unio tumidus isolate 203 haplogroup M mitochondrion, complete genome
16,607 bp circular DNA
KY021075.1 GI:1143271601

203. Unio tumidus isolate 207 haplogroup F mitochondrion, complete genome
15,769 bp circular DNA
KY021076.1 GI:1143271616

204. Unio tumidus isolate 155 haplogroup F mitochondrion, complete genome
15,770 bp circular DNA
KY021077.1 GI:1143271631

205. Unio tumidus isolate 158 haplogroup F mitochondrion, complete genome
15,769 bp circular DNA
KY021078.1 GI:1143271646

206. Venustaconcha ellipsiformis haplotype male mitochondrion, complete genome
17,174 bp circular DNA
FJ809752.1 GI:229915676

207. Venustaconcha ellipsiformis haplotype female mitochondrion, complete genome
15,975 bp circular DNA
FJ809753.1 GI:229915690

208. Margaritifera falcata isolate H2912 mitochondrion, complete genome
16,121 bp circular DNA
HM856634.1 GI:302702098

209. UNVERIFIED: Westralunio carteri isolate WesCar_F mitochondrion sequence, sequence
16,766 bp linear DNA
MK761145.1 GI:1803023769

210. UNVERIFIED: Westralunio carteri isolate WesCar_M mitochondrion sequence, sequence
17,942 bp linear DNA
MK761146.1 GI:1803023770

ıplete genome

Supplementary File 2 – Results of BLASTP search of the BRAKER2 predicted protein against RefSeq database.
The results are in tabular format obtaining using DIAMOND with parameters:
"-k 1 -b 20 -e 1e-5 --sensitive
--outfmt 6 qseqid staxids bitscore qseqid sseqid pident length mismatch gapopen qstart qend sstart send evalue bitscore".
Additionally, taxonomic classification was added to the last column of the table, using the taxids list, with TaxonKit v.0.8.0.

UdeM00000044471 225164 409.5 UdeM00000044471 XP_009052905.1 84 231 37 0 18 248 121 351 3.10E-111 409.5 225164 cellular organisms;Eukaryota;Opisthokonta;Metazoa;Eumetazoa;Bilateria;Protostomia;Spiralia;Lophotrochozoa;Mollusca;Gastropoda;Patellogastropoda;Lottioidea;Lottiidae;Lottia;Lottia gigantea
UdeM00000033513 29159 316.2 UdeM00000033513 XP_034317111.1 55.8 283 124 1 2 283 171 453 4.20E-83 316.2 29159 cellular organisms;Eukaryota;Opisthokonta;Metazoa;Eumetazoa;Bilateria;Protostomia;Spiralia;Lophotrochozoa;Mollusca;Bivalvia;Autobranchia;Pteriomorphia;Ostreida;Ostreoidea;Ostreidae;Crassostrea;Crassostrea gigas
UdeM00000033514 6596 301.6 UdeM00000033514 XP_045161140.1 64.3 221 78 1 8 227 10 230 9.90E-79 301.6 6596 cellular organisms;Eukaryota;Opisthokonta;Metazoa;Eumetazoa;Bilateria;Protostomia;Spiralia;Lophotrochozoa;Mollusca;Bivalvia;Autobranchia;Heteroconchia;Euheterodonta;Imparidentia;Venerida;Veneroidea;Veneridae;Mercenaria;Mercenaria mercenaria
UdeM00000033515 6573 110.5 UdeM00000033515 XP_021372591.1 65 80 24 1 39 118 30 105 1.50E-21 110.5 6573 cellular organisms;Eukaryota;Opisthokonta;Metazoa;Eumetazoa;Bilateria;Protostomia;Spiralia;Lophotrochozoa;Mollusca;Bivalvia;Autobranchia;Pteriomorphia;Pectinida;Pectinoidea;Pectinidae;Mizuhopecten;Mizuhopecten yessoensis
UdeM00000033516 6596 206.1 UdeM00000033516 XP_045161140.1 65.8 152 51 1 12 163 87 237 3.50E-50 206.1 6596 cellular organisms;Eukaryota;Opisthokonta;Metazoa;Eumetazoa;Bilateria;Protostomia;Spiralia;Lophotrochozoa;Mollusca;Bivalvia;Autobranchia;Heteroconchia;Euheterodonta;Imparidentia;Venerida;Veneroidea;Veneridae;Mercenaria;Mercenaria mercenaria
UdeM00000033517 6596 110.2 UdeM00000033517 XP_045161140.1 69 71 22 0 52 122 53 123 2.00E-21 110.2 6596 cellular organisms;Eukaryota;Opisthokonta;Metazoa;Eumetazoa;Bilateria;Protostomia;Spiralia;Lophotrochozoa;Mollusca;Bivalvia;Autobranchia;Heteroconchia;Euheterodonta;Imparidentia;Venerida;Veneroidea;Veneridae;Mercenaria;Mercenaria mercenaria
UdeM00000033518 6579 184.5 UdeM00000033518 XP_033763365.1 33.2 346 214 8 1 342 797 1129 2.40E-43 184.5 6579 cellular organisms;Eukaryota;Opisthokonta;Metazoa;Eumetazoa;Bilateria;Protostomia;Spiralia;Lophotrochozoa;Mollusca;Bivalvia;Autobranchia;Pteriomorphia;Pectinida;Pectinoidea;Pectinidae;Pecten;Pecten maximus
UdeM00000033519 6596 76.6 UdeM00000033519 XP_045162803.1 46.4 97 41 2 22 108 392 487 2.10E-11 76.6 6596 cellular organisms;Eukaryota;Opisthokonta;Metazoa;Eumetazoa;Bilateria;Protostomia;Spiralia;Lophotrochozoa;Mollusca;Bivalvia;Autobranchia;Heteroconchia;Euheterodonta;Imparidentia;Venerida;Veneroidea;Veneridae;Mercenaria;Mercenaria mercenaria
UdeM00000033521 6573 1373.2 UdeM00000033521 XP_021368707.1 43.2 1662 918 12 1 1650 1 1648 0.00E+00 1373.2 6573 cellular organisms;Eukaryota;Opisthokonta;Metazoa;Eumetazoa;Bilateria;Protostomia;Spiralia;Lophotrochozoa;Mollusca;Bivalvia;Autobranchia;Pteriomorphia;Pectinida;Pectinoidea;Pectinidae;Mizuhopecten;Mizuhopecten yessoensis
UdeM00000033522 6596 124.4 UdeM00000033522 XP_045190982.1 40.8 142 74 3 18 152 32 170 1.20E-25 124.4 6596 cellular organisms;Eukaryota;Opisthokonta;Metazoa;Eumetazoa;Bilateria;Protostomia;

Spiralia;Lophotrochozoa;Mollusca;Bivalvia;Autobranchia;Heteroconchia;Euheterodonta;Imparidentia;Venerida;Veneroidea;Veneridae;Mercenaria;Mercenaria mercenaria
UdeM00000033523 6596 139 UdeM00000033523 XP_045206881.1 42.7 164 82 5 1 159 1 157 5.10E-30 139 6596 cellular organisms;Eukaryota;Opisthokonta;Metazoa;Eumetazoa;Bilateria;Protostomia;Spiralia;Lophotrochozoa;Mollusca;Bivalvia;Autobranchia;Heteroconchia;Euheterodonta;Imparidentia;Venerida;Veneroidea;Veneridae;Mercenaria;Mercenaria mercenaria
UdeM00000033524 6573 1373.2 UdeM00000033524 XP_021368707.1 42.6 1672 925 12 1 1661 1 1648 0.00E+00 1373.2 6573 cellular organisms;Eukaryota;Opisthokonta;Metazoa;Eumetazoa;Bilateria;Protostomia;Spiralia;Lophotrochozoa;Mollusca;Bivalvia;Autobranchia;Pteriomorphia;Pectinida;Pectinoidea;Pectinidae;Mizuhopecten;Mizuhopecten yessoensis
UdeM00000033525 6573 1380.5 UdeM00000033525 XP_021368707.1 42.9 1661 925 11 1 1650 1 1648 0.00E+00 1380.5 6573 cellular organisms;Eukaryota;Opisthokonta;Metazoa;Eumetazoa;Bilateria;Protostomia;Spiralia;Lophotrochozoa;Mollusca;Bivalvia;Autobranchia;Pteriomorphia;Pectinida;Pectinoidea;Pectinidae;Mizuhopecten;Mizuhopecten yessoensis
UdeM00000033526 6565 247.7 UdeM00000033526 XP_022346198.1 53.4 223 96 2 79 293 43 265 1.90E-62 247.7 6565 cellular organisms;Eukaryota;Opisthokonta;Metazoa;Eumetazoa;Bilateria;Protostomia;Spiralia;Lophotrochozoa;Mollusca;Bivalvia;Autobranchia;Pteriomorphia;Ostreida;Ostreoidea;Ostreidae;Crassostrea;Crassostrea virginica
UdeM00000033527 6565 228 UdeM00000033527 XP_022346195.1 54.4 206 85 3 1 198 62 266 1.00E-56 228 6565 cellular organisms;Eukaryota;Opisthokonta;Metazoa;Eumetazoa;Bilateria;Protostomia;Spiralia;Lophotrochozoa;Mollusca;Bivalvia;Autobranchia;Pteriomorphia;Ostreida;Ostreoidea;Ostreidae;Crassostrea;Crassostrea virginica
UdeM00000033528 6565 225.7 UdeM00000033528 XP_022346195.1 57.8 185 71 2 6 184 83 266 4.80E-56 225.7 6565 cellular organisms;Eukaryota;Opisthokonta;Metazoa;Eumetazoa;Bilateria;Protostomia;Spiralia;Lophotrochozoa;Mollusca;Bivalvia;Autobranchia;Pteriomorphia;Ostreida;Ostreoidea;Ostreidae;Crassostrea;Crassostrea virginica
UdeM00000033529 6596 133.7 UdeM00000033529 XP_045207460.1 42.5 146 78 3 40 183 45 186 5.70E-28 133.7 6596 cellular organisms;Eukaryota;Opisthokonta;Metazoa;Eumetazoa;Bilateria;Protostomia;Spiralia;Lophotrochozoa;Mollusca;Bivalvia;Autobranchia;Heteroconchia;Euheterodonta;Imparidentia;Venerida;Veneroidea;Veneridae;Mercenaria;Mercenaria mercenaria
UdeM00000033530 1735272 299.3 UdeM00000033530 XP_041379055.1 62.4 221 83 0 24 244 35 255 4.50E-78 299.3 1735272 cellular organisms;Eukaryota;Opisthokonta;Metazoa;Eumetazoa;Bilateria;Protostomia;Spiralia;Lophotrochozoa;Mollusca;Gastropoda;Neomphalina;Neomphaloidea;Peltospiridae;Gigantopelta;Gigantopelta aegis
UdeM00000033531 400727 290.4 UdeM00000033531 XP_025092513.1 63.1 214 79 0 1 214 29 242 1.80E-75 290.4 400727 cellular organisms;Eukaryota;Opisthokonta;Metazoa;Eumetazoa;Bilateria;Protostomia;Spiralia;Lophotrochozoa;Mollusca;Gastropoda;Caenogastropoda;Architaenioglossa;Ampullarioidea;Ampullariidae;Pomacea;Pomacea canaliculata

UdeM00000033532	6596	143.3	UdeM00000033532	XP_045207460.1	40.1	172
	93	5	27	191	18	186	5.70E-31	143.3	6596	cellular
organisms;Eukaryota;Opisthokonta;Metazoa;Eumetazoa;Bilateria;Protostomia;
Spiralia;Lophotrochozoa;Mollusca;Bivalvia;Autobranchia;Heteroconchia;Euhe
terodonta;Imparidentia;Venerida;Veneroidea;Veneridae;Mercenaria;Mercenari
a mercenaria
UdeM00000033533	6596	143.3	UdeM00000033533	XP_045207460.1	40.1	172
	93	5	27	191	18	186	5.20E-31	143.3	6596	cellular
organisms;Eukaryota;Opisthokonta;Metazoa;Eumetazoa;Bilateria;Protostomia;
Spiralia;Lophotrochozoa;Mollusca;Bivalvia;Autobranchia;Heteroconchia;Euhe
terodonta;Imparidentia;Venerida;Veneroidea;Veneridae;Mercenaria;Mercenari
a mercenaria
UdeM00000033536	6596	387.1	UdeM00000033536	XP_045177776.1	33.1	818
	473	21	300	1072	402	1190	7.20E-104	387.1	6596	cellular
organisms;Eukaryota;Opisthokonta;Metazoa;Eumetazoa;Bilateria;Protostomia;
Spiralia;Lophotrochozoa;Mollusca;Bivalvia;Autobranchia;Heteroconchia;Euhe
terodonta;Imparidentia;Venerida;Veneroidea;Veneridae;Mercenaria;Mercenari
a mercenaria
UdeM00000033538	33528	87.4	UdeM00000033538	XP_043978578.1	28.5	214
	122	5	2	197	30	230	2.90E-14	87.4	33528	cellular
organisms;Eukaryota;Opisthokonta;Metazoa;Eumetazoa;Bilateria;Deuterostomi
a;Chordata;Craniata;Vertebrata;Gnathostomata;Teleostomi;Euteleostomi;Acti
nopterygii;Actinopteri;Neopterygii;Teleostei;Osteoglossocephalai;Clupeoce
phala;Euteleosteomorpha;Neoteleostei;Eurypterygia;Ctenosquamata;Acanthomo
rphata;Euacanthomorphacea;Percomorphaceae;Ovalentaria;Atherinomorphae;Cyp
rinodontiformes;Cyprinodontoidei;Poeciliidae;Poeciliinae;Gambusia;Gambusi
a affinis
UdeM00000033537	13397	88.6	UdeM00000033537	XP_041062505.1	26.9	305
	161	9	3	270	26	305	1.30E-14	88.6	13397	cellular
organisms;Eukaryota;Opisthokonta;Metazoa;Eumetazoa;Bilateria;Deuterostomi
a;Chordata;Craniata;Vertebrata;Gnathostomata;Chondrichthyes;Elasmobranchi
i;Selachii;Galeomorphii;Galeoidea;Lamniformes;Alopiidae;Lamninae;Carcharo
don;Carcharodon carcharias
UdeM00000033539	8245	59.7	UdeM00000033539	XP_040007666.1	34.4	90
	47	2	3	92	101	178	4.70E-06	59.7	8245	cellular
organisms;Eukaryota;Opisthokonta;Metazoa;Eumetazoa;Bilateria;Deuterostomi
a;Chordata;Craniata;Vertebrata;Gnathostomata;Teleostomi;Euteleostomi;Acti
nopterygii;Actinopteri;Neopterygii;Teleostei;Osteoglossocephalai;Clupeoce
phala;Euteleosteomorpha;Neoteleostei;Eurypterygia;Ctenosquamata;Acanthomo
rphata;Euacanthomorphacea;Percomorphaceae;Carangaria;Istiophoriformes;Xip
hiidae;Xiphias;Xiphias gladius
UdeM00000033540	941984	108.6	UdeM00000033540	XP_040922238.1	37.4
	163	88	3	4	165	34	183	1.20E-20	108.6	941984
	cellular
organisms;Eukaryota;Opisthokonta;Metazoa;Eumetazoa;Bilateria;Deuterostomi
a;Chordata;Craniata;Vertebrata;Gnathostomata;Teleostomi;Euteleostomi;Acti
nopterygii;Actinopteri;Neopterygii;Teleostei;Osteoglossocephalai;Clupeoce
phala;Euteleosteomorpha;Neoteleostei;Eurypterygia;Ctenosquamata;Acanthomo
rphata;Euacanthomorphacea;Percomorphaceae;Carangaria;Carangaria incertae
sedis;Toxotidae;Toxotes;Toxotes jaculatrix
UdeM00000033541	433405	81.6	UdeM00000033541	XP_031701220.1	28.5
	214	122	5	2	196	30	231	1.60E-12	81.6	433405
	cellular
organisms;Eukaryota;Opisthokonta;Metazoa;Eumetazoa;Bilateria;Deuterostomi

a;Chordata;Craniata;Vertebrata;Gnathostomata;Teleostomi;Euteleostomi;Actinopterygii;Actinopteri;Neopterygii;Teleostei;Osteoglossocephalai;Clupeocephala;Euteleosteomorpha;Neoteleostei;Eurypterygia;Ctenosquamata;Acanthomorphata;Euacanthomorphacea;Percomorphaceae;Eupercaria;Perciformes;Cottioidei;Zoarcales;Anarhichadidae;Anarrhichthys;Anarrhichthys ocellatus
UdeM00000033542 13397 80.5 UdeM00000033542 XP_041062505.1 26.8 302 164 9 3 269 26 305 3.60E-12 80.5 13397 cellular organisms;Eukaryota;Opisthokonta;Metazoa;Eumetazoa;Bilateria;Deuterostomia;Chordata;Craniata;Vertebrata;Gnathostomata;Chondrichthyes;Elasmobranchii;Selachii;Galeomorphii;Galeoidea;Lamniformes;Alopiidae;Lamninae;Carcharodon;Carcharodon carcharias
UdeM00000033543 6596 161.8 UdeM00000033543 XP_045163870.1 46.5 155 81 2 30 182 21 175 8.50E-37 161.8 6596 cellular organisms;Eukaryota;Opisthokonta;Metazoa;Eumetazoa;Bilateria;Protostomia;Spiralia;Lophotrochozoa;Mollusca;Bivalvia;Autobranchia;Heteroconchia;Euheterodonta;Imparidentia;Venerida;Veneroidea;Veneridae;Mercenaria;Mercenaria mercenaria
UdeM00000033544 29159 110.2 UdeM00000033544 XP_011456334.2 43.4 136 70 1 6 141 19 147 4.20E-21 110.2 29159 cellular organisms;Eukaryota;Opisthokonta;Metazoa;Eumetazoa;Bilateria;Protostomia;Spiralia;Lophotrochozoa;Mollusca;Bivalvia;Autobranchia;Pteriomorphia;Ostreida;Ostreoidea;Ostreidae;Crassostrea;Crassostrea gigas
UdeM00000033548 33528 89.4 UdeM00000033548 XP_043978588.1 29.1 213 121 4 2 197 30 229 7.60E-15 89.4 33528 cellular organisms;Eukaryota;Opisthokonta;Metazoa;Eumetazoa;Bilateria;Deuterostomia;Chordata;Craniata;Vertebrata;Gnathostomata;Teleostomi;Euteleostomi;Actinopterygii;Actinopteri;Neopterygii;Teleostei;Osteoglossocephalai;Clupeocephala;Euteleosteomorpha;Neoteleostei;Eurypterygia;Ctenosquamata;Acanthomorphata;Euacanthomorphacea;Percomorphaceae;Ovalentaria;Atherinomorphae;Cyprinodontiformes;Cyprinodontoidei;Poeciliidae;Poeciliinae;Gambusia;Gambusia affinis
UdeM00000033550 90988 90.9 UdeM00000033550 XP_039503614.1 27 278 158 7 3 268 26 270 2.70E-15 90.9 90988 cellular organisms;Eukaryota;Opisthokonta;Metazoa;Eumetazoa;Bilateria;Deuterostomia;Chordata;Craniata;Vertebrata;Gnathostomata;Teleostomi;Euteleostomi;Actinopterygii;Actinopteri;Neopterygii;Teleostei;Osteoglossocephalai;Clupeocephala;Otomorpha;Ostariophysi;Otophysi;Cypriniphysae;Cypriniformes;Cyprinoidei;Leuciscidae;Pogonichthyinae;Pimephales;Pimephales promelas
UdeM00000033551 6596 392.9 UdeM00000033551 XP_045162242.1 33.8 722 356 8 18 632 72 778 7.90E-106 392.9 6596 cellular organisms;Eukaryota;Opisthokonta;Metazoa;Eumetazoa;Bilateria;Protostomia;Spiralia;Lophotrochozoa;Mollusca;Bivalvia;Autobranchia;Heteroconchia;Euheterodonta;Imparidentia;Venerida;Veneroidea;Veneridae;Mercenaria;Mercenaria mercenaria
UdeM00000033552 6596 389.4 UdeM00000033552 XP_045162242.1 33.4 731 361 11 12 633 66 779 8.70E-105 389.4 6596 cellular organisms;Eukaryota;Opisthokonta;Metazoa;Eumetazoa;Bilateria;Protostomia;Spiralia;Lophotrochozoa;Mollusca;Bivalvia;Autobranchia;Heteroconchia;Euheterodonta;Imparidentia;Venerida;Veneroidea;Veneridae;Mercenaria;Mercenaria mercenaria
UdeM00000033553 6596 507.3 UdeM00000033553 XP_045204674.1 45.1 585 312 7 9 592 1 577 2.60E-140 507.3 6596 cellular organisms;Eukaryota;Opisthokonta;Metazoa;Eumetazoa;Bilateria;Protostomia;Spiralia;Lophotrochozoa;Mollusca;Bivalvia;Autobranchia;Heteroconchia;Euhe

terodonta;Imparidentia;Venerida;Veneroidea;Veneridae;Mercenaria;Mercenaria mercenaria
UdeM00000033554 6579 66.6 UdeM00000033554 XP_033742659.1 31.5 124 71 3 2 119 466 581 2.40E-08 66.6 6579 cellular organisms;Eukaryota;Opisthokonta;Metazoa;Eumetazoa;Bilateria;Protostomia;Spiralia;Lophotrochozoa;Mollusca;Bivalvia;Autobranchia;Pteriomorphia;Pectinida;Pectinoidea;Pectinidae;Pecten;Pecten maximus
UdeM00000033555 6596 86.3 UdeM00000033555 XP_045173396.1 52.5 80 37 1 1 80 92 170 2.70E-14 86.3 6596 cellular organisms;Eukaryota;Opisthokonta;Metazoa;Eumetazoa;Bilateria;Protostomia;Spiralia;Lophotrochozoa;Mollusca;Bivalvia;Autobranchia;Heteroconchia;Euheterodonta;Imparidentia;Venerida;Veneroidea;Veneridae;Mercenaria;Mercenaria mercenaria
UdeM00000033556 6596 205.3 UdeM00000033556 XP_045173396.1 55.9 170 74 1 42 211 2 170 7.90E-50 205.3 6596 cellular organisms;Eukaryota;Opisthokonta;Metazoa;Eumetazoa;Bilateria;Protostomia;Spiralia;Lophotrochozoa;Mollusca;Bivalvia;Autobranchia;Heteroconchia;Euheterodonta;Imparidentia;Venerida;Veneroidea;Veneridae;Mercenaria;Mercenaria mercenaria
UdeM00000033557 6596 470.7 UdeM00000033557 XP_045204106.1 52.3 455 213 3 48 499 5 458 2.50E-129 470.7 6596 cellular organisms;Eukaryota;Opisthokonta;Metazoa;Eumetazoa;Bilateria;Protostomia;Spiralia;Lophotrochozoa;Mollusca;Bivalvia;Autobranchia;Heteroconchia;Euheterodonta;Imparidentia;Venerida;Veneroidea;Veneridae;Mercenaria;Mercenaria mercenaria
UdeM00000033558 6596 237.3 UdeM00000033558 XP_045204271.1 35.8 338 213 4 1 337 245 579 2.90E-59 237.3 6596 cellular organisms;Eukaryota;Opisthokonta;Metazoa;Eumetazoa;Bilateria;Protostomia;Spiralia;Lophotrochozoa;Mollusca;Bivalvia;Autobranchia;Heteroconchia;Euheterodonta;Imparidentia;Venerida;Veneroidea;Veneridae;Mercenaria;Mercenaria mercenaria
UdeM00000033559 6596 253.4 UdeM00000033559 XP_045173396.1 54.2 227 100 3 7 231 2 226 2.90E-64 253.4 6596 cellular organisms;Eukaryota;Opisthokonta;Metazoa;Eumetazoa;Bilateria;Protostomia;Spiralia;Lophotrochozoa;Mollusca;Bivalvia;Autobranchia;Heteroconchia;Euheterodonta;Imparidentia;Venerida;Veneroidea;Veneridae;Mercenaria;Mercenaria mercenaria
UdeM00000033560 6596 378.3 UdeM00000033560 XP_045204271.1 41.4 466 265 5 3 465 119 579 1.50E-101 378.3 6596 cellular organisms;Eukaryota;Opisthokonta;Metazoa;Eumetazoa;Bilateria;Protostomia;Spiralia;Lophotrochozoa;Mollusca;Bivalvia;Autobranchia;Heteroconchia;Euheterodonta;Imparidentia;Venerida;Veneroidea;Veneridae;Mercenaria;Mercenaria mercenaria
UdeM00000033561 6596 379.8 UdeM00000033561 XP_045204673.1 43.4 470 259 5 1 469 115 578 5.00E-102 379.8 6596 cellular organisms;Eukaryota;Opisthokonta;Metazoa;Eumetazoa;Bilateria;Protostomia;Spiralia;Lophotrochozoa;Mollusca;Bivalvia;Autobranchia;Heteroconchia;Euheterodonta;Imparidentia;Venerida;Veneroidea;Veneridae;Mercenaria;Mercenaria mercenaria
UdeM00000033562 6596 287.3 UdeM00000033562 XP_045162913.1 86.5 171 22 1 1 170 1 171 1.20E-74 287.3 6596 cellular organisms;Eukaryota;Opisthokonta;Metazoa;Eumetazoa;Bilateria;Protostomia;Spiralia;Lophotrochozoa;Mollusca;Bivalvia;Autobranchia;Heteroconchia;Euhe

terodonta;Imparidentia;Venerida;Veneroidea;Veneridae;Mercenaria;Mercenaria mercenaria
UdeM00000033563 6596 319.3 UdeM00000033563 XP_045162913.1 87.6 185 23 0 1 185 1 185 3.20E-84 319.3 6596 cellular organisms;Eukaryota;Opisthokonta;Metazoa;Eumetazoa;Bilateria;Protostomia;Spiralia;Lophotrochozoa;Mollusca;Bivalvia;Autobranchia;Heteroconchia;Euheterodonta;Imparidentia;Venerida;Veneroidea;Veneridae;Mercenaria;Mercenaria mercenaria
UdeM00000033564 1735272 180.3 UdeM00000033564 XP_041378161.1 43.9 198 107 2 7 200 1 198 2.70E-42 180.3 1735272 cellular organisms;Eukaryota;Opisthokonta;Metazoa;Eumetazoa;Bilateria;Protostomia;Spiralia;Lophotrochozoa;Mollusca;Gastropoda;Neomphalina;Neomphaloidea;Peltospiridae;Gigantopelta;Gigantopelta aegis
UdeM00000033565 6565 1270.4 UdeM00000033565 XP_022342289.1 54.4 1551 622 25 1 1503 1 1513 0.00E+00 1270.4 6565 cellular organisms;Eukaryota;Opisthokonta;Metazoa;Eumetazoa;Bilateria;Protostomia;Spiralia;Lophotrochozoa;Mollusca;Bivalvia;Autobranchia;Pteriomorphia;Ostreida;Ostreoidea;Ostreidae;Crassostrea;Crassostrea virginica
UdeM00000033566 6565 588.6 UdeM00000033566 XP_022342288.1 46.7 1104 492 27 1 1056 383 1438 1.60E-164 588.6 6565 cellular organisms;Eukaryota;Opisthokonta;Metazoa;Eumetazoa;Bilateria;Protostomia;Spiralia;Lophotrochozoa;Mollusca;Bivalvia;Autobranchia;Pteriomorphia;Ostreida;Ostreoidea;Ostreidae;Crassostrea;Crassostrea virginica
UdeM00000033567 6596 399.1 UdeM00000033567 XP_045162447.1 75.9 261 60 3 1 259 34 293 4.40E-108 399.1 6596 cellular organisms;Eukaryota;Opisthokonta;Metazoa;Eumetazoa;Bilateria;Protostomia;Spiralia;Lophotrochozoa;Mollusca;Bivalvia;Autobranchia;Heteroconchia;Euheterodonta;Imparidentia;Venerida;Veneroidea;Veneridae;Mercenaria;Mercenaria mercenaria
UdeM00000033569 6579 1069.7 UdeM00000033569 XP_033763612.1 75.3 676 167 0 1 676 1 676 0.00E+00 1069.7 6579 cellular organisms;Eukaryota;Opisthokonta;Metazoa;Eumetazoa;Bilateria;Protostomia;Spiralia;Lophotrochozoa;Mollusca;Bivalvia;Autobranchia;Pteriomorphia;Pectinida;Pectinoidea;Pectinidae;Pecten;Pecten maximus
UdeM00000033570 6596 453.8 UdeM00000033570 XP_045179877.1 42.5 560 310 7 6 555 3 560 3.30E-124 453.8 6596 cellular organisms;Eukaryota;Opisthokonta;Metazoa;Eumetazoa;Bilateria;Protostomia;Spiralia;Lophotrochozoa;Mollusca;Bivalvia;Autobranchia;Heteroconchia;Euheterodonta;Imparidentia;Venerida;Veneroidea;Veneridae;Mercenaria;Mercenaria mercenaria
UdeM00000033571 6596 453.4 UdeM00000033571 XP_045179877.1 42.6 559 309 7 1 549 4 560 4.20E-124 453.4 6596 cellular organisms;Eukaryota;Opisthokonta;Metazoa;Eumetazoa;Bilateria;Protostomia;Spiralia;Lophotrochozoa;Mollusca;Bivalvia;Autobranchia;Heteroconchia;Euheterodonta;Imparidentia;Venerida;Veneroidea;Veneridae;Mercenaria;Mercenaria mercenaria
UdeM00000033574 6596 432.6 UdeM00000033574 XP_045163662.1 39.5 559 326 7 3 552 6 561 7.70E-118 432.6 6596 cellular organisms;Eukaryota;Opisthokonta;Metazoa;Eumetazoa;Bilateria;Protostomia;Spiralia;Lophotrochozoa;Mollusca;Bivalvia;Autobranchia;Heteroconchia;Euhe

terodonta;Imparidentia;Venerida;Veneroidea;Veneridae;Mercenaria;Mercenaria mercenaria
UdeM00000033576 1735272 210.7 UdeM00000033576 XP_041378362.1 43.3 630 302 17 21 622 21 623 5.40E-51 210.7 1735272 cellular organisms;Eukaryota;Opisthokonta;Metazoa;Eumetazoa;Bilateria;Protostomia;Spiralia;Lophotrochozoa;Mollusca;Gastropoda;Neomphalina;Neomphaloidea;Peltospiridae;Gigantopelta;Gigantopelta aegis
UdeM00000033577 6573 225.3 UdeM00000033577 XP_021363050.1 46.1 245 122 3 62 298 60 302 1.10E-55 225.3 6573 cellular organisms;Eukaryota;Opisthokonta;Metazoa;Eumetazoa;Bilateria;Protostomia;Spiralia;Lophotrochozoa;Mollusca;Bivalvia;Autobranchia;Pteriomorphia;Pectinida;Pectinoidea;Pectinidae;Mizuhopecten;Mizuhopecten yessoensis
UdeM00000033580 1735272 207.2 UdeM00000033580 XP_041356266.1 49.4 180 91 0 1 180 429 608 1.70E-50 207.2 1735272 cellular organisms;Eukaryota;Opisthokonta;Metazoa;Eumetazoa;Bilateria;Protostomia;Spiralia;Lophotrochozoa;Mollusca;Gastropoda;Neomphalina;Neomphaloidea;Peltospiridae;Gigantopelta;Gigantopelta aegis
UdeM00000033581 6596 80.5 UdeM00000033581 XP_045192251.1 52.7 91 39 3 133 220 330 419 3.00E-12 80.5 6596 cellular organisms;Eukaryota;Opisthokonta;Metazoa;Eumetazoa;Bilateria;Protostomia;Spiralia;Lophotrochozoa;Mollusca;Bivalvia;Autobranchia;Heteroconchia;Euheterodonta;Imparidentia;Venerida;Veneroidea;Veneridae;Mercenaria;Mercenaria mercenaria
UdeM00000033582 6596 603.2 UdeM00000033582 XP_045163606.1 69.5 426 92 2 5 395 98 520 2.40E-169 603.2 6596 cellular organisms;Eukaryota;Opisthokonta;Metazoa;Eumetazoa;Bilateria;Protostomia;Spiralia;Lophotrochozoa;Mollusca;Bivalvia;Autobranchia;Heteroconchia;Euheterodonta;Imparidentia;Venerida;Veneroidea;Veneridae;Mercenaria;Mercenaria mercenaria
UdeM00000033583 6579 117.1 UdeM00000033583 XP_033763904.1 34.6 211 113 5 906 1099 158 360 1.70E-22 117.1 6579 cellular organisms;Eukaryota;Opisthokonta;Metazoa;Eumetazoa;Bilateria;Protostomia;Spiralia;Lophotrochozoa;Mollusca;Bivalvia;Autobranchia;Pteriomorphia;Pectinida;Pectinoidea;Pectinidae;Pecten;Pecten maximus
UdeM00000033585 6573 230.3 UdeM00000033585 XP_021376553.1 45 260 138 2 1 256 189 447 3.00E-57 230.3 6573 cellular organisms;Eukaryota;Opisthokonta;Metazoa;Eumetazoa;Bilateria;Protostomia;Spiralia;Lophotrochozoa;Mollusca;Bivalvia;Autobranchia;Pteriomorphia;Pectinida;Pectinoidea;Pectinidae;Mizuhopecten;Mizuhopecten yessoensis
UdeM00000033586 6573 142.1 UdeM00000033586 XP_021376553.1 51.9 133 60 2 77 207 1 131 7.80E-31 142.1 6573 cellular organisms;Eukaryota;Opisthokonta;Metazoa;Eumetazoa;Bilateria;Protostomia;Spiralia;Lophotrochozoa;Mollusca;Bivalvia;Autobranchia;Pteriomorphia;Pectinida;Pectinoidea;Pectinidae;Mizuhopecten;Mizuhopecten yessoensis
UdeM00000033588 29159 68.2 UdeM00000033588 XP_019918807.2 22 428 276 12 271 691 650 1026 4.80E-08 68.2 29159 cellular organisms;Eukaryota;Opisthokonta;Metazoa;Eumetazoa;Bilateria;Protostomia;Spiralia;Lophotrochozoa;Mollusca;Bivalvia;Autobranchia;Pteriomorphia;Ostreida;Ostreoidea;Ostreidae;Crassostrea;Crassostrea gigas
UdeM00000033589 6596 748.8 UdeM00000033589 XP_045161121.1 51.5 699 332 5 214 907 82 778 8.00E-213 748.8 6596 cellular organisms;Eukaryota;Opisthokonta;Metazoa;Eumetazoa;Bilateria;Protostomia;

Spiralia;Lophotrochozoa;Mollusca;Bivalvia;Autobranchia;Heteroconchia;Euheterodonta;Imparidentia;Venerida;Veneroidea;Veneridae;Mercenaria;Mercenaria mercenaria
UdeM00000033590 6573 2988 UdeM00000033590 XP_021353290.1 44.4 3472
1847 34 1 3413 4784 8229 0.00E+00 2988 6573 cellular
organisms;Eukaryota;Opisthokonta;Metazoa;Eumetazoa;Bilateria;Protostomia;
Spiralia;Lophotrochozoa;Mollusca;Bivalvia;Autobranchia;Pteriomorphia;Pectinida;Pectinoidea;Pectinidae;Mizuhopecten;Mizuhopecten yessoensis
UdeM00000033591 6579 436.8 UdeM00000033591 XP_033724554.1 33.3 861
497 20 8 816 1397 2232 6.40E-119 436.8 6579 cellular
organisms;Eukaryota;Opisthokonta;Metazoa;Eumetazoa;Bilateria;Protostomia;
Spiralia;Lophotrochozoa;Mollusca;Bivalvia;Autobranchia;Pteriomorphia;Pectinida;Pectinoidea;Pectinidae;Pecten;Pecten maximus
UdeM00000033592 6596 1321.2 UdeM00000033592 XP_045156859.1 30.1
2542 1582 46 277 2708 4897 7352 0.00E+00 1321.2 6596
cellular
organisms;Eukaryota;Opisthokonta;Metazoa;Eumetazoa;Bilateria;Protostomia;
Spiralia;Lophotrochozoa;Mollusca;Bivalvia;Autobranchia;Heteroconchia;Euheterodonta;Imparidentia;Venerida;Veneroidea;Veneridae;Mercenaria;Mercenaria mercenaria
UdeM00000033593 6573 677.2 UdeM00000033593 XP_021353290.1 31.3 1339
852 29 9 1311 169 1475 4.30E-191 677.2 6573 cellular
organisms;Eukaryota;Opisthokonta;Metazoa;Eumetazoa;Bilateria;Protostomia;
Spiralia;Lophotrochozoa;Mollusca;Bivalvia;Autobranchia;Pteriomorphia;Pectinida;Pectinoidea;Pectinidae;Mizuhopecten;Mizuhopecten yessoensis
UdeM00000033594 6573 339.3 UdeM00000033594 XP_021353372.1 28.4 760
332 17 97 724 2445 3124 1.20E-89 339.3 6573 cellular
organisms;Eukaryota;Opisthokonta;Metazoa;Eumetazoa;Bilateria;Protostomia;
Spiralia;Lophotrochozoa;Mollusca;Bivalvia;Autobranchia;Pteriomorphia;Pectinida;Pectinoidea;Pectinidae;Mizuhopecten;Mizuhopecten yessoensis
UdeM00000033595 6565 500.4 UdeM00000033595 XP_022341753.1 25 1714
558 29 885 1877 658 2364 1.00E-137 500.4 6565 cellular
organisms;Eukaryota;Opisthokonta;Metazoa;Eumetazoa;Bilateria;Protostomia;
Spiralia;Lophotrochozoa;Mollusca;Bivalvia;Autobranchia;Pteriomorphia;Ostreida;Ostreoidea;Ostreidae;Crassostrea;Crassostrea virginica
UdeM00000041278 6596 326.2 UdeM00000041278 XP_045192847.1 67.2 262
84 2 9 270 11 270 4.70E-86 326.2 6596 cellular
organisms;Eukaryota;Opisthokonta;Metazoa;Eumetazoa;Bilateria;Protostomia;
Spiralia;Lophotrochozoa;Mollusca;Bivalvia;Autobranchia;Heteroconchia;Euheterodonta;Imparidentia;Venerida;Veneroidea;Veneridae;Mercenaria;Mercenaria mercenaria
UdeM00000041280 1735272 552.4 UdeM00000041280 XP_041376948.1 77.9
321 71 0 84 404 18 338 5.60E-154 552.4 1735272
cellular
organisms;Eukaryota;Opisthokonta;Metazoa;Eumetazoa;Bilateria;Protostomia;
Spiralia;Lophotrochozoa;Mollusca;Gastropoda;Neomphalina;Neomphaloidea;Peltospiridae;Gigantopelta;Gigantopelta aegis
UdeM00000043843 29159 305.4 UdeM00000043843 XP_034308217.1 35.9 443
257 4 32 448 8 449 1.20E-79 305.4 29159 cellular
organisms;Eukaryota;Opisthokonta;Metazoa;Eumetazoa;Bilateria;Protostomia;
Spiralia;Lophotrochozoa;Mollusca;Bivalvia;Autobranchia;Pteriomorphia;Ostreida;Ostreoidea;Ostreidae;Crassostrea;Crassostrea gigas
UdeM00000043844 29159 305.8 UdeM00000043844 XP_034308217.1 35.9 443
257 4 37 453 8 449 9.00E-80 305.8 29159 cellular

organisms;Eukaryota;Opisthokonta;Metazoa;Eumetazoa;Bilateria;Protostomia;
Spiralia;Lophotrochozoa;Mollusca;Bivalvia;Autobranchia;Pteriomorphia;Ostr
eida;Ostreoidea;Ostreidae;Crassostrea;Crassostrea gigas
UdeM00000043845 29159 288.1 UdeM00000043845 XP_034308217.1 34.8 443
262 4 81 497 8 449 2.20E-74 288.1 29159 cellular
organisms;Eukaryota;Opisthokonta;Metazoa;Eumetazoa;Bilateria;Protostomia;
Spiralia;Lophotrochozoa;Mollusca;Bivalvia;Autobranchia;Pteriomorphia;Ostr
eida;Ostreoidea;Ostreidae;Crassostrea;Crassostrea gigas
UdeM00000043846 29159 288.1 UdeM00000043846 XP_034308217.1 34.8 443
262 4 8 424 8 449 1.80E-74 288.1 29159 cellular
organisms;Eukaryota;Opisthokonta;Metazoa;Eumetazoa;Bilateria;Protostomia;
Spiralia;Lophotrochozoa;Mollusca;Bivalvia;Autobranchia;Pteriomorphia;Ostr
eida;Ostreoidea;Ostreidae;Crassostrea;Crassostrea gigas
UdeM00000043847 6596 528.5 UdeM00000043847 XP_045171564.1 35.7 1187
595 45 2 1132 499 1573 2.10E-146 528.5 6596 cellular
organisms;Eukaryota;Opisthokonta;Metazoa;Eumetazoa;Bilateria;Protostomia;
Spiralia;Lophotrochozoa;Mollusca;Bivalvia;Autobranchia;Heteroconchia;Euhe
terodonta;Imparidentia;Venerida;Veneroidea;Veneridae;Mercenaria;Mercenari
a mercenaria
UdeM00000043848 6565 209.9 UdeM00000043848 XP_022299727.1 46.5 228
119 2 1 226 46 272 3.60E-51 209.9 6565 cellular
organisms;Eukaryota;Opisthokonta;Metazoa;Eumetazoa;Bilateria;Protostomia;
Spiralia;Lophotrochozoa;Mollusca;Bivalvia;Autobranchia;Pteriomorphia;Ostr
eida;Ostreoidea;Ostreidae;Crassostrea;Crassostrea virginica
UdeM00000043849 6596 396 UdeM00000043849 XP_045171564.1 58.6 321
131 2 9 328 189 508 4.80E-107 396 6596 cellular
organisms;Eukaryota;Opisthokonta;Metazoa;Eumetazoa;Bilateria;Protostomia;
Spiralia;Lophotrochozoa;Mollusca;Bivalvia;Autobranchia;Heteroconchia;Euhe
terodonta;Imparidentia;Venerida;Veneroidea;Veneridae;Mercenaria;Mercenari
a mercenaria
UdeM00000043850 6565 134 UdeM00000043850 XP_022343716.1 43.8 201
95 6 6 203 4 189 2.10E-28 134 6565 cellular
organisms;Eukaryota;Opisthokonta;Metazoa;Eumetazoa;Bilateria;Protostomia;
Spiralia;Lophotrochozoa;Mollusca;Bivalvia;Autobranchia;Pteriomorphia;Ostr
eida;Ostreoidea;Ostreidae;Crassostrea;Crassostrea virginica
UdeM00000043852 29159 115.9 UdeM00000043852 XP_034339168.1 37.5 192
94 4 1 167 166 356 6.30E-23 115.9 29159 cellular
organisms;Eukaryota;Opisthokonta;Metazoa;Eumetazoa;Bilateria;Protostomia;
Spiralia;Lophotrochozoa;Mollusca;Bivalvia;Autobranchia;Pteriomorphia;Ostr
eida;Ostreoidea;Ostreidae;Crassostrea;Crassostrea gigas
UdeM00000044348 6596 369 UdeM00000044348 XP_045201497.1 54.5 321
136 1 1 321 1 311 6.10E-99 369 6596 cellular
organisms;Eukaryota;Opisthokonta;Metazoa;Eumetazoa;Bilateria;Protostomia;
Spiralia;Lophotrochozoa;Mollusca;Bivalvia;Autobranchia;Heteroconchia;Euhe
terodonta;Imparidentia;Venerida;Veneroidea;Veneridae;Mercenaria;Mercenari
a mercenaria
UdeM00000044350 6596 125.2 UdeM00000044350 XP_045171743.1 44.4 153
81 2 12 160 54 206 7.70E-26 125.2 6596 cellular
organisms;Eukaryota;Opisthokonta;Metazoa;Eumetazoa;Bilateria;Protostomia;
Spiralia;Lophotrochozoa;Mollusca;Bivalvia;Autobranchia;Heteroconchia;Euhe
terodonta;Imparidentia;Venerida;Veneroidea;Veneridae;Mercenaria;Mercenari
a mercenaria
UdeM00000041024 1735272 131.3 UdeM00000041024 XP_041360711.1 34.7
199 117 3 2 198 3 190 1.50E-27 131.3 1735272

cellular organisms;Eukaryota;Opisthokonta;Metazoa;Eumetazoa;Bilateria;Protostomia;Spiralia;Lophotrochozoa;Mollusca;Gastropoda;Neomphalina;Neomphaloidea;Peltospiridae;Gigantopelta;Gigantopelta aegis
UdeM00000041025 6565 87 UdeM00000041025 XP_022297828.1 54.5 77 31 3 13 87 70 144 1.30E-14 87 6565 cellular organisms;Eukaryota;Opisthokonta;Metazoa;Eumetazoa;Bilateria;Protostomia;Spiralia;Lophotrochozoa;Mollusca;Bivalvia;Autobranchia;Pteriomorphia;Ostreida;Ostreoidea;Ostreidae;Crassostrea;Crassostrea virginica
UdeM00000041026 6565 773.5 UdeM00000041026 XP_022299017.1 29.8 1861 1165 42 64 1900 377 2119 6.30E-220 773.5 6565 cellular organisms;Eukaryota;Opisthokonta;Metazoa;Eumetazoa;Bilateria;Protostomia;Spiralia;Lophotrochozoa;Mollusca;Bivalvia;Autobranchia;Pteriomorphia;Ostreida;Ostreoidea;Ostreidae;Crassostrea;Crassostrea virginica
UdeM00000041027 6573 72.8 UdeM00000041027 XP_021372396.1 27.1 192 126 6 183 366 281 466 1.10E-09 72.8 6573 cellular organisms;Eukaryota;Opisthokonta;Metazoa;Eumetazoa;Bilateria;Protostomia;Spiralia;Lophotrochozoa;Mollusca;Bivalvia;Autobranchia;Pteriomorphia;Pectinida;Pectinoidea;Pectinidae;Mizuhopecten;Mizuhopecten yessoensis
UdeM00000041032 6579 855.1 UdeM00000041032 XP_033739131.1 60.9 645 250 2 1 644 726 1369 5.60E-245 855.1 6579 cellular organisms;Eukaryota;Opisthokonta;Metazoa;Eumetazoa;Bilateria;Protostomia;Spiralia;Lophotrochozoa;Mollusca;Bivalvia;Autobranchia;Pteriomorphia;Pectinida;Pectinoidea;Pectinidae;Pecten;Pecten maximus
UdeM00000036145 6596 502.3 UdeM00000036145 XP_045194118.1 35.2 915 492 14 74 905 1655 2551 1.60E-138 502.3 6596 cellular organisms;Eukaryota;Opisthokonta;Metazoa;Eumetazoa;Bilateria;Protostomia;Spiralia;Lophotrochozoa;Mollusca;Bivalvia;Autobranchia;Heteroconchia;Euheterodonta;Imparidentia;Venerida;Veneroidea;Veneridae;Mercenaria;Mercenaria mercenaria
UdeM00000036147 88082 77 UdeM00000036147 XP_039177253.1 33.3 138 71 3 1 117 2499 2636 3.60E-11 77 88082 cellular organisms;Eukaryota;Opisthokonta;Metazoa;Eumetazoa;Bilateria;Deuterostomia;Chordata;Craniata;Vertebrata;Gnathostomata;Teleostomi;Euteleostomi;Sarcopterygii;Dipnotetrapodomorpha;Tetrapoda;Amniota;Sauropsida;Sauria;Lepidosauria;Squamata;Bifurcata;Unidentata;Episquamata;Toxicofera;Serpentes;Colubroidea;Viperidae;Crotalinae;Crotalus;Crotalus tigris
UdeM00000036150 6596 312 UdeM00000036150 XP_045194118.1 36.2 531 253 7 1 457 1708 2226 1.30E-81 312 6596 cellular organisms;Eukaryota;Opisthokonta;Metazoa;Eumetazoa;Bilateria;Protostomia;Spiralia;Lophotrochozoa;Mollusca;Bivalvia;Autobranchia;Heteroconchia;Euheterodonta;Imparidentia;Venerida;Veneroidea;Veneridae;Mercenaria;Mercenaria mercenaria
UdeM00000036151 6596 487.6 UdeM00000036151 XP_045194118.1 34.3 858 472 11 1 780 1708 2551 3.40E-134 487.6 6596 cellular organisms;Eukaryota;Opisthokonta;Metazoa;Eumetazoa;Bilateria;Protostomia;Spiralia;Lophotrochozoa;Mollusca;Bivalvia;Autobranchia;Heteroconchia;Euheterodonta;Imparidentia;Venerida;Veneroidea;Veneridae;Mercenaria;Mercenaria mercenaria
UdeM00000036152 6596 184.5 UdeM00000036152 XP_045194118.1 31.8 318 212 3 28 341 2235 2551 3.20E-43 184.5 6596 cellular organisms;Eukaryota;Opisthokonta;Metazoa;Eumetazoa;Bilateria;Protostomia;Spiralia;Lophotrochozoa;Mollusca;Bivalvia;Autobranchia;Heteroconchia;Euhe

terodonta;Imparidentia;Venerida;Veneroidea;Veneridae;Mercenaria;Mercenaria mercenaria
UdeM00000035004 6596 128.6 UdeM00000035004 XP_045204527.1 34.2 202 127 2 346 542 206 406 2.30E-26 128.6 6596 cellular organisms;Eukaryota;Opisthokonta;Metazoa;Eumetazoa;Bilateria;Protostomia;Spiralia;Lophotrochozoa;Mollusca;Bivalvia;Autobranchia;Heteroconchia;Euheterodonta;Imparidentia;Venerida;Veneroidea;Veneridae;Mercenaria;Mercenaria mercenaria
UdeM00000035007 6565 157.9 UdeM00000035007 XP_022320073.1 34.3 236 150 3 615 845 443 678 5.60E-35 157.9 6565 cellular organisms;Eukaryota;Opisthokonta;Metazoa;Eumetazoa;Bilateria;Protostomia;Spiralia;Lophotrochozoa;Mollusca;Bivalvia;Autobranchia;Pteriomorphia;Ostreida;Ostreoidea;Ostreidae;Crassostrea;Crassostrea virginica
UdeM00000035008 225164 354.8 UdeM00000035008 XP_009062152.1 51.5 338 145 4 24 356 362 685 1.30E-94 354.8 225164 cellular organisms;Eukaryota;Opisthokonta;Metazoa;Eumetazoa;Bilateria;Protostomia;Spiralia;Lophotrochozoa;Mollusca;Gastropoda;Patellogastropoda;Lottioidea;Lottiidae;Lottia;Lottia gigantea
UdeM00000035009 225164 362.8 UdeM00000035009 XP_009062152.1 53 328 145 3 24 346 362 685 4.70E-97 362.8 225164 cellular organisms;Eukaryota;Opisthokonta;Metazoa;Eumetazoa;Bilateria;Protostomia;Spiralia;Lophotrochozoa;Mollusca;Gastropoda;Patellogastropoda;Lottioidea;Lottiidae;Lottia;Lottia gigantea
UdeM00000035011 6596 169.1 UdeM00000035011 XP_045206665.1 36.8 269 143 8 263 521 54 305 1.50E-38 169.1 6596 cellular organisms;Eukaryota;Opisthokonta;Metazoa;Eumetazoa;Bilateria;Protostomia;Spiralia;Lophotrochozoa;Mollusca;Bivalvia;Autobranchia;Heteroconchia;Euheterodonta;Imparidentia;Venerida;Veneroidea;Veneridae;Mercenaria;Mercenaria mercenaria
UdeM00000035013 6596 169.1 UdeM00000035013 XP_045206665.1 36.8 269 143 8 61 319 54 305 9.20E-39 169.1 6596 cellular organisms;Eukaryota;Opisthokonta;Metazoa;Eumetazoa;Bilateria;Protostomia;Spiralia;Lophotrochozoa;Mollusca;Bivalvia;Autobranchia;Heteroconchia;Euheterodonta;Imparidentia;Venerida;Veneroidea;Veneridae;Mercenaria;Mercenaria mercenaria
UdeM00000035014 6565 134.4 UdeM00000035014 XP_022320730.1 32.5 249 143 5 318 544 273 518 4.30E-28 134.4 6565 cellular organisms;Eukaryota;Opisthokonta;Metazoa;Eumetazoa;Bilateria;Protostomia;Spiralia;Lophotrochozoa;Mollusca;Bivalvia;Autobranchia;Pteriomorphia;Ostreida;Ostreoidea;Ostreidae;Crassostrea;Crassostrea virginica
UdeM00000035016 7604 302.4 UdeM00000035016 XP_033633146.1 41 390 212 3 262 633 29 418 2.40E-78 302.4 7604 cellular organisms;Eukaryota;Opisthokonta;Metazoa;Eumetazoa;Bilateria;Deuterostomia;Echinodermata;Eleutherozoa;Asterozoa;Asteroidea;Forcipulatacea;Forcipulatida;Asteriidae;Asterias;Asterias rubens
UdeM00000035017 6596 149.4 UdeM00000035017 XP_045204527.1 30.9 236 159 2 48 283 173 404 6.80E-33 149.4 6596 cellular organisms;Eukaryota;Opisthokonta;Metazoa;Eumetazoa;Bilateria;Protostomia;Spiralia;Lophotrochozoa;Mollusca;Bivalvia;Autobranchia;Heteroconchia;Euheterodonta;Imparidentia;Venerida;Veneroidea;Veneridae;Mercenaria;Mercenaria mercenaria

UdeM00000035018 6596 293.1 UdeM00000035018 XP_045204528.1 44 336
173 4 80 413 74 396 5.50E-76 293.1 6596 cellular
organisms;Eukaryota;Opisthokonta;Metazoa;Eumetazoa;Bilateria;Protostomia;
Spiralia;Lophotrochozoa;Mollusca;Bivalvia;Autobranchia;Heteroconchia;Euhe
terodonta;Imparidentia;Venerida;Veneroidea;Veneridae;Mercenaria;Mercenari
a mercenaria
UdeM00000035019 6596 128.6 UdeM00000035019 XP_045204528.1 32.4 216
133 4 48 250 181 396 1.10E-26 128.6 6596 cellular
organisms;Eukaryota;Opisthokonta;Metazoa;Eumetazoa;Bilateria;Protostomia;
Spiralia;Lophotrochozoa;Mollusca;Bivalvia;Autobranchia;Heteroconchia;Euhe
terodonta;Imparidentia;Venerida;Veneroidea;Veneridae;Mercenaria;Mercenari
a mercenaria
UdeM00000035022 6596 662.5 UdeM00000035022 XP_045202954.1 55.1 577
247 4 2 567 26 601 4.70E-187 662.5 6596 cellular
organisms;Eukaryota;Opisthokonta;Metazoa;Eumetazoa;Bilateria;Protostomia;
Spiralia;Lophotrochozoa;Mollusca;Bivalvia;Autobranchia;Heteroconchia;Euhe
terodonta;Imparidentia;Venerida;Veneroidea;Veneridae;Mercenaria;Mercenari
a mercenaria
UdeM00000035023 75366 446 UdeM00000035023 XP_016132731.1 63.8 376
136 0 1 376 229 604 4.70E-122 446 75366 cellular
organisms;Eukaryota;Opisthokonta;Metazoa;Eumetazoa;Bilateria;Deuterostomi
a;Chordata;Craniata;Vertebrata;Gnathostomata;Teleostomi;Euteleostomi;Acti
nopterygii;Actinopteri;Neopterygii;Teleostei;Osteoglossocephalai;Clupeoce
phala;Otomorpha;Ostariophysi;Otophysi;Cypriniphysae;Cypriniformes;Cyprino
idei;Cyprinidae;Cyprininae;Sinocyclocheilus;Sinocyclocheilus grahami
UdeM00000035024 6596 455.7 UdeM00000035024 XP_045184046.1 43.9 508
272 4 27 528 11 511 8.90E-125 455.7 6596 cellular
organisms;Eukaryota;Opisthokonta;Metazoa;Eumetazoa;Bilateria;Protostomia;
Spiralia;Lophotrochozoa;Mollusca;Bivalvia;Autobranchia;Heteroconchia;Euhe
terodonta;Imparidentia;Venerida;Veneroidea;Veneridae;Mercenaria;Mercenari
a mercenaria
UdeM00000035025 6596 388.3 UdeM00000035025 XP_045184046.1 38.1 520
310 7 1 513 47 561 1.60E-104 388.3 6596 cellular
organisms;Eukaryota;Opisthokonta;Metazoa;Eumetazoa;Bilateria;Protostomia;
Spiralia;Lophotrochozoa;Mollusca;Bivalvia;Autobranchia;Heteroconchia;Euhe
terodonta;Imparidentia;Venerida;Veneroidea;Veneridae;Mercenaria;Mercenari
a mercenaria
UdeM00000035026 6596 433.7 UdeM00000035026 XP_045184046.1 40.6 534
302 8 9 534 9 535 3.30E-118 433.7 6596 cellular
organisms;Eukaryota;Opisthokonta;Metazoa;Eumetazoa;Bilateria;Protostomia;
Spiralia;Lophotrochozoa;Mollusca;Bivalvia;Autobranchia;Heteroconchia;Euhe
terodonta;Imparidentia;Venerida;Veneroidea;Veneridae;Mercenaria;Mercenari
a mercenaria
UdeM00000035027 6596 402.9 UdeM00000035027 XP_045184046.1 42.5 475
257 8 1 466 47 514 5.80E-109 402.9 6596 cellular
organisms;Eukaryota;Opisthokonta;Metazoa;Eumetazoa;Bilateria;Protostomia;
Spiralia;Lophotrochozoa;Mollusca;Bivalvia;Autobranchia;Heteroconchia;Euhe
terodonta;Imparidentia;Venerida;Veneroidea;Veneridae;Mercenaria;Mercenari
a mercenaria
UdeM00000035028 6596 383.6 UdeM00000035028 XP_045184046.1 38.2 524
291 9 1 496 39 557 3.80E-103 383.6 6596 cellular
organisms;Eukaryota;Opisthokonta;Metazoa;Eumetazoa;Bilateria;Protostomia;
Spiralia;Lophotrochozoa;Mollusca;Bivalvia;Autobranchia;Heteroconchia;Euhe

terodonta;Imparidentia;Venerida;Veneroidea;Veneridae;Mercenaria;Mercenaria mercenaria
UdeM00000035029 6596 621.3 UdeM00000035029 XP_045205200.1 41.6 896 427 16 331 1180 194 1039 2.50E-174 621.3 6596 cellular organisms;Eukaryota;Opisthokonta;Metazoa;Eumetazoa;Bilateria;Protostomia;Spiralia;Lophotrochozoa;Mollusca;Bivalvia;Autobranchia;Heteroconchia;Euheterodonta;Imparidentia;Venerida;Veneroidea;Veneridae;Mercenaria;Mercenaria mercenaria
UdeM00000035030 6596 612.1 UdeM00000035030 XP_045205200.1 40.4 913 447 17 331 1213 194 1039 1.60E-171 612.1 6596 cellular organisms;Eukaryota;Opisthokonta;Metazoa;Eumetazoa;Bilateria;Protostomia;Spiralia;Lophotrochozoa;Mollusca;Bivalvia;Autobranchia;Heteroconchia;Euheterodonta;Imparidentia;Venerida;Veneroidea;Veneridae;Mercenaria;Mercenaria mercenaria
UdeM00000035031 6596 626.3 UdeM00000035031 XP_045205200.1 41.1 913 448 16 331 1220 194 1039 8.00E-176 626.3 6596 cellular organisms;Eukaryota;Opisthokonta;Metazoa;Eumetazoa;Bilateria;Protostomia;Spiralia;Lophotrochozoa;Mollusca;Bivalvia;Autobranchia;Heteroconchia;Euheterodonta;Imparidentia;Venerida;Veneroidea;Veneridae;Mercenaria;Mercenaria mercenaria
UdeM00000035032 1735272 1637.1 UdeM00000035032 XP_041358787.1 60.6 1286 497 8 1 1283 3 1281 0.00E+00 1637.1 1735272 cellular organisms;Eukaryota;Opisthokonta;Metazoa;Eumetazoa;Bilateria;Protostomia;Spiralia;Lophotrochozoa;Mollusca;Gastropoda;Neomphalina;Neomphaloidea;Peltospiridae;Gigantopelta;Gigantopelta aegis
UdeM00000035033 29159 372.9 UdeM00000035033 XP_011456426.2 68.6 271 82 2 1 270 1 269 3.60E-100 372.9 29159 cellular organisms;Eukaryota;Opisthokonta;Metazoa;Eumetazoa;Bilateria;Protostomia;Spiralia;Lophotrochozoa;Mollusca;Bivalvia;Autobranchia;Pteriomorphia;Ostreida;Ostreoidea;Ostreidae;Crassostrea;Crassostrea gigas
UdeM00000035034 6596 318.2 UdeM00000035034 XP_045203457.1 63.9 244 87 1 1 244 1 243 9.50E-84 318.2 6596 cellular organisms;Eukaryota;Opisthokonta;Metazoa;Eumetazoa;Bilateria;Protostomia;Spiralia;Lophotrochozoa;Mollusca;Bivalvia;Autobranchia;Heteroconchia;Euheterodonta;Imparidentia;Venerida;Veneroidea;Veneridae;Mercenaria;Mercenaria mercenaria
UdeM00000035035 1735272 422.9 UdeM00000035035 XP_041359277.1 53.9 384 174 3 11 394 38 418 4.50E-115 422.9 1735272 cellular organisms;Eukaryota;Opisthokonta;Metazoa;Eumetazoa;Bilateria;Protostomia;Spiralia;Lophotrochozoa;Mollusca;Gastropoda;Neomphalina;Neomphaloidea;Peltospiridae;Gigantopelta;Gigantopelta aegis
UdeM00000035036 1735272 466.5 UdeM00000035036 XP_041359277.1 54.2 421 190 3 1 421 1 418 3.80E-128 466.5 1735272 cellular organisms;Eukaryota;Opisthokonta;Metazoa;Eumetazoa;Bilateria;Protostomia;Spiralia;Lophotrochozoa;Mollusca;Gastropoda;Neomphalina;Neomphaloidea;Peltospiridae;Gigantopelta;Gigantopelta aegis
UdeM00000035037 225164 374.4 UdeM00000035037 XP_009062152.1 53.4 328 144 3 73 395 362 685 1.90E-100 374.4 225164 cellular organisms;Eukaryota;Opisthokonta;Metazoa;Eumetazoa;Bilateria;Protostomia;

Spiralia;Lophotrochozoa;Mollusca;Gastropoda;Patellogastropoda;Lottioidea;
Lottiidae;Lottia;Lottia gigantea
UdeM00000035038 225164 366.7 UdeM00000035038 XP_009062152.1 49.6
359 169 4 50 403 334 685 3.80E-98 366.7 225164
cellular
organisms;Eukaryota;Opisthokonta;Metazoa;Eumetazoa;Bilateria;Protostomia;
Spiralia;Lophotrochozoa;Mollusca;Gastropoda;Patellogastropoda;Lottioidea;
Lottiidae;Lottia;Lottia gigantea
UdeM00000035039 6596 62 UdeM00000035039 XP_045203144.1 30.6 134
81 3 5 134 463 588 6.70E-07 62 6596 cellular
organisms;Eukaryota;Opisthokonta;Metazoa;Eumetazoa;Bilateria;Protostomia;
Spiralia;Lophotrochozoa;Mollusca;Bivalvia;Autobranchia;Heteroconchia;Euhe
terodonta;Imparidentia;Venerida;Veneroidea;Veneridae;Mercenaria;Mercenari
a mercenaria
UdeM00000035040 6596 127.9 UdeM00000035040 XP_045204673.1 39.2 148
89 1 1 148 284 430 1.10E-26 127.9 6596 cellular
organisms;Eukaryota;Opisthokonta;Metazoa;Eumetazoa;Bilateria;Protostomia;
Spiralia;Lophotrochozoa;Mollusca;Bivalvia;Autobranchia;Heteroconchia;Euhe
terodonta;Imparidentia;Venerida;Veneroidea;Veneridae;Mercenaria;Mercenari
a mercenaria
UdeM00000035041 6596 149.1 UdeM00000035041 XP_045208604.1 54.7 128
56 1 41 166 7 134 5.10E-33 149.1 6596 cellular
organisms;Eukaryota;Opisthokonta;Metazoa;Eumetazoa;Bilateria;Protostomia;
Spiralia;Lophotrochozoa;Mollusca;Bivalvia;Autobranchia;Heteroconchia;Euhe
terodonta;Imparidentia;Venerida;Veneroidea;Veneridae;Mercenaria;Mercenari
a mercenaria
UdeM00000035042 6596 496.5 UdeM00000035042 XP_045204674.1 44.9 581
310 8 7 585 4 576 4.60E-137 496.5 6596 cellular
organisms;Eukaryota;Opisthokonta;Metazoa;Eumetazoa;Bilateria;Protostomia;
Spiralia;Lophotrochozoa;Mollusca;Bivalvia;Autobranchia;Heteroconchia;Euhe
terodonta;Imparidentia;Venerida;Veneroidea;Veneridae;Mercenaria;Mercenari
a mercenaria
UdeM00000035044 6579 251.1 UdeM00000035044 XP_033752673.1 43.5 299
75 1 11 215 353 651 1.20E-63 251.1 6579 cellular
organisms;Eukaryota;Opisthokonta;Metazoa;Eumetazoa;Bilateria;Protostomia;
Spiralia;Lophotrochozoa;Mollusca;Bivalvia;Autobranchia;Pteriomorphia;Pect
inida;Pectinoidea;Pectinidae;Pecten;Pecten maximus
UdeM00000035043 225164 90.9 UdeM00000035043 XP_009058199.1 61.8
55 21 0 63 117 610 664 1.20E-15 90.9 225164
cellular
organisms;Eukaryota;Opisthokonta;Metazoa;Eumetazoa;Bilateria;Protostomia;
Spiralia;Lophotrochozoa;Mollusca;Gastropoda;Patellogastropoda;Lottioidea;
Lottiidae;Lottia;Lottia gigantea
UdeM00000035045 225164 67 UdeM00000035045 XP_009058199.1 46.8
79 35 2 1 79 31 102 1.30E-08 67 225164
cellular
organisms;Eukaryota;Opisthokonta;Metazoa;Eumetazoa;Bilateria;Protostomia;
Spiralia;Lophotrochozoa;Mollusca;Gastropoda;Patellogastropoda;Lottioidea;
Lottiidae;Lottia;Lottia gigantea
UdeM00000035047 69319 96.7 UdeM00000035047 XP_008557728.1 52.3 86
34 2 2 82 701 784 1.50E-17 96.7 69319 cellular
organisms;Eukaryota;Opisthokonta;Metazoa;Eumetazoa;Bilateria;Protostomia;
Ecdysozoa;Panarthropoda;Arthropoda;Mandibulata;Pancrustacea;Hexapoda;Inse
cta;Dicondylia;Pterygota;Neoptera;Endopterygota;Hymenoptera;Apocrita;Para

sitoida;Ichneumonoidea;Braconidae;Microgastrinae;Microplitis;Microplitis demolitor
UdeM00000035048 215358 164.9 UdeM00000035048 XP_027132200.1 33.8 293 173 7 1 284 1 281 1.60E-37 164.9 215358 cellular organisms;Eukaryota;Opisthokonta;Metazoa;Eumetazoa;Bilateria;Deuterostomia;Chordata;Craniata;Vertebrata;Gnathostomata;Teleostomi;Euteleostomi;Actinopterygii;Actinopteri;Neopterygii;Teleostei;Osteoglossocephalai;Clupeocephala;Euteleosteomorpha;Neoteleostei;Eurypterygia;Ctenosquamata;Acanthomorphata;Euacanthomorphacea;Percomorphaceae;Eupercaria;Eupercaria incertae sedis;Sciaenidae;Larimichthys;Larimichthys crocea
UdeM00000035049 30732 184.9 UdeM00000035049 XP_024118320.1 43.8 208 115 2 14 221 409 614 1.10E-43 184.9 30732 cellular organisms;Eukaryota;Opisthokonta;Metazoa;Eumetazoa;Bilateria;Deuterostomia;Chordata;Craniata;Vertebrata;Gnathostomata;Teleostomi;Euteleostomi;Actinopterygii;Actinopteri;Neopterygii;Teleostei;Osteoglossocephalai;Clupeocephala;Euteleosteomorpha;Neoteleostei;Eurypterygia;Ctenosquamata;Acanthomorphata;Euacanthomorphacea;Percomorphaceae;Ovalentaria;Atherinomorphae;Beloniformes;Adrianichthyoidei;Adrianichthyidae;Oryziinae;Oryzias;Oryzias melastigma
UdeM00000035050 375764 126.3 UdeM00000035050 XP_029977959.1 56.1 98 43 0 11 108 34 131 2.40E-26 126.3 375764 cellular organisms;Eukaryota;Opisthokonta;Metazoa;Eumetazoa;Bilateria;Deuterostomia;Chordata;Craniata;Vertebrata;Gnathostomata;Teleostomi;Euteleostomi;Actinopterygii;Actinopteri;Neopterygii;Teleostei;Osteoglossocephalai;Clupeocephala;Euteleosteomorpha;Neoteleostei;Eurypterygia;Ctenosquamata;Acanthomorphata;Euacanthomorphacea;Percomorphaceae;Gobiaria;Kurtiformes;Apogonoidei;Apogonidae;Apogoninae;Sphaeramia;Sphaeramia orbicularis
UdeM00000035051 6596 490.3 UdeM00000035051 XP_045204674.1 44.1 581 315 8 7 585 4 576 3.30E-135 490.3 6596 cellular organisms;Eukaryota;Opisthokonta;Metazoa;Eumetazoa;Bilateria;Protostomia;Spiralia;Lophotrochozoa;Mollusca;Bivalvia;Autobranchia;Heteroconchia;Euheterodonta;Imparidentia;Venerida;Veneroidea;Veneridae;Mercenaria;Mercenaria mercenaria
UdeM00000035052 6579 170.2 UdeM00000035052 XP_033752673.1 62.9 124 46 0 21 144 528 651 1.90E-39 170.2 6579 cellular organisms;Eukaryota;Opisthokonta;Metazoa;Eumetazoa;Bilateria;Protostomia;Spiralia;Lophotrochozoa;Mollusca;Bivalvia;Autobranchia;Pteriomorphia;Pectinida;Pectinoidea;Pectinidae;Pecten;Pecten maximus
UdeM00000035054 225164 357.8 UdeM00000035054 XP_009062152.1 53.6 323 138 4 2 316 367 685 1.40E-95 357.8 225164 cellular organisms;Eukaryota;Opisthokonta;Metazoa;Eumetazoa;Bilateria;Protostomia;Spiralia;Lophotrochozoa;Mollusca;Gastropoda;Patellogastropoda;Lottioidea;Lottiidae;Lottia;Lottia gigantea
UdeM00000035055 6596 112.1 UdeM00000035055 XP_045203146.1 36.2 185 114 2 1 185 398 578 7.80E-22 112.1 6596 cellular organisms;Eukaryota;Opisthokonta;Metazoa;Eumetazoa;Bilateria;Protostomia;Spiralia;Lophotrochozoa;Mollusca;Bivalvia;Autobranchia;Heteroconchia;Euheterodonta;Imparidentia;Venerida;Veneroidea;Veneridae;Mercenaria;Mercenaria mercenaria
UdeM00000035056 6596 131.7 UdeM00000035056 XP_045198473.1 58.8 102 42 0 16 117 13 114 6.10E-28 131.7 6596 cellular

organisms;Eukaryota;Opisthokonta;Metazoa;Eumetazoa;Bilateria;Protostomia;Spiralia;Lophotrochozoa;Mollusca;Bivalvia;Autobranchia;Heteroconchia;Euheterodonta;Imparidentia;Venerida;Veneroidea;Veneridae;Mercenaria;Mercenaria mercenaria
UdeM00000035057 6579 304.3 UdeM00000035057 XP_033742173.1 40.3 590 276 9 1 534 1 570 3.10E-79 304.3 6579 cellular organisms;Eukaryota;Opisthokonta;Metazoa;Eumetazoa;Bilateria;Protostomia;Spiralia;Lophotrochozoa;Mollusca;Bivalvia;Autobranchia;Pteriomorphia;Pectinida;Pectinoidea;Pectinidae;Pecten;Pecten maximus
UdeM00000035058 6565 139.8 UdeM00000035058 XP_022326746.1 70.3 91 27 0 1 91 1 91 1.70E-30 139.8 6565 cellular organisms;Eukaryota;Opisthokonta;Metazoa;Eumetazoa;Bilateria;Protostomia;Spiralia;Lophotrochozoa;Mollusca;Bivalvia;Autobranchia;Pteriomorphia;Ostreida;Ostreoidea;Ostreidae;Crassostrea;Crassostrea virginica
UdeM00000035059 6596 244.6 UdeM00000035059 XP_045204501.1 44.3 305 162 4 25 328 24 321 1.90E-61 244.6 6596 cellular organisms;Eukaryota;Opisthokonta;Metazoa;Eumetazoa;Bilateria;Protostomia;Spiralia;Lophotrochozoa;Mollusca;Bivalvia;Autobranchia;Heteroconchia;Euheterodonta;Imparidentia;Venerida;Veneroidea;Veneridae;Mercenaria;Mercenaria mercenaria
UdeM00000035061 6596 194.5 UdeM00000035061 XP_045205379.1 36.8 321 179 8 7 306 2 319 4.40E-46 194.5 6596 cellular organisms;Eukaryota;Opisthokonta;Metazoa;Eumetazoa;Bilateria;Protostomia;Spiralia;Lophotrochozoa;Mollusca;Bivalvia;Autobranchia;Heteroconchia;Euheterodonta;Imparidentia;Venerida;Veneroidea;Veneridae;Mercenaria;Mercenaria mercenaria
UdeM00000035062 6596 203 UdeM00000035062 XP_045205379.1 38 324 174 10 6 309 3 319 1.40E-48 203 6596 cellular organisms;Eukaryota;Opisthokonta;Metazoa;Eumetazoa;Bilateria;Protostomia;Spiralia;Lophotrochozoa;Mollusca;Bivalvia;Autobranchia;Heteroconchia;Euheterodonta;Imparidentia;Venerida;Veneroidea;Veneridae;Mercenaria;Mercenaria mercenaria
UdeM00000035063 6565 80.5 UdeM00000035063 XP_022336362.1 40.4 89 53 0 35 123 23 111 1.80E-12 80.5 6565 cellular organisms;Eukaryota;Opisthokonta;Metazoa;Eumetazoa;Bilateria;Protostomia;Spiralia;Lophotrochozoa;Mollusca;Bivalvia;Autobranchia;Pteriomorphia;Ostreida;Ostreoidea;Ostreidae;Crassostrea;Crassostrea virginica
UdeM00000035064 6565 129.8 UdeM00000035064 XP_022325109.1 35.4 198 127 1 5 202 1007 1203 1.50E-26 129.8 6565 cellular organisms;Eukaryota;Opisthokonta;Metazoa;Eumetazoa;Bilateria;Protostomia;Spiralia;Lophotrochozoa;Mollusca;Bivalvia;Autobranchia;Pteriomorphia;Ostreida;Ostreoidea;Ostreidae;Crassostrea;Crassostrea virginica
UdeM00000035065 6596 193 UdeM00000035065 XP_045204557.1 35.9 301 183 6 13 311 308 600 6.20E-46 193 6596 cellular organisms;Eukaryota;Opisthokonta;Metazoa;Eumetazoa;Bilateria;Protostomia;Spiralia;Lophotrochozoa;Mollusca;Bivalvia;Autobranchia;Heteroconchia;Euheterodonta;Imparidentia;Venerida;Veneroidea;Veneridae;Mercenaria;Mercenaria mercenaria
UdeM00000035066 6596 82.4 UdeM00000035066 XP_045202434.1 48.8 84 39 1 29 112 236 315 4.00E-13 82.4 6596 cellular organisms;Eukaryota;Opisthokonta;Metazoa;Eumetazoa;Bilateria;Protostomia;Spiralia;Lophotrochozoa;Mollusca;Bivalvia;Autobranchia;Heteroconchia;Euheterodonta;Imparidentia;Venerida;Veneroidea;Veneridae;Mercenaria;Mercenaria mercenaria

UdeM00000035067 6596 993.4 UdeM00000035067 XP_045204426.1 76.6 608
133 5 1 605 1 602 1.20E-286 993.4 6596 cellular
organisms;Eukaryota;Opisthokonta;Metazoa;Eumetazoa;Bilateria;Protostomia;
Spiralia;Lophotrochozoa;Mollusca;Bivalvia;Autobranchia;Heteroconchia;Euhe
terodonta;Imparidentia;Venerida;Veneroidea;Veneridae;Mercenaria;Mercenari
a mercenaria
UdeM00000035068 6573 267.7 UdeM00000035068 XP_021371193.1 60.1 198
78 1 13 209 13 210 1.20E-68 267.7 6573 cellular
organisms;Eukaryota;Opisthokonta;Metazoa;Eumetazoa;Bilateria;Protostomia;
Spiralia;Lophotrochozoa;Mollusca;Bivalvia;Autobranchia;Pteriomorphia;Pect
inida;Pectinoidea;Pectinidae;Mizuhopecten;Mizuhopecten yessoensis
UdeM00000035069 6573 264.6 UdeM00000035069 XP_021371194.1 59.7 196
78 1 13 207 13 208 1.00E-67 264.6 6573 cellular
organisms;Eukaryota;Opisthokonta;Metazoa;Eumetazoa;Bilateria;Protostomia;
Spiralia;Lophotrochozoa;Mollusca;Bivalvia;Autobranchia;Pteriomorphia;Pect
inida;Pectinoidea;Pectinidae;Mizuhopecten;Mizuhopecten yessoensis
UdeM00000035070 6596 509.6 UdeM00000035070 XP_045204674.1 45.6 581
306 8 11 589 4 576 5.30E-141 509.6 6596 cellular
organisms;Eukaryota;Opisthokonta;Metazoa;Eumetazoa;Bilateria;Protostomia;
Spiralia;Lophotrochozoa;Mollusca;Bivalvia;Autobranchia;Heteroconchia;Euhe
terodonta;Imparidentia;Venerida;Veneroidea;Veneridae;Mercenaria;Mercenari
a mercenaria
UdeM00000035071 6596 386.3 UdeM00000035071 XP_045204674.1 43.1 490
270 7 1 488 94 576 5.60E-104 386.3 6596 cellular
organisms;Eukaryota;Opisthokonta;Metazoa;Eumetazoa;Bilateria;Protostomia;
Spiralia;Lophotrochozoa;Mollusca;Bivalvia;Autobranchia;Heteroconchia;Euhe
terodonta;Imparidentia;Venerida;Veneroidea;Veneridae;Mercenaria;Mercenari
a mercenaria
UdeM00000035072 41139 171.8 UdeM00000035072 XP_044760115.1 50.6 170
75 3 2 166 735 900 7.40E-40 171.8 41139 cellular
organisms;Eukaryota;Opisthokonta;Metazoa;Eumetazoa;Bilateria;Protostomia;
Ecdysozoa;Panarthropoda;Arthropoda;Mandibulata;Pancrustacea;Hexapoda;Inse
cta;Dicondylia;Pterygota;Neoptera;Endopterygota;Coleoptera;Polyphaga;Cucu
jiformia;Cucujoidea;Coccinellidae;Coccinellinae;Coccinellini;Coccinella;C
occinella septempunctata
UdeM00000035075 6596 485.7 UdeM00000035075 XP_045173396.1 42.5 583
326 6 12 590 2 579 8.20E-134 485.7 6596 cellular
organisms;Eukaryota;Opisthokonta;Metazoa;Eumetazoa;Bilateria;Protostomia;
Spiralia;Lophotrochozoa;Mollusca;Bivalvia;Autobranchia;Heteroconchia;Euhe
terodonta;Imparidentia;Venerida;Veneroidea;Veneridae;Mercenaria;Mercenari
a mercenaria
UdeM00000035076 225164 365.9 UdeM00000035076 XP_009062152.1 54.8
323 137 3 2 319 367 685 5.10E-98 365.9 225164
cellular
organisms;Eukaryota;Opisthokonta;Metazoa;Eumetazoa;Bilateria;Protostomia;
Spiralia;Lophotrochozoa;Mollusca;Gastropoda;Patellogastropoda;Lottioidea;
Lottiidae;Lottia;Lottia gigantea
UdeM00000035077 6596 305.8 UdeM00000035077 XP_045204271.1 35.6 494
272 8 1 454 92 579 9.00E-80 305.8 6596 cellular
organisms;Eukaryota;Opisthokonta;Metazoa;Eumetazoa;Bilateria;Protostomia;
Spiralia;Lophotrochozoa;Mollusca;Bivalvia;Autobranchia;Heteroconchia;Euhe
terodonta;Imparidentia;Venerida;Veneroidea;Veneridae;Mercenaria;Mercenari
a mercenaria

UdeM00000035078 6596 375.2 UdeM00000035078 XP_045204106.1 56.1 328
140 3 3 326 8 335 9.20E-101 375.2 6596 cellular
organisms;Eukaryota;Opisthokonta;Metazoa;Eumetazoa;Bilateria;Protostomia;
Spiralia;Lophotrochozoa;Mollusca;Bivalvia;Autobranchia;Heteroconchia;Euhe
terodonta;Imparidentia;Venerida;Veneroidea;Veneridae;Mercenaria;Mercenari
a mercenaria
UdeM00000035079 6565 148.3 UdeM00000035079 XP_022321199.1 35.9 223
138 3 5 227 364 581 1.20E-32 148.3 6565 cellular
organisms;Eukaryota;Opisthokonta;Metazoa;Eumetazoa;Bilateria;Protostomia;
Spiralia;Lophotrochozoa;Mollusca;Bivalvia;Autobranchia;Pteriomorphia;Ostr
eida;Ostreoidea;Ostreidae;Crassostrea;Crassostrea virginica
UdeM00000035080 225164 370.9 UdeM00000035080 XP_009062152.1 54
328 142 3 23 345 362 685 1.70E-99 370.9 225164
cellular
organisms;Eukaryota;Opisthokonta;Metazoa;Eumetazoa;Bilateria;Protostomia;
Spiralia;Lophotrochozoa;Mollusca;Gastropoda;Patellogastropoda;Lottioidea;
Lottiidae;Lottia;Lottia gigantea
UdeM00000035082 6596 309.7 UdeM00000035082 XP_045204176.1 55.9 261
113 1 12 272 2 260 3.70E-81 309.7 6596 cellular
organisms;Eukaryota;Opisthokonta;Metazoa;Eumetazoa;Bilateria;Protostomia;
Spiralia;Lophotrochozoa;Mollusca;Bivalvia;Autobranchia;Heteroconchia;Euhe
terodonta;Imparidentia;Venerida;Veneroidea;Veneridae;Mercenaria;Mercenari
a mercenaria
UdeM00000035083 6596 163.3 UdeM00000035083 XP_045204673.1 38.1 194
119 1 1 194 284 476 3.10E-37 163.3 6596 cellular
organisms;Eukaryota;Opisthokonta;Metazoa;Eumetazoa;Bilateria;Protostomia;
Spiralia;Lophotrochozoa;Mollusca;Bivalvia;Autobranchia;Heteroconchia;Euhe
terodonta;Imparidentia;Venerida;Veneroidea;Veneridae;Mercenaria;Mercenari
a mercenaria
UdeM00000035084 225164 355.5 UdeM00000035084 XP_009062152.1 53.3
323 142 3 2 319 367 685 6.90E-95 355.5 225164
cellular
organisms;Eukaryota;Opisthokonta;Metazoa;Eumetazoa;Bilateria;Protostomia;
Spiralia;Lophotrochozoa;Mollusca;Gastropoda;Patellogastropoda;Lottioidea;
Lottiidae;Lottia;Lottia gigantea
UdeM00000035086 6596 501.1 UdeM00000035086 XP_045204106.1 50.3 497
242 4 1 493 1 496 1.90E-138 501.1 6596 cellular
organisms;Eukaryota;Opisthokonta;Metazoa;Eumetazoa;Bilateria;Protostomia;
Spiralia;Lophotrochozoa;Mollusca;Bivalvia;Autobranchia;Heteroconchia;Euhe
terodonta;Imparidentia;Venerida;Veneroidea;Veneridae;Mercenaria;Mercenari
a mercenaria
UdeM00000035087 400727 366.7 UdeM00000035087 XP_025109728.1 53.9
330 143 3 21 345 346 671 3.30E-98 366.7 400727
cellular
organisms;Eukaryota;Opisthokonta;Metazoa;Eumetazoa;Bilateria;Protostomia;
Spiralia;Lophotrochozoa;Mollusca;Gastropoda;Caenogastropoda;Architaeniogl
ossa;Ampullarioidea;Ampullariidae;Pomacea;Pomacea canaliculata
UdeM00000035089 6596 376.7 UdeM00000035089 XP_045204106.1 55.2 330
145 2 15 341 14 343 3.10E-101 376.7 6596 cellular
organisms;Eukaryota;Opisthokonta;Metazoa;Eumetazoa;Bilateria;Protostomia;
Spiralia;Lophotrochozoa;Mollusca;Bivalvia;Autobranchia;Heteroconchia;Euhe
terodonta;Imparidentia;Venerida;Veneroidea;Veneridae;Mercenaria;Mercenari
a mercenaria

UdeM00000035090 6565 124.4 UdeM00000035090 XP_022321199.1 36.4 184
114 1 1 184 401 581 1.50E-25 124.4 6565 cellular
organisms;Eukaryota;Opisthokonta;Metazoa;Eumetazoa;Bilateria;Protostomia;
Spiralia;Lophotrochozoa;Mollusca;Bivalvia;Autobranchia;Pteriomorphia;Ostr
eida;Ostreoidea;Ostreidae;Crassostrea;Crassostrea virginica
UdeM00000035091 6596 256.9 UdeM00000035091 XP_045204176.1 53.1 245
107 2 30 268 2 244 3.30E-65 256.9 6596 cellular
organisms;Eukaryota;Opisthokonta;Metazoa;Eumetazoa;Bilateria;Protostomia;
Spiralia;Lophotrochozoa;Mollusca;Bivalvia;Autobranchia;Heteroconchia;Euhe
terodonta;Imparidentia;Venerida;Veneroidea;Veneridae;Mercenaria;Mercenari
a mercenaria
UdeM00000035092 6596 498.8 UdeM00000035092 XP_045204106.1 50.2 488
239 3 23 507 10 496 9.60E-138 498.8 6596 cellular
organisms;Eukaryota;Opisthokonta;Metazoa;Eumetazoa;Bilateria;Protostomia;
Spiralia;Lophotrochozoa;Mollusca;Bivalvia;Autobranchia;Heteroconchia;Euhe
terodonta;Imparidentia;Venerida;Veneroidea;Veneridae;Mercenaria;Mercenari
a mercenaria
UdeM00000035093 225164 363.6 UdeM00000035093 XP_009062152.1 54.3
328 141 3 73 395 362 685 3.10E-97 363.6 225164
cellular
organisms;Eukaryota;Opisthokonta;Metazoa;Eumetazoa;Bilateria;Protostomia;
Spiralia;Lophotrochozoa;Mollusca;Gastropoda;Patellogastropoda;Lottioidea;
Lottiidae;Lottia;Lottia gigantea
UdeM00000035094 6596 246.9 UdeM00000035094 XP_045208604.1 52.4 225
105 1 31 253 2 226 2.80E-62 246.9 6596 cellular
organisms;Eukaryota;Opisthokonta;Metazoa;Eumetazoa;Bilateria;Protostomia;
Spiralia;Lophotrochozoa;Mollusca;Bivalvia;Autobranchia;Heteroconchia;Euhe
terodonta;Imparidentia;Venerida;Veneroidea;Veneridae;Mercenaria;Mercenari
a mercenaria
UdeM00000035095 6565 130.6 UdeM00000035095 XP_022326993.1 36.4 198
116 3 3 196 390 581 2.20E-27 130.6 6565 cellular
organisms;Eukaryota;Opisthokonta;Metazoa;Eumetazoa;Bilateria;Protostomia;
Spiralia;Lophotrochozoa;Mollusca;Bivalvia;Autobranchia;Pteriomorphia;Ostr
eida;Ostreoidea;Ostreidae;Crassostrea;Crassostrea virginica
UdeM00000035096 326594 93.6 UdeM00000035096 XP_011496778.1 46.4
110 52 2 43 147 582 689 2.30E-16 93.6 326594
cellular
organisms;Eukaryota;Opisthokonta;Metazoa;Eumetazoa;Bilateria;Protostomia;
Ecdysozoa;Panarthropoda;Arthropoda;Mandibulata;Pancrustacea;Hexapoda;Inse
cta;Dicondylia;Pterygota;Neoptera;Endopterygota;Hymenoptera;Apocrita;Para
sitoida;Chalcidoidea;Agaonidae;Agaoninae;Ceratosolen;Ceratosolen
solmsi;Ceratosolen solmsi marchali
UdeM00000035097 225164 275.4 UdeM00000035097 XP_009062152.1 59.4
207 84 0 23 229 362 568 6.60E-71 275.4 225164
cellular
organisms;Eukaryota;Opisthokonta;Metazoa;Eumetazoa;Bilateria;Protostomia;
Spiralia;Lophotrochozoa;Mollusca;Gastropoda;Patellogastropoda;Lottioidea;
Lottiidae;Lottia;Lottia gigantea
UdeM00000035099 6596 488 UdeM00000035099 XP_045204674.1 43.5 581
318 8 12 590 4 576 1.70E-134 488 6596 cellular
organisms;Eukaryota;Opisthokonta;Metazoa;Eumetazoa;Bilateria;Protostomia;
Spiralia;Lophotrochozoa;Mollusca;Bivalvia;Autobranchia;Heteroconchia;Euhe
terodonta;Imparidentia;Venerida;Veneroidea;Veneridae;Mercenaria;Mercenari
a mercenaria

UdeM00000029046 6596 795.4 UdeM00000029046 XP_045192328.1 70 544
157 4 1 540 1 542 6.70E-227 795.4 6596 cellular
organisms;Eukaryota;Opisthokonta;Metazoa;Eumetazoa;Bilateria;Protostomia;
Spiralia;Lophotrochozoa;Mollusca;Bivalvia;Autobranchia;Heteroconchia;Euhe
terodonta;Imparidentia;Venerida;Veneroidea;Veneridae;Mercenaria;Mercenari
a mercenaria
UdeM00000029047 6596 800.4 UdeM00000029047 XP_045192328.1 70.2 543
157 3 1 539 1 542 2.10E-228 800.4 6596 cellular
organisms;Eukaryota;Opisthokonta;Metazoa;Eumetazoa;Bilateria;Protostomia;
Spiralia;Lophotrochozoa;Mollusca;Bivalvia;Autobranchia;Heteroconchia;Euhe
terodonta;Imparidentia;Venerida;Veneroidea;Veneridae;Mercenaria;Mercenari
a mercenaria
UdeM00000029048 6573 96.3 UdeM00000029048 XP_021369989.1 61.7 81
26 2 1 77 323 402 8.20E-17 96.3 6573 cellular
organisms;Eukaryota;Opisthokonta;Metazoa;Eumetazoa;Bilateria;Protostomia;
Spiralia;Lophotrochozoa;Mollusca;Bivalvia;Autobranchia;Pteriomorphia;Pect
inida;Pectinoidea;Pectinidae;Mizuhopecten;Mizuhopecten yessoensis
UdeM00000029049 6596 64.3 UdeM00000029049 XP_045177042.1 37.9 87
50 3 1 85 29 113 1.10E-07 64.3 6596 cellular
organisms;Eukaryota;Opisthokonta;Metazoa;Eumetazoa;Bilateria;Protostomia;
Spiralia;Lophotrochozoa;Mollusca;Bivalvia;Autobranchia;Heteroconchia;Euhe
terodonta;Imparidentia;Venerida;Veneroidea;Veneridae;Mercenaria;Mercenari
a mercenaria
UdeM00000029050 1735272 458.8 UdeM00000029050 XP_041348317.1 56.6
392 169 1 6 397 148 538 7.50E-126 458.8 1735272
cellular
organisms;Eukaryota;Opisthokonta;Metazoa;Eumetazoa;Bilateria;Protostomia;
Spiralia;Lophotrochozoa;Mollusca;Gastropoda;Neomphalina;Neomphaloidea;Pel
tospiridae;Gigantopelta;Gigantopelta aegis
UdeM00000029051 6573 76.3 UdeM00000029051 XP_021370979.1 35.7 126
72 2 1 124 1 119 4.70E-11 76.3 6573 cellular
organisms;Eukaryota;Opisthokonta;Metazoa;Eumetazoa;Bilateria;Protostomia;
Spiralia;Lophotrochozoa;Mollusca;Bivalvia;Autobranchia;Pteriomorphia;Pect
inida;Pectinoidea;Pectinidae;Mizuhopecten;Mizuhopecten yessoensis
UdeM00000029052 6573 504.6 UdeM00000029052 XP_021370979.1 31.2 1167
617 29 1 1141 510 1516 3.30E-139 504.6 6573 cellular
organisms;Eukaryota;Opisthokonta;Metazoa;Eumetazoa;Bilateria;Protostomia;
Spiralia;Lophotrochozoa;Mollusca;Bivalvia;Autobranchia;Pteriomorphia;Pect
inida;Pectinoidea;Pectinidae;Mizuhopecten;Mizuhopecten yessoensis
UdeM00000029053 1735272 132.5 UdeM00000029053 XP_041350107.1 41
188 65 7 2 187 72 215 3.30E-27 132.5 1735272
cellular
organisms;Eukaryota;Opisthokonta;Metazoa;Eumetazoa;Bilateria;Protostomia;
Spiralia;Lophotrochozoa;Mollusca;Gastropoda;Neomphalina;Neomphaloidea;Pel
tospiridae;Gigantopelta;Gigantopelta aegis
UdeM00000029054 1735272 381.3 UdeM00000029054 XP_041348497.1 38.1
557 330 6 395 947 503 1048 3.50E-102 381.3 1735272
cellular
organisms;Eukaryota;Opisthokonta;Metazoa;Eumetazoa;Bilateria;Protostomia;
Spiralia;Lophotrochozoa;Mollusca;Gastropoda;Neomphalina;Neomphaloidea;Pel
tospiridae;Gigantopelta;Gigantopelta aegis
UdeM00000029056 6596 229.9 UdeM00000029056 XP_045164978.1 44.8 391
145 8 100 423 88 474 5.90E-57 229.9 6596 cellular
organisms;Eukaryota;Opisthokonta;Metazoa;Eumetazoa;Bilateria;Protostomia;

Spiralia;Lophotrochozoa;Mollusca;Bivalvia;Autobranchia;Heteroconchia;Euheterodonta;Imparidentia;Venerida;Veneroidea;Veneridae;Mercenaria;Mercenaria mercenaria
UdeM00000029057 6596 251.1 UdeM00000029057 XP_045164978.1 46.1 408 149 8 100 440 88 491 2.50E-63 251.1 6596 cellular organisms;Eukaryota;Opisthokonta;Metazoa;Eumetazoa;Bilateria;Protostomia;Spiralia;Lophotrochozoa;Mollusca;Bivalvia;Autobranchia;Heteroconchia;Euheterodonta;Imparidentia;Venerida;Veneroidea;Veneridae;Mercenaria;Mercenaria mercenaria
UdeM00000029058 29159 515.4 UdeM00000029058 XP_011414388.1 82.9 292 50 0 1 292 1 292 4.80E-143 515.4 29159 cellular organisms;Eukaryota;Opisthokonta;Metazoa;Eumetazoa;Bilateria;Protostomia;Spiralia;Lophotrochozoa;Mollusca;Bivalvia;Autobranchia;Pteriomorphia;Ostreida;Ostreoidea;Ostreidae;Crassostrea;Crassostrea gigas
UdeM00000029059 6596 404.8 UdeM00000029059 XP_045164764.1 71.7 290 72 2 1 289 1 281 9.00E-110 404.8 6596 cellular organisms;Eukaryota;Opisthokonta;Metazoa;Eumetazoa;Bilateria;Protostomia;Spiralia;Lophotrochozoa;Mollusca;Bivalvia;Autobranchia;Heteroconchia;Euheterodonta;Imparidentia;Venerida;Veneroidea;Veneridae;Mercenaria;Mercenaria mercenaria
UdeM00000029060 6596 573.2 UdeM00000029060 XP_045165956.1 68.2 400 126 1 48 446 46 445 3.00E-160 573.2 6596 cellular organisms;Eukaryota;Opisthokonta;Metazoa;Eumetazoa;Bilateria;Protostomia;Spiralia;Lophotrochozoa;Mollusca;Bivalvia;Autobranchia;Heteroconchia;Euheterodonta;Imparidentia;Venerida;Veneroidea;Veneridae;Mercenaria;Mercenaria mercenaria
UdeM00000029068 7739 58.5 UdeM00000029068 XP_035672209.1 55.3 47 20 1 23 69 505 550 9.50E-06 58.5 7739 cellular organisms;Eukaryota;Opisthokonta;Metazoa;Eumetazoa;Bilateria;Deuterostomia;Chordata;Cephalochordata;Leptocardii;Amphioxiformes;Branchiostomidae;Branchiostoma;Branchiostoma floridae
UdeM00000029069 6596 78.2 UdeM00000029069 XP_045164318.1 35.9 103 54 3 5 96 6 107 3.60E-11 78.2 6596 cellular organisms;Eukaryota;Opisthokonta;Metazoa;Eumetazoa;Bilateria;Protostomia;Spiralia;Lophotrochozoa;Mollusca;Bivalvia;Autobranchia;Heteroconchia;Euheterodonta;Imparidentia;Venerida;Veneroidea;Veneridae;Mercenaria;Mercenaria mercenaria
UdeM00000029070 6596 78.2 UdeM00000029070 XP_045164318.1 35.9 103 54 3 5 96 6 107 1.10E-11 78.2 6596 cellular organisms;Eukaryota;Opisthokonta;Metazoa;Eumetazoa;Bilateria;Protostomia;Spiralia;Lophotrochozoa;Mollusca;Bivalvia;Autobranchia;Heteroconchia;Euheterodonta;Imparidentia;Venerida;Veneroidea;Veneridae;Mercenaria;Mercenaria mercenaria
UdeM00000029071 6596 74.3 UdeM00000029071 XP_045164318.1 27.6 239 138 10 5 214 6 238 3.10E-10 74.3 6596 cellular organisms;Eukaryota;Opisthokonta;Metazoa;Eumetazoa;Bilateria;Protostomia;Spiralia;Lophotrochozoa;Mollusca;Bivalvia;Autobranchia;Heteroconchia;Euheterodonta;Imparidentia;Venerida;Veneroidea;Veneridae;Mercenaria;Mercenaria mercenaria
UdeM00000029074 6565 82.8 UdeM00000029074 XP_022302776.1 36.2 224 128 6 19 229 25 246 9.50E-13 82.8 6565 cellular organisms;Eukaryota;Opisthokonta;Metazoa;Eumetazoa;Bilateria;Protostomia;Spiralia;Lophotrochozoa;Mollusca;Bivalvia;Autobranchia;Pteriomorphia;Ostreida;Ostreoidea;Ostreidae;Crassostrea;Crassostrea virginica

UdeM00000029075 6596 70.5 UdeM00000029075 XP_045196479.1 40.9 88
48 2 4 91 109 192 1.30E-09 70.5 6596 cellular organisms;Eukaryota;Opisthokonta;Metazoa;Eumetazoa;Bilateria;Protostomia;Spiralia;Lophotrochozoa;Mollusca;Bivalvia;Autobranchia;Heteroconchia;Euheterodonta;Imparidentia;Venerida;Veneroidea;Veneridae;Mercenaria;Mercenaria mercenaria
UdeM00000029076 6596 289.3 UdeM00000029076 XP_045175277.1 45.8 319
162 3 26 333 11 329 6.40E-75 289.3 6596 cellular organisms;Eukaryota;Opisthokonta;Metazoa;Eumetazoa;Bilateria;Protostomia;Spiralia;Lophotrochozoa;Mollusca;Bivalvia;Autobranchia;Heteroconchia;Euheterodonta;Imparidentia;Venerida;Veneroidea;Veneridae;Mercenaria;Mercenaria mercenaria
UdeM00000029078 6596 248.4 UdeM00000029078 XP_045188938.1 43.9 280
155 2 4 282 20 298 1.10E-62 248.4 6596 cellular organisms;Eukaryota;Opisthokonta;Metazoa;Eumetazoa;Bilateria;Protostomia;Spiralia;Lophotrochozoa;Mollusca;Bivalvia;Autobranchia;Heteroconchia;Euheterodonta;Imparidentia;Venerida;Veneroidea;Veneridae;Mercenaria;Mercenaria mercenaria
UdeM00000029079 6596 203 UdeM00000029079 XP_045166785.1 40 290
158 10 62 344 39 319 6.30E-49 203 6596 cellular organisms;Eukaryota;Opisthokonta;Metazoa;Eumetazoa;Bilateria;Protostomia;Spiralia;Lophotrochozoa;Mollusca;Bivalvia;Autobranchia;Heteroconchia;Euheterodonta;Imparidentia;Venerida;Veneroidea;Veneridae;Mercenaria;Mercenaria mercenaria
UdeM00000029080 6596 271.2 UdeM00000029080 XP_045167324.1 31.6 561
313 17 59 570 41 579 3.40E-69 271.2 6596 cellular organisms;Eukaryota;Opisthokonta;Metazoa;Eumetazoa;Bilateria;Protostomia;Spiralia;Lophotrochozoa;Mollusca;Bivalvia;Autobranchia;Heteroconchia;Euheterodonta;Imparidentia;Venerida;Veneroidea;Veneridae;Mercenaria;Mercenaria mercenaria
UdeM00000029081 6596 548.5 UdeM00000029081 XP_045167324.1 35.5 941
539 28 59 962 41 950 1.70E-152 548.5 6596 cellular organisms;Eukaryota;Opisthokonta;Metazoa;Eumetazoa;Bilateria;Protostomia;Spiralia;Lophotrochozoa;Mollusca;Bivalvia;Autobranchia;Heteroconchia;Euheterodonta;Imparidentia;Venerida;Veneroidea;Veneridae;Mercenaria;Mercenaria mercenaria
UdeM00000029082 6596 542 UdeM00000029082 XP_045167324.1 34.6 940
550 26 31 935 41 950 1.50E-150 542 6596 cellular organisms;Eukaryota;Opisthokonta;Metazoa;Eumetazoa;Bilateria;Protostomia;Spiralia;Lophotrochozoa;Mollusca;Bivalvia;Autobranchia;Heteroconchia;Euheterodonta;Imparidentia;Venerida;Veneroidea;Veneridae;Mercenaria;Mercenaria mercenaria
UdeM00000029083 72036 196.4 UdeM00000029083 XP_040576246.1 30.7 469
181 9 3 335 1 461 5.70E-47 196.4 72036 cellular organisms;Eukaryota;Opisthokonta;Metazoa;Eumetazoa;Bilateria;Protostomia;Ecdysozoa;Panarthropoda;Arthropoda;Mandibulata;Pancrustacea;Crustacea;Multicrustacea;Hexanauplia;Copepoda;Neocopepoda;Podoplea;Siphonostomatoida;Caligidae;Lepeophtheirus;Lepeophtheirus salmonis
UdeM00000029084 6579 437.6 UdeM00000029084 XP_033727104.1 40.1 519
292 9 3 510 16 526 2.40E-119 437.6 6579 cellular organisms;Eukaryota;Opisthokonta;Metazoa;Eumetazoa;Bilateria;Protostomia;Spiralia;Lophotrochozoa;Mollusca;Bivalvia;Autobranchia;Pteriomorphia;Pectinida;Pectinoidea;Pectinidae;Pecten;Pecten maximus

UdeM00000029085 46731 82.4 UdeM00000029085 XP_027042029.1 40.5 121
68 2 25 144 32 149 5.90E-13 82.4 46731 cellular
organisms;Eukaryota;Opisthokonta;Metazoa;Eumetazoa;Cnidaria;Anthozoa;Hexacorallia;Scleractinia;Astrocoeniina;Pocilloporidae;Pocillopora;Pocillopora damicornis
UdeM00000029086 1735272 630.9 UdeM00000029086 XP_041374928.1 77.3
419 91 1 23 441 1078 1492 1.20E-177 630.9 1735272
cellular
organisms;Eukaryota;Opisthokonta;Metazoa;Eumetazoa;Bilateria;Protostomia;Spiralia;Lophotrochozoa;Mollusca;Gastropoda;Neomphalina;Neomphaloidea;Peltospiridae;Gigantopelta;Gigantopelta aegis
UdeM00000029089 151771 206.8 UdeM00000029089 XP_028415423.1 28.8
511 228 8 3 382 5 510 8.60E-50 206.8 151771
cellular
organisms;Eukaryota;Opisthokonta;Metazoa;Eumetazoa;Cnidaria;Anthozoa;Octocorallia;Alcyonacea;Alcyoniina;Nephtheidae;Dendronephthya;Dendronephthya gigantea
UdeM00000029090 6526 80.9 UdeM00000029090 XP_013062120.1 52.9 70
33 0 28 97 275 344 1.00E-12 80.9 6526 cellular
organisms;Eukaryota;Opisthokonta;Metazoa;Eumetazoa;Bilateria;Protostomia;Spiralia;Lophotrochozoa;Mollusca;Gastropoda;Heterobranchia;Euthyneura;Panpulmonata;Hygrophila;Lymnaeoidea;Planorbidae;Biomphalaria;Biomphalaria glabrata
UdeM00000029091 6105 74.3 UdeM00000029091 XP_031573684.1 41.1 112
59 2 179 290 45 149 2.80E-10 74.3 6105 cellular
organisms;Eukaryota;Opisthokonta;Metazoa;Eumetazoa;Cnidaria;Anthozoa;Hexacorallia;Actiniaria;Actiniidae;Actinia;Actinia tenebrosa
UdeM00000029093 2607531 70.5 UdeM00000029093 XP_029643184.1 34.3
137 78 3 1 134 1 128 2.90E-09 70.5 2607531
cellular
organisms;Eukaryota;Opisthokonta;Metazoa;Eumetazoa;Bilateria;Protostomia;Spiralia;Lophotrochozoa;Mollusca;Cephalopoda;Coleoidea;Octopodiformes;Octopoda;Incirrata;Octopodidae;Octopus;Octopus sinensis
UdeM00000029094 29159 173.3 UdeM00000029094 XP_011416080.2 75.8 99
24 0 13 111 4 102 1.70E-40 173.3 29159 cellular
organisms;Eukaryota;Opisthokonta;Metazoa;Eumetazoa;Bilateria;Protostomia;Spiralia;Lophotrochozoa;Mollusca;Bivalvia;Autobranchia;Pteriomorphia;Ostreida;Ostreoidea;Ostreidae;Crassostrea;Crassostrea gigas
UdeM00000029095 6573 86.7 UdeM00000029095 XP_021371694.1 34.9 195
84 7 58 225 34 212 4.30E-14 86.7 6573 cellular
organisms;Eukaryota;Opisthokonta;Metazoa;Eumetazoa;Bilateria;Protostomia;Spiralia;Lophotrochozoa;Mollusca;Bivalvia;Autobranchia;Pteriomorphia;Pectinida;Pectinoidea;Pectinidae;Mizuhopecten;Mizuhopecten yessoensis
UdeM00000029096 6573 86.7 UdeM00000029096 XP_021371694.1 34.9 195
84 7 25 192 34 212 3.70E-14 86.7 6573 cellular
organisms;Eukaryota;Opisthokonta;Metazoa;Eumetazoa;Bilateria;Protostomia;Spiralia;Lophotrochozoa;Mollusca;Bivalvia;Autobranchia;Pteriomorphia;Pectinida;Pectinoidea;Pectinidae;Mizuhopecten;Mizuhopecten yessoensis
UdeM00000029097 6596 58.2 UdeM00000029097 XP_045206071.1 26.2 141
75 2 2 113 184 324 9.40E-06 58.2 6596 cellular
organisms;Eukaryota;Opisthokonta;Metazoa;Eumetazoa;Bilateria;Protostomia;Spiralia;Lophotrochozoa;Mollusca;Bivalvia;Autobranchia;Heteroconchia;Euheterodonta;Imparidentia;Venerida;Veneroidea;Veneridae;Mercenaria;Mercenaria mercenaria

UdeM00000029098	6596	209.1	UdeM00000029098	XP_045172484.1	23.6	694
	462	17	62	731	74	723	1.80E-50	209.1	6596	cellular
organisms;Eukaryota;Opisthokonta;Metazoa;Eumetazoa;Bilateria;Protostomia;
Spiralia;Lophotrochozoa;Mollusca;Bivalvia;Autobranchia;Heteroconchia;Euhe
terodonta;Imparidentia;Venerida;Veneroidea;Veneridae;Mercenaria;Mercenari
a mercenaria
UdeM00000029099	6596	505.8	UdeM00000029099	XP_045165614.1	42.1	675
	309	12	47	705	14	622	9.10E-140	505.8	6596	cellular
organisms;Eukaryota;Opisthokonta;Metazoa;Eumetazoa;Bilateria;Protostomia;
Spiralia;Lophotrochozoa;Mollusca;Bivalvia;Autobranchia;Heteroconchia;Euhe
terodonta;Imparidentia;Venerida;Veneroidea;Veneridae;Mercenaria;Mercenari
a mercenaria
UdeM00000029100	29159	77.8	UdeM00000029100	XP_034305523.1	54.5	66
	30	0	1	66	794	859	7.10E-12	77.8	29159	cellular
organisms;Eukaryota;Opisthokonta;Metazoa;Eumetazoa;Bilateria;Protostomia;
Spiralia;Lophotrochozoa;Mollusca;Bivalvia;Autobranchia;Pteriomorphia;Ostr
eida;Ostreoidea;Ostreidae;Crassostrea;Crassostrea gigas
UdeM00000029101	6579	402.1	UdeM00000029101	XP_033739131.1	62.2	294
	111	0	1	294	1077	1370	6.00E-109	402.1	6579	cellular
organisms;Eukaryota;Opisthokonta;Metazoa;Eumetazoa;Bilateria;Protostomia;
Spiralia;Lophotrochozoa;Mollusca;Bivalvia;Autobranchia;Pteriomorphia;Pect
inida;Pectinoidea;Pectinidae;Pecten;Pecten maximus
UdeM00000029102	29159	130.2	UdeM00000029102	XP_034307199.1	76.5	81
	17	1	117	195	103	183	2.90E-27	130.2	29159	cellular
organisms;Eukaryota;Opisthokonta;Metazoa;Eumetazoa;Bilateria;Protostomia;
Spiralia;Lophotrochozoa;Mollusca;Bivalvia;Autobranchia;Pteriomorphia;Ostr
eida;Ostreoidea;Ostreidae;Crassostrea;Crassostrea gigas
UdeM00000029103	6596	664.1	UdeM00000029103	XP_045164809.1	84.7	366
	54	1	114	477	147	512	1.40E-187	664.1	6596	cellular
organisms;Eukaryota;Opisthokonta;Metazoa;Eumetazoa;Bilateria;Protostomia;
Spiralia;Lophotrochozoa;Mollusca;Bivalvia;Autobranchia;Heteroconchia;Euhe
terodonta;Imparidentia;Venerida;Veneroidea;Veneridae;Mercenaria;Mercenari
a mercenaria
UdeM00000029104	29159	357.5	UdeM00000029104	XP_034305429.1	59.6	401
	160	2	1	399	1	401	2.30E-95	357.5	29159	cellular
organisms;Eukaryota;Opisthokonta;Metazoa;Eumetazoa;Bilateria;Protostomia;
Spiralia;Lophotrochozoa;Mollusca;Bivalvia;Autobranchia;Pteriomorphia;Ostr
eida;Ostreoidea;Ostreidae;Crassostrea;Crassostrea gigas
UdeM00000029105	80427	118.2	UdeM00000029105	XP_032998211.1	35.5	197
	109	4	575	766	367	550	4.50E-23	118.2	80427	cellular
organisms;Eukaryota;Opisthokonta;Metazoa;Eumetazoa;Bilateria;Deuterostomi
a;Chordata;Craniata;Vertebrata;Gnathostomata;Teleostomi;Euteleostomi;Sarc
opterygii;Dipnotetrapodomorpha;Tetrapoda;Amniota;Sauropsida;Sauria;Lepido
sauria;Squamata;Bifurcata;Unidentata;Episquamata;Laterata;Lacertibaenia;L
acertidae;Lacertinae;Lacerta;Lacerta agilis
UdeM00000029106	6596	234.6	UdeM00000029106	XP_045169782.1	47.6	233
	121	1	1	232	56	288	1.30E-58	234.6	6596	cellular
organisms;Eukaryota;Opisthokonta;Metazoa;Eumetazoa;Bilateria;Protostomia;
Spiralia;Lophotrochozoa;Mollusca;Bivalvia;Autobranchia;Heteroconchia;Euhe
terodonta;Imparidentia;Venerida;Veneroidea;Veneridae;Mercenaria;Mercenari
a mercenaria
UdeM00000029107	6526	557.8	UdeM00000029107	XP_013085681.1	76.8	354
	81	1	1	353	20	373	1.00E-155	557.8	6526	cellular
organisms;Eukaryota;Opisthokonta;Metazoa;Eumetazoa;Bilateria;Protostomia;

Spiralia;Lophotrochozoa;Mollusca;Gastropoda;Heterobranchia;Euthyneura;Pan pulmonata;Hygrophila;Lymnaeoidea;Planorbidae;Biomphalaria;Biomphalaria glabrata
UdeM00000029108 6565 14710.4 UdeM00000029108 XP_022297807.1 44.2 21403 11001 239 5 20953 12 20917 0.00E+00 14710.4 6565 cellular organisms;Eukaryota;Opisthokonta;Metazoa;Eumetazoa;Bilateria;Protostomia; Spiralia;Lophotrochozoa;Mollusca;Bivalvia;Autobranchia;Pteriomorphia;Ostr eida;Ostreoidea;Ostreidae;Crassostrea;Crassostrea virginica
UdeM00000029109 6565 14745.8 UdeM00000029109 XP_022297807.1 44.2 21403 11001 238 5 20968 12 20917 0.00E+00 14745.8 6565 cellular organisms;Eukaryota;Opisthokonta;Metazoa;Eumetazoa;Bilateria;Protostomia; Spiralia;Lophotrochozoa;Mollusca;Bivalvia;Autobranchia;Pteriomorphia;Ostr eida;Ostreoidea;Ostreidae;Crassostrea;Crassostrea virginica
UdeM00000029110 6596 132.5 UdeM00000029110 XP_045164347.1 34.1 270 155 10 59 318 41 297 1.20E-27 132.5 6596 cellular organisms;Eukaryota;Opisthokonta;Metazoa;Eumetazoa;Bilateria;Protostomia; Spiralia;Lophotrochozoa;Mollusca;Bivalvia;Autobranchia;Heteroconchia;Euhe terodonta;Imparidentia;Venerida;Veneroidea;Veneridae;Mercenaria;Mercenari a mercenaria
UdeM00000029113 6596 429.5 UdeM00000029113 XP_045167324.1 36.2 674 370 18 40 668 292 950 7.90E-117 429.5 6596 cellular organisms;Eukaryota;Opisthokonta;Metazoa;Eumetazoa;Bilateria;Protostomia; Spiralia;Lophotrochozoa;Mollusca;Bivalvia;Autobranchia;Heteroconchia;Euhe terodonta;Imparidentia;Venerida;Veneroidea;Veneridae;Mercenaria;Mercenari a mercenaria
UdeM00000029114 6596 143.3 UdeM00000029114 XP_045167324.1 34.2 199 121 3 64 252 371 569 4.30E-31 143.3 6596 cellular organisms;Eukaryota;Opisthokonta;Metazoa;Eumetazoa;Bilateria;Protostomia; Spiralia;Lophotrochozoa;Mollusca;Bivalvia;Autobranchia;Heteroconchia;Euhe terodonta;Imparidentia;Venerida;Veneroidea;Veneridae;Mercenaria;Mercenari a mercenaria
UdeM00000029115 1735272 212.6 UdeM00000029115 XP_041378881.1 38 363 171 14 5 332 603 946 7.80E-52 212.6 1735272 cellular organisms;Eukaryota;Opisthokonta;Metazoa;Eumetazoa;Bilateria;Protostomia; Spiralia;Lophotrochozoa;Mollusca;Gastropoda;Neomphalina;Neomphaloidea;Pel tospiridae;Gigantopelta;Gigantopelta aegis
UdeM00000029116 1735272 228 UdeM00000029116 XP_041378881.1 38.8 363 174 13 5 338 603 946 1.80E-56 228 1735272 cellular organisms;Eukaryota;Opisthokonta;Metazoa;Eumetazoa;Bilateria;Protostomia; Spiralia;Lophotrochozoa;Mollusca;Gastropoda;Neomphalina;Neomphaloidea;Pel tospiridae;Gigantopelta;Gigantopelta aegis
UdeM00000029117 6565 63.5 UdeM00000029117 XP_022302312.1 34.7 75 49 0 2 76 22 96 1.30E-07 63.5 6565 cellular organisms;Eukaryota;Opisthokonta;Metazoa;Eumetazoa;Bilateria;Protostomia; Spiralia;Lophotrochozoa;Mollusca;Bivalvia;Autobranchia;Pteriomorphia;Ostr eida;Ostreoidea;Ostreidae;Crassostrea;Crassostrea virginica
UdeM00000029119 225164 201.8 UdeM00000029119 XP_009044734.1 73.5 136 28 3 1 132 14 145 5.80E-49 201.8 225164 cellular organisms;Eukaryota;Opisthokonta;Metazoa;Eumetazoa;Bilateria;Protostomia;

Spiralia;Lophotrochozoa;Mollusca;Gastropoda;Patellogastropoda;Lottioidea;
Lottiidae;Lottia;Lottia gigantea
UdeM00000029118 225164 293.9 UdeM00000029118 XP_009044734.1 75.3
186 38 3 1 182 14 195 1.90E-76 293.9 225164
cellular
organisms;Eukaryota;Opisthokonta;Metazoa;Eumetazoa;Bilateria;Protostomia;
Spiralia;Lophotrochozoa;Mollusca;Gastropoda;Patellogastropoda;Lottioidea;
Lottiidae;Lottia;Lottia gigantea
UdeM00000029120 6596 109 UdeM00000029120 XP_045165989.1 31.7 186
118 6 69 250 35 215 1.60E-20 109 6596 cellular
organisms;Eukaryota;Opisthokonta;Metazoa;Eumetazoa;Bilateria;Protostomia;
Spiralia;Lophotrochozoa;Mollusca;Bivalvia;Autobranchia;Heteroconchia;Euhe
terodonta;Imparidentia;Venerida;Veneroidea;Veneridae;Mercenaria;Mercenari
a mercenaria
UdeM00000029121 6596 107.1 UdeM00000029121 XP_045165989.1 30.5 210
134 7 309 512 38 241 6.90E-20 107.1 6596 cellular
organisms;Eukaryota;Opisthokonta;Metazoa;Eumetazoa;Bilateria;Protostomia;
Spiralia;Lophotrochozoa;Mollusca;Bivalvia;Autobranchia;Heteroconchia;Euhe
terodonta;Imparidentia;Venerida;Veneroidea;Veneridae;Mercenaria;Mercenari
a mercenaria
UdeM00000029122 1735272 204.5 UdeM00000029122 XP_041348725.1 57.9
195 63 4 9 200 296 474 1.20E-49 204.5 1735272
cellular
organisms;Eukaryota;Opisthokonta;Metazoa;Eumetazoa;Bilateria;Protostomia;
Spiralia;Lophotrochozoa;Mollusca;Gastropoda;Neomphalina;Neomphaloidea;Pel
tospiridae;Gigantopelta;Gigantopelta aegis
UdeM00000029123 7574 221.1 UdeM00000029123 XP_013408784.1 50.5 220
67 1 47 224 127 346 1.40E-54 221.1 7574 cellular
organisms;Eukaryota;Opisthokonta;Metazoa;Eumetazoa;Bilateria;Protostomia;
Spiralia;Lophotrochozoa;Brachiopoda;Linguliformea;Lingulata;Lingulida;Lin
guloidea;Lingulidae;Lingula;Lingula anatina
UdeM00000029125 6573 499.6 UdeM00000029125 XP_021371790.1 78 304
67 0 22 325 96 399 3.10E-138 499.6 6573 cellular
organisms;Eukaryota;Opisthokonta;Metazoa;Eumetazoa;Bilateria;Protostomia;
Spiralia;Lophotrochozoa;Mollusca;Bivalvia;Autobranchia;Pteriomorphia;Pect
inida;Pectinoidea;Pectinidae;Mizuhopecten;Mizuhopecten yessoensis
UdeM00000029124 6596 529.3 UdeM00000029124 XP_045165994.1 69.8 374
108 1 17 385 89 462 1.30E-146 529.3 6596 cellular
organisms;Eukaryota;Opisthokonta;Metazoa;Eumetazoa;Bilateria;Protostomia;
Spiralia;Lophotrochozoa;Mollusca;Bivalvia;Autobranchia;Heteroconchia;Euhe
terodonta;Imparidentia;Venerida;Veneroidea;Veneridae;Mercenaria;Mercenari
a mercenaria
UdeM00000029126 6573 233.8 UdeM00000029126 XP_021371790.1 69.4 160
48 1 19 177 277 436 9.70E-58 233.8 6573 cellular
organisms;Eukaryota;Opisthokonta;Metazoa;Eumetazoa;Bilateria;Protostomia;
Spiralia;Lophotrochozoa;Mollusca;Bivalvia;Autobranchia;Pteriomorphia;Pect
inida;Pectinoidea;Pectinidae;Mizuhopecten;Mizuhopecten yessoensis
UdeM00000029127 6596 807.4 UdeM00000029127 XP_045167278.1 57.7 664
278 2 1 662 10 672 1.40E-230 807.4 6596 cellular
organisms;Eukaryota;Opisthokonta;Metazoa;Eumetazoa;Bilateria;Protostomia;
Spiralia;Lophotrochozoa;Mollusca;Bivalvia;Autobranchia;Heteroconchia;Euhe
terodonta;Imparidentia;Venerida;Veneroidea;Veneridae;Mercenaria;Mercenari
a mercenaria

UdeM00000029128 6573 815.1 UdeM00000029128 XP_021364165.1 68 609
167 8 1 597 1 593 6.00E-233 815.1 6573 cellular
organisms;Eukaryota;Opisthokonta;Metazoa;Eumetazoa;Bilateria;Protostomia;Spiralia;Lophotrochozoa;Mollusca;Bivalvia;Autobranchia;Pteriomorphia;Pectinida;Pectinoidea;Pectinidae;Mizuhopecten;Mizuhopecten yessoensis
UdeM00000029129 6579 630.2 UdeM00000029129 XP_033745226.1 44.7 844
378 20 1 803 1 796 3.70E-177 630.2 6579 cellular
organisms;Eukaryota;Opisthokonta;Metazoa;Eumetazoa;Bilateria;Protostomia;Spiralia;Lophotrochozoa;Mollusca;Bivalvia;Autobranchia;Pteriomorphia;Pectinida;Pectinoidea;Pectinidae;Pecten;Pecten maximus
UdeM00000029130 1735272 221.5 UdeM00000029130 XP_041347818.1 40.5
326 185 4 66 384 69 392 1.90E-54 221.5 1735272
cellular
organisms;Eukaryota;Opisthokonta;Metazoa;Eumetazoa;Bilateria;Protostomia;Spiralia;Lophotrochozoa;Mollusca;Gastropoda;Neomphalina;Neomphaloidea;Peltospiridae;Gigantopelta;Gigantopelta aegis
UdeM00000029131 400727 257.3 UdeM00000029131 XP_025106490.1 81.9
149 27 0 1 149 1 149 1.20E-65 257.3 400727
cellular
organisms;Eukaryota;Opisthokonta;Metazoa;Eumetazoa;Bilateria;Protostomia;Spiralia;Lophotrochozoa;Mollusca;Gastropoda;Caenogastropoda;Architaenioglossa;Ampullarioidea;Ampullariidae;Pomacea;Pomacea canaliculata
UdeM00000029132 37653 168.7 UdeM00000029132 XP_014782898.1 72.6 106
26 1 17 119 331 436 6.40E-39 168.7 37653 cellular
organisms;Eukaryota;Opisthokonta;Metazoa;Eumetazoa;Bilateria;Protostomia;Spiralia;Lophotrochozoa;Mollusca;Cephalopoda;Coleoidea;Octopodiformes;Octopoda;Incirrata;Octopodidae;Octopus;Octopus bimaculoides
UdeM00000029133 29159 411.8 UdeM00000029133 XP_011446959.2 51.2 555
202 16 34 528 163 708 1.40E-111 411.8 29159 cellular
organisms;Eukaryota;Opisthokonta;Metazoa;Eumetazoa;Bilateria;Protostomia;Spiralia;Lophotrochozoa;Mollusca;Bivalvia;Autobranchia;Pteriomorphia;Ostreida;Ostreoidea;Ostreidae;Crassostrea;Crassostrea gigas
UdeM00000029134 1735272 334.3 UdeM00000029134 XP_041347144.1 64.1
231 83 0 1 231 1 231 1.20E-88 334.3 1735272
cellular
organisms;Eukaryota;Opisthokonta;Metazoa;Eumetazoa;Bilateria;Protostomia;Spiralia;Lophotrochozoa;Mollusca;Gastropoda;Neomphalina;Neomphaloidea;Peltospiridae;Gigantopelta;Gigantopelta aegis
UdeM00000029135 6579 775.8 UdeM00000029135 XP_033744943.1 80.5 488
74 7 1 481 1 474 3.20E-221 775.8 6579 cellular
organisms;Eukaryota;Opisthokonta;Metazoa;Eumetazoa;Bilateria;Protostomia;Spiralia;Lophotrochozoa;Mollusca;Bivalvia;Autobranchia;Pteriomorphia;Pectinida;Pectinoidea;Pectinidae;Pecten;Pecten maximus
UdeM00000029136 6596 376.7 UdeM00000029136 XP_045166545.1 59.1 291
117 1 9 297 3 293 2.80E-101 376.7 6596 cellular
organisms;Eukaryota;Opisthokonta;Metazoa;Eumetazoa;Bilateria;Protostomia;Spiralia;Lophotrochozoa;Mollusca;Bivalvia;Autobranchia;Heteroconchia;Euheterodonta;Imparidentia;Venerida;Veneroidea;Veneridae;Mercenaria;Mercenaria mercenaria
UdeM00000029137 6596 960.7 UdeM00000029137 XP_045167176.1 43.8 1269
649 16 1 1226 1 1248 1.80E-276 960.7 6596 cellular
organisms;Eukaryota;Opisthokonta;Metazoa;Eumetazoa;Bilateria;Protostomia;Spiralia;Lophotrochozoa;Mollusca;Bivalvia;Autobranchia;Heteroconchia;Euhe

terodonta;Imparidentia;Venerida;Veneroidea;Veneridae;Mercenaria;Mercenaria mercenaria
UdeM00000029138 6596 466.8 UdeM00000029138 XP_045167176.1 36.8 1325 792 14 1 1307 1275 2571 9.20E-128 466.8 6596 cellular organisms;Eukaryota;Opisthokonta;Metazoa;Eumetazoa;Bilateria;Protostomia;Spiralia;Lophotrochozoa;Mollusca;Bivalvia;Autobranchia;Heteroconchia;Euheterodonta;Imparidentia;Venerida;Veneroidea;Veneridae;Mercenaria;Mercenaria mercenaria
UdeM00000029139 6596 250 UdeM00000029139 XP_045165356.1 67.7 186 55 3 1 185 1 182 2.50E-63 250 6596 cellular organisms;Eukaryota;Opisthokonta;Metazoa;Eumetazoa;Bilateria;Protostomia;Spiralia;Lophotrochozoa;Mollusca;Bivalvia;Autobranchia;Heteroconchia;Euheterodonta;Imparidentia;Venerida;Veneroidea;Veneridae;Mercenaria;Mercenaria mercenaria
UdeM00000029140 6596 526.6 UdeM00000029140 XP_045165355.1 42.6 779 358 19 1 721 1 748 5.50E-146 526.6 6596 cellular organisms;Eukaryota;Opisthokonta;Metazoa;Eumetazoa;Bilateria;Protostomia;Spiralia;Lophotrochozoa;Mollusca;Bivalvia;Autobranchia;Heteroconchia;Euheterodonta;Imparidentia;Venerida;Veneroidea;Veneridae;Mercenaria;Mercenaria mercenaria
UdeM00000029141 6596 906 UdeM00000029141 XP_045165369.1 82.7 566 91 6 1 564 1 561 2.40E-260 906 6596 cellular organisms;Eukaryota;Opisthokonta;Metazoa;Eumetazoa;Bilateria;Protostomia;Spiralia;Lophotrochozoa;Mollusca;Bivalvia;Autobranchia;Heteroconchia;Euheterodonta;Imparidentia;Venerida;Veneroidea;Veneridae;Mercenaria;Mercenaria mercenaria
UdeM00000029142 6573 292 UdeM00000029142 XP_021344022.1 61.3 230 80 3 2 222 3 232 6.60E-76 292 6573 cellular organisms;Eukaryota;Opisthokonta;Metazoa;Eumetazoa;Bilateria;Protostomia;Spiralia;Lophotrochozoa;Mollusca;Bivalvia;Autobranchia;Pteriomorphia;Pectinida;Pectinoidea;Pectinidae;Mizuhopecten;Mizuhopecten yessoensis
UdeM00000029143 6573 444.9 UdeM00000029143 XP_021343957.1 73 281 76 0 6 286 8 288 7.80E-122 444.9 6573 cellular organisms;Eukaryota;Opisthokonta;Metazoa;Eumetazoa;Bilateria;Protostomia;Spiralia;Lophotrochozoa;Mollusca;Bivalvia;Autobranchia;Pteriomorphia;Pectinida;Pectinoidea;Pectinidae;Mizuhopecten;Mizuhopecten yessoensis
UdeM00000029144 6596 239.2 UdeM00000029144 XP_045164817.1 36.5 493 263 13 10 471 6 479 1.10E-59 239.2 6596 cellular organisms;Eukaryota;Opisthokonta;Metazoa;Eumetazoa;Bilateria;Protostomia;Spiralia;Lophotrochozoa;Mollusca;Bivalvia;Autobranchia;Heteroconchia;Euheterodonta;Imparidentia;Venerida;Veneroidea;Veneridae;Mercenaria;Mercenaria mercenaria
UdeM00000029145 6565 149.8 UdeM00000029145 XP_022296688.1 50.7 138 58 2 1 138 36 163 2.50E-33 149.8 6565 cellular organisms;Eukaryota;Opisthokonta;Metazoa;Eumetazoa;Bilateria;Protostomia;Spiralia;Lophotrochozoa;Mollusca;Bivalvia;Autobranchia;Pteriomorphia;Ostreida;Ostreoidea;Ostreidae;Crassostrea;Crassostrea virginica
UdeM00000029146 6689 186.8 UdeM00000029146 XP_027207501.1 49.2 177 89 1 46 222 1826 2001 3.00E-44 186.8 6689 cellular organisms;Eukaryota;Opisthokonta;Metazoa;Eumetazoa;Bilateria;Protostomia;Ecdysozoa;Panarthropoda;Arthropoda;Mandibulata;Pancrustacea;Crustacea;Multicrustacea;Malacostraca;Eumalacostraca;Eucarida;Decapoda;Dendrobranchiata;Penaeoidea;Penaeidae;Penaeus;Penaeus vannamei

UdeM00000043526 1735272 125.6 UdeM00000043526 XP_041366888.1 24.6 475 284 14 13 429 29 487 1.60E-25 125.6 1735272 cellular organisms;Eukaryota;Opisthokonta;Metazoa;Eumetazoa;Bilateria;Protostomia;Spiralia;Lophotrochozoa;Mollusca;Gastropoda;Neomphalina;Neomphaloidea;Peltospiridae;Gigantopelta;Gigantopelta aegis
UdeM00000043527 6596 197.2 UdeM00000043527 XP_045199754.1 41.5 337 144 8 1 289 1 332 2.90E-47 197.2 6596 cellular organisms;Eukaryota;Opisthokonta;Metazoa;Eumetazoa;Bilateria;Protostomia;Spiralia;Lophotrochozoa;Mollusca;Bivalvia;Autobranchia;Heteroconchia;Euheterodonta;Imparidentia;Venerida;Veneroidea;Veneridae;Mercenaria;Mercenaria mercenaria
UdeM00000043528 6596 266.9 UdeM00000043528 XP_045199754.1 46.7 338 151 7 1 317 1 330 3.20E-68 266.9 6596 cellular organisms;Eukaryota;Opisthokonta;Metazoa;Eumetazoa;Bilateria;Protostomia;Spiralia;Lophotrochozoa;Mollusca;Bivalvia;Autobranchia;Heteroconchia;Euheterodonta;Imparidentia;Venerida;Veneroidea;Veneridae;Mercenaria;Mercenaria mercenaria
UdeM00000043529 6565 76.6 UdeM00000043529 XP_022341888.1 48.6 72 37 0 15 86 812 883 6.20E-11 76.6 6565 cellular organisms;Eukaryota;Opisthokonta;Metazoa;Eumetazoa;Bilateria;Protostomia;Spiralia;Lophotrochozoa;Mollusca;Bivalvia;Autobranchia;Pteriomorphia;Ostreida;Ostreoidea;Ostreidae;Crassostrea;Crassostrea virginica
UdeM00000043530 6565 184.1 UdeM00000043530 XP_022299727.1 45.9 196 105 1 5 200 83 277 2.00E-43 184.1 6565 cellular organisms;Eukaryota;Opisthokonta;Metazoa;Eumetazoa;Bilateria;Protostomia;Spiralia;Lophotrochozoa;Mollusca;Bivalvia;Autobranchia;Pteriomorphia;Ostreida;Ostreoidea;Ostreidae;Crassostrea;Crassostrea virginica
UdeM00000043531 29159 511.5 UdeM00000043531 XP_011435928.2 40.6 835 437 15 1 814 1 797 1.90E-141 511.5 29159 cellular organisms;Eukaryota;Opisthokonta;Metazoa;Eumetazoa;Bilateria;Protostomia;Spiralia;Lophotrochozoa;Mollusca;Bivalvia;Autobranchia;Pteriomorphia;Ostreida;Ostreoidea;Ostreidae;Crassostrea;Crassostrea gigas
UdeM00000043532 6596 218 UdeM00000043532 XP_045199314.1 68.6 159 46 2 1 157 1 157 8.50E-54 218 6596 cellular organisms;Eukaryota;Opisthokonta;Metazoa;Eumetazoa;Bilateria;Protostomia;Spiralia;Lophotrochozoa;Mollusca;Bivalvia;Autobranchia;Heteroconchia;Euheterodonta;Imparidentia;Venerida;Veneroidea;Veneridae;Mercenaria;Mercenaria mercenaria
UdeM00000034244 6579 620.9 UdeM00000034244 XP_033733400.1 57.1 580 147 7 14 575 19 514 3.30E-174 620.9 6579 cellular organisms;Eukaryota;Opisthokonta;Metazoa;Eumetazoa;Bilateria;Protostomia;Spiralia;Lophotrochozoa;Mollusca;Bivalvia;Autobranchia;Pteriomorphia;Pectinida;Pectinoidea;Pectinidae;Pecten;Pecten maximus
UdeM00000034245 6596 1067.4 UdeM00000034245 XP_045209371.1 66.3 744 249 2 14 757 18 759 0.00E+00 1067.4 6596 cellular organisms;Eukaryota;Opisthokonta;Metazoa;Eumetazoa;Bilateria;Protostomia;Spiralia;Lophotrochozoa;Mollusca;Bivalvia;Autobranchia;Heteroconchia;Euheterodonta;Imparidentia;Venerida;Veneroidea;Veneridae;Mercenaria;Mercenaria mercenaria
UdeM00000034246 6596 1065.4 UdeM00000034246 XP_045209371.1 65.3 760 260 3 5 764 4 759 3.20E-308 1065.4 6596 cellular

organisms;Eukaryota;Opisthokonta;Metazoa;Eumetazoa;Bilateria;Protostomia;Spiralia;Lophotrochozoa;Mollusca;Bivalvia;Autobranchia;Heteroconchia;Euheterodonta;Imparidentia;Venerida;Veneroidea;Veneridae;Mercenaria;Mercenaria mercenaria
UdeM00000034247 6565 72 UdeM00000034247 XP_022326036.1 74.7 75 19 0 11 85 5 79 4.10E-10 72 6565 cellular organisms;Eukaryota;Opisthokonta;Metazoa;Eumetazoa;Bilateria;Protostomia;Spiralia;Lophotrochozoa;Mollusca;Bivalvia;Autobranchia;Pteriomorphia;Ostreida;Ostreoidea;Ostreidae;Crassostrea;Crassostrea virginica
UdeM00000034248 6596 617.8 UdeM00000034248 XP_045209831.1 73.2 403 107 1 29 431 4 405 1.00E-173 617.8 6596 cellular organisms;Eukaryota;Opisthokonta;Metazoa;Eumetazoa;Bilateria;Protostomia;Spiralia;Lophotrochozoa;Mollusca;Bivalvia;Autobranchia;Heteroconchia;Euheterodonta;Imparidentia;Venerida;Veneroidea;Veneridae;Mercenaria;Mercenaria mercenaria
UdeM00000034249 6596 208.8 UdeM00000034249 XP_045208536.1 32.1 389 211 7 6 369 1 361 1.20E-50 208.8 6596 cellular organisms;Eukaryota;Opisthokonta;Metazoa;Eumetazoa;Bilateria;Protostomia;Spiralia;Lophotrochozoa;Mollusca;Bivalvia;Autobranchia;Heteroconchia;Euheterodonta;Imparidentia;Venerida;Veneroidea;Veneridae;Mercenaria;Mercenaria mercenaria
UdeM00000034250 6596 136.7 UdeM00000034250 XP_045162869.1 38.7 186 108 4 52 232 189 373 3.70E-29 136.7 6596 cellular organisms;Eukaryota;Opisthokonta;Metazoa;Eumetazoa;Bilateria;Protostomia;Spiralia;Lophotrochozoa;Mollusca;Bivalvia;Autobranchia;Heteroconchia;Euheterodonta;Imparidentia;Venerida;Veneroidea;Veneridae;Mercenaria;Mercenaria mercenaria
UdeM00000034253 6579 1307.4 UdeM00000034253 XP_033733003.1 75 879 172 7 1 865 1 845 0.00E+00 1307.4 6579 cellular organisms;Eukaryota;Opisthokonta;Metazoa;Eumetazoa;Bilateria;Protostomia;Spiralia;Lophotrochozoa;Mollusca;Bivalvia;Autobranchia;Pteriomorphia;Pectinida;Pectinoidea;Pectinidae;Pecten;Pecten maximus
UdeM00000034254 6579 1323.5 UdeM00000034254 XP_033733003.1 75.5 879 174 6 1 872 1 845 0.00E+00 1323.5 6579 cellular organisms;Eukaryota;Opisthokonta;Metazoa;Eumetazoa;Bilateria;Protostomia;Spiralia;Lophotrochozoa;Mollusca;Bivalvia;Autobranchia;Pteriomorphia;Pectinida;Pectinoidea;Pectinidae;Pecten;Pecten maximus
UdeM00000034255 6596 1250.7 UdeM00000034255 XP_045210369.1 51.3 1256 583 11 1 1250 62 1294 0.00E+00 1250.7 6596 cellular organisms;Eukaryota;Opisthokonta;Metazoa;Eumetazoa;Bilateria;Protostomia;Spiralia;Lophotrochozoa;Mollusca;Bivalvia;Autobranchia;Heteroconchia;Euheterodonta;Imparidentia;Venerida;Veneroidea;Veneridae;Mercenaria;Mercenaria mercenaria
UdeM00000034256 6596 1329.7 UdeM00000034256 XP_045210369.1 51.8 1301 604 10 37 1331 11 1294 0.00E+00 1329.7 6596 cellular organisms;Eukaryota;Opisthokonta;Metazoa;Eumetazoa;Bilateria;Protostomia;Spiralia;Lophotrochozoa;Mollusca;Bivalvia;Autobranchia;Heteroconchia;Euheterodonta;Imparidentia;Venerida;Veneroidea;Veneridae;Mercenaria;Mercenaria mercenaria

UdeM00000034257 6596 578.6 UdeM00000034257 XP_045210369.1 57.8 479
202 0 1 479 816 1294 7.60E-162 578.6 6596 cellular organisms;Eukaryota;Opisthokonta;Metazoa;Eumetazoa;Bilateria;Protostomia;Spiralia;Lophotrochozoa;Mollusca;Bivalvia;Autobranchia;Heteroconchia;Euheterodonta;Imparidentia;Venerida;Veneroidea;Veneridae;Mercenaria;Mercenaria mercenaria
UdeM00000034258 6579 213.8 UdeM00000034258 XP_033745805.1 46.1 269
129 6 3 258 29 294 2.70E-52 213.8 6579 cellular organisms;Eukaryota;Opisthokonta;Metazoa;Eumetazoa;Bilateria;Protostomia;Spiralia;Lophotrochozoa;Mollusca;Bivalvia;Autobranchia;Pteriomorphia;Pectinida;Pectinoidea;Pectinidae;Pecten;Pecten maximus
UdeM00000034259 6579 308.5 UdeM00000034259 XP_033730215.1 46.6 322
167 3 6 322 264 585 1.00E-80 308.5 6579 cellular organisms;Eukaryota;Opisthokonta;Metazoa;Eumetazoa;Bilateria;Protostomia;Spiralia;Lophotrochozoa;Mollusca;Bivalvia;Autobranchia;Pteriomorphia;Pectinida;Pectinoidea;Pectinidae;Pecten;Pecten maximus
UdeM00000034260 6579 142.5 UdeM00000034260 XP_033745803.1 42 174
98 1 6 176 264 437 5.30E-31 142.5 6579 cellular organisms;Eukaryota;Opisthokonta;Metazoa;Eumetazoa;Bilateria;Protostomia;Spiralia;Lophotrochozoa;Mollusca;Bivalvia;Autobranchia;Pteriomorphia;Pectinida;Pectinoidea;Pectinidae;Pecten;Pecten maximus
UdeM00000034261 6579 249.2 UdeM00000034261 XP_033761616.1 55.5 218
95 1 4 219 8 225 4.80E-63 249.2 6579 cellular organisms;Eukaryota;Opisthokonta;Metazoa;Eumetazoa;Bilateria;Protostomia;Spiralia;Lophotrochozoa;Mollusca;Bivalvia;Autobranchia;Pteriomorphia;Pectinida;Pectinoidea;Pectinidae;Pecten;Pecten maximus
UdeM00000034262 6596 413.7 UdeM00000034262 XP_045210369.1 45.4 487
247 8 1 484 62 532 3.30E-112 413.7 6596 cellular organisms;Eukaryota;Opisthokonta;Metazoa;Eumetazoa;Bilateria;Protostomia;Spiralia;Lophotrochozoa;Mollusca;Bivalvia;Autobranchia;Heteroconchia;Euheterodonta;Imparidentia;Venerida;Veneroidea;Veneridae;Mercenaria;Mercenaria mercenaria
UdeM00000034263 6596 787.7 UdeM00000034263 XP_045210369.1 53.3 719
334 1 1 717 576 1294 1.20E-224 787.7 6596 cellular organisms;Eukaryota;Opisthokonta;Metazoa;Eumetazoa;Bilateria;Protostomia;Spiralia;Lophotrochozoa;Mollusca;Bivalvia;Autobranchia;Heteroconchia;Euheterodonta;Imparidentia;Venerida;Veneroidea;Veneridae;Mercenaria;Mercenaria mercenaria
UdeM00000034264 6596 827.4 UdeM00000034264 XP_045210369.1 53.9 748
343 1 3 748 547 1294 1.50E-236 827.4 6596 cellular organisms;Eukaryota;Opisthokonta;Metazoa;Eumetazoa;Bilateria;Protostomia;Spiralia;Lophotrochozoa;Mollusca;Bivalvia;Autobranchia;Heteroconchia;Euheterodonta;Imparidentia;Venerida;Veneroidea;Veneridae;Mercenaria;Mercenaria mercenaria
UdeM00000034265 6596 218.8 UdeM00000034265 XP_045209472.1 65.7 327
84 12 1 313 1 313 9.90E-54 218.8 6596 cellular organisms;Eukaryota;Opisthokonta;Metazoa;Eumetazoa;Bilateria;Protostomia;Spiralia;Lophotrochozoa;Mollusca;Bivalvia;Autobranchia;Heteroconchia;Euheterodonta;Imparidentia;Venerida;Veneroidea;Veneridae;Mercenaria;Mercenaria mercenaria
UdeM00000034266 6596 165.2 UdeM00000034266 XP_045209472.1 60.9 174
57 6 1 169 1 168 7.10E-38 165.2 6596 cellular organisms;Eukaryota;Opisthokonta;Metazoa;Eumetazoa;Bilateria;Protostomia;Spiralia;Lophotrochozoa;Mollusca;Bivalvia;Autobranchia;Heteroconchia;Euhe

terodonta;Imparidentia;Venerida;Veneroidea;Veneridae;Mercenaria;Mercenari
a mercenaria
UdeM00000034267 6526 79.7 UdeM00000034267 XP_013080019.1 46 215
108 2 1 213 1 209 4.90E-12 79.7 6526 cellular
organisms;Eukaryota;Opisthokonta;Metazoa;Eumetazoa;Bilateria;Protostomia;
Spiralia;Lophotrochozoa;Mollusca;Gastropoda;Heterobranchia;Euthyneura;Pan
pulmonata;Hygrophila;Lymnaeoidea;Planorbidae;Biomphalaria;Biomphalaria
glabrata
UdeM00000034268 7574 89 UdeM00000034268 XP_013384015.1 57.5 87
36 1 6 92 186 271 5.50E-15 89 7574 cellular
organisms;Eukaryota;Opisthokonta;Metazoa;Eumetazoa;Bilateria;Protostomia;
Spiralia;Lophotrochozoa;Brachiopoda;Linguliformea;Lingulata;Lingulida;Lin
guloidea;Lingulidae;Lingula;Lingula anatina
UdeM00000034271 6596 398.3 UdeM00000034271 XP_045209230.1 59.6 322
125 3 1 317 6 327 9.70E-108 398.3 6596 cellular
organisms;Eukaryota;Opisthokonta;Metazoa;Eumetazoa;Bilateria;Protostomia;
Spiralia;Lophotrochozoa;Mollusca;Bivalvia;Autobranchia;Heteroconchia;Euhe
terodonta;Imparidentia;Venerida;Veneroidea;Veneridae;Mercenaria;Mercenari
a mercenaria
UdeM00000034270 6596 490.3 UdeM00000034270 XP_045209230.1 62.8 379
135 4 1 373 6 384 2.20E-135 490.3 6596 cellular
organisms;Eukaryota;Opisthokonta;Metazoa;Eumetazoa;Bilateria;Protostomia;
Spiralia;Lophotrochozoa;Mollusca;Bivalvia;Autobranchia;Heteroconchia;Euhe
terodonta;Imparidentia;Venerida;Veneroidea;Veneridae;Mercenaria;Mercenari
a mercenaria
UdeM00000034272 6596 112.8 UdeM00000034272 XP_045180427.1 39 177
105 2 76 250 98 273 6.50E-22 112.8 6596 cellular
organisms;Eukaryota;Opisthokonta;Metazoa;Eumetazoa;Bilateria;Protostomia;
Spiralia;Lophotrochozoa;Mollusca;Bivalvia;Autobranchia;Heteroconchia;Euhe
terodonta;Imparidentia;Venerida;Veneroidea;Veneridae;Mercenaria;Mercenari
a mercenaria
UdeM00000034273 6596 1467.6 UdeM00000034273 XP_045209230.1 72.4
937 255 2 2 934 386 1322 0.00E+00 1467.6 6596
cellular
organisms;Eukaryota;Opisthokonta;Metazoa;Eumetazoa;Bilateria;Protostomia;
Spiralia;Lophotrochozoa;Mollusca;Bivalvia;Autobranchia;Heteroconchia;Euhe
terodonta;Imparidentia;Venerida;Veneroidea;Veneridae;Mercenaria;Mercenari
a mercenaria
UdeM00000034274 400727 216.1 UdeM00000034274 XP_025094200.1 43.2
234 131 1 1 234 1 232 4.80E-53 216.1 400727
cellular
organisms;Eukaryota;Opisthokonta;Metazoa;Eumetazoa;Bilateria;Protostomia;
Spiralia;Lophotrochozoa;Mollusca;Gastropoda;Caenogastropoda;Architaeniogl
ossa;Ampullarioidea;Ampullariidae;Pomacea;Pomacea canaliculata
UdeM00000034275 29159 213 UdeM00000034275 XP_011423583.2 45.6 206
110 2 2 207 29 232 3.60E-52 213 29159 cellular
organisms;Eukaryota;Opisthokonta;Metazoa;Eumetazoa;Bilateria;Protostomia;
Spiralia;Lophotrochozoa;Mollusca;Bivalvia;Autobranchia;Pteriomorphia;Ostr
eida;Ostreoidea;Ostreidae;Crassostrea;Crassostrea gigas
UdeM00000034277 6565 364.4 UdeM00000034277 XP_022329293.1 71.3 254
73 0 1 254 841 1094 1.20E-97 364.4 6565 cellular
organisms;Eukaryota;Opisthokonta;Metazoa;Eumetazoa;Bilateria;Protostomia;
Spiralia;Lophotrochozoa;Mollusca;Bivalvia;Autobranchia;Pteriomorphia;Ostr
eida;Ostreoidea;Ostreidae;Crassostrea;Crassostrea virginica

UdeM00000034276	6596	937.6	UdeM00000034276	XP_045208008.1	68.5	701	204	2	78	778	419	1102	1.00E-269	937.6	6596	cellular organisms;Eukaryota;Opisthokonta;Metazoa;Eumetazoa;Bilateria;Protostomia;Spiralia;Lophotrochozoa;Mollusca;Bivalvia;Autobranchia;Heteroconchia;Euheterodonta;Imparidentia;Venerida;Veneroidea;Veneridae;Mercenaria;Mercenaria mercenaria
UdeM00000034278	7906	119.4	UdeM00000034278	XP_033868128.2	35.9	198	112	8	9	206	8	190	6.20E-24	119.4	7906	cellular organisms;Eukaryota;Opisthokonta;Metazoa;Eumetazoa;Bilateria;Deuterostomia;Chordata;Craniata;Vertebrata;Gnathostomata;Teleostomi;Euteleostomi;Actinopterygii;Actinopteri;Chondrostei;Acipenseriformes;Acipenseroidei;Acipenseridae;Acipenserinae;Acipenserini;Acipenser;Acipenser ruthenus
UdeM00000034281	46514	134.4	UdeM00000034281	XP_038058294.1	80.8	78	15	0	1	78	1	78	6.60E-29	134.4	46514	cellular organisms;Eukaryota;Opisthokonta;Metazoa;Eumetazoa;Bilateria;Deuterostomia;Echinodermata;Eleutherozoa;Asterozoa;Asteroidea;Valvatacea;Valvatida;Asterinidae;Patiria;Patiria miniata
UdeM00000034283	29159	1144.4	UdeM00000034283	XP_011423958.1	71.1	922	225	12	14	909	59	965	0.00E+00	1144.4	29159	cellular organisms;Eukaryota;Opisthokonta;Metazoa;Eumetazoa;Bilateria;Protostomia;Spiralia;Lophotrochozoa;Mollusca;Bivalvia;Autobranchia;Pteriomorphia;Ostreida;Ostreoidea;Ostreidae;Crassostrea;Crassostrea gigas
UdeM00000034284	6596	237.3	UdeM00000034284	XP_045207983.1	62.5	192	69	2	1	192	16	204	6.10E-59	237.3	6596	cellular organisms;Eukaryota;Opisthokonta;Metazoa;Eumetazoa;Bilateria;Protostomia;Spiralia;Lophotrochozoa;Mollusca;Bivalvia;Autobranchia;Heteroconchia;Euheterodonta;Imparidentia;Venerida;Veneroidea;Veneridae;Mercenaria;Mercenaria mercenaria
UdeM00000034285	6596	375.6	UdeM00000034285	XP_045208652.1	68.1	301	90	4	1	297	1	299	6.00E-101	375.6	6596	cellular organisms;Eukaryota;Opisthokonta;Metazoa;Eumetazoa;Bilateria;Protostomia;Spiralia;Lophotrochozoa;Mollusca;Bivalvia;Autobranchia;Heteroconchia;Euheterodonta;Imparidentia;Venerida;Veneroidea;Veneridae;Mercenaria;Mercenaria mercenaria
UdeM00000034291	6596	168.7	UdeM00000034291	XP_045207153.1	46.2	171	80	5	4	174	33	191	1.00E-38	168.7	6596	cellular organisms;Eukaryota;Opisthokonta;Metazoa;Eumetazoa;Bilateria;Protostomia;Spiralia;Lophotrochozoa;Mollusca;Bivalvia;Autobranchia;Heteroconchia;Euheterodonta;Imparidentia;Venerida;Veneroidea;Veneridae;Mercenaria;Mercenaria mercenaria
UdeM00000034290	6596	175.6	UdeM00000034290	XP_045207153.1	45.4	185	89	5	10	194	19	191	8.90E-41	175.6	6596	cellular organisms;Eukaryota;Opisthokonta;Metazoa;Eumetazoa;Bilateria;Protostomia;Spiralia;Lophotrochozoa;Mollusca;Bivalvia;Autobranchia;Heteroconchia;Euheterodonta;Imparidentia;Venerida;Veneroidea;Veneridae;Mercenaria;Mercenaria mercenaria
UdeM00000034292	6596	322	UdeM00000034292	XP_045207150.1	45.5	719	333	13	10	719	6	674	2.00E-84	322	6596	cellular organisms;Eukaryota;Opisthokonta;Metazoa;Eumetazoa;Bilateria;Protostomia;Spiralia;Lophotrochozoa;Mollusca;Bivalvia;Autobranchia;Heteroconchia;Euheterodonta;Imparidentia;Venerida;Veneroidea;Veneridae;Mercenaria;Mercenaria mercenaria

UdeM00000034293 6596 119 UdeM00000034293 XP_045207189.1 72.9 70
19 0 1 70 412 481 2.70E-24 119 6596 cellular
organisms;Eukaryota;Opisthokonta;Metazoa;Eumetazoa;Bilateria;Protostomia;
Spiralia;Lophotrochozoa;Mollusca;Bivalvia;Autobranchia;Heteroconchia;Euhe
terodonta;Imparidentia;Venerida;Veneroidea;Veneridae;Mercenaria;Mercenari
a mercenaria
UdeM00000034294 6596 143.7 UdeM00000034294 XP_045207189.1 46.2 156
81 1 9 164 116 268 2.10E-31 143.7 6596 cellular
organisms;Eukaryota;Opisthokonta;Metazoa;Eumetazoa;Bilateria;Protostomia;
Spiralia;Lophotrochozoa;Mollusca;Bivalvia;Autobranchia;Heteroconchia;Euhe
terodonta;Imparidentia;Venerida;Veneroidea;Veneridae;Mercenaria;Mercenari
a mercenaria
UdeM00000034295 50390 79.3 UdeM00000034295 XP_028149250.1 33.1 172
104 6 1 169 58 221 5.20E-12 79.3 50390 cellular
organisms;Eukaryota;Opisthokonta;Metazoa;Eumetazoa;Bilateria;Protostomia;
Ecdysozoa;Panarthropoda;Arthropoda;Mandibulata;Pancrustacea;Hexapoda;Inse
cta;Dicondylia;Pterygota;Neoptera;Endopterygota;Coleoptera;Polyphaga;Cucu
jiformia;Chrysomeloidea;Chrysomelidae;Galerucinae;Luperini;Diabroticina;D
iabroticites;Diabrotica;Diabrotica virgifera;Diabrotica virgifera
virgifera
UdeM00000034296 6596 530 UdeM00000034296 XP_045207189.1 54.8 487
212 4 68 551 51 532 3.50E-147 530 6596 cellular
organisms;Eukaryota;Opisthokonta;Metazoa;Eumetazoa;Bilateria;Protostomia;
Spiralia;Lophotrochozoa;Mollusca;Bivalvia;Autobranchia;Heteroconchia;Euhe
terodonta;Imparidentia;Venerida;Veneroidea;Veneridae;Mercenaria;Mercenari
a mercenaria
UdeM00000034297 6596 394.4 UdeM00000034297 XP_045207189.1 51.8 398
186 3 60 456 51 443 2.00E-106 394.4 6596 cellular
organisms;Eukaryota;Opisthokonta;Metazoa;Eumetazoa;Bilateria;Protostomia;
Spiralia;Lophotrochozoa;Mollusca;Bivalvia;Autobranchia;Heteroconchia;Euhe
terodonta;Imparidentia;Venerida;Veneroidea;Veneridae;Mercenaria;Mercenari
a mercenaria
UdeM00000034298 6596 548.9 UdeM00000034298 XP_045207189.1 55.9 490
210 3 39 527 51 535 7.10E-153 548.9 6596 cellular
organisms;Eukaryota;Opisthokonta;Metazoa;Eumetazoa;Bilateria;Protostomia;
Spiralia;Lophotrochozoa;Mollusca;Bivalvia;Autobranchia;Heteroconchia;Euhe
terodonta;Imparidentia;Venerida;Veneroidea;Veneridae;Mercenaria;Mercenari
a mercenaria
UdeM00000034299 1735272 369 UdeM00000034299 XP_041372401.1 50.8
455 179 10 255 709 291 700 1.40E-98 369 1735272
cellular
organisms;Eukaryota;Opisthokonta;Metazoa;Eumetazoa;Bilateria;Protostomia;
Spiralia;Lophotrochozoa;Mollusca;Gastropoda;Neomphalina;Neomphaloidea;Pel
tospiridae;Gigantopelta;Gigantopelta aegis
UdeM00000034300 29159 145.6 UdeM00000034300 XP_034311318.1 48.6 142
69 3 13 151 1 141 5.80E-32 145.6 29159 cellular
organisms;Eukaryota;Opisthokonta;Metazoa;Eumetazoa;Bilateria;Protostomia;
Spiralia;Lophotrochozoa;Mollusca;Bivalvia;Autobranchia;Pteriomorphia;Ostr
eida;Ostreoidea;Ostreidae;Crassostrea;Crassostrea gigas
UdeM00000034301 29159 133.7 UdeM00000034301 XP_034311318.1 51.2 127
58 2 1 125 167 291 1.80E-28 133.7 29159 cellular
organisms;Eukaryota;Opisthokonta;Metazoa;Eumetazoa;Bilateria;Protostomia;
Spiralia;Lophotrochozoa;Mollusca;Bivalvia;Autobranchia;Pteriomorphia;Ostr
eida;Ostreoidea;Ostreidae;Crassostrea;Crassostrea gigas

UdeM00000034302 29159 98.2 UdeM00000034302 XP_034311318.1 53.8 91
40 1 1 91 322 410 6.00E-18 98.2 29159 cellular
organisms;Eukaryota;Opisthokonta;Metazoa;Eumetazoa;Bilateria;Protostomia;
Spiralia;Lophotrochozoa;Mollusca;Bivalvia;Autobranchia;Pteriomorphia;Ostr
eida;Ostreoidea;Ostreidae;Crassostrea;Crassostrea gigas
UdeM00000034303 6596 80.9 UdeM00000034303 XP_045206295.1 29.3 208
123 5 3 196 539 736 2.70E-12 80.9 6596 cellular
organisms;Eukaryota;Opisthokonta;Metazoa;Eumetazoa;Bilateria;Protostomia;
Spiralia;Lophotrochozoa;Mollusca;Bivalvia;Autobranchia;Heteroconchia;Euhe
terodonta;Imparidentia;Venerida;Veneroidea;Veneridae;Mercenaria;Mercenari
a mercenaria
UdeM00000034304 6579 70.9 UdeM00000034304 XP_033736448.1 38.8 85
50 1 5 87 10 94 3.40E-09 70.9 6579 cellular
organisms;Eukaryota;Opisthokonta;Metazoa;Eumetazoa;Bilateria;Protostomia;
Spiralia;Lophotrochozoa;Mollusca;Bivalvia;Autobranchia;Pteriomorphia;Pect
inida;Pectinoidea;Pectinidae;Pecten;Pecten maximus
UdeM00000034305 6596 632.9 UdeM00000034305 XP_045209776.1 70.1 435
129 1 1 434 1 435 3.10E-178 632.9 6596 cellular
organisms;Eukaryota;Opisthokonta;Metazoa;Eumetazoa;Bilateria;Protostomia;
Spiralia;Lophotrochozoa;Mollusca;Bivalvia;Autobranchia;Heteroconchia;Euhe
terodonta;Imparidentia;Venerida;Veneroidea;Veneridae;Mercenaria;Mercenari
a mercenaria
UdeM00000034306 6596 629.4 UdeM00000034306 XP_045209776.1 70.5 430
126 1 1 429 6 435 3.30E-177 629.4 6596 cellular
organisms;Eukaryota;Opisthokonta;Metazoa;Eumetazoa;Bilateria;Protostomia;
Spiralia;Lophotrochozoa;Mollusca;Bivalvia;Autobranchia;Heteroconchia;Euhe
terodonta;Imparidentia;Venerida;Veneroidea;Veneridae;Mercenaria;Mercenari
a mercenaria
UdeM00000034308 1735272 156.8 UdeM00000034308 XP_041357032.1 53.1
145 63 3 1 140 38 182 2.20E-35 156.8 1735272
cellular
organisms;Eukaryota;Opisthokonta;Metazoa;Eumetazoa;Bilateria;Protostomia;
Spiralia;Lophotrochozoa;Mollusca;Gastropoda;Neomphalina;Neomphaloidea;Pel
tospiridae;Gigantopelta;Gigantopelta aegis
UdeM00000034309 6596 94.4 UdeM00000034309 XP_045180326.1 47.6 103
49 1 34 136 62 159 1.40E-16 94.4 6596 cellular
organisms;Eukaryota;Opisthokonta;Metazoa;Eumetazoa;Bilateria;Protostomia;
Spiralia;Lophotrochozoa;Mollusca;Bivalvia;Autobranchia;Heteroconchia;Euhe
terodonta;Imparidentia;Venerida;Veneroidea;Veneridae;Mercenaria;Mercenari
a mercenaria
UdeM00000034311 6596 1054.3 UdeM00000034311 XP_045210469.1 50.8
1011 416 5 3 933 71 1080 9.10E-305 1054.3 6596
cellular
organisms;Eukaryota;Opisthokonta;Metazoa;Eumetazoa;Bilateria;Protostomia;
Spiralia;Lophotrochozoa;Mollusca;Bivalvia;Autobranchia;Heteroconchia;Euhe
terodonta;Imparidentia;Venerida;Veneroidea;Veneridae;Mercenaria;Mercenari
a mercenaria
UdeM00000034312 6579 1016.1 UdeM00000034312 XP_033724877.1 79.6
587 96 1 15 577 45 631 1.80E-293 1016.1 6579
cellular
organisms;Eukaryota;Opisthokonta;Metazoa;Eumetazoa;Bilateria;Protostomia;
Spiralia;Lophotrochozoa;Mollusca;Bivalvia;Autobranchia;Pteriomorphia;Pect
inida;Pectinoidea;Pectinidae;Pecten;Pecten maximus

UdeM00000034313	6500	262.3	UdeM00000034313	XP_005099787.2	50	264
125	4	222	482	63	322	1.20E-66	262.3	6500	cellular
organisms;Eukaryota;Opisthokonta;Metazoa;Eumetazoa;Bilateria;Protostomia;
Spiralia;Lophotrochozoa;Mollusca;Gastropoda;Heterobranchia;Euthyneura;Tec
tipleura;Aplysiida;Aplysioidea;Aplysiidae;Aplysia;Aplysia californica
UdeM00000034314	37621	201.1	UdeM00000034314	XP_014679850.1	51.7	207
89	4	1	199	1	204	1.40E-48	201.1	37621	cellular
organisms;Eukaryota;Opisthokonta;Metazoa;Eumetazoa;Bilateria;Protostomia;
Ecdysozoa;Scalidophora;Priapulida;Priapulimorpha;Priapulimorphida;Priapul
idae;Priapulus;Priapulus caudatus
UdeM00000034315	37621	334.3	UdeM00000034315	XP_014679849.1	53.9	282
117	4	4	273	343	623	1.40E-88	334.3	37621	cellular
organisms;Eukaryota;Opisthokonta;Metazoa;Eumetazoa;Bilateria;Protostomia;
Ecdysozoa;Scalidophora;Priapulida;Priapulimorpha;Priapulimorphida;Priapul
idae;Priapulus;Priapulus caudatus
UdeM00000034316	29159	66.6	UdeM00000034316	XP_034315424.1	35.4	130
73	4	12	134	213	338	2.90E-08	66.6	29159	cellular
organisms;Eukaryota;Opisthokonta;Metazoa;Eumetazoa;Bilateria;Protostomia;
Spiralia;Lophotrochozoa;Mollusca;Bivalvia;Autobranchia;Pteriomorphia;Ostr
eida;Ostreoidea;Ostreidae;Crassostrea;Crassostrea gigas
UdeM00000034318	6596	152.1	UdeM00000034318	XP_045207280.1	53.3	137
64	0	2	138	188	324	6.20E-34	152.1	6596	cellular
organisms;Eukaryota;Opisthokonta;Metazoa;Eumetazoa;Bilateria;Protostomia;
Spiralia;Lophotrochozoa;Mollusca;Bivalvia;Autobranchia;Heteroconchia;Euhe
terodonta;Imparidentia;Venerida;Veneroidea;Veneridae;Mercenaria;Mercenari
a mercenaria
UdeM00000034319	7604	94	UdeM00000034319	XP_033628627.1	52.9	85
39	1	35	119	504	587	1.50E-16	94	7604	cellular
organisms;Eukaryota;Opisthokonta;Metazoa;Eumetazoa;Bilateria;Deuterostomi
a;Echinodermata;Eleutherozoa;Asterozoa;Asteroidea;Forcipulatacea;Forcipul
atida;Asteriidae;Asterias;Asterias rubens
UdeM00000034320	6579	186	UdeM00000034320	XP_033761495.1	74.8	119
24	1	25	143	164	276	7.30E-44	186	6579	cellular
organisms;Eukaryota;Opisthokonta;Metazoa;Eumetazoa;Bilateria;Protostomia;
Spiralia;Lophotrochozoa;Mollusca;Bivalvia;Autobranchia;Pteriomorphia;Pect
inida;Pectinoidea;Pectinidae;Pecten;Pecten maximus
UdeM00000034321	70779	108.2	UdeM00000034321	XP_015759403.1	31.3	243
97	5	277	460	145	376	2.80E-20	108.2	70779	cellular
organisms;Eukaryota;Opisthokonta;Metazoa;Eumetazoa;Cnidaria;Anthozoa;Hexa
corallia;Scleractinia;Astrocoeniina;Acroporidae;Acropora;Acropora
digitifera
UdeM00000034323	6596	188	UdeM00000034323	XP_045185473.1	20.8	1507
992	42	162	1582	4	1395	1.20E-43	188	6596	cellular
organisms;Eukaryota;Opisthokonta;Metazoa;Eumetazoa;Bilateria;Protostomia;
Spiralia;Lophotrochozoa;Mollusca;Bivalvia;Autobranchia;Heteroconchia;Euhe
terodonta;Imparidentia;Venerida;Veneroidea;Veneridae;Mercenaria;Mercenari
a mercenaria
UdeM00000000267	6596	391.3	UdeM00000000267	XP_045168006.1	73.1	245
65	1	1	245	107	350	8.80E-106	391.3	6596	cellular
organisms;Eukaryota;Opisthokonta;Metazoa;Eumetazoa;Bilateria;Protostomia;
Spiralia;Lophotrochozoa;Mollusca;Bivalvia;Autobranchia;Heteroconchia;Euhe
terodonta;Imparidentia;Venerida;Veneroidea;Veneridae;Mercenaria;Mercenari
a mercenaria

UdeM00000000268	6579	448.4	UdeM00000000268	XP_033762641.1	55.1	405
	170	4	37	439	6	400	1.10E-122	448.4	6579	cellular
organisms;Eukaryota;Opisthokonta;Metazoa;Eumetazoa;Bilateria;Protostomia;Spiralia;Lophotrochozoa;Mollusca;Bivalvia;Autobranchia;Pteriomorphia;Pectinida;Pectinoidea;Pectinidae;Pecten;Pecten maximus
UdeM00000000269	6579	439.1	UdeM00000000269	XP_033762641.1	53.6	414
	171	5	37	448	6	400	6.70E-120	439.1	6579	cellular
organisms;Eukaryota;Opisthokonta;Metazoa;Eumetazoa;Bilateria;Protostomia;Spiralia;Lophotrochozoa;Mollusca;Bivalvia;Autobranchia;Pteriomorphia;Pectinida;Pectinoidea;Pectinidae;Pecten;Pecten maximus
UdeM00000000270	8005	569.7	UdeM00000000270	XP_035380990.1	48.8	627
	292	7	1	608	29	645	5.40E-159	569.7	8005	cellular
organisms;Eukaryota;Opisthokonta;Metazoa;Eumetazoa;Bilateria;Deuterostomia;Chordata;Craniata;Vertebrata;Gnathostomata;Teleostomi;Euteleostomi;Actinopterygii;Actinopteri;Neopterygii;Teleostei;Osteoglossocephalai;Clupeocephala;Otomorpha;Ostariophysi;Otophysi;Characiphysae;Gymnotiformes;Gymnotoidei;Gymnotidae;Electrophorus;Electrophorus electricus
UdeM00000000271	1735272	357.8	UdeM00000000271	XP_041366148.1	61.7
	266	99	1	1	263	78	343	1.20E-95	357.8	1735272
	cellular
organisms;Eukaryota;Opisthokonta;Metazoa;Eumetazoa;Bilateria;Protostomia;Spiralia;Lophotrochozoa;Mollusca;Gastropoda;Neomphalina;Neomphaloidea;Peltospiridae;Gigantopelta;Gigantopelta aegis
UdeM00000000272	7739	357.5	UdeM00000000272	XP_035696678.1	36.4	569
	195	2	1	402	335	903	2.50E-95	357.5	7739	cellular
organisms;Eukaryota;Opisthokonta;Metazoa;Eumetazoa;Bilateria;Deuterostomia;Chordata;Cephalochordata;Leptocardii;Amphioxiformes;Branchiostomidae;Branchiostoma;Branchiostoma floridae
UdeM00000000273	6596	260.4	UdeM00000000273	XP_045180379.1	68.6	175
	55	0	1	175	1	175	1.70E-66	260.4	6596	cellular
organisms;Eukaryota;Opisthokonta;Metazoa;Eumetazoa;Bilateria;Protostomia;Spiralia;Lophotrochozoa;Mollusca;Bivalvia;Autobranchia;Heteroconchia;Euheterodonta;Imparidentia;Venerida;Veneroidea;Veneridae;Mercenaria;Mercenaria mercenaria
UdeM00000000274	6596	702.2	UdeM00000000274	XP_045168651.1	64.2	567
	202	1	435	1001	47	612	9.50E-199	702.2	6596	cellular
organisms;Eukaryota;Opisthokonta;Metazoa;Eumetazoa;Bilateria;Protostomia;Spiralia;Lophotrochozoa;Mollusca;Bivalvia;Autobranchia;Heteroconchia;Euheterodonta;Imparidentia;Venerida;Veneroidea;Veneridae;Mercenaria;Mercenaria mercenaria
UdeM00000000275	7574	152.5	UdeM00000000275	XP_013396733.1	43.2	206
	115	2	67	270	43	248	7.50E-34	152.5	7574	cellular
organisms;Eukaryota;Opisthokonta;Metazoa;Eumetazoa;Bilateria;Protostomia;Spiralia;Lophotrochozoa;Brachiopoda;Linguliformea;Lingulata;Lingulida;Linguloidea;Lingulidae;Lingula;Lingula anatina
UdeM00000000278	6596	86.7	UdeM00000000278	XP_045170040.1	32.2	199
	106	8	10	205	8	180	3.90E-14	86.7	6596	cellular
organisms;Eukaryota;Opisthokonta;Metazoa;Eumetazoa;Bilateria;Protostomia;Spiralia;Lophotrochozoa;Mollusca;Bivalvia;Autobranchia;Heteroconchia;Euheterodonta;Imparidentia;Venerida;Veneroidea;Veneridae;Mercenaria;Mercenaria mercenaria
UdeM00000000279	6596	234.6	UdeM00000000279	XP_045167421.1	46.5	256
	120	4	49	289	59	312	5.00E-58	234.6	6596	cellular
organisms;Eukaryota;Opisthokonta;Metazoa;Eumetazoa;Bilateria;Protostomia;

Spiralia;Lophotrochozoa;Mollusca;Bivalvia;Autobranchia;Heteroconchia;Euheterodonta;Imparidentia;Venerida;Veneroidea;Veneridae;Mercenaria;Mercenaria mercenaria
UdeM00000000280 6596 346.3 UdeM00000000280 XP_045167418.1 51.6 341 135 2 43 353 347 687 4.80E-92 346.3 6596 cellular organisms;Eukaryota;Opisthokonta;Metazoa;Eumetazoa;Bilateria;Protostomia;Spiralia;Lophotrochozoa;Mollusca;Bivalvia;Autobranchia;Heteroconchia;Euheterodonta;Imparidentia;Venerida;Veneroidea;Veneridae;Mercenaria;Mercenaria mercenaria
UdeM00000000281 1735272 542 UdeM00000000281 XP_041365615.1 61.3 401 153 2 1 401 2 400 6.60E-151 542 1735272 cellular organisms;Eukaryota;Opisthokonta;Metazoa;Eumetazoa;Bilateria;Protostomia;Spiralia;Lophotrochozoa;Mollusca;Gastropoda;Neomphalina;Neomphaloidea;Peltospiridae;Gigantopelta;Gigantopelta aegis
UdeM00000000282 1735272 77.8 UdeM00000000282 XP_041349428.1 41.2 97 48 1 23 110 136 232 9.90E-12 77.8 1735272 cellular organisms;Eukaryota;Opisthokonta;Metazoa;Eumetazoa;Bilateria;Protostomia;Spiralia;Lophotrochozoa;Mollusca;Gastropoda;Neomphalina;Neomphaloidea;Peltospiridae;Gigantopelta;Gigantopelta aegis
UdeM00000000283 6596 275.4 UdeM00000000283 XP_045168190.1 70.6 177 52 0 4 180 5 181 5.20E-71 275.4 6596 cellular organisms;Eukaryota;Opisthokonta;Metazoa;Eumetazoa;Bilateria;Protostomia;Spiralia;Lophotrochozoa;Mollusca;Bivalvia;Autobranchia;Heteroconchia;Euheterodonta;Imparidentia;Venerida;Veneroidea;Veneridae;Mercenaria;Mercenaria mercenaria
UdeM00000000286 6596 637.5 UdeM00000000286 XP_045170379.1 64.2 508 169 5 34 533 21 523 1.50E-179 637.5 6596 cellular organisms;Eukaryota;Opisthokonta;Metazoa;Eumetazoa;Bilateria;Protostomia;Spiralia;Lophotrochozoa;Mollusca;Bivalvia;Autobranchia;Heteroconchia;Euheterodonta;Imparidentia;Venerida;Veneroidea;Veneridae;Mercenaria;Mercenaria mercenaria
UdeM00000000287 6579 353.2 UdeM00000000287 XP_033761881.1 50.2 426 183 10 1 410 1 413 2.30E-93 353.2 6579 cellular organisms;Eukaryota;Opisthokonta;Metazoa;Eumetazoa;Bilateria;Protostomia;Spiralia;Lophotrochozoa;Mollusca;Bivalvia;Autobranchia;Pteriomorphia;Pectinida;Pectinoidea;Pectinidae;Pecten;Pecten maximus
UdeM00000000288 6573 421.4 UdeM00000000288 XP_021376345.1 54.6 370 161 3 1 366 1 367 1.30E-114 421.4 6573 cellular organisms;Eukaryota;Opisthokonta;Metazoa;Eumetazoa;Bilateria;Protostomia;Spiralia;Lophotrochozoa;Mollusca;Bivalvia;Autobranchia;Pteriomorphia;Pectinida;Pectinoidea;Pectinidae;Mizuhopecten;Mizuhopecten yessoensis
UdeM00000000289 6579 790.8 UdeM00000000289 XP_033762188.1 78.9 465 98 0 1 465 1 465 9.50E-226 790.8 6579 cellular organisms;Eukaryota;Opisthokonta;Metazoa;Eumetazoa;Bilateria;Protostomia;Spiralia;Lophotrochozoa;Mollusca;Bivalvia;Autobranchia;Pteriomorphia;Pectinida;Pectinoidea;Pectinidae;Pecten;Pecten maximus
UdeM00000000290 6596 419.1 UdeM00000000290 XP_045168429.1 65.7 350 99 5 2 340 98 437 8.60E-114 419.1 6596 cellular organisms;Eukaryota;Opisthokonta;Metazoa;Eumetazoa;Bilateria;Protostomia;Spiralia;Lophotrochozoa;Mollusca;Bivalvia;Autobranchia;Heteroconchia;Euheterodonta;Imparidentia;Venerida;Veneroidea;Veneridae;Mercenaria;Mercenaria mercenaria

UdeM00000000291 6596 466.1 UdeM00000000291 XP_045168431.1 64.1 384
121 7 27 408 31 399 7.40E-128 466.1 6596 cellular organisms;Eukaryota;Opisthokonta;Metazoa;Eumetazoa;Bilateria;Protostomia;Spiralia;Lophotrochozoa;Mollusca;Bivalvia;Autobranchia;Heteroconchia;Euheterodonta;Imparidentia;Venerida;Veneroidea;Veneridae;Mercenaria;Mercenaria mercenaria
UdeM00000000293 7888 149.8 UdeM00000000293 XP_043916349.1 58.1 124
52 0 64 187 413 536 3.40E-33 149.8 7888 cellular organisms;Eukaryota;Opisthokonta;Metazoa;Eumetazoa;Bilateria;Deuterostomia;Chordata;Craniata;Vertebrata;Gnathostomata;Teleostomi;Euteleostomi;Sarcopterygii;Dipnotetrapodomorpha;Dipnomorpha;Ceratodontae;Ceratodontiformes;Lepidosirenoidei;Protopteridae;Protopterus;Protopterus annectens
UdeM00000000297 6596 94 UdeM00000000297 XP_045167967.1 38.2 136
74 3 39 174 57 182 2.10E-16 94 6596 cellular organisms;Eukaryota;Opisthokonta;Metazoa;Eumetazoa;Bilateria;Protostomia;Spiralia;Lophotrochozoa;Mollusca;Bivalvia;Autobranchia;Heteroconchia;Euheterodonta;Imparidentia;Venerida;Veneroidea;Veneridae;Mercenaria;Mercenaria mercenaria
UdeM00000000300 6596 138.3 UdeM00000000300 XP_045169969.1 43.6 243
122 5 1 238 1 233 1.30E-29 138.3 6596 cellular organisms;Eukaryota;Opisthokonta;Metazoa;Eumetazoa;Bilateria;Protostomia;Spiralia;Lophotrochozoa;Mollusca;Bivalvia;Autobranchia;Heteroconchia;Euheterodonta;Imparidentia;Venerida;Veneroidea;Veneridae;Mercenaria;Mercenaria mercenaria
UdeM00000000301 6596 212.2 UdeM00000000301 XP_045168681.1 36.3 267
168 2 457 722 605 870 2.20E-51 212.2 6596 cellular organisms;Eukaryota;Opisthokonta;Metazoa;Eumetazoa;Bilateria;Protostomia;Spiralia;Lophotrochozoa;Mollusca;Bivalvia;Autobranchia;Heteroconchia;Euheterodonta;Imparidentia;Venerida;Veneroidea;Veneridae;Mercenaria;Mercenaria mercenaria
UdeM00000000302 6565 182.6 UdeM00000000302 XP_022305129.1 36.5 345
203 4 1 334 1 340 9.60E-43 182.6 6565 cellular organisms;Eukaryota;Opisthokonta;Metazoa;Eumetazoa;Bilateria;Protostomia;Spiralia;Lophotrochozoa;Mollusca;Bivalvia;Autobranchia;Pteriomorphia;Ostreida;Ostreoidea;Ostreidae;Crassostrea;Crassostrea virginica
UdeM00000000303 6573 245 UdeM00000000303 XP_021356369.1 42.4 403
190 8 1089 1467 6127 6511 6.10E-61 245 6573 cellular organisms;Eukaryota;Opisthokonta;Metazoa;Eumetazoa;Bilateria;Protostomia;Spiralia;Lophotrochozoa;Mollusca;Bivalvia;Autobranchia;Pteriomorphia;Pectinida;Pectinoidea;Pectinidae;Mizuhopecten;Mizuhopecten yessoensis
UdeM00000000304 6573 245 UdeM00000000304 XP_021356369.1 42.4 403
190 8 104 482 6127 6511 2.00E-61 245 6573 cellular organisms;Eukaryota;Opisthokonta;Metazoa;Eumetazoa;Bilateria;Protostomia;Spiralia;Lophotrochozoa;Mollusca;Bivalvia;Autobranchia;Pteriomorphia;Pectinida;Pectinoidea;Pectinidae;Mizuhopecten;Mizuhopecten yessoensis
UdeM00000000305 6596 624 UdeM00000000305 XP_045167922.1 46 1381
646 23 1599 2928 2 1333 9.60E-175 624 6596 cellular organisms;Eukaryota;Opisthokonta;Metazoa;Eumetazoa;Bilateria;Protostomia;Spiralia;Lophotrochozoa;Mollusca;Bivalvia;Autobranchia;Heteroconchia;Euheterodonta;Imparidentia;Venerida;Veneroidea;Veneridae;Mercenaria;Mercenaria mercenaria
UdeM00000000306 6596 636.3 UdeM00000000306 XP_045167913.1 49.2 1186
534 20 1795 2939 352 1510 1.90E-178 636.3 6596 cellular organisms;Eukaryota;Opisthokonta;Metazoa;Eumetazoa;Bilateria;Protostomia;

Spiralia;Lophotrochozoa;Mollusca;Bivalvia;Autobranchia;Heteroconchia;Euhe
terodonta;Imparidentia;Venerida;Veneroidea;Veneridae;Mercenaria;Mercenari
a mercenaria
UdeM00000000307 29159 67.4 UdeM00000000307 XP_034310663.1 43.7 71
40 0 11 81 4 74 1.20E-08 67.4 29159 cellular
organisms;Eukaryota;Opisthokonta;Metazoa;Eumetazoa;Bilateria;Protostomia;
Spiralia;Lophotrochozoa;Mollusca;Bivalvia;Autobranchia;Pteriomorphia;Ostr
eida;Ostreoidea;Ostreidae;Crassostrea;Crassostrea gigas
UdeM00000000308 2607531 109.8 UdeM00000000308 XP_029633614.1 52.8
89 41 1 1 88 1 89 1.90E-21 109.8 2607531
cellular
organisms;Eukaryota;Opisthokonta;Metazoa;Eumetazoa;Bilateria;Protostomia;
Spiralia;Lophotrochozoa;Mollusca;Cephalopoda;Coleoidea;Octopodiformes;Oct
opoda;Incirrata;Octopodidae;Octopus;Octopus sinensis
UdeM00000000310 34632 94 UdeM00000000310 XP_037515180.1 38.4 125
77 0 6 130 269 393 1.60E-16 94 34632 cellular
organisms;Eukaryota;Opisthokonta;Metazoa;Eumetazoa;Bilateria;Protostomia;
Ecdysozoa;Panarthropoda;Arthropoda;Chelicerata;Arachnida;Acari;Parasitifo
rmes;Ixodida;Ixodoidea;Ixodidae;Rhipicephalinae;Rhipicephalus;Rhipicephal
us;Rhipicephalus sanguineus group;Rhipicephalus sanguineus
UdeM00000000311 37621 183 UdeM00000000311 XP_014676995.1 58.3 139
57 1 1 139 1 138 2.70E-43 183 37621 cellular
organisms;Eukaryota;Opisthokonta;Metazoa;Eumetazoa;Bilateria;Protostomia;
Ecdysozoa;Scalidophora;Priapulida;Priapulimorpha;Priapulimorphida;Priapul
idae;Priapulus;Priapulus caudatus
UdeM00000000312 6596 558.5 UdeM00000000312 XP_045214662.1 40.9 743
395 16 12 725 50 777 1.20E-155 558.5 6596 cellular
organisms;Eukaryota;Opisthokonta;Metazoa;Eumetazoa;Bilateria;Protostomia;
Spiralia;Lophotrochozoa;Mollusca;Bivalvia;Autobranchia;Heteroconchia;Euhe
terodonta;Imparidentia;Venerida;Veneroidea;Veneridae;Mercenaria;Mercenari
a mercenaria
UdeM00000000313 6596 314.7 UdeM00000000313 XP_045214662.1 42.7 398
203 7 2 383 389 777 1.60E-82 314.7 6596 cellular
organisms;Eukaryota;Opisthokonta;Metazoa;Eumetazoa;Bilateria;Protostomia;
Spiralia;Lophotrochozoa;Mollusca;Bivalvia;Autobranchia;Heteroconchia;Euhe
terodonta;Imparidentia;Venerida;Veneroidea;Veneridae;Mercenaria;Mercenari
a mercenaria
UdeM00000000315 7668 73.9 UdeM00000000315 XP_030841875.1 48.4 64
32 1 3 66 71 133 1.40E-10 73.9 7668 cellular
organisms;Eukaryota;Opisthokonta;Metazoa;Eumetazoa;Bilateria;Deuterostomi
a;Echinodermata;Eleutherozoa;Echinozoa;Echinoidea;Euechinoidea;Echinacea;
Camarodonta;Echinidea;Strongylocentrotidae;Strongylocentrotus;Strongyloce
ntrotus purpuratus
UdeM00000000316 225164 94 UdeM00000000316 XP_009052182.1 45
100 49 2 16 114 132 226 1.40E-16 94 225164
cellular
organisms;Eukaryota;Opisthokonta;Metazoa;Eumetazoa;Bilateria;Protostomia;
Spiralia;Lophotrochozoa;Mollusca;Gastropoda;Patellogastropoda;Lottioidea;
Lottiidae;Lottia;Lottia gigantea
UdeM00000000317 29159 156.8 UdeM00000000317 XP_034309946.1 54.5 176
67 3 382 548 5323 5494 9.00E-35 156.8 29159 cellular
organisms;Eukaryota;Opisthokonta;Metazoa;Eumetazoa;Bilateria;Protostomia;
Spiralia;Lophotrochozoa;Mollusca;Bivalvia;Autobranchia;Pteriomorphia;Ostr
eida;Ostreoidea;Ostreidae;Crassostrea;Crassostrea gigas

UdeM00000000318 6596 1018.5 UdeM00000000318 XP_045214662.1 45.2
1277 627 28 11 1234 11 1267 7.30E-294 1018.5 6596
cellular
organisms;Eukaryota;Opisthokonta;Metazoa;Eumetazoa;Bilateria;Protostomia;Spiralia;Lophotrochozoa;Mollusca;Bivalvia;Autobranchia;Heteroconchia;Euheterodonta;Imparidentia;Venerida;Veneroidea;Veneridae;Mercenaria;Mercenaria mercenaria
UdeM00000000319 6706 146 UdeM00000000319 XP_042235765.1 43.9 157
88 0 1 157 1 157 4.20E-32 146 6706 cellular
organisms;Eukaryota;Opisthokonta;Metazoa;Eumetazoa;Bilateria;Protostomia;Ecdysozoa;Panarthropoda;Arthropoda;Mandibulata;Pancrustacea;Crustacea;Multicrustacea;Malacostraca;Eumalacostraca;Eucarida;Decapoda;Pleocyemata;Astacidea;Nephropoidea;Nephropidae;Homarus;Homarus americanus
UdeM00000000320 72036 264.6 UdeM00000000320 XP_040576246.1 45.8 297
155 3 3 297 1 293 1.50E-67 264.6 72036 cellular
organisms;Eukaryota;Opisthokonta;Metazoa;Eumetazoa;Bilateria;Protostomia;Ecdysozoa;Panarthropoda;Arthropoda;Mandibulata;Pancrustacea;Crustacea;Multicrustacea;Hexanauplia;Copepoda;Neocopepoda;Podoplea;Siphonostomatoida;Caligidae;Lepeophtheirus;Lepeophtheirus salmonis
UdeM00000000322 6579 111.7 UdeM00000000322 XP_033761790.1 26.7 300
166 8 14 268 154 444 2.00E-21 111.7 6579 cellular
organisms;Eukaryota;Opisthokonta;Metazoa;Eumetazoa;Bilateria;Protostomia;Spiralia;Lophotrochozoa;Mollusca;Bivalvia;Autobranchia;Pteriomorphia;Pectinida;Pectinoidea;Pectinidae;Pecten;Pecten maximus
UdeM00000000323 6596 138.7 UdeM00000000323 XP_045168928.1 38.5 213
123 4 111 317 640 850 1.40E-29 138.7 6596 cellular
organisms;Eukaryota;Opisthokonta;Metazoa;Eumetazoa;Bilateria;Protostomia;Spiralia;Lophotrochozoa;Mollusca;Bivalvia;Autobranchia;Heteroconchia;Euheterodonta;Imparidentia;Venerida;Veneroidea;Veneridae;Mercenaria;Mercenaria mercenaria
UdeM00000000324 6596 104.8 UdeM00000000324 XP_045214662.1 67.9 78
21 2 1 78 1107 1180 5.30E-20 104.8 6596 cellular
organisms;Eukaryota;Opisthokonta;Metazoa;Eumetazoa;Bilateria;Protostomia;Spiralia;Lophotrochozoa;Mollusca;Bivalvia;Autobranchia;Heteroconchia;Euheterodonta;Imparidentia;Venerida;Veneroidea;Veneridae;Mercenaria;Mercenaria mercenaria
UdeM00000000325 6596 147.9 UdeM00000000325 XP_045214662.1 57.9 121
51 0 1 121 972 1092 8.40E-33 147.9 6596 cellular
organisms;Eukaryota;Opisthokonta;Metazoa;Eumetazoa;Bilateria;Protostomia;Spiralia;Lophotrochozoa;Mollusca;Bivalvia;Autobranchia;Heteroconchia;Euheterodonta;Imparidentia;Venerida;Veneroidea;Veneridae;Mercenaria;Mercenaria mercenaria
UdeM00000000327 6596 237.7 UdeM00000000327 XP_045214662.1 39.5 347
185 7 1 331 415 752 2.20E-59 237.7 6596 cellular
organisms;Eukaryota;Opisthokonta;Metazoa;Eumetazoa;Bilateria;Protostomia;Spiralia;Lophotrochozoa;Mollusca;Bivalvia;Autobranchia;Heteroconchia;Euheterodonta;Imparidentia;Venerida;Veneroidea;Veneridae;Mercenaria;Mercenaria mercenaria
UdeM00000000328 6596 1008.4 UdeM00000000328 XP_045214662.1 45.2
1278 585 27 11 1194 11 1267 7.40E-291 1008.4 6596
cellular
organisms;Eukaryota;Opisthokonta;Metazoa;Eumetazoa;Bilateria;Protostomia;Spiralia;Lophotrochozoa;Mollusca;Bivalvia;Autobranchia;Heteroconchia;Euhe

terodonta;Imparidentia;Venerida;Veneroidea;Veneridae;Mercenaria;Mercenaria mercenaria
UdeM00000000329 6596 693.7 UdeM00000000329 XP_045169298.1 43.9 852 438 17 26 855 25 858 2.90E-196 693.7 6596 cellular organisms;Eukaryota;Opisthokonta;Metazoa;Eumetazoa;Bilateria;Protostomia;Spiralia;Lophotrochozoa;Mollusca;Bivalvia;Autobranchia;Heteroconchia;Euheterodonta;Imparidentia;Venerida;Veneroidea;Veneridae;Mercenaria;Mercenaria mercenaria
UdeM00000000330 6596 84.3 UdeM00000000330 XP_045211012.1 44.4 90 49 1 10 98 321 410 9.40E-14 84.3 6596 cellular organisms;Eukaryota;Opisthokonta;Metazoa;Eumetazoa;Bilateria;Protostomia;Spiralia;Lophotrochozoa;Mollusca;Bivalvia;Autobranchia;Heteroconchia;Euheterodonta;Imparidentia;Venerida;Veneroidea;Veneridae;Mercenaria;Mercenaria mercenaria
UdeM00000000331 6596 1077 UdeM00000000331 XP_045168803.1 48.2 1142 548 15 30 1143 107 1233 0.00E+00 1077 6596 cellular organisms;Eukaryota;Opisthokonta;Metazoa;Eumetazoa;Bilateria;Protostomia;Spiralia;Lophotrochozoa;Mollusca;Bivalvia;Autobranchia;Heteroconchia;Euheterodonta;Imparidentia;Venerida;Veneroidea;Veneridae;Mercenaria;Mercenaria mercenaria
UdeM00000000332 1735272 346.7 UdeM00000000332 XP_041378840.1 51.1 319 155 1 100 418 2 319 4.20E-92 346.7 1735272 cellular organisms;Eukaryota;Opisthokonta;Metazoa;Eumetazoa;Bilateria;Protostomia;Spiralia;Lophotrochozoa;Mollusca;Gastropoda;Neomphalina;Neomphaloidea;Peltospiridae;Gigantopelta;Gigantopelta aegis
UdeM00000000333 1735272 424.5 UdeM00000000333 XP_041347065.1 66.3 309 103 1 1 308 179 487 1.20E-115 424.5 1735272 cellular organisms;Eukaryota;Opisthokonta;Metazoa;Eumetazoa;Bilateria;Protostomia;Spiralia;Lophotrochozoa;Mollusca;Gastropoda;Neomphalina;Neomphaloidea;Peltospiridae;Gigantopelta;Gigantopelta aegis
UdeM00000000334 1735272 97.8 UdeM00000000334 XP_041347065.1 37.9 140 44 2 1 97 1 140 8.40E-18 97.8 1735272 cellular organisms;Eukaryota;Opisthokonta;Metazoa;Eumetazoa;Bilateria;Protostomia;Spiralia;Lophotrochozoa;Mollusca;Gastropoda;Neomphalina;Neomphaloidea;Peltospiridae;Gigantopelta;Gigantopelta aegis
UdeM00000000335 6579 100.5 UdeM00000000335 XP_033725865.1 30 267 96 2 1 176 1 267 2.30E-18 100.5 6579 cellular organisms;Eukaryota;Opisthokonta;Metazoa;Eumetazoa;Bilateria;Protostomia;Spiralia;Lophotrochozoa;Mollusca;Bivalvia;Autobranchia;Pteriomorphia;Pectinida;Pectinoidea;Pectinidae;Pecten;Pecten maximus
UdeM00000000336 6596 882.5 UdeM00000000336 XP_045214662.1 43.5 1174 600 22 39 1164 104 1262 5.90E-253 882.5 6596 cellular organisms;Eukaryota;Opisthokonta;Metazoa;Eumetazoa;Bilateria;Protostomia;Spiralia;Lophotrochozoa;Mollusca;Bivalvia;Autobranchia;Heteroconchia;Euheterodonta;Imparidentia;Venerida;Veneroidea;Veneridae;Mercenaria;Mercenaria mercenaria
UdeM00000000337 400727 345.1 UdeM00000000337 XP_025109728.1 50.7 353 159 4 45 386 323 671 1.10E-91 345.1 400727 cellular organisms;Eukaryota;Opisthokonta;Metazoa;Eumetazoa;Bilateria;Protostomia;

Spiralia;Lophotrochozoa;Mollusca;Gastropoda;Caenogastropoda;Architaenioglossa;Ampullarioidea;Ampullariidae;Pomacea;Pomacea canaliculata
UdeM00000000338 6596 686.4 UdeM00000000338 XP_045167551.1 60.3 595
203 3 54 648 35 596 3.50E-194 686.4 6596 cellular organisms;Eukaryota;Opisthokonta;Metazoa;Eumetazoa;Bilateria;Protostomia;Spiralia;Lophotrochozoa;Mollusca;Bivalvia;Autobranchia;Heteroconchia;Euheterodonta;Imparidentia;Venerida;Veneroidea;Veneridae;Mercenaria;Mercenaria mercenaria
UdeM00000000339 6596 578.2 UdeM00000000339 XP_045167628.1 42 757
395 12 16 735 180 929 2.20E-161 578.2 6596 cellular organisms;Eukaryota;Opisthokonta;Metazoa;Eumetazoa;Bilateria;Protostomia;Spiralia;Lophotrochozoa;Mollusca;Bivalvia;Autobranchia;Heteroconchia;Euheterodonta;Imparidentia;Venerida;Veneroidea;Veneridae;Mercenaria;Mercenaria mercenaria
UdeM00000000340 6596 976.1 UdeM00000000340 XP_045168803.1 45.7 1117
550 16 18 1092 132 1233 3.70E-281 976.1 6596 cellular organisms;Eukaryota;Opisthokonta;Metazoa;Eumetazoa;Bilateria;Protostomia;Spiralia;Lophotrochozoa;Mollusca;Bivalvia;Autobranchia;Heteroconchia;Euheterodonta;Imparidentia;Venerida;Veneroidea;Veneridae;Mercenaria;Mercenaria mercenaria
UdeM00000000341 151771 240 UdeM00000000341 XP_028406951.1 34
441 242 7 3 397 148 585 5.50E-60 240 151771
cellular organisms;Eukaryota;Opisthokonta;Metazoa;Eumetazoa;Cnidaria;Anthozoa;Octocorallia;Alcyonacea;Alcyoniina;Nephtheidae;Dendronephthya;Dendronephthya gigantea
UdeM00000000344 451745 96.7 UdeM00000000344 XP_037534902.1 24.6
521 316 14 536 1022 170 647 3.00E-16 96.7 451745
cellular organisms;Eukaryota;Opisthokonta;Metazoa;Eumetazoa;Bilateria;Deuterostomia;Chordata;Craniata;Vertebrata;Gnathostomata;Teleostomi;Euteleostomi;Actinopterygii;Actinopteri;Neopterygii;Teleostei;Osteoglossocephalai;Clupeocephala;Euteleosteomorpha;Neoteleostei;Eurypterygia;Ctenosquamata;Acanthomorphata;Euacanthomorphacea;Percomorphaceae;Ovalentaria;Atherinomorphae;Cyprinodontiformes;Aplocheiloidei;Rivulidae;Nematolebias;Nematolebias whitei
UdeM00000000345 6596 66.2 UdeM00000000345 XP_045169919.1 63.2 144
23 2 42 155 63 206 4.10E-08 66.2 6596 cellular organisms;Eukaryota;Opisthokonta;Metazoa;Eumetazoa;Bilateria;Protostomia;Spiralia;Lophotrochozoa;Mollusca;Bivalvia;Autobranchia;Heteroconchia;Euheterodonta;Imparidentia;Venerida;Veneroidea;Veneridae;Mercenaria;Mercenaria mercenaria
UdeM00000000346 6596 114 UdeM00000000346 XP_045169918.1 70.5 149
40 2 36 182 63 209 2.00E-22 114 6596 cellular organisms;Eukaryota;Opisthokonta;Metazoa;Eumetazoa;Bilateria;Protostomia;Spiralia;Lophotrochozoa;Mollusca;Bivalvia;Autobranchia;Heteroconchia;Euheterodonta;Imparidentia;Venerida;Veneroidea;Veneridae;Mercenaria;Mercenaria mercenaria
UdeM00000000349 6412 184.1 UdeM00000000349 XP_009025211.1 98.9 89
1 0 1 89 1 89 7.80E-44 184.1 6412 cellular organisms;Eukaryota;Opisthokonta;Metazoa;Eumetazoa;Bilateria;Protostomia;Spiralia;Lophotrochozoa;Annelida;Clitellata;Hirudinea;Rhynchobdellida;Glossiphoniidae;Helobdella;Helobdella robusta
UdeM00000000351 6573 160.2 UdeM00000000351 XP_021350458.1 44.3 194
101 2 173 360 253 445 6.10E-36 160.2 6573 cellular

organisms;Eukaryota;Opisthokonta;Metazoa;Eumetazoa;Bilateria;Protostomia;
Spiralia;Lophotrochozoa;Mollusca;Bivalvia;Autobranchia;Pteriomorphia;Pect
inida;Pectinoidea;Pectinidae;Mizuhopecten;Mizuhopecten yessoensis
UdeM00000000353 6596 123.2 UdeM00000000353 XP_045169387.1 69.3 88
26 1 54 140 106 193 3.00E-25 123.2 6596 cellular
organisms;Eukaryota;Opisthokonta;Metazoa;Eumetazoa;Bilateria;Protostomia;
Spiralia;Lophotrochozoa;Mollusca;Bivalvia;Autobranchia;Heteroconchia;Euhe
terodonta;Imparidentia;Venerida;Veneroidea;Veneridae;Mercenaria;Mercenari
a mercenaria
UdeM00000000354 6579 204.9 UdeM00000000354 XP_033729790.1 39.8 274
160 2 10 283 17 285 2.50E-49 204.9 6579 cellular
organisms;Eukaryota;Opisthokonta;Metazoa;Eumetazoa;Bilateria;Protostomia;
Spiralia;Lophotrochozoa;Mollusca;Bivalvia;Autobranchia;Pteriomorphia;Pect
inida;Pectinoidea;Pectinidae;Pecten;Pecten maximus
UdeM00000000357 6596 518.5 UdeM00000000357 XP_045169387.1 64.3 389
135 1 4 388 193 581 7.70E-144 518.5 6596 cellular
organisms;Eukaryota;Opisthokonta;Metazoa;Eumetazoa;Bilateria;Protostomia;
Spiralia;Lophotrochozoa;Mollusca;Bivalvia;Autobranchia;Heteroconchia;Euhe
terodonta;Imparidentia;Venerida;Veneroidea;Veneridae;Mercenaria;Mercenari
a mercenaria
UdeM00000000358 6526 347.4 UdeM00000000358 XP_013079593.1 52.6 323
144 3 2 319 90 408 1.90E-92 347.4 6526 cellular
organisms;Eukaryota;Opisthokonta;Metazoa;Eumetazoa;Bilateria;Protostomia;
Spiralia;Lophotrochozoa;Mollusca;Gastropoda;Heterobranchia;Euthyneura;Pan
pulmonata;Hygrophila;Lymnaeoidea;Planorbidae;Biomphalaria;Biomphalaria
glabrata
UdeM00000000360 6596 295.8 UdeM00000000360 XP_045168877.1 34.4 572
337 9 1 553 1 553 1.30E-76 295.8 6596 cellular
organisms;Eukaryota;Opisthokonta;Metazoa;Eumetazoa;Bilateria;Protostomia;
Spiralia;Lophotrochozoa;Mollusca;Bivalvia;Autobranchia;Heteroconchia;Euhe
terodonta;Imparidentia;Venerida;Veneroidea;Veneridae;Mercenaria;Mercenari
a mercenaria
UdeM00000000361 6596 471.5 UdeM00000000361 XP_045167594.1 54.1 427
145 3 1 376 43 469 1.20E-129 471.5 6596 cellular
organisms;Eukaryota;Opisthokonta;Metazoa;Eumetazoa;Bilateria;Protostomia;
Spiralia;Lophotrochozoa;Mollusca;Bivalvia;Autobranchia;Heteroconchia;Euhe
terodonta;Imparidentia;Venerida;Veneroidea;Veneridae;Mercenaria;Mercenari
a mercenaria
UdeM00000000362 6596 6378.1 UdeM00000000362 XP_045170173.1 84.1
3887 570 14 8 3880 47 3899 0.00E+00 6378.1 6596
cellular
organisms;Eukaryota;Opisthokonta;Metazoa;Eumetazoa;Bilateria;Protostomia;
Spiralia;Lophotrochozoa;Mollusca;Bivalvia;Autobranchia;Heteroconchia;Euhe
terodonta;Imparidentia;Venerida;Veneroidea;Veneridae;Mercenaria;Mercenari
a mercenaria
UdeM00000000363 29159 194.9 UdeM00000000363 XP_034315421.1 51.9 206
93 3 122 323 271 474 1.60E-46 194.9 29159 cellular
organisms;Eukaryota;Opisthokonta;Metazoa;Eumetazoa;Bilateria;Protostomia;
Spiralia;Lophotrochozoa;Mollusca;Bivalvia;Autobranchia;Pteriomorphia;Ostr
eida;Ostreoidea;Ostreidae;Crassostrea;Crassostrea gigas
UdeM00000000364 6565 149.4 UdeM00000000364 XP_022306954.1 52.9 121
57 0 126 246 828 948 5.80E-33 149.4 6565 cellular
organisms;Eukaryota;Opisthokonta;Metazoa;Eumetazoa;Bilateria;Protostomia;

Spiralia;Lophotrochozoa;Mollusca;Bivalvia;Autobranchia;Pteriomorphia;Ostreida;Ostreoidea;Ostreidae;Crassostrea;Crassostrea virginica
UdeM00000000365 29159 383.6 UdeM00000000365 XP_034318666.1 60.1 288
115 0 1 288 482 769 2.30E-103 383.6 29159 cellular organisms;Eukaryota;Opisthokonta;Metazoa;Eumetazoa;Bilateria;Protostomia;Spiralia;Lophotrochozoa;Mollusca;Bivalvia;Autobranchia;Pteriomorphia;Ostreida;Ostreoidea;Ostreidae;Crassostrea;Crassostrea gigas
UdeM00000000366 37653 152.9 UdeM00000000366 XP_014777319.1 52.2 134
62 1 9 140 27 160 3.40E-34 152.9 37653 cellular organisms;Eukaryota;Opisthokonta;Metazoa;Eumetazoa;Bilateria;Protostomia;Spiralia;Lophotrochozoa;Mollusca;Cephalopoda;Coleoidea;Octopodiformes;Octopoda;Incirrata;Octopodidae;Octopus;Octopus bimaculoides
UdeM00000000367 6565 280 UdeM00000000367 XP_022326235.1 42.9 310
174 2 12 321 138 444 3.70E-72 280 6565 cellular organisms;Eukaryota;Opisthokonta;Metazoa;Eumetazoa;Bilateria;Protostomia;Spiralia;Lophotrochozoa;Mollusca;Bivalvia;Autobranchia;Pteriomorphia;Ostreida;Ostreoidea;Ostreidae;Crassostrea;Crassostrea virginica
UdeM00000000368 6596 194.5 UdeM00000000368 XP_045168504.1 29.8 876
403 34 3 720 11 832 1.00E-45 194.5 6596 cellular organisms;Eukaryota;Opisthokonta;Metazoa;Eumetazoa;Bilateria;Protostomia;Spiralia;Lophotrochozoa;Mollusca;Bivalvia;Autobranchia;Heteroconchia;Euheterodonta;Imparidentia;Venerida;Veneroidea;Veneridae;Mercenaria;Mercenaria mercenaria
UdeM00000000373 7604 303.5 UdeM00000000373 XP_033633146.1 41 390
212 3 262 633 29 418 7.00E-79 303.5 7604 cellular organisms;Eukaryota;Opisthokonta;Metazoa;Eumetazoa;Bilateria;Deuterostomia;Echinodermata;Eleutherozoa;Asterozoa;Asteroidea;Forcipulatacea;Forcipulatida;Asteriidae;Asterias;Asterias rubens
UdeM00000000375 6596 547 UdeM00000000375 XP_045213752.1 47.2 589
281 3 1 588 83 642 5.70E-152 547 6596 cellular organisms;Eukaryota;Opisthokonta;Metazoa;Eumetazoa;Bilateria;Protostomia;Spiralia;Lophotrochozoa;Mollusca;Bivalvia;Autobranchia;Heteroconchia;Euheterodonta;Imparidentia;Venerida;Veneroidea;Veneridae;Mercenaria;Mercenaria mercenaria
UdeM00000000376 6596 151.8 UdeM00000000376 XP_045188603.1 28.4 464
287 17 499 935 361 806 4.70E-33 151.8 6596 cellular organisms;Eukaryota;Opisthokonta;Metazoa;Eumetazoa;Bilateria;Protostomia;Spiralia;Lophotrochozoa;Mollusca;Bivalvia;Autobranchia;Heteroconchia;Euheterodonta;Imparidentia;Venerida;Veneroidea;Veneridae;Mercenaria;Mercenaria mercenaria
UdeM00000000377 6596 311.2 UdeM00000000377 XP_045169811.1 35.8 492
298 9 38 516 25 511 2.50E-81 311.2 6596 cellular organisms;Eukaryota;Opisthokonta;Metazoa;Eumetazoa;Bilateria;Protostomia;Spiralia;Lophotrochozoa;Mollusca;Bivalvia;Autobranchia;Heteroconchia;Euheterodonta;Imparidentia;Venerida;Veneroidea;Veneridae;Mercenaria;Mercenaria mercenaria
UdeM00000000378 6596 361.7 UdeM00000000378 XP_045167569.1 44.7 376
199 4 14 381 38 412 1.20E-96 361.7 6596 cellular organisms;Eukaryota;Opisthokonta;Metazoa;Eumetazoa;Bilateria;Protostomia;Spiralia;Lophotrochozoa;Mollusca;Bivalvia;Autobranchia;Heteroconchia;Euheterodonta;Imparidentia;Venerida;Veneroidea;Veneridae;Mercenaria;Mercenaria mercenaria
UdeM00000000379 6596 124 UdeM00000000379 XP_045167569.1 55 120
49 2 4 120 424 541 1.50E-25 124 6596 cellular

organisms;Eukaryota;Opisthokonta;Metazoa;Eumetazoa;Bilateria;Protostomia;Spiralia;Lophotrochozoa;Mollusca;Bivalvia;Autobranchia;Heteroconchia;Euheterodonta;Imparidentia;Venerida;Veneroidea;Veneridae;Mercenaria;Mercenaria mercenaria
UdeM00000000380 6596 591.3 UdeM00000000380 XP_045168540.1 39.6 849 400 17 35 791 117 944 1.90E-165 591.3 6596 cellular organisms;Eukaryota;Opisthokonta;Metazoa;Eumetazoa;Bilateria;Protostomia;Spiralia;Lophotrochozoa;Mollusca;Bivalvia;Autobranchia;Heteroconchia;Euheterodonta;Imparidentia;Venerida;Veneroidea;Veneridae;Mercenaria;Mercenaria mercenaria
UdeM00000000382 400727 916.4 UdeM00000000382 XP_025093892.1 69.9 638 180 5 5 633 2 636 2.00E-263 916.4 400727 cellular organisms;Eukaryota;Opisthokonta;Metazoa;Eumetazoa;Bilateria;Protostomia;Spiralia;Lophotrochozoa;Mollusca;Gastropoda;Caenogastropoda;Architaenioglossa;Ampullarioidea;Ampullariidae;Pomacea;Pomacea canaliculata
UdeM00000000383 6596 67.8 UdeM00000000383 XP_045169096.1 43.2 88 41 2 35 122 426 504 1.20E-08 67.8 6596 cellular organisms;Eukaryota;Opisthokonta;Metazoa;Eumetazoa;Bilateria;Protostomia;Spiralia;Lophotrochozoa;Mollusca;Bivalvia;Autobranchia;Heteroconchia;Euheterodonta;Imparidentia;Venerida;Veneroidea;Veneridae;Mercenaria;Mercenaria mercenaria
UdeM00000000385 6596 166.8 UdeM00000000385 XP_045169096.1 62.1 132 34 2 1 131 194 310 1.90E-38 166.8 6596 cellular organisms;Eukaryota;Opisthokonta;Metazoa;Eumetazoa;Bilateria;Protostomia;Spiralia;Lophotrochozoa;Mollusca;Bivalvia;Autobranchia;Heteroconchia;Euheterodonta;Imparidentia;Venerida;Veneroidea;Veneridae;Mercenaria;Mercenaria mercenaria
UdeM00000000384 6596 298.9 UdeM00000000384 XP_045169096.1 67 206 66 2 19 222 105 310 5.40E-78 298.9 6596 cellular organisms;Eukaryota;Opisthokonta;Metazoa;Eumetazoa;Bilateria;Protostomia;Spiralia;Lophotrochozoa;Mollusca;Bivalvia;Autobranchia;Heteroconchia;Euheterodonta;Imparidentia;Venerida;Veneroidea;Veneridae;Mercenaria;Mercenaria mercenaria
UdeM00000000386 6596 108.6 UdeM00000000386 XP_045170409.1 40.2 326 139 7 1 321 1 275 1.50E-20 108.6 6596 cellular organisms;Eukaryota;Opisthokonta;Metazoa;Eumetazoa;Bilateria;Protostomia;Spiralia;Lophotrochozoa;Mollusca;Bivalvia;Autobranchia;Heteroconchia;Euheterodonta;Imparidentia;Venerida;Veneroidea;Veneridae;Mercenaria;Mercenaria mercenaria
UdeM00000000387 6596 309.7 UdeM00000000387 XP_045169621.1 76.6 201 43 2 1 200 1 198 2.70E-81 309.7 6596 cellular organisms;Eukaryota;Opisthokonta;Metazoa;Eumetazoa;Bilateria;Protostomia;Spiralia;Lophotrochozoa;Mollusca;Bivalvia;Autobranchia;Heteroconchia;Euheterodonta;Imparidentia;Venerida;Veneroidea;Veneridae;Mercenaria;Mercenaria mercenaria
UdeM00000000388 218467 207.2 UdeM00000000388 XP_023229324.1 86.8 114 15 0 1 114 1 114 1.10E-50 207.2 218467 cellular organisms;Eukaryota;Opisthokonta;Metazoa;Eumetazoa;Bilateria;Protostomia;Ecdysozoa;Panarthropoda;Arthropoda;Chelicerata;Arachnida;Scorpiones;Buthida;Buthoidea;Buthidae;Centruroides;Centruroides sculpturatus
UdeM00000000389 6596 319.7 UdeM00000000389 XP_045168366.1 48.1 308 159 1 10 316 11 318 4.20E-84 319.7 6596 cellular

organisms;Eukaryota;Opisthokonta;Metazoa;Eumetazoa;Bilateria;Protostomia;Spiralia;Lophotrochozoa;Mollusca;Bivalvia;Autobranchia;Heteroconchia;Euheterodonta;Imparidentia;Venerida;Veneroidea;Veneridae;Mercenaria;Mercenaria mercenaria
UdeM00000000390 6596 1060.8 UdeM00000000390 XP_045169674.1 66.4 821 212 5 1 782 368 1163 8.30E-307 1060.8 6596 cellular organisms;Eukaryota;Opisthokonta;Metazoa;Eumetazoa;Bilateria;Protostomia;Spiralia;Lophotrochozoa;Mollusca;Bivalvia;Autobranchia;Heteroconchia;Euheterodonta;Imparidentia;Venerida;Veneroidea;Veneridae;Mercenaria;Mercenaria mercenaria
UdeM00000000391 6579 189.5 UdeM00000000391 XP_033762111.1 44.3 255 132 4 48 296 24 274 6.10E-45 189.5 6579 cellular organisms;Eukaryota;Opisthokonta;Metazoa;Eumetazoa;Bilateria;Protostomia;Spiralia;Lophotrochozoa;Mollusca;Bivalvia;Autobranchia;Pteriomorphia;Pectinida;Pectinoidea;Pectinidae;Pecten;Pecten maximus
UdeM00000000392 471704 149.4 UdeM00000000392 XP_018365076.1 35.2 256 98 2 1 188 233 488 4.50E-33 149.4 471704 cellular organisms;Eukaryota;Opisthokonta;Metazoa;Eumetazoa;Bilateria;Protostomia;Ecdysozoa;Panarthropoda;Arthropoda;Mandibulata;Pancrustacea;Hexapoda;Insecta;Dicondylia;Pterygota;Neoptera;Endopterygota;Hymenoptera;Apocrita;Aculeata;Formicoidea;Formicidae;Myrmicinae;Attini;Trachymyrmex;Trachymyrmex cornetzi
UdeM00000000393 6565 304.7 UdeM00000000393 XP_022306142.1 55 289 119 4 1 279 276 563 1.20E-79 304.7 6565 cellular organisms;Eukaryota;Opisthokonta;Metazoa;Eumetazoa;Bilateria;Protostomia;Spiralia;Lophotrochozoa;Mollusca;Bivalvia;Autobranchia;Pteriomorphia;Ostreida;Ostreoidea;Ostreidae;Crassostrea;Crassostrea virginica
UdeM00000000394 29159 548.1 UdeM00000000394 XP_034309569.1 54.6 538 217 7 6 522 30 561 1.20E-152 548.1 29159 cellular organisms;Eukaryota;Opisthokonta;Metazoa;Eumetazoa;Bilateria;Protostomia;Spiralia;Lophotrochozoa;Mollusca;Bivalvia;Autobranchia;Pteriomorphia;Ostreida;Ostreoidea;Ostreidae;Crassostrea;Crassostrea gigas
UdeM00000000395 6596 69.7 UdeM00000000395 XP_045169584.1 37.1 89 49 3 1 83 75 162 2.30E-09 69.7 6596 cellular organisms;Eukaryota;Opisthokonta;Metazoa;Eumetazoa;Bilateria;Protostomia;Spiralia;Lophotrochozoa;Mollusca;Bivalvia;Autobranchia;Heteroconchia;Euheterodonta;Imparidentia;Venerida;Veneroidea;Veneridae;Mercenaria;Mercenaria mercenaria
UdeM00000000396 6596 498.4 UdeM00000000396 XP_045181503.1 32 968 594 23 14 952 113 1045 2.00E-137 498.4 6596 cellular organisms;Eukaryota;Opisthokonta;Metazoa;Eumetazoa;Bilateria;Protostomia;Spiralia;Lophotrochozoa;Mollusca;Bivalvia;Autobranchia;Heteroconchia;Euheterodonta;Imparidentia;Venerida;Veneroidea;Veneridae;Mercenaria;Mercenaria mercenaria
UdeM00000000397 6596 501.5 UdeM00000000397 XP_045181503.1 32.2 970 592 25 14 954 113 1045 2.40E-138 501.5 6596 cellular organisms;Eukaryota;Opisthokonta;Metazoa;Eumetazoa;Bilateria;Protostomia;Spiralia;Lophotrochozoa;Mollusca;Bivalvia;Autobranchia;Heteroconchia;Euheterodonta;Imparidentia;Venerida;Veneroidea;Veneridae;Mercenaria;Mercenaria mercenaria
UdeM00000000399 6596 337.4 UdeM00000000399 XP_045169584.1 39 480 272 10 4 478 60 523 3.10E-89 337.4 6596 cellular

organisms;Eukaryota;Opisthokonta;Metazoa;Eumetazoa;Bilateria;Protostomia;Spiralia;Lophotrochozoa;Mollusca;Bivalvia;Autobranchia;Heteroconchia;Euheterodonta;Imparidentia;Venerida;Veneroidea;Veneridae;Mercenaria;Mercenaria mercenaria
UdeM00000000400 6596 325.5 UdeM00000000400 XP_045169584.1 36.6 511 272 10 4 509 60 523 1.30E-85 325.5 6596 cellular organisms;Eukaryota;Opisthokonta;Metazoa;Eumetazoa;Bilateria;Protostomia;Spiralia;Lophotrochozoa;Mollusca;Bivalvia;Autobranchia;Heteroconchia;Euheterodonta;Imparidentia;Venerida;Veneroidea;Veneridae;Mercenaria;Mercenaria mercenaria
UdeM00000000401 6596 212.2 UdeM00000000401 XP_045181503.1 28.8 510 334 10 47 539 544 1041 1.70E-51 212.2 6596 cellular organisms;Eukaryota;Opisthokonta;Metazoa;Eumetazoa;Bilateria;Protostomia;Spiralia;Lophotrochozoa;Mollusca;Bivalvia;Autobranchia;Heteroconchia;Euheterodonta;Imparidentia;Venerida;Veneroidea;Veneridae;Mercenaria;Mercenaria mercenaria
UdeM00000000402 6596 553.5 UdeM00000000402 XP_045167908.1 33 1001 612 23 3 964 24 1004 5.70E-154 553.5 6596 cellular organisms;Eukaryota;Opisthokonta;Metazoa;Eumetazoa;Bilateria;Protostomia;Spiralia;Lophotrochozoa;Mollusca;Bivalvia;Autobranchia;Heteroconchia;Euheterodonta;Imparidentia;Venerida;Veneroidea;Veneridae;Mercenaria;Mercenaria mercenaria
UdeM00000000403 6596 419.5 UdeM00000000403 XP_045169585.1 60.3 315 125 0 1 315 1 315 3.90E-114 419.5 6596 cellular organisms;Eukaryota;Opisthokonta;Metazoa;Eumetazoa;Bilateria;Protostomia;Spiralia;Lophotrochozoa;Mollusca;Bivalvia;Autobranchia;Heteroconchia;Euheterodonta;Imparidentia;Venerida;Veneroidea;Veneridae;Mercenaria;Mercenaria mercenaria
UdeM00000000404 6596 927.9 UdeM00000000404 XP_045166183.1 100 451 0 0 1 451 1 451 4.80E-267 927.9 6596 cellular organisms;Eukaryota;Opisthokonta;Metazoa;Eumetazoa;Bilateria;Protostomia;Spiralia;Lophotrochozoa;Mollusca;Bivalvia;Autobranchia;Heteroconchia;Euheterodonta;Imparidentia;Venerida;Veneroidea;Veneridae;Mercenaria;Mercenaria mercenaria
UdeM00000000405 6596 1543.1 UdeM00000000405 XP_045170179.1 75.3 983 237 3 18 995 30 1011 0.00E+00 1543.1 6596 cellular organisms;Eukaryota;Opisthokonta;Metazoa;Eumetazoa;Bilateria;Protostomia;Spiralia;Lophotrochozoa;Mollusca;Bivalvia;Autobranchia;Heteroconchia;Euheterodonta;Imparidentia;Venerida;Veneroidea;Veneridae;Mercenaria;Mercenaria mercenaria
UdeM00000000413 6579 439.1 UdeM00000000413 XP_033761282.1 70 303 75 3 1 296 1 294 4.40E-120 439.1 6579 cellular organisms;Eukaryota;Opisthokonta;Metazoa;Eumetazoa;Bilateria;Protostomia;Spiralia;Lophotrochozoa;Mollusca;Bivalvia;Autobranchia;Pteriomorphia;Pectinida;Pectinoidea;Pectinidae;Pecten;Pecten maximus
UdeM00000000414 6596 739.6 UdeM00000000414 XP_045168279.1 85.2 420 62 0 1 420 1 420 2.30E-210 739.6 6596 cellular organisms;Eukaryota;Opisthokonta;Metazoa;Eumetazoa;Bilateria;Protostomia;Spiralia;Lophotrochozoa;Mollusca;Bivalvia;Autobranchia;Heteroconchia;Euheterodonta;Imparidentia;Venerida;Veneroidea;Veneridae;Mercenaria;Mercenaria mercenaria
UdeM00000000415 6596 685.3 UdeM00000000415 XP_045170358.1 85.3 416 61 0 6 421 3 418 5.00E-194 685.3 6596 cellular

organisms;Eukaryota;Opisthokonta;Metazoa;Eumetazoa;Bilateria;Protostomia;Spiralia;Lophotrochozoa;Mollusca;Bivalvia;Autobranchia;Heteroconchia;Euheterodonta;Imparidentia;Venerida;Veneroidea;Veneridae;Mercenaria;Mercenaria mercenaria
UdeM00000000416 6579 142.1 UdeM00000000416 XP_033761302.1 76 100 24 0 7 106 4 103 4.20E-31 142.1 6579 cellular organisms;Eukaryota;Opisthokonta;Metazoa;Eumetazoa;Bilateria;Protostomia;Spiralia;Lophotrochozoa;Mollusca;Bivalvia;Autobranchia;Pteriomorphia;Pectinida;Pectinoidea;Pectinidae;Pecten;Pecten maximus
UdeM00000000417 6596 464.9 UdeM00000000417 XP_045167959.1 66.3 338 111 2 1 335 1 338 8.50E-128 464.9 6596 cellular organisms;Eukaryota;Opisthokonta;Metazoa;Eumetazoa;Bilateria;Protostomia;Spiralia;Lophotrochozoa;Mollusca;Bivalvia;Autobranchia;Heteroconchia;Euheterodonta;Imparidentia;Venerida;Veneroidea;Veneridae;Mercenaria;Mercenaria mercenaria
UdeM00000000418 6596 330.1 UdeM00000000418 XP_045168165.1 70.7 229 49 1 227 455 8 218 4.50E-87 330.1 6596 cellular organisms;Eukaryota;Opisthokonta;Metazoa;Eumetazoa;Bilateria;Protostomia;Spiralia;Lophotrochozoa;Mollusca;Bivalvia;Autobranchia;Heteroconchia;Euheterodonta;Imparidentia;Venerida;Veneroidea;Veneridae;Mercenaria;Mercenaria mercenaria
UdeM00000000420 6596 423.7 UdeM00000000420 XP_045167906.1 48.5 507 214 12 4 467 14 516 3.00E-115 423.7 6596 cellular organisms;Eukaryota;Opisthokonta;Metazoa;Eumetazoa;Bilateria;Protostomia;Spiralia;Lophotrochozoa;Mollusca;Bivalvia;Autobranchia;Heteroconchia;Euheterodonta;Imparidentia;Venerida;Veneroidea;Veneridae;Mercenaria;Mercenaria mercenaria
UdeM00000000421 29159 411 UdeM00000000421 XP_034309544.1 48.8 455 192 10 1 453 13 428 2.00E-111 411 29159 cellular organisms;Eukaryota;Opisthokonta;Metazoa;Eumetazoa;Bilateria;Protostomia;Spiralia;Lophotrochozoa;Mollusca;Bivalvia;Autobranchia;Pteriomorphia;Ostreida;Ostreoidea;Ostreidae;Crassostrea;Crassostrea gigas
UdeM00000000422 6596 422.2 UdeM00000000422 XP_045167906.1 47.9 507 207 11 4 457 14 516 8.60E-115 422.2 6596 cellular organisms;Eukaryota;Opisthokonta;Metazoa;Eumetazoa;Bilateria;Protostomia;Spiralia;Lophotrochozoa;Mollusca;Bivalvia;Autobranchia;Heteroconchia;Euheterodonta;Imparidentia;Venerida;Veneroidea;Veneridae;Mercenaria;Mercenaria mercenaria
UdeM00000000424 1735272 212.2 UdeM00000000424 XP_041367509.1 79.7 123 21 1 127 245 1 123 8.90E-52 212.2 1735272 cellular organisms;Eukaryota;Opisthokonta;Metazoa;Eumetazoa;Bilateria;Protostomia;Spiralia;Lophotrochozoa;Mollusca;Gastropoda;Neomphalina;Neomphaloidea;Peltospiridae;Gigantopelta;Gigantopelta aegis
UdeM00000000425 6579 359 UdeM00000000425 XP_033761765.1 25.4 1609 867 38 9 1395 2 1499 2.80E-95 359 6579 cellular organisms;Eukaryota;Opisthokonta;Metazoa;Eumetazoa;Bilateria;Protostomia;Spiralia;Lophotrochozoa;Mollusca;Bivalvia;Autobranchia;Pteriomorphia;Pectinida;Pectinoidea;Pectinidae;Pecten;Pecten maximus
UdeM00000000426 6579 220.3 UdeM00000000426 XP_033762438.1 43.3 342 171 7 6 342 4 327 4.00E-54 220.3 6579 cellular organisms;Eukaryota;Opisthokonta;Metazoa;Eumetazoa;Bilateria;Protostomia;Spiralia;Lophotrochozoa;Mollusca;Bivalvia;Autobranchia;Pteriomorphia;Pectinida;Pectinoidea;Pectinidae;Pecten;Pecten maximus

UdeM00000000427 6596 542 UdeM00000000427 XP_045170013.1 55.7 551
225 10 18 556 392 935 9.10E-151 542 6596 cellular
organisms;Eukaryota;Opisthokonta;Metazoa;Eumetazoa;Bilateria;Protostomia;
Spiralia;Lophotrochozoa;Mollusca;Bivalvia;Autobranchia;Heteroconchia;Euhe
terodonta;Imparidentia;Venerida;Veneroidea;Veneridae;Mercenaria;Mercenari
a mercenaria
UdeM00000000428 1735272 149.1 UdeM00000000428 XP_041349588.1 55
120 54 0 10 129 65 184 4.20E-33 149.1 1735272
cellular
organisms;Eukaryota;Opisthokonta;Metazoa;Eumetazoa;Bilateria;Protostomia;
Spiralia;Lophotrochozoa;Mollusca;Gastropoda;Neomphalina;Neomphaloidea;Pel
tospiridae;Gigantopelta;Gigantopelta aegis
UdeM00000000429 1735272 144.1 UdeM00000000429 XP_041357032.1 56.3
119 48 1 8 126 63 177 1.70E-31 144.1 1735272
cellular
organisms;Eukaryota;Opisthokonta;Metazoa;Eumetazoa;Bilateria;Protostomia;
Spiralia;Lophotrochozoa;Mollusca;Gastropoda;Neomphalina;Neomphaloidea;Pel
tospiridae;Gigantopelta;Gigantopelta aegis
UdeM00000000431 1735272 159.1 UdeM00000000431 XP_041367303.1 22.6
1104 650 31 6 1035 12 985 3.30E-35 159.1 1735272
cellular
organisms;Eukaryota;Opisthokonta;Metazoa;Eumetazoa;Bilateria;Protostomia;
Spiralia;Lophotrochozoa;Mollusca;Gastropoda;Neomphalina;Neomphaloidea;Pel
tospiridae;Gigantopelta;Gigantopelta aegis
UdeM00000000432 6573 237.3 UdeM00000000432 XP_021375283.1 31.2 541
319 8 5 530 756 1258 4.80E-59 237.3 6573 cellular
organisms;Eukaryota;Opisthokonta;Metazoa;Eumetazoa;Bilateria;Protostomia;
Spiralia;Lophotrochozoa;Mollusca;Bivalvia;Autobranchia;Pteriomorphia;Pect
inida;Pectinoidea;Pectinidae;Mizuhopecten;Mizuhopecten yessoensis
UdeM00000000433 29159 111.3 UdeM00000000433 XP_011421628.2 28.4 313
189 9 36 324 558 859 2.40E-21 111.3 29159 cellular
organisms;Eukaryota;Opisthokonta;Metazoa;Eumetazoa;Bilateria;Protostomia;
Spiralia;Lophotrochozoa;Mollusca;Bivalvia;Autobranchia;Pteriomorphia;Ostr
eida;Ostreoidea;Ostreidae;Crassostrea;Crassostrea gigas
UdeM00000000434 6573 235.7 UdeM00000000434 XP_021375283.1 31.2 539
338 10 5 538 756 1266 1.40E-58 235.7 6573 cellular
organisms;Eukaryota;Opisthokonta;Metazoa;Eumetazoa;Bilateria;Protostomia;
Spiralia;Lophotrochozoa;Mollusca;Bivalvia;Autobranchia;Pteriomorphia;Pect
inida;Pectinoidea;Pectinidae;Mizuhopecten;Mizuhopecten yessoensis
UdeM00000000435 29159 88.2 UdeM00000000435 XP_019928550.2 43 86
49 0 46 131 173 258 1.10E-14 88.2 29159 cellular
organisms;Eukaryota;Opisthokonta;Metazoa;Eumetazoa;Bilateria;Protostomia;
Spiralia;Lophotrochozoa;Mollusca;Bivalvia;Autobranchia;Pteriomorphia;Ostr
eida;Ostreoidea;Ostreidae;Crassostrea;Crassostrea gigas
UdeM00000000436 6573 69.3 UdeM00000000436 XP_021375283.1 33.3 90
49 2 36 124 244 323 4.60E-09 69.3 6573 cellular
organisms;Eukaryota;Opisthokonta;Metazoa;Eumetazoa;Bilateria;Protostomia;
Spiralia;Lophotrochozoa;Mollusca;Bivalvia;Autobranchia;Pteriomorphia;Pect
inida;Pectinoidea;Pectinidae;Mizuhopecten;Mizuhopecten yessoensis
UdeM00000000438 6573 228.8 UdeM00000000438 XP_021358437.1 29.8 557
350 12 2 546 756 1283 1.70E-56 228.8 6573 cellular
organisms;Eukaryota;Opisthokonta;Metazoa;Eumetazoa;Bilateria;Protostomia;
Spiralia;Lophotrochozoa;Mollusca;Bivalvia;Autobranchia;Pteriomorphia;Pect
inida;Pectinoidea;Pectinidae;Mizuhopecten;Mizuhopecten yessoensis

UdeM00000000439 7604 462.6 UdeM00000000439 XP_033641066.1 56.3 378
165 0 4 381 3 380 4.90E-127 462.6 7604 cellular
organisms;Eukaryota;Opisthokonta;Metazoa;Eumetazoa;Bilateria;Deuterostomia;Echinodermata;Eleutherozoa;Asterozoa;Asteroidea;Forcipulatacea;Forcipulatida;Asteriidae;Asterias;Asterias rubens
UdeM00000000443 6573 432.6 UdeM00000000443 XP_021375283.1 26.8 1328
838 33 38 1327 49 1280 1.90E-117 432.6 6573 cellular
organisms;Eukaryota;Opisthokonta;Metazoa;Eumetazoa;Bilateria;Protostomia;Spiralia;Lophotrochozoa;Mollusca;Bivalvia;Autobranchia;Pteriomorphia;Pectinida;Pectinoidea;Pectinidae;Mizuhopecten;Mizuhopecten yessoensis
UdeM00000000444 6573 132.1 UdeM00000000444 XP_021353057.1 61.6 99
38 0 1 99 1 99 4.70E-28 132.1 6573 cellular
organisms;Eukaryota;Opisthokonta;Metazoa;Eumetazoa;Bilateria;Protostomia;Spiralia;Lophotrochozoa;Mollusca;Bivalvia;Autobranchia;Pteriomorphia;Pectinida;Pectinoidea;Pectinidae;Mizuhopecten;Mizuhopecten yessoensis
UdeM00000000445 6565 249.2 UdeM00000000445 XP_022343208.1 31.2 560
327 21 24 543 21 562 1.20E-62 249.2 6565 cellular
organisms;Eukaryota;Opisthokonta;Metazoa;Eumetazoa;Bilateria;Protostomia;Spiralia;Lophotrochozoa;Mollusca;Bivalvia;Autobranchia;Pteriomorphia;Ostreida;Ostreoidea;Ostreidae;Crassostrea;Crassostrea virginica
UdeM00000000446 29159 508.8 UdeM00000000446 XP_034310304.1 82.6 299
52 0 1 299 1 299 4.60E-141 508.8 29159 cellular
organisms;Eukaryota;Opisthokonta;Metazoa;Eumetazoa;Bilateria;Protostomia;Spiralia;Lophotrochozoa;Mollusca;Bivalvia;Autobranchia;Pteriomorphia;Ostreida;Ostreoidea;Ostreidae;Crassostrea;Crassostrea gigas
UdeM00000000447 6579 75.9 UdeM00000000447 XP_033762025.1 32.1 212
118 9 3 200 43 242 7.20E-11 75.9 6579 cellular
organisms;Eukaryota;Opisthokonta;Metazoa;Eumetazoa;Bilateria;Protostomia;Spiralia;Lophotrochozoa;Mollusca;Bivalvia;Autobranchia;Pteriomorphia;Pectinida;Pectinoidea;Pectinidae;Pecten;Pecten maximus
UdeM00000000448 6565 726.9 UdeM00000000448 XP_022309008.1 90.7 389
36 0 77 465 132 520 1.70E-206 726.9 6565 cellular
organisms;Eukaryota;Opisthokonta;Metazoa;Eumetazoa;Bilateria;Protostomia;Spiralia;Lophotrochozoa;Mollusca;Bivalvia;Autobranchia;Pteriomorphia;Ostreida;Ostreoidea;Ostreidae;Crassostrea;Crassostrea virginica
UdeM00000000449 29159 275 UdeM00000000449 XP_034329844.1 32.6 503
313 7 32 519 5 496 1.90E-70 275 29159 cellular
organisms;Eukaryota;Opisthokonta;Metazoa;Eumetazoa;Bilateria;Protostomia;Spiralia;Lophotrochozoa;Mollusca;Bivalvia;Autobranchia;Pteriomorphia;Ostreida;Ostreoidea;Ostreidae;Crassostrea;Crassostrea gigas
UdeM00000000450 29159 273.5 UdeM00000000450 XP_034329844.1 32.6 503
313 7 4 491 5 496 5.40E-70 273.5 29159 cellular
organisms;Eukaryota;Opisthokonta;Metazoa;Eumetazoa;Bilateria;Protostomia;Spiralia;Lophotrochozoa;Mollusca;Bivalvia;Autobranchia;Pteriomorphia;Ostreida;Ostreoidea;Ostreidae;Crassostrea;Crassostrea gigas
UdeM00000000451 6596 563.9 UdeM00000000451 XP_045169405.1 89.6 299
31 0 1 299 1 299 1.20E-157 563.9 6596 cellular
organisms;Eukaryota;Opisthokonta;Metazoa;Eumetazoa;Bilateria;Protostomia;Spiralia;Lophotrochozoa;Mollusca;Bivalvia;Autobranchia;Heteroconchia;Euheterodonta;Imparidentia;Venerida;Veneroidea;Veneridae;Mercenaria;Mercenaria mercenaria
UdeM00000000452 1735272 226.1 UdeM00000000452 XP_041365495.1 60.1
193 77 0 1 193 4 196 4.60E-56 226.1 1735272
cellular

organisms;Eukaryota;Opisthokonta;Metazoa;Eumetazoa;Bilateria;Protostomia;Spiralia;Lophotrochozoa;Mollusca;Gastropoda;Neomphalina;Neomphaloidea;Peltospiridae;Gigantopelta;Gigantopelta aegis
UdeM00000000453 6565 258.8 UdeM00000000453 XP_022327962.1 49.3 272 134 3 1 269 448 718 7.50E-66 258.8 6565 cellular organisms;Eukaryota;Opisthokonta;Metazoa;Eumetazoa;Bilateria;Protostomia;Spiralia;Lophotrochozoa;Mollusca;Bivalvia;Autobranchia;Pteriomorphia;Ostreida;Ostreoidea;Ostreidae;Crassostrea;Crassostrea virginica
UdeM00000000454 6565 65.5 UdeM00000000454 XP_022327962.1 35.3 139 23 2 1 72 295 433 3.30E-08 65.5 6565 cellular organisms;Eukaryota;Opisthokonta;Metazoa;Eumetazoa;Bilateria;Protostomia;Spiralia;Lophotrochozoa;Mollusca;Bivalvia;Autobranchia;Pteriomorphia;Ostreida;Ostreoidea;Ostreidae;Crassostrea;Crassostrea virginica
UdeM00000000455 6596 148.7 UdeM00000000455 XP_045198374.1 45.2 177 83 4 1 173 1 167 7.00E-33 148.7 6596 cellular organisms;Eukaryota;Opisthokonta;Metazoa;Eumetazoa;Bilateria;Protostomia;Spiralia;Lophotrochozoa;Mollusca;Bivalvia;Autobranchia;Heteroconchia;Euheterodonta;Imparidentia;Venerida;Veneroidea;Veneridae;Mercenaria;Mercenaria mercenaria
UdeM00000000458 2607531 163.3 UdeM00000000458 XP_036365360.1 79.6 98 20 0 13 110 305 402 1.80E-37 163.3 2607531 cellular organisms;Eukaryota;Opisthokonta;Metazoa;Eumetazoa;Bilateria;Protostomia;Spiralia;Lophotrochozoa;Mollusca;Cephalopoda;Coleoidea;Octopodiformes;Octopoda;Incirrata;Octopodidae;Octopus;Octopus sinensis
UdeM00000000459 6596 472.6 UdeM00000000459 XP_045170613.1 74.4 316 74 4 31 340 281 595 4.50E-130 472.6 6596 cellular organisms;Eukaryota;Opisthokonta;Metazoa;Eumetazoa;Bilateria;Protostomia;Spiralia;Lophotrochozoa;Mollusca;Bivalvia;Autobranchia;Heteroconchia;Euheterodonta;Imparidentia;Venerida;Veneroidea;Veneridae;Mercenaria;Mercenaria mercenaria
UdeM00000000460 6596 846.3 UdeM00000000460 XP_045170613.1 45.2 1274 565 28 1 1177 792 2029 4.80E-242 846.3 6596 cellular organisms;Eukaryota;Opisthokonta;Metazoa;Eumetazoa;Bilateria;Protostomia;Spiralia;Lophotrochozoa;Mollusca;Bivalvia;Autobranchia;Heteroconchia;Euheterodonta;Imparidentia;Venerida;Veneroidea;Veneridae;Mercenaria;Mercenaria mercenaria
UdeM00000000461 6596 154.1 UdeM00000000461 XP_045169926.1 35.1 305 161 9 16 298 11 300 2.90E-34 154.1 6596 cellular organisms;Eukaryota;Opisthokonta;Metazoa;Eumetazoa;Bilateria;Protostomia;Spiralia;Lophotrochozoa;Mollusca;Bivalvia;Autobranchia;Heteroconchia;Euheterodonta;Imparidentia;Venerida;Veneroidea;Veneridae;Mercenaria;Mercenaria mercenaria
UdeM00000000462 6596 370.2 UdeM00000000462 XP_045177776.1 41.4 527 293 9 1 519 405 923 6.70E-99 370.2 6596 cellular organisms;Eukaryota;Opisthokonta;Metazoa;Eumetazoa;Bilateria;Protostomia;Spiralia;Lophotrochozoa;Mollusca;Bivalvia;Autobranchia;Heteroconchia;Euheterodonta;Imparidentia;Venerida;Veneroidea;Veneridae;Mercenaria;Mercenaria mercenaria
UdeM00000000463 6579 694.1 UdeM00000000463 XP_033729085.1 35.7 1657 780 44 1 1507 1 1522 3.90E-196 694.1 6579 cellular organisms;Eukaryota;Opisthokonta;Metazoa;Eumetazoa;Bilateria;Protostomia;Spiralia;Lophotrochozoa;Mollusca;Bivalvia;Autobranchia;Pteriomorphia;Pectinida;Pectinoidea;Pectinidae;Pecten;Pecten maximus

UdeM00000000464	6573	568.5	UdeM00000000464	XP_021363465.1	39.3	1033
481	19	1	929	1	991	1.50E-158	568.5	6573	cellular organisms;Eukaryota;Opisthokonta;Metazoa;Eumetazoa;Bilateria;Protostomia;Spiralia;Lophotrochozoa;Mollusca;Bivalvia;Autobranchia;Pteriomorphia;Pectinida;Pectinoidea;Pectinidae;Mizuhopecten;Mizuhopecten yessoensis
UdeM00000000465	6565	233	UdeM00000000465	XP_022320421.1	53.7	231
97	4	3	233	8	228	3.80E-58	233	6565	cellular organisms;Eukaryota;Opisthokonta;Metazoa;Eumetazoa;Bilateria;Protostomia;Spiralia;Lophotrochozoa;Mollusca;Bivalvia;Autobranchia;Pteriomorphia;Ostreida;Ostreoidea;Ostreidae;Crassostrea;Crassostrea virginica
UdeM00000000466	6596	567.4	UdeM00000000466	XP_045178508.1	79.3	363
69	4	1	358	1	362	1.30E-158	567.4	6596	cellular organisms;Eukaryota;Opisthokonta;Metazoa;Eumetazoa;Bilateria;Protostomia;Spiralia;Lophotrochozoa;Mollusca;Bivalvia;Autobranchia;Heteroconchia;Euheterodonta;Imparidentia;Venerida;Veneroidea;Veneridae;Mercenaria;Mercenaria mercenaria
UdeM00000000467	29159	547.4	UdeM00000000467	XP_034330259.1	46.7	585
294	8	6	588	133	701	2.30E-152	547.4	29159	cellular organisms;Eukaryota;Opisthokonta;Metazoa;Eumetazoa;Bilateria;Protostomia;Spiralia;Lophotrochozoa;Mollusca;Bivalvia;Autobranchia;Pteriomorphia;Ostreida;Ostreoidea;Ostreidae;Crassostrea;Crassostrea gigas
UdeM00000000470	6596	215.3	UdeM00000000470	XP_045172074.1	71.2	132
38	0	2	133	249	380	4.90E-53	215.3	6596	cellular organisms;Eukaryota;Opisthokonta;Metazoa;Eumetazoa;Bilateria;Protostomia;Spiralia;Lophotrochozoa;Mollusca;Bivalvia;Autobranchia;Heteroconchia;Euheterodonta;Imparidentia;Venerida;Veneroidea;Veneridae;Mercenaria;Mercenaria mercenaria
UdeM00000000471	6596	184.9	UdeM00000000471	XP_045172453.1	56.4	156
68	0	1	156	7	162	9.60E-44	184.9	6596	cellular organisms;Eukaryota;Opisthokonta;Metazoa;Eumetazoa;Bilateria;Protostomia;Spiralia;Lophotrochozoa;Mollusca;Bivalvia;Autobranchia;Heteroconchia;Euheterodonta;Imparidentia;Venerida;Veneroidea;Veneridae;Mercenaria;Mercenaria mercenaria
UdeM00000000473	6579	273.5	UdeM00000000473	XP_033728617.1	62.7	212
69	2	1	211	1	203	2.30E-70	273.5	6579	cellular organisms;Eukaryota;Opisthokonta;Metazoa;Eumetazoa;Bilateria;Protostomia;Spiralia;Lophotrochozoa;Mollusca;Bivalvia;Autobranchia;Pteriomorphia;Pectinida;Pectinoidea;Pectinidae;Pecten;Pecten maximus
UdeM00000000472	6596	342.8	UdeM00000000472	XP_045178232.1	62.9	259
87	1	1	259	1	250	3.80E-91	342.8	6596	cellular organisms;Eukaryota;Opisthokonta;Metazoa;Eumetazoa;Bilateria;Protostomia;Spiralia;Lophotrochozoa;Mollusca;Bivalvia;Autobranchia;Heteroconchia;Euheterodonta;Imparidentia;Venerida;Veneroidea;Veneridae;Mercenaria;Mercenaria mercenaria
UdeM00000000474	6596	424.1	UdeM00000000474	XP_045178964.1	76.1	243
58	0	23	265	240	482	1.30E-115	424.1	6596	cellular organisms;Eukaryota;Opisthokonta;Metazoa;Eumetazoa;Bilateria;Protostomia;Spiralia;Lophotrochozoa;Mollusca;Bivalvia;Autobranchia;Heteroconchia;Euheterodonta;Imparidentia;Venerida;Veneroidea;Veneridae;Mercenaria;Mercenaria mercenaria
UdeM00000000475	6573	156	UdeM00000000475	XP_021342433.1	78.4	88
19	0	17	104	75	162	2.80E-35	156	6573	cellular organisms;Eukaryota;Opisthokonta;Metazoa;Eumetazoa;Bilateria;Protostomia;

Spiralia;Lophotrochozoa;Mollusca;Bivalvia;Autobranchia;Pteriomorphia;Pectinida;Pectinoidea;Pectinidae;Mizuhopecten;Mizuhopecten yessoensis
UdeM00000000478 400727 496.9 UdeM00000000478 XP_025086682.1 72.2 313 85 1 1 311 33 345 1.90E-137 496.9 400727 cellular organisms;Eukaryota;Opisthokonta;Metazoa;Eumetazoa;Bilateria;Protostomia;Spiralia;Lophotrochozoa;Mollusca;Gastropoda;Caenogastropoda;Architaenioglossa;Ampullarioidea;Ampullariidae;Pomacea;Pomacea canaliculata
UdeM00000000479 6596 623.6 UdeM00000000479 XP_045178482.1 75.1 377 91 2 1 374 1 377 1.60E-175 623.6 6596 cellular organisms;Eukaryota;Opisthokonta;Metazoa;Eumetazoa;Bilateria;Protostomia;Spiralia;Lophotrochozoa;Mollusca;Bivalvia;Autobranchia;Heteroconchia;Euheterodonta;Imparidentia;Venerida;Veneroidea;Veneridae;Mercenaria;Mercenaria mercenaria
UdeM00000000480 6596 889.8 UdeM00000000480 XP_045177600.1 60.4 793 268 12 8 783 51 814 2.50E-255 889.8 6596 cellular organisms;Eukaryota;Opisthokonta;Metazoa;Eumetazoa;Bilateria;Protostomia;Spiralia;Lophotrochozoa;Mollusca;Bivalvia;Autobranchia;Heteroconchia;Euheterodonta;Imparidentia;Venerida;Veneroidea;Veneridae;Mercenaria;Mercenaria mercenaria
UdeM00000000481 6596 479.6 UdeM00000000481 XP_045178607.1 52.5 434 191 3 11 439 1 424 4.40E-132 479.6 6596 cellular organisms;Eukaryota;Opisthokonta;Metazoa;Eumetazoa;Bilateria;Protostomia;Spiralia;Lophotrochozoa;Mollusca;Bivalvia;Autobranchia;Heteroconchia;Euheterodonta;Imparidentia;Venerida;Veneroidea;Veneridae;Mercenaria;Mercenaria mercenaria
UdeM00000000482 6596 920.2 UdeM00000000482 XP_045199866.1 39.6 1211 647 19 13 1150 308 1506 2.80E-264 920.2 6596 cellular organisms;Eukaryota;Opisthokonta;Metazoa;Eumetazoa;Bilateria;Protostomia;Spiralia;Lophotrochozoa;Mollusca;Bivalvia;Autobranchia;Heteroconchia;Euheterodonta;Imparidentia;Venerida;Veneroidea;Veneridae;Mercenaria;Mercenaria mercenaria
UdeM00000000483 29159 676.4 UdeM00000000483 XP_011414437.2 44.8 734 373 16 43 765 29 741 4.40E-191 676.4 29159 cellular organisms;Eukaryota;Opisthokonta;Metazoa;Eumetazoa;Bilateria;Protostomia;Spiralia;Lophotrochozoa;Mollusca;Bivalvia;Autobranchia;Pteriomorphia;Ostreida;Ostreoidea;Ostreidae;Crassostrea;Crassostrea gigas
UdeM00000000484 29159 359.4 UdeM00000000484 XP_011414437.2 47.3 353 178 4 16 368 343 687 6.40E-96 359.4 29159 cellular organisms;Eukaryota;Opisthokonta;Metazoa;Eumetazoa;Bilateria;Protostomia;Spiralia;Lophotrochozoa;Mollusca;Bivalvia;Autobranchia;Pteriomorphia;Ostreida;Ostreoidea;Ostreidae;Crassostrea;Crassostrea gigas
UdeM00000000485 6579 191 UdeM00000000485 XP_033727198.1 37.1 259 119 5 30 245 518 775 1.90E-45 191 6579 cellular organisms;Eukaryota;Opisthokonta;Metazoa;Eumetazoa;Bilateria;Protostomia;Spiralia;Lophotrochozoa;Mollusca;Bivalvia;Autobranchia;Pteriomorphia;Pectinida;Pectinoidea;Pectinidae;Pecten;Pecten maximus
UdeM00000000486 29159 434.5 UdeM00000000486 XP_011414437.2 44.9 470 243 7 14 477 291 750 1.80E-118 434.5 29159 cellular organisms;Eukaryota;Opisthokonta;Metazoa;Eumetazoa;Bilateria;Protostomia;Spiralia;Lophotrochozoa;Mollusca;Bivalvia;Autobranchia;Pteriomorphia;Ostreida;Ostreoidea;Ostreidae;Crassostrea;Crassostrea gigas
UdeM00000000487 46514 601.7 UdeM00000000487 XP_038061742.1 52 612 263 14 2 599 92 686 1.10E-168 601.7 46514 cellular

organisms;Eukaryota;Opisthokonta;Metazoa;Eumetazoa;Bilateria;Deuterostomia;Echinodermata;Eleutherozoa;Asterozoa;Asteroidea;Valvatacea;Valvatida;Asterinidae;Patiria;Patiria miniata
UdeM00000000488 6596 307.4 UdeM00000000488 XP_045179280.1 39.2 429 241 6 3 427 17 429 2.90E-80 307.4 6596 cellular organisms;Eukaryota;Opisthokonta;Metazoa;Eumetazoa;Bilateria;Protostomia;Spiralia;Lophotrochozoa;Mollusca;Bivalvia;Autobranchia;Heteroconchia;Euheterodonta;Imparidentia;Venerida;Veneroidea;Veneridae;Mercenaria;Mercenaria mercenaria
UdeM00000000490 6565 252.3 UdeM00000000490 XP_022327962.1 36 472 100 4 1 270 247 718 7.00E-64 252.3 6565 cellular organisms;Eukaryota;Opisthokonta;Metazoa;Eumetazoa;Bilateria;Protostomia;Spiralia;Lophotrochozoa;Mollusca;Bivalvia;Autobranchia;Pteriomorphia;Ostreida;Ostreoidea;Ostreidae;Crassostrea;Crassostrea virginica
UdeM00000000491 6579 671 UdeM00000000491 XP_033729078.1 63.6 566 177 13 260 803 57 615 1.90E-189 671 6579 cellular organisms;Eukaryota;Opisthokonta;Metazoa;Eumetazoa;Bilateria;Protostomia;Spiralia;Lophotrochozoa;Mollusca;Bivalvia;Autobranchia;Pteriomorphia;Pectinida;Pectinoidea;Pectinidae;Pecten;Pecten maximus
UdeM00000000493 6596 548.5 UdeM00000000493 XP_045180074.1 47.3 579 280 9 9 576 3 567 1.00E-152 548.5 6596 cellular organisms;Eukaryota;Opisthokonta;Metazoa;Eumetazoa;Bilateria;Protostomia;Spiralia;Lophotrochozoa;Mollusca;Bivalvia;Autobranchia;Heteroconchia;Euheterodonta;Imparidentia;Venerida;Veneroidea;Veneridae;Mercenaria;Mercenaria mercenaria
UdeM00000000494 6596 149.4 UdeM00000000494 XP_045179100.1 32.4 293 193 4 21 309 46 337 7.80E-33 149.4 6596 cellular organisms;Eukaryota;Opisthokonta;Metazoa;Eumetazoa;Bilateria;Protostomia;Spiralia;Lophotrochozoa;Mollusca;Bivalvia;Autobranchia;Heteroconchia;Euheterodonta;Imparidentia;Venerida;Veneroidea;Veneridae;Mercenaria;Mercenaria mercenaria
UdeM00000000495 1735272 426.8 UdeM00000000495 XP_041374782.1 69.4 288 86 1 1 288 1 286 2.20E-116 426.8 1735272 cellular organisms;Eukaryota;Opisthokonta;Metazoa;Eumetazoa;Bilateria;Protostomia;Spiralia;Lophotrochozoa;Mollusca;Gastropoda;Neomphalina;Neomphaloidea;Peltospiridae;Gigantopelta;Gigantopelta aegis
UdeM00000000496 1735272 2044.2 UdeM00000000496 XP_041374199.1 40.5 3100 1444 47 14 2951 1494 4353 0.00E+00 2044.2 1735272 cellular organisms;Eukaryota;Opisthokonta;Metazoa;Eumetazoa;Bilateria;Protostomia;Spiralia;Lophotrochozoa;Mollusca;Gastropoda;Neomphalina;Neomphaloidea;Peltospiridae;Gigantopelta;Gigantopelta aegis
UdeM00000000497 6596 1812 UdeM00000000497 XP_045179037.1 47.4 2012 831 14 44 1843 2309 4304 0.00E+00 1812 6596 cellular organisms;Eukaryota;Opisthokonta;Metazoa;Eumetazoa;Bilateria;Protostomia;Spiralia;Lophotrochozoa;Mollusca;Bivalvia;Autobranchia;Heteroconchia;Euheterodonta;Imparidentia;Venerida;Veneroidea;Veneridae;Mercenaria;Mercenaria mercenaria
UdeM00000000498 400727 70.1 UdeM00000000498 XP_025091829.1 37.5 88 55 0 30 117 35 122 2.50E-09 70.1 400727 cellular organisms;Eukaryota;Opisthokonta;Metazoa;Eumetazoa;Bilateria;Protostomia;

Spiralia;Lophotrochozoa;Mollusca;Gastropoda;Caenogastropoda;Architaenioglossa;Ampullarioidea;Ampullariidae;Pomacea;Pomacea canaliculata
UdeM00000000499 29159 836.3 UdeM00000000499 XP_034301291.1 39.6 1262 613 24 150 1335 230 1418 6.20E-239 836.3 29159 cellular organisms;Eukaryota;Opisthokonta;Metazoa;Eumetazoa;Bilateria;Protostomia;Spiralia;Lophotrochozoa;Mollusca;Bivalvia;Autobranchia;Pteriomorphia;Ostreida;Ostreoidea;Ostreidae;Crassostrea;Crassostrea gigas
UdeM00000000500 29159 890.2 UdeM00000000500 XP_034301293.1 38.5 1403 701 29 117 1438 62 1383 3.90E-255 890.2 29159 cellular organisms;Eukaryota;Opisthokonta;Metazoa;Eumetazoa;Bilateria;Protostomia;Spiralia;Lophotrochozoa;Mollusca;Bivalvia;Autobranchia;Pteriomorphia;Ostreida;Ostreoidea;Ostreidae;Crassostrea;Crassostrea gigas
UdeM00000000501 6579 802.7 UdeM00000000501 XP_033728979.1 36.9 1385 676 29 115 1376 70 1379 7.90E-229 802.7 6579 cellular organisms;Eukaryota;Opisthokonta;Metazoa;Eumetazoa;Bilateria;Protostomia;Spiralia;Lophotrochozoa;Mollusca;Bivalvia;Autobranchia;Pteriomorphia;Pectinida;Pectinoidea;Pectinidae;Pecten;Pecten maximus
UdeM00000000502 29159 945.7 UdeM00000000502 XP_034301291.1 40 1405 677 28 116 1437 97 1418 7.80E-272 945.7 29159 cellular organisms;Eukaryota;Opisthokonta;Metazoa;Eumetazoa;Bilateria;Protostomia;Spiralia;Lophotrochozoa;Mollusca;Bivalvia;Autobranchia;Pteriomorphia;Ostreida;Ostreoidea;Ostreidae;Crassostrea;Crassostrea gigas
UdeM00000000504 29159 178.3 UdeM00000000504 XP_034317111.1 29.1 471 221 11 2 363 171 637 4.00E-41 178.3 29159 cellular organisms;Eukaryota;Opisthokonta;Metazoa;Eumetazoa;Bilateria;Protostomia;Spiralia;Lophotrochozoa;Mollusca;Bivalvia;Autobranchia;Pteriomorphia;Ostreida;Ostreoidea;Ostreidae;Crassostrea;Crassostrea gigas
UdeM00000000505 6596 526.2 UdeM00000000505 XP_045177776.1 37.2 833 453 16 107 921 153 933 9.50E-146 526.2 6596 cellular organisms;Eukaryota;Opisthokonta;Metazoa;Eumetazoa;Bilateria;Protostomia;Spiralia;Lophotrochozoa;Mollusca;Bivalvia;Autobranchia;Heteroconchia;Euheterodonta;Imparidentia;Venerida;Veneroidea;Veneridae;Mercenaria;Mercenaria mercenaria
UdeM00000000506 6596 317.4 UdeM00000000506 XP_045177903.1 55.1 272 114 2 1049 1314 907 1176 8.60E-83 317.4 6596 cellular organisms;Eukaryota;Opisthokonta;Metazoa;Eumetazoa;Bilateria;Protostomia;Spiralia;Lophotrochozoa;Mollusca;Bivalvia;Autobranchia;Heteroconchia;Euheterodonta;Imparidentia;Venerida;Veneroidea;Veneridae;Mercenaria;Mercenaria mercenaria
UdeM00000000507 6596 78.2 UdeM00000000507 XP_045212464.1 46.6 73 38 1 90 162 31 102 1.20E-11 78.2 6596 cellular organisms;Eukaryota;Opisthokonta;Metazoa;Eumetazoa;Bilateria;Protostomia;Spiralia;Lophotrochozoa;Mollusca;Bivalvia;Autobranchia;Heteroconchia;Euheterodonta;Imparidentia;Venerida;Veneroidea;Veneridae;Mercenaria;Mercenaria mercenaria
UdeM00000000509 6565 84.7 UdeM00000000509 XP_022288562.1 46.6 88 47 0 1 88 73 160 1.30E-13 84.7 6565 cellular organisms;Eukaryota;Opisthokonta;Metazoa;Eumetazoa;Bilateria;Protostomia;Spiralia;Lophotrochozoa;Mollusca;Bivalvia;Autobranchia;Pteriomorphia;Ostreida;Ostreoidea;Ostreidae;Crassostrea;Crassostrea virginica
UdeM00000000510 6573 218.4 UdeM00000000510 XP_021346189.1 56.1 189 77 2 1 187 22 206 7.90E-54 218.4 6573 cellular organisms;Eukaryota;Opisthokonta;Metazoa;Eumetazoa;Bilateria;Protostomia;

Spiralia;Lophotrochozoa;Mollusca;Bivalvia;Autobranchia;Pteriomorphia;Pectinida;Pectinoidea;Pectinidae;Mizuhopecten;Mizuhopecten yessoensis
UdeM00000000511 6596 335.9 UdeM00000000511 XP_045179152.1 55.1 314 131 3 1 307 71 381 6.10E-89 335.9 6596 cellular organisms;Eukaryota;Opisthokonta;Metazoa;Eumetazoa;Bilateria;Protostomia;Spiralia;Lophotrochozoa;Mollusca;Bivalvia;Autobranchia;Heteroconchia;Euheterodonta;Imparidentia;Venerida;Veneroidea;Veneridae;Mercenaria;Mercenaria mercenaria
UdeM00000000513 6596 325.5 UdeM00000000513 XP_045179603.1 47.2 377 181 5 1 363 1 373 9.60E-86 325.5 6596 cellular organisms;Eukaryota;Opisthokonta;Metazoa;Eumetazoa;Bilateria;Protostomia;Spiralia;Lophotrochozoa;Mollusca;Bivalvia;Autobranchia;Heteroconchia;Euheterodonta;Imparidentia;Venerida;Veneroidea;Veneridae;Mercenaria;Mercenaria mercenaria
UdeM00000000514 6596 166.4 UdeM00000000514 XP_045164742.1 52 125 60 0 8 132 6 130 2.50E-38 166.4 6596 cellular organisms;Eukaryota;Opisthokonta;Metazoa;Eumetazoa;Bilateria;Protostomia;Spiralia;Lophotrochozoa;Mollusca;Bivalvia;Autobranchia;Heteroconchia;Euheterodonta;Imparidentia;Venerida;Veneroidea;Veneridae;Mercenaria;Mercenaria mercenaria
UdeM00000000515 6565 186.8 UdeM00000000515 XP_022324251.1 48.6 181 89 3 25 203 18 196 2.80E-44 186.8 6565 cellular organisms;Eukaryota;Opisthokonta;Metazoa;Eumetazoa;Bilateria;Protostomia;Spiralia;Lophotrochozoa;Mollusca;Bivalvia;Autobranchia;Pteriomorphia;Ostreida;Ostreoidea;Ostreidae;Crassostrea;Crassostrea virginica
UdeM00000000516 6565 206.8 UdeM00000000516 XP_022324251.1 54.6 183 79 3 24 204 18 198 2.60E-50 206.8 6565 cellular organisms;Eukaryota;Opisthokonta;Metazoa;Eumetazoa;Bilateria;Protostomia;Spiralia;Lophotrochozoa;Mollusca;Bivalvia;Autobranchia;Pteriomorphia;Ostreida;Ostreoidea;Ostreidae;Crassostrea;Crassostrea virginica
UdeM00000000517 6412 111.7 UdeM00000000517 XP_009015452.1 51 98 48 0 1 98 1 98 5.60E-21 111.7 6412 cellular organisms;Eukaryota;Opisthokonta;Metazoa;Eumetazoa;Bilateria;Protostomia;Spiralia;Lophotrochozoa;Annelida;Clitellata;Hirudinea;Rhynchobdellida;Glossiphoniidae;Helobdella;Helobdella robusta
UdeM00000000518 6596 625.2 UdeM00000000518 XP_045180021.1 71.6 405 113 1 11 415 3 405 6.20E-176 625.2 6596 cellular organisms;Eukaryota;Opisthokonta;Metazoa;Eumetazoa;Bilateria;Protostomia;Spiralia;Lophotrochozoa;Mollusca;Bivalvia;Autobranchia;Heteroconchia;Euheterodonta;Imparidentia;Venerida;Veneroidea;Veneridae;Mercenaria;Mercenaria mercenaria
UdeM00000000519 6596 535.8 UdeM00000000519 XP_045180026.1 66.5 388 125 2 1 388 27 409 4.60E-149 535.8 6596 cellular organisms;Eukaryota;Opisthokonta;Metazoa;Eumetazoa;Bilateria;Protostomia;Spiralia;Lophotrochozoa;Mollusca;Bivalvia;Autobranchia;Heteroconchia;Euheterodonta;Imparidentia;Venerida;Veneroidea;Veneridae;Mercenaria;Mercenaria mercenaria
UdeM00000000520 6596 605.1 UdeM00000000520 XP_045180021.1 69.8 404 120 1 26 429 5 406 6.80E-170 605.1 6596 cellular organisms;Eukaryota;Opisthokonta;Metazoa;Eumetazoa;Bilateria;Protostomia;Spiralia;Lophotrochozoa;Mollusca;Bivalvia;Autobranchia;Heteroconchia;Euheterodonta;Imparidentia;Venerida;Veneroidea;Veneridae;Mercenaria;Mercenaria mercenaria

UdeM00000000521 6565 147.9 UdeM00000000521 XP_022342298.1 55.4 130
53 1 156 285 399 523 2.10E-32 147.9 6565 cellular
organisms;Eukaryota;Opisthokonta;Metazoa;Eumetazoa;Bilateria;Protostomia;
Spiralia;Lophotrochozoa;Mollusca;Bivalvia;Autobranchia;Pteriomorphia;Ostr
eida;Ostreoidea;Ostreidae;Crassostrea;Crassostrea virginica
UdeM00000000522 202533 336.3 UdeM00000000522 XP_035218899.1 38.3
470 263 8 1 447 1 466 6.10E-89 336.3 202533
cellular
organisms;Eukaryota;Opisthokonta;Metazoa;Eumetazoa;Bilateria;Protostomia;
Ecdysozoa;Panarthropoda;Arthropoda;Chelicerata;Arachnida;Araneae;Araneomo
rphae;Entelegynae;Eresoidea;Eresidae;Stegodyphus;Stegodyphus dumicola
UdeM00000000523 6596 105.1 UdeM00000000523 XP_045178915.1 30.5 643
323 15 1 547 1 615 2.80E-19 105.1 6596 cellular
organisms;Eukaryota;Opisthokonta;Metazoa;Eumetazoa;Bilateria;Protostomia;
Spiralia;Lophotrochozoa;Mollusca;Bivalvia;Autobranchia;Heteroconchia;Euhe
terodonta;Imparidentia;Venerida;Veneroidea;Veneridae;Mercenaria;Mercenari
a mercenaria
UdeM00000000524 6596 622.1 UdeM00000000524 XP_045178916.1 69.7 403
121 1 1 403 1 402 5.00E-175 622.1 6596 cellular
organisms;Eukaryota;Opisthokonta;Metazoa;Eumetazoa;Bilateria;Protostomia;
Spiralia;Lophotrochozoa;Mollusca;Bivalvia;Autobranchia;Heteroconchia;Euhe
terodonta;Imparidentia;Venerida;Veneroidea;Veneridae;Mercenaria;Mercenari
a mercenaria
UdeM00000000525 6596 65.9 UdeM00000000525 XP_045179474.1 52.9 85
18 4 1 82 1 66 2.90E-08 65.9 6596 cellular
organisms;Eukaryota;Opisthokonta;Metazoa;Eumetazoa;Bilateria;Protostomia;
Spiralia;Lophotrochozoa;Mollusca;Bivalvia;Autobranchia;Heteroconchia;Euhe
terodonta;Imparidentia;Venerida;Veneroidea;Veneridae;Mercenaria;Mercenari
a mercenaria
UdeM00000000526 6596 268.5 UdeM00000000526 XP_045179474.1 95.4 130
6 0 100 229 68 197 8.00E-69 268.5 6596 cellular
organisms;Eukaryota;Opisthokonta;Metazoa;Eumetazoa;Bilateria;Protostomia;
Spiralia;Lophotrochozoa;Mollusca;Bivalvia;Autobranchia;Heteroconchia;Euhe
terodonta;Imparidentia;Venerida;Veneroidea;Veneridae;Mercenaria;Mercenari
a mercenaria
UdeM00000000527 6596 242.7 UdeM00000000527 XP_045179765.1 51.5 233
106 4 8 233 39 271 4.80E-61 242.7 6596 cellular
organisms;Eukaryota;Opisthokonta;Metazoa;Eumetazoa;Bilateria;Protostomia;
Spiralia;Lophotrochozoa;Mollusca;Bivalvia;Autobranchia;Heteroconchia;Euhe
terodonta;Imparidentia;Venerida;Veneroidea;Veneridae;Mercenaria;Mercenari
a mercenaria
UdeM00000000528 6596 261.5 UdeM00000000528 XP_045179766.1 52 248
108 5 10 246 18 265 1.10E-66 261.5 6596 cellular
organisms;Eukaryota;Opisthokonta;Metazoa;Eumetazoa;Bilateria;Protostomia;
Spiralia;Lophotrochozoa;Mollusca;Bivalvia;Autobranchia;Heteroconchia;Euhe
terodonta;Imparidentia;Venerida;Veneroidea;Veneridae;Mercenaria;Mercenari
a mercenaria
UdeM00000000529 6596 107.8 UdeM00000000529 XP_045194227.1 48.4 126
61 3 2 125 16 139 1.40E-20 107.8 6596 cellular
organisms;Eukaryota;Opisthokonta;Metazoa;Eumetazoa;Bilateria;Protostomia;
Spiralia;Lophotrochozoa;Mollusca;Bivalvia;Autobranchia;Heteroconchia;Euhe
terodonta;Imparidentia;Venerida;Veneroidea;Veneridae;Mercenaria;Mercenari
a mercenaria

UdeM00000000530 6596 85.5 UdeM00000000530 XP_045194227.1 44.9 107
55 3 2 106 35 139 5.90E-14 85.5 6596 cellular
organisms;Eukaryota;Opisthokonta;Metazoa;Eumetazoa;Bilateria;Protostomia;Spiralia;Lophotrochozoa;Mollusca;Bivalvia;Autobranchia;Heteroconchia;Euheterodonta;Imparidentia;Venerida;Veneroidea;Veneridae;Mercenaria;Mercenaria mercenaria
UdeM00000000531 29159 374.4 UdeM00000000531 XP_034299474.1 45.2 445
227 5 6 447 247 677 2.10E-100 374.4 29159 cellular
organisms;Eukaryota;Opisthokonta;Metazoa;Eumetazoa;Bilateria;Protostomia;Spiralia;Lophotrochozoa;Mollusca;Bivalvia;Autobranchia;Pteriomorphia;Ostreida;Ostreoidea;Ostreidae;Crassostrea;Crassostrea gigas
UdeM00000000532 6565 168.3 UdeM00000000532 XP_022290343.1 50 180
80 5 1 171 126 304 8.50E-39 168.3 6565 cellular
organisms;Eukaryota;Opisthokonta;Metazoa;Eumetazoa;Bilateria;Protostomia;Spiralia;Lophotrochozoa;Mollusca;Bivalvia;Autobranchia;Pteriomorphia;Ostreida;Ostreoidea;Ostreidae;Crassostrea;Crassostrea virginica
UdeM00000000533 29159 142.5 UdeM00000000533 XP_034330911.1 52.9 138
62 2 1 137 1 136 4.40E-31 142.5 29159 cellular
organisms;Eukaryota;Opisthokonta;Metazoa;Eumetazoa;Bilateria;Protostomia;Spiralia;Lophotrochozoa;Mollusca;Bivalvia;Autobranchia;Pteriomorphia;Ostreida;Ostreoidea;Ostreidae;Crassostrea;Crassostrea gigas
UdeM00000000534 1735272 174.1 UdeM00000000534 XP_041347065.1 57.9
145 59 2 1 143 1 145 1.40E-40 174.1 1735272
cellular
organisms;Eukaryota;Opisthokonta;Metazoa;Eumetazoa;Bilateria;Protostomia;Spiralia;Lophotrochozoa;Mollusca;Gastropoda;Neomphalina;Neomphaloidea;Peltospiridae;Gigantopelta;Gigantopelta aegis
UdeM00000000535 7757 287.7 UdeM00000000535 XP_032835197.1 80.1 176
35 0 1 176 52 227 9.90E-75 287.7 7757 cellular
organisms;Eukaryota;Opisthokonta;Metazoa;Eumetazoa;Bilateria;Deuterostomia;Chordata;Craniata;Vertebrata;Cyclostomata;Hyperoartia;Petromyzontiformes;Petromyzontidae;Petromyzon;Petromyzon marinus
UdeM00000000537 7574 338.2 UdeM00000000537 XP_013382110.1 51.5 328
148 4 14 340 45 362 1.30E-89 338.2 7574 cellular
organisms;Eukaryota;Opisthokonta;Metazoa;Eumetazoa;Bilateria;Protostomia;Spiralia;Lophotrochozoa;Brachiopoda;Linguliformea;Lingulata;Lingulida;Linguloidea;Lingulidae;Lingula;Lingula anatina
UdeM00000000538 7574 168.3 UdeM00000000538 XP_013384628.1 65.8 120
39 1 1 120 33 150 8.10E-39 168.3 7574 cellular
organisms;Eukaryota;Opisthokonta;Metazoa;Eumetazoa;Bilateria;Protostomia;Spiralia;Lophotrochozoa;Brachiopoda;Linguliformea;Lingulata;Lingulida;Linguloidea;Lingulidae;Lingula;Lingula anatina
UdeM00000000538 7574 53.5 UdeM00000000538 XP_013384628.1 75 32
8 0 134 165 529 560 2.90E-04 53.5 7574 cellular
organisms;Eukaryota;Opisthokonta;Metazoa;Eumetazoa;Bilateria;Protostomia;Spiralia;Lophotrochozoa;Brachiopoda;Linguliformea;Lingulata;Lingulida;Linguloidea;Lingulidae;Lingula;Lingula anatina
UdeM00000000540 1735272 82.8 UdeM00000000540 XP_041363681.1 39.6
101 56 1 3 103 513 608 3.10E-13 82.8 1735272
cellular
organisms;Eukaryota;Opisthokonta;Metazoa;Eumetazoa;Bilateria;Protostomia;Spiralia;Lophotrochozoa;Mollusca;Gastropoda;Neomphalina;Neomphaloidea;Peltospiridae;Gigantopelta;Gigantopelta aegis

UdeM00000000541 8078 94 UdeM00000000541 XP_036006874.1 32.3 186
111 5 38 209 21 205 3.10E-16 94 8078 cellular organisms;Eukaryota;Opisthokonta;Metazoa;Eumetazoa;Bilateria;Deuterostomia;Chordata;Craniata;Vertebrata;Gnathostomata;Teleostomi;Euteleostomi;Actinopterygii;Actinopteri;Neopterygii;Teleostei;Osteoglossocephalai;Clupeocephala;Euteleosteomorpha;Neoteleostei;Eurypterygia;Ctenosquamata;Acanthomorphata;Euacanthomorphacea;Percomorphaceae;Ovalentaria;Atherinomorphae;Cyprinodontiformes;Cyprinodontoidei;Fundulidae;Fundulus;Fundulus heteroclitus
UdeM00000000542 6596 374 UdeM00000000542 XP_045179225.1 64 317
90 5 1 307 39 341 1.80E-100 374 6596 cellular organisms;Eukaryota;Opisthokonta;Metazoa;Eumetazoa;Bilateria;Protostomia;Spiralia;Lophotrochozoa;Mollusca;Bivalvia;Autobranchia;Heteroconchia;Euheterodonta;Imparidentia;Venerida;Veneroidea;Veneridae;Mercenaria;Mercenaria mercenaria
UdeM00000000543 6596 109.4 UdeM00000000543 XP_045192592.1 26.5 298
193 7 11 290 348 637 8.40E-21 109.4 6596 cellular organisms;Eukaryota;Opisthokonta;Metazoa;Eumetazoa;Bilateria;Protostomia;Spiralia;Lophotrochozoa;Mollusca;Bivalvia;Autobranchia;Heteroconchia;Euheterodonta;Imparidentia;Venerida;Veneroidea;Veneridae;Mercenaria;Mercenaria mercenaria
UdeM00000000544 6596 110.2 UdeM00000000544 XP_045191996.1 28.6 234
159 2 33 261 354 584 4.40E-21 110.2 6596 cellular organisms;Eukaryota;Opisthokonta;Metazoa;Eumetazoa;Bilateria;Protostomia;Spiralia;Lophotrochozoa;Mollusca;Bivalvia;Autobranchia;Heteroconchia;Euheterodonta;Imparidentia;Venerida;Veneroidea;Veneridae;Mercenaria;Mercenaria mercenaria
UdeM00000000545 6579 194.5 UdeM00000000545 XP_033758968.1 25.6 609
412 15 13 610 63 641 4.20E-46 194.5 6579 cellular organisms;Eukaryota;Opisthokonta;Metazoa;Eumetazoa;Bilateria;Protostomia;Spiralia;Lophotrochozoa;Mollusca;Bivalvia;Autobranchia;Pteriomorphia;Pectinida;Pectinoidea;Pectinidae;Pecten;Pecten maximus
UdeM00000000546 6596 152.9 UdeM00000000546 XP_045215484.1 31.8 318
171 7 1 278 6 317 5.90E-34 152.9 6596 cellular organisms;Eukaryota;Opisthokonta;Metazoa;Eumetazoa;Bilateria;Protostomia;Spiralia;Lophotrochozoa;Mollusca;Bivalvia;Autobranchia;Heteroconchia;Euheterodonta;Imparidentia;Venerida;Veneroidea;Veneridae;Mercenaria;Mercenaria mercenaria
UdeM00000000547 6579 161.8 UdeM00000000547 XP_033758968.1 23.8 646
441 16 1 605 44 679 2.80E-36 161.8 6579 cellular organisms;Eukaryota;Opisthokonta;Metazoa;Eumetazoa;Bilateria;Protostomia;Spiralia;Lophotrochozoa;Mollusca;Bivalvia;Autobranchia;Pteriomorphia;Pectinida;Pectinoidea;Pectinidae;Pecten;Pecten maximus
UdeM00000000548 6573 216.1 UdeM00000000548 XP_021341731.1 23.9 695
473 11 10 679 8 671 1.40E-52 216.1 6573 cellular organisms;Eukaryota;Opisthokonta;Metazoa;Eumetazoa;Bilateria;Protostomia;Spiralia;Lophotrochozoa;Mollusca;Bivalvia;Autobranchia;Pteriomorphia;Pectinida;Pectinoidea;Pectinidae;Mizuhopecten;Mizuhopecten yessoensis
UdeM00000000549 6596 222.2 UdeM00000000549 XP_045172484.1 25 688
455 18 5 677 66 707 2.00E-54 222.2 6596 cellular organisms;Eukaryota;Opisthokonta;Metazoa;Eumetazoa;Bilateria;Protostomia;Spiralia;Lophotrochozoa;Mollusca;Bivalvia;Autobranchia;Heteroconchia;Euheterodonta;Imparidentia;Venerida;Veneroidea;Veneridae;Mercenaria;Mercenaria mercenaria

UdeM00000000550 7493 126.3 UdeM00000000550 XP_014238511.1 25.6 425
253 13 904 1301 25 413 4.40E-25 126.3 7493 cellular
organisms;Eukaryota;Opisthokonta;Metazoa;Eumetazoa;Bilateria;Protostomia;
Ecdysozoa;Panarthropoda;Arthropoda;Mandibulata;Pancrustacea;Hexapoda;Inse
cta;Dicondylia;Pterygota;Neoptera;Endopterygota;Hymenoptera;Apocrita;Para
sitoida;Chalcidoidea;Trichogrammatidae;Trichogramma;Trichogramma
pretiosum
UdeM00000000551 210409 81.3 UdeM00000000551 XP_045125380.1 25.1
315 187 11 1584 1881 82 364 1.50E-11 81.3 210409
cellular
organisms;Eukaryota;Opisthokonta;Metazoa;Eumetazoa;Bilateria;Protostomia;
Ecdysozoa;Panarthropoda;Arthropoda;Mandibulata;Pancrustacea;Crustacea;Mul
ticrustacea;Malacostraca;Eumalacostraca;Eucarida;Decapoda;Pleocyemata;Bra
chyura;Eubrachyura;Heterotremata;Portunoidea;Portunidae;Portunus;Portunus
trituberculatus
UdeM00000000552 6579 537.3 UdeM00000000552 XP_033728415.1 51.7 565
247 8 19 580 14 555 2.30E-149 537.3 6579 cellular
organisms;Eukaryota;Opisthokonta;Metazoa;Eumetazoa;Bilateria;Protostomia;
Spiralia;Lophotrochozoa;Mollusca;Bivalvia;Autobranchia;Pteriomorphia;Pect
inida;Pectinoidea;Pectinidae;Pecten;Pecten maximus
UdeM00000000553 6596 370.9 UdeM00000000553 XP_045178425.1 41.7 472
234 6 41 506 38 474 2.60E-99 370.9 6596 cellular
organisms;Eukaryota;Opisthokonta;Metazoa;Eumetazoa;Bilateria;Protostomia;
Spiralia;Lophotrochozoa;Mollusca;Bivalvia;Autobranchia;Heteroconchia;Euhe
terodonta;Imparidentia;Venerida;Veneroidea;Veneridae;Mercenaria;Mercenari
a mercenaria
UdeM00000000554 6596 287.7 UdeM00000000554 XP_045187594.1 44.9 356
162 10 36 371 35 376 2.10E-74 287.7 6596 cellular
organisms;Eukaryota;Opisthokonta;Metazoa;Eumetazoa;Bilateria;Protostomia;
Spiralia;Lophotrochozoa;Mollusca;Bivalvia;Autobranchia;Heteroconchia;Euhe
terodonta;Imparidentia;Venerida;Veneroidea;Veneridae;Mercenaria;Mercenari
a mercenaria
UdeM00000000555 6596 287.7 UdeM00000000555 XP_045187594.1 44.9 356
162 10 45 380 35 376 2.20E-74 287.7 6596 cellular
organisms;Eukaryota;Opisthokonta;Metazoa;Eumetazoa;Bilateria;Protostomia;
Spiralia;Lophotrochozoa;Mollusca;Bivalvia;Autobranchia;Heteroconchia;Euhe
terodonta;Imparidentia;Venerida;Veneroidea;Veneridae;Mercenaria;Mercenari
a mercenaria
UdeM00000000556 35525 249.2 UdeM00000000556 XP_032777945.1 41.6 351
170 10 74 406 35 368 9.10E-63 249.2 35525 cellular
organisms;Eukaryota;Opisthokonta;Metazoa;Eumetazoa;Bilateria;Protostomia;
Ecdysozoa;Panarthropoda;Arthropoda;Mandibulata;Pancrustacea;Crustacea;Bra
nchiopoda;Phyllopoda;Diplostraca;Cladocera;Anomopoda;Daphniidae;Daphnia;D
aphnia magna
UdeM00000000557 35525 264.6 UdeM00000000557 XP_032777945.1 43.3 351
171 10 74 413 35 368 2.10E-67 264.6 35525 cellular
organisms;Eukaryota;Opisthokonta;Metazoa;Eumetazoa;Bilateria;Protostomia;
Ecdysozoa;Panarthropoda;Arthropoda;Mandibulata;Pancrustacea;Crustacea;Bra
nchiopoda;Phyllopoda;Diplostraca;Cladocera;Anomopoda;Daphniidae;Daphnia;D
aphnia magna
UdeM00000000558 6596 521.9 UdeM00000000558 XP_045178985.1 60.4 386
153 0 21 406 5 390 7.50E-145 521.9 6596 cellular
organisms;Eukaryota;Opisthokonta;Metazoa;Eumetazoa;Bilateria;Protostomia;
Spiralia;Lophotrochozoa;Mollusca;Bivalvia;Autobranchia;Heteroconchia;Euhe

terodonta;Imparidentia;Venerida;Veneroidea;Veneridae;Mercenaria;Mercenari
a mercenaria
UdeM00000000559 6573 394 UdeM00000000559 XP_021351760.1 39.6 904
404 36 1 808 1 858 6.80E-106 394 6573 cellular
organisms;Eukaryota;Opisthokonta;Metazoa;Eumetazoa;Bilateria;Protostomia;
Spiralia;Lophotrochozoa;Mollusca;Bivalvia;Autobranchia;Pteriomorphia;Pect
inida;Pectinoidea;Pectinidae;Mizuhopecten;Mizuhopecten yessoensis
UdeM00000000559 6573 65.5 UdeM00000000559 XP_021351760.1 57.4 54
23 0 1187 1240 1158 1211 5.60E-07 65.5 6573 cellular
organisms;Eukaryota;Opisthokonta;Metazoa;Eumetazoa;Bilateria;Protostomia;
Spiralia;Lophotrochozoa;Mollusca;Bivalvia;Autobranchia;Pteriomorphia;Pect
inida;Pectinoidea;Pectinidae;Mizuhopecten;Mizuhopecten yessoensis
UdeM00000000560 6579 220.3 UdeM00000000560 XP_033758968.1 24.8 686
468 13 15 679 13 671 7.50E-54 220.3 6579 cellular
organisms;Eukaryota;Opisthokonta;Metazoa;Eumetazoa;Bilateria;Protostomia;
Spiralia;Lophotrochozoa;Mollusca;Bivalvia;Autobranchia;Pteriomorphia;Pect
inida;Pectinoidea;Pectinidae;Pecten;Pecten maximus
UdeM00000000562 6579 119.8 UdeM00000000562 XP_033758968.1 23.7 405
284 5 8 411 141 521 8.40E-24 119.8 6579 cellular
organisms;Eukaryota;Opisthokonta;Metazoa;Eumetazoa;Bilateria;Protostomia;
Spiralia;Lophotrochozoa;Mollusca;Bivalvia;Autobranchia;Pteriomorphia;Pect
inida;Pectinoidea;Pectinidae;Pecten;Pecten maximus
UdeM00000000563 6573 227.3 UdeM00000000563 XP_021351761.1 41.8 390
181 12 14 382 761 1125 3.40E-56 227.3 6573 cellular
organisms;Eukaryota;Opisthokonta;Metazoa;Eumetazoa;Bilateria;Protostomia;
Spiralia;Lophotrochozoa;Mollusca;Bivalvia;Autobranchia;Pteriomorphia;Pect
inida;Pectinoidea;Pectinidae;Mizuhopecten;Mizuhopecten yessoensis
UdeM00000000564 6596 305.4 UdeM00000000564 XP_045179164.1 53.3 418
176 6 20 433 19 421 1.10E-79 305.4 6596 cellular
organisms;Eukaryota;Opisthokonta;Metazoa;Eumetazoa;Bilateria;Protostomia;
Spiralia;Lophotrochozoa;Mollusca;Bivalvia;Autobranchia;Heteroconchia;Euhe
terodonta;Imparidentia;Venerida;Veneroidea;Veneridae;Mercenaria;Mercenari
a mercenaria
UdeM00000000565 6573 248.4 UdeM00000000565 XP_021373014.1 25.7 1331
735 40 3 1184 10 1235 4.40E-62 248.4 6573 cellular
organisms;Eukaryota;Opisthokonta;Metazoa;Eumetazoa;Bilateria;Protostomia;
Spiralia;Lophotrochozoa;Mollusca;Bivalvia;Autobranchia;Pteriomorphia;Pect
inida;Pectinoidea;Pectinidae;Mizuhopecten;Mizuhopecten yessoensis
UdeM00000000566 6573 394.8 UdeM00000000566 XP_021341105.1 44.6 894
329 24 1 837 1 785 2.70E-106 394.8 6573 cellular
organisms;Eukaryota;Opisthokonta;Metazoa;Eumetazoa;Bilateria;Protostomia;
Spiralia;Lophotrochozoa;Mollusca;Bivalvia;Autobranchia;Pteriomorphia;Pect
inida;Pectinoidea;Pectinidae;Mizuhopecten;Mizuhopecten yessoensis
UdeM00000000569 6565 172.6 UdeM00000000569 XP_022327991.1 58.7 126
52 0 36 161 34 159 4.70E-40 172.6 6565 cellular
organisms;Eukaryota;Opisthokonta;Metazoa;Eumetazoa;Bilateria;Protostomia;
Spiralia;Lophotrochozoa;Mollusca;Bivalvia;Autobranchia;Pteriomorphia;Ostr
eida;Ostreoidea;Ostreidae;Crassostrea;Crassostrea virginica
UdeM00000000571 6596 458 UdeM00000000571 XP_045180079.1 76.2 281
67 0 1 281 1 281 8.70E-126 458 6596 cellular
organisms;Eukaryota;Opisthokonta;Metazoa;Eumetazoa;Bilateria;Protostomia;
Spiralia;Lophotrochozoa;Mollusca;Bivalvia;Autobranchia;Heteroconchia;Euhe
terodonta;Imparidentia;Venerida;Veneroidea;Veneridae;Mercenaria;Mercenari
a mercenaria

UdeM00000000572 29159 60.8 UdeM00000000572 XP_034313251.1 43.3 67
38 0 76 142 6 72 1.70E-06 60.8 29159 cellular
organisms;Eukaryota;Opisthokonta;Metazoa;Eumetazoa;Bilateria;Protostomia;
Spiralia;Lophotrochozoa;Mollusca;Bivalvia;Autobranchia;Pteriomorphia;Ostr
eida;Ostreoidea;Ostreidae;Crassostrea;Crassostrea gigas
UdeM00000000573 6573 554.3 UdeM00000000573 XP_021351753.1 60.4 450
153 4 4 428 3 452 1.40E-154 554.3 6573 cellular
organisms;Eukaryota;Opisthokonta;Metazoa;Eumetazoa;Bilateria;Protostomia;
Spiralia;Lophotrochozoa;Mollusca;Bivalvia;Autobranchia;Pteriomorphia;Pect
inida;Pectinoidea;Pectinidae;Mizuhopecten;Mizuhopecten yessoensis
UdeM00000000574 1735272 462.6 UdeM00000000574 XP_041374046.1 61
362 132 3 13 367 13 372 4.60E-127 462.6 1735272
cellular
organisms;Eukaryota;Opisthokonta;Metazoa;Eumetazoa;Bilateria;Protostomia;
Spiralia;Lophotrochozoa;Mollusca;Gastropoda;Neomphalina;Neomphaloidea;Pel
tospiridae;Gigantopelta;Gigantopelta aegis
UdeM00000000576 90988 118.2 UdeM00000000576 XP_039535825.1 58.9 95
38 1 1 95 111 204 6.20E-24 118.2 90988 cellular
organisms;Eukaryota;Opisthokonta;Metazoa;Eumetazoa;Bilateria;Deuterostomi
a;Chordata;Craniata;Vertebrata;Gnathostomata;Teleostomi;Euteleostomi;Acti
nopterygii;Actinopteri;Neopterygii;Teleostei;Osteoglossocephalai;Clupeoce
phala;Otomorpha;Ostariophysi;Otophysi;Cypriniphysae;Cypriniformes;Cyprino
idei;Leuciscidae;Pogonichthyinae;Pimephales;Pimephales promelas
UdeM00000000578 6579 1333.2 UdeM00000000578 XP_033728526.1 85.3
842 97 15 1 821 1 836 0.00E+00 1333.2 6579
cellular
organisms;Eukaryota;Opisthokonta;Metazoa;Eumetazoa;Bilateria;Protostomia;
Spiralia;Lophotrochozoa;Mollusca;Bivalvia;Autobranchia;Pteriomorphia;Pect
inida;Pectinoidea;Pectinidae;Pecten;Pecten maximus
UdeM00000000579 6579 1327.4 UdeM00000000579 XP_033728526.1 85.8
830 94 14 5 816 13 836 0.00E+00 1327.4 6579
cellular
organisms;Eukaryota;Opisthokonta;Metazoa;Eumetazoa;Bilateria;Protostomia;
Spiralia;Lophotrochozoa;Mollusca;Bivalvia;Autobranchia;Pteriomorphia;Pect
inida;Pectinoidea;Pectinidae;Pecten;Pecten maximus
UdeM00000000580 6565 398.3 UdeM00000000580 XP_022327156.1 61.4 308
102 1 1 308 1 291 9.10E-108 398.3 6565 cellular
organisms;Eukaryota;Opisthokonta;Metazoa;Eumetazoa;Bilateria;Protostomia;
Spiralia;Lophotrochozoa;Mollusca;Bivalvia;Autobranchia;Pteriomorphia;Ostr
eida;Ostreoidea;Ostreidae;Crassostrea;Crassostrea virginica
UdeM00000000581 6596 146.7 UdeM00000000581 XP_045178328.1 67.6 105
34 0 39 143 47 151 2.20E-32 146.7 6596 cellular
organisms;Eukaryota;Opisthokonta;Metazoa;Eumetazoa;Bilateria;Protostomia;
Spiralia;Lophotrochozoa;Mollusca;Bivalvia;Autobranchia;Heteroconchia;Euhe
terodonta;Imparidentia;Venerida;Veneroidea;Veneridae;Mercenaria;Mercenari
a mercenaria
UdeM00000000583 6596 239.6 UdeM00000000583 XP_045179812.1 26.8 1226
581 40 1 936 1 1199 1.80E-59 239.6 6596 cellular
organisms;Eukaryota;Opisthokonta;Metazoa;Eumetazoa;Bilateria;Protostomia;
Spiralia;Lophotrochozoa;Mollusca;Bivalvia;Autobranchia;Heteroconchia;Euhe
terodonta;Imparidentia;Venerida;Veneroidea;Veneridae;Mercenaria;Mercenari
a mercenaria
UdeM00000000584 6596 1248.4 UdeM00000000584 XP_045178526.1 58.2
1160 428 15 8 1145 11 1135 0.00E+00 1248.4 6596

cellular
organisms;Eukaryota;Opisthokonta;Metazoa;Eumetazoa;Bilateria;Protostomia;
Spiralia;Lophotrochozoa;Mollusca;Bivalvia;Autobranchia;Heteroconchia;Euhe
terodonta;Imparidentia;Venerida;Veneroidea;Veneridae;Mercenaria;Mercenari
a mercenaria
UdeM00000000585 6596 167.9 UdeM00000000585 XP_045178526.1 52.7 169
67 3 1 162 936 1098 1.10E-38 167.9 6596 cellular
organisms;Eukaryota;Opisthokonta;Metazoa;Eumetazoa;Bilateria;Protostomia;
Spiralia;Lophotrochozoa;Mollusca;Bivalvia;Autobranchia;Heteroconchia;Euhe
terodonta;Imparidentia;Venerida;Veneroidea;Veneridae;Mercenaria;Mercenari
a mercenaria
UdeM00000000589 29159 1440.6 UdeM00000000589 XP_034302242.1 82.9
875 149 1 1 874 1 875 0.00E+00 1440.6
29159 cellular
organisms;Eukaryota;Opisthokonta;Metazoa;Eumetazoa;Bilateria;Protostomia;
Spiralia;Lophotrochozoa;Mollusca;Bivalvia;Autobranchia;Pteriomorphia;Ostr
eida;Ostreoidea;Ostreidae;Crassostrea;Crassostrea gigas
UdeM00000000590 29159 367.5 UdeM00000000590 XP_034302240.1 78.5 228
48 1 6 232 651 878 1.30E-98 367.5 29159 cellular
organisms;Eukaryota;Opisthokonta;Metazoa;Eumetazoa;Bilateria;Protostomia;
Spiralia;Lophotrochozoa;Mollusca;Bivalvia;Autobranchia;Pteriomorphia;Ostr
eida;Ostreoidea;Ostreidae;Crassostrea;Crassostrea gigas
UdeM00000000591 29159 614.4 UdeM00000000591 XP_034302950.1 79.1 422
85 2 3 421 68 489 1.10E-172 614.4 29159 cellular
organisms;Eukaryota;Opisthokonta;Metazoa;Eumetazoa;Bilateria;Protostomia;
Spiralia;Lophotrochozoa;Mollusca;Bivalvia;Autobranchia;Pteriomorphia;Ostr
eida;Ostreoidea;Ostreidae;Crassostrea;Crassostrea gigas
UdeM00000000592 6579 407.1 UdeM00000000592 XP_033728032.1 38.6 741
368 21 1 679 1 716 4.30E-110 407.1 6579 cellular
organisms;Eukaryota;Opisthokonta;Metazoa;Eumetazoa;Bilateria;Protostomia;
Spiralia;Lophotrochozoa;Mollusca;Bivalvia;Autobranchia;Pteriomorphia;Pect
inida;Pectinoidea;Pectinidae;Pecten;Pecten maximus
UdeM00000000593 6579 67.4 UdeM00000000593 XP_033728710.1 26.5 181
128 2 193 370 307 485 4.40E-08 67.4 6579 cellular
organisms;Eukaryota;Opisthokonta;Metazoa;Eumetazoa;Bilateria;Protostomia;
Spiralia;Lophotrochozoa;Mollusca;Bivalvia;Autobranchia;Pteriomorphia;Pect
inida;Pectinoidea;Pectinidae;Pecten;Pecten maximus
UdeM00000000594 6579 67.4 UdeM00000000594 XP_033728710.1 25.9 185
130 3 193 376 307 485 4.50E-08 67.4 6579 cellular
organisms;Eukaryota;Opisthokonta;Metazoa;Eumetazoa;Bilateria;Protostomia;
Spiralia;Lophotrochozoa;Mollusca;Bivalvia;Autobranchia;Pteriomorphia;Pect
inida;Pectinoidea;Pectinidae;Pecten;Pecten maximus
UdeM00000000595 6579 73.2 UdeM00000000595 XP_033728710.1 22.2 334
193 6 4 270 724 1057 7.80E-10 73.2 6579 cellular
organisms;Eukaryota;Opisthokonta;Metazoa;Eumetazoa;Bilateria;Protostomia;
Spiralia;Lophotrochozoa;Mollusca;Bivalvia;Autobranchia;Pteriomorphia;Pect
inida;Pectinoidea;Pectinidae;Pecten;Pecten maximus
UdeM00000000596 7054 238.4 UdeM00000000596 XP_031350480.1 54.3 186
85 0 44 229 224 409 9.10E-60 238.4 7054 cellular
organisms;Eukaryota;Opisthokonta;Metazoa;Eumetazoa;Bilateria;Protostomia;
Ecdysozoa;Panarthropoda;Arthropoda;Mandibulata;Pancrustacea;Hexapoda;Inse
cta;Dicondylia;Pterygota;Neoptera;Endopterygota;Coleoptera;Polyphaga;Elat
eriformia;Elateroidea;Lampyridae;Lampyrinae;Photinus;Photinus pyralis

UdeM00000000597 310752 62.8 UdeM00000000597 XP_028019109.1 44.2
77 37 3 24 98 297 369 1.60E-06 62.8 310752
cellular
organisms;Eukaryota;Opisthokonta;Metazoa;Eumetazoa;Bilateria;Deuterostomia;Chordata;Craniata;Vertebrata;Gnathostomata;Teleostomi;Euteleostomi;Sarcopterygii;Dipnotetrapodomorpha;Tetrapoda;Amniota;Mammalia;Theria;Eutheria;Boreoeutheria;Laurasiatheria;Artiodactyla;Whippomorpha;Cetacea;Mysticeti;Balaenopteridae;Balaenoptera;Balaenoptera acutorostrata;Balaenoptera acutorostrata scammoni
UdeM00000000600 6596 115.9 UdeM00000000600 XP_045179133.1 72 75
20 1 1 74 41 115 2.20E-23 115.9 6596 cellular
organisms;Eukaryota;Opisthokonta;Metazoa;Eumetazoa;Bilateria;Protostomia;Spiralia;Lophotrochozoa;Mollusca;Bivalvia;Autobranchia;Heteroconchia;Euheterodonta;Imparidentia;Venerida;Veneroidea;Veneridae;Mercenaria;Mercenaria mercenaria
UdeM00000000601 6596 965.7 UdeM00000000601 XP_045180000.1 71.2 677
193 2 1 675 1 677 3.10E-278 965.7 6596 cellular
organisms;Eukaryota;Opisthokonta;Metazoa;Eumetazoa;Bilateria;Protostomia;Spiralia;Lophotrochozoa;Mollusca;Bivalvia;Autobranchia;Heteroconchia;Euheterodonta;Imparidentia;Venerida;Veneroidea;Veneridae;Mercenaria;Mercenaria mercenaria
UdeM00000000602 6573 63.2 UdeM00000000602 XP_021366893.1 34.3 102
61 3 65 166 11 106 3.90E-07 63.2 6573 cellular
organisms;Eukaryota;Opisthokonta;Metazoa;Eumetazoa;Bilateria;Protostomia;Spiralia;Lophotrochozoa;Mollusca;Bivalvia;Autobranchia;Pteriomorphia;Pectinida;Pectinoidea;Pectinidae;Mizuhopecten;Mizuhopecten yessoensis
UdeM00000000603 6573 62.8 UdeM00000000603 XP_021366893.1 34.3 102
61 3 10 111 11 106 3.50E-07 62.8 6573 cellular
organisms;Eukaryota;Opisthokonta;Metazoa;Eumetazoa;Bilateria;Protostomia;Spiralia;Lophotrochozoa;Mollusca;Bivalvia;Autobranchia;Pteriomorphia;Pectinida;Pectinoidea;Pectinidae;Mizuhopecten;Mizuhopecten yessoensis
UdeM00000000604 6573 278.9 UdeM00000000604 XP_021355158.1 49.3 361
150 9 1 347 1 342 9.90E-72 278.9 6573 cellular
organisms;Eukaryota;Opisthokonta;Metazoa;Eumetazoa;Bilateria;Protostomia;Spiralia;Lophotrochozoa;Mollusca;Bivalvia;Autobranchia;Pteriomorphia;Pectinida;Pectinoidea;Pectinidae;Mizuhopecten;Mizuhopecten yessoensis
UdeM00000000605 6565 211.8 UdeM00000000605 XP_022321940.1 24.5 575
427 5 5 578 114 682 2.30E-51 211.8 6565 cellular
organisms;Eukaryota;Opisthokonta;Metazoa;Eumetazoa;Bilateria;Protostomia;Spiralia;Lophotrochozoa;Mollusca;Bivalvia;Autobranchia;Pteriomorphia;Ostreida;Ostreoidea;Ostreidae;Crassostrea;Crassostrea virginica
UdeM00000000607 6596 1067 UdeM00000000607 XP_045178438.1 64.4 944
294 20 1 920 1 926 0.00E+00 1067 6596 cellular
organisms;Eukaryota;Opisthokonta;Metazoa;Eumetazoa;Bilateria;Protostomia;Spiralia;Lophotrochozoa;Mollusca;Bivalvia;Autobranchia;Heteroconchia;Euheterodonta;Imparidentia;Venerida;Veneroidea;Veneridae;Mercenaria;Mercenaria mercenaria
UdeM00000000608 278856 185.7 UdeM00000000608 XP_032528089.1 37.7
300 162 10 151 433 323 614 2.00E-43 185.7 278856
cellular
organisms;Eukaryota;Opisthokonta;Metazoa;Eumetazoa;Bilateria;Protostomia;Ecdysozoa;Panarthropoda;Arthropoda;Mandibulata;Pancrustacea;Hexapoda;Insecta;Dicondylia;Pterygota;Neoptera;Endopterygota;Amphiesmenoptera;Lepidoptera;Glossata;Neolepidoptera;Heteroneura;Ditrysia;Obtectomera;Papilionoide

a;Nymphalidae;Danainae;Danaini;Danaina;Danaus;Danaus;Danaus plexippus;Danaus plexippus plexippus
UdeM00000000609 6596 431.8 UdeM00000000609 XP_045178776.1 56 389 170 1 107 495 134 521 1.20E-117 431.8 6596 cellular organisms;Eukaryota;Opisthokonta;Metazoa;Eumetazoa;Bilateria;Protostomia;Spiralia;Lophotrochozoa;Mollusca;Bivalvia;Autobranchia;Heteroconchia;Euheterodonta;Imparidentia;Venerida;Veneroidea;Veneridae;Mercenaria;Mercenaria mercenaria
UdeM00000000611 400727 296.2 UdeM00000000611 XP_025086782.1 44.5 330 183 0 17 346 28 357 5.50E-77 296.2 400727 cellular organisms;Eukaryota;Opisthokonta;Metazoa;Eumetazoa;Bilateria;Protostomia;Spiralia;Lophotrochozoa;Mollusca;Gastropoda;Caenogastropoda;Architaenioglossa;Ampullarioidea;Ampullariidae;Pomacea;Pomacea canaliculata
UdeM00000000612 6596 411.8 UdeM00000000612 XP_045179478.1 69.6 299 91 0 13 311 5 303 8.00E-112 411.8 6596 cellular organisms;Eukaryota;Opisthokonta;Metazoa;Eumetazoa;Bilateria;Protostomia;Spiralia;Lophotrochozoa;Mollusca;Bivalvia;Autobranchia;Heteroconchia;Euheterodonta;Imparidentia;Venerida;Veneroidea;Veneridae;Mercenaria;Mercenaria mercenaria
UdeM00000000613 6573 700.7 UdeM00000000613 XP_021365386.1 52.4 704 313 9 2 687 4 703 2.10E-198 700.7 6573 cellular organisms;Eukaryota;Opisthokonta;Metazoa;Eumetazoa;Bilateria;Protostomia;Spiralia;Lophotrochozoa;Mollusca;Bivalvia;Autobranchia;Pteriomorphia;Pectinida;Pectinoidea;Pectinidae;Mizuhopecten;Mizuhopecten yessoensis
UdeM00000000616 6596 547 UdeM00000000616 XP_045177552.1 73.4 379 92 3 11 389 39 408 2.00E-152 547 6596 cellular organisms;Eukaryota;Opisthokonta;Metazoa;Eumetazoa;Bilateria;Protostomia;Spiralia;Lophotrochozoa;Mollusca;Bivalvia;Autobranchia;Heteroconchia;Euheterodonta;Imparidentia;Venerida;Veneroidea;Veneridae;Mercenaria;Mercenaria mercenaria
UdeM00000000615 6596 546.2 UdeM00000000615 XP_045177552.1 73.4 379 92 3 11 389 39 408 3.60E-152 546.2 6596 cellular organisms;Eukaryota;Opisthokonta;Metazoa;Eumetazoa;Bilateria;Protostomia;Spiralia;Lophotrochozoa;Mollusca;Bivalvia;Autobranchia;Heteroconchia;Euheterodonta;Imparidentia;Venerida;Veneroidea;Veneridae;Mercenaria;Mercenaria mercenaria
UdeM00000000622 6596 112.8 UdeM00000000622 XP_045178614.1 50 118 55 2 1 116 1 116 2.90E-22 112.8 6596 cellular organisms;Eukaryota;Opisthokonta;Metazoa;Eumetazoa;Bilateria;Protostomia;Spiralia;Lophotrochozoa;Mollusca;Bivalvia;Autobranchia;Heteroconchia;Euheterodonta;Imparidentia;Venerida;Veneroidea;Veneridae;Mercenaria;Mercenaria mercenaria
UdeM00000000623 6596 1531.9 UdeM00000000623 XP_045177754.1 58.9 1384 449 25 1 1326 1 1322 0.00E+00 1531.9 6596 cellular organisms;Eukaryota;Opisthokonta;Metazoa;Eumetazoa;Bilateria;Protostomia;Spiralia;Lophotrochozoa;Mollusca;Bivalvia;Autobranchia;Heteroconchia;Euheterodonta;Imparidentia;Venerida;Veneroidea;Veneridae;Mercenaria;Mercenaria mercenaria
UdeM00000000624 6579 141 UdeM00000000624 XP_033727998.1 54 161 59 5 1 152 2 156 1.30E-30 141 6579 cellular organisms;Eukaryota;Opisthokonta;Metazoa;Eumetazoa;Bilateria;Protostomia;

Spiralia;Lophotrochozoa;Mollusca;Bivalvia;Autobranchia;Pteriomorphia;Pectinida;Pectinoidea;Pectinidae;Pecten;Pecten maximus
UdeM00000000625 6596 165.6 UdeM00000000625 XP_045177846.1 76 100 24 0 46 145 73 172 4.90E-38 165.6 6596 cellular organisms;Eukaryota;Opisthokonta;Metazoa;Eumetazoa;Bilateria;Protostomia;Spiralia;Lophotrochozoa;Mollusca;Bivalvia;Autobranchia;Heteroconchia;Euheterodonta;Imparidentia;Venerida;Veneroidea;Veneridae;Mercenaria;Mercenaria mercenaria
UdeM00000000626 6596 221.9 UdeM00000000626 XP_045177846.1 76.6 137 32 0 46 182 73 209 6.90E-55 221.9 6596 cellular organisms;Eukaryota;Opisthokonta;Metazoa;Eumetazoa;Bilateria;Protostomia;Spiralia;Lophotrochozoa;Mollusca;Bivalvia;Autobranchia;Heteroconchia;Euheterodonta;Imparidentia;Venerida;Veneroidea;Veneridae;Mercenaria;Mercenaria mercenaria
UdeM00000000627 1735272 228 UdeM00000000627 XP_041379265.1 48.2 220 88 2 148 343 282 499 1.80E-56 228 1735272 cellular organisms;Eukaryota;Opisthokonta;Metazoa;Eumetazoa;Bilateria;Protostomia;Spiralia;Lophotrochozoa;Mollusca;Gastropoda;Neomphalina;Neomphaloidea;Peltospiridae;Gigantopelta;Gigantopelta aegis
UdeM00000000628 6596 750.4 UdeM00000000628 XP_045177846.1 67.2 579 189 1 45 623 248 825 2.50E-213 750.4 6596 cellular organisms;Eukaryota;Opisthokonta;Metazoa;Eumetazoa;Bilateria;Protostomia;Spiralia;Lophotrochozoa;Mollusca;Bivalvia;Autobranchia;Heteroconchia;Euheterodonta;Imparidentia;Venerida;Veneroidea;Veneridae;Mercenaria;Mercenaria mercenaria
UdeM00000000629 6596 5659.7 UdeM00000000629 XP_045177846.1 60.1 5422 1917 60 8 5341 1218 6481 0.00E+00 5659.7 6596 cellular organisms;Eukaryota;Opisthokonta;Metazoa;Eumetazoa;Bilateria;Protostomia;Spiralia;Lophotrochozoa;Mollusca;Bivalvia;Autobranchia;Heteroconchia;Euheterodonta;Imparidentia;Venerida;Veneroidea;Veneridae;Mercenaria;Mercenaria mercenaria
UdeM00000000630 6596 5659.7 UdeM00000000630 XP_045177846.1 60.1 5422 1917 60 8 5341 1218 6481 0.00E+00 5659.7 6596 cellular organisms;Eukaryota;Opisthokonta;Metazoa;Eumetazoa;Bilateria;Protostomia;Spiralia;Lophotrochozoa;Mollusca;Bivalvia;Autobranchia;Heteroconchia;Euheterodonta;Imparidentia;Venerida;Veneroidea;Veneridae;Mercenaria;Mercenaria mercenaria
UdeM00000000631 6573 9050.2 UdeM00000000631 XP_021365920.1 45.7 10458 4131 52 15 9319 10840 20900 0.00E+00 9050.2 6573 cellular organisms;Eukaryota;Opisthokonta;Metazoa;Eumetazoa;Bilateria;Protostomia;Spiralia;Lophotrochozoa;Mollusca;Bivalvia;Autobranchia;Pteriomorphia;Pectinida;Pectinoidea;Pectinidae;Mizuhopecten;Mizuhopecten yessoensis
UdeM00000000632 6596 256.5 UdeM00000000632 XP_045177641.1 38 469 215 13 1 411 1 451 5.60E-65 256.5 6596 cellular organisms;Eukaryota;Opisthokonta;Metazoa;Eumetazoa;Bilateria;Protostomia;Spiralia;Lophotrochozoa;Mollusca;Bivalvia;Autobranchia;Heteroconchia;Euheterodonta;Imparidentia;Venerida;Veneroidea;Veneridae;Mercenaria;Mercenaria mercenaria
UdeM00000000633 6596 557 UdeM00000000633 XP_045177635.1 61.6 445 161 4 61 504 10 445 2.50E-155 557 6596 cellular

organisms;Eukaryota;Opisthokonta;Metazoa;Eumetazoa;Bilateria;Protostomia;Spiralia;Lophotrochozoa;Mollusca;Bivalvia;Autobranchia;Heteroconchia;Euheterodonta;Imparidentia;Venerida;Veneroidea;Veneridae;Mercenaria;Mercenaria mercenaria
UdeM00000000634 6596 870.9 UdeM00000000634 XP_045177632.1 52.3 961 414 14 326 1257 1110 2055 1.90E-249 870.9 6596 cellular organisms;Eukaryota;Opisthokonta;Metazoa;Eumetazoa;Bilateria;Protostomia;Spiralia;Lophotrochozoa;Mollusca;Bivalvia;Autobranchia;Heteroconchia;Euheterodonta;Imparidentia;Venerida;Veneroidea;Veneridae;Mercenaria;Mercenaria mercenaria
UdeM00000000634 6596 68.6 UdeM00000000634 XP_045177632.1 33.3 99 63 1 22 117 586 684 6.70E-08 68.6 6596 cellular organisms;Eukaryota;Opisthokonta;Metazoa;Eumetazoa;Bilateria;Protostomia;Spiralia;Lophotrochozoa;Mollusca;Bivalvia;Autobranchia;Heteroconchia;Euheterodonta;Imparidentia;Venerida;Veneroidea;Veneridae;Mercenaria;Mercenaria mercenaria
UdeM00000000635 6579 347.1 UdeM00000000635 XP_033748001.1 45.9 381 186 6 10 383 9 376 3.00E-92 347.1 6579 cellular organisms;Eukaryota;Opisthokonta;Metazoa;Eumetazoa;Bilateria;Protostomia;Spiralia;Lophotrochozoa;Mollusca;Bivalvia;Autobranchia;Pteriomorphia;Pectinida;Pectinoidea;Pectinidae;Pecten;Pecten maximus
UdeM00000000636 6500 291.2 UdeM00000000636 XP_035826907.1 42.6 385 212 5 1 383 300 677 2.40E-75 291.2 6500 cellular organisms;Eukaryota;Opisthokonta;Metazoa;Eumetazoa;Bilateria;Protostomia;Spiralia;Lophotrochozoa;Mollusca;Gastropoda;Heterobranchia;Euthyneura;Tectipleura;Aplysiida;Aplysioidea;Aplysiidae;Aplysia;Aplysia californica
UdeM00000000637 6579 343.2 UdeM00000000637 XP_033748001.1 45.1 377 196 4 46 420 9 376 4.70E-91 343.2 6579 cellular organisms;Eukaryota;Opisthokonta;Metazoa;Eumetazoa;Bilateria;Protostomia;Spiralia;Lophotrochozoa;Mollusca;Bivalvia;Autobranchia;Pteriomorphia;Pectinida;Pectinoidea;Pectinidae;Pecten;Pecten maximus
UdeM00000000638 6573 330.5 UdeM00000000638 XP_021364790.1 40.8 380 214 4 5 382 6 376 2.90E-87 330.5 6573 cellular organisms;Eukaryota;Opisthokonta;Metazoa;Eumetazoa;Bilateria;Protostomia;Spiralia;Lophotrochozoa;Mollusca;Bivalvia;Autobranchia;Pteriomorphia;Pectinida;Pectinoidea;Pectinidae;Mizuhopecten;Mizuhopecten yessoensis
UdeM00000000639 400727 316.6 UdeM00000000639 XP_025110836.1 42.3 385 198 7 13 383 53 427 4.30E-83 316.6 400727 cellular organisms;Eukaryota;Opisthokonta;Metazoa;Eumetazoa;Bilateria;Protostomia;Spiralia;Lophotrochozoa;Mollusca;Gastropoda;Caenogastropoda;Architaenioglossa;Ampullarioidea;Ampullariidae;Pomacea;Pomacea canaliculata
UdeM00000000641 6579 305.1 UdeM00000000641 XP_033748001.1 41.2 381 210 7 7 383 6 376 1.30E-79 305.1 6579 cellular organisms;Eukaryota;Opisthokonta;Metazoa;Eumetazoa;Bilateria;Protostomia;Spiralia;Lophotrochozoa;Mollusca;Bivalvia;Autobranchia;Pteriomorphia;Pectinida;Pectinoidea;Pectinidae;Pecten;Pecten maximus
UdeM00000000642 6579 314.3 UdeM00000000642 XP_033748001.1 42 381 207 7 7 383 6 376 2.10E-82 314.3 6579 cellular organisms;Eukaryota;Opisthokonta;Metazoa;Eumetazoa;Bilateria;Protostomia;Spiralia;Lophotrochozoa;Mollusca;Bivalvia;Autobranchia;Pteriomorphia;Pectinida;Pectinoidea;Pectinidae;Pecten;Pecten maximus
UdeM00000000643 6579 321.2 UdeM00000000643 XP_033748001.1 41.3 380 208 4 2 375 6 376 1.70E-84 321.2 6579 cellular

organisms;Eukaryota;Opisthokonta;Metazoa;Eumetazoa;Bilateria;Protostomia;Spiralia;Lophotrochozoa;Mollusca;Bivalvia;Autobranchia;Pteriomorphia;Pectinida;Pectinoidea;Pectinidae;Pecten;Pecten maximus
UdeM00000000644 6596 384 UdeM00000000644 XP_045179786.1 50.8 366 178 2 1 364 1 366 2.10E-103 384 6596 cellular organisms;Eukaryota;Opisthokonta;Metazoa;Eumetazoa;Bilateria;Protostomia;Spiralia;Lophotrochozoa;Mollusca;Bivalvia;Autobranchia;Heteroconchia;Euheterodonta;Imparidentia;Venerida;Veneroidea;Veneridae;Mercenaria;Mercenaria mercenaria
UdeM00000000645 6526 749.2 UdeM00000000645 XP_013081401.1 62.9 620 225 2 51 669 43 658 4.60E-213 749.2 6526 cellular organisms;Eukaryota;Opisthokonta;Metazoa;Eumetazoa;Bilateria;Protostomia;Spiralia;Lophotrochozoa;Mollusca;Gastropoda;Heterobranchia;Euthyneura;Panpulmonata;Hygrophila;Lymnaeoidea;Planorbidae;Biomphalaria;Biomphalaria glabrata
UdeM00000000646 6596 271.9 UdeM00000000646 XP_045179024.1 69.3 192 59 0 13 204 874 1065 6.60E-70 271.9 6596 cellular organisms;Eukaryota;Opisthokonta;Metazoa;Eumetazoa;Bilateria;Protostomia;Spiralia;Lophotrochozoa;Mollusca;Bivalvia;Autobranchia;Heteroconchia;Euheterodonta;Imparidentia;Venerida;Veneroidea;Veneridae;Mercenaria;Mercenaria mercenaria
UdeM00000000647 6500 723 UdeM00000000647 XP_035828416.1 33.4 1361 779 20 20 1262 179 1530 6.50E-205 723 6500 cellular organisms;Eukaryota;Opisthokonta;Metazoa;Eumetazoa;Bilateria;Protostomia;Spiralia;Lophotrochozoa;Mollusca;Gastropoda;Heterobranchia;Euthyneura;Tectipleura;Aplysiida;Aplysioidea;Aplysiidae;Aplysia;Aplysia californica
UdeM00000000648 6596 104.4 UdeM00000000648 XP_045179786.1 45 109 59 1 31 138 258 366 1.20E-19 104.4 6596 cellular organisms;Eukaryota;Opisthokonta;Metazoa;Eumetazoa;Bilateria;Protostomia;Spiralia;Lophotrochozoa;Mollusca;Bivalvia;Autobranchia;Heteroconchia;Euheterodonta;Imparidentia;Venerida;Veneroidea;Veneridae;Mercenaria;Mercenaria mercenaria
UdeM00000000649 6596 79.7 UdeM00000000649 XP_045179786.1 46.9 96 42 2 3 89 143 238 2.10E-12 79.7 6596 cellular organisms;Eukaryota;Opisthokonta;Metazoa;Eumetazoa;Bilateria;Protostomia;Spiralia;Lophotrochozoa;Mollusca;Bivalvia;Autobranchia;Heteroconchia;Euheterodonta;Imparidentia;Venerida;Veneroidea;Veneridae;Mercenaria;Mercenaria mercenaria
UdeM00000000650 6596 114 UdeM00000000650 XP_045179786.1 56.7 90 39 0 16 105 79 168 1.30E-22 114 6596 cellular organisms;Eukaryota;Opisthokonta;Metazoa;Eumetazoa;Bilateria;Protostomia;Spiralia;Lophotrochozoa;Mollusca;Bivalvia;Autobranchia;Heteroconchia;Euheterodonta;Imparidentia;Venerida;Veneroidea;Veneridae;Mercenaria;Mercenaria mercenaria
UdeM00000000651 400727 214.2 UdeM00000000651 XP_025081052.1 92.5 120 7 1 1 120 1 118 9.40E-53 214.2 400727 cellular organisms;Eukaryota;Opisthokonta;Metazoa;Eumetazoa;Bilateria;Protostomia;Spiralia;Lophotrochozoa;Mollusca;Gastropoda;Caenogastropoda;Architaenioglossa;Ampullarioidea;Ampullariidae;Pomacea;Pomacea canaliculata
UdeM00000000652 400727 154.1 UdeM00000000652 XP_025087839.1 45.2 155 83 2 19 171 116 270 1.70E-34 154.1 400727 cellular organisms;Eukaryota;Opisthokonta;Metazoa;Eumetazoa;Bilateria;Protostomia;

Spiralia;Lophotrochozoa;Mollusca;Gastropoda;Caenogastropoda;Architaeniogl
ossa;Ampullarioidea;Ampullariidae;Pomacea;Pomacea canaliculata
UdeM00000000655 6596 1947.9 UdeM00000000655 XP_045179002.1 49.1
2505 1030 52 74 2502 2087 4423 0.00E+00 1947.9 6596
cellular
organisms;Eukaryota;Opisthokonta;Metazoa;Eumetazoa;Bilateria;Protostomia;
Spiralia;Lophotrochozoa;Mollusca;Bivalvia;Autobranchia;Heteroconchia;Euhe
terodonta;Imparidentia;Venerida;Veneroidea;Veneridae;Mercenaria;Mercenari
a mercenaria
UdeM00000000659 6596 639.8 UdeM00000000659 XP_045177666.1 64.8 494
165 5 247 733 50 541 4.20E-180 639.8 6596 cellular
organisms;Eukaryota;Opisthokonta;Metazoa;Eumetazoa;Bilateria;Protostomia;
Spiralia;Lophotrochozoa;Mollusca;Bivalvia;Autobranchia;Heteroconchia;Euhe
terodonta;Imparidentia;Venerida;Veneroidea;Veneridae;Mercenaria;Mercenari
a mercenaria
UdeM00000000660 6573 101.3 UdeM00000000660 XP_021354117.1 40.3 119
71 0 59 177 12 130 1.70E-18 101.3 6573 cellular
organisms;Eukaryota;Opisthokonta;Metazoa;Eumetazoa;Bilateria;Protostomia;
Spiralia;Lophotrochozoa;Mollusca;Bivalvia;Autobranchia;Pteriomorphia;Pect
inida;Pectinoidea;Pectinidae;Mizuhopecten;Mizuhopecten yessoensis
UdeM00000000661 6596 74.7 UdeM00000000661 XP_045179604.1 55.9 59
26 0 29 87 123 181 7.70E-11 74.7 6596 cellular
organisms;Eukaryota;Opisthokonta;Metazoa;Eumetazoa;Bilateria;Protostomia;
Spiralia;Lophotrochozoa;Mollusca;Bivalvia;Autobranchia;Heteroconchia;Euhe
terodonta;Imparidentia;Venerida;Veneroidea;Veneridae;Mercenaria;Mercenari
a mercenaria
UdeM00000000662 6579 95.9 UdeM00000000662 XP_033728432.1 33 188
116 4 3 190 415 592 6.50E-17 95.9 6579 cellular
organisms;Eukaryota;Opisthokonta;Metazoa;Eumetazoa;Bilateria;Protostomia;
Spiralia;Lophotrochozoa;Mollusca;Bivalvia;Autobranchia;Pteriomorphia;Pect
inida;Pectinoidea;Pectinidae;Pecten;Pecten maximus
UdeM00000000664 6596 78.2 UdeM00000000664 XP_045178180.1 30.5 239
134 8 34 270 245 453 3.90E-11 78.2 6596 cellular
organisms;Eukaryota;Opisthokonta;Metazoa;Eumetazoa;Bilateria;Protostomia;
Spiralia;Lophotrochozoa;Mollusca;Bivalvia;Autobranchia;Heteroconchia;Euhe
terodonta;Imparidentia;Venerida;Veneroidea;Veneridae;Mercenaria;Mercenari
a mercenaria
UdeM00000000665 6596 264.2 UdeM00000000665 XP_045178181.1 28 1283
614 44 30 1275 12 1021 8.40E-67 264.2 6596 cellular
organisms;Eukaryota;Opisthokonta;Metazoa;Eumetazoa;Bilateria;Protostomia;
Spiralia;Lophotrochozoa;Mollusca;Bivalvia;Autobranchia;Heteroconchia;Euhe
terodonta;Imparidentia;Venerida;Veneroidea;Veneridae;Mercenaria;Mercenari
a mercenaria
UdeM00000000668 1735272 591.7 UdeM00000000668 XP_041374670.1 76
425 85 9 16 430 28 445 7.80E-166 591.7 1735272
cellular
organisms;Eukaryota;Opisthokonta;Metazoa;Eumetazoa;Bilateria;Protostomia;
Spiralia;Lophotrochozoa;Mollusca;Gastropoda;Neomphalina;Neomphaloidea;Pel
tospiridae;Gigantopelta;Gigantopelta aegis
UdeM00000000666 6596 627.1 UdeM00000000666 XP_045179012.1 74.8 456
102 7 1 444 1 455 1.70E-176 627.1 6596 cellular
organisms;Eukaryota;Opisthokonta;Metazoa;Eumetazoa;Bilateria;Protostomia;
Spiralia;Lophotrochozoa;Mollusca;Bivalvia;Autobranchia;Heteroconchia;Euhe

terodonta;Imparidentia;Venerida;Veneroidea;Veneridae;Mercenaria;Mercenaria mercenaria
UdeM00000000667 6596 622.5 UdeM00000000667 XP_045179012.1 74.8 456 101 8 1 443 1 455 4.20E-175 622.5 6596 cellular organisms;Eukaryota;Opisthokonta;Metazoa;Eumetazoa;Bilateria;Protostomia;Spiralia;Lophotrochozoa;Mollusca;Bivalvia;Autobranchia;Heteroconchia;Euheterodonta;Imparidentia;Venerida;Veneroidea;Veneridae;Mercenaria;Mercenaria mercenaria
UdeM00000000669 114398 213.4 UdeM00000000669 XP_015930873.1 36.2 348 137 4 1 267 1 344 4.10E-52 213.4 114398 cellular organisms;Eukaryota;Opisthokonta;Metazoa;Eumetazoa;Bilateria;Protostomia;Ecdysozoa;Panarthropoda;Arthropoda;Chelicerata;Arachnida;Araneae;Araneomorphae;Entelegynae;Orbiculariae;Araneoidea;Theridiidae;Parasteatoda;Parasteatoda tepidariorum
UdeM00000000670 1529436 126.7 UdeM00000000670 XP_033114794.1 40.9 193 78 6 1 160 581 770 3.60E-26 126.7 1529436 cellular organisms;Eukaryota;Opisthokonta;Metazoa;Eumetazoa;Bilateria;Deuterostomia;Echinodermata;Pelmatozoa;Crinoidea;Articulata;Comatulida;Comatulidae;Comatulinae;Anneissia;Anneissia japonica
UdeM00000000671 1735272 79.7 UdeM00000000671 XP_041347149.1 43.6 101 50 2 1 96 257 355 2.40E-12 79.7 1735272 cellular organisms;Eukaryota;Opisthokonta;Metazoa;Eumetazoa;Bilateria;Protostomia;Spiralia;Lophotrochozoa;Mollusca;Gastropoda;Neomphalina;Neomphaloidea;Peltospiridae;Gigantopelta;Gigantopelta aegis
UdeM00000000672 37621 140.6 UdeM00000000672 XP_014679100.1 37.4 230 130 7 129 353 82 302 3.90E-30 140.6 37621 cellular organisms;Eukaryota;Opisthokonta;Metazoa;Eumetazoa;Bilateria;Protostomia;Ecdysozoa;Scalidophora;Priapulida;Priapulimorpha;Priapulimorphida;Priapulidae;Priapulus;Priapulus caudatus
UdeM00000000674 6565 166.4 UdeM00000000674 XP_022287521.1 46 187 66 1 275 426 180 366 8.00E-38 166.4 6565 cellular organisms;Eukaryota;Opisthokonta;Metazoa;Eumetazoa;Bilateria;Protostomia;Spiralia;Lophotrochozoa;Mollusca;Bivalvia;Autobranchia;Pteriomorphia;Ostreida;Ostreoidea;Ostreidae;Crassostrea;Crassostrea virginica
UdeM00000000676 6596 188.3 UdeM00000000676 XP_045161133.1 81.9 94 17 0 1 94 1 94 4.40E-45 188.3 6596 cellular organisms;Eukaryota;Opisthokonta;Metazoa;Eumetazoa;Bilateria;Protostomia;Spiralia;Lophotrochozoa;Mollusca;Bivalvia;Autobranchia;Heteroconchia;Euheterodonta;Imparidentia;Venerida;Veneroidea;Veneridae;Mercenaria;Mercenaria mercenaria
UdeM00000000678 1529436 203.8 UdeM00000000678 XP_033114794.1 44.9 263 130 6 1 251 581 840 3.10E-49 203.8 1529436 cellular organisms;Eukaryota;Opisthokonta;Metazoa;Eumetazoa;Bilateria;Deuterostomia;Echinodermata;Pelmatozoa;Crinoidea;Articulata;Comatulida;Comatulidae;Comatulinae;Anneissia;Anneissia japonica
UdeM00000000681 6565 61.6 UdeM00000000681 XP_022329522.1 70.3 37 11 0 138 174 426 462 1.10E-06 61.6 6565 cellular organisms;Eukaryota;Opisthokonta;Metazoa;Eumetazoa;Bilateria;Protostomia;Spiralia;Lophotrochozoa;Mollusca;Bivalvia;Autobranchia;Pteriomorphia;Ostreida;Ostreoidea;Ostreidae;Crassostrea;Crassostrea virginica

UdeM00000000683 6596 71.2 UdeM00000000683 XP_045192251.1 40.4 99
58 1 77 175 323 420 1.50E-09 71.2 6596 cellular
organisms;Eukaryota;Opisthokonta;Metazoa;Eumetazoa;Bilateria;Protostomia;
Spiralia;Lophotrochozoa;Mollusca;Bivalvia;Autobranchia;Heteroconchia;Euhe
terodonta;Imparidentia;Venerida;Veneroidea;Veneridae;Mercenaria;Mercenari
a mercenaria
UdeM00000000684 7739 78.2 UdeM00000000684 XP_035661789.1 29.8 248
140 8 1 228 354 587 1.60E-11 78.2 7739 cellular
organisms;Eukaryota;Opisthokonta;Metazoa;Eumetazoa;Bilateria;Deuterostomi
a;Chordata;Cephalochordata;Leptocardii;Amphioxiformes;Branchiostomidae;Br
anchiostoma;Branchiostoma floridae
UdeM00000000687 6596 2239.9 UdeM00000000687 XP_045179806.1 48
2541 1141 60 19 2449 102 2572 0.00E+00 2239.9 6596
cellular
organisms;Eukaryota;Opisthokonta;Metazoa;Eumetazoa;Bilateria;Protostomia;
Spiralia;Lophotrochozoa;Mollusca;Bivalvia;Autobranchia;Heteroconchia;Euhe
terodonta;Imparidentia;Venerida;Veneroidea;Veneridae;Mercenaria;Mercenari
a mercenaria
UdeM00000000686 6596 2266.9 UdeM00000000686 XP_045179806.1 46
2729 1194 62 19 2606 102 2692 0.00E+00 2266.9 6596
cellular
organisms;Eukaryota;Opisthokonta;Metazoa;Eumetazoa;Bilateria;Protostomia;
Spiralia;Lophotrochozoa;Mollusca;Bivalvia;Autobranchia;Heteroconchia;Euhe
terodonta;Imparidentia;Venerida;Veneroidea;Veneridae;Mercenaria;Mercenari
a mercenaria
UdeM00000000688 6573 1255.7 UdeM00000000688 XP_021351871.1 46.2
1733 798 47 63 1724 2575 4243 0.00E+00 1255.7 6573
cellular
organisms;Eukaryota;Opisthokonta;Metazoa;Eumetazoa;Bilateria;Protostomia;
Spiralia;Lophotrochozoa;Mollusca;Bivalvia;Autobranchia;Pteriomorphia;Pect
inida;Pectinoidea;Pectinidae;Mizuhopecten;Mizuhopecten yessoensis
UdeM00000000689 6579 286.6 UdeM00000000689 XP_033731015.1 37.3 440
271 3 1 439 6 441 5.40E-74 286.6 6579 cellular
organisms;Eukaryota;Opisthokonta;Metazoa;Eumetazoa;Bilateria;Protostomia;
Spiralia;Lophotrochozoa;Mollusca;Bivalvia;Autobranchia;Pteriomorphia;Pect
inida;Pectinoidea;Pectinidae;Pecten;Pecten maximus
UdeM00000000690 6579 295 UdeM00000000690 XP_033731015.1 39.3 440
258 5 7 446 13 443 1.60E-76 295 6579 cellular
organisms;Eukaryota;Opisthokonta;Metazoa;Eumetazoa;Bilateria;Protostomia;
Spiralia;Lophotrochozoa;Mollusca;Bivalvia;Autobranchia;Pteriomorphia;Pect
inida;Pectinoidea;Pectinidae;Pecten;Pecten maximus
UdeM00000000691 6596 293.1 UdeM00000000691 XP_045179280.1 39.9 406
220 3 5 410 48 429 5.50E-76 293.1 6596 cellular
organisms;Eukaryota;Opisthokonta;Metazoa;Eumetazoa;Bilateria;Protostomia;
Spiralia;Lophotrochozoa;Mollusca;Bivalvia;Autobranchia;Heteroconchia;Euhe
terodonta;Imparidentia;Venerida;Veneroidea;Veneridae;Mercenaria;Mercenari
a mercenaria
UdeM00000000692 7741 285 UdeM00000000692 XP_019622111.1 51.1 280
134 3 5 283 1 278 1.00E-73 285 7741 cellular
organisms;Eukaryota;Opisthokonta;Metazoa;Eumetazoa;Bilateria;Deuterostomi
a;Chordata;Cephalochordata;Leptocardii;Amphioxiformes;Branchiostomidae;Br
anchiostoma;Branchiostoma belcheri
UdeM00000000693 6596 987.6 UdeM00000000693 XP_045178348.1 67 815
220 13 5 797 60 847 1.10E-284 987.6 6596 cellular

organisms;Eukaryota;Opisthokonta;Metazoa;Eumetazoa;Bilateria;Protostomia;Spiralia;Lophotrochozoa;Mollusca;Bivalvia;Autobranchia;Heteroconchia;Euheterodonta;Imparidentia;Venerida;Veneroidea;Veneridae;Mercenaria;Mercenaria mercenaria
UdeM00000000694 6596 1048.1 UdeM00000000694 XP_045178348.1 65.3 886 256 15 5 867 60 917 7.70E-303 1048.1 6596 cellular organisms;Eukaryota;Opisthokonta;Metazoa;Eumetazoa;Bilateria;Protostomia;Spiralia;Lophotrochozoa;Mollusca;Bivalvia;Autobranchia;Heteroconchia;Euheterodonta;Imparidentia;Venerida;Veneroidea;Veneridae;Mercenaria;Mercenaria mercenaria
UdeM00000000696 6596 380.9 UdeM00000000696 XP_045162792.1 46.5 477 235 5 11 487 14 470 2.40E-102 380.9 6596 cellular organisms;Eukaryota;Opisthokonta;Metazoa;Eumetazoa;Bilateria;Protostomia;Spiralia;Lophotrochozoa;Mollusca;Bivalvia;Autobranchia;Heteroconchia;Euheterodonta;Imparidentia;Venerida;Veneroidea;Veneridae;Mercenaria;Mercenaria mercenaria
UdeM00000000697 1735272 481.9 UdeM00000000697 XP_041365458.1 44.3 578 302 7 6 580 31 591 1.20E-132 481.9 1735272 cellular organisms;Eukaryota;Opisthokonta;Metazoa;Eumetazoa;Bilateria;Protostomia;Spiralia;Lophotrochozoa;Mollusca;Gastropoda;Neomphalina;Neomphaloidea;Peltospiridae;Gigantopelta;Gigantopelta aegis
UdeM00000000698 6596 469.2 UdeM00000000698 XP_045177468.1 50.6 539 223 11 1 533 2 503 7.20E-129 469.2 6596 cellular organisms;Eukaryota;Opisthokonta;Metazoa;Eumetazoa;Bilateria;Protostomia;Spiralia;Lophotrochozoa;Mollusca;Bivalvia;Autobranchia;Heteroconchia;Euheterodonta;Imparidentia;Venerida;Veneroidea;Veneridae;Mercenaria;Mercenaria mercenaria
UdeM00000000699 6596 129.4 UdeM00000000699 XP_045168144.1 29.6 324 208 7 77 390 551 864 1.10E-26 129.4 6596 cellular organisms;Eukaryota;Opisthokonta;Metazoa;Eumetazoa;Bilateria;Protostomia;Spiralia;Lophotrochozoa;Mollusca;Bivalvia;Autobranchia;Heteroconchia;Euheterodonta;Imparidentia;Venerida;Veneroidea;Veneridae;Mercenaria;Mercenaria mercenaria
UdeM00000000699 6596 79.3 UdeM00000000699 XP_045168144.1 33.7 104 69 0 4 107 17 120 1.30E-11 79.3 6596 cellular organisms;Eukaryota;Opisthokonta;Metazoa;Eumetazoa;Bilateria;Protostomia;Spiralia;Lophotrochozoa;Mollusca;Bivalvia;Autobranchia;Heteroconchia;Euheterodonta;Imparidentia;Venerida;Veneroidea;Veneridae;Mercenaria;Mercenaria mercenaria
UdeM00000000700 6596 129.4 UdeM00000000700 XP_045168144.1 29.6 324 208 7 77 390 551 864 1.10E-26 129.4 6596 cellular organisms;Eukaryota;Opisthokonta;Metazoa;Eumetazoa;Bilateria;Protostomia;Spiralia;Lophotrochozoa;Mollusca;Bivalvia;Autobranchia;Heteroconchia;Euheterodonta;Imparidentia;Venerida;Veneroidea;Veneridae;Mercenaria;Mercenaria mercenaria
UdeM00000000700 6596 79.3 UdeM00000000700 XP_045168144.1 33.7 104 69 0 4 107 17 120 1.30E-11 79.3 6596 cellular organisms;Eukaryota;Opisthokonta;Metazoa;Eumetazoa;Bilateria;Protostomia;Spiralia;Lophotrochozoa;Mollusca;Bivalvia;Autobranchia;Heteroconchia;Euheterodonta;Imparidentia;Venerida;Veneroidea;Veneridae;Mercenaria;Mercenaria mercenaria

UdeM00000000701 6596 129.4 UdeM00000000701 XP_045168144.1 29.6 324
208 7 77 390 551 864 1.10E-26 129.4 6596 cellular
organisms;Eukaryota;Opisthokonta;Metazoa;Eumetazoa;Bilateria;Protostomia;
Spiralia;Lophotrochozoa;Mollusca;Bivalvia;Autobranchia;Heteroconchia;Euhe
terodonta;Imparidentia;Venerida;Veneroidea;Veneridae;Mercenaria;Mercenari
a mercenaria
UdeM00000000701 6596 79.3 UdeM00000000701 XP_045168144.1 33.7 104
69 0 4 107 17 120 1.30E-11 79.3 6596 cellular
organisms;Eukaryota;Opisthokonta;Metazoa;Eumetazoa;Bilateria;Protostomia;
Spiralia;Lophotrochozoa;Mollusca;Bivalvia;Autobranchia;Heteroconchia;Euhe
terodonta;Imparidentia;Venerida;Veneroidea;Veneridae;Mercenaria;Mercenari
a mercenaria
UdeM00000000702 6596 328.9 UdeM00000000702 XP_045162799.1 45 456
222 5 12 439 15 469 9.90E-87 328.9 6596 cellular
organisms;Eukaryota;Opisthokonta;Metazoa;Eumetazoa;Bilateria;Protostomia;
Spiralia;Lophotrochozoa;Mollusca;Bivalvia;Autobranchia;Heteroconchia;Euhe
terodonta;Imparidentia;Venerida;Veneroidea;Veneridae;Mercenaria;Mercenari
a mercenaria
UdeM00000000703 6596 401.7 UdeM00000000703 XP_045162799.1 48.2 483
220 4 10 487 13 470 1.30E-108 401.7 6596 cellular
organisms;Eukaryota;Opisthokonta;Metazoa;Eumetazoa;Bilateria;Protostomia;
Spiralia;Lophotrochozoa;Mollusca;Bivalvia;Autobranchia;Heteroconchia;Euhe
terodonta;Imparidentia;Venerida;Veneroidea;Veneridae;Mercenaria;Mercenari
a mercenaria
UdeM00000000704 6573 283.9 UdeM00000000704 XP_021354145.1 45 322
171 3 1 318 269 588 2.60E-73 283.9 6573 cellular
organisms;Eukaryota;Opisthokonta;Metazoa;Eumetazoa;Bilateria;Protostomia;
Spiralia;Lophotrochozoa;Mollusca;Bivalvia;Autobranchia;Pteriomorphia;Pect
inida;Pectinoidea;Pectinidae;Mizuhopecten;Mizuhopecten yessoensis
UdeM00000000705 400727 369 UdeM00000000705 XP_025091384.1 42.8
458 247 6 5 459 148 593 8.70E-99 369 400727
cellular
organisms;Eukaryota;Opisthokonta;Metazoa;Eumetazoa;Bilateria;Protostomia;
Spiralia;Lophotrochozoa;Mollusca;Gastropoda;Caenogastropoda;Architaeniogl
ossa;Ampullarioidea;Ampullariidae;Pomacea;Pomacea canaliculata
UdeM00000000706 307959 235.7 UdeM00000000706 XP_016384368.1 40.6
281 136 2 34 314 83 332 1.10E-58 235.7 307959
cellular
organisms;Eukaryota;Opisthokonta;Metazoa;Eumetazoa;Bilateria;Deuterostomi
a;Chordata;Craniata;Vertebrata;Gnathostomata;Teleostomi;Euteleostomi;Acti
nopterygii;Actinopteri;Neopterygii;Teleostei;Osteoglossocephalai;Clupeoce
phala;Otomorpha;Ostariophysi;Otophysi;Cypriniphysae;Cypriniformes;Cyprino
idei;Cyprinidae;Cyprininae;Sinocyclocheilus;Sinocyclocheilus rhinocerous
UdeM00000000707 6596 426 UdeM00000000707 XP_045209095.1 50.1 441
205 7 1 430 1 437 8.70E-116 426 6596 cellular
organisms;Eukaryota;Opisthokonta;Metazoa;Eumetazoa;Bilateria;Protostomia;
Spiralia;Lophotrochozoa;Mollusca;Bivalvia;Autobranchia;Heteroconchia;Euhe
terodonta;Imparidentia;Venerida;Veneroidea;Veneridae;Mercenaria;Mercenari
a mercenaria
UdeM00000000708 6565 683.3 UdeM00000000708 XP_022327155.1 44.7 1095
488 16 4139 5138 3239 4311 2.30E-192 683.3 6565 cellular
organisms;Eukaryota;Opisthokonta;Metazoa;Eumetazoa;Bilateria;Protostomia;
Spiralia;Lophotrochozoa;Mollusca;Bivalvia;Autobranchia;Pteriomorphia;Ostr
eida;Ostreoidea;Ostreidae;Crassostrea;Crassostrea virginica

UdeM00000000709 29159 177.6 UdeM00000000709 XP_011423571.1 74.4 117
26 2 1 116 1 114 9.50E-42 177.6 29159 cellular
organisms;Eukaryota;Opisthokonta;Metazoa;Eumetazoa;Bilateria;Protostomia;
Spiralia;Lophotrochozoa;Mollusca;Bivalvia;Autobranchia;Pteriomorphia;Ostr
eida;Ostreoidea;Ostreidae;Crassostrea;Crassostrea gigas
UdeM00000000710 6579 269.6 UdeM00000000710 XP_033729083.1 48.3 478
222 8 15 476 10 478 9.50E-69 269.6 6579 cellular
organisms;Eukaryota;Opisthokonta;Metazoa;Eumetazoa;Bilateria;Protostomia;
Spiralia;Lophotrochozoa;Mollusca;Bivalvia;Autobranchia;Pteriomorphia;Pect
inida;Pectinoidea;Pectinidae;Pecten;Pecten maximus
UdeM00000000711 6596 347.4 UdeM00000000711 XP_045178021.1 47 696
258 19 83 690 584 1256 5.60E-92 347.4 6596 cellular
organisms;Eukaryota;Opisthokonta;Metazoa;Eumetazoa;Bilateria;Protostomia;
Spiralia;Lophotrochozoa;Mollusca;Bivalvia;Autobranchia;Heteroconchia;Euhe
terodonta;Imparidentia;Venerida;Veneroidea;Veneridae;Mercenaria;Mercenari
a mercenaria
UdeM00000000712 7654 81.3 UdeM00000000712 XP_041484611.1 37.1 132
62 2 11 121 392 523 1.10E-12 81.3 7654 cellular
organisms;Eukaryota;Opisthokonta;Metazoa;Eumetazoa;Bilateria;Deuterostomi
a;Echinodermata;Eleutherozoa;Echinozoa;Echinoidea;Euechinoidea;Echinacea;
Temnopleuroida;Toxopneustidae;Lytechinus;Lytechinus variegatus
UdeM00000000713 6596 236.1 UdeM00000000713 XP_045178682.1 49.2 242
122 1 21 262 31 271 5.00E-59 236.1 6596 cellular
organisms;Eukaryota;Opisthokonta;Metazoa;Eumetazoa;Bilateria;Protostomia;
Spiralia;Lophotrochozoa;Mollusca;Bivalvia;Autobranchia;Heteroconchia;Euhe
terodonta;Imparidentia;Venerida;Veneroidea;Veneridae;Mercenaria;Mercenari
a mercenaria
UdeM00000000714 29159 463 UdeM00000000714 XP_011442807.3 48.9 481
214 8 9 486 10 461 4.80E-127 463 29159 cellular
organisms;Eukaryota;Opisthokonta;Metazoa;Eumetazoa;Bilateria;Protostomia;
Spiralia;Lophotrochozoa;Mollusca;Bivalvia;Autobranchia;Pteriomorphia;Ostr
eida;Ostreoidea;Ostreidae;Crassostrea;Crassostrea gigas
UdeM00000000715 37653 657.5 UdeM00000000715 XP_014768102.1 65.8 447
142 2 1 438 1 445 1.20E-185 657.5 37653 cellular
organisms;Eukaryota;Opisthokonta;Metazoa;Eumetazoa;Bilateria;Protostomia;
Spiralia;Lophotrochozoa;Mollusca;Cephalopoda;Coleoidea;Octopodiformes;Oct
opoda;Incirrata;Octopodidae;Octopus;Octopus bimaculoides
UdeM00000000716 6565 725.3 UdeM00000000716 XP_022336131.1 36.3 1323
738 49 120 1401 147 1405 1.50E-205 725.3 6565 cellular
organisms;Eukaryota;Opisthokonta;Metazoa;Eumetazoa;Bilateria;Protostomia;
Spiralia;Lophotrochozoa;Mollusca;Bivalvia;Autobranchia;Pteriomorphia;Ostr
eida;Ostreoidea;Ostreidae;Crassostrea;Crassostrea virginica
UdeM00000000717 6579 150.2 UdeM00000000717 XP_033756913.1 38.5 200
110 5 80 276 132 321 4.00E-33 150.2 6579 cellular
organisms;Eukaryota;Opisthokonta;Metazoa;Eumetazoa;Bilateria;Protostomia;
Spiralia;Lophotrochozoa;Mollusca;Bivalvia;Autobranchia;Pteriomorphia;Pect
inida;Pectinoidea;Pectinidae;Pecten;Pecten maximus
UdeM00000000718 6596 268.5 UdeM00000000718 XP_045216525.1 42.3 385
196 14 1 378 315 680 1.50E-68 268.5 6596 cellular
organisms;Eukaryota;Opisthokonta;Metazoa;Eumetazoa;Bilateria;Protostomia;
Spiralia;Lophotrochozoa;Mollusca;Bivalvia;Autobranchia;Heteroconchia;Euhe
terodonta;Imparidentia;Venerida;Veneroidea;Veneridae;Mercenaria;Mercenari
a mercenaria

UdeM00000000719 6596 303.9 UdeM00000000719 XP_045216525.1 40.9 491
227 21 77 529 160 625 4.20E-79 303.9 6596 cellular
organisms;Eukaryota;Opisthokonta;Metazoa;Eumetazoa;Bilateria;Protostomia;
Spiralia;Lophotrochozoa;Mollusca;Bivalvia;Autobranchia;Heteroconchia;Euhe
terodonta;Imparidentia;Venerida;Veneroidea;Veneridae;Mercenaria;Mercenari
a mercenaria
UdeM00000044262 6573 109.4 UdeM00000044262 XP_021359218.1 50 104
52 0 82 185 375 478 5.10E-21 109.4 6573 cellular
organisms;Eukaryota;Opisthokonta;Metazoa;Eumetazoa;Bilateria;Protostomia;
Spiralia;Lophotrochozoa;Mollusca;Bivalvia;Autobranchia;Pteriomorphia;Pect
inida;Pectinoidea;Pectinidae;Mizuhopecten;Mizuhopecten yessoensis
UdeM00000044263 6596 126.7 UdeM00000044263 XP_045204839.1 29.8 305
191 8 217 506 325 621 8.50E-26 126.7 6596 cellular
organisms;Eukaryota;Opisthokonta;Metazoa;Eumetazoa;Bilateria;Protostomia;
Spiralia;Lophotrochozoa;Mollusca;Bivalvia;Autobranchia;Heteroconchia;Euhe
terodonta;Imparidentia;Venerida;Veneroidea;Veneridae;Mercenaria;Mercenari
a mercenaria
UdeM00000044264 6596 93.6 UdeM00000044264 XP_045204839.1 34.7 118
73 1 619 736 508 621 1.20E-15 93.6 6596 cellular
organisms;Eukaryota;Opisthokonta;Metazoa;Eumetazoa;Bilateria;Protostomia;
Spiralia;Lophotrochozoa;Mollusca;Bivalvia;Autobranchia;Heteroconchia;Euhe
terodonta;Imparidentia;Venerida;Veneroidea;Veneridae;Mercenaria;Mercenari
a mercenaria
UdeM00000044265 6596 93.6 UdeM00000044265 XP_045204839.1 34.7 118
73 1 616 733 508 621 1.10E-15 93.6 6596 cellular
organisms;Eukaryota;Opisthokonta;Metazoa;Eumetazoa;Bilateria;Protostomia;
Spiralia;Lophotrochozoa;Mollusca;Bivalvia;Autobranchia;Heteroconchia;Euhe
terodonta;Imparidentia;Venerida;Veneroidea;Veneridae;Mercenaria;Mercenari
a mercenaria
UdeM00000044267 6596 93.6 UdeM00000044267 XP_045204839.1 34.7 118
73 1 380 497 508 621 7.80E-16 93.6 6596 cellular
organisms;Eukaryota;Opisthokonta;Metazoa;Eumetazoa;Bilateria;Protostomia;
Spiralia;Lophotrochozoa;Mollusca;Bivalvia;Autobranchia;Heteroconchia;Euhe
terodonta;Imparidentia;Venerida;Veneroidea;Veneridae;Mercenaria;Mercenari
a mercenaria
UdeM00000044268 6565 100.1 UdeM00000044268 XP_022324784.1 49 102
52 0 537 638 594 695 1.40E-17 100.1 6565 cellular
organisms;Eukaryota;Opisthokonta;Metazoa;Eumetazoa;Bilateria;Protostomia;
Spiralia;Lophotrochozoa;Mollusca;Bivalvia;Autobranchia;Pteriomorphia;Ostr
eida;Ostreoidea;Ostreidae;Crassostrea;Crassostrea virginica
UdeM00000044269 6565 100.1 UdeM00000044269 XP_022324784.1 49 102
52 0 546 647 594 695 1.40E-17 100.1 6565 cellular
organisms;Eukaryota;Opisthokonta;Metazoa;Eumetazoa;Bilateria;Protostomia;
Spiralia;Lophotrochozoa;Mollusca;Bivalvia;Autobranchia;Pteriomorphia;Ostr
eida;Ostreoidea;Ostreidae;Crassostrea;Crassostrea virginica
UdeM00000044270 6565 100.1 UdeM00000044270 XP_022324784.1 49 102
52 0 484 585 594 695 1.30E-17 100.1 6565 cellular
organisms;Eukaryota;Opisthokonta;Metazoa;Eumetazoa;Bilateria;Protostomia;
Spiralia;Lophotrochozoa;Mollusca;Bivalvia;Autobranchia;Pteriomorphia;Ostr
eida;Ostreoidea;Ostreidae;Crassostrea;Crassostrea virginica
UdeM00000041109 6579 124.4 UdeM00000041109 XP_033742496.1 37.4 163
98 2 1 159 255 417 1.30E-25 124.4 6579 cellular
organisms;Eukaryota;Opisthokonta;Metazoa;Eumetazoa;Bilateria;Protostomia;

Spiralia;Lophotrochozoa;Mollusca;Bivalvia;Autobranchia;Pteriomorphia;Pectinida;Pectinoidea;Pectinidae;Pecten;Pecten maximus
UdeM00000033841 6579 515.8 UdeM00000033841 XP_033734426.1 77.1 423 85 4 83 502 99 512 6.30E-143 515.8 6579 cellular organisms;Eukaryota;Opisthokonta;Metazoa;Eumetazoa;Bilateria;Protostomia;Spiralia;Lophotrochozoa;Mollusca;Bivalvia;Autobranchia;Pteriomorphia;Pectinida;Pectinoidea;Pectinidae;Pecten;Pecten maximus
UdeM00000033842 6596 956.8 UdeM00000033842 XP_045182299.1 58.9 757 303 3 18 773 15 764 1.60E-275 956.8 6596 cellular organisms;Eukaryota;Opisthokonta;Metazoa;Eumetazoa;Bilateria;Protostomia;Spiralia;Lophotrochozoa;Mollusca;Bivalvia;Autobranchia;Heteroconchia;Euheterodonta;Imparidentia;Venerida;Veneroidea;Veneridae;Mercenaria;Mercenaria mercenaria
UdeM00000033843 29159 84.7 UdeM00000033843 XP_011415139.2 34.2 158 33 1 41 127 61 218 9.10E-14 84.7 29159 cellular organisms;Eukaryota;Opisthokonta;Metazoa;Eumetazoa;Bilateria;Protostomia;Spiralia;Lophotrochozoa;Mollusca;Bivalvia;Autobranchia;Pteriomorphia;Ostreida;Ostreoidea;Ostreidae;Crassostrea;Crassostrea gigas
UdeM00000033844 6596 871.7 UdeM00000033844 XP_045182299.1 55.7 734 288 5 35 738 38 764 6.60E-250 871.7 6596 cellular organisms;Eukaryota;Opisthokonta;Metazoa;Eumetazoa;Bilateria;Protostomia;Spiralia;Lophotrochozoa;Mollusca;Bivalvia;Autobranchia;Heteroconchia;Euheterodonta;Imparidentia;Venerida;Veneroidea;Veneridae;Mercenaria;Mercenaria mercenaria
UdeM00000033848 6596 106.7 UdeM00000033848 XP_045213058.1 28.3 279 113 8 286 481 87 361 9.20E-20 106.7 6596 cellular organisms;Eukaryota;Opisthokonta;Metazoa;Eumetazoa;Bilateria;Protostomia;Spiralia;Lophotrochozoa;Mollusca;Bivalvia;Autobranchia;Heteroconchia;Euheterodonta;Imparidentia;Venerida;Veneroidea;Veneridae;Mercenaria;Mercenaria mercenaria
UdeM00000033849 6596 223.8 UdeM00000033849 XP_045198257.1 37.2 285 177 1 3 287 123 405 2.80E-55 223.8 6596 cellular organisms;Eukaryota;Opisthokonta;Metazoa;Eumetazoa;Bilateria;Protostomia;Spiralia;Lophotrochozoa;Mollusca;Bivalvia;Autobranchia;Heteroconchia;Euheterodonta;Imparidentia;Venerida;Veneroidea;Veneridae;Mercenaria;Mercenaria mercenaria
UdeM00000033850 6573 72 UdeM00000033850 XP_021352480.1 30.2 278 169 11 34 302 49 310 1.40E-09 72 6573 cellular organisms;Eukaryota;Opisthokonta;Metazoa;Eumetazoa;Bilateria;Protostomia;Spiralia;Lophotrochozoa;Mollusca;Bivalvia;Autobranchia;Pteriomorphia;Pectinida;Pectinoidea;Pectinidae;Mizuhopecten;Mizuhopecten yessoensis
UdeM00000033851 6596 157.5 UdeM00000033851 XP_045165790.1 58.5 118 49 0 1 118 61 178 1.10E-35 157.5 6596 cellular organisms;Eukaryota;Opisthokonta;Metazoa;Eumetazoa;Bilateria;Protostomia;Spiralia;Lophotrochozoa;Mollusca;Bivalvia;Autobranchia;Heteroconchia;Euheterodonta;Imparidentia;Venerida;Veneroidea;Veneridae;Mercenaria;Mercenaria mercenaria
UdeM00000033852 6596 240 UdeM00000033852 XP_045165790.1 65.5 168 57 1 1 167 11 178 2.30E-60 240 6596 cellular organisms;Eukaryota;Opisthokonta;Metazoa;Eumetazoa;Bilateria;Protostomia;Spiralia;Lophotrochozoa;Mollusca;Bivalvia;Autobranchia;Heteroconchia;Euheterodonta;Imparidentia;Venerida;Veneroidea;Veneridae;Mercenaria;Mercenaria mercenaria

UdeM00000033853 6596 182.2 UdeM00000033853 XP_045188028.1 57.5 134
57 0 1 134 12 145 4.50E-43 182.2 6596 cellular
organisms;Eukaryota;Opisthokonta;Metazoa;Eumetazoa;Bilateria;Protostomia;
Spiralia;Lophotrochozoa;Mollusca;Bivalvia;Autobranchia;Heteroconchia;Euhe
terodonta;Imparidentia;Venerida;Veneroidea;Veneridae;Mercenaria;Mercenari
a mercenaria
UdeM00000033854 6596 160.6 UdeM00000033854 XP_045188028.1 53.6 125
58 0 21 145 21 145 1.50E-36 160.6 6596 cellular
organisms;Eukaryota;Opisthokonta;Metazoa;Eumetazoa;Bilateria;Protostomia;
Spiralia;Lophotrochozoa;Mollusca;Bivalvia;Autobranchia;Heteroconchia;Euhe
terodonta;Imparidentia;Venerida;Veneroidea;Veneridae;Mercenaria;Mercenari
a mercenaria
UdeM00000033855 6596 361.3 UdeM00000033855 XP_045167151.1 35.3 641
394 13 201 832 136 764 3.70E-96 361.3 6596 cellular
organisms;Eukaryota;Opisthokonta;Metazoa;Eumetazoa;Bilateria;Protostomia;
Spiralia;Lophotrochozoa;Mollusca;Bivalvia;Autobranchia;Heteroconchia;Euhe
terodonta;Imparidentia;Venerida;Veneroidea;Veneridae;Mercenaria;Mercenari
a mercenaria
UdeM00000033856 6596 1015 UdeM00000033856 XP_045187456.1 74 701
180 2 10 709 8 707 4.70E-293 1015 6596 cellular
organisms;Eukaryota;Opisthokonta;Metazoa;Eumetazoa;Bilateria;Protostomia;
Spiralia;Lophotrochozoa;Mollusca;Bivalvia;Autobranchia;Heteroconchia;Euhe
terodonta;Imparidentia;Venerida;Veneroidea;Veneridae;Mercenaria;Mercenari
a mercenaria
UdeM00000033857 6596 402.1 UdeM00000033857 XP_045187456.1 74.8 305
76 1 6 310 404 707 6.30E-109 402.1 6596 cellular
organisms;Eukaryota;Opisthokonta;Metazoa;Eumetazoa;Bilateria;Protostomia;
Spiralia;Lophotrochozoa;Mollusca;Bivalvia;Autobranchia;Heteroconchia;Euhe
terodonta;Imparidentia;Venerida;Veneroidea;Veneridae;Mercenaria;Mercenari
a mercenaria
UdeM00000033858 6565 530 UdeM00000033858 XP_022331125.1 82.6 299
51 1 12 309 57 355 2.00E-147 530 6565 cellular
organisms;Eukaryota;Opisthokonta;Metazoa;Eumetazoa;Bilateria;Protostomia;
Spiralia;Lophotrochozoa;Mollusca;Bivalvia;Autobranchia;Pteriomorphia;Ostr
eida;Ostreoidea;Ostreidae;Crassostrea;Crassostrea virginica
UdeM00000033859 29159 186.8 UdeM00000033859 XP_034331441.1 43.4 219
119 3 15 230 1 217 6.40E-44 186.8 29159 cellular
organisms;Eukaryota;Opisthokonta;Metazoa;Eumetazoa;Bilateria;Protostomia;
Spiralia;Lophotrochozoa;Mollusca;Bivalvia;Autobranchia;Pteriomorphia;Ostr
eida;Ostreoidea;Ostreidae;Crassostrea;Crassostrea gigas
UdeM00000033860 7739 154.5 UdeM00000033860 XP_035659254.1 43.3 180
96 2 5 178 753 932 1.80E-34 154.5 7739 cellular
organisms;Eukaryota;Opisthokonta;Metazoa;Eumetazoa;Bilateria;Deuterostomi
a;Chordata;Cephalochordata;Leptocardii;Amphioxiformes;Branchiostomidae;Br
anchiostoma;Branchiostoma floridae
UdeM00000033861 6596 231.9 UdeM00000033861 XP_045187458.1 56.6 272
110 2 91 354 53 324 1.40E-57 231.9 6596 cellular
organisms;Eukaryota;Opisthokonta;Metazoa;Eumetazoa;Bilateria;Protostomia;
Spiralia;Lophotrochozoa;Mollusca;Bivalvia;Autobranchia;Heteroconchia;Euhe
terodonta;Imparidentia;Venerida;Veneroidea;Veneridae;Mercenaria;Mercenari
a mercenaria
UdeM00000033862 6596 171.8 UdeM00000033862 XP_045186984.1 39.1 294
126 10 1 246 5 293 1.20E-39 171.8 6596 cellular
organisms;Eukaryota;Opisthokonta;Metazoa;Eumetazoa;Bilateria;Protostomia;

Spiralia;Lophotrochozoa;Mollusca;Bivalvia;Autobranchia;Heteroconchia;Euheterodonta;Imparidentia;Venerida;Veneroidea;Veneridae;Mercenaria;Mercenaria mercenaria
UdeM00000033863 6596 200.7 UdeM00000033863 XP_045186985.1 41.9 296 127 11 1 256 5 295 2.30E-48 200.7 6596 cellular organisms;Eukaryota;Opisthokonta;Metazoa;Eumetazoa;Bilateria;Protostomia;Spiralia;Lophotrochozoa;Mollusca;Bivalvia;Autobranchia;Heteroconchia;Euheterodonta;Imparidentia;Venerida;Veneroidea;Veneridae;Mercenaria;Mercenaria mercenaria
UdeM00000033864 6565 107.5 UdeM00000033864 XP_022331458.1 31.5 248 151 5 41 284 63 295 4.40E-20 107.5 6565 cellular organisms;Eukaryota;Opisthokonta;Metazoa;Eumetazoa;Bilateria;Protostomia;Spiralia;Lophotrochozoa;Mollusca;Bivalvia;Autobranchia;Pteriomorphia;Ostreida;Ostreoidea;Ostreidae;Crassostrea;Crassostrea virginica
UdeM00000033865 1735272 59.7 UdeM00000033865 XP_041373661.1 38.8 67 40 1 1 66 4 70 4.30E-06 59.7 1735272 cellular organisms;Eukaryota;Opisthokonta;Metazoa;Eumetazoa;Bilateria;Protostomia;Spiralia;Lophotrochozoa;Mollusca;Gastropoda;Neomphalina;Neomphaloidea;Peltospiridae;Gigantopelta;Gigantopelta aegis
UdeM00000033866 6579 1127.5 UdeM00000033866 XP_033734163.1 51.4 1085 512 8 1 1078 1 1077 0.00E+00 1127.5 6579 cellular organisms;Eukaryota;Opisthokonta;Metazoa;Eumetazoa;Bilateria;Protostomia;Spiralia;Lophotrochozoa;Mollusca;Bivalvia;Autobranchia;Pteriomorphia;Pectinida;Pectinoidea;Pectinidae;Pecten;Pecten maximus
UdeM00000033868 225164 139 UdeM00000033868 XP_009060494.1 35.4 209 123 4 33 229 62 270 7.60E-30 139 225164 cellular organisms;Eukaryota;Opisthokonta;Metazoa;Eumetazoa;Bilateria;Protostomia;Spiralia;Lophotrochozoa;Mollusca;Gastropoda;Patellogastropoda;Lottioidea;Lottiidae;Lottia;Lottia gigantea
UdeM00000033869 6565 162.2 UdeM00000033869 XP_022333543.1 33.9 239 151 3 14 249 51 285 8.80E-37 162.2 6565 cellular organisms;Eukaryota;Opisthokonta;Metazoa;Eumetazoa;Bilateria;Protostomia;Spiralia;Lophotrochozoa;Mollusca;Bivalvia;Autobranchia;Pteriomorphia;Ostreida;Ostreoidea;Ostreidae;Crassostrea;Crassostrea virginica
UdeM00000033870 1735272 157.9 UdeM00000033870 XP_041351938.1 39.8 259 140 7 3 249 464 718 1.70E-35 157.9 1735272 cellular organisms;Eukaryota;Opisthokonta;Metazoa;Eumetazoa;Bilateria;Protostomia;Spiralia;Lophotrochozoa;Mollusca;Gastropoda;Neomphalina;Neomphaloidea;Peltospiridae;Gigantopelta;Gigantopelta aegis
UdeM00000033871 1735272 63.9 UdeM00000033871 XP_041351938.1 23.7 262 178 7 35 286 223 472 4.20E-07 63.9 1735272 cellular organisms;Eukaryota;Opisthokonta;Metazoa;Eumetazoa;Bilateria;Protostomia;Spiralia;Lophotrochozoa;Mollusca;Gastropoda;Neomphalina;Neomphaloidea;Peltospiridae;Gigantopelta;Gigantopelta aegis
UdeM00000033872 400727 125.2 UdeM00000033872 XP_025105569.1 41.2 148 86 1 15 162 49 195 7.90E-26 125.2 400727 cellular organisms;Eukaryota;Opisthokonta;Metazoa;Eumetazoa;Bilateria;Protostomia;

Spiralia;Lophotrochozoa;Mollusca;Gastropoda;Caenogastropoda;Architaenioglossa;Ampullarioidea;Ampullariidae;Pomacea;Pomacea canaliculata
UdeM00000033873 6565 1333.2 UdeM00000033873 XP_022332215.1 68.9 936 279 4 1 933 16 942 0.00E+00 1333.2 6565 cellular organisms;Eukaryota;Opisthokonta;Metazoa;Eumetazoa;Bilateria;Protostomia;Spiralia;Lophotrochozoa;Mollusca;Bivalvia;Autobranchia;Pteriomorphia;Ostreida;Ostreoidea;Ostreidae;Crassostrea;Crassostrea virginica
UdeM00000033874 6579 365.5 UdeM00000033874 XP_033735988.1 64.4 250 89 0 7 256 38 287 5.40E-98 365.5 6579 cellular organisms;Eukaryota;Opisthokonta;Metazoa;Eumetazoa;Bilateria;Protostomia;Spiralia;Lophotrochozoa;Mollusca;Bivalvia;Autobranchia;Pteriomorphia;Pectinida;Pectinoidea;Pectinidae;Pecten;Pecten maximus
UdeM00000033875 29159 64.7 UdeM00000033875 XP_034336196.1 28.6 112 75 2 35 143 565 674 1.20E-07 64.7 29159 cellular organisms;Eukaryota;Opisthokonta;Metazoa;Eumetazoa;Bilateria;Protostomia;Spiralia;Lophotrochozoa;Mollusca;Bivalvia;Autobranchia;Pteriomorphia;Ostreida;Ostreoidea;Ostreidae;Crassostrea;Crassostrea gigas
UdeM00000033877 6565 65.5 UdeM00000033877 XP_022334490.1 36.6 101 59 3 45 144 245 341 6.70E-08 65.5 6565 cellular organisms;Eukaryota;Opisthokonta;Metazoa;Eumetazoa;Bilateria;Protostomia;Spiralia;Lophotrochozoa;Mollusca;Bivalvia;Autobranchia;Pteriomorphia;Ostreida;Ostreoidea;Ostreidae;Crassostrea;Crassostrea virginica
UdeM00000033878 6596 82.4 UdeM00000033878 XP_045177449.1 42.2 135 63 7 14 138 368 497 5.00E-13 82.4 6596 cellular organisms;Eukaryota;Opisthokonta;Metazoa;Eumetazoa;Bilateria;Protostomia;Spiralia;Lophotrochozoa;Mollusca;Bivalvia;Autobranchia;Heteroconchia;Euheterodonta;Imparidentia;Venerida;Veneroidea;Veneridae;Mercenaria;Mercenaria mercenaria
UdeM00000033879 7739 775 UdeM00000033879 XP_035696678.1 55.3 683 299 3 208 884 202 884 1.00E-220 775 7739 cellular organisms;Eukaryota;Opisthokonta;Metazoa;Eumetazoa;Bilateria;Deuterostomia;Chordata;Cephalochordata;Leptocardii;Amphioxiformes;Branchiostomidae;Branchiostoma;Branchiostoma floridae
UdeM00000033880 6596 379.4 UdeM00000033880 XP_045169901.1 41.2 514 292 4 8 518 5 511 7.50E-102 379.4 6596 cellular organisms;Eukaryota;Opisthokonta;Metazoa;Eumetazoa;Bilateria;Protostomia;Spiralia;Lophotrochozoa;Mollusca;Bivalvia;Autobranchia;Heteroconchia;Euheterodonta;Imparidentia;Venerida;Veneroidea;Veneridae;Mercenaria;Mercenaria mercenaria
UdeM00000033881 6596 112.1 UdeM00000033881 XP_045179939.1 42.3 130 72 2 57 183 251 380 7.70E-22 112.1 6596 cellular organisms;Eukaryota;Opisthokonta;Metazoa;Eumetazoa;Bilateria;Protostomia;Spiralia;Lophotrochozoa;Mollusca;Bivalvia;Autobranchia;Heteroconchia;Euheterodonta;Imparidentia;Venerida;Veneroidea;Veneridae;Mercenaria;Mercenaria mercenaria
UdeM00000033883 6596 118.2 UdeM00000033883 XP_045179939.1 43.8 130 70 2 260 386 251 380 2.30E-23 118.2 6596 cellular organisms;Eukaryota;Opisthokonta;Metazoa;Eumetazoa;Bilateria;Protostomia;Spiralia;Lophotrochozoa;Mollusca;Bivalvia;Autobranchia;Heteroconchia;Euheterodonta;Imparidentia;Venerida;Veneroidea;Veneridae;Mercenaria;Mercenaria mercenaria
UdeM00000033884 6565 276.2 UdeM00000033884 XP_022341974.1 49.8 281 120 6 14 293 198 458 5.00E-71 276.2 6565 cellular

organisms;Eukaryota;Opisthokonta;Metazoa;Eumetazoa;Bilateria;Protostomia;
Spiralia;Lophotrochozoa;Mollusca;Bivalvia;Autobranchia;Pteriomorphia;Ostr
eida;Ostreoidea;Ostreidae;Crassostrea;Crassostrea virginica
UdeM00000033885 6565 275.4 UdeM00000033885 XP_022341974.1 49.8 281
120 6 14 293 198 458 8.50E-71 275.4 6565 cellular
organisms;Eukaryota;Opisthokonta;Metazoa;Eumetazoa;Bilateria;Protostomia;
Spiralia;Lophotrochozoa;Mollusca;Bivalvia;Autobranchia;Pteriomorphia;Ostr
eida;Ostreoidea;Ostreidae;Crassostrea;Crassostrea virginica
UdeM00000033886 6596 263.1 UdeM00000033886 XP_045175116.1 34.9 373
227 3 18 376 25 395 5.50E-67 263.1 6596 cellular
organisms;Eukaryota;Opisthokonta;Metazoa;Eumetazoa;Bilateria;Protostomia;
Spiralia;Lophotrochozoa;Mollusca;Bivalvia;Autobranchia;Heteroconchia;Euhe
terodonta;Imparidentia;Venerida;Veneroidea;Veneridae;Mercenaria;Mercenari
a mercenaria
UdeM00000033887 6596 308.9 UdeM00000033887 XP_045212714.1 30.5 696
452 11 2 670 3 693 1.60E-80 308.9 6596 cellular
organisms;Eukaryota;Opisthokonta;Metazoa;Eumetazoa;Bilateria;Protostomia;
Spiralia;Lophotrochozoa;Mollusca;Bivalvia;Autobranchia;Heteroconchia;Euhe
terodonta;Imparidentia;Venerida;Veneroidea;Veneridae;Mercenaria;Mercenari
a mercenaria
UdeM00000033888 6105 82.8 UdeM00000033888 XP_031557194.1 44.6 83
46 0 1 83 299 381 2.80E-13 82.8 6105 cellular
organisms;Eukaryota;Opisthokonta;Metazoa;Eumetazoa;Cnidaria;Anthozoa;Hexa
corallia;Actiniaria;Actiniidae;Actinia;Actinia tenebrosa
UdeM00000033889 6596 69.7 UdeM00000033889 XP_045202967.1 46.3 67
34 1 3 67 9 75 2.00E-09 69.7 6596 cellular
organisms;Eukaryota;Opisthokonta;Metazoa;Eumetazoa;Bilateria;Protostomia;
Spiralia;Lophotrochozoa;Mollusca;Bivalvia;Autobranchia;Heteroconchia;Euhe
terodonta;Imparidentia;Venerida;Veneroidea;Veneridae;Mercenaria;Mercenari
a mercenaria
UdeM00000033890 6596 310.5 UdeM00000033890 XP_045212714.1 31.8 695
444 11 4 674 5 693 5.50E-81 310.5 6596 cellular
organisms;Eukaryota;Opisthokonta;Metazoa;Eumetazoa;Bilateria;Protostomia;
Spiralia;Lophotrochozoa;Mollusca;Bivalvia;Autobranchia;Heteroconchia;Euhe
terodonta;Imparidentia;Venerida;Veneroidea;Veneridae;Mercenaria;Mercenari
a mercenaria
UdeM00000033891 6596 209.9 UdeM00000033891 XP_045201862.1 31.1 472
280 11 2 442 222 679 7.10E-51 209.9 6596 cellular
organisms;Eukaryota;Opisthokonta;Metazoa;Eumetazoa;Bilateria;Protostomia;
Spiralia;Lophotrochozoa;Mollusca;Bivalvia;Autobranchia;Heteroconchia;Euhe
terodonta;Imparidentia;Venerida;Veneroidea;Veneridae;Mercenaria;Mercenari
a mercenaria
UdeM00000033892 6596 75.9 UdeM00000033892 XP_045160626.1 34 150
97 2 2 150 17 165 5.60E-11 75.9 6596 cellular
organisms;Eukaryota;Opisthokonta;Metazoa;Eumetazoa;Bilateria;Protostomia;
Spiralia;Lophotrochozoa;Mollusca;Bivalvia;Autobranchia;Heteroconchia;Euhe
terodonta;Imparidentia;Venerida;Veneroidea;Veneridae;Mercenaria;Mercenari
a mercenaria
UdeM00000033893 6105 104.4 UdeM00000033893 XP_031557194.1 45.6 114
59 2 1 114 299 409 1.00E-19 104.4 6105 cellular
organisms;Eukaryota;Opisthokonta;Metazoa;Eumetazoa;Cnidaria;Anthozoa;Hexa
corallia;Actiniaria;Actiniidae;Actinia;Actinia tenebrosa
UdeM00000033894 6105 148.3 UdeM00000033894 XP_031557194.1 38.4 245
128 6 7 248 4 228 1.30E-32 148.3 6105 cellular

organisms;Eukaryota;Opisthokonta;Metazoa;Eumetazoa;Cnidaria;Anthozoa;Hexacorallia;Actiniaria;Actiniidae;Actinia;Actinia tenebrosa
UdeM00000033895 6573 359 UdeM00000033895 XP_021358036.1 79.6 206 42 0 1 206 1 206 4.10E-96 359 6573 cellular organisms;Eukaryota;Opisthokonta;Metazoa;Eumetazoa;Bilateria;Protostomia;Spiralia;Lophotrochozoa;Mollusca;Bivalvia;Autobranchia;Pteriomorphia;Pectinida;Pectinoidea;Pectinidae;Mizuhopecten;Mizuhopecten yessoensis
UdeM00000033896 1735272 353.6 UdeM00000033896 XP_041376080.1 57.5 360 103 5 2 361 3 312 3.00E-94 353.6 1735272 cellular organisms;Eukaryota;Opisthokonta;Metazoa;Eumetazoa;Bilateria;Protostomia;Spiralia;Lophotrochozoa;Mollusca;Gastropoda;Neomphalina;Neomphaloidea;Peltospiridae;Gigantopelta;Gigantopelta aegis
UdeM00000033897 6596 498 UdeM00000033897 XP_045192822.1 86.4 273 37 0 1 273 1 273 7.40E-138 498 6596 cellular organisms;Eukaryota;Opisthokonta;Metazoa;Eumetazoa;Bilateria;Protostomia;Spiralia;Lophotrochozoa;Mollusca;Bivalvia;Autobranchia;Heteroconchia;Euheterodonta;Imparidentia;Venerida;Veneroidea;Veneridae;Mercenaria;Mercenaria mercenaria
UdeM00000033898 6596 260.4 UdeM00000033898 XP_045192822.1 82.3 147 26 0 23 169 127 273 1.60E-66 260.4 6596 cellular organisms;Eukaryota;Opisthokonta;Metazoa;Eumetazoa;Bilateria;Protostomia;Spiralia;Lophotrochozoa;Mollusca;Bivalvia;Autobranchia;Heteroconchia;Euheterodonta;Imparidentia;Venerida;Veneroidea;Veneridae;Mercenaria;Mercenaria mercenaria
UdeM00000041513 400727 468.8 UdeM00000041513 XP_025092305.1 53.3 420 186 6 13 427 29 443 7.50E-129 468.8 400727 cellular organisms;Eukaryota;Opisthokonta;Metazoa;Eumetazoa;Bilateria;Protostomia;Spiralia;Lophotrochozoa;Mollusca;Gastropoda;Caenogastropoda;Architaenioglossa;Ampullarioidea;Ampullariidae;Pomacea;Pomacea canaliculata
UdeM00000041515 1735272 80.1 UdeM00000041515 XP_041360874.1 30.1 186 115 6 10 184 16 197 3.20E-12 80.1 1735272 cellular organisms;Eukaryota;Opisthokonta;Metazoa;Eumetazoa;Bilateria;Protostomia;Spiralia;Lophotrochozoa;Mollusca;Gastropoda;Neomphalina;Neomphaloidea;Peltospiridae;Gigantopelta;Gigantopelta aegis
UdeM00000041516 1735272 270.8 UdeM00000041516 XP_041360874.1 39.3 420 240 7 12 429 13 419 3.00E-69 270.8 1735272 cellular organisms;Eukaryota;Opisthokonta;Metazoa;Eumetazoa;Bilateria;Protostomia;Spiralia;Lophotrochozoa;Mollusca;Gastropoda;Neomphalina;Neomphaloidea;Peltospiridae;Gigantopelta;Gigantopelta aegis
UdeM00000041517 2607531 274.6 UdeM00000041517 XP_036368557.1 40.1 387 220 8 35 413 44 426 2.00E-70 274.6 2607531 cellular organisms;Eukaryota;Opisthokonta;Metazoa;Eumetazoa;Bilateria;Protostomia;Spiralia;Lophotrochozoa;Mollusca;Cephalopoda;Coleoidea;Octopodiformes;Octopoda;Incirrata;Octopodidae;Octopus;Octopus sinensis
UdeM00000041518 1735272 288.5 UdeM00000041518 XP_041360874.1 43.9 367 198 5 1 361 55 419 1.20E-74 288.5 1735272 cellular organisms;Eukaryota;Opisthokonta;Metazoa;Eumetazoa;Bilateria;Protostomia;

Spiralia;Lophotrochozoa;Mollusca;Gastropoda;Neomphalina;Neomphaloidea;Peltospiridae;Gigantopelta;Gigantopelta aegis
UdeM00000041519 29159 245.7 UdeM00000041519 XP_019919189.2 31.2 641 329 17 82 712 85 623 1.80E-61 245.7 29159 cellular organisms;Eukaryota;Opisthokonta;Metazoa;Eumetazoa;Bilateria;Protostomia;Spiralia;Lophotrochozoa;Mollusca;Bivalvia;Autobranchia;Pteriomorphia;Ostreida;Ostreoidea;Ostreidae;Crassostrea;Crassostrea gigas
UdeM00000041520 6596 496.9 UdeM00000041520 XP_045182242.1 53.4 470 188 5 9 459 16 473 3.10E-137 496.9 6596 cellular organisms;Eukaryota;Opisthokonta;Metazoa;Eumetazoa;Bilateria;Protostomia;Spiralia;Lophotrochozoa;Mollusca;Bivalvia;Autobranchia;Heteroconchia;Euheterodonta;Imparidentia;Venerida;Veneroidea;Veneridae;Mercenaria;Mercenaria mercenaria
UdeM00000041521 6596 212.2 UdeM00000041521 XP_045181187.1 62.3 183 57 3 13 191 1 175 5.70E-52 212.2 6596 cellular organisms;Eukaryota;Opisthokonta;Metazoa;Eumetazoa;Bilateria;Protostomia;Spiralia;Lophotrochozoa;Mollusca;Bivalvia;Autobranchia;Heteroconchia;Euheterodonta;Imparidentia;Venerida;Veneroidea;Veneridae;Mercenaria;Mercenaria mercenaria
UdeM00000041522 6596 186.4 UdeM00000041522 XP_045181193.1 63.5 159 48 2 8 162 11 163 2.80E-44 186.4 6596 cellular organisms;Eukaryota;Opisthokonta;Metazoa;Eumetazoa;Bilateria;Protostomia;Spiralia;Lophotrochozoa;Mollusca;Bivalvia;Autobranchia;Heteroconchia;Euheterodonta;Imparidentia;Venerida;Veneroidea;Veneridae;Mercenaria;Mercenaria mercenaria
UdeM00000041523 6596 295.8 UdeM00000041523 XP_045180623.1 51.6 285 106 4 8 262 132 414 5.40E-77 295.8 6596 cellular organisms;Eukaryota;Opisthokonta;Metazoa;Eumetazoa;Bilateria;Protostomia;Spiralia;Lophotrochozoa;Mollusca;Bivalvia;Autobranchia;Heteroconchia;Euheterodonta;Imparidentia;Venerida;Veneroidea;Veneridae;Mercenaria;Mercenaria mercenaria
UdeM00000041524 6596 300.4 UdeM00000041524 XP_045180623.1 53.3 285 104 4 8 265 132 414 2.20E-78 300.4 6596 cellular organisms;Eukaryota;Opisthokonta;Metazoa;Eumetazoa;Bilateria;Protostomia;Spiralia;Lophotrochozoa;Mollusca;Bivalvia;Autobranchia;Heteroconchia;Euheterodonta;Imparidentia;Venerida;Veneroidea;Veneridae;Mercenaria;Mercenaria mercenaria
UdeM00000041527 6573 535 UdeM00000041527 XP_021373061.1 77 330 75 1 1 329 507 836 6.60E-149 535 6573 cellular organisms;Eukaryota;Opisthokonta;Metazoa;Eumetazoa;Bilateria;Protostomia;Spiralia;Lophotrochozoa;Mollusca;Bivalvia;Autobranchia;Pteriomorphia;Pectinida;Pectinoidea;Pectinidae;Mizuhopecten;Mizuhopecten yessoensis
UdeM00000041526 6573 657.9 UdeM00000041526 XP_021373065.1 76.5 413 96 1 19 430 404 816 8.80E-186 657.9 6573 cellular organisms;Eukaryota;Opisthokonta;Metazoa;Eumetazoa;Bilateria;Protostomia;Spiralia;Lophotrochozoa;Mollusca;Bivalvia;Autobranchia;Pteriomorphia;Pectinida;Pectinoidea;Pectinidae;Mizuhopecten;Mizuhopecten yessoensis
UdeM00000041528 29159 213.8 UdeM00000041528 XP_034317505.1 84 125 20 0 265 389 288 412 4.00E-52 213.8 29159 cellular organisms;Eukaryota;Opisthokonta;Metazoa;Eumetazoa;Bilateria;Protostomia;Spiralia;Lophotrochozoa;Mollusca;Bivalvia;Autobranchia;Pteriomorphia;Ostreida;Ostreoidea;Ostreidae;Crassostrea;Crassostrea gigas
UdeM00000041529 6573 445.7 UdeM00000041529 XP_021373061.1 83.3 257 43 0 20 276 21 277 4.50E-122 445.7 6573 cellular

organisms;Eukaryota;Opisthokonta;Metazoa;Eumetazoa;Bilateria;Protostomia;Spiralia;Lophotrochozoa;Mollusca;Bivalvia;Autobranchia;Pteriomorphia;Pectinida;Pectinoidea;Pectinidae;Mizuhopecten;Mizuhopecten yessoensis
UdeM00000041530 400727 330.1 UdeM00000041530 XP_025091729.1 87.8 197 24 0 1 197 1 197 1.90E-87 330.1 400727 cellular organisms;Eukaryota;Opisthokonta;Metazoa;Eumetazoa;Bilateria;Protostomia;Spiralia;Lophotrochozoa;Mollusca;Gastropoda;Caenogastropoda;Architaenioglossa;Ampullarioidea;Ampullariidae;Pomacea;Pomacea canaliculata
UdeM00000041531 6596 313.5 UdeM00000041531 XP_045166991.1 41.6 411 161 5 57 423 265 640 8.70E-82 313.5 6596 cellular organisms;Eukaryota;Opisthokonta;Metazoa;Eumetazoa;Bilateria;Protostomia;Spiralia;Lophotrochozoa;Mollusca;Bivalvia;Autobranchia;Heteroconchia;Euheterodonta;Imparidentia;Venerida;Veneroidea;Veneridae;Mercenaria;Mercenaria mercenaria
UdeM00000041532 29159 242.7 UdeM00000041532 XP_019919189.2 29.7 663 342 12 98 760 85 623 1.60E-60 242.7 29159 cellular organisms;Eukaryota;Opisthokonta;Metazoa;Eumetazoa;Bilateria;Protostomia;Spiralia;Lophotrochozoa;Mollusca;Bivalvia;Autobranchia;Pteriomorphia;Ostreida;Ostreoidea;Ostreidae;Crassostrea;Crassostrea gigas
UdeM00000041533 6596 725.7 UdeM00000041533 XP_045180675.1 50.3 748 341 8 5 734 301 1035 5.90E-206 725.7 6596 cellular organisms;Eukaryota;Opisthokonta;Metazoa;Eumetazoa;Bilateria;Protostomia;Spiralia;Lophotrochozoa;Mollusca;Bivalvia;Autobranchia;Heteroconchia;Euheterodonta;Imparidentia;Venerida;Veneroidea;Veneridae;Mercenaria;Mercenaria mercenaria
UdeM00000041534 1735272 113.6 UdeM00000041534 XP_041361447.1 41.7 163 76 5 82 244 116 259 3.70E-22 113.6 1735272 cellular organisms;Eukaryota;Opisthokonta;Metazoa;Eumetazoa;Bilateria;Protostomia;Spiralia;Lophotrochozoa;Mollusca;Gastropoda;Neomphalina;Neomphaloidea;Peltospiridae;Gigantopelta;Gigantopelta aegis
UdeM00000041535 6579 451.4 UdeM00000041535 XP_033727368.1 50.6 462 158 6 1 393 1 461 1.10E-123 451.4 6579 cellular organisms;Eukaryota;Opisthokonta;Metazoa;Eumetazoa;Bilateria;Protostomia;Spiralia;Lophotrochozoa;Mollusca;Bivalvia;Autobranchia;Pteriomorphia;Pectinida;Pectinoidea;Pectinidae;Pecten;Pecten maximus
UdeM00000041536 45351 92.4 UdeM00000041536 XP_032236999.1 35.8 151 81 2 37 172 11 160 6.20E-16 92.4 45351 cellular organisms;Eukaryota;Opisthokonta;Metazoa;Eumetazoa;Cnidaria;Anthozoa;Hexacorallia;Actiniaria;Edwardsiidae;Nematostella;Nematostella vectensis
UdeM00000041537 6596 290.4 UdeM00000041537 XP_045177776.1 40.6 433 235 8 1 427 405 821 3.70E-75 290.4 6596 cellular organisms;Eukaryota;Opisthokonta;Metazoa;Eumetazoa;Bilateria;Protostomia;Spiralia;Lophotrochozoa;Mollusca;Bivalvia;Autobranchia;Heteroconchia;Euheterodonta;Imparidentia;Venerida;Veneroidea;Veneridae;Mercenaria;Mercenaria mercenaria
UdeM00000041538 6596 108.6 UdeM00000041538 XP_045209482.1 41.7 168 86 2 149 316 7 162 1.60E-20 108.6 6596 cellular organisms;Eukaryota;Opisthokonta;Metazoa;Eumetazoa;Bilateria;Protostomia;Spiralia;Lophotrochozoa;Mollusca;Bivalvia;Autobranchia;Heteroconchia;Euheterodonta;Imparidentia;Venerida;Veneroidea;Veneridae;Mercenaria;Mercenaria mercenaria

UdeM00000041539 6596 270.8 UdeM00000041539 XP_045195540.1 49.2 256
129 1 5 260 46 300 2.10E-69 270.8 6596 cellular
organisms;Eukaryota;Opisthokonta;Metazoa;Eumetazoa;Bilateria;Protostomia;
Spiralia;Lophotrochozoa;Mollusca;Bivalvia;Autobranchia;Heteroconchia;Euhe
terodonta;Imparidentia;Venerida;Veneroidea;Veneridae;Mercenaria;Mercenari
a mercenaria
UdeM00000041540 1735272 115.5 UdeM00000041540 XP_041374221.1 34.6
214 124 1 25 238 25 222 1.60E-22 115.5 1735272
cellular
organisms;Eukaryota;Opisthokonta;Metazoa;Eumetazoa;Bilateria;Protostomia;
Spiralia;Lophotrochozoa;Mollusca;Gastropoda;Neomphalina;Neomphaloidea;Pel
tospiridae;Gigantopelta;Gigantopelta aegis
UdeM00000041541 6596 390.6 UdeM00000041541 XP_045177776.1 41.4 553
304 10 1 539 405 951 4.80E-105 390.6 6596 cellular
organisms;Eukaryota;Opisthokonta;Metazoa;Eumetazoa;Bilateria;Protostomia;
Spiralia;Lophotrochozoa;Mollusca;Bivalvia;Autobranchia;Heteroconchia;Euhe
terodonta;Imparidentia;Venerida;Veneroidea;Veneridae;Mercenaria;Mercenari
a mercenaria
UdeM00000041542 6596 295.4 UdeM00000041542 XP_045177779.1 41 432
223 8 1 406 97 522 1.10E-76 295.4 6596 cellular
organisms;Eukaryota;Opisthokonta;Metazoa;Eumetazoa;Bilateria;Protostomia;
Spiralia;Lophotrochozoa;Mollusca;Bivalvia;Autobranchia;Heteroconchia;Euhe
terodonta;Imparidentia;Venerida;Veneroidea;Veneridae;Mercenaria;Mercenari
a mercenaria
UdeM00000041543 6579 559.7 UdeM00000041543 XP_033761495.1 77.1 336
77 0 36 371 24 359 3.70E-156 559.7 6579 cellular
organisms;Eukaryota;Opisthokonta;Metazoa;Eumetazoa;Bilateria;Protostomia;
Spiralia;Lophotrochozoa;Mollusca;Bivalvia;Autobranchia;Pteriomorphia;Pect
inida;Pectinoidea;Pectinidae;Pecten;Pecten maximus
UdeM00000041544 6596 111.3 UdeM00000041544 XP_045188177.1 53.5 99
40 1 1 99 65 157 7.30E-22 111.3 6596 cellular
organisms;Eukaryota;Opisthokonta;Metazoa;Eumetazoa;Bilateria;Protostomia;
Spiralia;Lophotrochozoa;Mollusca;Bivalvia;Autobranchia;Heteroconchia;Euhe
terodonta;Imparidentia;Venerida;Veneroidea;Veneridae;Mercenaria;Mercenari
a mercenaria
UdeM00000041545 6596 98.6 UdeM00000041545 XP_045162187.1 46.6 103
55 0 26 128 26 128 9.80E-18 98.6 6596 cellular
organisms;Eukaryota;Opisthokonta;Metazoa;Eumetazoa;Bilateria;Protostomia;
Spiralia;Lophotrochozoa;Mollusca;Bivalvia;Autobranchia;Heteroconchia;Euhe
terodonta;Imparidentia;Venerida;Veneroidea;Veneridae;Mercenaria;Mercenari
a mercenaria
UdeM00000041552 6596 172.6 UdeM00000041552 XP_045200961.1 35.5 279
158 6 2 271 3 268 7.10E-40 172.6 6596 cellular
organisms;Eukaryota;Opisthokonta;Metazoa;Eumetazoa;Bilateria;Protostomia;
Spiralia;Lophotrochozoa;Mollusca;Bivalvia;Autobranchia;Heteroconchia;Euhe
terodonta;Imparidentia;Venerida;Veneroidea;Veneridae;Mercenaria;Mercenari
a mercenaria
UdeM00000041553 6596 161 UdeM00000041553 XP_045200961.1 35.3 252
141 6 1 243 30 268 1.90E-36 161 6596 cellular
organisms;Eukaryota;Opisthokonta;Metazoa;Eumetazoa;Bilateria;Protostomia;
Spiralia;Lophotrochozoa;Mollusca;Bivalvia;Autobranchia;Heteroconchia;Euhe
terodonta;Imparidentia;Venerida;Veneroidea;Veneridae;Mercenaria;Mercenari
a mercenaria

UdeM00000041554	29159	91.3	UdeM00000041554	XP_011453058.2	26.5	238
157	9	26	248	76	310	1.90E-15	91.3	29159	cellular organisms;Eukaryota;Opisthokonta;Metazoa;Eumetazoa;Bilateria;Protostomia;Spiralia;Lophotrochozoa;Mollusca;Bivalvia;Autobranchia;Pteriomorphia;Ostreida;Ostreoidea;Ostreidae;Crassostrea;Crassostrea gigas
UdeM00000041555	1735272	64.3	UdeM00000041555	XP_041375509.1	50
62	31	0	33	94	931	992	1.20E-07	64.3	1735272
cellular
organisms;Eukaryota;Opisthokonta;Metazoa;Eumetazoa;Bilateria;Protostomia;Spiralia;Lophotrochozoa;Mollusca;Gastropoda;Neomphalina;Neomphaloidea;Peltospiridae;Gigantopelta;Gigantopelta aegis
UdeM00000041557	6596	367.5	UdeM00000041557	XP_045175691.1	47.8	389
199	4	3	387	17	405	2.20E-98	367.5	6596	cellular organisms;Eukaryota;Opisthokonta;Metazoa;Eumetazoa;Bilateria;Protostomia;Spiralia;Lophotrochozoa;Mollusca;Bivalvia;Autobranchia;Heteroconchia;Euheterodonta;Imparidentia;Venerida;Veneroidea;Veneridae;Mercenaria;Mercenaria mercenaria
UdeM00000041558	6565	427.6	UdeM00000041558	XP_022291405.1	65.9	299
102	0	14	312	12	310	1.40E-116	427.6	6565	cellular organisms;Eukaryota;Opisthokonta;Metazoa;Eumetazoa;Bilateria;Protostomia;Spiralia;Lophotrochozoa;Mollusca;Bivalvia;Autobranchia;Pteriomorphia;Ostreida;Ostreoidea;Ostreidae;Crassostrea;Crassostrea virginica
UdeM00000041559	29159	248.8	UdeM00000041559	XP_011449844.1	23.3	716
490	14	3	695	6	685	2.00E-62	248.8	29159	cellular organisms;Eukaryota;Opisthokonta;Metazoa;Eumetazoa;Bilateria;Protostomia;Spiralia;Lophotrochozoa;Mollusca;Bivalvia;Autobranchia;Pteriomorphia;Ostreida;Ostreoidea;Ostreidae;Crassostrea;Crassostrea gigas
UdeM00000041560	6596	199.1	UdeM00000041560	XP_045169960.1	21.8	705
434	16	8	696	7	610	1.80E-47	199.1	6596	cellular organisms;Eukaryota;Opisthokonta;Metazoa;Eumetazoa;Bilateria;Protostomia;Spiralia;Lophotrochozoa;Mollusca;Bivalvia;Autobranchia;Heteroconchia;Euheterodonta;Imparidentia;Venerida;Veneroidea;Veneridae;Mercenaria;Mercenaria mercenaria
UdeM00000041561	6596	172.9	UdeM00000041561	XP_045181403.1	58.2	158
61	2	1	154	765	921	3.10E-40	172.9	6596	cellular organisms;Eukaryota;Opisthokonta;Metazoa;Eumetazoa;Bilateria;Protostomia;Spiralia;Lophotrochozoa;Mollusca;Bivalvia;Autobranchia;Heteroconchia;Euheterodonta;Imparidentia;Venerida;Veneroidea;Veneridae;Mercenaria;Mercenaria mercenaria
UdeM00000041562	6596	407.9	UdeM00000041562	XP_045181407.1	50.6	468
177	14	3	446	1	438	1.70E-110	407.9	6596	cellular organisms;Eukaryota;Opisthokonta;Metazoa;Eumetazoa;Bilateria;Protostomia;Spiralia;Lophotrochozoa;Mollusca;Bivalvia;Autobranchia;Heteroconchia;Euheterodonta;Imparidentia;Venerida;Veneroidea;Veneridae;Mercenaria;Mercenaria mercenaria
UdeM00000041564	6596	429.9	UdeM00000041564	XP_045181968.1	58.8	328
134	1	1	327	1	328	3.00E-117	429.9	6596	cellular organisms;Eukaryota;Opisthokonta;Metazoa;Eumetazoa;Bilateria;Protostomia;Spiralia;Lophotrochozoa;Mollusca;Bivalvia;Autobranchia;Heteroconchia;Euheterodonta;Imparidentia;Venerida;Veneroidea;Veneridae;Mercenaria;Mercenaria mercenaria
UdeM00000041565	1735272	205.3	UdeM00000041565	XP_041360720.1	37.8
378	209	9	1076	1444	1387	1747	5.50E-49	205.3	1735272
cellular

organisms;Eukaryota;Opisthokonta;Metazoa;Eumetazoa;Bilateria;Protostomia;Spiralia;Lophotrochozoa;Mollusca;Gastropoda;Neomphalina;Neomphaloidea;Peltospiridae;Gigantopelta;Gigantopelta aegis
UdeM00000041565 1735272 132.5 UdeM00000041565 XP_041360720.1 39.4 198 109 3 1 198 1 187 4.50E-27 132.5 1735272 cellular organisms;Eukaryota;Opisthokonta;Metazoa;Eumetazoa;Bilateria;Protostomia;Spiralia;Lophotrochozoa;Mollusca;Gastropoda;Neomphalina;Neomphaloidea;Peltospiridae;Gigantopelta;Gigantopelta aegis
UdeM00000041569 6596 424.1 UdeM00000041569 XP_045181968.1 57.9 328 137 1 1 327 1 328 1.60E-115 424.1 6596 cellular organisms;Eukaryota;Opisthokonta;Metazoa;Eumetazoa;Bilateria;Protostomia;Spiralia;Lophotrochozoa;Mollusca;Bivalvia;Autobranchia;Heteroconchia;Euheterodonta;Imparidentia;Venerida;Veneroidea;Veneridae;Mercenaria;Mercenaria mercenaria
UdeM00000041570 6596 275.4 UdeM00000041570 XP_045181968.1 54.8 228 102 1 1 227 1 228 7.00E-71 275.4 6596 cellular organisms;Eukaryota;Opisthokonta;Metazoa;Eumetazoa;Bilateria;Protostomia;Spiralia;Lophotrochozoa;Mollusca;Bivalvia;Autobranchia;Heteroconchia;Euheterodonta;Imparidentia;Venerida;Veneroidea;Veneridae;Mercenaria;Mercenaria mercenaria
UdeM00000041571 6596 417.9 UdeM00000041571 XP_045206084.1 30.5 931 593 21 155 1057 79 983 5.90E-113 417.9 6596 cellular organisms;Eukaryota;Opisthokonta;Metazoa;Eumetazoa;Bilateria;Protostomia;Spiralia;Lophotrochozoa;Mollusca;Bivalvia;Autobranchia;Heteroconchia;Euheterodonta;Imparidentia;Venerida;Veneroidea;Veneridae;Mercenaria;Mercenaria mercenaria
UdeM00000041572 6596 403.7 UdeM00000041572 XP_045206084.1 30.2 933 593 21 131 1033 79 983 8.50E-109 403.7 6596 cellular organisms;Eukaryota;Opisthokonta;Metazoa;Eumetazoa;Bilateria;Protostomia;Spiralia;Lophotrochozoa;Mollusca;Bivalvia;Autobranchia;Heteroconchia;Euheterodonta;Imparidentia;Venerida;Veneroidea;Veneridae;Mercenaria;Mercenaria mercenaria
UdeM00000041574 6596 186.4 UdeM00000041574 XP_045206055.1 33.7 365 192 11 382 699 79 440 1.20E-43 186.4 6596 cellular organisms;Eukaryota;Opisthokonta;Metazoa;Eumetazoa;Bilateria;Protostomia;Spiralia;Lophotrochozoa;Mollusca;Bivalvia;Autobranchia;Heteroconchia;Euheterodonta;Imparidentia;Venerida;Veneroidea;Veneridae;Mercenaria;Mercenaria mercenaria
UdeM00000041575 6596 112.5 UdeM00000041575 XP_045206084.1 32 222 139 8 39 250 221 440 8.40E-22 112.5 6596 cellular organisms;Eukaryota;Opisthokonta;Metazoa;Eumetazoa;Bilateria;Protostomia;Spiralia;Lophotrochozoa;Mollusca;Bivalvia;Autobranchia;Heteroconchia;Euheterodonta;Imparidentia;Venerida;Veneroidea;Veneridae;Mercenaria;Mercenaria mercenaria
UdeM00000041576 118200 60.1 UdeM00000041576 XP_009906001.1 26.4 129 93 2 34 162 1954 2080 3.50E-06 60.1 118200 cellular organisms;Eukaryota;Opisthokonta;Metazoa;Eumetazoa;Bilateria;Deuterostomia;Chordata;Craniata;Vertebrata;Gnathostomata;Teleostomi;Euteleostomi;Sarcopterygii;Dipnotetrapodomorpha;Tetrapoda;Amniota;Sauropsida;Sauria;Archelosauria;Archosauria;Dinosauria;Saurischia;Theropoda;Coelurosauria;Aves;Neognathae;Piciformes;Picidae;Dryobates;Dryobates pubescens

UdeM00000041577 6565 86.3 UdeM00000041577 XP_022324486.1 36.1 133
74 6 6 131 319 447 3.30E-14 86.3 6565 cellular
organisms;Eukaryota;Opisthokonta;Metazoa;Eumetazoa;Bilateria;Protostomia;
Spiralia;Lophotrochozoa;Mollusca;Bivalvia;Autobranchia;Pteriomorphia;Ostr
eida;Ostreoidea;Ostreidae;Crassostrea;Crassostrea virginica
UdeM00000041578 6596 412.9 UdeM00000041578 XP_045206084.1 30.8 933
588 21 131 1033 79 983 1.90E-111 412.9 6596 cellular
organisms;Eukaryota;Opisthokonta;Metazoa;Eumetazoa;Bilateria;Protostomia;
Spiralia;Lophotrochozoa;Mollusca;Bivalvia;Autobranchia;Heteroconchia;Euhe
terodonta;Imparidentia;Venerida;Veneroidea;Veneridae;Mercenaria;Mercenari
a mercenaria
UdeM00000041579 6565 167.9 UdeM00000041579 XP_022326775.1 26.7 430
289 13 2 414 202 622 2.70E-38 167.9 6565 cellular
organisms;Eukaryota;Opisthokonta;Metazoa;Eumetazoa;Bilateria;Protostomia;
Spiralia;Lophotrochozoa;Mollusca;Bivalvia;Autobranchia;Pteriomorphia;Ostr
eida;Ostreoidea;Ostreidae;Crassostrea;Crassostrea virginica
UdeM00000041582 6596 265.4 UdeM00000041582 XP_045206055.1 32.8 518
317 15 383 879 79 586 2.70E-67 265.4 6596 cellular
organisms;Eukaryota;Opisthokonta;Metazoa;Eumetazoa;Bilateria;Protostomia;
Spiralia;Lophotrochozoa;Mollusca;Bivalvia;Autobranchia;Heteroconchia;Euhe
terodonta;Imparidentia;Venerida;Veneroidea;Veneridae;Mercenaria;Mercenari
a mercenaria
UdeM00000041583 29159 219.9 UdeM00000041583 XP_019927830.2 27.9 559
341 15 377 881 74 624 1.30E-53 219.9 29159 cellular
organisms;Eukaryota;Opisthokonta;Metazoa;Eumetazoa;Bilateria;Protostomia;
Spiralia;Lophotrochozoa;Mollusca;Bivalvia;Autobranchia;Pteriomorphia;Ostr
eida;Ostreoidea;Ostreidae;Crassostrea;Crassostrea gigas
UdeM00000041584 6596 413.3 UdeM00000041584 XP_045206084.1 30.8 933
588 21 43 945 79 983 1.20E-111 413.3 6596 cellular
organisms;Eukaryota;Opisthokonta;Metazoa;Eumetazoa;Bilateria;Protostomia;
Spiralia;Lophotrochozoa;Mollusca;Bivalvia;Autobranchia;Heteroconchia;Euhe
terodonta;Imparidentia;Venerida;Veneroidea;Veneridae;Mercenaria;Mercenari
a mercenaria
UdeM00000041589 6596 371.7 UdeM00000041589 XP_045206065.1 29.1 925
552 21 43 877 79 989 3.90E-99 371.7 6596 cellular
organisms;Eukaryota;Opisthokonta;Metazoa;Eumetazoa;Bilateria;Protostomia;
Spiralia;Lophotrochozoa;Mollusca;Bivalvia;Autobranchia;Heteroconchia;Euhe
terodonta;Imparidentia;Venerida;Veneroidea;Veneridae;Mercenaria;Mercenari
a mercenaria
UdeM00000041588 6596 413.3 UdeM00000041588 XP_045206084.1 30.8 933
588 21 133 1035 79 983 1.30E-111 413.3 6596 cellular
organisms;Eukaryota;Opisthokonta;Metazoa;Eumetazoa;Bilateria;Protostomia;
Spiralia;Lophotrochozoa;Mollusca;Bivalvia;Autobranchia;Heteroconchia;Euhe
terodonta;Imparidentia;Venerida;Veneroidea;Veneridae;Mercenaria;Mercenari
a mercenaria
UdeM00000041590 6596 413.7 UdeM00000041590 XP_045206084.1 30.8 933
588 21 131 1033 79 983 8.00E-112 413.7 6596 cellular
organisms;Eukaryota;Opisthokonta;Metazoa;Eumetazoa;Bilateria;Protostomia;
Spiralia;Lophotrochozoa;Mollusca;Bivalvia;Autobranchia;Heteroconchia;Euhe
terodonta;Imparidentia;Venerida;Veneroidea;Veneridae;Mercenaria;Mercenari
a mercenaria
UdeM00000041591 6596 123.2 UdeM00000041591 XP_045206055.1 25.8 396
233 13 2 342 197 586 7.00E-25 123.2 6596 cellular
organisms;Eukaryota;Opisthokonta;Metazoa;Eumetazoa;Bilateria;Protostomia;

Spiralia;Lophotrochozoa;Mollusca;Bivalvia;Autobranchia;Heteroconchia;Euheterodonta;Imparidentia;Venerida;Veneroidea;Veneridae;Mercenaria;Mercenaria mercenaria
UdeM00000041592 6596 413.7 UdeM00000041592 XP_045206084.1 30.8 933 588 21 133 1035 79 983 1.00E-111 413.7 6596 cellular organisms;Eukaryota;Opisthokonta;Metazoa;Eumetazoa;Bilateria;Protostomia;Spiralia;Lophotrochozoa;Mollusca;Bivalvia;Autobranchia;Heteroconchia;Euheterodonta;Imparidentia;Venerida;Veneroidea;Veneridae;Mercenaria;Mercenaria mercenaria
UdeM00000043816 6596 209.5 UdeM00000043816 XP_045177053.1 33.4 338 209 7 61 390 92 421 7.90E-51 209.5 6596 cellular organisms;Eukaryota;Opisthokonta;Metazoa;Eumetazoa;Bilateria;Protostomia;Spiralia;Lophotrochozoa;Mollusca;Bivalvia;Autobranchia;Heteroconchia;Euheterodonta;Imparidentia;Venerida;Veneroidea;Veneridae;Mercenaria;Mercenaria mercenaria
UdeM00000043818 6596 229.2 UdeM00000043818 XP_045176664.1 31.4 506 293 13 44 518 32 514 1.30E-56 229.2 6596 cellular organisms;Eukaryota;Opisthokonta;Metazoa;Eumetazoa;Bilateria;Protostomia;Spiralia;Lophotrochozoa;Mollusca;Bivalvia;Autobranchia;Heteroconchia;Euheterodonta;Imparidentia;Venerida;Veneroidea;Veneridae;Mercenaria;Mercenaria mercenaria
UdeM00000043819 6596 127.1 UdeM00000043819 XP_045176094.1 43.4 152 81 3 212 359 83 233 4.60E-26 127.1 6596 cellular organisms;Eukaryota;Opisthokonta;Metazoa;Eumetazoa;Bilateria;Protostomia;Spiralia;Lophotrochozoa;Mollusca;Bivalvia;Autobranchia;Heteroconchia;Euheterodonta;Imparidentia;Venerida;Veneroidea;Veneridae;Mercenaria;Mercenaria mercenaria
UdeM00000043820 6596 77 UdeM00000043820 XP_045176664.1 25.4 256 177 6 47 291 35 287 4.40E-11 77 6596 cellular organisms;Eukaryota;Opisthokonta;Metazoa;Eumetazoa;Bilateria;Protostomia;Spiralia;Lophotrochozoa;Mollusca;Bivalvia;Autobranchia;Heteroconchia;Euheterodonta;Imparidentia;Venerida;Veneroidea;Veneridae;Mercenaria;Mercenaria mercenaria
UdeM00000043821 6596 153.7 UdeM00000043821 XP_045176094.1 43.6 188 98 4 17 200 83 266 2.50E-34 153.7 6596 cellular organisms;Eukaryota;Opisthokonta;Metazoa;Eumetazoa;Bilateria;Protostomia;Spiralia;Lophotrochozoa;Mollusca;Bivalvia;Autobranchia;Heteroconchia;Euheterodonta;Imparidentia;Venerida;Veneroidea;Veneridae;Mercenaria;Mercenaria mercenaria
UdeM00000043826 10228 191.8 UdeM00000043826 XP_002118406.1 30.9 433 266 11 12 428 172 587 2.00E-45 191.8 10228 cellular organisms;Eukaryota;Opisthokonta;Metazoa;Eumetazoa;Placozoa;Trichoplacidae;Trichoplax;Trichoplax adhaerens
UdeM00000040979 6596 202.2 UdeM00000040979 XP_045213493.1 51.1 182 85 2 2 180 137 317 5.60E-49 202.2 6596 cellular organisms;Eukaryota;Opisthokonta;Metazoa;Eumetazoa;Bilateria;Protostomia;Spiralia;Lophotrochozoa;Mollusca;Bivalvia;Autobranchia;Heteroconchia;Euheterodonta;Imparidentia;Venerida;Veneroidea;Veneridae;Mercenaria;Mercenaria mercenaria
UdeM00000009145 6596 668.7 UdeM00000009145 XP_045162867.1 50.8 711 271 9 18 660 363 1062 7.60E-189 668.7 6596 cellular organisms;Eukaryota;Opisthokonta;Metazoa;Eumetazoa;Bilateria;Protostomia;Spiralia;Lophotrochozoa;Mollusca;Bivalvia;Autobranchia;Heteroconchia;Euhe

terodonta;Imparidentia;Venerida;Veneroidea;Veneridae;Mercenaria;Mercenaria mercenaria
UdeM00000009146 6596 251.5 UdeM00000009146 XP_045207443.1 69 171 53 0 1 171 880 1050 7.60E-64 251.5 6596 cellular organisms;Eukaryota;Opisthokonta;Metazoa;Eumetazoa;Bilateria;Protostomia;Spiralia;Lophotrochozoa;Mollusca;Bivalvia;Autobranchia;Heteroconchia;Euheterodonta;Imparidentia;Venerida;Veneroidea;Veneridae;Mercenaria;Mercenaria mercenaria
UdeM00000009147 6596 926.8 UdeM00000009147 XP_045207446.1 52.1 887 410 9 6 888 7 882 2.10E-266 926.8 6596 cellular organisms;Eukaryota;Opisthokonta;Metazoa;Eumetazoa;Bilateria;Protostomia;Spiralia;Lophotrochozoa;Mollusca;Bivalvia;Autobranchia;Heteroconchia;Euheterodonta;Imparidentia;Venerida;Veneroidea;Veneridae;Mercenaria;Mercenaria mercenaria
UdeM00000009149 6596 567 UdeM00000009149 XP_045161482.1 70.7 423 91 6 1 421 380 771 2.00E-158 567 6596 cellular organisms;Eukaryota;Opisthokonta;Metazoa;Eumetazoa;Bilateria;Protostomia;Spiralia;Lophotrochozoa;Mollusca;Bivalvia;Autobranchia;Heteroconchia;Euheterodonta;Imparidentia;Venerida;Veneroidea;Veneridae;Mercenaria;Mercenaria mercenaria
UdeM00000009150 6596 577.8 UdeM00000009150 XP_045161482.1 71.1 426 89 6 1 426 380 771 1.10E-161 577.8 6596 cellular organisms;Eukaryota;Opisthokonta;Metazoa;Eumetazoa;Bilateria;Protostomia;Spiralia;Lophotrochozoa;Mollusca;Bivalvia;Autobranchia;Heteroconchia;Euheterodonta;Imparidentia;Venerida;Veneroidea;Veneridae;Mercenaria;Mercenaria mercenaria
UdeM00000009151 6596 484.2 UdeM00000009151 XP_045161482.1 67.6 373 120 1 1 372 1 373 1.50E-133 484.2 6596 cellular organisms;Eukaryota;Opisthokonta;Metazoa;Eumetazoa;Bilateria;Protostomia;Spiralia;Lophotrochozoa;Mollusca;Bivalvia;Autobranchia;Heteroconchia;Euheterodonta;Imparidentia;Venerida;Veneroidea;Veneridae;Mercenaria;Mercenaria mercenaria
UdeM00000009152 6579 411.4 UdeM00000009152 XP_033724832.1 86.9 213 28 0 5 217 3 215 7.30E-112 411.4 6579 cellular organisms;Eukaryota;Opisthokonta;Metazoa;Eumetazoa;Bilateria;Protostomia;Spiralia;Lophotrochozoa;Mollusca;Bivalvia;Autobranchia;Pteriomorphia;Pectinida;Pectinoidea;Pectinidae;Pecten;Pecten maximus
UdeM00000009153 29159 85.5 UdeM00000009153 XP_034317111.1 31.1 164 107 3 28 188 445 605 1.30E-13 85.5 29159 cellular organisms;Eukaryota;Opisthokonta;Metazoa;Eumetazoa;Bilateria;Protostomia;Spiralia;Lophotrochozoa;Mollusca;Bivalvia;Autobranchia;Pteriomorphia;Ostreida;Ostreoidea;Ostreidae;Crassostrea;Crassostrea gigas
UdeM00000009154 29159 157.5 UdeM00000009154 XP_034317111.1 59 122 50 0 1 122 1 122 1.10E-35 157.5 29159 cellular organisms;Eukaryota;Opisthokonta;Metazoa;Eumetazoa;Bilateria;Protostomia;Spiralia;Lophotrochozoa;Mollusca;Bivalvia;Autobranchia;Pteriomorphia;Ostreida;Ostreoidea;Ostreidae;Crassostrea;Crassostrea gigas
UdeM00000009155 1735272 136 UdeM00000009155 XP_041377984.1 88 75 9 0 33 107 20 94 3.00E-29 136 1735272 cellular organisms;Eukaryota;Opisthokonta;Metazoa;Eumetazoa;Bilateria;Protostomia;Spiralia;Lophotrochozoa;Mollusca;Gastropoda;Neomphalina;Neomphaloidea;Peltospiridae;Gigantopelta;Gigantopelta aegis

UdeM00000009156 29159 77 UdeM00000009156 XP_034335954.1 32.2 146
93 4 63 204 32 175 1.80E-10 77 29159 cellular
organisms;Eukaryota;Opisthokonta;Metazoa;Eumetazoa;Bilateria;Protostomia;
Spiralia;Lophotrochozoa;Mollusca;Bivalvia;Autobranchia;Pteriomorphia;Ostr
eida;Ostreoidea;Ostreidae;Crassostrea;Crassostrea gigas
UdeM00000009157 29159 77 UdeM00000009157 XP_034335954.1 32.2 146
93 4 92 233 32 175 1.90E-10 77 29159 cellular
organisms;Eukaryota;Opisthokonta;Metazoa;Eumetazoa;Bilateria;Protostomia;
Spiralia;Lophotrochozoa;Mollusca;Bivalvia;Autobranchia;Pteriomorphia;Ostr
eida;Ostreoidea;Ostreidae;Crassostrea;Crassostrea gigas
UdeM00000009158 29159 84.7 UdeM00000009158 XP_034335954.1 36.4 140
82 4 99 233 38 175 4.70E-13 84.7 29159 cellular
organisms;Eukaryota;Opisthokonta;Metazoa;Eumetazoa;Bilateria;Protostomia;
Spiralia;Lophotrochozoa;Mollusca;Bivalvia;Autobranchia;Pteriomorphia;Ostr
eida;Ostreoidea;Ostreidae;Crassostrea;Crassostrea gigas
UdeM00000009160 29159 84.3 UdeM00000009160 XP_034335954.1 36.4 140
82 4 92 226 38 175 6.00E-13 84.3 29159 cellular
organisms;Eukaryota;Opisthokonta;Metazoa;Eumetazoa;Bilateria;Protostomia;
Spiralia;Lophotrochozoa;Mollusca;Bivalvia;Autobranchia;Pteriomorphia;Ostr
eida;Ostreoidea;Ostreidae;Crassostrea;Crassostrea gigas
UdeM00000009159 29159 84.3 UdeM00000009159 XP_034335954.1 36.4 140
82 4 99 233 38 175 6.00E-13 84.3 29159 cellular
organisms;Eukaryota;Opisthokonta;Metazoa;Eumetazoa;Bilateria;Protostomia;
Spiralia;Lophotrochozoa;Mollusca;Bivalvia;Autobranchia;Pteriomorphia;Ostr
eida;Ostreoidea;Ostreidae;Crassostrea;Crassostrea gigas
UdeM00000009161 6596 86.3 UdeM00000009161 XP_045215532.1 32.2 152
100 2 87 236 24 174 1.80E-13 86.3 6596 cellular
organisms;Eukaryota;Opisthokonta;Metazoa;Eumetazoa;Bilateria;Protostomia;
Spiralia;Lophotrochozoa;Mollusca;Bivalvia;Autobranchia;Heteroconchia;Euhe
terodonta;Imparidentia;Venerida;Veneroidea;Veneridae;Mercenaria;Mercenari
a mercenaria
UdeM00000009162 6596 607.1 UdeM00000009162 XP_045163368.1 63.9 463
163 3 1 460 69 530 1.90E-170 607.1 6596 cellular
organisms;Eukaryota;Opisthokonta;Metazoa;Eumetazoa;Bilateria;Protostomia;
Spiralia;Lophotrochozoa;Mollusca;Bivalvia;Autobranchia;Heteroconchia;Euhe
terodonta;Imparidentia;Venerida;Veneroidea;Veneridae;Mercenaria;Mercenari
a mercenaria
UdeM00000009163 6596 256.1 UdeM00000009163 XP_045163368.1 60.8 199
78 0 22 220 24 222 4.10E-65 256.1 6596 cellular
organisms;Eukaryota;Opisthokonta;Metazoa;Eumetazoa;Bilateria;Protostomia;
Spiralia;Lophotrochozoa;Mollusca;Bivalvia;Autobranchia;Heteroconchia;Euhe
terodonta;Imparidentia;Venerida;Veneroidea;Veneridae;Mercenaria;Mercenari
a mercenaria
UdeM00000009164 6596 604 UdeM00000009164 XP_045163368.1 58.9 511
202 5 22 526 24 532 1.90E-169 604 6596 cellular
organisms;Eukaryota;Opisthokonta;Metazoa;Eumetazoa;Bilateria;Protostomia;
Spiralia;Lophotrochozoa;Mollusca;Bivalvia;Autobranchia;Heteroconchia;Euhe
terodonta;Imparidentia;Venerida;Veneroidea;Veneridae;Mercenaria;Mercenari
a mercenaria
UdeM00000009165 6573 256.1 UdeM00000009165 XP_021360957.1 59.9 202
79 1 1 200 8 209 4.10E-65 256.1 6573 cellular
organisms;Eukaryota;Opisthokonta;Metazoa;Eumetazoa;Bilateria;Protostomia;
Spiralia;Lophotrochozoa;Mollusca;Bivalvia;Autobranchia;Pteriomorphia;Pect
inida;Pectinoidea;Pectinidae;Mizuhopecten;Mizuhopecten yessoensis

UdeM00000009166 6850 139.8 UdeM00000009166 XP_022252575.1 71.9 96
27 0 1 96 126 221 1.90E-30 139.8 6850 cellular
organisms;Eukaryota;Opisthokonta;Metazoa;Eumetazoa;Bilateria;Protostomia;
Ecdysozoa;Panarthropoda;Arthropoda;Chelicerata;Merostomata;Xiphosura;Limu
lidae;Limulus;Limulus polyphemus
UdeM00000009167 6850 95.9 UdeM00000009167 XP_022254320.1 66.2 71
21 1 19 86 413 483 3.70E-17 95.9 6850 cellular
organisms;Eukaryota;Opisthokonta;Metazoa;Eumetazoa;Bilateria;Protostomia;
Ecdysozoa;Panarthropoda;Arthropoda;Chelicerata;Merostomata;Xiphosura;Limu
lidae;Limulus;Limulus polyphemus
UdeM00000009168 6596 148.7 UdeM00000009168 XP_045163015.1 30.8 273
154 3 24 262 24 295 1.10E-32 148.7 6596 cellular
organisms;Eukaryota;Opisthokonta;Metazoa;Eumetazoa;Bilateria;Protostomia;
Spiralia;Lophotrochozoa;Mollusca;Bivalvia;Autobranchia;Heteroconchia;Euhe
terodonta;Imparidentia;Venerida;Veneroidea;Veneridae;Mercenaria;Mercenari
a mercenaria
UdeM00000009169 6565 852.8 UdeM00000009169 XP_022346113.1 97.5 433
11 0 1 433 1 433 1.90E-244 852.8 6565 cellular
organisms;Eukaryota;Opisthokonta;Metazoa;Eumetazoa;Bilateria;Protostomia;
Spiralia;Lophotrochozoa;Mollusca;Bivalvia;Autobranchia;Pteriomorphia;Ostr
eida;Ostreoidea;Ostreidae;Crassostrea;Crassostrea virginica
UdeM00000009170 6565 680.6 UdeM00000009170 XP_022286351.1 55.5 650
279 5 1 645 1 645 1.90E-192 680.6 6565 cellular
organisms;Eukaryota;Opisthokonta;Metazoa;Eumetazoa;Bilateria;Protostomia;
Spiralia;Lophotrochozoa;Mollusca;Bivalvia;Autobranchia;Pteriomorphia;Ostr
eida;Ostreoidea;Ostreidae;Crassostrea;Crassostrea virginica
UdeM00000009171 1232801 191.8 UdeM00000009171 XP_043193789.1 36
292 178 3 559 848 32 316 3.50E-45 191.8 1232801
cellular
organisms;Eukaryota;Opisthokonta;Metazoa;Eumetazoa;Bilateria;Protostomia;
Ecdysozoa;Panarthropoda;Arthropoda;Mandibulata;Pancrustacea;Crustacea;Mul
ticrustacea;Hexanauplia;Thecostraca;Cirripedia;Thoracica;Sessilia;Balanid
ae;Amphibalanus;Amphibalanus amphitrite
UdeM00000009172 6412 200.7 UdeM00000009172 XP_009014029.1 73.9 119
31 0 16 134 73 191 1.20E-48 200.7 6412 cellular
organisms;Eukaryota;Opisthokonta;Metazoa;Eumetazoa;Bilateria;Protostomia;
Spiralia;Lophotrochozoa;Annelida;Clitellata;Hirudinea;Rhynchobdellida;Glo
ssiphoniidae;Helobdella;Helobdella robusta
UdeM00000009173 1735272 319.7 UdeM00000009173 XP_041377683.1 77.9
190 42 0 3 192 5 194 2.50E-84 319.7 1735272
cellular
organisms;Eukaryota;Opisthokonta;Metazoa;Eumetazoa;Bilateria;Protostomia;
Spiralia;Lophotrochozoa;Mollusca;Gastropoda;Neomphalina;Neomphaloidea;Pel
tospiridae;Gigantopelta;Gigantopelta aegis
UdeM00000009174 6596 842 UdeM00000009174 XP_045162561.1 78.5 498
96 4 45 537 8 499 5.30E-241 842 6596 cellular
organisms;Eukaryota;Opisthokonta;Metazoa;Eumetazoa;Bilateria;Protostomia;
Spiralia;Lophotrochozoa;Mollusca;Bivalvia;Autobranchia;Heteroconchia;Euhe
terodonta;Imparidentia;Venerida;Veneroidea;Veneridae;Mercenaria;Mercenari
a mercenaria
UdeM00000009175 6596 736.1 UdeM00000009175 XP_045163744.1 45.6 1004
362 23 453 1304 766 1737 7.70E-209 736.1 6596 cellular
organisms;Eukaryota;Opisthokonta;Metazoa;Eumetazoa;Bilateria;Protostomia;
Spiralia;Lophotrochozoa;Mollusca;Bivalvia;Autobranchia;Heteroconchia;Euhe

terodonta;Imparidentia;Venerida;Veneroidea;Veneridae;Mercenaria;Mercenaria mercenaria
UdeM00000009177 34506 165.2 UdeM00000009177 XP_024502180.1 36.1 233 145 3 102 333 14 243 1.80E-37 165.2 34506 cellular organisms;Eukaryota;Opisthokonta;Metazoa;Eumetazoa;Bilateria;Protostomia;Ecdysozoa;Nematoda;Chromadorea;Rhabditida;Tylenchina;Panagrolaimomorpha;Strongyloidoidea;Strongyloididae;Strongyloides;Strongyloides ratti
UdeM00000009178 45264 336.3 UdeM00000009178 XP_044179091.1 63.1 233 85 1 1 233 202 433 3.20E-89 336.3 45264 cellular organisms;Eukaryota;Opisthokonta;Metazoa;Eumetazoa;Cnidaria;Anthozoa;Hexacorallia;Scleractinia;Astrocoeniina;Acroporidae;Acropora;Acropora millepora
UdeM00000009179 36176 218 UdeM00000009179 XP_043534371.1 65.5 139 48 0 1 139 279 417 7.70E-54 218 36176 cellular organisms;Eukaryota;Opisthokonta;Metazoa;Eumetazoa;Bilateria;Deuterostomia;Chordata;Craniata;Vertebrata;Gnathostomata;Chondrichthyes;Elasmobranchii;Selachii;Galeomorphii;Galeoidea;Orectolobiformes;Hemiscylliidae;Chiloscyllium;Chiloscyllium plagiosum
UdeM00000009180 50390 362.5 UdeM00000009180 XP_028142380.1 55 260 117 0 1 260 31 290 4.60E-97 362.5 50390 cellular organisms;Eukaryota;Opisthokonta;Metazoa;Eumetazoa;Bilateria;Protostomia;Ecdysozoa;Panarthropoda;Arthropoda;Mandibulata;Pancrustacea;Hexapoda;Insecta;Dicondylia;Pterygota;Neoptera;Endopterygota;Coleoptera;Polyphaga;Cucujiformia;Chrysomeloidea;Chrysomelidae;Galerucinae;Luperini;Diabroticina;Diabroticites;Diabrotica;Diabrotica virgifera;Diabrotica virgifera virgifera
UdeM00000009181 50390 362.5 UdeM00000009181 XP_028142380.1 55.4 258 115 0 2 259 33 290 4.60E-97 362.5 50390 cellular organisms;Eukaryota;Opisthokonta;Metazoa;Eumetazoa;Bilateria;Protostomia;Ecdysozoa;Panarthropoda;Arthropoda;Mandibulata;Pancrustacea;Hexapoda;Insecta;Dicondylia;Pterygota;Neoptera;Endopterygota;Coleoptera;Polyphaga;Cucujiformia;Chrysomeloidea;Chrysomelidae;Galerucinae;Luperini;Diabroticina;Diabroticites;Diabrotica;Diabrotica virgifera;Diabrotica virgifera virgifera
UdeM00000009183 481459 182.2 UdeM00000009183 XP_040031014.1 34.8 267 163 3 2 268 198 453 8.90E-43 182.2 481459 cellular organisms;Eukaryota;Opisthokonta;Metazoa;Eumetazoa;Bilateria;Deuterostomia;Chordata;Craniata;Vertebrata;Gnathostomata;Teleostomi;Euteleostomi;Actinopterygii;Actinopteri;Neopterygii;Teleostei;Osteoglossocephalai;Clupeocephala;Euteleosteomorpha;Neoteleostei;Eurypterygia;Ctenosquamata;Acanthomorphata;Euacanthomorphacea;Percomorphaceae;Eupercaria;Perciformes;Cottioidei;Gasterosteales;Gasterosteidae;Gasterosteus;Gasterosteus aculeatus;Gasterosteus aculeatus aculeatus
UdeM00000009184 6573 509.6 UdeM00000009184 XP_021367898.1 68.1 376 105 4 1 375 1 362 3.40E-141 509.6 6573 cellular organisms;Eukaryota;Opisthokonta;Metazoa;Eumetazoa;Bilateria;Protostomia;Spiralia;Lophotrochozoa;Mollusca;Bivalvia;Autobranchia;Pteriomorphia;Pectinida;Pectinoidea;Pectinidae;Mizuhopecten;Mizuhopecten yessoensis
UdeM00000009186 6573 222.6 UdeM00000009186 XP_021372552.1 63.1 187 32 3 30 215 30 180 7.60E-55 222.6 6573 cellular organisms;Eukaryota;Opisthokonta;Metazoa;Eumetazoa;Bilateria;Protostomia;Spiralia;Lophotrochozoa;Mollusca;Bivalvia;Autobranchia;Pteriomorphia;Pectinida;Pectinoidea;Pectinidae;Mizuhopecten;Mizuhopecten yessoensis

UdeM00000009185 6573 273.9 UdeM00000009185 XP_021372552.1 66.7 213
34 3 6 217 4 180 2.90E-70 273.9 6573 cellular
organisms;Eukaryota;Opisthokonta;Metazoa;Eumetazoa;Bilateria;Protostomia;
Spiralia;Lophotrochozoa;Mollusca;Bivalvia;Autobranchia;Pteriomorphia;Pect
inida;Pectinoidea;Pectinidae;Mizuhopecten;Mizuhopecten yessoensis
UdeM00000009188 6565 284.6 UdeM00000009188 XP_022337637.1 42.1 667
286 21 213 848 187 784 4.60E-73 284.6 6565 cellular
organisms;Eukaryota;Opisthokonta;Metazoa;Eumetazoa;Bilateria;Protostomia;
Spiralia;Lophotrochozoa;Mollusca;Bivalvia;Autobranchia;Pteriomorphia;Ostr
eida;Ostreoidea;Ostreidae;Crassostrea;Crassostrea virginica
UdeM00000009189 6596 228.8 UdeM00000009189 XP_045161222.1 67.6 179
57 1 9 187 6 183 6.00E-57 228.8 6596 cellular
organisms;Eukaryota;Opisthokonta;Metazoa;Eumetazoa;Bilateria;Protostomia;
Spiralia;Lophotrochozoa;Mollusca;Bivalvia;Autobranchia;Heteroconchia;Euhe
terodonta;Imparidentia;Venerida;Veneroidea;Veneridae;Mercenaria;Mercenari
a mercenaria
UdeM00000009190 29159 119.4 UdeM00000009190 XP_011422610.1 46.2 173
69 6 1 169 1 153 4.50E-24 119.4 29159 cellular
organisms;Eukaryota;Opisthokonta;Metazoa;Eumetazoa;Bilateria;Protostomia;
Spiralia;Lophotrochozoa;Mollusca;Bivalvia;Autobranchia;Pteriomorphia;Ostr
eida;Ostreoidea;Ostreidae;Crassostrea;Crassostrea gigas
UdeM00000009191 6596 114 UdeM00000009191 XP_045163784.1 46.9 147
71 4 7 152 18 158 1.70E-22 114 6596 cellular
organisms;Eukaryota;Opisthokonta;Metazoa;Eumetazoa;Bilateria;Protostomia;
Spiralia;Lophotrochozoa;Mollusca;Bivalvia;Autobranchia;Heteroconchia;Euhe
terodonta;Imparidentia;Venerida;Veneroidea;Veneridae;Mercenaria;Mercenari
a mercenaria
UdeM00000009192 6596 1376.7 UdeM00000009192 XP_045163785.1 60.8
1200 440 11 160 1353 6 1180 0.00E+00 1376.7 6596
cellular
organisms;Eukaryota;Opisthokonta;Metazoa;Eumetazoa;Bilateria;Protostomia;
Spiralia;Lophotrochozoa;Mollusca;Bivalvia;Autobranchia;Heteroconchia;Euhe
terodonta;Imparidentia;Venerida;Veneroidea;Veneridae;Mercenaria;Mercenari
a mercenaria
UdeM00000009193 6500 411.8 UdeM00000009193 XP_012939659.1 66.6 314
103 1 80 393 15 326 1.00E-111 411.8 6500 cellular
organisms;Eukaryota;Opisthokonta;Metazoa;Eumetazoa;Bilateria;Protostomia;
Spiralia;Lophotrochozoa;Mollusca;Gastropoda;Heterobranchia;Euthyneura;Tec
tipleura;Aplysiida;Aplysioidea;Aplysiidae;Aplysia;Aplysia californica
UdeM00000009194 6500 411.8 UdeM00000009194 XP_012939659.1 66.6 314
103 1 23 336 15 326 8.70E-112 411.8 6500 cellular
organisms;Eukaryota;Opisthokonta;Metazoa;Eumetazoa;Bilateria;Protostomia;
Spiralia;Lophotrochozoa;Mollusca;Gastropoda;Heterobranchia;Euthyneura;Tec
tipleura;Aplysiida;Aplysioidea;Aplysiidae;Aplysia;Aplysia californica
UdeM00000009195 400727 1048.9 UdeM00000009195 XP_025081660.1
74.9 676 167 1 13 688 13 685 2.80E-303 1048.9
400727 cellular
organisms;Eukaryota;Opisthokonta;Metazoa;Eumetazoa;Bilateria;Protostomia;
Spiralia;Lophotrochozoa;Mollusca;Gastropoda;Caenogastropoda;Architaeniogl
ossa;Ampullarioidea;Ampullariidae;Pomacea;Pomacea canaliculata
UdeM00000009196 29159 335.5 UdeM00000009196 XP_011429477.2 60.3 247
95 2 31 275 2 247 6.50E-89 335.5 29159 cellular
organisms;Eukaryota;Opisthokonta;Metazoa;Eumetazoa;Bilateria;Protostomia;

Spiralia;Lophotrochozoa;Mollusca;Bivalvia;Autobranchia;Pteriomorphia;Ostreida;Ostreoidea;Ostreidae;Crassostrea;Crassostrea gigas
UdeM00000009197 29159 335.9 UdeM00000009197 XP_011429477.2 60.3 247 95 2 6 250 2 247 4.50E-89 335.9 29159 cellular organisms;Eukaryota;Opisthokonta;Metazoa;Eumetazoa;Bilateria;Protostomia;Spiralia;Lophotrochozoa;Mollusca;Bivalvia;Autobranchia;Pteriomorphia;Ostreida;Ostreoidea;Ostreidae;Crassostrea;Crassostrea gigas
UdeM00000009199 6596 672.5 UdeM00000009199 XP_045161419.1 50.9 636 301 3 17 647 32 661 5.20E-190 672.5 6596 cellular organisms;Eukaryota;Opisthokonta;Metazoa;Eumetazoa;Bilateria;Protostomia;Spiralia;Lophotrochozoa;Mollusca;Bivalvia;Autobranchia;Heteroconchia;Euheterodonta;Imparidentia;Venerida;Veneroidea;Veneridae;Mercenaria;Mercenaria mercenaria
UdeM00000009200 6596 847.4 UdeM00000009200 XP_045161419.1 51.1 792 309 3 24 778 23 773 1.40E-242 847.4 6596 cellular organisms;Eukaryota;Opisthokonta;Metazoa;Eumetazoa;Bilateria;Protostomia;Spiralia;Lophotrochozoa;Mollusca;Bivalvia;Autobranchia;Heteroconchia;Euheterodonta;Imparidentia;Venerida;Veneroidea;Veneridae;Mercenaria;Mercenaria mercenaria
UdeM00000009203 6596 906.4 UdeM00000009203 XP_045161419.1 55.7 752 332 1 8 759 23 773 2.50E-260 906.4 6596 cellular organisms;Eukaryota;Opisthokonta;Metazoa;Eumetazoa;Bilateria;Protostomia;Spiralia;Lophotrochozoa;Mollusca;Bivalvia;Autobranchia;Heteroconchia;Euheterodonta;Imparidentia;Venerida;Veneroidea;Veneridae;Mercenaria;Mercenaria mercenaria
UdeM00000009204 9337 62 UdeM00000009204 XP_036612368.1 30.4 135 67 4 58 168 170 301 8.40E-07 62 9337 cellular organisms;Eukaryota;Opisthokonta;Metazoa;Eumetazoa;Bilateria;Deuterostomia;Chordata;Craniata;Vertebrata;Gnathostomata;Teleostomi;Euteleostomi;Sarcopterygii;Dipnotetrapodomorpha;Tetrapoda;Amniota;Mammalia;Theria;Metatheria;Diprotodontia;Phalangeridae;Trichosurus;Trichosurus vulpecula
UdeM00000009205 6573 223.4 UdeM00000009205 XP_021362041.1 40.1 279 164 3 21 298 15 291 3.90E-55 223.4 6573 cellular organisms;Eukaryota;Opisthokonta;Metazoa;Eumetazoa;Bilateria;Protostomia;Spiralia;Lophotrochozoa;Mollusca;Bivalvia;Autobranchia;Pteriomorphia;Pectinida;Pectinoidea;Pectinidae;Mizuhopecten;Mizuhopecten yessoensis
UdeM00000009206 6573 190.7 UdeM00000009206 XP_021362041.1 32.4 346 164 4 21 365 15 291 3.40E-45 190.7 6573 cellular organisms;Eukaryota;Opisthokonta;Metazoa;Eumetazoa;Bilateria;Protostomia;Spiralia;Lophotrochozoa;Mollusca;Bivalvia;Autobranchia;Pteriomorphia;Pectinida;Pectinoidea;Pectinidae;Mizuhopecten;Mizuhopecten yessoensis
UdeM00000009209 6596 1371.3 UdeM00000009209 XP_045161717.1 51.9 1426 499 22 62 1355 469 1839 0.00E+00 1371.3 6596 cellular organisms;Eukaryota;Opisthokonta;Metazoa;Eumetazoa;Bilateria;Protostomia;Spiralia;Lophotrochozoa;Mollusca;Bivalvia;Autobranchia;Heteroconchia;Euheterodonta;Imparidentia;Venerida;Veneroidea;Veneridae;Mercenaria;Mercenaria mercenaria
UdeM00000009210 6596 85.5 UdeM00000009210 XP_045161717.1 30.7 469 280 13 4 447 3 451 1.90E-13 85.5 6596 cellular organisms;Eukaryota;Opisthokonta;Metazoa;Eumetazoa;Bilateria;Protostomia;Spiralia;Lophotrochozoa;Mollusca;Bivalvia;Autobranchia;Heteroconchia;Euheterodonta;Imparidentia;Venerida;Veneroidea;Veneridae;Mercenaria;Mercenaria mercenaria

UdeM00000009211 225164 409.5 UdeM00000009211 XP_009060553.1 73.5
268 71 0 76 343 48 315 5.00E-111 409.5 225164
cellular
organisms;Eukaryota;Opisthokonta;Metazoa;Eumetazoa;Bilateria;Protostomia;
Spiralia;Lophotrochozoa;Mollusca;Gastropoda;Patellogastropoda;Lottioidea;
Lottiidae;Lottia;Lottia gigantea
UdeM00000009213 225164 421.4 UdeM00000009213 XP_009060553.1 72.5
287 79 0 76 362 48 334 1.20E-114 421.4 225164
cellular
organisms;Eukaryota;Opisthokonta;Metazoa;Eumetazoa;Bilateria;Protostomia;
Spiralia;Lophotrochozoa;Mollusca;Gastropoda;Patellogastropoda;Lottioidea;
Lottiidae;Lottia;Lottia gigantea
UdeM00000009212 225164 417.5 UdeM00000009212 XP_009060553.1 73.2
276 74 0 76 351 48 323 1.70E-113 417.5 225164
cellular
organisms;Eukaryota;Opisthokonta;Metazoa;Eumetazoa;Bilateria;Protostomia;
Spiralia;Lophotrochozoa;Mollusca;Gastropoda;Patellogastropoda;Lottioidea;
Lottiidae;Lottia;Lottia gigantea
UdeM00000009215 6596 388.3 UdeM00000009215 XP_045161723.1 65.6 302
93 1 51 352 51 341 1.10E-104 388.3 6596 cellular
organisms;Eukaryota;Opisthokonta;Metazoa;Eumetazoa;Bilateria;Protostomia;
Spiralia;Lophotrochozoa;Mollusca;Bivalvia;Autobranchia;Heteroconchia;Euhe
terodonta;Imparidentia;Venerida;Veneroidea;Veneridae;Mercenaria;Mercenari
a mercenaria
UdeM00000009214 6596 389 UdeM00000009214 XP_045161723.1 68.9 280
87 0 51 330 51 330 6.20E-105 389 6596 cellular
organisms;Eukaryota;Opisthokonta;Metazoa;Eumetazoa;Bilateria;Protostomia;
Spiralia;Lophotrochozoa;Mollusca;Bivalvia;Autobranchia;Heteroconchia;Euhe
terodonta;Imparidentia;Venerida;Veneroidea;Veneridae;Mercenaria;Mercenari
a mercenaria
UdeM00000009216 6596 142.5 UdeM00000009216 XP_045162514.1 67.9 106
33 1 6 111 491 595 3.40E-31 142.5 6596 cellular
organisms;Eukaryota;Opisthokonta;Metazoa;Eumetazoa;Bilateria;Protostomia;
Spiralia;Lophotrochozoa;Mollusca;Bivalvia;Autobranchia;Heteroconchia;Euhe
terodonta;Imparidentia;Venerida;Veneroidea;Veneridae;Mercenaria;Mercenari
a mercenaria
UdeM00000009217 6596 142.5 UdeM00000009217 XP_045162514.1 67.9 106
33 1 96 201 491 595 6.00E-31 142.5 6596 cellular
organisms;Eukaryota;Opisthokonta;Metazoa;Eumetazoa;Bilateria;Protostomia;
Spiralia;Lophotrochozoa;Mollusca;Bivalvia;Autobranchia;Heteroconchia;Euhe
terodonta;Imparidentia;Venerida;Veneroidea;Veneridae;Mercenaria;Mercenari
a mercenaria
UdeM00000009218 6596 1052 UdeM00000009218 XP_045162514.1 84.7 587
87 2 23 607 10 595 3.00E-304 1052 6596 cellular
organisms;Eukaryota;Opisthokonta;Metazoa;Eumetazoa;Bilateria;Protostomia;
Spiralia;Lophotrochozoa;Mollusca;Bivalvia;Autobranchia;Heteroconchia;Euhe
terodonta;Imparidentia;Venerida;Veneroidea;Veneridae;Mercenaria;Mercenari
a mercenaria
UdeM00000009219 29159 312.8 UdeM00000009219 XP_011456876.2 31.8 629
266 16 23 638 114 592 1.00E-81 312.8 29159 cellular
organisms;Eukaryota;Opisthokonta;Metazoa;Eumetazoa;Bilateria;Protostomia;
Spiralia;Lophotrochozoa;Mollusca;Bivalvia;Autobranchia;Pteriomorphia;Ostr
eida;Ostreoidea;Ostreidae;Crassostrea;Crassostrea gigas

UdeM00000009220 6596 85.5 UdeM00000009220 XP_045162568.1 56.2 89
33 3 2 89 548 631 4.30E-14 85.5 6596 cellular
organisms;Eukaryota;Opisthokonta;Metazoa;Eumetazoa;Bilateria;Protostomia;
Spiralia;Lophotrochozoa;Mollusca;Bivalvia;Autobranchia;Heteroconchia;Euhe
terodonta;Imparidentia;Venerida;Veneroidea;Veneridae;Mercenaria;Mercenari
a mercenaria
UdeM00000009222 29159 162.2 UdeM00000009222 XP_011456876.2 41.4 203
112 4 91 288 404 604 1.00E-36 162.2 29159 cellular
organisms;Eukaryota;Opisthokonta;Metazoa;Eumetazoa;Bilateria;Protostomia;
Spiralia;Lophotrochozoa;Mollusca;Bivalvia;Autobranchia;Pteriomorphia;Ostr
eida;Ostreoidea;Ostreidae;Crassostrea;Crassostrea gigas
UdeM00000009223 29159 521.2 UdeM00000009223 XP_011456876.2 37.1 901
367 22 24 899 116 841 2.70E-144 521.2 29159 cellular
organisms;Eukaryota;Opisthokonta;Metazoa;Eumetazoa;Bilateria;Protostomia;
Spiralia;Lophotrochozoa;Mollusca;Bivalvia;Autobranchia;Pteriomorphia;Ostr
eida;Ostreoidea;Ostreidae;Crassostrea;Crassostrea gigas
UdeM00000009224 151771 318.5 UdeM00000009224 XP_028415423.1 32.6
607 273 8 2 477 23 624 1.40E-83 318.5 151771
cellular
organisms;Eukaryota;Opisthokonta;Metazoa;Eumetazoa;Cnidaria;Anthozoa;Octo
corallia;Alcyonacea;Alcyoniina;Nephtheidae;Dendronephthya;Dendronephthya
gigantea
UdeM00000009225 29159 169.9 UdeM00000009225 XP_011456876.2 64.6 130
45 1 1 130 713 841 2.30E-39 169.9 29159 cellular
organisms;Eukaryota;Opisthokonta;Metazoa;Eumetazoa;Bilateria;Protostomia;
Spiralia;Lophotrochozoa;Mollusca;Bivalvia;Autobranchia;Pteriomorphia;Ostr
eida;Ostreoidea;Ostreidae;Crassostrea;Crassostrea gigas
UdeM00000009226 6573 87.4 UdeM00000009226 XP_021365625.1 51.2 86
42 0 16 101 41 126 2.40E-14 87.4 6573 cellular
organisms;Eukaryota;Opisthokonta;Metazoa;Eumetazoa;Bilateria;Protostomia;
Spiralia;Lophotrochozoa;Mollusca;Bivalvia;Autobranchia;Pteriomorphia;Pect
inida;Pectinoidea;Pectinidae;Mizuhopecten;Mizuhopecten yessoensis
UdeM00000009227 29159 400.6 UdeM00000009227 XP_011456876.2 36.1 675
329 18 24 671 116 715 4.50E-108 400.6 29159 cellular
organisms;Eukaryota;Opisthokonta;Metazoa;Eumetazoa;Bilateria;Protostomia;
Spiralia;Lophotrochozoa;Mollusca;Bivalvia;Autobranchia;Pteriomorphia;Ostr
eida;Ostreoidea;Ostreidae;Crassostrea;Crassostrea gigas
UdeM00000009228 400727 288.1 UdeM00000009228 XP_025107489.1 53.2
293 133 3 88 377 147 438 1.60E-74 288.1 400727
cellular
organisms;Eukaryota;Opisthokonta;Metazoa;Eumetazoa;Bilateria;Protostomia;
Spiralia;Lophotrochozoa;Mollusca;Gastropoda;Caenogastropoda;Architaeniogl
ossa;Ampullarioidea;Ampullariidae;Pomacea;Pomacea canaliculata
UdeM00000009229 29159 522.3 UdeM00000009229 XP_011456876.2 42.2 747
370 24 22 731 121 842 9.90E-145 522.3 29159 cellular
organisms;Eukaryota;Opisthokonta;Metazoa;Eumetazoa;Bilateria;Protostomia;
Spiralia;Lophotrochozoa;Mollusca;Bivalvia;Autobranchia;Pteriomorphia;Ostr
eida;Ostreoidea;Ostreidae;Crassostrea;Crassostrea gigas
UdeM00000009230 6573 210.3 UdeM00000009230 XP_021376553.1 36.5 323
149 1 1 267 81 403 3.00E-51 210.3 6573 cellular
organisms;Eukaryota;Opisthokonta;Metazoa;Eumetazoa;Bilateria;Protostomia;
Spiralia;Lophotrochozoa;Mollusca;Bivalvia;Autobranchia;Pteriomorphia;Pect
inida;Pectinoidea;Pectinidae;Mizuhopecten;Mizuhopecten yessoensis

UdeM00000009234	6596	916.4	UdeM00000009234	XP_045163454.1	73.4	623
153	4	2	615	11	629	2.00E-263	916.4	6596	cellular
organisms;Eukaryota;Opisthokonta;Metazoa;Eumetazoa;Bilateria;Protostomia;
Spiralia;Lophotrochozoa;Mollusca;Bivalvia;Autobranchia;Heteroconchia;Euhe
terodonta;Imparidentia;Venerida;Veneroidea;Veneridae;Mercenaria;Mercenari
a mercenaria
UdeM00000009235	6565	193	UdeM00000009235	XP_022299727.1	31.8	377
230	9	39	401	434	797	8.00E-46	193	6565	cellular
organisms;Eukaryota;Opisthokonta;Metazoa;Eumetazoa;Bilateria;Protostomia;
Spiralia;Lophotrochozoa;Mollusca;Bivalvia;Autobranchia;Pteriomorphia;Ostr
eida;Ostreoidea;Ostreidae;Crassostrea;Crassostrea virginica
UdeM00000009236	29159	245.7	UdeM00000009236	XP_011430393.1	46.4	276
138	3	20	290	11	281	7.10E-62	245.7	29159	cellular
organisms;Eukaryota;Opisthokonta;Metazoa;Eumetazoa;Bilateria;Protostomia;
Spiralia;Lophotrochozoa;Mollusca;Bivalvia;Autobranchia;Pteriomorphia;Ostr
eida;Ostreoidea;Ostreidae;Crassostrea;Crassostrea gigas
UdeM00000009237	6596	98.2	UdeM00000009237	XP_045162188.1	34.6	153
95	3	25	173	74	225	2.60E-17	98.2	6596	cellular
organisms;Eukaryota;Opisthokonta;Metazoa;Eumetazoa;Bilateria;Protostomia;
Spiralia;Lophotrochozoa;Mollusca;Bivalvia;Autobranchia;Heteroconchia;Euhe
terodonta;Imparidentia;Venerida;Veneroidea;Veneridae;Mercenaria;Mercenari
a mercenaria
UdeM00000009241	1735272	193.7	UdeM00000009241	XP_041355121.1	50.2
215	96	5	1	207	1	212	3.10E-46	193.7	1735272
cellular
organisms;Eukaryota;Opisthokonta;Metazoa;Eumetazoa;Bilateria;Protostomia;
Spiralia;Lophotrochozoa;Mollusca;Gastropoda;Neomphalina;Neomphaloidea;Pel
tospiridae;Gigantopelta;Gigantopelta aegis
UdeM00000009248	45264	270.4	UdeM00000009248	XP_044165143.1	40	412
149	7	27	361	35	425	3.30E-69	270.4	45264	cellular
organisms;Eukaryota;Opisthokonta;Metazoa;Eumetazoa;Cnidaria;Anthozoa;Hexa
corallia;Scleractinia;Astrocoeniina;Acroporidae;Acropora;Acropora
millepora
UdeM00000009249	45264	172.9	UdeM00000009249	XP_044184031.1	48.7	191
88	5	1	185	300	486	3.70E-40	172.9	45264	cellular
organisms;Eukaryota;Opisthokonta;Metazoa;Eumetazoa;Cnidaria;Anthozoa;Hexa
corallia;Scleractinia;Astrocoeniina;Acroporidae;Acropora;Acropora
millepora
UdeM00000009250	6596	456.8	UdeM00000009250	XP_045162568.1	46	658
304	19	3	639	4	631	4.50E-125	456.8	6596	cellular
organisms;Eukaryota;Opisthokonta;Metazoa;Eumetazoa;Bilateria;Protostomia;
Spiralia;Lophotrochozoa;Mollusca;Bivalvia;Autobranchia;Heteroconchia;Euhe
terodonta;Imparidentia;Venerida;Veneroidea;Veneridae;Mercenaria;Mercenari
a mercenaria
UdeM00000009251	6596	409.1	UdeM00000009251	XP_045162570.1	56.1	367
151	4	19	383	19	377	6.30E-111	409.1	6596	cellular
organisms;Eukaryota;Opisthokonta;Metazoa;Eumetazoa;Bilateria;Protostomia;
Spiralia;Lophotrochozoa;Mollusca;Bivalvia;Autobranchia;Heteroconchia;Euhe
terodonta;Imparidentia;Venerida;Veneroidea;Veneridae;Mercenaria;Mercenari
a mercenaria
UdeM00000009252	1735272	105.1	UdeM00000009252	XP_041378199.1	58.3
108	42	2	1	107	1	106	5.50E-20	105.1	1735272
cellular
organisms;Eukaryota;Opisthokonta;Metazoa;Eumetazoa;Bilateria;Protostomia;

Spiralia;Lophotrochozoa;Mollusca;Gastropoda;Neomphalina;Neomphaloidea;Peltospiridae;Gigantopelta;Gigantopelta aegis
UdeM00000009253 6579 396.4 UdeM00000009253 XP_033764004.1 41.2 537 291 9 1 519 1 530 6.10E-107 396.4 6579 cellular organisms;Eukaryota;Opisthokonta;Metazoa;Eumetazoa;Bilateria;Protostomia;Spiralia;Lophotrochozoa;Mollusca;Bivalvia;Autobranchia;Pteriomorphia;Pectinida;Pectinoidea;Pectinidae;Pecten;Pecten maximus
UdeM00000009254 6596 280 UdeM00000009254 XP_045161114.1 86.3 153 21 0 34 186 655 807 2.20E-72 280 6596 cellular organisms;Eukaryota;Opisthokonta;Metazoa;Eumetazoa;Bilateria;Protostomia;Spiralia;Lophotrochozoa;Mollusca;Bivalvia;Autobranchia;Heteroconchia;Euheterodonta;Imparidentia;Venerida;Veneroidea;Veneridae;Mercenaria;Mercenaria mercenaria
UdeM00000009255 6596 281.2 UdeM00000009255 XP_045161115.1 50.8 368 150 11 15 363 176 531 2.30E-72 281.2 6596 cellular organisms;Eukaryota;Opisthokonta;Metazoa;Eumetazoa;Bilateria;Protostomia;Spiralia;Lophotrochozoa;Mollusca;Bivalvia;Autobranchia;Heteroconchia;Euheterodonta;Imparidentia;Venerida;Veneroidea;Veneridae;Mercenaria;Mercenaria mercenaria
UdeM00000009256 6579 125.9 UdeM00000009256 XP_033763686.1 73.8 80 20 1 5 84 117 195 2.40E-26 125.9 6579 cellular organisms;Eukaryota;Opisthokonta;Metazoa;Eumetazoa;Bilateria;Protostomia;Spiralia;Lophotrochozoa;Mollusca;Bivalvia;Autobranchia;Pteriomorphia;Pectinida;Pectinoidea;Pectinidae;Pecten;Pecten maximus
UdeM00000009257 6596 1410.6 UdeM00000009257 XP_045161334.1 58.9 1256 432 12 11 1205 17 1249 0.00E+00 1410.6 6596 cellular organisms;Eukaryota;Opisthokonta;Metazoa;Eumetazoa;Bilateria;Protostomia;Spiralia;Lophotrochozoa;Mollusca;Bivalvia;Autobranchia;Heteroconchia;Euheterodonta;Imparidentia;Venerida;Veneroidea;Veneridae;Mercenaria;Mercenaria mercenaria
UdeM00000009259 6596 262.3 UdeM00000009259 XP_045192050.1 50.6 239 118 0 123 361 18 256 9.20E-67 262.3 6596 cellular organisms;Eukaryota;Opisthokonta;Metazoa;Eumetazoa;Bilateria;Protostomia;Spiralia;Lophotrochozoa;Mollusca;Bivalvia;Autobranchia;Heteroconchia;Euheterodonta;Imparidentia;Venerida;Veneroidea;Veneridae;Mercenaria;Mercenaria mercenaria
UdeM00000009260 6596 1218.8 UdeM00000009260 XP_045161330.1 62.5 1018 285 7 16 1024 201 1130 0.00E+00 1218.8 6596 cellular organisms;Eukaryota;Opisthokonta;Metazoa;Eumetazoa;Bilateria;Protostomia;Spiralia;Lophotrochozoa;Mollusca;Bivalvia;Autobranchia;Heteroconchia;Euheterodonta;Imparidentia;Venerida;Veneroidea;Veneridae;Mercenaria;Mercenaria mercenaria
UdeM00000009261 6596 1406 UdeM00000009261 XP_045161330.1 59.1 1231 435 11 14 1190 3 1219 0.00E+00 1406 6596 cellular organisms;Eukaryota;Opisthokonta;Metazoa;Eumetazoa;Bilateria;Protostomia;Spiralia;Lophotrochozoa;Mollusca;Bivalvia;Autobranchia;Heteroconchia;Euheterodonta;Imparidentia;Venerida;Veneroidea;Veneridae;Mercenaria;Mercenaria mercenaria
UdeM00000009262 6596 1379.4 UdeM00000009262 XP_045161330.1 59 1214 427 11 9 1165 20 1219 0.00E+00 1379.4 6596 cellular organisms;Eukaryota;Opisthokonta;Metazoa;Eumetazoa;Bilateria;Protostomia;

Spiralia;Lophotrochozoa;Mollusca;Bivalvia;Autobranchia;Heteroconchia;Euheterodonta;Imparidentia;Venerida;Veneroidea;Veneridae;Mercenaria;Mercenaria mercenaria
UdeM00000009263 6596 1379.4 UdeM00000009263 XP_045161330.1 59 1214 427 11 9 1165 20 1219 0.00E+00 1379.4 6596 cellular organisms;Eukaryota;Opisthokonta;Metazoa;Eumetazoa;Bilateria;Protostomia;Spiralia;Lophotrochozoa;Mollusca;Bivalvia;Autobranchia;Heteroconchia;Euheterodonta;Imparidentia;Venerida;Veneroidea;Veneridae;Mercenaria;Mercenaria mercenaria
UdeM00000009265 45264 393.3 UdeM00000009265 XP_044165535.1 49.6 367 181 1 1 363 158 524 5.80E-106 393.3 45264 cellular organisms;Eukaryota;Opisthokonta;Metazoa;Eumetazoa;Cnidaria;Anthozoa;Hexacorallia;Scleractinia;Astrocoeniina;Acroporidae;Acropora;Acropora millepora
UdeM00000009267 6596 119.8 UdeM00000009267 XP_045161772.1 35.6 267 131 8 1 261 1 232 5.30E-24 119.8 6596 cellular organisms;Eukaryota;Opisthokonta;Metazoa;Eumetazoa;Bilateria;Protostomia;Spiralia;Lophotrochozoa;Mollusca;Bivalvia;Autobranchia;Heteroconchia;Euheterodonta;Imparidentia;Venerida;Veneroidea;Veneridae;Mercenaria;Mercenaria mercenaria
UdeM00000009268 225164 248.4 UdeM00000009268 XP_009058076.1 58 212 89 0 5 216 4 215 8.10E-63 248.4 225164 cellular organisms;Eukaryota;Opisthokonta;Metazoa;Eumetazoa;Bilateria;Protostomia;Spiralia;Lophotrochozoa;Mollusca;Gastropoda;Patellogastropoda;Lottioidea;Lottiidae;Lottia;Lottia gigantea
UdeM00000009269 6565 684.9 UdeM00000009269 XP_022343652.1 77.7 489 103 3 107 589 138 626 9.40E-194 684.9 6565 cellular organisms;Eukaryota;Opisthokonta;Metazoa;Eumetazoa;Bilateria;Protostomia;Spiralia;Lophotrochozoa;Mollusca;Bivalvia;Autobranchia;Pteriomorphia;Ostreida;Ostreoidea;Ostreidae;Crassostrea;Crassostrea virginica
UdeM00000009270 6596 125.9 UdeM00000009270 XP_045180427.1 33.6 274 127 4 1 220 1 273 6.60E-26 125.9 6596 cellular organisms;Eukaryota;Opisthokonta;Metazoa;Eumetazoa;Bilateria;Protostomia;Spiralia;Lophotrochozoa;Mollusca;Bivalvia;Autobranchia;Heteroconchia;Euheterodonta;Imparidentia;Venerida;Veneroidea;Veneridae;Mercenaria;Mercenaria mercenaria
UdeM00000009271 6596 90.9 UdeM00000009271 XP_045163075.1 40.7 140 78 3 1 137 1 138 2.80E-15 90.9 6596 cellular organisms;Eukaryota;Opisthokonta;Metazoa;Eumetazoa;Bilateria;Protostomia;Spiralia;Lophotrochozoa;Mollusca;Bivalvia;Autobranchia;Heteroconchia;Euheterodonta;Imparidentia;Venerida;Veneroidea;Veneridae;Mercenaria;Mercenaria mercenaria
UdeM00000009272 6596 283.1 UdeM00000009272 XP_045162299.1 67.8 208 58 2 1 199 1 208 2.80E-73 283.1 6596 cellular organisms;Eukaryota;Opisthokonta;Metazoa;Eumetazoa;Bilateria;Protostomia;Spiralia;Lophotrochozoa;Mollusca;Bivalvia;Autobranchia;Heteroconchia;Euheterodonta;Imparidentia;Venerida;Veneroidea;Veneridae;Mercenaria;Mercenaria mercenaria
UdeM00000009273 6596 112.1 UdeM00000009273 XP_045161599.1 29.7 263 154 7 20 260 27 280 1.40E-21 112.1 6596 cellular organisms;Eukaryota;Opisthokonta;Metazoa;Eumetazoa;Bilateria;Protostomia;Spiralia;Lophotrochozoa;Mollusca;Bivalvia;Autobranchia;Heteroconchia;Euhe

terodonta;Imparidentia;Venerida;Veneroidea;Veneridae;Mercenaria;Mercenaria mercenaria
UdeM00000009274 7574 143.7 UdeM00000009274 XP_013405563.1 34.5 267 138 6 1 264 146 378 3.50E-31 143.7 7574 cellular organisms;Eukaryota;Opisthokonta;Metazoa;Eumetazoa;Bilateria;Protostomia;Spiralia;Lophotrochozoa;Brachiopoda;Linguliformea;Lingulata;Lingulida;Linguloidea;Lingulidae;Lingula;Lingula anatina
UdeM00000009275 45264 328.9 UdeM00000009275 XP_044182736.1 39.4 485 261 8 2 469 385 853 1.00E-86 328.9 45264 cellular organisms;Eukaryota;Opisthokonta;Metazoa;Eumetazoa;Cnidaria;Anthozoa;Hexacorallia;Scleractinia;Astrocoeniina;Acroporidae;Acropora;Acropora millepora
UdeM00000009276 6579 243.8 UdeM00000009276 XP_033739209.1 48.4 258 117 2 9 261 2 248 2.40E-61 243.8 6579 cellular organisms;Eukaryota;Opisthokonta;Metazoa;Eumetazoa;Bilateria;Protostomia;Spiralia;Lophotrochozoa;Mollusca;Bivalvia;Autobranchia;Pteriomorphia;Pectinida;Pectinoidea;Pectinidae;Pecten;Pecten maximus
UdeM00000009277 6579 331.3 UdeM00000009277 XP_033724599.1 51 402 175 8 12 405 14 401 1.80E-87 331.3 6579 cellular organisms;Eukaryota;Opisthokonta;Metazoa;Eumetazoa;Bilateria;Protostomia;Spiralia;Lophotrochozoa;Mollusca;Bivalvia;Autobranchia;Pteriomorphia;Pectinida;Pectinoidea;Pectinidae;Pecten;Pecten maximus
UdeM00000009278 6596 312.8 UdeM00000009278 XP_045162550.1 43.1 429 226 7 4 416 3 429 6.70E-82 312.8 6596 cellular organisms;Eukaryota;Opisthokonta;Metazoa;Eumetazoa;Bilateria;Protostomia;Spiralia;Lophotrochozoa;Mollusca;Bivalvia;Autobranchia;Heteroconchia;Euheterodonta;Imparidentia;Venerida;Veneroidea;Veneridae;Mercenaria;Mercenaria mercenaria
UdeM00000009279 6596 889.8 UdeM00000009279 XP_045162356.1 69.8 625 187 1 11 635 12 634 2.00E-255 889.8 6596 cellular organisms;Eukaryota;Opisthokonta;Metazoa;Eumetazoa;Bilateria;Protostomia;Spiralia;Lophotrochozoa;Mollusca;Bivalvia;Autobranchia;Heteroconchia;Euheterodonta;Imparidentia;Venerida;Veneroidea;Veneridae;Mercenaria;Mercenaria mercenaria
UdeM00000009281 29159 259.6 UdeM00000009281 XP_034309752.1 49.1 269 133 3 2 266 689 957 4.30E-66 259.6 29159 cellular organisms;Eukaryota;Opisthokonta;Metazoa;Eumetazoa;Bilateria;Protostomia;Spiralia;Lophotrochozoa;Mollusca;Bivalvia;Autobranchia;Pteriomorphia;Ostreida;Ostreoidea;Ostreidae;Crassostrea;Crassostrea gigas
UdeM00000009280 6565 224.6 UdeM00000009280 XP_022306954.1 40.3 303 144 6 165 435 651 948 2.50E-55 224.6 6565 cellular organisms;Eukaryota;Opisthokonta;Metazoa;Eumetazoa;Bilateria;Protostomia;Spiralia;Lophotrochozoa;Mollusca;Bivalvia;Autobranchia;Pteriomorphia;Ostreida;Ostreoidea;Ostreidae;Crassostrea;Crassostrea virginica
UdeM00000009280 6565 120.2 UdeM00000009280 XP_022306954.1 33.5 227 118 4 1 214 4 210 6.70E-24 120.2 6565 cellular organisms;Eukaryota;Opisthokonta;Metazoa;Eumetazoa;Bilateria;Protostomia;Spiralia;Lophotrochozoa;Mollusca;Bivalvia;Autobranchia;Pteriomorphia;Ostreida;Ostreoidea;Ostreidae;Crassostrea;Crassostrea virginica
UdeM00000009283 29159 194.5 UdeM00000009283 XP_034315421.1 51.9 206 93 3 135 336 271 474 2.20E-46 194.5 29159 cellular organisms;Eukaryota;Opisthokonta;Metazoa;Eumetazoa;Bilateria;Protostomia;Spiralia;Lophotrochozoa;Mollusca;Bivalvia;Autobranchia;Pteriomorphia;Ostreida;Ostreoidea;Ostreidae;Crassostrea;Crassostrea gigas

UdeM00000009287	6565	559.3	UdeM00000009287	XP_022306197.1	47	698
	314	14	9	681	16	682	6.70E-156	559.3	6565	cellular
organisms;Eukaryota;Opisthokonta;Metazoa;Eumetazoa;Bilateria;Protostomia;
Spiralia;Lophotrochozoa;Mollusca;Bivalvia;Autobranchia;Pteriomorphia;Ostr
eida;Ostreoidea;Ostreidae;Crassostrea;Crassostrea virginica
UdeM00000009288	6596	152.9	UdeM00000009288	XP_045161746.1	88.8	89
	10	0	48	136	47	135	2.90E-34	152.9	6596	cellular
organisms;Eukaryota;Opisthokonta;Metazoa;Eumetazoa;Bilateria;Protostomia;
Spiralia;Lophotrochozoa;Mollusca;Bivalvia;Autobranchia;Heteroconchia;Euhe
terodonta;Imparidentia;Venerida;Veneroidea;Veneridae;Mercenaria;Mercenari
a mercenaria
UdeM00000009290	1735272	86.3	UdeM00000009290	XP_041366951.1	44.4
	81	45	0	44	124	163	243	3.50E-14	86.3	1735272
	cellular
organisms;Eukaryota;Opisthokonta;Metazoa;Eumetazoa;Bilateria;Protostomia;
Spiralia;Lophotrochozoa;Mollusca;Gastropoda;Neomphalina;Neomphaloidea;Pel
tospiridae;Gigantopelta;Gigantopelta aegis
UdeM00000009291	1735272	158.3	UdeM00000009291	XP_041362472.1	35.8
	243	150	2	224	462	3	243	2.40E-35	158.3	1735272
	cellular
organisms;Eukaryota;Opisthokonta;Metazoa;Eumetazoa;Bilateria;Protostomia;
Spiralia;Lophotrochozoa;Mollusca;Gastropoda;Neomphalina;Neomphaloidea;Pel
tospiridae;Gigantopelta;Gigantopelta aegis
UdeM00000009292	400727	819.3	UdeM00000009292	XP_025081017.1	93.9
	441	25	1	1	441	1	439	2.30E-234	819.3	400727
	cellular
organisms;Eukaryota;Opisthokonta;Metazoa;Eumetazoa;Bilateria;Protostomia;
Spiralia;Lophotrochozoa;Mollusca;Gastropoda;Caenogastropoda;Architaeniogl
ossa;Ampullarioidea;Ampullariidae;Pomacea;Pomacea canaliculata
UdeM00000009293	1735272	333.2	UdeM00000009293	XP_041377542.1	84.1
	176	28	0	4	179	6	181	2.10E-88	333.2	1735272
	cellular
organisms;Eukaryota;Opisthokonta;Metazoa;Eumetazoa;Bilateria;Protostomia;
Spiralia;Lophotrochozoa;Mollusca;Gastropoda;Neomphalina;Neomphaloidea;Pel
tospiridae;Gigantopelta;Gigantopelta aegis
UdeM00000009295	1735272	188	UdeM00000009295	XP_041377542.1	82.8
	99	17	0	8	106	83	181	6.40E-45	188	1735272
	cellular
organisms;Eukaryota;Opisthokonta;Metazoa;Eumetazoa;Bilateria;Protostomia;
Spiralia;Lophotrochozoa;Mollusca;Gastropoda;Neomphalina;Neomphaloidea;Pel
tospiridae;Gigantopelta;Gigantopelta aegis
UdeM00000009294	6579	127.1	UdeM00000009294	XP_033741736.1	58.6	99
	41	0	24	122	796	894	1.70E-26	127.1	6579	cellular
organisms;Eukaryota;Opisthokonta;Metazoa;Eumetazoa;Bilateria;Protostomia;
Spiralia;Lophotrochozoa;Mollusca;Bivalvia;Autobranchia;Pteriomorphia;Pect
inida;Pectinoidea;Pectinidae;Pecten;Pecten maximus
UdeM00000009297	6596	329.3	UdeM00000009297	XP_045161674.1	89.2	158
	17	0	1	158	180	337	2.70E-87	329.3	6596	cellular
organisms;Eukaryota;Opisthokonta;Metazoa;Eumetazoa;Bilateria;Protostomia;
Spiralia;Lophotrochozoa;Mollusca;Bivalvia;Autobranchia;Heteroconchia;Euhe
terodonta;Imparidentia;Venerida;Veneroidea;Veneridae;Mercenaria;Mercenari
a mercenaria
UdeM00000009296	6596	369	UdeM00000009296	XP_045161675.1	87.8	181
	22	0	67	247	127	307	4.70E-99	369	6596	cellular

organisms;Eukaryota;Opisthokonta;Metazoa;Eumetazoa;Bilateria;Protostomia;
Spiralia;Lophotrochozoa;Mollusca;Bivalvia;Autobranchia;Heteroconchia;Euheterodonta;Imparidentia;Venerida;Veneroidea;Veneridae;Mercenaria;Mercenaria mercenaria
UdeM00000009298 6573 89.4 UdeM00000009298 XP_021366544.1 47.3 93
49 0 6 98 5 97 2.90E-15 89.4 6573 cellular
organisms;Eukaryota;Opisthokonta;Metazoa;Eumetazoa;Bilateria;Protostomia;
Spiralia;Lophotrochozoa;Mollusca;Bivalvia;Autobranchia;Pteriomorphia;Pectinida;Pectinoidea;Pectinidae;Mizuhopecten;Mizuhopecten yessoensis
UdeM00000009299 2652724 139.4 UdeM00000009299 XP_028513771.1 36.2
243 123 4 1 243 73 283 6.70E-30 139.4 2652724
cellular
organisms;Eukaryota;Opisthokonta;Metazoa;Eumetazoa;Cnidaria;Anthozoa;Hexacorallia;Actiniaria;Aiptasiidae;Exaiptasia;Exaiptasia diaphana
UdeM00000009300 6579 228 UdeM00000009300 XP_033724701.1 50.5 202
100 0 313 514 134 335 2.70E-56 228 6579 cellular
organisms;Eukaryota;Opisthokonta;Metazoa;Eumetazoa;Bilateria;Protostomia;
Spiralia;Lophotrochozoa;Mollusca;Bivalvia;Autobranchia;Pteriomorphia;Pectinida;Pectinoidea;Pectinidae;Pecten;Pecten maximus
UdeM00000009301 6596 404.1 UdeM00000009301 XP_045163841.1 58.9 355
137 3 16 364 7 358 2.00E-109 404.1 6596 cellular
organisms;Eukaryota;Opisthokonta;Metazoa;Eumetazoa;Bilateria;Protostomia;
Spiralia;Lophotrochozoa;Mollusca;Bivalvia;Autobranchia;Heteroconchia;Euheterodonta;Imparidentia;Venerida;Veneroidea;Veneridae;Mercenaria;Mercenaria mercenaria
UdeM00000009302 6579 639.8 UdeM00000009302 XP_033741736.1 55.5 535
226 4 82 610 347 875 3.70E-180 639.8 6579 cellular
organisms;Eukaryota;Opisthokonta;Metazoa;Eumetazoa;Bilateria;Protostomia;
Spiralia;Lophotrochozoa;Mollusca;Bivalvia;Autobranchia;Pteriomorphia;Pectinida;Pectinoidea;Pectinidae;Pecten;Pecten maximus
UdeM00000009303 6579 292.7 UdeM00000009303 XP_033744278.1 53.4 247
114 1 1 246 726 972 4.40E-76 292.7 6579 cellular
organisms;Eukaryota;Opisthokonta;Metazoa;Eumetazoa;Bilateria;Protostomia;
Spiralia;Lophotrochozoa;Mollusca;Bivalvia;Autobranchia;Pteriomorphia;Pectinida;Pectinoidea;Pectinidae;Pecten;Pecten maximus
UdeM00000009304 6579 211.5 UdeM00000009304 XP_033763899.1 40.1 374
202 9 3 368 299 658 2.10E-51 211.5 6579 cellular
organisms;Eukaryota;Opisthokonta;Metazoa;Eumetazoa;Bilateria;Protostomia;
Spiralia;Lophotrochozoa;Mollusca;Bivalvia;Autobranchia;Pteriomorphia;Pectinida;Pectinoidea;Pectinidae;Pecten;Pecten maximus
UdeM00000009305 6596 161.8 UdeM00000009305 XP_045162477.1 50.6 247
101 7 2 245 296 524 1.20E-36 161.8 6596 cellular
organisms;Eukaryota;Opisthokonta;Metazoa;Eumetazoa;Bilateria;Protostomia;
Spiralia;Lophotrochozoa;Mollusca;Bivalvia;Autobranchia;Heteroconchia;Euheterodonta;Imparidentia;Venerida;Veneroidea;Veneridae;Mercenaria;Mercenaria mercenaria
UdeM00000009306 6579 464.9 UdeM00000009306 XP_033763474.1 74.2 310
67 6 6 311 7 307 8.50E-128 464.9 6579 cellular
organisms;Eukaryota;Opisthokonta;Metazoa;Eumetazoa;Bilateria;Protostomia;
Spiralia;Lophotrochozoa;Mollusca;Bivalvia;Autobranchia;Pteriomorphia;Pectinida;Pectinoidea;Pectinidae;Pecten;Pecten maximus
UdeM00000009307 29159 132.1 UdeM00000009307 XP_034331441.1 41.4 181
103 3 1 181 1 178 7.60E-28 132.1 29159 cellular
organisms;Eukaryota;Opisthokonta;Metazoa;Eumetazoa;Bilateria;Protostomia;

Spiralia;Lophotrochozoa;Mollusca;Bivalvia;Autobranchia;Pteriomorphia;Ostreida;Ostreoidea;Ostreidae;Crassostrea;Crassostrea gigas
UdeM00000009309 6596 94.7 UdeM00000009309 XP_045163642.1 40 160 79 9 11 160 13 165 1.20E-16 94.7 6596 cellular organisms;Eukaryota;Opisthokonta;Metazoa;Eumetazoa;Bilateria;Protostomia;Spiralia;Lophotrochozoa;Mollusca;Bivalvia;Autobranchia;Heteroconchia;Euheterodonta;Imparidentia;Venerida;Veneroidea;Veneridae;Mercenaria;Mercenaria mercenaria
UdeM00000009310 6579 496.1 UdeM00000009310 XP_033731203.1 60.1 376 147 2 2 376 4 377 3.90E-137 496.1 6579 cellular organisms;Eukaryota;Opisthokonta;Metazoa;Eumetazoa;Bilateria;Protostomia;Spiralia;Lophotrochozoa;Mollusca;Bivalvia;Autobranchia;Pteriomorphia;Pectinida;Pectinoidea;Pectinidae;Pecten;Pecten maximus
UdeM00000008863 6573 201.8 UdeM00000008863 XP_021355224.1 36.4 341 163 7 9 340 17 312 2.80E-48 201.8 6573 cellular organisms;Eukaryota;Opisthokonta;Metazoa;Eumetazoa;Bilateria;Protostomia;Spiralia;Lophotrochozoa;Mollusca;Bivalvia;Autobranchia;Pteriomorphia;Pectinida;Pectinoidea;Pectinidae;Mizuhopecten;Mizuhopecten yessoensis
UdeM00000008864 6573 205.7 UdeM00000008864 XP_021355224.1 39.5 334 155 10 9 333 17 312 2.00E-49 205.7 6573 cellular organisms;Eukaryota;Opisthokonta;Metazoa;Eumetazoa;Bilateria;Protostomia;Spiralia;Lophotrochozoa;Mollusca;Bivalvia;Autobranchia;Pteriomorphia;Pectinida;Pectinoidea;Pectinidae;Mizuhopecten;Mizuhopecten yessoensis
UdeM00000008866 1735272 167.5 UdeM00000008866 XP_041361370.1 24 730 485 21 8 695 8 709 6.30E-38 167.5 1735272 cellular organisms;Eukaryota;Opisthokonta;Metazoa;Eumetazoa;Bilateria;Protostomia;Spiralia;Lophotrochozoa;Mollusca;Gastropoda;Neomphalina;Neomphaloidea;Peltospiridae;Gigantopelta;Gigantopelta aegis
UdeM00000008867 1735272 173.7 UdeM00000008867 XP_041361369.1 25.1 740 484 23 6 705 7 716 8.80E-40 173.7 1735272 cellular organisms;Eukaryota;Opisthokonta;Metazoa;Eumetazoa;Bilateria;Protostomia;Spiralia;Lophotrochozoa;Mollusca;Gastropoda;Neomphalina;Neomphaloidea;Peltospiridae;Gigantopelta;Gigantopelta aegis
UdeM00000008868 29159 146 UdeM00000008868 XP_034320422.1 75 84 21 0 46 129 141 224 3.70E-32 146 29159 cellular organisms;Eukaryota;Opisthokonta;Metazoa;Eumetazoa;Bilateria;Protostomia;Spiralia;Lophotrochozoa;Mollusca;Bivalvia;Autobranchia;Pteriomorphia;Ostreida;Ostreoidea;Ostreidae;Crassostrea;Crassostrea gigas
UdeM00000008869 6596 837 UdeM00000008869 XP_045209790.1 55 789 326 9 217 981 1 784 2.50E-239 837 6596 cellular organisms;Eukaryota;Opisthokonta;Metazoa;Eumetazoa;Bilateria;Protostomia;Spiralia;Lophotrochozoa;Mollusca;Bivalvia;Autobranchia;Heteroconchia;Euheterodonta;Imparidentia;Venerida;Veneroidea;Veneridae;Mercenaria;Mercenaria mercenaria
UdeM00000008872 6579 76.3 UdeM00000008872 XP_033744239.1 53.2 79 35 1 54 132 356 432 3.80E-11 76.3 6579 cellular organisms;Eukaryota;Opisthokonta;Metazoa;Eumetazoa;Bilateria;Protostomia;Spiralia;Lophotrochozoa;Mollusca;Bivalvia;Autobranchia;Pteriomorphia;Pectinida;Pectinoidea;Pectinidae;Pecten;Pecten maximus
UdeM00000008874 2652724 167.5 UdeM00000008874 XP_028514361.1 58.1 117 49 0 10 126 790 906 1.20E-38 167.5 2652724 cellular

organisms;Eukaryota;Opisthokonta;Metazoa;Eumetazoa;Cnidaria;Anthozoa;Hexacorallia;Actiniaria;Aiptasiidae;Exaiptasia;Exaiptasia diaphana
UdeM00000008875 6596 234.6 UdeM00000008875 XP_045199866.1 46.1 228 122 1 4 231 1914 2140 1.40E-58 234.6 6596 cellular organisms;Eukaryota;Opisthokonta;Metazoa;Eumetazoa;Bilateria;Protostomia;Spiralia;Lophotrochozoa;Mollusca;Bivalvia;Autobranchia;Heteroconchia;Euheterodonta;Imparidentia;Venerida;Veneroidea;Veneridae;Mercenaria;Mercenaria mercenaria
UdeM00000008876 29159 85.1 UdeM00000008876 XP_011433261.2 54.1 74 34 0 52 125 1972 2045 7.20E-14 85.1 29159 cellular organisms;Eukaryota;Opisthokonta;Metazoa;Eumetazoa;Bilateria;Protostomia;Spiralia;Lophotrochozoa;Mollusca;Bivalvia;Autobranchia;Pteriomorphia;Ostreida;Ostreoidea;Ostreidae;Crassostrea;Crassostrea gigas
UdeM00000008877 1735272 342.4 UdeM00000008877 XP_041357551.1 39.5 433 258 3 1 433 41 469 8.20E-91 342.4 1735272 cellular organisms;Eukaryota;Opisthokonta;Metazoa;Eumetazoa;Bilateria;Protostomia;Spiralia;Lophotrochozoa;Mollusca;Gastropoda;Neomphalina;Neomphaloidea;Peltospiridae;Gigantopelta;Gigantopelta aegis
UdeM00000008878 6579 62 UdeM00000008878 XP_033725862.1 40.8 76 45 0 125 200 215 290 1.00E-06 62 6579 cellular organisms;Eukaryota;Opisthokonta;Metazoa;Eumetazoa;Bilateria;Protostomia;Spiralia;Lophotrochozoa;Mollusca;Bivalvia;Autobranchia;Pteriomorphia;Pectinida;Pectinoidea;Pectinidae;Pecten;Pecten maximus
UdeM00000008879 6596 1195.3 UdeM00000008879 XP_045157300.1 38.6 1584 912 31 197 1755 3 1551 0.00E+00 1195.3 6596 cellular organisms;Eukaryota;Opisthokonta;Metazoa;Eumetazoa;Bilateria;Protostomia;Spiralia;Lophotrochozoa;Mollusca;Bivalvia;Autobranchia;Heteroconchia;Euheterodonta;Imparidentia;Venerida;Veneroidea;Veneridae;Mercenaria;Mercenaria mercenaria
UdeM00000008880 6596 1129.4 UdeM00000008880 XP_045157300.1 37.4 1584 887 32 197 1710 3 1551 0.00E+00 1129.4 6596 cellular organisms;Eukaryota;Opisthokonta;Metazoa;Eumetazoa;Bilateria;Protostomia;Spiralia;Lophotrochozoa;Mollusca;Bivalvia;Autobranchia;Heteroconchia;Euheterodonta;Imparidentia;Venerida;Veneroidea;Veneridae;Mercenaria;Mercenaria mercenaria
UdeM00000008884 1735272 480.3 UdeM00000008884 XP_041357551.1 39.3 616 368 5 14 629 25 634 3.70E-132 480.3 1735272 cellular organisms;Eukaryota;Opisthokonta;Metazoa;Eumetazoa;Bilateria;Protostomia;Spiralia;Lophotrochozoa;Mollusca;Gastropoda;Neomphalina;Neomphaloidea;Peltospiridae;Gigantopelta;Gigantopelta aegis
UdeM00000008882 1735272 481.5 UdeM00000008882 XP_041357551.1 39.1 632 375 6 11 642 13 634 1.70E-132 481.5 1735272 cellular organisms;Eukaryota;Opisthokonta;Metazoa;Eumetazoa;Bilateria;Protostomia;Spiralia;Lophotrochozoa;Mollusca;Gastropoda;Neomphalina;Neomphaloidea;Peltospiridae;Gigantopelta;Gigantopelta aegis
UdeM00000008883 1735272 481.5 UdeM00000008883 XP_041357551.1 39.1 632 375 6 11 642 13 634 1.70E-132 481.5 1735272 cellular organisms;Eukaryota;Opisthokonta;Metazoa;Eumetazoa;Bilateria;Protostomia;

Spiralia;Lophotrochozoa;Mollusca;Gastropoda;Neomphalina;Neomphaloidea;Peltospiridae;Gigantopelta;Gigantopelta aegis
UdeM00000008887 6579 807 UdeM00000008887 XP_033724854.1 38 1321 695 26 34 1272 59 1337 3.70E-230 807 6579 cellular organisms;Eukaryota;Opisthokonta;Metazoa;Eumetazoa;Bilateria;Protostomia;Spiralia;Lophotrochozoa;Mollusca;Bivalvia;Autobranchia;Pteriomorphia;Pectinida;Pectinoidea;Pectinidae;Pecten;Pecten maximus
UdeM00000008888 6579 685.6 UdeM00000008888 XP_033724854.1 32.8 1480 690 32 19 1402 54 1324 1.30E-193 685.6 6579 cellular organisms;Eukaryota;Opisthokonta;Metazoa;Eumetazoa;Bilateria;Protostomia;Spiralia;Lophotrochozoa;Mollusca;Bivalvia;Autobranchia;Pteriomorphia;Pectinida;Pectinoidea;Pectinidae;Pecten;Pecten maximus
UdeM00000008889 1735272 392.1 UdeM00000008889 XP_041371976.1 35.5 662 357 18 43 660 55 690 1.40E-105 392.1 1735272 cellular organisms;Eukaryota;Opisthokonta;Metazoa;Eumetazoa;Bilateria;Protostomia;Spiralia;Lophotrochozoa;Mollusca;Gastropoda;Neomphalina;Neomphaloidea;Peltospiridae;Gigantopelta;Gigantopelta aegis
UdeM00000008890 6596 241.9 UdeM00000008890 XP_045207155.1 51 296 115 5 471 762 145 414 2.70E-60 241.9 6596 cellular organisms;Eukaryota;Opisthokonta;Metazoa;Eumetazoa;Bilateria;Protostomia;Spiralia;Lophotrochozoa;Mollusca;Bivalvia;Autobranchia;Heteroconchia;Euheterodonta;Imparidentia;Venerida;Veneroidea;Veneridae;Mercenaria;Mercenaria mercenaria
UdeM00000008891 6596 78.6 UdeM00000008891 XP_045207155.1 62.9 62 23 0 91 152 414 475 1.00E-11 78.6 6596 cellular organisms;Eukaryota;Opisthokonta;Metazoa;Eumetazoa;Bilateria;Protostomia;Spiralia;Lophotrochozoa;Mollusca;Bivalvia;Autobranchia;Heteroconchia;Euheterodonta;Imparidentia;Venerida;Veneroidea;Veneridae;Mercenaria;Mercenaria mercenaria
UdeM00000008892 6573 335.1 UdeM00000008892 XP_021368827.1 44.2 387 190 9 22 387 11 392 1.20E-88 335.1 6573 cellular organisms;Eukaryota;Opisthokonta;Metazoa;Eumetazoa;Bilateria;Protostomia;Spiralia;Lophotrochozoa;Mollusca;Bivalvia;Autobranchia;Pteriomorphia;Pectinida;Pectinoidea;Pectinidae;Mizuhopecten;Mizuhopecten yessoensis
UdeM00000008893 6565 401.4 UdeM00000008893 XP_022344684.1 50.6 389 191 1 12 399 19 407 1.50E-108 401.4 6565 cellular organisms;Eukaryota;Opisthokonta;Metazoa;Eumetazoa;Bilateria;Protostomia;Spiralia;Lophotrochozoa;Mollusca;Bivalvia;Autobranchia;Pteriomorphia;Ostreida;Ostreoidea;Ostreidae;Crassostrea;Crassostrea virginica
UdeM00000008894 6565 343.6 UdeM00000008894 XP_022344684.1 42.9 399 190 5 10 373 19 414 3.70E-91 343.6 6565 cellular organisms;Eukaryota;Opisthokonta;Metazoa;Eumetazoa;Bilateria;Protostomia;Spiralia;Lophotrochozoa;Mollusca;Bivalvia;Autobranchia;Pteriomorphia;Ostreida;Ostreoidea;Ostreidae;Crassostrea;Crassostrea virginica
UdeM00000008895 6579 141.7 UdeM00000008895 XP_033733231.1 29.9 395 257 10 53 434 73 460 3.20E-30 141.7 6579 cellular organisms;Eukaryota;Opisthokonta;Metazoa;Eumetazoa;Bilateria;Protostomia;Spiralia;Lophotrochozoa;Mollusca;Bivalvia;Autobranchia;Pteriomorphia;Pectinida;Pectinoidea;Pectinidae;Pecten;Pecten maximus
UdeM00000008896 6596 167.9 UdeM00000008896 XP_045207757.1 50 168 82 2 8 174 19 185 1.20E-38 167.9 6596 cellular organisms;Eukaryota;Opisthokonta;Metazoa;Eumetazoa;Bilateria;Protostomia;Spiralia;Lophotrochozoa;Mollusca;Bivalvia;Autobranchia;Heteroconchia;Euhe

terodonta;Imparidentia;Venerida;Veneroidea;Veneridae;Mercenaria;Mercenaria mercenaria
UdeM00000008897 6596 452.2 UdeM00000008897 XP_045208451.1 65.6 314 105 1 4 314 3 316 5.40E-124 452.2 6596 cellular organisms;Eukaryota;Opisthokonta;Metazoa;Eumetazoa;Bilateria;Protostomia;Spiralia;Lophotrochozoa;Mollusca;Bivalvia;Autobranchia;Heteroconchia;Euheterodonta;Imparidentia;Venerida;Veneroidea;Veneridae;Mercenaria;Mercenaria mercenaria
UdeM00000008898 6579 620.2 UdeM00000008898 XP_033732800.1 50.1 609 290 9 50 652 41 641 3.10E-174 620.2 6579 cellular organisms;Eukaryota;Opisthokonta;Metazoa;Eumetazoa;Bilateria;Protostomia;Spiralia;Lophotrochozoa;Mollusca;Bivalvia;Autobranchia;Pteriomorphia;Pectinida;Pectinoidea;Pectinidae;Pecten;Pecten maximus
UdeM00000008899 6596 181.8 UdeM00000008899 XP_045162238.1 29 341 225 5 2 336 141 470 1.50E-42 181.8 6596 cellular organisms;Eukaryota;Opisthokonta;Metazoa;Eumetazoa;Bilateria;Protostomia;Spiralia;Lophotrochozoa;Mollusca;Bivalvia;Autobranchia;Heteroconchia;Euheterodonta;Imparidentia;Venerida;Veneroidea;Veneridae;Mercenaria;Mercenaria mercenaria
UdeM00000008900 6596 782.3 UdeM00000008900 XP_045208328.1 34.2 1432 766 28 19 1369 12 1348 1.00E-222 782.3 6596 cellular organisms;Eukaryota;Opisthokonta;Metazoa;Eumetazoa;Bilateria;Protostomia;Spiralia;Lophotrochozoa;Mollusca;Bivalvia;Autobranchia;Heteroconchia;Euheterodonta;Imparidentia;Venerida;Veneroidea;Veneridae;Mercenaria;Mercenaria mercenaria
UdeM00000008901 6573 66.6 UdeM00000008901 XP_021364034.1 38.2 68 42 0 3 70 1219 1286 1.60E-08 66.6 6573 cellular organisms;Eukaryota;Opisthokonta;Metazoa;Eumetazoa;Bilateria;Protostomia;Spiralia;Lophotrochozoa;Mollusca;Bivalvia;Autobranchia;Pteriomorphia;Pectinida;Pectinoidea;Pectinidae;Mizuhopecten;Mizuhopecten yessoensis
UdeM00000008902 6565 284.3 UdeM00000008902 XP_022341886.1 75.4 179 42 1 3 179 2 180 1.10E-73 284.3 6565 cellular organisms;Eukaryota;Opisthokonta;Metazoa;Eumetazoa;Bilateria;Protostomia;Spiralia;Lophotrochozoa;Mollusca;Bivalvia;Autobranchia;Pteriomorphia;Ostreida;Ostreoidea;Ostreidae;Crassostrea;Crassostrea virginica
UdeM00000008903 6596 586.6 UdeM00000008903 XP_045210366.1 51.5 623 261 12 20 621 19 621 3.60E-164 586.6 6596 cellular organisms;Eukaryota;Opisthokonta;Metazoa;Eumetazoa;Bilateria;Protostomia;Spiralia;Lophotrochozoa;Mollusca;Bivalvia;Autobranchia;Heteroconchia;Euheterodonta;Imparidentia;Venerida;Veneroidea;Veneridae;Mercenaria;Mercenaria mercenaria
UdeM00000008905 6596 207.2 UdeM00000008905 XP_045208495.1 52.7 169 73 3 1 168 10 172 1.60E-50 207.2 6596 cellular organisms;Eukaryota;Opisthokonta;Metazoa;Eumetazoa;Bilateria;Protostomia;Spiralia;Lophotrochozoa;Mollusca;Bivalvia;Autobranchia;Heteroconchia;Euheterodonta;Imparidentia;Venerida;Veneroidea;Veneridae;Mercenaria;Mercenaria mercenaria
UdeM00000008906 400727 59.7 UdeM00000008906 XP_025082576.1 45.8 83 36 3 1 83 1 74 3.30E-06 59.7 400727 cellular organisms;Eukaryota;Opisthokonta;Metazoa;Eumetazoa;Bilateria;Protostomia;Spiralia;Lophotrochozoa;Mollusca;Gastropoda;Caenogastropoda;Architaenioglossa;Ampullarioidea;Ampullariidae;Pomacea;Pomacea canaliculata

UdeM00000008907 400727 780 UdeM00000008907 XP_025081089.1 65.4
584 196 2 20 603 321 898 2.20E-222 780 400727
cellular
organisms;Eukaryota;Opisthokonta;Metazoa;Eumetazoa;Bilateria;Protostomia;
Spiralia;Lophotrochozoa;Mollusca;Gastropoda;Caenogastropoda;Architaeniogl
ossa;Ampullarioidea;Ampullariidae;Pomacea;Pomacea canaliculata
UdeM00000008908 29159 104.8 UdeM00000008908 XP_011450739.2 70.1 67
20 0 1 67 844 910 4.50E-20 104.8 29159 cellular
organisms;Eukaryota;Opisthokonta;Metazoa;Eumetazoa;Bilateria;Protostomia;
Spiralia;Lophotrochozoa;Mollusca;Bivalvia;Autobranchia;Pteriomorphia;Ostr
eida;Ostreoidea;Ostreidae;Crassostrea;Crassostrea gigas
UdeM00000008909 6596 308.9 UdeM00000008909 XP_045207456.1 36.7 507
181 6 240 607 339 844 1.40E-80 308.9 6596 cellular
organisms;Eukaryota;Opisthokonta;Metazoa;Eumetazoa;Bilateria;Protostomia;
Spiralia;Lophotrochozoa;Mollusca;Bivalvia;Autobranchia;Heteroconchia;Euhe
terodonta;Imparidentia;Venerida;Veneroidea;Veneridae;Mercenaria;Mercenari
a mercenaria
UdeM00000008910 6596 469.9 UdeM00000008910 XP_045207456.1 47.3 791
354 17 45 792 75 845 6.20E-129 469.9 6596 cellular
organisms;Eukaryota;Opisthokonta;Metazoa;Eumetazoa;Bilateria;Protostomia;
Spiralia;Lophotrochozoa;Mollusca;Bivalvia;Autobranchia;Heteroconchia;Euhe
terodonta;Imparidentia;Venerida;Veneroidea;Veneridae;Mercenaria;Mercenari
a mercenaria
UdeM00000008911 6596 251.1 UdeM00000008911 XP_045207456.1 50.7 278
112 7 4 269 581 845 1.60E-63 251.1 6596 cellular
organisms;Eukaryota;Opisthokonta;Metazoa;Eumetazoa;Bilateria;Protostomia;
Spiralia;Lophotrochozoa;Mollusca;Bivalvia;Autobranchia;Heteroconchia;Euhe
terodonta;Imparidentia;Venerida;Veneroidea;Veneridae;Mercenaria;Mercenari
a mercenaria
UdeM00000008912 6573 67.4 UdeM00000008912 XP_021354703.1 26.3 175
95 2 21 162 17 190 2.20E-08 67.4 6573 cellular
organisms;Eukaryota;Opisthokonta;Metazoa;Eumetazoa;Bilateria;Protostomia;
Spiralia;Lophotrochozoa;Mollusca;Bivalvia;Autobranchia;Pteriomorphia;Pect
inida;Pectinoidea;Pectinidae;Mizuhopecten;Mizuhopecten yessoensis
UdeM00000008913 6596 870.2 UdeM00000008913 XP_045188050.1 46 1035
501 9 7 1022 421 1416 2.70E-249 870.2 6596 cellular
organisms;Eukaryota;Opisthokonta;Metazoa;Eumetazoa;Bilateria;Protostomia;
Spiralia;Lophotrochozoa;Mollusca;Bivalvia;Autobranchia;Heteroconchia;Euhe
terodonta;Imparidentia;Venerida;Veneroidea;Veneridae;Mercenaria;Mercenari
a mercenaria
UdeM00000008914 2607531 288.9 UdeM00000008914 XP_036359723.1 32.8
545 355 7 89 628 119 657 1.60E-74 288.9 2607531
cellular
organisms;Eukaryota;Opisthokonta;Metazoa;Eumetazoa;Bilateria;Protostomia;
Spiralia;Lophotrochozoa;Mollusca;Cephalopoda;Coleoidea;Octopodiformes;Oct
opoda;Incirrata;Octopodidae;Octopus;Octopus sinensis
UdeM00000008915 2607531 288.9 UdeM00000008915 XP_036359723.1 32.8
545 355 7 79 618 119 657 1.60E-74 288.9 2607531
cellular
organisms;Eukaryota;Opisthokonta;Metazoa;Eumetazoa;Bilateria;Protostomia;
Spiralia;Lophotrochozoa;Mollusca;Cephalopoda;Coleoidea;Octopodiformes;Oct
opoda;Incirrata;Octopodidae;Octopus;Octopus sinensis
UdeM00000008916 6596 184.1 UdeM00000008916 XP_045176613.1 46.2 199
100 5 53 247 147 342 2.30E-43 184.1 6596 cellular

organisms;Eukaryota;Opisthokonta;Metazoa;Eumetazoa;Bilateria;Protostomia;Spiralia;Lophotrochozoa;Mollusca;Bivalvia;Autobranchia;Heteroconchia;Euheterodonta;Imparidentia;Venerida;Veneroidea;Veneridae;Mercenaria;Mercenaria mercenaria
UdeM00000008917 6565 143.7 UdeM00000008917 XP_022344143.1 47.7 153 77 3 13 164 17 167 2.20E-31 143.7 6565 cellular organisms;Eukaryota;Opisthokonta;Metazoa;Eumetazoa;Bilateria;Protostomia;Spiralia;Lophotrochozoa;Mollusca;Bivalvia;Autobranchia;Pteriomorphia;Ostreida;Ostreoidea;Ostreidae;Crassostrea;Crassostrea virginica
UdeM00000008918 6596 332.8 UdeM00000008918 XP_045176613.1 48.6 346 168 8 3 343 4 344 5.20E-88 332.8 6596 cellular organisms;Eukaryota;Opisthokonta;Metazoa;Eumetazoa;Bilateria;Protostomia;Spiralia;Lophotrochozoa;Mollusca;Bivalvia;Autobranchia;Heteroconchia;Euheterodonta;Imparidentia;Venerida;Veneroidea;Veneridae;Mercenaria;Mercenaria mercenaria
UdeM00000008920 6596 64.7 UdeM00000008920 XP_045208681.1 34.5 142 76 5 14 141 5 143 1.40E-07 64.7 6596 cellular organisms;Eukaryota;Opisthokonta;Metazoa;Eumetazoa;Bilateria;Protostomia;Spiralia;Lophotrochozoa;Mollusca;Bivalvia;Autobranchia;Heteroconchia;Euheterodonta;Imparidentia;Venerida;Veneroidea;Veneridae;Mercenaria;Mercenaria mercenaria
UdeM00000008922 6596 117.9 UdeM00000008922 XP_045202576.1 55.6 108 47 1 1 107 87 194 1.40E-23 117.9 6596 cellular organisms;Eukaryota;Opisthokonta;Metazoa;Eumetazoa;Bilateria;Protostomia;Spiralia;Lophotrochozoa;Mollusca;Bivalvia;Autobranchia;Heteroconchia;Euheterodonta;Imparidentia;Venerida;Veneroidea;Veneridae;Mercenaria;Mercenaria mercenaria
UdeM00000008923 6596 101.3 UdeM00000008923 XP_045202346.1 36.2 152 93 2 6 156 16 164 1.20E-18 101.3 6596 cellular organisms;Eukaryota;Opisthokonta;Metazoa;Eumetazoa;Bilateria;Protostomia;Spiralia;Lophotrochozoa;Mollusca;Bivalvia;Autobranchia;Heteroconchia;Euheterodonta;Imparidentia;Venerida;Veneroidea;Veneridae;Mercenaria;Mercenaria mercenaria
UdeM00000008924 6596 65.9 UdeM00000008924 XP_045202346.1 50 54 27 0 2 55 94 147 3.50E-08 65.9 6596 cellular organisms;Eukaryota;Opisthokonta;Metazoa;Eumetazoa;Bilateria;Protostomia;Spiralia;Lophotrochozoa;Mollusca;Bivalvia;Autobranchia;Heteroconchia;Euheterodonta;Imparidentia;Venerida;Veneroidea;Veneridae;Mercenaria;Mercenaria mercenaria
UdeM00000008929 29159 452.2 UdeM00000008929 XP_034330911.1 48.8 461 217 8 8 456 13 466 7.80E-124 452.2 29159 cellular organisms;Eukaryota;Opisthokonta;Metazoa;Eumetazoa;Bilateria;Protostomia;Spiralia;Lophotrochozoa;Mollusca;Bivalvia;Autobranchia;Pteriomorphia;Ostreida;Ostreoidea;Ostreidae;Crassostrea;Crassostrea gigas
UdeM00000008930 6565 102.1 UdeM00000008930 XP_022322655.1 48.5 99 51 0 2 100 650 748 4.40E-19 102.1 6565 cellular organisms;Eukaryota;Opisthokonta;Metazoa;Eumetazoa;Bilateria;Protostomia;Spiralia;Lophotrochozoa;Mollusca;Bivalvia;Autobranchia;Pteriomorphia;Ostreida;Ostreoidea;Ostreidae;Crassostrea;Crassostrea virginica
UdeM00000008931 6565 144.8 UdeM00000008931 XP_022322655.1 35.8 246 103 5 1 194 417 659 1.20E-31 144.8 6565 cellular organisms;Eukaryota;Opisthokonta;Metazoa;Eumetazoa;Bilateria;Protostomia;Spiralia;Lophotrochozoa;Mollusca;Bivalvia;Autobranchia;Pteriomorphia;Ostreida;Ostreoidea;Ostreidae;Crassostrea;Crassostrea virginica

UdeM00000008932 29159 62 UdeM00000008932 XP_011426086.2 25.2 139
99 3 3 137 35 172 6.90E-07 62 29159 cellular
organisms;Eukaryota;Opisthokonta;Metazoa;Eumetazoa;Bilateria;Protostomia;
Spiralia;Lophotrochozoa;Mollusca;Bivalvia;Autobranchia;Pteriomorphia;Ostr
eida;Ostreoidea;Ostreidae;Crassostrea;Crassostrea gigas
UdeM00000008938 29159 59.7 UdeM00000008938 XP_011426086.2 26.7 135
94 3 3 133 35 168 4.50E-06 59.7 29159 cellular
organisms;Eukaryota;Opisthokonta;Metazoa;Eumetazoa;Bilateria;Protostomia;
Spiralia;Lophotrochozoa;Mollusca;Bivalvia;Autobranchia;Pteriomorphia;Ostr
eida;Ostreoidea;Ostreidae;Crassostrea;Crassostrea gigas
UdeM00000008940 29159 73.2 UdeM00000008940 XP_011426086.2 26.6 154
111 2 33 184 35 188 1.10E-09 73.2 29159 cellular
organisms;Eukaryota;Opisthokonta;Metazoa;Eumetazoa;Bilateria;Protostomia;
Spiralia;Lophotrochozoa;Mollusca;Bivalvia;Autobranchia;Pteriomorphia;Ostr
eida;Ostreoidea;Ostreidae;Crassostrea;Crassostrea gigas
UdeM00000008941 1735272 268.5 UdeM00000008941 XP_041357551.1 28.8
619 397 13 4 600 38 634 2.40E-68 268.5 1735272
cellular
organisms;Eukaryota;Opisthokonta;Metazoa;Eumetazoa;Bilateria;Protostomia;
Spiralia;Lophotrochozoa;Mollusca;Gastropoda;Neomphalina;Neomphaloidea;Pel
tospiridae;Gigantopelta;Gigantopelta aegis
UdeM00000008943 6573 94.7 UdeM00000008943 XP_021340830.1 43.8 121
61 3 4 119 1929 2047 8.90E-17 94.7 6573 cellular
organisms;Eukaryota;Opisthokonta;Metazoa;Eumetazoa;Bilateria;Protostomia;
Spiralia;Lophotrochozoa;Mollusca;Bivalvia;Autobranchia;Pteriomorphia;Pect
inida;Pectinoidea;Pectinidae;Mizuhopecten;Mizuhopecten yessoensis
UdeM00000008946 29159 73.9 UdeM00000008946 XP_011426086.2 22.8 311
200 11 6 298 35 323 4.00E-10 73.9 29159 cellular
organisms;Eukaryota;Opisthokonta;Metazoa;Eumetazoa;Bilateria;Protostomia;
Spiralia;Lophotrochozoa;Mollusca;Bivalvia;Autobranchia;Pteriomorphia;Ostr
eida;Ostreoidea;Ostreidae;Crassostrea;Crassostrea gigas
UdeM00000008947 412133 81.3 UdeM00000008947 XP_001580069.1 33.8
142 88 3 532 670 326 464 5.40E-12 81.3 412133
cellular
organisms;Eukaryota;Metamonada;Parabasalia;Trichomonadida;Trichomonadidae
;Trichomonas;Trichomonas vaginalis;Trichomonas vaginalis G3
UdeM00000008948 29159 216.1 UdeM00000008948 XP_011433261.2 42.1 252
140 4 336 581 1973 2224 1.40E-52 216.1 29159 cellular
organisms;Eukaryota;Opisthokonta;Metazoa;Eumetazoa;Bilateria;Protostomia;
Spiralia;Lophotrochozoa;Mollusca;Bivalvia;Autobranchia;Pteriomorphia;Ostr
eida;Ostreoidea;Ostreidae;Crassostrea;Crassostrea gigas
UdeM00000008949 1735272 260.4 UdeM00000008949 XP_041357551.1 29
601 404 13 7 596 46 634 6.60E-66 260.4 1735272
cellular
organisms;Eukaryota;Opisthokonta;Metazoa;Eumetazoa;Bilateria;Protostomia;
Spiralia;Lophotrochozoa;Mollusca;Gastropoda;Neomphalina;Neomphaloidea;Pel
tospiridae;Gigantopelta;Gigantopelta aegis
UdeM00000008950 6596 452.6 UdeM00000008950 XP_045168479.1 36.4 707
422 7 11 709 30 716 9.30E-124 452.6 6596 cellular
organisms;Eukaryota;Opisthokonta;Metazoa;Eumetazoa;Bilateria;Protostomia;
Spiralia;Lophotrochozoa;Mollusca;Bivalvia;Autobranchia;Heteroconchia;Euhe
terodonta;Imparidentia;Venerida;Veneroidea;Veneridae;Mercenaria;Mercenari
a mercenaria

UdeM00000008951 6565 69.7 UdeM00000008951 XP_022291841.1 39.8 83
50 0 52 134 53 135 7.20E-09 69.7 6565 cellular
organisms;Eukaryota;Opisthokonta;Metazoa;Eumetazoa;Bilateria;Protostomia;
Spiralia;Lophotrochozoa;Mollusca;Bivalvia;Autobranchia;Pteriomorphia;Ostr
eida;Ostreoidea;Ostreidae;Crassostrea;Crassostrea virginica
UdeM00000008952 6596 300.4 UdeM00000008952 XP_045168479.1 36.9 472
277 5 3 467 259 716 3.90E-78 300.4 6596 cellular
organisms;Eukaryota;Opisthokonta;Metazoa;Eumetazoa;Bilateria;Protostomia;
Spiralia;Lophotrochozoa;Mollusca;Bivalvia;Autobranchia;Heteroconchia;Euhe
terodonta;Imparidentia;Venerida;Veneroidea;Veneridae;Mercenaria;Mercenari
a mercenaria
UdeM00000008953 6596 84 UdeM00000008953 XP_045169256.1 29.2 185
120 4 5 185 3 180 2.30E-13 84 6596 cellular
organisms;Eukaryota;Opisthokonta;Metazoa;Eumetazoa;Bilateria;Protostomia;
Spiralia;Lophotrochozoa;Mollusca;Bivalvia;Autobranchia;Heteroconchia;Euhe
terodonta;Imparidentia;Venerida;Veneroidea;Veneridae;Mercenaria;Mercenari
a mercenaria
UdeM00000008954 6105 157.5 UdeM00000008954 XP_031551063.1 31.8 277
168 4 3 261 449 722 2.30E-35 157.5 6105 cellular
organisms;Eukaryota;Opisthokonta;Metazoa;Eumetazoa;Cnidaria;Anthozoa;Hexa
corallia;Actiniaria;Actiniidae;Actinia;Actinia tenebrosa
UdeM00000008955 6596 312 UdeM00000008955 XP_045168479.1 37.9 404
238 3 11 414 30 420 1.20E-81 312 6596 cellular
organisms;Eukaryota;Opisthokonta;Metazoa;Eumetazoa;Bilateria;Protostomia;
Spiralia;Lophotrochozoa;Mollusca;Bivalvia;Autobranchia;Heteroconchia;Euhe
terodonta;Imparidentia;Venerida;Veneroidea;Veneridae;Mercenaria;Mercenari
a mercenaria
UdeM00000008956 6596 463.4 UdeM00000008956 XP_045168479.1 37.1 715
423 6 3 710 22 716 5.30E-127 463.4 6596 cellular
organisms;Eukaryota;Opisthokonta;Metazoa;Eumetazoa;Bilateria;Protostomia;
Spiralia;Lophotrochozoa;Mollusca;Bivalvia;Autobranchia;Heteroconchia;Euhe
terodonta;Imparidentia;Venerida;Veneroidea;Veneridae;Mercenaria;Mercenari
a mercenaria
UdeM00000008957 6105 116.7 UdeM00000008957 XP_031551063.1 41 139
74 1 34 164 584 722 2.90E-23 116.7 6105 cellular
organisms;Eukaryota;Opisthokonta;Metazoa;Eumetazoa;Cnidaria;Anthozoa;Hexa
corallia;Actiniaria;Actiniidae;Actinia;Actinia tenebrosa
UdeM00000008958 6596 134.4 UdeM00000008958 XP_045168479.1 30.9 223
153 1 43 265 298 519 2.10E-28 134.4 6596 cellular
organisms;Eukaryota;Opisthokonta;Metazoa;Eumetazoa;Bilateria;Protostomia;
Spiralia;Lophotrochozoa;Mollusca;Bivalvia;Autobranchia;Heteroconchia;Euhe
terodonta;Imparidentia;Venerida;Veneroidea;Veneridae;Mercenaria;Mercenari
a mercenaria
UdeM00000008959 6596 88.2 UdeM00000008959 XP_045168478.1 36.8 133
68 3 39 171 103 219 1.10E-14 88.2 6596 cellular
organisms;Eukaryota;Opisthokonta;Metazoa;Eumetazoa;Bilateria;Protostomia;
Spiralia;Lophotrochozoa;Mollusca;Bivalvia;Autobranchia;Heteroconchia;Euhe
terodonta;Imparidentia;Venerida;Veneroidea;Veneridae;Mercenaria;Mercenari
a mercenaria
UdeM00000008961 6596 75.1 UdeM00000008961 XP_045168479.1 42.5 80
46 0 55 134 583 662 8.00E-11 75.1 6596 cellular
organisms;Eukaryota;Opisthokonta;Metazoa;Eumetazoa;Bilateria;Protostomia;
Spiralia;Lophotrochozoa;Mollusca;Bivalvia;Autobranchia;Heteroconchia;Euhe

terodonta;Imparidentia;Venerida;Veneroidea;Veneridae;Mercenaria;Mercenaria mercenaria
UdeM00000008962 6105 125.9 UdeM00000008962 XP_031551063.1 46.9 130 61 1 1 122 591 720 3.60E-26 125.9 6105 cellular organisms;Eukaryota;Opisthokonta;Metazoa;Eumetazoa;Cnidaria;Anthozoa;Hexacorallia;Actiniaria;Actiniidae;Actinia;Actinia tenebrosa
UdeM00000008963 6596 287.7 UdeM00000008963 XP_045168479.1 40.6 342 202 1 1 342 170 510 1.90E-74 287.7 6596 cellular organisms;Eukaryota;Opisthokonta;Metazoa;Eumetazoa;Bilateria;Protostomia;Spiralia;Lophotrochozoa;Mollusca;Bivalvia;Autobranchia;Heteroconchia;Euheterodonta;Imparidentia;Venerida;Veneroidea;Veneridae;Mercenaria;Mercenaria mercenaria
UdeM00000008964 6596 58.2 UdeM00000008964 XP_045169256.1 31.3 99 68 0 3 101 4 102 7.40E-06 58.2 6596 cellular organisms;Eukaryota;Opisthokonta;Metazoa;Eumetazoa;Bilateria;Protostomia;Spiralia;Lophotrochozoa;Mollusca;Bivalvia;Autobranchia;Heteroconchia;Euheterodonta;Imparidentia;Venerida;Veneroidea;Veneridae;Mercenaria;Mercenaria mercenaria
UdeM00000008965 6596 457.2 UdeM00000008965 XP_045168479.1 34.7 709 443 6 11 719 30 718 3.80E-125 457.2 6596 cellular organisms;Eukaryota;Opisthokonta;Metazoa;Eumetazoa;Bilateria;Protostomia;Spiralia;Lophotrochozoa;Mollusca;Bivalvia;Autobranchia;Heteroconchia;Euheterodonta;Imparidentia;Venerida;Veneroidea;Veneridae;Mercenaria;Mercenaria mercenaria
UdeM00000008966 6596 458 UdeM00000008966 XP_045168479.1 35.1 713 439 7 11 723 30 718 2.30E-125 458 6596 cellular organisms;Eukaryota;Opisthokonta;Metazoa;Eumetazoa;Bilateria;Protostomia;Spiralia;Lophotrochozoa;Mollusca;Bivalvia;Autobranchia;Heteroconchia;Euheterodonta;Imparidentia;Venerida;Veneroidea;Veneridae;Mercenaria;Mercenaria mercenaria
UdeM00000008967 7654 176.4 UdeM00000008967 XP_041483378.1 27.7 415 274 5 1120 1524 532 930 2.80E-40 176.4 7654 cellular organisms;Eukaryota;Opisthokonta;Metazoa;Eumetazoa;Bilateria;Deuterostomia;Echinodermata;Eleutherozoa;Echinozoa;Echinoidea;Euechinoidea;Echinacea;Temnopleuroida;Toxopneustidae;Lytechinus;Lytechinus variegatus
UdeM00000008968 6596 483 UdeM00000008968 XP_045208579.1 48.9 483 245 1 16 498 16 496 4.60E-133 483 6596 cellular organisms;Eukaryota;Opisthokonta;Metazoa;Eumetazoa;Bilateria;Protostomia;Spiralia;Lophotrochozoa;Mollusca;Bivalvia;Autobranchia;Heteroconchia;Euheterodonta;Imparidentia;Venerida;Veneroidea;Veneridae;Mercenaria;Mercenaria mercenaria
UdeM00000008969 37653 256.1 UdeM00000008969 XP_014770564.1 90 140 14 0 1 140 1 140 2.50E-65 256.1 37653 cellular organisms;Eukaryota;Opisthokonta;Metazoa;Eumetazoa;Bilateria;Protostomia;Spiralia;Lophotrochozoa;Mollusca;Cephalopoda;Coleoidea;Octopodiformes;Octopoda;Incirrata;Octopodidae;Octopus;Octopus bimaculoides
UdeM00000008970 6596 220.3 UdeM00000008970 XP_045209851.1 72.5 138 38 0 2 139 4 141 1.80E-54 220.3 6596 cellular organisms;Eukaryota;Opisthokonta;Metazoa;Eumetazoa;Bilateria;Protostomia;Spiralia;Lophotrochozoa;Mollusca;Bivalvia;Autobranchia;Heteroconchia;Euheterodonta;Imparidentia;Venerida;Veneroidea;Veneridae;Mercenaria;Mercenaria mercenaria
UdeM00000008971 6596 198.7 UdeM00000008971 XP_045209839.1 62 229 70 7 21 232 187 415 7.90E-48 198.7 6596 cellular

organisms;Eukaryota;Opisthokonta;Metazoa;Eumetazoa;Bilateria;Protostomia;
Spiralia;Lophotrochozoa;Mollusca;Bivalvia;Autobranchia;Heteroconchia;Euheterodonta;Imparidentia;Venerida;Veneroidea;Veneridae;Mercenaria;Mercenaria mercenaria
UdeM00000008972 7574 84.7 UdeM00000008972 XP_013401704.1 42.9 84 43 1 17 95 748 831 7.10E-14 84.7 7574 cellular organisms;Eukaryota;Opisthokonta;Metazoa;Eumetazoa;Bilateria;Protostomia;
Spiralia;Lophotrochozoa;Brachiopoda;Linguliformea;Lingulata;Lingulida;Linguloidea;Lingulidae;Lingula;Lingula anatina
UdeM00000008973 6565 155.2 UdeM00000008973 XP_022290947.1 43.1 197 110 2 5 200 26 221 8.60E-35 155.2 6565 cellular organisms;Eukaryota;Opisthokonta;Metazoa;Eumetazoa;Bilateria;Protostomia;
Spiralia;Lophotrochozoa;Mollusca;Bivalvia;Autobranchia;Pteriomorphia;Ostreida;Ostreoidea;Ostreidae;Crassostrea;Crassostrea virginica
UdeM00000008974 1735272 127.1 UdeM00000008974 XP_041372244.1 39.1 156 95 0 6 161 6 161 2.10E-26 127.1 1735272 cellular organisms;Eukaryota;Opisthokonta;Metazoa;Eumetazoa;Bilateria;Protostomia;
Spiralia;Lophotrochozoa;Mollusca;Gastropoda;Neomphalina;Neomphaloidea;Peltospiridae;Gigantopelta;Gigantopelta aegis
UdeM00000008975 6596 439.1 UdeM00000008975 XP_045210465.1 70.7 294 79 2 51 339 37 328 5.10E-120 439.1 6596 cellular organisms;Eukaryota;Opisthokonta;Metazoa;Eumetazoa;Bilateria;Protostomia;
Spiralia;Lophotrochozoa;Mollusca;Bivalvia;Autobranchia;Heteroconchia;Euheterodonta;Imparidentia;Venerida;Veneroidea;Veneridae;Mercenaria;Mercenaria mercenaria
UdeM00000008976 6596 192.6 UdeM00000008976 XP_045213108.1 38.3 371 190 11 18 359 11 371 8.90E-46 192.6 6596 cellular organisms;Eukaryota;Opisthokonta;Metazoa;Eumetazoa;Bilateria;Protostomia;
Spiralia;Lophotrochozoa;Mollusca;Bivalvia;Autobranchia;Heteroconchia;Euheterodonta;Imparidentia;Venerida;Veneroidea;Veneridae;Mercenaria;Mercenaria mercenaria
UdeM00000008977 6596 481.9 UdeM00000008977 XP_045208245.1 78.3 290 62 1 1 289 1 290 5.90E-133 481.9 6596 cellular organisms;Eukaryota;Opisthokonta;Metazoa;Eumetazoa;Bilateria;Protostomia;
Spiralia;Lophotrochozoa;Mollusca;Bivalvia;Autobranchia;Heteroconchia;Euheterodonta;Imparidentia;Venerida;Veneroidea;Veneridae;Mercenaria;Mercenaria mercenaria
UdeM00000008978 6596 870.5 UdeM00000008978 XP_045209202.1 37.9 1243 689 28 374 1559 631 1847 3.30E-249 870.5 6596 cellular organisms;Eukaryota;Opisthokonta;Metazoa;Eumetazoa;Bilateria;Protostomia;
Spiralia;Lophotrochozoa;Mollusca;Bivalvia;Autobranchia;Heteroconchia;Euheterodonta;Imparidentia;Venerida;Veneroidea;Veneridae;Mercenaria;Mercenaria mercenaria
UdeM00000008979 6596 134.8 UdeM00000008979 XP_045189898.1 29.2 332 214 8 537 861 2017 2334 5.60E-28 134.8 6596 cellular organisms;Eukaryota;Opisthokonta;Metazoa;Eumetazoa;Bilateria;Protostomia;
Spiralia;Lophotrochozoa;Mollusca;Bivalvia;Autobranchia;Heteroconchia;Euheterodonta;Imparidentia;Venerida;Veneroidea;Veneridae;Mercenaria;Mercenaria mercenaria
UdeM00000008980 6596 597 UdeM00000008980 XP_045209202.1 32.9 1084 582 23 22 983 631 1691 4.20E-167 597 6596 cellular organisms;Eukaryota;Opisthokonta;Metazoa;Eumetazoa;Bilateria;Protostomia;
Spiralia;Lophotrochozoa;Mollusca;Bivalvia;Autobranchia;Heteroconchia;Euhe

terodonta;Imparidentia;Venerida;Veneroidea;Veneridae;Mercenaria;Mercenari
a mercenaria
UdeM00000008981 6596 78.6 UdeM00000008981 XP_045208001.1 31 142
97 1 261 401 2204 2345 2.10E-11 78.6 6596 cellular
organisms;Eukaryota;Opisthokonta;Metazoa;Eumetazoa;Bilateria;Protostomia;
Spiralia;Lophotrochozoa;Mollusca;Bivalvia;Autobranchia;Heteroconchia;Euhe
terodonta;Imparidentia;Venerida;Veneroidea;Veneridae;Mercenaria;Mercenari
a mercenaria
UdeM00000008982 6596 201.8 UdeM00000008982 XP_045207645.1 33 352
95 5 299 510 774 1124 3.30E-48 201.8 6596 cellular
organisms;Eukaryota;Opisthokonta;Metazoa;Eumetazoa;Bilateria;Protostomia;
Spiralia;Lophotrochozoa;Mollusca;Bivalvia;Autobranchia;Heteroconchia;Euhe
terodonta;Imparidentia;Venerida;Veneroidea;Veneridae;Mercenaria;Mercenari
a mercenaria
UdeM00000008983 6596 894.4 UdeM00000008983 XP_045189898.1 35.2 1409
857 22 376 1750 289 1675 3.80E-256 894.4 6596 cellular
organisms;Eukaryota;Opisthokonta;Metazoa;Eumetazoa;Bilateria;Protostomia;
Spiralia;Lophotrochozoa;Mollusca;Bivalvia;Autobranchia;Heteroconchia;Euhe
terodonta;Imparidentia;Venerida;Veneroidea;Veneridae;Mercenaria;Mercenari
a mercenaria
UdeM00000008984 6596 187.6 UdeM00000008984 XP_045209202.1 25.3 479
163 7 206 490 1275 1752 5.30E-44 187.6 6596 cellular
organisms;Eukaryota;Opisthokonta;Metazoa;Eumetazoa;Bilateria;Protostomia;
Spiralia;Lophotrochozoa;Mollusca;Bivalvia;Autobranchia;Heteroconchia;Euhe
terodonta;Imparidentia;Venerida;Veneroidea;Veneridae;Mercenaria;Mercenari
a mercenaria
UdeM00000008985 6500 291.2 UdeM00000008985 XP_035826907.1 42.6 385
212 5 1 383 300 677 2.30E-75 291.2 6500 cellular
organisms;Eukaryota;Opisthokonta;Metazoa;Eumetazoa;Bilateria;Protostomia;
Spiralia;Lophotrochozoa;Mollusca;Gastropoda;Heterobranchia;Euthyneura;Tec
tipleura;Aplysiida;Aplysioidea;Aplysiidae;Aplysia;Aplysia californica
UdeM00000008986 1735272 287.7 UdeM00000008986 XP_041361403.1 44.5
346 178 8 11 348 23 362 2.00E-74 287.7 1735272
cellular
organisms;Eukaryota;Opisthokonta;Metazoa;Eumetazoa;Bilateria;Protostomia;
Spiralia;Lophotrochozoa;Mollusca;Gastropoda;Neomphalina;Neomphaloidea;Pel
tospiridae;Gigantopelta;Gigantopelta aegis
UdeM00000008987 1529436 298.9 UdeM00000008987 XP_033103324.1 43.5
372 200 6 1 366 12 379 1.30E-77 298.9 1529436
cellular
organisms;Eukaryota;Opisthokonta;Metazoa;Eumetazoa;Bilateria;Deuterostomi
a;Echinodermata;Pelmatozoa;Crinoidea;Articulata;Comatulida;Comatulidae;Co
matulinae;Anneissia;Anneissia japonica
UdeM00000008988 225164 120.9 UdeM00000008988 XP_009052905.1 80.9
68 13 0 30 97 284 351 8.80E-25 120.9 225164
cellular
organisms;Eukaryota;Opisthokonta;Metazoa;Eumetazoa;Bilateria;Protostomia;
Spiralia;Lophotrochozoa;Mollusca;Gastropoda;Patellogastropoda;Lottioidea;
Lottiidae;Lottia;Lottia gigantea
UdeM00000008989 1735272 257.7 UdeM00000008989 XP_041347279.1 34.2
430 248 9 1 400 1 425 2.80E-65 257.7 1735272
cellular
organisms;Eukaryota;Opisthokonta;Metazoa;Eumetazoa;Bilateria;Protostomia;

Spiralia;Lophotrochozoa;Mollusca;Gastropoda;Neomphalina;Neomphaloidea;Peltospiridae;Gigantopelta;Gigantopelta aegis
UdeM00000008990 6573 65.5 UdeM00000008990 XP_021368863.1 71.4 42 12 0 1 42 41 82 7.40E-08 65.5 6573 cellular organisms;Eukaryota;Opisthokonta;Metazoa;Eumetazoa;Bilateria;Protostomia;Spiralia;Lophotrochozoa;Mollusca;Bivalvia;Autobranchia;Pteriomorphia;Pectinida;Pectinoidea;Pectinidae;Mizuhopecten;Mizuhopecten yessoensis
UdeM00000008991 1529436 304.7 UdeM00000008991 XP_033103324.1 43.5 379 201 7 3 373 6 379 2.50E-79 304.7 1529436 cellular organisms;Eukaryota;Opisthokonta;Metazoa;Eumetazoa;Bilateria;Deuterostomia;Echinodermata;Pelmatozoa;Crinoidea;Articulata;Comatulida;Comatulidae;Comatulinae;Anneissia;Anneissia japonica
UdeM00000008992 6596 168.3 UdeM00000008992 XP_045209201.1 34.3 271 138 5 318 549 4008 4277 3.20E-38 168.3 6596 cellular organisms;Eukaryota;Opisthokonta;Metazoa;Eumetazoa;Bilateria;Protostomia;Spiralia;Lophotrochozoa;Mollusca;Bivalvia;Autobranchia;Heteroconchia;Euheterodonta;Imparidentia;Venerida;Veneroidea;Veneridae;Mercenaria;Mercenaria mercenaria
UdeM00000008995 6596 147.5 UdeM00000008995 XP_045207645.1 30.9 265 177 4 3 264 1812 2073 3.50E-32 147.5 6596 cellular organisms;Eukaryota;Opisthokonta;Metazoa;Eumetazoa;Bilateria;Protostomia;Spiralia;Lophotrochozoa;Mollusca;Bivalvia;Autobranchia;Heteroconchia;Euheterodonta;Imparidentia;Venerida;Veneroidea;Veneridae;Mercenaria;Mercenaria mercenaria
UdeM00000008996 225164 588.2 UdeM00000008996 XP_009052905.1 78.6 355 72 1 1 355 1 351 7.10E-165 588.2 225164 cellular organisms;Eukaryota;Opisthokonta;Metazoa;Eumetazoa;Bilateria;Protostomia;Spiralia;Lophotrochozoa;Mollusca;Gastropoda;Patellogastropoda;Lottioidea;Lottiidae;Lottia;Lottia gigantea
UdeM00000008997 1735272 284.6 UdeM00000008997 XP_041361403.1 42 357 193 7 7 354 11 362 1.70E-73 284.6 1735272 cellular organisms;Eukaryota;Opisthokonta;Metazoa;Eumetazoa;Bilateria;Protostomia;Spiralia;Lophotrochozoa;Mollusca;Gastropoda;Neomphalina;Neomphaloidea;Peltospiridae;Gigantopelta;Gigantopelta aegis
UdeM00000008998 1735272 302 UdeM00000008998 XP_041361403.1 47.5 337 169 6 3 337 47 377 1.30E-78 302 1735272 cellular organisms;Eukaryota;Opisthokonta;Metazoa;Eumetazoa;Bilateria;Protostomia;Spiralia;Lophotrochozoa;Mollusca;Gastropoda;Neomphalina;Neomphaloidea;Peltospiridae;Gigantopelta;Gigantopelta aegis
UdeM00000008999 225164 596.3 UdeM00000008999 XP_009052905.1 80.1 351 70 0 1 351 1 351 2.60E-167 596.3 225164 cellular organisms;Eukaryota;Opisthokonta;Metazoa;Eumetazoa;Bilateria;Protostomia;Spiralia;Lophotrochozoa;Mollusca;Gastropoda;Patellogastropoda;Lottioidea;Lottiidae;Lottia;Lottia gigantea
UdeM00000009000 6596 727.2 UdeM00000009000 XP_045208088.1 64 586 177 6 4 576 99 663 1.60E-206 727.2 6596 cellular organisms;Eukaryota;Opisthokonta;Metazoa;Eumetazoa;Bilateria;Protostomia;Spiralia;Lophotrochozoa;Mollusca;Bivalvia;Autobranchia;Heteroconchia;Euhe

terodonta;Imparidentia;Venerida;Veneroidea;Veneridae;Mercenaria;Mercenaria mercenaria
UdeM00000009001 6596 132.5 UdeM00000009001 XP_045163868.1 50.7 136 60 2 4 132 3 138 4.00E-28 132.5 6596 cellular organisms;Eukaryota;Opisthokonta;Metazoa;Eumetazoa;Bilateria;Protostomia;Spiralia;Lophotrochozoa;Mollusca;Bivalvia;Autobranchia;Heteroconchia;Euheterodonta;Imparidentia;Venerida;Veneroidea;Veneridae;Mercenaria;Mercenaria mercenaria
UdeM00000009005 6596 466.5 UdeM00000009005 XP_045207527.1 79.5 292 59 1 8 299 12 302 2.70E-128 466.5 6596 cellular organisms;Eukaryota;Opisthokonta;Metazoa;Eumetazoa;Bilateria;Protostomia;Spiralia;Lophotrochozoa;Mollusca;Bivalvia;Autobranchia;Heteroconchia;Euheterodonta;Imparidentia;Venerida;Veneroidea;Veneridae;Mercenaria;Mercenaria mercenaria
UdeM00000009006 6596 401.4 UdeM00000009006 XP_045207527.1 67.2 302 92 3 5 300 8 308 1.00E-108 401.4 6596 cellular organisms;Eukaryota;Opisthokonta;Metazoa;Eumetazoa;Bilateria;Protostomia;Spiralia;Lophotrochozoa;Mollusca;Bivalvia;Autobranchia;Heteroconchia;Euheterodonta;Imparidentia;Venerida;Veneroidea;Veneridae;Mercenaria;Mercenaria mercenaria
UdeM00000009007 6596 94.4 UdeM00000009007 XP_045188729.1 25.5 424 254 14 29 404 1085 1494 3.80E-16 94.4 6596 cellular organisms;Eukaryota;Opisthokonta;Metazoa;Eumetazoa;Bilateria;Protostomia;Spiralia;Lophotrochozoa;Mollusca;Bivalvia;Autobranchia;Heteroconchia;Euheterodonta;Imparidentia;Venerida;Veneroidea;Veneridae;Mercenaria;Mercenaria mercenaria
UdeM00000009008 7574 80.5 UdeM00000009008 XP_013384015.1 51.1 94 45 1 6 99 186 278 1.50E-12 80.5 7574 cellular organisms;Eukaryota;Opisthokonta;Metazoa;Eumetazoa;Bilateria;Protostomia;Spiralia;Lophotrochozoa;Brachiopoda;Linguliformea;Lingulata;Lingulida;Linguloidea;Lingulidae;Lingula;Lingula anatina
UdeM00000009009 9545 96.3 UdeM00000009009 XP_011720398.1 24.3 412 269 13 17 414 1000 1382 9.90E-17 96.3 9545 cellular organisms;Eukaryota;Opisthokonta;Metazoa;Eumetazoa;Bilateria;Deuterostomia;Chordata;Craniata;Vertebrata;Gnathostomata;Teleostomi;Euteleostomi;Sarcopterygii;Dipnotetrapodomorpha;Tetrapoda;Amniota;Mammalia;Theria;Eutheria;Boreoeutheria;Euarchontoglires;Primates;Haplorrhini;Simiiformes;Catarrhini;Cercopithecoidea;Cercopithecidae;Cercopithecinae;Macaca;Macaca nemestrina
UdeM00000009010 6596 302.4 UdeM00000009010 XP_045209334.1 52.3 325 130 7 4 322 9 314 7.00E-79 302.4 6596 cellular organisms;Eukaryota;Opisthokonta;Metazoa;Eumetazoa;Bilateria;Protostomia;Spiralia;Lophotrochozoa;Mollusca;Bivalvia;Autobranchia;Heteroconchia;Euheterodonta;Imparidentia;Venerida;Veneroidea;Veneridae;Mercenaria;Mercenaria mercenaria
UdeM00000009011 29159 586.3 UdeM00000009011 XP_011449002.2 61.6 445 171 0 1 445 1 445 3.40E-164 586.3 29159 cellular organisms;Eukaryota;Opisthokonta;Metazoa;Eumetazoa;Bilateria;Protostomia;Spiralia;Lophotrochozoa;Mollusca;Bivalvia;Autobranchia;Pteriomorphia;Ostreida;Ostreoidea;Ostreidae;Crassostrea;Crassostrea gigas
UdeM00000009012 6596 169.5 UdeM00000009012 XP_045210186.1 64.2 120 39 2 34 153 55 170 4.10E-39 169.5 6596 cellular organisms;Eukaryota;Opisthokonta;Metazoa;Eumetazoa;Bilateria;Protostomia;Spiralia;Lophotrochozoa;Mollusca;Bivalvia;Autobranchia;Heteroconchia;Euhe

terodonta;Imparidentia;Venerida;Veneroidea;Veneridae;Mercenaria;Mercenari
a mercenaria
UdeM00000009013 6579 340.1 UdeM00000009013 XP_033732980.1 51.7 360
140 10 1 336 1 350 3.20E-90 340.1 6579 cellular
organisms;Eukaryota;Opisthokonta;Metazoa;Eumetazoa;Bilateria;Protostomia;
Spiralia;Lophotrochozoa;Mollusca;Bivalvia;Autobranchia;Pteriomorphia;Pect
inida;Pectinoidea;Pectinidae;Pecten;Pecten maximus
UdeM00000009014 6579 709.9 UdeM00000009014 XP_033729842.1 65.3 519
178 1 7 525 8 524 2.40E-201 709.9 6579 cellular
organisms;Eukaryota;Opisthokonta;Metazoa;Eumetazoa;Bilateria;Protostomia;
Spiralia;Lophotrochozoa;Mollusca;Bivalvia;Autobranchia;Pteriomorphia;Pect
inida;Pectinoidea;Pectinidae;Pecten;Pecten maximus
UdeM00000009015 6596 482.3 UdeM00000009015 XP_045208023.1 49.9 537
257 5 1 529 1 533 8.30E-133 482.3 6596 cellular
organisms;Eukaryota;Opisthokonta;Metazoa;Eumetazoa;Bilateria;Protostomia;
Spiralia;Lophotrochozoa;Mollusca;Bivalvia;Autobranchia;Heteroconchia;Euhe
terodonta;Imparidentia;Venerida;Veneroidea;Veneridae;Mercenaria;Mercenari
a mercenaria
UdeM00000009017 7574 79.3 UdeM00000009017 XP_013405563.1 34.7 144
81 1 2 145 247 377 4.50E-12 79.3 7574 cellular
organisms;Eukaryota;Opisthokonta;Metazoa;Eumetazoa;Bilateria;Protostomia;
Spiralia;Lophotrochozoa;Brachiopoda;Linguliformea;Lingulata;Lingulida;Lin
guloidea;Lingulidae;Lingula;Lingula anatina
UdeM00000009018 6596 345.5 UdeM00000009018 XP_045170511.1 31 670
402 18 365 995 281 929 2.60E-91 345.5 6596 cellular
organisms;Eukaryota;Opisthokonta;Metazoa;Eumetazoa;Bilateria;Protostomia;
Spiralia;Lophotrochozoa;Mollusca;Bivalvia;Autobranchia;Heteroconchia;Euhe
terodonta;Imparidentia;Venerida;Veneroidea;Veneridae;Mercenaria;Mercenari
a mercenaria
UdeM00000009022 6596 80.5 UdeM00000009022 XP_045208558.1 28.6 238
149 10 14 245 63 285 4.60E-12 80.5 6596 cellular
organisms;Eukaryota;Opisthokonta;Metazoa;Eumetazoa;Bilateria;Protostomia;
Spiralia;Lophotrochozoa;Mollusca;Bivalvia;Autobranchia;Heteroconchia;Euhe
terodonta;Imparidentia;Venerida;Veneroidea;Veneridae;Mercenaria;Mercenari
a mercenaria
UdeM00000009023 218467 61.6 UdeM00000009023 XP_023231487.1 27.8
180 119 4 297 468 928 1104 3.50E-06 61.6 218467
cellular
organisms;Eukaryota;Opisthokonta;Metazoa;Eumetazoa;Bilateria;Protostomia;
Ecdysozoa;Panarthropoda;Arthropoda;Chelicerata;Arachnida;Scorpiones;Buthi
da;Buthoidea;Buthidae;Centruroides;Centruroides sculpturatus
UdeM00000009024 218467 61.6 UdeM00000009024 XP_023231487.1 27.8
180 119 4 195 366 928 1104 2.80E-06 61.6 218467
cellular
organisms;Eukaryota;Opisthokonta;Metazoa;Eumetazoa;Bilateria;Protostomia;
Ecdysozoa;Panarthropoda;Arthropoda;Chelicerata;Arachnida;Scorpiones;Buthi
da;Buthoidea;Buthidae;Centruroides;Centruroides sculpturatus
UdeM00000009027 6596 72.4 UdeM00000009027 XP_045207213.1 28.1 146
83 5 46 188 594 720 7.60E-10 72.4 6596 cellular
organisms;Eukaryota;Opisthokonta;Metazoa;Eumetazoa;Bilateria;Protostomia;
Spiralia;Lophotrochozoa;Mollusca;Bivalvia;Autobranchia;Heteroconchia;Euhe
terodonta;Imparidentia;Venerida;Veneroidea;Veneridae;Mercenaria;Mercenari
a mercenaria

UdeM00000009028 6596 72.4 UdeM00000009028 XP_045207213.1 28.1 146
83 5 95 237 594 720 9.40E-10 72.4 6596 cellular
organisms;Eukaryota;Opisthokonta;Metazoa;Eumetazoa;Bilateria;Protostomia;
Spiralia;Lophotrochozoa;Mollusca;Bivalvia;Autobranchia;Heteroconchia;Euhe
terodonta;Imparidentia;Venerida;Veneroidea;Veneridae;Mercenaria;Mercenari
a mercenaria
UdeM00000009031 118141 65.9 UdeM00000009031 XP_036394471.1 30.2
149 88 4 159 299 908 1048 1.40E-07 65.9 118141
cellular
organisms;Eukaryota;Opisthokonta;Metazoa;Eumetazoa;Bilateria;Deuterostomi
a;Chordata;Craniata;Vertebrata;Gnathostomata;Teleostomi;Euteleostomi;Acti
nopterygii;Actinopteri;Neopterygii;Teleostei;Elopocephalai;Elopocephala;E
lopomorpha;Elopiformes;Megalopidae;Megalops;Megalops cyprinoides
UdeM00000009034 6596 77 UdeM00000009034 XP_045207213.1 21.6 306
212 9 46 348 594 874 9.50E-11 77 6596 cellular
organisms;Eukaryota;Opisthokonta;Metazoa;Eumetazoa;Bilateria;Protostomia;
Spiralia;Lophotrochozoa;Mollusca;Bivalvia;Autobranchia;Heteroconchia;Euhe
terodonta;Imparidentia;Venerida;Veneroidea;Veneridae;Mercenaria;Mercenari
a mercenaria
UdeM00000009035 29159 58.9 UdeM00000009035 XP_034322739.1 32.1 134
83 4 52 181 589 718 8.80E-06 58.9 29159 cellular
organisms;Eukaryota;Opisthokonta;Metazoa;Eumetazoa;Bilateria;Protostomia;
Spiralia;Lophotrochozoa;Mollusca;Bivalvia;Autobranchia;Pteriomorphia;Ostr
eida;Ostreoidea;Ostreidae;Crassostrea;Crassostrea gigas
UdeM00000009036 6596 100.1 UdeM00000009036 XP_045210115.1 27.4 266
154 11 46 294 328 571 7.90E-18 100.1 6596 cellular
organisms;Eukaryota;Opisthokonta;Metazoa;Eumetazoa;Bilateria;Protostomia;
Spiralia;Lophotrochozoa;Mollusca;Bivalvia;Autobranchia;Heteroconchia;Euhe
terodonta;Imparidentia;Venerida;Veneroidea;Veneridae;Mercenaria;Mercenari
a mercenaria
UdeM00000009039 6596 67 UdeM00000009039 XP_045208558.1 32.7 104
59 3 14 117 63 155 4.20E-08 67 6596 cellular
organisms;Eukaryota;Opisthokonta;Metazoa;Eumetazoa;Bilateria;Protostomia;
Spiralia;Lophotrochozoa;Mollusca;Bivalvia;Autobranchia;Heteroconchia;Euhe
terodonta;Imparidentia;Venerida;Veneroidea;Veneridae;Mercenaria;Mercenari
a mercenaria
UdeM00000009042 7957 63.2 UdeM00000009042 XP_026135560.1 33 103
64 2 50 152 202 299 4.60E-07 63.2 7957 cellular
organisms;Eukaryota;Opisthokonta;Metazoa;Eumetazoa;Bilateria;Deuterostomi
a;Chordata;Craniata;Vertebrata;Gnathostomata;Teleostomi;Euteleostomi;Acti
nopterygii;Actinopteri;Neopterygii;Teleostei;Osteoglossocephalai;Clupeoce
phala;Otomorpha;Ostariophysi;Otophysi;Cypriniphysae;Cypriniformes;Cyprino
idei;Cyprinidae;Cyprininae;Carassius;Carassius auratus
UdeM00000009046 6596 189.1 UdeM00000009046 XP_045183440.1 52.6 190
85 3 1 189 198 383 7.20E-45 189.1 6596 cellular
organisms;Eukaryota;Opisthokonta;Metazoa;Eumetazoa;Bilateria;Protostomia;
Spiralia;Lophotrochozoa;Mollusca;Bivalvia;Autobranchia;Heteroconchia;Euhe
terodonta;Imparidentia;Venerida;Veneroidea;Veneridae;Mercenaria;Mercenari
a mercenaria
UdeM00000009047 151771 120.9 UdeM00000009047 XP_028417951.1 43.2
139 78 1 43 181 7 144 1.60E-24 120.9 151771
cellular
organisms;Eukaryota;Opisthokonta;Metazoa;Eumetazoa;Cnidaria;Anthozoa;Octo

corallia;Alcyonacea;Alcyoniina;Nephtheidae;Dendronephthya;Dendronephthya gigantea
UdeM00000009050 6596 430.6 UdeM00000009050 XP_045208747.1 67.2 287 85 4 280 565 273 551 3.30E-117 430.6 6596 cellular organisms;Eukaryota;Opisthokonta;Metazoa;Eumetazoa;Bilateria;Protostomia;Spiralia;Lophotrochozoa;Mollusca;Bivalvia;Autobranchia;Heteroconchia;Euheterodonta;Imparidentia;Venerida;Veneroidea;Veneridae;Mercenaria;Mercenaria mercenaria
UdeM00000009051 6573 1038.9 UdeM00000009051 XP_021368837.1 34.7 6630 2801 131 103 5769 919 6983 2.70E-299 1038.9 6573 cellular organisms;Eukaryota;Opisthokonta;Metazoa;Eumetazoa;Bilateria;Protostomia;Spiralia;Lophotrochozoa;Mollusca;Bivalvia;Autobranchia;Pteriomorphia;Pectinida;Pectinoidea;Pectinidae;Mizuhopecten;Mizuhopecten yessoensis
UdeM00000009052 6579 668.7 UdeM00000009052 XP_033732984.1 74.8 420 103 3 5 423 6599 7016 4.90E-189 668.7 6579 cellular organisms;Eukaryota;Opisthokonta;Metazoa;Eumetazoa;Bilateria;Protostomia;Spiralia;Lophotrochozoa;Mollusca;Bivalvia;Autobranchia;Pteriomorphia;Pectinida;Pectinoidea;Pectinidae;Pecten;Pecten maximus
UdeM00000009053 6579 328.2 UdeM00000009053 XP_033733025.1 49 484 198 18 6 463 29 489 2.60E-86 328.2 6579 cellular organisms;Eukaryota;Opisthokonta;Metazoa;Eumetazoa;Bilateria;Protostomia;Spiralia;Lophotrochozoa;Mollusca;Bivalvia;Autobranchia;Pteriomorphia;Pectinida;Pectinoidea;Pectinidae;Pecten;Pecten maximus
UdeM00000009054 6579 329.3 UdeM00000009054 XP_033733025.1 49.3 483 197 18 6 462 29 489 1.20E-86 329.3 6579 cellular organisms;Eukaryota;Opisthokonta;Metazoa;Eumetazoa;Bilateria;Protostomia;Spiralia;Lophotrochozoa;Mollusca;Bivalvia;Autobranchia;Pteriomorphia;Pectinida;Pectinoidea;Pectinidae;Pecten;Pecten maximus
UdeM00000009055 6579 93.6 UdeM00000009055 XP_033733025.1 33 312 155 12 1107 1407 789 1057 2.20E-15 93.6 6579 cellular organisms;Eukaryota;Opisthokonta;Metazoa;Eumetazoa;Bilateria;Protostomia;Spiralia;Lophotrochozoa;Mollusca;Bivalvia;Autobranchia;Pteriomorphia;Pectinida;Pectinoidea;Pectinidae;Pecten;Pecten maximus
UdeM00000009056 6596 127.5 UdeM00000009056 XP_045202576.1 56.5 124 46 2 1 116 87 210 2.00E-26 127.5 6596 cellular organisms;Eukaryota;Opisthokonta;Metazoa;Eumetazoa;Bilateria;Protostomia;Spiralia;Lophotrochozoa;Mollusca;Bivalvia;Autobranchia;Heteroconchia;Euheterodonta;Imparidentia;Venerida;Veneroidea;Veneridae;Mercenaria;Mercenaria mercenaria
UdeM00000009057 6596 172.6 UdeM00000009057 XP_045206316.1 53.6 151 69 1 2 151 128 278 4.00E-40 172.6 6596 cellular organisms;Eukaryota;Opisthokonta;Metazoa;Eumetazoa;Bilateria;Protostomia;Spiralia;Lophotrochozoa;Mollusca;Bivalvia;Autobranchia;Heteroconchia;Euheterodonta;Imparidentia;Venerida;Veneroidea;Veneridae;Mercenaria;Mercenaria mercenaria
UdeM00000009059 90988 125.2 UdeM00000009059 XP_039535825.1 58.8 102 40 1 1 100 111 212 5.20E-26 125.2 90988 cellular organisms;Eukaryota;Opisthokonta;Metazoa;Eumetazoa;Bilateria;Deuterostomia;Chordata;Craniata;Vertebrata;Gnathostomata;Teleostomi;Euteleostomi;Actinopterygii;Actinopteri;Neopterygii;Teleostei;Osteoglossocephalai;Clupeocephala;Otomorpha;Ostariophysi;Otophysi;Cypriniphysae;Cypriniformes;Cyprinoidei;Leuciscidae;Pogonichthyinae;Pimephales;Pimephales promelas

UdeM00000009060 260615 300.1 UdeM00000009060 XP_039386738.1 88.9
180 18 1 1 178 347 526 1.90E-78 300.1 260615
cellular
organisms;Eukaryota;Opisthokonta;Metazoa;Eumetazoa;Bilateria;Deuterostomia;Chordata;Craniata;Vertebrata;Gnathostomata;Teleostomi;Euteleostomi;Sarcopterygii;Dipnotetrapodomorpha;Tetrapoda;Amniota;Sauropsida;Sauria;Archelosauria;Testudinata;Testudines;Cryptodira;Durocryptodira;Testudinoidea;Geoemydidae;Geoemydinae;Mauremys;Mauremys reevesii
UdeM00000009061 225164 404.4 UdeM00000009061 XP_009052844.1 63.2
323 118 1 39 360 25 347 1.50E-109 404.4 225164
cellular
organisms;Eukaryota;Opisthokonta;Metazoa;Eumetazoa;Bilateria;Protostomia;Spiralia;Lophotrochozoa;Mollusca;Gastropoda;Patellogastropoda;Lottioidea;Lottiidae;Lottia;Lottia gigantea
UdeM00000009062 70779 189.5 UdeM00000009062 XP_015749887.1 38.8 325
152 9 30 316 22 337 6.50E-45 189.5 70779 cellular
organisms;Eukaryota;Opisthokonta;Metazoa;Eumetazoa;Cnidaria;Anthozoa;Hexacorallia;Scleractinia;Astrocoeniina;Acroporidae;Acropora;Acropora digitifera
UdeM00000009063 27687 155.2 UdeM00000009063 XP_028657992.1 30.5 377
229 8 25 385 104 463 1.70E-34 155.2 27687 cellular
organisms;Eukaryota;Opisthokonta;Metazoa;Eumetazoa;Bilateria;Deuterostomia;Chordata;Craniata;Vertebrata;Gnathostomata;Teleostomi;Euteleostomi;Actinopterygii;Cladistia;Polypteriformes;Polypteridae;Erpetoichthys;Erpetoichthys calabaricus
UdeM00000009064 6596 1280.4 UdeM00000009064 XP_045157300.1 36.9
1988 1073 45 46 1898 6 1946 0.00E+00 1280.4 6596
cellular
organisms;Eukaryota;Opisthokonta;Metazoa;Eumetazoa;Bilateria;Protostomia;Spiralia;Lophotrochozoa;Mollusca;Bivalvia;Autobranchia;Heteroconchia;Euheterodonta;Imparidentia;Venerida;Veneroidea;Veneridae;Mercenaria;Mercenaria mercenaria
UdeM00000009065 7757 376.7 UdeM00000009065 XP_032835197.1 80.3 233
46 0 77 309 3 235 2.80E-101 376.7 7757 cellular
organisms;Eukaryota;Opisthokonta;Metazoa;Eumetazoa;Bilateria;Deuterostomia;Chordata;Craniata;Vertebrata;Cyclostomata;Hyperoartia;Petromyzontiformes;Petromyzontidae;Petromyzon;Petromyzon marinus
UdeM00000009066 7757 109.8 UdeM00000009066 XP_032835197.1 67.9 81
19 1 1 74 251 331 1.70E-21 109.8 7757 cellular
organisms;Eukaryota;Opisthokonta;Metazoa;Eumetazoa;Bilateria;Deuterostomia;Chordata;Craniata;Vertebrata;Cyclostomata;Hyperoartia;Petromyzontiformes;Petromyzontidae;Petromyzon;Petromyzon marinus
UdeM00000009067 1415580 170.6 UdeM00000009067 XP_030075314.1 46.7
214 96 3 1 211 427 625 2.10E-39 170.6 1415580
cellular
organisms;Eukaryota;Opisthokonta;Metazoa;Eumetazoa;Bilateria;Deuterostomia;Chordata;Craniata;Vertebrata;Gnathostomata;Teleostomi;Euteleostomi;Sarcopterygii;Dipnotetrapodomorpha;Tetrapoda;Amphibia;Gymnophiona;Siphonopidae;Microcaecilia;Microcaecilia unicolor
UdeM00000009068 6596 1310.8 UdeM00000009068 XP_045157300.1 35.7
2084 1107 39 97 2094 10 1946 0.00E+00 1310.8 6596
cellular
organisms;Eukaryota;Opisthokonta;Metazoa;Eumetazoa;Bilateria;Protostomia;Spiralia;Lophotrochozoa;Mollusca;Bivalvia;Autobranchia;Heteroconchia;Euhe

terodonta;Imparidentia;Venerida;Veneroidea;Veneridae;Mercenaria;Mercenaria mercenaria
UdeM00000009069 6596 418.7 UdeM00000009069 XP_045208069.1 35.9 647 383 15 105 733 369 1001 3.20E-113 418.7 6596 cellular organisms;Eukaryota;Opisthokonta;Metazoa;Eumetazoa;Bilateria;Protostomia;Spiralia;Lophotrochozoa;Mollusca;Bivalvia;Autobranchia;Heteroconchia;Euheterodonta;Imparidentia;Venerida;Veneroidea;Veneridae;Mercenaria;Mercenaria mercenaria
UdeM00000009070 6596 103.6 UdeM00000009070 XP_045209997.1 44.4 108 57 1 4 108 37 144 1.60E-19 103.6 6596 cellular organisms;Eukaryota;Opisthokonta;Metazoa;Eumetazoa;Bilateria;Protostomia;Spiralia;Lophotrochozoa;Mollusca;Bivalvia;Autobranchia;Heteroconchia;Euheterodonta;Imparidentia;Venerida;Veneroidea;Veneridae;Mercenaria;Mercenaria mercenaria
UdeM00000009071 29159 265.8 UdeM00000009071 XP_034316161.1 45 291 159 1 1 290 718 1008 6.60E-68 265.8 29159 cellular organisms;Eukaryota;Opisthokonta;Metazoa;Eumetazoa;Bilateria;Protostomia;Spiralia;Lophotrochozoa;Mollusca;Bivalvia;Autobranchia;Pteriomorphia;Ostreida;Ostreoidea;Ostreidae;Crassostrea;Crassostrea gigas
UdeM00000009072 6596 360.1 UdeM00000009072 XP_045208459.1 52.8 305 144 0 11 315 7 311 2.80E-96 360.1 6596 cellular organisms;Eukaryota;Opisthokonta;Metazoa;Eumetazoa;Bilateria;Protostomia;Spiralia;Lophotrochozoa;Mollusca;Bivalvia;Autobranchia;Heteroconchia;Euheterodonta;Imparidentia;Venerida;Veneroidea;Veneridae;Mercenaria;Mercenaria mercenaria
UdeM00000009073 400727 93.2 UdeM00000009073 XP_025084528.1 49.5 95 39 1 1 86 46 140 1.80E-16 93.2 400727 cellular organisms;Eukaryota;Opisthokonta;Metazoa;Eumetazoa;Bilateria;Protostomia;Spiralia;Lophotrochozoa;Mollusca;Gastropoda;Caenogastropoda;Architaenioglossa;Ampullarioidea;Ampullariidae;Pomacea;Pomacea canaliculata
UdeM00000009074 6596 413.3 UdeM00000009074 XP_045207043.1 67 291 93 2 12 302 307 594 2.70E-112 413.3 6596 cellular organisms;Eukaryota;Opisthokonta;Metazoa;Eumetazoa;Bilateria;Protostomia;Spiralia;Lophotrochozoa;Mollusca;Bivalvia;Autobranchia;Heteroconchia;Euheterodonta;Imparidentia;Venerida;Veneroidea;Veneridae;Mercenaria;Mercenaria mercenaria
UdeM00000009075 6579 402.9 UdeM00000009075 XP_033732370.1 69.6 293 87 2 1 291 1 293 3.60E-109 402.9 6579 cellular organisms;Eukaryota;Opisthokonta;Metazoa;Eumetazoa;Bilateria;Protostomia;Spiralia;Lophotrochozoa;Mollusca;Bivalvia;Autobranchia;Pteriomorphia;Pectinida;Pectinoidea;Pectinidae;Pecten;Pecten maximus
UdeM00000009077 6596 286.2 UdeM00000009077 XP_045210146.1 74 192 43 2 11 200 3 189 3.20E-74 286.2 6596 cellular organisms;Eukaryota;Opisthokonta;Metazoa;Eumetazoa;Bilateria;Protostomia;Spiralia;Lophotrochozoa;Mollusca;Bivalvia;Autobranchia;Heteroconchia;Euheterodonta;Imparidentia;Venerida;Veneroidea;Veneridae;Mercenaria;Mercenaria mercenaria
UdeM00000009078 6596 116.3 UdeM00000009078 XP_045210141.1 62.1 87 33 0 1 87 169 255 1.90E-23 116.3 6596 cellular organisms;Eukaryota;Opisthokonta;Metazoa;Eumetazoa;Bilateria;Protostomia;Spiralia;Lophotrochozoa;Mollusca;Bivalvia;Autobranchia;Heteroconchia;Euheterodonta;Imparidentia;Venerida;Veneroidea;Veneridae;Mercenaria;Mercenaria mercenaria

UdeM00000009080 6579 114.4 UdeM00000009080 XP_033733263.1 63.4 82
30 0 1 82 37 118 8.90E-23 114.4 6579 cellular
organisms;Eukaryota;Opisthokonta;Metazoa;Eumetazoa;Bilateria;Protostomia;
Spiralia;Lophotrochozoa;Mollusca;Bivalvia;Autobranchia;Pteriomorphia;Pect
inida;Pectinoidea;Pectinidae;Pecten;Pecten maximus
UdeM00000009081 6596 493.4 UdeM00000009081 XP_045207548.1 74.5 318
80 1 1 317 1 318 2.10E-136 493.4 6596 cellular
organisms;Eukaryota;Opisthokonta;Metazoa;Eumetazoa;Bilateria;Protostomia;
Spiralia;Lophotrochozoa;Mollusca;Bivalvia;Autobranchia;Heteroconchia;Euhe
terodonta;Imparidentia;Venerida;Veneroidea;Veneridae;Mercenaria;Mercenari
a mercenaria
UdeM00000009082 29159 105.1 UdeM00000009082 XP_019919120.2 24.2 400
274 11 20 412 46 423 2.20E-19 105.1 29159 cellular
organisms;Eukaryota;Opisthokonta;Metazoa;Eumetazoa;Bilateria;Protostomia;
Spiralia;Lophotrochozoa;Mollusca;Bivalvia;Autobranchia;Pteriomorphia;Ostr
eida;Ostreoidea;Ostreidae;Crassostrea;Crassostrea gigas
UdeM00000009083 2607531 101.7 UdeM00000009083 XP_029658399.1 30.2
192 126 4 67 255 104 290 1.50E-18 101.7 2607531
cellular
organisms;Eukaryota;Opisthokonta;Metazoa;Eumetazoa;Bilateria;Protostomia;
Spiralia;Lophotrochozoa;Mollusca;Cephalopoda;Coleoidea;Octopodiformes;Oct
opoda;Incirrata;Octopodidae;Octopus;Octopus sinensis
UdeM00000009087 6579 182.2 UdeM00000009087 XP_033729137.1 31.5 362
220 9 6 354 13 359 1.20E-42 182.2 6579 cellular
organisms;Eukaryota;Opisthokonta;Metazoa;Eumetazoa;Bilateria;Protostomia;
Spiralia;Lophotrochozoa;Mollusca;Bivalvia;Autobranchia;Pteriomorphia;Pect
inida;Pectinoidea;Pectinidae;Pecten;Pecten maximus
UdeM00000009088 225164 443 UdeM00000009088 XP_009063875.1 65
306 107 0 1 306 1 306 3.20E-121 443 225164
cellular
organisms;Eukaryota;Opisthokonta;Metazoa;Eumetazoa;Bilateria;Protostomia;
Spiralia;Lophotrochozoa;Mollusca;Gastropoda;Patellogastropoda;Lottioidea;
Lottiidae;Lottia;Lottia gigantea
UdeM00000009089 6596 278.1 UdeM00000009089 XP_045210361.1 76.2 164
39 0 4 167 3 166 7.90E-72 278.1 6596 cellular
organisms;Eukaryota;Opisthokonta;Metazoa;Eumetazoa;Bilateria;Protostomia;
Spiralia;Lophotrochozoa;Mollusca;Bivalvia;Autobranchia;Heteroconchia;Euhe
terodonta;Imparidentia;Venerida;Veneroidea;Veneridae;Mercenaria;Mercenari
a mercenaria
UdeM00000009090 6596 481.1 UdeM00000009090 XP_045208226.1 58.9 423
155 3 17 438 8 412 1.50E-132 481.1 6596 cellular
organisms;Eukaryota;Opisthokonta;Metazoa;Eumetazoa;Bilateria;Protostomia;
Spiralia;Lophotrochozoa;Mollusca;Bivalvia;Autobranchia;Heteroconchia;Euhe
terodonta;Imparidentia;Venerida;Veneroidea;Veneridae;Mercenaria;Mercenari
a mercenaria
UdeM00000009091 6500 200.7 UdeM00000009091 XP_012935509.1 43.6 250
127 7 1157 1405 1072 1308 1.30E-47 200.7 6500 cellular
organisms;Eukaryota;Opisthokonta;Metazoa;Eumetazoa;Bilateria;Protostomia;
Spiralia;Lophotrochozoa;Mollusca;Gastropoda;Heterobranchia;Euthyneura;Tec
tipleura;Aplysiida;Aplysioidea;Aplysiidae;Aplysia;Aplysia californica
UdeM00000009092 6573 658.7 UdeM00000009092 XP_021342727.1 59.4 493
199 1 5 497 32 523 6.00E-186 658.7 6573 cellular
organisms;Eukaryota;Opisthokonta;Metazoa;Eumetazoa;Bilateria;Protostomia;

Spiralia;Lophotrochozoa;Mollusca;Bivalvia;Autobranchia;Pteriomorphia;Pectinida;Pectinoidea;Pectinidae;Mizuhopecten;Mizuhopecten yessoensis
UdeM00000009093 6565 210.3 UdeM00000009093 XP_022341069.1 39.2 260 155 1 1186 1445 1181 1437 1.60E-50 210.3 6565 cellular organisms;Eukaryota;Opisthokonta;Metazoa;Eumetazoa;Bilateria;Protostomia;Spiralia;Lophotrochozoa;Mollusca;Bivalvia;Autobranchia;Pteriomorphia;Ostreida;Ostreoidea;Ostreidae;Crassostrea;Crassostrea virginica
UdeM00000009095 6579 228 UdeM00000009095 XP_033726761.1 62.9 197 69 2 1 193 7 203 1.00E-56 228 6579 cellular organisms;Eukaryota;Opisthokonta;Metazoa;Eumetazoa;Bilateria;Protostomia;Spiralia;Lophotrochozoa;Mollusca;Bivalvia;Autobranchia;Pteriomorphia;Pectinida;Pectinoidea;Pectinidae;Pecten;Pecten maximus
UdeM00000009094 6579 240.7 UdeM00000009094 XP_033726761.1 62.9 205 72 2 1 201 7 211 1.60E-60 240.7 6579 cellular organisms;Eukaryota;Opisthokonta;Metazoa;Eumetazoa;Bilateria;Protostomia;Spiralia;Lophotrochozoa;Mollusca;Bivalvia;Autobranchia;Pteriomorphia;Pectinida;Pectinoidea;Pectinidae;Pecten;Pecten maximus
UdeM00000009096 6596 904 UdeM00000009096 XP_045209739.1 77.4 636 140 4 1 633 1 635 1.00E-259 904 6596 cellular organisms;Eukaryota;Opisthokonta;Metazoa;Eumetazoa;Bilateria;Protostomia;Spiralia;Lophotrochozoa;Mollusca;Bivalvia;Autobranchia;Heteroconchia;Euheterodonta;Imparidentia;Venerida;Veneroidea;Veneridae;Mercenaria;Mercenaria mercenaria
UdeM00000009097 29159 215.7 UdeM00000009097 XP_011420009.2 24.6 670 451 19 21 668 16 653 1.80E-52 215.7 29159 cellular organisms;Eukaryota;Opisthokonta;Metazoa;Eumetazoa;Bilateria;Protostomia;Spiralia;Lophotrochozoa;Mollusca;Bivalvia;Autobranchia;Pteriomorphia;Ostreida;Ostreoidea;Ostreidae;Crassostrea;Crassostrea gigas
UdeM00000009098 6565 102.8 UdeM00000009098 XP_022321940.1 25.6 250 178 5 24 270 428 672 7.20E-19 102.8 6565 cellular organisms;Eukaryota;Opisthokonta;Metazoa;Eumetazoa;Bilateria;Protostomia;Spiralia;Lophotrochozoa;Mollusca;Bivalvia;Autobranchia;Pteriomorphia;Ostreida;Ostreoidea;Ostreidae;Crassostrea;Crassostrea virginica
UdeM00000009099 29159 64.3 UdeM00000009099 XP_034315424.1 36.1 108 60 3 12 116 213 314 1.40E-07 64.3 29159 cellular organisms;Eukaryota;Opisthokonta;Metazoa;Eumetazoa;Bilateria;Protostomia;Spiralia;Lophotrochozoa;Mollusca;Bivalvia;Autobranchia;Pteriomorphia;Ostreida;Ostreoidea;Ostreidae;Crassostrea;Crassostrea gigas
UdeM00000009100 1735272 330.5 UdeM00000009100 XP_041372048.1 54.8 301 127 3 9 303 10 307 2.80E-87 330.5 1735272 cellular organisms;Eukaryota;Opisthokonta;Metazoa;Eumetazoa;Bilateria;Protostomia;Spiralia;Lophotrochozoa;Mollusca;Gastropoda;Neomphalina;Neomphaloidea;Peltospiridae;Gigantopelta;Gigantopelta aegis
UdeM00000009102 1735272 86.3 UdeM00000009102 XP_041379412.1 40.6 106 62 1 29 133 11 116 3.50E-14 86.3 1735272 cellular organisms;Eukaryota;Opisthokonta;Metazoa;Eumetazoa;Bilateria;Protostomia;Spiralia;Lophotrochozoa;Mollusca;Gastropoda;Neomphalina;Neomphaloidea;Peltospiridae;Gigantopelta;Gigantopelta aegis
UdeM00000009103 8005 72.4 UdeM00000009103 XP_026860382.2 40.9 93 52 2 51 140 367 459 5.60E-10 72.4 8005 cellular organisms;Eukaryota;Opisthokonta;Metazoa;Eumetazoa;Bilateria;Deuterostomia;Chordata;Craniata;Vertebrata;Gnathostomata;Teleostomi;Euteleostomi;Acti

nopterygii;Actinopteri;Neopterygii;Teleostei;Osteoglossocephalai;Clupeocephala;Otomorpha;Ostariophysi;Otophysi;Characiphysae;Gymnotiformes;Gymnotoidei;Gymnotidae;Electrophorus;Electrophorus electricus
UdeM00000009104 6579 72.4 UdeM00000009104 XP_033732841.1 60 45 18 0 3 47 6 50 2.20E-09 72.4 6579 cellular organisms;Eukaryota;Opisthokonta;Metazoa;Eumetazoa;Bilateria;Protostomia;Spiralia;Lophotrochozoa;Mollusca;Bivalvia;Autobranchia;Pteriomorphia;Pectinida;Pectinoidea;Pectinidae;Pecten;Pecten maximus
UdeM00000009106 134920 132.5 UdeM00000009106 XP_037324962.1 55.8 113 49 1 4 116 133 244 3.70E-28 132.5 134920 cellular organisms;Eukaryota;Opisthokonta;Metazoa;Eumetazoa;Bilateria;Deuterostomia;Chordata;Craniata;Vertebrata;Gnathostomata;Teleostomi;Euteleostomi;Actinopterygii;Actinopteri;Neopterygii;Teleostei;Osteoglossocephalai;Clupeocephala;Euteleosteomorpha;Neoteleostei;Eurypterygia;Ctenosquamata;Acanthomorphata;Euacanthomorphacea;Percomorphaceae;Eupercaria;Perciformes;Cottioidei;Gasterosteales;Gasterosteidae;Pungitius;Pungitius pungitius
UdeM00000009107 260995 130.6 UdeM00000009107 XP_033809097.1 48.3 120 62 0 1 120 136 255 1.40E-27 130.6 260995 cellular organisms;Eukaryota;Opisthokonta;Metazoa;Eumetazoa;Bilateria;Deuterostomia;Chordata;Craniata;Vertebrata;Gnathostomata;Teleostomi;Euteleostomi;Sarcopterygii;Dipnotetrapodomorpha;Tetrapoda;Amphibia;Gymnophiona;Dermophiidae;Geotrypetes;Geotrypetes seraphini
UdeM00000009108 260995 130.6 UdeM00000009108 XP_033809097.1 48.3 120 62 0 1 120 136 255 1.40E-27 130.6 260995 cellular organisms;Eukaryota;Opisthokonta;Metazoa;Eumetazoa;Bilateria;Deuterostomia;Chordata;Craniata;Vertebrata;Gnathostomata;Teleostomi;Euteleostomi;Sarcopterygii;Dipnotetrapodomorpha;Tetrapoda;Amphibia;Gymnophiona;Dermophiidae;Geotrypetes;Geotrypetes seraphini
UdeM00000009109 6596 209.9 UdeM00000009109 XP_045208340.1 63.1 233 75 4 1 225 6 235 3.40E-51 209.9 6596 cellular organisms;Eukaryota;Opisthokonta;Metazoa;Eumetazoa;Bilateria;Protostomia;Spiralia;Lophotrochozoa;Mollusca;Bivalvia;Autobranchia;Heteroconchia;Euheterodonta;Imparidentia;Venerida;Veneroidea;Veneridae;Mercenaria;Mercenaria mercenaria
UdeM00000009110 6573 756.1 UdeM00000009110 XP_021352765.1 55.8 650 274 7 33 671 31 678 3.70E-215 756.1 6573 cellular organisms;Eukaryota;Opisthokonta;Metazoa;Eumetazoa;Bilateria;Protostomia;Spiralia;Lophotrochozoa;Mollusca;Bivalvia;Autobranchia;Pteriomorphia;Pectinida;Pectinoidea;Pectinidae;Mizuhopecten;Mizuhopecten yessoensis
UdeM00000009111 6596 422.9 UdeM00000009111 XP_045207726.1 62.2 328 115 1 10 337 9 327 3.70E-115 422.9 6596 cellular organisms;Eukaryota;Opisthokonta;Metazoa;Eumetazoa;Bilateria;Protostomia;Spiralia;Lophotrochozoa;Mollusca;Bivalvia;Autobranchia;Heteroconchia;Euheterodonta;Imparidentia;Venerida;Veneroidea;Veneridae;Mercenaria;Mercenaria mercenaria
UdeM00000009112 6596 423.3 UdeM00000009112 XP_045207726.1 62.2 328 115 1 7 334 9 327 2.80E-115 423.3 6596 cellular organisms;Eukaryota;Opisthokonta;Metazoa;Eumetazoa;Bilateria;Protostomia;Spiralia;Lophotrochozoa;Mollusca;Bivalvia;Autobranchia;Heteroconchia;Euheterodonta;Imparidentia;Venerida;Veneroidea;Veneridae;Mercenaria;Mercenaria mercenaria

UdeM00000009113 6596 367.1 UdeM00000009113 XP_045208156.1 77.7 211
47 0 10 220 6 216 1.60E-98 367.1 6596 cellular
organisms;Eukaryota;Opisthokonta;Metazoa;Eumetazoa;Bilateria;Protostomia;
Spiralia;Lophotrochozoa;Mollusca;Bivalvia;Autobranchia;Heteroconchia;Euheterodonta;Imparidentia;Venerida;Veneroidea;Veneridae;Mercenaria;Mercenaria mercenaria
UdeM00000009115 29159 93.6 UdeM00000009115 XP_011453423.2 35.6 101
64 1 1 100 714 814 1.60E-16 93.6 29159 cellular
organisms;Eukaryota;Opisthokonta;Metazoa;Eumetazoa;Bilateria;Protostomia;
Spiralia;Lophotrochozoa;Mollusca;Bivalvia;Autobranchia;Pteriomorphia;Ostreida;Ostreoidea;Ostreidae;Crassostrea;Crassostrea gigas
UdeM00000009116 6573 84.3 UdeM00000009116 XP_021374089.1 30.3 132
58 3 50 148 548 678 1.40E-13 84.3 6573 cellular
organisms;Eukaryota;Opisthokonta;Metazoa;Eumetazoa;Bilateria;Protostomia;
Spiralia;Lophotrochozoa;Mollusca;Bivalvia;Autobranchia;Pteriomorphia;Pectinida;Pectinoidea;Pectinidae;Mizuhopecten;Mizuhopecten yessoensis
UdeM00000009118 6596 181.8 UdeM00000009118 XP_045194326.1 43.5 207
106 4 20 215 23 229 9.60E-43 181.8 6596 cellular
organisms;Eukaryota;Opisthokonta;Metazoa;Eumetazoa;Bilateria;Protostomia;
Spiralia;Lophotrochozoa;Mollusca;Bivalvia;Autobranchia;Heteroconchia;Euheterodonta;Imparidentia;Venerida;Veneroidea;Veneridae;Mercenaria;Mercenaria mercenaria
UdeM00000009119 6573 187.2 UdeM00000009119 XP_021359532.1 32.5 302
193 5 4 295 346 646 3.00E-44 187.2 6573 cellular
organisms;Eukaryota;Opisthokonta;Metazoa;Eumetazoa;Bilateria;Protostomia;
Spiralia;Lophotrochozoa;Mollusca;Bivalvia;Autobranchia;Pteriomorphia;Pectinida;Pectinoidea;Pectinidae;Mizuhopecten;Mizuhopecten yessoensis
UdeM00000009120 6596 207.6 UdeM00000009120 XP_045209563.1 32.7 336
162 6 82 373 19 334 2.90E-50 207.6 6596 cellular
organisms;Eukaryota;Opisthokonta;Metazoa;Eumetazoa;Bilateria;Protostomia;
Spiralia;Lophotrochozoa;Mollusca;Bivalvia;Autobranchia;Heteroconchia;Euheterodonta;Imparidentia;Venerida;Veneroidea;Veneridae;Mercenaria;Mercenaria mercenaria
UdeM00000009121 6596 80.1 UdeM00000009121 XP_045209326.1 47.7 132
49 6 52 171 60 183 3.20E-12 80.1 6596 cellular
organisms;Eukaryota;Opisthokonta;Metazoa;Eumetazoa;Bilateria;Protostomia;
Spiralia;Lophotrochozoa;Mollusca;Bivalvia;Autobranchia;Heteroconchia;Euheterodonta;Imparidentia;Venerida;Veneroidea;Veneridae;Mercenaria;Mercenaria mercenaria
UdeM00000034008 6596 248.4 UdeM00000034008 XP_045179850.1 56.2 208
90 1 25 232 19 225 8.80E-63 248.4 6596 cellular
organisms;Eukaryota;Opisthokonta;Metazoa;Eumetazoa;Bilateria;Protostomia;
Spiralia;Lophotrochozoa;Mollusca;Bivalvia;Autobranchia;Heteroconchia;Euheterodonta;Imparidentia;Venerida;Veneroidea;Veneridae;Mercenaria;Mercenaria mercenaria
UdeM00000034009 6596 220.3 UdeM00000034009 XP_045198179.1 33.1 405
237 6 11 382 675 1078 4.20E-54 220.3 6596 cellular
organisms;Eukaryota;Opisthokonta;Metazoa;Eumetazoa;Bilateria;Protostomia;
Spiralia;Lophotrochozoa;Mollusca;Bivalvia;Autobranchia;Heteroconchia;Euheterodonta;Imparidentia;Venerida;Veneroidea;Veneridae;Mercenaria;Mercenaria mercenaria
UdeM00000034010 6596 432.2 UdeM00000034010 XP_045156280.1 54 411
183 5 1 408 389 796 7.70E-118 432.2 6596 cellular
organisms;Eukaryota;Opisthokonta;Metazoa;Eumetazoa;Bilateria;Protostomia;

Spiralia;Lophotrochozoa;Mollusca;Bivalvia;Autobranchia;Heteroconchia;Euheterodonta;Imparidentia;Venerida;Veneroidea;Veneridae;Mercenaria;Mercenaria mercenaria
UdeM00000034011 6573 362.1 UdeM00000034011 XP_021349151.1 52.9 333 154 2 2 334 30 359 7.90E-97 362.1 6573 cellular organisms;Eukaryota;Opisthokonta;Metazoa;Eumetazoa;Bilateria;Protostomia;Spiralia;Lophotrochozoa;Mollusca;Bivalvia;Autobranchia;Pteriomorphia;Pectinida;Pectinoidea;Pectinidae;Mizuhopecten;Mizuhopecten yessoensis
UdeM00000034012 29159 97.4 UdeM00000034012 XP_034330259.1 34 150 82 5 8 153 403 539 1.70E-17 97.4 29159 cellular organisms;Eukaryota;Opisthokonta;Metazoa;Eumetazoa;Bilateria;Protostomia;Spiralia;Lophotrochozoa;Mollusca;Bivalvia;Autobranchia;Pteriomorphia;Ostreida;Ostreoidea;Ostreidae;Crassostrea;Crassostrea gigas
UdeM00000034013 6596 174.9 UdeM00000034013 XP_045193479.1 53.9 152 70 0 1 152 28 179 8.00E-41 174.9 6596 cellular organisms;Eukaryota;Opisthokonta;Metazoa;Eumetazoa;Bilateria;Protostomia;Spiralia;Lophotrochozoa;Mollusca;Bivalvia;Autobranchia;Heteroconchia;Euheterodonta;Imparidentia;Venerida;Veneroidea;Veneridae;Mercenaria;Mercenaria mercenaria
UdeM00000034014 6573 134.4 UdeM00000034014 XP_021357372.1 53.1 128 54 4 1 128 154 275 1.10E-28 134.4 6573 cellular organisms;Eukaryota;Opisthokonta;Metazoa;Eumetazoa;Bilateria;Protostomia;Spiralia;Lophotrochozoa;Mollusca;Bivalvia;Autobranchia;Pteriomorphia;Pectinida;Pectinoidea;Pectinidae;Mizuhopecten;Mizuhopecten yessoensis
UdeM00000034016 7668 149.8 UdeM00000034016 XP_030845483.1 61.1 126 49 0 1 126 384 509 2.30E-33 149.8 7668 cellular organisms;Eukaryota;Opisthokonta;Metazoa;Eumetazoa;Bilateria;Deuterostomia;Echinodermata;Eleutherozoa;Echinozoa;Echinoidea;Euechinoidea;Echinacea;Camarodonta;Echinidea;Strongylocentrotidae;Strongylocentrotus;Strongylocentrotus purpuratus
UdeM00000034017 6579 110.5 UdeM00000034017 XP_033753599.1 57.5 106 34 3 12 106 174 279 1.30E-21 110.5 6579 cellular organisms;Eukaryota;Opisthokonta;Metazoa;Eumetazoa;Bilateria;Protostomia;Spiralia;Lophotrochozoa;Mollusca;Bivalvia;Autobranchia;Pteriomorphia;Pectinida;Pectinoidea;Pectinidae;Pecten;Pecten maximus
UdeM00000034018 37653 184.5 UdeM00000034018 XP_014784938.1 53.9 152 69 1 5 156 212 362 1.10E-43 184.5 37653 cellular organisms;Eukaryota;Opisthokonta;Metazoa;Eumetazoa;Bilateria;Protostomia;Spiralia;Lophotrochozoa;Mollusca;Cephalopoda;Coleoidea;Octopodiformes;Octopoda;Incirrata;Octopodidae;Octopus;Octopus bimaculoides
UdeM00000034019 151771 90.5 UdeM00000034019 XP_028397269.1 51.2 80 33 1 1 74 206 285 3.10E-15 90.5 151771 cellular organisms;Eukaryota;Opisthokonta;Metazoa;Eumetazoa;Cnidaria;Anthozoa;Octocorallia;Alcyonacea;Alcyoniina;Nephtheidae;Dendronephthya;Dendronephthya gigantea
UdeM00000034020 6565 76.6 UdeM00000034020 XP_022298030.1 30.5 131 91 0 6 136 11 141 3.10E-11 76.6 6565 cellular organisms;Eukaryota;Opisthokonta;Metazoa;Eumetazoa;Bilateria;Protostomia;Spiralia;Lophotrochozoa;Mollusca;Bivalvia;Autobranchia;Pteriomorphia;Ostreida;Ostreoidea;Ostreidae;Crassostrea;Crassostrea virginica
UdeM00000034021 6565 130.2 UdeM00000034021 XP_022297037.1 64.5 93 33 0 1 93 228 320 1.50E-27 130.2 6565 cellular organisms;Eukaryota;Opisthokonta;Metazoa;Eumetazoa;Bilateria;Protostomia;

Spiralia;Lophotrochozoa;Mollusca;Bivalvia;Autobranchia;Pteriomorphia;Ostreida;Ostreoidea;Ostreidae;Crassostrea;Crassostrea virginica
UdeM00000034022 6565 223.8 UdeM00000034022 XP_022286598.1 40.8 348 140 7 1 292 1 338 3.50E-55 223.8 6565 cellular organisms;Eukaryota;Opisthokonta;Metazoa;Eumetazoa;Bilateria;Protostomia;Spiralia;Lophotrochozoa;Mollusca;Bivalvia;Autobranchia;Pteriomorphia;Ostreida;Ostreoidea;Ostreidae;Crassostrea;Crassostrea virginica
UdeM00000034023 6579 221.5 UdeM00000034023 XP_033729837.1 58.8 170 70 0 14 183 17 186 9.70E-55 221.5 6579 cellular organisms;Eukaryota;Opisthokonta;Metazoa;Eumetazoa;Bilateria;Protostomia;Spiralia;Lophotrochozoa;Mollusca;Bivalvia;Autobranchia;Pteriomorphia;Pectinida;Pectinoidea;Pectinidae;Pecten;Pecten maximus
UdeM00000034024 10224 62.4 UdeM00000034024 XP_002737779.1 57.1 49 21 0 1 49 437 485 3.00E-07 62.4 10224 cellular organisms;Eukaryota;Opisthokonta;Metazoa;Eumetazoa;Bilateria;Deuterostomia;Hemichordata;Enteropneusta;Harrimaniidae;Saccoglossus;Saccoglossus kowalevskii
UdeM00000034025 6565 179.5 UdeM00000034025 XP_022286598.1 50 174 85 1 40 213 266 437 4.90E-42 179.5 6565 cellular organisms;Eukaryota;Opisthokonta;Metazoa;Eumetazoa;Bilateria;Protostomia;Spiralia;Lophotrochozoa;Mollusca;Bivalvia;Autobranchia;Pteriomorphia;Ostreida;Ostreoidea;Ostreidae;Crassostrea;Crassostrea virginica
UdeM00000034026 6565 122.5 UdeM00000034026 XP_022342298.1 31.4 318 98 4 48 249 200 513 8.60E-25 122.5 6565 cellular organisms;Eukaryota;Opisthokonta;Metazoa;Eumetazoa;Bilateria;Protostomia;Spiralia;Lophotrochozoa;Mollusca;Bivalvia;Autobranchia;Pteriomorphia;Ostreida;Ostreoidea;Ostreidae;Crassostrea;Crassostrea virginica
UdeM00000034027 390379 95.1 UdeM00000034027 XP_034039899.1 51.4 70 34 0 5 74 4 73 4.50E-17 95.1 390379 cellular organisms;Eukaryota;Opisthokonta;Metazoa;Eumetazoa;Bilateria;Deuterostomia;Chordata;Craniata;Vertebrata;Gnathostomata;Teleostomi;Euteleostomi;Actinopterygii;Actinopteri;Neopterygii;Teleostei;Osteoglossocephalai;Clupeocephala;Euteleosteomorpha;Neoteleostei;Eurypterygia;Ctenosquamata;Acanthomorphata;Euacanthomorphacea;Percomorphaceae;Batrachoidaria;Batrachoidiformes;Batrachoididae;Thalassophyrininae;Thalassophryne;Thalassophryne amazonica
UdeM00000034028 2652724 393.7 UdeM00000034028 XP_020903442.1 41.8 519 284 8 9 515 2 514 3.80E-106 393.7 2652724 cellular organisms;Eukaryota;Opisthokonta;Metazoa;Eumetazoa;Cnidaria;Anthozoa;Hexacorallia;Actiniaria;Aiptasiidae;Exaiptasia;Exaiptasia diaphana
UdeM00000034030 1203425 71.6 UdeM00000034030 XP_034530357.1 60.8 51 20 0 1 51 72 122 3.70E-10 71.6 1203425 cellular organisms;Eukaryota;Opisthokonta;Metazoa;Eumetazoa;Bilateria;Deuterostomia;Chordata;Craniata;Vertebrata;Gnathostomata;Teleostomi;Euteleostomi;Actinopterygii;Actinopteri;Neopterygii;Teleostei;Osteoglossocephalai;Clupeocephala;Euteleosteomorpha;Neoteleostei;Eurypterygia;Ctenosquamata;Acanthomorphata;Euacanthomorphacea;Percomorphaceae;Eupercaria;Labriformes;Labridae;Notolabrus;Notolabrus celidotus
UdeM00000034029 29159 82.8 UdeM00000034029 XP_034334268.1 62.3 61 20 1 1 61 160 217 1.90E-13 82.8 29159 cellular organisms;Eukaryota;Opisthokonta;Metazoa;Eumetazoa;Bilateria;Protostomia;

Spiralia;Lophotrochozoa;Mollusca;Bivalvia;Autobranchia;Pteriomorphia;Ostreida;Ostreoidea;Ostreidae;Crassostrea;Crassostrea gigas
UdeM00000034031 29159 119 UdeM00000034031 XP_034334268.1 59.4 96 36 1 1 96 160 252 3.30E-24 119 29159 cellular organisms;Eukaryota;Opisthokonta;Metazoa;Eumetazoa;Bilateria;Protostomia;Spiralia;Lophotrochozoa;Mollusca;Bivalvia;Autobranchia;Pteriomorphia;Ostreida;Ostreoidea;Ostreidae;Crassostrea;Crassostrea gigas
UdeM00000034033 46514 132.9 UdeM00000034033 XP_038073135.1 36.4 530 231 9 1 434 14 533 1.00E-27 132.9 46514 cellular organisms;Eukaryota;Opisthokonta;Metazoa;Eumetazoa;Bilateria;Deuterostomia;Echinodermata;Eleutherozoa;Asterozoa;Asteroidea;Valvatacea;Valvatida;Asterinidae;Patiria;Patiria miniata
UdeM00000034034 6596 198.7 UdeM00000034034 XP_045179821.1 40.4 277 161 3 9 283 31 305 9.70E-48 198.7 6596 cellular organisms;Eukaryota;Opisthokonta;Metazoa;Eumetazoa;Bilateria;Protostomia;Spiralia;Lophotrochozoa;Mollusca;Bivalvia;Autobranchia;Heteroconchia;Euheterodonta;Imparidentia;Venerida;Veneroidea;Veneridae;Mercenaria;Mercenaria mercenaria
UdeM00000034035 1735272 61.6 UdeM00000034035 XP_041359107.1 27.6 214 111 7 4 191 8 203 1.30E-06 61.6 1735272 cellular organisms;Eukaryota;Opisthokonta;Metazoa;Eumetazoa;Bilateria;Protostomia;Spiralia;Lophotrochozoa;Mollusca;Gastropoda;Neomphalina;Neomphaloidea;Peltospiridae;Gigantopelta;Gigantopelta aegis
UdeM00000034036 1735272 61.6 UdeM00000034036 XP_041359107.1 27.6 217 113 7 22 212 5 203 1.40E-06 61.6 1735272 cellular organisms;Eukaryota;Opisthokonta;Metazoa;Eumetazoa;Bilateria;Protostomia;Spiralia;Lophotrochozoa;Mollusca;Gastropoda;Neomphalina;Neomphaloidea;Peltospiridae;Gigantopelta;Gigantopelta aegis
UdeM00000034038 6579 373.6 UdeM00000034038 XP_033743002.1 50 344 171 1 92 434 10 353 3.40E-100 373.6 6579 cellular organisms;Eukaryota;Opisthokonta;Metazoa;Eumetazoa;Bilateria;Protostomia;Spiralia;Lophotrochozoa;Mollusca;Bivalvia;Autobranchia;Pteriomorphia;Pectinida;Pectinoidea;Pectinidae;Pecten;Pecten maximus
UdeM00000034037 6579 373.6 UdeM00000034037 XP_033743002.1 50 344 171 1 113 455 10 353 3.60E-100 373.6 6579 cellular organisms;Eukaryota;Opisthokonta;Metazoa;Eumetazoa;Bilateria;Protostomia;Spiralia;Lophotrochozoa;Mollusca;Bivalvia;Autobranchia;Pteriomorphia;Pectinida;Pectinoidea;Pectinidae;Pecten;Pecten maximus
UdeM00000034039 6596 198.7 UdeM00000034039 XP_045175762.1 36.4 368 202 10 4 354 5 357 1.20E-47 198.7 6596 cellular organisms;Eukaryota;Opisthokonta;Metazoa;Eumetazoa;Bilateria;Protostomia;Spiralia;Lophotrochozoa;Mollusca;Bivalvia;Autobranchia;Heteroconchia;Euheterodonta;Imparidentia;Venerida;Veneroidea;Veneridae;Mercenaria;Mercenaria mercenaria
UdeM00000034040 6596 188 UdeM00000034040 XP_045175763.1 32.1 399 208 10 54 436 5 356 2.60E-44 188 6596 cellular organisms;Eukaryota;Opisthokonta;Metazoa;Eumetazoa;Bilateria;Protostomia;Spiralia;Lophotrochozoa;Mollusca;Bivalvia;Autobranchia;Heteroconchia;Euheterodonta;Imparidentia;Venerida;Veneroidea;Veneridae;Mercenaria;Mercenaria mercenaria
UdeM00000034041 6596 168.7 UdeM00000034041 XP_045175761.1 34.9 292 162 8 54 329 82 361 1.30E-38 168.7 6596 cellular

organisms;Eukaryota;Opisthokonta;Metazoa;Eumetazoa;Bilateria;Protostomia;Spiralia;Lophotrochozoa;Mollusca;Bivalvia;Autobranchia;Heteroconchia;Euheterodonta;Imparidentia;Venerida;Veneroidea;Veneridae;Mercenaria;Mercenaria mercenaria
UdeM00000034042 34632 65.5 UdeM00000034042 XP_037504739.1 42.4 66 38 0 8 73 365 430 3.30E-08 65.5 34632 cellular organisms;Eukaryota;Opisthokonta;Metazoa;Eumetazoa;Bilateria;Protostomia;Ecdysozoa;Panarthropoda;Arthropoda;Chelicerata;Arachnida;Acari;Parasitiformes;Ixodida;Ixodoidea;Ixodidae;Rhipicephalinae;Rhipicephalus;Rhipicephalus;Rhipicephalus sanguineus group;Rhipicephalus sanguineus
UdeM00000034043 6596 87 UdeM00000034043 XP_045175117.1 37.4 147 89 2 3 148 4 148 3.70E-14 87 6596 cellular organisms;Eukaryota;Opisthokonta;Metazoa;Eumetazoa;Bilateria;Protostomia;Spiralia;Lophotrochozoa;Mollusca;Bivalvia;Autobranchia;Heteroconchia;Euheterodonta;Imparidentia;Venerida;Veneroidea;Veneridae;Mercenaria;Mercenaria mercenaria
UdeM00000034044 6596 273.9 UdeM00000034044 XP_045206316.1 51.9 268 125 2 11 277 169 433 3.10E-70 273.9 6596 cellular organisms;Eukaryota;Opisthokonta;Metazoa;Eumetazoa;Bilateria;Protostomia;Spiralia;Lophotrochozoa;Mollusca;Bivalvia;Autobranchia;Heteroconchia;Euheterodonta;Imparidentia;Venerida;Veneroidea;Veneridae;Mercenaria;Mercenaria mercenaria
UdeM00000034045 6596 114.8 UdeM00000034045 XP_045175117.1 40.5 158 80 5 12 167 192 337 1.20E-22 114.8 6596 cellular organisms;Eukaryota;Opisthokonta;Metazoa;Eumetazoa;Bilateria;Protostomia;Spiralia;Lophotrochozoa;Mollusca;Bivalvia;Autobranchia;Heteroconchia;Euheterodonta;Imparidentia;Venerida;Veneroidea;Veneridae;Mercenaria;Mercenaria mercenaria
UdeM00000034046 6596 298.1 UdeM00000034046 XP_045201856.1 56.6 244 101 2 27 266 25 267 1.10E-77 298.1 6596 cellular organisms;Eukaryota;Opisthokonta;Metazoa;Eumetazoa;Bilateria;Protostomia;Spiralia;Lophotrochozoa;Mollusca;Bivalvia;Autobranchia;Heteroconchia;Euheterodonta;Imparidentia;Venerida;Veneroidea;Veneridae;Mercenaria;Mercenaria mercenaria
UdeM00000034047 6596 299.7 UdeM00000034047 XP_045201856.1 56.6 242 99 2 29 266 28 267 3.80E-78 299.7 6596 cellular organisms;Eukaryota;Opisthokonta;Metazoa;Eumetazoa;Bilateria;Protostomia;Spiralia;Lophotrochozoa;Mollusca;Bivalvia;Autobranchia;Heteroconchia;Euheterodonta;Imparidentia;Venerida;Veneroidea;Veneridae;Mercenaria;Mercenaria mercenaria
UdeM00000034048 6596 232.6 UdeM00000034048 XP_045201856.1 57.6 184 76 1 2 185 86 267 3.90E-58 232.6 6596 cellular organisms;Eukaryota;Opisthokonta;Metazoa;Eumetazoa;Bilateria;Protostomia;Spiralia;Lophotrochozoa;Mollusca;Bivalvia;Autobranchia;Heteroconchia;Euheterodonta;Imparidentia;Venerida;Veneroidea;Veneridae;Mercenaria;Mercenaria mercenaria
UdeM00000034050 6596 150.6 UdeM00000034050 XP_045175117.1 36.3 273 146 9 3 259 77 337 3.00E-33 150.6 6596 cellular organisms;Eukaryota;Opisthokonta;Metazoa;Eumetazoa;Bilateria;Protostomia;Spiralia;Lophotrochozoa;Mollusca;Bivalvia;Autobranchia;Heteroconchia;Euheterodonta;Imparidentia;Venerida;Veneroidea;Veneridae;Mercenaria;Mercenaria mercenaria
UdeM00000034051 6596 244.2 UdeM00000034051 XP_045201856.1 52.5 217 98 3 12 224 52 267 1.60E-61 244.2 6596 cellular

organisms;Eukaryota;Opisthokonta;Metazoa;Eumetazoa;Bilateria;Protostomia;Spiralia;Lophotrochozoa;Mollusca;Bivalvia;Autobranchia;Heteroconchia;Euheterodonta;Imparidentia;Venerida;Veneroidea;Veneridae;Mercenaria;Mercenaria mercenaria
UdeM00000034052 6596 253.8 UdeM00000034052 XP_045201856.1 56 207 85 2 9 211 63 267 1.90E-64 253.8 6596 cellular organisms;Eukaryota;Opisthokonta;Metazoa;Eumetazoa;Bilateria;Protostomia;Spiralia;Lophotrochozoa;Mollusca;Bivalvia;Autobranchia;Heteroconchia;Euheterodonta;Imparidentia;Venerida;Veneroidea;Veneridae;Mercenaria;Mercenaria mercenaria
UdeM00000034053 2652724 153.7 UdeM00000034053 XP_028512668.1 45.2 166 86 2 29 193 97 258 3.40E-34 153.7 2652724 cellular organisms;Eukaryota;Opisthokonta;Metazoa;Eumetazoa;Cnidaria;Anthozoa;Hexacorallia;Actiniaria;Aiptasiidae;Exaiptasia;Exaiptasia diaphana
UdeM00000034054 6596 101.3 UdeM00000034054 XP_045179845.1 31.9 160 107 2 8 166 1325 1483 1.40E-18 101.3 6596 cellular organisms;Eukaryota;Opisthokonta;Metazoa;Eumetazoa;Bilateria;Protostomia;Spiralia;Lophotrochozoa;Mollusca;Bivalvia;Autobranchia;Heteroconchia;Euheterodonta;Imparidentia;Venerida;Veneroidea;Veneridae;Mercenaria;Mercenaria mercenaria
UdeM00000034055 6573 58.2 UdeM00000034055 XP_021364203.1 31.2 109 71 3 7 114 193 298 8.20E-06 58.2 6573 cellular organisms;Eukaryota;Opisthokonta;Metazoa;Eumetazoa;Bilateria;Protostomia;Spiralia;Lophotrochozoa;Mollusca;Bivalvia;Autobranchia;Pteriomorphia;Pectinida;Pectinoidea;Pectinidae;Mizuhopecten;Mizuhopecten yessoensis
UdeM00000034056 6596 213.4 UdeM00000034056 XP_045179019.1 34.5 412 235 11 14 407 23 417 6.50E-52 213.4 6596 cellular organisms;Eukaryota;Opisthokonta;Metazoa;Eumetazoa;Bilateria;Protostomia;Spiralia;Lophotrochozoa;Mollusca;Bivalvia;Autobranchia;Heteroconchia;Euheterodonta;Imparidentia;Venerida;Veneroidea;Veneridae;Mercenaria;Mercenaria mercenaria
UdeM00000034058 6596 908.3 UdeM00000034058 XP_045177368.1 55.7 795 336 10 38 821 44 833 7.10E-261 908.3 6596 cellular organisms;Eukaryota;Opisthokonta;Metazoa;Eumetazoa;Bilateria;Protostomia;Spiralia;Lophotrochozoa;Mollusca;Bivalvia;Autobranchia;Heteroconchia;Euheterodonta;Imparidentia;Venerida;Veneroidea;Veneridae;Mercenaria;Mercenaria mercenaria
UdeM00000034057 6596 908.3 UdeM00000034057 XP_045177368.1 55.7 795 336 10 59 842 44 833 7.30E-261 908.3 6596 cellular organisms;Eukaryota;Opisthokonta;Metazoa;Eumetazoa;Bilateria;Protostomia;Spiralia;Lophotrochozoa;Mollusca;Bivalvia;Autobranchia;Heteroconchia;Euheterodonta;Imparidentia;Venerida;Veneroidea;Veneridae;Mercenaria;Mercenaria mercenaria
UdeM00000034059 37621 102.8 UdeM00000034059 XP_014670052.1 50 102 42 1 2 94 6 107 3.70E-19 102.8 37621 cellular organisms;Eukaryota;Opisthokonta;Metazoa;Eumetazoa;Bilateria;Protostomia;Ecdysozoa;Scalidophora;Priapulida;Priapulimorpha;Priapulimorphida;Priapulidae;Priapulus;Priapulus caudatus
UdeM00000034060 210409 102.4 UdeM00000034060 XP_045107160.1 40.7 113 65 1 1 111 171 283 3.80E-19 102.4 210409 cellular organisms;Eukaryota;Opisthokonta;Metazoa;Eumetazoa;Bilateria;Protostomia;Ecdysozoa;Panarthropoda;Arthropoda;Mandibulata;Pancrustacea;Crustacea;Mul

ticrustacea;Malacostraca;Eumalacostraca;Eucarida;Decapoda;Pleocyemata;Brachyura;Eubrachyura;Heterotremata;Portunoidea;Portunidae;Portunus;Portunus trituberculatus
UdeM00000034063 6500 504.6 UdeM00000034063 XP_005100247.1 80.4 285 56 0 1 285 53 337 8.40E-140 504.6 6500 cellular organisms;Eukaryota;Opisthokonta;Metazoa;Eumetazoa;Bilateria;Protostomia;Spiralia;Lophotrochozoa;Mollusca;Gastropoda;Heterobranchia;Euthyneura;Tectipleura;Aplysiida;Aplysioidea;Aplysiidae;Aplysia;Aplysia californica
UdeM00000034064 6596 298.1 UdeM00000034064 XP_045177776.1 38.9 460 249 11 3 446 481 924 1.90E-77 298.1 6596 cellular organisms;Eukaryota;Opisthokonta;Metazoa;Eumetazoa;Bilateria;Protostomia;Spiralia;Lophotrochozoa;Mollusca;Bivalvia;Autobranchia;Heteroconchia;Euheterodonta;Imparidentia;Venerida;Veneroidea;Veneridae;Mercenaria;Mercenaria mercenaria
UdeM00000034065 6238 112.1 UdeM00000034065 XP_002638250.1 31.9 226 127 5 2 202 4 227 9.20E-22 112.1 6238 cellular organisms;Eukaryota;Opisthokonta;Metazoa;Eumetazoa;Bilateria;Protostomia;Ecdysozoa;Nematoda;Chromadorea;Rhabditida;Rhabditina;Rhabditomorpha;Rhabditoidea;Rhabditidae;Peloderinae;Caenorhabditis;Caenorhabditis briggsae
UdeM00000034066 45351 84.3 UdeM00000034066 XP_001623471.2 27.6 221 131 5 3 207 6 213 2.00E-13 84.3 45351 cellular organisms;Eukaryota;Opisthokonta;Metazoa;Eumetazoa;Cnidaria;Anthozoa;Hexacorallia;Actiniaria;Edwardsiidae;Nematostella;Nematostella vectensis
UdeM00000034067 6596 303.5 UdeM00000034067 XP_045177776.1 42.9 378 202 7 9 378 552 923 5.80E-79 303.5 6596 cellular organisms;Eukaryota;Opisthokonta;Metazoa;Eumetazoa;Bilateria;Protostomia;Spiralia;Lophotrochozoa;Mollusca;Bivalvia;Autobranchia;Heteroconchia;Euheterodonta;Imparidentia;Venerida;Veneroidea;Veneridae;Mercenaria;Mercenaria mercenaria
UdeM00000034068 6579 232.6 UdeM00000034068 XP_033729769.1 42.7 288 130 4 48 304 32 315 7.60E-58 232.6 6579 cellular organisms;Eukaryota;Opisthokonta;Metazoa;Eumetazoa;Bilateria;Protostomia;Spiralia;Lophotrochozoa;Mollusca;Bivalvia;Autobranchia;Pteriomorphia;Pectinida;Pectinoidea;Pectinidae;Pecten;Pecten maximus
UdeM00000034069 6579 146 UdeM00000034069 XP_033747958.1 58.4 101 42 0 1 101 57 157 3.30E-32 146 6579 cellular organisms;Eukaryota;Opisthokonta;Metazoa;Eumetazoa;Bilateria;Protostomia;Spiralia;Lophotrochozoa;Mollusca;Bivalvia;Autobranchia;Pteriomorphia;Pectinida;Pectinoidea;Pectinidae;Pecten;Pecten maximus
UdeM00000034070 6579 159.8 UdeM00000034070 XP_033747958.1 58.2 110 46 0 18 127 48 157 2.70E-36 159.8 6579 cellular organisms;Eukaryota;Opisthokonta;Metazoa;Eumetazoa;Bilateria;Protostomia;Spiralia;Lophotrochozoa;Mollusca;Bivalvia;Autobranchia;Pteriomorphia;Pectinida;Pectinoidea;Pectinidae;Pecten;Pecten maximus
UdeM00000034073 6526 219.2 UdeM00000034073 XP_013063620.1 34.8 480 226 12 14 416 60 529 1.00E-53 219.2 6526 cellular organisms;Eukaryota;Opisthokonta;Metazoa;Eumetazoa;Bilateria;Protostomia;Spiralia;Lophotrochozoa;Mollusca;Gastropoda;Heterobranchia;Euthyneura;Panpulmonata;Hygrophila;Lymnaeoidea;Planorbidae;Biomphalaria;Biomphalaria glabrata
UdeM00000034075 29159 443 UdeM00000034075 XP_019924536.2 43.8 537 282 7 90 624 680 1198 6.70E-121 443 29159 cellular organisms;Eukaryota;Opisthokonta;Metazoa;Eumetazoa;Bilateria;Protostomia;

Spiralia;Lophotrochozoa;Mollusca;Bivalvia;Autobranchia;Pteriomorphia;Ostreida;Ostreoidea;Ostreidae;Crassostrea;Crassostrea gigas
UdeM00000034076 6573 530.4 UdeM00000034076 XP_021362500.1 33.9 1097 525 24 81 997 103 1179 5.40E-147 530.4 6573 cellular organisms;Eukaryota;Opisthokonta;Metazoa;Eumetazoa;Bilateria;Protostomia;Spiralia;Lophotrochozoa;Mollusca;Bivalvia;Autobranchia;Pteriomorphia;Pectinida;Pectinoidea;Pectinidae;Mizuhopecten;Mizuhopecten yessoensis
UdeM00000034077 6526 231.5 UdeM00000034077 XP_013063620.1 34.6 514 233 14 45 469 30 529 2.20E-57 231.5 6526 cellular organisms;Eukaryota;Opisthokonta;Metazoa;Eumetazoa;Bilateria;Protostomia;Spiralia;Lophotrochozoa;Mollusca;Gastropoda;Heterobranchia;Euthyneura;Panpulmonata;Hygrophila;Lymnaeoidea;Planorbidae;Biomphalaria;Biomphalaria glabrata
UdeM00000034079 6573 389.8 UdeM00000034079 XP_021362500.1 38.7 571 275 9 79 582 652 1214 6.40E-105 389.8 6573 cellular organisms;Eukaryota;Opisthokonta;Metazoa;Eumetazoa;Bilateria;Protostomia;Spiralia;Lophotrochozoa;Mollusca;Bivalvia;Autobranchia;Pteriomorphia;Pectinida;Pectinoidea;Pectinidae;Mizuhopecten;Mizuhopecten yessoensis
UdeM00000034080 7739 194.9 UdeM00000034080 XP_035700323.1 31.2 528 252 15 85 506 134 656 4.70E-46 194.9 7739 cellular organisms;Eukaryota;Opisthokonta;Metazoa;Eumetazoa;Bilateria;Deuterostomia;Chordata;Cephalochordata;Leptocardii;Amphioxiformes;Branchiostomidae;Branchiostoma;Branchiostoma floridae
UdeM00000034081 6596 116.7 UdeM00000034081 XP_045189228.1 53 100 47 0 77 176 236 335 4.70E-23 116.7 6596 cellular organisms;Eukaryota;Opisthokonta;Metazoa;Eumetazoa;Bilateria;Protostomia;Spiralia;Lophotrochozoa;Mollusca;Bivalvia;Autobranchia;Heteroconchia;Euheterodonta;Imparidentia;Venerida;Veneroidea;Veneridae;Mercenaria;Mercenaria mercenaria
UdeM00000034084 6596 112.1 UdeM00000034084 XP_045202349.1 24.1 370 252 9 585 929 404 769 4.00E-21 112.1 6596 cellular organisms;Eukaryota;Opisthokonta;Metazoa;Eumetazoa;Bilateria;Protostomia;Spiralia;Lophotrochozoa;Mollusca;Bivalvia;Autobranchia;Heteroconchia;Euheterodonta;Imparidentia;Venerida;Veneroidea;Veneridae;Mercenaria;Mercenaria mercenaria
UdeM00000034085 29159 147.1 UdeM00000034085 XP_034320422.1 65 103 33 1 86 185 122 224 2.30E-32 147.1 29159 cellular organisms;Eukaryota;Opisthokonta;Metazoa;Eumetazoa;Bilateria;Protostomia;Spiralia;Lophotrochozoa;Mollusca;Bivalvia;Autobranchia;Pteriomorphia;Ostreida;Ostreoidea;Ostreidae;Crassostrea;Crassostrea gigas
UdeM00000034086 6573 124 UdeM00000034086 XP_021355633.1 48.5 130 66 1 9 137 11 140 2.90E-25 124 6573 cellular organisms;Eukaryota;Opisthokonta;Metazoa;Eumetazoa;Bilateria;Protostomia;Spiralia;Lophotrochozoa;Mollusca;Bivalvia;Autobranchia;Pteriomorphia;Pectinida;Pectinoidea;Pectinidae;Mizuhopecten;Mizuhopecten yessoensis
UdeM00000034088 6596 201.8 UdeM00000034088 XP_045200918.1 23 1071 737 32 1993 3005 1723 2763 1.20E-47 201.8 6596 cellular organisms;Eukaryota;Opisthokonta;Metazoa;Eumetazoa;Bilateria;Protostomia;Spiralia;Lophotrochozoa;Mollusca;Bivalvia;Autobranchia;Heteroconchia;Euheterodonta;Imparidentia;Venerida;Veneroidea;Veneridae;Mercenaria;Mercenaria mercenaria
UdeM00000034091 6573 79 UdeM00000034091 XP_021372170.1 27.1 255 121 9 518 711 809 1059 2.90E-11 79 6573 cellular organisms;Eukaryota;Opisthokonta;Metazoa;Eumetazoa;Bilateria;Protostomia;

Spiralia;Lophotrochozoa;Mollusca;Bivalvia;Autobranchia;Pteriomorphia;Pectinida;Pectinoidea;Pectinidae;Mizuhopecten;Mizuhopecten yessoensis
UdeM00000034092 6573 139 UdeM00000034092 XP_021372109.1 37.1 178 106 1 11 182 57 234 7.70E-30 139 6573 cellular organisms;Eukaryota;Opisthokonta;Metazoa;Eumetazoa;Bilateria;Protostomia;Spiralia;Lophotrochozoa;Mollusca;Bivalvia;Autobranchia;Pteriomorphia;Pectinida;Pectinoidea;Pectinidae;Mizuhopecten;Mizuhopecten yessoensis
UdeM00000034093 6596 276.6 UdeM00000034093 XP_045182756.1 64.2 204 72 1 1 203 1 204 2.60E-71 276.6 6596 cellular organisms;Eukaryota;Opisthokonta;Metazoa;Eumetazoa;Bilateria;Protostomia;Spiralia;Lophotrochozoa;Mollusca;Bivalvia;Autobranchia;Heteroconchia;Euheterodonta;Imparidentia;Venerida;Veneroidea;Veneridae;Mercenaria;Mercenaria mercenaria
UdeM00000034094 6596 189.5 UdeM00000034094 XP_045171694.1 61.1 144 55 1 80 223 223 365 4.60E-45 189.5 6596 cellular organisms;Eukaryota;Opisthokonta;Metazoa;Eumetazoa;Bilateria;Protostomia;Spiralia;Lophotrochozoa;Mollusca;Bivalvia;Autobranchia;Heteroconchia;Euheterodonta;Imparidentia;Venerida;Veneroidea;Veneridae;Mercenaria;Mercenaria mercenaria
UdeM00000034096 6565 197.6 UdeM00000034096 XP_022315923.1 39.4 259 156 1 5 262 149 407 2.10E-47 197.6 6565 cellular organisms;Eukaryota;Opisthokonta;Metazoa;Eumetazoa;Bilateria;Protostomia;Spiralia;Lophotrochozoa;Mollusca;Bivalvia;Autobranchia;Pteriomorphia;Ostreida;Ostreoidea;Ostreidae;Crassostrea;Crassostrea virginica
UdeM00000034097 6596 188.3 UdeM00000034097 XP_045214901.1 31.8 400 262 4 15 410 61 453 3.70E-44 188.3 6596 cellular organisms;Eukaryota;Opisthokonta;Metazoa;Eumetazoa;Bilateria;Protostomia;Spiralia;Lophotrochozoa;Mollusca;Bivalvia;Autobranchia;Heteroconchia;Euheterodonta;Imparidentia;Venerida;Veneroidea;Veneridae;Mercenaria;Mercenaria mercenaria
UdeM00000034098 45351 83.6 UdeM00000034098 XP_001623471.2 35.9 145 75 3 11 139 14 156 3.40E-13 83.6 45351 cellular organisms;Eukaryota;Opisthokonta;Metazoa;Eumetazoa;Cnidaria;Anthozoa;Hexacorallia;Actiniaria;Edwardsiidae;Nematostella;Nematostella vectensis
UdeM00000034099 1735272 206.1 UdeM00000034099 XP_041374260.1 65.8 155 46 2 1 151 244 395 3.20E-50 206.1 1735272 cellular organisms;Eukaryota;Opisthokonta;Metazoa;Eumetazoa;Bilateria;Protostomia;Spiralia;Lophotrochozoa;Mollusca;Gastropoda;Neomphalina;Neomphaloidea;Peltospiridae;Gigantopelta;Gigantopelta aegis
UdeM00000034100 6573 449.1 UdeM00000034100 XP_021370830.1 82.3 249 44 0 6 254 7 255 3.80E-123 449.1 6573 cellular organisms;Eukaryota;Opisthokonta;Metazoa;Eumetazoa;Bilateria;Protostomia;Spiralia;Lophotrochozoa;Mollusca;Bivalvia;Autobranchia;Pteriomorphia;Pectinida;Pectinoidea;Pectinidae;Mizuhopecten;Mizuhopecten yessoensis
UdeM00000034101 6596 191.8 UdeM00000034101 XP_045164125.1 41.8 273 91 2 1 205 320 592 8.50E-46 191.8 6596 cellular organisms;Eukaryota;Opisthokonta;Metazoa;Eumetazoa;Bilateria;Protostomia;Spiralia;Lophotrochozoa;Mollusca;Bivalvia;Autobranchia;Heteroconchia;Euheterodonta;Imparidentia;Venerida;Veneroidea;Veneridae;Mercenaria;Mercenaria mercenaria
UdeM00000034102 6573 278.1 UdeM00000034102 XP_021351807.1 59.8 229 83 1 1 220 242 470 1.00E-71 278.1 6573 cellular organisms;Eukaryota;Opisthokonta;Metazoa;Eumetazoa;Bilateria;Protostomia;

Spiralia;Lophotrochozoa;Mollusca;Bivalvia;Autobranchia;Pteriomorphia;Pectinida;Pectinoidea;Pectinidae;Mizuhopecten;Mizuhopecten yessoensis
UdeM00000034103 225164 200.7 UdeM00000034103 XP_009047395.1 44.1 204 113 1 4 207 1 203 1.90E-48 200.7 225164 cellular organisms;Eukaryota;Opisthokonta;Metazoa;Eumetazoa;Bilateria;Protostomia;Spiralia;Lophotrochozoa;Mollusca;Gastropoda;Patellogastropoda;Lottioidea;Lottiidae;Lottia;Lottia gigantea
UdeM00000034104 6596 4286.9 UdeM00000034104 XP_045177825.1 64.2 3399 1169 16 36 3421 24 3388 0.00E+00 4286.9 6596 cellular organisms;Eukaryota;Opisthokonta;Metazoa;Eumetazoa;Bilateria;Protostomia;Spiralia;Lophotrochozoa;Mollusca;Bivalvia;Autobranchia;Heteroconchia;Euheterodonta;Imparidentia;Venerida;Veneroidea;Veneridae;Mercenaria;Mercenaria mercenaria
UdeM00000034105 6573 1775.8 UdeM00000034105 XP_021351806.1 68.5 1320 399 9 1 1314 1 1309 0.00E+00 1775.8 6573 cellular organisms;Eukaryota;Opisthokonta;Metazoa;Eumetazoa;Bilateria;Protostomia;Spiralia;Lophotrochozoa;Mollusca;Bivalvia;Autobranchia;Pteriomorphia;Pectinida;Pectinoidea;Pectinidae;Mizuhopecten;Mizuhopecten yessoensis
UdeM00000034106 6573 1776.9 UdeM00000034106 XP_021351806.1 68.5 1319 400 8 1 1313 1 1309 0.00E+00 1776.9 6573 cellular organisms;Eukaryota;Opisthokonta;Metazoa;Eumetazoa;Bilateria;Protostomia;Spiralia;Lophotrochozoa;Mollusca;Bivalvia;Autobranchia;Pteriomorphia;Pectinida;Pectinoidea;Pectinidae;Mizuhopecten;Mizuhopecten yessoensis
UdeM00000034107 6596 686.8 UdeM00000034107 XP_045178323.1 72.7 479 123 3 27 499 27 503 2.10E-194 686.8 6596 cellular organisms;Eukaryota;Opisthokonta;Metazoa;Eumetazoa;Bilateria;Protostomia;Spiralia;Lophotrochozoa;Mollusca;Bivalvia;Autobranchia;Heteroconchia;Euheterodonta;Imparidentia;Venerida;Veneroidea;Veneridae;Mercenaria;Mercenaria mercenaria
UdeM00000034108 29159 261.5 UdeM00000034108 XP_034300378.1 44 327 178 2 6 328 5 330 1.40E-66 261.5 29159 cellular organisms;Eukaryota;Opisthokonta;Metazoa;Eumetazoa;Bilateria;Protostomia;Spiralia;Lophotrochozoa;Mollusca;Bivalvia;Autobranchia;Pteriomorphia;Ostreida;Ostreoidea;Ostreidae;Crassostrea;Crassostrea gigas
UdeM00000034109 6596 2400.9 UdeM00000034109 XP_045177982.1 52.7 2889 1145 21 7 2879 7094 9776 0.00E+00 2400.9 6596 cellular organisms;Eukaryota;Opisthokonta;Metazoa;Eumetazoa;Bilateria;Protostomia;Spiralia;Lophotrochozoa;Mollusca;Bivalvia;Autobranchia;Heteroconchia;Euheterodonta;Imparidentia;Venerida;Veneroidea;Veneridae;Mercenaria;Mercenaria mercenaria
UdeM00000034110 29159 1822.8 UdeM00000034110 XP_011451330.2 57.8 1548 640 6 173 1717 5545 7081 0.00E+00 1822.8 29159 cellular organisms;Eukaryota;Opisthokonta;Metazoa;Eumetazoa;Bilateria;Protostomia;Spiralia;Lophotrochozoa;Mollusca;Bivalvia;Autobranchia;Pteriomorphia;Ostreida;Ostreoidea;Ostreidae;Crassostrea;Crassostrea gigas
UdeM00000034111 6565 795.4 UdeM00000034111 XP_022322952.1 56.8 703 303 1 1 703 4836 5537 5.80E-227 795.4 6565 cellular organisms;Eukaryota;Opisthokonta;Metazoa;Eumetazoa;Bilateria;Protostomia;

Spiralia;Lophotrochozoa;Mollusca;Bivalvia;Autobranchia;Pteriomorphia;Ostreida;Ostreoidea;Ostreidae;Crassostrea;Crassostrea virginica
UdeM00000034112 6565 831.6 UdeM00000034112 XP_022322952.1 56.9 728 313 1 6 733 4811 5537 7.60E-238 831.6 6565 cellular organisms;Eukaryota;Opisthokonta;Metazoa;Eumetazoa;Bilateria;Protostomia;Spiralia;Lophotrochozoa;Mollusca;Bivalvia;Autobranchia;Pteriomorphia;Ostreida;Ostreoidea;Ostreidae;Crassostrea;Crassostrea virginica
UdeM00000034113 1735272 3068.9 UdeM00000034113 XP_041374694.1 58.9 2391 960 6 7 2377 2435 4823 0.00E+00 3068.9 1735272 cellular organisms;Eukaryota;Opisthokonta;Metazoa;Eumetazoa;Bilateria;Protostomia;Spiralia;Lophotrochozoa;Mollusca;Gastropoda;Neomphalina;Neomphaloidea;Peltospiridae;Gigantopelta;Gigantopelta aegis
UdeM00000034114 6565 5623.5 UdeM00000034114 XP_022322952.1 56.5 4768 1934 18 4 4635 54 4815 0.00E+00 5623.5 6565 cellular organisms;Eukaryota;Opisthokonta;Metazoa;Eumetazoa;Bilateria;Protostomia;Spiralia;Lophotrochozoa;Mollusca;Bivalvia;Autobranchia;Pteriomorphia;Ostreida;Ostreoidea;Ostreidae;Crassostrea;Crassostrea virginica
UdeM00000034115 6596 1136.7 UdeM00000034115 XP_045177827.1 77.1 737 168 1 41 777 36 771 0.00E+00 1136.7 6596 cellular organisms;Eukaryota;Opisthokonta;Metazoa;Eumetazoa;Bilateria;Protostomia;Spiralia;Lophotrochozoa;Mollusca;Bivalvia;Autobranchia;Heteroconchia;Euheterodonta;Imparidentia;Venerida;Veneroidea;Veneridae;Mercenaria;Mercenaria mercenaria
UdeM00000034116 6596 73.9 UdeM00000034116 XP_045207416.1 32.1 137 90 1 3 139 1 134 2.40E-10 73.9 6596 cellular organisms;Eukaryota;Opisthokonta;Metazoa;Eumetazoa;Bilateria;Protostomia;Spiralia;Lophotrochozoa;Mollusca;Bivalvia;Autobranchia;Heteroconchia;Euheterodonta;Imparidentia;Venerida;Veneroidea;Veneridae;Mercenaria;Mercenaria mercenaria
UdeM00000034117 6596 73.6 UdeM00000034117 XP_045207416.1 32.4 170 104 4 6 175 4 162 3.20E-10 73.6 6596 cellular organisms;Eukaryota;Opisthokonta;Metazoa;Eumetazoa;Bilateria;Protostomia;Spiralia;Lophotrochozoa;Mollusca;Bivalvia;Autobranchia;Heteroconchia;Euheterodonta;Imparidentia;Venerida;Veneroidea;Veneridae;Mercenaria;Mercenaria mercenaria
UdeM00000034119 7719 70.5 UdeM00000034119 XP_004227365.1 50.9 57 28 0 37 93 106 162 1.30E-09 70.5 7719 cellular organisms;Eukaryota;Opisthokonta;Metazoa;Eumetazoa;Bilateria;Deuterostomia;Chordata;Tunicata;Ascidiacea;Phlebobranchia;Cionidae;Ciona;Ciona intestinalis
UdeM00000034118 7719 70.5 UdeM00000034118 XP_004227365.1 50.9 57 28 0 21 77 106 162 1.10E-09 70.5 7719 cellular organisms;Eukaryota;Opisthokonta;Metazoa;Eumetazoa;Bilateria;Deuterostomia;Chordata;Tunicata;Ascidiacea;Phlebobranchia;Cionidae;Ciona;Ciona intestinalis
UdeM00000034120 7054 112.1 UdeM00000034120 XP_031346849.1 45.2 135 74 0 19 153 33 167 7.40E-22 112.1 7054 cellular organisms;Eukaryota;Opisthokonta;Metazoa;Eumetazoa;Bilateria;Protostomia;Ecdysozoa;Panarthropoda;Arthropoda;Mandibulata;Pancrustacea;Hexapoda;Insecta;Dicondylia;Pterygota;Neoptera;Endopterygota;Coleoptera;Polyphaga;Elateriformia;Elateroidea;Lampyridae;Lampyrinae;Photinus;Photinus pyralis

UdeM00000034121 225164 198.4 UdeM00000034121 XP_009057312.1 62.5
152 57 0 13 164 13 164 7.40E-48 198.4 225164
cellular
organisms;Eukaryota;Opisthokonta;Metazoa;Eumetazoa;Bilateria;Protostomia;
Spiralia;Lophotrochozoa;Mollusca;Gastropoda;Patellogastropoda;Lottioidea;
Lottiidae;Lottia;Lottia gigantea
UdeM00000034122 6579 1022.7 UdeM00000034122 XP_033728096.1 66.4
742 221 4 1 737 1 719 2.30E-295 1022.7 6579
cellular
organisms;Eukaryota;Opisthokonta;Metazoa;Eumetazoa;Bilateria;Protostomia;
Spiralia;Lophotrochozoa;Mollusca;Bivalvia;Autobranchia;Pteriomorphia;Pect
inida;Pectinoidea;Pectinidae;Pecten;Pecten maximus
UdeM00000034123 6573 726.1 UdeM00000034123 XP_021348393.1 48.9 880
184 5 21 899 29 643 5.60E-206 726.1 6573 cellular
organisms;Eukaryota;Opisthokonta;Metazoa;Eumetazoa;Bilateria;Protostomia;
Spiralia;Lophotrochozoa;Mollusca;Bivalvia;Autobranchia;Pteriomorphia;Pect
inida;Pectinoidea;Pectinidae;Mizuhopecten;Mizuhopecten yessoensis
UdeM00000034125 6596 252.7 UdeM00000034125 XP_045177475.1 47.2 324
145 7 2 309 11 324 7.10E-64 252.7 6596 cellular
organisms;Eukaryota;Opisthokonta;Metazoa;Eumetazoa;Bilateria;Protostomia;
Spiralia;Lophotrochozoa;Mollusca;Bivalvia;Autobranchia;Heteroconchia;Euhe
terodonta;Imparidentia;Venerida;Veneroidea;Veneridae;Mercenaria;Mercenari
a mercenaria
UdeM00000034126 6596 263.5 UdeM00000034126 XP_045178904.1 67 188
62 0 4 191 2 189 2.20E-67 263.5 6596 cellular
organisms;Eukaryota;Opisthokonta;Metazoa;Eumetazoa;Bilateria;Protostomia;
Spiralia;Lophotrochozoa;Mollusca;Bivalvia;Autobranchia;Heteroconchia;Euhe
terodonta;Imparidentia;Venerida;Veneroidea;Veneridae;Mercenaria;Mercenari
a mercenaria
UdeM00000034127 6596 339 UdeM00000034127 XP_045180077.1 52.9 363
125 6 26 348 23 379 9.80E-90 339 6596 cellular
organisms;Eukaryota;Opisthokonta;Metazoa;Eumetazoa;Bilateria;Protostomia;
Spiralia;Lophotrochozoa;Mollusca;Bivalvia;Autobranchia;Heteroconchia;Euhe
terodonta;Imparidentia;Venerida;Veneroidea;Veneridae;Mercenaria;Mercenari
a mercenaria
UdeM00000034128 6573 251.9 UdeM00000034128 XP_021366101.1 64.2 165
59 0 1 165 447 611 5.60E-64 251.9 6573 cellular
organisms;Eukaryota;Opisthokonta;Metazoa;Eumetazoa;Bilateria;Protostomia;
Spiralia;Lophotrochozoa;Mollusca;Bivalvia;Autobranchia;Pteriomorphia;Pect
inida;Pectinoidea;Pectinidae;Mizuhopecten;Mizuhopecten yessoensis
UdeM00000034130 6596 252.7 UdeM00000034130 XP_045179257.1 69.9 163
49 0 1 163 48 210 3.20E-64 252.7 6596 cellular
organisms;Eukaryota;Opisthokonta;Metazoa;Eumetazoa;Bilateria;Protostomia;
Spiralia;Lophotrochozoa;Mollusca;Bivalvia;Autobranchia;Heteroconchia;Euhe
terodonta;Imparidentia;Venerida;Veneroidea;Veneridae;Mercenaria;Mercenari
a mercenaria
UdeM00000034129 6596 286.2 UdeM00000034129 XP_045179257.1 64.9 205
71 1 10 214 7 210 3.50E-74 286.2 6596 cellular
organisms;Eukaryota;Opisthokonta;Metazoa;Eumetazoa;Bilateria;Protostomia;
Spiralia;Lophotrochozoa;Mollusca;Bivalvia;Autobranchia;Heteroconchia;Euhe
terodonta;Imparidentia;Venerida;Veneroidea;Veneridae;Mercenaria;Mercenari
a mercenaria
UdeM00000034132 6579 63.9 UdeM00000034132 XP_033745803.1 38.6 88
51 1 17 101 284 371 2.40E-07 63.9 6579 cellular

organisms;Eukaryota;Opisthokonta;Metazoa;Eumetazoa;Bilateria;Protostomia;Spiralia;Lophotrochozoa;Mollusca;Bivalvia;Autobranchia;Pteriomorphia;Pectinida;Pectinoidea;Pectinidae;Pecten;Pecten maximus
UdeM00000034133 29159 129.8 UdeM00000034133 XP_011443561.2 45.5 178 67 4 11 164 10 181 3.20E-27 129.8 29159 cellular organisms;Eukaryota;Opisthokonta;Metazoa;Eumetazoa;Bilateria;Protostomia;Spiralia;Lophotrochozoa;Mollusca;Bivalvia;Autobranchia;Pteriomorphia;Ostreida;Ostreoidea;Ostreidae;Crassostrea;Crassostrea gigas
UdeM00000034134 29159 124.8 UdeM00000034134 XP_011443561.2 45.5 134 49 2 32 141 48 181 8.80E-26 124.8 29159 cellular organisms;Eukaryota;Opisthokonta;Metazoa;Eumetazoa;Bilateria;Protostomia;Spiralia;Lophotrochozoa;Mollusca;Bivalvia;Autobranchia;Pteriomorphia;Ostreida;Ostreoidea;Ostreidae;Crassostrea;Crassostrea gigas
UdeM00000034135 6596 367.9 UdeM00000034135 XP_045201099.1 65.2 250 87 0 25 274 1 250 1.20E-98 367.9 6596 cellular organisms;Eukaryota;Opisthokonta;Metazoa;Eumetazoa;Bilateria;Protostomia;Spiralia;Lophotrochozoa;Mollusca;Bivalvia;Autobranchia;Heteroconchia;Euheterodonta;Imparidentia;Venerida;Veneroidea;Veneridae;Mercenaria;Mercenaria mercenaria
UdeM00000034136 6596 96.7 UdeM00000034136 XP_045188256.1 40.9 132 72 3 145 270 245 376 5.10E-17 96.7 6596 cellular organisms;Eukaryota;Opisthokonta;Metazoa;Eumetazoa;Bilateria;Protostomia;Spiralia;Lophotrochozoa;Mollusca;Bivalvia;Autobranchia;Heteroconchia;Euheterodonta;Imparidentia;Venerida;Veneroidea;Veneridae;Mercenaria;Mercenaria mercenaria
UdeM00000034137 6579 167.5 UdeM00000034137 XP_033729693.1 66.7 105 35 0 1 105 68 172 3.40E-38 167.5 6579 cellular organisms;Eukaryota;Opisthokonta;Metazoa;Eumetazoa;Bilateria;Protostomia;Spiralia;Lophotrochozoa;Mollusca;Bivalvia;Autobranchia;Pteriomorphia;Pectinida;Pectinoidea;Pectinidae;Pecten;Pecten maximus
UdeM00000034138 6573 99.4 UdeM00000034138 XP_021365625.1 47.6 105 55 0 27 131 22 126 6.40E-18 99.4 6573 cellular organisms;Eukaryota;Opisthokonta;Metazoa;Eumetazoa;Bilateria;Protostomia;Spiralia;Lophotrochozoa;Mollusca;Bivalvia;Autobranchia;Pteriomorphia;Pectinida;Pectinoidea;Pectinidae;Mizuhopecten;Mizuhopecten yessoensis
UdeM00000034139 37621 150.6 UdeM00000034139 XP_014677462.1 69.6 92 28 0 1 92 1 92 9.90E-34 150.6 37621 cellular organisms;Eukaryota;Opisthokonta;Metazoa;Eumetazoa;Bilateria;Protostomia;Ecdysozoa;Scalidophora;Priapulida;Priapulimorpha;Priapulimorphida;Priapulidae;Priapulus;Priapulus caudatus
UdeM00000034141 6579 259.2 UdeM00000034141 XP_033754238.1 57.6 217 91 1 1 217 1 216 4.60E-66 259.2 6579 cellular organisms;Eukaryota;Opisthokonta;Metazoa;Eumetazoa;Bilateria;Protostomia;Spiralia;Lophotrochozoa;Mollusca;Bivalvia;Autobranchia;Pteriomorphia;Pectinida;Pectinoidea;Pectinidae;Pecten;Pecten maximus
UdeM00000034143 1529436 147.1 UdeM00000034143 XP_033121366.1 45.9 135 73 0 1 135 85 219 1.70E-32 147.1 1529436 cellular organisms;Eukaryota;Opisthokonta;Metazoa;Eumetazoa;Bilateria;Deuterostomia;Echinodermata;Pelmatozoa;Crinoidea;Articulata;Comatulida;Comatulidae;Comatulinae;Anneissia;Anneissia japonica
UdeM00000034144 29159 228.8 UdeM00000034144 XP_034305523.1 50.5 200 97 2 1 198 737 936 6.60E-57 228.8 29159 cellular organisms;Eukaryota;Opisthokonta;Metazoa;Eumetazoa;Bilateria;Protostomia;

Spiralia;Lophotrochozoa;Mollusca;Bivalvia;Autobranchia;Pteriomorphia;Ostreida;Ostreoidea;Ostreidae;Crassostrea;Crassostrea gigas
UdeM00000034145 37653 88.6 UdeM00000034145 XP_014784938.1 57.1 63 27 0 1 63 300 362 3.70E-15 88.6 37653 cellular organisms;Eukaryota;Opisthokonta;Metazoa;Eumetazoa;Bilateria;Protostomia;Spiralia;Lophotrochozoa;Mollusca;Cephalopoda;Coleoidea;Octopodiformes;Octopoda;Incirrata;Octopodidae;Octopus;Octopus bimaculoides
UdeM00000034146 6573 165.6 UdeM00000034146 XP_021373558.1 54.7 161 70 3 114 273 158 316 8.90E-38 165.6 6573 cellular organisms;Eukaryota;Opisthokonta;Metazoa;Eumetazoa;Bilateria;Protostomia;Spiralia;Lophotrochozoa;Mollusca;Bivalvia;Autobranchia;Pteriomorphia;Pectinida;Pectinoidea;Pectinidae;Mizuhopecten;Mizuhopecten yessoensis
UdeM00000034147 6565 301.2 UdeM00000034147 XP_022314529.1 40 412 162 4 1 327 37 448 1.60E-78 301.2 6565 cellular organisms;Eukaryota;Opisthokonta;Metazoa;Eumetazoa;Bilateria;Protostomia;Spiralia;Lophotrochozoa;Mollusca;Bivalvia;Autobranchia;Pteriomorphia;Ostreida;Ostreoidea;Ostreidae;Crassostrea;Crassostrea virginica
UdeM00000034148 6573 263.8 UdeM00000034148 XP_021342558.1 36 483 255 11 1 442 1 470 3.90E-67 263.8 6573 cellular organisms;Eukaryota;Opisthokonta;Metazoa;Eumetazoa;Bilateria;Protostomia;Spiralia;Lophotrochozoa;Mollusca;Bivalvia;Autobranchia;Pteriomorphia;Pectinida;Pectinoidea;Pectinidae;Mizuhopecten;Mizuhopecten yessoensis
UdeM00000034149 6565 1171 UdeM00000034149 XP_022320464.1 75 853 201 7 1 848 1 846 0.00E+00 1171 6565 cellular organisms;Eukaryota;Opisthokonta;Metazoa;Eumetazoa;Bilateria;Protostomia;Spiralia;Lophotrochozoa;Mollusca;Bivalvia;Autobranchia;Pteriomorphia;Ostreida;Ostreoidea;Ostreidae;Crassostrea;Crassostrea virginica
UdeM00000034150 6565 1163.7 UdeM00000034150 XP_022320464.1 74.5 859 201 8 1 854 1 846 0.00E+00 1163.7 6565 cellular organisms;Eukaryota;Opisthokonta;Metazoa;Eumetazoa;Bilateria;Protostomia;Spiralia;Lophotrochozoa;Mollusca;Bivalvia;Autobranchia;Pteriomorphia;Ostreida;Ostreoidea;Ostreidae;Crassostrea;Crassostrea virginica
UdeM00000034151 6596 399.8 UdeM00000034151 XP_045179125.1 47.8 473 213 9 1 462 1 450 4.70E-108 399.8 6596 cellular organisms;Eukaryota;Opisthokonta;Metazoa;Eumetazoa;Bilateria;Protostomia;Spiralia;Lophotrochozoa;Mollusca;Bivalvia;Autobranchia;Heteroconchia;Euheterodonta;Imparidentia;Venerida;Veneroidea;Veneridae;Mercenaria;Mercenaria mercenaria
UdeM00000034152 6579 3677.9 UdeM00000034152 XP_033727987.1 80.2 2242 428 6 1 2237 1 2230 0.00E+00 3677.9 6579 cellular organisms;Eukaryota;Opisthokonta;Metazoa;Eumetazoa;Bilateria;Protostomia;Spiralia;Lophotrochozoa;Mollusca;Bivalvia;Autobranchia;Pteriomorphia;Pectinida;Pectinoidea;Pectinidae;Pecten;Pecten maximus
UdeM00000034153 6579 884.8 UdeM00000034153 XP_033728365.1 58.9 740 301 1 5 744 39 775 7.60E-254 884.8 6579 cellular organisms;Eukaryota;Opisthokonta;Metazoa;Eumetazoa;Bilateria;Protostomia;Spiralia;Lophotrochozoa;Mollusca;Bivalvia;Autobranchia;Pteriomorphia;Pectinida;Pectinoidea;Pectinidae;Pecten;Pecten maximus
UdeM00000034154 6596 301.6 UdeM00000034154 XP_045177776.1 33.2 639 243 12 1 623 405 875 2.30E-78 301.6 6596 cellular organisms;Eukaryota;Opisthokonta;Metazoa;Eumetazoa;Bilateria;Protostomia;Spiralia;Lophotrochozoa;Mollusca;Bivalvia;Autobranchia;Heteroconchia;Euhe

terodonta;Imparidentia;Venerida;Veneroidea;Veneridae;Mercenaria;Mercenaria mercenaria
UdeM00000034155 45351 86.7 UdeM00000034155 XP_001623471.2 31.9 235 127 7 3 208 6 236 4.00E-14 86.7 45351 cellular organisms;Eukaryota;Opisthokonta;Metazoa;Eumetazoa;Cnidaria;Anthozoa;Hexacorallia;Actiniaria;Edwardsiidae;Nematostella;Nematostella vectensis
UdeM00000034156 6596 534.6 UdeM00000034156 XP_045177700.1 76.5 378 80 4 2 379 3 371 1.10E-148 534.6 6596 cellular organisms;Eukaryota;Opisthokonta;Metazoa;Eumetazoa;Bilateria;Protostomia;Spiralia;Lophotrochozoa;Mollusca;Bivalvia;Autobranchia;Heteroconchia;Euheterodonta;Imparidentia;Venerida;Veneroidea;Veneridae;Mercenaria;Mercenaria mercenaria
UdeM00000034157 6596 815.5 UdeM00000034157 XP_045177696.1 54.2 708 303 4 1 693 7 708 5.30E-233 815.5 6596 cellular organisms;Eukaryota;Opisthokonta;Metazoa;Eumetazoa;Bilateria;Protostomia;Spiralia;Lophotrochozoa;Mollusca;Bivalvia;Autobranchia;Heteroconchia;Euheterodonta;Imparidentia;Venerida;Veneroidea;Veneridae;Mercenaria;Mercenaria mercenaria
UdeM00000034158 6573 147.5 UdeM00000034158 XP_021341696.1 54.6 152 49 5 1 148 483 618 1.30E-32 147.5 6573 cellular organisms;Eukaryota;Opisthokonta;Metazoa;Eumetazoa;Bilateria;Protostomia;Spiralia;Lophotrochozoa;Mollusca;Bivalvia;Autobranchia;Pteriomorphia;Pectinida;Pectinoidea;Pectinidae;Mizuhopecten;Mizuhopecten yessoensis
UdeM00000034159 29159 340.9 UdeM00000034159 XP_034301291.1 37.7 509 294 11 89 579 97 600 3.20E-90 340.9 29159 cellular organisms;Eukaryota;Opisthokonta;Metazoa;Eumetazoa;Bilateria;Protostomia;Spiralia;Lophotrochozoa;Mollusca;Bivalvia;Autobranchia;Pteriomorphia;Ostreida;Ostreoidea;Ostreidae;Crassostrea;Crassostrea gigas
UdeM00000034160 29159 1046.2 UdeM00000034160 XP_034301291.1 54.8 1044 427 18 2 1032 618 1629 3.10E-302 1046.2 29159 cellular organisms;Eukaryota;Opisthokonta;Metazoa;Eumetazoa;Bilateria;Protostomia;Spiralia;Lophotrochozoa;Mollusca;Bivalvia;Autobranchia;Pteriomorphia;Ostreida;Ostreoidea;Ostreidae;Crassostrea;Crassostrea gigas
UdeM00000034161 6596 193.7 UdeM00000034161 XP_045178320.1 24.8 1265 685 41 25 1118 849 2018 1.20E-45 193.7 6596 cellular organisms;Eukaryota;Opisthokonta;Metazoa;Eumetazoa;Bilateria;Protostomia;Spiralia;Lophotrochozoa;Mollusca;Bivalvia;Autobranchia;Heteroconchia;Euheterodonta;Imparidentia;Venerida;Veneroidea;Veneridae;Mercenaria;Mercenaria mercenaria
UdeM00000034162 6596 93.6 UdeM00000034162 XP_045178322.1 41.9 117 62 3 14 126 708 822 4.80E-16 93.6 6596 cellular organisms;Eukaryota;Opisthokonta;Metazoa;Eumetazoa;Bilateria;Protostomia;Spiralia;Lophotrochozoa;Mollusca;Bivalvia;Autobranchia;Heteroconchia;Euheterodonta;Imparidentia;Venerida;Veneroidea;Veneridae;Mercenaria;Mercenaria mercenaria
UdeM00000034164 6526 102.1 UdeM00000034164 XP_013078966.1 26.8 317 223 5 72 379 56 372 1.70E-18 102.1 6526 cellular organisms;Eukaryota;Opisthokonta;Metazoa;Eumetazoa;Bilateria;Protostomia;Spiralia;Lophotrochozoa;Mollusca;Gastropoda;Heterobranchia;Euthyneura;Panpulmonata;Hygrophila;Lymnaeoidea;Planorbidae;Biomphalaria;Biomphalaria glabrata
UdeM00000040543 6596 191 UdeM00000040543 XP_045180677.1 76.3 118 24 1 3 116 1090 1207 2.10E-45 191 6596 cellular

organisms;Eukaryota;Opisthokonta;Metazoa;Eumetazoa;Bilateria;Protostomia;Spiralia;Lophotrochozoa;Mollusca;Bivalvia;Autobranchia;Heteroconchia;Euheterodonta;Imparidentia;Venerida;Veneroidea;Veneridae;Mercenaria;Mercenaria mercenaria
UdeM00000040543 6596 162.2 UdeM00000040543 XP_045180677.1 62.3 154 51 3 143 290 2724 2876 1.00E-36 162.2 6596 cellular organisms;Eukaryota;Opisthokonta;Metazoa;Eumetazoa;Bilateria;Protostomia;Spiralia;Lophotrochozoa;Mollusca;Bivalvia;Autobranchia;Heteroconchia;Euheterodonta;Imparidentia;Venerida;Veneroidea;Veneridae;Mercenaria;Mercenaria mercenaria
UdeM00000040544 6565 146 UdeM00000040544 XP_022289882.1 88 75 9 0 2 76 2700 2774 2.20E-32 146 6565 cellular organisms;Eukaryota;Opisthokonta;Metazoa;Eumetazoa;Bilateria;Protostomia;Spiralia;Lophotrochozoa;Mollusca;Bivalvia;Autobranchia;Pteriomorphia;Ostreida;Ostreoidea;Ostreidae;Crassostrea;Crassostrea virginica
UdeM00000040545 6573 300.1 UdeM00000040545 XP_021377534.1 92.3 156 12 0 1 156 2893 3048 1.70E-78 300.1 6573 cellular organisms;Eukaryota;Opisthokonta;Metazoa;Eumetazoa;Bilateria;Protostomia;Spiralia;Lophotrochozoa;Mollusca;Bivalvia;Autobranchia;Pteriomorphia;Pectinida;Pectinoidea;Pectinidae;Mizuhopecten;Mizuhopecten yessoensis
UdeM00000040546 6565 766.5 UdeM00000040546 XP_022311347.1 59.5 657 248 8 1 656 148 787 2.70E-218 766.5 6565 cellular organisms;Eukaryota;Opisthokonta;Metazoa;Eumetazoa;Bilateria;Protostomia;Spiralia;Lophotrochozoa;Mollusca;Bivalvia;Autobranchia;Pteriomorphia;Ostreida;Ostreoidea;Ostreidae;Crassostrea;Crassostrea virginica
UdeM00000040547 6565 65.5 UdeM00000040547 XP_022316733.1 66.7 39 13 0 1 39 188 226 8.80E-08 65.5 6565 cellular organisms;Eukaryota;Opisthokonta;Metazoa;Eumetazoa;Bilateria;Protostomia;Spiralia;Lophotrochozoa;Mollusca;Bivalvia;Autobranchia;Pteriomorphia;Ostreida;Ostreoidea;Ostreidae;Crassostrea;Crassostrea virginica
UdeM00000040548 6596 96.7 UdeM00000040548 XP_045180822.1 48.5 99 38 2 1 86 1 99 1.80E-17 96.7 6596 cellular organisms;Eukaryota;Opisthokonta;Metazoa;Eumetazoa;Bilateria;Protostomia;Spiralia;Lophotrochozoa;Mollusca;Bivalvia;Autobranchia;Heteroconchia;Euheterodonta;Imparidentia;Venerida;Veneroidea;Veneridae;Mercenaria;Mercenaria mercenaria
UdeM00000040551 2607531 193.7 UdeM00000040551 XP_036371126.1 62.3 130 49 0 2 131 453 582 1.50E-46 193.7 2607531 cellular organisms;Eukaryota;Opisthokonta;Metazoa;Eumetazoa;Bilateria;Protostomia;Spiralia;Lophotrochozoa;Mollusca;Cephalopoda;Coleoidea;Octopodiformes;Octopoda;Incirrata;Octopodidae;Octopus;Octopus sinensis
UdeM00000040552 441366 611.3 UdeM00000040552 XP_028318211.1 56.5 455 197 1 1 454 163 617 1.00E-171 611.3 441366 cellular organisms;Eukaryota;Opisthokonta;Metazoa;Eumetazoa;Bilateria;Deuterostomia;Chordata;Craniata;Vertebrata;Gnathostomata;Teleostomi;Euteleostomi;Actinopterygii;Actinopteri;Neopterygii;Teleostei;Osteoglossocephalai;Clupeocephala;Euteleosteomorpha;Neoteleostei;Eurypterygia;Ctenosquamata;Acanthomorphata;Euacanthomorphacea;Percomorphaceae;Ovalentaria;Blenniimorphae;Blenniiformes;Gobiesocoidei;Gobiesocidae;Gobiesocinae;Gouania;Gouania willdenowi
UdeM00000040553 1735272 255.8 UdeM00000040553 XP_041352907.1 48.2 257 119 4 92 337 93 346 7.90E-65 255.8 1735272

cellular
organisms;Eukaryota;Opisthokonta;Metazoa;Eumetazoa;Bilateria;Protostomia;Spiralia;Lophotrochozoa;Mollusca;Gastropoda;Neomphalina;Neomphaloidea;Peltospiridae;Gigantopelta;Gigantopelta aegis
UdeM00000005100 6565 184.5 UdeM00000005100 XP_022313739.1 65.1 146 47 2 81 223 180 324 1.50E-43 184.5 6565 cellular organisms;Eukaryota;Opisthokonta;Metazoa;Eumetazoa;Bilateria;Protostomia;Spiralia;Lophotrochozoa;Mollusca;Bivalvia;Autobranchia;Pteriomorphia;Ostreida;Ostreoidea;Ostreidae;Crassostrea;Crassostrea virginica
UdeM00000005101 6565 229.9 UdeM00000005101 XP_022317541.1 34.7 714 448 10 9 707 7 717 2.10E-56 229.9 6565 cellular organisms;Eukaryota;Opisthokonta;Metazoa;Eumetazoa;Bilateria;Protostomia;Spiralia;Lophotrochozoa;Mollusca;Bivalvia;Autobranchia;Pteriomorphia;Ostreida;Ostreoidea;Ostreidae;Crassostrea;Crassostrea virginica
UdeM00000005102 6573 840.9 UdeM00000005102 XP_021343599.1 65.5 663 209 7 1 655 1 651 1.10E-240 840.9 6573 cellular organisms;Eukaryota;Opisthokonta;Metazoa;Eumetazoa;Bilateria;Protostomia;Spiralia;Lophotrochozoa;Mollusca;Bivalvia;Autobranchia;Pteriomorphia;Pectinida;Pectinoidea;Pectinidae;Mizuhopecten;Mizuhopecten yessoensis
UdeM00000005103 6565 313.9 UdeM00000005103 XP_022314297.1 53.5 271 125 1 1 271 116 385 2.00E-82 313.9 6565 cellular organisms;Eukaryota;Opisthokonta;Metazoa;Eumetazoa;Bilateria;Protostomia;Spiralia;Lophotrochozoa;Mollusca;Bivalvia;Autobranchia;Pteriomorphia;Ostreida;Ostreoidea;Ostreidae;Crassostrea;Crassostrea virginica
UdeM00000005104 6596 152.9 UdeM00000005104 XP_045158376.1 71.7 99 27 1 14 111 10 108 2.40E-34 152.9 6596 cellular organisms;Eukaryota;Opisthokonta;Metazoa;Eumetazoa;Bilateria;Protostomia;Spiralia;Lophotrochozoa;Mollusca;Bivalvia;Autobranchia;Heteroconchia;Euheterodonta;Imparidentia;Venerida;Veneroidea;Veneridae;Mercenaria;Mercenaria mercenaria
UdeM00000005105 6596 704.1 UdeM00000005105 XP_045158955.1 78.2 440 95 1 1 440 1 439 1.10E-199 704.1 6596 cellular organisms;Eukaryota;Opisthokonta;Metazoa;Eumetazoa;Bilateria;Protostomia;Spiralia;Lophotrochozoa;Mollusca;Bivalvia;Autobranchia;Heteroconchia;Euheterodonta;Imparidentia;Venerida;Veneroidea;Veneridae;Mercenaria;Mercenaria mercenaria
UdeM00000005106 6596 320.5 UdeM00000005106 XP_045158957.1 50.7 341 158 6 2 338 461 795 2.60E-84 320.5 6596 cellular organisms;Eukaryota;Opisthokonta;Metazoa;Eumetazoa;Bilateria;Protostomia;Spiralia;Lophotrochozoa;Mollusca;Bivalvia;Autobranchia;Heteroconchia;Euheterodonta;Imparidentia;Venerida;Veneroidea;Veneridae;Mercenaria;Mercenaria mercenaria
UdeM00000005107 31138 79.3 UdeM00000005107 XP_034615470.1 40.4 114 58 1 26 129 949 1062 4.70E-12 79.3 31138 cellular organisms;Eukaryota;Opisthokonta;Metazoa;Eumetazoa;Bilateria;Deuterostomia;Chordata;Craniata;Vertebrata;Gnathostomata;Teleostomi;Euteleostomi;Sarcopterygii;Dipnotetrapodomorpha;Tetrapoda;Amniota;Sauropsida;Sauria;Archelosauria;Testudinata;Testudines;Cryptodira;Durocryptodira;Testudinoidea;Emydidae;Trachemys;Trachemys scripta;Trachemys scripta elegans
UdeM00000005108 6573 143.7 UdeM00000005108 XP_021363148.1 43.8 320 153 7 23 332 4 306 4.80E-31 143.7 6573 cellular organisms;Eukaryota;Opisthokonta;Metazoa;Eumetazoa;Bilateria;Protostomia;Spiralia;Lophotrochozoa;Mollusca;Bivalvia;Autobranchia;Pteriomorphia;Pectinida;Pectinoidea;Pectinidae;Mizuhopecten;Mizuhopecten yessoensis

UdeM00000005109 6565 126.3 UdeM00000005109 XP_022318049.1 63.5 115
33 3 1 115 1 106 2.50E-26 126.3 6565 cellular
organisms;Eukaryota;Opisthokonta;Metazoa;Eumetazoa;Bilateria;Protostomia;
Spiralia;Lophotrochozoa;Mollusca;Bivalvia;Autobranchia;Pteriomorphia;Ostr
eida;Ostreoidea;Ostreidae;Crassostrea;Crassostrea virginica
UdeM00000005110 6565 110.2 UdeM00000005110 XP_022318050.1 60.7 107
33 3 1 107 8 105 1.70E-21 110.2 6565 cellular
organisms;Eukaryota;Opisthokonta;Metazoa;Eumetazoa;Bilateria;Protostomia;
Spiralia;Lophotrochozoa;Mollusca;Bivalvia;Autobranchia;Pteriomorphia;Ostr
eida;Ostreoidea;Ostreidae;Crassostrea;Crassostrea virginica
UdeM00000005111 6596 238.4 UdeM00000005111 XP_045160421.1 46.3 283
151 1 43 324 3 285 1.30E-59 238.4 6596 cellular
organisms;Eukaryota;Opisthokonta;Metazoa;Eumetazoa;Bilateria;Protostomia;
Spiralia;Lophotrochozoa;Mollusca;Bivalvia;Autobranchia;Heteroconchia;Euhe
terodonta;Imparidentia;Venerida;Veneroidea;Veneridae;Mercenaria;Mercenari
a mercenaria
UdeM00000005112 6596 196.4 UdeM00000005112 XP_045160427.1 45.2 434
210 11 1 425 24 438 7.20E-47 196.4 6596 cellular
organisms;Eukaryota;Opisthokonta;Metazoa;Eumetazoa;Bilateria;Protostomia;
Spiralia;Lophotrochozoa;Mollusca;Bivalvia;Autobranchia;Heteroconchia;Euhe
terodonta;Imparidentia;Venerida;Veneroidea;Veneridae;Mercenaria;Mercenari
a mercenaria
UdeM00000005113 400727 241.1 UdeM00000005113 XP_025109700.1 52.5
219 101 2 48 265 25 241 2.00E-60 241.1 400727
cellular
organisms;Eukaryota;Opisthokonta;Metazoa;Eumetazoa;Bilateria;Protostomia;
Spiralia;Lophotrochozoa;Mollusca;Gastropoda;Caenogastropoda;Architaeniogl
ossa;Ampullarioidea;Ampullariidae;Pomacea;Pomacea canaliculata
UdeM00000005114 6596 132.1 UdeM00000005114 XP_045160951.1 66.3 98
29 2 42 138 979 1073 5.80E-28 132.1 6596 cellular
organisms;Eukaryota;Opisthokonta;Metazoa;Eumetazoa;Bilateria;Protostomia;
Spiralia;Lophotrochozoa;Mollusca;Bivalvia;Autobranchia;Heteroconchia;Euhe
terodonta;Imparidentia;Venerida;Veneroidea;Veneridae;Mercenaria;Mercenari
a mercenaria
UdeM00000005115 6596 131.7 UdeM00000005115 XP_045160951.1 66.3 98
29 2 42 138 979 1073 8.10E-28 131.7 6596 cellular
organisms;Eukaryota;Opisthokonta;Metazoa;Eumetazoa;Bilateria;Protostomia;
Spiralia;Lophotrochozoa;Mollusca;Bivalvia;Autobranchia;Heteroconchia;Euhe
terodonta;Imparidentia;Venerida;Veneroidea;Veneridae;Mercenaria;Mercenari
a mercenaria
UdeM00000005117 6596 79.3 UdeM00000005117 XP_045160951.1 45.8 120
49 5 81 193 862 972 6.90E-12 79.3 6596 cellular
organisms;Eukaryota;Opisthokonta;Metazoa;Eumetazoa;Bilateria;Protostomia;
Spiralia;Lophotrochozoa;Mollusca;Bivalvia;Autobranchia;Heteroconchia;Euhe
terodonta;Imparidentia;Venerida;Veneroidea;Veneridae;Mercenaria;Mercenari
a mercenaria
UdeM00000005116 6596 79.3 UdeM00000005116 XP_045160951.1 45.8 120
49 5 119 231 862 972 8.10E-12 79.3 6596 cellular
organisms;Eukaryota;Opisthokonta;Metazoa;Eumetazoa;Bilateria;Protostomia;
Spiralia;Lophotrochozoa;Mollusca;Bivalvia;Autobranchia;Heteroconchia;Euhe
terodonta;Imparidentia;Venerida;Veneroidea;Veneridae;Mercenaria;Mercenari
a mercenaria
UdeM00000005118 6596 494.6 UdeM00000005118 XP_045159996.1 56.3 449
192 2 1 446 19 466 1.30E-136 494.6 6596 cellular

organisms;Eukaryota;Opisthokonta;Metazoa;Eumetazoa;Bilateria;Protostomia;
Spiralia;Lophotrochozoa;Mollusca;Bivalvia;Autobranchia;Heteroconchia;Euhe
terodonta;Imparidentia;Venerida;Veneroidea;Veneridae;Mercenaria;Mercenari
a mercenaria
UdeM00000005119 6596 295 UdeM00000005119 XP_045160951.1 44.4 396
167 10 1 375 401 764 1.40E-76 295 6596 cellular
organisms;Eukaryota;Opisthokonta;Metazoa;Eumetazoa;Bilateria;Protostomia;
Spiralia;Lophotrochozoa;Mollusca;Bivalvia;Autobranchia;Heteroconchia;Euhe
terodonta;Imparidentia;Venerida;Veneroidea;Veneridae;Mercenaria;Mercenari
a mercenaria
UdeM00000005120 6579 645.6 UdeM00000005120 XP_033738021.1 75 400
100 0 1 400 1 400 4.30E-182 645.6 6579 cellular
organisms;Eukaryota;Opisthokonta;Metazoa;Eumetazoa;Bilateria;Protostomia;
Spiralia;Lophotrochozoa;Mollusca;Bivalvia;Autobranchia;Pteriomorphia;Pect
inida;Pectinoidea;Pectinidae;Pecten;Pecten maximus
UdeM00000005121 400727 327.8 UdeM00000005121 XP_025079053.1 55.7
316 136 2 1 315 1 313 1.50E-86 327.8 400727
cellular
organisms;Eukaryota;Opisthokonta;Metazoa;Eumetazoa;Bilateria;Protostomia;
Spiralia;Lophotrochozoa;Mollusca;Gastropoda;Caenogastropoda;Architaeniogl
ossa;Ampullarioidea;Ampullariidae;Pomacea;Pomacea canaliculata
UdeM00000005122 6596 390.6 UdeM00000005122 XP_045159547.1 30.5 2180
1097 72 42 2121 11 1871 1.30E-104 390.6 6596 cellular
organisms;Eukaryota;Opisthokonta;Metazoa;Eumetazoa;Bilateria;Protostomia;
Spiralia;Lophotrochozoa;Mollusca;Bivalvia;Autobranchia;Heteroconchia;Euhe
terodonta;Imparidentia;Venerida;Veneroidea;Veneridae;Mercenaria;Mercenari
a mercenaria
UdeM00000005123 6596 389 UdeM00000005123 XP_045159547.1 30.4 2152
1086 70 1 2059 11 1843 3.70E-104 389 6596 cellular
organisms;Eukaryota;Opisthokonta;Metazoa;Eumetazoa;Bilateria;Protostomia;
Spiralia;Lophotrochozoa;Mollusca;Bivalvia;Autobranchia;Heteroconchia;Euhe
terodonta;Imparidentia;Venerida;Veneroidea;Veneridae;Mercenaria;Mercenari
a mercenaria
UdeM00000005124 6596 603.6 UdeM00000005124 XP_045160646.1 69.5 410
124 1 50 459 4 412 2.10E-169 603.6 6596 cellular
organisms;Eukaryota;Opisthokonta;Metazoa;Eumetazoa;Bilateria;Protostomia;
Spiralia;Lophotrochozoa;Mollusca;Bivalvia;Autobranchia;Heteroconchia;Euhe
terodonta;Imparidentia;Venerida;Veneroidea;Veneridae;Mercenaria;Mercenari
a mercenaria
UdeM00000005125 1735272 761.5 UdeM00000005125 XP_041357023.1 46.7
836 404 20 1 808 37 858 1.10E-216 761.5 1735272
cellular
organisms;Eukaryota;Opisthokonta;Metazoa;Eumetazoa;Bilateria;Protostomia;
Spiralia;Lophotrochozoa;Mollusca;Gastropoda;Neomphalina;Neomphaloidea;Pel
tospiridae;Gigantopelta;Gigantopelta aegis
UdeM00000005126 6596 887.9 UdeM00000005126 XP_045159523.1 63.5 657
235 3 19 673 7 660 8.20E-255 887.9 6596 cellular
organisms;Eukaryota;Opisthokonta;Metazoa;Eumetazoa;Bilateria;Protostomia;
Spiralia;Lophotrochozoa;Mollusca;Bivalvia;Autobranchia;Heteroconchia;Euhe
terodonta;Imparidentia;Venerida;Veneroidea;Veneridae;Mercenaria;Mercenari
a mercenaria
UdeM00000005127 7574 121.3 UdeM00000005127 XP_013404165.1 53.2 111
52 0 2 112 666 776 8.20E-25 121.3 7574 cellular
organisms;Eukaryota;Opisthokonta;Metazoa;Eumetazoa;Bilateria;Protostomia;

Spiralia;Lophotrochozoa;Brachiopoda;Linguliformea;Lingulata;Lingulida;Linguloidea;Lingulidae;Lingula;Lingula anatina
UdeM00000005128 7574 121.7 UdeM00000005128 XP_013404165.1 53.2 111 52 0 2 112 666 776 7.20E-25 121.7 7574 cellular organisms;Eukaryota;Opisthokonta;Metazoa;Eumetazoa;Bilateria;Protostomia;Spiralia;Lophotrochozoa;Brachiopoda;Linguliformea;Lingulata;Lingulida;Linguloidea;Lingulidae;Lingula;Lingula anatina
UdeM00000005129 6596 1010 UdeM00000005129 XP_045159523.1 61.3 776 294 4 9 781 3 775 1.70E-291 1010 6596 cellular organisms;Eukaryota;Opisthokonta;Metazoa;Eumetazoa;Bilateria;Protostomia;Spiralia;Lophotrochozoa;Mollusca;Bivalvia;Autobranchia;Heteroconchia;Euheterodonta;Imparidentia;Venerida;Veneroidea;Veneridae;Mercenaria;Mercenaria mercenaria
UdeM00000005130 6596 794.7 UdeM00000005130 XP_045160278.1 70.5 563 149 2 140 693 1 555 9.70E-227 794.7 6596 cellular organisms;Eukaryota;Opisthokonta;Metazoa;Eumetazoa;Bilateria;Protostomia;Spiralia;Lophotrochozoa;Mollusca;Bivalvia;Autobranchia;Heteroconchia;Euheterodonta;Imparidentia;Venerida;Veneroidea;Veneridae;Mercenaria;Mercenaria mercenaria
UdeM00000005132 400727 927.2 UdeM00000005132 XP_025111001.1 58.2 1017 399 7 86 1096 70 1066 2.00E-266 927.2 400727 cellular organisms;Eukaryota;Opisthokonta;Metazoa;Eumetazoa;Bilateria;Protostomia;Spiralia;Lophotrochozoa;Mollusca;Gastropoda;Caenogastropoda;Architaenioglossa;Ampullarioidea;Ampullariidae;Pomacea;Pomacea canaliculata
UdeM00000005133 6579 326.2 UdeM00000005133 XP_033738116.1 60 240 96 0 1 240 7 246 3.40E-86 326.2 6579 cellular organisms;Eukaryota;Opisthokonta;Metazoa;Eumetazoa;Bilateria;Protostomia;Spiralia;Lophotrochozoa;Mollusca;Bivalvia;Autobranchia;Pteriomorphia;Pectinida;Pectinoidea;Pectinidae;Pecten;Pecten maximus
UdeM00000005134 6565 170.2 UdeM00000005134 XP_022313153.1 52.8 176 69 4 57 228 55 220 3.00E-39 170.2 6565 cellular organisms;Eukaryota;Opisthokonta;Metazoa;Eumetazoa;Bilateria;Protostomia;Spiralia;Lophotrochozoa;Mollusca;Bivalvia;Autobranchia;Pteriomorphia;Ostreida;Ostreoidea;Ostreidae;Crassostrea;Crassostrea virginica
UdeM00000005135 29159 511.5 UdeM00000005135 XP_034313958.1 55.7 521 201 7 3 506 2 509 6.10E-141 511.5 29159 cellular organisms;Eukaryota;Opisthokonta;Metazoa;Eumetazoa;Bilateria;Protostomia;Spiralia;Lophotrochozoa;Mollusca;Bivalvia;Autobranchia;Pteriomorphia;Ostreida;Ostreoidea;Ostreidae;Crassostrea;Crassostrea gigas
UdeM00000005136 6596 1707.2 UdeM00000005136 XP_045160034.1 74.4 1094 276 4 7 1098 19 1110 0.00E+00 1707.2 6596 cellular organisms;Eukaryota;Opisthokonta;Metazoa;Eumetazoa;Bilateria;Protostomia;Spiralia;Lophotrochozoa;Mollusca;Bivalvia;Autobranchia;Heteroconchia;Euheterodonta;Imparidentia;Venerida;Veneroidea;Veneridae;Mercenaria;Mercenaria mercenaria
UdeM00000005137 6596 291.6 UdeM00000005137 XP_045159157.1 34.9 519 272 15 10 471 35 544 1.90E-75 291.6 6596 cellular organisms;Eukaryota;Opisthokonta;Metazoa;Eumetazoa;Bilateria;Protostomia;Spiralia;Lophotrochozoa;Mollusca;Bivalvia;Autobranchia;Heteroconchia;Euheterodonta;Imparidentia;Venerida;Veneroidea;Veneridae;Mercenaria;Mercenaria mercenaria

UdeM00000005138 6579 278.1 UdeM00000005138 XP_033737835.1 33.6 547
284 17 26 511 56 584 2.30E-71 278.1 6579 cellular
organisms;Eukaryota;Opisthokonta;Metazoa;Eumetazoa;Bilateria;Protostomia;
Spiralia;Lophotrochozoa;Mollusca;Bivalvia;Autobranchia;Pteriomorphia;Pect
inida;Pectinoidea;Pectinidae;Pecten;Pecten maximus
UdeM00000005139 6579 305.8 UdeM00000005139 XP_033737835.1 47.8 335
154 6 124 445 272 598 1.00E-79 305.8 6579 cellular
organisms;Eukaryota;Opisthokonta;Metazoa;Eumetazoa;Bilateria;Protostomia;
Spiralia;Lophotrochozoa;Mollusca;Bivalvia;Autobranchia;Pteriomorphia;Pect
inida;Pectinoidea;Pectinidae;Pecten;Pecten maximus
UdeM00000005141 29159 91.7 UdeM00000005141 XP_034324494.1 35.9 156
92 3 1 155 675 823 9.70E-16 91.7 29159 cellular
organisms;Eukaryota;Opisthokonta;Metazoa;Eumetazoa;Bilateria;Protostomia;
Spiralia;Lophotrochozoa;Mollusca;Bivalvia;Autobranchia;Pteriomorphia;Ostr
eida;Ostreoidea;Ostreidae;Crassostrea;Crassostrea gigas
UdeM00000005142 6579 307.4 UdeM00000005142 XP_033737835.1 51.1 311
136 5 36 338 282 584 2.80E-80 307.4 6579 cellular
organisms;Eukaryota;Opisthokonta;Metazoa;Eumetazoa;Bilateria;Protostomia;
Spiralia;Lophotrochozoa;Mollusca;Bivalvia;Autobranchia;Pteriomorphia;Pect
inida;Pectinoidea;Pectinidae;Pecten;Pecten maximus
UdeM00000005143 6579 307.4 UdeM00000005143 XP_033737835.1 51.1 311
136 5 23 325 282 584 2.70E-80 307.4 6579 cellular
organisms;Eukaryota;Opisthokonta;Metazoa;Eumetazoa;Bilateria;Protostomia;
Spiralia;Lophotrochozoa;Mollusca;Bivalvia;Autobranchia;Pteriomorphia;Pect
inida;Pectinoidea;Pectinidae;Pecten;Pecten maximus
UdeM00000005144 6579 305.4 UdeM00000005144 XP_033737835.1 50.8 311
137 5 36 338 282 584 1.00E-79 305.4 6579 cellular
organisms;Eukaryota;Opisthokonta;Metazoa;Eumetazoa;Bilateria;Protostomia;
Spiralia;Lophotrochozoa;Mollusca;Bivalvia;Autobranchia;Pteriomorphia;Pect
inida;Pectinoidea;Pectinidae;Pecten;Pecten maximus
UdeM00000005145 50429 268.1 UdeM00000005145 XP_022786616.1 78.7 164
35 0 32 195 29 192 9.10E-69 268.1 50429 cellular
organisms;Eukaryota;Opisthokonta;Metazoa;Eumetazoa;Cnidaria;Anthozoa;Hexa
corallia;Scleractinia;Astrocoeniina;Pocilloporidae;Stylophora;Stylophora
pistillata
UdeM00000005146 6596 68.2 UdeM00000005146 XP_045157989.1 32.7 107
69 1 1 104 11 117 9.90E-09 68.2 6596 cellular
organisms;Eukaryota;Opisthokonta;Metazoa;Eumetazoa;Bilateria;Protostomia;
Spiralia;Lophotrochozoa;Mollusca;Bivalvia;Autobranchia;Heteroconchia;Euhe
terodonta;Imparidentia;Venerida;Veneroidea;Veneridae;Mercenaria;Mercenari
a mercenaria
UdeM00000005147 6596 97.8 UdeM00000005147 XP_045157989.1 54.2 96
42 1 3 96 174 269 1.10E-17 97.8 6596 cellular
organisms;Eukaryota;Opisthokonta;Metazoa;Eumetazoa;Bilateria;Protostomia;
Spiralia;Lophotrochozoa;Mollusca;Bivalvia;Autobranchia;Heteroconchia;Euhe
terodonta;Imparidentia;Venerida;Veneroidea;Veneridae;Mercenaria;Mercenari
a mercenaria
UdeM00000005148 6596 195.3 UdeM00000005148 XP_045157989.1 41.3 259
147 3 1 254 11 269 1.10E-46 195.3 6596 cellular
organisms;Eukaryota;Opisthokonta;Metazoa;Eumetazoa;Bilateria;Protostomia;
Spiralia;Lophotrochozoa;Mollusca;Bivalvia;Autobranchia;Heteroconchia;Euhe
terodonta;Imparidentia;Venerida;Veneroidea;Veneridae;Mercenaria;Mercenari
a mercenaria

UdeM00000005149	133434	495.4	UdeM00000005149	XP_022109439.1	74.9
319	80	0	1	319	207	525	5.70E-137	495.4	133434
cellular
organisms;Eukaryota;Opisthokonta;Metazoa;Eumetazoa;Bilateria;Deuterostomia;Echinodermata;Eleutherozoa;Asterozoa;Asteroidea;Valvatacea;Valvatida;Acanthasteridae;Acanthaster;Acanthaster planci
UdeM00000005150	6596	244.2	UdeM00000005150	XP_045158063.1	32.4	534
280	12	23	543	23	488	3.90E-61	244.2	6596	cellular
organisms;Eukaryota;Opisthokonta;Metazoa;Eumetazoa;Bilateria;Protostomia;Spiralia;Lophotrochozoa;Mollusca;Bivalvia;Autobranchia;Heteroconchia;Euheterodonta;Imparidentia;Venerida;Veneroidea;Veneridae;Mercenaria;Mercenaria mercenaria
UdeM00000005152	225164	366.7	UdeM00000005152	XP_009043916.1	90.9
198	17	1	1	198	1	197	1.90E-98	366.7	225164
cellular
organisms;Eukaryota;Opisthokonta;Metazoa;Eumetazoa;Bilateria;Protostomia;Spiralia;Lophotrochozoa;Mollusca;Gastropoda;Patellogastropoda;Lottioidea;Lottiidae;Lottia;Lottia gigantea
UdeM00000005153	6596	699.1	UdeM00000005153	XP_045157372.1	71.7	580
151	10	1	574	1	573	4.60E-198	699.1	6596	cellular
organisms;Eukaryota;Opisthokonta;Metazoa;Eumetazoa;Bilateria;Protostomia;Spiralia;Lophotrochozoa;Mollusca;Bivalvia;Autobranchia;Heteroconchia;Euheterodonta;Imparidentia;Venerida;Veneroidea;Veneridae;Mercenaria;Mercenaria mercenaria
UdeM00000005154	6579	923.3	UdeM00000005154	XP_033737731.1	53	895
329	14	765	1638	629	1452	4.30E-265	923.3	6579	cellular
organisms;Eukaryota;Opisthokonta;Metazoa;Eumetazoa;Bilateria;Protostomia;Spiralia;Lophotrochozoa;Mollusca;Bivalvia;Autobranchia;Pteriomorphia;Pectinida;Pectinoidea;Pectinidae;Pecten;Pecten maximus
UdeM00000005155	6579	945.3	UdeM00000005155	XP_033737731.1	55.8	850
329	13	765	1593	629	1452	1.00E-271	945.3	6579	cellular
organisms;Eukaryota;Opisthokonta;Metazoa;Eumetazoa;Bilateria;Protostomia;Spiralia;Lophotrochozoa;Mollusca;Bivalvia;Autobranchia;Pteriomorphia;Pectinida;Pectinoidea;Pectinidae;Pecten;Pecten maximus
UdeM00000005156	6596	349	UdeM00000005156	XP_045157375.1	82.5	194
34	0	1	194	1	194	1.20E-92	349	6596	cellular
organisms;Eukaryota;Opisthokonta;Metazoa;Eumetazoa;Bilateria;Protostomia;Spiralia;Lophotrochozoa;Mollusca;Bivalvia;Autobranchia;Heteroconchia;Euheterodonta;Imparidentia;Venerida;Veneroidea;Veneridae;Mercenaria;Mercenaria mercenaria
UdeM00000005157	400727	111.3	UdeM00000005157	XP_025108844.1	32.8
436	237	15	2	411	195	600	2.90E-21	111.3	400727
cellular
organisms;Eukaryota;Opisthokonta;Metazoa;Eumetazoa;Bilateria;Protostomia;Spiralia;Lophotrochozoa;Mollusca;Gastropoda;Caenogastropoda;Architaenioglossa;Ampullarioidea;Ampullariidae;Pomacea;Pomacea canaliculata
UdeM00000005158	6573	674.9	UdeM00000005158	XP_021363122.1	46.2	1150
453	26	3	1074	133	1194	1.70E-190	674.9	6573	cellular
organisms;Eukaryota;Opisthokonta;Metazoa;Eumetazoa;Bilateria;Protostomia;Spiralia;Lophotrochozoa;Mollusca;Bivalvia;Autobranchia;Pteriomorphia;Pectinida;Pectinoidea;Pectinidae;Mizuhopecten;Mizuhopecten yessoensis
UdeM00000005159	6579	435.6	UdeM00000005159	XP_033737724.1	78.5	251
53	1	27	277	133	382	4.60E-119	435.6	6579	cellular
organisms;Eukaryota;Opisthokonta;Metazoa;Eumetazoa;Bilateria;Protostomia;

Spiralia;Lophotrochozoa;Mollusca;Bivalvia;Autobranchia;Pteriomorphia;Pectinida;Pectinoidea;Pectinidae;Pecten;Pecten maximus
UdeM00000005160 6596 222.6 UdeM00000005160 XP_045157932.1 72.1 147 41 0 1 147 1 147 4.50E-55 222.6 6596 cellular organisms;Eukaryota;Opisthokonta;Metazoa;Eumetazoa;Bilateria;Protostomia;Spiralia;Lophotrochozoa;Mollusca;Bivalvia;Autobranchia;Heteroconchia;Euheterodonta;Imparidentia;Venerida;Veneroidea;Veneridae;Mercenaria;Mercenaria mercenaria
UdeM00000005161 6573 638.6 UdeM00000005161 XP_021363080.1 43.7 935 380 23 1 889 2 836 1.10E-179 638.6 6573 cellular organisms;Eukaryota;Opisthokonta;Metazoa;Eumetazoa;Bilateria;Protostomia;Spiralia;Lophotrochozoa;Mollusca;Bivalvia;Autobranchia;Pteriomorphia;Pectinida;Pectinoidea;Pectinidae;Mizuhopecten;Mizuhopecten yessoensis
UdeM00000005162 6565 173.7 UdeM00000005162 XP_022319806.1 34.8 290 177 4 22 308 1 281 3.90E-40 173.7 6565 cellular organisms;Eukaryota;Opisthokonta;Metazoa;Eumetazoa;Bilateria;Protostomia;Spiralia;Lophotrochozoa;Mollusca;Bivalvia;Autobranchia;Pteriomorphia;Ostreida;Ostreoidea;Ostreidae;Crassostrea;Crassostrea virginica
UdeM00000005163 6565 303.9 UdeM00000005163 XP_022314461.1 53.7 246 112 2 62 306 64 308 2.30E-79 303.9 6565 cellular organisms;Eukaryota;Opisthokonta;Metazoa;Eumetazoa;Bilateria;Protostomia;Spiralia;Lophotrochozoa;Mollusca;Bivalvia;Autobranchia;Pteriomorphia;Ostreida;Ostreoidea;Ostreidae;Crassostrea;Crassostrea virginica
UdeM00000005164 29159 193.4 UdeM00000005164 XP_034317111.1 52.2 186 87 1 2 185 171 356 2.70E-46 193.4 29159 cellular organisms;Eukaryota;Opisthokonta;Metazoa;Eumetazoa;Bilateria;Protostomia;Spiralia;Lophotrochozoa;Mollusca;Bivalvia;Autobranchia;Pteriomorphia;Ostreida;Ostreoidea;Ostreidae;Crassostrea;Crassostrea gigas
UdeM00000005165 6596 899.4 UdeM00000005165 XP_045157954.1 63.3 660 209 9 12 649 10 658 2.60E-258 899.4 6596 cellular organisms;Eukaryota;Opisthokonta;Metazoa;Eumetazoa;Bilateria;Protostomia;Spiralia;Lophotrochozoa;Mollusca;Bivalvia;Autobranchia;Heteroconchia;Euheterodonta;Imparidentia;Venerida;Veneroidea;Veneridae;Mercenaria;Mercenaria mercenaria
UdeM00000005166 6596 269.6 UdeM00000005166 XP_045184088.1 34.6 471 284 4 3 454 12 477 7.50E-69 269.6 6596 cellular organisms;Eukaryota;Opisthokonta;Metazoa;Eumetazoa;Bilateria;Protostomia;Spiralia;Lophotrochozoa;Mollusca;Bivalvia;Autobranchia;Heteroconchia;Euheterodonta;Imparidentia;Venerida;Veneroidea;Veneridae;Mercenaria;Mercenaria mercenaria
UdeM00000005167 6596 267.7 UdeM00000005167 XP_045184088.1 36.7 428 270 1 26 452 48 475 2.70E-68 267.7 6596 cellular organisms;Eukaryota;Opisthokonta;Metazoa;Eumetazoa;Bilateria;Protostomia;Spiralia;Lophotrochozoa;Mollusca;Bivalvia;Autobranchia;Heteroconchia;Euheterodonta;Imparidentia;Venerida;Veneroidea;Veneridae;Mercenaria;Mercenaria mercenaria
UdeM00000005168 6596 276.2 UdeM00000005168 XP_045184088.1 37.4 439 266 3 26 459 48 482 8.00E-71 276.2 6596 cellular organisms;Eukaryota;Opisthokonta;Metazoa;Eumetazoa;Bilateria;Protostomia;Spiralia;Lophotrochozoa;Mollusca;Bivalvia;Autobranchia;Heteroconchia;Euheterodonta;Imparidentia;Venerida;Veneroidea;Veneridae;Mercenaria;Mercenaria mercenaria
UdeM00000005169 6573 288.1 UdeM00000005169 XP_021360416.1 36.4 431 268 4 27 453 50 478 1.90E-74 288.1 6573 cellular

organisms;Eukaryota;Opisthokonta;Metazoa;Eumetazoa;Bilateria;Protostomia;Spiralia;Lophotrochozoa;Mollusca;Bivalvia;Autobranchia;Pteriomorphia;Pectinida;Pectinoidea;Pectinidae;Mizuhopecten;Mizuhopecten yessoensis
UdeM00000005170 6596 712.6 UdeM00000005170 XP_045160623.1 65.2 537 174 4 19 547 66 597 3.80E-202 712.6 6596 cellular organisms;Eukaryota;Opisthokonta;Metazoa;Eumetazoa;Bilateria;Protostomia;Spiralia;Lophotrochozoa;Mollusca;Bivalvia;Autobranchia;Heteroconchia;Euheterodonta;Imparidentia;Venerida;Veneroidea;Veneridae;Mercenaria;Mercenaria mercenaria
UdeM00000005171 6596 85.5 UdeM00000005171 XP_045159333.1 26.5 306 179 12 158 436 54 340 2.00E-13 85.5 6596 cellular organisms;Eukaryota;Opisthokonta;Metazoa;Eumetazoa;Bilateria;Protostomia;Spiralia;Lophotrochozoa;Mollusca;Bivalvia;Autobranchia;Heteroconchia;Euheterodonta;Imparidentia;Venerida;Veneroidea;Veneridae;Mercenaria;Mercenaria mercenaria
UdeM00000005173 6596 228.4 UdeM00000005173 XP_045159063.1 38.3 347 206 5 3 344 177 520 1.40E-56 228.4 6596 cellular organisms;Eukaryota;Opisthokonta;Metazoa;Eumetazoa;Bilateria;Protostomia;Spiralia;Lophotrochozoa;Mollusca;Bivalvia;Autobranchia;Heteroconchia;Euheterodonta;Imparidentia;Venerida;Veneroidea;Veneridae;Mercenaria;Mercenaria mercenaria
UdeM00000005174 6596 109.4 UdeM00000005174 XP_045159063.1 48 98 48 1 27 121 35 132 3.40E-21 109.4 6596 cellular organisms;Eukaryota;Opisthokonta;Metazoa;Eumetazoa;Bilateria;Protostomia;Spiralia;Lophotrochozoa;Mollusca;Bivalvia;Autobranchia;Heteroconchia;Euheterodonta;Imparidentia;Venerida;Veneroidea;Veneridae;Mercenaria;Mercenaria mercenaria
UdeM00000005175 1735272 307.8 UdeM00000005175 XP_041358755.1 56.5 269 110 2 1 269 1 262 1.40E-80 307.8 1735272 cellular organisms;Eukaryota;Opisthokonta;Metazoa;Eumetazoa;Bilateria;Protostomia;Spiralia;Lophotrochozoa;Mollusca;Gastropoda;Neomphalina;Neomphaloidea;Peltospiridae;Gigantopelta;Gigantopelta aegis
UdeM00000005176 1735272 663.3 UdeM00000005176 XP_041358489.1 52.2 712 315 9 102 792 78 785 3.90E-187 663.3 1735272 cellular organisms;Eukaryota;Opisthokonta;Metazoa;Eumetazoa;Bilateria;Protostomia;Spiralia;Lophotrochozoa;Mollusca;Gastropoda;Neomphalina;Neomphaloidea;Peltospiridae;Gigantopelta;Gigantopelta aegis
UdeM00000005177 29159 104.4 UdeM00000005177 XP_011419361.2 39.7 131 66 3 4 125 3 129 1.80E-19 104.4 29159 cellular organisms;Eukaryota;Opisthokonta;Metazoa;Eumetazoa;Bilateria;Protostomia;Spiralia;Lophotrochozoa;Mollusca;Bivalvia;Autobranchia;Pteriomorphia;Ostreida;Ostreoidea;Ostreidae;Crassostrea;Crassostrea gigas
UdeM00000005178 1735272 504.6 UdeM00000005178 XP_041358411.1 81.7 290 53 0 1 290 1 290 8.50E-140 504.6 1735272 cellular organisms;Eukaryota;Opisthokonta;Metazoa;Eumetazoa;Bilateria;Protostomia;Spiralia;Lophotrochozoa;Mollusca;Gastropoda;Neomphalina;Neomphaloidea;Peltospiridae;Gigantopelta;Gigantopelta aegis
UdeM00000005179 6596 1382.1 UdeM00000005179 XP_045160228.1 78.2 870 190 0 1 870 295 1164 0.00E+00 1382.1 6596 cellular organisms;Eukaryota;Opisthokonta;Metazoa;Eumetazoa;Bilateria;Protostomia;

Spiralia;Lophotrochozoa;Mollusca;Bivalvia;Autobranchia;Heteroconchia;Euheterodonta;Imparidentia;Venerida;Veneroidea;Veneridae;Mercenaria;Mercenaria mercenaria
UdeM00000005180 6596 316.2 UdeM00000005180 XP_045160217.1 71.2 208 60 0 1 208 1 208 3.00E-83 316.2 6596 cellular organisms;Eukaryota;Opisthokonta;Metazoa;Eumetazoa;Bilateria;Protostomia;Spiralia;Lophotrochozoa;Mollusca;Bivalvia;Autobranchia;Heteroconchia;Euheterodonta;Imparidentia;Venerida;Veneroidea;Veneridae;Mercenaria;Mercenaria mercenaria
UdeM00000005182 29159 280.8 UdeM00000005182 XP_019918003.1 70.6 187 55 0 1 187 1 187 1.30E-72 280.8 29159 cellular organisms;Eukaryota;Opisthokonta;Metazoa;Eumetazoa;Bilateria;Protostomia;Spiralia;Lophotrochozoa;Mollusca;Bivalvia;Autobranchia;Pteriomorphia;Ostreida;Ostreoidea;Ostreidae;Crassostrea;Crassostrea gigas
UdeM00000005181 6596 317.4 UdeM00000005181 XP_045160214.1 71.3 209 60 0 1 209 1 209 1.40E-83 317.4 6596 cellular organisms;Eukaryota;Opisthokonta;Metazoa;Eumetazoa;Bilateria;Protostomia;Spiralia;Lophotrochozoa;Mollusca;Bivalvia;Autobranchia;Heteroconchia;Euheterodonta;Imparidentia;Venerida;Veneroidea;Veneridae;Mercenaria;Mercenaria mercenaria
UdeM00000005183 6579 224.2 UdeM00000005183 XP_033737551.1 44.3 323 166 5 883 1195 1381 1699 9.10E-55 224.2 6579 cellular organisms;Eukaryota;Opisthokonta;Metazoa;Eumetazoa;Bilateria;Protostomia;Spiralia;Lophotrochozoa;Mollusca;Bivalvia;Autobranchia;Pteriomorphia;Pectinida;Pectinoidea;Pectinidae;Pecten;Pecten maximus
UdeM00000005185 29159 232.3 UdeM00000005185 XP_019926412.2 52.4 231 103 3 155 383 2 227 1.10E-57 232.3 29159 cellular organisms;Eukaryota;Opisthokonta;Metazoa;Eumetazoa;Bilateria;Protostomia;Spiralia;Lophotrochozoa;Mollusca;Bivalvia;Autobranchia;Pteriomorphia;Ostreida;Ostreoidea;Ostreidae;Crassostrea;Crassostrea gigas
UdeM00000005184 1735272 140.2 UdeM00000005184 XP_041349280.1 38.3 235 126 6 53 277 51 276 5.10E-30 140.2 1735272 cellular organisms;Eukaryota;Opisthokonta;Metazoa;Eumetazoa;Bilateria;Protostomia;Spiralia;Lophotrochozoa;Mollusca;Gastropoda;Neomphalina;Neomphaloidea;Peltospiridae;Gigantopelta;Gigantopelta aegis
UdeM00000005186 6579 188 UdeM00000005186 XP_033761495.1 62.6 139 52 0 8 146 24 162 9.00E-45 188 6579 cellular organisms;Eukaryota;Opisthokonta;Metazoa;Eumetazoa;Bilateria;Protostomia;Spiralia;Lophotrochozoa;Mollusca;Bivalvia;Autobranchia;Pteriomorphia;Pectinida;Pectinoidea;Pectinidae;Pecten;Pecten maximus
UdeM00000005187 80966 184.5 UdeM00000005187 XP_022077561.1 55.8 156 69 0 1 156 116 271 1.00E-43 184.5 80966 cellular organisms;Eukaryota;Opisthokonta;Metazoa;Eumetazoa;Bilateria;Deuterostomia;Chordata;Craniata;Vertebrata;Gnathostomata;Teleostomi;Euteleostomi;Actinopterygii;Actinopteri;Neopterygii;Teleostei;Osteoglossocephalai;Clupeocephala;Euteleosteomorpha;Neoteleostei;Eurypterygia;Ctenosquamata;Acanthomorphata;Euacanthomorphacea;Percomorphaceae;Ovalentaria;Ovalentaria incertae sedis;Pomacentridae;Acanthochromis;Acanthochromis polyacanthus
UdeM00000005188 6573 71.6 UdeM00000005188 XP_021377276.1 35.5 121 69 2 25 136 98 218 1.10E-09 71.6 6573 cellular organisms;Eukaryota;Opisthokonta;Metazoa;Eumetazoa;Bilateria;Protostomia;Spiralia;Lophotrochozoa;Mollusca;Bivalvia;Autobranchia;Pteriomorphia;Pectinida;Pectinoidea;Pectinidae;Mizuhopecten;Mizuhopecten yessoensis

UdeM00000005189	6596	622.1	UdeM00000005189	XP_045160227.1	52.5	628
283	2	1	628	1	613	7.80E-175	622.1	6596	cellular organisms;Eukaryota;Opisthokonta;Metazoa;Eumetazoa;Bilateria;Protostomia;Spiralia;Lophotrochozoa;Mollusca;Bivalvia;Autobranchia;Heteroconchia;Euheterodonta;Imparidentia;Venerida;Veneroidea;Veneridae;Mercenaria;Mercenaria mercenaria
UdeM00000005190	6573	812	UdeM00000005190	XP_021370693.1	71.7	555
147	4	220	766	217	769	6.70E-232	812	6573	cellular organisms;Eukaryota;Opisthokonta;Metazoa;Eumetazoa;Bilateria;Protostomia;Spiralia;Lophotrochozoa;Mollusca;Bivalvia;Autobranchia;Pteriomorphia;Pectinida;Pectinoidea;Pectinidae;Mizuhopecten;Mizuhopecten yessoensis
UdeM00000005191	260615	176.8	UdeM00000005191	XP_039398742.1	74.5
137	11	1	1	113	246	382	1.70E-41	176.8	260615
cellular organisms;Eukaryota;Opisthokonta;Metazoa;Eumetazoa;Bilateria;Deuterostomia;Chordata;Craniata;Vertebrata;Gnathostomata;Teleostomi;Euteleostomi;Sarcopterygii;Dipnotetrapodomorpha;Tetrapoda;Amniota;Sauropsida;Sauria;Archelosauria;Testudinata;Testudines;Cryptodira;Durocryptodira;Testudinoidea;Geoemydidae;Geoemydinae;Mauremys;Mauremys reevesii
UdeM00000005192	6573	657.1	UdeM00000005192	XP_021370781.1	74.5	423
107	1	1	423	1	422	1.50E-185	657.1	6573	cellular organisms;Eukaryota;Opisthokonta;Metazoa;Eumetazoa;Bilateria;Protostomia;Spiralia;Lophotrochozoa;Mollusca;Bivalvia;Autobranchia;Pteriomorphia;Pectinida;Pectinoidea;Pectinidae;Mizuhopecten;Mizuhopecten yessoensis
UdeM00000005193	6579	1382.9	UdeM00000005193	XP_033738813.1	71.9
980	264	5	1	978	895	1865	0.00E+00	1382.9	6579
cellular organisms;Eukaryota;Opisthokonta;Metazoa;Eumetazoa;Bilateria;Protostomia;Spiralia;Lophotrochozoa;Mollusca;Bivalvia;Autobranchia;Pteriomorphia;Pectinida;Pectinoidea;Pectinidae;Pecten;Pecten maximus
UdeM00000005194	218467	105.9	UdeM00000005194	XP_023212519.1	53.3
105	48	1	1	105	1	104	4.70E-20	105.9	218467
cellular organisms;Eukaryota;Opisthokonta;Metazoa;Eumetazoa;Bilateria;Protostomia;Ecdysozoa;Panarthropoda;Arthropoda;Chelicerata;Arachnida;Scorpiones;Buthida;Buthoidea;Buthidae;Centruroides;Centruroides sculpturatus
UdeM00000005195	6573	829.7	UdeM00000005195	XP_021370716.1	59.5	923
305	13	1	913	4	867	3.60E-237	829.7	6573	cellular organisms;Eukaryota;Opisthokonta;Metazoa;Eumetazoa;Bilateria;Protostomia;Spiralia;Lophotrochozoa;Mollusca;Bivalvia;Autobranchia;Pteriomorphia;Pectinida;Pectinoidea;Pectinidae;Mizuhopecten;Mizuhopecten yessoensis
UdeM00000005196	6579	876.3	UdeM00000005196	XP_033738818.1	76.3	540
125	1	2	538	1	540	2.50E-251	876.3	6579	cellular organisms;Eukaryota;Opisthokonta;Metazoa;Eumetazoa;Bilateria;Protostomia;Spiralia;Lophotrochozoa;Mollusca;Bivalvia;Autobranchia;Pteriomorphia;Pectinida;Pectinoidea;Pectinidae;Pecten;Pecten maximus
UdeM00000005197	6579	876.3	UdeM00000005197	XP_033738818.1	76.3	540
125	1	1	537	1	540	2.50E-251	876.3	6579	cellular organisms;Eukaryota;Opisthokonta;Metazoa;Eumetazoa;Bilateria;Protostomia;Spiralia;Lophotrochozoa;Mollusca;Bivalvia;Autobranchia;Pteriomorphia;Pectinida;Pectinoidea;Pectinidae;Pecten;Pecten maximus
UdeM00000005198	6579	557.8	UdeM00000005198	XP_033738819.1	50.8	506
242	3	10	515	40	538	1.50E-155	557.8	6579	cellular organisms;Eukaryota;Opisthokonta;Metazoa;Eumetazoa;Bilateria;Protostomia;

Spiralia;Lophotrochozoa;Mollusca;Bivalvia;Autobranchia;Pteriomorphia;Pectinida;Pectinoidea;Pectinidae;Pecten;Pecten maximus
UdeM00000005199 37653 501.5 UdeM00000005199 XP_014784610.1 60.3 408 148 4 1 395 1 407 9.80E-139 501.5 37653 cellular organisms;Eukaryota;Opisthokonta;Metazoa;Eumetazoa;Bilateria;Protostomia;Spiralia;Lophotrochozoa;Mollusca;Cephalopoda;Coleoidea;Octopodiformes;Octopoda;Incirrata;Octopodidae;Octopus;Octopus bimaculoides
UdeM00000005200 6596 523.5 UdeM00000005200 XP_045160651.1 56 464 196 3 37 498 24 481 3.10E-145 523.5 6596 cellular organisms;Eukaryota;Opisthokonta;Metazoa;Eumetazoa;Bilateria;Protostomia;Spiralia;Lophotrochozoa;Mollusca;Bivalvia;Autobranchia;Heteroconchia;Euheterodonta;Imparidentia;Venerida;Veneroidea;Veneridae;Mercenaria;Mercenaria mercenaria
UdeM00000005201 6526 332.4 UdeM00000005201 XP_013078356.1 52.6 369 98 5 1 363 1 298 7.10E-88 332.4 6526 cellular organisms;Eukaryota;Opisthokonta;Metazoa;Eumetazoa;Bilateria;Protostomia;Spiralia;Lophotrochozoa;Mollusca;Gastropoda;Heterobranchia;Euthyneura;Panpulmonata;Hygrophila;Lymnaeoidea;Planorbidae;Biomphalaria;Biomphalaria glabrata
UdeM00000005202 6526 359.4 UdeM00000005202 XP_013078356.1 63 308 98 4 1 302 1 298 4.50E-96 359.4 6526 cellular organisms;Eukaryota;Opisthokonta;Metazoa;Eumetazoa;Bilateria;Protostomia;Spiralia;Lophotrochozoa;Mollusca;Gastropoda;Heterobranchia;Euthyneura;Panpulmonata;Hygrophila;Lymnaeoidea;Planorbidae;Biomphalaria;Biomphalaria glabrata
UdeM00000005203 1735272 215.3 UdeM00000005203 XP_041359327.1 57.8 185 74 2 5 189 3 183 6.70E-53 215.3 1735272 cellular organisms;Eukaryota;Opisthokonta;Metazoa;Eumetazoa;Bilateria;Protostomia;Spiralia;Lophotrochozoa;Mollusca;Gastropoda;Neomphalina;Neomphaloidea;Peltospiridae;Gigantopelta;Gigantopelta aegis
UdeM00000005204 6579 192.6 UdeM00000005204 XP_033730245.1 40.8 233 134 4 24 254 22 252 6.20E-46 192.6 6579 cellular organisms;Eukaryota;Opisthokonta;Metazoa;Eumetazoa;Bilateria;Protostomia;Spiralia;Lophotrochozoa;Mollusca;Bivalvia;Autobranchia;Pteriomorphia;Pectinida;Pectinoidea;Pectinidae;Pecten;Pecten maximus
UdeM00000005205 6596 148.3 UdeM00000005205 XP_045171942.1 39.6 192 113 3 18 208 16 205 1.10E-32 148.3 6596 cellular organisms;Eukaryota;Opisthokonta;Metazoa;Eumetazoa;Bilateria;Protostomia;Spiralia;Lophotrochozoa;Mollusca;Bivalvia;Autobranchia;Heteroconchia;Euheterodonta;Imparidentia;Venerida;Veneroidea;Veneridae;Mercenaria;Mercenaria mercenaria
UdeM00000005206 6596 201.8 UdeM00000005206 XP_045173451.1 41.2 255 140 6 6 255 8 257 1.00E-48 201.8 6596 cellular organisms;Eukaryota;Opisthokonta;Metazoa;Eumetazoa;Bilateria;Protostomia;Spiralia;Lophotrochozoa;Mollusca;Bivalvia;Autobranchia;Heteroconchia;Euheterodonta;Imparidentia;Venerida;Veneroidea;Veneridae;Mercenaria;Mercenaria mercenaria
UdeM00000005207 6565 130.2 UdeM00000005207 XP_022318637.1 54.9 102 44 1 7 106 3 104 1.60E-27 130.2 6565 cellular organisms;Eukaryota;Opisthokonta;Metazoa;Eumetazoa;Bilateria;Protostomia;Spiralia;Lophotrochozoa;Mollusca;Bivalvia;Autobranchia;Pteriomorphia;Ostreida;Ostreoidea;Ostreidae;Crassostrea;Crassostrea virginica

UdeM00000005208 6579 211.5 UdeM00000005208 XP_033730245.1 42.5 233
130 4 24 254 22 252 1.30E-51 211.5 6579 cellular
organisms;Eukaryota;Opisthokonta;Metazoa;Eumetazoa;Bilateria;Protostomia;
Spiralia;Lophotrochozoa;Mollusca;Bivalvia;Autobranchia;Pteriomorphia;Pect
inida;Pectinoidea;Pectinidae;Pecten;Pecten maximus
UdeM00000005209 6579 204.9 UdeM00000005209 XP_033730245.1 42.9 231
126 5 27 254 25 252 1.20E-49 204.9 6579 cellular
organisms;Eukaryota;Opisthokonta;Metazoa;Eumetazoa;Bilateria;Protostomia;
Spiralia;Lophotrochozoa;Mollusca;Bivalvia;Autobranchia;Pteriomorphia;Pect
inida;Pectinoidea;Pectinidae;Pecten;Pecten maximus
UdeM00000005210 6565 766.9 UdeM00000005210 XP_022318582.1 61.5 702
260 6 1 694 1 700 2.20E-218 766.9 6565 cellular
organisms;Eukaryota;Opisthokonta;Metazoa;Eumetazoa;Bilateria;Protostomia;
Spiralia;Lophotrochozoa;Mollusca;Bivalvia;Autobranchia;Pteriomorphia;Ostr
eida;Ostreoidea;Ostreidae;Crassostrea;Crassostrea virginica
UdeM00000005211 6565 773.1 UdeM00000005211 XP_022318582.1 61.5 709
261 7 1 701 1 705 3.10E-220 773.1 6565 cellular
organisms;Eukaryota;Opisthokonta;Metazoa;Eumetazoa;Bilateria;Protostomia;
Spiralia;Lophotrochozoa;Mollusca;Bivalvia;Autobranchia;Pteriomorphia;Ostr
eida;Ostreoidea;Ostreidae;Crassostrea;Crassostrea virginica
UdeM00000005213 6565 87.8 UdeM00000005213 XP_022313560.1 54.4 79
35 1 193 271 95 172 2.30E-14 87.8 6565 cellular
organisms;Eukaryota;Opisthokonta;Metazoa;Eumetazoa;Bilateria;Protostomia;
Spiralia;Lophotrochozoa;Mollusca;Bivalvia;Autobranchia;Pteriomorphia;Ostr
eida;Ostreoidea;Ostreidae;Crassostrea;Crassostrea virginica
UdeM00000005214 6596 134 UdeM00000005214 XP_045157588.1 58.4 113
45 2 79 189 84 196 1.90E-28 134 6596 cellular
organisms;Eukaryota;Opisthokonta;Metazoa;Eumetazoa;Bilateria;Protostomia;
Spiralia;Lophotrochozoa;Mollusca;Bivalvia;Autobranchia;Heteroconchia;Euhe
terodonta;Imparidentia;Venerida;Veneroidea;Veneridae;Mercenaria;Mercenari
a mercenaria
UdeM00000005215 6596 589 UdeM00000005215 XP_045157590.1 44.4 730
371 11 1 704 6 726 8.20E-165 589 6596 cellular
organisms;Eukaryota;Opisthokonta;Metazoa;Eumetazoa;Bilateria;Protostomia;
Spiralia;Lophotrochozoa;Mollusca;Bivalvia;Autobranchia;Heteroconchia;Euhe
terodonta;Imparidentia;Venerida;Veneroidea;Veneridae;Mercenaria;Mercenari
a mercenaria
UdeM00000005217 6573 120.6 UdeM00000005217 XP_021350883.1 68.3 82
23 2 1 80 23 103 1.10E-24 120.6 6573 cellular
organisms;Eukaryota;Opisthokonta;Metazoa;Eumetazoa;Bilateria;Protostomia;
Spiralia;Lophotrochozoa;Mollusca;Bivalvia;Autobranchia;Pteriomorphia;Pect
inida;Pectinoidea;Pectinidae;Mizuhopecten;Mizuhopecten yessoensis
UdeM00000005218 400727 137.1 UdeM00000005218 XP_025109747.1 66.4
107 31 1 1 107 138 239 1.60E-29 137.1 400727
cellular
organisms;Eukaryota;Opisthokonta;Metazoa;Eumetazoa;Bilateria;Protostomia;
Spiralia;Lophotrochozoa;Mollusca;Gastropoda;Caenogastropoda;Architaeniogl
ossa;Ampullarioidea;Ampullariidae;Pomacea;Pomacea canaliculata
UdeM00000005219 6526 321.6 UdeM00000005219 XP_013089196.1 62.6 396
125 6 32 415 34 418 1.40E-84 321.6 6526 cellular
organisms;Eukaryota;Opisthokonta;Metazoa;Eumetazoa;Bilateria;Protostomia;
Spiralia;Lophotrochozoa;Mollusca;Gastropoda;Heterobranchia;Euthyneura;Pan
pulmonata;Hygrophila;Lymnaeoidea;Planorbidae;Biomphalaria;Biomphalaria
glabrata

UdeM00000005221 6596 117.1 UdeM00000005221 XP_045159813.1 59.1 93
38 0 7 99 145 237 1.30E-23 117.1 6596 cellular
organisms;Eukaryota;Opisthokonta;Metazoa;Eumetazoa;Bilateria;Protostomia;
Spiralia;Lophotrochozoa;Mollusca;Bivalvia;Autobranchia;Heteroconchia;Euhe
terodonta;Imparidentia;Venerida;Veneroidea;Veneridae;Mercenaria;Mercenari
a mercenaria
UdeM00000005223 6596 121.3 UdeM00000005223 XP_045159813.1 50 110
55 0 4 113 364 473 9.60E-25 121.3 6596 cellular
organisms;Eukaryota;Opisthokonta;Metazoa;Eumetazoa;Bilateria;Protostomia;
Spiralia;Lophotrochozoa;Mollusca;Bivalvia;Autobranchia;Heteroconchia;Euhe
terodonta;Imparidentia;Venerida;Veneroidea;Veneridae;Mercenaria;Mercenari
a mercenaria
UdeM00000005224 6596 438.7 UdeM00000005224 XP_045159814.1 52.7 419
194 4 48 464 60 476 9.10E-120 438.7 6596 cellular
organisms;Eukaryota;Opisthokonta;Metazoa;Eumetazoa;Bilateria;Protostomia;
Spiralia;Lophotrochozoa;Mollusca;Bivalvia;Autobranchia;Heteroconchia;Euhe
terodonta;Imparidentia;Venerida;Veneroidea;Veneridae;Mercenaria;Mercenari
a mercenaria
UdeM00000005225 6596 464.5 UdeM00000005225 XP_045159814.1 50.1 483
232 6 1 481 1 476 1.60E-127 464.5 6596 cellular
organisms;Eukaryota;Opisthokonta;Metazoa;Eumetazoa;Bilateria;Protostomia;
Spiralia;Lophotrochozoa;Mollusca;Bivalvia;Autobranchia;Heteroconchia;Euhe
terodonta;Imparidentia;Venerida;Veneroidea;Veneridae;Mercenaria;Mercenari
a mercenaria
UdeM00000005226 6596 463.8 UdeM00000005226 XP_045159814.1 50.3 483
231 6 1 481 1 476 2.70E-127 463.8 6596 cellular
organisms;Eukaryota;Opisthokonta;Metazoa;Eumetazoa;Bilateria;Protostomia;
Spiralia;Lophotrochozoa;Mollusca;Bivalvia;Autobranchia;Heteroconchia;Euhe
terodonta;Imparidentia;Venerida;Veneroidea;Veneridae;Mercenaria;Mercenari
a mercenaria
UdeM00000005227 6573 179.1 UdeM00000005227 XP_021363161.1 38.1 270
154 4 11 271 6 271 7.70E-42 179.1 6573 cellular
organisms;Eukaryota;Opisthokonta;Metazoa;Eumetazoa;Bilateria;Protostomia;
Spiralia;Lophotrochozoa;Mollusca;Bivalvia;Autobranchia;Pteriomorphia;Pect
inida;Pectinoidea;Pectinidae;Mizuhopecten;Mizuhopecten yessoensis
UdeM00000005228 6596 533.9 UdeM00000005228 XP_045160558.1 56.7 432
173 5 1 431 1 419 1.90E-148 533.9 6596 cellular
organisms;Eukaryota;Opisthokonta;Metazoa;Eumetazoa;Bilateria;Protostomia;
Spiralia;Lophotrochozoa;Mollusca;Bivalvia;Autobranchia;Heteroconchia;Euhe
terodonta;Imparidentia;Venerida;Veneroidea;Veneridae;Mercenaria;Mercenari
a mercenaria
UdeM00000005229 1735272 145.6 UdeM00000005229 XP_041358954.1 55
131 58 1 1 131 4 133 4.90E-32 145.6 1735272
cellular
organisms;Eukaryota;Opisthokonta;Metazoa;Eumetazoa;Bilateria;Protostomia;
Spiralia;Lophotrochozoa;Mollusca;Gastropoda;Neomphalina;Neomphaloidea;Pel
tospiridae;Gigantopelta;Gigantopelta aegis
UdeM00000005230 6565 167.5 UdeM00000005230 XP_022308193.1 43.4 205
60 3 1 151 352 554 1.30E-38 167.5 6565 cellular
organisms;Eukaryota;Opisthokonta;Metazoa;Eumetazoa;Bilateria;Protostomia;
Spiralia;Lophotrochozoa;Mollusca;Bivalvia;Autobranchia;Pteriomorphia;Ostr
eida;Ostreoidea;Ostreidae;Crassostrea;Crassostrea virginica
UdeM00000005232 64793 59.7 UdeM00000005232 XP_011690608.1 34.3 102
60 1 40 141 1963 2057 3.50E-06 59.7 64793 cellular

organisms;Eukaryota;Opisthokonta;Metazoa;Eumetazoa;Bilateria;Protostomia;Ecdysozoa;Panarthropoda;Arthropoda;Mandibulata;Pancrustacea;Hexapoda;Insecta;Dicondylia;Pterygota;Neoptera;Endopterygota;Hymenoptera;Apocrita;Aculeata;Formicoidea;Formicidae;Myrmicinae;Blepharidattini;Wasmannia;Wasmannia auropunctata
UdeM00000005233 6573 242.7 UdeM00000005233 XP_021362424.1 48.7 273
128 7 9 276 2 267 5.80E-61 242.7 6573 cellular
organisms;Eukaryota;Opisthokonta;Metazoa;Eumetazoa;Bilateria;Protostomia;Spiralia;Lophotrochozoa;Mollusca;Bivalvia;Autobranchia;Pteriomorphia;Pectinida;Pectinoidea;Pectinidae;Mizuhopecten;Mizuhopecten yessoensis
UdeM00000005234 6596 239.6 UdeM00000005234 XP_045159970.1 52.8 252
117 2 2 252 169 419 4.50E-60 239.6 6596 cellular
organisms;Eukaryota;Opisthokonta;Metazoa;Eumetazoa;Bilateria;Protostomia;Spiralia;Lophotrochozoa;Mollusca;Bivalvia;Autobranchia;Heteroconchia;Euheterodonta;Imparidentia;Venerida;Veneroidea;Veneridae;Mercenaria;Mercenaria mercenaria
UdeM00000005235 6573 394 UdeM00000005235 XP_021373425.1 52.3 478
198 10 1 459 1 467 2.60E-106 394 6573 cellular
organisms;Eukaryota;Opisthokonta;Metazoa;Eumetazoa;Bilateria;Protostomia;Spiralia;Lophotrochozoa;Mollusca;Bivalvia;Autobranchia;Pteriomorphia;Pectinida;Pectinoidea;Pectinidae;Mizuhopecten;Mizuhopecten yessoensis
UdeM00000005236 400727 369.4 UdeM00000005236 XP_025109371.1 52
446 177 10 1 412 1 443 6.10E-99 369.4 400727
cellular
organisms;Eukaryota;Opisthokonta;Metazoa;Eumetazoa;Bilateria;Protostomia;Spiralia;Lophotrochozoa;Mollusca;Gastropoda;Caenogastropoda;Architaenioglossa;Ampullarioidea;Ampullariidae;Pomacea;Pomacea canaliculata
UdeM00000005237 400727 370.2 UdeM00000005237 XP_025109371.1 48.8
496 177 11 1 472 1 443 4.10E-99 370.2 400727
cellular
organisms;Eukaryota;Opisthokonta;Metazoa;Eumetazoa;Bilateria;Protostomia;Spiralia;Lophotrochozoa;Mollusca;Gastropoda;Caenogastropoda;Architaenioglossa;Ampullarioidea;Ampullariidae;Pomacea;Pomacea canaliculata
UdeM00000005238 6596 1560.8 UdeM00000005238 XP_045157497.1 56.7
1347 500 12 1 1282 167 1495 0.00E+00 1560.8 6596
cellular
organisms;Eukaryota;Opisthokonta;Metazoa;Eumetazoa;Bilateria;Protostomia;Spiralia;Lophotrochozoa;Mollusca;Bivalvia;Autobranchia;Heteroconchia;Euheterodonta;Imparidentia;Venerida;Veneroidea;Veneridae;Mercenaria;Mercenaria mercenaria
UdeM00000005244 6565 479.6 UdeM00000005244 XP_022313144.1 51.2 480
227 6 3 479 302 777 4.90E-132 479.6 6565 cellular
organisms;Eukaryota;Opisthokonta;Metazoa;Eumetazoa;Bilateria;Protostomia;Spiralia;Lophotrochozoa;Mollusca;Bivalvia;Autobranchia;Pteriomorphia;Ostreida;Ostreoidea;Ostreidae;Crassostrea;Crassostrea virginica
UdeM00000005245 37621 149.8 UdeM00000005245 XP_014673981.1 43.9 198
71 5 9 169 24 218 3.40E-33 149.8 37621 cellular
organisms;Eukaryota;Opisthokonta;Metazoa;Eumetazoa;Bilateria;Protostomia;Ecdysozoa;Scalidophora;Priapulida;Priapulimorpha;Priapulimorphida;Priapulidae;Priapulus;Priapulus caudatus
UdeM00000005246 37621 210.7 UdeM00000005246 XP_014672567.1 51.7 174
84 0 10 183 345 518 2.00E-51 210.7 37621 cellular
organisms;Eukaryota;Opisthokonta;Metazoa;Eumetazoa;Bilateria;Protostomia;

Ecdysozoa;Scalidophora;Priapulida;Priapulimorpha;Priapulimorphida;Priapulidae;Priapulus;Priapulus caudatus
UdeM00000005247 6596 483.8 UdeM00000005247 XP_045157493.1 76.9 286 66 0 1 286 1 286 1.50E-133 483.8 6596 cellular organisms;Eukaryota;Opisthokonta;Metazoa;Eumetazoa;Bilateria;Protostomia;Spiralia;Lophotrochozoa;Mollusca;Bivalvia;Autobranchia;Heteroconchia;Euheterodonta;Imparidentia;Venerida;Veneroidea;Veneridae;Mercenaria;Mercenaria mercenaria
UdeM00000005248 6579 981.9 UdeM00000005248 XP_033737646.1 47.8 1037 516 6 20 1048 20 1039 6.50E-283 981.9 6579 cellular organisms;Eukaryota;Opisthokonta;Metazoa;Eumetazoa;Bilateria;Protostomia;Spiralia;Lophotrochozoa;Mollusca;Bivalvia;Autobranchia;Pteriomorphia;Pectinida;Pectinoidea;Pectinidae;Pecten;Pecten maximus
UdeM00000005249 225164 352.1 UdeM00000005249 XP_009056741.1 55.2 335 131 2 13 336 60 386 8.50E-94 352.1 225164 cellular organisms;Eukaryota;Opisthokonta;Metazoa;Eumetazoa;Bilateria;Protostomia;Spiralia;Lophotrochozoa;Mollusca;Gastropoda;Patellogastropoda;Lottioidea;Lottiidae;Lottia;Lottia gigantea
UdeM00000005250 6579 494.6 UdeM00000005250 XP_033737646.1 54.2 463 202 3 15 468 20 481 1.40E-136 494.6 6579 cellular organisms;Eukaryota;Opisthokonta;Metazoa;Eumetazoa;Bilateria;Protostomia;Spiralia;Lophotrochozoa;Mollusca;Bivalvia;Autobranchia;Pteriomorphia;Pectinida;Pectinoidea;Pectinidae;Pecten;Pecten maximus
UdeM00000005251 6573 342.8 UdeM00000005251 XP_021359218.1 54.5 297 134 1 1 297 469 764 4.60E-91 342.8 6573 cellular organisms;Eukaryota;Opisthokonta;Metazoa;Eumetazoa;Bilateria;Protostomia;Spiralia;Lophotrochozoa;Mollusca;Bivalvia;Autobranchia;Pteriomorphia;Pectinida;Pectinoidea;Pectinidae;Mizuhopecten;Mizuhopecten yessoensis
UdeM00000005252 6565 126.3 UdeM00000005252 XP_022314272.1 47.5 141 70 3 1 140 1 138 4.10E-26 126.3 6565 cellular organisms;Eukaryota;Opisthokonta;Metazoa;Eumetazoa;Bilateria;Protostomia;Spiralia;Lophotrochozoa;Mollusca;Bivalvia;Autobranchia;Pteriomorphia;Ostreida;Ostreoidea;Ostreidae;Crassostrea;Crassostrea virginica
UdeM00000005254 6565 125.6 UdeM00000005254 XP_022314272.1 53.7 108 50 0 1 108 1 108 5.00E-26 125.6 6565 cellular organisms;Eukaryota;Opisthokonta;Metazoa;Eumetazoa;Bilateria;Protostomia;Spiralia;Lophotrochozoa;Mollusca;Bivalvia;Autobranchia;Pteriomorphia;Ostreida;Ostreoidea;Ostreidae;Crassostrea;Crassostrea virginica
UdeM00000005253 6573 122.1 UdeM00000005253 XP_021359218.1 51.9 108 52 0 1 108 352 459 5.10E-25 122.1 6573 cellular organisms;Eukaryota;Opisthokonta;Metazoa;Eumetazoa;Bilateria;Protostomia;Spiralia;Lophotrochozoa;Mollusca;Bivalvia;Autobranchia;Pteriomorphia;Pectinida;Pectinoidea;Pectinidae;Mizuhopecten;Mizuhopecten yessoensis
UdeM00000005255 6596 447.2 UdeM00000005255 XP_045158333.1 42.4 540 302 4 1 537 517 1050 2.90E-122 447.2 6596 cellular organisms;Eukaryota;Opisthokonta;Metazoa;Eumetazoa;Bilateria;Protostomia;Spiralia;Lophotrochozoa;Mollusca;Bivalvia;Autobranchia;Heteroconchia;Euheterodonta;Imparidentia;Venerida;Veneroidea;Veneridae;Mercenaria;Mercenaria mercenaria
UdeM00000005256 6579 590.5 UdeM00000005256 XP_033737643.1 73.1 372 100 0 5 376 4 375 1.50E-165 590.5 6579 cellular organisms;Eukaryota;Opisthokonta;Metazoa;Eumetazoa;Bilateria;Protostomia;

Spiralia;Lophotrochozoa;Mollusca;Bivalvia;Autobranchia;Pteriomorphia;Pectinida;Pectinoidea;Pectinidae;Pecten;Pecten maximus
UdeM00000005257 6573 424.5 UdeM00000005257 XP_021372819.1 55.7 352 149 4 196 541 56 406 2.10E-115 424.5 6573 cellular organisms;Eukaryota;Opisthokonta;Metazoa;Eumetazoa;Bilateria;Protostomia;Spiralia;Lophotrochozoa;Mollusca;Bivalvia;Autobranchia;Pteriomorphia;Pectinida;Pectinoidea;Pectinidae;Mizuhopecten;Mizuhopecten yessoensis
UdeM00000005258 6596 336.7 UdeM00000005258 XP_045157461.1 63 257 88 2 15 269 4 255 3.30E-89 336.7 6596 cellular organisms;Eukaryota;Opisthokonta;Metazoa;Eumetazoa;Bilateria;Protostomia;Spiralia;Lophotrochozoa;Mollusca;Bivalvia;Autobranchia;Heteroconchia;Euheterodonta;Imparidentia;Venerida;Veneroidea;Veneridae;Mercenaria;Mercenaria mercenaria
UdeM00000005259 6565 185.3 UdeM00000005259 XP_022318671.1 45.6 180 98 0 18 197 10 189 8.30E-44 185.3 6565 cellular organisms;Eukaryota;Opisthokonta;Metazoa;Eumetazoa;Bilateria;Protostomia;Spiralia;Lophotrochozoa;Mollusca;Bivalvia;Autobranchia;Pteriomorphia;Ostreida;Ostreoidea;Ostreidae;Crassostrea;Crassostrea virginica
UdeM00000005260 151771 255.4 UdeM00000005260 XP_028408207.1 49.4 235 117 2 2 236 280 512 8.20E-65 255.4 151771 cellular organisms;Eukaryota;Opisthokonta;Metazoa;Eumetazoa;Cnidaria;Anthozoa;Octocorallia;Alcyonacea;Alcyoniina;Nephtheidae;Dendronephthya;Dendronephthya gigantea
UdeM00000005262 6565 62.4 UdeM00000005262 XP_022290403.1 53 66 23 2 1 66 73 130 3.60E-07 62.4 6565 cellular organisms;Eukaryota;Opisthokonta;Metazoa;Eumetazoa;Bilateria;Protostomia;Spiralia;Lophotrochozoa;Mollusca;Bivalvia;Autobranchia;Pteriomorphia;Ostreida;Ostreoidea;Ostreidae;Crassostrea;Crassostrea virginica
UdeM00000005263 6596 516.5 UdeM00000005263 XP_045157890.1 73.9 348 85 4 1 345 1 345 2.50E-143 516.5 6596 cellular organisms;Eukaryota;Opisthokonta;Metazoa;Eumetazoa;Bilateria;Protostomia;Spiralia;Lophotrochozoa;Mollusca;Bivalvia;Autobranchia;Heteroconchia;Euheterodonta;Imparidentia;Venerida;Veneroidea;Veneridae;Mercenaria;Mercenaria mercenaria
UdeM00000005264 29159 380.9 UdeM00000005264 XP_011428029.1 76.5 234 55 0 1 234 115 348 1.10E-102 380.9 29159 cellular organisms;Eukaryota;Opisthokonta;Metazoa;Eumetazoa;Bilateria;Protostomia;Spiralia;Lophotrochozoa;Mollusca;Bivalvia;Autobranchia;Pteriomorphia;Ostreida;Ostreoidea;Ostreidae;Crassostrea;Crassostrea gigas
UdeM00000005266 6565 233.4 UdeM00000005266 XP_022314854.1 26.7 746 458 32 23 729 28 723 1.50E-57 233.4 6565 cellular organisms;Eukaryota;Opisthokonta;Metazoa;Eumetazoa;Bilateria;Protostomia;Spiralia;Lophotrochozoa;Mollusca;Bivalvia;Autobranchia;Pteriomorphia;Ostreida;Ostreoidea;Ostreidae;Crassostrea;Crassostrea virginica
UdeM00000005267 6565 218.4 UdeM00000005267 XP_022314854.1 28.4 630 383 25 46 650 8 594 2.70E-53 218.4 6565 cellular organisms;Eukaryota;Opisthokonta;Metazoa;Eumetazoa;Bilateria;Protostomia;Spiralia;Lophotrochozoa;Mollusca;Bivalvia;Autobranchia;Pteriomorphia;Ostreida;Ostreoidea;Ostreidae;Crassostrea;Crassostrea virginica
UdeM00000005268 6565 219.9 UdeM00000005268 XP_022314854.1 28.7 627 384 24 46 649 8 594 9.30E-54 219.9 6565 cellular organisms;Eukaryota;Opisthokonta;Metazoa;Eumetazoa;Bilateria;Protostomia;

Spiralia;Lophotrochozoa;Mollusca;Bivalvia;Autobranchia;Pteriomorphia;Ostreida;Ostreoidea;Ostreidae;Crassostrea;Crassostrea virginica
UdeM00000005271 6579 218.8 UdeM00000005271 XP_033738178.1 26 731 441 29 2 699 9 672 3.20E-53 218.8 6579 cellular organisms;Eukaryota;Opisthokonta;Metazoa;Eumetazoa;Bilateria;Protostomia;Spiralia;Lophotrochozoa;Mollusca;Bivalvia;Autobranchia;Pteriomorphia;Pectinida;Pectinoidea;Pectinidae;Pecten;Pecten maximus
UdeM00000005272 6565 237.3 UdeM00000005272 XP_022314854.1 28.5 720 434 29 34 721 28 698 9.60E-59 237.3 6565 cellular organisms;Eukaryota;Opisthokonta;Metazoa;Eumetazoa;Bilateria;Protostomia;Spiralia;Lophotrochozoa;Mollusca;Bivalvia;Autobranchia;Pteriomorphia;Ostreida;Ostreoidea;Ostreidae;Crassostrea;Crassostrea virginica
UdeM00000005273 6596 495 UdeM00000005273 XP_045159893.1 61.8 382 141 3 8 384 9 390 9.20E-137 495 6596 cellular organisms;Eukaryota;Opisthokonta;Metazoa;Eumetazoa;Bilateria;Protostomia;Spiralia;Lophotrochozoa;Mollusca;Bivalvia;Autobranchia;Heteroconchia;Euheterodonta;Imparidentia;Venerida;Veneroidea;Veneridae;Mercenaria;Mercenaria mercenaria
UdeM00000005274 6596 272.3 UdeM00000005274 XP_045156993.1 48.3 360 163 6 1 344 2 354 9.50E-70 272.3 6596 cellular organisms;Eukaryota;Opisthokonta;Metazoa;Eumetazoa;Bilateria;Protostomia;Spiralia;Lophotrochozoa;Mollusca;Bivalvia;Autobranchia;Heteroconchia;Euheterodonta;Imparidentia;Venerida;Veneroidea;Veneridae;Mercenaria;Mercenaria mercenaria
UdeM00000005276 114398 428.7 UdeM00000005276 XP_042903861.1 39.1 609 360 6 1 605 1 602 1.20E-116 428.7 114398 cellular organisms;Eukaryota;Opisthokonta;Metazoa;Eumetazoa;Bilateria;Protostomia;Ecdysozoa;Panarthropoda;Arthropoda;Chelicerata;Arachnida;Araneae;Araneomorphae;Entelegynae;Orbiculariae;Araneoidea;Theridiidae;Parasteatoda;Parasteatoda tepidariorum
UdeM00000005277 400727 198.7 UdeM00000005277 XP_025110918.1 65.1 146 50 1 1 145 28 173 4.90E-48 198.7 400727 cellular organisms;Eukaryota;Opisthokonta;Metazoa;Eumetazoa;Bilateria;Protostomia;Spiralia;Lophotrochozoa;Mollusca;Gastropoda;Caenogastropoda;Architaenioglossa;Ampullarioidea;Ampullariidae;Pomacea;Pomacea canaliculata
UdeM00000005278 29159 532.3 UdeM00000005278 XP_019926413.2 53.5 559 142 4 4 445 52 609 6.30E-148 532.3 29159 cellular organisms;Eukaryota;Opisthokonta;Metazoa;Eumetazoa;Bilateria;Protostomia;Spiralia;Lophotrochozoa;Mollusca;Bivalvia;Autobranchia;Pteriomorphia;Ostreida;Ostreoidea;Ostreidae;Crassostrea;Crassostrea gigas
UdeM00000005279 6596 1529.6 UdeM00000005279 XP_045158550.1 36.8 3458 1558 62 6 2928 6 3371 0.00E+00 1529.6 6596 cellular organisms;Eukaryota;Opisthokonta;Metazoa;Eumetazoa;Bilateria;Protostomia;Spiralia;Lophotrochozoa;Mollusca;Bivalvia;Autobranchia;Heteroconchia;Euheterodonta;Imparidentia;Venerida;Veneroidea;Veneridae;Mercenaria;Mercenaria mercenaria
UdeM00000005281 6565 82.4 UdeM00000005281 XP_022335959.1 41.9 93 46 3 15 104 681 768 3.70E-13 82.4 6565 cellular organisms;Eukaryota;Opisthokonta;Metazoa;Eumetazoa;Bilateria;Protostomia;Spiralia;Lophotrochozoa;Mollusca;Bivalvia;Autobranchia;Pteriomorphia;Ostreida;Ostreoidea;Ostreidae;Crassostrea;Crassostrea virginica

UdeM00000005282 6596 186.4 UdeM00000005282 XP_045158553.1 38.5 291
142 8 49 302 80 370 5.70E-44 186.4 6596 cellular
organisms;Eukaryota;Opisthokonta;Metazoa;Eumetazoa;Bilateria;Protostomia;
Spiralia;Lophotrochozoa;Mollusca;Bivalvia;Autobranchia;Heteroconchia;Euhe
terodonta;Imparidentia;Venerida;Veneroidea;Veneridae;Mercenaria;Mercenari
a mercenaria
UdeM00000005283 6596 227.6 UdeM00000005283 XP_045157040.1 48.7 238
108 4 5 229 118 354 1.60E-56 227.6 6596 cellular
organisms;Eukaryota;Opisthokonta;Metazoa;Eumetazoa;Bilateria;Protostomia;
Spiralia;Lophotrochozoa;Mollusca;Bivalvia;Autobranchia;Heteroconchia;Euhe
terodonta;Imparidentia;Venerida;Veneroidea;Veneridae;Mercenaria;Mercenari
a mercenaria
UdeM00000005284 6596 228.4 UdeM00000005284 XP_045157040.1 48.5 239
113 4 5 235 118 354 9.70E-57 228.4 6596 cellular
organisms;Eukaryota;Opisthokonta;Metazoa;Eumetazoa;Bilateria;Protostomia;
Spiralia;Lophotrochozoa;Mollusca;Bivalvia;Autobranchia;Heteroconchia;Euhe
terodonta;Imparidentia;Venerida;Veneroidea;Veneridae;Mercenaria;Mercenari
a mercenaria
UdeM00000005285 6596 310.1 UdeM00000005285 XP_045158553.1 57.9 252
104 1 26 275 110 361 6.70E-81 310.1 6596 cellular
organisms;Eukaryota;Opisthokonta;Metazoa;Eumetazoa;Bilateria;Protostomia;
Spiralia;Lophotrochozoa;Mollusca;Bivalvia;Autobranchia;Heteroconchia;Euhe
terodonta;Imparidentia;Venerida;Veneroidea;Veneridae;Mercenaria;Mercenari
a mercenaria
UdeM00000005286 6596 317.8 UdeM00000005286 XP_045157040.1 47.1 331
159 4 17 332 25 354 1.70E-83 317.8 6596 cellular
organisms;Eukaryota;Opisthokonta;Metazoa;Eumetazoa;Bilateria;Protostomia;
Spiralia;Lophotrochozoa;Mollusca;Bivalvia;Autobranchia;Heteroconchia;Euhe
terodonta;Imparidentia;Venerida;Veneroidea;Veneridae;Mercenaria;Mercenari
a mercenaria
UdeM00000005287 6596 298.9 UdeM00000005287 XP_045158432.1 49.4 328
152 5 24 339 26 351 8.40E-78 298.9 6596 cellular
organisms;Eukaryota;Opisthokonta;Metazoa;Eumetazoa;Bilateria;Protostomia;
Spiralia;Lophotrochozoa;Mollusca;Bivalvia;Autobranchia;Heteroconchia;Euhe
terodonta;Imparidentia;Venerida;Veneroidea;Veneridae;Mercenaria;Mercenari
a mercenaria
UdeM00000005288 6596 317 UdeM00000005288 XP_045157040.1 47.7 331
157 4 23 338 25 354 2.90E-83 317 6596 cellular
organisms;Eukaryota;Opisthokonta;Metazoa;Eumetazoa;Bilateria;Protostomia;
Spiralia;Lophotrochozoa;Mollusca;Bivalvia;Autobranchia;Heteroconchia;Euhe
terodonta;Imparidentia;Venerida;Veneroidea;Veneridae;Mercenaria;Mercenari
a mercenaria
UdeM00000005289 29159 288.5 UdeM00000005289 XP_019926217.2 46.1 343
150 7 324 650 25 348 2.20E-74 288.5 29159 cellular
organisms;Eukaryota;Opisthokonta;Metazoa;Eumetazoa;Bilateria;Protostomia;
Spiralia;Lophotrochozoa;Mollusca;Bivalvia;Autobranchia;Pteriomorphia;Ostr
eida;Ostreoidea;Ostreidae;Crassostrea;Crassostrea gigas
UdeM00000005290 6596 304.3 UdeM00000005290 XP_045157040.1 47.3 332
159 5 2 318 22 352 1.90E-79 304.3 6596 cellular
organisms;Eukaryota;Opisthokonta;Metazoa;Eumetazoa;Bilateria;Protostomia;
Spiralia;Lophotrochozoa;Mollusca;Bivalvia;Autobranchia;Heteroconchia;Euhe
terodonta;Imparidentia;Venerida;Veneroidea;Veneridae;Mercenaria;Mercenari
a mercenaria

UdeM00000005291 6596 96.7 UdeM00000005291 XP_045157040.1 59 83
33 1 85 167 119 200 3.00E-17 96.7 6596 cellular
organisms;Eukaryota;Opisthokonta;Metazoa;Eumetazoa;Bilateria;Protostomia;
Spiralia;Lophotrochozoa;Mollusca;Bivalvia;Autobranchia;Heteroconchia;Euhe
terodonta;Imparidentia;Venerida;Veneroidea;Veneridae;Mercenaria;Mercenari
a mercenaria
UdeM00000005292 29159 61.2 UdeM00000005292 XP_034315424.1 36.4 107
61 3 12 115 213 315 1.00E-06 61.2 29159 cellular
organisms;Eukaryota;Opisthokonta;Metazoa;Eumetazoa;Bilateria;Protostomia;
Spiralia;Lophotrochozoa;Mollusca;Bivalvia;Autobranchia;Pteriomorphia;Ostr
eida;Ostreoidea;Ostreidae;Crassostrea;Crassostrea gigas
UdeM00000005293 6565 144.8 UdeM00000005293 XP_022306954.1 51.7 116
56 0 1 116 833 948 6.90E-32 144.8 6565 cellular
organisms;Eukaryota;Opisthokonta;Metazoa;Eumetazoa;Bilateria;Protostomia;
Spiralia;Lophotrochozoa;Mollusca;Bivalvia;Autobranchia;Pteriomorphia;Ostr
eida;Ostreoidea;Ostreidae;Crassostrea;Crassostrea virginica
UdeM00000005294 29159 84.7 UdeM00000005294 XP_034319609.1 57.4 61
26 0 86 146 894 954 1.10E-13 84.7 29159 cellular
organisms;Eukaryota;Opisthokonta;Metazoa;Eumetazoa;Bilateria;Protostomia;
Spiralia;Lophotrochozoa;Mollusca;Bivalvia;Autobranchia;Pteriomorphia;Ostr
eida;Ostreoidea;Ostreidae;Crassostrea;Crassostrea gigas
UdeM00000005295 29159 103.6 UdeM00000005295 XP_034318948.1 40.2 132
77 1 1 130 686 817 1.90E-19 103.6 29159 cellular
organisms;Eukaryota;Opisthokonta;Metazoa;Eumetazoa;Bilateria;Protostomia;
Spiralia;Lophotrochozoa;Mollusca;Bivalvia;Autobranchia;Pteriomorphia;Ostr
eida;Ostreoidea;Ostreidae;Crassostrea;Crassostrea gigas
UdeM00000005297 29159 194.9 UdeM00000005297 XP_034315421.1 51.9 206
93 3 194 395 271 474 1.90E-46 194.9 29159 cellular
organisms;Eukaryota;Opisthokonta;Metazoa;Eumetazoa;Bilateria;Protostomia;
Spiralia;Lophotrochozoa;Mollusca;Bivalvia;Autobranchia;Pteriomorphia;Ostr
eida;Ostreoidea;Ostreidae;Crassostrea;Crassostrea gigas
UdeM00000005298 6596 306.2 UdeM00000005298 XP_045157040.1 47.8 339
160 5 5 328 18 354 5.00E-80 306.2 6596 cellular
organisms;Eukaryota;Opisthokonta;Metazoa;Eumetazoa;Bilateria;Protostomia;
Spiralia;Lophotrochozoa;Mollusca;Bivalvia;Autobranchia;Heteroconchia;Euhe
terodonta;Imparidentia;Venerida;Veneroidea;Veneridae;Mercenaria;Mercenari
a mercenaria
UdeM00000005300 6596 316.6 UdeM00000005300 XP_045158432.1 50.5 333
146 6 23 339 22 351 4.40E-83 316.6 6596 cellular
organisms;Eukaryota;Opisthokonta;Metazoa;Eumetazoa;Bilateria;Protostomia;
Spiralia;Lophotrochozoa;Mollusca;Bivalvia;Autobranchia;Heteroconchia;Euhe
terodonta;Imparidentia;Venerida;Veneroidea;Veneridae;Mercenaria;Mercenari
a mercenaria
UdeM00000005301 6596 285 UdeM00000005301 XP_045158432.1 50.7 304
135 5 36 327 51 351 1.20E-73 285 6596 cellular
organisms;Eukaryota;Opisthokonta;Metazoa;Eumetazoa;Bilateria;Protostomia;
Spiralia;Lophotrochozoa;Mollusca;Bivalvia;Autobranchia;Heteroconchia;Euhe
terodonta;Imparidentia;Venerida;Veneroidea;Veneridae;Mercenaria;Mercenari
a mercenaria
UdeM00000005302 6565 516.2 UdeM00000005302 XP_022320668.1 59.5 405
164 0 23 427 310 714 1.60E-142 516.2 6565 cellular
organisms;Eukaryota;Opisthokonta;Metazoa;Eumetazoa;Bilateria;Protostomia;
Spiralia;Lophotrochozoa;Mollusca;Bivalvia;Autobranchia;Pteriomorphia;Ostr
eida;Ostreoidea;Ostreidae;Crassostrea;Crassostrea virginica

UdeM00000005304 6596 118.2 UdeM00000005304 XP_045157040.1 42.6 122
61 2 12 125 234 354 7.80E-24 118.2 6596 cellular
organisms;Eukaryota;Opisthokonta;Metazoa;Eumetazoa;Bilateria;Protostomia;
Spiralia;Lophotrochozoa;Mollusca;Bivalvia;Autobranchia;Heteroconchia;Euhe
terodonta;Imparidentia;Venerida;Veneroidea;Veneridae;Mercenaria;Mercenari
a mercenaria
UdeM00000005305 6596 93.2 UdeM00000005305 XP_045216961.1 26.6 379
237 14 395 751 417 776 2.60E-15 93.2 6596 cellular
organisms;Eukaryota;Opisthokonta;Metazoa;Eumetazoa;Bilateria;Protostomia;
Spiralia;Lophotrochozoa;Mollusca;Bivalvia;Autobranchia;Heteroconchia;Euhe
terodonta;Imparidentia;Venerida;Veneroidea;Veneridae;Mercenaria;Mercenari
a mercenaria
UdeM00000005307 6596 317.4 UdeM00000005307 XP_045157040.1 45.8 354
173 5 3 338 2 354 2.20E-83 317.4 6596 cellular
organisms;Eukaryota;Opisthokonta;Metazoa;Eumetazoa;Bilateria;Protostomia;
Spiralia;Lophotrochozoa;Mollusca;Bivalvia;Autobranchia;Heteroconchia;Euhe
terodonta;Imparidentia;Venerida;Veneroidea;Veneridae;Mercenaria;Mercenari
a mercenaria
UdeM00000005308 6596 291.2 UdeM00000005308 XP_045158432.1 49.2 329
149 7 32 345 26 351 1.80E-75 291.2 6596 cellular
organisms;Eukaryota;Opisthokonta;Metazoa;Eumetazoa;Bilateria;Protostomia;
Spiralia;Lophotrochozoa;Mollusca;Bivalvia;Autobranchia;Heteroconchia;Euhe
terodonta;Imparidentia;Venerida;Veneroidea;Veneridae;Mercenaria;Mercenari
a mercenaria
UdeM00000005309 6596 226.9 UdeM00000005309 XP_045157040.1 48.7 232
105 3 2 220 122 352 2.70E-56 226.9 6596 cellular
organisms;Eukaryota;Opisthokonta;Metazoa;Eumetazoa;Bilateria;Protostomia;
Spiralia;Lophotrochozoa;Mollusca;Bivalvia;Autobranchia;Heteroconchia;Euhe
terodonta;Imparidentia;Venerida;Veneroidea;Veneridae;Mercenaria;Mercenari
a mercenaria
UdeM00000005310 6596 170.2 UdeM00000005310 XP_045157040.1 54.7 159
66 2 27 180 119 276 2.40E-39 170.2 6596 cellular
organisms;Eukaryota;Opisthokonta;Metazoa;Eumetazoa;Bilateria;Protostomia;
Spiralia;Lophotrochozoa;Mollusca;Bivalvia;Autobranchia;Heteroconchia;Euhe
terodonta;Imparidentia;Venerida;Veneroidea;Veneridae;Mercenaria;Mercenari
a mercenaria
UdeM00000005311 6596 307 UdeM00000005311 XP_045158553.1 57.5 252
105 1 8 257 110 361 5.50E-80 307 6596 cellular
organisms;Eukaryota;Opisthokonta;Metazoa;Eumetazoa;Bilateria;Protostomia;
Spiralia;Lophotrochozoa;Mollusca;Bivalvia;Autobranchia;Heteroconchia;Euhe
terodonta;Imparidentia;Venerida;Veneroidea;Veneridae;Mercenaria;Mercenari
a mercenaria
UdeM00000005312 1735272 72.8 UdeM00000005312 XP_041347149.1 50.6
87 36 3 1 82 257 341 3.30E-10 72.8 1735272
cellular
organisms;Eukaryota;Opisthokonta;Metazoa;Eumetazoa;Bilateria;Protostomia;
Spiralia;Lophotrochozoa;Mollusca;Gastropoda;Neomphalina;Neomphaloidea;Pel
tospiridae;Gigantopelta;Gigantopelta aegis
UdeM00000005313 37621 181.8 UdeM00000005313 XP_014676995.1 58.2 141
56 2 1 141 1 138 6.10E-43 181.8 37621 cellular
organisms;Eukaryota;Opisthokonta;Metazoa;Eumetazoa;Bilateria;Protostomia;
Ecdysozoa;Scalidophora;Priapulida;Priapulimorpha;Priapulimorphida;Priapul
idae;Priapulus;Priapulus caudatus

UdeM00000005314 29159 181.8 UdeM00000005314 XP_019926217.2 42.5 247
93 5 21 221 11 254 1.20E-42 181.8 29159 cellular
organisms;Eukaryota;Opisthokonta;Metazoa;Eumetazoa;Bilateria;Protostomia;
Spiralia;Lophotrochozoa;Mollusca;Bivalvia;Autobranchia;Pteriomorphia;Ostr
eida;Ostreoidea;Ostreidae;Crassostrea;Crassostrea gigas
UdeM00000005315 6596 316.6 UdeM00000005315 XP_045157040.1 49.1 330
152 4 26 340 26 354 3.80E-83 316.6 6596 cellular
organisms;Eukaryota;Opisthokonta;Metazoa;Eumetazoa;Bilateria;Protostomia;
Spiralia;Lophotrochozoa;Mollusca;Bivalvia;Autobranchia;Heteroconchia;Euhe
terodonta;Imparidentia;Venerida;Veneroidea;Veneridae;Mercenaria;Mercenari
a mercenaria
UdeM00000005316 6596 95.5 UdeM00000005316 XP_045158553.1 48.8 121
60 1 22 142 94 212 6.10E-17 95.5 6596 cellular
organisms;Eukaryota;Opisthokonta;Metazoa;Eumetazoa;Bilateria;Protostomia;
Spiralia;Lophotrochozoa;Mollusca;Bivalvia;Autobranchia;Heteroconchia;Euhe
terodonta;Imparidentia;Venerida;Veneroidea;Veneridae;Mercenaria;Mercenari
a mercenaria
UdeM00000005318 6579 148.7 UdeM00000005318 XP_033738723.1 59.4 128
48 2 4 128 106 232 6.30E-33 148.7 6579 cellular
organisms;Eukaryota;Opisthokonta;Metazoa;Eumetazoa;Bilateria;Protostomia;
Spiralia;Lophotrochozoa;Mollusca;Bivalvia;Autobranchia;Pteriomorphia;Pect
inida;Pectinoidea;Pectinidae;Pecten;Pecten maximus
UdeM00000005319 6579 147.5 UdeM00000005319 XP_033738723.1 61.5 122
43 2 4 122 106 226 1.20E-32 147.5 6579 cellular
organisms;Eukaryota;Opisthokonta;Metazoa;Eumetazoa;Bilateria;Protostomia;
Spiralia;Lophotrochozoa;Mollusca;Bivalvia;Autobranchia;Pteriomorphia;Pect
inida;Pectinoidea;Pectinidae;Pecten;Pecten maximus
UdeM00000005321 6596 95.1 UdeM00000005321 XP_045216961.1 26.1 379
239 14 395 751 417 776 6.70E-16 95.1 6596 cellular
organisms;Eukaryota;Opisthokonta;Metazoa;Eumetazoa;Bilateria;Protostomia;
Spiralia;Lophotrochozoa;Mollusca;Bivalvia;Autobranchia;Heteroconchia;Euhe
terodonta;Imparidentia;Venerida;Veneroidea;Veneridae;Mercenaria;Mercenari
a mercenaria
UdeM00000005322 1735272 97.1 UdeM00000005322 XP_041359644.1 26.6
350 224 14 12 357 354 674 5.10E-17 97.1 1735272
cellular
organisms;Eukaryota;Opisthokonta;Metazoa;Eumetazoa;Bilateria;Protostomia;
Spiralia;Lophotrochozoa;Mollusca;Gastropoda;Neomphalina;Neomphaloidea;Pel
tospiridae;Gigantopelta;Gigantopelta aegis
UdeM00000005323 6596 260 UdeM00000005323 XP_045158432.1 51 261
117 4 17 269 92 349 3.40E-66 260 6596 cellular
organisms;Eukaryota;Opisthokonta;Metazoa;Eumetazoa;Bilateria;Protostomia;
Spiralia;Lophotrochozoa;Mollusca;Bivalvia;Autobranchia;Heteroconchia;Euhe
terodonta;Imparidentia;Venerida;Veneroidea;Veneridae;Mercenaria;Mercenari
a mercenaria
UdeM00000005324 6596 99.8 UdeM00000005324 XP_045216883.1 26.9 375
247 12 370 729 10 372 2.70E-17 99.8 6596 cellular
organisms;Eukaryota;Opisthokonta;Metazoa;Eumetazoa;Bilateria;Protostomia;
Spiralia;Lophotrochozoa;Mollusca;Bivalvia;Autobranchia;Heteroconchia;Euhe
terodonta;Imparidentia;Venerida;Veneroidea;Veneridae;Mercenaria;Mercenari
a mercenaria
UdeM00000005325 10224 210.7 UdeM00000005325 XP_002736880.1 51.3 195
93 1 14 206 36 230 2.20E-51 210.7 10224 cellular
organisms;Eukaryota;Opisthokonta;Metazoa;Eumetazoa;Bilateria;Deuterostomi

a;Hemichordata;Enteropneusta;Harrimaniidae;Saccoglossus;Saccoglossus kowalevskii
UdeM00000005327 6579 148.7 UdeM00000005327 XP_033738723.1 59.4 128 48 2 4 128 106 232 6.30E-33 148.7 6579 cellular organisms;Eukaryota;Opisthokonta;Metazoa;Eumetazoa;Bilateria;Protostomia;Spiralia;Lophotrochozoa;Mollusca;Bivalvia;Autobranchia;Pteriomorphia;Pectinida;Pectinoidea;Pectinidae;Pecten;Pecten maximus
UdeM00000005329 6596 94.7 UdeM00000005329 XP_045216961.1 26.1 379 239 14 395 751 417 776 8.80E-16 94.7 6596 cellular organisms;Eukaryota;Opisthokonta;Metazoa;Eumetazoa;Bilateria;Protostomia;Spiralia;Lophotrochozoa;Mollusca;Bivalvia;Autobranchia;Heteroconchia;Euheterodonta;Imparidentia;Venerida;Veneroidea;Veneridae;Mercenaria;Mercenaria mercenaria
UdeM00000005331 6596 253.1 UdeM00000005331 XP_045157040.1 49.8 255 109 4 1 247 111 354 3.80E-64 253.1 6596 cellular organisms;Eukaryota;Opisthokonta;Metazoa;Eumetazoa;Bilateria;Protostomia;Spiralia;Lophotrochozoa;Mollusca;Bivalvia;Autobranchia;Heteroconchia;Euheterodonta;Imparidentia;Venerida;Veneroidea;Veneridae;Mercenaria;Mercenaria mercenaria
UdeM00000005332 6596 198.4 UdeM00000005332 XP_045157040.1 44.5 245 81 3 1 191 111 354 8.70E-48 198.4 6596 cellular organisms;Eukaryota;Opisthokonta;Metazoa;Eumetazoa;Bilateria;Protostomia;Spiralia;Lophotrochozoa;Mollusca;Bivalvia;Autobranchia;Heteroconchia;Euheterodonta;Imparidentia;Venerida;Veneroidea;Veneridae;Mercenaria;Mercenaria mercenaria
UdeM00000005334 6596 82.4 UdeM00000005334 XP_045185328.1 23.2 457 296 16 254 686 9 434 4.20E-12 82.4 6596 cellular organisms;Eukaryota;Opisthokonta;Metazoa;Eumetazoa;Bilateria;Protostomia;Spiralia;Lophotrochozoa;Mollusca;Bivalvia;Autobranchia;Heteroconchia;Euheterodonta;Imparidentia;Venerida;Veneroidea;Veneridae;Mercenaria;Mercenaria mercenaria
UdeM00000005335 6596 82.4 UdeM00000005335 XP_045185328.1 23.2 457 296 16 249 681 9 434 4.20E-12 82.4 6596 cellular organisms;Eukaryota;Opisthokonta;Metazoa;Eumetazoa;Bilateria;Protostomia;Spiralia;Lophotrochozoa;Mollusca;Bivalvia;Autobranchia;Heteroconchia;Euheterodonta;Imparidentia;Venerida;Veneroidea;Veneridae;Mercenaria;Mercenaria mercenaria
UdeM00000005337 6596 251.5 UdeM00000005337 XP_045158432.1 42.9 347 127 5 23 315 22 351 1.40E-63 251.5 6596 cellular organisms;Eukaryota;Opisthokonta;Metazoa;Eumetazoa;Bilateria;Protostomia;Spiralia;Lophotrochozoa;Mollusca;Bivalvia;Autobranchia;Heteroconchia;Euheterodonta;Imparidentia;Venerida;Veneroidea;Veneridae;Mercenaria;Mercenaria mercenaria
UdeM00000005339 6596 91.3 UdeM00000005339 XP_045185328.1 23.2 457 296 16 289 721 9 434 9.40E-15 91.3 6596 cellular organisms;Eukaryota;Opisthokonta;Metazoa;Eumetazoa;Bilateria;Protostomia;Spiralia;Lophotrochozoa;Mollusca;Bivalvia;Autobranchia;Heteroconchia;Euheterodonta;Imparidentia;Venerida;Veneroidea;Veneridae;Mercenaria;Mercenaria mercenaria
UdeM00000005340 1735272 116.3 UdeM00000005340 XP_041356266.1 49.5 91 46 0 33 123 473 563 2.70E-23 116.3 1735272 cellular organisms;Eukaryota;Opisthokonta;Metazoa;Eumetazoa;Bilateria;Protostomia;

Spiralia;Lophotrochozoa;Mollusca;Gastropoda;Neomphalina;Neomphaloidea;Peltospiridae;Gigantopelta;Gigantopelta aegis
UdeM00000005341 6596 91.3 UdeM00000005341 XP_045160186.1 28.2 202 130 3 132 330 62 251 2.50E-15 91.3 6596 cellular organisms;Eukaryota;Opisthokonta;Metazoa;Eumetazoa;Bilateria;Protostomia;Spiralia;Lophotrochozoa;Mollusca;Bivalvia;Autobranchia;Heteroconchia;Euheterodonta;Imparidentia;Venerida;Veneroidea;Veneridae;Mercenaria;Mercenaria mercenaria
UdeM00000005342 6596 117.5 UdeM00000005342 XP_045160186.1 26.9 249 163 3 92 339 21 251 3.40E-23 117.5 6596 cellular organisms;Eukaryota;Opisthokonta;Metazoa;Eumetazoa;Bilateria;Protostomia;Spiralia;Lophotrochozoa;Mollusca;Bivalvia;Autobranchia;Heteroconchia;Euheterodonta;Imparidentia;Venerida;Veneroidea;Veneridae;Mercenaria;Mercenaria mercenaria
UdeM00000005343 29159 153.3 UdeM00000005343 XP_034299474.1 53.4 148 66 2 1 145 329 476 2.60E-34 153.3 29159 cellular organisms;Eukaryota;Opisthokonta;Metazoa;Eumetazoa;Bilateria;Protostomia;Spiralia;Lophotrochozoa;Mollusca;Bivalvia;Autobranchia;Pteriomorphia;Ostreida;Ostreoidea;Ostreidae;Crassostrea;Crassostrea gigas
UdeM00000005346 6565 90.1 UdeM00000005346 XP_022322655.1 40.9 110 62 2 1 107 639 748 1.80E-15 90.1 6565 cellular organisms;Eukaryota;Opisthokonta;Metazoa;Eumetazoa;Bilateria;Protostomia;Spiralia;Lophotrochozoa;Mollusca;Bivalvia;Autobranchia;Pteriomorphia;Ostreida;Ostreoidea;Ostreidae;Crassostrea;Crassostrea virginica
UdeM00000005347 6596 95.1 UdeM00000005347 XP_045160186.1 27.1 210 130 3 85 291 62 251 1.50E-16 95.1 6596 cellular organisms;Eukaryota;Opisthokonta;Metazoa;Eumetazoa;Bilateria;Protostomia;Spiralia;Lophotrochozoa;Mollusca;Bivalvia;Autobranchia;Heteroconchia;Euheterodonta;Imparidentia;Venerida;Veneroidea;Veneridae;Mercenaria;Mercenaria mercenaria
UdeM00000005348 37653 149.1 UdeM00000005348 XP_014773381.1 37.2 215 105 5 96 309 58 243 9.70E-33 149.1 37653 cellular organisms;Eukaryota;Opisthokonta;Metazoa;Eumetazoa;Bilateria;Protostomia;Spiralia;Lophotrochozoa;Mollusca;Cephalopoda;Coleoidea;Octopodiformes;Octopoda;Incirrata;Octopodidae;Octopus;Octopus bimaculoides
UdeM00000005349 6579 100.1 UdeM00000005349 XP_033736975.1 28.6 175 118 4 46 216 69 240 3.70E-18 100.1 6579 cellular organisms;Eukaryota;Opisthokonta;Metazoa;Eumetazoa;Bilateria;Protostomia;Spiralia;Lophotrochozoa;Mollusca;Bivalvia;Autobranchia;Pteriomorphia;Pectinida;Pectinoidea;Pectinidae;Pecten;Pecten maximus
UdeM00000005351 7574 97.1 UdeM00000005351 XP_013396873.1 29.4 177 120 3 42 216 61 234 3.10E-17 97.1 7574 cellular organisms;Eukaryota;Opisthokonta;Metazoa;Eumetazoa;Bilateria;Protostomia;Spiralia;Lophotrochozoa;Brachiopoda;Linguliformea;Lingulata;Lingulida;Linguloidea;Lingulidae;Lingula;Lingula anatina
UdeM00000005352 29053 118.2 UdeM00000005352 XP_023246665.1 51.8 110 53 0 9 118 216 325 6.90E-24 118.2 29053 cellular organisms;Eukaryota;Opisthokonta;Metazoa;Eumetazoa;Bilateria;Protostomia;Ecdysozoa;Panarthropoda;Arthropoda;Mandibulata;Pancrustacea;Hexapoda;Insecta;Dicondylia;Pterygota;Neoptera;Endopterygota;Hymenoptera;Apocrita;Parasitoida;Chalcidoidea;Encyrtidae;Encyrtinae;Copidosoma;Copidosoma floridanum
UdeM00000005353 6596 419.5 UdeM00000005353 XP_045159559.1 52.2 379 174 6 31 409 22 393 5.00E-114 419.5 6596 cellular

organisms;Eukaryota;Opisthokonta;Metazoa;Eumetazoa;Bilateria;Protostomia;Spiralia;Lophotrochozoa;Mollusca;Bivalvia;Autobranchia;Heteroconchia;Euheterodonta;Imparidentia;Venerida;Veneroidea;Veneridae;Mercenaria;Mercenaria mercenaria
UdeM00000005354 6596 565.5 UdeM00000005354 XP_045159560.1 53.8 545 227 4 1 524 1 541 7.20E-158 565.5 6596 cellular organisms;Eukaryota;Opisthokonta;Metazoa;Eumetazoa;Bilateria;Protostomia;Spiralia;Lophotrochozoa;Mollusca;Bivalvia;Autobranchia;Heteroconchia;Euheterodonta;Imparidentia;Venerida;Veneroidea;Veneridae;Mercenaria;Mercenaria mercenaria
UdeM00000005355 6573 197.2 UdeM00000005355 XP_021341342.1 98.9 93 1 0 1 93 1 93 1.40E-47 197.2 6573 cellular organisms;Eukaryota;Opisthokonta;Metazoa;Eumetazoa;Bilateria;Protostomia;Spiralia;Lophotrochozoa;Mollusca;Bivalvia;Autobranchia;Pteriomorphia;Pectinida;Pectinoidea;Pectinidae;Mizuhopecten;Mizuhopecten yessoensis
UdeM00000005356 6596 341.3 UdeM00000005356 XP_045160898.1 74.5 204 51 1 6 208 162 365 9.20E-91 341.3 6596 cellular organisms;Eukaryota;Opisthokonta;Metazoa;Eumetazoa;Bilateria;Protostomia;Spiralia;Lophotrochozoa;Mollusca;Bivalvia;Autobranchia;Heteroconchia;Euheterodonta;Imparidentia;Venerida;Veneroidea;Veneridae;Mercenaria;Mercenaria mercenaria
UdeM00000005357 6596 320.5 UdeM00000005357 XP_045160898.1 53.6 293 131 3 14 303 365 655 2.50E-84 320.5 6596 cellular organisms;Eukaryota;Opisthokonta;Metazoa;Eumetazoa;Bilateria;Protostomia;Spiralia;Lophotrochozoa;Mollusca;Bivalvia;Autobranchia;Heteroconchia;Euheterodonta;Imparidentia;Venerida;Veneroidea;Veneridae;Mercenaria;Mercenaria mercenaria
UdeM00000005359 161584 95.9 UdeM00000005359 XP_037104184.1 51 100 49 0 2 101 215 314 4.00E-17 95.9 161584 cellular organisms;Eukaryota;Opisthokonta;Metazoa;Eumetazoa;Bilateria;Deuterostomia;Chordata;Craniata;Vertebrata;Gnathostomata;Teleostomi;Euteleostomi;Actinopterygii;Actinopteri;Neopterygii;Teleostei;Osteoglossocephalai;Clupeocephala;Euteleosteomorpha;Neoteleostei;Eurypterygia;Ctenosquamata;Acanthomorphata;Euacanthomorphacea;Percomorphaceae;Syngnathiaria;Syngnathiformes;Syngnathoidei;Syngnathidae;Syngnathinae;Syngnathus;Syngnathus acus
UdeM00000005360 77115 63.5 UdeM00000005360 XP_038157148.1 38.1 84 50 1 7 90 2 83 1.90E-07 63.5 77115 cellular organisms;Eukaryota;Opisthokonta;Metazoa;Eumetazoa;Bilateria;Deuterostomia;Chordata;Craniata;Vertebrata;Gnathostomata;Teleostomi;Euteleostomi;Actinopterygii;Actinopteri;Neopterygii;Teleostei;Osteoglossocephalai;Clupeocephala;Euteleosteomorpha;Neoteleostei;Eurypterygia;Ctenosquamata;Acanthomorphata;Euacanthomorphacea;Percomorphaceae;Ovalentaria;Atherinomorphae;Cyprinodontiformes;Cyprinodontoidei;Cyprinodontidae;Cyprinodontinae;Cyprinodontini;Cyprinodon;Cyprinodon tularosa
UdeM00000005361 6565 308.1 UdeM00000005361 XP_022317798.1 85.7 161 23 0 1 161 1 161 6.40E-81 308.1 6565 cellular organisms;Eukaryota;Opisthokonta;Metazoa;Eumetazoa;Bilateria;Protostomia;Spiralia;Lophotrochozoa;Mollusca;Bivalvia;Autobranchia;Pteriomorphia;Ostreida;Ostreoidea;Ostreidae;Crassostrea;Crassostrea virginica
UdeM00000005362 6596 405.2 UdeM00000005362 XP_045159561.1 63.5 293 100 3 4 294 13 300 7.50E-110 405.2 6596 cellular organisms;Eukaryota;Opisthokonta;Metazoa;Eumetazoa;Bilateria;Protostomia;Spiralia;Lophotrochozoa;Mollusca;Bivalvia;Autobranchia;Heteroconchia;Euhe

terodonta;Imparidentia;Venerida;Veneroidea;Veneridae;Mercenaria;Mercenari a mercenaria
UdeM00000005363 6573 455.3 UdeM00000005363 XP_021374817.1 39.7 695 344 15 17 656 9 683 1.30E-124 455.3 6573 cellular organisms;Eukaryota;Opisthokonta;Metazoa;Eumetazoa;Bilateria;Protostomia;Spiralia;Lophotrochozoa;Mollusca;Bivalvia;Autobranchia;Pteriomorphia;Pectinida;Pectinoidea;Pectinidae;Mizuhopecten;Mizuhopecten yessoensis
UdeM00000005365 6596 274.2 UdeM00000005365 XP_045160931.1 50.5 277 135 1 1 277 201 475 1.80E-70 274.2 6596 cellular organisms;Eukaryota;Opisthokonta;Metazoa;Eumetazoa;Bilateria;Protostomia;Spiralia;Lophotrochozoa;Mollusca;Bivalvia;Autobranchia;Heteroconchia;Euheterodonta;Imparidentia;Venerida;Veneroidea;Veneridae;Mercenaria;Mercenaria mercenaria
UdeM00000005364 6596 391.3 UdeM00000005364 XP_045160931.1 45 471 245 4 14 480 15 475 1.70E-105 391.3 6596 cellular organisms;Eukaryota;Opisthokonta;Metazoa;Eumetazoa;Bilateria;Protostomia;Spiralia;Lophotrochozoa;Mollusca;Bivalvia;Autobranchia;Heteroconchia;Euheterodonta;Imparidentia;Venerida;Veneroidea;Veneridae;Mercenaria;Mercenaria mercenaria
UdeM00000005366 6565 124.8 UdeM00000005366 XP_022315755.1 50.7 136 60 2 67 201 65 194 1.30E-25 124.8 6565 cellular organisms;Eukaryota;Opisthokonta;Metazoa;Eumetazoa;Bilateria;Protostomia;Spiralia;Lophotrochozoa;Mollusca;Bivalvia;Autobranchia;Pteriomorphia;Ostreida;Ostreoidea;Ostreidae;Crassostrea;Crassostrea virginica
UdeM00000005367 6596 1052.4 UdeM00000005367 XP_045158822.1 92.7 549 40 0 1 549 1 549 2.00E-304 1052.4 6596 cellular organisms;Eukaryota;Opisthokonta;Metazoa;Eumetazoa;Bilateria;Protostomia;Spiralia;Lophotrochozoa;Mollusca;Bivalvia;Autobranchia;Heteroconchia;Euheterodonta;Imparidentia;Venerida;Veneroidea;Veneridae;Mercenaria;Mercenaria mercenaria
UdeM00000005369 1735272 409.5 UdeM00000005369 XP_041358009.1 64 292 105 0 4 295 17 308 3.70E-111 409.5 1735272 cellular organisms;Eukaryota;Opisthokonta;Metazoa;Eumetazoa;Bilateria;Protostomia;Spiralia;Lophotrochozoa;Mollusca;Gastropoda;Neomphalina;Neomphaloidea;Peltospiridae;Gigantopelta;Gigantopelta aegis
UdeM00000005370 6573 170.2 UdeM00000005370 XP_021377013.1 67.9 131 41 1 1 131 1 130 1.90E-39 170.2 6573 cellular organisms;Eukaryota;Opisthokonta;Metazoa;Eumetazoa;Bilateria;Protostomia;Spiralia;Lophotrochozoa;Mollusca;Bivalvia;Autobranchia;Pteriomorphia;Pectinida;Pectinoidea;Pectinidae;Mizuhopecten;Mizuhopecten yessoensis
UdeM00000005371 1735272 112.8 UdeM00000005371 XP_041366822.1 44.4 133 74 0 10 142 2 134 1.10E-21 112.8 1735272 cellular organisms;Eukaryota;Opisthokonta;Metazoa;Eumetazoa;Bilateria;Protostomia;Spiralia;Lophotrochozoa;Mollusca;Gastropoda;Neomphalina;Neomphaloidea;Peltospiridae;Gigantopelta;Gigantopelta aegis
UdeM00000005372 6596 957.2 UdeM00000005372 XP_045160538.1 65.2 784 195 11 1 773 1 717 1.30E-275 957.2 6596 cellular organisms;Eukaryota;Opisthokonta;Metazoa;Eumetazoa;Bilateria;Protostomia;Spiralia;Lophotrochozoa;Mollusca;Bivalvia;Autobranchia;Heteroconchia;Euheterodonta;Imparidentia;Venerida;Veneroidea;Veneridae;Mercenaria;Mercenaria mercenaria

UdeM00000005373	6596	253.8	UdeM00000005373	XP_045172766.1	41.7	319
179	4	67	384	138	450	3.50E-64	253.8	6596	cellular organisms;Eukaryota;Opisthokonta;Metazoa;Eumetazoa;Bilateria;Protostomia;Spiralia;Lophotrochozoa;Mollusca;Bivalvia;Autobranchia;Heteroconchia;Euheterodonta;Imparidentia;Venerida;Veneroidea;Veneridae;Mercenaria;Mercenaria mercenaria
UdeM00000005374	6573	157.1	UdeM00000005374	XP_021377015.1	62	100
38	0	2	101	1455	1554	1.30E-35	157.1	6573	cellular organisms;Eukaryota;Opisthokonta;Metazoa;Eumetazoa;Bilateria;Protostomia;Spiralia;Lophotrochozoa;Mollusca;Bivalvia;Autobranchia;Pteriomorphia;Pectinida;Pectinoidea;Pectinidae;Mizuhopecten;Mizuhopecten yessoensis
UdeM00000005375	6573	2129	UdeM00000005375	XP_021377015.1	64.1	1559
549	8	1	1553	1	1554	0.00E+00	2129	6573	cellular organisms;Eukaryota;Opisthokonta;Metazoa;Eumetazoa;Bilateria;Protostomia;Spiralia;Lophotrochozoa;Mollusca;Bivalvia;Autobranchia;Pteriomorphia;Pectinida;Pectinoidea;Pectinidae;Mizuhopecten;Mizuhopecten yessoensis
UdeM00000005378	1735272	68.2	UdeM00000005378	XP_041364761.1	42.1
76	44	0	37	112	70	145	8.30E-09	68.2	1735272
cellular organisms;Eukaryota;Opisthokonta;Metazoa;Eumetazoa;Bilateria;Protostomia;Spiralia;Lophotrochozoa;Mollusca;Gastropoda;Neomphalina;Neomphaloidea;Peltospiridae;Gigantopelta;Gigantopelta aegis
UdeM00000005379	1735272	82	UdeM00000005379	XP_041364761.1	45
80	44	0	37	116	70	149	5.50E-13	82	1735272
cellular organisms;Eukaryota;Opisthokonta;Metazoa;Eumetazoa;Bilateria;Protostomia;Spiralia;Lophotrochozoa;Mollusca;Gastropoda;Neomphalina;Neomphaloidea;Peltospiridae;Gigantopelta;Gigantopelta aegis
UdeM00000005380	6596	291.6	UdeM00000005380	XP_045159679.1	44.9	312
161	3	35	346	35	335	1.40E-75	291.6	6596	cellular organisms;Eukaryota;Opisthokonta;Metazoa;Eumetazoa;Bilateria;Protostomia;Spiralia;Lophotrochozoa;Mollusca;Bivalvia;Autobranchia;Heteroconchia;Euheterodonta;Imparidentia;Venerida;Veneroidea;Veneridae;Mercenaria;Mercenaria mercenaria
UdeM00000005381	742174	216.1	UdeM00000005381	XP_025199557.1	34.8
342	201	6	1	320	12	353	6.60E-53	216.1	742174
cellular organisms;Eukaryota;Opisthokonta;Metazoa;Eumetazoa;Bilateria;Protostomia;Ecdysozoa;Panarthropoda;Arthropoda;Mandibulata;Pancrustacea;Hexapoda;Insecta;Dicondylia;Pterygota;Neoptera;Paraneoptera;Hemiptera;Sternorrhyncha;Aphidomorpha;Aphidoidea;Aphididae;Aphidinae;Aphidini;Melanaphis;Melanaphis sacchari
UdeM00000005382	29159	773.9	UdeM00000005382	XP_011436709.1	69	603
166	8	1	592	1	593	1.50E-220	773.9	29159	cellular organisms;Eukaryota;Opisthokonta;Metazoa;Eumetazoa;Bilateria;Protostomia;Spiralia;Lophotrochozoa;Mollusca;Bivalvia;Autobranchia;Pteriomorphia;Ostreida;Ostreoidea;Ostreidae;Crassostrea;Crassostrea gigas
UdeM00000005383	29159	785.8	UdeM00000005383	XP_011436709.1	69.9	601
161	9	1	589	1	593	3.80E-224	785.8	29159	cellular organisms;Eukaryota;Opisthokonta;Metazoa;Eumetazoa;Bilateria;Protostomia;Spiralia;Lophotrochozoa;Mollusca;Bivalvia;Autobranchia;Pteriomorphia;Ostreida;Ostreoidea;Ostreidae;Crassostrea;Crassostrea gigas
UdeM00000005384	7741	295.8	UdeM00000005384	XP_019645671.1	46.2	344
162	4	187	526	96	420	1.10E-76	295.8	7741	cellular

organisms;Eukaryota;Opisthokonta;Metazoa;Eumetazoa;Bilateria;Deuterostomia;Chordata;Cephalochordata;Leptocardii;Amphioxiformes;Branchiostomidae;Branchiostoma;Branchiostoma belcheri
UdeM00000005385 6579 289.7 UdeM00000005385 XP_033737045.1 39.6 419 206 8 122 502 12 421 7.60E-75 289.7 6579 cellular organisms;Eukaryota;Opisthokonta;Metazoa;Eumetazoa;Bilateria;Protostomia;Spiralia;Lophotrochozoa;Mollusca;Bivalvia;Autobranchia;Pteriomorphia;Pectinida;Pectinoidea;Pectinidae;Pecten;Pecten maximus
UdeM00000005386 6596 221.9 UdeM00000005386 XP_045157090.1 48.6 218 110 1 4 219 2 219 8.40E-55 221.9 6596 cellular organisms;Eukaryota;Opisthokonta;Metazoa;Eumetazoa;Bilateria;Protostomia;Spiralia;Lophotrochozoa;Mollusca;Bivalvia;Autobranchia;Heteroconchia;Euheterodonta;Imparidentia;Venerida;Veneroidea;Veneridae;Mercenaria;Mercenaria mercenaria
UdeM00000005387 6596 216.5 UdeM00000005387 XP_045157090.1 49.8 205 101 1 4 206 15 219 3.30E-53 216.5 6596 cellular organisms;Eukaryota;Opisthokonta;Metazoa;Eumetazoa;Bilateria;Protostomia;Spiralia;Lophotrochozoa;Mollusca;Bivalvia;Autobranchia;Heteroconchia;Euheterodonta;Imparidentia;Venerida;Veneroidea;Veneridae;Mercenaria;Mercenaria mercenaria
UdeM00000005388 6596 330.1 UdeM00000005388 XP_045159695.1 59.6 260 104 1 1 259 1 260 2.60E-87 330.1 6596 cellular organisms;Eukaryota;Opisthokonta;Metazoa;Eumetazoa;Bilateria;Protostomia;Spiralia;Lophotrochozoa;Mollusca;Bivalvia;Autobranchia;Heteroconchia;Euheterodonta;Imparidentia;Venerida;Veneroidea;Veneridae;Mercenaria;Mercenaria mercenaria
UdeM00000005389 6596 91.3 UdeM00000005389 XP_045159473.1 30.7 163 81 3 396 556 273 405 4.50E-15 91.3 6596 cellular organisms;Eukaryota;Opisthokonta;Metazoa;Eumetazoa;Bilateria;Protostomia;Spiralia;Lophotrochozoa;Mollusca;Bivalvia;Autobranchia;Heteroconchia;Euheterodonta;Imparidentia;Venerida;Veneroidea;Veneridae;Mercenaria;Mercenaria mercenaria
UdeM00000005391 6573 198 UdeM00000005391 XP_021372817.1 31.8 478 273 18 145 593 249 702 4.60E-47 198 6573 cellular organisms;Eukaryota;Opisthokonta;Metazoa;Eumetazoa;Bilateria;Protostomia;Spiralia;Lophotrochozoa;Mollusca;Bivalvia;Autobranchia;Pteriomorphia;Pectinida;Pectinoidea;Pectinidae;Mizuhopecten;Mizuhopecten yessoensis
UdeM00000005392 6596 124.4 UdeM00000005392 XP_045160870.1 65.8 76 26 0 140 215 356 431 1.80E-25 124.4 6596 cellular organisms;Eukaryota;Opisthokonta;Metazoa;Eumetazoa;Bilateria;Protostomia;Spiralia;Lophotrochozoa;Mollusca;Bivalvia;Autobranchia;Heteroconchia;Euheterodonta;Imparidentia;Venerida;Veneroidea;Veneridae;Mercenaria;Mercenaria mercenaria
UdeM00000005394 6596 176.4 UdeM00000005394 XP_045159473.1 24.7 510 247 16 192 639 42 476 1.50E-40 176.4 6596 cellular organisms;Eukaryota;Opisthokonta;Metazoa;Eumetazoa;Bilateria;Protostomia;Spiralia;Lophotrochozoa;Mollusca;Bivalvia;Autobranchia;Heteroconchia;Euheterodonta;Imparidentia;Venerida;Veneroidea;Veneridae;Mercenaria;Mercenaria mercenaria
UdeM00000005395 6596 124.4 UdeM00000005395 XP_045160870.1 43 151 45 3 105 219 286 431 1.80E-25 124.4 6596 cellular organisms;Eukaryota;Opisthokonta;Metazoa;Eumetazoa;Bilateria;Protostomia;Spiralia;Lophotrochozoa;Mollusca;Bivalvia;Autobranchia;Heteroconchia;Euhe

terodonta;Imparidentia;Venerida;Veneroidea;Veneridae;Mercenaria;Mercenari
a mercenaria
UdeM00000005396 6565 171.8 UdeM00000005396 XP_022311830.1 32 306
159 10 331 633 58 317 3.70E-39 171.8 6565 cellular
organisms;Eukaryota;Opisthokonta;Metazoa;Eumetazoa;Bilateria;Protostomia;
Spiralia;Lophotrochozoa;Mollusca;Bivalvia;Autobranchia;Pteriomorphia;Ostr
eida;Ostreoidea;Ostreidae;Crassostrea;Crassostrea virginica
UdeM00000005397 6596 127.5 UdeM00000005397 XP_045160870.1 67.1 76
25 0 140 215 356 431 2.10E-26 127.5 6596 cellular
organisms;Eukaryota;Opisthokonta;Metazoa;Eumetazoa;Bilateria;Protostomia;
Spiralia;Lophotrochozoa;Mollusca;Bivalvia;Autobranchia;Heteroconchia;Euhe
terodonta;Imparidentia;Venerida;Veneroidea;Veneridae;Mercenaria;Mercenari
a mercenaria
UdeM00000005399 6596 182.2 UdeM00000005399 XP_045159473.1 28.5 365
201 11 329 638 117 476 2.80E-42 182.2 6596 cellular
organisms;Eukaryota;Opisthokonta;Metazoa;Eumetazoa;Bilateria;Protostomia;
Spiralia;Lophotrochozoa;Mollusca;Bivalvia;Autobranchia;Heteroconchia;Euhe
terodonta;Imparidentia;Venerida;Veneroidea;Veneridae;Mercenaria;Mercenari
a mercenaria
UdeM00000005400 6596 182.2 UdeM00000005400 XP_045159473.1 28.5 365
201 11 329 638 117 476 2.80E-42 182.2 6596 cellular
organisms;Eukaryota;Opisthokonta;Metazoa;Eumetazoa;Bilateria;Protostomia;
Spiralia;Lophotrochozoa;Mollusca;Bivalvia;Autobranchia;Heteroconchia;Euhe
terodonta;Imparidentia;Venerida;Veneroidea;Veneridae;Mercenaria;Mercenari
a mercenaria
UdeM00000005403 6596 153.3 UdeM00000005403 XP_045159473.1 35 254
140 10 2 232 103 354 3.80E-34 153.3 6596 cellular
organisms;Eukaryota;Opisthokonta;Metazoa;Eumetazoa;Bilateria;Protostomia;
Spiralia;Lophotrochozoa;Mollusca;Bivalvia;Autobranchia;Heteroconchia;Euhe
terodonta;Imparidentia;Venerida;Veneroidea;Veneridae;Mercenaria;Mercenari
a mercenaria
UdeM00000005405 6565 180.6 UdeM00000005405 XP_022311830.1 35 286
150 10 359 633 57 317 8.00E-42 180.6 6565 cellular
organisms;Eukaryota;Opisthokonta;Metazoa;Eumetazoa;Bilateria;Protostomia;
Spiralia;Lophotrochozoa;Mollusca;Bivalvia;Autobranchia;Pteriomorphia;Ostr
eida;Ostreoidea;Ostreidae;Crassostrea;Crassostrea virginica
UdeM00000005406 6565 180.6 UdeM00000005406 XP_022311830.1 35 286
150 10 320 594 57 317 7.70E-42 180.6 6565 cellular
organisms;Eukaryota;Opisthokonta;Metazoa;Eumetazoa;Bilateria;Protostomia;
Spiralia;Lophotrochozoa;Mollusca;Bivalvia;Autobranchia;Pteriomorphia;Ostr
eida;Ostreoidea;Ostreidae;Crassostrea;Crassostrea virginica
UdeM00000005407 6596 492.7 UdeM00000005407 XP_045169782.1 50.7 477
205 2 1 448 56 531 5.30E-136 492.7 6596 cellular
organisms;Eukaryota;Opisthokonta;Metazoa;Eumetazoa;Bilateria;Protostomia;
Spiralia;Lophotrochozoa;Mollusca;Bivalvia;Autobranchia;Heteroconchia;Euhe
terodonta;Imparidentia;Venerida;Veneroidea;Veneridae;Mercenaria;Mercenari
a mercenaria
UdeM00000005409 6573 206.1 UdeM00000005409 XP_021353855.1 43 416
202 12 6 403 6 404 8.60E-50 206.1 6573 cellular
organisms;Eukaryota;Opisthokonta;Metazoa;Eumetazoa;Bilateria;Protostomia;
Spiralia;Lophotrochozoa;Mollusca;Bivalvia;Autobranchia;Pteriomorphia;Pect
inida;Pectinoidea;Pectinidae;Mizuhopecten;Mizuhopecten yessoensis
UdeM00000005410 6596 429.9 UdeM00000005410 XP_045158303.1 54.8 526
194 15 1 508 1 500 4.60E-117 429.9 6596 cellular

organisms;Eukaryota;Opisthokonta;Metazoa;Eumetazoa;Bilateria;Protostomia;Spiralia;Lophotrochozoa;Mollusca;Bivalvia;Autobranchia;Heteroconchia;Euheterodonta;Imparidentia;Venerida;Veneroidea;Veneridae;Mercenaria;Mercenaria mercenaria
UdeM00000005411 6565 94.7 UdeM00000005411 XP_022308193.1 42.1 126 69 2 10 133 6 129 1.00E-16 94.7 6565 cellular organisms;Eukaryota;Opisthokonta;Metazoa;Eumetazoa;Bilateria;Protostomia;Spiralia;Lophotrochozoa;Mollusca;Bivalvia;Autobranchia;Pteriomorphia;Ostreida;Ostreoidea;Ostreidae;Crassostrea;Crassostrea virginica
UdeM00000005412 6565 134.8 UdeM00000005412 XP_022308193.1 53.1 113 52 1 1 113 445 556 7.00E-29 134.8 6565 cellular organisms;Eukaryota;Opisthokonta;Metazoa;Eumetazoa;Bilateria;Protostomia;Spiralia;Lophotrochozoa;Mollusca;Bivalvia;Autobranchia;Pteriomorphia;Ostreida;Ostreoidea;Ostreidae;Crassostrea;Crassostrea virginica
UdeM00000005413 6565 162.5 UdeM00000005413 XP_022299727.1 48 173 89 1 1 173 106 277 5.40E-37 162.5 6565 cellular organisms;Eukaryota;Opisthokonta;Metazoa;Eumetazoa;Bilateria;Protostomia;Spiralia;Lophotrochozoa;Mollusca;Bivalvia;Autobranchia;Pteriomorphia;Ostreida;Ostreoidea;Ostreidae;Crassostrea;Crassostrea virginica
UdeM00000005414 6596 555.1 UdeM00000005414 XP_045158296.1 58.9 443 176 3 1 442 1 438 8.30E-155 555.1 6596 cellular organisms;Eukaryota;Opisthokonta;Metazoa;Eumetazoa;Bilateria;Protostomia;Spiralia;Lophotrochozoa;Mollusca;Bivalvia;Autobranchia;Heteroconchia;Euheterodonta;Imparidentia;Venerida;Veneroidea;Veneridae;Mercenaria;Mercenaria mercenaria
UdeM00000005415 30033 127.1 UdeM00000005415 XP_041631336.1 59.8 97 37 1 3 99 20 114 1.30E-26 127.1 30033 cellular organisms;Eukaryota;Opisthokonta;Metazoa;Eumetazoa;Bilateria;Protostomia;Ecdysozoa;Panarthropoda;Arthropoda;Mandibulata;Pancrustacea;Hexapoda;Insecta;Dicondylia;Pterygota;Neoptera;Endopterygota;Diptera;Brachycera;Muscomorpha;Eremoneura;Cyclorrhapha;Schizophora;Acalyptratae;Ephydroidea;Drosophilidae;Drosophilinae;Drosophilini;Drosophila;Sophophora;melanogaster group;montium subgroup;Drosophila kikkawai
UdeM00000005416 6579 100.1 UdeM00000005416 XP_033737997.1 26 647 366 27 1 585 14 609 1.20E-17 100.1 6579 cellular organisms;Eukaryota;Opisthokonta;Metazoa;Eumetazoa;Bilateria;Protostomia;Spiralia;Lophotrochozoa;Mollusca;Bivalvia;Autobranchia;Pteriomorphia;Pectinida;Pectinoidea;Pectinidae;Pecten;Pecten maximus
UdeM00000005417 6573 75.5 UdeM00000005417 XP_021342304.1 29 231 129 6 60 264 8 229 1.40E-10 75.5 6573 cellular organisms;Eukaryota;Opisthokonta;Metazoa;Eumetazoa;Bilateria;Protostomia;Spiralia;Lophotrochozoa;Mollusca;Bivalvia;Autobranchia;Pteriomorphia;Pectinida;Pectinoidea;Pectinidae;Mizuhopecten;Mizuhopecten yessoensis
UdeM00000005418 6579 635.2 UdeM00000005418 XP_033738335.1 55.6 572 249 2 6 576 4 571 8.30E-179 635.2 6579 cellular organisms;Eukaryota;Opisthokonta;Metazoa;Eumetazoa;Bilateria;Protostomia;Spiralia;Lophotrochozoa;Mollusca;Bivalvia;Autobranchia;Pteriomorphia;Pectinida;Pectinoidea;Pectinidae;Pecten;Pecten maximus
UdeM00000005419 6596 616.7 UdeM00000005419 XP_045160765.1 49.9 851 399 3 1 827 1 848 5.20E-173 616.7 6596 cellular organisms;Eukaryota;Opisthokonta;Metazoa;Eumetazoa;Bilateria;Protostomia;Spiralia;Lophotrochozoa;Mollusca;Bivalvia;Autobranchia;Heteroconchia;Euheterodonta;Imparidentia;Venerida;Veneroidea;Veneridae;Mercenaria;Mercenaria mercenaria

UdeM00000005420 1735272 290.8 UdeM00000005420 XP_041356784.1 49.8
321 161 0 11 331 1057 1377 6.90E-75 290.8 1735272
cellular
organisms;Eukaryota;Opisthokonta;Metazoa;Eumetazoa;Bilateria;Protostomia;Spiralia;Lophotrochozoa;Mollusca;Gastropoda;Neomphalina;Neomphaloidea;Peltospiridae;Gigantopelta;Gigantopelta aegis
UdeM00000005421 46514 185.3 UdeM00000005421 XP_038075667.1 39.4 274
140 5 1 258 301 564 1.00E-43 185.3 46514 cellular
organisms;Eukaryota;Opisthokonta;Metazoa;Eumetazoa;Bilateria;Deuterostomia;Echinodermata;Eleutherozoa;Asterozoa;Asteroidea;Valvatacea;Valvatida;Asterinidae;Patiria;Patiria miniata
UdeM00000005422 441366 203.4 UdeM00000005422 XP_028296753.1 41.3
305 162 9 6 308 32 321 4.30E-49 203.4 441366
cellular
organisms;Eukaryota;Opisthokonta;Metazoa;Eumetazoa;Bilateria;Deuterostomia;Chordata;Craniata;Vertebrata;Gnathostomata;Teleostomi;Euteleostomi;Actinopterygii;Actinopteri;Neopterygii;Teleostei;Osteoglossocephalai;Clupeocephala;Euteleosteomorpha;Neoteleostei;Eurypterygia;Ctenosquamata;Acanthomorphata;Euacanthomorphacea;Percomorphaceae;Ovalentaria;Blenniimorphae;Blenniiformes;Gobiesocoidei;Gobiesocidae;Gobiesocinae;Gouania;Gouania willdenowi
UdeM00000005424 29159 142.5 UdeM00000005424 XP_034314103.1 50.6 176
73 5 10 181 2186 2351 1.00E-30 142.5 29159 cellular
organisms;Eukaryota;Opisthokonta;Metazoa;Eumetazoa;Bilateria;Protostomia;Spiralia;Lophotrochozoa;Mollusca;Bivalvia;Autobranchia;Pteriomorphia;Ostreida;Ostreoidea;Ostreidae;Crassostrea;Crassostrea gigas
UdeM00000005425 6565 713.8 UdeM00000005425 XP_022314135.1 87.2 422
54 0 1 422 1 422 1.30E-202 713.8 6565 cellular
organisms;Eukaryota;Opisthokonta;Metazoa;Eumetazoa;Bilateria;Protostomia;Spiralia;Lophotrochozoa;Mollusca;Bivalvia;Autobranchia;Pteriomorphia;Ostreida;Ostreoidea;Ostreidae;Crassostrea;Crassostrea virginica
UdeM00000005426 6596 235.3 UdeM00000005426 XP_045158518.1 29.5 675
362 16 1 670 1 566 2.30E-58 235.3 6596 cellular
organisms;Eukaryota;Opisthokonta;Metazoa;Eumetazoa;Bilateria;Protostomia;Spiralia;Lophotrochozoa;Mollusca;Bivalvia;Autobranchia;Heteroconchia;Euheterodonta;Imparidentia;Venerida;Veneroidea;Veneridae;Mercenaria;Mercenaria mercenaria
UdeM00000005427 6596 155.6 UdeM00000005427 XP_045158523.1 54.9 142
57 3 11 149 442 579 4.90E-35 155.6 6596 cellular
organisms;Eukaryota;Opisthokonta;Metazoa;Eumetazoa;Bilateria;Protostomia;Spiralia;Lophotrochozoa;Mollusca;Bivalvia;Autobranchia;Heteroconchia;Euheterodonta;Imparidentia;Venerida;Veneroidea;Veneridae;Mercenaria;Mercenaria mercenaria
UdeM00000005428 6596 396 UdeM00000005428 XP_045158523.1 62 337
113 8 3 332 251 579 4.80E-107 396 6596 cellular
organisms;Eukaryota;Opisthokonta;Metazoa;Eumetazoa;Bilateria;Protostomia;Spiralia;Lophotrochozoa;Mollusca;Bivalvia;Autobranchia;Heteroconchia;Euheterodonta;Imparidentia;Venerida;Veneroidea;Veneridae;Mercenaria;Mercenaria mercenaria
UdeM00000005429 2607531 72.8 UdeM00000005429 XP_029640194.1 57.9
57 20 1 28 80 221 277 2.30E-10 72.8 2607531
cellular
organisms;Eukaryota;Opisthokonta;Metazoa;Eumetazoa;Bilateria;Protostomia;

Spiralia;Lophotrochozoa;Mollusca;Cephalopoda;Coleoidea;Octopodiformes;Octopoda;Incirrata;Octopodidae;Octopus;Octopus sinensis
UdeM00000005431 6579 184.5 UdeM00000005431 XP_033737234.1 58.2 196 69 8 1 187 57 248 1.20E-43 184.5 6579 cellular organisms;Eukaryota;Opisthokonta;Metazoa;Eumetazoa;Bilateria;Protostomia;Spiralia;Lophotrochozoa;Mollusca;Bivalvia;Autobranchia;Pteriomorphia;Pectinida;Pectinoidea;Pectinidae;Pecten;Pecten maximus
UdeM00000005430 6596 234.6 UdeM00000005430 XP_045158523.1 57.4 223 86 5 1 219 1 218 1.20E-58 234.6 6596 cellular organisms;Eukaryota;Opisthokonta;Metazoa;Eumetazoa;Bilateria;Protostomia;Spiralia;Lophotrochozoa;Mollusca;Bivalvia;Autobranchia;Heteroconchia;Euheterodonta;Imparidentia;Venerida;Veneroidea;Veneridae;Mercenaria;Mercenaria mercenaria
UdeM00000005432 6596 138.7 UdeM00000005432 XP_045158522.1 34.5 594 337 16 23 582 6 581 2.40E-29 138.7 6596 cellular organisms;Eukaryota;Opisthokonta;Metazoa;Eumetazoa;Bilateria;Protostomia;Spiralia;Lophotrochozoa;Mollusca;Bivalvia;Autobranchia;Heteroconchia;Euheterodonta;Imparidentia;Venerida;Veneroidea;Veneridae;Mercenaria;Mercenaria mercenaria
UdeM00000005433 6596 359.4 UdeM00000005433 XP_045176367.1 44.6 361 196 4 139 498 335 692 7.80E-96 359.4 6596 cellular organisms;Eukaryota;Opisthokonta;Metazoa;Eumetazoa;Bilateria;Protostomia;Spiralia;Lophotrochozoa;Mollusca;Bivalvia;Autobranchia;Heteroconchia;Euheterodonta;Imparidentia;Venerida;Veneroidea;Veneridae;Mercenaria;Mercenaria mercenaria
UdeM00000005434 6579 309.7 UdeM00000005434 XP_033737835.1 46.3 376 169 9 48 410 235 590 6.00E-81 309.7 6579 cellular organisms;Eukaryota;Opisthokonta;Metazoa;Eumetazoa;Bilateria;Protostomia;Spiralia;Lophotrochozoa;Mollusca;Bivalvia;Autobranchia;Pteriomorphia;Pectinida;Pectinoidea;Pectinidae;Pecten;Pecten maximus
UdeM00000005435 6565 83.2 UdeM00000005435 XP_022314056.1 29.4 211 111 10 12 188 8 214 4.00E-13 83.2 6565 cellular organisms;Eukaryota;Opisthokonta;Metazoa;Eumetazoa;Bilateria;Protostomia;Spiralia;Lophotrochozoa;Mollusca;Bivalvia;Autobranchia;Pteriomorphia;Ostreida;Ostreoidea;Ostreidae;Crassostrea;Crassostrea virginica
UdeM00000005436 6596 508.4 UdeM00000005436 XP_045159515.1 63.7 383 139 0 33 415 274 656 8.40E-141 508.4 6596 cellular organisms;Eukaryota;Opisthokonta;Metazoa;Eumetazoa;Bilateria;Protostomia;Spiralia;Lophotrochozoa;Mollusca;Bivalvia;Autobranchia;Heteroconchia;Euheterodonta;Imparidentia;Venerida;Veneroidea;Veneridae;Mercenaria;Mercenaria mercenaria
UdeM00000005437 6596 275.4 UdeM00000005437 XP_045159519.1 40.3 392 223 5 4 393 32 414 1.10E-70 275.4 6596 cellular organisms;Eukaryota;Opisthokonta;Metazoa;Eumetazoa;Bilateria;Protostomia;Spiralia;Lophotrochozoa;Mollusca;Bivalvia;Autobranchia;Heteroconchia;Euheterodonta;Imparidentia;Venerida;Veneroidea;Veneridae;Mercenaria;Mercenaria mercenaria
UdeM00000005438 6596 157.5 UdeM00000005438 XP_045159515.1 50.6 178 83 3 9 185 102 275 1.70E-35 157.5 6596 cellular organisms;Eukaryota;Opisthokonta;Metazoa;Eumetazoa;Bilateria;Protostomia;Spiralia;Lophotrochozoa;Mollusca;Bivalvia;Autobranchia;Heteroconchia;Euheterodonta;Imparidentia;Venerida;Veneroidea;Veneridae;Mercenaria;Mercenaria mercenaria

UdeM00000005439 6596 83.6 UdeM00000005439 XP_045201747.1   27.2 228
130 6 14 235 11 208 3.80E-13 83.6 6596 cellular organisms;Eukaryota;Opisthokonta;Metazoa;Eumetazoa;Bilateria;Protostomia;Spiralia;Lophotrochozoa;Mollusca;Bivalvia;Autobranchia;Heteroconchia;Euheterodonta;Imparidentia;Venerida;Veneroidea;Veneridae;Mercenaria;Mercenaria mercenaria
UdeM00000005440 6596 88.6 UdeM00000005440 XP_045198326.1   27.8 227
121 6 6 224 159 350 1.20E-14 88.6 6596 cellular organisms;Eukaryota;Opisthokonta;Metazoa;Eumetazoa;Bilateria;Protostomia;Spiralia;Lophotrochozoa;Mollusca;Bivalvia;Autobranchia;Heteroconchia;Euheterodonta;Imparidentia;Venerida;Veneroidea;Veneridae;Mercenaria;Mercenaria mercenaria
UdeM00000005441 6579 608.6 UdeM00000005441 XP_033737507.1   64.1 501
162 4 1 487 1 497 8.20E-171 608.6 6579 cellular organisms;Eukaryota;Opisthokonta;Metazoa;Eumetazoa;Bilateria;Protostomia;Spiralia;Lophotrochozoa;Mollusca;Bivalvia;Autobranchia;Pteriomorphia;Pectinida;Pectinoidea;Pectinidae;Pecten;Pecten maximus
UdeM00000005442 6579 379 UdeM00000005442 XP_033729593.1   50.6 391
191 1 81 469 852 1242 8.60E-102 379 6579 cellular organisms;Eukaryota;Opisthokonta;Metazoa;Eumetazoa;Bilateria;Protostomia;Spiralia;Lophotrochozoa;Mollusca;Bivalvia;Autobranchia;Pteriomorphia;Pectinida;Pectinoidea;Pectinidae;Pecten;Pecten maximus
UdeM00000005443 6579 384.8 UdeM00000005443 XP_033729593.1   50.9 391
190 1 81 469 852 1242 1.60E-103 384.8 6579 cellular organisms;Eukaryota;Opisthokonta;Metazoa;Eumetazoa;Bilateria;Protostomia;Spiralia;Lophotrochozoa;Mollusca;Bivalvia;Autobranchia;Pteriomorphia;Pectinida;Pectinoidea;Pectinidae;Pecten;Pecten maximus
UdeM00000005444 6579 419.5 UdeM00000005444 XP_033729593.1   51.8 425
203 1 47 469 818 1242 5.70E-114 419.5 6579 cellular organisms;Eukaryota;Opisthokonta;Metazoa;Eumetazoa;Bilateria;Protostomia;Spiralia;Lophotrochozoa;Mollusca;Bivalvia;Autobranchia;Pteriomorphia;Pectinida;Pectinoidea;Pectinidae;Pecten;Pecten maximus
UdeM00000005445 29159 78.2 UdeM00000005445 XP_034316161.1   37.4 91
57 0 56 146 644 734 9.80E-12 78.2 29159 cellular organisms;Eukaryota;Opisthokonta;Metazoa;Eumetazoa;Bilateria;Protostomia;Spiralia;Lophotrochozoa;Mollusca;Bivalvia;Autobranchia;Pteriomorphia;Ostreida;Ostreoidea;Ostreidae;Crassostrea;Crassostrea gigas
UdeM00000005446 6596 461.5 UdeM00000005446 XP_045160038.1   60.9 373
140 3 1 368 1 372 1.00E-126 461.5 6596 cellular organisms;Eukaryota;Opisthokonta;Metazoa;Eumetazoa;Bilateria;Protostomia;Spiralia;Lophotrochozoa;Mollusca;Bivalvia;Autobranchia;Heteroconchia;Euheterodonta;Imparidentia;Venerida;Veneroidea;Veneridae;Mercenaria;Mercenaria mercenaria
UdeM00000005447 6412 110.2 UdeM00000005447 XP_009012269.1   37.9 161
93 2 62 216 2 161 4.20E-21 110.2 6412 cellular organisms;Eukaryota;Opisthokonta;Metazoa;Eumetazoa;Bilateria;Protostomia;Spiralia;Lophotrochozoa;Annelida;Clitellata;Hirudinea;Rhynchobdellida;Glossiphoniidae;Helobdella;Helobdella robusta
UdeM00000005448 6596 270.8 UdeM00000005448 XP_045160546.1   44.6 287
154 2 1 282 1 287 2.00E-69 270.8 6596 cellular organisms;Eukaryota;Opisthokonta;Metazoa;Eumetazoa;Bilateria;Protostomia;Spiralia;Lophotrochozoa;Mollusca;Bivalvia;Autobranchia;Heteroconchia;Euheterodonta;Imparidentia;Venerida;Veneroidea;Veneridae;Mercenaria;Mercenaria mercenaria

UdeM00000005449 225164 278.5 UdeM00000005449 XP_009044134.1 48.2
284 146 1 29 312 18 300 1.10E-71 278.5 225164
cellular
organisms;Eukaryota;Opisthokonta;Metazoa;Eumetazoa;Bilateria;Protostomia;
Spiralia;Lophotrochozoa;Mollusca;Gastropoda;Patellogastropoda;Lottioidea;
Lottiidae;Lottia;Lottia gigantea
UdeM00000005450 29159 153.3 UdeM00000005450 XP_011414040.3 66.4 128
40 2 73 198 98 224 3.80E-34 153.3 29159 cellular
organisms;Eukaryota;Opisthokonta;Metazoa;Eumetazoa;Bilateria;Protostomia;
Spiralia;Lophotrochozoa;Mollusca;Bivalvia;Autobranchia;Pteriomorphia;Ostr
eida;Ostreoidea;Ostreidae;Crassostrea;Crassostrea gigas
UdeM00000005451 6596 320.1 UdeM00000005451 XP_045160399.1 57.7 239
99 2 43 281 14 250 2.80E-84 320.1 6596 cellular
organisms;Eukaryota;Opisthokonta;Metazoa;Eumetazoa;Bilateria;Protostomia;
Spiralia;Lophotrochozoa;Mollusca;Bivalvia;Autobranchia;Heteroconchia;Euhe
terodonta;Imparidentia;Venerida;Veneroidea;Veneridae;Mercenaria;Mercenari
a mercenaria
UdeM00000005452 6565 578.2 UdeM00000005452 XP_022306197.1 47.9 698
311 13 9 684 16 682 1.40E-161 578.2 6565 cellular
organisms;Eukaryota;Opisthokonta;Metazoa;Eumetazoa;Bilateria;Protostomia;
Spiralia;Lophotrochozoa;Mollusca;Bivalvia;Autobranchia;Pteriomorphia;Ostr
eida;Ostreoidea;Ostreidae;Crassostrea;Crassostrea virginica
UdeM00000005453 6565 174.1 UdeM00000005453 XP_022316207.1 41.4 526
253 16 1 524 1 473 4.70E-40 174.1 6565 cellular
organisms;Eukaryota;Opisthokonta;Metazoa;Eumetazoa;Bilateria;Protostomia;
Spiralia;Lophotrochozoa;Mollusca;Bivalvia;Autobranchia;Pteriomorphia;Ostr
eida;Ostreoidea;Ostreidae;Crassostrea;Crassostrea virginica
UdeM00000005454 6596 374 UdeM00000005454 XP_045160273.1 37.3 526
244 5 5 447 21 543 3.00E-100 374 6596 cellular
organisms;Eukaryota;Opisthokonta;Metazoa;Eumetazoa;Bilateria;Protostomia;
Spiralia;Lophotrochozoa;Mollusca;Bivalvia;Autobranchia;Heteroconchia;Euhe
terodonta;Imparidentia;Venerida;Veneroidea;Veneridae;Mercenaria;Mercenari
a mercenaria
UdeM00000040756 6573 93.2 UdeM00000040756 XP_021365625.1 51.7 89
43 0 17 105 40 128 2.70E-16 93.2 6573 cellular
organisms;Eukaryota;Opisthokonta;Metazoa;Eumetazoa;Bilateria;Protostomia;
Spiralia;Lophotrochozoa;Mollusca;Bivalvia;Autobranchia;Pteriomorphia;Pect
inida;Pectinoidea;Pectinidae;Mizuhopecten;Mizuhopecten yessoensis
UdeM00000040757 29159 97.4 UdeM00000040757 XP_034307807.1 32.7 199
123 6 65 261 21 210 2.90E-17 97.4 29159 cellular
organisms;Eukaryota;Opisthokonta;Metazoa;Eumetazoa;Bilateria;Protostomia;
Spiralia;Lophotrochozoa;Mollusca;Bivalvia;Autobranchia;Pteriomorphia;Ostr
eida;Ostreoidea;Ostreidae;Crassostrea;Crassostrea gigas
UdeM00000040758 6596 294.3 UdeM00000040758 XP_045177776.1 36.8 508
270 9 1 461 405 908 2.80E-76 294.3 6596 cellular
organisms;Eukaryota;Opisthokonta;Metazoa;Eumetazoa;Bilateria;Protostomia;
Spiralia;Lophotrochozoa;Mollusca;Bivalvia;Autobranchia;Heteroconchia;Euhe
terodonta;Imparidentia;Venerida;Veneroidea;Veneridae;Mercenaria;Mercenari
a mercenaria
UdeM00000040759 29159 117.5 UdeM00000040759 XP_034307341.1 34 197
115 3 8 190 57 252 2.80E-23 117.5 29159 cellular
organisms;Eukaryota;Opisthokonta;Metazoa;Eumetazoa;Bilateria;Protostomia;
Spiralia;Lophotrochozoa;Mollusca;Bivalvia;Autobranchia;Pteriomorphia;Ostr
eida;Ostreoidea;Ostreidae;Crassostrea;Crassostrea gigas

UdeM00000040762 6565 90.5 UdeM00000040762 XP_022318775.1 46.4 110
55 3 1 107 1 109 1.70E-15 90.5 6565 cellular
organisms;Eukaryota;Opisthokonta;Metazoa;Eumetazoa;Bilateria;Protostomia;
Spiralia;Lophotrochozoa;Mollusca;Bivalvia;Autobranchia;Pteriomorphia;Ostr
eida;Ostreoidea;Ostreidae;Crassostrea;Crassostrea virginica
UdeM00000040763 37621 250 UdeM00000040763 XP_014673981.1 48.9 278
124 8 53 314 37 312 4.10E-63 250 37621 cellular
organisms;Eukaryota;Opisthokonta;Metazoa;Eumetazoa;Bilateria;Protostomia;
Ecdysozoa;Scalidophora;Priapulida;Priapulimorpha;Priapulimorphida;Priapul
idae;Priapulus;Priapulus caudatus
UdeM00000040764 6596 93.2 UdeM00000040764 XP_045179120.1 57.3 75
31 1 1 75 1 74 1.60E-16 93.2 6596 cellular
organisms;Eukaryota;Opisthokonta;Metazoa;Eumetazoa;Bilateria;Protostomia;
Spiralia;Lophotrochozoa;Mollusca;Bivalvia;Autobranchia;Heteroconchia;Euhe
terodonta;Imparidentia;Venerida;Veneroidea;Veneridae;Mercenaria;Mercenari
a mercenaria
UdeM00000040765 50429 158.7 UdeM00000040765 XP_022801251.1 39.3 224
123 7 2 217 798 1016 9.10E-36 158.7 50429 cellular
organisms;Eukaryota;Opisthokonta;Metazoa;Eumetazoa;Cnidaria;Anthozoa;Hexa
corallia;Scleractinia;Astrocoeniina;Pocilloporidae;Stylophora;Stylophora
pistillata
UdeM00000040767 7574 138.3 UdeM00000040767 XP_013390464.1 36.9 225
123 7 2 215 770 986 1.20E-29 138.3 7574 cellular
organisms;Eukaryota;Opisthokonta;Metazoa;Eumetazoa;Bilateria;Protostomia;
Spiralia;Lophotrochozoa;Brachiopoda;Linguliformea;Lingulata;Lingulida;Lin
guloidea;Lingulidae;Lingula;Lingula anatina
UdeM00000040766 7574 137.9 UdeM00000040766 XP_013390464.1 36.9 225
123 7 2 215 770 986 1.60E-29 137.9 7574 cellular
organisms;Eukaryota;Opisthokonta;Metazoa;Eumetazoa;Bilateria;Protostomia;
Spiralia;Lophotrochozoa;Brachiopoda;Linguliformea;Lingulata;Lingulida;Lin
guloidea;Lingulidae;Lingula;Lingula anatina
UdeM00000040768 7574 134 UdeM00000040768 XP_013390464.1 35.2 219
132 5 2 212 770 986 2.30E-28 134 7574 cellular
organisms;Eukaryota;Opisthokonta;Metazoa;Eumetazoa;Bilateria;Protostomia;
Spiralia;Lophotrochozoa;Brachiopoda;Linguliformea;Lingulata;Lingulida;Lin
guloidea;Lingulidae;Lingula;Lingula anatina
UdeM00000040770 6596 159.5 UdeM00000040770 XP_045189228.1 33.3 336
178 7 1 334 50 341 1.40E-35 159.5 6596 cellular
organisms;Eukaryota;Opisthokonta;Metazoa;Eumetazoa;Bilateria;Protostomia;
Spiralia;Lophotrochozoa;Mollusca;Bivalvia;Autobranchia;Heteroconchia;Euhe
terodonta;Imparidentia;Venerida;Veneroidea;Veneridae;Mercenaria;Mercenari
a mercenaria
UdeM00000040771 6573 114.4 UdeM00000040771 XP_021354477.1 43.2 132
75 0 85 216 1988 2119 1.80E-22 114.4 6573 cellular
organisms;Eukaryota;Opisthokonta;Metazoa;Eumetazoa;Bilateria;Protostomia;
Spiralia;Lophotrochozoa;Mollusca;Bivalvia;Autobranchia;Pteriomorphia;Pect
inida;Pectinoidea;Pectinidae;Mizuhopecten;Mizuhopecten yessoensis
UdeM00000040772 6596 226.9 UdeM00000040772 XP_045164107.1 52.9 221
100 2 2 221 2620 2837 2.60E-56 226.9 6596 cellular
organisms;Eukaryota;Opisthokonta;Metazoa;Eumetazoa;Bilateria;Protostomia;
Spiralia;Lophotrochozoa;Mollusca;Bivalvia;Autobranchia;Heteroconchia;Euhe
terodonta;Imparidentia;Venerida;Veneroidea;Veneridae;Mercenaria;Mercenari
a mercenaria

UdeM00000040773	6596	172.6 UdeM00000040773	XP_045164107.1	48.9	180
84	1	44	223	2239	2410	5.90E-40	172.6 6596	cellular organisms;Eukaryota;Opisthokonta;Metazoa;Eumetazoa;Bilateria;Protostomia;Spiralia;Lophotrochozoa;Mollusca;Bivalvia;Autobranchia;Heteroconchia;Euheterodonta;Imparidentia;Venerida;Veneroidea;Veneridae;Mercenaria;Mercenaria mercenaria
UdeM00000040774	6596	359.8 UdeM00000040774	XP_045177776.1	39.4	528
313	6	1	523	405	930	8.90E-96	359.8 6596	cellular organisms;Eukaryota;Opisthokonta;Metazoa;Eumetazoa;Bilateria;Protostomia;Spiralia;Lophotrochozoa;Mollusca;Bivalvia;Autobranchia;Heteroconchia;Euheterodonta;Imparidentia;Venerida;Veneroidea;Veneridae;Mercenaria;Mercenaria mercenaria
UdeM00000040775	6579	508.8 UdeM00000040775	XP_033761495.1	78.9	294
62	0	53	346	66	359	8.60E-141	508.8 6579	cellular organisms;Eukaryota;Opisthokonta;Metazoa;Eumetazoa;Bilateria;Protostomia;Spiralia;Lophotrochozoa;Mollusca;Bivalvia;Autobranchia;Pteriomorphia;Pectinida;Pectinoidea;Pectinidae;Pecten;Pecten maximus
UdeM00000040776	7574	132.1 UdeM00000040776	XP_013389395.1	37.1	224
107	7	2	198	749	965	8.30E-28	132.1 7574	cellular organisms;Eukaryota;Opisthokonta;Metazoa;Eumetazoa;Bilateria;Protostomia;Spiralia;Lophotrochozoa;Brachiopoda;Linguliformea;Lingulata;Lingulida;Linguloidea;Lingulidae;Lingula;Lingula anatina
UdeM00000040777	7574	146	UdeM00000040777	XP_013390464.1	37.3	225
122	7	2	215	770	986	6.00E-32	146	7574	cellular organisms;Eukaryota;Opisthokonta;Metazoa;Eumetazoa;Bilateria;Protostomia;Spiralia;Lophotrochozoa;Brachiopoda;Linguliformea;Lingulata;Lingulida;Linguloidea;Lingulidae;Lingula;Lingula anatina
UdeM00000040778	7574	134.4 UdeM00000040778	XP_013389395.1	35.3	221
129	6	2	212	749	965	1.80E-28	134.4 7574	cellular organisms;Eukaryota;Opisthokonta;Metazoa;Eumetazoa;Bilateria;Protostomia;Spiralia;Lophotrochozoa;Brachiopoda;Linguliformea;Lingulata;Lingulida;Linguloidea;Lingulidae;Lingula;Lingula anatina
UdeM00000040779	7574	139.8 UdeM00000040779	XP_013389395.1	36.1	219
130	5	2	212	749	965	4.30E-30	139.8 7574	cellular organisms;Eukaryota;Opisthokonta;Metazoa;Eumetazoa;Bilateria;Protostomia;Spiralia;Lophotrochozoa;Brachiopoda;Linguliformea;Lingulata;Lingulida;Linguloidea;Lingulidae;Lingula;Lingula anatina
UdeM00000040780	37653 201.8 UdeM00000040780	XP_014767494.1	81.5	119
22	0	34	152	939	1057	6.10E-49	201.8 37653 cellular organisms;Eukaryota;Opisthokonta;Metazoa;Eumetazoa;Bilateria;Protostomia;Spiralia;Lophotrochozoa;Mollusca;Cephalopoda;Coleoidea;Octopodiformes;Octopoda;Incirrata;Octopodidae;Octopus;Octopus bimaculoides
UdeM00000040781	37621 184.5 UdeM00000040781	XP_014679849.1	38.7	230
55	2	2	145	394	623	9.70E-44	184.5 37621 cellular organisms;Eukaryota;Opisthokonta;Metazoa;Eumetazoa;Bilateria;Protostomia;Ecdysozoa;Scalidophora;Priapulida;Priapulimorpha;Priapulimorphida;Priapulidae;Priapulus;Priapulus caudatus
UdeM00000040782	37621 144.8 UdeM00000040782	XP_014679849.1	40.4	213
51	2	1	137	17	229	8.50E-32	144.8 37621 cellular organisms;Eukaryota;Opisthokonta;Metazoa;Eumetazoa;Bilateria;Protostomia;Ecdysozoa;Scalidophora;Priapulida;Priapulimorpha;Priapulimorphida;Priapulidae;Priapulus;Priapulus caudatus
UdeM00000040783	6596	309.3 UdeM00000040783	XP_045189084.1	32.3	626
317	12	1	616	1350	1878	1.40E-80	309.3 6596	cellular

organisms;Eukaryota;Opisthokonta;Metazoa;Eumetazoa;Bilateria;Protostomia;Spiralia;Lophotrochozoa;Mollusca;Bivalvia;Autobranchia;Heteroconchia;Euheterodonta;Imparidentia;Venerida;Veneroidea;Veneridae;Mercenaria;Mercenaria mercenaria
UdeM00000040784 6579 92.8 UdeM00000040784 XP_033729770.1 41.4 133 51 4 1 117 1 122 3.20E-16 92.8 6579 cellular organisms;Eukaryota;Opisthokonta;Metazoa;Eumetazoa;Bilateria;Protostomia;Spiralia;Lophotrochozoa;Mollusca;Bivalvia;Autobranchia;Pteriomorphia;Pectinida;Pectinoidea;Pectinidae;Pecten;Pecten maximus
UdeM00000040785 6579 197.2 UdeM00000040785 XP_033764274.1 40.3 233 138 1 2 234 238 469 2.40E-47 197.2 6579 cellular organisms;Eukaryota;Opisthokonta;Metazoa;Eumetazoa;Bilateria;Protostomia;Spiralia;Lophotrochozoa;Mollusca;Bivalvia;Autobranchia;Pteriomorphia;Pectinida;Pectinoidea;Pectinidae;Pecten;Pecten maximus
UdeM00000040786 6579 165.6 UdeM00000040786 XP_033747958.1 55.3 132 59 0 15 146 28 159 4.70E-38 165.6 6579 cellular organisms;Eukaryota;Opisthokonta;Metazoa;Eumetazoa;Bilateria;Protostomia;Spiralia;Lophotrochozoa;Mollusca;Bivalvia;Autobranchia;Pteriomorphia;Pectinida;Pectinoidea;Pectinidae;Pecten;Pecten maximus
UdeM00000040787 6596 94.4 UdeM00000040787 XP_045164107.1 47.9 94 49 0 1 94 1241 1334 8.70E-17 94.4 6596 cellular organisms;Eukaryota;Opisthokonta;Metazoa;Eumetazoa;Bilateria;Protostomia;Spiralia;Lophotrochozoa;Mollusca;Bivalvia;Autobranchia;Heteroconchia;Euheterodonta;Imparidentia;Venerida;Veneroidea;Veneridae;Mercenaria;Mercenaria mercenaria
UdeM00000040790 6596 385.6 UdeM00000040790 XP_045177776.1 38.7 550 307 12 1 541 405 933 1.50E-103 385.6 6596 cellular organisms;Eukaryota;Opisthokonta;Metazoa;Eumetazoa;Bilateria;Protostomia;Spiralia;Lophotrochozoa;Mollusca;Bivalvia;Autobranchia;Heteroconchia;Euheterodonta;Imparidentia;Venerida;Veneroidea;Veneridae;Mercenaria;Mercenaria mercenaria
UdeM00000040791 6596 888.6 UdeM00000040791 XP_045164107.1 54.5 846 324 6 26 865 2180 2970 6.20E-255 888.6 6596 cellular organisms;Eukaryota;Opisthokonta;Metazoa;Eumetazoa;Bilateria;Protostomia;Spiralia;Lophotrochozoa;Mollusca;Bivalvia;Autobranchia;Heteroconchia;Euheterodonta;Imparidentia;Venerida;Veneroidea;Veneridae;Mercenaria;Mercenaria mercenaria
UdeM00000040792 1735272 459.1 UdeM00000040792 XP_041360663.1 35.3 914 475 24 115 969 848 1704 1.40E-125 459.1 1735272 cellular organisms;Eukaryota;Opisthokonta;Metazoa;Eumetazoa;Bilateria;Protostomia;Spiralia;Lophotrochozoa;Mollusca;Gastropoda;Neomphalina;Neomphaloidea;Peltospiridae;Gigantopelta;Gigantopelta aegis
UdeM00000040793 6596 70.1 UdeM00000040793 XP_045206414.1 30.9 97 67 0 44 140 109 205 4.60E-09 70.1 6596 cellular organisms;Eukaryota;Opisthokonta;Metazoa;Eumetazoa;Bilateria;Protostomia;Spiralia;Lophotrochozoa;Mollusca;Bivalvia;Autobranchia;Heteroconchia;Euheterodonta;Imparidentia;Venerida;Veneroidea;Veneridae;Mercenaria;Mercenaria mercenaria
UdeM00000040794 6596 161.8 UdeM00000040794 XP_045156273.1 34.9 352 188 10 1 347 569 884 2.30E-36 161.8 6596 cellular organisms;Eukaryota;Opisthokonta;Metazoa;Eumetazoa;Bilateria;Protostomia;Spiralia;Lophotrochozoa;Mollusca;Bivalvia;Autobranchia;Heteroconchia;Euhe

terodonta;Imparidentia;Venerida;Veneroidea;Veneridae;Mercenaria;Mercenari
a mercenaria
UdeM00000040795 6596 153.7 UdeM00000040795 XP_045177776.1 39.8 201
101 2 1 201 695 875 2.70E-34 153.7 6596 cellular
organisms;Eukaryota;Opisthokonta;Metazoa;Eumetazoa;Bilateria;Protostomia;
Spiralia;Lophotrochozoa;Mollusca;Bivalvia;Autobranchia;Heteroconchia;Euhe
terodonta;Imparidentia;Venerida;Veneroidea;Veneridae;Mercenaria;Mercenari
a mercenaria
UdeM00000040796 6565 95.5 UdeM00000040796 XP_022308598.1 37.7 162
92 5 170 331 15 167 1.40E-16 95.5 6565 cellular
organisms;Eukaryota;Opisthokonta;Metazoa;Eumetazoa;Bilateria;Protostomia;
Spiralia;Lophotrochozoa;Mollusca;Bivalvia;Autobranchia;Pteriomorphia;Ostr
eida;Ostreoidea;Ostreidae;Crassostrea;Crassostrea virginica
UdeM00000040797 6565 95.5 UdeM00000040797 XP_022308598.1 37.7 162
92 5 23 184 15 167 8.40E-17 95.5 6565 cellular
organisms;Eukaryota;Opisthokonta;Metazoa;Eumetazoa;Bilateria;Protostomia;
Spiralia;Lophotrochozoa;Mollusca;Bivalvia;Autobranchia;Pteriomorphia;Ostr
eida;Ostreoidea;Ostreidae;Crassostrea;Crassostrea virginica
UdeM00000040798 7739 723 UdeM00000040798 XP_035696678.1 56.9 613
261 1 7 616 299 911 3.30E-205 723 7739 cellular
organisms;Eukaryota;Opisthokonta;Metazoa;Eumetazoa;Bilateria;Deuterostomi
a;Chordata;Cephalochordata;Leptocardii;Amphioxiformes;Branchiostomidae;Br
anchiostoma;Branchiostoma floridae
UdeM00000040799 6565 229.9 UdeM00000040799 XP_022327931.1 36.8 359
208 7 38 387 26 374 5.40E-57 229.9 6565 cellular
organisms;Eukaryota;Opisthokonta;Metazoa;Eumetazoa;Bilateria;Protostomia;
Spiralia;Lophotrochozoa;Mollusca;Bivalvia;Autobranchia;Pteriomorphia;Ostr
eida;Ostreoidea;Ostreidae;Crassostrea;Crassostrea virginica
UdeM00000040800 29159 109.8 UdeM00000040800 XP_034305547.1 32.4 204
127 6 25 226 21 215 4.90E-21 109.8 29159 cellular
organisms;Eukaryota;Opisthokonta;Metazoa;Eumetazoa;Bilateria;Protostomia;
Spiralia;Lophotrochozoa;Mollusca;Bivalvia;Autobranchia;Pteriomorphia;Ostr
eida;Ostreoidea;Ostreidae;Crassostrea;Crassostrea gigas
UdeM00000040801 6596 179.1 UdeM00000040801 XP_045164107.1 32.1 439
104 4 22 268 1811 2247 1.30E-41 179.1 6596 cellular
organisms;Eukaryota;Opisthokonta;Metazoa;Eumetazoa;Bilateria;Protostomia;
Spiralia;Lophotrochozoa;Mollusca;Bivalvia;Autobranchia;Heteroconchia;Euhe
terodonta;Imparidentia;Venerida;Veneroidea;Veneridae;Mercenaria;Mercenari
a mercenaria
UdeM00000040801 6596 124.8 UdeM00000040801 XP_045164107.1 55.6 108
48 0 308 415 2730 2837 2.90E-25 124.8 6596 cellular
organisms;Eukaryota;Opisthokonta;Metazoa;Eumetazoa;Bilateria;Protostomia;
Spiralia;Lophotrochozoa;Mollusca;Bivalvia;Autobranchia;Heteroconchia;Euhe
terodonta;Imparidentia;Venerida;Veneroidea;Veneridae;Mercenaria;Mercenari
a mercenaria
UdeM00000044273 6579 430.3 UdeM00000044273 XP_033761812.1 40.7 575
319 12 123 686 151 714 4.80E-117 430.3 6579 cellular
organisms;Eukaryota;Opisthokonta;Metazoa;Eumetazoa;Bilateria;Protostomia;
Spiralia;Lophotrochozoa;Mollusca;Bivalvia;Autobranchia;Pteriomorphia;Pect
inida;Pectinoidea;Pectinidae;Pecten;Pecten maximus
UdeM00000044274 7574 79 UdeM00000044274 XP_013401006.1 60.6 66
26 0 19 84 422 487 4.60E-12 79 7574 cellular
organisms;Eukaryota;Opisthokonta;Metazoa;Eumetazoa;Bilateria;Protostomia;

Spiralia;Lophotrochozoa;Brachiopoda;Linguliformea;Lingulata;Lingulida;Linguloidea;Lingulidae;Lingula;Lingula anatina
UdeM00000044275 6579 306.6 UdeM00000044275 XP_033757668.1 42.2 405 222 8 1 397 310 710 4.70E-80 306.6 6579 cellular organisms;Eukaryota;Opisthokonta;Metazoa;Eumetazoa;Bilateria;Protostomia;Spiralia;Lophotrochozoa;Mollusca;Bivalvia;Autobranchia;Pteriomorphia;Pectinida;Pectinoidea;Pectinidae;Pecten;Pecten maximus
UdeM00000044190 29159 117.5 UdeM00000044190 XP_034329650.1 45.6 103 56 0 1 103 276 378 1.00E-23 117.5 29159 cellular organisms;Eukaryota;Opisthokonta;Metazoa;Eumetazoa;Bilateria;Protostomia;Spiralia;Lophotrochozoa;Mollusca;Bivalvia;Autobranchia;Pteriomorphia;Ostreida;Ostreoidea;Ostreidae;Crassostrea;Crassostrea gigas
UdeM00000044191 6573 258.1 UdeM00000044191 XP_021352044.1 68 172 55 0 1 172 98 269 9.10E-66 258.1 6573 cellular organisms;Eukaryota;Opisthokonta;Metazoa;Eumetazoa;Bilateria;Protostomia;Spiralia;Lophotrochozoa;Mollusca;Bivalvia;Autobranchia;Pteriomorphia;Pectinida;Pectinoidea;Pectinidae;Mizuhopecten;Mizuhopecten yessoensis
UdeM00000034378 6579 137.1 UdeM00000034378 XP_033736448.1 35.8 218 129 5 5 213 10 225 2.60E-29 137.1 6579 cellular organisms;Eukaryota;Opisthokonta;Metazoa;Eumetazoa;Bilateria;Protostomia;Spiralia;Lophotrochozoa;Mollusca;Bivalvia;Autobranchia;Pteriomorphia;Pectinida;Pectinoidea;Pectinidae;Pecten;Pecten maximus
UdeM00000034379 29159 338.2 UdeM00000034379 XP_034305965.1 44.2 416 204 12 6 412 6 402 1.50E-89 338.2 29159 cellular organisms;Eukaryota;Opisthokonta;Metazoa;Eumetazoa;Bilateria;Protostomia;Spiralia;Lophotrochozoa;Mollusca;Bivalvia;Autobranchia;Pteriomorphia;Ostreida;Ostreoidea;Ostreidae;Crassostrea;Crassostrea gigas
UdeM00000034381 6596 88.2 UdeM00000034381 XP_045175388.1 34.1 132 72 3 5 121 257 388 8.30E-15 88.2 6596 cellular organisms;Eukaryota;Opisthokonta;Metazoa;Eumetazoa;Bilateria;Protostomia;Spiralia;Lophotrochozoa;Mollusca;Bivalvia;Autobranchia;Heteroconchia;Euheterodonta;Imparidentia;Venerida;Veneroidea;Veneridae;Mercenaria;Mercenaria mercenaria
UdeM00000034384 6565 515 UdeM00000034384 XP_022322118.1 37.8 1293 475 22 17 1273 16 1015 2.90E-142 515 6565 cellular organisms;Eukaryota;Opisthokonta;Metazoa;Eumetazoa;Bilateria;Protostomia;Spiralia;Lophotrochozoa;Mollusca;Bivalvia;Autobranchia;Pteriomorphia;Ostreida;Ostreoidea;Ostreidae;Crassostrea;Crassostrea virginica
UdeM00000034385 46514 63.9 UdeM00000034385 XP_038062328.1 29.6 152 105 1 1 150 225 376 2.00E-07 63.9 46514 cellular organisms;Eukaryota;Opisthokonta;Metazoa;Eumetazoa;Bilateria;Deuterostomia;Echinodermata;Eleutherozoa;Asterozoa;Asteroidea;Valvatacea;Valvatida;Asterinidae;Patiria;Patiria miniata
UdeM00000034387 6596 919.5 UdeM00000034387 XP_045207427.1 66.3 664 221 2 56 716 8 671 2.70E-264 919.5 6596 cellular organisms;Eukaryota;Opisthokonta;Metazoa;Eumetazoa;Bilateria;Protostomia;Spiralia;Lophotrochozoa;Mollusca;Bivalvia;Autobranchia;Heteroconchia;Euheterodonta;Imparidentia;Venerida;Veneroidea;Veneridae;Mercenaria;Mercenaria mercenaria
UdeM00000034388 6596 183 UdeM00000034388 XP_045209523.1 72.8 114 31 0 18 131 11 124 2.60E-43 183 6596 cellular organisms;Eukaryota;Opisthokonta;Metazoa;Eumetazoa;Bilateria;Protostomia;Spiralia;Lophotrochozoa;Mollusca;Bivalvia;Autobranchia;Heteroconchia;Euhe

terodonta;Imparidentia;Venerida;Veneroidea;Veneridae;Mercenaria;Mercenaria mercenaria
UdeM00000034390 1735272 63.5 UdeM00000034390 XP_041350990.1 46.8 62 33 0 125 186 461 522 3.20E-07 63.5 1735272 cellular organisms;Eukaryota;Opisthokonta;Metazoa;Eumetazoa;Bilateria;Protostomia;Spiralia;Lophotrochozoa;Mollusca;Gastropoda;Neomphalina;Neomphaloidea;Peltospiridae;Gigantopelta;Gigantopelta aegis
UdeM00000034391 6105 120.6 UdeM00000034391 XP_031551974.1 49.6 129 64 1 1 129 525 652 1.70E-24 120.6 6105 cellular organisms;Eukaryota;Opisthokonta;Metazoa;Eumetazoa;Cnidaria;Anthozoa;Hexacorallia;Actiniaria;Actiniidae;Actinia;Actinia tenebrosa
UdeM00000034394 50390 221.5 UdeM00000034394 XP_028150043.1 41.5 275 153 3 1 268 26 299 1.40E-54 221.5 50390 cellular organisms;Eukaryota;Opisthokonta;Metazoa;Eumetazoa;Bilateria;Protostomia;Ecdysozoa;Panarthropoda;Arthropoda;Mandibulata;Pancrustacea;Hexapoda;Insecta;Dicondylia;Pterygota;Neoptera;Endopterygota;Coleoptera;Polyphaga;Cucujiformia;Chrysomeloidea;Chrysomelidae;Galerucinae;Luperini;Diabroticina;Diabroticites;Diabrotica;Diabrotica virgifera;Diabrotica virgifera virgifera
UdeM00000034395 6596 172.6 UdeM00000034395 XP_045160979.1 45.9 209 109 2 6 212 8 214 5.80E-40 172.6 6596 cellular organisms;Eukaryota;Opisthokonta;Metazoa;Eumetazoa;Bilateria;Protostomia;Spiralia;Lophotrochozoa;Mollusca;Bivalvia;Autobranchia;Heteroconchia;Euheterodonta;Imparidentia;Venerida;Veneroidea;Veneridae;Mercenaria;Mercenaria mercenaria
UdeM00000034396 6105 193.7 UdeM00000034396 XP_031571719.1 26 792 490 22 234 969 40 791 1.30E-45 193.7 6105 cellular organisms;Eukaryota;Opisthokonta;Metazoa;Eumetazoa;Cnidaria;Anthozoa;Hexacorallia;Actiniaria;Actiniidae;Actinia;Actinia tenebrosa
UdeM00000034400 6579 1328.2 UdeM00000034400 XP_033750824.1 55.3 1208 490 13 1 1196 302 1471 0.00E+00 1328.2 6579 cellular organisms;Eukaryota;Opisthokonta;Metazoa;Eumetazoa;Bilateria;Protostomia;Spiralia;Lophotrochozoa;Mollusca;Bivalvia;Autobranchia;Pteriomorphia;Pectinida;Pectinoidea;Pectinidae;Pecten;Pecten maximus
UdeM00000034402 1437191 172.9 UdeM00000034402 XP_029054816.1 29.5 403 160 6 3 282 61 462 5.70E-40 172.9 1437191 cellular organisms;Eukaryota;Opisthokonta;Metazoa;Eumetazoa;Bilateria;Protostomia;Ecdysozoa;Panarthropoda;Arthropoda;Mandibulata;Pancrustacea;Hexapoda;Insecta;Dicondylia;Pterygota;Neoptera;Endopterygota;Hymenoptera;Apocrita;Aculeata;Apoidea;Megachilidae;Megachilinae;Osmiini;Osmia;Osmia bicornis;Osmia bicornis bicornis
UdeM00000034404 6596 915.2 UdeM00000034404 XP_045210363.1 54.6 852 378 4 206 1053 118 964 7.50E-263 915.2 6596 cellular organisms;Eukaryota;Opisthokonta;Metazoa;Eumetazoa;Bilateria;Protostomia;Spiralia;Lophotrochozoa;Mollusca;Bivalvia;Autobranchia;Heteroconchia;Euheterodonta;Imparidentia;Venerida;Veneroidea;Veneridae;Mercenaria;Mercenaria mercenaria
UdeM00000034406 6596 278.1 UdeM00000034406 XP_045183805.1 59 205 83 1 1 204 45 249 9.00E-72 278.1 6596 cellular organisms;Eukaryota;Opisthokonta;Metazoa;Eumetazoa;Bilateria;Protostomia;Spiralia;Lophotrochozoa;Mollusca;Bivalvia;Autobranchia;Heteroconchia;Euhe

terodonta;Imparidentia;Venerida;Veneroidea;Veneridae;Mercenaria;Mercenari
a mercenaria
UdeM00000034405 6596 279.6 UdeM00000034405 XP_045183805.1 58.7 206
84 1 20 224 44 249 3.40E-72 279.6 6596 cellular
organisms;Eukaryota;Opisthokonta;Metazoa;Eumetazoa;Bilateria;Protostomia;
Spiralia;Lophotrochozoa;Mollusca;Bivalvia;Autobranchia;Heteroconchia;Euhe
terodonta;Imparidentia;Venerida;Veneroidea;Veneridae;Mercenaria;Mercenari
a mercenaria
UdeM00000034407 6596 309.3 UdeM00000034407 XP_045183805.1 65.6 209
71 1 1 208 41 249 3.70E-81 309.3 6596 cellular
organisms;Eukaryota;Opisthokonta;Metazoa;Eumetazoa;Bilateria;Protostomia;
Spiralia;Lophotrochozoa;Mollusca;Bivalvia;Autobranchia;Heteroconchia;Euhe
terodonta;Imparidentia;Venerida;Veneroidea;Veneridae;Mercenaria;Mercenari
a mercenaria
UdeM00000034408 6596 415.2 UdeM00000034408 XP_045184513.1 44.5 1013
503 14 17 988 19 1013 2.50E-112 415.2 6596 cellular
organisms;Eukaryota;Opisthokonta;Metazoa;Eumetazoa;Bilateria;Protostomia;
Spiralia;Lophotrochozoa;Mollusca;Bivalvia;Autobranchia;Heteroconchia;Euhe
terodonta;Imparidentia;Venerida;Veneroidea;Veneridae;Mercenaria;Mercenari
a mercenaria
UdeM00000034409 6596 187.2 UdeM00000034409 XP_045183513.1 36.3 582
338 7 544 1105 416 984 1.40E-43 187.2 6596 cellular
organisms;Eukaryota;Opisthokonta;Metazoa;Eumetazoa;Bilateria;Protostomia;
Spiralia;Lophotrochozoa;Mollusca;Bivalvia;Autobranchia;Heteroconchia;Euhe
terodonta;Imparidentia;Venerida;Veneroidea;Veneridae;Mercenaria;Mercenari
a mercenaria
UdeM00000034410 6596 295.4 UdeM00000034410 XP_045173349.1 27.7 860
481 23 142 878 69 910 2.80E-76 295.4 6596 cellular
organisms;Eukaryota;Opisthokonta;Metazoa;Eumetazoa;Bilateria;Protostomia;
Spiralia;Lophotrochozoa;Mollusca;Bivalvia;Autobranchia;Heteroconchia;Euhe
terodonta;Imparidentia;Venerida;Veneroidea;Veneridae;Mercenaria;Mercenari
a mercenaria
UdeM00000034411 225164 325.5 UdeM00000034411 XP_009057189.1 42.8
397 223 2 60 456 9 401 1.10E-85 325.5 225164
cellular
organisms;Eukaryota;Opisthokonta;Metazoa;Eumetazoa;Bilateria;Protostomia;
Spiralia;Lophotrochozoa;Mollusca;Gastropoda;Patellogastropoda;Lottioidea;
Lottiidae;Lottia;Lottia gigantea
UdeM00000034412 6596 503.8 UdeM00000034412 XP_045207654.1 51.7 536
234 9 263 784 156 680 3.90E-139 503.8 6596 cellular
organisms;Eukaryota;Opisthokonta;Metazoa;Eumetazoa;Bilateria;Protostomia;
Spiralia;Lophotrochozoa;Mollusca;Bivalvia;Autobranchia;Heteroconchia;Euhe
terodonta;Imparidentia;Venerida;Veneroidea;Veneridae;Mercenaria;Mercenari
a mercenaria
UdeM00000034413 6596 172.2 UdeM00000034413 XP_045207747.1 34.8 287
177 5 1 278 1 286 9.60E-40 172.2 6596 cellular
organisms;Eukaryota;Opisthokonta;Metazoa;Eumetazoa;Bilateria;Protostomia;
Spiralia;Lophotrochozoa;Mollusca;Bivalvia;Autobranchia;Heteroconchia;Euhe
terodonta;Imparidentia;Venerida;Veneroidea;Veneridae;Mercenaria;Mercenari
a mercenaria
UdeM00000034414 6596 298.9 UdeM00000034414 XP_045209765.1 26 850
463 20 533 1342 251 974 3.30E-77 298.9 6596 cellular
organisms;Eukaryota;Opisthokonta;Metazoa;Eumetazoa;Bilateria;Protostomia;
Spiralia;Lophotrochozoa;Mollusca;Bivalvia;Autobranchia;Heteroconchia;Euhe

terodonta;Imparidentia;Venerida;Veneroidea;Veneridae;Mercenaria;Mercenaria mercenaria
UdeM00000034415 6596 172.9 UdeM00000034415 XP_045209769.1 23.8 583 394 12 45 587 449 1021 1.20E-39 172.9 6596 cellular organisms;Eukaryota;Opisthokonta;Metazoa;Eumetazoa;Bilateria;Protostomia;Spiralia;Lophotrochozoa;Mollusca;Bivalvia;Autobranchia;Heteroconchia;Euheterodonta;Imparidentia;Venerida;Veneroidea;Veneridae;Mercenaria;Mercenaria mercenaria
UdeM00000034416 6596 131 UdeM00000034416 XP_045209768.1 23.3 520 357 11 122 609 449 958 5.70E-27 131 6596 cellular organisms;Eukaryota;Opisthokonta;Metazoa;Eumetazoa;Bilateria;Protostomia;Spiralia;Lophotrochozoa;Mollusca;Bivalvia;Autobranchia;Heteroconchia;Euheterodonta;Imparidentia;Venerida;Veneroidea;Veneridae;Mercenaria;Mercenaria mercenaria
UdeM00000034417 6596 414.1 UdeM00000034417 XP_045209768.1 28.8 1060 606 26 223 1249 33 976 7.00E-112 414.1 6596 cellular organisms;Eukaryota;Opisthokonta;Metazoa;Eumetazoa;Bilateria;Protostomia;Spiralia;Lophotrochozoa;Mollusca;Bivalvia;Autobranchia;Heteroconchia;Euheterodonta;Imparidentia;Venerida;Veneroidea;Veneridae;Mercenaria;Mercenaria mercenaria
UdeM00000034419 6596 171 UdeM00000034419 XP_045209765.1 24 578 393 10 149 687 560 1130 5.20E-39 171 6596 cellular organisms;Eukaryota;Opisthokonta;Metazoa;Eumetazoa;Bilateria;Protostomia;Spiralia;Lophotrochozoa;Mollusca;Bivalvia;Autobranchia;Heteroconchia;Euheterodonta;Imparidentia;Venerida;Veneroidea;Veneridae;Mercenaria;Mercenaria mercenaria
UdeM00000034420 6596 155.6 UdeM00000034420 XP_045209769.1 25.7 460 310 10 64 501 474 923 2.10E-34 155.6 6596 cellular organisms;Eukaryota;Opisthokonta;Metazoa;Eumetazoa;Bilateria;Protostomia;Spiralia;Lophotrochozoa;Mollusca;Bivalvia;Autobranchia;Heteroconchia;Euheterodonta;Imparidentia;Venerida;Veneroidea;Veneridae;Mercenaria;Mercenaria mercenaria
UdeM00000034422 6596 162.2 UdeM00000034422 XP_045209760.1 52.6 133 63 0 1027 1159 533 665 4.70E-36 162.2 6596 cellular organisms;Eukaryota;Opisthokonta;Metazoa;Eumetazoa;Bilateria;Protostomia;Spiralia;Lophotrochozoa;Mollusca;Bivalvia;Autobranchia;Heteroconchia;Euheterodonta;Imparidentia;Venerida;Veneroidea;Veneridae;Mercenaria;Mercenaria mercenaria
UdeM00000034423 6596 94.4 UdeM00000034423 XP_045210122.1 38.8 134 72 5 129 258 57 184 2.40E-16 94.4 6596 cellular organisms;Eukaryota;Opisthokonta;Metazoa;Eumetazoa;Bilateria;Protostomia;Spiralia;Lophotrochozoa;Mollusca;Bivalvia;Autobranchia;Heteroconchia;Euheterodonta;Imparidentia;Venerida;Veneroidea;Veneridae;Mercenaria;Mercenaria mercenaria
UdeM00000034424 6956 63.5 UdeM00000034424 XP_027204985.1 28.8 125 73 4 35 145 131 253 2.80E-07 63.5 6956 cellular organisms;Eukaryota;Opisthokonta;Metazoa;Eumetazoa;Bilateria;Protostomia;Ecdysozoa;Panarthropoda;Arthropoda;Chelicerata;Arachnida;Acari;Acariformes;Sarcoptiformes;Astigmata;Psoroptidia;Analgoidea;Pyroglyphidae;Dermatophagoidinae;Dermatophagoides;Dermatophagoides pteronyssinus
UdeM00000034425 6565 5670.5 UdeM00000034425 XP_022322144.1 41.4 7220 3597 92 2 6821 215 7198 0.00E+00 5670.5 6565 cellular
organisms;Eukaryota;Opisthokonta;Metazoa;Eumetazoa;Bilateria;Protostomia;

Spiralia;Lophotrochozoa;Mollusca;Bivalvia;Autobranchia;Pteriomorphia;Ostreida;Ostreoidea;Ostreidae;Crassostrea;Crassostrea virginica
UdeM00000034426 6596 220.3 UdeM00000034426 XP_045208827.1 52.4 210 95 2 9 218 9 213 2.50E-54 220.3 6596 cellular organisms;Eukaryota;Opisthokonta;Metazoa;Eumetazoa;Bilateria;Protostomia;Spiralia;Lophotrochozoa;Mollusca;Bivalvia;Autobranchia;Heteroconchia;Euheterodonta;Imparidentia;Venerida;Veneroidea;Veneridae;Mercenaria;Mercenaria mercenaria
UdeM00000034429 6596 96.3 UdeM00000034429 XP_045180326.1 40.3 144 80 2 20 162 32 170 5.10E-17 96.3 6596 cellular organisms;Eukaryota;Opisthokonta;Metazoa;Eumetazoa;Bilateria;Protostomia;Spiralia;Lophotrochozoa;Mollusca;Bivalvia;Autobranchia;Heteroconchia;Euheterodonta;Imparidentia;Venerida;Veneroidea;Veneridae;Mercenaria;Mercenaria mercenaria
UdeM00000034430 6596 96.3 UdeM00000034430 XP_045180326.1 40.3 144 80 2 20 162 32 170 4.50E-17 96.3 6596 cellular organisms;Eukaryota;Opisthokonta;Metazoa;Eumetazoa;Bilateria;Protostomia;Spiralia;Lophotrochozoa;Mollusca;Bivalvia;Autobranchia;Heteroconchia;Euheterodonta;Imparidentia;Venerida;Veneroidea;Veneridae;Mercenaria;Mercenaria mercenaria
UdeM00000034431 32264 141.7 UdeM00000034431 XP_015790859.1 41.1 192 49 1 5 132 119 310 6.60E-31 141.7 32264 cellular organisms;Eukaryota;Opisthokonta;Metazoa;Eumetazoa;Bilateria;Protostomia;Ecdysozoa;Panarthropoda;Arthropoda;Chelicerata;Arachnida;Acari;Acariformes;Trombidiformes;Prostigmata;Eleutherengona;Raphignathae;Tetranychoidea;Tetranychidae;Tetranychus;Tetranychus urticae
UdeM00000034432 88015 65.5 UdeM00000034432 XP_023337768.1 45.5 66 31 1 27 92 277 337 4.20E-08 65.5 88015 cellular organisms;Eukaryota;Opisthokonta;Metazoa;Eumetazoa;Bilateria;Protostomia;Ecdysozoa;Panarthropoda;Arthropoda;Mandibulata;Pancrustacea;Crustacea;Multicrustacea;Hexanauplia;Copepoda;Neocopepoda;Gymnoplea;Calanoida;Temoridae;Eurytemora;Eurytemora affinis
UdeM00000034433 29159 384.8 UdeM00000034433 XP_034331235.1 55.6 342 132 6 25 349 22 360 1.20E-103 384.8 29159 cellular organisms;Eukaryota;Opisthokonta;Metazoa;Eumetazoa;Bilateria;Protostomia;Spiralia;Lophotrochozoa;Mollusca;Bivalvia;Autobranchia;Pteriomorphia;Ostreida;Ostreoidea;Ostreidae;Crassostrea;Crassostrea gigas
UdeM00000034434 225164 334.7 UdeM00000034434 XP_009063479.1 50.8 321 137 6 2 321 77 377 1.30E-88 334.7 225164 cellular organisms;Eukaryota;Opisthokonta;Metazoa;Eumetazoa;Bilateria;Protostomia;Spiralia;Lophotrochozoa;Mollusca;Gastropoda;Patellogastropoda;Lottioidea;Lottiidae;Lottia;Lottia gigantea
UdeM00000034436 6596 509.6 UdeM00000034436 XP_045207945.1 53.5 548 226 8 17 547 64 599 5.10E-141 509.6 6596 cellular organisms;Eukaryota;Opisthokonta;Metazoa;Eumetazoa;Bilateria;Protostomia;Spiralia;Lophotrochozoa;Mollusca;Bivalvia;Autobranchia;Heteroconchia;Euheterodonta;Imparidentia;Venerida;Veneroidea;Veneridae;Mercenaria;Mercenaria mercenaria
UdeM00000034437 6596 442.2 UdeM00000034437 XP_045209937.1 59.5 373 143 6 14 379 15 386 6.70E-121 442.2 6596 cellular organisms;Eukaryota;Opisthokonta;Metazoa;Eumetazoa;Bilateria;Protostomia;Spiralia;Lophotrochozoa;Mollusca;Bivalvia;Autobranchia;Heteroconchia;Euhe

terodonta;Imparidentia;Venerida;Veneroidea;Veneridae;Mercenaria;Mercenaria mercenaria
UdeM00000034438 6596 724.9 UdeM00000034438 XP_045209936.1 74.8 511 121 4 2 507 5 512 6.90E-206 724.9 6596 cellular organisms;Eukaryota;Opisthokonta;Metazoa;Eumetazoa;Bilateria;Protostomia;Spiralia;Lophotrochozoa;Mollusca;Bivalvia;Autobranchia;Heteroconchia;Euheterodonta;Imparidentia;Venerida;Veneroidea;Veneridae;Mercenaria;Mercenaria mercenaria
UdeM00000034439 6596 1246.1 UdeM00000034439 XP_045208750.1 36.4 2970 1319 81 39 2784 100 2723 0.00E+00 1246.1 6596 cellular organisms;Eukaryota;Opisthokonta;Metazoa;Eumetazoa;Bilateria;Protostomia;Spiralia;Lophotrochozoa;Mollusca;Bivalvia;Autobranchia;Heteroconchia;Euheterodonta;Imparidentia;Venerida;Veneroidea;Veneridae;Mercenaria;Mercenaria mercenaria
UdeM00000034440 6596 244.2 UdeM00000034440 XP_045209546.1 83.9 137 22 0 1 137 430 566 9.70E-62 244.2 6596 cellular organisms;Eukaryota;Opisthokonta;Metazoa;Eumetazoa;Bilateria;Protostomia;Spiralia;Lophotrochozoa;Mollusca;Bivalvia;Autobranchia;Heteroconchia;Euheterodonta;Imparidentia;Venerida;Veneroidea;Veneridae;Mercenaria;Mercenaria mercenaria
UdeM00000034441 6596 490.7 UdeM00000034441 XP_045209546.1 71.3 369 99 3 48 415 77 439 1.80E-135 490.7 6596 cellular organisms;Eukaryota;Opisthokonta;Metazoa;Eumetazoa;Bilateria;Protostomia;Spiralia;Lophotrochozoa;Mollusca;Bivalvia;Autobranchia;Heteroconchia;Euheterodonta;Imparidentia;Venerida;Veneroidea;Veneridae;Mercenaria;Mercenaria mercenaria
UdeM00000034442 441366 277.3 UdeM00000034442 XP_028318799.1 60.3 199 79 0 2 200 271 469 1.50E-71 277.3 441366 cellular organisms;Eukaryota;Opisthokonta;Metazoa;Eumetazoa;Bilateria;Deuterostomia;Chordata;Craniata;Vertebrata;Gnathostomata;Teleostomi;Euteleostomi;Actinopterygii;Actinopteri;Neopterygii;Teleostei;Osteoglossocephalai;Clupeocephala;Euteleosteomorpha;Neoteleostei;Eurypterygia;Ctenosquamata;Acanthomorphata;Euacanthomorphacea;Percomorphaceae;Ovalentaria;Blenniimorphae;Blenniiformes;Gobiesocoidei;Gobiesocidae;Gobiesocinae;Gouania;Gouania willdenowi
UdeM00000034443 441366 149.1 UdeM00000034443 XP_028298353.1 61.9 105 40 0 1 105 558 662 3.40E-33 149.1 441366 cellular organisms;Eukaryota;Opisthokonta;Metazoa;Eumetazoa;Bilateria;Deuterostomia;Chordata;Craniata;Vertebrata;Gnathostomata;Teleostomi;Euteleostomi;Actinopterygii;Actinopteri;Neopterygii;Teleostei;Osteoglossocephalai;Clupeocephala;Euteleosteomorpha;Neoteleostei;Eurypterygia;Ctenosquamata;Acanthomorphata;Euacanthomorphacea;Percomorphaceae;Ovalentaria;Blenniimorphae;Blenniiformes;Gobiesocoidei;Gobiesocidae;Gobiesocinae;Gouania;Gouania willdenowi
UdeM00000034444 50429 78.2 UdeM00000034444 XP_022790680.1 48.6 72 36 1 3 74 40 110 1.20E-11 78.2 50429 cellular organisms;Eukaryota;Opisthokonta;Metazoa;Eumetazoa;Cnidaria;Anthozoa;Hexacorallia;Scleractinia;Astrocoeniina;Pocilloporidae;Stylophora;Stylophora pistillata
UdeM00000034445 7654 73.9 UdeM00000034445 XP_041463467.1 41.6 101 55 3 42 139 162 261 2.00E-10 73.9 7654 cellular

organisms;Eukaryota;Opisthokonta;Metazoa;Eumetazoa;Bilateria;Deuterostomia;Echinodermata;Eleutherozoa;Echinozoa;Echinoidea;Euechinoidea;Echinacea;Temnopleuroida;Toxopneustidae;Lytechinus;Lytechinus variegatus
UdeM00000034446 6596 308.9 UdeM00000034446 XP_045215232.1 47.6 347 176 4 61 405 111 453 1.00E-80 308.9 6596 cellular organisms;Eukaryota;Opisthokonta;Metazoa;Eumetazoa;Bilateria;Protostomia;Spiralia;Lophotrochozoa;Mollusca;Bivalvia;Autobranchia;Heteroconchia;Euheterodonta;Imparidentia;Venerida;Veneroidea;Veneridae;Mercenaria;Mercenaria mercenaria
UdeM00000034447 6596 307.4 UdeM00000034447 XP_045215232.1 47.8 345 174 4 61 403 111 451 2.70E-80 307.4 6596 cellular organisms;Eukaryota;Opisthokonta;Metazoa;Eumetazoa;Bilateria;Protostomia;Spiralia;Lophotrochozoa;Mollusca;Bivalvia;Autobranchia;Heteroconchia;Euheterodonta;Imparidentia;Venerida;Veneroidea;Veneridae;Mercenaria;Mercenaria mercenaria
UdeM00000034448 441366 550.4 UdeM00000034448 XP_028318216.1 59.7 402 162 0 8 409 154 555 1.90E-153 550.4 441366 cellular organisms;Eukaryota;Opisthokonta;Metazoa;Eumetazoa;Bilateria;Deuterostomia;Chordata;Craniata;Vertebrata;Gnathostomata;Teleostomi;Euteleostomi;Actinopterygii;Actinopteri;Neopterygii;Teleostei;Osteoglossocephalai;Clupeocephala;Euteleosteomorpha;Neoteleostei;Eurypterygia;Ctenosquamata;Acanthomorphata;Euacanthomorphacea;Percomorphaceae;Ovalentaria;Blenniimorphae;Blenniiformes;Gobiesocoidei;Gobiesocidae;Gobiesocinae;Gouania;Gouania willdenowi
UdeM00000034450 6596 308.9 UdeM00000034450 XP_045215232.1 47.6 347 176 4 61 405 111 453 1.00E-80 308.9 6596 cellular organisms;Eukaryota;Opisthokonta;Metazoa;Eumetazoa;Bilateria;Protostomia;Spiralia;Lophotrochozoa;Mollusca;Bivalvia;Autobranchia;Heteroconchia;Euheterodonta;Imparidentia;Venerida;Veneroidea;Veneridae;Mercenaria;Mercenaria mercenaria
UdeM00000034449 6596 307.4 UdeM00000034449 XP_045215232.1 47.8 345 174 4 61 403 111 451 2.70E-80 307.4 6596 cellular organisms;Eukaryota;Opisthokonta;Metazoa;Eumetazoa;Bilateria;Protostomia;Spiralia;Lophotrochozoa;Mollusca;Bivalvia;Autobranchia;Heteroconchia;Euheterodonta;Imparidentia;Venerida;Veneroidea;Veneridae;Mercenaria;Mercenaria mercenaria
UdeM00000034451 441366 548.9 UdeM00000034451 XP_028318216.1 59.5 402 163 0 8 409 154 555 5.50E-153 548.9 441366 cellular organisms;Eukaryota;Opisthokonta;Metazoa;Eumetazoa;Bilateria;Deuterostomia;Chordata;Craniata;Vertebrata;Gnathostomata;Teleostomi;Euteleostomi;Actinopterygii;Actinopteri;Neopterygii;Teleostei;Osteoglossocephalai;Clupeocephala;Euteleosteomorpha;Neoteleostei;Eurypterygia;Ctenosquamata;Acanthomorphata;Euacanthomorphacea;Percomorphaceae;Ovalentaria;Blenniimorphae;Blenniiformes;Gobiesocoidei;Gobiesocidae;Gobiesocinae;Gouania;Gouania willdenowi
UdeM00000034453 6596 308.9 UdeM00000034453 XP_045215232.1 47.6 347 176 4 61 405 111 453 1.00E-80 308.9 6596 cellular organisms;Eukaryota;Opisthokonta;Metazoa;Eumetazoa;Bilateria;Protostomia;Spiralia;Lophotrochozoa;Mollusca;Bivalvia;Autobranchia;Heteroconchia;Euheterodonta;Imparidentia;Venerida;Veneroidea;Veneridae;Mercenaria;Mercenaria mercenaria

UdeM00000034452 6596 307.4 UdeM00000034452 XP_045215232.1 47.8 345
174 4 61 403 111 451 2.70E-80 307.4 6596 cellular
organisms;Eukaryota;Opisthokonta;Metazoa;Eumetazoa;Bilateria;Protostomia;Spiralia;Lophotrochozoa;Mollusca;Bivalvia;Autobranchia;Heteroconchia;Euheterodonta;Imparidentia;Venerida;Veneroidea;Veneridae;Mercenaria;Mercenaria mercenaria
UdeM00000034454 50390 393.7 UdeM00000034454 XP_028142380.1 52.3 300
143 0 2 301 49 348 2.20E-106 393.7 50390 cellular
organisms;Eukaryota;Opisthokonta;Metazoa;Eumetazoa;Bilateria;Protostomia;Ecdysozoa;Panarthropoda;Arthropoda;Mandibulata;Pancrustacea;Hexapoda;Insecta;Dicondylia;Pterygota;Neoptera;Endopterygota;Coleoptera;Polyphaga;Cucujiformia;Chrysomeloidea;Chrysomelidae;Galerucinae;Luperini;Diabroticina;Diabroticites;Diabrotica;Diabrotica virgifera;Diabrotica virgifera virgifera
UdeM00000034455 50429 77.4 UdeM00000034455 XP_022790680.1 47.2 72
37 1 3 74 40 110 2.10E-11 77.4 50429 cellular
organisms;Eukaryota;Opisthokonta;Metazoa;Eumetazoa;Cnidaria;Anthozoa;Hexacorallia;Scleractinia;Astrocoeniina;Pocilloporidae;Stylophora;Stylophora pistillata
UdeM00000034456 7741 186.4 UdeM00000034456 XP_019626645.1 44.9 236
69 3 1 178 96 328 3.20E-44 186.4 7741 cellular
organisms;Eukaryota;Opisthokonta;Metazoa;Eumetazoa;Bilateria;Deuterostomia;Chordata;Cephalochordata;Leptocardii;Amphioxiformes;Branchiostomidae;Branchiostoma;Branchiostoma belcheri
UdeM00000034457 52239 147.5 UdeM00000034457 XP_033947251.1 58.1 105
44 0 2 106 124 228 9.70E-33 147.5 52239 cellular
organisms;Eukaryota;Opisthokonta;Metazoa;Eumetazoa;Bilateria;Deuterostomia;Chordata;Craniata;Vertebrata;Gnathostomata;Teleostomi;Euteleostomi;Actinopterygii;Actinopteri;Neopterygii;Teleostei;Osteoglossocephalai;Clupeocephala;Euteleosteomorpha;Neoteleostei;Eurypterygia;Ctenosquamata;Acanthomorphata;Euacanthomorphacea;Percomorphaceae;Eupercaria;Perciformes;Notothenioidei;Channichthyidae;Pseudochaenichthys;Pseudochaenichthys georgianus
UdeM00000034458 400682 73.6 UdeM00000034458 XP_019861952.1 38.2
89 53 2 3 90 336 423 1.90E-10 73.6 400682
cellular
organisms;Eukaryota;Opisthokonta;Metazoa;Porifera;Demospongiae;Heteroscleromorpha;Haplosclerida;Niphatidae;Amphimedon;Amphimedon queenslandica
UdeM00000034461 7739 129 UdeM00000034461 XP_035659254.1 39.3 168
96 2 1 162 765 932 6.40E-27 129 7739 cellular
organisms;Eukaryota;Opisthokonta;Metazoa;Eumetazoa;Bilateria;Deuterostomia;Chordata;Cephalochordata;Leptocardii;Amphioxiformes;Branchiostomidae;Branchiostoma;Branchiostoma floridae
UdeM00000034462 7574 73.2 UdeM00000034462 XP_013400898.1 38.9 90
53 1 100 189 212 299 4.10E-10 73.2 7574 cellular
organisms;Eukaryota;Opisthokonta;Metazoa;Eumetazoa;Bilateria;Protostomia;Spiralia;Lophotrochozoa;Brachiopoda;Linguliformea;Lingulata;Lingulida;Linguloidea;Lingulidae;Lingula;Lingula anatina
UdeM00000034463 441366 226.9 UdeM00000034463 XP_028318792.1 63.5
159 56 1 2 160 612 768 1.90E-56 226.9 441366
cellular
organisms;Eukaryota;Opisthokonta;Metazoa;Eumetazoa;Bilateria;Deuterostomia;Chordata;Craniata;Vertebrata;Gnathostomata;Teleostomi;Euteleostomi;Actinopterygii;Actinopteri;Neopterygii;Teleostei;Osteoglossocephalai;Clupeocephala;Euteleosteomorpha;Neoteleostei;Eurypterygia;Ctenosquamata;Acanthomo

rphata;Euacanthomorphacea;Percomorphaceae;Ovalentaria;Blenniimorphae;Blenniiformes;Gobiesocoidei;Gobiesocidae;Gobiesocinae;Gouania;Gouania willdenowi
UdeM00000034465 6565 91.3 UdeM00000034465 XP_022307899.1 35.1 168 87 5 17 183 11 157 1.60E-15 91.3 6565 cellular organisms;Eukaryota;Opisthokonta;Metazoa;Eumetazoa;Bilateria;Protostomia;Spiralia;Lophotrochozoa;Mollusca;Bivalvia;Autobranchia;Pteriomorphia;Ostreida;Ostreoidea;Ostreidae;Crassostrea;Crassostrea virginica
UdeM00000034464 6565 91.3 UdeM00000034464 XP_022307899.1 35.1 168 87 5 133 299 11 157 2.50E-15 91.3 6565 cellular organisms;Eukaryota;Opisthokonta;Metazoa;Eumetazoa;Bilateria;Protostomia;Spiralia;Lophotrochozoa;Mollusca;Bivalvia;Autobranchia;Pteriomorphia;Ostreida;Ostreoidea;Ostreidae;Crassostrea;Crassostrea virginica
UdeM00000034466 6596 345.1 UdeM00000034466 XP_045177776.1 38.4 524 261 8 9 514 398 877 1.50E-91 345.1 6596 cellular organisms;Eukaryota;Opisthokonta;Metazoa;Eumetazoa;Bilateria;Protostomia;Spiralia;Lophotrochozoa;Mollusca;Bivalvia;Autobranchia;Heteroconchia;Euheterodonta;Imparidentia;Venerida;Veneroidea;Veneridae;Mercenaria;Mercenaria mercenaria
UdeM00000034467 50390 330.9 UdeM00000034467 XP_028152422.1 54.9 255 114 1 2 256 188 441 1.50E-87 330.9 50390 cellular organisms;Eukaryota;Opisthokonta;Metazoa;Eumetazoa;Bilateria;Protostomia;Ecdysozoa;Panarthropoda;Arthropoda;Mandibulata;Pancrustacea;Hexapoda;Insecta;Dicondylia;Pterygota;Neoptera;Endopterygota;Coleoptera;Polyphaga;Cucujiformia;Chrysomeloidea;Chrysomelidae;Galerucinae;Luperini;Diabroticina;Diabroticites;Diabrotica;Diabrotica virgifera;Diabrotica virgifera virgifera
UdeM00000034468 6565 419.9 UdeM00000034468 XP_022309566.1 54.8 301 136 0 2 302 717 1017 2.80E-114 419.9 6565 cellular organisms;Eukaryota;Opisthokonta;Metazoa;Eumetazoa;Bilateria;Protostomia;Spiralia;Lophotrochozoa;Mollusca;Bivalvia;Autobranchia;Pteriomorphia;Ostreida;Ostreoidea;Ostreidae;Crassostrea;Crassostrea virginica
UdeM00000034469 50390 552 UdeM00000034469 XP_028154228.1 51.8 452 217 1 4 454 158 609 7.20E-154 552 50390 cellular organisms;Eukaryota;Opisthokonta;Metazoa;Eumetazoa;Bilateria;Protostomia;Ecdysozoa;Panarthropoda;Arthropoda;Mandibulata;Pancrustacea;Hexapoda;Insecta;Dicondylia;Pterygota;Neoptera;Endopterygota;Coleoptera;Polyphaga;Cucujiformia;Chrysomeloidea;Chrysomelidae;Galerucinae;Luperini;Diabroticina;Diabroticites;Diabrotica;Diabrotica virgifera;Diabrotica virgifera virgifera
UdeM00000034470 29159 309.3 UdeM00000034470 XP_011419884.2 71.4 203 58 0 1 203 1 203 3.70E-81 309.3 29159 cellular organisms;Eukaryota;Opisthokonta;Metazoa;Eumetazoa;Bilateria;Protostomia;Spiralia;Lophotrochozoa;Mollusca;Bivalvia;Autobranchia;Pteriomorphia;Ostreida;Ostreoidea;Ostreidae;Crassostrea;Crassostrea gigas
UdeM00000034472 6573 160.6 UdeM00000034472 XP_021358714.1 58.3 139 54 2 1 139 1 135 1.90E-36 160.6 6573 cellular organisms;Eukaryota;Opisthokonta;Metazoa;Eumetazoa;Bilateria;Protostomia;Spiralia;Lophotrochozoa;Mollusca;Bivalvia;Autobranchia;Pteriomorphia;Pectinida;Pectinoidea;Pectinidae;Mizuhopecten;Mizuhopecten yessoensis
UdeM00000034473 29159 69.7 UdeM00000034473 XP_011435817.2 56.1 57 25 0 7 63 146 202 5.90E-09 69.7 29159 cellular organisms;Eukaryota;Opisthokonta;Metazoa;Eumetazoa;Bilateria;Protostomia;

Spiralia;Lophotrochozoa;Mollusca;Bivalvia;Autobranchia;Pteriomorphia;Ostreida;Ostreoidea;Ostreidae;Crassostrea;Crassostrea gigas
UdeM00000034474 6596 786.2 UdeM00000034474 XP_045183696.1 78.6 509 104 3 1 507 2738 3243 2.50E-224 786.2 6596 cellular organisms;Eukaryota;Opisthokonta;Metazoa;Eumetazoa;Bilateria;Protostomia;Spiralia;Lophotrochozoa;Mollusca;Bivalvia;Autobranchia;Heteroconchia;Euheterodonta;Imparidentia;Venerida;Veneroidea;Veneridae;Mercenaria;Mercenaria mercenaria
UdeM00000034475 6596 564.7 UdeM00000034475 XP_045183696.1 68.3 398 123 3 14 410 2327 2722 9.90E-158 564.7 6596 cellular organisms;Eukaryota;Opisthokonta;Metazoa;Eumetazoa;Bilateria;Protostomia;Spiralia;Lophotrochozoa;Mollusca;Bivalvia;Autobranchia;Heteroconchia;Euheterodonta;Imparidentia;Venerida;Veneroidea;Veneridae;Mercenaria;Mercenaria mercenaria
UdeM00000034476 6596 1375.5 UdeM00000034476 XP_045183696.1 53 1396 585 20 13 1391 945 2286 0.00E+00 1375.5 6596 cellular organisms;Eukaryota;Opisthokonta;Metazoa;Eumetazoa;Bilateria;Protostomia;Spiralia;Lophotrochozoa;Mollusca;Bivalvia;Autobranchia;Heteroconchia;Euheterodonta;Imparidentia;Venerida;Veneroidea;Veneridae;Mercenaria;Mercenaria mercenaria
UdeM00000034477 6596 875.9 UdeM00000034477 XP_045183696.1 68.4 712 207 6 39 736 65 772 6.00E-251 875.9 6596 cellular organisms;Eukaryota;Opisthokonta;Metazoa;Eumetazoa;Bilateria;Protostomia;Spiralia;Lophotrochozoa;Mollusca;Bivalvia;Autobranchia;Heteroconchia;Euheterodonta;Imparidentia;Venerida;Veneroidea;Veneridae;Mercenaria;Mercenaria mercenaria
UdeM00000034478 6565 89.7 UdeM00000034478 XP_022327962.1 47.3 93 47 1 3 93 626 718 2.10E-15 89.7 6565 cellular organisms;Eukaryota;Opisthokonta;Metazoa;Eumetazoa;Bilateria;Protostomia;Spiralia;Lophotrochozoa;Mollusca;Bivalvia;Autobranchia;Pteriomorphia;Ostreida;Ostreoidea;Ostreidae;Crassostrea;Crassostrea virginica
UdeM00000034479 6565 231.9 UdeM00000034479 XP_022327962.1 52.4 227 106 2 73 297 361 587 1.10E-57 231.9 6565 cellular organisms;Eukaryota;Opisthokonta;Metazoa;Eumetazoa;Bilateria;Protostomia;Spiralia;Lophotrochozoa;Mollusca;Bivalvia;Autobranchia;Pteriomorphia;Ostreida;Ostreoidea;Ostreidae;Crassostrea;Crassostrea virginica
UdeM00000034480 160734 96.7 UdeM00000034480 XP_042370962.1 50 78 38 1 429 505 177 254 9.20E-17 96.7 160734 cellular organisms;Eukaryota;Opisthokonta;Metazoa;Eumetazoa;Bilateria;Deuterostomia;Chordata;Craniata;Vertebrata;Gnathostomata;Teleostomi;Euteleostomi;Actinopterygii;Actinopteri;Neopterygii;Teleostei;Osteoglossocephalai;Clupeocephala;Euteleosteomorpha;Neoteleostei;Eurypterygia;Ctenosquamata;Acanthomorphata;Euacanthomorphacea;Percomorphaceae;Eupercaria;Perciformes;Serranoidei;Serranidae;Epinephelinae;Epinephelini;Plectropomus;Plectropomus leopardus
UdeM00000034481 7054 101.3 UdeM00000034481 XP_031332239.1 42.2 102 59 0 597 698 464 565 5.20E-18 101.3 7054 cellular organisms;Eukaryota;Opisthokonta;Metazoa;Eumetazoa;Bilateria;Protostomia;Ecdysozoa;Panarthropoda;Arthropoda;Mandibulata;Pancrustacea;Hexapoda;Insecta;Dicondylia;Pterygota;Neoptera;Endopterygota;Coleoptera;Polyphaga;Elateriformia;Elateroidea;Lampyridae;Lampyrinae;Photinus;Photinus pyralis

UdeM00000034482 45351 110.5 UdeM00000034482 XP_032232593.1 53.6 84
39 0 493 576 441 524 7.80E-21 110.5 45351 cellular
organisms;Eukaryota;Opisthokonta;Metazoa;Eumetazoa;Cnidaria;Anthozoa;Hexa
corallia;Actiniaria;Edwardsiidae;Nematostella;Nematostella vectensis
UdeM00000034483 37653 122.5 UdeM00000034483 XP_014769226.1 40.6 143
77 3 648 785 333 472 2.40E-24 122.5 37653 cellular
organisms;Eukaryota;Opisthokonta;Metazoa;Eumetazoa;Bilateria;Protostomia;
Spiralia;Lophotrochozoa;Mollusca;Cephalopoda;Coleoidea;Octopodiformes;Oct
opoda;Incirrata;Octopodidae;Octopus;Octopus bimaculoides
UdeM00000034484 6596 317 UdeM00000034484 XP_045216733.1 50.3 288
141 2 47 334 227 512 2.90E-83 317 6596 cellular
organisms;Eukaryota;Opisthokonta;Metazoa;Eumetazoa;Bilateria;Protostomia;
Spiralia;Lophotrochozoa;Mollusca;Bivalvia;Autobranchia;Heteroconchia;Euhe
terodonta;Imparidentia;Venerida;Veneroidea;Veneridae;Mercenaria;Mercenari
a mercenaria
UdeM00000034485 6596 275 UdeM00000034485 XP_045216732.1 50 272
126 4 24 295 201 462 1.10E-70 275 6596 cellular
organisms;Eukaryota;Opisthokonta;Metazoa;Eumetazoa;Bilateria;Protostomia;
Spiralia;Lophotrochozoa;Mollusca;Bivalvia;Autobranchia;Heteroconchia;Euhe
terodonta;Imparidentia;Venerida;Veneroidea;Veneridae;Mercenaria;Mercenari
a mercenaria
UdeM00000034486 6596 274.6 UdeM00000034486 XP_045216732.1 49.8 271
126 4 24 294 201 461 1.80E-70 274.6 6596 cellular
organisms;Eukaryota;Opisthokonta;Metazoa;Eumetazoa;Bilateria;Protostomia;
Spiralia;Lophotrochozoa;Mollusca;Bivalvia;Autobranchia;Heteroconchia;Euhe
terodonta;Imparidentia;Venerida;Veneroidea;Veneridae;Mercenaria;Mercenari
a mercenaria
UdeM00000034487 29159 177.6 UdeM00000034487 XP_034314481.1 48.9 184
93 1 173 356 224 406 3.00E-41 177.6 29159 cellular
organisms;Eukaryota;Opisthokonta;Metazoa;Eumetazoa;Bilateria;Protostomia;
Spiralia;Lophotrochozoa;Mollusca;Bivalvia;Autobranchia;Pteriomorphia;Ostr
eida;Ostreoidea;Ostreidae;Crassostrea;Crassostrea gigas
UdeM00000034489 29159 352.8 UdeM00000034489 XP_034318412.1 52.6 344
161 2 39 381 11 353 5.50E-94 352.8 29159 cellular
organisms;Eukaryota;Opisthokonta;Metazoa;Eumetazoa;Bilateria;Protostomia;
Spiralia;Lophotrochozoa;Mollusca;Bivalvia;Autobranchia;Pteriomorphia;Ostr
eida;Ostreoidea;Ostreidae;Crassostrea;Crassostrea gigas
UdeM00000034490 6596 244.6 UdeM00000034490 XP_045206286.1 47.9 238
85 1 1 199 663 900 1.20E-61 244.6 6596 cellular
organisms;Eukaryota;Opisthokonta;Metazoa;Eumetazoa;Bilateria;Protostomia;
Spiralia;Lophotrochozoa;Mollusca;Bivalvia;Autobranchia;Heteroconchia;Euhe
terodonta;Imparidentia;Venerida;Veneroidea;Veneridae;Mercenaria;Mercenari
a mercenaria
UdeM00000034493 29159 154.8 UdeM00000034493 XP_034327176.1 33.8 328
135 11 52 369 407 662 2.30E-34 154.8 29159 cellular
organisms;Eukaryota;Opisthokonta;Metazoa;Eumetazoa;Bilateria;Protostomia;
Spiralia;Lophotrochozoa;Mollusca;Bivalvia;Autobranchia;Pteriomorphia;Ostr
eida;Ostreoidea;Ostreidae;Crassostrea;Crassostrea gigas
UdeM00000034492 6596 472.2 UdeM00000034492 XP_045204155.1 38.6 721
353 13 43 741 592 1244 1.20E-129 472.2 6596 cellular
organisms;Eukaryota;Opisthokonta;Metazoa;Eumetazoa;Bilateria;Protostomia;
Spiralia;Lophotrochozoa;Mollusca;Bivalvia;Autobranchia;Heteroconchia;Euhe
terodonta;Imparidentia;Venerida;Veneroidea;Veneridae;Mercenaria;Mercenari
a mercenaria

UdeM00000034494 29159 235 UdeM00000034494 XP_034327176.1 36.8 402
170 13 22 407 407 740 1.90E-58 235 29159 cellular
organisms;Eukaryota;Opisthokonta;Metazoa;Eumetazoa;Bilateria;Protostomia;
Spiralia;Lophotrochozoa;Mollusca;Bivalvia;Autobranchia;Pteriomorphia;Ostr
eida;Ostreoidea;Ostreidae;Crassostrea;Crassostrea gigas
UdeM00000034495 6596 317 UdeM00000034495 XP_045206286.1 58.2 244
101 1 1 244 663 905 2.30E-83 317 6596 cellular
organisms;Eukaryota;Opisthokonta;Metazoa;Eumetazoa;Bilateria;Protostomia;
Spiralia;Lophotrochozoa;Mollusca;Bivalvia;Autobranchia;Heteroconchia;Euhe
terodonta;Imparidentia;Venerida;Veneroidea;Veneridae;Mercenaria;Mercenari
a mercenaria
UdeM00000034496 6596 316.6 UdeM00000034496 XP_045206286.1 59.4 239
96 1 1 239 663 900 3.00E-83 316.6 6596 cellular
organisms;Eukaryota;Opisthokonta;Metazoa;Eumetazoa;Bilateria;Protostomia;
Spiralia;Lophotrochozoa;Mollusca;Bivalvia;Autobranchia;Heteroconchia;Euhe
terodonta;Imparidentia;Venerida;Veneroidea;Veneridae;Mercenaria;Mercenari
a mercenaria
UdeM00000034497 6565 216.5 UdeM00000034497 XP_022324984.1 47.6 212
107 3 5 214 65 274 3.80E-53 216.5 6565 cellular
organisms;Eukaryota;Opisthokonta;Metazoa;Eumetazoa;Bilateria;Protostomia;
Spiralia;Lophotrochozoa;Mollusca;Bivalvia;Autobranchia;Pteriomorphia;Ostr
eida;Ostreoidea;Ostreidae;Crassostrea;Crassostrea virginica
UdeM00000044433 224308 105.9 UdeM00000044433 NP_390979.1 32.5 191
118 4 4 184 27 216 1.10E-19 105.9 224308
cellular organisms;Bacteria;Terrabacteria
group;Firmicutes;Bacilli;Bacillales;Bacillaceae;Bacillus;Bacillus
subtilis group;Bacillus subtilis;Bacillus subtilis subsp.
subtilis;Bacillus subtilis subsp. subtilis str. 168
UdeM00000043808 6565 102.1 UdeM00000043808 XP_022325431.1 28.1 217
132 8 17 227 1134 1332 1.70E-18 102.1 6565 cellular
organisms;Eukaryota;Opisthokonta;Metazoa;Eumetazoa;Bilateria;Protostomia;
Spiralia;Lophotrochozoa;Mollusca;Bivalvia;Autobranchia;Pteriomorphia;Ostr
eida;Ostreoidea;Ostreidae;Crassostrea;Crassostrea virginica
UdeM00000043809 6579 116.7 UdeM00000043809 XP_033744257.1 41.4 140
82 0 1 140 154 293 2.60E-23 116.7 6579 cellular
organisms;Eukaryota;Opisthokonta;Metazoa;Eumetazoa;Bilateria;Protostomia;
Spiralia;Lophotrochozoa;Mollusca;Bivalvia;Autobranchia;Pteriomorphia;Pect
inida;Pectinoidea;Pectinidae;Pecten;Pecten maximus
UdeM00000043812 6565 85.5 UdeM00000043812 XP_022288855.1 39.5 119
62 5 12 129 979 1088 1.20E-13 85.5 6565 cellular
organisms;Eukaryota;Opisthokonta;Metazoa;Eumetazoa;Bilateria;Protostomia;
Spiralia;Lophotrochozoa;Mollusca;Bivalvia;Autobranchia;Pteriomorphia;Ostr
eida;Ostreoidea;Ostreidae;Crassostrea;Crassostrea virginica
UdeM00000044443 7574 352.4 UdeM00000044443 XP_013399327.1 92.7 193
14 0 1 193 2 194 3.60E-94 352.4 7574 cellular
organisms;Eukaryota;Opisthokonta;Metazoa;Eumetazoa;Bilateria;Protostomia;
Spiralia;Lophotrochozoa;Brachiopoda;Linguliformea;Lingulata;Lingulida;Lin
guloidea;Lingulidae;Lingula;Lingula anatina
UdeM00000038944 6596 285.8 UdeM00000038944 XP_045213047.1 29.3 607
392 20 11 593 1168 1761 3.00E-73 285.8 6596 cellular
organisms;Eukaryota;Opisthokonta;Metazoa;Eumetazoa;Bilateria;Protostomia;
Spiralia;Lophotrochozoa;Mollusca;Bivalvia;Autobranchia;Heteroconchia;Euhe
terodonta;Imparidentia;Venerida;Veneroidea;Veneridae;Mercenaria;Mercenari
a mercenaria

UdeM00000038945 400727 391.7 UdeM00000038945 XP_025103830.1 43.4
431 233 4 41 464 23 449 1.30E-105 391.7 400727
cellular
organisms;Eukaryota;Opisthokonta;Metazoa;Eumetazoa;Bilateria;Protostomia;Spiralia;Lophotrochozoa;Mollusca;Gastropoda;Caenogastropoda;Architaenioglossa;Ampullarioidea;Ampullariidae;Pomacea;Pomacea canaliculata
UdeM00000038946 400727 387.1 UdeM00000038946 XP_025103830.1 43
449 245 4 1 442 1 445 3.00E-104 387.1 400727
cellular
organisms;Eukaryota;Opisthokonta;Metazoa;Eumetazoa;Bilateria;Protostomia;Spiralia;Lophotrochozoa;Mollusca;Gastropoda;Caenogastropoda;Architaenioglossa;Ampullarioidea;Ampullariidae;Pomacea;Pomacea canaliculata
UdeM00000038947 400727 387.9 UdeM00000038947 XP_025103830.1 45.3
428 220 7 41 458 23 446 1.80E-104 387.9 400727
cellular
organisms;Eukaryota;Opisthokonta;Metazoa;Eumetazoa;Bilateria;Protostomia;Spiralia;Lophotrochozoa;Mollusca;Gastropoda;Caenogastropoda;Architaenioglossa;Ampullarioidea;Ampullariidae;Pomacea;Pomacea canaliculata
UdeM00000038948 400727 391 UdeM00000038948 XP_025103830.1 45.3
428 221 6 41 459 23 446 2.20E-105 391 400727
cellular
organisms;Eukaryota;Opisthokonta;Metazoa;Eumetazoa;Bilateria;Protostomia;Spiralia;Lophotrochozoa;Mollusca;Gastropoda;Caenogastropoda;Architaenioglossa;Ampullarioidea;Ampullariidae;Pomacea;Pomacea canaliculata
UdeM00000038949 6596 1001.1 UdeM00000038949 XP_045187389.1 73.4
627 155 5 1 621 1 621 6.10E-289 1001.1 6596
cellular
organisms;Eukaryota;Opisthokonta;Metazoa;Eumetazoa;Bilateria;Protostomia;Spiralia;Lophotrochozoa;Mollusca;Bivalvia;Autobranchia;Heteroconchia;Euheterodonta;Imparidentia;Venerida;Veneroidea;Veneridae;Mercenaria;Mercenaria mercenaria
UdeM00000038950 400727 75.9 UdeM00000038950 XP_025104291.1 40
75 45 0 30 104 2 76 3.90E-11 75.9 400727
cellular
organisms;Eukaryota;Opisthokonta;Metazoa;Eumetazoa;Bilateria;Protostomia;Spiralia;Lophotrochozoa;Mollusca;Gastropoda;Caenogastropoda;Architaenioglossa;Ampullarioidea;Ampullariidae;Pomacea;Pomacea canaliculata
UdeM00000038951 225164 501.5 UdeM00000038951 XP_009055041.1 62.1
396 143 4 1 391 1 394 9.60E-139 501.5 225164
cellular
organisms;Eukaryota;Opisthokonta;Metazoa;Eumetazoa;Bilateria;Protostomia;Spiralia;Lophotrochozoa;Mollusca;Gastropoda;Patellogastropoda;Lottioidea;Lottiidae;Lottia;Lottia gigantea
UdeM00000038952 6573 848.2 UdeM00000038952 XP_021378004.1 41.3 2427
1163 35 5 2329 28 2295 2.50E-242 848.2 6573 cellular
organisms;Eukaryota;Opisthokonta;Metazoa;Eumetazoa;Bilateria;Protostomia;Spiralia;Lophotrochozoa;Mollusca;Bivalvia;Autobranchia;Pteriomorphia;Pectinida;Pectinoidea;Pectinidae;Mizuhopecten;Mizuhopecten yessoensis
UdeM00000038954 6596 699.5 UdeM00000038954 XP_045164128.1 67.3 514
90 4 1 509 1 441 3.10E-198 699.5 6596 cellular
organisms;Eukaryota;Opisthokonta;Metazoa;Eumetazoa;Bilateria;Protostomia;Spiralia;Lophotrochozoa;Mollusca;Bivalvia;Autobranchia;Heteroconchia;Euheterodonta;Imparidentia;Venerida;Veneroidea;Veneridae;Mercenaria;Mercenaria mercenaria

UdeM00000038955 6596 245 UdeM00000038955 XP_045168446.1 62 192
54 3 26 215 18 192 9.40E-62 245 6596 cellular
organisms;Eukaryota;Opisthokonta;Metazoa;Eumetazoa;Bilateria;Protostomia;
Spiralia;Lophotrochozoa;Mollusca;Bivalvia;Autobranchia;Heteroconchia;Euhe
terodonta;Imparidentia;Venerida;Veneroidea;Veneridae;Mercenaria;Mercenari
a mercenaria
UdeM00000038956 6596 309.7 UdeM00000038956 XP_045168360.1 32.1 676
366 15 1 660 1 599 9.50E-81 309.7 6596 cellular
organisms;Eukaryota;Opisthokonta;Metazoa;Eumetazoa;Bilateria;Protostomia;
Spiralia;Lophotrochozoa;Mollusca;Bivalvia;Autobranchia;Heteroconchia;Euhe
terodonta;Imparidentia;Venerida;Veneroidea;Veneridae;Mercenaria;Mercenari
a mercenaria
UdeM00000038957 6596 503.4 UdeM00000038957 XP_045168379.1 45.2 639
258 11 5 568 599 1220 3.70E-139 503.4 6596 cellular
organisms;Eukaryota;Opisthokonta;Metazoa;Eumetazoa;Bilateria;Protostomia;
Spiralia;Lophotrochozoa;Mollusca;Bivalvia;Autobranchia;Heteroconchia;Euhe
terodonta;Imparidentia;Venerida;Veneroidea;Veneridae;Mercenaria;Mercenari
a mercenaria
UdeM00000038958 6579 387.5 UdeM00000038958 XP_033734740.1 51.5 412
182 7 1 400 11 416 2.10E-104 387.5 6579 cellular
organisms;Eukaryota;Opisthokonta;Metazoa;Eumetazoa;Bilateria;Protostomia;
Spiralia;Lophotrochozoa;Mollusca;Bivalvia;Autobranchia;Pteriomorphia;Pect
inida;Pectinoidea;Pectinidae;Pecten;Pecten maximus
UdeM00000038960 6596 424.1 UdeM00000038960 XP_045214148.1 59.8 343
134 3 4 345 565 904 1.70E-115 424.1 6596 cellular
organisms;Eukaryota;Opisthokonta;Metazoa;Eumetazoa;Bilateria;Protostomia;
Spiralia;Lophotrochozoa;Mollusca;Bivalvia;Autobranchia;Heteroconchia;Euhe
terodonta;Imparidentia;Venerida;Veneroidea;Veneridae;Mercenaria;Mercenari
a mercenaria
UdeM00000038961 6596 326.6 UdeM00000038961 XP_045214148.1 42.6 350
177 3 8 335 145 492 3.80E-86 326.6 6596 cellular
organisms;Eukaryota;Opisthokonta;Metazoa;Eumetazoa;Bilateria;Protostomia;
Spiralia;Lophotrochozoa;Mollusca;Bivalvia;Autobranchia;Heteroconchia;Euhe
terodonta;Imparidentia;Venerida;Veneroidea;Veneridae;Mercenaria;Mercenari
a mercenaria
UdeM00000038962 6596 71.2 UdeM00000038962 XP_045214148.1 38.7 106
51 4 24 117 40 143 1.30E-09 71.2 6596 cellular
organisms;Eukaryota;Opisthokonta;Metazoa;Eumetazoa;Bilateria;Protostomia;
Spiralia;Lophotrochozoa;Mollusca;Bivalvia;Autobranchia;Heteroconchia;Euhe
terodonta;Imparidentia;Venerida;Veneroidea;Veneridae;Mercenaria;Mercenari
a mercenaria
UdeM00000038963 1735272 156.4 UdeM00000038963 XP_041354039.1 55.4
148 59 2 2 144 3 148 2.80E-35 156.4 1735272
cellular
organisms;Eukaryota;Opisthokonta;Metazoa;Eumetazoa;Bilateria;Protostomia;
Spiralia;Lophotrochozoa;Mollusca;Gastropoda;Neomphalina;Neomphaloidea;Pel
tospiridae;Gigantopelta;Gigantopelta aegis
UdeM00000038964 6573 235.7 UdeM00000038964 XP_021349576.1 45.3 413
178 10 5 388 1 394 9.80E-59 235.7 6573 cellular
organisms;Eukaryota;Opisthokonta;Metazoa;Eumetazoa;Bilateria;Protostomia;
Spiralia;Lophotrochozoa;Mollusca;Bivalvia;Autobranchia;Pteriomorphia;Pect
inida;Pectinoidea;Pectinidae;Mizuhopecten;Mizuhopecten yessoensis
UdeM00000038965 6573 236.9 UdeM00000038965 XP_021349576.1 45.8 413
175 11 5 387 1 394 4.40E-59 236.9 6573 cellular

organisms;Eukaryota;Opisthokonta;Metazoa;Eumetazoa;Bilateria;Protostomia;Spiralia;Lophotrochozoa;Mollusca;Bivalvia;Autobranchia;Pteriomorphia;Pectinida;Pectinoidea;Pectinidae;Mizuhopecten;Mizuhopecten yessoensis
UdeM00000038966 1735272 91.7 UdeM00000038966 XP_041354510.1 38.5 104 64 0 7 110 11 114 8.00E-16 91.7 1735272 cellular organisms;Eukaryota;Opisthokonta;Metazoa;Eumetazoa;Bilateria;Protostomia;Spiralia;Lophotrochozoa;Mollusca;Gastropoda;Neomphalina;Neomphaloidea;Peltospiridae;Gigantopelta;Gigantopelta aegis
UdeM00000038967 6596 487.6 UdeM00000038967 XP_045168176.1 64.4 376 128 2 1 372 1 374 1.50E-134 487.6 6596 cellular organisms;Eukaryota;Opisthokonta;Metazoa;Eumetazoa;Bilateria;Protostomia;Spiralia;Lophotrochozoa;Mollusca;Bivalvia;Autobranchia;Heteroconchia;Euheterodonta;Imparidentia;Venerida;Veneroidea;Veneridae;Mercenaria;Mercenaria mercenaria
UdeM00000038969 6596 140.2 UdeM00000038969 XP_045165354.1 54.5 123 55 1 2 124 3 124 1.80E-30 140.2 6596 cellular organisms;Eukaryota;Opisthokonta;Metazoa;Eumetazoa;Bilateria;Protostomia;Spiralia;Lophotrochozoa;Mollusca;Bivalvia;Autobranchia;Heteroconchia;Euheterodonta;Imparidentia;Venerida;Veneroidea;Veneridae;Mercenaria;Mercenaria mercenaria
UdeM00000038970 6596 548.1 UdeM00000038970 XP_045165354.1 51.7 551 257 5 1 547 127 672 1.20E-152 548.1 6596 cellular organisms;Eukaryota;Opisthokonta;Metazoa;Eumetazoa;Bilateria;Protostomia;Spiralia;Lophotrochozoa;Mollusca;Bivalvia;Autobranchia;Heteroconchia;Euheterodonta;Imparidentia;Venerida;Veneroidea;Veneridae;Mercenaria;Mercenaria mercenaria
UdeM00000038971 6596 1025.8 UdeM00000038971 XP_045165343.1 56.1 906 380 9 1 899 148 1042 3.40E-296 1025.8 6596 cellular organisms;Eukaryota;Opisthokonta;Metazoa;Eumetazoa;Bilateria;Protostomia;Spiralia;Lophotrochozoa;Mollusca;Bivalvia;Autobranchia;Heteroconchia;Euheterodonta;Imparidentia;Venerida;Veneroidea;Veneridae;Mercenaria;Mercenaria mercenaria
UdeM00000038972 6596 103.2 UdeM00000038972 XP_045165343.1 72.7 66 18 0 1 66 1 66 2.90E-19 103.2 6596 cellular organisms;Eukaryota;Opisthokonta;Metazoa;Eumetazoa;Bilateria;Protostomia;Spiralia;Lophotrochozoa;Mollusca;Bivalvia;Autobranchia;Heteroconchia;Euheterodonta;Imparidentia;Venerida;Veneroidea;Veneridae;Mercenaria;Mercenaria mercenaria
UdeM00000038973 6596 300.8 UdeM00000038973 XP_045187985.1 42.8 339 193 1 41 379 3 340 2.40E-78 300.8 6596 cellular organisms;Eukaryota;Opisthokonta;Metazoa;Eumetazoa;Bilateria;Protostomia;Spiralia;Lophotrochozoa;Mollusca;Bivalvia;Autobranchia;Heteroconchia;Euheterodonta;Imparidentia;Venerida;Veneroidea;Veneridae;Mercenaria;Mercenaria mercenaria
UdeM00000038974 6596 132.1 UdeM00000038974 XP_045187703.1 24.1 506 256 13 13 506 57 446 2.20E-27 132.1 6596 cellular organisms;Eukaryota;Opisthokonta;Metazoa;Eumetazoa;Bilateria;Protostomia;Spiralia;Lophotrochozoa;Mollusca;Bivalvia;Autobranchia;Heteroconchia;Euheterodonta;Imparidentia;Venerida;Veneroidea;Veneridae;Mercenaria;Mercenaria mercenaria
UdeM00000038976 6596 614.4 UdeM00000038976 XP_045188035.1 60.3 532 196 6 18 539 19 545 1.50E-172 614.4 6596 cellular

organisms;Eukaryota;Opisthokonta;Metazoa;Eumetazoa;Bilateria;Protostomia;Spiralia;Lophotrochozoa;Mollusca;Bivalvia;Autobranchia;Heteroconchia;Euheterodonta;Imparidentia;Venerida;Veneroidea;Veneridae;Mercenaria;Mercenaria mercenaria
UdeM00000038977 451745 105.9 UdeM00000038977 XP_037531102.1 31.5 181 118 3 73 253 212 386 7.80E-20 105.9 451745 cellular organisms;Eukaryota;Opisthokonta;Metazoa;Eumetazoa;Bilateria;Deuterostomia;Chordata;Craniata;Vertebrata;Gnathostomata;Teleostomi;Euteleostomi;Actinopterygii;Actinopteri;Neopterygii;Teleostei;Osteoglossocephalai;Clupeocephala;Euteleosteomorpha;Neoteleostei;Eurypterygia;Ctenosquamata;Acanthomorphata;Euacanthomorphacea;Percomorphaceae;Ovalentaria;Atherinomorphae;Cyprinodontiformes;Aplocheiloidei;Rivulidae;Nematolebias;Nematolebias whitei
UdeM00000038978 6596 672.9 UdeM00000038978 XP_045172382.1 64.2 565 175 9 3 561 33 576 3.60E-190 672.9 6596 cellular organisms;Eukaryota;Opisthokonta;Metazoa;Eumetazoa;Bilateria;Protostomia;Spiralia;Lophotrochozoa;Mollusca;Bivalvia;Autobranchia;Heteroconchia;Euheterodonta;Imparidentia;Venerida;Veneroidea;Veneridae;Mercenaria;Mercenaria mercenaria
UdeM00000038979 6596 700.7 UdeM00000038979 XP_045172382.1 65.2 586 173 8 20 599 19 579 1.70E-198 700.7 6596 cellular organisms;Eukaryota;Opisthokonta;Metazoa;Eumetazoa;Bilateria;Protostomia;Spiralia;Lophotrochozoa;Mollusca;Bivalvia;Autobranchia;Heteroconchia;Euheterodonta;Imparidentia;Venerida;Veneroidea;Veneridae;Mercenaria;Mercenaria mercenaria
UdeM00000038980 6596 694.5 UdeM00000038980 XP_045172382.1 64.3 594 180 10 68 655 19 586 1.30E-196 694.5 6596 cellular organisms;Eukaryota;Opisthokonta;Metazoa;Eumetazoa;Bilateria;Protostomia;Spiralia;Lophotrochozoa;Mollusca;Bivalvia;Autobranchia;Heteroconchia;Euheterodonta;Imparidentia;Venerida;Veneroidea;Veneridae;Mercenaria;Mercenaria mercenaria
UdeM00000038981 6596 682.6 UdeM00000038981 XP_045172400.1 65.7 566 184 7 77 637 39 599 5.00E-193 682.6 6596 cellular organisms;Eukaryota;Opisthokonta;Metazoa;Eumetazoa;Bilateria;Protostomia;Spiralia;Lophotrochozoa;Mollusca;Bivalvia;Autobranchia;Heteroconchia;Euheterodonta;Imparidentia;Venerida;Veneroidea;Veneridae;Mercenaria;Mercenaria mercenaria
UdeM00000038982 6596 515.8 UdeM00000038982 XP_045172365.1 67 352 116 0 1 352 64 415 4.40E-143 515.8 6596 cellular organisms;Eukaryota;Opisthokonta;Metazoa;Eumetazoa;Bilateria;Protostomia;Spiralia;Lophotrochozoa;Mollusca;Bivalvia;Autobranchia;Heteroconchia;Euheterodonta;Imparidentia;Venerida;Veneroidea;Veneridae;Mercenaria;Mercenaria mercenaria
UdeM00000038983 6596 547.4 UdeM00000038983 XP_045172365.1 67.7 371 120 0 5 375 45 415 1.50E-152 547.4 6596 cellular organisms;Eukaryota;Opisthokonta;Metazoa;Eumetazoa;Bilateria;Protostomia;Spiralia;Lophotrochozoa;Mollusca;Bivalvia;Autobranchia;Heteroconchia;Euheterodonta;Imparidentia;Venerida;Veneroidea;Veneridae;Mercenaria;Mercenaria mercenaria
UdeM00000038984 6596 280 UdeM00000038984 XP_045162613.1 59.7 216 85 1 1 214 1 216 2.60E-72 280 6596 cellular organisms;Eukaryota;Opisthokonta;Metazoa;Eumetazoa;Bilateria;Protostomia;Spiralia;Lophotrochozoa;Mollusca;Bivalvia;Autobranchia;Heteroconchia;Euhe

terodonta;Imparidentia;Venerida;Veneroidea;Veneridae;Mercenaria;Mercenaria mercenaria
UdeM00000038985 7741 292.4 UdeM00000038985 XP_019647976.1 63.8 210 76 0 6 215 358 567 4.90E-76 292.4 7741 cellular organisms;Eukaryota;Opisthokonta;Metazoa;Eumetazoa;Bilateria;Deuterostomia;Chordata;Cephalochordata;Leptocardii;Amphioxiformes;Branchiostomidae;Branchiostoma;Branchiostoma belcheri
UdeM00000038986 6579 113.2 UdeM00000038986 XP_033733871.1 70.7 75 22 0 1 75 1 75 1.40E-22 113.2 6579 cellular organisms;Eukaryota;Opisthokonta;Metazoa;Eumetazoa;Bilateria;Protostomia;Spiralia;Lophotrochozoa;Mollusca;Bivalvia;Autobranchia;Pteriomorphia;Pectinida;Pectinoidea;Pectinidae;Pecten;Pecten maximus
UdeM00000038987 1735272 991.1 UdeM00000038987 XP_041353357.1 62.1 766 285 4 1 765 7 768 7.80E-286 991.1 1735272 cellular organisms;Eukaryota;Opisthokonta;Metazoa;Eumetazoa;Bilateria;Protostomia;Spiralia;Lophotrochozoa;Mollusca;Gastropoda;Neomphalina;Neomphaloidea;Peltospiridae;Gigantopelta;Gigantopelta aegis
UdeM00000038988 6596 322.8 UdeM00000038988 XP_045174735.1 41.5 414 219 8 15 427 28 419 6.70E-85 322.8 6596 cellular organisms;Eukaryota;Opisthokonta;Metazoa;Eumetazoa;Bilateria;Protostomia;Spiralia;Lophotrochozoa;Mollusca;Bivalvia;Autobranchia;Heteroconchia;Euheterodonta;Imparidentia;Venerida;Veneroidea;Veneridae;Mercenaria;Mercenaria mercenaria
UdeM00000038989 48498 993 UdeM00000038989 XP_020618976.1 46.1 942 492 5 215 1154 1327 2254 3.30E-286 993 48498 cellular organisms;Eukaryota;Opisthokonta;Metazoa;Eumetazoa;Cnidaria;Anthozoa;Hexacorallia;Scleractinia;Faviina;Merulinidae;Orbicella;Orbicella faveolata
UdeM00000038991 29159 472.2 UdeM00000038991 XP_034336072.1 52.4 389 182 2 334 719 1447 1835 1.20E-129 472.2 29159 cellular organisms;Eukaryota;Opisthokonta;Metazoa;Eumetazoa;Bilateria;Protostomia;Spiralia;Lophotrochozoa;Mollusca;Bivalvia;Autobranchia;Pteriomorphia;Ostreida;Ostreoidea;Ostreidae;Crassostrea;Crassostrea gigas
UdeM00000038991 29159 155.6 UdeM00000038991 XP_034336072.1 27.6 655 396 21 31 645 221 837 2.50E-34 155.6 29159 cellular organisms;Eukaryota;Opisthokonta;Metazoa;Eumetazoa;Bilateria;Protostomia;Spiralia;Lophotrochozoa;Mollusca;Bivalvia;Autobranchia;Pteriomorphia;Ostreida;Ostreoidea;Ostreidae;Crassostrea;Crassostrea gigas
UdeM00000038992 6596 1689.9 UdeM00000038992 XP_045164211.1 59.7 1283 503 9 1 1273 1 1279 0.00E+00 1689.9 6596 cellular organisms;Eukaryota;Opisthokonta;Metazoa;Eumetazoa;Bilateria;Protostomia;Spiralia;Lophotrochozoa;Mollusca;Bivalvia;Autobranchia;Heteroconchia;Euheterodonta;Imparidentia;Venerida;Veneroidea;Veneridae;Mercenaria;Mercenaria mercenaria
UdeM00000038993 6573 110.9 UdeM00000038993 XP_021340561.1 44.4 135 66 4 1 127 34 167 3.10E-21 110.9 6573 cellular organisms;Eukaryota;Opisthokonta;Metazoa;Eumetazoa;Bilateria;Protostomia;Spiralia;Lophotrochozoa;Mollusca;Bivalvia;Autobranchia;Pteriomorphia;Pectinida;Pectinoidea;Pectinidae;Mizuhopecten;Mizuhopecten yessoensis
UdeM00000038994 225164 274.6 UdeM00000038994 XP_009063678.1 79 162 34 0 43 204 1 162 1.00E-70 274.6 225164 cellular organisms;Eukaryota;Opisthokonta;Metazoa;Eumetazoa;Bilateria;Protostomia;

Spiralia;Lophotrochozoa;Mollusca;Gastropoda;Patellogastropoda;Lottioidea;
Lottiidae;Lottia;Lottia gigantea
UdeM00000038995 6596 1165.2 UdeM00000038995 XP_045178583.1 67.9
920 269 9 1 902 188 1099 0.00E+00 1165.2 6596
cellular
organisms;Eukaryota;Opisthokonta;Metazoa;Eumetazoa;Bilateria;Protostomia;
Spiralia;Lophotrochozoa;Mollusca;Bivalvia;Autobranchia;Heteroconchia;Euhe
terodonta;Imparidentia;Venerida;Veneroidea;Veneridae;Mercenaria;Mercenari
a mercenaria
UdeM00000038997 6526 68.2 UdeM00000038997 XP_013094521.1 39.1 128
77 1 1 127 169 296 1.60E-08 68.2 6526 cellular
organisms;Eukaryota;Opisthokonta;Metazoa;Eumetazoa;Bilateria;Protostomia;
Spiralia;Lophotrochozoa;Mollusca;Gastropoda;Heterobranchia;Euthyneura;Pan
pulmonata;Hygrophila;Lymnaeoidea;Planorbidae;Biomphalaria;Biomphalaria
glabrata
UdeM00000038998 6596 317.4 UdeM00000038998 XP_045176506.1 58 264
103 4 1 257 125 387 1.70E-83 317.4 6596 cellular
organisms;Eukaryota;Opisthokonta;Metazoa;Eumetazoa;Bilateria;Protostomia;
Spiralia;Lophotrochozoa;Mollusca;Bivalvia;Autobranchia;Heteroconchia;Euhe
terodonta;Imparidentia;Venerida;Veneroidea;Veneridae;Mercenaria;Mercenari
a mercenaria
UdeM00000038999 6526 178.7 UdeM00000038999 XP_013079253.1 57.7 142
60 0 1 142 1 142 5.20E-42 178.7 6526 cellular
organisms;Eukaryota;Opisthokonta;Metazoa;Eumetazoa;Bilateria;Protostomia;
Spiralia;Lophotrochozoa;Mollusca;Gastropoda;Heterobranchia;Euthyneura;Pan
pulmonata;Hygrophila;Lymnaeoidea;Planorbidae;Biomphalaria;Biomphalaria
glabrata
UdeM00000039000 6565 133.3 UdeM00000039000 XP_022329085.1 68.2 110
32 1 27 136 8 114 2.40E-28 133.3 6565 cellular
organisms;Eukaryota;Opisthokonta;Metazoa;Eumetazoa;Bilateria;Protostomia;
Spiralia;Lophotrochozoa;Mollusca;Bivalvia;Autobranchia;Pteriomorphia;Ostr
eida;Ostreoidea;Ostreidae;Crassostrea;Crassostrea virginica
UdeM00000039001 29159 575.9 UdeM00000039001 XP_034325001.1 63.5 414
148 2 22 433 18 430 4.40E-161 575.9 29159 cellular
organisms;Eukaryota;Opisthokonta;Metazoa;Eumetazoa;Bilateria;Protostomia;
Spiralia;Lophotrochozoa;Mollusca;Bivalvia;Autobranchia;Pteriomorphia;Ostr
eida;Ostreoidea;Ostreidae;Crassostrea;Crassostrea gigas
UdeM00000039002 29159 550.8 UdeM00000039002 XP_034325001.1 63.5 394
141 2 1 392 38 430 1.40E-153 550.8 29159 cellular
organisms;Eukaryota;Opisthokonta;Metazoa;Eumetazoa;Bilateria;Protostomia;
Spiralia;Lophotrochozoa;Mollusca;Bivalvia;Autobranchia;Pteriomorphia;Ostr
eida;Ostreoidea;Ostreidae;Crassostrea;Crassostrea gigas
UdeM00000039003 29159 253.4 UdeM00000039003 XP_011426298.2 62.5 200
67 3 1 193 1 199 2.60E-64 253.4 29159 cellular
organisms;Eukaryota;Opisthokonta;Metazoa;Eumetazoa;Bilateria;Protostomia;
Spiralia;Lophotrochozoa;Mollusca;Bivalvia;Autobranchia;Pteriomorphia;Ostr
eida;Ostreoidea;Ostreidae;Crassostrea;Crassostrea gigas
UdeM00000039004 6596 1399 UdeM00000039004 XP_045187193.1 71.1 1000
274 6 1 986 1 999 0.00E+00 1399 6596 cellular
organisms;Eukaryota;Opisthokonta;Metazoa;Eumetazoa;Bilateria;Protostomia;
Spiralia;Lophotrochozoa;Mollusca;Bivalvia;Autobranchia;Heteroconchia;Euhe
terodonta;Imparidentia;Venerida;Veneroidea;Veneridae;Mercenaria;Mercenari
a mercenaria

UdeM00000039005 6565 304.7 UdeM00000039005 XP_022333584.1 53.9 282
118 4 9 287 385 657 1.30E-79 304.7 6565 cellular
organisms;Eukaryota;Opisthokonta;Metazoa;Eumetazoa;Bilateria;Protostomia;
Spiralia;Lophotrochozoa;Mollusca;Bivalvia;Autobranchia;Pteriomorphia;Ostr
eida;Ostreoidea;Ostreidae;Crassostrea;Crassostrea virginica
UdeM00000039006 6565 132.9 UdeM00000039006 XP_022333584.1 51.2 127
62 0 14 140 262 388 3.20E-28 132.9 6565 cellular
organisms;Eukaryota;Opisthokonta;Metazoa;Eumetazoa;Bilateria;Protostomia;
Spiralia;Lophotrochozoa;Mollusca;Bivalvia;Autobranchia;Pteriomorphia;Ostr
eida;Ostreoidea;Ostreidae;Crassostrea;Crassostrea virginica
UdeM00000039007 6565 132.9 UdeM00000039007 XP_022333584.1 51.2 127
62 0 17 143 262 388 3.30E-28 132.9 6565 cellular
organisms;Eukaryota;Opisthokonta;Metazoa;Eumetazoa;Bilateria;Protostomia;
Spiralia;Lophotrochozoa;Mollusca;Bivalvia;Autobranchia;Pteriomorphia;Ostr
eida;Ostreoidea;Ostreidae;Crassostrea;Crassostrea virginica
UdeM00000039008 6565 151.8 UdeM00000039008 XP_022310266.1 33.4 353
192 10 1 337 1 326 1.60E-33 151.8 6565 cellular
organisms;Eukaryota;Opisthokonta;Metazoa;Eumetazoa;Bilateria;Protostomia;
Spiralia;Lophotrochozoa;Mollusca;Bivalvia;Autobranchia;Pteriomorphia;Ostr
eida;Ostreoidea;Ostreidae;Crassostrea;Crassostrea virginica
UdeM00000039009 6565 151.8 UdeM00000039009 XP_022310266.1 33.4 353
192 10 1 337 1 326 1.60E-33 151.8 6565 cellular
organisms;Eukaryota;Opisthokonta;Metazoa;Eumetazoa;Bilateria;Protostomia;
Spiralia;Lophotrochozoa;Mollusca;Bivalvia;Autobranchia;Pteriomorphia;Ostr
eida;Ostreoidea;Ostreidae;Crassostrea;Crassostrea virginica
UdeM00000039010 6565 151.8 UdeM00000039010 XP_022310266.1 33.4 353
192 10 1 337 1 326 1.60E-33 151.8 6565 cellular
organisms;Eukaryota;Opisthokonta;Metazoa;Eumetazoa;Bilateria;Protostomia;
Spiralia;Lophotrochozoa;Mollusca;Bivalvia;Autobranchia;Pteriomorphia;Ostr
eida;Ostreoidea;Ostreidae;Crassostrea;Crassostrea virginica
UdeM00000039011 6565 151.8 UdeM00000039011 XP_022310266.1 33.4 353
192 10 1 337 1 326 1.60E-33 151.8 6565 cellular
organisms;Eukaryota;Opisthokonta;Metazoa;Eumetazoa;Bilateria;Protostomia;
Spiralia;Lophotrochozoa;Mollusca;Bivalvia;Autobranchia;Pteriomorphia;Ostr
eida;Ostreoidea;Ostreidae;Crassostrea;Crassostrea virginica
UdeM00000039012 6579 132.9 UdeM00000039012 XP_033736434.1 72.8 81
22 0 36 116 197 277 2.70E-28 132.9 6579 cellular
organisms;Eukaryota;Opisthokonta;Metazoa;Eumetazoa;Bilateria;Protostomia;
Spiralia;Lophotrochozoa;Mollusca;Bivalvia;Autobranchia;Pteriomorphia;Pect
inida;Pectinoidea;Pectinidae;Pecten;Pecten maximus
UdeM00000039013 6579 179.9 UdeM00000039013 XP_033734181.1 57.3 157
57 2 34 190 19 165 3.40E-42 179.9 6579 cellular
organisms;Eukaryota;Opisthokonta;Metazoa;Eumetazoa;Bilateria;Protostomia;
Spiralia;Lophotrochozoa;Mollusca;Bivalvia;Autobranchia;Pteriomorphia;Pect
inida;Pectinoidea;Pectinidae;Pecten;Pecten maximus
UdeM00000039014 13164 70.1 UdeM00000039014 XP_022164283.1 35.1 94
61 0 1 94 1 94 2.10E-09 70.1 13164 cellular
organisms;Eukaryota;Opisthokonta;Metazoa;Eumetazoa;Bilateria;Protostomia;
Ecdysozoa;Panarthropoda;Arthropoda;Mandibulata;Pancrustacea;Hexapoda;Inse
cta;Dicondylia;Pterygota;Neoptera;Paraneoptera;Hemiptera;Sternorrhyncha;A
phidomorpha;Aphidoidea;Aphididae;Aphidinae;Macrosiphini;Myzus;Myzus
persicae
UdeM00000039015 6596 278.1 UdeM00000039015 XP_045213934.1 50.3 300
133 3 5 300 4 291 1.30E-71 278.1 6596 cellular

organisms;Eukaryota;Opisthokonta;Metazoa;Eumetazoa;Bilateria;Protostomia;Spiralia;Lophotrochozoa;Mollusca;Bivalvia;Autobranchia;Heteroconchia;Euheterodonta;Imparidentia;Venerida;Veneroidea;Veneridae;Mercenaria;Mercenaria mercenaria
UdeM00000039017 146911 57.4 UdeM00000039017 XP_015270826.1 38.6 83 47 2 1 79 1 83 9.80E-06 57.4 146911 cellular organisms;Eukaryota;Opisthokonta;Metazoa;Eumetazoa;Bilateria;Deuterostomia;Chordata;Craniata;Vertebrata;Gnathostomata;Teleostomi;Euteleostomi;Sarcopterygii;Dipnotetrapodomorpha;Tetrapoda;Amniota;Sauropsida;Sauria;Lepidosauria;Squamata;Bifurcata;Gekkota;Gekkonidae;Gekkoninae;Gekko;Gekko japonicus
UdeM00000039020 29144 365.2 UdeM00000039020 XP_030650271.1 59.4 325 130 2 7 331 6 328 1.40E-97 365.2 29144 cellular organisms;Eukaryota;Opisthokonta;Metazoa;Eumetazoa;Bilateria;Deuterostomia;Chordata;Craniata;Vertebrata;Gnathostomata;Teleostomi;Euteleostomi;Actinopterygii;Actinopteri;Neopterygii;Teleostei;Osteoglossocephalai;Clupeocephala;Otomorpha;Ostariophysi;Anotophysi;Gonorynchiformes;Chanoidei;Chanidae;Chanos;Chanos chanos
UdeM00000039021 6596 648.7 UdeM00000039021 XP_045162993.1 56.6 722 258 15 73 771 95 784 9.60E-183 648.7 6596 cellular organisms;Eukaryota;Opisthokonta;Metazoa;Eumetazoa;Bilateria;Protostomia;Spiralia;Lophotrochozoa;Mollusca;Bivalvia;Autobranchia;Heteroconchia;Euheterodonta;Imparidentia;Venerida;Veneroidea;Veneridae;Mercenaria;Mercenaria mercenaria
UdeM00000039022 6596 639 UdeM00000039022 XP_045162993.1 56.2 726 261 15 73 777 95 784 7.70E-180 639 6596 cellular organisms;Eukaryota;Opisthokonta;Metazoa;Eumetazoa;Bilateria;Protostomia;Spiralia;Lophotrochozoa;Mollusca;Bivalvia;Autobranchia;Heteroconchia;Euheterodonta;Imparidentia;Venerida;Veneroidea;Veneridae;Mercenaria;Mercenaria mercenaria
UdeM00000039023 6579 426.8 UdeM00000039023 XP_033733718.1 43.6 582 282 13 1 553 22 586 4.30E-116 426.8 6579 cellular organisms;Eukaryota;Opisthokonta;Metazoa;Eumetazoa;Bilateria;Protostomia;Spiralia;Lophotrochozoa;Mollusca;Bivalvia;Autobranchia;Pteriomorphia;Pectinida;Pectinoidea;Pectinidae;Pecten;Pecten maximus
UdeM00000039025 6596 287 UdeM00000039025 XP_045184098.1 43.8 322 170 5 84 405 48 358 3.90E-74 287 6596 cellular organisms;Eukaryota;Opisthokonta;Metazoa;Eumetazoa;Bilateria;Protostomia;Spiralia;Lophotrochozoa;Mollusca;Bivalvia;Autobranchia;Heteroconchia;Euheterodonta;Imparidentia;Venerida;Veneroidea;Veneridae;Mercenaria;Mercenaria mercenaria
UdeM00000039026 6596 167.2 UdeM00000039026 XP_045169960.1 24.5 633 424 21 13 629 16 610 6.90E-38 167.2 6596 cellular organisms;Eukaryota;Opisthokonta;Metazoa;Eumetazoa;Bilateria;Protostomia;Spiralia;Lophotrochozoa;Mollusca;Bivalvia;Autobranchia;Heteroconchia;Euheterodonta;Imparidentia;Venerida;Veneroidea;Veneridae;Mercenaria;Mercenaria mercenaria
UdeM00000039027 9515 146.7 UdeM00000039027 XP_032127860.1 63.5 115 40 2 39 153 24 136 2.50E-32 146.7 9515 cellular organisms;Eukaryota;Opisthokonta;Metazoa;Eumetazoa;Bilateria;Deuterostomia;Chordata;Craniata;Vertebrata;Gnathostomata;Teleostomi;Euteleostomi;Sarcopterygii;Dipnotetrapodomorpha;Tetrapoda;Amniota;Mammalia;Theria;Eutheria

;Boreoeutheria;Euarchontoglires;Primates;Haplorrhini;Simiiformes;Platyrrhini;Cebidae;Cebinae;Sapajus;Sapajus apella
UdeM00000039028 9515 146.7 UdeM00000039028 XP_032127860.1 63.5 115 40 2 27 141 24 136 2.30E-32 146.7 9515 cellular organisms;Eukaryota;Opisthokonta;Metazoa;Eumetazoa;Bilateria;Deuterostomia;Chordata;Craniata;Vertebrata;Gnathostomata;Teleostomi;Euteleostomi;Sarcopterygii;Dipnotetrapodomorpha;Tetrapoda;Amniota;Mammalia;Theria;Eutheria;Boreoeutheria;Euarchontoglires;Primates;Haplorrhini;Simiiformes;Platyrrhini;Cebidae;Cebinae;Sapajus;Sapajus apella
UdeM00000039030 6573 205.7 UdeM00000039030 XP_021370611.1 71.1 152 44 0 1 152 1 152 4.20E-50 205.7 6573 cellular organisms;Eukaryota;Opisthokonta;Metazoa;Eumetazoa;Bilateria;Protostomia;Spiralia;Lophotrochozoa;Mollusca;Bivalvia;Autobranchia;Pteriomorphia;Pectinida;Pectinoidea;Pectinidae;Mizuhopecten;Mizuhopecten yessoensis
UdeM00000039029 6596 300.4 UdeM00000039029 XP_045164917.1 56.6 362 142 4 6 360 11 364 3.00E-78 300.4 6596 cellular organisms;Eukaryota;Opisthokonta;Metazoa;Eumetazoa;Bilateria;Protostomia;Spiralia;Lophotrochozoa;Mollusca;Bivalvia;Autobranchia;Heteroconchia;Euheterodonta;Imparidentia;Venerida;Veneroidea;Veneridae;Mercenaria;Mercenaria mercenaria
UdeM00000039031 6579 326.2 UdeM00000039031 XP_033734833.1 47.7 478 221 12 1 462 1 465 8.70E-86 326.2 6579 cellular organisms;Eukaryota;Opisthokonta;Metazoa;Eumetazoa;Bilateria;Protostomia;Spiralia;Lophotrochozoa;Mollusca;Bivalvia;Autobranchia;Pteriomorphia;Pectinida;Pectinoidea;Pectinidae;Pecten;Pecten maximus
UdeM00000039032 6579 330.9 UdeM00000039032 XP_033734833.1 47.7 478 222 11 1 463 1 465 3.50E-87 330.9 6579 cellular organisms;Eukaryota;Opisthokonta;Metazoa;Eumetazoa;Bilateria;Protostomia;Spiralia;Lophotrochozoa;Mollusca;Bivalvia;Autobranchia;Pteriomorphia;Pectinida;Pectinoidea;Pectinidae;Pecten;Pecten maximus
UdeM00000039033 6596 707.6 UdeM00000039033 XP_045164872.1 81.8 495 88 1 1 495 1 493 1.20E-200 707.6 6596 cellular organisms;Eukaryota;Opisthokonta;Metazoa;Eumetazoa;Bilateria;Protostomia;Spiralia;Lophotrochozoa;Mollusca;Bivalvia;Autobranchia;Heteroconchia;Euheterodonta;Imparidentia;Venerida;Veneroidea;Veneridae;Mercenaria;Mercenaria mercenaria
UdeM00000039034 6573 292.4 UdeM00000039034 XP_021370558.1 70 180 54 0 25 204 533 712 4.70E-76 292.4 6573 cellular organisms;Eukaryota;Opisthokonta;Metazoa;Eumetazoa;Bilateria;Protostomia;Spiralia;Lophotrochozoa;Mollusca;Bivalvia;Autobranchia;Pteriomorphia;Pectinida;Pectinoidea;Pectinidae;Mizuhopecten;Mizuhopecten yessoensis
UdeM00000039035 400727 492.7 UdeM00000039035 XP_025104969.1 66.8 346 68 2 1 342 390 692 3.90E-136 492.7 400727 cellular organisms;Eukaryota;Opisthokonta;Metazoa;Eumetazoa;Bilateria;Protostomia;Spiralia;Lophotrochozoa;Mollusca;Gastropoda;Caenogastropoda;Architaenioglossa;Ampullarioidea;Ampullariidae;Pomacea;Pomacea canaliculata
UdeM00000039036 6579 163.3 UdeM00000039036 XP_033734829.1 74.1 112 29 0 74 185 276 387 3.40E-37 163.3 6579 cellular organisms;Eukaryota;Opisthokonta;Metazoa;Eumetazoa;Bilateria;Protostomia;Spiralia;Lophotrochozoa;Mollusca;Bivalvia;Autobranchia;Pteriomorphia;Pectinida;Pectinoidea;Pectinidae;Pecten;Pecten maximus
UdeM00000039037 6596 365.9 UdeM00000039037 XP_045164833.1 65.4 257 84 3 1 252 1 257 4.30E-98 365.9 6596 cellular

organisms;Eukaryota;Opisthokonta;Metazoa;Eumetazoa;Bilateria;Protostomia;Spiralia;Lophotrochozoa;Mollusca;Bivalvia;Autobranchia;Heteroconchia;Euheterodonta;Imparidentia;Venerida;Veneroidea;Veneridae;Mercenaria;Mercenaria mercenaria
UdeM00000039038 6579 669.5 UdeM00000039038 XP_033734828.1 68.5 461 139 3 1 457 1 459 3.10E-189 669.5 6579 cellular organisms;Eukaryota;Opisthokonta;Metazoa;Eumetazoa;Bilateria;Protostomia;Spiralia;Lophotrochozoa;Mollusca;Bivalvia;Autobranchia;Pteriomorphia;Pectinida;Pectinoidea;Pectinidae;Pecten;Pecten maximus
UdeM00000039039 6596 666 UdeM00000039039 XP_045164800.1 59.4 498 201 1 36 533 42 538 4.00E-188 666 6596 cellular organisms;Eukaryota;Opisthokonta;Metazoa;Eumetazoa;Bilateria;Protostomia;Spiralia;Lophotrochozoa;Mollusca;Bivalvia;Autobranchia;Heteroconchia;Euheterodonta;Imparidentia;Venerida;Veneroidea;Veneridae;Mercenaria;Mercenaria mercenaria
UdeM00000039040 6596 666 UdeM00000039040 XP_045164800.1 59.4 498 201 1 27 524 42 538 3.90E-188 666 6596 cellular organisms;Eukaryota;Opisthokonta;Metazoa;Eumetazoa;Bilateria;Protostomia;Spiralia;Lophotrochozoa;Mollusca;Bivalvia;Autobranchia;Heteroconchia;Euheterodonta;Imparidentia;Venerida;Veneroidea;Veneridae;Mercenaria;Mercenaria mercenaria
UdeM00000039041 29159 323.2 UdeM00000039041 XP_034326737.1 56.8 324 124 6 10 328 7 319 4.20E-85 323.2 29159 cellular organisms;Eukaryota;Opisthokonta;Metazoa;Eumetazoa;Bilateria;Protostomia;Spiralia;Lophotrochozoa;Mollusca;Bivalvia;Autobranchia;Pteriomorphia;Ostreida;Ostreoidea;Ostreidae;Crassostrea;Crassostrea gigas
UdeM00000039042 6565 203 UdeM00000039042 XP_022334649.1 38.7 315 180 5 24 330 4 313 6.20E-49 203 6565 cellular organisms;Eukaryota;Opisthokonta;Metazoa;Eumetazoa;Bilateria;Protostomia;Spiralia;Lophotrochozoa;Mollusca;Bivalvia;Autobranchia;Pteriomorphia;Ostreida;Ostreoidea;Ostreidae;Crassostrea;Crassostrea virginica
UdeM00000039043 6596 166 UdeM00000039043 XP_045166071.1 38.1 286 161 7 14 291 321 598 7.60E-38 166 6596 cellular organisms;Eukaryota;Opisthokonta;Metazoa;Eumetazoa;Bilateria;Protostomia;Spiralia;Lophotrochozoa;Mollusca;Bivalvia;Autobranchia;Heteroconchia;Euheterodonta;Imparidentia;Venerida;Veneroidea;Veneridae;Mercenaria;Mercenaria mercenaria
UdeM00000039044 6596 388.3 UdeM00000039044 XP_045166082.1 32.7 947 521 20 23 958 17 858 3.00E-104 388.3 6596 cellular organisms;Eukaryota;Opisthokonta;Metazoa;Eumetazoa;Bilateria;Protostomia;Spiralia;Lophotrochozoa;Mollusca;Bivalvia;Autobranchia;Heteroconchia;Euheterodonta;Imparidentia;Venerida;Veneroidea;Veneridae;Mercenaria;Mercenaria mercenaria
UdeM00000039045 6579 303.1 UdeM00000039045 XP_033756871.1 31.8 655 410 16 4 642 22 655 1.00E-78 303.1 6579 cellular organisms;Eukaryota;Opisthokonta;Metazoa;Eumetazoa;Bilateria;Protostomia;Spiralia;Lophotrochozoa;Mollusca;Bivalvia;Autobranchia;Pteriomorphia;Pectinida;Pectinoidea;Pectinidae;Pecten;Pecten maximus
UdeM00000039047 6596 352.1 UdeM00000039047 XP_045166082.1 31.6 895 487 19 23 900 17 803 2.20E-93 352.1 6596 cellular organisms;Eukaryota;Opisthokonta;Metazoa;Eumetazoa;Bilateria;Protostomia;Spiralia;Lophotrochozoa;Mollusca;Bivalvia;Autobranchia;Heteroconchia;Euheterodonta;Imparidentia;Venerida;Veneroidea;Veneridae;Mercenaria;Mercenaria mercenaria

UdeM00000039049 29159 284.3 UdeM00000039049 XP_011428416.3 32.4 584
360 16 29 588 20 592 3.80E-73 284.3 29159 cellular organisms;Eukaryota;Opisthokonta;Metazoa;Eumetazoa;Bilateria;Protostomia;Spiralia;Lophotrochozoa;Mollusca;Bivalvia;Autobranchia;Pteriomorphia;Ostreida;Ostreoidea;Ostreidae;Crassostrea;Crassostrea gigas
UdeM00000039050 29159 266.9 UdeM00000039050 XP_011428416.3 31.1 575
347 16 29 565 20 583 5.90E-68 266.9 29159 cellular organisms;Eukaryota;Opisthokonta;Metazoa;Eumetazoa;Bilateria;Protostomia;Spiralia;Lophotrochozoa;Mollusca;Bivalvia;Autobranchia;Pteriomorphia;Ostreida;Ostreoidea;Ostreidae;Crassostrea;Crassostrea gigas
UdeM00000044202 554065 165.6 UdeM00000044202 XP_005849672.1 39.9
253 144 4 6 255 3 250 8.90E-38 165.6 554065
cellular organisms;Eukaryota;Viridiplantae;Chlorophyta;core chlorophytes;Trebouxiophyceae;Chlorellales;Chlorellaceae;Chlorella clade;Chlorella;Chlorella variabilis
UdeM00000025039 6596 531.2 UdeM00000025039 XP_045204475.1 72.8 334
90 1 22 355 23 355 1.00E-147 531.2 6596 cellular organisms;Eukaryota;Opisthokonta;Metazoa;Eumetazoa;Bilateria;Protostomia;Spiralia;Lophotrochozoa;Mollusca;Bivalvia;Autobranchia;Heteroconchia;Euheterodonta;Imparidentia;Venerida;Veneroidea;Veneridae;Mercenaria;Mercenaria mercenaria
UdeM00000025038 6596 805.4 UdeM00000025038 XP_045204475.1 61.4 622
223 8 22 635 23 635 5.20E-230 805.4 6596 cellular organisms;Eukaryota;Opisthokonta;Metazoa;Eumetazoa;Bilateria;Protostomia;Spiralia;Lophotrochozoa;Mollusca;Bivalvia;Autobranchia;Heteroconchia;Euheterodonta;Imparidentia;Venerida;Veneroidea;Veneridae;Mercenaria;Mercenaria mercenaria
UdeM00000025040 400727 341.3 UdeM00000025040 XP_025094507.1 53.4
313 142 4 5 315 325 635 1.40E-90 341.3 400727
cellular
organisms;Eukaryota;Opisthokonta;Metazoa;Eumetazoa;Bilateria;Protostomia;Spiralia;Lophotrochozoa;Mollusca;Gastropoda;Caenogastropoda;Architaenioglossa;Ampullarioidea;Ampullariidae;Pomacea;Pomacea canaliculata
UdeM00000025041 6573 322 UdeM00000025041 XP_021346252.1 35.5 625
317 19 1 560 1 604 1.50E-84 322 6573 cellular organisms;Eukaryota;Opisthokonta;Metazoa;Eumetazoa;Bilateria;Protostomia;Spiralia;Lophotrochozoa;Mollusca;Bivalvia;Autobranchia;Pteriomorphia;Pectinida;Pectinoidea;Pectinidae;Mizuhopecten;Mizuhopecten yessoensis
UdeM00000025042 6573 624.8 UdeM00000025042 XP_021346253.1 87 355
46 0 1 355 1 355 6.80E-176 624.8 6573 cellular organisms;Eukaryota;Opisthokonta;Metazoa;Eumetazoa;Bilateria;Protostomia;Spiralia;Lophotrochozoa;Mollusca;Bivalvia;Autobranchia;Pteriomorphia;Pectinida;Pectinoidea;Pectinidae;Mizuhopecten;Mizuhopecten yessoensis
UdeM00000025044 10224 59.3 UdeM00000025044 XP_002733294.1 53.7 54
25 0 35 88 23 76 6.40E-06 59.3 10224 cellular organisms;Eukaryota;Opisthokonta;Metazoa;Eumetazoa;Bilateria;Deuterostomia;Hemichordata;Enteropneusta;Harrimaniidae;Saccoglossus;Saccoglossus kowalevskii
UdeM00000025046 6573 851.3 UdeM00000025046 XP_021346254.1 79.7 512
100 2 15 523 342 852 6.90E-244 851.3 6573 cellular organisms;Eukaryota;Opisthokonta;Metazoa;Eumetazoa;Bilateria;Protostomia;Spiralia;Lophotrochozoa;Mollusca;Bivalvia;Autobranchia;Pteriomorphia;Pectinida;Pectinoidea;Pectinidae;Mizuhopecten;Mizuhopecten yessoensis

UdeM00000025047	29159	481.9	UdeM00000025047	XP_034318405.1	76.7	318
68	3	1	313	38	354	6.50E-133	481.9	29159	cellular
organisms;Eukaryota;Opisthokonta;Metazoa;Eumetazoa;Bilateria;Protostomia;
Spiralia;Lophotrochozoa;Mollusca;Bivalvia;Autobranchia;Pteriomorphia;Ostr
eida;Ostreoidea;Ostreidae;Crassostrea;Crassostrea gigas
UdeM00000025050	6596	300.1	UdeM00000025050	XP_045204216.1	49	294
136	4	26	318	36	316	3.50E-78	300.1	6596	cellular
organisms;Eukaryota;Opisthokonta;Metazoa;Eumetazoa;Bilateria;Protostomia;
Spiralia;Lophotrochozoa;Mollusca;Bivalvia;Autobranchia;Heteroconchia;Euhe
terodonta;Imparidentia;Venerida;Veneroidea;Veneridae;Mercenaria;Mercenari
a mercenaria
UdeM00000025052	6573	181.4	UdeM00000025052	XP_021364077.1	45.3	278
131	6	690	955	1136	1404	5.40E-42	181.4	6573	cellular
organisms;Eukaryota;Opisthokonta;Metazoa;Eumetazoa;Bilateria;Protostomia;
Spiralia;Lophotrochozoa;Mollusca;Bivalvia;Autobranchia;Pteriomorphia;Pect
inida;Pectinoidea;Pectinidae;Mizuhopecten;Mizuhopecten yessoensis
UdeM00000025053	8040	70.9	UdeM00000025053	XP_038848645.1	28.7	178
126	1	180	357	1306	1482	6.50E-09	70.9	8040	cellular
organisms;Eukaryota;Opisthokonta;Metazoa;Eumetazoa;Bilateria;Deuterostomi
a;Chordata;Craniata;Vertebrata;Gnathostomata;Teleostomi;Euteleostomi;Acti
nopterygii;Actinopteri;Neopterygii;Teleostei;Osteoglossocephalai;Clupeoce
phala;Euteleosteomorpha;Protacanthopterygii;Salmoniformes;Salmonidae;Salm
oninae;Salvelinus;Salvelinus namaycush
UdeM00000025056	6596	684.1	UdeM00000025056	XP_045203147.1	70.2	473
136	1	1	468	1	473	1.20E-193	684.1	6596	cellular
organisms;Eukaryota;Opisthokonta;Metazoa;Eumetazoa;Bilateria;Protostomia;
Spiralia;Lophotrochozoa;Mollusca;Bivalvia;Autobranchia;Heteroconchia;Euhe
terodonta;Imparidentia;Venerida;Veneroidea;Veneridae;Mercenaria;Mercenari
a mercenaria
UdeM00000025057	2587831	130.2	UdeM00000025057	XP_026503620.1	45.3
139	76	0	8	146	4	142	2.60E-27	130.2	2587831
cellular
organisms;Eukaryota;Opisthokonta;Metazoa;Eumetazoa;Bilateria;Deuterostomi
a;Chordata;Craniata;Vertebrata;Gnathostomata;Teleostomi;Euteleostomi;Sarc
opterygii;Dipnotetrapodomorpha;Tetrapoda;Amniota;Sauropsida;Sauria;Archel
osauria;Testudinata;Testudines;Cryptodira;Durocryptodira;Testudinoidea;Em
ydidae;Terrapene;Terrapene carolina;Terrapene carolina triunguis
UdeM00000025058	6596	1109	UdeM00000025058	XP_045202911.1	78	699
125	2	1	698	1	671	0.00E+00	1109	6596	cellular
organisms;Eukaryota;Opisthokonta;Metazoa;Eumetazoa;Bilateria;Protostomia;
Spiralia;Lophotrochozoa;Mollusca;Bivalvia;Autobranchia;Heteroconchia;Euhe
terodonta;Imparidentia;Venerida;Veneroidea;Veneridae;Mercenaria;Mercenari
a mercenaria
UdeM00000025059	6596	1125.5	UdeM00000025059	XP_045202910.1	81.2
672	124	2	1	671	1	671	0.00E+00	1125.5	6596
cellular
organisms;Eukaryota;Opisthokonta;Metazoa;Eumetazoa;Bilateria;Protostomia;
Spiralia;Lophotrochozoa;Mollusca;Bivalvia;Autobranchia;Heteroconchia;Euhe
terodonta;Imparidentia;Venerida;Veneroidea;Veneridae;Mercenaria;Mercenari
a mercenaria
UdeM00000025060	6579	120.2	UdeM00000025060	XP_033736263.1	45.2	124
67	1	11	133	451	574	2.10E-24	120.2	6579	cellular
organisms;Eukaryota;Opisthokonta;Metazoa;Eumetazoa;Bilateria;Protostomia;

Spiralia;Lophotrochozoa;Mollusca;Bivalvia;Autobranchia;Pteriomorphia;Pectinida;Pectinoidea;Pectinidae;Pecten;Pecten maximus
UdeM00000025061 6596 194.5 UdeM00000025061 XP_045177053.1 39.1 253 139 6 267 514 189 431 3.30E-46 194.5 6596 cellular organisms;Eukaryota;Opisthokonta;Metazoa;Eumetazoa;Bilateria;Protostomia;Spiralia;Lophotrochozoa;Mollusca;Bivalvia;Autobranchia;Heteroconchia;Euheterodonta;Imparidentia;Venerida;Veneroidea;Veneridae;Mercenaria;Mercenaria mercenaria
UdeM00000025062 6579 112.1 UdeM00000025062 XP_033736263.1 45.5 112 60 1 20 130 463 574 5.50E-22 112.1 6579 cellular organisms;Eukaryota;Opisthokonta;Metazoa;Eumetazoa;Bilateria;Protostomia;Spiralia;Lophotrochozoa;Mollusca;Bivalvia;Autobranchia;Pteriomorphia;Pectinida;Pectinoidea;Pectinidae;Pecten;Pecten maximus
UdeM00000025063 6596 199.9 UdeM00000025063 XP_045181277.1 37.3 292 167 8 134 418 139 421 6.40E-48 199.9 6596 cellular organisms;Eukaryota;Opisthokonta;Metazoa;Eumetazoa;Bilateria;Protostomia;Spiralia;Lophotrochozoa;Mollusca;Bivalvia;Autobranchia;Heteroconchia;Euheterodonta;Imparidentia;Venerida;Veneroidea;Veneridae;Mercenaria;Mercenaria mercenaria
UdeM00000025064 6579 110.9 UdeM00000025064 XP_033736263.1 45.5 110 59 1 9 117 465 574 1.10E-21 110.9 6579 cellular organisms;Eukaryota;Opisthokonta;Metazoa;Eumetazoa;Bilateria;Protostomia;Spiralia;Lophotrochozoa;Mollusca;Bivalvia;Autobranchia;Pteriomorphia;Pectinida;Pectinoidea;Pectinidae;Pecten;Pecten maximus
UdeM00000025065 6596 154.1 UdeM00000025065 XP_045177053.1 42.2 185 95 4 303 483 255 431 4.60E-34 154.1 6596 cellular organisms;Eukaryota;Opisthokonta;Metazoa;Eumetazoa;Bilateria;Protostomia;Spiralia;Lophotrochozoa;Mollusca;Bivalvia;Autobranchia;Heteroconchia;Euheterodonta;Imparidentia;Venerida;Veneroidea;Veneridae;Mercenaria;Mercenaria mercenaria
UdeM00000025066 225164 129.4 UdeM00000025066 XP_009046882.1 53.8 106 49 0 218 323 27 132 8.20E-27 129.4 225164 cellular organisms;Eukaryota;Opisthokonta;Metazoa;Eumetazoa;Bilateria;Protostomia;Spiralia;Lophotrochozoa;Mollusca;Gastropoda;Patellogastropoda;Lottioidea;Lottiidae;Lottia;Lottia gigantea
UdeM00000025067 6596 120.2 UdeM00000025067 XP_045205692.1 28.8 386 185 9 627 996 540 851 1.50E-23 120.2 6596 cellular organisms;Eukaryota;Opisthokonta;Metazoa;Eumetazoa;Bilateria;Protostomia;Spiralia;Lophotrochozoa;Mollusca;Bivalvia;Autobranchia;Heteroconchia;Euheterodonta;Imparidentia;Venerida;Veneroidea;Veneridae;Mercenaria;Mercenaria mercenaria
UdeM00000025068 6596 120.2 UdeM00000025068 XP_045205692.1 28.8 386 185 9 600 969 540 851 1.50E-23 120.2 6596 cellular organisms;Eukaryota;Opisthokonta;Metazoa;Eumetazoa;Bilateria;Protostomia;Spiralia;Lophotrochozoa;Mollusca;Bivalvia;Autobranchia;Heteroconchia;Euheterodonta;Imparidentia;Venerida;Veneroidea;Veneridae;Mercenaria;Mercenaria mercenaria
UdeM00000025069 6596 973.4 UdeM00000025069 XP_045206013.1 46.9 897 466 6 177 1068 953 1844 3.40E-280 973.4 6596 cellular organisms;Eukaryota;Opisthokonta;Metazoa;Eumetazoa;Bilateria;Protostomia;Spiralia;Lophotrochozoa;Mollusca;Bivalvia;Autobranchia;Heteroconchia;Euheterodonta;Imparidentia;Venerida;Veneroidea;Veneridae;Mercenaria;Mercenaria mercenaria

UdeM00000025070 6596 1239.2 UdeM00000025070 XP_045205123.1 77.9 757 152 3 39 782 78 832 0.00E+00 1239.2 6596 cellular organisms;Eukaryota;Opisthokonta;Metazoa;Eumetazoa;Bilateria;Protostomia;Spiralia;Lophotrochozoa;Mollusca;Bivalvia;Autobranchia;Heteroconchia;Euheterodonta;Imparidentia;Venerida;Veneroidea;Veneridae;Mercenaria;Mercenaria mercenaria
UdeM00000025072 7574 98.6 UdeM00000025072 XP_023931656.1 36.8 144 88 3 7 149 119 260 8.90E-18 98.6 7574 cellular organisms;Eukaryota;Opisthokonta;Metazoa;Eumetazoa;Bilateria;Protostomia;Spiralia;Lophotrochozoa;Brachiopoda;Linguliformea;Lingulata;Lingulida;Linguloidea;Lingulidae;Lingula;Lingula anatina
UdeM00000025073 6596 278.9 UdeM00000025073 XP_045176230.1 32.6 565 338 19 9 556 36 574 1.50E-71 278.9 6596 cellular organisms;Eukaryota;Opisthokonta;Metazoa;Eumetazoa;Bilateria;Protostomia;Spiralia;Lophotrochozoa;Mollusca;Bivalvia;Autobranchia;Heteroconchia;Euheterodonta;Imparidentia;Venerida;Veneroidea;Veneridae;Mercenaria;Mercenaria mercenaria
UdeM00000025074 6596 117.9 UdeM00000025074 XP_045167526.1 30.6 252 158 8 6 241 196 446 1.90E-23 117.9 6596 cellular organisms;Eukaryota;Opisthokonta;Metazoa;Eumetazoa;Bilateria;Protostomia;Spiralia;Lophotrochozoa;Mollusca;Bivalvia;Autobranchia;Heteroconchia;Euheterodonta;Imparidentia;Venerida;Veneroidea;Veneridae;Mercenaria;Mercenaria mercenaria
UdeM00000025075 6596 58.2 UdeM00000025075 XP_045161740.1 33.3 96 54 2 43 132 26 117 9.90E-06 58.2 6596 cellular organisms;Eukaryota;Opisthokonta;Metazoa;Eumetazoa;Bilateria;Protostomia;Spiralia;Lophotrochozoa;Mollusca;Bivalvia;Autobranchia;Heteroconchia;Euheterodonta;Imparidentia;Venerida;Veneroidea;Veneridae;Mercenaria;Mercenaria mercenaria
UdeM00000025076 6596 232.6 UdeM00000025076 XP_045176230.1 30.4 487 303 14 106 578 57 521 1.20E-57 232.6 6596 cellular organisms;Eukaryota;Opisthokonta;Metazoa;Eumetazoa;Bilateria;Protostomia;Spiralia;Lophotrochozoa;Mollusca;Bivalvia;Autobranchia;Heteroconchia;Euheterodonta;Imparidentia;Venerida;Veneroidea;Veneridae;Mercenaria;Mercenaria mercenaria
UdeM00000025077 6596 94.4 UdeM00000025077 XP_045181544.1 30 180 113 4 3 171 280 457 1.50E-16 94.4 6596 cellular organisms;Eukaryota;Opisthokonta;Metazoa;Eumetazoa;Bilateria;Protostomia;Spiralia;Lophotrochozoa;Mollusca;Bivalvia;Autobranchia;Heteroconchia;Euheterodonta;Imparidentia;Venerida;Veneroidea;Veneridae;Mercenaria;Mercenaria mercenaria
UdeM00000025080 6596 304.3 UdeM00000025080 XP_045208657.1 32.9 638 369 24 14 623 21 627 3.60E-79 304.3 6596 cellular organisms;Eukaryota;Opisthokonta;Metazoa;Eumetazoa;Bilateria;Protostomia;Spiralia;Lophotrochozoa;Mollusca;Bivalvia;Autobranchia;Heteroconchia;Euheterodonta;Imparidentia;Venerida;Veneroidea;Veneridae;Mercenaria;Mercenaria mercenaria
UdeM00000025081 6596 115.9 UdeM00000025081 XP_045176230.1 36.7 169 104 3 1 167 193 360 5.10E-23 115.9 6596 cellular organisms;Eukaryota;Opisthokonta;Metazoa;Eumetazoa;Bilateria;Protostomia;Spiralia;Lophotrochozoa;Mollusca;Bivalvia;Autobranchia;Heteroconchia;Euheterodonta;Imparidentia;Venerida;Veneroidea;Veneridae;Mercenaria;Mercenaria mercenaria

UdeM00000025082 6596 189.5 UdeM00000025082 XP_045176230.1 31.6 393
241 12 36 419 57 430 9.20E-45 189.5 6596 cellular
organisms;Eukaryota;Opisthokonta;Metazoa;Eumetazoa;Bilateria;Protostomia;
Spiralia;Lophotrochozoa;Mollusca;Bivalvia;Autobranchia;Heteroconchia;Euhe
terodonta;Imparidentia;Venerida;Veneroidea;Veneridae;Mercenaria;Mercenari
a mercenaria
UdeM00000025083 6687 78.2 UdeM00000025083 XP_037797021.1 42.9 112
57 5 16 122 41 150 8.20E-12 78.2 6687 cellular
organisms;Eukaryota;Opisthokonta;Metazoa;Eumetazoa;Bilateria;Protostomia;
Ecdysozoa;Panarthropoda;Arthropoda;Mandibulata;Pancrustacea;Crustacea;Mul
ticrustacea;Malacostraca;Eumalacostraca;Eucarida;Decapoda;Dendrobranchiat
a;Penaeoidea;Penaeidae;Penaeus;Penaeus monodon
UdeM00000025084 441366 75.1 UdeM00000025084 XP_028296543.1 45.1
71 39 0 15 85 768 838 5.00E-11 75.1 441366
cellular
organisms;Eukaryota;Opisthokonta;Metazoa;Eumetazoa;Bilateria;Deuterostomi
a;Chordata;Craniata;Vertebrata;Gnathostomata;Teleostomi;Euteleostomi;Acti
nopterygii;Actinopteri;Neopterygii;Teleostei;Osteoglossocephalai;Clupeoce
phala;Euteleosteomorpha;Neoteleostei;Eurypterygia;Ctenosquamata;Acanthomo
rphata;Euacanthomorphacea;Percomorphaceae;Ovalentaria;Blenniimorphae;Blen
niiformes;Gobiesocoidei;Gobiesocidae;Gobiesocinae;Gouania;Gouania
willdenowi
UdeM00000025087 29159 70.1 UdeM00000025087 XP_034322711.1 33.1 163
102 4 7 168 168 324 3.40E-09 70.1 29159 cellular
organisms;Eukaryota;Opisthokonta;Metazoa;Eumetazoa;Bilateria;Protostomia;
Spiralia;Lophotrochozoa;Mollusca;Bivalvia;Autobranchia;Pteriomorphia;Ostr
eida;Ostreoidea;Ostreidae;Crassostrea;Crassostrea gigas
UdeM00000025088 1232801 97.8 UdeM00000025088 XP_043203304.1 41.1
141 81 2 8 147 597 736 1.90E-17 97.8 1232801
cellular
organisms;Eukaryota;Opisthokonta;Metazoa;Eumetazoa;Bilateria;Protostomia;
Ecdysozoa;Panarthropoda;Arthropoda;Mandibulata;Pancrustacea;Crustacea;Mul
ticrustacea;Hexanauplia;Thecostraca;Cirripedia;Thoracica;Sessilia;Balanid
ae;Amphibalanus;Amphibalanus amphitrite
UdeM00000025090 37653 121.3 UdeM00000025090 XP_014784938.1 52.4 103
48 1 1 102 164 266 9.00E-25 121.3 37653 cellular
organisms;Eukaryota;Opisthokonta;Metazoa;Eumetazoa;Bilateria;Protostomia;
Spiralia;Lophotrochozoa;Mollusca;Cephalopoda;Coleoidea;Octopodiformes;Oct
opoda;Incirrata;Octopodidae;Octopus;Octopus bimaculoides
UdeM00000025091 6596 651 UdeM00000025091 XP_045204508.1 37.6 1185
668 25 57 1220 8 1142 3.00E-183 651 6596 cellular
organisms;Eukaryota;Opisthokonta;Metazoa;Eumetazoa;Bilateria;Protostomia;
Spiralia;Lophotrochozoa;Mollusca;Bivalvia;Autobranchia;Heteroconchia;Euhe
terodonta;Imparidentia;Venerida;Veneroidea;Veneridae;Mercenaria;Mercenari
a mercenaria
UdeM00000025092 6596 129.4 UdeM00000025092 XP_045180427.1 33.3 273
117 4 1 208 1 273 5.70E-27 129.4 6596 cellular
organisms;Eukaryota;Opisthokonta;Metazoa;Eumetazoa;Bilateria;Protostomia;
Spiralia;Lophotrochozoa;Mollusca;Bivalvia;Autobranchia;Heteroconchia;Euhe
terodonta;Imparidentia;Venerida;Veneroidea;Veneridae;Mercenaria;Mercenari
a mercenaria
UdeM00000025093 29159 72.4 UdeM00000025093 XP_034307860.1 64.2 53
19 0 1 53 1 53 3.50E-10 72.4 29159 cellular
organisms;Eukaryota;Opisthokonta;Metazoa;Eumetazoa;Bilateria;Protostomia;

Spiralia;Lophotrochozoa;Mollusca;Bivalvia;Autobranchia;Pteriomorphia;Ostreida;Ostreoidea;Ostreidae;Crassostrea;Crassostrea gigas
UdeM00000025094 6596 188 UdeM00000025094 XP_045167526.1 31.1 409 264 8 60 462 50 446 2.80E-44 188 6596 cellular organisms;Eukaryota;Opisthokonta;Metazoa;Eumetazoa;Bilateria;Protostomia;Spiralia;Lophotrochozoa;Mollusca;Bivalvia;Autobranchia;Heteroconchia;Euheterodonta;Imparidentia;Venerida;Veneroidea;Veneridae;Mercenaria;Mercenaria mercenaria
UdeM00000025095 6526 187.6 UdeM00000025095 XP_013072228.1 29.8 486 223 9 15 387 19 499 3.00E-44 187.6 6526 cellular organisms;Eukaryota;Opisthokonta;Metazoa;Eumetazoa;Bilateria;Protostomia;Spiralia;Lophotrochozoa;Mollusca;Gastropoda;Heterobranchia;Euthyneura;Panpulmonata;Hygrophila;Lymnaeoidea;Planorbidae;Biomphalaria;Biomphalaria glabrata
UdeM00000025096 6596 184.9 UdeM00000025096 XP_045176230.1 29.5 417 264 11 12 417 33 430 2.30E-43 184.9 6596 cellular organisms;Eukaryota;Opisthokonta;Metazoa;Eumetazoa;Bilateria;Protostomia;Spiralia;Lophotrochozoa;Mollusca;Bivalvia;Autobranchia;Heteroconchia;Euheterodonta;Imparidentia;Venerida;Veneroidea;Veneridae;Mercenaria;Mercenaria mercenaria
UdeM00000025097 6596 243.8 UdeM00000025097 XP_045176230.1 29.7 556 316 15 30 548 57 574 5.50E-61 243.8 6596 cellular organisms;Eukaryota;Opisthokonta;Metazoa;Eumetazoa;Bilateria;Protostomia;Spiralia;Lophotrochozoa;Mollusca;Bivalvia;Autobranchia;Heteroconchia;Euheterodonta;Imparidentia;Venerida;Veneroidea;Veneridae;Mercenaria;Mercenaria mercenaria
UdeM00000025098 6596 146 UdeM00000025098 XP_045184527.1 39.8 171 95 5 1 166 111 278 4.60E-32 146 6596 cellular organisms;Eukaryota;Opisthokonta;Metazoa;Eumetazoa;Bilateria;Protostomia;Spiralia;Lophotrochozoa;Mollusca;Bivalvia;Autobranchia;Heteroconchia;Euheterodonta;Imparidentia;Venerida;Veneroidea;Veneridae;Mercenaria;Mercenaria mercenaria
UdeM00000025100 6579 578.9 UdeM00000025100 XP_033742376.1 38.2 997 570 15 22 1010 14 972 1.40E-161 578.9 6579 cellular organisms;Eukaryota;Opisthokonta;Metazoa;Eumetazoa;Bilateria;Protostomia;Spiralia;Lophotrochozoa;Mollusca;Bivalvia;Autobranchia;Pteriomorphia;Pectinida;Pectinoidea;Pectinidae;Pecten;Pecten maximus
UdeM00000025101 6579 578.9 UdeM00000025101 XP_033742376.1 38.2 997 570 15 7 995 14 972 1.40E-161 578.9 6579 cellular organisms;Eukaryota;Opisthokonta;Metazoa;Eumetazoa;Bilateria;Protostomia;Spiralia;Lophotrochozoa;Mollusca;Bivalvia;Autobranchia;Pteriomorphia;Pectinida;Pectinoidea;Pectinidae;Pecten;Pecten maximus
UdeM00000025102 50390 76.3 UdeM00000025102 XP_028127913.1 44.9 78 42 1 1 78 1 77 2.00E-11 76.3 50390 cellular organisms;Eukaryota;Opisthokonta;Metazoa;Eumetazoa;Bilateria;Protostomia;Ecdysozoa;Panarthropoda;Arthropoda;Mandibulata;Pancrustacea;Hexapoda;Insecta;Dicondylia;Pterygota;Neoptera;Endopterygota;Coleoptera;Polyphaga;Cucujiformia;Chrysomeloidea;Chrysomelidae;Galerucinae;Luperini;Diabroticina;Diabroticites;Diabrotica;Diabrotica virgifera;Diabrotica virgifera virgifera
UdeM00000025103 2607531 242.3 UdeM00000025103 XP_029657406.1 56.7 208 90 0 4 211 43 250 5.70E-61 242.3 2607531 cellular organisms;Eukaryota;Opisthokonta;Metazoa;Eumetazoa;Bilateria;Protostomia;

Spiralia;Lophotrochozoa;Mollusca;Cephalopoda;Coleoidea;Octopodiformes;Octopoda;Incirrata;Octopodidae;Octopus;Octopus sinensis
UdeM00000025104 7725 146 UdeM00000025104 XP_039263221.1 62.5 112 42 0 48 159 9 120 5.10E-32 146 7725 cellular organisms;Eukaryota;Opisthokonta;Metazoa;Eumetazoa;Bilateria;Deuterostomia;Chordata;Tunicata;Ascidiacea;Stolidobranchia;Styelidae;Styela;Styela clava
UdeM00000025106 6579 585.5 UdeM00000025106 XP_033742376.1 37.7 1002 569 16 18 1010 15 970 1.50E-163 585.5 6579 cellular organisms;Eukaryota;Opisthokonta;Metazoa;Eumetazoa;Bilateria;Protostomia;Spiralia;Lophotrochozoa;Mollusca;Bivalvia;Autobranchia;Pteriomorphia;Pectinida;Pectinoidea;Pectinidae;Pecten;Pecten maximus
UdeM00000025107 6579 580.1 UdeM00000025107 XP_033742376.1 38.9 978 545 15 32 999 23 957 6.20E-162 580.1 6579 cellular organisms;Eukaryota;Opisthokonta;Metazoa;Eumetazoa;Bilateria;Protostomia;Spiralia;Lophotrochozoa;Mollusca;Bivalvia;Autobranchia;Pteriomorphia;Pectinida;Pectinoidea;Pectinidae;Pecten;Pecten maximus
UdeM00000025108 6579 579.7 UdeM00000025108 XP_033742376.1 38.7 951 543 13 27 960 24 951 7.80E-162 579.7 6579 cellular organisms;Eukaryota;Opisthokonta;Metazoa;Eumetazoa;Bilateria;Protostomia;Spiralia;Lophotrochozoa;Mollusca;Bivalvia;Autobranchia;Pteriomorphia;Pectinida;Pectinoidea;Pectinidae;Pecten;Pecten maximus
UdeM00000025109 6573 184.5 UdeM00000025109 XP_021354853.1 29.6 439 116 3 1 246 355 793 2.50E-43 184.5 6573 cellular organisms;Eukaryota;Opisthokonta;Metazoa;Eumetazoa;Bilateria;Protostomia;Spiralia;Lophotrochozoa;Mollusca;Bivalvia;Autobranchia;Pteriomorphia;Pectinida;Pectinoidea;Pectinidae;Mizuhopecten;Mizuhopecten yessoensis
UdeM00000025110 29159 61.6 UdeM00000025110 XP_034322709.1 42.2 64 37 0 18 81 66 129 1.30E-06 61.6 29159 cellular organisms;Eukaryota;Opisthokonta;Metazoa;Eumetazoa;Bilateria;Protostomia;Spiralia;Lophotrochozoa;Mollusca;Bivalvia;Autobranchia;Pteriomorphia;Ostreida;Ostreoidea;Ostreidae;Crassostrea;Crassostrea gigas
UdeM00000025115 6596 592 UdeM00000025115 XP_045204974.1 37.7 1002 608 10 6 1001 15 1006 1.60E-165 592 6596 cellular organisms;Eukaryota;Opisthokonta;Metazoa;Eumetazoa;Bilateria;Protostomia;Spiralia;Lophotrochozoa;Mollusca;Bivalvia;Autobranchia;Heteroconchia;Euheterodonta;Imparidentia;Venerida;Veneroidea;Veneridae;Mercenaria;Mercenaria mercenaria
UdeM00000025119 6579 544.3 UdeM00000025119 XP_033760558.1 30.5 942 655 0 745 1686 1852 2793 5.60E-151 544.3 6579 cellular organisms;Eukaryota;Opisthokonta;Metazoa;Eumetazoa;Bilateria;Protostomia;Spiralia;Lophotrochozoa;Mollusca;Bivalvia;Autobranchia;Pteriomorphia;Pectinida;Pectinoidea;Pectinidae;Pecten;Pecten maximus
UdeM00000025122 6596 757.3 UdeM00000025122 XP_045205574.1 43.8 1116 559 17 9 1086 21 1106 2.70E-215 757.3 6596 cellular organisms;Eukaryota;Opisthokonta;Metazoa;Eumetazoa;Bilateria;Protostomia;Spiralia;Lophotrochozoa;Mollusca;Bivalvia;Autobranchia;Heteroconchia;Euheterodonta;Imparidentia;Venerida;Veneroidea;Veneridae;Mercenaria;Mercenaria mercenaria
UdeM00000025124 6596 714.9 UdeM00000025124 XP_045205573.1 40.6 1094 604 16 5 1074 41 1112 1.50E-202 714.9 6596 cellular organisms;Eukaryota;Opisthokonta;Metazoa;Eumetazoa;Bilateria;Protostomia;Spiralia;Lophotrochozoa;Mollusca;Bivalvia;Autobranchia;Heteroconchia;Euhe

terodonta;Imparidentia;Venerida;Veneroidea;Veneridae;Mercenaria;Mercenaria mercenaria
UdeM00000025123 6596 728.4 UdeM00000025123 XP_045205573.1 40.5 1122 622 16 8 1105 13 1112 1.40E-206 728.4 6596 cellular organisms;Eukaryota;Opisthokonta;Metazoa;Eumetazoa;Bilateria;Protostomia;Spiralia;Lophotrochozoa;Mollusca;Bivalvia;Autobranchia;Heteroconchia;Euheterodonta;Imparidentia;Venerida;Veneroidea;Veneridae;Mercenaria;Mercenaria mercenaria
UdeM00000025125 6596 768.5 UdeM00000025125 XP_045205574.1 48.4 953 470 12 9 953 11 949 1.20E-218 768.5 6596 cellular organisms;Eukaryota;Opisthokonta;Metazoa;Eumetazoa;Bilateria;Protostomia;Spiralia;Lophotrochozoa;Mollusca;Bivalvia;Autobranchia;Heteroconchia;Euheterodonta;Imparidentia;Venerida;Veneroidea;Veneridae;Mercenaria;Mercenaria mercenaria
UdeM00000025127 6596 96.3 UdeM00000025127 XP_045215739.1 34.5 177 101 4 2 174 328 493 5.30E-17 96.3 6596 cellular organisms;Eukaryota;Opisthokonta;Metazoa;Eumetazoa;Bilateria;Protostomia;Spiralia;Lophotrochozoa;Mollusca;Bivalvia;Autobranchia;Heteroconchia;Euheterodonta;Imparidentia;Venerida;Veneroidea;Veneridae;Mercenaria;Mercenaria mercenaria
UdeM00000025128 6573 206.8 UdeM00000025128 XP_021378643.1 37.4 297 180 4 38 330 939 1233 4.80E-50 206.8 6573 cellular organisms;Eukaryota;Opisthokonta;Metazoa;Eumetazoa;Bilateria;Protostomia;Spiralia;Lophotrochozoa;Mollusca;Bivalvia;Autobranchia;Pteriomorphia;Pectinida;Pectinoidea;Pectinidae;Mizuhopecten;Mizuhopecten yessoensis
UdeM00000025129 29159 352.1 UdeM00000025129 XP_034322969.1 31.9 626 340 14 29 594 210 809 1.40E-93 352.1 29159 cellular organisms;Eukaryota;Opisthokonta;Metazoa;Eumetazoa;Bilateria;Protostomia;Spiralia;Lophotrochozoa;Mollusca;Bivalvia;Autobranchia;Pteriomorphia;Ostreida;Ostreoidea;Ostreidae;Crassostrea;Crassostrea gigas
UdeM00000025131 6596 61.6 UdeM00000025131 XP_045183346.1 46.6 58 27 1 10 63 76 133 1.10E-06 61.6 6596 cellular organisms;Eukaryota;Opisthokonta;Metazoa;Eumetazoa;Bilateria;Protostomia;Spiralia;Lophotrochozoa;Mollusca;Bivalvia;Autobranchia;Heteroconchia;Euheterodonta;Imparidentia;Venerida;Veneroidea;Veneridae;Mercenaria;Mercenaria mercenaria
UdeM00000025132 400727 452.2 UdeM00000025132 XP_025087586.1 64 317 113 1 24 340 5 320 5.80E-124 452.2 400727 cellular organisms;Eukaryota;Opisthokonta;Metazoa;Eumetazoa;Bilateria;Protostomia;Spiralia;Lophotrochozoa;Mollusca;Gastropoda;Caenogastropoda;Architaenioglossa;Ampullarioidea;Ampullariidae;Pomacea;Pomacea canaliculata
UdeM00000025133 42434 58.9 UdeM00000025133 XP_039453121.1 42.5 73 37 2 92 160 494 565 9.10E-06 58.9 42434 cellular organisms;Eukaryota;Opisthokonta;Metazoa;Eumetazoa;Bilateria;Protostomia;Ecdysozoa;Panarthropoda;Arthropoda;Mandibulata;Pancrustacea;Hexapoda;Insecta;Dicondylia;Pterygota;Neoptera;Endopterygota;Diptera;Nematocera;Culicomorpha;Culicoidea;Culicidae;Culicinae;Culicini;Culex;Culex;Culex pipiens complex;Culex pipiens;Culex pipiens pallens
UdeM00000025134 6573 933.7 UdeM00000025134 XP_021346238.1 67.1 812 251 2 1 805 1 803 1.70E-268 933.7 6573 cellular organisms;Eukaryota;Opisthokonta;Metazoa;Eumetazoa;Bilateria;Protostomia;Spiralia;Lophotrochozoa;Mollusca;Bivalvia;Autobranchia;Pteriomorphia;Pectinida;Pectinoidea;Pectinidae;Mizuhopecten;Mizuhopecten yessoensis

UdeM00000025135 6596 137.9 UdeM00000025135 XP_045204103.1 67.8 121
37 2 1 121 3 121 8.70E-30 137.9 6596 cellular
organisms;Eukaryota;Opisthokonta;Metazoa;Eumetazoa;Bilateria;Protostomia;
Spiralia;Lophotrochozoa;Mollusca;Bivalvia;Autobranchia;Heteroconchia;Euheterodonta;Imparidentia;Venerida;Veneroidea;Veneridae;Mercenaria;Mercenaria mercenaria
UdeM00000025136 6565 224.2 UdeM00000025136 XP_022327024.1 76 129
31 0 29 157 35 163 1.20E-55 224.2 6565 cellular
organisms;Eukaryota;Opisthokonta;Metazoa;Eumetazoa;Bilateria;Protostomia;
Spiralia;Lophotrochozoa;Mollusca;Bivalvia;Autobranchia;Pteriomorphia;Ostreida;Ostreoidea;Ostreidae;Crassostrea;Crassostrea virginica
UdeM00000025137 1735272 463.8 UdeM00000025137 XP_041357055.1 69.9
336 100 1 55 390 178 512 2.40E-127 463.8 1735272
cellular
organisms;Eukaryota;Opisthokonta;Metazoa;Eumetazoa;Bilateria;Protostomia;
Spiralia;Lophotrochozoa;Mollusca;Gastropoda;Neomphalina;Neomphaloidea;Peltospiridae;Gigantopelta;Gigantopelta aegis
UdeM00000025138 6573 349.7 UdeM00000025138 XP_021346245.1 31.7 1244
628 36 7 1182 595 1684 1.40E-92 349.7 6573 cellular
organisms;Eukaryota;Opisthokonta;Metazoa;Eumetazoa;Bilateria;Protostomia;
Spiralia;Lophotrochozoa;Mollusca;Bivalvia;Autobranchia;Pteriomorphia;Pectinida;Pectinoidea;Pectinidae;Mizuhopecten;Mizuhopecten yessoensis
UdeM00000025139 7574 280 UdeM00000025139 XP_023932810.1 38.4 440
240 15 15 431 51 482 6.00E-72 280 7574 cellular
organisms;Eukaryota;Opisthokonta;Metazoa;Eumetazoa;Bilateria;Protostomia;
Spiralia;Lophotrochozoa;Brachiopoda;Linguliformea;Lingulata;Lingulida;Linguloidea;Lingulidae;Lingula;Lingula anatina
UdeM00000025140 6573 261.9 UdeM00000025140 XP_021378643.1 45 311
153 6 32 338 562 858 1.10E-66 261.9 6573 cellular
organisms;Eukaryota;Opisthokonta;Metazoa;Eumetazoa;Bilateria;Protostomia;
Spiralia;Lophotrochozoa;Mollusca;Bivalvia;Autobranchia;Pteriomorphia;Pectinida;Pectinoidea;Pectinidae;Mizuhopecten;Mizuhopecten yessoensis
UdeM00000025141 6573 481.1 UdeM00000025141 XP_021378643.1 38.7 693
387 14 32 707 562 1233 2.60E-132 481.1 6573 cellular
organisms;Eukaryota;Opisthokonta;Metazoa;Eumetazoa;Bilateria;Protostomia;
Spiralia;Lophotrochozoa;Mollusca;Bivalvia;Autobranchia;Pteriomorphia;Pectinida;Pectinoidea;Pectinidae;Mizuhopecten;Mizuhopecten yessoensis
UdeM00000025142 6579 253.8 UdeM00000025142 XP_033751112.1 42.8 283
154 5 92 371 84 361 3.40E-64 253.8 6579 cellular
organisms;Eukaryota;Opisthokonta;Metazoa;Eumetazoa;Bilateria;Protostomia;
Spiralia;Lophotrochozoa;Mollusca;Bivalvia;Autobranchia;Pteriomorphia;Pectinida;Pectinoidea;Pectinidae;Pecten;Pecten maximus
UdeM00000025143 6579 250.8 UdeM00000025143 XP_033751112.1 42.3 293
160 5 69 359 76 361 2.80E-63 250.8 6579 cellular
organisms;Eukaryota;Opisthokonta;Metazoa;Eumetazoa;Bilateria;Protostomia;
Spiralia;Lophotrochozoa;Mollusca;Bivalvia;Autobranchia;Pteriomorphia;Pectinida;Pectinoidea;Pectinidae;Pecten;Pecten maximus
UdeM00000025144 6579 255.8 UdeM00000025144 XP_033751112.1 43.4 281
154 4 90 368 84 361 9.00E-65 255.8 6579 cellular
organisms;Eukaryota;Opisthokonta;Metazoa;Eumetazoa;Bilateria;Protostomia;
Spiralia;Lophotrochozoa;Mollusca;Bivalvia;Autobranchia;Pteriomorphia;Pectinida;Pectinoidea;Pectinidae;Pecten;Pecten maximus
UdeM00000025146 6579 265.4 UdeM00000025146 XP_033751112.1 44.5 281
151 4 90 368 84 361 1.10E-67 265.4 6579 cellular

organisms;Eukaryota;Opisthokonta;Metazoa;Eumetazoa;Bilateria;Protostomia;
Spiralia;Lophotrochozoa;Mollusca;Bivalvia;Autobranchia;Pteriomorphia;Pect
inida;Pectinoidea;Pectinidae;Pecten;Pecten maximus
UdeM00000025145 6579 234.6 UdeM00000025145 XP_033751112.1 49.3 207
103 1 90 296 84 288 1.70E-58 234.6 6579 cellular
organisms;Eukaryota;Opisthokonta;Metazoa;Eumetazoa;Bilateria;Protostomia;
Spiralia;Lophotrochozoa;Mollusca;Bivalvia;Autobranchia;Pteriomorphia;Pect
inida;Pectinoidea;Pectinidae;Pecten;Pecten maximus
UdeM00000025147 6596 823.9 UdeM00000025147 XP_045206700.1 61.8 662
240 5 1 657 1 654 1.50E-235 823.9 6596 cellular
organisms;Eukaryota;Opisthokonta;Metazoa;Eumetazoa;Bilateria;Protostomia;
Spiralia;Lophotrochozoa;Mollusca;Bivalvia;Autobranchia;Heteroconchia;Euhe
terodonta;Imparidentia;Venerida;Veneroidea;Veneridae;Mercenaria;Mercenari
a mercenaria
UdeM00000025148 6565 1092 UdeM00000025148 XP_022322186.1 65.6 785
267 3 33 815 28 811 0.00E+00 1092 6565 cellular
organisms;Eukaryota;Opisthokonta;Metazoa;Eumetazoa;Bilateria;Protostomia;
Spiralia;Lophotrochozoa;Mollusca;Bivalvia;Autobranchia;Pteriomorphia;Ostr
eida;Ostreoidea;Ostreidae;Crassostrea;Crassostrea virginica
UdeM00000025150 6596 440.3 UdeM00000025150 XP_045205536.1 43.9 709
290 17 13 639 15 697 4.40E-120 440.3 6596 cellular
organisms;Eukaryota;Opisthokonta;Metazoa;Eumetazoa;Bilateria;Protostomia;
Spiralia;Lophotrochozoa;Mollusca;Bivalvia;Autobranchia;Heteroconchia;Euhe
terodonta;Imparidentia;Venerida;Veneroidea;Veneridae;Mercenaria;Mercenari
a mercenaria
UdeM00000025151 8496 86.3 UdeM00000025151 XP_014461102.1 36.1 122
74 3 4 122 228 348 3.10E-14 86.3 8496 cellular
organisms;Eukaryota;Opisthokonta;Metazoa;Eumetazoa;Bilateria;Deuterostomi
a;Chordata;Craniata;Vertebrata;Gnathostomata;Teleostomi;Euteleostomi;Sarc
opterygii;Dipnotetrapodomorpha;Tetrapoda;Amniota;Sauropsida;Sauria;Archel
osauria;Archosauria;Crocodylia;Alligatoridae;Alligatorinae;Alligator;Alli
gator mississippiensis
UdeM00000025152 6596 392.9 UdeM00000025152 XP_045205536.1 38.3 724
344 22 1 697 753 1400 8.70E-106 392.9 6596 cellular
organisms;Eukaryota;Opisthokonta;Metazoa;Eumetazoa;Bilateria;Protostomia;
Spiralia;Lophotrochozoa;Mollusca;Bivalvia;Autobranchia;Heteroconchia;Euhe
terodonta;Imparidentia;Venerida;Veneroidea;Veneridae;Mercenaria;Mercenari
a mercenaria
UdeM00000025153 6596 150.6 UdeM00000025153 XP_045205536.1 33.9 448
194 21 3 424 1029 1400 4.60E-33 150.6 6596 cellular
organisms;Eukaryota;Opisthokonta;Metazoa;Eumetazoa;Bilateria;Protostomia;
Spiralia;Lophotrochozoa;Mollusca;Bivalvia;Autobranchia;Heteroconchia;Euhe
terodonta;Imparidentia;Venerida;Veneroidea;Veneridae;Mercenaria;Mercenari
a mercenaria
UdeM00000025155 6596 540 UdeM00000025155 XP_045203192.1 58.9 467
192 0 34 500 19 485 3.10E-150 540 6596 cellular
organisms;Eukaryota;Opisthokonta;Metazoa;Eumetazoa;Bilateria;Protostomia;
Spiralia;Lophotrochozoa;Mollusca;Bivalvia;Autobranchia;Heteroconchia;Euhe
terodonta;Imparidentia;Venerida;Veneroidea;Veneridae;Mercenaria;Mercenari
a mercenaria
UdeM00000025156 6573 557 UdeM00000025156 XP_021358726.1 53.7 521
218 7 1 510 1 509 2.90E-155 557 6573 cellular
organisms;Eukaryota;Opisthokonta;Metazoa;Eumetazoa;Bilateria;Protostomia;

Spiralia;Lophotrochozoa;Mollusca;Bivalvia;Autobranchia;Pteriomorphia;Pectinida;Pectinoidea;Pectinidae;Mizuhopecten;Mizuhopecten yessoensis
UdeM00000025157 1735272 164.5 UdeM00000025157 XP_041359832.1 30 444 264 17 9 422 7 433 4.00E-37 164.5 1735272 cellular organisms;Eukaryota;Opisthokonta;Metazoa;Eumetazoa;Bilateria;Protostomia;Spiralia;Lophotrochozoa;Mollusca;Gastropoda;Neomphalina;Neomphaloidea;Peltospiridae;Gigantopelta;Gigantopelta aegis
UdeM00000025158 29159 189.9 UdeM00000025158 XP_034302756.1 62.5 136 51 0 4 139 9 144 2.20E-45 189.9 29159 cellular organisms;Eukaryota;Opisthokonta;Metazoa;Eumetazoa;Bilateria;Protostomia;Spiralia;Lophotrochozoa;Mollusca;Bivalvia;Autobranchia;Pteriomorphia;Ostreida;Ostreoidea;Ostreidae;Crassostrea;Crassostrea gigas
UdeM00000025159 6596 1158.3 UdeM00000025159 XP_045205163.1 46 1466 654 37 27 1399 28 1448 0.00E+00 1158.3 6596 cellular organisms;Eukaryota;Opisthokonta;Metazoa;Eumetazoa;Bilateria;Protostomia;Spiralia;Lophotrochozoa;Mollusca;Bivalvia;Autobranchia;Heteroconchia;Euheterodonta;Imparidentia;Venerida;Veneroidea;Veneridae;Mercenaria;Mercenaria mercenaria
UdeM00000025160 6596 1060.1 UdeM00000025160 XP_045205832.1 79.5 658 122 5 1 645 1 658 1.20E-306 1060.1 6596 cellular organisms;Eukaryota;Opisthokonta;Metazoa;Eumetazoa;Bilateria;Protostomia;Spiralia;Lophotrochozoa;Mollusca;Bivalvia;Autobranchia;Heteroconchia;Euheterodonta;Imparidentia;Venerida;Veneroidea;Veneridae;Mercenaria;Mercenaria mercenaria
UdeM00000025161 6596 5430.1 UdeM00000025161 XP_045204551.1 57.4 5222 1883 92 14 5064 6 5056 0.00E+00 5430.1 6596 cellular organisms;Eukaryota;Opisthokonta;Metazoa;Eumetazoa;Bilateria;Protostomia;Spiralia;Lophotrochozoa;Mollusca;Bivalvia;Autobranchia;Heteroconchia;Euheterodonta;Imparidentia;Venerida;Veneroidea;Veneridae;Mercenaria;Mercenaria mercenaria
UdeM00000025162 6596 526.2 UdeM00000025162 XP_045204541.1 78.7 497 96 3 1 489 212 706 4.50E-146 526.2 6596 cellular organisms;Eukaryota;Opisthokonta;Metazoa;Eumetazoa;Bilateria;Protostomia;Spiralia;Lophotrochozoa;Mollusca;Bivalvia;Autobranchia;Heteroconchia;Euheterodonta;Imparidentia;Venerida;Veneroidea;Veneridae;Mercenaria;Mercenaria mercenaria
UdeM00000025163 6596 685.6 UdeM00000025163 XP_045204541.1 79.2 600 101 4 1 578 109 706 5.30E-194 685.6 6596 cellular organisms;Eukaryota;Opisthokonta;Metazoa;Eumetazoa;Bilateria;Protostomia;Spiralia;Lophotrochozoa;Mollusca;Bivalvia;Autobranchia;Heteroconchia;Euheterodonta;Imparidentia;Venerida;Veneroidea;Veneridae;Mercenaria;Mercenaria mercenaria
UdeM00000025164 303518 129 UdeM00000025164 XP_005751641.1 49.3 142 69 2 3 141 291 432 4.70E-27 129 303518 cellular organisms;Eukaryota;Opisthokonta;Metazoa;Eumetazoa;Bilateria;Deuterostomia;Chordata;Craniata;Vertebrata;Gnathostomata;Teleostomi;Euteleostomi;Actinopterygii;Actinopteri;Neopterygii;Teleostei;Osteoglossocephalai;Clupeocephala;Euteleosteomorpha;Neoteleostei;Eurypterygia;Ctenosquamata;Acanthomorphata;Euacanthomorphacea;Percomorphaceae;Ovalentaria;Cichlomorphae;Cichl

iformes;Cichlidae;African
cichlids;Pseudocrenilabrinae;Haplochromini;Pundamilia;Pundamilia nyererei
UdeM00000025165 7574 137.1 UdeM00000025165 XP_013384614.1 71.7 92
23 1 1 89 1 92 1.50E-29 137.1 7574 cellular
organisms;Eukaryota;Opisthokonta;Metazoa;Eumetazoa;Bilateria;Protostomia;
Spiralia;Lophotrochozoa;Brachiopoda;Linguliformea;Lingulata;Lingulida;Lin
guloidea;Lingulidae;Lingula;Lingula anatina
UdeM00000025166 400727 115.2 UdeM00000025166 XP_025086911.1 63.8
80 26 1 5 84 9 85 4.30E-23 115.2 400727
cellular
organisms;Eukaryota;Opisthokonta;Metazoa;Eumetazoa;Bilateria;Protostomia;
Spiralia;Lophotrochozoa;Mollusca;Gastropoda;Caenogastropoda;Architaeniogl
ossa;Ampullarioidea;Ampullariidae;Pomacea;Pomacea canaliculata
UdeM00000025167 6596 692.6 UdeM00000025167 XP_045204430.1 77.5 512
97 3 992 1500 487 983 1.10E-195 692.6 6596 cellular
organisms;Eukaryota;Opisthokonta;Metazoa;Eumetazoa;Bilateria;Protostomia;
Spiralia;Lophotrochozoa;Mollusca;Bivalvia;Autobranchia;Heteroconchia;Euhe
terodonta;Imparidentia;Venerida;Veneroidea;Veneridae;Mercenaria;Mercenari
a mercenaria
UdeM00000025168 29159 718 UdeM00000025168 XP_011448163.2 84.8 461
65 1 1 461 1 456 7.70E-204 718 29159 cellular
organisms;Eukaryota;Opisthokonta;Metazoa;Eumetazoa;Bilateria;Protostomia;
Spiralia;Lophotrochozoa;Mollusca;Bivalvia;Autobranchia;Pteriomorphia;Ostr
eida;Ostreoidea;Ostreidae;Crassostrea;Crassostrea gigas
UdeM00000025169 151771 111.3 UdeM00000025169 XP_028408207.1 39.5
157 94 1 84 240 138 293 1.70E-21 111.3 151771
cellular
organisms;Eukaryota;Opisthokonta;Metazoa;Eumetazoa;Cnidaria;Anthozoa;Octo
corallia;Alcyonacea;Alcyoniina;Nephtheidae;Dendronephthya;Dendronephthya
gigantea
UdeM00000025170 6596 140.2 UdeM00000025170 XP_045204433.1 44 159
84 3 1 155 1 158 2.40E-30 140.2 6596 cellular
organisms;Eukaryota;Opisthokonta;Metazoa;Eumetazoa;Bilateria;Protostomia;
Spiralia;Lophotrochozoa;Mollusca;Bivalvia;Autobranchia;Heteroconchia;Euhe
terodonta;Imparidentia;Venerida;Veneroidea;Veneridae;Mercenaria;Mercenari
a mercenaria
UdeM00000025171 29159 520.4 UdeM00000025171 XP_011456694.2 68.2 381
118 1 1 381 1 378 1.90E-144 520.4 29159 cellular
organisms;Eukaryota;Opisthokonta;Metazoa;Eumetazoa;Bilateria;Protostomia;
Spiralia;Lophotrochozoa;Mollusca;Bivalvia;Autobranchia;Pteriomorphia;Ostr
eida;Ostreoidea;Ostreidae;Crassostrea;Crassostrea gigas
UdeM00000025172 6526 73.9 UdeM00000025172 XP_013095497.1 38.3 94
56 2 10 102 30 122 1.50E-10 73.9 6526 cellular
organisms;Eukaryota;Opisthokonta;Metazoa;Eumetazoa;Bilateria;Protostomia;
Spiralia;Lophotrochozoa;Mollusca;Gastropoda;Heterobranchia;Euthyneura;Pan
pulmonata;Hygrophila;Lymnaeoidea;Planorbidae;Biomphalaria;Biomphalaria
glabrata
UdeM00000025173 1735272 604.7 UdeM00000025173 XP_041358113.1 60
493 179 5 74 548 17 509 1.10E-169 604.7 1735272
cellular
organisms;Eukaryota;Opisthokonta;Metazoa;Eumetazoa;Bilateria;Protostomia;
Spiralia;Lophotrochozoa;Mollusca;Gastropoda;Neomphalina;Neomphaloidea;Pel
tospiridae;Gigantopelta;Gigantopelta aegis

UdeM00000025176	6596	390.6 UdeM00000025176	XP_045203973.1	42.8	479
	269	4	27	503	39	514	3.10E-105	390.6 6596	cellular organisms;Eukaryota;Opisthokonta;Metazoa;Eumetazoa;Bilateria;Protostomia;Spiralia;Lophotrochozoa;Mollusca;Bivalvia;Autobranchia;Heteroconchia;Euheterodonta;Imparidentia;Venerida;Veneroidea;Veneridae;Mercenaria;Mercenaria mercenaria
UdeM00000025177	6596	145.6 UdeM00000025177	XP_045203974.1	65	100
	35	0	10	109	36	135	3.70E-32	145.6 6596	cellular organisms;Eukaryota;Opisthokonta;Metazoa;Eumetazoa;Bilateria;Protostomia;Spiralia;Lophotrochozoa;Mollusca;Bivalvia;Autobranchia;Heteroconchia;Euheterodonta;Imparidentia;Venerida;Veneroidea;Veneridae;Mercenaria;Mercenaria mercenaria
UdeM00000025178	6596	285	UdeM00000025178	XP_045203961.1	71.5	193
	55	0	2	194	6	198	7.00E-74	285	6596	cellular organisms;Eukaryota;Opisthokonta;Metazoa;Eumetazoa;Bilateria;Protostomia;Spiralia;Lophotrochozoa;Mollusca;Bivalvia;Autobranchia;Heteroconchia;Euheterodonta;Imparidentia;Venerida;Veneroidea;Veneridae;Mercenaria;Mercenaria mercenaria
UdeM00000025179	6573	1024.6	UdeM00000025179	XP_021375935.1	78.9
	587	121	1	43	626	25	611	5.20E-296	1024.6	6573
	cellular organisms;Eukaryota;Opisthokonta;Metazoa;Eumetazoa;Bilateria;Protostomia;Spiralia;Lophotrochozoa;Mollusca;Bivalvia;Autobranchia;Pteriomorphia;Pectinida;Pectinoidea;Pectinidae;Mizuhopecten;Mizuhopecten yessoensis
UdeM00000025180	6596	482.6 UdeM00000025180	XP_045205224.1	57.6	422
	171	3	8	421	17	438	5.00E-133	482.6 6596	cellular organisms;Eukaryota;Opisthokonta;Metazoa;Eumetazoa;Bilateria;Protostomia;Spiralia;Lophotrochozoa;Mollusca;Bivalvia;Autobranchia;Heteroconchia;Euheterodonta;Imparidentia;Venerida;Veneroidea;Veneridae;Mercenaria;Mercenaria mercenaria
UdeM00000025181	6579	339.3 UdeM00000025181	XP_033743800.1	53.8	329
	152	0	2	330	6	334	5.30E-90	339.3 6579	cellular organisms;Eukaryota;Opisthokonta;Metazoa;Eumetazoa;Bilateria;Protostomia;Spiralia;Lophotrochozoa;Mollusca;Bivalvia;Autobranchia;Pteriomorphia;Pectinida;Pectinoidea;Pectinidae;Pecten;Pecten maximus
UdeM00000025182	6596	857.4 UdeM00000025182	XP_045204610.1	71.9	563
	143	3	17	564	59	621	1.10E-245	857.4 6596	cellular organisms;Eukaryota;Opisthokonta;Metazoa;Eumetazoa;Bilateria;Protostomia;Spiralia;Lophotrochozoa;Mollusca;Bivalvia;Autobranchia;Heteroconchia;Euheterodonta;Imparidentia;Venerida;Veneroidea;Veneridae;Mercenaria;Mercenaria mercenaria
UdeM00000025183	37653 94	UdeM00000025183	XP_014781215.1	62.7	67
	25	0	14	80	31	97	9.80E-17	94	37653 cellular organisms;Eukaryota;Opisthokonta;Metazoa;Eumetazoa;Bilateria;Protostomia;Spiralia;Lophotrochozoa;Mollusca;Cephalopoda;Coleoidea;Octopodiformes;Octopoda;Incirrata;Octopodidae;Octopus;Octopus bimaculoides
UdeM00000025184	225164	623.6 UdeM00000025184	XP_009047116.1	73.1
	417	111	1	12	427	204	620	1.90E-175	623.6 225164
	cellular organisms;Eukaryota;Opisthokonta;Metazoa;Eumetazoa;Bilateria;Protostomia;Spiralia;Lophotrochozoa;Mollusca;Gastropoda;Patellogastropoda;Lottioidea;Lottiidae;Lottia;Lottia gigantea
UdeM00000025185	1735272	272.7 UdeM00000025185	XP_041356928.1	62.3
	223	70	4	44	264	29	239	4.90E-70	272.7 1735272

cellular organisms;Eukaryota;Opisthokonta;Metazoa;Eumetazoa;Bilateria;Protostomia;Spiralia;Lophotrochozoa;Mollusca;Gastropoda;Neomphalina;Neomphaloidea;Peltospiridae;Gigantopelta;Gigantopelta aegis
UdeM00000025186 29159 1017.3 UdeM00000025186 XP_011423016.2 79.9 616 124 0 792 1407 623 1238 1.90E-293 1017.3 29159 cellular organisms;Eukaryota;Opisthokonta;Metazoa;Eumetazoa;Bilateria;Protostomia;Spiralia;Lophotrochozoa;Mollusca;Bivalvia;Autobranchia;Pteriomorphia;Ostreida;Ostreoidea;Ostreidae;Crassostrea;Crassostrea gigas
UdeM00000025189 6596 124.4 UdeM00000025189 XP_045202738.1 45.7 164 74 4 22 181 19 171 2.20E-25 124.4 6596 cellular organisms;Eukaryota;Opisthokonta;Metazoa;Eumetazoa;Bilateria;Protostomia;Spiralia;Lophotrochozoa;Mollusca;Bivalvia;Autobranchia;Heteroconchia;Euheterodonta;Imparidentia;Venerida;Veneroidea;Veneridae;Mercenaria;Mercenaria mercenaria
UdeM00000025191 6596 238.8 UdeM00000025191 XP_045202740.1 59.5 205 71 4 13 214 10 205 6.40E-60 238.8 6596 cellular organisms;Eukaryota;Opisthokonta;Metazoa;Eumetazoa;Bilateria;Protostomia;Spiralia;Lophotrochozoa;Mollusca;Bivalvia;Autobranchia;Heteroconchia;Euheterodonta;Imparidentia;Venerida;Veneroidea;Veneridae;Mercenaria;Mercenaria mercenaria
UdeM00000025194 6596 394.4 UdeM00000025194 XP_045204909.1 47 428 210 5 3 425 10 425 2.00E-106 394.4 6596 cellular organisms;Eukaryota;Opisthokonta;Metazoa;Eumetazoa;Bilateria;Protostomia;Spiralia;Lophotrochozoa;Mollusca;Bivalvia;Autobranchia;Heteroconchia;Euheterodonta;Imparidentia;Venerida;Veneroidea;Veneridae;Mercenaria;Mercenaria mercenaria
UdeM00000025195 6573 513.5 UdeM00000025195 XP_021339020.1 67 424 131 1 1 424 1 415 2.70E-142 513.5 6573 cellular organisms;Eukaryota;Opisthokonta;Metazoa;Eumetazoa;Bilateria;Protostomia;Spiralia;Lophotrochozoa;Mollusca;Bivalvia;Autobranchia;Pteriomorphia;Pectinida;Pectinoidea;Pectinidae;Mizuhopecten;Mizuhopecten yessoensis
UdeM00000025196 6596 698 UdeM00000025196 XP_045203692.1 64.1 577 196 3 1 577 1 566 1.00E-197 698 6596 cellular organisms;Eukaryota;Opisthokonta;Metazoa;Eumetazoa;Bilateria;Protostomia;Spiralia;Lophotrochozoa;Mollusca;Bivalvia;Autobranchia;Heteroconchia;Euheterodonta;Imparidentia;Venerida;Veneroidea;Veneridae;Mercenaria;Mercenaria mercenaria
UdeM00000025197 6596 195.3 UdeM00000025197 XP_045203692.1 55.8 163 71 1 28 190 405 566 7.20E-47 195.3 6596 cellular organisms;Eukaryota;Opisthokonta;Metazoa;Eumetazoa;Bilateria;Protostomia;Spiralia;Lophotrochozoa;Mollusca;Bivalvia;Autobranchia;Heteroconchia;Euheterodonta;Imparidentia;Venerida;Veneroidea;Veneridae;Mercenaria;Mercenaria mercenaria
UdeM00000025198 6579 95.9 UdeM00000025198 XP_033743710.1 83.3 48 8 0 9 56 11 58 4.90E-17 95.9 6579 cellular organisms;Eukaryota;Opisthokonta;Metazoa;Eumetazoa;Bilateria;Protostomia;Spiralia;Lophotrochozoa;Mollusca;Bivalvia;Autobranchia;Pteriomorphia;Pectinida;Pectinoidea;Pectinidae;Pecten;Pecten maximus
UdeM00000025199 77115 118.6 UdeM00000025199 XP_038140435.1 22.2 535 316 17 332 775 12 537 4.50E-23 118.6 77115 cellular organisms;Eukaryota;Opisthokonta;Metazoa;Eumetazoa;Bilateria;Deuterostomia;Chordata;Craniata;Vertebrata;Gnathostomata;Teleostomi;Euteleostomi;Acti

nopterygii;Actinopteri;Neopterygii;Teleostei;Osteoglossocephalai;Clupeocephala;Euteleosteomorpha;Neoteleostei;Eurypterygia;Ctenosquamata;Acanthomorphata;Euacanthomorphacea;Percomorphaceae;Ovalentaria;Atherinomorphae;Cyprinodontiformes;Cyprinodontoidei;Cyprinodontidae;Cyprinodontinae;Cyprinodontini;Cyprinodon;Cyprinodon tularosa
UdeM00000025200 77115 86.7 UdeM00000025200 XP_038140435.1 29.4 204 127 8 91 284 341 537 6.70E-14 86.7 77115 cellular organisms;Eukaryota;Opisthokonta;Metazoa;Eumetazoa;Bilateria;Deuterostomia;Chordata;Craniata;Vertebrata;Gnathostomata;Teleostomi;Euteleostomi;Actinopterygii;Actinopteri;Neopterygii;Teleostei;Osteoglossocephalai;Clupeocephala;Euteleosteomorpha;Neoteleostei;Eurypterygia;Ctenosquamata;Acanthomorphata;Euacanthomorphacea;Percomorphaceae;Ovalentaria;Atherinomorphae;Cyprinodontiformes;Cyprinodontoidei;Cyprinodontidae;Cyprinodontinae;Cyprinodontini;Cyprinodon;Cyprinodon tularosa
UdeM00000025202 6596 604 UdeM00000025202 XP_045206270.1 41.6 844 415 17 58 864 27 829 3.50E-169 604 6596 cellular organisms;Eukaryota;Opisthokonta;Metazoa;Eumetazoa;Bilateria;Protostomia;Spiralia;Lophotrochozoa;Mollusca;Bivalvia;Autobranchia;Heteroconchia;Euheterodonta;Imparidentia;Venerida;Veneroidea;Veneridae;Mercenaria;Mercenaria mercenaria
UdeM00000025205 6565 842.4 UdeM00000025205 XP_022323119.1 56.8 701 267 9 5 682 15 702 4.00E-241 842.4 6565 cellular organisms;Eukaryota;Opisthokonta;Metazoa;Eumetazoa;Bilateria;Protostomia;Spiralia;Lophotrochozoa;Mollusca;Bivalvia;Autobranchia;Pteriomorphia;Ostreida;Ostreoidea;Ostreidae;Crassostrea;Crassostrea virginica
UdeM00000025206 6565 1037.3 UdeM00000025206 XP_022323119.1 70.1 712 200 5 3 713 4 703 8.80E-300 1037.3 6565 cellular organisms;Eukaryota;Opisthokonta;Metazoa;Eumetazoa;Bilateria;Protostomia;Spiralia;Lophotrochozoa;Mollusca;Bivalvia;Autobranchia;Pteriomorphia;Ostreida;Ostreoidea;Ostreidae;Crassostrea;Crassostrea virginica
UdeM00000025207 6565 1042 UdeM00000025207 XP_022323119.1 69.5 727 194 5 3 728 4 703 3.70E-301 1042 6565 cellular organisms;Eukaryota;Opisthokonta;Metazoa;Eumetazoa;Bilateria;Protostomia;Spiralia;Lophotrochozoa;Mollusca;Bivalvia;Autobranchia;Pteriomorphia;Ostreida;Ostreoidea;Ostreidae;Crassostrea;Crassostrea virginica
UdeM00000025208 6573 1947.6 UdeM00000025208 XP_021348278.1 58.9 2043 658 28 1 1993 1 1911 0.00E+00 1947.6 6573 cellular organisms;Eukaryota;Opisthokonta;Metazoa;Eumetazoa;Bilateria;Protostomia;Spiralia;Lophotrochozoa;Mollusca;Bivalvia;Autobranchia;Pteriomorphia;Pectinida;Pectinoidea;Pectinidae;Mizuhopecten;Mizuhopecten yessoensis
UdeM00000025209 400727 312.8 UdeM00000025209 XP_025088528.1 60.3 297 105 8 1 289 1 292 5.20E-82 312.8 400727 cellular organisms;Eukaryota;Opisthokonta;Metazoa;Eumetazoa;Bilateria;Protostomia;Spiralia;Lophotrochozoa;Mollusca;Gastropoda;Caenogastropoda;Architaenioglossa;Ampullarioidea;Ampullariidae;Pomacea;Pomacea canaliculata
UdeM00000025224 6500 408.3 UdeM00000025224 XP_005103080.1 58.1 375 146 4 2 365 110 484 1.30E-110 408.3 6500 cellular organisms;Eukaryota;Opisthokonta;Metazoa;Eumetazoa;Bilateria;Protostomia;Spiralia;Lophotrochozoa;Mollusca;Gastropoda;Heterobranchia;Euthyneura;Tectipleura;Aplysiida;Aplysioidea;Aplysiidae;Aplysia;Aplysia californica

UdeM00000025225	6565	360.5	UdeM00000025225	XP_022324860.1	49.4	401
188	4	54	449	314	704	3.20E-96	360.5	6565	cellular organisms;Eukaryota;Opisthokonta;Metazoa;Eumetazoa;Bilateria;Protostomia;Spiralia;Lophotrochozoa;Mollusca;Bivalvia;Autobranchia;Pteriomorphia;Ostreida;Ostreoidea;Ostreidae;Crassostrea;Crassostrea virginica
UdeM00000025227	6579	308.1	UdeM00000025227	XP_033743895.1	50.9	383
148	10	1	376	1	350	1.50E-80	308.1	6579	cellular organisms;Eukaryota;Opisthokonta;Metazoa;Eumetazoa;Bilateria;Protostomia;Spiralia;Lophotrochozoa;Mollusca;Bivalvia;Autobranchia;Pteriomorphia;Pectinida;Pectinoidea;Pectinidae;Pecten;Pecten maximus
UdeM00000025228	6579	99.4	UdeM00000025228	XP_033743219.1	46.2	93
49	1	2	93	140	232	2.60E-18	99.4	6579	cellular organisms;Eukaryota;Opisthokonta;Metazoa;Eumetazoa;Bilateria;Protostomia;Spiralia;Lophotrochozoa;Mollusca;Bivalvia;Autobranchia;Pteriomorphia;Pectinida;Pectinoidea;Pectinidae;Pecten;Pecten maximus
UdeM00000025229	6573	342.4	UdeM00000025229	XP_021361341.1	46.2	539
208	17	56	559	92	583	1.10E-90	342.4	6573	cellular organisms;Eukaryota;Opisthokonta;Metazoa;Eumetazoa;Bilateria;Protostomia;Spiralia;Lophotrochozoa;Mollusca;Bivalvia;Autobranchia;Pteriomorphia;Pectinida;Pectinoidea;Pectinidae;Mizuhopecten;Mizuhopecten yessoensis
UdeM00000025230	6573	455.3	UdeM00000025230	XP_021360284.1	57.3	393
163	4	260	651	50	438	1.30E-124	455.3	6573	cellular organisms;Eukaryota;Opisthokonta;Metazoa;Eumetazoa;Bilateria;Protostomia;Spiralia;Lophotrochozoa;Mollusca;Bivalvia;Autobranchia;Pteriomorphia;Pectinida;Pectinoidea;Pectinidae;Mizuhopecten;Mizuhopecten yessoensis
UdeM00000025231	2607531	1501.9	UdeM00000025231	XP_029638531.1
57.7	1316	465	13	23	1322	469	1708	0.00E+00	1501.9
2607531	cellular organisms;Eukaryota;Opisthokonta;Metazoa;Eumetazoa;Bilateria;Protostomia;Spiralia;Lophotrochozoa;Mollusca;Cephalopoda;Coleoidea;Octopodiformes;Octopoda;Incirrata;Octopodidae;Octopus;Octopus sinensis
UdeM00000025232	6596	550.8	UdeM00000025232	XP_045204753.1	63	414
149	3	3	413	1437	1849	1.50E-153	550.8	6596	cellular organisms;Eukaryota;Opisthokonta;Metazoa;Eumetazoa;Bilateria;Protostomia;Spiralia;Lophotrochozoa;Mollusca;Bivalvia;Autobranchia;Heteroconchia;Euheterodonta;Imparidentia;Venerida;Veneroidea;Veneridae;Mercenaria;Mercenaria mercenaria
UdeM00000025233	6579	209.1	UdeM00000025233	XP_033744344.1	54.4	160
73	0	2	161	149	308	4.10E-51	209.1	6579	cellular organisms;Eukaryota;Opisthokonta;Metazoa;Eumetazoa;Bilateria;Protostomia;Spiralia;Lophotrochozoa;Mollusca;Bivalvia;Autobranchia;Pteriomorphia;Pectinida;Pectinoidea;Pectinidae;Pecten;Pecten maximus
UdeM00000025234	6579	107.8	UdeM00000025234	XP_033744344.1	45.5	145
65	6	3	134	1	144	1.10E-20	107.8	6579	cellular organisms;Eukaryota;Opisthokonta;Metazoa;Eumetazoa;Bilateria;Protostomia;Spiralia;Lophotrochozoa;Mollusca;Bivalvia;Autobranchia;Pteriomorphia;Pectinida;Pectinoidea;Pectinidae;Pecten;Pecten maximus
UdeM00000025235	6596	357.5	UdeM00000025235	XP_045206384.1	50.3	340
123	4	14	352	36	330	2.00E-95	357.5	6596	cellular organisms;Eukaryota;Opisthokonta;Metazoa;Eumetazoa;Bilateria;Protostomia;Spiralia;Lophotrochozoa;Mollusca;Bivalvia;Autobranchia;Heteroconchia;Euheterodonta;Imparidentia;Venerida;Veneroidea;Veneridae;Mercenaria;Mercenaria mercenaria

UdeM00000025236 6573 394.8 UdeM00000025236 XP_021352157.1 45.1 812
364 15 1 763 1 779 3.90E-106 394.8 6573 cellular organisms;Eukaryota;Opisthokonta;Metazoa;Eumetazoa;Bilateria;Protostomia;Spiralia;Lophotrochozoa;Mollusca;Bivalvia;Autobranchia;Pteriomorphia;Pectinida;Pectinoidea;Pectinidae;Mizuhopecten;Mizuhopecten yessoensis
UdeM00000025237 29159 184.9 UdeM00000025237 XP_011429648.2 68.9 122
33 1 65 186 961 1077 9.80E-44 184.9 29159 cellular organisms;Eukaryota;Opisthokonta;Metazoa;Eumetazoa;Bilateria;Protostomia;Spiralia;Lophotrochozoa;Mollusca;Bivalvia;Autobranchia;Pteriomorphia;Ostreida;Ostreoidea;Ostreidae;Crassostrea;Crassostrea gigas
UdeM00000025238 6596 117.9 UdeM00000025238 XP_045203996.1 61.9 97
36 1 1098 1194 420 515 9.10E-23 117.9 6596 cellular organisms;Eukaryota;Opisthokonta;Metazoa;Eumetazoa;Bilateria;Protostomia;Spiralia;Lophotrochozoa;Mollusca;Bivalvia;Autobranchia;Heteroconchia;Euheterodonta;Imparidentia;Venerida;Veneroidea;Veneridae;Mercenaria;Mercenaria mercenaria
UdeM00000025239 6596 834.7 UdeM00000025239 XP_045204006.1 59.4 756
260 15 1 754 56 766 9.20E-239 834.7 6596 cellular organisms;Eukaryota;Opisthokonta;Metazoa;Eumetazoa;Bilateria;Protostomia;Spiralia;Lophotrochozoa;Mollusca;Bivalvia;Autobranchia;Heteroconchia;Euheterodonta;Imparidentia;Venerida;Veneroidea;Veneridae;Mercenaria;Mercenaria mercenaria
UdeM00000025240 6596 615.9 UdeM00000025240 XP_045204007.1 61.1 537
195 6 2 532 161 689 4.70E-173 615.9 6596 cellular organisms;Eukaryota;Opisthokonta;Metazoa;Eumetazoa;Bilateria;Protostomia;Spiralia;Lophotrochozoa;Mollusca;Bivalvia;Autobranchia;Heteroconchia;Euheterodonta;Imparidentia;Venerida;Veneroidea;Veneridae;Mercenaria;Mercenaria mercenaria
UdeM00000025241 6596 644 UdeM00000025241 XP_045205866.1 51.5 681
293 11 4 670 7 664 2.10E-181 644 6596 cellular organisms;Eukaryota;Opisthokonta;Metazoa;Eumetazoa;Bilateria;Protostomia;Spiralia;Lophotrochozoa;Mollusca;Bivalvia;Autobranchia;Heteroconchia;Euheterodonta;Imparidentia;Venerida;Veneroidea;Veneridae;Mercenaria;Mercenaria mercenaria
UdeM00000025242 6596 365.2 UdeM00000025242 XP_045206603.1 43.7 428
236 4 2 429 11 433 1.30E-97 365.2 6596 cellular organisms;Eukaryota;Opisthokonta;Metazoa;Eumetazoa;Bilateria;Protostomia;Spiralia;Lophotrochozoa;Mollusca;Bivalvia;Autobranchia;Heteroconchia;Euheterodonta;Imparidentia;Venerida;Veneroidea;Veneridae;Mercenaria;Mercenaria mercenaria
UdeM00000025243 6596 371.3 UdeM00000025243 XP_045206603.1 45.3 422
225 4 4 424 13 429 1.70E-99 371.3 6596 cellular organisms;Eukaryota;Opisthokonta;Metazoa;Eumetazoa;Bilateria;Protostomia;Spiralia;Lophotrochozoa;Mollusca;Bivalvia;Autobranchia;Heteroconchia;Euheterodonta;Imparidentia;Venerida;Veneroidea;Veneridae;Mercenaria;Mercenaria mercenaria
UdeM00000025244 6596 741.5 UdeM00000025244 XP_045203583.1 47 879
397 18 34 878 38 881 1.20E-210 741.5 6596 cellular organisms;Eukaryota;Opisthokonta;Metazoa;Eumetazoa;Bilateria;Protostomia;Spiralia;Lophotrochozoa;Mollusca;Bivalvia;Autobranchia;Heteroconchia;Euheterodonta;Imparidentia;Venerida;Veneroidea;Veneridae;Mercenaria;Mercenaria mercenaria
UdeM00000025245 1937702 68.9 UdeM00000025245 XP_043053154.1 26.9
171 115 4 292 455 210 377 1.90E-08 68.9 1937702

cellular
organisms;Eukaryota;Opisthokonta;Fungi;Dikarya;Ascomycota;saccharomyceta;Saccharomycotina;Saccharomycetes;Saccharomycetales;Pichiaceae;Ogataea;Ogataea haglerorum
UdeM00000025246 6596 84.7 UdeM00000025246 XP_045204036.1 48.8 86
37 2 28 109 135 217 8.40E-14 84.7 6596 cellular
organisms;Eukaryota;Opisthokonta;Metazoa;Eumetazoa;Bilateria;Protostomia;Spiralia;Lophotrochozoa;Mollusca;Bivalvia;Autobranchia;Heteroconchia;Euheterodonta;Imparidentia;Venerida;Veneroidea;Veneridae;Mercenaria;Mercenaria mercenaria
UdeM00000025247 2652724 114.4 UdeM00000025247 XP_020900102.1 32.5
209 100 6 27 197 45 250 1.80E-22 114.4 2652724
cellular
organisms;Eukaryota;Opisthokonta;Metazoa;Eumetazoa;Cnidaria;Anthozoa;Hexacorallia;Actiniaria;Aiptasiidae;Exaiptasia;Exaiptasia diaphana
UdeM00000025248 6596 150.2 UdeM00000025248 XP_045204036.1 57 114
48 1 31 144 105 217 2.10E-33 150.2 6596 cellular
organisms;Eukaryota;Opisthokonta;Metazoa;Eumetazoa;Bilateria;Protostomia;Spiralia;Lophotrochozoa;Mollusca;Bivalvia;Autobranchia;Heteroconchia;Euheterodonta;Imparidentia;Venerida;Veneroidea;Veneridae;Mercenaria;Mercenaria mercenaria
UdeM00000025249 1735272 160.6 UdeM00000025249 XP_041367532.1 39.9
258 142 3 87 338 30 280 3.50E-36 160.6 1735272
cellular
organisms;Eukaryota;Opisthokonta;Metazoa;Eumetazoa;Bilateria;Protostomia;Spiralia;Lophotrochozoa;Mollusca;Gastropoda;Neomphalina;Neomphaloidea;Peltospiridae;Gigantopelta;Gigantopelta aegis
UdeM00000025250 1735272 183.7 UdeM00000025250 XP_041354040.1 45.3
212 101 1 109 305 150 361 3.60E-43 183.7 1735272
cellular
organisms;Eukaryota;Opisthokonta;Metazoa;Eumetazoa;Bilateria;Protostomia;Spiralia;Lophotrochozoa;Mollusca;Gastropoda;Neomphalina;Neomphaloidea;Peltospiridae;Gigantopelta;Gigantopelta aegis
UdeM00000025251 1735272 186.8 UdeM00000025251 XP_041354040.1 45.3
214 102 1 95 293 148 361 4.20E-44 186.8 1735272
cellular
organisms;Eukaryota;Opisthokonta;Metazoa;Eumetazoa;Bilateria;Protostomia;Spiralia;Lophotrochozoa;Mollusca;Gastropoda;Neomphalina;Neomphaloidea;Peltospiridae;Gigantopelta;Gigantopelta aegis
UdeM00000025252 6596 114.8 UdeM00000025252 XP_045156958.1 39.1 197
107 4 9 203 7 192 1.30E-22 114.8 6596 cellular
organisms;Eukaryota;Opisthokonta;Metazoa;Eumetazoa;Bilateria;Protostomia;Spiralia;Lophotrochozoa;Mollusca;Bivalvia;Autobranchia;Heteroconchia;Euheterodonta;Imparidentia;Venerida;Veneroidea;Veneridae;Mercenaria;Mercenaria mercenaria
UdeM00000025254 6565 357.1 UdeM00000025254 XP_022327050.1 55.6 306
136 0 1 306 1 306 2.40E-95 357.1 6565 cellular
organisms;Eukaryota;Opisthokonta;Metazoa;Eumetazoa;Bilateria;Protostomia;Spiralia;Lophotrochozoa;Mollusca;Bivalvia;Autobranchia;Pteriomorphia;Ostreida;Ostreoidea;Ostreidae;Crassostrea;Crassostrea virginica
UdeM00000025255 1735272 645.6 UdeM00000025255 XP_041358501.1 53.1
606 258 6 1 592 318 911 7.10E-182 645.6 1735272
cellular
organisms;Eukaryota;Opisthokonta;Metazoa;Eumetazoa;Bilateria;Protostomia;

Spiralia;Lophotrochozoa;Mollusca;Gastropoda;Neomphalina;Neomphaloidea;Peltospiridae;Gigantopelta;Gigantopelta aegis
UdeM00000025256 6565 96.7 UdeM00000025256 XP_022327050.1 75.8 62 15 0 126 187 907 968 3.40E-17 96.7 6565 cellular organisms;Eukaryota;Opisthokonta;Metazoa;Eumetazoa;Bilateria;Protostomia;Spiralia;Lophotrochozoa;Mollusca;Bivalvia;Autobranchia;Pteriomorphia;Ostreida;Ostreoidea;Ostreidae;Crassostrea;Crassostrea virginica
UdeM00000025257 6596 230.7 UdeM00000025257 XP_045189699.1 42.6 495 187 9 54 451 859 1353 3.70E-57 230.7 6596 cellular organisms;Eukaryota;Opisthokonta;Metazoa;Eumetazoa;Bilateria;Protostomia;Spiralia;Lophotrochozoa;Mollusca;Bivalvia;Autobranchia;Heteroconchia;Euheterodonta;Imparidentia;Venerida;Veneroidea;Veneridae;Mercenaria;Mercenaria mercenaria
UdeM00000025258 6596 230.7 UdeM00000025258 XP_045189699.1 42.6 495 187 9 54 451 859 1353 3.70E-57 230.7 6596 cellular organisms;Eukaryota;Opisthokonta;Metazoa;Eumetazoa;Bilateria;Protostomia;Spiralia;Lophotrochozoa;Mollusca;Bivalvia;Autobranchia;Heteroconchia;Euheterodonta;Imparidentia;Venerida;Veneroidea;Veneridae;Mercenaria;Mercenaria mercenaria
UdeM00000025259 6596 159.5 UdeM00000025259 XP_045171063.1 35.3 252 158 5 15 264 7 255 6.10E-36 159.5 6596 cellular organisms;Eukaryota;Opisthokonta;Metazoa;Eumetazoa;Bilateria;Protostomia;Spiralia;Lophotrochozoa;Mollusca;Bivalvia;Autobranchia;Heteroconchia;Euheterodonta;Imparidentia;Venerida;Veneroidea;Veneridae;Mercenaria;Mercenaria mercenaria
UdeM00000025260 6596 130.6 UdeM00000025260 XP_045171063.1 36 211 130 5 14 222 48 255 2.60E-27 130.6 6596 cellular organisms;Eukaryota;Opisthokonta;Metazoa;Eumetazoa;Bilateria;Protostomia;Spiralia;Lophotrochozoa;Mollusca;Bivalvia;Autobranchia;Heteroconchia;Euheterodonta;Imparidentia;Venerida;Veneroidea;Veneridae;Mercenaria;Mercenaria mercenaria
UdeM00000025261 7994 163.3 UdeM00000025261 XP_022537161.1 94.2 86 5 0 32 117 1 86 1.90E-37 163.3 7994 cellular organisms;Eukaryota;Opisthokonta;Metazoa;Eumetazoa;Bilateria;Deuterostomia;Chordata;Craniata;Vertebrata;Gnathostomata;Teleostomi;Euteleostomi;Actinopterygii;Actinopteri;Neopterygii;Teleostei;Osteoglossocephalai;Clupeocephala;Otomorpha;Ostariophysi;Otophysi;Characiphysae;Characiformes;Characoidei;Characidae;Stethaprioninae;Astyanax;Astyanax mexicanus
UdeM00000025262 37003 87 UdeM00000025262 XP_017260744.1 55 60 27 0 141 200 78 137 2.90E-14 87 37003 cellular organisms;Eukaryota;Opisthokonta;Metazoa;Eumetazoa;Bilateria;Deuterostomia;Chordata;Craniata;Vertebrata;Gnathostomata;Teleostomi;Euteleostomi;Actinopterygii;Actinopteri;Neopterygii;Teleostei;Osteoglossocephalai;Clupeocephala;Euteleosteomorpha;Neoteleostei;Eurypterygia;Ctenosquamata;Acanthomorphata;Euacanthomorphacea;Percomorphaceae;Ovalentaria;Atherinomorphae;Cyprinodontiformes;Aplocheiloidei;Rivulidae;Kryptolebias;Kryptolebias marmoratus
UdeM00000025264 6596 583.2 UdeM00000025264 XP_045203022.1 43.9 585 284 6 19 599 29 573 4.20E-163 583.2 6596 cellular organisms;Eukaryota;Opisthokonta;Metazoa;Eumetazoa;Bilateria;Protostomia;Spiralia;Lophotrochozoa;Mollusca;Bivalvia;Autobranchia;Heteroconchia;Euheterodonta;Imparidentia;Venerida;Veneroidea;Veneridae;Mercenaria;Mercenaria mercenaria

UdeM00000025265 6596 142.9 UdeM00000025265 XP_045202791.1 41.9 198
98 6 5 199 7 190 4.40E-31 142.9 6596 cellular organisms;Eukaryota;Opisthokonta;Metazoa;Eumetazoa;Bilateria;Protostomia;Spiralia;Lophotrochozoa;Mollusca;Bivalvia;Autobranchia;Heteroconchia;Euheterodonta;Imparidentia;Venerida;Veneroidea;Veneridae;Mercenaria;Mercenaria mercenaria
UdeM00000025266 6579 248.8 UdeM00000025266 XP_033742790.1 37 395
234 6 71 461 16 399 2.40E-62 248.8 6579 cellular organisms;Eukaryota;Opisthokonta;Metazoa;Eumetazoa;Bilateria;Protostomia;Spiralia;Lophotrochozoa;Mollusca;Bivalvia;Autobranchia;Pteriomorphia;Pectinida;Pectinoidea;Pectinidae;Pecten;Pecten maximus
UdeM00000025268 6565 535 UdeM00000025268 XP_022327844.1 58.5 487
190 3 1 482 58 537 9.80E-149 535 6565 cellular organisms;Eukaryota;Opisthokonta;Metazoa;Eumetazoa;Bilateria;Protostomia;Spiralia;Lophotrochozoa;Mollusca;Bivalvia;Autobranchia;Pteriomorphia;Ostreida;Ostreoidea;Ostreidae;Crassostrea;Crassostrea virginica
UdeM00000025269 6565 559.7 UdeM00000025269 XP_022328337.1 56.5 522
213 4 26 540 21 535 6.80E-156 559.7 6565 cellular organisms;Eukaryota;Opisthokonta;Metazoa;Eumetazoa;Bilateria;Protostomia;Spiralia;Lophotrochozoa;Mollusca;Bivalvia;Autobranchia;Pteriomorphia;Ostreida;Ostreoidea;Ostreidae;Crassostrea;Crassostrea virginica
UdeM00000025270 6596 439.5 UdeM00000025270 XP_045204896.1 58.3 393
147 6 34 420 28 409 4.80E-120 439.5 6596 cellular organisms;Eukaryota;Opisthokonta;Metazoa;Eumetazoa;Bilateria;Protostomia;Spiralia;Lophotrochozoa;Mollusca;Bivalvia;Autobranchia;Heteroconchia;Euheterodonta;Imparidentia;Venerida;Veneroidea;Veneridae;Mercenaria;Mercenaria mercenaria
UdeM00000025271 6565 176.4 UdeM00000025271 XP_022320066.1 49.5 182
88 3 4 185 2 179 3.50E-41 176.4 6565 cellular organisms;Eukaryota;Opisthokonta;Metazoa;Eumetazoa;Bilateria;Protostomia;Spiralia;Lophotrochozoa;Mollusca;Bivalvia;Autobranchia;Pteriomorphia;Ostreida;Ostreoidea;Ostreidae;Crassostrea;Crassostrea virginica
UdeM00000025272 6596 467.6 UdeM00000025272 XP_045204917.1 58.7 407
159 3 5 406 37 439 1.60E-128 467.6 6596 cellular organisms;Eukaryota;Opisthokonta;Metazoa;Eumetazoa;Bilateria;Protostomia;Spiralia;Lophotrochozoa;Mollusca;Bivalvia;Autobranchia;Heteroconchia;Euheterodonta;Imparidentia;Venerida;Veneroidea;Veneridae;Mercenaria;Mercenaria mercenaria
UdeM00000025273 6596 487.6 UdeM00000025273 XP_045204271.1 42.5 595
313 9 55 638 4 580 2.30E-134 487.6 6596 cellular organisms;Eukaryota;Opisthokonta;Metazoa;Eumetazoa;Bilateria;Protostomia;Spiralia;Lophotrochozoa;Mollusca;Bivalvia;Autobranchia;Heteroconchia;Euheterodonta;Imparidentia;Venerida;Veneroidea;Veneridae;Mercenaria;Mercenaria mercenaria
UdeM00000025274 6596 610.9 UdeM00000025274 XP_045202781.1 53 577
261 6 6 575 8 581 1.60E-171 610.9 6596 cellular organisms;Eukaryota;Opisthokonta;Metazoa;Eumetazoa;Bilateria;Protostomia;Spiralia;Lophotrochozoa;Mollusca;Bivalvia;Autobranchia;Heteroconchia;Euheterodonta;Imparidentia;Venerida;Veneroidea;Veneridae;Mercenaria;Mercenaria mercenaria
UdeM00000025275 6596 600.9 UdeM00000025275 XP_045203759.1 51 574
275 5 7 576 19 590 1.70E-168 600.9 6596 cellular organisms;Eukaryota;Opisthokonta;Metazoa;Eumetazoa;Bilateria;Protostomia;Spiralia;Lophotrochozoa;Mollusca;Bivalvia;Autobranchia;Heteroconchia;Euhe

terodonta;Imparidentia;Venerida;Veneroidea;Veneridae;Mercenaria;Mercenaria mercenaria
UdeM00000025276 6412 195.7 UdeM00000025276 XP_009008669.1 39.1 266
150 4 137 402 133 386 1.20E-46 195.7 6412 cellular organisms;Eukaryota;Opisthokonta;Metazoa;Eumetazoa;Bilateria;Protostomia;Spiralia;Lophotrochozoa;Annelida;Clitellata;Hirudinea;Rhynchobdellida;Glossiphoniidae;Helobdella;Helobdella robusta
UdeM00000025278 6596 315.5 UdeM00000025278 XP_045205084.1 46.9 343
166 3 8 350 11 337 1.20E-82 315.5 6596 cellular organisms;Eukaryota;Opisthokonta;Metazoa;Eumetazoa;Bilateria;Protostomia;Spiralia;Lophotrochozoa;Mollusca;Bivalvia;Autobranchia;Heteroconchia;Euheterodonta;Imparidentia;Venerida;Veneroidea;Veneridae;Mercenaria;Mercenaria mercenaria
UdeM00000025277 6596 262.7 UdeM00000025277 XP_045205084.1 46.3 298
144 3 32 329 56 337 8.60E-67 262.7 6596 cellular organisms;Eukaryota;Opisthokonta;Metazoa;Eumetazoa;Bilateria;Protostomia;Spiralia;Lophotrochozoa;Mollusca;Bivalvia;Autobranchia;Heteroconchia;Euheterodonta;Imparidentia;Venerida;Veneroidea;Veneridae;Mercenaria;Mercenaria mercenaria
UdeM00000025280 6573 119.8 UdeM00000025280 XP_021345821.1 38.8 196
112 2 6 196 5 197 3.60E-23 119.8 6573 cellular organisms;Eukaryota;Opisthokonta;Metazoa;Eumetazoa;Bilateria;Protostomia;Spiralia;Lophotrochozoa;Mollusca;Bivalvia;Autobranchia;Pteriomorphia;Pectinida;Pectinoidea;Pectinidae;Mizuhopecten;Mizuhopecten yessoensis
UdeM00000025281 6596 291.6 UdeM00000025281 XP_045203447.1 60.7 270
94 4 24 283 347 614 2.00E-75 291.6 6596 cellular organisms;Eukaryota;Opisthokonta;Metazoa;Eumetazoa;Bilateria;Protostomia;Spiralia;Lophotrochozoa;Mollusca;Bivalvia;Autobranchia;Heteroconchia;Euheterodonta;Imparidentia;Venerida;Veneroidea;Veneridae;Mercenaria;Mercenaria mercenaria
UdeM00000025282 6573 102.1 UdeM00000025282 XP_021371245.1 46.9 179
91 3 42 219 1242 1417 1.50E-18 102.1 6573 cellular organisms;Eukaryota;Opisthokonta;Metazoa;Eumetazoa;Bilateria;Protostomia;Spiralia;Lophotrochozoa;Mollusca;Bivalvia;Autobranchia;Pteriomorphia;Pectinida;Pectinoidea;Pectinidae;Mizuhopecten;Mizuhopecten yessoensis
UdeM00000025283 6596 354.8 UdeM00000025283 XP_045206000.1 41.4 967
389 29 63 889 134 1062 4.10E-94 354.8 6596 cellular organisms;Eukaryota;Opisthokonta;Metazoa;Eumetazoa;Bilateria;Protostomia;Spiralia;Lophotrochozoa;Mollusca;Bivalvia;Autobranchia;Heteroconchia;Euheterodonta;Imparidentia;Venerida;Veneroidea;Veneridae;Mercenaria;Mercenaria mercenaria
UdeM00000025284 29159 329.7 UdeM00000025284 XP_011442674.1 63.4 265
97 0 1 265 1 265 3.40E-87 329.7 29159 cellular organisms;Eukaryota;Opisthokonta;Metazoa;Eumetazoa;Bilateria;Protostomia;Spiralia;Lophotrochozoa;Mollusca;Bivalvia;Autobranchia;Pteriomorphia;Ostreida;Ostreoidea;Ostreidae;Crassostrea;Crassostrea gigas
UdeM00000025286 6596 304.3 UdeM00000025286 XP_045206720.1 63 216
68 1 197 412 240 443 2.40E-79 304.3 6596 cellular organisms;Eukaryota;Opisthokonta;Metazoa;Eumetazoa;Bilateria;Protostomia;Spiralia;Lophotrochozoa;Mollusca;Bivalvia;Autobranchia;Heteroconchia;Euheterodonta;Imparidentia;Venerida;Veneroidea;Veneridae;Mercenaria;Mercenaria mercenaria
UdeM00000025287 6596 301.2 UdeM00000025287 XP_045206720.1 63.6 217
64 3 197 411 240 443 2.00E-78 301.2 6596 cellular

organisms;Eukaryota;Opisthokonta;Metazoa;Eumetazoa;Bilateria;Protostomia;Spiralia;Lophotrochozoa;Mollusca;Bivalvia;Autobranchia;Heteroconchia;Euheterodonta;Imparidentia;Venerida;Veneroidea;Veneridae;Mercenaria;Mercenaria mercenaria
UdeM00000025289 6596 291.6 UdeM00000025289 XP_045204760.1 43 874 420 22 1 844 1 826 3.20E-75 291.6 6596 cellular organisms;Eukaryota;Opisthokonta;Metazoa;Eumetazoa;Bilateria;Protostomia;Spiralia;Lophotrochozoa;Mollusca;Bivalvia;Autobranchia;Heteroconchia;Euheterodonta;Imparidentia;Venerida;Veneroidea;Veneridae;Mercenaria;Mercenaria mercenaria
UdeM00000025290 6579 250 UdeM00000025290 XP_033751799.1 37.2 398 233 8 114 504 21 408 7.20E-63 250 6579 cellular organisms;Eukaryota;Opisthokonta;Metazoa;Eumetazoa;Bilateria;Protostomia;Spiralia;Lophotrochozoa;Mollusca;Bivalvia;Autobranchia;Pteriomorphia;Pectinida;Pectinoidea;Pectinidae;Pecten;Pecten maximus
UdeM00000025291 6579 250 UdeM00000025291 XP_033751799.1 37.2 398 233 8 115 505 21 408 7.20E-63 250 6579 cellular organisms;Eukaryota;Opisthokonta;Metazoa;Eumetazoa;Bilateria;Protostomia;Spiralia;Lophotrochozoa;Mollusca;Bivalvia;Autobranchia;Pteriomorphia;Pectinida;Pectinoidea;Pectinidae;Pecten;Pecten maximus
UdeM00000025292 6596 364 UdeM00000025292 XP_045205172.1 45.6 406 215 5 227 629 197 599 3.80E-97 364 6596 cellular organisms;Eukaryota;Opisthokonta;Metazoa;Eumetazoa;Bilateria;Protostomia;Spiralia;Lophotrochozoa;Mollusca;Bivalvia;Autobranchia;Heteroconchia;Euheterodonta;Imparidentia;Venerida;Veneroidea;Veneridae;Mercenaria;Mercenaria mercenaria
UdeM00000025293 1735272 1406.3 UdeM00000025293 XP_041360351.1 39 2478 1298 50 102 2439 2560 4963 0.00E+00 1406.3 1735272 cellular organisms;Eukaryota;Opisthokonta;Metazoa;Eumetazoa;Bilateria;Protostomia;Spiralia;Lophotrochozoa;Mollusca;Gastropoda;Neomphalina;Neomphaloidea;Peltospiridae;Gigantopelta;Gigantopelta aegis
UdeM00000025294 6850 235.7 UdeM00000025294 XP_022249667.1 48.4 248 126 2 33 278 1696 1943 7.30E-59 235.7 6850 cellular organisms;Eukaryota;Opisthokonta;Metazoa;Eumetazoa;Bilateria;Protostomia;Ecdysozoa;Panarthropoda;Arthropoda;Chelicerata;Merostomata;Xiphosura;Limulidae;Limulus;Limulus polyphemus
UdeM00000025295 6573 1369.4 UdeM00000025295 XP_021352384.1 44.5 1731 925 20 14 1729 14 1724 0.00E+00 1369.4 6573 cellular organisms;Eukaryota;Opisthokonta;Metazoa;Eumetazoa;Bilateria;Protostomia;Spiralia;Lophotrochozoa;Mollusca;Bivalvia;Autobranchia;Pteriomorphia;Pectinida;Pectinoidea;Pectinidae;Mizuhopecten;Mizuhopecten yessoensis
UdeM00000025299 1735272 820.1 UdeM00000025299 XP_041359038.1 66.3 576 191 3 37 612 112 684 1.90E-234 820.1 1735272 cellular organisms;Eukaryota;Opisthokonta;Metazoa;Eumetazoa;Bilateria;Protostomia;Spiralia;Lophotrochozoa;Mollusca;Gastropoda;Neomphalina;Neomphaloidea;Peltospiridae;Gigantopelta;Gigantopelta aegis
UdeM00000025300 6500 364 UdeM00000025300 XP_005109735.2 54.5 347 150 5 8 349 20 363 2.50E-97 364 6500 cellular organisms;Eukaryota;Opisthokonta;Metazoa;Eumetazoa;Bilateria;Protostomia;Spiralia;Lophotrochozoa;Mollusca;Gastropoda;Heterobranchia;Euthyneura;Tectipleura;Aplysiida;Aplysioidea;Aplysiidae;Aplysia;Aplysia californica

UdeM00000025301 6579 692.2 UdeM00000025301 XP_033742952.1 47 956
423 20 151 1081 169 1065 1.10E-195 692.2 6579 cellular
organisms;Eukaryota;Opisthokonta;Metazoa;Eumetazoa;Bilateria;Protostomia;
Spiralia;Lophotrochozoa;Mollusca;Bivalvia;Autobranchia;Pteriomorphia;Pect
inida;Pectinoidea;Pectinidae;Pecten;Pecten maximus
UdeM00000025302 6596 474.9 UdeM00000025302 XP_045202907.1 77.5 298
66 1 1 298 27 323 7.30E-131 474.9 6596 cellular
organisms;Eukaryota;Opisthokonta;Metazoa;Eumetazoa;Bilateria;Protostomia;
Spiralia;Lophotrochozoa;Mollusca;Bivalvia;Autobranchia;Heteroconchia;Euhe
terodonta;Imparidentia;Venerida;Veneroidea;Veneridae;Mercenaria;Mercenari
a mercenaria
UdeM00000025303 7159 156.4 UdeM00000025303 XP_021712114.1 26.6 403
277 6 11 409 1191 1578 9.90E-35 156.4 7159 cellular
organisms;Eukaryota;Opisthokonta;Metazoa;Eumetazoa;Bilateria;Protostomia;
Ecdysozoa;Panarthropoda;Arthropoda;Mandibulata;Pancrustacea;Hexapoda;Inse
cta;Dicondylia;Pterygota;Neoptera;Endopterygota;Diptera;Nematocera;Culico
morpha;Culicoidea;Culicidae;Culicinae;Aedini;Aedes;Stegomyia;Aedes
aegypti
UdeM00000025304 1220207 71.6 UdeM00000025304 XP_041549944.1 32.7
113 75 1 14 125 635 747 9.50E-10 71.6 1220207
cellular
organisms;Eukaryota;Opisthokonta;Fungi;Dikarya;Ascomycota;saccharomyceta;
Pezizomycotina;leotiomyceta;Eurotiomycetes;Eurotiomycetidae;Eurotiales;As
pergillaceae;Aspergillus;Aspergillus puulaauensis
UdeM00000025305 50390 196.4 UdeM00000025305 XP_028127913.1 43.7 231
129 1 1 231 1 230 4.10E-47 196.4 50390 cellular
organisms;Eukaryota;Opisthokonta;Metazoa;Eumetazoa;Bilateria;Protostomia;
Ecdysozoa;Panarthropoda;Arthropoda;Mandibulata;Pancrustacea;Hexapoda;Inse
cta;Dicondylia;Pterygota;Neoptera;Endopterygota;Coleoptera;Polyphaga;Cucu
jiformia;Chrysomeloidea;Chrysomelidae;Galerucinae;Luperini;Diabroticina;D
iabroticites;Diabrotica;Diabrotica virgifera;Diabrotica virgifera
virgifera
UdeM00000025306 8267 233.8 UdeM00000025306 XP_034459057.1 39.2 344
199 4 1 341 485 821 3.30E-58 233.8 8267 cellular
organisms;Eukaryota;Opisthokonta;Metazoa;Eumetazoa;Bilateria;Deuterostomi
a;Chordata;Craniata;Vertebrata;Gnathostomata;Teleostomi;Euteleostomi;Acti
nopterygii;Actinopteri;Neopterygii;Teleostei;Osteoglossocephalai;Clupeoce
phala;Euteleosteomorpha;Neoteleostei;Eurypterygia;Ctenosquamata;Acanthomo
rphata;Euacanthomorphacea;Percomorphaceae;Carangaria;Pleuronectiformes;Pl
euronectoidei;Pleuronectidae;Hippoglossus;Hippoglossus hippoglossus
UdeM00000025307 6596 592 UdeM00000025307 XP_045205529.1 80.2 435
63 2 1 414 1 433 5.70E-166 592 6596 cellular
organisms;Eukaryota;Opisthokonta;Metazoa;Eumetazoa;Bilateria;Protostomia;
Spiralia;Lophotrochozoa;Mollusca;Bivalvia;Autobranchia;Heteroconchia;Euhe
terodonta;Imparidentia;Venerida;Veneroidea;Veneridae;Mercenaria;Mercenari
a mercenaria
UdeM00000025308 6596 2356.3 UdeM00000025308 XP_045206430.1 67.7
2134 567 16 1 2115 1 2031 0.00E+00 2356.3 6596
cellular
organisms;Eukaryota;Opisthokonta;Metazoa;Eumetazoa;Bilateria;Protostomia;
Spiralia;Lophotrochozoa;Mollusca;Bivalvia;Autobranchia;Heteroconchia;Euhe
terodonta;Imparidentia;Venerida;Veneroidea;Veneridae;Mercenaria;Mercenari
a mercenaria

UdeM00000025309 6565 701.8 UdeM00000025309 XP_022326504.1 91 368
33 0 1 368 1 368 4.50E-199 701.8 6565 cellular
organisms;Eukaryota;Opisthokonta;Metazoa;Eumetazoa;Bilateria;Protostomia;
Spiralia;Lophotrochozoa;Mollusca;Bivalvia;Autobranchia;Pteriomorphia;Ostr
eida;Ostreoidea;Ostreidae;Crassostrea;Crassostrea virginica
UdeM00000025311 6596 705.3 UdeM00000025311 XP_045206430.1 82.9 403
68 1 42 443 1961 2363 5.40E-200 705.3 6596 cellular
organisms;Eukaryota;Opisthokonta;Metazoa;Eumetazoa;Bilateria;Protostomia;
Spiralia;Lophotrochozoa;Mollusca;Bivalvia;Autobranchia;Heteroconchia;Euhe
terodonta;Imparidentia;Venerida;Veneroidea;Veneridae;Mercenaria;Mercenari
a mercenaria
UdeM00000025310 6579 1330.5 UdeM00000025310 XP_033742934.1 81.7
765 137 2 42 805 2163 2925 0.00E+00 1330.5 6579
cellular
organisms;Eukaryota;Opisthokonta;Metazoa;Eumetazoa;Bilateria;Protostomia;
Spiralia;Lophotrochozoa;Mollusca;Bivalvia;Autobranchia;Pteriomorphia;Pect
inida;Pectinoidea;Pectinidae;Pecten;Pecten maximus
UdeM00000025312 2607531 670.6 UdeM00000025312 XP_029643971.1 43.2
850 407 15 1 835 1 789 2.50E-189 670.6 2607531
cellular
organisms;Eukaryota;Opisthokonta;Metazoa;Eumetazoa;Bilateria;Protostomia;
Spiralia;Lophotrochozoa;Mollusca;Cephalopoda;Coleoidea;Octopodiformes;Oct
opoda;Incirrata;Octopodidae;Octopus;Octopus sinensis
UdeM00000025313 6596 197.6 UdeM00000025313 XP_045206368.1 61.4 127
48 1 10 135 1 127 1.20E-47 197.6 6596 cellular
organisms;Eukaryota;Opisthokonta;Metazoa;Eumetazoa;Bilateria;Protostomia;
Spiralia;Lophotrochozoa;Mollusca;Bivalvia;Autobranchia;Heteroconchia;Euhe
terodonta;Imparidentia;Venerida;Veneroidea;Veneridae;Mercenaria;Mercenari
a mercenaria
UdeM00000025314 7574 152.5 UdeM00000025314 XP_023932663.1 29.6 429
274 11 15 426 39 456 1.40E-33 152.5 7574 cellular
organisms;Eukaryota;Opisthokonta;Metazoa;Eumetazoa;Bilateria;Protostomia;
Spiralia;Lophotrochozoa;Brachiopoda;Linguliformea;Lingulata;Lingulida;Lin
guloidea;Lingulidae;Lingula;Lingula anatina
UdeM00000025315 6573 268.5 UdeM00000025315 XP_021359539.1 42.5 400
186 11 215 579 282 672 2.10E-68 268.5 6573 cellular
organisms;Eukaryota;Opisthokonta;Metazoa;Eumetazoa;Bilateria;Protostomia;
Spiralia;Lophotrochozoa;Mollusca;Bivalvia;Autobranchia;Pteriomorphia;Pect
inida;Pectinoidea;Pectinidae;Mizuhopecten;Mizuhopecten yessoensis
UdeM00000025317 6596 110.2 UdeM00000025317 XP_045203123.1 57.9 95
34 2 209 300 668 759 8.00E-21 110.2 6596 cellular
organisms;Eukaryota;Opisthokonta;Metazoa;Eumetazoa;Bilateria;Protostomia;
Spiralia;Lophotrochozoa;Mollusca;Bivalvia;Autobranchia;Heteroconchia;Euhe
terodonta;Imparidentia;Venerida;Veneroidea;Veneridae;Mercenaria;Mercenari
a mercenaria
UdeM00000025316 6596 153.3 UdeM00000025316 XP_045203123.1 30.6 467
242 15 37 476 348 759 1.10E-33 153.3 6596 cellular
organisms;Eukaryota;Opisthokonta;Metazoa;Eumetazoa;Bilateria;Protostomia;
Spiralia;Lophotrochozoa;Mollusca;Bivalvia;Autobranchia;Heteroconchia;Euhe
terodonta;Imparidentia;Venerida;Veneroidea;Veneridae;Mercenaria;Mercenari
a mercenaria
UdeM00000025318 6596 66.2 UdeM00000025318 XP_045203123.1 34.7 150
74 5 41 175 348 488 4.90E-08 66.2 6596 cellular
organisms;Eukaryota;Opisthokonta;Metazoa;Eumetazoa;Bilateria;Protostomia;

Spiralia;Lophotrochozoa;Mollusca;Bivalvia;Autobranchia;Heteroconchia;Euheterodonta;Imparidentia;Venerida;Veneroidea;Veneridae;Mercenaria;Mercenaria mercenaria
UdeM00000025319 52670 82.4 UdeM00000025319 XP_013874439.1 56.7 60 26 0 46 105 1714 1773 3.90E-13 82.4 52670 cellular organisms;Eukaryota;Opisthokonta;Metazoa;Eumetazoa;Bilateria;Deuterostomia;Chordata;Craniata;Vertebrata;Gnathostomata;Teleostomi;Euteleostomi;Actinopterygii;Actinopteri;Neopterygii;Teleostei;Osteoglossocephalai;Clupeocephala;Euteleosteomorpha;Neoteleostei;Eurypterygia;Ctenosquamata;Acanthomorphata;Euacanthomorphacea;Percomorphaceae;Ovalentaria;Atherinomorphae;Cyprinodontiformes;Aplocheiloidei;Rivulidae;Austrofundulus;Austrofundulus limnaeus
UdeM00000025320 52670 82.4 UdeM00000025320 XP_013874439.1 56.7 60 26 0 44 103 1714 1773 3.80E-13 82.4 52670 cellular organisms;Eukaryota;Opisthokonta;Metazoa;Eumetazoa;Bilateria;Deuterostomia;Chordata;Craniata;Vertebrata;Gnathostomata;Teleostomi;Euteleostomi;Actinopterygii;Actinopteri;Neopterygii;Teleostei;Osteoglossocephalai;Clupeocephala;Euteleosteomorpha;Neoteleostei;Eurypterygia;Ctenosquamata;Acanthomorphata;Euacanthomorphacea;Percomorphaceae;Ovalentaria;Atherinomorphae;Cyprinodontiformes;Aplocheiloidei;Rivulidae;Austrofundulus;Austrofundulus limnaeus
UdeM00000025321 6596 183.7 UdeM00000025321 XP_045203123.1 55.8 165 65 3 12 175 83 240 3.20E-43 183.7 6596 cellular organisms;Eukaryota;Opisthokonta;Metazoa;Eumetazoa;Bilateria;Protostomia;Spiralia;Lophotrochozoa;Mollusca;Bivalvia;Autobranchia;Heteroconchia;Euheterodonta;Imparidentia;Venerida;Veneroidea;Veneridae;Mercenaria;Mercenaria mercenaria
UdeM00000025322 6596 95.1 UdeM00000025322 XP_045203123.1 64.2 67 24 0 1 67 5 71 4.60E-17 95.1 6596 cellular organisms;Eukaryota;Opisthokonta;Metazoa;Eumetazoa;Bilateria;Protostomia;Spiralia;Lophotrochozoa;Mollusca;Bivalvia;Autobranchia;Heteroconchia;Euheterodonta;Imparidentia;Venerida;Veneroidea;Veneridae;Mercenaria;Mercenaria mercenaria
UdeM00000025323 13686 204.1 UdeM00000025323 XP_039302821.1 40.8 262 153 1 1 262 364 623 2.10E-49 204.1 13686 cellular organisms;Eukaryota;Opisthokonta;Metazoa;Eumetazoa;Bilateria;Protostomia;Ecdysozoa;Panarthropoda;Arthropoda;Mandibulata;Pancrustacea;Hexapoda;Insecta;Dicondylia;Pterygota;Neoptera;Endopterygota;Hymenoptera;Apocrita;Aculeata;Formicoidea;Formicidae;Myrmicinae;Solenopsidini;Solenopsis;Solenopsis invicta
UdeM00000025324 13686 175.6 UdeM00000025324 XP_011170408.3 38.9 283 146 10 110 373 115 389 1.20E-40 175.6 13686 cellular organisms;Eukaryota;Opisthokonta;Metazoa;Eumetazoa;Bilateria;Protostomia;Ecdysozoa;Panarthropoda;Arthropoda;Mandibulata;Pancrustacea;Hexapoda;Insecta;Dicondylia;Pterygota;Neoptera;Endopterygota;Hymenoptera;Apocrita;Aculeata;Formicoidea;Formicidae;Myrmicinae;Solenopsidini;Solenopsis;Solenopsis invicta
UdeM00000025325 6596 625.9 UdeM00000025325 XP_045203122.1 49.6 1015 446 23 1 992 1 972 8.50E-176 625.9 6596 cellular organisms;Eukaryota;Opisthokonta;Metazoa;Eumetazoa;Bilateria;Protostomia;Spiralia;Lophotrochozoa;Mollusca;Bivalvia;Autobranchia;Heteroconchia;Euheterodonta;Imparidentia;Venerida;Veneroidea;Veneridae;Mercenaria;Mercenaria mercenaria

UdeM00000025327 6596 647.1 UdeM00000025327 XP_045204100.1 58.3 581
224 7 22 596 10 578 2.30E-182 647.1 6596 cellular organisms;Eukaryota;Opisthokonta;Metazoa;Eumetazoa;Bilateria;Protostomia;Spiralia;Lophotrochozoa;Mollusca;Bivalvia;Autobranchia;Heteroconchia;Euheterodonta;Imparidentia;Venerida;Veneroidea;Veneridae;Mercenaria;Mercenaria mercenaria
UdeM00000025326 6596 647.9 UdeM00000025326 XP_045204100.1 59.2 571
215 7 150 714 20 578 1.60E-182 647.9 6596 cellular organisms;Eukaryota;Opisthokonta;Metazoa;Eumetazoa;Bilateria;Protostomia;Spiralia;Lophotrochozoa;Mollusca;Bivalvia;Autobranchia;Heteroconchia;Euheterodonta;Imparidentia;Venerida;Veneroidea;Veneridae;Mercenaria;Mercenaria mercenaria
UdeM00000025328 6596 667.9 UdeM00000025328 XP_045179825.1 79.9 394
79 0 1 394 1 394 7.80E-189 667.9 6596 cellular organisms;Eukaryota;Opisthokonta;Metazoa;Eumetazoa;Bilateria;Protostomia;Spiralia;Lophotrochozoa;Mollusca;Bivalvia;Autobranchia;Heteroconchia;Euheterodonta;Imparidentia;Venerida;Veneroidea;Veneridae;Mercenaria;Mercenaria mercenaria
UdeM00000025329 50390 380.9 UdeM00000025329 XP_028154228.1 52.3 323
145 2 1 320 126 442 1.50E-102 380.9 50390 cellular organisms;Eukaryota;Opisthokonta;Metazoa;Eumetazoa;Bilateria;Protostomia;Ecdysozoa;Panarthropoda;Arthropoda;Mandibulata;Pancrustacea;Hexapoda;Insecta;Dicondylia;Pterygota;Neoptera;Endopterygota;Coleoptera;Polyphaga;Cucujiformia;Chrysomeloidea;Chrysomelidae;Galerucinae;Luperini;Diabroticina;Diabroticites;Diabrotica;Diabrotica virgifera;Diabrotica virgifera virgifera
UdeM00000025330 105023 92 UdeM00000025330 XP_015797448.1 33.9
118 77 1 106 222 29 146 1.00E-15 92 105023
cellular organisms;Eukaryota;Opisthokonta;Metazoa;Eumetazoa;Bilateria;Deuterostomia;Chordata;Craniata;Vertebrata;Gnathostomata;Teleostomi;Euteleostomi;Actinopterygii;Actinopteri;Neopterygii;Teleostei;Osteoglossocephalai;Clupeocephala;Euteleosteomorpha;Neoteleostei;Eurypterygia;Ctenosquamata;Acanthomorphata;Euacanthomorphacea;Percomorphaceae;Ovalentaria;Atherinomorphae;Cyprinodontiformes;Aplocheiloidei;Nothobranchiidae;Nothobranchius;Nothobranchius furzeri
UdeM00000025331 105023 92 UdeM00000025331 XP_015797448.1 33.9
118 77 1 75 191 29 146 8.80E-16 92 105023
cellular organisms;Eukaryota;Opisthokonta;Metazoa;Eumetazoa;Bilateria;Deuterostomia;Chordata;Craniata;Vertebrata;Gnathostomata;Teleostomi;Euteleostomi;Actinopterygii;Actinopteri;Neopterygii;Teleostei;Osteoglossocephalai;Clupeocephala;Euteleosteomorpha;Neoteleostei;Eurypterygia;Ctenosquamata;Acanthomorphata;Euacanthomorphacea;Percomorphaceae;Ovalentaria;Atherinomorphae;Cyprinodontiformes;Aplocheiloidei;Nothobranchiidae;Nothobranchius;Nothobranchius furzeri
UdeM00000025332 50390 456.4 UdeM00000025332 XP_028142380.1 52.3 348
166 0 1 348 5 352 3.10E-125 456.4 50390 cellular organisms;Eukaryota;Opisthokonta;Metazoa;Eumetazoa;Bilateria;Protostomia;Ecdysozoa;Panarthropoda;Arthropoda;Mandibulata;Pancrustacea;Hexapoda;Insecta;Dicondylia;Pterygota;Neoptera;Endopterygota;Coleoptera;Polyphaga;Cucujiformia;Chrysomeloidea;Chrysomelidae;Galerucinae;Luperini;Diabroticina;Diabroticites;Diabrotica;Diabrotica virgifera;Diabrotica virgifera virgifera

UdeM00000025333 6596 226.9 UdeM00000025333 XP_045187824.1 40.7 295 171 2 10 304 42 332 4.10E-56 226.9 6596 cellular organisms;Eukaryota;Opisthokonta;Metazoa;Eumetazoa;Bilateria;Protostomia;Spiralia;Lophotrochozoa;Mollusca;Bivalvia;Autobranchia;Heteroconchia;Euheterodonta;Imparidentia;Venerida;Veneroidea;Veneridae;Mercenaria;Mercenaria mercenaria
UdeM00000025334 50390 416.8 UdeM00000025334 XP_028142380.1 55 320 140 1 1 316 89 408 2.50E-113 416.8 50390 cellular organisms;Eukaryota;Opisthokonta;Metazoa;Eumetazoa;Bilateria;Protostomia;Ecdysozoa;Panarthropoda;Arthropoda;Mandibulata;Pancrustacea;Hexapoda;Insecta;Dicondylia;Pterygota;Neoptera;Endopterygota;Coleoptera;Polyphaga;Cucujiformia;Chrysomeloidea;Chrysomelidae;Galerucinae;Luperini;Diabroticina;Diabroticites;Diabrotica;Diabrotica virgifera;Diabrotica virgifera virgifera
UdeM00000025335 50390 475.3 UdeM00000025335 XP_028142380.1 53.1 377 167 3 26 392 2 378 7.40E-131 475.3 50390 cellular organisms;Eukaryota;Opisthokonta;Metazoa;Eumetazoa;Bilateria;Protostomia;Ecdysozoa;Panarthropoda;Arthropoda;Mandibulata;Pancrustacea;Hexapoda;Insecta;Dicondylia;Pterygota;Neoptera;Endopterygota;Coleoptera;Polyphaga;Cucujiformia;Chrysomeloidea;Chrysomelidae;Galerucinae;Luperini;Diabroticina;Diabroticites;Diabrotica;Diabrotica virgifera;Diabrotica virgifera virgifera
UdeM00000025336 29159 197.2 UdeM00000025336 XP_034331441.1 44.7 219 116 3 1 216 1 217 4.50E-47 197.2 29159 cellular organisms;Eukaryota;Opisthokonta;Metazoa;Eumetazoa;Bilateria;Protostomia;Spiralia;Lophotrochozoa;Mollusca;Bivalvia;Autobranchia;Pteriomorphia;Ostreida;Ostreoidea;Ostreidae;Crassostrea;Crassostrea gigas
UdeM00000025337 50390 547.4 UdeM00000025337 XP_028154228.1 56.4 413 180 0 1 413 154 566 1.60E-152 547.4 50390 cellular organisms;Eukaryota;Opisthokonta;Metazoa;Eumetazoa;Bilateria;Protostomia;Ecdysozoa;Panarthropoda;Arthropoda;Mandibulata;Pancrustacea;Hexapoda;Insecta;Dicondylia;Pterygota;Neoptera;Endopterygota;Coleoptera;Polyphaga;Cucujiformia;Chrysomeloidea;Chrysomelidae;Galerucinae;Luperini;Diabroticina;Diabroticites;Diabrotica;Diabrotica virgifera;Diabrotica virgifera virgifera
UdeM00000025338 50390 612.5 UdeM00000025338 XP_028154228.1 54.9 481 212 1 2 482 91 566 4.70E-172 612.5 50390 cellular organisms;Eukaryota;Opisthokonta;Metazoa;Eumetazoa;Bilateria;Protostomia;Ecdysozoa;Panarthropoda;Arthropoda;Mandibulata;Pancrustacea;Hexapoda;Insecta;Dicondylia;Pterygota;Neoptera;Endopterygota;Coleoptera;Polyphaga;Cucujiformia;Chrysomeloidea;Chrysomelidae;Galerucinae;Luperini;Diabroticina;Diabroticites;Diabrotica;Diabrotica virgifera;Diabrotica virgifera virgifera
UdeM00000025339 6573 125.6 UdeM00000025339 XP_021346496.1 46.3 134 68 1 1 134 406 535 9.10E-26 125.6 6573 cellular organisms;Eukaryota;Opisthokonta;Metazoa;Eumetazoa;Bilateria;Protostomia;Spiralia;Lophotrochozoa;Mollusca;Bivalvia;Autobranchia;Pteriomorphia;Pectinida;Pectinoidea;Pectinidae;Mizuhopecten;Mizuhopecten yessoensis
UdeM00000025340 6579 251.5 UdeM00000025340 XP_033739209.1 50.4 258 112 2 9 261 2 248 2.20E-63 251.5 6579 cellular organisms;Eukaryota;Opisthokonta;Metazoa;Eumetazoa;Bilateria;Protostomia;Spiralia;Lophotrochozoa;Mollusca;Bivalvia;Autobranchia;Pteriomorphia;Pectinida;Pectinoidea;Pectinidae;Pecten;Pecten maximus

UdeM00000025341 50390 417.5 UdeM00000025341 XP_028154228.1 52 344
142 1 1 321 126 469 1.50E-113 417.5 50390 cellular
organisms;Eukaryota;Opisthokonta;Metazoa;Eumetazoa;Bilateria;Protostomia;
Ecdysozoa;Panarthropoda;Arthropoda;Mandibulata;Pancrustacea;Hexapoda;Inse
cta;Dicondylia;Pterygota;Neoptera;Endopterygota;Coleoptera;Polyphaga;Cucu
jiformia;Chrysomeloidea;Chrysomelidae;Galerucinae;Luperini;Diabroticina;D
iabroticites;Diabrotica;Diabrotica virgifera;Diabrotica virgifera
virgifera
UdeM00000025342 45264 238.4 UdeM00000025342 XP_044177087.1 50.2 231
112 2 2 232 234 461 9.20E-60 238.4 45264 cellular
organisms;Eukaryota;Opisthokonta;Metazoa;Eumetazoa;Cnidaria;Anthozoa;Hexa
corallia;Scleractinia;Astrocoeniina;Acroporidae;Acropora;Acropora
millepora
UdeM00000025343 29159 95.1 UdeM00000025343 XP_011428553.1 42.3 123
64 3 12 134 11 126 7.20E-17 95.1 29159 cellular
organisms;Eukaryota;Opisthokonta;Metazoa;Eumetazoa;Bilateria;Protostomia;
Spiralia;Lophotrochozoa;Mollusca;Bivalvia;Autobranchia;Pteriomorphia;Ostr
eida;Ostreoidea;Ostreidae;Crassostrea;Crassostrea gigas
UdeM00000025344 6573 906 UdeM00000025344 XP_021352139.1 49.1 1416
587 33 1 1369 1 1329 6.00E-260 906 6573 cellular
organisms;Eukaryota;Opisthokonta;Metazoa;Eumetazoa;Bilateria;Protostomia;
Spiralia;Lophotrochozoa;Mollusca;Bivalvia;Autobranchia;Pteriomorphia;Pect
inida;Pectinoidea;Pectinidae;Mizuhopecten;Mizuhopecten yessoensis
UdeM00000025345 6573 902.9 UdeM00000025345 XP_021352139.1 49.6 1412
578 33 1 1361 1 1329 5.10E-259 902.9 6573 cellular
organisms;Eukaryota;Opisthokonta;Metazoa;Eumetazoa;Bilateria;Protostomia;
Spiralia;Lophotrochozoa;Mollusca;Bivalvia;Autobranchia;Pteriomorphia;Pect
inida;Pectinoidea;Pectinidae;Mizuhopecten;Mizuhopecten yessoensis
UdeM00000025346 6596 441.8 UdeM00000025346 XP_045202893.1 80.4 271
53 0 1 271 1 271 6.30E-121 441.8 6596 cellular
organisms;Eukaryota;Opisthokonta;Metazoa;Eumetazoa;Bilateria;Protostomia;
Spiralia;Lophotrochozoa;Mollusca;Bivalvia;Autobranchia;Heteroconchia;Euhe
terodonta;Imparidentia;Venerida;Veneroidea;Veneridae;Mercenaria;Mercenari
a mercenaria
UdeM00000025347 6579 292.7 UdeM00000025347 XP_033742514.1 83.2 173
27 2 1 172 77 248 3.00E-76 292.7 6579 cellular
organisms;Eukaryota;Opisthokonta;Metazoa;Eumetazoa;Bilateria;Protostomia;
Spiralia;Lophotrochozoa;Mollusca;Bivalvia;Autobranchia;Pteriomorphia;Pect
inida;Pectinoidea;Pectinidae;Pecten;Pecten maximus
UdeM00000025348 133901 153.3 UdeM00000025348 XP_026278027.1 53
166 76 1 1 164 183 348 3.60E-34 153.3 133901
cellular
organisms;Eukaryota;Opisthokonta;Metazoa;Eumetazoa;Bilateria;Protostomia;
Ecdysozoa;Panarthropoda;Arthropoda;Mandibulata;Pancrustacea;Hexapoda;Inse
cta;Dicondylia;Pterygota;Neoptera;Paraneoptera;Thysanoptera;Terebrantia;T
hripoidea;Thripidae;Thripinae;Frankliniella;Frankliniella occidentalis
UdeM00000025349 6596 310.1 UdeM00000025349 XP_045202898.1 52.1 267
123 1 104 365 82 348 3.80E-81 310.1 6596 cellular
organisms;Eukaryota;Opisthokonta;Metazoa;Eumetazoa;Bilateria;Protostomia;
Spiralia;Lophotrochozoa;Mollusca;Bivalvia;Autobranchia;Heteroconchia;Euhe
terodonta;Imparidentia;Venerida;Veneroidea;Veneridae;Mercenaria;Mercenari
a mercenaria
UdeM00000025350 6596 375.9 UdeM00000025350 XP_045203987.1 33.3 768
457 17 5 745 7 746 1.20E-100 375.9 6596 cellular

organisms;Eukaryota;Opisthokonta;Metazoa;Eumetazoa;Bilateria;Protostomia;Spiralia;Lophotrochozoa;Mollusca;Bivalvia;Autobranchia;Heteroconchia;Euheterodonta;Imparidentia;Venerida;Veneroidea;Veneridae;Mercenaria;Mercenaria mercenaria
UdeM00000025351 225164 145.2 UdeM00000025351 XP_009047492.1 50 134 66 1 1 134 52 184 6.00E-32 145.2 225164 cellular organisms;Eukaryota;Opisthokonta;Metazoa;Eumetazoa;Bilateria;Protostomia;Spiralia;Lophotrochozoa;Mollusca;Gastropoda;Patellogastropoda;Lottioidea;Lottiidae;Lottia;Lottia gigantea
UdeM00000025352 6596 236.9 UdeM00000025352 XP_045167641.1 54.4 195 86 1 75 269 31 222 3.00E-59 236.9 6596 cellular organisms;Eukaryota;Opisthokonta;Metazoa;Eumetazoa;Bilateria;Protostomia;Spiralia;Lophotrochozoa;Mollusca;Bivalvia;Autobranchia;Heteroconchia;Euheterodonta;Imparidentia;Venerida;Veneroidea;Veneridae;Mercenaria;Mercenaria mercenaria
UdeM00000025354 7719 189.9 UdeM00000025354 XP_004227365.1 54 150 69 0 49 198 13 162 3.10E-45 189.9 7719 cellular organisms;Eukaryota;Opisthokonta;Metazoa;Eumetazoa;Bilateria;Deuterostomia;Chordata;Tunicata;Ascidiacea;Phlebobranchia;Cionidae;Ciona;Ciona intestinalis
UdeM00000025355 6596 154.1 UdeM00000025355 XP_045203987.1 46.9 177 86 3 56 224 298 474 2.90E-34 154.1 6596 cellular organisms;Eukaryota;Opisthokonta;Metazoa;Eumetazoa;Bilateria;Protostomia;Spiralia;Lophotrochozoa;Mollusca;Bivalvia;Autobranchia;Heteroconchia;Euheterodonta;Imparidentia;Venerida;Veneroidea;Veneridae;Mercenaria;Mercenaria mercenaria
UdeM00000025356 225164 161.4 UdeM00000025356 XP_009047492.1 51 145 70 1 11 155 41 184 9.40E-37 161.4 225164 cellular organisms;Eukaryota;Opisthokonta;Metazoa;Eumetazoa;Bilateria;Protostomia;Spiralia;Lophotrochozoa;Mollusca;Gastropoda;Patellogastropoda;Lottioidea;Lottiidae;Lottia;Lottia gigantea
UdeM00000025357 6596 229.6 UdeM00000025357 XP_045167641.1 53.9 193 86 1 131 323 33 222 5.80E-57 229.6 6596 cellular organisms;Eukaryota;Opisthokonta;Metazoa;Eumetazoa;Bilateria;Protostomia;Spiralia;Lophotrochozoa;Mollusca;Bivalvia;Autobranchia;Heteroconchia;Euheterodonta;Imparidentia;Venerida;Veneroidea;Veneridae;Mercenaria;Mercenaria mercenaria
UdeM00000025358 6596 232.3 UdeM00000025358 XP_045203987.1 44.4 304 153 6 2 289 224 527 1.60E-57 232.3 6596 cellular organisms;Eukaryota;Opisthokonta;Metazoa;Eumetazoa;Bilateria;Protostomia;Spiralia;Lophotrochozoa;Mollusca;Bivalvia;Autobranchia;Heteroconchia;Euheterodonta;Imparidentia;Venerida;Veneroidea;Veneridae;Mercenaria;Mercenaria mercenaria
UdeM00000025359 225164 162.5 UdeM00000025359 XP_009047492.1 51.7 145 69 1 18 162 41 184 4.40E-37 162.5 225164 cellular organisms;Eukaryota;Opisthokonta;Metazoa;Eumetazoa;Bilateria;Protostomia;Spiralia;Lophotrochozoa;Mollusca;Gastropoda;Patellogastropoda;Lottioidea;Lottiidae;Lottia;Lottia gigantea
UdeM00000025360 6596 228.8 UdeM00000025360 XP_045167641.1 56.1 187 79 1 137 323 39 222 9.90E-57 228.8 6596 cellular organisms;Eukaryota;Opisthokonta;Metazoa;Eumetazoa;Bilateria;Protostomia;

Spiralia;Lophotrochozoa;Mollusca;Bivalvia;Autobranchia;Heteroconchia;Euheterodonta;Imparidentia;Venerida;Veneroidea;Veneridae;Mercenaria;Mercenaria mercenaria
UdeM00000025361 6596 224.6 UdeM00000025361 XP_045176520.1 55.7 185 77 3 96 280 41 220 1.60E-55 224.6 6596 cellular organisms;Eukaryota;Opisthokonta;Metazoa;Eumetazoa;Bilateria;Protostomia;Spiralia;Lophotrochozoa;Mollusca;Bivalvia;Autobranchia;Heteroconchia;Euheterodonta;Imparidentia;Venerida;Veneroidea;Veneridae;Mercenaria;Mercenaria mercenaria
UdeM00000025362 299321 75.1 UdeM00000025362 XP_028821593.1 39 100 52 4 276 368 315 412 2.10E-10 75.1 299321 cellular organisms;Eukaryota;Opisthokonta;Metazoa;Eumetazoa;Bilateria;Deuterostomia;Chordata;Craniata;Vertebrata;Gnathostomata;Teleostomi;Euteleostomi;Actinopterygii;Actinopteri;Neopterygii;Teleostei;Osteoglossocephalai;Clupeocephala;Otomorpha;Clupei;Clupeiformes;Denticipitoidei;Denticipitidae;Denticeps;Denticeps clupeoides
UdeM00000044401 83332 1920.6 UdeM00000044401 NP_216564.2 52.1 2042 926 29 12 2039 32 2034 0.00E+00 1920.6 83332 cellular organisms;Bacteria;Terrabacteria group;Actinobacteria;Actinomycetia;Corynebacteriales;Mycobacteriaceae;Mycobacterium;Mycobacterium tuberculosis complex;Mycobacterium tuberculosis;Mycobacterium tuberculosis H37Rv
UdeM00000044402 83332 431.4 UdeM00000044402 NP_217615.1 75.2 278 69 0 4 281 6 283 8.80E-118 431.4 83332 cellular organisms;Bacteria;Terrabacteria group;Actinobacteria;Actinomycetia;Corynebacteriales;Mycobacteriaceae;Mycobacterium;Mycobacterium tuberculosis complex;Mycobacterium tuberculosis;Mycobacterium tuberculosis H37Rv
UdeM00000044403 83332 717.6 UdeM00000044403 NP_217655.1 87.7 407 50 0 1 407 62 468 8.90E-204 717.6 83332 cellular organisms;Bacteria;Terrabacteria group;Actinobacteria;Actinomycetia;Corynebacteriales;Mycobacteriaceae;Mycobacterium;Mycobacterium tuberculosis complex;Mycobacterium tuberculosis;Mycobacterium tuberculosis H37Rv
UdeM00000044272 6596 275.4 UdeM00000044272 XP_045164400.1 46.3 354 166 9 1 336 1 348 9.80E-71 275.4 6596 cellular organisms;Eukaryota;Opisthokonta;Metazoa;Eumetazoa;Bilateria;Protostomia;Spiralia;Lophotrochozoa;Mollusca;Bivalvia;Autobranchia;Heteroconchia;Euheterodonta;Imparidentia;Venerida;Veneroidea;Veneridae;Mercenaria;Mercenaria mercenaria
UdeM00000042648 29159 297 UdeM00000042648 XP_034308217.1 36.2 450 260 4 27 450 1 449 4.20E-77 297 29159 cellular organisms;Eukaryota;Opisthokonta;Metazoa;Eumetazoa;Bilateria;Protostomia;Spiralia;Lophotrochozoa;Mollusca;Bivalvia;Autobranchia;Pteriomorphia;Ostreida;Ostreoidea;Ostreidae;Crassostrea;Crassostrea gigas
UdeM00000042649 6596 227.3 UdeM00000042649 XP_045172539.1 29.4 449 271 6 16 419 24 471 3.70E-56 227.3 6596 cellular organisms;Eukaryota;Opisthokonta;Metazoa;Eumetazoa;Bilateria;Protostomia;Spiralia;Lophotrochozoa;Mollusca;Bivalvia;Autobranchia;Heteroconchia;Euheterodonta;Imparidentia;Venerida;Veneroidea;Veneridae;Mercenaria;Mercenaria mercenaria
UdeM00000042650 6596 226.5 UdeM00000042650 XP_045172539.1 29.5 448 270 6 1 403 25 471 6.10E-56 226.5 6596 cellular

organisms;Eukaryota;Opisthokonta;Metazoa;Eumetazoa;Bilateria;Protostomia;Spiralia;Lophotrochozoa;Mollusca;Bivalvia;Autobranchia;Heteroconchia;Euheterodonta;Imparidentia;Venerida;Veneroidea;Veneridae;Mercenaria;Mercenaria mercenaria
UdeM00000042651 6596 1432.2 UdeM00000042651 XP_045171548.1 67.5 1290 368 8 35 1278 24 1308 0.00E+00 1432.2 6596 cellular organisms;Eukaryota;Opisthokonta;Metazoa;Eumetazoa;Bilateria;Protostomia;Spiralia;Lophotrochozoa;Mollusca;Bivalvia;Autobranchia;Heteroconchia;Euheterodonta;Imparidentia;Venerida;Veneroidea;Veneridae;Mercenaria;Mercenaria mercenaria
UdeM00000042652 6573 469.2 UdeM00000042652 XP_021357899.1 61.5 403 142 4 1 397 1 396 7.70E-129 469.2 6573 cellular organisms;Eukaryota;Opisthokonta;Metazoa;Eumetazoa;Bilateria;Protostomia;Spiralia;Lophotrochozoa;Mollusca;Bivalvia;Autobranchia;Pteriomorphia;Pectinida;Pectinoidea;Pectinidae;Mizuhopecten;Mizuhopecten yessoensis
UdeM00000042653 6579 560.5 UdeM00000042653 XP_033748878.1 39.3 1008 383 28 54 1042 20 817 4.90E-156 560.5 6579 cellular organisms;Eukaryota;Opisthokonta;Metazoa;Eumetazoa;Bilateria;Protostomia;Spiralia;Lophotrochozoa;Mollusca;Bivalvia;Autobranchia;Pteriomorphia;Pectinida;Pectinoidea;Pectinidae;Pecten;Pecten maximus
UdeM00000042654 6596 179.5 UdeM00000042654 XP_045173316.1 30.8 701 329 24 30 640 905 1539 1.40E-41 179.5 6596 cellular organisms;Eukaryota;Opisthokonta;Metazoa;Eumetazoa;Bilateria;Protostomia;Spiralia;Lophotrochozoa;Mollusca;Bivalvia;Autobranchia;Heteroconchia;Euheterodonta;Imparidentia;Venerida;Veneroidea;Veneridae;Mercenaria;Mercenaria mercenaria
UdeM00000042655 7741 168.3 UdeM00000042655 XP_019633445.1 34.6 269 148 3 3 243 13 281 1.50E-38 168.3 7741 cellular organisms;Eukaryota;Opisthokonta;Metazoa;Eumetazoa;Bilateria;Deuterostomia;Chordata;Cephalochordata;Leptocardii;Amphioxiformes;Branchiostomidae;Branchiostoma;Branchiostoma belcheri
UdeM00000042656 6565 72.4 UdeM00000042656 XP_022345136.1 71.8 294 75 3 10 300 11 299 1.30E-09 72.4 6565 cellular organisms;Eukaryota;Opisthokonta;Metazoa;Eumetazoa;Bilateria;Protostomia;Spiralia;Lophotrochozoa;Mollusca;Bivalvia;Autobranchia;Pteriomorphia;Ostreida;Ostreoidea;Ostreidae;Crassostrea;Crassostrea virginica
UdeM00000042657 6596 194.5 UdeM00000042657 XP_045172099.1 77 178 29 4 13 181 25 199 1.20E-46 194.5 6596 cellular organisms;Eukaryota;Opisthokonta;Metazoa;Eumetazoa;Bilateria;Protostomia;Spiralia;Lophotrochozoa;Mollusca;Bivalvia;Autobranchia;Heteroconchia;Euheterodonta;Imparidentia;Venerida;Veneroidea;Veneridae;Mercenaria;Mercenaria mercenaria
UdeM00000042658 29159 78.2 UdeM00000042658 XP_034339368.1 68.5 143 40 2 1 139 1 142 9.50E-12 78.2 29159 cellular organisms;Eukaryota;Opisthokonta;Metazoa;Eumetazoa;Bilateria;Protostomia;Spiralia;Lophotrochozoa;Mollusca;Bivalvia;Autobranchia;Pteriomorphia;Ostreida;Ostreoidea;Ostreidae;Crassostrea;Crassostrea gigas
UdeM00000042659 29159 327 UdeM00000042659 XP_034339368.1 57.5 327 118 6 18 333 364 680 2.80E-86 327 29159 cellular organisms;Eukaryota;Opisthokonta;Metazoa;Eumetazoa;Bilateria;Protostomia;Spiralia;Lophotrochozoa;Mollusca;Bivalvia;Autobranchia;Pteriomorphia;Ostreida;Ostreoidea;Ostreidae;Crassostrea;Crassostrea gigas

UdeM00000042660 6596 235 UdeM00000042660 XP_045172084.1 45.6 298
152 3 6 297 1311 1604 1.40E-58 235 6596 cellular
organisms;Eukaryota;Opisthokonta;Metazoa;Eumetazoa;Bilateria;Protostomia;
Spiralia;Lophotrochozoa;Mollusca;Bivalvia;Autobranchia;Heteroconchia;Euhe
terodonta;Imparidentia;Venerida;Veneroidea;Veneridae;Mercenaria;Mercenari
a mercenaria
UdeM00000042661 6596 362.8 UdeM00000042661 XP_045172084.1 57.2 341
126 6 206 531 1819 2154 7.20E-97 362.8 6596 cellular
organisms;Eukaryota;Opisthokonta;Metazoa;Eumetazoa;Bilateria;Protostomia;
Spiralia;Lophotrochozoa;Mollusca;Bivalvia;Autobranchia;Heteroconchia;Euhe
terodonta;Imparidentia;Venerida;Veneroidea;Veneridae;Mercenaria;Mercenari
a mercenaria
UdeM00000042662 6596 176.8 UdeM00000042662 XP_045187622.1 36 328
187 5 1 309 72 395 4.30E-41 176.8 6596 cellular
organisms;Eukaryota;Opisthokonta;Metazoa;Eumetazoa;Bilateria;Protostomia;
Spiralia;Lophotrochozoa;Mollusca;Bivalvia;Autobranchia;Heteroconchia;Euhe
terodonta;Imparidentia;Venerida;Veneroidea;Veneridae;Mercenaria;Mercenari
a mercenaria
UdeM00000042663 6596 239.6 UdeM00000042663 XP_045165062.1 38.6 370
203 3 10 373 7 358 6.60E-60 239.6 6596 cellular
organisms;Eukaryota;Opisthokonta;Metazoa;Eumetazoa;Bilateria;Protostomia;
Spiralia;Lophotrochozoa;Mollusca;Bivalvia;Autobranchia;Heteroconchia;Euhe
terodonta;Imparidentia;Venerida;Veneroidea;Veneridae;Mercenaria;Mercenari
a mercenaria
UdeM00000042664 6596 284.6 UdeM00000042664 XP_045173842.1 81.7 164
30 0 45 208 413 576 9.90E-74 284.6 6596 cellular
organisms;Eukaryota;Opisthokonta;Metazoa;Eumetazoa;Bilateria;Protostomia;
Spiralia;Lophotrochozoa;Mollusca;Bivalvia;Autobranchia;Heteroconchia;Euhe
terodonta;Imparidentia;Venerida;Veneroidea;Veneridae;Mercenaria;Mercenari
a mercenaria
UdeM00000042665 6596 217.6 UdeM00000042665 XP_045165713.1 36.2 398
224 6 7 383 4 392 2.80E-53 217.6 6596 cellular
organisms;Eukaryota;Opisthokonta;Metazoa;Eumetazoa;Bilateria;Protostomia;
Spiralia;Lophotrochozoa;Mollusca;Bivalvia;Autobranchia;Heteroconchia;Euhe
terodonta;Imparidentia;Venerida;Veneroidea;Veneridae;Mercenaria;Mercenari
a mercenaria
UdeM00000042666 6596 475.3 UdeM00000042666 XP_045173842.1 65.2 405
124 6 2 390 9 412 9.90E-131 475.3 6596 cellular
organisms;Eukaryota;Opisthokonta;Metazoa;Eumetazoa;Bilateria;Protostomia;
Spiralia;Lophotrochozoa;Mollusca;Bivalvia;Autobranchia;Heteroconchia;Euhe
terodonta;Imparidentia;Venerida;Veneroidea;Veneridae;Mercenaria;Mercenari
a mercenaria
UdeM00000042667 6596 576.6 UdeM00000042667 XP_045171724.1 40.5 1112
437 22 16 1007 24 1030 6.10E-161 576.6 6596 cellular
organisms;Eukaryota;Opisthokonta;Metazoa;Eumetazoa;Bilateria;Protostomia;
Spiralia;Lophotrochozoa;Mollusca;Bivalvia;Autobranchia;Heteroconchia;Euhe
terodonta;Imparidentia;Venerida;Veneroidea;Veneridae;Mercenaria;Mercenari
a mercenaria
UdeM00000042668 6596 417.9 UdeM00000042668 XP_045171724.1 39.1 911
377 18 1 792 179 1030 2.80E-113 417.9 6596 cellular
organisms;Eukaryota;Opisthokonta;Metazoa;Eumetazoa;Bilateria;Protostomia;
Spiralia;Lophotrochozoa;Mollusca;Bivalvia;Autobranchia;Heteroconchia;Euhe
terodonta;Imparidentia;Venerida;Veneroidea;Veneridae;Mercenaria;Mercenari
a mercenaria

UdeM00000042670 6596 489.2 UdeM00000042670 XP_045173683.1 55.4 415
175 3 36 440 40 454 5.60E-135 489.2 6596 cellular
organisms;Eukaryota;Opisthokonta;Metazoa;Eumetazoa;Bilateria;Protostomia;
Spiralia;Lophotrochozoa;Mollusca;Bivalvia;Autobranchia;Heteroconchia;Euhe
terodonta;Imparidentia;Venerida;Veneroidea;Veneridae;Mercenaria;Mercenari
a mercenaria
UdeM00000042669 6596 493.8 UdeM00000042669 XP_045173683.1 51.5 470
193 5 5 463 9 454 2.40E-136 493.8 6596 cellular
organisms;Eukaryota;Opisthokonta;Metazoa;Eumetazoa;Bilateria;Protostomia;
Spiralia;Lophotrochozoa;Mollusca;Bivalvia;Autobranchia;Heteroconchia;Euhe
terodonta;Imparidentia;Venerida;Veneroidea;Veneridae;Mercenaria;Mercenari
a mercenaria
UdeM00000042671 6596 450.7 UdeM00000042671 XP_045171372.1 59.4 340
131 3 308 642 226 563 3.70E-123 450.7 6596 cellular
organisms;Eukaryota;Opisthokonta;Metazoa;Eumetazoa;Bilateria;Protostomia;
Spiralia;Lophotrochozoa;Mollusca;Bivalvia;Autobranchia;Heteroconchia;Euhe
terodonta;Imparidentia;Venerida;Veneroidea;Veneridae;Mercenaria;Mercenari
a mercenaria
UdeM00000042672 6596 81.6 UdeM00000042672 XP_045173596.1 40 115
69 0 15 129 12 126 2.60E-12 81.6 6596 cellular
organisms;Eukaryota;Opisthokonta;Metazoa;Eumetazoa;Bilateria;Protostomia;
Spiralia;Lophotrochozoa;Mollusca;Bivalvia;Autobranchia;Heteroconchia;Euhe
terodonta;Imparidentia;Venerida;Veneroidea;Veneridae;Mercenaria;Mercenari
a mercenaria
UdeM00000042673 6596 334 UdeM00000042673 XP_045171372.1 38.5 410
112 1 39 448 268 537 3.80E-88 334 6596 cellular
organisms;Eukaryota;Opisthokonta;Metazoa;Eumetazoa;Bilateria;Protostomia;
Spiralia;Lophotrochozoa;Mollusca;Bivalvia;Autobranchia;Heteroconchia;Euhe
terodonta;Imparidentia;Venerida;Veneroidea;Veneridae;Mercenaria;Mercenari
a mercenaria
UdeM00000042674 6565 116.3 UdeM00000042674 XP_022336588.1 37.7 159
79 3 5 163 88 226 3.80E-23 116.3 6565 cellular
organisms;Eukaryota;Opisthokonta;Metazoa;Eumetazoa;Bilateria;Protostomia;
Spiralia;Lophotrochozoa;Mollusca;Bivalvia;Autobranchia;Pteriomorphia;Ostr
eida;Ostreoidea;Ostreidae;Crassostrea;Crassostrea virginica
UdeM00000042675 6596 312.4 UdeM00000042675 XP_045171505.1 41.5 407
224 6 1 397 180 582 8.40E-82 312.4 6596 cellular
organisms;Eukaryota;Opisthokonta;Metazoa;Eumetazoa;Bilateria;Protostomia;
Spiralia;Lophotrochozoa;Mollusca;Bivalvia;Autobranchia;Heteroconchia;Euhe
terodonta;Imparidentia;Venerida;Veneroidea;Veneridae;Mercenaria;Mercenari
a mercenaria
UdeM00000042676 45351 1013.8 UdeM00000042676 XP_032218909.1 43.3
1029 499 3 89 1112 850 1799 1.90E-292 1013.8
45351 cellular
organisms;Eukaryota;Opisthokonta;Metazoa;Eumetazoa;Cnidaria;Anthozoa;Hexa
corallia;Actiniaria;Edwardsiidae;Nematostella;Nematostella vectensis
UdeM00000043793 6579 500.4 UdeM00000043793 XP_033742825.1 49.7 541
183 8 19 492 304 822 2.70E-138 500.4 6579 cellular
organisms;Eukaryota;Opisthokonta;Metazoa;Eumetazoa;Bilateria;Protostomia;
Spiralia;Lophotrochozoa;Mollusca;Bivalvia;Autobranchia;Pteriomorphia;Pect
inida;Pectinoidea;Pectinidae;Pecten;Pecten maximus
UdeM00000043794 6500 118.2 UdeM00000043794 XP_012944219.2 50.8 118
44 1 1 104 720 837 1.10E-23 118.2 6500 cellular
organisms;Eukaryota;Opisthokonta;Metazoa;Eumetazoa;Bilateria;Protostomia;

Spiralia;Lophotrochozoa;Mollusca;Gastropoda;Heterobranchia;Euthyneura;Tectipleura;Aplysiida;Aplysioidea;Aplysiidae;Aplysia;Aplysia californica
UdeM00000043795 6596 136 UdeM00000043795 XP_045165299.1 37.6 189 110 6 2 187 110 293 5.30E-29 136 6596 cellular organisms;Eukaryota;Opisthokonta;Metazoa;Eumetazoa;Bilateria;Protostomia;Spiralia;Lophotrochozoa;Mollusca;Bivalvia;Autobranchia;Heteroconchia;Euheterodonta;Imparidentia;Venerida;Veneroidea;Veneridae;Mercenaria;Mercenaria mercenaria
UdeM00000043796 6579 436.8 UdeM00000043796 XP_033742373.1 43.7 460 253 3 12 467 24 481 3.50E-119 436.8 6579 cellular organisms;Eukaryota;Opisthokonta;Metazoa;Eumetazoa;Bilateria;Protostomia;Spiralia;Lophotrochozoa;Mollusca;Bivalvia;Autobranchia;Pteriomorphia;Pectinida;Pectinoidea;Pectinidae;Pecten;Pecten maximus
UdeM00000043797 7604 760.4 UdeM00000043797 XP_033647599.1 31.2 2202 1308 58 636 2712 326 2446 8.10E-216 760.4 7604 cellular organisms;Eukaryota;Opisthokonta;Metazoa;Eumetazoa;Bilateria;Deuterostomia;Echinodermata;Eleutherozoa;Asterozoa;Asteroidea;Forcipulatacea;Forcipulatida;Asteriidae;Asterias;Asterias rubens
UdeM00000043798 6579 138.3 UdeM00000043798 XP_033743120.1 38.1 215 91 5 1 180 1 208 1.70E-29 138.3 6579 cellular organisms;Eukaryota;Opisthokonta;Metazoa;Eumetazoa;Bilateria;Protostomia;Spiralia;Lophotrochozoa;Mollusca;Bivalvia;Autobranchia;Pteriomorphia;Pectinida;Pectinoidea;Pectinidae;Pecten;Pecten maximus
UdeM00000043801 6596 137.5 UdeM00000043801 XP_045204388.1 35.5 273 156 8 24 280 22 290 2.80E-29 137.5 6596 cellular organisms;Eukaryota;Opisthokonta;Metazoa;Eumetazoa;Bilateria;Protostomia;Spiralia;Lophotrochozoa;Mollusca;Bivalvia;Autobranchia;Heteroconchia;Euheterodonta;Imparidentia;Venerida;Veneroidea;Veneridae;Mercenaria;Mercenaria mercenaria
UdeM00000043802 6565 112.5 UdeM00000043802 XP_022334997.1 39.9 153 85 3 33 181 29 178 9.70E-22 112.5 6565 cellular organisms;Eukaryota;Opisthokonta;Metazoa;Eumetazoa;Bilateria;Protostomia;Spiralia;Lophotrochozoa;Mollusca;Bivalvia;Autobranchia;Pteriomorphia;Ostreida;Ostreoidea;Ostreidae;Crassostrea;Crassostrea virginica
UdeM00000043803 7604 114.8 UdeM00000043803 XP_033647599.1 39.9 288 157 1 3 274 1405 1692 1.80E-22 114.8 7604 cellular organisms;Eukaryota;Opisthokonta;Metazoa;Eumetazoa;Bilateria;Deuterostomia;Echinodermata;Eleutherozoa;Asterozoa;Asteroidea;Forcipulatacea;Forcipulatida;Asteriidae;Asterias;Asterias rubens
UdeM00000043805 6596 122.5 UdeM00000043805 XP_045202576.1 59.3 108 43 1 1 107 87 194 3.80E-25 122.5 6596 cellular organisms;Eukaryota;Opisthokonta;Metazoa;Eumetazoa;Bilateria;Protostomia;Spiralia;Lophotrochozoa;Mollusca;Bivalvia;Autobranchia;Heteroconchia;Euheterodonta;Imparidentia;Venerida;Veneroidea;Veneridae;Mercenaria;Mercenaria mercenaria
UdeM00000043806 6596 177.6 UdeM00000043806 XP_045206316.1 47.6 191 99 1 13 202 70 260 1.60E-41 177.6 6596 cellular organisms;Eukaryota;Opisthokonta;Metazoa;Eumetazoa;Bilateria;Protostomia;Spiralia;Lophotrochozoa;Mollusca;Bivalvia;Autobranchia;Heteroconchia;Euheterodonta;Imparidentia;Venerida;Veneroidea;Veneridae;Mercenaria;Mercenaria mercenaria
UdeM00000035947 29159 62.8 UdeM00000035947 XP_034307341.1 30.5 128 63 2 2 109 48 169 3.20E-07 62.8 29159 cellular organisms;Eukaryota;Opisthokonta;Metazoa;Eumetazoa;Bilateria;Protostomia;

Spiralia;Lophotrochozoa;Mollusca;Bivalvia;Autobranchia;Pteriomorphia;Ostreida;Ostreoidea;Ostreidae;Crassostrea;Crassostrea gigas
UdeM00000035948 1735272 80.9 UdeM00000035948 XP_041353177.1 44.3 79 44 0 1 79 148 226 1.90E-12 80.9 1735272 cellular organisms;Eukaryota;Opisthokonta;Metazoa;Eumetazoa;Bilateria;Protostomia;Spiralia;Lophotrochozoa;Mollusca;Gastropoda;Neomphalina;Neomphaloidea;Peltospiridae;Gigantopelta;Gigantopelta aegis
UdeM00000035951 29159 310.8 UdeM00000035951 XP_034305965.1 38.8 466 215 15 6 433 7 440 2.80E-81 310.8 29159 cellular organisms;Eukaryota;Opisthokonta;Metazoa;Eumetazoa;Bilateria;Protostomia;Spiralia;Lophotrochozoa;Mollusca;Bivalvia;Autobranchia;Pteriomorphia;Ostreida;Ostreoidea;Ostreidae;Crassostrea;Crassostrea gigas
UdeM00000035953 6565 198.7 UdeM00000035953 XP_022333382.1 52.6 173 81 1 1 173 189 360 9.90E-48 198.7 6565 cellular organisms;Eukaryota;Opisthokonta;Metazoa;Eumetazoa;Bilateria;Protostomia;Spiralia;Lophotrochozoa;Mollusca;Bivalvia;Autobranchia;Pteriomorphia;Ostreida;Ostreoidea;Ostreidae;Crassostrea;Crassostrea virginica
UdeM00000035954 46514 115.5 UdeM00000035954 XP_038071378.1 56.5 108 47 0 7 114 6 113 4.40E-23 115.5 46514 cellular organisms;Eukaryota;Opisthokonta;Metazoa;Eumetazoa;Bilateria;Deuterostomia;Echinodermata;Eleutherozoa;Asterozoa;Asteroidea;Valvatacea;Valvatida;Asterinidae;Patiria;Patiria miniata
UdeM00000035955 6706 152.1 UdeM00000035955 XP_042235765.1 43.9 157 88 0 1 157 1 157 5.80E-34 152.1 6706 cellular organisms;Eukaryota;Opisthokonta;Metazoa;Eumetazoa;Bilateria;Protostomia;Ecdysozoa;Panarthropoda;Arthropoda;Mandibulata;Pancrustacea;Crustacea;Multicrustacea;Malacostraca;Eumalacostraca;Eucarida;Decapoda;Pleocyemata;Astacidea;Nephropoidea;Nephropidae;Homarus;Homarus americanus
UdeM00000035959 6565 216.9 UdeM00000035959 XP_022298930.1 42 276 142 6 26 287 10 281 3.70E-53 216.9 6565 cellular organisms;Eukaryota;Opisthokonta;Metazoa;Eumetazoa;Bilateria;Protostomia;Spiralia;Lophotrochozoa;Mollusca;Bivalvia;Autobranchia;Pteriomorphia;Ostreida;Ostreoidea;Ostreidae;Crassostrea;Crassostrea virginica
UdeM00000035960 7998 437.2 UdeM00000035960 XP_017334791.1 64.6 319 113 0 1 319 62 380 1.80E-119 437.2 7998 cellular organisms;Eukaryota;Opisthokonta;Metazoa;Eumetazoa;Bilateria;Deuterostomia;Chordata;Craniata;Vertebrata;Gnathostomata;Teleostomi;Euteleostomi;Actinopterygii;Actinopteri;Neopterygii;Teleostei;Osteoglossocephalai;Clupeocephala;Otomorpha;Ostariophysi;Otophysi;Characiphysae;Siluriformes;Siluroidei;Ictaluridae;Ictalurus;Ictalurus punctatus
UdeM00000035961 6579 157.9 UdeM00000035961 XP_033752778.1 40.9 264 128 7 2 255 15 260 1.70E-35 157.9 6579 cellular organisms;Eukaryota;Opisthokonta;Metazoa;Eumetazoa;Bilateria;Protostomia;Spiralia;Lophotrochozoa;Mollusca;Bivalvia;Autobranchia;Pteriomorphia;Pectinida;Pectinoidea;Pectinidae;Pecten;Pecten maximus
UdeM00000035962 6565 93.6 UdeM00000035962 XP_022312527.1 44.8 96 52 1 69 163 70 165 2.70E-16 93.6 6565 cellular organisms;Eukaryota;Opisthokonta;Metazoa;Eumetazoa;Bilateria;Protostomia;Spiralia;Lophotrochozoa;Mollusca;Bivalvia;Autobranchia;Pteriomorphia;Ostreida;Ostreoidea;Ostreidae;Crassostrea;Crassostrea virginica
UdeM00000035963 7054 480.7 UdeM00000035963 XP_031328250.1 49.7 467 233 2 1 465 1 467 2.10E-132 480.7 7054 cellular organisms;Eukaryota;Opisthokonta;Metazoa;Eumetazoa;Bilateria;Protostomia;

Ecdysozoa;Panarthropoda;Arthropoda;Mandibulata;Pancrustacea;Hexapoda;Insecta;Dicondylia;Pterygota;Neoptera;Endopterygota;Coleoptera;Polyphaga;Elateriformia;Elateroidea;Lampyridae;Lampyrinae;Photinus;Photinus pyralis
UdeM00000035965 6706 151 UdeM00000035965 XP_042235765.1 43.9 157
88 0 1 157 1 157 1.30E-33 151 6706 cellular
organisms;Eukaryota;Opisthokonta;Metazoa;Eumetazoa;Bilateria;Protostomia;Ecdysozoa;Panarthropoda;Arthropoda;Mandibulata;Pancrustacea;Crustacea;Multicrustacea;Malacostraca;Eumalacostraca;Eucarida;Decapoda;Pleocyemata;Astacidea;Nephropoidea;Nephropidae;Homarus;Homarus americanus
UdeM00000035966 6596 62.4 UdeM00000035966 XP_045210831.1 31.2 141
40 2 1 84 1 141 3.20E-07 62.4 6596 cellular
organisms;Eukaryota;Opisthokonta;Metazoa;Eumetazoa;Bilateria;Protostomia;Spiralia;Lophotrochozoa;Mollusca;Bivalvia;Autobranchia;Heteroconchia;Euheterodonta;Imparidentia;Venerida;Veneroidea;Veneridae;Mercenaria;Mercenaria mercenaria
UdeM00000035967 6596 481.1 UdeM00000035967 XP_045187150.1 49.4 488
242 2 1 488 135 617 1.70E-132 481.1 6596 cellular
organisms;Eukaryota;Opisthokonta;Metazoa;Eumetazoa;Bilateria;Protostomia;Spiralia;Lophotrochozoa;Mollusca;Bivalvia;Autobranchia;Heteroconchia;Euheterodonta;Imparidentia;Venerida;Veneroidea;Veneridae;Mercenaria;Mercenaria mercenaria
UdeM00000035968 6596 622.1 UdeM00000035968 XP_045190527.1 48 656
330 5 57 707 3 652 8.80E-175 622.1 6596 cellular
organisms;Eukaryota;Opisthokonta;Metazoa;Eumetazoa;Bilateria;Protostomia;Spiralia;Lophotrochozoa;Mollusca;Bivalvia;Autobranchia;Heteroconchia;Euheterodonta;Imparidentia;Venerida;Veneroidea;Veneridae;Mercenaria;Mercenaria mercenaria
UdeM00000035969 6565 836.6 UdeM00000035969 XP_022334993.1 49.4 887
413 13 39 914 8 869 2.90E-239 836.6 6565 cellular
organisms;Eukaryota;Opisthokonta;Metazoa;Eumetazoa;Bilateria;Protostomia;Spiralia;Lophotrochozoa;Mollusca;Bivalvia;Autobranchia;Pteriomorphia;Ostreida;Ostreoidea;Ostreidae;Crassostrea;Crassostrea virginica
UdeM00000035970 6565 212.6 UdeM00000035970 XP_022287303.1 34.2 310
190 7 6 303 443 750 6.90E-52 212.6 6565 cellular
organisms;Eukaryota;Opisthokonta;Metazoa;Eumetazoa;Bilateria;Protostomia;Spiralia;Lophotrochozoa;Mollusca;Bivalvia;Autobranchia;Pteriomorphia;Ostreida;Ostreoidea;Ostreidae;Crassostrea;Crassostrea virginica
UdeM00000035971 6565 132.1 UdeM00000035971 XP_022334997.1 44.8 154
80 3 6 156 29 180 6.20E-28 132.1 6565 cellular
organisms;Eukaryota;Opisthokonta;Metazoa;Eumetazoa;Bilateria;Protostomia;Spiralia;Lophotrochozoa;Mollusca;Bivalvia;Autobranchia;Pteriomorphia;Ostreida;Ostreoidea;Ostreidae;Crassostrea;Crassostrea virginica
UdeM00000035972 6565 129.8 UdeM00000035972 XP_022334997.1 44.7 152
79 3 6 154 29 178 8.60E-27 129.8 6565 cellular
organisms;Eukaryota;Opisthokonta;Metazoa;Eumetazoa;Bilateria;Protostomia;Spiralia;Lophotrochozoa;Mollusca;Bivalvia;Autobranchia;Pteriomorphia;Ostreida;Ostreoidea;Ostreidae;Crassostrea;Crassostrea virginica
UdeM00000035973 6565 746.9 UdeM00000035973 XP_022334994.1 46.8 870
364 13 36 811 4 868 2.70E-212 746.9 6565 cellular
organisms;Eukaryota;Opisthokonta;Metazoa;Eumetazoa;Bilateria;Protostomia;Spiralia;Lophotrochozoa;Mollusca;Bivalvia;Autobranchia;Pteriomorphia;Ostreida;Ostreoidea;Ostreidae;Crassostrea;Crassostrea virginica
UdeM00000035974 6596 145.2 UdeM00000035974 XP_045209729.1 46.4 168
77 5 2 157 570 736 7.00E-32 145.2 6596 cellular

organisms;Eukaryota;Opisthokonta;Metazoa;Eumetazoa;Bilateria;Protostomia;Spiralia;Lophotrochozoa;Mollusca;Bivalvia;Autobranchia;Heteroconchia;Euheterodonta;Imparidentia;Venerida;Veneroidea;Veneridae;Mercenaria;Mercenaria mercenaria
UdeM00000035975 29159 68.6 UdeM00000035975 XP_034318137.1 42.6 61 35 0 39 99 811 871 5.30E-09 68.6 29159 cellular organisms;Eukaryota;Opisthokonta;Metazoa;Eumetazoa;Bilateria;Protostomia;Spiralia;Lophotrochozoa;Mollusca;Bivalvia;Autobranchia;Pteriomorphia;Ostreida;Ostreoidea;Ostreidae;Crassostrea;Crassostrea gigas
UdeM00000035976 6596 86.3 UdeM00000035976 XP_045208792.1 37.6 109 67 1 1 109 1 108 2.80E-14 86.3 6596 cellular organisms;Eukaryota;Opisthokonta;Metazoa;Eumetazoa;Bilateria;Protostomia;Spiralia;Lophotrochozoa;Mollusca;Bivalvia;Autobranchia;Heteroconchia;Euheterodonta;Imparidentia;Venerida;Veneroidea;Veneridae;Mercenaria;Mercenaria mercenaria
UdeM00000035977 6565 169.1 UdeM00000035977 XP_022337267.1 40.1 267 99 6 56 261 300 566 7.80E-39 169.1 6565 cellular organisms;Eukaryota;Opisthokonta;Metazoa;Eumetazoa;Bilateria;Protostomia;Spiralia;Lophotrochozoa;Mollusca;Bivalvia;Autobranchia;Pteriomorphia;Ostreida;Ostreoidea;Ostreidae;Crassostrea;Crassostrea virginica
UdeM00000035978 6565 163.3 UdeM00000035978 XP_022337267.1 50.3 167 81 1 49 213 400 566 3.50E-37 163.3 6565 cellular organisms;Eukaryota;Opisthokonta;Metazoa;Eumetazoa;Bilateria;Protostomia;Spiralia;Lophotrochozoa;Mollusca;Bivalvia;Autobranchia;Pteriomorphia;Ostreida;Ostreoidea;Ostreidae;Crassostrea;Crassostrea virginica
UdeM00000035979 6565 154.1 UdeM00000035979 XP_022337267.1 65.5 113 39 0 2 114 454 566 1.20E-34 154.1 6565 cellular organisms;Eukaryota;Opisthokonta;Metazoa;Eumetazoa;Bilateria;Protostomia;Spiralia;Lophotrochozoa;Mollusca;Bivalvia;Autobranchia;Pteriomorphia;Ostreida;Ostreoidea;Ostreidae;Crassostrea;Crassostrea virginica
UdeM00000035982 6596 375.2 UdeM00000035982 XP_045169782.1 53.9 332 151 2 1 331 56 386 8.90E-101 375.2 6596 cellular organisms;Eukaryota;Opisthokonta;Metazoa;Eumetazoa;Bilateria;Protostomia;Spiralia;Lophotrochozoa;Mollusca;Bivalvia;Autobranchia;Heteroconchia;Euheterodonta;Imparidentia;Venerida;Veneroidea;Veneridae;Mercenaria;Mercenaria mercenaria
UdeM00000035986 6596 326.6 UdeM00000035986 XP_045181000.1 60 452 113 4 2 399 1140 1577 4.40E-86 326.6 6596 cellular organisms;Eukaryota;Opisthokonta;Metazoa;Eumetazoa;Bilateria;Protostomia;Spiralia;Lophotrochozoa;Mollusca;Bivalvia;Autobranchia;Heteroconchia;Euheterodonta;Imparidentia;Venerida;Veneroidea;Veneridae;Mercenaria;Mercenaria mercenaria
UdeM00000035987 6596 192.2 UdeM00000035987 XP_045181000.1 56 193 49 4 2 176 868 1042 5.80E-46 192.2 6596 cellular organisms;Eukaryota;Opisthokonta;Metazoa;Eumetazoa;Bilateria;Protostomia;Spiralia;Lophotrochozoa;Mollusca;Bivalvia;Autobranchia;Heteroconchia;Euheterodonta;Imparidentia;Venerida;Veneroidea;Veneridae;Mercenaria;Mercenaria mercenaria
UdeM00000035989 7054 402.1 UdeM00000035989 XP_031328250.1 45 467 221 4 1 431 1 467 8.70E-109 402.1 7054 cellular organisms;Eukaryota;Opisthokonta;Metazoa;Eumetazoa;Bilateria;Protostomia;Ecdysozoa;Panarthropoda;Arthropoda;Mandibulata;Pancrustacea;Hexapoda;Insecta;Dicondylia;Pterygota;Neoptera;Endopterygota;Coleoptera;Polyphaga;Elateriformia;Elateroidea;Lampyridae;Lampyrinae;Photinus;Photinus pyralis

UdeM00000028285	50390	427.9	UdeM00000028285	XP_028154228.1	58.4	296
123	0	3	298	326	621	1.00E-116	427.9	50390	cellular organisms;Eukaryota;Opisthokonta;Metazoa;Eumetazoa;Bilateria;Protostomia;Ecdysozoa;Panarthropoda;Arthropoda;Mandibulata;Pancrustacea;Hexapoda;Insecta;Dicondylia;Pterygota;Neoptera;Endopterygota;Coleoptera;Polyphaga;Cucujiformia;Chrysomeloidea;Chrysomelidae;Galerucinae;Luperini;Diabroticina;Diabroticites;Diabrotica;Diabrotica virgifera;Diabrotica virgifera virgifera
UdeM00000028286	50390	401.4	UdeM00000028286	XP_028142044.1	57.1	294
126	0	2	295	334	627	1.00E-108	401.4	50390	cellular organisms;Eukaryota;Opisthokonta;Metazoa;Eumetazoa;Bilateria;Protostomia;Ecdysozoa;Panarthropoda;Arthropoda;Mandibulata;Pancrustacea;Hexapoda;Insecta;Dicondylia;Pterygota;Neoptera;Endopterygota;Coleoptera;Polyphaga;Cucujiformia;Chrysomeloidea;Chrysomelidae;Galerucinae;Luperini;Diabroticina;Diabroticites;Diabrotica;Diabrotica virgifera;Diabrotica virgifera virgifera
UdeM00000028287	80966	184.5	UdeM00000028287	XP_022077561.1	54.5	156
71	0	1	156	116	271	1.00E-43	184.5	80966	cellular organisms;Eukaryota;Opisthokonta;Metazoa;Eumetazoa;Bilateria;Deuterostomia;Chordata;Craniata;Vertebrata;Gnathostomata;Teleostomi;Euteleostomi;Actinopterygii;Actinopteri;Neopterygii;Teleostei;Osteoglossocephalai;Clupeocephala;Euteleosteomorpha;Neoteleostei;Eurypterygia;Ctenosquamata;Acanthomorphata;Euacanthomorphacea;Percomorphaceae;Ovalentaria;Ovalentaria incertae sedis;Pomacentridae;Acanthochromis;Acanthochromis polyacanthus
UdeM00000028288	6596	426.4	UdeM00000028288	XP_045159152.1	66.1	327
111	0	55	381	52	378	3.90E-116	426.4	6596	cellular organisms;Eukaryota;Opisthokonta;Metazoa;Eumetazoa;Bilateria;Protostomia;Spiralia;Lophotrochozoa;Mollusca;Bivalvia;Autobranchia;Heteroconchia;Euheterodonta;Imparidentia;Venerida;Veneroidea;Veneridae;Mercenaria;Mercenaria mercenaria
UdeM00000028291	6596	1008.1	UdeM00000028291	XP_045162416.1	70.5
803	199	8	1	784	1	784	6.30E-291	1008.1	6596
cellular
organisms;Eukaryota;Opisthokonta;Metazoa;Eumetazoa;Bilateria;Protostomia;Spiralia;Lophotrochozoa;Mollusca;Bivalvia;Autobranchia;Heteroconchia;Euheterodonta;Imparidentia;Venerida;Veneroidea;Veneridae;Mercenaria;Mercenaria mercenaria
UdeM00000028290	6596	989.6	UdeM00000028290	XP_045162416.1	70.1	792
199	8	1	773	1	773	2.40E-285	989.6	6596	cellular organisms;Eukaryota;Opisthokonta;Metazoa;Eumetazoa;Bilateria;Protostomia;Spiralia;Lophotrochozoa;Mollusca;Bivalvia;Autobranchia;Heteroconchia;Euheterodonta;Imparidentia;Venerida;Veneroidea;Veneridae;Mercenaria;Mercenaria mercenaria
UdeM00000028292	225164	367.9	UdeM00000028292	XP_009058784.1	39
549	321	10	15	555	5	547	2.40E-98	367.9	225164
cellular
organisms;Eukaryota;Opisthokonta;Metazoa;Eumetazoa;Bilateria;Protostomia;Spiralia;Lophotrochozoa;Mollusca;Gastropoda;Patellogastropoda;Lottioidea;Lottiidae;Lottia;Lottia gigantea
UdeM00000028293	7574	72.8	UdeM00000028293	XP_013383769.1	32.3	124
80	2	12	131	172	295	3.80E-10	72.8	7574	cellular organisms;Eukaryota;Opisthokonta;Metazoa;Eumetazoa;Bilateria;Protostomia;Spiralia;Lophotrochozoa;Brachiopoda;Linguliformea;Lingulata;Lingulida;Linguloidea;Lingulidae;Lingula;Lingula anatina

UdeM00000028294 7668 83.6 UdeM00000028294 XP_030847066.1 42.9 105
52 1 5 101 469 573 1.60E-13 83.6 7668 cellular
organisms;Eukaryota;Opisthokonta;Metazoa;Eumetazoa;Bilateria;Deuterostomi
a;Echinodermata;Eleutherozoa;Echinozoa;Echinoidea;Euechinoidea;Echinacea;
Camarodonta;Echinidea;Strongylocentrotidae;Strongylocentrotus;Strongyloce
ntrotus purpuratus
UdeM00000028295 6573 168.3 UdeM00000028295 XP_021372870.1 40 205
121 2 6 209 624 827 1.10E-38 168.3 6573 cellular
organisms;Eukaryota;Opisthokonta;Metazoa;Eumetazoa;Bilateria;Protostomia;
Spiralia;Lophotrochozoa;Mollusca;Bivalvia;Autobranchia;Pteriomorphia;Pect
inida;Pectinoidea;Pectinidae;Mizuhopecten;Mizuhopecten yessoensis
UdeM00000028296 27687 448 UdeM00000028296 XP_028667911.1 28.8 1082
684 27 10 1062 29 1053 3.40E-122 448 27687 cellular
organisms;Eukaryota;Opisthokonta;Metazoa;Eumetazoa;Bilateria;Deuterostomi
a;Chordata;Craniata;Vertebrata;Gnathostomata;Teleostomi;Euteleostomi;Acti
nopterygii;Cladistia;Polypteriformes;Polypteridae;Erpetoichthys;Erpetoich
thys calabaricus
UdeM00000028297 45264 146.4 UdeM00000028297 XP_044165535.1 39 205
117 4 99 297 322 524 6.00E-32 146.4 45264 cellular
organisms;Eukaryota;Opisthokonta;Metazoa;Eumetazoa;Cnidaria;Anthozoa;Hexa
corallia;Scleractinia;Astrocoeniina;Acroporidae;Acropora;Acropora
millepora
UdeM00000028298 29159 802.4 UdeM00000028298 XP_034298913.1 70.7 543
156 2 351 893 1155 1694 6.00E-229 802.4 29159 cellular
organisms;Eukaryota;Opisthokonta;Metazoa;Eumetazoa;Bilateria;Protostomia;
Spiralia;Lophotrochozoa;Mollusca;Bivalvia;Autobranchia;Pteriomorphia;Ostr
eida;Ostreoidea;Ostreidae;Crassostrea;Crassostrea gigas
UdeM00000028299 6565 692.2 UdeM00000028299 XP_022343829.1 97.1 341
10 0 1 341 1 341 3.30E-196 692.2 6565 cellular
organisms;Eukaryota;Opisthokonta;Metazoa;Eumetazoa;Bilateria;Protostomia;
Spiralia;Lophotrochozoa;Mollusca;Bivalvia;Autobranchia;Pteriomorphia;Ostr
eida;Ostreoidea;Ostreidae;Crassostrea;Crassostrea virginica
UdeM00000028300 6565 369.4 UdeM00000028300 XP_022343829.1 98.3 174
3 0 8 181 168 341 2.60E-99 369.4 6565 cellular
organisms;Eukaryota;Opisthokonta;Metazoa;Eumetazoa;Bilateria;Protostomia;
Spiralia;Lophotrochozoa;Mollusca;Bivalvia;Autobranchia;Pteriomorphia;Ostr
eida;Ostreoidea;Ostreidae;Crassostrea;Crassostrea virginica
UdeM00000028301 6579 436.8 UdeM00000028301 XP_033763717.1 45.3 523
223 9 28 525 28 512 3.90E-119 436.8 6579 cellular
organisms;Eukaryota;Opisthokonta;Metazoa;Eumetazoa;Bilateria;Protostomia;
Spiralia;Lophotrochozoa;Mollusca;Bivalvia;Autobranchia;Pteriomorphia;Pect
inida;Pectinoidea;Pectinidae;Pecten;Pecten maximus
UdeM00000028302 6596 456.1 UdeM00000028302 XP_045161195.1 70.7 311
91 0 1 311 1 311 3.70E-125 456.1 6596 cellular
organisms;Eukaryota;Opisthokonta;Metazoa;Eumetazoa;Bilateria;Protostomia;
Spiralia;Lophotrochozoa;Mollusca;Bivalvia;Autobranchia;Heteroconchia;Euhe
terodonta;Imparidentia;Venerida;Veneroidea;Veneridae;Mercenaria;Mercenari
a mercenaria
UdeM00000028303 6565 92.4 UdeM00000028303 XP_022303441.1 29.7 306
174 7 1 291 1 280 1.00E-15 92.4 6565 cellular
organisms;Eukaryota;Opisthokonta;Metazoa;Eumetazoa;Bilateria;Protostomia;
Spiralia;Lophotrochozoa;Mollusca;Bivalvia;Autobranchia;Pteriomorphia;Ostr
eida;Ostreoidea;Ostreidae;Crassostrea;Crassostrea virginica

UdeM00000028304 6579 271.6 UdeM00000028304 XP_033724809.1 45.3 327
160 4 1 326 1 309 1.40E-69 271.6 6579 cellular
organisms;Eukaryota;Opisthokonta;Metazoa;Eumetazoa;Bilateria;Protostomia;Spiralia;Lophotrochozoa;Mollusca;Bivalvia;Autobranchia;Pteriomorphia;Pectinida;Pectinoidea;Pectinidae;Pecten;Pecten maximus
UdeM00000028305 6596 510.8 UdeM00000028305 XP_045162872.1 73.1 316
85 0 71 386 5 320 1.60E-141 510.8 6596 cellular
organisms;Eukaryota;Opisthokonta;Metazoa;Eumetazoa;Bilateria;Protostomia;Spiralia;Lophotrochozoa;Mollusca;Bivalvia;Autobranchia;Heteroconchia;Euheterodonta;Imparidentia;Venerida;Veneroidea;Veneridae;Mercenaria;Mercenaria mercenaria
UdeM00000028306 6596 89.4 UdeM00000028306 XP_045161495.1 55.1 78
32 1 77 154 46 120 4.50E-15 89.4 6596 cellular
organisms;Eukaryota;Opisthokonta;Metazoa;Eumetazoa;Bilateria;Protostomia;Spiralia;Lophotrochozoa;Mollusca;Bivalvia;Autobranchia;Heteroconchia;Euheterodonta;Imparidentia;Venerida;Veneroidea;Veneridae;Mercenaria;Mercenaria mercenaria
UdeM00000028307 6596 374 UdeM00000028307 XP_045177776.1 31.4 781
463 18 285 1047 398 1123 7.00E-100 374 6596 cellular
organisms;Eukaryota;Opisthokonta;Metazoa;Eumetazoa;Bilateria;Protostomia;Spiralia;Lophotrochozoa;Mollusca;Bivalvia;Autobranchia;Heteroconchia;Euheterodonta;Imparidentia;Venerida;Veneroidea;Veneridae;Mercenaria;Mercenaria mercenaria
UdeM00000028308 50429 158.3 UdeM00000028308 XP_022789961.1 68.5 92
29 0 1 92 78 169 4.70E-36 158.3 50429 cellular
organisms;Eukaryota;Opisthokonta;Metazoa;Eumetazoa;Cnidaria;Anthozoa;Hexacorallia;Scleractinia;Astrocoeniina;Pocilloporidae;Stylophora;Stylophora pistillata
UdeM00000028309 6596 410.2 UdeM00000028309 XP_045170511.1 31.3 951
485 32 19 861 90 980 6.70E-111 410.2 6596 cellular
organisms;Eukaryota;Opisthokonta;Metazoa;Eumetazoa;Bilateria;Protostomia;Spiralia;Lophotrochozoa;Mollusca;Bivalvia;Autobranchia;Heteroconchia;Euheterodonta;Imparidentia;Venerida;Veneroidea;Veneridae;Mercenaria;Mercenaria mercenaria
UdeM00000028310 6579 69.3 UdeM00000028310 XP_033761099.1 22.1 249
171 4 134 363 68 312 1.20E-08 69.3 6579 cellular
organisms;Eukaryota;Opisthokonta;Metazoa;Eumetazoa;Bilateria;Protostomia;Spiralia;Lophotrochozoa;Mollusca;Bivalvia;Autobranchia;Pteriomorphia;Pectinida;Pectinoidea;Pectinidae;Pecten;Pecten maximus
UdeM00000028311 6579 321.6 UdeM00000028311 XP_033724622.1 77.7 202
45 0 18 219 1482 1683 7.60E-85 321.6 6579 cellular
organisms;Eukaryota;Opisthokonta;Metazoa;Eumetazoa;Bilateria;Protostomia;Spiralia;Lophotrochozoa;Mollusca;Bivalvia;Autobranchia;Pteriomorphia;Pectinida;Pectinoidea;Pectinidae;Pecten;Pecten maximus
UdeM00000028312 390379 96.3 UdeM00000028312 XP_034039899.1 52.1
73 34 1 3 74 1 73 2.40E-17 96.3 390379
cellular
organisms;Eukaryota;Opisthokonta;Metazoa;Eumetazoa;Bilateria;Deuterostomia;Chordata;Craniata;Vertebrata;Gnathostomata;Teleostomi;Euteleostomi;Actinopterygii;Actinopteri;Neopterygii;Teleostei;Osteoglossocephalai;Clupeocephala;Euteleosteomorpha;Neoteleostei;Eurypterygia;Ctenosquamata;Acanthomorphata;Euacanthomorphacea;Percomorphaceae;Batrachoidaria;Batrachoidiformes;Batrachoididae;Thalassophryninae;Thalassophryne;Thalassophryne amazonica

UdeM00000028313 6565 631.7 UdeM00000028313 XP_022343862.1 46.1 647
286 5 24 610 661 1304 1.20E-177 631.7 6565 cellular
organisms;Eukaryota;Opisthokonta;Metazoa;Eumetazoa;Bilateria;Protostomia;
Spiralia;Lophotrochozoa;Mollusca;Bivalvia;Autobranchia;Pteriomorphia;Ostr
eida;Ostreoidea;Ostreidae;Crassostrea;Crassostrea virginica
UdeM00000028316 6596 235 UdeM00000028316 XP_045185723.1 42.4 304
157 5 7 308 455 742 2.00E-58 235 6596 cellular
organisms;Eukaryota;Opisthokonta;Metazoa;Eumetazoa;Bilateria;Protostomia;
Spiralia;Lophotrochozoa;Mollusca;Bivalvia;Autobranchia;Heteroconchia;Euhe
terodonta;Imparidentia;Venerida;Veneroidea;Veneridae;Mercenaria;Mercenari
a mercenaria
UdeM00000028317 6596 122.1 UdeM00000028317 XP_045206982.1 51 104
51 0 4 107 80 183 4.80E-25 122.1 6596 cellular
organisms;Eukaryota;Opisthokonta;Metazoa;Eumetazoa;Bilateria;Protostomia;
Spiralia;Lophotrochozoa;Mollusca;Bivalvia;Autobranchia;Heteroconchia;Euhe
terodonta;Imparidentia;Venerida;Veneroidea;Veneridae;Mercenaria;Mercenari
a mercenaria
UdeM00000028318 6565 191 UdeM00000028318 XP_022343862.1 32.9 295
191 6 82 371 275 567 3.10E-45 191 6565 cellular
organisms;Eukaryota;Opisthokonta;Metazoa;Eumetazoa;Bilateria;Protostomia;
Spiralia;Lophotrochozoa;Mollusca;Bivalvia;Autobranchia;Pteriomorphia;Ostr
eida;Ostreoidea;Ostreidae;Crassostrea;Crassostrea virginica
UdeM00000028319 6579 166 UdeM00000028319 XP_033724622.1 37.8 270
138 7 4 256 140 396 6.30E-38 166 6579 cellular
organisms;Eukaryota;Opisthokonta;Metazoa;Eumetazoa;Bilateria;Protostomia;
Spiralia;Lophotrochozoa;Mollusca;Bivalvia;Autobranchia;Pteriomorphia;Pect
inida;Pectinoidea;Pectinidae;Pecten;Pecten maximus
UdeM00000028320 151771 325.9 UdeM00000028320 XP_028406951.1 36
555 311 10 1 518 119 666 9.60E-86 325.9 151771
cellular
organisms;Eukaryota;Opisthokonta;Metazoa;Eumetazoa;Cnidaria;Anthozoa;Octo
corallia;Alcyonacea;Alcyoniina;Nephtheidae;Dendronephthya;Dendronephthya
gigantea
UdeM00000028321 45264 403.3 UdeM00000028321 XP_044165143.1 49 443
171 5 42 433 35 473 4.00E-109 403.3 45264 cellular
organisms;Eukaryota;Opisthokonta;Metazoa;Eumetazoa;Cnidaria;Anthozoa;Hexa
corallia;Scleractinia;Astrocoeniina;Acroporidae;Acropora;Acropora
millepora
UdeM00000028322 29159 428.3 UdeM00000028322 XP_034336553.1 58.8 369
144 3 22 383 27 394 1.00E-116 428.3 29159 cellular
organisms;Eukaryota;Opisthokonta;Metazoa;Eumetazoa;Bilateria;Protostomia;
Spiralia;Lophotrochozoa;Mollusca;Bivalvia;Autobranchia;Pteriomorphia;Ostr
eida;Ostreoidea;Ostreidae;Crassostrea;Crassostrea gigas
UdeM00000028323 29159 500 UdeM00000028323 XP_034336553.1 60.3 416
157 3 22 430 27 441 3.10E-138 500 29159 cellular
organisms;Eukaryota;Opisthokonta;Metazoa;Eumetazoa;Bilateria;Protostomia;
Spiralia;Lophotrochozoa;Mollusca;Bivalvia;Autobranchia;Pteriomorphia;Ostr
eida;Ostreoidea;Ostreidae;Crassostrea;Crassostrea gigas
UdeM00000028324 6579 242.7 UdeM00000028324 XP_033763289.1 75.7 152
36 1 6 156 289 440 3.30E-61 242.7 6579 cellular
organisms;Eukaryota;Opisthokonta;Metazoa;Eumetazoa;Bilateria;Protostomia;
Spiralia;Lophotrochozoa;Mollusca;Bivalvia;Autobranchia;Pteriomorphia;Pect
inida;Pectinoidea;Pectinidae;Pecten;Pecten maximus

UdeM00000028325 37621 331.3 UdeM00000028325 XP_014679474.1 42.9 464
150 6 1 349 1 464 1.50E-87 331.3 37621 cellular organisms;Eukaryota;Opisthokonta;Metazoa;Eumetazoa;Bilateria;Protostomia;Ecdysozoa;Scalidophora;Priapulida;Priapulimorpha;Priapulimorphida;Priapulidae;Priapulus;Priapulus caudatus
UdeM00000028326 1735272 402.5 UdeM00000028326 XP_041366048.1 47.9
443 188 4 7 443 8 413 6.90E-109 402.5 1735272
cellular
organisms;Eukaryota;Opisthokonta;Metazoa;Eumetazoa;Bilateria;Protostomia;Spiralia;Lophotrochozoa;Mollusca;Gastropoda;Neomphalina;Neomphaloidea;Peltospiridae;Gigantopelta;Gigantopelta aegis
UdeM00000028327 6579 916 UdeM00000028327 XP_033724659.1 60.7 792
299 5 40 820 478 1268 3.80E-263 916 6579 cellular organisms;Eukaryota;Opisthokonta;Metazoa;Eumetazoa;Bilateria;Protostomia;Spiralia;Lophotrochozoa;Mollusca;Bivalvia;Autobranchia;Pteriomorphia;Pectinida;Pectinoidea;Pectinidae;Pecten;Pecten maximus
UdeM00000028328 6579 191 UdeM00000028328 XP_033724608.1 60.1 153
56 1 1 148 389 541 1.10E-45 191 6579 cellular organisms;Eukaryota;Opisthokonta;Metazoa;Eumetazoa;Bilateria;Protostomia;Spiralia;Lophotrochozoa;Mollusca;Bivalvia;Autobranchia;Pteriomorphia;Pectinida;Pectinoidea;Pectinidae;Pecten;Pecten maximus
UdeM00000028329 6579 189.1 UdeM00000028329 XP_033724608.1 68 128
41 0 14 141 414 541 3.80E-45 189.1 6579 cellular organisms;Eukaryota;Opisthokonta;Metazoa;Eumetazoa;Bilateria;Protostomia;Spiralia;Lophotrochozoa;Mollusca;Bivalvia;Autobranchia;Pteriomorphia;Pectinida;Pectinoidea;Pectinidae;Pecten;Pecten maximus
UdeM00000028330 6596 213.8 UdeM00000028330 XP_045162854.1 39 315
159 2 1 282 1 315 3.00E-52 213.8 6596 cellular organisms;Eukaryota;Opisthokonta;Metazoa;Eumetazoa;Bilateria;Protostomia;Spiralia;Lophotrochozoa;Mollusca;Bivalvia;Autobranchia;Heteroconchia;Euheterodonta;Imparidentia;Venerida;Veneroidea;Veneridae;Mercenaria;Mercenaria mercenaria
UdeM00000028331 6596 287.7 UdeM00000028331 XP_045162854.1 50.6 314
152 2 5 315 4 317 1.90E-74 287.7 6596 cellular organisms;Eukaryota;Opisthokonta;Metazoa;Eumetazoa;Bilateria;Protostomia;Spiralia;Lophotrochozoa;Mollusca;Bivalvia;Autobranchia;Heteroconchia;Euheterodonta;Imparidentia;Venerida;Veneroidea;Veneridae;Mercenaria;Mercenaria mercenaria
UdeM00000028332 1735272 961.1 UdeM00000028332 XP_041378073.1 83.5
546 90 0 27 572 26 571 6.50E-277 961.1 1735272
cellular
organisms;Eukaryota;Opisthokonta;Metazoa;Eumetazoa;Bilateria;Protostomia;Spiralia;Lophotrochozoa;Mollusca;Gastropoda;Neomphalina;Neomphaloidea;Peltospiridae;Gigantopelta;Gigantopelta aegis
UdeM00000028333 6500 93.2 UdeM00000028333 XP_012940823.2 29.6 189
133 0 19 207 98 286 1.00E-15 93.2 6500 cellular organisms;Eukaryota;Opisthokonta;Metazoa;Eumetazoa;Bilateria;Protostomia;Spiralia;Lophotrochozoa;Mollusca;Gastropoda;Heterobranchia;Euthyneura;Tectipleura;Aplysiida;Aplysioidea;Aplysiidae;Aplysia;Aplysia californica
UdeM00000028336 29159 756.5 UdeM00000028336 XP_011446697.1 80.8 556
106 1 1 555 1 556 2.30E-215 756.5 29159 cellular organisms;Eukaryota;Opisthokonta;Metazoa;Eumetazoa;Bilateria;Protostomia;Spiralia;Lophotrochozoa;Mollusca;Bivalvia;Autobranchia;Pteriomorphia;Ostreida;Ostreoidea;Ostreidae;Crassostrea;Crassostrea gigas

UdeM00000028337 6596 1681.8 UdeM00000028337 XP_045161165.1 57.6
1508 557 22 61 1549 72 1516 0.00E+00 1681.8 6596
cellular
organisms;Eukaryota;Opisthokonta;Metazoa;Eumetazoa;Bilateria;Protostomia;
Spiralia;Lophotrochozoa;Mollusca;Bivalvia;Autobranchia;Heteroconchia;Euhe
terodonta;Imparidentia;Venerida;Veneroidea;Veneridae;Mercenaria;Mercenari
a mercenaria
UdeM00000028338 1735272 375.6 UdeM00000028338 XP_041377628.1 60.3
290 109 3 18 302 11 299 6.10E-101 375.6 1735272
cellular
organisms;Eukaryota;Opisthokonta;Metazoa;Eumetazoa;Bilateria;Protostomia;
Spiralia;Lophotrochozoa;Mollusca;Gastropoda;Neomphalina;Neomphaloidea;Pel
tospiridae;Gigantopelta;Gigantopelta aegis
UdeM00000028339 6573 849.4 UdeM00000028339 XP_021372599.1 67 628
189 9 1 621 1 617 3.00E-243 849.4 6573 cellular
organisms;Eukaryota;Opisthokonta;Metazoa;Eumetazoa;Bilateria;Protostomia;
Spiralia;Lophotrochozoa;Mollusca;Bivalvia;Autobranchia;Pteriomorphia;Pect
inida;Pectinoidea;Pectinidae;Mizuhopecten;Mizuhopecten yessoensis
UdeM00000028340 400727 244.2 UdeM00000028340 XP_025081221.1 89.3
131 14 0 1 131 17 147 9.30E-62 244.2 400727
cellular
organisms;Eukaryota;Opisthokonta;Metazoa;Eumetazoa;Bilateria;Protostomia;
Spiralia;Lophotrochozoa;Mollusca;Gastropoda;Caenogastropoda;Architaeniogl
ossa;Ampullarioidea;Ampullariidae;Pomacea;Pomacea canaliculata
UdeM00000028341 400727 123.2 UdeM00000028341 XP_025081502.1 70.4
81 23 1 1 81 13 92 1.70E-25 123.2 400727
cellular
organisms;Eukaryota;Opisthokonta;Metazoa;Eumetazoa;Bilateria;Protostomia;
Spiralia;Lophotrochozoa;Mollusca;Gastropoda;Caenogastropoda;Architaeniogl
ossa;Ampullarioidea;Ampullariidae;Pomacea;Pomacea canaliculata
UdeM00000028343 6573 429.1 UdeM00000028343 XP_021366465.1 97.2 216
6 0 1 216 1 216 7.00E-117 429.1 6573 cellular
organisms;Eukaryota;Opisthokonta;Metazoa;Eumetazoa;Bilateria;Protostomia;
Spiralia;Lophotrochozoa;Mollusca;Bivalvia;Autobranchia;Pteriomorphia;Pect
inida;Pectinoidea;Pectinidae;Mizuhopecten;Mizuhopecten yessoensis
UdeM00000028344 6596 786.9 UdeM00000028344 XP_045160149.1 34.2 1687
846 42 6 1506 3 1611 5.10E-224 786.9 6596 cellular
organisms;Eukaryota;Opisthokonta;Metazoa;Eumetazoa;Bilateria;Protostomia;
Spiralia;Lophotrochozoa;Mollusca;Bivalvia;Autobranchia;Heteroconchia;Euhe
terodonta;Imparidentia;Venerida;Veneroidea;Veneridae;Mercenaria;Mercenari
a mercenaria
UdeM00000028345 6596 1040.8 UdeM00000028345 XP_045162321.1 60.3
882 270 7 396 1268 692 1502 1.40E-300 1040.8 6596
cellular
organisms;Eukaryota;Opisthokonta;Metazoa;Eumetazoa;Bilateria;Protostomia;
Spiralia;Lophotrochozoa;Mollusca;Bivalvia;Autobranchia;Heteroconchia;Euhe
terodonta;Imparidentia;Venerida;Veneroidea;Veneridae;Mercenaria;Mercenari
a mercenaria
UdeM00000028347 6596 333.2 UdeM00000028347 XP_045162321.1 66.8 241
79 1 1 241 1263 1502 2.80E-88 333.2 6596 cellular
organisms;Eukaryota;Opisthokonta;Metazoa;Eumetazoa;Bilateria;Protostomia;
Spiralia;Lophotrochozoa;Mollusca;Bivalvia;Autobranchia;Heteroconchia;Euhe
terodonta;Imparidentia;Venerida;Veneroidea;Veneridae;Mercenaria;Mercenari
a mercenaria

UdeM00000028346	7574	359.8	UdeM00000028346	XP_023933247.1	78.8	222
	47	0	77	298	848	1069	3.50E-96	359.8	7574	cellular
organisms;Eukaryota;Opisthokonta;Metazoa;Eumetazoa;Bilateria;Protostomia;
Spiralia;Lophotrochozoa;Brachiopoda;Linguliformea;Lingulata;Lingulida;Lin
guloidea;Lingulidae;Lingula;Lingula anatina
UdeM00000028348	6596	127.9	UdeM00000028348	XP_045200228.1	30.5	311
	181	8	54	336	30	333	3.00E-26	127.9	6596	cellular
organisms;Eukaryota;Opisthokonta;Metazoa;Eumetazoa;Bilateria;Protostomia;
Spiralia;Lophotrochozoa;Mollusca;Bivalvia;Autobranchia;Heteroconchia;Euhe
terodonta;Imparidentia;Venerida;Veneroidea;Veneridae;Mercenaria;Mercenari
a mercenaria
UdeM00000028349	6579	745.7	UdeM00000028349	XP_033739131.1	59.2	588
	237	2	1	585	783	1370	4.40E-212	745.7	6579	cellular
organisms;Eukaryota;Opisthokonta;Metazoa;Eumetazoa;Bilateria;Protostomia;
Spiralia;Lophotrochozoa;Mollusca;Bivalvia;Autobranchia;Pteriomorphia;Pect
inida;Pectinoidea;Pectinidae;Pecten;Pecten maximus
UdeM00000041777	7574	186.4	UdeM00000041777	XP_013384628.1	58.4	161
	58	2	1	161	33	184	3.20E-44	186.4	7574	cellular
organisms;Eukaryota;Opisthokonta;Metazoa;Eumetazoa;Bilateria;Protostomia;
Spiralia;Lophotrochozoa;Brachiopoda;Linguliformea;Lingulata;Lingulida;Lin
guloidea;Lingulidae;Lingula;Lingula anatina
UdeM00000041778	7574	255.8	UdeM00000041778	XP_013382110.1	47.4	234
	119	2	3	236	386	615	5.50E-65	255.8	7574	cellular
organisms;Eukaryota;Opisthokonta;Metazoa;Eumetazoa;Bilateria;Protostomia;
Spiralia;Lophotrochozoa;Brachiopoda;Linguliformea;Lingulata;Lingulida;Lin
guloidea;Lingulidae;Lingula;Lingula anatina
UdeM00000041780	7574	260.8	UdeM00000041780	XP_013382110.1	61.9	194
	74	0	14	207	45	238	1.50E-66	260.8	7574	cellular
organisms;Eukaryota;Opisthokonta;Metazoa;Eumetazoa;Bilateria;Protostomia;
Spiralia;Lophotrochozoa;Brachiopoda;Linguliformea;Lingulata;Lingulida;Lin
guloidea;Lingulidae;Lingula;Lingula anatina
UdeM00000041779	7574	260.8	UdeM00000041779	XP_013382110.1	59.6	208
	78	1	1	208	37	238	1.50E-66	260.8	7574	cellular
organisms;Eukaryota;Opisthokonta;Metazoa;Eumetazoa;Bilateria;Protostomia;
Spiralia;Lophotrochozoa;Brachiopoda;Linguliformea;Lingulata;Lingulida;Lin
guloidea;Lingulidae;Lingula;Lingula anatina
UdeM00000041781	6596	82.4	UdeM00000041781	XP_045192251.1	51.1	92
	42	2	122	210	328	419	7.50E-13	82.4	6596	cellular
organisms;Eukaryota;Opisthokonta;Metazoa;Eumetazoa;Bilateria;Protostomia;
Spiralia;Lophotrochozoa;Mollusca;Bivalvia;Autobranchia;Heteroconchia;Euhe
terodonta;Imparidentia;Venerida;Veneroidea;Veneridae;Mercenaria;Mercenari
a mercenaria
UdeM00000041782	6573	264.2	UdeM00000041782	XP_021354853.1	50	266
	123	3	185	447	147	405	2.90E-67	264.2	6573	cellular
organisms;Eukaryota;Opisthokonta;Metazoa;Eumetazoa;Bilateria;Protostomia;
Spiralia;Lophotrochozoa;Mollusca;Bivalvia;Autobranchia;Pteriomorphia;Pect
inida;Pectinoidea;Pectinidae;Mizuhopecten;Mizuhopecten yessoensis
UdeM00000041783	6573	442.2	UdeM00000041783	XP_021354853.1	46.3	497
	208	4	4	445	396	888	7.80E-121	442.2	6573	cellular
organisms;Eukaryota;Opisthokonta;Metazoa;Eumetazoa;Bilateria;Protostomia;
Spiralia;Lophotrochozoa;Mollusca;Bivalvia;Autobranchia;Pteriomorphia;Pect
inida;Pectinoidea;Pectinidae;Mizuhopecten;Mizuhopecten yessoensis
UdeM00000041784	6596	176.4	UdeM00000041784	XP_045206152.1	52.6	175
	77	2	70	244	381	549	4.50E-41	176.4	6596	cellular

organisms;Eukaryota;Opisthokonta;Metazoa;Eumetazoa;Bilateria;Protostomia;Spiralia;Lophotrochozoa;Mollusca;Bivalvia;Autobranchia;Heteroconchia;Euheterodonta;Imparidentia;Venerida;Veneroidea;Veneridae;Mercenaria;Mercenaria mercenaria
UdeM00000041786 6596 82.8 UdeM00000041786 XP_045186008.1 42 81 46 1 4 83 221 301 5.80E-13 82.8 6596 cellular organisms;Eukaryota;Opisthokonta;Metazoa;Eumetazoa;Bilateria;Protostomia;Spiralia;Lophotrochozoa;Mollusca;Bivalvia;Autobranchia;Heteroconchia;Euheterodonta;Imparidentia;Venerida;Veneroidea;Veneridae;Mercenaria;Mercenaria mercenaria
UdeM00000041787 7739 105.1 UdeM00000041787 XP_035662552.1 38.8 129 77 1 2 130 390 516 1.30E-19 105.1 7739 cellular organisms;Eukaryota;Opisthokonta;Metazoa;Eumetazoa;Bilateria;Deuterostomia;Chordata;Cephalochordata;Leptocardii;Amphioxiformes;Branchiostomidae;Branchiostoma;Branchiostoma floridae
UdeM00000041788 34773 106.7 UdeM00000041788 XP_041958816.1 32.8 180 102 6 3 165 153 330 3.40E-20 106.7 34773 cellular organisms;Eukaryota;Opisthokonta;Metazoa;Eumetazoa;Bilateria;Deuterostomia;Chordata;Craniata;Vertebrata;Gnathostomata;Teleostomi;Euteleostomi;Actinopterygii;Actinopteri;Neopterygii;Teleostei;Osteoglossocephalai;Clupeocephala;Otomorpha;Clupei;Clupeiformes;Clupeoidei;Clupeidae;Alosinae;Alosa;Alosa sapidissima
UdeM00000031609 6596 184.9 UdeM00000031609 XP_045176664.1 26.5 543 309 14 32 496 35 565 2.50E-43 184.9 6596 cellular organisms;Eukaryota;Opisthokonta;Metazoa;Eumetazoa;Bilateria;Protostomia;Spiralia;Lophotrochozoa;Mollusca;Bivalvia;Autobranchia;Heteroconchia;Euheterodonta;Imparidentia;Venerida;Veneroidea;Veneridae;Mercenaria;Mercenaria mercenaria
UdeM00000031610 6596 129 UdeM00000031610 XP_045214418.1 40.8 147 84 2 154 298 110 255 1.00E-26 129 6596 cellular organisms;Eukaryota;Opisthokonta;Metazoa;Eumetazoa;Bilateria;Protostomia;Spiralia;Lophotrochozoa;Mollusca;Bivalvia;Autobranchia;Heteroconchia;Euheterodonta;Imparidentia;Venerida;Veneroidea;Veneridae;Mercenaria;Mercenaria mercenaria
UdeM00000031611 6565 63.9 UdeM00000031611 XP_022322825.1 46.9 64 34 0 4 67 401 464 1.40E-07 63.9 6565 cellular organisms;Eukaryota;Opisthokonta;Metazoa;Eumetazoa;Bilateria;Protostomia;Spiralia;Lophotrochozoa;Mollusca;Bivalvia;Autobranchia;Pteriomorphia;Ostreida;Ostreoidea;Ostreidae;Crassostrea;Crassostrea virginica
UdeM00000031612 29159 67.8 UdeM00000031612 XP_034322772.1 28.8 205 94 6 1 187 1 171 1.70E-08 67.8 29159 cellular organisms;Eukaryota;Opisthokonta;Metazoa;Eumetazoa;Bilateria;Protostomia;Spiralia;Lophotrochozoa;Mollusca;Bivalvia;Autobranchia;Pteriomorphia;Ostreida;Ostreoidea;Ostreidae;Crassostrea;Crassostrea gigas
UdeM00000031613 6596 464.9 UdeM00000031613 XP_045188551.1 30 1320 821 39 2 1278 2218 3477 3.20E-127 464.9 6596 cellular organisms;Eukaryota;Opisthokonta;Metazoa;Eumetazoa;Bilateria;Protostomia;Spiralia;Lophotrochozoa;Mollusca;Bivalvia;Autobranchia;Heteroconchia;Euheterodonta;Imparidentia;Venerida;Veneroidea;Veneridae;Mercenaria;Mercenaria mercenaria
UdeM00000031614 6596 199.9 UdeM00000031614 XP_045190673.1 43.6 243 130 4 11 248 589 829 5.40E-48 199.9 6596 cellular organisms;Eukaryota;Opisthokonta;Metazoa;Eumetazoa;Bilateria;Protostomia;Spiralia;Lophotrochozoa;Mollusca;Bivalvia;Autobranchia;Heteroconchia;Euhe

terodonta;Imparidentia;Venerida;Veneroidea;Veneridae;Mercenaria;Mercenaria mercenaria
UdeM00000031617 151771 420.6 UdeM00000031617 XP_028419301.1 54.5 380 144 3 42 394 34 411 2.30E-114 420.6 151771 cellular organisms;Eukaryota;Opisthokonta;Metazoa;Eumetazoa;Cnidaria;Anthozoa;Octocorallia;Alcyonacea;Alcyoniina;Nephtheidae;Dendronephthya;Dendronephthya gigantea
UdeM00000031618 45264 172.9 UdeM00000031618 XP_044184031.1 48.7 191 88 5 1 185 300 486 3.70E-40 172.9 45264 cellular organisms;Eukaryota;Opisthokonta;Metazoa;Eumetazoa;Cnidaria;Anthozoa;Hexacorallia;Scleractinia;Astrocoeniina;Acroporidae;Acropora;Acropora millepora
UdeM00000031619 6596 198.4 UdeM00000031619 XP_045190673.1 42.6 242 133 4 11 248 589 828 1.10E-47 198.4 6596 cellular organisms;Eukaryota;Opisthokonta;Metazoa;Eumetazoa;Bilateria;Protostomia;Spiralia;Lophotrochozoa;Mollusca;Bivalvia;Autobranchia;Heteroconchia;Euheterodonta;Imparidentia;Venerida;Veneroidea;Veneridae;Mercenaria;Mercenaria mercenaria
UdeM00000031620 6579 208.8 UdeM00000031620 XP_033764133.1 27.5 665 415 15 484 1093 490 1142 3.60E-50 208.8 6579 cellular organisms;Eukaryota;Opisthokonta;Metazoa;Eumetazoa;Bilateria;Protostomia;Spiralia;Lophotrochozoa;Mollusca;Bivalvia;Autobranchia;Pteriomorphia;Pectinida;Pectinoidea;Pectinidae;Pecten;Pecten maximus
UdeM00000031621 6573 303.9 UdeM00000031621 XP_021355423.1 40.7 427 221 9 13 424 2093 2502 3.20E-79 303.9 6573 cellular organisms;Eukaryota;Opisthokonta;Metazoa;Eumetazoa;Bilateria;Protostomia;Spiralia;Lophotrochozoa;Mollusca;Bivalvia;Autobranchia;Pteriomorphia;Pectinida;Pectinoidea;Pectinidae;Mizuhopecten;Mizuhopecten yessoensis
UdeM00000031622 1735272 337.4 UdeM00000031622 XP_041347204.1 39.8 465 252 14 2 460 207 649 2.80E-89 337.4 1735272 cellular organisms;Eukaryota;Opisthokonta;Metazoa;Eumetazoa;Bilateria;Protostomia;Spiralia;Lophotrochozoa;Mollusca;Gastropoda;Neomphalina;Neomphaloidea;Peltospiridae;Gigantopelta;Gigantopelta aegis
UdeM00000031623 1735272 492.7 UdeM00000031623 XP_041347279.1 35 907 521 28 265 1131 1 878 1.30E-135 492.7 1735272 cellular organisms;Eukaryota;Opisthokonta;Metazoa;Eumetazoa;Bilateria;Protostomia;Spiralia;Lophotrochozoa;Mollusca;Gastropoda;Neomphalina;Neomphaloidea;Peltospiridae;Gigantopelta;Gigantopelta aegis
UdeM00000031625 6565 266.5 UdeM00000031625 XP_022335825.1 24 1279 772 40 20 1248 550 1678 2.00E-67 266.5 6565 cellular organisms;Eukaryota;Opisthokonta;Metazoa;Eumetazoa;Bilateria;Protostomia;Spiralia;Lophotrochozoa;Mollusca;Bivalvia;Autobranchia;Pteriomorphia;Ostreida;Ostreoidea;Ostreidae;Crassostrea;Crassostrea virginica
UdeM00000031626 7574 73.2 UdeM00000031626 XP_023932230.1 46.1 89 47 1 2 89 780 868 6.70E-10 73.2 7574 cellular organisms;Eukaryota;Opisthokonta;Metazoa;Eumetazoa;Bilateria;Protostomia;Spiralia;Lophotrochozoa;Brachiopoda;Linguliformea;Lingulata;Lingulida;Linguloidea;Lingulidae;Lingula;Lingula anatina
UdeM00000031627 6565 201.1 UdeM00000031627 XP_022335825.1 42.7 241 133 4 11 249 550 787 5.30E-48 201.1 6565 cellular organisms;Eukaryota;Opisthokonta;Metazoa;Eumetazoa;Bilateria;Protostomia;

Spiralia;Lophotrochozoa;Mollusca;Bivalvia;Autobranchia;Pteriomorphia;Ostreida;Ostreoidea;Ostreidae;Crassostrea;Crassostrea virginica
UdeM00000031629 29159 438.7 UdeM00000031629 XP_011425943.2 25.4 1757 974 44 474 1972 809 2486 3.90E-119 438.7 29159 cellular organisms;Eukaryota;Opisthokonta;Metazoa;Eumetazoa;Bilateria;Protostomia;Spiralia;Lophotrochozoa;Mollusca;Bivalvia;Autobranchia;Pteriomorphia;Ostreida;Ostreoidea;Ostreidae;Crassostrea;Crassostrea gigas
UdeM00000031630 6573 198 UdeM00000031630 XP_021354329.1 42 245 137 4 10 251 481 723 4.00E-47 198 6573 cellular organisms;Eukaryota;Opisthokonta;Metazoa;Eumetazoa;Bilateria;Protostomia;Spiralia;Lophotrochozoa;Mollusca;Bivalvia;Autobranchia;Pteriomorphia;Pectinida;Pectinoidea;Pectinidae;Mizuhopecten;Mizuhopecten yessoensis
UdeM00000031631 29159 146.7 UdeM00000031631 XP_034317326.1 31.4 370 215 10 408 740 1640 2007 1.50E-31 146.7 29159 cellular organisms;Eukaryota;Opisthokonta;Metazoa;Eumetazoa;Bilateria;Protostomia;Spiralia;Lophotrochozoa;Mollusca;Bivalvia;Autobranchia;Pteriomorphia;Ostreida;Ostreoidea;Ostreidae;Crassostrea;Crassostrea gigas
UdeM00000031632 6596 123.6 UdeM00000031632 XP_045188551.1 59.4 101 40 1 3 102 3377 3477 1.50E-25 123.6 6596 cellular organisms;Eukaryota;Opisthokonta;Metazoa;Eumetazoa;Bilateria;Protostomia;Spiralia;Lophotrochozoa;Mollusca;Bivalvia;Autobranchia;Heteroconchia;Euheterodonta;Imparidentia;Venerida;Veneroidea;Veneridae;Mercenaria;Mercenaria mercenaria
UdeM00000031634 6565 177.9 UdeM00000031634 XP_022335825.1 40.8 240 128 5 26 253 550 787 2.70E-41 177.9 6565 cellular organisms;Eukaryota;Opisthokonta;Metazoa;Eumetazoa;Bilateria;Protostomia;Spiralia;Lophotrochozoa;Mollusca;Bivalvia;Autobranchia;Pteriomorphia;Ostreida;Ostreoidea;Ostreidae;Crassostrea;Crassostrea virginica
UdeM00000031636 114398 258.1 UdeM00000031636 XP_042913678.1 41.2 323 184 2 1 319 108 428 1.50E-65 258.1 114398 cellular organisms;Eukaryota;Opisthokonta;Metazoa;Eumetazoa;Bilateria;Protostomia;Ecdysozoa;Panarthropoda;Arthropoda;Chelicerata;Arachnida;Araneae;Araneomorphae;Entelegynae;Orbiculariae;Araneoidea;Theridiidae;Parasteatoda;Parasteatoda tepidariorum
UdeM00000031637 6579 357.1 UdeM00000031637 XP_033764133.1 30.4 951 516 27 1 823 491 1423 6.20E-95 357.1 6579 cellular organisms;Eukaryota;Opisthokonta;Metazoa;Eumetazoa;Bilateria;Protostomia;Spiralia;Lophotrochozoa;Mollusca;Bivalvia;Autobranchia;Pteriomorphia;Pectinida;Pectinoidea;Pectinidae;Pecten;Pecten maximus
UdeM00000031638 6596 200.7 UdeM00000031638 XP_045190515.1 33.5 394 222 11 809 1164 1582 1973 1.00E-47 200.7 6596 cellular organisms;Eukaryota;Opisthokonta;Metazoa;Eumetazoa;Bilateria;Protostomia;Spiralia;Lophotrochozoa;Mollusca;Bivalvia;Autobranchia;Heteroconchia;Euheterodonta;Imparidentia;Venerida;Veneroidea;Veneridae;Mercenaria;Mercenaria mercenaria
UdeM00000031640 6596 186.4 UdeM00000031640 XP_045190673.1 40.6 261 131 5 11 267 589 829 6.10E-44 186.4 6596 cellular organisms;Eukaryota;Opisthokonta;Metazoa;Eumetazoa;Bilateria;Protostomia;Spiralia;Lophotrochozoa;Mollusca;Bivalvia;Autobranchia;Heteroconchia;Euheterodonta;Imparidentia;Venerida;Veneroidea;Veneridae;Mercenaria;Mercenaria mercenaria
UdeM00000031641 6596 235 UdeM00000031641 XP_045188486.1 38.7 390 216 7 89 459 1693 2078 2.70E-58 235 6596 cellular

organisms;Eukaryota;Opisthokonta;Metazoa;Eumetazoa;Bilateria;Protostomia;Spiralia;Lophotrochozoa;Mollusca;Bivalvia;Autobranchia;Heteroconchia;Euheterodonta;Imparidentia;Venerida;Veneroidea;Veneridae;Mercenaria;Mercenaria mercenaria
UdeM00000031642 6596 202.6 UdeM00000031642 XP_045190673.1 43.6 243 131 4 20 258 589 829 2.20E-48 202.6 6596 cellular organisms;Eukaryota;Opisthokonta;Metazoa;Eumetazoa;Bilateria;Protostomia;Spiralia;Lophotrochozoa;Mollusca;Bivalvia;Autobranchia;Heteroconchia;Euheterodonta;Imparidentia;Venerida;Veneroidea;Veneridae;Mercenaria;Mercenaria mercenaria
UdeM00000031643 6596 479.6 UdeM00000031643 XP_045188486.1 38.2 759 437 16 3 742 1804 2549 7.40E-132 479.6 6596 cellular organisms;Eukaryota;Opisthokonta;Metazoa;Eumetazoa;Bilateria;Protostomia;Spiralia;Lophotrochozoa;Mollusca;Bivalvia;Autobranchia;Heteroconchia;Euheterodonta;Imparidentia;Venerida;Veneroidea;Veneridae;Mercenaria;Mercenaria mercenaria
UdeM00000031644 6573 261.2 UdeM00000031644 XP_021355423.1 34.3 502 280 13 11 481 1965 2447 2.70E-66 261.2 6573 cellular organisms;Eukaryota;Opisthokonta;Metazoa;Eumetazoa;Bilateria;Protostomia;Spiralia;Lophotrochozoa;Mollusca;Bivalvia;Autobranchia;Pteriomorphia;Pectinida;Pectinoidea;Pectinidae;Mizuhopecten;Mizuhopecten yessoensis
UdeM00000031649 6596 154.8 UdeM00000031649 XP_045188551.1 55.2 125 56 0 9 133 3353 3477 7.60E-35 154.8 6596 cellular organisms;Eukaryota;Opisthokonta;Metazoa;Eumetazoa;Bilateria;Protostomia;Spiralia;Lophotrochozoa;Mollusca;Bivalvia;Autobranchia;Heteroconchia;Euheterodonta;Imparidentia;Venerida;Veneroidea;Veneridae;Mercenaria;Mercenaria mercenaria
UdeM00000031645 6596 497.3 UdeM00000031645 XP_045188551.1 28.8 1615 890 47 594 1997 1912 3477 9.20E-137 497.3 6596 cellular organisms;Eukaryota;Opisthokonta;Metazoa;Eumetazoa;Bilateria;Protostomia;Spiralia;Lophotrochozoa;Mollusca;Bivalvia;Autobranchia;Heteroconchia;Euheterodonta;Imparidentia;Venerida;Veneroidea;Veneridae;Mercenaria;Mercenaria mercenaria
UdeM00000031646 6596 497.3 UdeM00000031646 XP_045188551.1 28.8 1615 890 47 609 2012 1912 3477 9.30E-137 497.3 6596 cellular organisms;Eukaryota;Opisthokonta;Metazoa;Eumetazoa;Bilateria;Protostomia;Spiralia;Lophotrochozoa;Mollusca;Bivalvia;Autobranchia;Heteroconchia;Euheterodonta;Imparidentia;Venerida;Veneroidea;Veneridae;Mercenaria;Mercenaria mercenaria
UdeM00000031647 6596 500.4 UdeM00000031647 XP_045188551.1 28.5 1657 919 49 496 1937 1872 3477 1.10E-137 500.4 6596 cellular organisms;Eukaryota;Opisthokonta;Metazoa;Eumetazoa;Bilateria;Protostomia;Spiralia;Lophotrochozoa;Mollusca;Bivalvia;Autobranchia;Heteroconchia;Euheterodonta;Imparidentia;Venerida;Veneroidea;Veneridae;Mercenaria;Mercenaria mercenaria
UdeM00000031648 6596 497.7 UdeM00000031648 XP_045188551.1 28.8 1615 890 47 549 1952 1912 3477 6.90E-137 497.7 6596 cellular organisms;Eukaryota;Opisthokonta;Metazoa;Eumetazoa;Bilateria;Protostomia;Spiralia;Lophotrochozoa;Mollusca;Bivalvia;Autobranchia;Heteroconchia;Euheterodonta;Imparidentia;Venerida;Veneroidea;Veneridae;Mercenaria;Mercenaria mercenaria
UdeM00000031651 7962 203.8 UdeM00000031651 XP_042578941.1 32 397 246 8 125 507 38 424 6.60E-49 203.8 7962 cellular organisms;Eukaryota;Opisthokonta;Metazoa;Eumetazoa;Bilateria;Deuterostomi

a;Chordata;Craniata;Vertebrata;Gnathostomata;Teleostomi;Euteleostomi;Actinopterygii;Actinopteri;Neopterygii;Teleostei;Osteoglossocephalai;Clupeocephala;Otomorpha;Ostariophysi;Otophysi;Cypriniphysae;Cypriniformes;Cyprinoidei;Cyprinidae;Cyprininae;Cyprinus;Cyprinus carpio
UdeM00000031652 6565 359.8 UdeM00000031652 XP_022316307.1 38.4 570 320 10 1 550 5 563 6.70E-96 359.8 6565 cellular organisms;Eukaryota;Opisthokonta;Metazoa;Eumetazoa;Bilateria;Protostomia;Spiralia;Lophotrochozoa;Mollusca;Bivalvia;Autobranchia;Pteriomorphia;Ostreida;Ostreoidea;Ostreidae;Crassostrea;Crassostrea virginica
UdeM00000031654 6565 199.5 UdeM00000031654 XP_022335825.1 44 243 129 5 23 263 550 787 6.90E-48 199.5 6565 cellular organisms;Eukaryota;Opisthokonta;Metazoa;Eumetazoa;Bilateria;Protostomia;Spiralia;Lophotrochozoa;Mollusca;Bivalvia;Autobranchia;Pteriomorphia;Ostreida;Ostreoidea;Ostreidae;Crassostrea;Crassostrea virginica
UdeM00000031656 29159 152.5 UdeM00000031656 XP_034336526.1 69.1 97 30 0 223 319 1839 1935 8.90E-34 152.5 29159 cellular organisms;Eukaryota;Opisthokonta;Metazoa;Eumetazoa;Bilateria;Protostomia;Spiralia;Lophotrochozoa;Mollusca;Bivalvia;Autobranchia;Pteriomorphia;Ostreida;Ostreoidea;Ostreidae;Crassostrea;Crassostrea gigas
UdeM00000031657 1735272 95.1 UdeM00000031657 XP_041347065.1 32.8 204 101 6 1 171 1 201 9.40E-17 95.1 1735272 cellular organisms;Eukaryota;Opisthokonta;Metazoa;Eumetazoa;Bilateria;Protostomia;Spiralia;Lophotrochozoa;Mollusca;Gastropoda;Neomphalina;Neomphaloidea;Peltospiridae;Gigantopelta;Gigantopelta aegis
UdeM00000031658 37621 389.4 UdeM00000031658 XP_014679849.1 35.8 614 225 6 10 464 14 617 6.30E-105 389.4 37621 cellular organisms;Eukaryota;Opisthokonta;Metazoa;Eumetazoa;Bilateria;Protostomia;Ecdysozoa;Scalidophora;Priapulida;Priapulimorpha;Priapulimorphida;Priapulidae;Priapulus;Priapulus caudatus
UdeM00000031659 6579 142.5 UdeM00000031659 XP_033764133.1 29.5 424 254 13 158 543 493 909 1.60E-30 142.5 6579 cellular organisms;Eukaryota;Opisthokonta;Metazoa;Eumetazoa;Bilateria;Protostomia;Spiralia;Lophotrochozoa;Mollusca;Bivalvia;Autobranchia;Pteriomorphia;Pectinida;Pectinoidea;Pectinidae;Pecten;Pecten maximus
UdeM00000031660 6596 327.4 UdeM00000031660 XP_045190515.1 34.7 547 323 11 14 549 1450 1973 3.50E-86 327.4 6596 cellular organisms;Eukaryota;Opisthokonta;Metazoa;Eumetazoa;Bilateria;Protostomia;Spiralia;Lophotrochozoa;Mollusca;Bivalvia;Autobranchia;Heteroconchia;Euheterodonta;Imparidentia;Venerida;Veneroidea;Veneridae;Mercenaria;Mercenaria mercenaria
UdeM00000031661 6573 154.1 UdeM00000031661 XP_021361223.1 71.1 97 28 0 291 387 975 1071 3.70E-34 154.1 6573 cellular organisms;Eukaryota;Opisthokonta;Metazoa;Eumetazoa;Bilateria;Protostomia;Spiralia;Lophotrochozoa;Mollusca;Bivalvia;Autobranchia;Pteriomorphia;Pectinida;Pectinoidea;Pectinidae;Mizuhopecten;Mizuhopecten yessoensis
UdeM00000031662 6573 98.6 UdeM00000031662 XP_021371061.1 36.7 188 90 7 29 196 58 236 9.60E-18 98.6 6573 cellular organisms;Eukaryota;Opisthokonta;Metazoa;Eumetazoa;Bilateria;Protostomia;Spiralia;Lophotrochozoa;Mollusca;Bivalvia;Autobranchia;Pteriomorphia;Pectinida;Pectinoidea;Pectinidae;Mizuhopecten;Mizuhopecten yessoensis
UdeM00000031663 37621 216.1 UdeM00000031663 XP_014679849.1 51.9 181 73 3 16 192 447 617 4.00E-53 216.1 37621 cellular organisms;Eukaryota;Opisthokonta;Metazoa;Eumetazoa;Bilateria;Protostomia;

Ecdysozoa;Scalidophora;Priapulida;Priapulimorpha;Priapulimorphida;Priapulidae;Priapulus;Priapulus caudatus
UdeM00000031664 6573 137.1 UdeM00000031664 XP_021375729.1 57.8 102 43 0 10 111 9 110 1.50E-29 137.1 6573 cellular organisms;Eukaryota;Opisthokonta;Metazoa;Eumetazoa;Bilateria;Protostomia;Spiralia;Lophotrochozoa;Mollusca;Bivalvia;Autobranchia;Pteriomorphia;Pectinida;Pectinoidea;Pectinidae;Mizuhopecten;Mizuhopecten yessoensis
UdeM00000031665 6579 350.9 UdeM00000031665 XP_033731448.1 26.1 1363 833 36 658 1875 81 1414 1.00E-92 350.9 6579 cellular organisms;Eukaryota;Opisthokonta;Metazoa;Eumetazoa;Bilateria;Protostomia;Spiralia;Lophotrochozoa;Mollusca;Bivalvia;Autobranchia;Pteriomorphia;Pectinida;Pectinoidea;Pectinidae;Pecten;Pecten maximus
UdeM00000031667 6596 330.5 UdeM00000031667 XP_045158356.1 32.4 608 391 8 1 588 959 1566 4.40E-87 330.5 6596 cellular organisms;Eukaryota;Opisthokonta;Metazoa;Eumetazoa;Bilateria;Protostomia;Spiralia;Lophotrochozoa;Mollusca;Bivalvia;Autobranchia;Heteroconchia;Euheterodonta;Imparidentia;Venerida;Veneroidea;Veneridae;Mercenaria;Mercenaria mercenaria
UdeM00000031668 8932 100.5 UdeM00000031668 XP_021146887.1 44.6 92 51 0 18 109 532 623 1.60E-18 100.5 8932 cellular organisms;Eukaryota;Opisthokonta;Metazoa;Eumetazoa;Bilateria;Deuterostomia;Chordata;Craniata;Vertebrata;Gnathostomata;Teleostomi;Euteleostomi;Sarcopterygii;Dipnotetrapodomorpha;Tetrapoda;Amniota;Sauropsida;Sauria;Archelosauria;Archosauria;Dinosauria;Saurischia;Theropoda;Coelurosauria;Aves;Neognathae;Columbiformes;Columbidae;Columba;Columba livia
UdeM00000031669 6579 304.7 UdeM00000031669 XP_033731448.1 27 1245 748 43 172 1287 80 1292 5.70E-79 304.7 6579 cellular organisms;Eukaryota;Opisthokonta;Metazoa;Eumetazoa;Bilateria;Protostomia;Spiralia;Lophotrochozoa;Mollusca;Bivalvia;Autobranchia;Pteriomorphia;Pectinida;Pectinoidea;Pectinidae;Pecten;Pecten maximus
UdeM00000044057 595528 246.5 UdeM00000044057 XP_004349202.2 44.2 308 150 4 4 310 2 288 4.60E-62 246.5 595528 cellular organisms;Eukaryota;Opisthokonta;Filasterea;Capsaspora;Capsaspora owczarzaki;Capsaspora owczarzaki ATCC 30864
UdeM00000044058 83332 434.1 UdeM00000044058 NP_216556.1 81.4 263 49 0 1 263 37 299 1.30E-118 434.1 83332 cellular organisms;Bacteria;Terrabacteria group;Actinobacteria;Actinomycetia;Corynebacteriales;Mycobacteriaceae;Mycobacterium;Mycobacterium tuberculosis complex;Mycobacterium tuberculosis;Mycobacterium tuberculosis H37Rv
UdeM00000044059 83332 427.9 UdeM00000044059 NP_216554.1 82.2 264 47 0 1 264 92 355 9.20E-117 427.9 83332 cellular organisms;Bacteria;Terrabacteria group;Actinobacteria;Actinomycetia;Corynebacteriales;Mycobacteriaceae;Mycobacterium;Mycobacterium tuberculosis complex;Mycobacterium tuberculosis;Mycobacterium tuberculosis H37Rv
UdeM00000044060 208964 352.8 UdeM00000044060 NP_251247.1 39.3 544 293 10 24 537 18 554 7.60E-94 352.8 208964 cellular organisms;Bacteria;Proteobacteria;Gammaproteobacteria;Pseudomonadales;Pseudomonadaceae;Pseudomonas;Pseudomonas aeruginosa group;Pseudomonas aeruginosa;Pseudomonas aeruginosa PAO1

UdeM00000007718 6596 126.7 UdeM00000007718 XP_045181277.1 26.2 390
199 12 9 325 46 419 5.40E-26 126.7 6596 cellular
organisms;Eukaryota;Opisthokonta;Metazoa;Eumetazoa;Bilateria;Protostomia;
Spiralia;Lophotrochozoa;Mollusca;Bivalvia;Autobranchia;Heteroconchia;Euheterodonta;Imparidentia;Venerida;Veneroidea;Veneridae;Mercenaria;Mercenaria mercenaria
UdeM00000007719 6596 137.1 UdeM00000007719 XP_045181277.1 32.8 256
155 7 112 355 143 393 4.30E-29 137.1 6596 cellular
organisms;Eukaryota;Opisthokonta;Metazoa;Eumetazoa;Bilateria;Protostomia;
Spiralia;Lophotrochozoa;Mollusca;Bivalvia;Autobranchia;Heteroconchia;Euheterodonta;Imparidentia;Venerida;Veneroidea;Veneridae;Mercenaria;Mercenaria mercenaria
UdeM00000007720 6596 131.7 UdeM00000007720 XP_045181277.1 32.5 234
147 6 99 325 143 372 1.80E-27 131.7 6596 cellular
organisms;Eukaryota;Opisthokonta;Metazoa;Eumetazoa;Bilateria;Protostomia;
Spiralia;Lophotrochozoa;Mollusca;Bivalvia;Autobranchia;Heteroconchia;Euheterodonta;Imparidentia;Venerida;Veneroidea;Veneridae;Mercenaria;Mercenaria mercenaria
UdeM00000007722 6596 87.8 UdeM00000007722 XP_045177372.1 40.2 102
56 1 12 108 401 502 1.10E-14 87.8 6596 cellular
organisms;Eukaryota;Opisthokonta;Metazoa;Eumetazoa;Bilateria;Protostomia;
Spiralia;Lophotrochozoa;Mollusca;Bivalvia;Autobranchia;Heteroconchia;Euheterodonta;Imparidentia;Venerida;Veneroidea;Veneridae;Mercenaria;Mercenaria mercenaria
UdeM00000007723 6596 80.5 UdeM00000007723 XP_045202346.1 46.8 77
41 0 8 84 68 144 1.10E-12 80.5 6596 cellular
organisms;Eukaryota;Opisthokonta;Metazoa;Eumetazoa;Bilateria;Protostomia;
Spiralia;Lophotrochozoa;Mollusca;Bivalvia;Autobranchia;Heteroconchia;Euheterodonta;Imparidentia;Venerida;Veneroidea;Veneridae;Mercenaria;Mercenaria mercenaria
UdeM00000007724 6596 179.1 UdeM00000007724 XP_045183440.1 42.3 239
106 3 2 211 145 380 7.90E-42 179.1 6596 cellular
organisms;Eukaryota;Opisthokonta;Metazoa;Eumetazoa;Bilateria;Protostomia;
Spiralia;Lophotrochozoa;Mollusca;Bivalvia;Autobranchia;Heteroconchia;Euheterodonta;Imparidentia;Venerida;Veneroidea;Veneridae;Mercenaria;Mercenaria mercenaria
UdeM00000007725 6596 132.1 UdeM00000007725 XP_045181277.1 31.5 260
141 9 67 304 143 387 1.20E-27 132.1 6596 cellular
organisms;Eukaryota;Opisthokonta;Metazoa;Eumetazoa;Bilateria;Protostomia;
Spiralia;Lophotrochozoa;Mollusca;Bivalvia;Autobranchia;Heteroconchia;Euheterodonta;Imparidentia;Venerida;Veneroidea;Veneridae;Mercenaria;Mercenaria mercenaria
UdeM00000007726 6596 97.4 UdeM00000007726 XP_045202346.1 41.3 109
62 1 26 134 36 142 1.50E-17 97.4 6596 cellular
organisms;Eukaryota;Opisthokonta;Metazoa;Eumetazoa;Bilateria;Protostomia;
Spiralia;Lophotrochozoa;Mollusca;Bivalvia;Autobranchia;Heteroconchia;Euheterodonta;Imparidentia;Venerida;Veneroidea;Veneridae;Mercenaria;Mercenaria mercenaria
UdeM00000007728 6596 186 UdeM00000007728 XP_045187052.1 52.6 175
78 3 2 175 145 315 4.40E-44 186 6596 cellular
organisms;Eukaryota;Opisthokonta;Metazoa;Eumetazoa;Bilateria;Protostomia;
Spiralia;Lophotrochozoa;Mollusca;Bivalvia;Autobranchia;Heteroconchia;Euheterodonta;Imparidentia;Venerida;Veneroidea;Veneridae;Mercenaria;Mercenaria mercenaria

UdeM00000007727	6596	187.2	UdeM00000007727	XP_045187052.1	50	188
89	3	2	188	145	328	2.60E-44	187.2	6596	cellular
organisms;Eukaryota;Opisthokonta;Metazoa;Eumetazoa;Bilateria;Protostomia;Spiralia;Lophotrochozoa;Mollusca;Bivalvia;Autobranchia;Heteroconchia;Euheterodonta;Imparidentia;Venerida;Veneroidea;Veneridae;Mercenaria;Mercenaria mercenaria
UdeM00000007731	50429	139	UdeM00000007731	XP_022808032.1	50.4	131
61	2	1	127	556	686	4.20E-30	139	50429	cellular
organisms;Eukaryota;Opisthokonta;Metazoa;Eumetazoa;Cnidaria;Anthozoa;Hexacorallia;Scleractinia;Astrocoeniina;Pocilloporidae;Stylophora;Stylophora pistillata
UdeM00000007732	151771	81.3	UdeM00000007732	XP_028412660.1	38.2
131	80	1	2	132	942	1071	1.10E-12	81.3	151771
cellular
organisms;Eukaryota;Opisthokonta;Metazoa;Eumetazoa;Cnidaria;Anthozoa;Octocorallia;Alcyonacea;Alcyoniina;Nephtheidae;Dendronephthya;Dendronephthya gigantea
UdeM00000007733	7654	117.9	UdeM00000007733	XP_041481100.1	38.1	147
88	3	156	302	1460	1603	2.30E-23	117.9	7654	cellular
organisms;Eukaryota;Opisthokonta;Metazoa;Eumetazoa;Bilateria;Deuterostomia;Echinodermata;Eleutherozoa;Echinozoa;Echinoidea;Euechinoidea;Echinacea;Temnopleuroida;Toxopneustidae;Lytechinus;Lytechinus variegatus
UdeM00000007735	6596	78.6	UdeM00000007735	XP_045176094.1	59.7	62
25	0	58	119	108	169	9.80E-12	78.6	6596	cellular
organisms;Eukaryota;Opisthokonta;Metazoa;Eumetazoa;Bilateria;Protostomia;Spiralia;Lophotrochozoa;Mollusca;Bivalvia;Autobranchia;Heteroconchia;Euheterodonta;Imparidentia;Venerida;Veneroidea;Veneridae;Mercenaria;Mercenaria mercenaria
UdeM00000007736	6596	78.6	UdeM00000007736	XP_045176094.1	59.7	62
25	0	14	75	108	169	7.60E-12	78.6	6596	cellular
organisms;Eukaryota;Opisthokonta;Metazoa;Eumetazoa;Bilateria;Protostomia;Spiralia;Lophotrochozoa;Mollusca;Bivalvia;Autobranchia;Heteroconchia;Euheterodonta;Imparidentia;Venerida;Veneroidea;Veneridae;Mercenaria;Mercenaria mercenaria
UdeM00000007737	6596	155.6	UdeM00000007737	XP_045181277.1	34.3	280
158	9	19	285	106	372	1.00E-34	155.6	6596	cellular
organisms;Eukaryota;Opisthokonta;Metazoa;Eumetazoa;Bilateria;Protostomia;Spiralia;Lophotrochozoa;Mollusca;Bivalvia;Autobranchia;Heteroconchia;Euheterodonta;Imparidentia;Venerida;Veneroidea;Veneridae;Mercenaria;Mercenaria mercenaria
UdeM00000007738	6596	97.1	UdeM00000007738	XP_045192683.1	55.4	83
37	0	1	83	199	281	1.20E-17	97.1	6596	cellular
organisms;Eukaryota;Opisthokonta;Metazoa;Eumetazoa;Bilateria;Protostomia;Spiralia;Lophotrochozoa;Mollusca;Bivalvia;Autobranchia;Heteroconchia;Euheterodonta;Imparidentia;Venerida;Veneroidea;Veneridae;Mercenaria;Mercenaria mercenaria
UdeM00000007739	6596	133.7	UdeM00000007739	XP_045181277.1	35.8	201
122	5	17	214	176	372	4.00E-28	133.7	6596	cellular
organisms;Eukaryota;Opisthokonta;Metazoa;Eumetazoa;Bilateria;Protostomia;Spiralia;Lophotrochozoa;Mollusca;Bivalvia;Autobranchia;Heteroconchia;Euheterodonta;Imparidentia;Venerida;Veneroidea;Veneridae;Mercenaria;Mercenaria mercenaria
UdeM00000007740	6596	164.5	UdeM00000007740	XP_045192683.1	42.7	232
123	2	34	257	52	281	1.80E-37	164.5	6596	cellular

organisms;Eukaryota;Opisthokonta;Metazoa;Eumetazoa;Bilateria;Protostomia;
Spiralia;Lophotrochozoa;Mollusca;Bivalvia;Autobranchia;Heteroconchia;Euhe
terodonta;Imparidentia;Venerida;Veneroidea;Veneridae;Mercenaria;Mercenari
a mercenaria
UdeM00000007741 6579 78.6 UdeM00000007741 XP_033739131.1 46.4 69
37 0 14 82 1118 1186 4.20E-12 78.6 6579 cellular
organisms;Eukaryota;Opisthokonta;Metazoa;Eumetazoa;Bilateria;Protostomia;
Spiralia;Lophotrochozoa;Mollusca;Bivalvia;Autobranchia;Pteriomorphia;Pect
inida;Pectinoidea;Pectinidae;Pecten;Pecten maximus
UdeM00000007742 6596 184.1 UdeM00000007742 XP_045192683.1 42.2 249
123 5 18 251 39 281 2.20E-43 184.1 6596 cellular
organisms;Eukaryota;Opisthokonta;Metazoa;Eumetazoa;Bilateria;Protostomia;
Spiralia;Lophotrochozoa;Mollusca;Bivalvia;Autobranchia;Heteroconchia;Euhe
terodonta;Imparidentia;Venerida;Veneroidea;Veneridae;Mercenaria;Mercenari
a mercenaria
UdeM00000007743 6596 182.2 UdeM00000007743 XP_045192683.1 44.5 245
101 6 19 240 49 281 7.90E-43 182.2 6596 cellular
organisms;Eukaryota;Opisthokonta;Metazoa;Eumetazoa;Bilateria;Protostomia;
Spiralia;Lophotrochozoa;Mollusca;Bivalvia;Autobranchia;Heteroconchia;Euhe
terodonta;Imparidentia;Venerida;Veneroidea;Veneridae;Mercenaria;Mercenari
a mercenaria
UdeM00000007744 6596 346.3 UdeM00000007744 XP_045212389.1 44.9 365
191 6 54 415 61 418 5.50E-92 346.3 6596 cellular
organisms;Eukaryota;Opisthokonta;Metazoa;Eumetazoa;Bilateria;Protostomia;
Spiralia;Lophotrochozoa;Mollusca;Bivalvia;Autobranchia;Heteroconchia;Euhe
terodonta;Imparidentia;Venerida;Veneroidea;Veneridae;Mercenaria;Mercenari
a mercenaria
UdeM00000007746 6573 219.9 UdeM00000007746 XP_021377528.1 66.2 157
53 0 1 157 18 174 2.30E-54 219.9 6573 cellular
organisms;Eukaryota;Opisthokonta;Metazoa;Eumetazoa;Bilateria;Protostomia;
Spiralia;Lophotrochozoa;Mollusca;Bivalvia;Autobranchia;Pteriomorphia;Pect
inida;Pectinoidea;Pectinidae;Mizuhopecten;Mizuhopecten yessoensis
UdeM00000007748 6565 629 UdeM00000007748 XP_022328027.1 84.1 383
59 1 1 381 174 556 3.90E-177 629 6565 cellular
organisms;Eukaryota;Opisthokonta;Metazoa;Eumetazoa;Bilateria;Protostomia;
Spiralia;Lophotrochozoa;Mollusca;Bivalvia;Autobranchia;Pteriomorphia;Ostr
eida;Ostreoidea;Ostreidae;Crassostrea;Crassostrea virginica
UdeM00000007747 6565 927.2 UdeM00000007747 XP_022328027.1 85.4 556
79 1 1 554 1 556 1.00E-266 927.2 6565 cellular
organisms;Eukaryota;Opisthokonta;Metazoa;Eumetazoa;Bilateria;Protostomia;
Spiralia;Lophotrochozoa;Mollusca;Bivalvia;Autobranchia;Pteriomorphia;Ostr
eida;Ostreoidea;Ostreidae;Crassostrea;Crassostrea virginica
UdeM00000007749 29159 254.2 UdeM00000007749 XP_034303596.1 89.5 143
15 0 19 161 24 166 1.10E-64 254.2 29159 cellular
organisms;Eukaryota;Opisthokonta;Metazoa;Eumetazoa;Bilateria;Protostomia;
Spiralia;Lophotrochozoa;Mollusca;Bivalvia;Autobranchia;Pteriomorphia;Ostr
eida;Ostreoidea;Ostreidae;Crassostrea;Crassostrea gigas
UdeM00000007750 225164 76.3 UdeM00000007750 XP_009063340.1 60
60 24 0 4 63 28 87 1.70E-11 76.3 225164
cellular
organisms;Eukaryota;Opisthokonta;Metazoa;Eumetazoa;Bilateria;Protostomia;
Spiralia;Lophotrochozoa;Mollusca;Gastropoda;Patellogastropoda;Lottioidea;
Lottiidae;Lottia;Lottia gigantea

UdeM00000007751 29159 292.4 UdeM00000007751 XP_034333743.1 67.5 209
68 0 47 255 86 294 5.80E-76 292.4 29159 cellular
organisms;Eukaryota;Opisthokonta;Metazoa;Eumetazoa;Bilateria;Protostomia;
Spiralia;Lophotrochozoa;Mollusca;Bivalvia;Autobranchia;Pteriomorphia;Ostr
eida;Ostreoidea;Ostreidae;Crassostrea;Crassostrea gigas
UdeM00000007752 6596 328.6 UdeM00000007752 XP_045177776.1 38.7 475
271 10 1 470 405 864 2.10E-86 328.6 6596 cellular
organisms;Eukaryota;Opisthokonta;Metazoa;Eumetazoa;Bilateria;Protostomia;
Spiralia;Lophotrochozoa;Mollusca;Bivalvia;Autobranchia;Heteroconchia;Euhe
terodonta;Imparidentia;Venerida;Veneroidea;Veneridae;Mercenaria;Mercenari
a mercenaria
UdeM00000007753 6596 78.2 UdeM00000007753 XP_045179120.1 53.8 65
29 1 1 65 1 64 4.50E-12 78.2 6596 cellular
organisms;Eukaryota;Opisthokonta;Metazoa;Eumetazoa;Bilateria;Protostomia;
Spiralia;Lophotrochozoa;Mollusca;Bivalvia;Autobranchia;Heteroconchia;Euhe
terodonta;Imparidentia;Venerida;Veneroidea;Veneridae;Mercenaria;Mercenari
a mercenaria
UdeM00000007754 6596 81.6 UdeM00000007754 XP_045179120.1 53.7 67
30 1 1 67 1 66 6.40E-13 81.6 6596 cellular
organisms;Eukaryota;Opisthokonta;Metazoa;Eumetazoa;Bilateria;Protostomia;
Spiralia;Lophotrochozoa;Mollusca;Bivalvia;Autobranchia;Heteroconchia;Euhe
terodonta;Imparidentia;Venerida;Veneroidea;Veneridae;Mercenaria;Mercenari
a mercenaria
UdeM00000007757 6596 166.8 UdeM00000007757 XP_045194272.1 61.3 142
54 1 3 144 23 163 2.10E-38 166.8 6596 cellular
organisms;Eukaryota;Opisthokonta;Metazoa;Eumetazoa;Bilateria;Protostomia;
Spiralia;Lophotrochozoa;Mollusca;Bivalvia;Autobranchia;Heteroconchia;Euhe
terodonta;Imparidentia;Venerida;Veneroidea;Veneridae;Mercenaria;Mercenari
a mercenaria
UdeM00000007758 1735272 88.6 UdeM00000007758 XP_041361245.1 56
75 33 0 123 197 217 291 1.00E-14 88.6 1735272
cellular
organisms;Eukaryota;Opisthokonta;Metazoa;Eumetazoa;Bilateria;Protostomia;
Spiralia;Lophotrochozoa;Mollusca;Gastropoda;Neomphalina;Neomphaloidea;Pel
tospiridae;Gigantopelta;Gigantopelta aegis
UdeM00000007759 6596 87.4 UdeM00000007759 XP_045182578.1 50.6 79
39 0 121 199 214 292 2.20E-14 87.4 6596 cellular
organisms;Eukaryota;Opisthokonta;Metazoa;Eumetazoa;Bilateria;Protostomia;
Spiralia;Lophotrochozoa;Mollusca;Bivalvia;Autobranchia;Heteroconchia;Euhe
terodonta;Imparidentia;Venerida;Veneroidea;Veneridae;Mercenaria;Mercenari
a mercenaria
UdeM00000007761 29159 219.9 UdeM00000007761 XP_034339165.1 37.7 334
194 8 1 331 186 508 4.70E-54 219.9 29159 cellular
organisms;Eukaryota;Opisthokonta;Metazoa;Eumetazoa;Bilateria;Protostomia;
Spiralia;Lophotrochozoa;Mollusca;Bivalvia;Autobranchia;Pteriomorphia;Ostr
eida;Ostreoidea;Ostreidae;Crassostrea;Crassostrea gigas
UdeM00000007762 29159 502.3 UdeM00000007762 XP_034299474.1 43.6 610
328 8 94 696 155 755 1.00E-138 502.3 29159 cellular
organisms;Eukaryota;Opisthokonta;Metazoa;Eumetazoa;Bilateria;Protostomia;
Spiralia;Lophotrochozoa;Mollusca;Bivalvia;Autobranchia;Pteriomorphia;Ostr
eida;Ostreoidea;Ostreidae;Crassostrea;Crassostrea gigas
UdeM00000007765 6579 177.2 UdeM00000007765 XP_033744238.1 37.2 331
161 10 329 649 133 426 7.10E-41 177.2 6579 cellular
organisms;Eukaryota;Opisthokonta;Metazoa;Eumetazoa;Bilateria;Protostomia;

Spiralia;Lophotrochozoa;Mollusca;Bivalvia;Autobranchia;Pteriomorphia;Pectinida;Pectinoidea;Pectinidae;Pecten;Pecten maximus
UdeM00000007767 30019 58.9 UdeM00000007767 XP_017846261.1 34.5 139 74 5 1 132 168 296 5.60E-06 58.9 30019 cellular organisms;Eukaryota;Opisthokonta;Metazoa;Eumetazoa;Bilateria;Protostomia;Ecdysozoa;Panarthropoda;Arthropoda;Mandibulata;Pancrustacea;Hexapoda;Insecta;Dicondylia;Pterygota;Neoptera;Endopterygota;Diptera;Brachycera;Muscomorpha;Eremoneura;Cyclorrhapha;Schizophora;Acalyptratae;Ephydroidea;Drosophilidae;Drosophilinae;Drosophilini;Drosophila;Dorsilopha;Drosophila busckii
UdeM00000007768 1735272 153.7 UdeM00000007768 XP_041361243.1 49 198 89 5 18 205 81 276 3.10E-33 153.7 1735272 cellular organisms;Eukaryota;Opisthokonta;Metazoa;Eumetazoa;Bilateria;Protostomia;Spiralia;Lophotrochozoa;Mollusca;Gastropoda;Neomphalina;Neomphaloidea;Peltospiridae;Gigantopelta;Gigantopelta aegis
UdeM00000007769 29159 180.3 UdeM00000007769 XP_019926413.2 81.8 110 18 2 1 108 97 206 1.40E-42 180.3 29159 cellular organisms;Eukaryota;Opisthokonta;Metazoa;Eumetazoa;Bilateria;Protostomia;Spiralia;Lophotrochozoa;Mollusca;Bivalvia;Autobranchia;Pteriomorphia;Ostreida;Ostreoidea;Ostreidae;Crassostrea;Crassostrea gigas
UdeM00000007770 29159 369.4 UdeM00000007770 XP_019926413.2 83.6 219 35 1 1 219 292 509 3.40E-99 369.4 29159 cellular organisms;Eukaryota;Opisthokonta;Metazoa;Eumetazoa;Bilateria;Protostomia;Spiralia;Lophotrochozoa;Mollusca;Bivalvia;Autobranchia;Pteriomorphia;Ostreida;Ostreoidea;Ostreidae;Crassostrea;Crassostrea gigas
UdeM00000007771 7574 97.8 UdeM00000007771 XP_013401704.1 46.4 84 45 0 17 100 748 831 8.50E-18 97.8 7574 cellular organisms;Eukaryota;Opisthokonta;Metazoa;Eumetazoa;Bilateria;Protostomia;Spiralia;Lophotrochozoa;Brachiopoda;Linguliformea;Lingulata;Lingulida;Linguloidea;Lingulidae;Lingula;Lingula anatina
UdeM00000007773 45264 198.4 UdeM00000007773 XP_044165143.1 76.9 117 27 0 1 117 217 333 5.30E-48 198.4 45264 cellular organisms;Eukaryota;Opisthokonta;Metazoa;Eumetazoa;Cnidaria;Anthozoa;Hexacorallia;Scleractinia;Astrocoeniina;Acroporidae;Acropora;Acropora millepora
UdeM00000007775 6565 281.2 UdeM00000007775 XP_022303385.1 44.9 303 155 7 29 322 547 846 1.70E-72 281.2 6565 cellular organisms;Eukaryota;Opisthokonta;Metazoa;Eumetazoa;Bilateria;Protostomia;Spiralia;Lophotrochozoa;Mollusca;Bivalvia;Autobranchia;Pteriomorphia;Ostreida;Ostreoidea;Ostreidae;Crassostrea;Crassostrea virginica
UdeM00000007776 6565 108.2 UdeM00000007776 XP_022321940.1 26 292 199 7 31 312 399 683 1.90E-20 108.2 6565 cellular organisms;Eukaryota;Opisthokonta;Metazoa;Eumetazoa;Bilateria;Protostomia;Spiralia;Lophotrochozoa;Mollusca;Bivalvia;Autobranchia;Pteriomorphia;Ostreida;Ostreoidea;Ostreidae;Crassostrea;Crassostrea virginica
UdeM00000037872 1735272 126.3 UdeM00000037872 XP_041371877.1 82.1 67 12 0 2 68 952 1018 1.60E-26 126.3 1735272 cellular organisms;Eukaryota;Opisthokonta;Metazoa;Eumetazoa;Bilateria;Protostomia;Spiralia;Lophotrochozoa;Mollusca;Gastropoda;Neomphalina;Neomphaloidea;Peltospiridae;Gigantopelta;Gigantopelta aegis
UdeM00000037873 6596 402.5 UdeM00000037873 XP_045156903.1 39.4 872 512 2 88 957 1981 2838 1.60E-108 402.5 6596 cellular

organisms;Eukaryota;Opisthokonta;Metazoa;Eumetazoa;Bilateria;Protostomia;Spiralia;Lophotrochozoa;Mollusca;Bivalvia;Autobranchia;Heteroconchia;Euheterodonta;Imparidentia;Venerida;Veneroidea;Veneridae;Mercenaria;Mercenaria mercenaria
UdeM00000037874 6596 357.1 UdeM00000037874 XP_045156903.1 38.2 872 388 10 3967 4831 1155 1882 3.60E-94 357.1 6596 cellular organisms;Eukaryota;Opisthokonta;Metazoa;Eumetazoa;Bilateria;Protostomia;Spiralia;Lophotrochozoa;Mollusca;Bivalvia;Autobranchia;Heteroconchia;Euheterodonta;Imparidentia;Venerida;Veneroidea;Veneridae;Mercenaria;Mercenaria mercenaria
UdeM00000037875 37621 186.4 UdeM00000037875 XP_014673010.1 52.3 176 81 2 3 176 363 537 3.50E-44 186.4 37621 cellular organisms;Eukaryota;Opisthokonta;Metazoa;Eumetazoa;Bilateria;Protostomia;Ecdysozoa;Scalidophora;Priapulida;Priapulimorpha;Priapulimorphida;Priapulidae;Priapulus;Priapulus caudatus
UdeM00000037876 1735272 975.7 UdeM00000037876 XP_041372967.1 79.1 728 125 10 1 715 1 714 3.20E-281 975.7 1735272 cellular organisms;Eukaryota;Opisthokonta;Metazoa;Eumetazoa;Bilateria;Protostomia;Spiralia;Lophotrochozoa;Mollusca;Gastropoda;Neomphalina;Neomphaloidea;Peltospiridae;Gigantopelta;Gigantopelta aegis
UdeM00000037877 1735272 956.1 UdeM00000037877 XP_041372967.1 77.7 726 146 7 1 722 1 714 2.60E-275 956.1 1735272 cellular organisms;Eukaryota;Opisthokonta;Metazoa;Eumetazoa;Bilateria;Protostomia;Spiralia;Lophotrochozoa;Mollusca;Gastropoda;Neomphalina;Neomphaloidea;Peltospiridae;Gigantopelta;Gigantopelta aegis
UdeM00000037878 6596 1127.1 UdeM00000037878 XP_045216647.1 62 1091 388 10 1 1081 1 1074 0.00E+00 1127.1 6596 cellular organisms;Eukaryota;Opisthokonta;Metazoa;Eumetazoa;Bilateria;Protostomia;Spiralia;Lophotrochozoa;Mollusca;Bivalvia;Autobranchia;Heteroconchia;Euheterodonta;Imparidentia;Venerida;Veneroidea;Veneridae;Mercenaria;Mercenaria mercenaria
UdeM00000037879 6565 1119.4 UdeM00000037879 XP_022332341.1 48.5 1207 614 5 28 1232 62 1262 0.00E+00 1119.4 6565 cellular organisms;Eukaryota;Opisthokonta;Metazoa;Eumetazoa;Bilateria;Protostomia;Spiralia;Lophotrochozoa;Mollusca;Bivalvia;Autobranchia;Pteriomorphia;Ostreida;Ostreoidea;Ostreidae;Crassostrea;Crassostrea virginica
UdeM00000037880 6596 356.3 UdeM00000037880 XP_045215300.1 55.2 335 144 2 22 351 1504 1837 5.70E-95 356.3 6596 cellular organisms;Eukaryota;Opisthokonta;Metazoa;Eumetazoa;Bilateria;Protostomia;Spiralia;Lophotrochozoa;Mollusca;Bivalvia;Autobranchia;Heteroconchia;Euheterodonta;Imparidentia;Venerida;Veneroidea;Veneridae;Mercenaria;Mercenaria mercenaria
UdeM00000037881 1735272 205.7 UdeM00000037881 XP_041373245.1 45.8 216 103 3 23 225 44 258 6.30E-50 205.7 1735272 cellular organisms;Eukaryota;Opisthokonta;Metazoa;Eumetazoa;Bilateria;Protostomia;Spiralia;Lophotrochozoa;Mollusca;Gastropoda;Neomphalina;Neomphaloidea;Peltospiridae;Gigantopelta;Gigantopelta aegis
UdeM00000037882 1735272 205.7 UdeM00000037882 XP_041373245.1 45.8 216 103 3 72 274 44 258 7.70E-50 205.7 1735272

cellular
organisms;Eukaryota;Opisthokonta;Metazoa;Eumetazoa;Bilateria;Protostomia;Spiralia;Lophotrochozoa;Mollusca;Gastropoda;Neomphalina;Neomphaloidea;Peltospiridae;Gigantopelta;Gigantopelta aegis
UdeM00000037883 6596 476.5 UdeM00000037883 XP_045215300.1 51.2 488
235 2 8 495 1835 2319 4.30E-131 476.5 6596 cellular
organisms;Eukaryota;Opisthokonta;Metazoa;Eumetazoa;Bilateria;Protostomia;Spiralia;Lophotrochozoa;Mollusca;Bivalvia;Autobranchia;Heteroconchia;Euheterodonta;Imparidentia;Venerida;Veneroidea;Veneridae;Mercenaria;Mercenaria mercenaria
UdeM00000037884 6596 723.8 UdeM00000037884 XP_045215300.1 51.6 733
301 3 24 702 2322 3054 2.10E-205 723.8 6596 cellular
organisms;Eukaryota;Opisthokonta;Metazoa;Eumetazoa;Bilateria;Protostomia;Spiralia;Lophotrochozoa;Mollusca;Bivalvia;Autobranchia;Heteroconchia;Euheterodonta;Imparidentia;Venerida;Veneroidea;Veneridae;Mercenaria;Mercenaria mercenaria
UdeM00000037885 6596 3783 UdeM00000037885 XP_045215300.1 52.2 3770
1310 10 13 3769 3054 6344 0.00E+00 3783 6596 cellular
organisms;Eukaryota;Opisthokonta;Metazoa;Eumetazoa;Bilateria;Protostomia;Spiralia;Lophotrochozoa;Mollusca;Bivalvia;Autobranchia;Heteroconchia;Euheterodonta;Imparidentia;Venerida;Veneroidea;Veneridae;Mercenaria;Mercenaria mercenaria
UdeM00000037886 29159 631.3 UdeM00000037886 XP_011453961.2 50.7 598
291 3 18 611 28 625 1.30E-177 631.3 29159 cellular
organisms;Eukaryota;Opisthokonta;Metazoa;Eumetazoa;Bilateria;Protostomia;Spiralia;Lophotrochozoa;Mollusca;Bivalvia;Autobranchia;Pteriomorphia;Ostreida;Ostreoidea;Ostreidae;Crassostrea;Crassostrea gigas
UdeM00000037887 6579 161 UdeM00000037887 XP_033750444.1 59.5 131
51 2 31 161 36 164 1.30E-36 161 6579 cellular
organisms;Eukaryota;Opisthokonta;Metazoa;Eumetazoa;Bilateria;Protostomia;Spiralia;Lophotrochozoa;Mollusca;Bivalvia;Autobranchia;Pteriomorphia;Pectinida;Pectinoidea;Pectinidae;Pecten;Pecten maximus
UdeM00000037888 6596 191 UdeM00000037888 XP_045156705.1 97.4 78
2 0 4 81 24 101 5.90E-46 191 6596 cellular
organisms;Eukaryota;Opisthokonta;Metazoa;Eumetazoa;Bilateria;Protostomia;Spiralia;Lophotrochozoa;Mollusca;Bivalvia;Autobranchia;Heteroconchia;Euheterodonta;Imparidentia;Venerida;Veneroidea;Veneridae;Mercenaria;Mercenaria mercenaria
UdeM00000037889 6596 334.7 UdeM00000037889 XP_045156707.1 30.3 801
477 20 5 764 27 787 3.90E-88 334.7 6596 cellular
organisms;Eukaryota;Opisthokonta;Metazoa;Eumetazoa;Bilateria;Protostomia;Spiralia;Lophotrochozoa;Mollusca;Bivalvia;Autobranchia;Heteroconchia;Euheterodonta;Imparidentia;Venerida;Veneroidea;Veneridae;Mercenaria;Mercenaria mercenaria
UdeM00000037890 6596 434.9 UdeM00000037890 XP_045215244.1 67.3 330
106 2 9 336 5 334 1.00E-118 434.9 6596 cellular
organisms;Eukaryota;Opisthokonta;Metazoa;Eumetazoa;Bilateria;Protostomia;Spiralia;Lophotrochozoa;Mollusca;Bivalvia;Autobranchia;Heteroconchia;Euheterodonta;Imparidentia;Venerida;Veneroidea;Veneridae;Mercenaria;Mercenaria mercenaria
UdeM00000037891 1735272 392.9 UdeM00000037891 XP_041367886.1 72.3
267 72 1 106 372 115 379 4.60E-106 392.9 1735272
cellular
organisms;Eukaryota;Opisthokonta;Metazoa;Eumetazoa;Bilateria;Protostomia;

Spiralia;Lophotrochozoa;Mollusca;Gastropoda;Neomphalina;Neomphaloidea;Peltospiridae;Gigantopelta;Gigantopelta aegis
UdeM00000037892 1735272 92 UdeM00000037892 XP_041367886.1 63 73 25 1 106 178 115 185 8.70E-16 92 1735272 cellular organisms;Eukaryota;Opisthokonta;Metazoa;Eumetazoa;Bilateria;Protostomia;Spiralia;Lophotrochozoa;Mollusca;Gastropoda;Neomphalina;Neomphaloidea;Peltospiridae;Gigantopelta;Gigantopelta aegis
UdeM00000037894 6579 452.2 UdeM00000037894 XP_033750186.1 75.2 282 69 1 8 289 95 375 6.00E-123 452.2 6579 cellular organisms;Eukaryota;Opisthokonta;Metazoa;Eumetazoa;Bilateria;Protostomia;Spiralia;Lophotrochozoa;Mollusca;Bivalvia;Autobranchia;Pteriomorphia;Pectinida;Pectinoidea;Pectinidae;Pecten;Pecten maximus
UdeM00000037896 29159 216.9 UdeM00000037896 XP_011450518.2 59.3 172 70 0 147 318 58 229 1.50E-52 216.9 29159 cellular organisms;Eukaryota;Opisthokonta;Metazoa;Eumetazoa;Bilateria;Protostomia;Spiralia;Lophotrochozoa;Mollusca;Bivalvia;Autobranchia;Pteriomorphia;Ostreida;Ostreoidea;Ostreidae;Crassostrea;Crassostrea gigas
UdeM00000037898 29159 170.6 UdeM00000037898 XP_011450518.2 39.7 277 150 4 1 271 1 266 4.80E-39 170.6 29159 cellular organisms;Eukaryota;Opisthokonta;Metazoa;Eumetazoa;Bilateria;Protostomia;Spiralia;Lophotrochozoa;Mollusca;Bivalvia;Autobranchia;Pteriomorphia;Ostreida;Ostreoidea;Ostreidae;Crassostrea;Crassostrea gigas
UdeM00000037897 29159 170.6 UdeM00000037897 XP_011450518.2 39.7 277 150 4 1 271 1 266 7.30E-39 170.6 29159 cellular organisms;Eukaryota;Opisthokonta;Metazoa;Eumetazoa;Bilateria;Protostomia;Spiralia;Lophotrochozoa;Mollusca;Bivalvia;Autobranchia;Pteriomorphia;Ostreida;Ostreoidea;Ostreidae;Crassostrea;Crassostrea gigas
UdeM00000037900 29159 159.1 UdeM00000037900 XP_011450518.2 48.2 166 84 1 4 167 63 228 1.90E-35 159.1 29159 cellular organisms;Eukaryota;Opisthokonta;Metazoa;Eumetazoa;Bilateria;Protostomia;Spiralia;Lophotrochozoa;Mollusca;Bivalvia;Autobranchia;Pteriomorphia;Ostreida;Ostreoidea;Ostreidae;Crassostrea;Crassostrea gigas
UdeM00000037901 1735272 273.5 UdeM00000037901 XP_041371855.1 39.7 421 215 7 2 421 1 383 4.60E-70 273.5 1735272 cellular organisms;Eukaryota;Opisthokonta;Metazoa;Eumetazoa;Bilateria;Protostomia;Spiralia;Lophotrochozoa;Mollusca;Gastropoda;Neomphalina;Neomphaloidea;Peltospiridae;Gigantopelta;Gigantopelta aegis
UdeM00000037902 6573 404.4 UdeM00000037902 XP_021359356.1 70.4 406 115 2 1 405 1 402 1.70E-109 404.4 6573 cellular organisms;Eukaryota;Opisthokonta;Metazoa;Eumetazoa;Bilateria;Protostomia;Spiralia;Lophotrochozoa;Mollusca;Bivalvia;Autobranchia;Pteriomorphia;Pectinida;Pectinoidea;Pectinidae;Mizuhopecten;Mizuhopecten yessoensis
UdeM00000037903 6579 109.4 UdeM00000037903 XP_033751022.1 60 80 32 0 2 81 288 367 2.30E-21 109.4 6579 cellular organisms;Eukaryota;Opisthokonta;Metazoa;Eumetazoa;Bilateria;Protostomia;Spiralia;Lophotrochozoa;Mollusca;Bivalvia;Autobranchia;Pteriomorphia;Pectinida;Pectinoidea;Pectinidae;Pecten;Pecten maximus
UdeM00000037904 6579 238 UdeM00000037904 XP_033751022.1 67.9 162 52 0 1 162 206 367 8.40E-60 238 6579 cellular organisms;Eukaryota;Opisthokonta;Metazoa;Eumetazoa;Bilateria;Protostomia;Spiralia;Lophotrochozoa;Mollusca;Bivalvia;Autobranchia;Pteriomorphia;Pectinida;Pectinoidea;Pectinidae;Pecten;Pecten maximus

UdeM00000037905 6596 464.2 UdeM00000037905 XP_045156914.1 61.5 364
132 4 6 364 2578 2938 1.80E-127 464.2 6596 cellular
organisms;Eukaryota;Opisthokonta;Metazoa;Eumetazoa;Bilateria;Protostomia;
Spiralia;Lophotrochozoa;Mollusca;Bivalvia;Autobranchia;Heteroconchia;Euhe
terodonta;Imparidentia;Venerida;Veneroidea;Veneridae;Mercenaria;Mercenari
a mercenaria
UdeM00000037906 6596 3640.1 UdeM00000037906 XP_045156914.1 62.5
2466 898 15 11 2470 120 2564 0.00E+00 3640.1 6596
cellular
organisms;Eukaryota;Opisthokonta;Metazoa;Eumetazoa;Bilateria;Protostomia;
Spiralia;Lophotrochozoa;Mollusca;Bivalvia;Autobranchia;Heteroconchia;Euhe
terodonta;Imparidentia;Venerida;Veneroidea;Veneridae;Mercenaria;Mercenari
a mercenaria
UdeM00000037907 6596 3618.2 UdeM00000037907 XP_045156914.1 61.8
2493 887 16 1 2449 93 2564 0.00E+00 3618.2 6596
cellular
organisms;Eukaryota;Opisthokonta;Metazoa;Eumetazoa;Bilateria;Protostomia;
Spiralia;Lophotrochozoa;Mollusca;Bivalvia;Autobranchia;Heteroconchia;Euhe
terodonta;Imparidentia;Venerida;Veneroidea;Veneridae;Mercenaria;Mercenari
a mercenaria
UdeM00000037908 6579 429.9 UdeM00000037908 XP_033725915.1 52.7 419
193 5 10 426 2 417 3.90E-117 429.9 6579 cellular
organisms;Eukaryota;Opisthokonta;Metazoa;Eumetazoa;Bilateria;Protostomia;
Spiralia;Lophotrochozoa;Mollusca;Bivalvia;Autobranchia;Pteriomorphia;Pect
inida;Pectinoidea;Pectinidae;Pecten;Pecten maximus
UdeM00000037909 6579 412.9 UdeM00000037909 XP_033725915.1 52.4 410
190 5 1 408 11 417 4.80E-112 412.9 6579 cellular
organisms;Eukaryota;Opisthokonta;Metazoa;Eumetazoa;Bilateria;Protostomia;
Spiralia;Lophotrochozoa;Mollusca;Bivalvia;Autobranchia;Pteriomorphia;Pect
inida;Pectinoidea;Pectinidae;Pecten;Pecten maximus
UdeM00000037910 6579 407.1 UdeM00000037910 XP_033725915.1 49.3 418
207 5 17 432 2 416 2.80E-110 407.1 6579 cellular
organisms;Eukaryota;Opisthokonta;Metazoa;Eumetazoa;Bilateria;Protostomia;
Spiralia;Lophotrochozoa;Mollusca;Bivalvia;Autobranchia;Pteriomorphia;Pect
inida;Pectinoidea;Pectinidae;Pecten;Pecten maximus
UdeM00000037911 6573 180.6 UdeM00000037911 XP_021365673.1 32.7 269
177 4 5 273 153 417 2.70E-42 180.6 6573 cellular
organisms;Eukaryota;Opisthokonta;Metazoa;Eumetazoa;Bilateria;Protostomia;
Spiralia;Lophotrochozoa;Mollusca;Bivalvia;Autobranchia;Pteriomorphia;Pect
inida;Pectinoidea;Pectinidae;Mizuhopecten;Mizuhopecten yessoensis
UdeM00000037912 6596 276.9 UdeM00000037912 XP_045216411.1 35.9 429
259 9 1 428 9 422 5.00E-71 276.9 6596 cellular
organisms;Eukaryota;Opisthokonta;Metazoa;Eumetazoa;Bilateria;Protostomia;
Spiralia;Lophotrochozoa;Mollusca;Bivalvia;Autobranchia;Heteroconchia;Euhe
terodonta;Imparidentia;Venerida;Veneroidea;Veneridae;Mercenaria;Mercenari
a mercenaria
UdeM00000037913 6596 129.8 UdeM00000037913 XP_045216866.1 54.5 290
107 7 1 284 1 271 5.50E-27 129.8 6596 cellular
organisms;Eukaryota;Opisthokonta;Metazoa;Eumetazoa;Bilateria;Protostomia;
Spiralia;Lophotrochozoa;Mollusca;Bivalvia;Autobranchia;Heteroconchia;Euhe
terodonta;Imparidentia;Venerida;Veneroidea;Veneridae;Mercenaria;Mercenari
a mercenaria
UdeM00000037914 6565 487.3 UdeM00000037914 XP_022332212.1 57.5 452
157 9 658 1102 590 1013 5.30E-134 487.3 6565 cellular

organisms;Eukaryota;Opisthokonta;Metazoa;Eumetazoa;Bilateria;Protostomia;Spiralia;Lophotrochozoa;Mollusca;Bivalvia;Autobranchia;Pteriomorphia;Ostreida;Ostreoidea;Ostreidae;Crassostrea;Crassostrea virginica
UdeM00000037915 6565 627.9 UdeM00000037915 XP_022334577.1 48.3 650 207 4 12 659 8 530 1.50E-176 627.9 6565 cellular organisms;Eukaryota;Opisthokonta;Metazoa;Eumetazoa;Bilateria;Protostomia;Spiralia;Lophotrochozoa;Mollusca;Bivalvia;Autobranchia;Pteriomorphia;Ostreida;Ostreoidea;Ostreidae;Crassostrea;Crassostrea virginica
UdeM00000037916 6579 323.6 UdeM00000037916 XP_033750900.1 56.5 336 135 5 1 327 2 335 3.10E-85 323.6 6579 cellular organisms;Eukaryota;Opisthokonta;Metazoa;Eumetazoa;Bilateria;Protostomia;Spiralia;Lophotrochozoa;Mollusca;Bivalvia;Autobranchia;Pteriomorphia;Pectinida;Pectinoidea;Pectinidae;Pecten;Pecten maximus
UdeM00000037917 6579 589 UdeM00000037917 XP_033749902.1 77.6 370 81 1 6 373 1 370 4.40E-165 589 6579 cellular organisms;Eukaryota;Opisthokonta;Metazoa;Eumetazoa;Bilateria;Protostomia;Spiralia;Lophotrochozoa;Mollusca;Bivalvia;Autobranchia;Pteriomorphia;Pectinida;Pectinoidea;Pectinidae;Pecten;Pecten maximus
UdeM00000037918 6579 205.7 UdeM00000037918 XP_033746397.1 38.5 265 161 1 1 263 1 265 7.40E-50 205.7 6579 cellular organisms;Eukaryota;Opisthokonta;Metazoa;Eumetazoa;Bilateria;Protostomia;Spiralia;Lophotrochozoa;Mollusca;Bivalvia;Autobranchia;Pteriomorphia;Pectinida;Pectinoidea;Pectinidae;Pecten;Pecten maximus
UdeM00000037919 54343 113.2 UdeM00000037919 XP_032366298.1 27.9 301 78 3 1 163 184 483 3.20E-22 113.2 54343 cellular organisms;Eukaryota;Opisthokonta;Metazoa;Eumetazoa;Bilateria;Deuterostomia;Chordata;Craniata;Vertebrata;Gnathostomata;Teleostomi;Euteleostomi;Actinopterygii;Actinopteri;Neopterygii;Teleostei;Osteoglossocephalai;Clupeocephala;Euteleosteomorpha;Neoteleostei;Eurypterygia;Ctenosquamata;Acanthomorphata;Euacanthomorphacea;Percomorphaceae;Eupercaria;Perciformes;Percoidei;Percidae;Etheostomatinae;Etheostoma;Oligocephalus;Etheostoma spectabile
UdeM00000037920 6596 224.6 UdeM00000037920 XP_045216171.1 48.1 231 99 2 39 269 35 244 1.80E-55 224.6 6596 cellular organisms;Eukaryota;Opisthokonta;Metazoa;Eumetazoa;Bilateria;Protostomia;Spiralia;Lophotrochozoa;Mollusca;Bivalvia;Autobranchia;Heteroconchia;Euheterodonta;Imparidentia;Venerida;Veneroidea;Veneridae;Mercenaria;Mercenaria mercenaria
UdeM00000023390 176946 65.5 UdeM00000023390 XP_015745591.2 41.2 85 43 2 1 78 2514 2598 5.00E-08 65.5 176946 cellular organisms;Eukaryota;Opisthokonta;Metazoa;Eumetazoa;Bilateria;Deuterostomia;Chordata;Craniata;Vertebrata;Gnathostomata;Teleostomi;Euteleostomi;Sarcopterygii;Dipnotetrapodomorpha;Tetrapoda;Amniota;Sauropsida;Sauria;Lepidosauria;Squamata;Bifurcata;Unidentata;Episquamata;Toxicofera;Serpentes;Henophidia;Pythonidae;Python;Python bivittatus
UdeM00000023391 6596 228.8 UdeM00000023391 XP_045194118.1 39 372 202 10 41 399 1544 1903 1.20E-56 228.8 6596 cellular organisms;Eukaryota;Opisthokonta;Metazoa;Eumetazoa;Bilateria;Protostomia;Spiralia;Lophotrochozoa;Mollusca;Bivalvia;Autobranchia;Heteroconchia;Euheterodonta;Imparidentia;Venerida;Veneroidea;Veneridae;Mercenaria;Mercenaria mercenaria
UdeM00000023392 6596 3577.7 UdeM00000023392 XP_045189836.1 61.9 3061 1074 32 1 3022 1 3007 0.00E+00 3577.7 6596 cellular

organisms;Eukaryota;Opisthokonta;Metazoa;Eumetazoa;Bilateria;Protostomia;Spiralia;Lophotrochozoa;Mollusca;Bivalvia;Autobranchia;Heteroconchia;Euheterodonta;Imparidentia;Venerida;Veneroidea;Veneridae;Mercenaria;Mercenaria mercenaria
UdeM00000023393 6596 281.2 UdeM00000023393 XP_045188529.1 52.2 356 103 10 1 302 8 350 1.60E-72 281.2 6596 cellular organisms;Eukaryota;Opisthokonta;Metazoa;Eumetazoa;Bilateria;Protostomia;Spiralia;Lophotrochozoa;Mollusca;Bivalvia;Autobranchia;Heteroconchia;Euheterodonta;Imparidentia;Venerida;Veneroidea;Veneridae;Mercenaria;Mercenaria mercenaria
UdeM00000023394 6579 238.4 UdeM00000023394 XP_033757210.1 54.8 263 93 6 58 303 65 318 1.20E-59 238.4 6579 cellular organisms;Eukaryota;Opisthokonta;Metazoa;Eumetazoa;Bilateria;Protostomia;Spiralia;Lophotrochozoa;Mollusca;Bivalvia;Autobranchia;Pteriomorphia;Pectinida;Pectinoidea;Pectinidae;Pecten;Pecten maximus
UdeM00000023395 6565 232.3 UdeM00000023395 XP_022339812.1 56.1 239 94 7 58 289 59 293 8.00E-58 232.3 6565 cellular organisms;Eukaryota;Opisthokonta;Metazoa;Eumetazoa;Bilateria;Protostomia;Spiralia;Lophotrochozoa;Mollusca;Bivalvia;Autobranchia;Pteriomorphia;Ostreida;Ostreoidea;Ostreidae;Crassostrea;Crassostrea virginica
UdeM00000023396 6565 232.6 UdeM00000023396 XP_022339812.1 56.1 239 94 7 58 289 59 293 6.20E-58 232.6 6565 cellular organisms;Eukaryota;Opisthokonta;Metazoa;Eumetazoa;Bilateria;Protostomia;Spiralia;Lophotrochozoa;Mollusca;Bivalvia;Autobranchia;Pteriomorphia;Ostreida;Ostreoidea;Ostreidae;Crassostrea;Crassostrea virginica
UdeM00000023397 6579 228.4 UdeM00000023397 XP_033757210.1 58.2 244 92 7 48 283 65 306 1.10E-56 228.4 6579 cellular organisms;Eukaryota;Opisthokonta;Metazoa;Eumetazoa;Bilateria;Protostomia;Spiralia;Lophotrochozoa;Mollusca;Bivalvia;Autobranchia;Pteriomorphia;Pectinida;Pectinoidea;Pectinidae;Pecten;Pecten maximus
UdeM00000023398 218467 87.4 UdeM00000023398 XP_023232316.1 36.2 138 84 2 61 197 64 198 2.30E-14 87.4 218467 cellular organisms;Eukaryota;Opisthokonta;Metazoa;Eumetazoa;Bilateria;Protostomia;Ecdysozoa;Panarthropoda;Arthropoda;Chelicerata;Arachnida;Scorpiones;Buthida;Buthoidea;Buthidae;Centruroides;Centruroides sculpturatus
UdeM00000023401 6596 322.8 UdeM00000023401 XP_045167151.1 33.1 707 438 14 28 725 94 774 1.20E-84 322.8 6596 cellular organisms;Eukaryota;Opisthokonta;Metazoa;Eumetazoa;Bilateria;Protostomia;Spiralia;Lophotrochozoa;Mollusca;Bivalvia;Autobranchia;Heteroconchia;Euheterodonta;Imparidentia;Venerida;Veneroidea;Veneridae;Mercenaria;Mercenaria mercenaria
UdeM00000023402 29159 295.8 UdeM00000023402 XP_019922954.2 46.7 347 159 7 18 342 632 974 7.10E-77 295.8 29159 cellular organisms;Eukaryota;Opisthokonta;Metazoa;Eumetazoa;Bilateria;Protostomia;Spiralia;Lophotrochozoa;Mollusca;Bivalvia;Autobranchia;Pteriomorphia;Ostreida;Ostreoidea;Ostreidae;Crassostrea;Crassostrea gigas
UdeM00000023404 29159 335.9 UdeM00000023404 XP_034333436.1 31.7 854 517 22 3 838 73 878 1.60E-88 335.9 29159 cellular organisms;Eukaryota;Opisthokonta;Metazoa;Eumetazoa;Bilateria;Protostomia;Spiralia;Lophotrochozoa;Mollusca;Bivalvia;Autobranchia;Pteriomorphia;Ostreida;Ostreoidea;Ostreidae;Crassostrea;Crassostrea gigas
UdeM00000023405 6579 290.8 UdeM00000023405 XP_033756471.1 44.8 337 173 4 18 342 657 992 2.30E-75 290.8 6579 cellular

organisms;Eukaryota;Opisthokonta;Metazoa;Eumetazoa;Bilateria;Protostomia;Spiralia;Lophotrochozoa;Mollusca;Bivalvia;Autobranchia;Pteriomorphia;Pectinida;Pectinoidea;Pectinidae;Pecten;Pecten maximus
UdeM00000023406 6945 87.8 UdeM00000023406 XP_040065097.1 29.5 210 117 6 24 207 50 254 1.80E-14 87.8 6945 cellular organisms;Eukaryota;Opisthokonta;Metazoa;Eumetazoa;Bilateria;Protostomia;Ecdysozoa;Panarthropoda;Arthropoda;Chelicerata;Arachnida;Acari;Parasitiformes;Ixodida;Ixodoidea;Ixodidae;Ixodinae;Ixodes;Ixodes scapularis
UdeM00000023407 218467 67.8 UdeM00000023407 XP_023219246.1 31.1 132 74 2 47 161 87 218 1.80E-08 67.8 218467 cellular organisms;Eukaryota;Opisthokonta;Metazoa;Eumetazoa;Bilateria;Protostomia;Ecdysozoa;Panarthropoda;Arthropoda;Chelicerata;Arachnida;Scorpiones;Buthida;Buthoidea;Buthidae;Centruroides;Centruroides sculpturatus
UdeM00000023408 6945 87 UdeM00000023408 XP_040065097.1 31.2 170 91 4 59 202 85 254 4.50E-14 87 6945 cellular organisms;Eukaryota;Opisthokonta;Metazoa;Eumetazoa;Bilateria;Protostomia;Ecdysozoa;Panarthropoda;Arthropoda;Chelicerata;Arachnida;Acari;Parasitiformes;Ixodida;Ixodoidea;Ixodidae;Ixodinae;Ixodes;Ixodes scapularis
UdeM00000023409 6579 84.3 UdeM00000023409 XP_033727573.1 31.3 179 111 3 28 195 66 243 2.90E-13 84.3 6579 cellular organisms;Eukaryota;Opisthokonta;Metazoa;Eumetazoa;Bilateria;Protostomia;Spiralia;Lophotrochozoa;Mollusca;Bivalvia;Autobranchia;Pteriomorphia;Pectinida;Pectinoidea;Pectinidae;Pecten;Pecten maximus
UdeM00000023410 29159 345.1 UdeM00000023410 XP_034333436.1 31.2 901 547 25 36 912 27 878 2.90E-91 345.1 29159 cellular organisms;Eukaryota;Opisthokonta;Metazoa;Eumetazoa;Bilateria;Protostomia;Spiralia;Lophotrochozoa;Mollusca;Bivalvia;Autobranchia;Pteriomorphia;Ostreida;Ostreoidea;Ostreidae;Crassostrea;Crassostrea gigas
UdeM00000023411 29159 297 UdeM00000023411 XP_019922954.2 46.4 347 160 7 18 342 632 974 3.20E-77 297 29159 cellular organisms;Eukaryota;Opisthokonta;Metazoa;Eumetazoa;Bilateria;Protostomia;Spiralia;Lophotrochozoa;Mollusca;Bivalvia;Autobranchia;Pteriomorphia;Ostreida;Ostreoidea;Ostreidae;Crassostrea;Crassostrea gigas
UdeM00000023412 6565 349.7 UdeM00000023412 XP_022295767.1 41.6 490 271 10 38 516 43 528 6.50E-93 349.7 6565 cellular organisms;Eukaryota;Opisthokonta;Metazoa;Eumetazoa;Bilateria;Protostomia;Spiralia;Lophotrochozoa;Mollusca;Bivalvia;Autobranchia;Pteriomorphia;Ostreida;Ostreoidea;Ostreidae;Crassostrea;Crassostrea virginica
UdeM00000023413 6565 133.7 UdeM00000023413 XP_022292556.1 53.4 116 53 1 7 121 61 176 1.70E-28 133.7 6565 cellular organisms;Eukaryota;Opisthokonta;Metazoa;Eumetazoa;Bilateria;Protostomia;Spiralia;Lophotrochozoa;Mollusca;Bivalvia;Autobranchia;Pteriomorphia;Ostreida;Ostreoidea;Ostreidae;Crassostrea;Crassostrea virginica
UdeM00000023414 29159 347.8 UdeM00000023414 XP_034302097.1 32.7 712 325 13 4 597 188 863 2.80E-92 347.8 29159 cellular organisms;Eukaryota;Opisthokonta;Metazoa;Eumetazoa;Bilateria;Protostomia;Spiralia;Lophotrochozoa;Mollusca;Bivalvia;Autobranchia;Pteriomorphia;Ostreida;Ostreoidea;Ostreidae;Crassostrea;Crassostrea gigas
UdeM00000023416 6573 147.1 UdeM00000023416 XP_021355903.1 25 643 307 14 5 488 67 693 6.60E-32 147.1 6573 cellular organisms;Eukaryota;Opisthokonta;Metazoa;Eumetazoa;Bilateria;Protostomia;Spiralia;Lophotrochozoa;Mollusca;Bivalvia;Autobranchia;Pteriomorphia;Pectinida;Pectinoidea;Pectinidae;Mizuhopecten;Mizuhopecten yessoensis

UdeM00000023417 6596 944.9 UdeM00000023417 XP_045189722.1 51.5 956
434 12 3 952 6 937 8.00E-272 944.9 6596 cellular organisms;Eukaryota;Opisthokonta;Metazoa;Eumetazoa;Bilateria;Protostomia;Spiralia;Lophotrochozoa;Mollusca;Bivalvia;Autobranchia;Heteroconchia;Euheterodonta;Imparidentia;Venerida;Veneroidea;Veneridae;Mercenaria;Mercenaria mercenaria
UdeM00000023418 6596 632.5 UdeM00000023418 XP_045189404.1 69.1 489
149 1 7 493 47 535 4.60E-178 632.5 6596 cellular organisms;Eukaryota;Opisthokonta;Metazoa;Eumetazoa;Bilateria;Protostomia;Spiralia;Lophotrochozoa;Mollusca;Bivalvia;Autobranchia;Heteroconchia;Euheterodonta;Imparidentia;Venerida;Veneroidea;Veneridae;Mercenaria;Mercenaria mercenaria
UdeM00000023419 6565 147.1 UdeM00000023419 XP_022338736.1 35.8 218
134 4 64 279 261 474 4.00E-32 147.1 6565 cellular organisms;Eukaryota;Opisthokonta;Metazoa;Eumetazoa;Bilateria;Protostomia;Spiralia;Lophotrochozoa;Mollusca;Bivalvia;Autobranchia;Pteriomorphia;Ostreida;Ostreoidea;Ostreidae;Crassostrea;Crassostrea virginica
UdeM00000023420 6596 70.9 UdeM00000023420 XP_045190852.1 33.3 207
110 8 27 216 465 660 3.60E-09 70.9 6596 cellular organisms;Eukaryota;Opisthokonta;Metazoa;Eumetazoa;Bilateria;Protostomia;Spiralia;Lophotrochozoa;Mollusca;Bivalvia;Autobranchia;Heteroconchia;Euheterodonta;Imparidentia;Venerida;Veneroidea;Veneridae;Mercenaria;Mercenaria mercenaria
UdeM00000023421 6579 684.1 UdeM00000023421 XP_033757509.1 69.1 476
143 2 75 548 22 495 1.80E-193 684.1 6579 cellular organisms;Eukaryota;Opisthokonta;Metazoa;Eumetazoa;Bilateria;Protostomia;Spiralia;Lophotrochozoa;Mollusca;Bivalvia;Autobranchia;Pteriomorphia;Pectinida;Pectinoidea;Pectinidae;Pecten;Pecten maximus
UdeM00000023422 7936 90.5 UdeM00000023422 XP_035247672.1 30.7 202
120 3 4 205 22 203 4.40E-15 90.5 7936 cellular organisms;Eukaryota;Opisthokonta;Metazoa;Eumetazoa;Bilateria;Deuterostomia;Chordata;Craniata;Vertebrata;Gnathostomata;Teleostomi;Euteleostomi;Actinopterygii;Actinopteri;Neopterygii;Teleostei;Elopocephalai;Elopocephala;Elopomorpha;Anguilliformes;Anguillidae;Anguilla;Anguilla anguilla
UdeM00000023423 6596 354.4 UdeM00000023423 XP_045190446.1 59 317
116 5 38 344 5 317 1.70E-94 354.4 6596 cellular organisms;Eukaryota;Opisthokonta;Metazoa;Eumetazoa;Bilateria;Protostomia;Spiralia;Lophotrochozoa;Mollusca;Bivalvia;Autobranchia;Heteroconchia;Euheterodonta;Imparidentia;Venerida;Veneroidea;Veneridae;Mercenaria;Mercenaria mercenaria
UdeM00000023424 6596 640.6 UdeM00000023424 XP_045190456.1 59.3 533
196 6 40 553 66 596 1.90E-180 640.6 6596 cellular organisms;Eukaryota;Opisthokonta;Metazoa;Eumetazoa;Bilateria;Protostomia;Spiralia;Lophotrochozoa;Mollusca;Bivalvia;Autobranchia;Heteroconchia;Euheterodonta;Imparidentia;Venerida;Veneroidea;Veneridae;Mercenaria;Mercenaria mercenaria
UdeM00000023425 29159 86.3 UdeM00000023425 XP_011423601.2 23.6 301
204 9 29 323 4 284 8.30E-14 86.3 29159 cellular organisms;Eukaryota;Opisthokonta;Metazoa;Eumetazoa;Bilateria;Protostomia;Spiralia;Lophotrochozoa;Mollusca;Bivalvia;Autobranchia;Pteriomorphia;Ostreida;Ostreoidea;Ostreidae;Crassostrea;Crassostrea gigas
UdeM00000023426 6596 692.2 UdeM00000023426 XP_045189100.1 66 664
179 13 1 659 1 622 6.50E-196 692.2 6596 cellular organisms;Eukaryota;Opisthokonta;Metazoa;Eumetazoa;Bilateria;Protostomia;

Spiralia;Lophotrochozoa;Mollusca;Bivalvia;Autobranchia;Heteroconchia;Euheterodonta;Imparidentia;Venerida;Veneroidea;Veneridae;Mercenaria;Mercenaria mercenaria
UdeM00000023429 225164 224.6 UdeM00000023429 XP_009052279.1 44.7 284 130 6 1 268 1 273 2.00E-55 224.6 225164 cellular organisms;Eukaryota;Opisthokonta;Metazoa;Eumetazoa;Bilateria;Protostomia;Spiralia;Lophotrochozoa;Mollusca;Gastropoda;Patellogastropoda;Lottioidea;Lottiidae;Lottia;Lottia gigantea
UdeM00000023432 6573 196.8 UdeM00000023432 XP_021364000.1 33.3 480 279 14 1 463 1 456 7.00E-47 196.8 6573 cellular organisms;Eukaryota;Opisthokonta;Metazoa;Eumetazoa;Bilateria;Protostomia;Spiralia;Lophotrochozoa;Mollusca;Bivalvia;Autobranchia;Pteriomorphia;Pectinida;Pectinoidea;Pectinidae;Mizuhopecten;Mizuhopecten yessoensis
UdeM00000023433 46514 120.6 UdeM00000023433 XP_038064356.1 33.6 220 141 4 7 226 45 259 3.00E-24 120.6 46514 cellular organisms;Eukaryota;Opisthokonta;Metazoa;Eumetazoa;Bilateria;Deuterostomia;Echinodermata;Eleutherozoa;Asterozoa;Asteroidea;Valvatacea;Valvatida;Asterinidae;Patiria;Patiria miniata
UdeM00000023434 7668 156 UdeM00000023434 XP_798461.5 52.2 134 63 1 236 369 24 156 1.90E-34 156 7668 cellular organisms;Eukaryota;Opisthokonta;Metazoa;Eumetazoa;Bilateria;Deuterostomia;Echinodermata;Eleutherozoa;Echinozoa;Echinoidea;Euechinoidea;Echinacea;Camarodonta;Echinidea;Strongylocentrotidae;Strongylocentrotus;Strongylocentrotus purpuratus
UdeM00000023435 7668 205.7 UdeM00000023435 XP_798461.5 51.6 192 87 2 1 187 14 204 9.00E-50 205.7 7668 cellular organisms;Eukaryota;Opisthokonta;Metazoa;Eumetazoa;Bilateria;Deuterostomia;Echinodermata;Eleutherozoa;Echinozoa;Echinoidea;Euechinoidea;Echinacea;Camarodonta;Echinidea;Strongylocentrotidae;Strongylocentrotus;Strongylocentrotus purpuratus
UdeM00000023436 50429 663.3 UdeM00000023436 XP_022788227.1 35.5 1055 591 26 24 999 30 1074 4.90E-187 663.3 50429 cellular organisms;Eukaryota;Opisthokonta;Metazoa;Eumetazoa;Cnidaria;Anthozoa;Hexacorallia;Scleractinia;Astrocoeniina;Pocilloporidae;Stylophora;Stylophora pistillata
UdeM00000023437 6596 271.2 UdeM00000023437 XP_045189601.1 44.9 356 182 3 4 346 22 376 1.90E-69 271.2 6596 cellular organisms;Eukaryota;Opisthokonta;Metazoa;Eumetazoa;Bilateria;Protostomia;Spiralia;Lophotrochozoa;Mollusca;Bivalvia;Autobranchia;Heteroconchia;Euheterodonta;Imparidentia;Venerida;Veneroidea;Veneridae;Mercenaria;Mercenaria mercenaria
UdeM00000023438 6596 131.3 UdeM00000023438 XP_045189601.1 52.9 121 55 1 11 131 45 163 1.00E-27 131.3 6596 cellular organisms;Eukaryota;Opisthokonta;Metazoa;Eumetazoa;Bilateria;Protostomia;Spiralia;Lophotrochozoa;Mollusca;Bivalvia;Autobranchia;Heteroconchia;Euheterodonta;Imparidentia;Venerida;Veneroidea;Veneridae;Mercenaria;Mercenaria mercenaria
UdeM00000023440 1735272 449.9 UdeM00000023440 XP_041370240.1 61.1 352 131 1 5 350 13 364 3.30E-123 449.9 1735272 cellular organisms;Eukaryota;Opisthokonta;Metazoa;Eumetazoa;Bilateria;Protostomia;Spiralia;Lophotrochozoa;Mollusca;Gastropoda;Neomphalina;Neomphaloidea;Peltospiridae;Gigantopelta;Gigantopelta aegis

UdeM00000023439 1735272 455.3 UdeM00000023439 XP_041370240.1 60.5
362 137 1 5 360 13 374 9.30E-125 455.3 1735272
cellular
organisms;Eukaryota;Opisthokonta;Metazoa;Eumetazoa;Bilateria;Protostomia;
Spiralia;Lophotrochozoa;Mollusca;Gastropoda;Neomphalina;Neomphaloidea;Pel
tospiridae;Gigantopelta;Gigantopelta aegis
UdeM00000023441 1735272 705.3 UdeM00000023441 XP_041370245.1 43.2
907 376 15 23 898 76 874 1.00E-199 705.3 1735272
cellular
organisms;Eukaryota;Opisthokonta;Metazoa;Eumetazoa;Bilateria;Protostomia;
Spiralia;Lophotrochozoa;Mollusca;Gastropoda;Neomphalina;Neomphaloidea;Pel
tospiridae;Gigantopelta;Gigantopelta aegis
UdeM00000023442 1735272 706.4 UdeM00000023442 XP_041370245.1 43.3
906 375 15 35 909 77 874 4.60E-200 706.4 1735272
cellular
organisms;Eukaryota;Opisthokonta;Metazoa;Eumetazoa;Bilateria;Protostomia;
Spiralia;Lophotrochozoa;Mollusca;Gastropoda;Neomphalina;Neomphaloidea;Pel
tospiridae;Gigantopelta;Gigantopelta aegis
UdeM00000023443 6579 593.6 UdeM00000023443 XP_033757345.1 54.7 547
171 3 1 545 1 472 2.60E-166 593.6 6579 cellular
organisms;Eukaryota;Opisthokonta;Metazoa;Eumetazoa;Bilateria;Protostomia;
Spiralia;Lophotrochozoa;Mollusca;Bivalvia;Autobranchia;Pteriomorphia;Pect
inida;Pectinoidea;Pectinidae;Pecten;Pecten maximus
UdeM00000023444 6596 344.7 UdeM00000023444 XP_045188673.1 35.9 496
312 3 124 618 186 676 2.40E-91 344.7 6596 cellular
organisms;Eukaryota;Opisthokonta;Metazoa;Eumetazoa;Bilateria;Protostomia;
Spiralia;Lophotrochozoa;Mollusca;Bivalvia;Autobranchia;Heteroconchia;Euhe
terodonta;Imparidentia;Venerida;Veneroidea;Veneridae;Mercenaria;Mercenari
a mercenaria
UdeM00000023445 6596 365.2 UdeM00000023445 XP_045188673.1 36.2 497
309 5 113 607 186 676 1.70E-97 365.2 6596 cellular
organisms;Eukaryota;Opisthokonta;Metazoa;Eumetazoa;Bilateria;Protostomia;
Spiralia;Lophotrochozoa;Mollusca;Bivalvia;Autobranchia;Heteroconchia;Euhe
terodonta;Imparidentia;Venerida;Veneroidea;Veneridae;Mercenaria;Mercenari
a mercenaria
UdeM00000023446 6596 334.7 UdeM00000023446 XP_045188673.1 32.4 612
385 7 10 612 85 676 2.40E-88 334.7 6596 cellular
organisms;Eukaryota;Opisthokonta;Metazoa;Eumetazoa;Bilateria;Protostomia;
Spiralia;Lophotrochozoa;Mollusca;Bivalvia;Autobranchia;Heteroconchia;Euhe
terodonta;Imparidentia;Venerida;Veneroidea;Veneridae;Mercenaria;Mercenari
a mercenaria
UdeM00000023447 6596 337 UdeM00000023447 XP_045188673.1 32.3 592
376 5 35 618 102 676 4.90E-89 337 6596 cellular
organisms;Eukaryota;Opisthokonta;Metazoa;Eumetazoa;Bilateria;Protostomia;
Spiralia;Lophotrochozoa;Mollusca;Bivalvia;Autobranchia;Heteroconchia;Euhe
terodonta;Imparidentia;Venerida;Veneroidea;Veneridae;Mercenaria;Mercenari
a mercenaria
UdeM00000023448 6596 365.5 UdeM00000023448 XP_045188673.1 36.4 497
308 5 113 607 186 676 1.30E-97 365.5 6596 cellular
organisms;Eukaryota;Opisthokonta;Metazoa;Eumetazoa;Bilateria;Protostomia;
Spiralia;Lophotrochozoa;Mollusca;Bivalvia;Autobranchia;Heteroconchia;Euhe
terodonta;Imparidentia;Venerida;Veneroidea;Veneridae;Mercenaria;Mercenari
a mercenaria

UdeM00000023449 6596 336.7 UdeM00000023449 XP_045188673.1 32.5 616
379 7 10 612 85 676 6.40E-89 336.7 6596 cellular
organisms;Eukaryota;Opisthokonta;Metazoa;Eumetazoa;Bilateria;Protostomia;
Spiralia;Lophotrochozoa;Mollusca;Bivalvia;Autobranchia;Heteroconchia;Euhe
terodonta;Imparidentia;Venerida;Veneroidea;Veneridae;Mercenaria;Mercenari
a mercenaria
UdeM00000023450 6596 312.4 UdeM00000023450 XP_045188673.1 38 416
252 3 8 422 266 676 8.90E-82 312.4 6596 cellular
organisms;Eukaryota;Opisthokonta;Metazoa;Eumetazoa;Bilateria;Protostomia;
Spiralia;Lophotrochozoa;Mollusca;Bivalvia;Autobranchia;Heteroconchia;Euhe
terodonta;Imparidentia;Venerida;Veneroidea;Veneridae;Mercenaria;Mercenari
a mercenaria
UdeM00000023451 6596 362.5 UdeM00000023451 XP_045188673.1 36.2 497
309 5 113 607 186 676 1.10E-96 362.5 6596 cellular
organisms;Eukaryota;Opisthokonta;Metazoa;Eumetazoa;Bilateria;Protostomia;
Spiralia;Lophotrochozoa;Mollusca;Bivalvia;Autobranchia;Heteroconchia;Euhe
terodonta;Imparidentia;Venerida;Veneroidea;Veneridae;Mercenaria;Mercenari
a mercenaria
UdeM00000023452 6596 393.7 UdeM00000023452 XP_045189606.1 70.7 263
77 0 1 263 5 267 1.90E-106 393.7 6596 cellular
organisms;Eukaryota;Opisthokonta;Metazoa;Eumetazoa;Bilateria;Protostomia;
Spiralia;Lophotrochozoa;Mollusca;Bivalvia;Autobranchia;Heteroconchia;Euhe
terodonta;Imparidentia;Venerida;Veneroidea;Veneridae;Mercenaria;Mercenari
a mercenaria
UdeM00000023453 6573 168.7 UdeM00000023453 XP_021373558.1 51.7 172
82 1 8 178 170 341 6.80E-39 168.7 6573 cellular
organisms;Eukaryota;Opisthokonta;Metazoa;Eumetazoa;Bilateria;Protostomia;
Spiralia;Lophotrochozoa;Mollusca;Bivalvia;Autobranchia;Pteriomorphia;Pect
inida;Pectinoidea;Pectinidae;Mizuhopecten;Mizuhopecten yessoensis
UdeM00000023454 6573 256.5 UdeM00000023454 XP_021374592.1 57.6 236
87 1 5 227 1 236 3.10E-65 256.5 6573 cellular
organisms;Eukaryota;Opisthokonta;Metazoa;Eumetazoa;Bilateria;Protostomia;
Spiralia;Lophotrochozoa;Mollusca;Bivalvia;Autobranchia;Pteriomorphia;Pect
inida;Pectinoidea;Pectinidae;Mizuhopecten;Mizuhopecten yessoensis
UdeM00000023455 6596 672.9 UdeM00000023455 XP_045189092.1 69.6 494
126 6 4 497 41 510 3.10E-190 672.9 6596 cellular
organisms;Eukaryota;Opisthokonta;Metazoa;Eumetazoa;Bilateria;Protostomia;
Spiralia;Lophotrochozoa;Mollusca;Bivalvia;Autobranchia;Heteroconchia;Euhe
terodonta;Imparidentia;Venerida;Veneroidea;Veneridae;Mercenaria;Mercenari
a mercenaria
UdeM00000023456 6573 316.2 UdeM00000023456 XP_021376429.1 59.3 243
99 0 17 259 18 260 3.80E-83 316.2 6573 cellular
organisms;Eukaryota;Opisthokonta;Metazoa;Eumetazoa;Bilateria;Protostomia;
Spiralia;Lophotrochozoa;Mollusca;Bivalvia;Autobranchia;Pteriomorphia;Pect
inida;Pectinoidea;Pectinidae;Mizuhopecten;Mizuhopecten yessoensis
UdeM00000023457 6596 317 UdeM00000023457 XP_045189438.1 59.6 277
112 0 34 310 32 308 2.70E-83 317 6596 cellular
organisms;Eukaryota;Opisthokonta;Metazoa;Eumetazoa;Bilateria;Protostomia;
Spiralia;Lophotrochozoa;Mollusca;Bivalvia;Autobranchia;Heteroconchia;Euhe
terodonta;Imparidentia;Venerida;Veneroidea;Veneridae;Mercenaria;Mercenari
a mercenaria
UdeM00000023458 6596 75.9 UdeM00000023458 XP_045207486.1 42.2 83
47 1 1 82 95 177 5.70E-11 75.9 6596 cellular
organisms;Eukaryota;Opisthokonta;Metazoa;Eumetazoa;Bilateria;Protostomia;

Spiralia;Lophotrochozoa;Mollusca;Bivalvia;Autobranchia;Heteroconchia;Euheterodonta;Imparidentia;Venerida;Veneroidea;Veneridae;Mercenaria;Mercenaria mercenaria
UdeM00000023459 50429 104.8 UdeM00000023459 XP_022783431.1 54.3 94 43 0 1 94 627 720 7.50E-20 104.8 50429 cellular organisms;Eukaryota;Opisthokonta;Metazoa;Eumetazoa;Cnidaria;Anthozoa;Hexacorallia;Scleractinia;Astrocoeniina;Pocilloporidae;Stylophora;Stylophora pistillata
UdeM00000023460 29159 136 UdeM00000023460 XP_011451097.2 28.2 369 204 11 1 330 1 347 1.20E-28 136 29159 cellular organisms;Eukaryota;Opisthokonta;Metazoa;Eumetazoa;Bilateria;Protostomia;Spiralia;Lophotrochozoa;Mollusca;Bivalvia;Autobranchia;Pteriomorphia;Ostreida;Ostreoidea;Ostreidae;Crassostrea;Crassostrea gigas
UdeM00000023461 70779 68.6 UdeM00000023461 XP_015777172.1 41.1 73 43 0 7 79 65 137 4.80E-09 68.6 70779 cellular organisms;Eukaryota;Opisthokonta;Metazoa;Eumetazoa;Cnidaria;Anthozoa;Hexacorallia;Scleractinia;Astrocoeniina;Acroporidae;Acropora;Acropora digitifera
UdeM00000023462 6596 632.1 UdeM00000023462 XP_045188501.1 76.5 387 91 0 44 430 40 426 5.20E-178 632.1 6596 cellular organisms;Eukaryota;Opisthokonta;Metazoa;Eumetazoa;Bilateria;Protostomia;Spiralia;Lophotrochozoa;Mollusca;Bivalvia;Autobranchia;Heteroconchia;Euheterodonta;Imparidentia;Venerida;Veneroidea;Veneridae;Mercenaria;Mercenaria mercenaria
UdeM00000023463 7574 73.6 UdeM00000023463 XP_013393409.1 48.7 76 34 1 19 89 200 275 2.80E-10 73.6 7574 cellular organisms;Eukaryota;Opisthokonta;Metazoa;Eumetazoa;Bilateria;Protostomia;Spiralia;Lophotrochozoa;Brachiopoda;Linguliformea;Lingulata;Lingulida;Linguloidea;Lingulidae;Lingula;Lingula anatina
UdeM00000023464 1735272 100.9 UdeM00000023464 XP_041370402.1 46.7 107 52 2 12 118 103 204 1.40E-18 100.9 1735272 cellular organisms;Eukaryota;Opisthokonta;Metazoa;Eumetazoa;Bilateria;Protostomia;Spiralia;Lophotrochozoa;Mollusca;Gastropoda;Neomphalina;Neomphaloidea;Peltospiridae;Gigantopelta;Gigantopelta aegis
UdeM00000023465 1735272 576.2 UdeM00000023465 XP_041369149.1 70.1 391 117 0 1 391 1 391 3.10E-161 576.2 1735272 cellular organisms;Eukaryota;Opisthokonta;Metazoa;Eumetazoa;Bilateria;Protostomia;Spiralia;Lophotrochozoa;Mollusca;Gastropoda;Neomphalina;Neomphaloidea;Peltospiridae;Gigantopelta;Gigantopelta aegis
UdeM00000023466 6596 371.3 UdeM00000023466 XP_045190075.1 54.3 300 134 1 1 300 1 297 1.30E-99 371.3 6596 cellular organisms;Eukaryota;Opisthokonta;Metazoa;Eumetazoa;Bilateria;Protostomia;Spiralia;Lophotrochozoa;Mollusca;Bivalvia;Autobranchia;Heteroconchia;Euheterodonta;Imparidentia;Venerida;Veneroidea;Veneridae;Mercenaria;Mercenaria mercenaria
UdeM00000023467 6596 205.7 UdeM00000023467 XP_045190219.1 62.3 162 57 2 1 158 485 646 4.40E-50 205.7 6596 cellular organisms;Eukaryota;Opisthokonta;Metazoa;Eumetazoa;Bilateria;Protostomia;Spiralia;Lophotrochozoa;Mollusca;Bivalvia;Autobranchia;Heteroconchia;Euheterodonta;Imparidentia;Venerida;Veneroidea;Veneridae;Mercenaria;Mercenaria mercenaria

UdeM00000023468 6596 399.1 UdeM00000023468 XP_045190220.1 63.7 322
109 2 75 388 41 362 6.70E-108 399.1 6596 cellular organisms;Eukaryota;Opisthokonta;Metazoa;Eumetazoa;Bilateria;Protostomia;Spiralia;Lophotrochozoa;Mollusca;Bivalvia;Autobranchia;Heteroconchia;Euheterodonta;Imparidentia;Venerida;Veneroidea;Veneridae;Mercenaria;Mercenaria mercenaria
UdeM00000023469 6596 384 UdeM00000023469 XP_045190220.1 66 297
93 2 75 363 41 337 2.20E-103 384 6596 cellular organisms;Eukaryota;Opisthokonta;Metazoa;Eumetazoa;Bilateria;Protostomia;Spiralia;Lophotrochozoa;Mollusca;Bivalvia;Autobranchia;Heteroconchia;Euheterodonta;Imparidentia;Venerida;Veneroidea;Veneridae;Mercenaria;Mercenaria mercenaria
UdeM00000023470 6596 230.3 UdeM00000023470 XP_045188559.1 31.2 504
306 9 1 484 1 483 5.40E-57 230.3 6596 cellular organisms;Eukaryota;Opisthokonta;Metazoa;Eumetazoa;Bilateria;Protostomia;Spiralia;Lophotrochozoa;Mollusca;Bivalvia;Autobranchia;Heteroconchia;Euheterodonta;Imparidentia;Venerida;Veneroidea;Veneridae;Mercenaria;Mercenaria mercenaria
UdeM00000023472 6596 276.9 UdeM00000023472 XP_045189058.1 29.4 718
411 18 1 694 1 646 7.00E-71 276.9 6596 cellular organisms;Eukaryota;Opisthokonta;Metazoa;Eumetazoa;Bilateria;Protostomia;Spiralia;Lophotrochozoa;Mollusca;Bivalvia;Autobranchia;Heteroconchia;Euheterodonta;Imparidentia;Venerida;Veneroidea;Veneridae;Mercenaria;Mercenaria mercenaria
UdeM00000023473 6596 272.7 UdeM00000023473 XP_045189058.1 24.4 1215
613 36 1 1146 1 978 2.10E-69 272.7 6596 cellular organisms;Eukaryota;Opisthokonta;Metazoa;Eumetazoa;Bilateria;Protostomia;Spiralia;Lophotrochozoa;Mollusca;Bivalvia;Autobranchia;Heteroconchia;Euheterodonta;Imparidentia;Venerida;Veneroidea;Veneridae;Mercenaria;Mercenaria mercenaria
UdeM00000023474 6596 388.7 UdeM00000023474 XP_045189058.1 26.8 1271
631 35 1 1202 35 1075 2.80E-104 388.7 6596 cellular organisms;Eukaryota;Opisthokonta;Metazoa;Eumetazoa;Bilateria;Protostomia;Spiralia;Lophotrochozoa;Mollusca;Bivalvia;Autobranchia;Heteroconchia;Euheterodonta;Imparidentia;Venerida;Veneroidea;Veneridae;Mercenaria;Mercenaria mercenaria
UdeM00000023475 6579 521.5 UdeM00000023475 XP_033752369.1 38.2 1014
497 27 3 926 29 1002 2.10E-144 521.5 6579 cellular organisms;Eukaryota;Opisthokonta;Metazoa;Eumetazoa;Bilateria;Protostomia;Spiralia;Lophotrochozoa;Mollusca;Bivalvia;Autobranchia;Pteriomorphia;Pectinida;Pectinoidea;Pectinidae;Pecten;Pecten maximus
UdeM00000023476 29159 242.3 UdeM00000023476 XP_034329372.1 24.5 1079
648 25 89 1086 7 999 3.80E-60 242.3 29159 cellular organisms;Eukaryota;Opisthokonta;Metazoa;Eumetazoa;Bilateria;Protostomia;Spiralia;Lophotrochozoa;Mollusca;Bivalvia;Autobranchia;Pteriomorphia;Ostreida;Ostreoidea;Ostreidae;Crassostrea;Crassostrea gigas
UdeM00000023481 6596 239.2 UdeM00000023481 XP_045185723.1 43.1 304
155 5 7 308 455 742 1.00E-59 239.2 6596 cellular organisms;Eukaryota;Opisthokonta;Metazoa;Eumetazoa;Bilateria;Protostomia;Spiralia;Lophotrochozoa;Mollusca;Bivalvia;Autobranchia;Heteroconchia;Euheterodonta;Imparidentia;Venerida;Veneroidea;Veneridae;Mercenaria;Mercenaria mercenaria
UdeM00000023480 6596 458 UdeM00000023480 XP_045188223.1 48.9 454
228 2 408 861 216 665 2.70E-125 458 6596 cellular

organisms;Eukaryota;Opisthokonta;Metazoa;Eumetazoa;Bilateria;Protostomia;Spiralia;Lophotrochozoa;Mollusca;Bivalvia;Autobranchia;Heteroconchia;Euheterodonta;Imparidentia;Venerida;Veneroidea;Veneridae;Mercenaria;Mercenaria mercenaria
UdeM00000023482 6596 123.2 UdeM00000023482 XP_045206982.1 52.9 104 49 0 4 107 80 183 2.20E-25 123.2 6596 cellular organisms;Eukaryota;Opisthokonta;Metazoa;Eumetazoa;Bilateria;Protostomia;Spiralia;Lophotrochozoa;Mollusca;Bivalvia;Autobranchia;Heteroconchia;Euheterodonta;Imparidentia;Venerida;Veneroidea;Veneridae;Mercenaria;Mercenaria mercenaria
UdeM00000023486 6596 239.2 UdeM00000023486 XP_045185723.1 43.1 304 155 5 7 308 455 742 1.00E-59 239.2 6596 cellular organisms;Eukaryota;Opisthokonta;Metazoa;Eumetazoa;Bilateria;Protostomia;Spiralia;Lophotrochozoa;Mollusca;Bivalvia;Autobranchia;Heteroconchia;Euheterodonta;Imparidentia;Venerida;Veneroidea;Veneridae;Mercenaria;Mercenaria mercenaria
UdeM00000023485 6596 458 UdeM00000023485 XP_045188223.1 48.9 454 228 2 408 861 216 665 2.70E-125 458 6596 cellular organisms;Eukaryota;Opisthokonta;Metazoa;Eumetazoa;Bilateria;Protostomia;Spiralia;Lophotrochozoa;Mollusca;Bivalvia;Autobranchia;Heteroconchia;Euheterodonta;Imparidentia;Venerida;Veneroidea;Veneridae;Mercenaria;Mercenaria mercenaria
UdeM00000023487 6596 123.2 UdeM00000023487 XP_045206982.1 52.9 104 49 0 4 107 80 183 2.20E-25 123.2 6596 cellular organisms;Eukaryota;Opisthokonta;Metazoa;Eumetazoa;Bilateria;Protostomia;Spiralia;Lophotrochozoa;Mollusca;Bivalvia;Autobranchia;Heteroconchia;Euheterodonta;Imparidentia;Venerida;Veneroidea;Veneridae;Mercenaria;Mercenaria mercenaria
UdeM00000023490 6565 224.2 UdeM00000023490 XP_022317300.1 33.4 401 247 5 6 392 14 408 3.00E-55 224.2 6565 cellular organisms;Eukaryota;Opisthokonta;Metazoa;Eumetazoa;Bilateria;Protostomia;Spiralia;Lophotrochozoa;Mollusca;Bivalvia;Autobranchia;Pteriomorphia;Ostreida;Ostreoidea;Ostreidae;Crassostrea;Crassostrea virginica
UdeM00000023492 6596 306.6 UdeM00000023492 XP_045190922.1 59.2 260 106 0 1 260 163 422 3.00E-80 306.6 6596 cellular organisms;Eukaryota;Opisthokonta;Metazoa;Eumetazoa;Bilateria;Protostomia;Spiralia;Lophotrochozoa;Mollusca;Bivalvia;Autobranchia;Heteroconchia;Euheterodonta;Imparidentia;Venerida;Veneroidea;Veneridae;Mercenaria;Mercenaria mercenaria
UdeM00000023493 29159 294.3 UdeM00000023493 XP_011431649.2 50.9 279 128 5 3 279 8 279 1.70E-76 294.3 29159 cellular organisms;Eukaryota;Opisthokonta;Metazoa;Eumetazoa;Bilateria;Protostomia;Spiralia;Lophotrochozoa;Mollusca;Bivalvia;Autobranchia;Pteriomorphia;Ostreida;Ostreoidea;Ostreidae;Crassostrea;Crassostrea gigas
UdeM00000023494 6579 287.7 UdeM00000023494 XP_033756999.1 51.1 272 126 4 10 279 12 278 1.60E-74 287.7 6579 cellular organisms;Eukaryota;Opisthokonta;Metazoa;Eumetazoa;Bilateria;Protostomia;Spiralia;Lophotrochozoa;Mollusca;Bivalvia;Autobranchia;Pteriomorphia;Pectinida;Pectinoidea;Pectinidae;Pecten;Pecten maximus
UdeM00000023495 6579 308.9 UdeM00000023495 XP_033756999.1 53.7 272 120 3 10 280 12 278 6.50E-81 308.9 6579 cellular organisms;Eukaryota;Opisthokonta;Metazoa;Eumetazoa;Bilateria;Protostomia;Spiralia;Lophotrochozoa;Mollusca;Bivalvia;Autobranchia;Pteriomorphia;Pectinida;Pectinoidea;Pectinidae;Pecten;Pecten maximus

UdeM00000023496 6596 545.8 UdeM00000023496 XP_045188477.1 62.9 456
155 7 26 474 27 475 5.40E-152 545.8 6596 cellular
organisms;Eukaryota;Opisthokonta;Metazoa;Eumetazoa;Bilateria;Protostomia;
Spiralia;Lophotrochozoa;Mollusca;Bivalvia;Autobranchia;Heteroconchia;Euhe
terodonta;Imparidentia;Venerida;Veneroidea;Veneridae;Mercenaria;Mercenari
a mercenaria
UdeM00000023498 6596 1019.6 UdeM00000023498 XP_045190725.1 33.8
3313 1627 63 634 3657 295 3329 9.80E-294 1019.6 6596
cellular
organisms;Eukaryota;Opisthokonta;Metazoa;Eumetazoa;Bilateria;Protostomia;
Spiralia;Lophotrochozoa;Mollusca;Bivalvia;Autobranchia;Heteroconchia;Euhe
terodonta;Imparidentia;Venerida;Veneroidea;Veneridae;Mercenaria;Mercenari
a mercenaria
UdeM00000023499 6596 573.9 UdeM00000023499 XP_045188783.1 82.1 319
57 0 1 319 1 319 1.20E-160 573.9 6596 cellular
organisms;Eukaryota;Opisthokonta;Metazoa;Eumetazoa;Bilateria;Protostomia;
Spiralia;Lophotrochozoa;Mollusca;Bivalvia;Autobranchia;Heteroconchia;Euhe
terodonta;Imparidentia;Venerida;Veneroidea;Veneridae;Mercenaria;Mercenari
a mercenaria
UdeM00000023500 6565 178.7 UdeM00000023500 XP_022289764.1 72.6 117
30 2 1 116 1 116 4.20E-42 178.7 6565 cellular
organisms;Eukaryota;Opisthokonta;Metazoa;Eumetazoa;Bilateria;Protostomia;
Spiralia;Lophotrochozoa;Mollusca;Bivalvia;Autobranchia;Pteriomorphia;Ostr
eida;Ostreoidea;Ostreidae;Crassostrea;Crassostrea virginica
UdeM00000023501 6565 176.8 UdeM00000023501 XP_022289764.1 72.4 116
30 2 1 115 2 116 1.60E-41 176.8 6565 cellular
organisms;Eukaryota;Opisthokonta;Metazoa;Eumetazoa;Bilateria;Protostomia;
Spiralia;Lophotrochozoa;Mollusca;Bivalvia;Autobranchia;Pteriomorphia;Ostr
eida;Ostreoidea;Ostreidae;Crassostrea;Crassostrea virginica
UdeM00000023502 6596 335.1 UdeM00000023502 XP_045190578.1 69.6 227
65 2 33 259 43 265 7.90E-89 335.1 6596 cellular
organisms;Eukaryota;Opisthokonta;Metazoa;Eumetazoa;Bilateria;Protostomia;
Spiralia;Lophotrochozoa;Mollusca;Bivalvia;Autobranchia;Heteroconchia;Euhe
terodonta;Imparidentia;Venerida;Veneroidea;Veneridae;Mercenaria;Mercenari
a mercenaria
UdeM00000023503 45264 353.6 UdeM00000023503 XP_044165143.1 55.9 313
135 3 20 329 161 473 2.80E-94 353.6 45264 cellular
organisms;Eukaryota;Opisthokonta;Metazoa;Eumetazoa;Cnidaria;Anthozoa;Hexa
corallia;Scleractinia;Astrocoeniina;Acroporidae;Acropora;Acropora
millepora
UdeM00000023505 45264 127.5 UdeM00000023505 XP_044164583.1 49.3 146
67 3 1 143 130 271 1.40E-26 127.5 45264 cellular
organisms;Eukaryota;Opisthokonta;Metazoa;Eumetazoa;Cnidaria;Anthozoa;Hexa
corallia;Scleractinia;Astrocoeniina;Acroporidae;Acropora;Acropora
millepora
UdeM00000023504 1529436 140.6 UdeM00000023504 XP_033095325.1 45.8
155 82 1 57 209 26 180 3.20E-30 140.6 1529436
cellular
organisms;Eukaryota;Opisthokonta;Metazoa;Eumetazoa;Bilateria;Deuterostomi
a;Echinodermata;Pelmatozoa;Crinoidea;Articulata;Comatulida;Comatulidae;Co
matulinae;Anneissia;Anneissia japonica
UdeM00000023506 1529436 173.7 UdeM00000023506 XP_033095325.1 49.7
171 84 1 57 225 26 196 2.60E-40 173.7 1529436
cellular

organisms;Eukaryota;Opisthokonta;Metazoa;Eumetazoa;Bilateria;Deuterostomia;Echinodermata;Pelmatozoa;Crinoidea;Articulata;Comatulida;Comatulidae;Comatulinae;Anneissia;Anneissia japonica
UdeM00000023507 1735272 250 UdeM00000023507 XP_041371067.1 32.5 511 334 7 1 506 2 506 6.50E-63 250 1735272 cellular
organisms;Eukaryota;Opisthokonta;Metazoa;Eumetazoa;Bilateria;Protostomia;Spiralia;Lophotrochozoa;Mollusca;Gastropoda;Neomphalina;Neomphaloidea;Peltospiridae;Gigantopelta;Gigantopelta aegis
UdeM00000023508 6596 193.4 UdeM00000023508 XP_045191266.1 84.3 121 15 2 160 280 198 314 4.10E-46 193.4 6596 cellular
organisms;Eukaryota;Opisthokonta;Metazoa;Eumetazoa;Bilateria;Protostomia;Spiralia;Lophotrochozoa;Mollusca;Bivalvia;Autobranchia;Heteroconchia;Euheterodonta;Imparidentia;Venerida;Veneroidea;Veneridae;Mercenaria;Mercenaria mercenaria
UdeM00000023509 6596 62 UdeM00000023509 XP_045191266.1 37.3 102 46 3 16 99 21 122 8.30E-07 62 6596 cellular
organisms;Eukaryota;Opisthokonta;Metazoa;Eumetazoa;Bilateria;Protostomia;Spiralia;Lophotrochozoa;Mollusca;Bivalvia;Autobranchia;Heteroconchia;Euheterodonta;Imparidentia;Venerida;Veneroidea;Veneridae;Mercenaria;Mercenaria mercenaria
UdeM00000023510 6596 165.2 UdeM00000023510 XP_045188238.1 62.3 162 48 4 136 295 166 316 1.20E-37 165.2 6596 cellular
organisms;Eukaryota;Opisthokonta;Metazoa;Eumetazoa;Bilateria;Protostomia;Spiralia;Lophotrochozoa;Mollusca;Bivalvia;Autobranchia;Heteroconchia;Euheterodonta;Imparidentia;Venerida;Veneroidea;Veneridae;Mercenaria;Mercenaria mercenaria
UdeM00000023511 6596 738 UdeM00000023511 XP_045188471.1 71.7 526 134 5 1 525 3036 3547 8.80E-210 738 6596 cellular
organisms;Eukaryota;Opisthokonta;Metazoa;Eumetazoa;Bilateria;Protostomia;Spiralia;Lophotrochozoa;Mollusca;Bivalvia;Autobranchia;Heteroconchia;Euheterodonta;Imparidentia;Venerida;Veneroidea;Veneridae;Mercenaria;Mercenaria mercenaria
UdeM00000023512 6596 1843.6 UdeM00000023512 XP_045188471.1 63.8 1569 533 15 2 1551 1485 3037 0.00E+00 1843.6 6596 cellular
organisms;Eukaryota;Opisthokonta;Metazoa;Eumetazoa;Bilateria;Protostomia;Spiralia;Lophotrochozoa;Mollusca;Bivalvia;Autobranchia;Heteroconchia;Euheterodonta;Imparidentia;Venerida;Veneroidea;Veneridae;Mercenaria;Mercenaria mercenaria
UdeM00000023513 6596 443 UdeM00000023513 XP_045188471.1 49.1 584 244 16 1 570 942 1486 6.10E-121 443 6596 cellular
organisms;Eukaryota;Opisthokonta;Metazoa;Eumetazoa;Bilateria;Protostomia;Spiralia;Lophotrochozoa;Mollusca;Bivalvia;Autobranchia;Heteroconchia;Euheterodonta;Imparidentia;Venerida;Veneroidea;Veneridae;Mercenaria;Mercenaria mercenaria
UdeM00000023514 6596 297.4 UdeM00000023514 XP_045188471.1 39.8 565 289 14 1 547 1 532 3.80E-77 297.4 6596 cellular
organisms;Eukaryota;Opisthokonta;Metazoa;Eumetazoa;Bilateria;Protostomia;Spiralia;Lophotrochozoa;Mollusca;Bivalvia;Autobranchia;Heteroconchia;Euheterodonta;Imparidentia;Venerida;Veneroidea;Veneridae;Mercenaria;Mercenaria mercenaria
UdeM00000023515 6596 263.1 UdeM00000023515 XP_045183440.1 54.2 240 105 3 2 240 145 380 4.30E-67 263.1 6596 cellular

organisms;Eukaryota;Opisthokonta;Metazoa;Eumetazoa;Bilateria;Protostomia;
Spiralia;Lophotrochozoa;Mollusca;Bivalvia;Autobranchia;Heteroconchia;Euheterodonta;Imparidentia;Venerida;Veneroidea;Veneridae;Mercenaria;Mercenaria mercenaria
UdeM00000023516 6596 263.1 UdeM00000023516 XP_045183440.1 54.2 240
105 3 2 240 145 380 4.30E-67 263.1 6596 cellular
organisms;Eukaryota;Opisthokonta;Metazoa;Eumetazoa;Bilateria;Protostomia;
Spiralia;Lophotrochozoa;Mollusca;Bivalvia;Autobranchia;Heteroconchia;Euheterodonta;Imparidentia;Venerida;Veneroidea;Veneridae;Mercenaria;Mercenaria mercenaria
UdeM00000023517 6596 69.7 UdeM00000023517 XP_045202346.1 37.4 91
53 2 25 114 35 122 3.10E-09 69.7 6596 cellular
organisms;Eukaryota;Opisthokonta;Metazoa;Eumetazoa;Bilateria;Protostomia;
Spiralia;Lophotrochozoa;Mollusca;Bivalvia;Autobranchia;Heteroconchia;Euheterodonta;Imparidentia;Venerida;Veneroidea;Veneridae;Mercenaria;Mercenaria mercenaria
UdeM00000023519 6596 599 UdeM00000023519 XP_045191253.1 61.1 475
175 3 1 472 37 504 5.40E-168 599 6596 cellular
organisms;Eukaryota;Opisthokonta;Metazoa;Eumetazoa;Bilateria;Protostomia;
Spiralia;Lophotrochozoa;Mollusca;Bivalvia;Autobranchia;Heteroconchia;Euheterodonta;Imparidentia;Venerida;Veneroidea;Veneridae;Mercenaria;Mercenaria mercenaria
UdeM00000023518 6596 645.2 UdeM00000023518 XP_045191253.1 61.7 506
184 3 3 505 6 504 7.00E-182 645.2 6596 cellular
organisms;Eukaryota;Opisthokonta;Metazoa;Eumetazoa;Bilateria;Protostomia;
Spiralia;Lophotrochozoa;Mollusca;Bivalvia;Autobranchia;Heteroconchia;Euheterodonta;Imparidentia;Venerida;Veneroidea;Veneridae;Mercenaria;Mercenaria mercenaria
UdeM00000023520 6596 72 UdeM00000023520 XP_045191040.1 38.5 91
56 0 175 265 238 328 1.30E-09 72 6596 cellular
organisms;Eukaryota;Opisthokonta;Metazoa;Eumetazoa;Bilateria;Protostomia;
Spiralia;Lophotrochozoa;Mollusca;Bivalvia;Autobranchia;Heteroconchia;Euheterodonta;Imparidentia;Venerida;Veneroidea;Veneridae;Mercenaria;Mercenaria mercenaria
UdeM00000023521 6596 75.5 UdeM00000023521 XP_045191040.1 37.4 91
57 0 169 259 238 328 1.10E-10 75.5 6596 cellular
organisms;Eukaryota;Opisthokonta;Metazoa;Eumetazoa;Bilateria;Protostomia;
Spiralia;Lophotrochozoa;Mollusca;Bivalvia;Autobranchia;Heteroconchia;Euheterodonta;Imparidentia;Venerida;Veneroidea;Veneridae;Mercenaria;Mercenaria mercenaria
UdeM00000023522 6596 73.9 UdeM00000023522 XP_045191040.1 37.5 88
55 0 171 258 241 328 3.30E-10 73.9 6596 cellular
organisms;Eukaryota;Opisthokonta;Metazoa;Eumetazoa;Bilateria;Protostomia;
Spiralia;Lophotrochozoa;Mollusca;Bivalvia;Autobranchia;Heteroconchia;Euheterodonta;Imparidentia;Venerida;Veneroidea;Veneridae;Mercenaria;Mercenaria mercenaria
UdeM00000023523 6596 74.3 UdeM00000023523 XP_045191040.1 33.6 146
88 3 113 254 185 325 2.50E-10 74.3 6596 cellular
organisms;Eukaryota;Opisthokonta;Metazoa;Eumetazoa;Bilateria;Protostomia;
Spiralia;Lophotrochozoa;Mollusca;Bivalvia;Autobranchia;Heteroconchia;Euheterodonta;Imparidentia;Venerida;Veneroidea;Veneridae;Mercenaria;Mercenaria mercenaria
UdeM00000023524 7741 193 UdeM00000023524 XP_019622111.1 52.2 180
84 2 17 196 101 278 3.70E-46 193 7741 cellular

organisms;Eukaryota;Opisthokonta;Metazoa;Eumetazoa;Bilateria;Deuterostomia;Chordata;Cephalochordata;Leptocardii;Amphioxiformes;Branchiostomidae;Branchiostoma;Branchiostoma belcheri
UdeM00000023525 6596 421.4 UdeM00000023525 XP_045197972.1 59.8 321 127 2 6 325 3 322 1.00E-114 421.4 6596 cellular organisms;Eukaryota;Opisthokonta;Metazoa;Eumetazoa;Bilateria;Protostomia;Spiralia;Lophotrochozoa;Mollusca;Bivalvia;Autobranchia;Heteroconchia;Euheterodonta;Imparidentia;Venerida;Veneroidea;Veneridae;Mercenaria;Mercenaria mercenaria
UdeM00000023526 6596 166.4 UdeM00000023526 XP_045176664.1 46.9 177 88 4 13 185 393 567 3.50E-38 166.4 6596 cellular organisms;Eukaryota;Opisthokonta;Metazoa;Eumetazoa;Bilateria;Protostomia;Spiralia;Lophotrochozoa;Mollusca;Bivalvia;Autobranchia;Heteroconchia;Euheterodonta;Imparidentia;Venerida;Veneroidea;Veneridae;Mercenaria;Mercenaria mercenaria
UdeM00000023527 114398 304.7 UdeM00000023527 XP_015906392.2 39.1 422 250 4 1 420 1 417 2.00E-79 304.7 114398 cellular organisms;Eukaryota;Opisthokonta;Metazoa;Eumetazoa;Bilateria;Protostomia;Ecdysozoa;Panarthropoda;Arthropoda;Chelicerata;Arachnida;Araneae;Araneomorphae;Entelegynae;Orbiculariae;Araneoidea;Theridiidae;Parasteatoda;Parasteatoda tepidariorum
UdeM00000023528 1735272 132.1 UdeM00000023528 XP_041373570.1 52.1 121 58 0 6 126 5 125 5.10E-28 132.1 1735272 cellular organisms;Eukaryota;Opisthokonta;Metazoa;Eumetazoa;Bilateria;Protostomia;Spiralia;Lophotrochozoa;Mollusca;Gastropoda;Neomphalina;Neomphaloidea;Peltospiridae;Gigantopelta;Gigantopelta aegis
UdeM00000023529 225164 1060.1 UdeM00000023529 XP_009046752.1 68.8 794 248 0 8 801 17 810 1.50E-306 1060.1 225164 cellular organisms;Eukaryota;Opisthokonta;Metazoa;Eumetazoa;Bilateria;Protostomia;Spiralia;Lophotrochozoa;Mollusca;Gastropoda;Patellogastropoda;Lottioidea;Lottiidae;Lottia;Lottia gigantea
UdeM00000023530 6596 1865.1 UdeM00000023530 XP_045170889.1 48.7 1911 939 21 340 2228 77 1968 0.00E+00 1865.1 6596 cellular organisms;Eukaryota;Opisthokonta;Metazoa;Eumetazoa;Bilateria;Protostomia;Spiralia;Lophotrochozoa;Mollusca;Bivalvia;Autobranchia;Heteroconchia;Euheterodonta;Imparidentia;Venerida;Veneroidea;Veneridae;Mercenaria;Mercenaria mercenaria
UdeM00000023531 6573 745.3 UdeM00000023531 XP_021358192.1 47.7 841 407 17 1 825 1977 2800 8.10E-212 745.3 6573 cellular organisms;Eukaryota;Opisthokonta;Metazoa;Eumetazoa;Bilateria;Protostomia;Spiralia;Lophotrochozoa;Mollusca;Bivalvia;Autobranchia;Pteriomorphia;Pectinida;Pectinoidea;Pectinidae;Mizuhopecten;Mizuhopecten yessoensis
UdeM00000023532 6573 792.3 UdeM00000023532 XP_021358192.1 47.8 901 441 15 7 893 1918 2803 6.20E-226 792.3 6573 cellular organisms;Eukaryota;Opisthokonta;Metazoa;Eumetazoa;Bilateria;Protostomia;Spiralia;Lophotrochozoa;Mollusca;Bivalvia;Autobranchia;Pteriomorphia;Pectinida;Pectinoidea;Pectinidae;Mizuhopecten;Mizuhopecten yessoensis
UdeM00000023533 6596 2074.7 UdeM00000023533 XP_045170890.1 50.6 2055 930 19 1 2025 1 1999 0.00E+00 2074.7 6596 cellular

organisms;Eukaryota;Opisthokonta;Metazoa;Eumetazoa;Bilateria;Protostomia;Spiralia;Lophotrochozoa;Mollusca;Bivalvia;Autobranchia;Heteroconchia;Euheterodonta;Imparidentia;Venerida;Veneroidea;Veneridae;Mercenaria;Mercenaria mercenaria
UdeM00000023534 6596 1089.7 UdeM00000023534 XP_045170890.1 61.8 828 314 1 5 832 1999 2824 0.00E+00 1089.7 6596 cellular organisms;Eukaryota;Opisthokonta;Metazoa;Eumetazoa;Bilateria;Protostomia;Spiralia;Lophotrochozoa;Mollusca;Bivalvia;Autobranchia;Heteroconchia;Euheterodonta;Imparidentia;Venerida;Veneroidea;Veneridae;Mercenaria;Mercenaria mercenaria
UdeM00000023536 6596 312.8 UdeM00000023536 XP_045174044.1 37.9 485 271 11 11 477 7 479 7.90E-82 312.8 6596 cellular organisms;Eukaryota;Opisthokonta;Metazoa;Eumetazoa;Bilateria;Protostomia;Spiralia;Lophotrochozoa;Mollusca;Bivalvia;Autobranchia;Heteroconchia;Euheterodonta;Imparidentia;Venerida;Veneroidea;Veneridae;Mercenaria;Mercenaria mercenaria
UdeM00000023535 6596 961.8 UdeM00000023535 XP_045174045.1 52.7 1011 429 21 11 988 7 1001 6.50E-277 961.8 6596 cellular organisms;Eukaryota;Opisthokonta;Metazoa;Eumetazoa;Bilateria;Protostomia;Spiralia;Lophotrochozoa;Mollusca;Bivalvia;Autobranchia;Heteroconchia;Euheterodonta;Imparidentia;Venerida;Veneroidea;Veneridae;Mercenaria;Mercenaria mercenaria
UdeM00000023537 6579 89.7 UdeM00000023537 XP_033732752.1 39.2 148 74 6 220 362 216 352 8.30E-15 89.7 6579 cellular organisms;Eukaryota;Opisthokonta;Metazoa;Eumetazoa;Bilateria;Protostomia;Spiralia;Lophotrochozoa;Mollusca;Bivalvia;Autobranchia;Pteriomorphia;Pectinida;Pectinoidea;Pectinidae;Pecten;Pecten maximus
UdeM00000023538 6596 90.9 UdeM00000023538 XP_045168532.1 32.2 177 105 3 234 410 46 207 4.20E-15 90.9 6596 cellular organisms;Eukaryota;Opisthokonta;Metazoa;Eumetazoa;Bilateria;Protostomia;Spiralia;Lophotrochozoa;Mollusca;Bivalvia;Autobranchia;Heteroconchia;Euheterodonta;Imparidentia;Venerida;Veneroidea;Veneridae;Mercenaria;Mercenaria mercenaria
UdeM00000023539 6573 83.2 UdeM00000023539 XP_021348414.1 31 203 116 6 8 207 23 204 4.50E-13 83.2 6573 cellular organisms;Eukaryota;Opisthokonta;Metazoa;Eumetazoa;Bilateria;Protostomia;Spiralia;Lophotrochozoa;Mollusca;Bivalvia;Autobranchia;Pteriomorphia;Pectinida;Pectinoidea;Pectinidae;Mizuhopecten;Mizuhopecten yessoensis
UdeM00000023540 6579 81.3 UdeM00000023540 XP_033732751.1 37 146 79 6 9 149 226 363 1.30E-12 81.3 6579 cellular organisms;Eukaryota;Opisthokonta;Metazoa;Eumetazoa;Bilateria;Protostomia;Spiralia;Lophotrochozoa;Mollusca;Bivalvia;Autobranchia;Pteriomorphia;Pectinida;Pectinoidea;Pectinidae;Pecten;Pecten maximus
UdeM00000023545 6579 144.1 UdeM00000023545 XP_033748699.1 51.4 177 69 5 14 181 17 185 2.20E-31 144.1 6579 cellular organisms;Eukaryota;Opisthokonta;Metazoa;Eumetazoa;Bilateria;Protostomia;Spiralia;Lophotrochozoa;Mollusca;Bivalvia;Autobranchia;Pteriomorphia;Pectinida;Pectinoidea;Pectinidae;Pecten;Pecten maximus
UdeM00000023544 6596 299.3 UdeM00000023544 XP_045171445.1 83.4 181 29 1 1 180 1 181 4.10E-78 299.3 6596 cellular organisms;Eukaryota;Opisthokonta;Metazoa;Eumetazoa;Bilateria;Protostomia;Spiralia;Lophotrochozoa;Mollusca;Bivalvia;Autobranchia;Heteroconchia;Euhe

terodonta;Imparidentia;Venerida;Veneroidea;Veneridae;Mercenaria;Mercenari a mercenaria
UdeM00000023546 1735272 572.4 UdeM00000023546 XP_041348257.1 79.2 336 67 1 10 342 4 339 3.90E-160 572.4 1735272 cellular organisms;Eukaryota;Opisthokonta;Metazoa;Eumetazoa;Bilateria;Protostomia;Spiralia;Lophotrochozoa;Mollusca;Gastropoda;Neomphalina;Neomphaloidea;Peltospiridae;Gigantopelta;Gigantopelta aegis
UdeM00000023547 400727 490 UdeM00000023547 XP_025091190.1 82.9 269 46 0 1 269 68 336 2.00E-135 490 400727 cellular organisms;Eukaryota;Opisthokonta;Metazoa;Eumetazoa;Bilateria;Protostomia;Spiralia;Lophotrochozoa;Mollusca;Gastropoda;Caenogastropoda;Architaenioglossa;Ampullarioidea;Ampullariidae;Pomacea;Pomacea canaliculata
UdeM00000023548 6596 492.7 UdeM00000023548 XP_045170916.1 70.8 332 87 4 34 360 51 377 5.60E-136 492.7 6596 cellular organisms;Eukaryota;Opisthokonta;Metazoa;Eumetazoa;Bilateria;Protostomia;Spiralia;Lophotrochozoa;Mollusca;Bivalvia;Autobranchia;Heteroconchia;Euheterodonta;Imparidentia;Venerida;Veneroidea;Veneridae;Mercenaria;Mercenaria mercenaria
UdeM00000023549 6596 978.8 UdeM00000023549 XP_045171420.1 54.3 899 332 13 118 982 132 985 5.30E-282 978.8 6596 cellular organisms;Eukaryota;Opisthokonta;Metazoa;Eumetazoa;Bilateria;Protostomia;Spiralia;Lophotrochozoa;Mollusca;Bivalvia;Autobranchia;Heteroconchia;Euheterodonta;Imparidentia;Venerida;Veneroidea;Veneridae;Mercenaria;Mercenaria mercenaria
UdeM00000023550 6596 193.7 UdeM00000023550 XP_045177053.1 42.2 244 129 6 114 350 193 431 3.90E-46 193.7 6596 cellular organisms;Eukaryota;Opisthokonta;Metazoa;Eumetazoa;Bilateria;Protostomia;Spiralia;Lophotrochozoa;Mollusca;Bivalvia;Autobranchia;Heteroconchia;Euheterodonta;Imparidentia;Venerida;Veneroidea;Veneridae;Mercenaria;Mercenaria mercenaria
UdeM00000023551 29159 152.1 UdeM00000023551 XP_034310219.1 32.1 352 208 13 459 803 268 595 3.00E-33 152.1 29159 cellular organisms;Eukaryota;Opisthokonta;Metazoa;Eumetazoa;Bilateria;Protostomia;Spiralia;Lophotrochozoa;Mollusca;Bivalvia;Autobranchia;Pteriomorphia;Ostreida;Ostreoidea;Ostreidae;Crassostrea;Crassostrea gigas
UdeM00000023552 29159 220.7 UdeM00000023552 XP_034337829.1 89.4 132 14 0 1 132 1 132 1.10E-54 220.7 29159 cellular organisms;Eukaryota;Opisthokonta;Metazoa;Eumetazoa;Bilateria;Protostomia;Spiralia;Lophotrochozoa;Mollusca;Bivalvia;Autobranchia;Pteriomorphia;Ostreida;Ostreoidea;Ostreidae;Crassostrea;Crassostrea gigas
UdeM00000023553 6596 478.8 UdeM00000023553 XP_045170652.1 80.4 291 55 1 1 291 1 289 5.00E-132 478.8 6596 cellular organisms;Eukaryota;Opisthokonta;Metazoa;Eumetazoa;Bilateria;Protostomia;Spiralia;Lophotrochozoa;Mollusca;Bivalvia;Autobranchia;Heteroconchia;Euheterodonta;Imparidentia;Venerida;Veneroidea;Veneridae;Mercenaria;Mercenaria mercenaria
UdeM00000023554 6596 395.6 UdeM00000023554 XP_045197399.1 59.9 314 126 0 1 314 1 314 5.90E-107 395.6 6596 cellular organisms;Eukaryota;Opisthokonta;Metazoa;Eumetazoa;Bilateria;Protostomia;Spiralia;Lophotrochozoa;Mollusca;Bivalvia;Autobranchia;Heteroconchia;Euheterodonta;Imparidentia;Venerida;Veneroidea;Veneridae;Mercenaria;Mercenaria mercenaria

UdeM00000023556	1735272	647.9	UdeM00000023556	XP_041348893.1	74.1
397	103	0	1	397	1	397	1.10E-182	647.9	1735272
cellular
organisms;Eukaryota;Opisthokonta;Metazoa;Eumetazoa;Bilateria;Protostomia;
Spiralia;Lophotrochozoa;Mollusca;Gastropoda;Neomphalina;Neomphaloidea;Pel
tospiridae;Gigantopelta;Gigantopelta aegis
UdeM00000023557	6596	154.1	UdeM00000023557	XP_045172863.1	63.7	102
37	0	5	106	4	105	1.00E-34	154.1	6596	cellular
organisms;Eukaryota;Opisthokonta;Metazoa;Eumetazoa;Bilateria;Protostomia;
Spiralia;Lophotrochozoa;Mollusca;Bivalvia;Autobranchia;Heteroconchia;Euhe
terodonta;Imparidentia;Venerida;Veneroidea;Veneridae;Mercenaria;Mercenari
a mercenaria
UdeM00000023558	29159	201.8	UdeM00000023558	XP_011444790.1	72.3	130
34	1	57	184	76	205	7.50E-49	201.8	29159	cellular
organisms;Eukaryota;Opisthokonta;Metazoa;Eumetazoa;Bilateria;Protostomia;
Spiralia;Lophotrochozoa;Mollusca;Bivalvia;Autobranchia;Pteriomorphia;Ostr
eida;Ostreoidea;Ostreidae;Crassostrea;Crassostrea gigas
UdeM00000023559	1735272	273.1	UdeM00000023559	XP_041367923.1	56.3
229	98	1	1	229	46	272	5.70E-70	273.1	1735272
cellular
organisms;Eukaryota;Opisthokonta;Metazoa;Eumetazoa;Bilateria;Protostomia;
Spiralia;Lophotrochozoa;Mollusca;Gastropoda;Neomphalina;Neomphaloidea;Pel
tospiridae;Gigantopelta;Gigantopelta aegis
UdeM00000023560	29159	74.3	UdeM00000023560	XP_019926413.2	42	69
40	0	1	69	153	221	7.60E-11	74.3	29159	cellular
organisms;Eukaryota;Opisthokonta;Metazoa;Eumetazoa;Bilateria;Protostomia;
Spiralia;Lophotrochozoa;Mollusca;Bivalvia;Autobranchia;Pteriomorphia;Ostr
eida;Ostreoidea;Ostreidae;Crassostrea;Crassostrea gigas
UdeM00000023562	6596	150.6	UdeM00000023562	XP_045172863.1	61.8	102
39	0	5	106	4	105	1.10E-33	150.6	6596	cellular
organisms;Eukaryota;Opisthokonta;Metazoa;Eumetazoa;Bilateria;Protostomia;
Spiralia;Lophotrochozoa;Mollusca;Bivalvia;Autobranchia;Heteroconchia;Euhe
terodonta;Imparidentia;Venerida;Veneroidea;Veneridae;Mercenaria;Mercenari
a mercenaria
UdeM00000023563	6573	365.5	UdeM00000023563	XP_021346288.1	62.2	288
85	2	1	288	1	264	6.10E-98	365.5	6573	cellular
organisms;Eukaryota;Opisthokonta;Metazoa;Eumetazoa;Bilateria;Protostomia;
Spiralia;Lophotrochozoa;Mollusca;Bivalvia;Autobranchia;Pteriomorphia;Pect
inida;Pectinoidea;Pectinidae;Mizuhopecten;Mizuhopecten yessoensis
UdeM00000023564	6579	396	UdeM00000023564	XP_033747923.1	56.4	362
154	2	1	362	1	358	5.50E-107	396	6579	cellular
organisms;Eukaryota;Opisthokonta;Metazoa;Eumetazoa;Bilateria;Protostomia;
Spiralia;Lophotrochozoa;Mollusca;Bivalvia;Autobranchia;Pteriomorphia;Pect
inida;Pectinoidea;Pectinidae;Pecten;Pecten maximus
UdeM00000023565	1735272	148.7	UdeM00000023565	XP_041350324.1	46.3
175	79	3	1	167	16	183	7.80E-33	148.7	1735272
cellular
organisms;Eukaryota;Opisthokonta;Metazoa;Eumetazoa;Bilateria;Protostomia;
Spiralia;Lophotrochozoa;Mollusca;Gastropoda;Neomphalina;Neomphaloidea;Pel
tospiridae;Gigantopelta;Gigantopelta aegis
UdeM00000023566	1735272	167.9	UdeM00000023566	XP_041350324.1	47.9
190	84	3	1	182	1	183	1.30E-38	167.9	1735272
cellular
organisms;Eukaryota;Opisthokonta;Metazoa;Eumetazoa;Bilateria;Protostomia;

Spiralia;Lophotrochozoa;Mollusca;Gastropoda;Neomphalina;Neomphaloidea;Peltospiridae;Gigantopelta;Gigantopelta aegis
UdeM00000023567 6596 275.8 UdeM00000023567 XP_045170696.1 39.1 603 255 20 1 566 1 528 1.30E-70 275.8 6596 cellular organisms;Eukaryota;Opisthokonta;Metazoa;Eumetazoa;Bilateria;Protostomia;Spiralia;Lophotrochozoa;Mollusca;Bivalvia;Autobranchia;Heteroconchia;Euheterodonta;Imparidentia;Venerida;Veneroidea;Veneridae;Mercenaria;Mercenaria mercenaria
UdeM00000023569 29159 199.5 UdeM00000023569 XP_034338217.1 72.3 130 35 1 1 130 595 723 2.60E-48 199.5 29159 cellular organisms;Eukaryota;Opisthokonta;Metazoa;Eumetazoa;Bilateria;Protostomia;Spiralia;Lophotrochozoa;Mollusca;Bivalvia;Autobranchia;Pteriomorphia;Ostreida;Ostreoidea;Ostreidae;Crassostrea;Crassostrea gigas
UdeM00000023568 6596 1100.9 UdeM00000023568 XP_045172714.1 72.2 712 195 2 19 728 16 726 0.00E+00 1100.9 6596 cellular organisms;Eukaryota;Opisthokonta;Metazoa;Eumetazoa;Bilateria;Protostomia;Spiralia;Lophotrochozoa;Mollusca;Bivalvia;Autobranchia;Heteroconchia;Euheterodonta;Imparidentia;Venerida;Veneroidea;Veneridae;Mercenaria;Mercenaria mercenaria
UdeM00000023570 6565 300.8 UdeM00000023570 XP_022340977.1 83.8 167 27 0 1 167 1 167 1.10E-78 300.8 6565 cellular organisms;Eukaryota;Opisthokonta;Metazoa;Eumetazoa;Bilateria;Protostomia;Spiralia;Lophotrochozoa;Mollusca;Bivalvia;Autobranchia;Pteriomorphia;Ostreida;Ostreoidea;Ostreidae;Crassostrea;Crassostrea virginica
UdeM00000023571 29159 265.8 UdeM00000023571 XP_034324494.1 45.8 321 167 4 12 328 252 569 7.60E-68 265.8 29159 cellular organisms;Eukaryota;Opisthokonta;Metazoa;Eumetazoa;Bilateria;Protostomia;Spiralia;Lophotrochozoa;Mollusca;Bivalvia;Autobranchia;Pteriomorphia;Ostreida;Ostreoidea;Ostreidae;Crassostrea;Crassostrea gigas
UdeM00000023572 29159 218 UdeM00000023572 XP_034324494.1 54.6 207 93 1 12 217 252 458 1.60E-53 218 29159 cellular organisms;Eukaryota;Opisthokonta;Metazoa;Eumetazoa;Bilateria;Protostomia;Spiralia;Lophotrochozoa;Mollusca;Bivalvia;Autobranchia;Pteriomorphia;Ostreida;Ostreoidea;Ostreidae;Crassostrea;Crassostrea gigas
UdeM00000023573 6573 283.9 UdeM00000023573 XP_021360672.1 37.1 396 229 7 12 388 13 407 3.10E-73 283.9 6573 cellular organisms;Eukaryota;Opisthokonta;Metazoa;Eumetazoa;Bilateria;Protostomia;Spiralia;Lophotrochozoa;Mollusca;Bivalvia;Autobranchia;Pteriomorphia;Pectinida;Pectinoidea;Pectinidae;Mizuhopecten;Mizuhopecten yessoensis
UdeM00000023574 6596 551.6 UdeM00000023574 XP_045172568.1 70.8 356 103 1 1 356 1 355 7.50E-154 551.6 6596 cellular organisms;Eukaryota;Opisthokonta;Metazoa;Eumetazoa;Bilateria;Protostomia;Spiralia;Lophotrochozoa;Mollusca;Bivalvia;Autobranchia;Heteroconchia;Euheterodonta;Imparidentia;Venerida;Veneroidea;Veneridae;Mercenaria;Mercenaria mercenaria
UdeM00000023575 6596 485 UdeM00000023575 XP_045172568.1 62.1 356 134 1 3 358 1 355 8.70E-134 485 6596 cellular organisms;Eukaryota;Opisthokonta;Metazoa;Eumetazoa;Bilateria;Protostomia;Spiralia;Lophotrochozoa;Mollusca;Bivalvia;Autobranchia;Heteroconchia;Euheterodonta;Imparidentia;Venerida;Veneroidea;Veneridae;Mercenaria;Mercenaria mercenaria
UdeM00000023576 6596 441.8 UdeM00000023576 XP_045172568.1 68.9 289 90 0 1 289 67 355 6.80E-121 441.8 6596 cellular

organisms;Eukaryota;Opisthokonta;Metazoa;Eumetazoa;Bilateria;Protostomia;Spiralia;Lophotrochozoa;Mollusca;Bivalvia;Autobranchia;Heteroconchia;Euheterodonta;Imparidentia;Venerida;Veneroidea;Veneridae;Mercenaria;Mercenaria mercenaria
UdeM00000023577 6596 672.5 UdeM00000023577 XP_045172811.1 64.4 562 176 8 80 620 127 685 5.10E-190 672.5 6596 cellular organisms;Eukaryota;Opisthokonta;Metazoa;Eumetazoa;Bilateria;Protostomia;Spiralia;Lophotrochozoa;Mollusca;Bivalvia;Autobranchia;Heteroconchia;Euheterodonta;Imparidentia;Venerida;Veneroidea;Veneridae;Mercenaria;Mercenaria mercenaria
UdeM00000023578 6573 1008.4 UdeM00000023578 XP_021375644.1 56 1069 463 3 1 1067 1 1064 6.70E-291 1008.4 6573 cellular organisms;Eukaryota;Opisthokonta;Metazoa;Eumetazoa;Bilateria;Protostomia;Spiralia;Lophotrochozoa;Mollusca;Bivalvia;Autobranchia;Pteriomorphia;Pectinida;Pectinoidea;Pectinidae;Mizuhopecten;Mizuhopecten yessoensis
UdeM00000023579 6573 1084.3 UdeM00000023579 XP_021375638.1 50 1161 540 11 344 1476 44 1191 0.00E+00 1084.3 6573 cellular organisms;Eukaryota;Opisthokonta;Metazoa;Eumetazoa;Bilateria;Protostomia;Spiralia;Lophotrochozoa;Mollusca;Bivalvia;Autobranchia;Pteriomorphia;Pectinida;Pectinoidea;Pectinidae;Mizuhopecten;Mizuhopecten yessoensis
UdeM00000023580 6596 397.1 UdeM00000023580 XP_045171678.1 60.3 340 127 4 1 337 1 335 2.20E-107 397.1 6596 cellular organisms;Eukaryota;Opisthokonta;Metazoa;Eumetazoa;Bilateria;Protostomia;Spiralia;Lophotrochozoa;Mollusca;Bivalvia;Autobranchia;Heteroconchia;Euheterodonta;Imparidentia;Venerida;Veneroidea;Veneridae;Mercenaria;Mercenaria mercenaria
UdeM00000023581 6565 302 UdeM00000023581 XP_022342998.1 87.9 198 20 2 4 201 3 196 7.00E-79 302 6565 cellular organisms;Eukaryota;Opisthokonta;Metazoa;Eumetazoa;Bilateria;Protostomia;Spiralia;Lophotrochozoa;Mollusca;Bivalvia;Autobranchia;Pteriomorphia;Ostreida;Ostreoidea;Ostreidae;Crassostrea;Crassostrea virginica
UdeM00000023582 50390 360.1 UdeM00000023582 XP_028154228.1 55.8 267 114 1 2 264 340 606 2.40E-96 360.1 50390 cellular organisms;Eukaryota;Opisthokonta;Metazoa;Eumetazoa;Bilateria;Protostomia;Ecdysozoa;Panarthropoda;Arthropoda;Mandibulata;Pancrustacea;Hexapoda;Insecta;Dicondylia;Pterygota;Neoptera;Endopterygota;Coleoptera;Polyphaga;Cucujiformia;Chrysomeloidea;Chrysomelidae;Galerucinae;Luperini;Diabroticina;Diabroticites;Diabrotica;Diabrotica virgifera;Diabrotica virgifera virgifera
UdeM00000023583 1735272 63.2 UdeM00000023583 XP_041347447.1 46.7 60 32 0 71 130 38 97 3.80E-07 63.2 1735272 cellular organisms;Eukaryota;Opisthokonta;Metazoa;Eumetazoa;Bilateria;Protostomia;Spiralia;Lophotrochozoa;Mollusca;Gastropoda;Neomphalina;Neomphaloidea;Peltospiridae;Gigantopelta;Gigantopelta aegis
UdeM00000023584 6573 256.1 UdeM00000023584 XP_021373211.1 32.5 495 319 7 119 608 550 1034 1.10E-64 256.1 6573 cellular organisms;Eukaryota;Opisthokonta;Metazoa;Eumetazoa;Bilateria;Protostomia;Spiralia;Lophotrochozoa;Mollusca;Bivalvia;Autobranchia;Pteriomorphia;Pectinida;Pectinoidea;Pectinidae;Mizuhopecten;Mizuhopecten yessoensis
UdeM00000023585 6596 295.8 UdeM00000023585 XP_045171284.1 90.6 160 15 0 1 160 45 204 3.30E-77 295.8 6596 cellular

organisms;Eukaryota;Opisthokonta;Metazoa;Eumetazoa;Bilateria;Protostomia;Spiralia;Lophotrochozoa;Mollusca;Bivalvia;Autobranchia;Heteroconchia;Euheterodonta;Imparidentia;Venerida;Veneroidea;Veneridae;Mercenaria;Mercenaria mercenaria
UdeM00000023586 6596 287.3 UdeM00000023586 XP_045171195.1 51.4 383 177 3 1 379 1 378 2.80E-74 287.3 6596 cellular organisms;Eukaryota;Opisthokonta;Metazoa;Eumetazoa;Bilateria;Protostomia;Spiralia;Lophotrochozoa;Mollusca;Bivalvia;Autobranchia;Heteroconchia;Euheterodonta;Imparidentia;Venerida;Veneroidea;Veneridae;Mercenaria;Mercenaria mercenaria
UdeM00000023587 6596 299.3 UdeM00000023587 XP_045171191.1 49.9 415 196 4 1 405 1 413 7.90E-78 299.3 6596 cellular organisms;Eukaryota;Opisthokonta;Metazoa;Eumetazoa;Bilateria;Protostomia;Spiralia;Lophotrochozoa;Mollusca;Bivalvia;Autobranchia;Heteroconchia;Euheterodonta;Imparidentia;Venerida;Veneroidea;Veneridae;Mercenaria;Mercenaria mercenaria
UdeM00000023588 29159 788.9 UdeM00000023588 XP_034338517.1 69.4 581 137 3 1 581 1049 1588 4.50E-225 788.9 29159 cellular organisms;Eukaryota;Opisthokonta;Metazoa;Eumetazoa;Bilateria;Protostomia;Spiralia;Lophotrochozoa;Mollusca;Bivalvia;Autobranchia;Pteriomorphia;Ostreida;Ostreoidea;Ostreidae;Crassostrea;Crassostrea gigas
UdeM00000023589 6596 902.9 UdeM00000023589 XP_045171259.1 54.6 1004 292 27 3 880 2 967 3.70E-259 902.9 6596 cellular organisms;Eukaryota;Opisthokonta;Metazoa;Eumetazoa;Bilateria;Protostomia;Spiralia;Lophotrochozoa;Mollusca;Bivalvia;Autobranchia;Heteroconchia;Euheterodonta;Imparidentia;Venerida;Veneroidea;Veneridae;Mercenaria;Mercenaria mercenaria
UdeM00000023590 6596 262.7 UdeM00000023590 XP_045171124.1 48.2 334 153 5 11 333 10 334 6.40E-67 262.7 6596 cellular organisms;Eukaryota;Opisthokonta;Metazoa;Eumetazoa;Bilateria;Protostomia;Spiralia;Lophotrochozoa;Mollusca;Bivalvia;Autobranchia;Heteroconchia;Euheterodonta;Imparidentia;Venerida;Veneroidea;Veneridae;Mercenaria;Mercenaria mercenaria
UdeM00000023591 6596 157.5 UdeM00000023591 XP_045171799.1 66 103 35 0 3 105 315 417 9.20E-36 157.5 6596 cellular organisms;Eukaryota;Opisthokonta;Metazoa;Eumetazoa;Bilateria;Protostomia;Spiralia;Lophotrochozoa;Mollusca;Bivalvia;Autobranchia;Heteroconchia;Euheterodonta;Imparidentia;Venerida;Veneroidea;Veneridae;Mercenaria;Mercenaria mercenaria
UdeM00000023592 6596 111.3 UdeM00000023592 XP_045171801.1 43 149 70 3 161 299 123 266 2.20E-21 111.3 6596 cellular organisms;Eukaryota;Opisthokonta;Metazoa;Eumetazoa;Bilateria;Protostomia;Spiralia;Lophotrochozoa;Mollusca;Bivalvia;Autobranchia;Heteroconchia;Euheterodonta;Imparidentia;Venerida;Veneroidea;Veneridae;Mercenaria;Mercenaria mercenaria
UdeM00000023593 6596 418.3 UdeM00000023593 XP_045171800.1 66.7 312 97 2 4 314 3 308 8.60E-114 418.3 6596 cellular organisms;Eukaryota;Opisthokonta;Metazoa;Eumetazoa;Bilateria;Protostomia;Spiralia;Lophotrochozoa;Mollusca;Bivalvia;Autobranchia;Heteroconchia;Euheterodonta;Imparidentia;Venerida;Veneroidea;Veneridae;Mercenaria;Mercenaria mercenaria
UdeM00000023594 6573 227.6 UdeM00000023594 XP_021374761.1 75 128 32 0 12 139 5 132 9.70E-57 227.6 6573 cellular organisms;Eukaryota;Opisthokonta;Metazoa;Eumetazoa;Bilateria;Protostomia;

Spiralia;Lophotrochozoa;Mollusca;Bivalvia;Autobranchia;Pteriomorphia;Pectinida;Pectinoidea;Pectinidae;Mizuhopecten;Mizuhopecten yessoensis
UdeM00000023595 6573 373.6 UdeM00000023595 XP_021374724.1 80 240 47 1 1 239 1 240 1.90E-100 373.6 6573 cellular organisms;Eukaryota;Opisthokonta;Metazoa;Eumetazoa;Bilateria;Protostomia;Spiralia;Lophotrochozoa;Mollusca;Bivalvia;Autobranchia;Pteriomorphia;Pectinida;Pectinoidea;Pectinidae;Mizuhopecten;Mizuhopecten yessoensis
UdeM00000023596 6573 273.1 UdeM00000023596 XP_021374724.1 84.6 162 24 1 1 161 1 162 2.30E-70 273.1 6573 cellular organisms;Eukaryota;Opisthokonta;Metazoa;Eumetazoa;Bilateria;Protostomia;Spiralia;Lophotrochozoa;Mollusca;Bivalvia;Autobranchia;Pteriomorphia;Pectinida;Pectinoidea;Pectinidae;Mizuhopecten;Mizuhopecten yessoensis
UdeM00000023597 6596 128.3 UdeM00000023597 XP_045175390.1 37.6 205 113 6 106 297 185 387 1.70E-26 128.3 6596 cellular organisms;Eukaryota;Opisthokonta;Metazoa;Eumetazoa;Bilateria;Protostomia;Spiralia;Lophotrochozoa;Mollusca;Bivalvia;Autobranchia;Heteroconchia;Euheterodonta;Imparidentia;Venerida;Veneroidea;Veneridae;Mercenaria;Mercenaria mercenaria
UdeM00000023598 6596 109.8 UdeM00000023598 XP_045175390.1 45.1 133 70 2 6 137 257 387 2.90E-21 109.8 6596 cellular organisms;Eukaryota;Opisthokonta;Metazoa;Eumetazoa;Bilateria;Protostomia;Spiralia;Lophotrochozoa;Mollusca;Bivalvia;Autobranchia;Heteroconchia;Euheterodonta;Imparidentia;Venerida;Veneroidea;Veneridae;Mercenaria;Mercenaria mercenaria
UdeM00000023599 6596 126.3 UdeM00000023599 XP_045175390.1 34.2 269 159 7 56 312 125 387 6.80E-26 126.3 6596 cellular organisms;Eukaryota;Opisthokonta;Metazoa;Eumetazoa;Bilateria;Protostomia;Spiralia;Lophotrochozoa;Mollusca;Bivalvia;Autobranchia;Heteroconchia;Euheterodonta;Imparidentia;Venerida;Veneroidea;Veneridae;Mercenaria;Mercenaria mercenaria
UdeM00000023601 6596 122.1 UdeM00000023601 XP_045175390.1 32.3 316 187 11 12 307 79 387 1.30E-24 122.1 6596 cellular organisms;Eukaryota;Opisthokonta;Metazoa;Eumetazoa;Bilateria;Protostomia;Spiralia;Lophotrochozoa;Mollusca;Bivalvia;Autobranchia;Heteroconchia;Euheterodonta;Imparidentia;Venerida;Veneroidea;Veneridae;Mercenaria;Mercenaria mercenaria
UdeM00000023602 6596 124 UdeM00000023602 XP_045175390.1 33.2 268 152 8 18 274 136 387 2.90E-25 124 6596 cellular organisms;Eukaryota;Opisthokonta;Metazoa;Eumetazoa;Bilateria;Protostomia;Spiralia;Lophotrochozoa;Mollusca;Bivalvia;Autobranchia;Heteroconchia;Euheterodonta;Imparidentia;Venerida;Veneroidea;Veneridae;Mercenaria;Mercenaria mercenaria
UdeM00000023603 6596 146.7 UdeM00000023603 XP_045171683.1 41.1 202 112 5 4 203 2 198 4.30E-32 146.7 6596 cellular organisms;Eukaryota;Opisthokonta;Metazoa;Eumetazoa;Bilateria;Protostomia;Spiralia;Lophotrochozoa;Mollusca;Bivalvia;Autobranchia;Heteroconchia;Euheterodonta;Imparidentia;Venerida;Veneroidea;Veneridae;Mercenaria;Mercenaria mercenaria
UdeM00000023604 29159 102.4 UdeM00000023604 XP_034315421.1 51.8 114 48 3 3 112 364 474 3.80E-19 102.4 29159 cellular organisms;Eukaryota;Opisthokonta;Metazoa;Eumetazoa;Bilateria;Protostomia;Spiralia;Lophotrochozoa;Mollusca;Bivalvia;Autobranchia;Pteriomorphia;Ostreida;Ostreoidea;Ostreidae;Crassostrea;Crassostrea gigas

UdeM00000023605 29159 161.4 UdeM00000023605 XP_034318948.1   72.9 96
      26   0   1   96   374   469   5.90E-37   161.4 29159 cellular
organisms;Eukaryota;Opisthokonta;Metazoa;Eumetazoa;Bilateria;Protostomia;
Spiralia;Lophotrochozoa;Mollusca;Bivalvia;Autobranchia;Pteriomorphia;Ostr
eida;Ostreoidea;Ostreidae;Crassostrea;Crassostrea gigas
UdeM00000023606 29159 375.6 UdeM00000023606 XP_034319609.1   41.6 473
      140   2   1   337   482   954   6.90E-101   375.6 29159 cellular
organisms;Eukaryota;Opisthokonta;Metazoa;Eumetazoa;Bilateria;Protostomia;
Spiralia;Lophotrochozoa;Mollusca;Bivalvia;Autobranchia;Pteriomorphia;Ostr
eida;Ostreoidea;Ostreidae;Crassostrea;Crassostrea gigas
UdeM00000023607 6596 117.9 UdeM00000023607 XP_045175388.1   43.2 139
      77   2   175   312   251   388   2.40E-23   117.9 6596 cellular
organisms;Eukaryota;Opisthokonta;Metazoa;Eumetazoa;Bilateria;Protostomia;
Spiralia;Lophotrochozoa;Mollusca;Bivalvia;Autobranchia;Heteroconchia;Euhe
terodonta;Imparidentia;Venerida;Veneroidea;Veneridae;Mercenaria;Mercenari
a mercenaria
UdeM00000023608 6596 117.9 UdeM00000023608 XP_045175388.1   43.2 139
      77   2   137   274   251   388   2.10E-23   117.9 6596 cellular
organisms;Eukaryota;Opisthokonta;Metazoa;Eumetazoa;Bilateria;Protostomia;
Spiralia;Lophotrochozoa;Mollusca;Bivalvia;Autobranchia;Heteroconchia;Euhe
terodonta;Imparidentia;Venerida;Veneroidea;Veneridae;Mercenaria;Mercenari
a mercenaria
UdeM00000023609 6596 164.1 UdeM00000023609 XP_045171683.1   42.2 199
      107   3   8   199   7   204   2.50E-37   164.1 6596 cellular
organisms;Eukaryota;Opisthokonta;Metazoa;Eumetazoa;Bilateria;Protostomia;
Spiralia;Lophotrochozoa;Mollusca;Bivalvia;Autobranchia;Heteroconchia;Euhe
terodonta;Imparidentia;Venerida;Veneroidea;Veneridae;Mercenaria;Mercenari
a mercenaria
UdeM00000023610 6596 152.1 UdeM00000023610 XP_045171683.1   40.6 202
      114   4   8   207   7   204   1.00E-33   152.1 6596 cellular
organisms;Eukaryota;Opisthokonta;Metazoa;Eumetazoa;Bilateria;Protostomia;
Spiralia;Lophotrochozoa;Mollusca;Bivalvia;Autobranchia;Heteroconchia;Euhe
terodonta;Imparidentia;Venerida;Veneroidea;Veneridae;Mercenaria;Mercenari
a mercenaria
UdeM00000023611 6596 61.2 UdeM00000023611 XP_045209009.1   37   92
      51   3   27   112   12   102   1.80E-06   61.2 6596 cellular
organisms;Eukaryota;Opisthokonta;Metazoa;Eumetazoa;Bilateria;Protostomia;
Spiralia;Lophotrochozoa;Mollusca;Bivalvia;Autobranchia;Heteroconchia;Euhe
terodonta;Imparidentia;Venerida;Veneroidea;Veneridae;Mercenaria;Mercenari
a mercenaria
UdeM00000023612 6573 123.6 UdeM00000023612 XP_021354173.1   39.5 185
      106   5   1   182   1   182   3.20E-25   123.6 6573 cellular
organisms;Eukaryota;Opisthokonta;Metazoa;Eumetazoa;Bilateria;Protostomia;
Spiralia;Lophotrochozoa;Mollusca;Bivalvia;Autobranchia;Pteriomorphia;Pect
inida;Pectinoidea;Pectinidae;Mizuhopecten;Mizuhopecten yessoensis
UdeM00000023613 6596 119.8 UdeM00000023613 XP_045172473.1   40.4 178
      97   4   7   181   6   177   4.50E-24   119.8 6596 cellular
organisms;Eukaryota;Opisthokonta;Metazoa;Eumetazoa;Bilateria;Protostomia;
Spiralia;Lophotrochozoa;Mollusca;Bivalvia;Autobranchia;Heteroconchia;Euhe
terodonta;Imparidentia;Venerida;Veneroidea;Veneridae;Mercenaria;Mercenari
a mercenaria
UdeM00000023614 6596 152.1 UdeM00000023614 XP_045171683.1   45.3 181
      92   5   5   183   32   207   9.20E-34   152.1 6596 cellular
organisms;Eukaryota;Opisthokonta;Metazoa;Eumetazoa;Bilateria;Protostomia;

Spiralia;Lophotrochozoa;Mollusca;Bivalvia;Autobranchia;Heteroconchia;Euheterodonta;Imparidentia;Venerida;Veneroidea;Veneridae;Mercenaria;Mercenaria mercenaria
UdeM00000023615 6573 207.2 UdeM00000023615 XP_021342392.1 27.3 933 522 24 15 873 10 860 8.60E-50 207.2 6573 cellular organisms;Eukaryota;Opisthokonta;Metazoa;Eumetazoa;Bilateria;Protostomia;Spiralia;Lophotrochozoa;Mollusca;Bivalvia;Autobranchia;Pteriomorphia;Pectinida;Pectinoidea;Pectinidae;Mizuhopecten;Mizuhopecten yessoensis
UdeM00000023616 29159 394 UdeM00000023616 XP_034298998.1 47.6 466 191 7 6 419 135 599 2.30E-106 394 29159 cellular organisms;Eukaryota;Opisthokonta;Metazoa;Eumetazoa;Bilateria;Protostomia;Spiralia;Lophotrochozoa;Mollusca;Bivalvia;Autobranchia;Pteriomorphia;Ostreida;Ostreoidea;Ostreidae;Crassostrea;Crassostrea gigas
UdeM00000023617 6573 167.2 UdeM00000023617 XP_021378264.1 57 151 59 2 1 145 509 659 1.60E-38 167.2 6573 cellular organisms;Eukaryota;Opisthokonta;Metazoa;Eumetazoa;Bilateria;Protostomia;Spiralia;Lophotrochozoa;Mollusca;Bivalvia;Autobranchia;Pteriomorphia;Pectinida;Pectinoidea;Pectinidae;Mizuhopecten;Mizuhopecten yessoensis
UdeM00000023618 1735272 232.6 UdeM00000023618 XP_041350169.1 65.5 177 55 2 14 184 13 189 3.90E-58 232.6 1735272 cellular organisms;Eukaryota;Opisthokonta;Metazoa;Eumetazoa;Bilateria;Protostomia;Spiralia;Lophotrochozoa;Mollusca;Gastropoda;Neomphalina;Neomphaloidea;Peltospiridae;Gigantopelta;Gigantopelta aegis
UdeM00000023619 1735272 250.8 UdeM00000023619 XP_041350169.1 69.7 178 52 2 14 190 13 189 1.40E-63 250.8 1735272 cellular organisms;Eukaryota;Opisthokonta;Metazoa;Eumetazoa;Bilateria;Protostomia;Spiralia;Lophotrochozoa;Mollusca;Gastropoda;Neomphalina;Neomphaloidea;Peltospiridae;Gigantopelta;Gigantopelta aegis
UdeM00000023620 29159 185.3 UdeM00000023620 XP_034298998.1 59.3 167 65 1 30 193 433 599 7.60E-44 185.3 29159 cellular organisms;Eukaryota;Opisthokonta;Metazoa;Eumetazoa;Bilateria;Protostomia;Spiralia;Lophotrochozoa;Mollusca;Bivalvia;Autobranchia;Pteriomorphia;Ostreida;Ostreoidea;Ostreidae;Crassostrea;Crassostrea gigas
UdeM00000023621 29159 270.8 UdeM00000023621 XP_034298998.1 46.1 317 153 4 1 300 125 440 2.20E-69 270.8 29159 cellular organisms;Eukaryota;Opisthokonta;Metazoa;Eumetazoa;Bilateria;Protostomia;Spiralia;Lophotrochozoa;Mollusca;Bivalvia;Autobranchia;Pteriomorphia;Ostreida;Ostreoidea;Ostreidae;Crassostrea;Crassostrea gigas
UdeM00000023622 6565 384.8 UdeM00000023622 XP_022335717.1 45.9 377 196 5 940 1314 1195 1565 4.40E-103 384.8 6565 cellular organisms;Eukaryota;Opisthokonta;Metazoa;Eumetazoa;Bilateria;Protostomia;Spiralia;Lophotrochozoa;Mollusca;Bivalvia;Autobranchia;Pteriomorphia;Ostreida;Ostreoidea;Ostreidae;Crassostrea;Crassostrea virginica
UdeM00000023625 6596 211.8 UdeM00000023625 XP_045171930.1 40.7 236 138 1 4 237 697 932 9.40E-52 211.8 6596 cellular organisms;Eukaryota;Opisthokonta;Metazoa;Eumetazoa;Bilateria;Protostomia;Spiralia;Lophotrochozoa;Mollusca;Bivalvia;Autobranchia;Heteroconchia;Euheterodonta;Imparidentia;Venerida;Veneroidea;Veneridae;Mercenaria;Mercenaria mercenaria
UdeM00000023626 6596 478.8 UdeM00000023626 XP_045171930.1 32.6 932 519 14 7 838 10 932 1.40E-131 478.8 6596 cellular organisms;Eukaryota;Opisthokonta;Metazoa;Eumetazoa;Bilateria;Protostomia;

Spiralia;Lophotrochozoa;Mollusca;Bivalvia;Autobranchia;Heteroconchia;Euheterodonta;Imparidentia;Venerida;Veneroidea;Veneridae;Mercenaria;Mercenaria mercenaria
UdeM00000023627 6596 117.1 UdeM00000023627 XP_045173872.1 71.8 71 20 0 1 71 18 88 1.10E-23 117.1 6596 cellular organisms;Eukaryota;Opisthokonta;Metazoa;Eumetazoa;Bilateria;Protostomia;Spiralia;Lophotrochozoa;Mollusca;Bivalvia;Autobranchia;Heteroconchia;Euheterodonta;Imparidentia;Venerida;Veneroidea;Veneridae;Mercenaria;Mercenaria mercenaria
UdeM00000023629 7574 309.3 UdeM00000023629 XP_013381563.1 47.7 342 162 7 1 332 60 394 6.20E-81 309.3 7574 cellular organisms;Eukaryota;Opisthokonta;Metazoa;Eumetazoa;Bilateria;Protostomia;Spiralia;Lophotrochozoa;Brachiopoda;Linguliformea;Lingulata;Lingulida;Linguloidea;Lingulidae;Lingula;Lingula anatina
UdeM00000023630 10224 112.8 UdeM00000023630 XP_006816668.1 35.5 169 106 2 40 208 291 456 7.60E-22 112.8 10224 cellular organisms;Eukaryota;Opisthokonta;Metazoa;Eumetazoa;Bilateria;Deuterostomia;Hemichordata;Enteropneusta;Harrimaniidae;Saccoglossus;Saccoglossus kowalevskii
UdeM00000023631 29159 112.1 UdeM00000023631 XP_034305965.1 43.2 111 63 0 53 163 63 173 7.50E-22 112.1 29159 cellular organisms;Eukaryota;Opisthokonta;Metazoa;Eumetazoa;Bilateria;Protostomia;Spiralia;Lophotrochozoa;Mollusca;Bivalvia;Autobranchia;Pteriomorphia;Ostreida;Ostreoidea;Ostreidae;Crassostrea;Crassostrea gigas
UdeM00000023632 29159 115.5 UdeM00000023632 XP_034305965.1 42.6 115 66 0 53 167 63 177 6.40E-23 115.5 29159 cellular organisms;Eukaryota;Opisthokonta;Metazoa;Eumetazoa;Bilateria;Protostomia;Spiralia;Lophotrochozoa;Mollusca;Bivalvia;Autobranchia;Pteriomorphia;Ostreida;Ostreoidea;Ostreidae;Crassostrea;Crassostrea gigas
UdeM00000023633 29159 112.1 UdeM00000023633 XP_034305965.1 43.2 111 63 0 53 163 63 173 7.60E-22 112.1 29159 cellular organisms;Eukaryota;Opisthokonta;Metazoa;Eumetazoa;Bilateria;Protostomia;Spiralia;Lophotrochozoa;Mollusca;Bivalvia;Autobranchia;Pteriomorphia;Ostreida;Ostreoidea;Ostreidae;Crassostrea;Crassostrea gigas
UdeM00000023634 7574 308.1 UdeM00000023634 XP_013381563.1 44.1 365 182 8 62 411 55 412 1.60E-80 308.1 7574 cellular organisms;Eukaryota;Opisthokonta;Metazoa;Eumetazoa;Bilateria;Protostomia;Spiralia;Lophotrochozoa;Brachiopoda;Linguliformea;Lingulata;Lingulida;Linguloidea;Lingulidae;Lingula;Lingula anatina
UdeM00000023635 6596 544.7 UdeM00000023635 XP_045171251.1 45.7 788 376 17 3 780 4 749 2.00E-151 544.7 6596 cellular organisms;Eukaryota;Opisthokonta;Metazoa;Eumetazoa;Bilateria;Protostomia;Spiralia;Lophotrochozoa;Mollusca;Bivalvia;Autobranchia;Heteroconchia;Euheterodonta;Imparidentia;Venerida;Veneroidea;Veneridae;Mercenaria;Mercenaria mercenaria
UdeM00000023636 6596 253.8 UdeM00000023636 XP_045172696.1 32.6 487 242 12 21 438 21 490 3.90E-64 253.8 6596 cellular organisms;Eukaryota;Opisthokonta;Metazoa;Eumetazoa;Bilateria;Protostomia;Spiralia;Lophotrochozoa;Mollusca;Bivalvia;Autobranchia;Heteroconchia;Euheterodonta;Imparidentia;Venerida;Veneroidea;Veneridae;Mercenaria;Mercenaria mercenaria
UdeM00000023637 6579 490.3 UdeM00000023637 XP_033747378.1 56.3 481 167 10 2 453 3 469 5.30E-135 490.3 6579 cellular organisms;Eukaryota;Opisthokonta;Metazoa;Eumetazoa;Bilateria;Protostomia;

Spiralia;Lophotrochozoa;Mollusca;Bivalvia;Autobranchia;Pteriomorphia;Pectinida;Pectinoidea;Pectinidae;Pecten;Pecten maximus
UdeM00000023638 6596 156.4 UdeM00000023638 XP_045172783.1 51.4 140 68 0 56 195 52 191 3.80E-35 156.4 6596 cellular organisms;Eukaryota;Opisthokonta;Metazoa;Eumetazoa;Bilateria;Protostomia;Spiralia;Lophotrochozoa;Mollusca;Bivalvia;Autobranchia;Heteroconchia;Euheterodonta;Imparidentia;Venerida;Veneroidea;Veneridae;Mercenaria;Mercenaria mercenaria
UdeM00000023640 6596 219.2 UdeM00000023640 XP_045173266.1 35.7 311 185 6 10 319 12 308 7.80E-54 219.2 6596 cellular organisms;Eukaryota;Opisthokonta;Metazoa;Eumetazoa;Bilateria;Protostomia;Spiralia;Lophotrochozoa;Mollusca;Bivalvia;Autobranchia;Heteroconchia;Euheterodonta;Imparidentia;Venerida;Veneroidea;Veneridae;Mercenaria;Mercenaria mercenaria
UdeM00000023641 1735272 531.9 UdeM00000023641 XP_041348019.1 57 433 183 2 40 472 1 430 8.00E-148 531.9 1735272 cellular organisms;Eukaryota;Opisthokonta;Metazoa;Eumetazoa;Bilateria;Protostomia;Spiralia;Lophotrochozoa;Mollusca;Gastropoda;Neomphalina;Neomphaloidea;Peltospiridae;Gigantopelta;Gigantopelta aegis
UdeM00000023642 29159 131.3 UdeM00000023642 XP_034337601.1 50.9 116 48 3 14 120 8 123 8.20E-28 131.3 29159 cellular organisms;Eukaryota;Opisthokonta;Metazoa;Eumetazoa;Bilateria;Protostomia;Spiralia;Lophotrochozoa;Mollusca;Bivalvia;Autobranchia;Pteriomorphia;Ostreida;Ostreoidea;Ostreidae;Crassostrea;Crassostrea gigas
UdeM00000023643 6596 446.4 UdeM00000023643 XP_045172121.1 45.7 588 271 11 8 560 113 687 5.70E-122 446.4 6596 cellular organisms;Eukaryota;Opisthokonta;Metazoa;Eumetazoa;Bilateria;Protostomia;Spiralia;Lophotrochozoa;Mollusca;Bivalvia;Autobranchia;Heteroconchia;Euheterodonta;Imparidentia;Venerida;Veneroidea;Veneridae;Mercenaria;Mercenaria mercenaria
UdeM00000023644 6596 132.9 UdeM00000023644 XP_045163627.1 25.4 480 323 13 59 519 507 970 1.60E-27 132.9 6596 cellular organisms;Eukaryota;Opisthokonta;Metazoa;Eumetazoa;Bilateria;Protostomia;Spiralia;Lophotrochozoa;Mollusca;Bivalvia;Autobranchia;Heteroconchia;Euheterodonta;Imparidentia;Venerida;Veneroidea;Veneridae;Mercenaria;Mercenaria mercenaria
UdeM00000023645 6596 117.5 UdeM00000023645 XP_045162069.1 22.9 733 494 23 4 698 282 981 7.60E-23 117.5 6596 cellular organisms;Eukaryota;Opisthokonta;Metazoa;Eumetazoa;Bilateria;Protostomia;Spiralia;Lophotrochozoa;Mollusca;Bivalvia;Autobranchia;Heteroconchia;Euheterodonta;Imparidentia;Venerida;Veneroidea;Veneridae;Mercenaria;Mercenaria mercenaria
UdeM00000023646 33528 134.4 UdeM00000023646 XP_043957434.1 45.3 139 75 1 1 139 247 384 1.10E-28 134.4 33528 cellular organisms;Eukaryota;Opisthokonta;Metazoa;Eumetazoa;Bilateria;Deuterostomia;Chordata;Craniata;Vertebrata;Gnathostomata;Teleostomi;Euteleostomi;Actinopterygii;Actinopteri;Neopterygii;Teleostei;Osteoglossocephalai;Clupeocephala;Euteleosteomorpha;Neoteleostei;Eurypterygia;Ctenosquamata;Acanthomorphata;Euacanthomorphacea;Percomorphaceae;Ovalentaria;Atherinomorphae;Cyprinodontiformes;Cyprinodontoidei;Poeciliidae;Poeciliinae;Gambusia;Gambusia affinis
UdeM00000023647 29159 292.7 UdeM00000023647 XP_034334268.1 62.6 211 76 1 32 242 1 208 4.20E-76 292.7 29159 cellular

organisms;Eukaryota;Opisthokonta;Metazoa;Eumetazoa;Bilateria;Protostomia;Spiralia;Lophotrochozoa;Mollusca;Bivalvia;Autobranchia;Pteriomorphia;Ostreida;Ostreoidea;Ostreidae;Crassostrea;Crassostrea gigas
UdeM00000041679 6596 763.1 UdeM00000041679 XP_045201368.1 52.4 825 362 10 4 815 3 809 4.40E-217 763.1 6596 cellular organisms;Eukaryota;Opisthokonta;Metazoa;Eumetazoa;Bilateria;Protostomia;Spiralia;Lophotrochozoa;Mollusca;Bivalvia;Autobranchia;Heteroconchia;Euheterodonta;Imparidentia;Venerida;Veneroidea;Veneridae;Mercenaria;Mercenaria mercenaria
UdeM00000041681 6596 429.1 UdeM00000041681 XP_045201815.1 37.4 708 409 12 14 707 17 704 1.30E-116 429.1 6596 cellular organisms;Eukaryota;Opisthokonta;Metazoa;Eumetazoa;Bilateria;Protostomia;Spiralia;Lophotrochozoa;Mollusca;Bivalvia;Autobranchia;Heteroconchia;Euheterodonta;Imparidentia;Venerida;Veneroidea;Veneridae;Mercenaria;Mercenaria mercenaria
UdeM00000041682 6596 423.7 UdeM00000041682 XP_045201815.1 37.1 712 412 15 14 710 17 707 5.40E-115 423.7 6596 cellular organisms;Eukaryota;Opisthokonta;Metazoa;Eumetazoa;Bilateria;Protostomia;Spiralia;Lophotrochozoa;Mollusca;Bivalvia;Autobranchia;Heteroconchia;Euheterodonta;Imparidentia;Venerida;Veneroidea;Veneridae;Mercenaria;Mercenaria mercenaria
UdeM00000041683 6596 73.9 UdeM00000041683 XP_045170001.1 47.5 101 48 3 1 98 242 340 3.30E-10 73.9 6596 cellular organisms;Eukaryota;Opisthokonta;Metazoa;Eumetazoa;Bilateria;Protostomia;Spiralia;Lophotrochozoa;Mollusca;Bivalvia;Autobranchia;Heteroconchia;Euheterodonta;Imparidentia;Venerida;Veneroidea;Veneridae;Mercenaria;Mercenaria mercenaria
UdeM00000041684 6596 146 UdeM00000041684 XP_045201815.1 36.3 259 150 6 4 259 261 507 7.10E-32 146 6596 cellular organisms;Eukaryota;Opisthokonta;Metazoa;Eumetazoa;Bilateria;Protostomia;Spiralia;Lophotrochozoa;Mollusca;Bivalvia;Autobranchia;Heteroconchia;Euheterodonta;Imparidentia;Venerida;Veneroidea;Veneridae;Mercenaria;Mercenaria mercenaria
UdeM00000041686 6596 118.2 UdeM00000041686 XP_045201815.1 37.6 181 88 3 1 172 166 330 1.10E-23 118.2 6596 cellular organisms;Eukaryota;Opisthokonta;Metazoa;Eumetazoa;Bilateria;Protostomia;Spiralia;Lophotrochozoa;Mollusca;Bivalvia;Autobranchia;Heteroconchia;Euheterodonta;Imparidentia;Venerida;Veneroidea;Veneridae;Mercenaria;Mercenaria mercenaria
UdeM00000041688 6596 436.4 UdeM00000041688 XP_045201815.1 38.2 725 408 14 14 723 17 716 8.10E-119 436.4 6596 cellular organisms;Eukaryota;Opisthokonta;Metazoa;Eumetazoa;Bilateria;Protostomia;Spiralia;Lophotrochozoa;Mollusca;Bivalvia;Autobranchia;Heteroconchia;Euheterodonta;Imparidentia;Venerida;Veneroidea;Veneridae;Mercenaria;Mercenaria mercenaria
UdeM00000041689 6596 442.2 UdeM00000041689 XP_045201815.1 38.6 725 405 14 14 723 17 716 1.50E-120 442.2 6596 cellular organisms;Eukaryota;Opisthokonta;Metazoa;Eumetazoa;Bilateria;Protostomia;Spiralia;Lophotrochozoa;Mollusca;Bivalvia;Autobranchia;Heteroconchia;Euheterodonta;Imparidentia;Venerida;Veneroidea;Veneridae;Mercenaria;Mercenaria mercenaria
UdeM00000041690 6596 438 UdeM00000041690 XP_045201815.1 38.8 706 399 12 14 704 17 704 2.80E-119 438 6596 cellular organisms;Eukaryota;Opisthokonta;Metazoa;Eumetazoa;Bilateria;Protostomia;

Spiralia;Lophotrochozoa;Mollusca;Bivalvia;Autobranchia;Heteroconchia;Euheterodonta;Imparidentia;Venerida;Veneroidea;Veneridae;Mercenaria;Mercenaria mercenaria
UdeM00000041691 6573 263.8 UdeM00000041691 XP_021380242.1 55.3 282 112 6 1 279 1 271 2.40E-67 263.8 6573 cellular organisms;Eukaryota;Opisthokonta;Metazoa;Eumetazoa;Bilateria;Protostomia;Spiralia;Lophotrochozoa;Mollusca;Bivalvia;Autobranchia;Pteriomorphia;Pectinida;Pectinoidea;Pectinidae;Mizuhopecten;Mizuhopecten yessoensis
UdeM00000041692 6579 179.5 UdeM00000041692 XP_033732123.1 56.5 138 60 0 15 152 3 140 3.50E-42 179.5 6579 cellular organisms;Eukaryota;Opisthokonta;Metazoa;Eumetazoa;Bilateria;Protostomia;Spiralia;Lophotrochozoa;Mollusca;Bivalvia;Autobranchia;Pteriomorphia;Pectinida;Pectinoidea;Pectinidae;Pecten;Pecten maximus
UdeM00000041693 1735272 510 UdeM00000041693 XP_041368427.1 43.5 545 283 6 16 544 32 567 4.10E-141 510 1735272 cellular organisms;Eukaryota;Opisthokonta;Metazoa;Eumetazoa;Bilateria;Protostomia;Spiralia;Lophotrochozoa;Mollusca;Gastropoda;Neomphalina;Neomphaloidea;Peltospiridae;Gigantopelta;Gigantopelta aegis
UdeM00000041694 1735272 287 UdeM00000041694 XP_041368427.1 38.7 331 181 4 3 317 243 567 3.40E-74 287 1735272 cellular organisms;Eukaryota;Opisthokonta;Metazoa;Eumetazoa;Bilateria;Protostomia;Spiralia;Lophotrochozoa;Mollusca;Gastropoda;Neomphalina;Neomphaloidea;Peltospiridae;Gigantopelta;Gigantopelta aegis
UdeM00000041696 6579 580.5 UdeM00000041696 XP_033732123.1 49.1 534 253 5 20 542 13 538 2.80E-162 580.5 6579 cellular organisms;Eukaryota;Opisthokonta;Metazoa;Eumetazoa;Bilateria;Protostomia;Spiralia;Lophotrochozoa;Mollusca;Bivalvia;Autobranchia;Pteriomorphia;Pectinida;Pectinoidea;Pectinidae;Pecten;Pecten maximus
UdeM00000041697 6579 438.7 UdeM00000041697 XP_033732123.1 45.9 460 232 7 4 455 88 538 9.80E-120 438.7 6579 cellular organisms;Eukaryota;Opisthokonta;Metazoa;Eumetazoa;Bilateria;Protostomia;Spiralia;Lophotrochozoa;Mollusca;Bivalvia;Autobranchia;Pteriomorphia;Pectinida;Pectinoidea;Pectinidae;Pecten;Pecten maximus
UdeM00000041698 6579 584.7 UdeM00000041698 XP_033732123.1 49.1 538 260 5 10 539 6 537 1.30E-163 584.7 6579 cellular organisms;Eukaryota;Opisthokonta;Metazoa;Eumetazoa;Bilateria;Protostomia;Spiralia;Lophotrochozoa;Mollusca;Bivalvia;Autobranchia;Pteriomorphia;Pectinida;Pectinoidea;Pectinidae;Pecten;Pecten maximus
UdeM00000041699 6579 601.7 UdeM00000041699 XP_033732123.1 49.8 544 259 6 10 545 6 543 1.00E-168 601.7 6579 cellular organisms;Eukaryota;Opisthokonta;Metazoa;Eumetazoa;Bilateria;Protostomia;Spiralia;Lophotrochozoa;Mollusca;Bivalvia;Autobranchia;Pteriomorphia;Pectinida;Pectinoidea;Pectinidae;Pecten;Pecten maximus
UdeM00000041700 64144 199.9 UdeM00000041700 XP_026225028.1 34.2 292 172 3 202 477 5 292 7.30E-48 199.9 64144 cellular organisms;Eukaryota;Opisthokonta;Metazoa;Eumetazoa;Bilateria;Deuterostomia;Chordata;Craniata;Vertebrata;Gnathostomata;Teleostomi;Euteleostomi;Actinopterygii;Actinopteri;Neopterygii;Teleostei;Osteoglossocephalai;Clupeocephala;Euteleosteomorpha;Neoteleostei;Eurypterygia;Ctenosquamata;Acanthomorphata;Euacanthomorphacea;Percomorphaceae;Anabantaria;Anabantiformes;Anabantoidei;Anabantidae;Anabas;Anabas testudineus

UdeM00000041701 6596 661.8 UdeM00000041701 XP_045204505.1 74.8 405
102 0 4 408 17 421 5.90E-187 661.8 6596 cellular
organisms;Eukaryota;Opisthokonta;Metazoa;Eumetazoa;Bilateria;Protostomia;
Spiralia;Lophotrochozoa;Mollusca;Bivalvia;Autobranchia;Heteroconchia;Euhe
terodonta;Imparidentia;Venerida;Veneroidea;Veneridae;Mercenaria;Mercenari
a mercenaria
UdeM00000041702 29159 87.4 UdeM00000041702 XP_034331441.1 47.8 90
45 2 1 89 722 810 9.90E-15 87.4 29159 cellular
organisms;Eukaryota;Opisthokonta;Metazoa;Eumetazoa;Bilateria;Protostomia;
Spiralia;Lophotrochozoa;Mollusca;Bivalvia;Autobranchia;Pteriomorphia;Ostr
eida;Ostreoidea;Ostreidae;Crassostrea;Crassostrea gigas
UdeM00000041703 29159 133.7 UdeM00000041703 XP_034331441.1 46 139
72 3 1 138 674 810 1.90E-28 133.7 29159 cellular
organisms;Eukaryota;Opisthokonta;Metazoa;Eumetazoa;Bilateria;Protostomia;
Spiralia;Lophotrochozoa;Mollusca;Bivalvia;Autobranchia;Pteriomorphia;Ostr
eida;Ostreoidea;Ostreidae;Crassostrea;Crassostrea gigas
UdeM00000041705 6596 58.2 UdeM00000041705 XP_045199492.1 31.1 106
69 3 21 123 97 201 8.90E-06 58.2 6596 cellular
organisms;Eukaryota;Opisthokonta;Metazoa;Eumetazoa;Bilateria;Protostomia;
Spiralia;Lophotrochozoa;Mollusca;Bivalvia;Autobranchia;Heteroconchia;Euhe
terodonta;Imparidentia;Venerida;Veneroidea;Veneridae;Mercenaria;Mercenari
a mercenaria
UdeM00000041706 6596 58.5 UdeM00000041706 XP_045208321.1 27.2 114
67 3 67 173 137 241 9.60E-06 58.5 6596 cellular
organisms;Eukaryota;Opisthokonta;Metazoa;Eumetazoa;Bilateria;Protostomia;
Spiralia;Lophotrochozoa;Mollusca;Bivalvia;Autobranchia;Heteroconchia;Euhe
terodonta;Imparidentia;Venerida;Veneroidea;Veneridae;Mercenaria;Mercenari
a mercenaria
UdeM00000041708 6596 123.2 UdeM00000041708 XP_045194453.1 34.1 211
128 5 1 204 56 262 3.80E-25 123.2 6596 cellular
organisms;Eukaryota;Opisthokonta;Metazoa;Eumetazoa;Bilateria;Protostomia;
Spiralia;Lophotrochozoa;Mollusca;Bivalvia;Autobranchia;Heteroconchia;Euhe
terodonta;Imparidentia;Venerida;Veneroidea;Veneridae;Mercenaria;Mercenari
a mercenaria
UdeM00000041709 6596 118.6 UdeM00000041709 XP_045194453.1 33.2 196
120 5 1 189 56 247 8.50E-24 118.6 6596 cellular
organisms;Eukaryota;Opisthokonta;Metazoa;Eumetazoa;Bilateria;Protostomia;
Spiralia;Lophotrochozoa;Mollusca;Bivalvia;Autobranchia;Heteroconchia;Euhe
terodonta;Imparidentia;Venerida;Veneroidea;Veneridae;Mercenaria;Mercenari
a mercenaria
UdeM00000041710 6573 176.8 UdeM00000041710 XP_021356708.1 60.4 144
55 1 4 145 6 149 2.10E-41 176.8 6573 cellular
organisms;Eukaryota;Opisthokonta;Metazoa;Eumetazoa;Bilateria;Protostomia;
Spiralia;Lophotrochozoa;Mollusca;Bivalvia;Autobranchia;Pteriomorphia;Pect
inida;Pectinoidea;Pectinidae;Mizuhopecten;Mizuhopecten yessoensis
UdeM00000041711 400727 164.5 UdeM00000041711 XP_025111853.1 58.9
146 59 1 7 152 8 152 1.10E-37 164.5 400727
cellular
organisms;Eukaryota;Opisthokonta;Metazoa;Eumetazoa;Bilateria;Protostomia;
Spiralia;Lophotrochozoa;Mollusca;Gastropoda;Caenogastropoda;Architaeniogl
ossa;Ampullarioidea;Ampullariidae;Pomacea;Pomacea canaliculata
UdeM00000041712 6565 953.4 UdeM00000041712 XP_022324969.1 68 716
201 7 7 710 3 702 1.70E-274 953.4 6565 cellular
organisms;Eukaryota;Opisthokonta;Metazoa;Eumetazoa;Bilateria;Protostomia;

Spiralia;Lophotrochozoa;Mollusca;Bivalvia;Autobranchia;Pteriomorphia;Ostreida;Ostreoidea;Ostreidae;Crassostrea;Crassostrea virginica
UdeM00000041713 6579 330.9 UdeM00000041713 XP_033730706.1 38.1 533 240 8 2 532 12 456 3.10E-87 330.9 6579 cellular organisms;Eukaryota;Opisthokonta;Metazoa;Eumetazoa;Bilateria;Protostomia;Spiralia;Lophotrochozoa;Mollusca;Bivalvia;Autobranchia;Pteriomorphia;Pectinida;Pectinoidea;Pectinidae;Pecten;Pecten maximus
UdeM00000041714 6565 142.5 UdeM00000041714 XP_022321863.1 33.7 279 159 11 78 348 275 535 1.10E-30 142.5 6565 cellular organisms;Eukaryota;Opisthokonta;Metazoa;Eumetazoa;Bilateria;Protostomia;Spiralia;Lophotrochozoa;Mollusca;Bivalvia;Autobranchia;Pteriomorphia;Ostreida;Ostreoidea;Ostreidae;Crassostrea;Crassostrea virginica
UdeM00000041715 6596 67 UdeM00000041715 XP_045171512.1 24.6 260 161 12 39 289 403 636 4.70E-08 67 6596 cellular organisms;Eukaryota;Opisthokonta;Metazoa;Eumetazoa;Bilateria;Protostomia;Spiralia;Lophotrochozoa;Mollusca;Bivalvia;Autobranchia;Heteroconchia;Euheterodonta;Imparidentia;Venerida;Veneroidea;Veneridae;Mercenaria;Mercenaria mercenaria
UdeM00000025567 29159 302.4 UdeM00000025567 XP_034303860.1 41.2 393 160 7 11 342 110 492 7.50E-79 302.4 29159 cellular organisms;Eukaryota;Opisthokonta;Metazoa;Eumetazoa;Bilateria;Protostomia;Spiralia;Lophotrochozoa;Mollusca;Bivalvia;Autobranchia;Pteriomorphia;Ostreida;Ostreoidea;Ostreidae;Crassostrea;Crassostrea gigas
UdeM00000025568 29159 125.9 UdeM00000025568 XP_011451530.1 48.3 116 58 2 45 158 96 211 4.40E-26 125.9 29159 cellular organisms;Eukaryota;Opisthokonta;Metazoa;Eumetazoa;Bilateria;Protostomia;Spiralia;Lophotrochozoa;Mollusca;Bivalvia;Autobranchia;Pteriomorphia;Ostreida;Ostreoidea;Ostreidae;Crassostrea;Crassostrea gigas
UdeM00000025570 6596 117.9 UdeM00000025570 XP_045205318.1 32 281 162 10 1 257 1 276 2.00E-23 117.9 6596 cellular organisms;Eukaryota;Opisthokonta;Metazoa;Eumetazoa;Bilateria;Protostomia;Spiralia;Lophotrochozoa;Mollusca;Bivalvia;Autobranchia;Heteroconchia;Euheterodonta;Imparidentia;Venerida;Veneroidea;Veneridae;Mercenaria;Mercenaria mercenaria
UdeM00000025571 50429 167.2 UdeM00000025571 XP_022807755.1 53.5 159 71 2 2 160 5 160 1.90E-38 167.2 50429 cellular organisms;Eukaryota;Opisthokonta;Metazoa;Eumetazoa;Cnidaria;Anthozoa;Hexacorallia;Scleractinia;Astrocoeniina;Pocilloporidae;Stylophora;Stylophora pistillata
UdeM00000025572 6573 282.3 UdeM00000025572 XP_021346251.1 54.6 249 109 2 1 249 1 245 5.90E-73 282.3 6573 cellular organisms;Eukaryota;Opisthokonta;Metazoa;Eumetazoa;Bilateria;Protostomia;Spiralia;Lophotrochozoa;Mollusca;Bivalvia;Autobranchia;Pteriomorphia;Pectinida;Pectinoidea;Pectinidae;Mizuhopecten;Mizuhopecten yessoensis
UdeM00000025573 6579 543.1 UdeM00000025573 XP_033743925.1 64.5 408 143 1 19 426 27 432 3.10E-151 543.1 6579 cellular organisms;Eukaryota;Opisthokonta;Metazoa;Eumetazoa;Bilateria;Protostomia;Spiralia;Lophotrochozoa;Mollusca;Bivalvia;Autobranchia;Pteriomorphia;Pectinida;Pectinoidea;Pectinidae;Pecten;Pecten maximus
UdeM00000025574 6596 84.3 UdeM00000025574 XP_045158901.1 48.1 81 41 1 1 80 10 90 7.50E-14 84.3 6596 cellular organisms;Eukaryota;Opisthokonta;Metazoa;Eumetazoa;Bilateria;Protostomia;Spiralia;Lophotrochozoa;Mollusca;Bivalvia;Autobranchia;Heteroconchia;Euhe

terodonta;Imparidentia;Venerida;Veneroidea;Veneridae;Mercenaria;Mercenari
a mercenaria
UdeM00000025575 6596 405.2 UdeM00000025575 XP_045187150.1 45.1 477
226 5 1 449 135 603 1.10E-109 405.2 6596 cellular
organisms;Eukaryota;Opisthokonta;Metazoa;Eumetazoa;Bilateria;Protostomia;
Spiralia;Lophotrochozoa;Mollusca;Bivalvia;Autobranchia;Heteroconchia;Euhe
terodonta;Imparidentia;Venerida;Veneroidea;Veneridae;Mercenaria;Mercenari
a mercenaria
UdeM00000025576 29159 301.2 UdeM00000025576 XP_011426907.2 55 309
107 9 10 301 9 302 4.20E-78 301.2 29159 cellular
organisms;Eukaryota;Opisthokonta;Metazoa;Eumetazoa;Bilateria;Protostomia;
Spiralia;Lophotrochozoa;Mollusca;Bivalvia;Autobranchia;Pteriomorphia;Ostr
eida;Ostreoidea;Ostreidae;Crassostrea;Crassostrea gigas
UdeM00000025577 6596 862.8 UdeM00000025577 XP_045204260.1 63.6 681
229 11 25 694 111 783 2.90E-247 862.8 6596 cellular
organisms;Eukaryota;Opisthokonta;Metazoa;Eumetazoa;Bilateria;Protostomia;
Spiralia;Lophotrochozoa;Mollusca;Bivalvia;Autobranchia;Heteroconchia;Euhe
terodonta;Imparidentia;Venerida;Veneroidea;Veneridae;Mercenaria;Mercenari
a mercenaria
UdeM00000025578 6579 149.1 UdeM00000025578 XP_033743043.1 46.7 152
75 2 1 151 119 265 4.90E-33 149.1 6579 cellular
organisms;Eukaryota;Opisthokonta;Metazoa;Eumetazoa;Bilateria;Protostomia;
Spiralia;Lophotrochozoa;Mollusca;Bivalvia;Autobranchia;Pteriomorphia;Pect
inida;Pectinoidea;Pectinidae;Pecten;Pecten maximus
UdeM00000025579 6573 592.4 UdeM00000025579 XP_021359067.1 49.3 604
290 9 1 594 10 607 6.30E-166 592.4 6573 cellular
organisms;Eukaryota;Opisthokonta;Metazoa;Eumetazoa;Bilateria;Protostomia;
Spiralia;Lophotrochozoa;Mollusca;Bivalvia;Autobranchia;Pteriomorphia;Pect
inida;Pectinoidea;Pectinidae;Mizuhopecten;Mizuhopecten yessoensis
UdeM00000025580 6596 308.1 UdeM00000025580 XP_045204011.1 82.4 187
3 1 1 187 1 157 7.50E-81 308.1 6596 cellular
organisms;Eukaryota;Opisthokonta;Metazoa;Eumetazoa;Bilateria;Protostomia;
Spiralia;Lophotrochozoa;Mollusca;Bivalvia;Autobranchia;Heteroconchia;Euhe
terodonta;Imparidentia;Venerida;Veneroidea;Veneridae;Mercenaria;Mercenari
a mercenaria
UdeM00000018225 6596 788.5 UdeM00000018225 XP_045199770.1 52 777
322 13 73 837 78 815 8.40E-225 788.5 6596 cellular
organisms;Eukaryota;Opisthokonta;Metazoa;Eumetazoa;Bilateria;Protostomia;
Spiralia;Lophotrochozoa;Mollusca;Bivalvia;Autobranchia;Heteroconchia;Euhe
terodonta;Imparidentia;Venerida;Veneroidea;Veneridae;Mercenaria;Mercenari
a mercenaria
UdeM00000018226 6596 748.4 UdeM00000018226 XP_045199770.1 53 727
292 12 1 715 127 815 8.20E-213 748.4 6596 cellular
organisms;Eukaryota;Opisthokonta;Metazoa;Eumetazoa;Bilateria;Protostomia;
Spiralia;Lophotrochozoa;Mollusca;Bivalvia;Autobranchia;Heteroconchia;Euhe
terodonta;Imparidentia;Venerida;Veneroidea;Veneridae;Mercenaria;Mercenari
a mercenaria
UdeM00000018227 6596 501.5 UdeM00000018227 XP_045199771.1 36.3 1836
799 54 1 1633 31 1699 4.70E-138 501.5 6596 cellular
organisms;Eukaryota;Opisthokonta;Metazoa;Eumetazoa;Bilateria;Protostomia;
Spiralia;Lophotrochozoa;Mollusca;Bivalvia;Autobranchia;Heteroconchia;Euhe
terodonta;Imparidentia;Venerida;Veneroidea;Veneridae;Mercenaria;Mercenari
a mercenaria

UdeM00000018228	6579	229.9	UdeM00000018228	XP_033758968.1	24.6	679
467	14	15	670	14	670	9.60E-57	229.9	6579	cellular
organisms;Eukaryota;Opisthokonta;Metazoa;Eumetazoa;Bilateria;Protostomia;
Spiralia;Lophotrochozoa;Mollusca;Bivalvia;Autobranchia;Pteriomorphia;Pect
inida;Pectinoidea;Pectinidae;Pecten;Pecten maximus
UdeM00000018229	6596	595.9	UdeM00000018229	XP_045200069.1	78.3	374
76	3	1	370	1	373	3.70E-167	595.9	6596	cellular
organisms;Eukaryota;Opisthokonta;Metazoa;Eumetazoa;Bilateria;Protostomia;
Spiralia;Lophotrochozoa;Mollusca;Bivalvia;Autobranchia;Heteroconchia;Euhe
terodonta;Imparidentia;Venerida;Veneroidea;Veneridae;Mercenaria;Mercenari
a mercenaria
UdeM00000018230	29159	142.9	UdeM00000018230	XP_011450416.1	68	103
33	0	1	103	381	483	2.40E-31	142.9	29159	cellular
organisms;Eukaryota;Opisthokonta;Metazoa;Eumetazoa;Bilateria;Protostomia;
Spiralia;Lophotrochozoa;Mollusca;Bivalvia;Autobranchia;Pteriomorphia;Ostr
eida;Ostreoidea;Ostreidae;Crassostrea;Crassostrea gigas
UdeM00000018232	29159	547	UdeM00000018232	XP_011450418.2	75.1	354
87	1	24	376	54	407	1.90E-152	547	29159	cellular
organisms;Eukaryota;Opisthokonta;Metazoa;Eumetazoa;Bilateria;Protostomia;
Spiralia;Lophotrochozoa;Mollusca;Bivalvia;Autobranchia;Pteriomorphia;Ostr
eida;Ostreoidea;Ostreidae;Crassostrea;Crassostrea gigas
UdeM00000018234	6596	632.1	UdeM00000018234	XP_045201105.1	70.3	481
115	8	1	465	1	469	5.60E-178	632.1	6596	cellular
organisms;Eukaryota;Opisthokonta;Metazoa;Eumetazoa;Bilateria;Protostomia;
Spiralia;Lophotrochozoa;Mollusca;Bivalvia;Autobranchia;Heteroconchia;Euhe
terodonta;Imparidentia;Venerida;Veneroidea;Veneridae;Mercenaria;Mercenari
a mercenaria
UdeM00000018235	1735272	200.3	UdeM00000018235	XP_041351430.1	70.1
134	36	2	9	139	18	150	1.60E-48	200.3	1735272
cellular
organisms;Eukaryota;Opisthokonta;Metazoa;Eumetazoa;Bilateria;Protostomia;
Spiralia;Lophotrochozoa;Mollusca;Gastropoda;Neomphalina;Neomphaloidea;Pel
tospiridae;Gigantopelta;Gigantopelta aegis
UdeM00000018236	6596	604.7	UdeM00000018236	XP_045200638.1	44.3	778
181	7	2	702	155	757	1.40E-169	604.7	6596	cellular
organisms;Eukaryota;Opisthokonta;Metazoa;Eumetazoa;Bilateria;Protostomia;
Spiralia;Lophotrochozoa;Mollusca;Bivalvia;Autobranchia;Heteroconchia;Euhe
terodonta;Imparidentia;Venerida;Veneroidea;Veneridae;Mercenaria;Mercenari
a mercenaria
UdeM00000018237	29159	240.7	UdeM00000018237	XP_011412587.1	78	223
40	2	1	220	1	217	1.70E-60	240.7	29159	cellular
organisms;Eukaryota;Opisthokonta;Metazoa;Eumetazoa;Bilateria;Protostomia;
Spiralia;Lophotrochozoa;Mollusca;Bivalvia;Autobranchia;Pteriomorphia;Ostr
eida;Ostreoidea;Ostreidae;Crassostrea;Crassostrea gigas
UdeM00000018239	1735272	228	UdeM00000018239	XP_041356261.1	59
217	76	4	11	227	27	230	1.90E-56	228	1735272
cellular
organisms;Eukaryota;Opisthokonta;Metazoa;Eumetazoa;Bilateria;Protostomia;
Spiralia;Lophotrochozoa;Mollusca;Gastropoda;Neomphalina;Neomphaloidea;Pel
tospiridae;Gigantopelta;Gigantopelta aegis
UdeM00000018240	6579	510.8	UdeM00000018240	XP_033760964.1	70.5	359
88	4	85	441	250	592	1.80E-141	510.8	6579	cellular
organisms;Eukaryota;Opisthokonta;Metazoa;Eumetazoa;Bilateria;Protostomia;

Spiralia;Lophotrochozoa;Mollusca;Bivalvia;Autobranchia;Pteriomorphia;Pectinida;Pectinoidea;Pectinidae;Pecten;Pecten maximus
UdeM00000018241 6596 1229.2 UdeM00000018241 XP_045199640.1 57.1 1148 428 15 37 1156 21 1132 0.00E+00 1229.2 6596 cellular organisms;Eukaryota;Opisthokonta;Metazoa;Eumetazoa;Bilateria;Protostomia;Spiralia;Lophotrochozoa;Mollusca;Bivalvia;Autobranchia;Heteroconchia;Euheterodonta;Imparidentia;Venerida;Veneroidea;Veneridae;Mercenaria;Mercenaria mercenaria
UdeM00000018242 29159 90.9 UdeM00000018242 XP_011453058.2 26.5 245 165 7 44 276 67 308 2.80E-15 90.9 29159 cellular organisms;Eukaryota;Opisthokonta;Metazoa;Eumetazoa;Bilateria;Protostomia;Spiralia;Lophotrochozoa;Mollusca;Bivalvia;Autobranchia;Pteriomorphia;Ostreida;Ostreoidea;Ostreidae;Crassostrea;Crassostrea gigas
UdeM00000018243 6596 159.8 UdeM00000018243 XP_045174908.1 40 235 136 3 153 387 340 569 6.90E-36 159.8 6596 cellular organisms;Eukaryota;Opisthokonta;Metazoa;Eumetazoa;Bilateria;Protostomia;Spiralia;Lophotrochozoa;Mollusca;Bivalvia;Autobranchia;Heteroconchia;Euheterodonta;Imparidentia;Venerida;Veneroidea;Veneridae;Mercenaria;Mercenaria mercenaria
UdeM00000018244 6596 158.7 UdeM00000018244 XP_045174908.1 40.4 235 135 3 153 387 340 569 1.50E-35 158.7 6596 cellular organisms;Eukaryota;Opisthokonta;Metazoa;Eumetazoa;Bilateria;Protostomia;Spiralia;Lophotrochozoa;Mollusca;Bivalvia;Autobranchia;Heteroconchia;Euheterodonta;Imparidentia;Venerida;Veneroidea;Veneridae;Mercenaria;Mercenaria mercenaria
UdeM00000018245 6596 155.6 UdeM00000018245 XP_045174908.1 42 231 125 5 161 387 344 569 1.30E-34 155.6 6596 cellular organisms;Eukaryota;Opisthokonta;Metazoa;Eumetazoa;Bilateria;Protostomia;Spiralia;Lophotrochozoa;Mollusca;Bivalvia;Autobranchia;Heteroconchia;Euheterodonta;Imparidentia;Venerida;Veneroidea;Veneridae;Mercenaria;Mercenaria mercenaria
UdeM00000018247 6596 135.6 UdeM00000018247 XP_045174908.1 45.1 175 91 3 2 176 400 569 6.40E-29 135.6 6596 cellular organisms;Eukaryota;Opisthokonta;Metazoa;Eumetazoa;Bilateria;Protostomia;Spiralia;Lophotrochozoa;Mollusca;Bivalvia;Autobranchia;Heteroconchia;Euheterodonta;Imparidentia;Venerida;Veneroidea;Veneridae;Mercenaria;Mercenaria mercenaria
UdeM00000018248 6596 418.7 UdeM00000018248 XP_045201065.1 63.1 317 115 1 1 317 1 315 6.60E-114 418.7 6596 cellular organisms;Eukaryota;Opisthokonta;Metazoa;Eumetazoa;Bilateria;Protostomia;Spiralia;Lophotrochozoa;Mollusca;Bivalvia;Autobranchia;Heteroconchia;Euheterodonta;Imparidentia;Venerida;Veneroidea;Veneridae;Mercenaria;Mercenaria mercenaria
UdeM00000018249 6596 109.8 UdeM00000018249 XP_045201067.1 26.5 502 273 12 3 430 4 483 1.40E-20 109.8 6596 cellular organisms;Eukaryota;Opisthokonta;Metazoa;Eumetazoa;Bilateria;Protostomia;Spiralia;Lophotrochozoa;Mollusca;Bivalvia;Autobranchia;Heteroconchia;Euheterodonta;Imparidentia;Venerida;Veneroidea;Veneridae;Mercenaria;Mercenaria mercenaria
UdeM00000018250 6565 183.7 UdeM00000018250 XP_022297447.1 52.5 158 73 1 1 158 211 366 1.80E-43 183.7 6565 cellular organisms;Eukaryota;Opisthokonta;Metazoa;Eumetazoa;Bilateria;Protostomia;

Spiralia;Lophotrochozoa;Mollusca;Bivalvia;Autobranchia;Pteriomorphia;Ostreida;Ostreoidea;Ostreidae;Crassostrea;Crassostrea virginica
UdeM00000018253 6596 207.6 UdeM00000018253 XP_045184729.1 28.8 513 305 10 1 460 193 698 3.40E-50 207.6 6596 cellular organisms;Eukaryota;Opisthokonta;Metazoa;Eumetazoa;Bilateria;Protostomia;Spiralia;Lophotrochozoa;Mollusca;Bivalvia;Autobranchia;Heteroconchia;Euheterodonta;Imparidentia;Venerida;Veneroidea;Veneridae;Mercenaria;Mercenaria mercenaria
UdeM00000018254 6596 81.6 UdeM00000018254 XP_045194475.1 39 118 72 0 4 121 3 120 1.00E-12 81.6 6596 cellular organisms;Eukaryota;Opisthokonta;Metazoa;Eumetazoa;Bilateria;Protostomia;Spiralia;Lophotrochozoa;Mollusca;Bivalvia;Autobranchia;Heteroconchia;Euheterodonta;Imparidentia;Venerida;Veneroidea;Veneridae;Mercenaria;Mercenaria mercenaria
UdeM00000018255 6596 100.5 UdeM00000018255 XP_045156670.1 33.7 163 107 1 43 205 541 702 2.70E-18 100.5 6596 cellular organisms;Eukaryota;Opisthokonta;Metazoa;Eumetazoa;Bilateria;Protostomia;Spiralia;Lophotrochozoa;Mollusca;Bivalvia;Autobranchia;Heteroconchia;Euheterodonta;Imparidentia;Venerida;Veneroidea;Veneridae;Mercenaria;Mercenaria mercenaria
UdeM00000018256 6596 292.7 UdeM00000018256 XP_045199387.1 30.6 722 447 18 6 704 5 695 1.20E-75 292.7 6596 cellular organisms;Eukaryota;Opisthokonta;Metazoa;Eumetazoa;Bilateria;Protostomia;Spiralia;Lophotrochozoa;Mollusca;Bivalvia;Autobranchia;Heteroconchia;Euheterodonta;Imparidentia;Venerida;Veneroidea;Veneridae;Mercenaria;Mercenaria mercenaria
UdeM00000018257 6596 138.7 UdeM00000018257 XP_045159530.1 38.8 165 100 1 5 169 226 389 1.30E-29 138.7 6596 cellular organisms;Eukaryota;Opisthokonta;Metazoa;Eumetazoa;Bilateria;Protostomia;Spiralia;Lophotrochozoa;Mollusca;Bivalvia;Autobranchia;Heteroconchia;Euheterodonta;Imparidentia;Venerida;Veneroidea;Veneridae;Mercenaria;Mercenaria mercenaria
UdeM00000018258 6596 82 UdeM00000018258 XP_045194475.1 39 118 72 0 4 121 3 120 8.00E-13 82 6596 cellular organisms;Eukaryota;Opisthokonta;Metazoa;Eumetazoa;Bilateria;Protostomia;Spiralia;Lophotrochozoa;Mollusca;Bivalvia;Autobranchia;Heteroconchia;Euheterodonta;Imparidentia;Venerida;Veneroidea;Veneridae;Mercenaria;Mercenaria mercenaria
UdeM00000018259 6596 122.5 UdeM00000018259 XP_045160626.1 31.5 257 168 3 87 343 442 690 1.10E-24 122.5 6596 cellular organisms;Eukaryota;Opisthokonta;Metazoa;Eumetazoa;Bilateria;Protostomia;Spiralia;Lophotrochozoa;Mollusca;Bivalvia;Autobranchia;Heteroconchia;Euheterodonta;Imparidentia;Venerida;Veneroidea;Veneridae;Mercenaria;Mercenaria mercenaria
UdeM00000018260 6596 290.8 UdeM00000018260 XP_045157317.1 30.2 725 455 14 4 704 3 700 4.70E-75 290.8 6596 cellular organisms;Eukaryota;Opisthokonta;Metazoa;Eumetazoa;Bilateria;Protostomia;Spiralia;Lophotrochozoa;Mollusca;Bivalvia;Autobranchia;Heteroconchia;Euheterodonta;Imparidentia;Venerida;Veneroidea;Veneridae;Mercenaria;Mercenaria mercenaria
UdeM00000018261 6596 291.2 UdeM00000018261 XP_045199387.1 30.7 720 462 11 6 716 5 696 3.60E-75 291.2 6596 cellular organisms;Eukaryota;Opisthokonta;Metazoa;Eumetazoa;Bilateria;Protostomia;Spiralia;Lophotrochozoa;Mollusca;Bivalvia;Autobranchia;Heteroconchia;Euhe

terodonta;Imparidentia;Venerida;Veneroidea;Veneridae;Mercenaria;Mercenari
a mercenaria
UdeM00000018262 6596 284.6 UdeM00000018262 XP_045199387.1 29.9 718
456 16 6 703 5 695 3.40E-73 284.6 6596 cellular
organisms;Eukaryota;Opisthokonta;Metazoa;Eumetazoa;Bilateria;Protostomia;
Spiralia;Lophotrochozoa;Mollusca;Bivalvia;Autobranchia;Heteroconchia;Euhe
terodonta;Imparidentia;Venerida;Veneroidea;Veneridae;Mercenaria;Mercenari
a mercenaria
UdeM00000018264 6596 290 UdeM00000018264 XP_045202537.1 64.8 386
122 8 1 380 1320 1697 6.20E-75 290 6596 cellular
organisms;Eukaryota;Opisthokonta;Metazoa;Eumetazoa;Bilateria;Protostomia;
Spiralia;Lophotrochozoa;Mollusca;Bivalvia;Autobranchia;Heteroconchia;Euhe
terodonta;Imparidentia;Venerida;Veneroidea;Veneridae;Mercenaria;Mercenari
a mercenaria
UdeM00000018263 6579 2232.6 UdeM00000018263 XP_033759998.1 65.3
1765 575 21 1 1752 1 1740 0.00E+00 2232.6 6579
cellular
organisms;Eukaryota;Opisthokonta;Metazoa;Eumetazoa;Bilateria;Protostomia;
Spiralia;Lophotrochozoa;Mollusca;Bivalvia;Autobranchia;Pteriomorphia;Pect
inida;Pectinoidea;Pectinidae;Pecten;Pecten maximus
UdeM00000018265 6579 237.7 UdeM00000018265 XP_033761214.1 52.8 216
101 1 1 215 1 216 1.40E-59 237.7 6579 cellular
organisms;Eukaryota;Opisthokonta;Metazoa;Eumetazoa;Bilateria;Protostomia;
Spiralia;Lophotrochozoa;Mollusca;Bivalvia;Autobranchia;Pteriomorphia;Pect
inida;Pectinoidea;Pectinidae;Pecten;Pecten maximus
UdeM00000018266 6596 147.5 UdeM00000018266 XP_045164042.1 55 131
58 1 178 308 107 236 2.80E-32 147.5 6596 cellular
organisms;Eukaryota;Opisthokonta;Metazoa;Eumetazoa;Bilateria;Protostomia;
Spiralia;Lophotrochozoa;Mollusca;Bivalvia;Autobranchia;Heteroconchia;Euhe
terodonta;Imparidentia;Venerida;Veneroidea;Veneridae;Mercenaria;Mercenari
a mercenaria
UdeM00000018267 6596 104.4 UdeM00000018267 XP_045157086.1 93.9 49
3 0 1 49 19 67 6.70E-20 104.4 6596 cellular
organisms;Eukaryota;Opisthokonta;Metazoa;Eumetazoa;Bilateria;Protostomia;
Spiralia;Lophotrochozoa;Mollusca;Bivalvia;Autobranchia;Heteroconchia;Euhe
terodonta;Imparidentia;Venerida;Veneroidea;Veneridae;Mercenaria;Mercenari
a mercenaria
UdeM00000018268 6596 443 UdeM00000018268 XP_045157086.1 55.9 442
135 4 1 440 76 459 4.60E-121 443 6596 cellular
organisms;Eukaryota;Opisthokonta;Metazoa;Eumetazoa;Bilateria;Protostomia;
Spiralia;Lophotrochozoa;Mollusca;Bivalvia;Autobranchia;Heteroconchia;Euhe
terodonta;Imparidentia;Venerida;Veneroidea;Veneridae;Mercenaria;Mercenari
a mercenaria
UdeM00000018269 6596 330.5 UdeM00000018269 XP_045157086.1 52.8 360
110 4 1 358 158 459 2.70E-87 330.5 6596 cellular
organisms;Eukaryota;Opisthokonta;Metazoa;Eumetazoa;Bilateria;Protostomia;
Spiralia;Lophotrochozoa;Mollusca;Bivalvia;Autobranchia;Heteroconchia;Euhe
terodonta;Imparidentia;Venerida;Veneroidea;Veneridae;Mercenaria;Mercenari
a mercenaria
UdeM00000018270 6596 506.5 UdeM00000018270 XP_045157086.1 53.7 495
190 8 1 492 1 459 3.80E-140 506.5 6596 cellular
organisms;Eukaryota;Opisthokonta;Metazoa;Eumetazoa;Bilateria;Protostomia;
Spiralia;Lophotrochozoa;Mollusca;Bivalvia;Autobranchia;Heteroconchia;Euhe

terodonta;Imparidentia;Venerida;Veneroidea;Veneridae;Mercenaria;Mercenaria mercenaria
UdeM00000018271 6500 113.2 UdeM00000018271 XP_012942302.1 30.3 304 190 5 5 286 7 310 5.50E-22 113.2 6500 cellular organisms;Eukaryota;Opisthokonta;Metazoa;Eumetazoa;Bilateria;Protostomia;Spiralia;Lophotrochozoa;Mollusca;Gastropoda;Heterobranchia;Euthyneura;Tectipleura;Aplysiida;Aplysioidea;Aplysiidae;Aplysia;Aplysia californica
UdeM00000018272 225164 376.3 UdeM00000018272 XP_009063422.1 45.2 440 226 7 38 475 1 427 5.90E-101 376.3 225164 cellular organisms;Eukaryota;Opisthokonta;Metazoa;Eumetazoa;Bilateria;Protostomia;Spiralia;Lophotrochozoa;Mollusca;Gastropoda;Patellogastropoda;Lottioidea;Lottiidae;Lottia;Lottia gigantea
UdeM00000018273 6596 160.2 UdeM00000018273 XP_045174734.1 32.8 299 179 8 8 297 11 296 6.00E-36 160.2 6596 cellular organisms;Eukaryota;Opisthokonta;Metazoa;Eumetazoa;Bilateria;Protostomia;Spiralia;Lophotrochozoa;Mollusca;Bivalvia;Autobranchia;Heteroconchia;Euheterodonta;Imparidentia;Venerida;Veneroidea;Veneridae;Mercenaria;Mercenaria mercenaria
UdeM00000018274 6573 78.6 UdeM00000018274 XP_021374915.1 33.7 98 65 0 8 105 15 112 7.00E-12 78.6 6573 cellular organisms;Eukaryota;Opisthokonta;Metazoa;Eumetazoa;Bilateria;Protostomia;Spiralia;Lophotrochozoa;Mollusca;Bivalvia;Autobranchia;Pteriomorphia;Pectinida;Pectinoidea;Pectinidae;Mizuhopecten;Mizuhopecten yessoensis
UdeM00000018275 6596 146.4 UdeM00000018275 XP_045201917.1 30.2 262 176 4 8 264 14 273 1.00E-31 146.4 6596 cellular organisms;Eukaryota;Opisthokonta;Metazoa;Eumetazoa;Bilateria;Protostomia;Spiralia;Lophotrochozoa;Mollusca;Bivalvia;Autobranchia;Heteroconchia;Euheterodonta;Imparidentia;Venerida;Veneroidea;Veneridae;Mercenaria;Mercenaria mercenaria
UdeM00000018276 6596 146.7 UdeM00000018276 XP_045174734.1 28.6 350 199 10 1 333 4 319 5.60E-32 146.7 6596 cellular organisms;Eukaryota;Opisthokonta;Metazoa;Eumetazoa;Bilateria;Protostomia;Spiralia;Lophotrochozoa;Mollusca;Bivalvia;Autobranchia;Heteroconchia;Euheterodonta;Imparidentia;Venerida;Veneroidea;Veneridae;Mercenaria;Mercenaria mercenaria
UdeM00000018277 29159 57.8 UdeM00000018277 XP_034339171.1 34.1 91 57 2 1 91 653 740 9.50E-06 57.8 29159 cellular organisms;Eukaryota;Opisthokonta;Metazoa;Eumetazoa;Bilateria;Protostomia;Spiralia;Lophotrochozoa;Mollusca;Bivalvia;Autobranchia;Pteriomorphia;Ostreida;Ostreoidea;Ostreidae;Crassostrea;Crassostrea gigas
UdeM00000018278 6596 158.3 UdeM00000018278 XP_045174734.1 30 307 187 6 8 303 11 300 2.30E-35 158.3 6596 cellular organisms;Eukaryota;Opisthokonta;Metazoa;Eumetazoa;Bilateria;Protostomia;Spiralia;Lophotrochozoa;Mollusca;Bivalvia;Autobranchia;Heteroconchia;Euheterodonta;Imparidentia;Venerida;Veneroidea;Veneridae;Mercenaria;Mercenaria mercenaria
UdeM00000018279 7955 81.6 UdeM00000018279 NP_001092898.1 33.7 163 83 6 1 139 1 162 2.30E-12 81.6 7955 cellular organisms;Eukaryota;Opisthokonta;Metazoa;Eumetazoa;Bilateria;Deuterostomia;Chordata;Craniata;Vertebrata;Gnathostomata;Teleostomi;Euteleostomi;Actinopterygii;Actinopteri;Neopterygii;Teleostei;Osteoglossocephalai;Clupeocephala;Otomorpha;Ostariophysi;Otophysi;Cypriniphysae;Cypriniformes;Cyprinoidei;Danionidae;Danioninae;Danio;Danio rerio

UdeM00000018280 6596 151.4 UdeM00000018280 XP_045174734.1 29.2 305
205 4 1 302 4 300 2.70E-33 151.4 6596 cellular
organisms;Eukaryota;Opisthokonta;Metazoa;Eumetazoa;Bilateria;Protostomia;
Spiralia;Lophotrochozoa;Mollusca;Bivalvia;Autobranchia;Heteroconchia;Euheterodonta;Imparidentia;Venerida;Veneroidea;Veneridae;Mercenaria;Mercenaria mercenaria
UdeM00000018281 6596 151.4 UdeM00000018281 XP_045174734.1 29.2 305
205 4 1 302 4 300 2.70E-33 151.4 6596 cellular
organisms;Eukaryota;Opisthokonta;Metazoa;Eumetazoa;Bilateria;Protostomia;
Spiralia;Lophotrochozoa;Mollusca;Bivalvia;Autobranchia;Heteroconchia;Euheterodonta;Imparidentia;Venerida;Veneroidea;Veneridae;Mercenaria;Mercenaria mercenaria
UdeM00000018282 6596 171 UdeM00000018282 XP_045201090.1 61.9 139
51 2 1 137 1 139 1.00E-39 171 6596 cellular
organisms;Eukaryota;Opisthokonta;Metazoa;Eumetazoa;Bilateria;Protostomia;
Spiralia;Lophotrochozoa;Mollusca;Bivalvia;Autobranchia;Heteroconchia;Euheterodonta;Imparidentia;Venerida;Veneroidea;Veneridae;Mercenaria;Mercenaria mercenaria
UdeM00000018284 1735272 98.6 UdeM00000018284 XP_041349006.1 39.5
114 69 0 358 471 46 159 2.30E-17 98.6 1735272
cellular
organisms;Eukaryota;Opisthokonta;Metazoa;Eumetazoa;Bilateria;Protostomia;
Spiralia;Lophotrochozoa;Mollusca;Gastropoda;Neomphalina;Neomphaloidea;Peltospiridae;Gigantopelta;Gigantopelta aegis
UdeM00000018285 6596 547 UdeM00000018285 XP_045201547.1 43.6 615
332 7 59 668 48 652 3.90E-152 547 6596 cellular
organisms;Eukaryota;Opisthokonta;Metazoa;Eumetazoa;Bilateria;Protostomia;
Spiralia;Lophotrochozoa;Mollusca;Bivalvia;Autobranchia;Heteroconchia;Euheterodonta;Imparidentia;Venerida;Veneroidea;Veneridae;Mercenaria;Mercenaria mercenaria
UdeM00000018286 29159 65.5 UdeM00000018286 XP_011412090.2 41 83
34 2 1 69 222 303 6.40E-08 65.5 29159 cellular
organisms;Eukaryota;Opisthokonta;Metazoa;Eumetazoa;Bilateria;Protostomia;
Spiralia;Lophotrochozoa;Mollusca;Bivalvia;Autobranchia;Pteriomorphia;Ostreida;Ostreoidea;Ostreidae;Crassostrea;Crassostrea gigas
UdeM00000018287 6596 305.1 UdeM00000018287 XP_045200551.1 69.1 207
64 0 11 217 1 207 8.10E-80 305.1 6596 cellular
organisms;Eukaryota;Opisthokonta;Metazoa;Eumetazoa;Bilateria;Protostomia;
Spiralia;Lophotrochozoa;Mollusca;Bivalvia;Autobranchia;Heteroconchia;Euheterodonta;Imparidentia;Venerida;Veneroidea;Veneridae;Mercenaria;Mercenaria mercenaria
UdeM00000018288 1735272 198.7 UdeM00000018288 XP_041357818.1 48.4
219 98 4 4 220 2 207 7.50E-48 198.7 1735272
cellular
organisms;Eukaryota;Opisthokonta;Metazoa;Eumetazoa;Bilateria;Protostomia;
Spiralia;Lophotrochozoa;Mollusca;Gastropoda;Neomphalina;Neomphaloidea;Peltospiridae;Gigantopelta;Gigantopelta aegis
UdeM00000018289 6573 470.7 UdeM00000018289 XP_021377752.1 32.7 887
543 9 4 867 1 856 4.00E-129 470.7 6573 cellular
organisms;Eukaryota;Opisthokonta;Metazoa;Eumetazoa;Bilateria;Protostomia;
Spiralia;Lophotrochozoa;Mollusca;Bivalvia;Autobranchia;Pteriomorphia;Pectinida;Pectinoidea;Pectinidae;Mizuhopecten;Mizuhopecten yessoensis
UdeM00000018291 6579 475.3 UdeM00000018291 XP_033730327.1 69.5 361
110 0 1 361 19 379 7.10E-131 475.3 6579 cellular

organisms;Eukaryota;Opisthokonta;Metazoa;Eumetazoa;Bilateria;Protostomia;Spiralia;Lophotrochozoa;Mollusca;Bivalvia;Autobranchia;Pteriomorphia;Pectinida;Pectinoidea;Pectinidae;Pecten;Pecten maximus
UdeM00000018290 6579 486.1 UdeM00000018290 XP_033730327.1 69.4 369 113 0 40 408 11 379 4.50E-134 486.1 6579 cellular organisms;Eukaryota;Opisthokonta;Metazoa;Eumetazoa;Bilateria;Protostomia;Spiralia;Lophotrochozoa;Mollusca;Bivalvia;Autobranchia;Pteriomorphia;Pectinida;Pectinoidea;Pectinidae;Pecten;Pecten maximus
UdeM00000018292 6526 213.8 UdeM00000018292 XP_013067574.1 82.8 122 21 0 4 125 2 123 1.30E-52 213.8 6526 cellular organisms;Eukaryota;Opisthokonta;Metazoa;Eumetazoa;Bilateria;Protostomia;Spiralia;Lophotrochozoa;Mollusca;Gastropoda;Heterobranchia;Euthyneura;Panpulmonata;Hygrophila;Lymnaeoidea;Planorbidae;Biomphalaria;Biomphalaria glabrata
UdeM00000018293 1735272 169.9 UdeM00000018293 XP_041371917.1 39 336 183 7 55 383 99 419 6.60E-39 169.9 1735272 cellular organisms;Eukaryota;Opisthokonta;Metazoa;Eumetazoa;Bilateria;Protostomia;Spiralia;Lophotrochozoa;Mollusca;Gastropoda;Neomphalina;Neomphaloidea;Peltospiridae;Gigantopelta;Gigantopelta aegis
UdeM00000018294 7668 120.9 UdeM00000018294 XP_003729577.2 32.5 209 88 2 9 168 712 916 1.60E-24 120.9 7668 cellular organisms;Eukaryota;Opisthokonta;Metazoa;Eumetazoa;Bilateria;Deuterostomia;Echinodermata;Eleutherozoa;Echinozoa;Echinoidea;Euechinoidea;Echinacea;Camarodonta;Echinidea;Strongylocentrotidae;Strongylocentrotus;Strongylocentrotus purpuratus
UdeM00000018295 37621 96.3 UdeM00000018295 XP_014678940.1 61.4 70 27 0 1 70 499 568 2.10E-17 96.3 37621 cellular organisms;Eukaryota;Opisthokonta;Metazoa;Eumetazoa;Bilateria;Protostomia;Ecdysozoa;Scalidophora;Priapulida;Priapulimorpha;Priapulimorphida;Priapulidae;Priapulus;Priapulus caudatus
UdeM00000018296 37621 98.2 UdeM00000018296 XP_014678940.1 53.3 92 42 1 1 92 499 589 8.30E-18 98.2 37621 cellular organisms;Eukaryota;Opisthokonta;Metazoa;Eumetazoa;Bilateria;Protostomia;Ecdysozoa;Scalidophora;Priapulida;Priapulimorpha;Priapulimorphida;Priapulidae;Priapulus;Priapulus caudatus
UdeM00000018297 37621 104 UdeM00000018297 XP_014673981.1 63.2 76 28 0 1 76 76 151 8.70E-20 104 37621 cellular organisms;Eukaryota;Opisthokonta;Metazoa;Eumetazoa;Bilateria;Protostomia;Ecdysozoa;Scalidophora;Priapulida;Priapulimorpha;Priapulimorphida;Priapulidae;Priapulus;Priapulus caudatus
UdeM00000018298 6596 192.2 UdeM00000018298 XP_045202462.1 40.6 286 150 7 1 271 151 431 8.70E-46 192.2 6596 cellular organisms;Eukaryota;Opisthokonta;Metazoa;Eumetazoa;Bilateria;Protostomia;Spiralia;Lophotrochozoa;Mollusca;Bivalvia;Autobranchia;Heteroconchia;Euheterodonta;Imparidentia;Venerida;Veneroidea;Veneridae;Mercenaria;Mercenaria mercenaria
UdeM00000018299 29159 153.7 UdeM00000018299 XP_034320422.1 71.7 92 26 0 28 119 133 224 1.70E-34 153.7 29159 cellular organisms;Eukaryota;Opisthokonta;Metazoa;Eumetazoa;Bilateria;Protostomia;Spiralia;Lophotrochozoa;Mollusca;Bivalvia;Autobranchia;Pteriomorphia;Ostreida;Ostreoidea;Ostreidae;Crassostrea;Crassostrea gigas
UdeM00000018300 6573 124 UdeM00000018300 XP_021355633.1 48.5 130 66 1 9 137 11 140 2.90E-25 124 6573 cellular

organisms;Eukaryota;Opisthokonta;Metazoa;Eumetazoa;Bilateria;Protostomia;
Spiralia;Lophotrochozoa;Mollusca;Bivalvia;Autobranchia;Pteriomorphia;Pectinida;Pectinoidea;Pectinidae;Mizuhopecten;Mizuhopecten yessoensis
UdeM00000018301 6573 124 UdeM00000018301 XP_021355633.1 48.5 130 66 1 9 137 11 140 2.90E-25 124 6573 cellular organisms;Eukaryota;Opisthokonta;Metazoa;Eumetazoa;Bilateria;Protostomia;Spiralia;Lophotrochozoa;Mollusca;Bivalvia;Autobranchia;Pteriomorphia;Pectinida;Pectinoidea;Pectinidae;Mizuhopecten;Mizuhopecten yessoensis
UdeM00000018302 1735272 73.6 UdeM00000018302 XP_041358777.1 27 230 150 6 1 220 1 222 6.10E-10 73.6 1735272 cellular organisms;Eukaryota;Opisthokonta;Metazoa;Eumetazoa;Bilateria;Protostomia;Spiralia;Lophotrochozoa;Mollusca;Gastropoda;Neomphalina;Neomphaloidea;Peltospiridae;Gigantopelta;Gigantopelta aegis
UdeM00000018303 1735272 172.6 UdeM00000018303 XP_041356772.1 29.1 385 251 7 24 402 39 407 1.10E-39 172.6 1735272 cellular organisms;Eukaryota;Opisthokonta;Metazoa;Eumetazoa;Bilateria;Protostomia;Spiralia;Lophotrochozoa;Mollusca;Gastropoda;Neomphalina;Neomphaloidea;Peltospiridae;Gigantopelta;Gigantopelta aegis
UdeM00000018304 6565 282.7 UdeM00000018304 XP_022290234.1 53.6 248 109 3 26 273 44 285 5.00E-73 282.7 6565 cellular organisms;Eukaryota;Opisthokonta;Metazoa;Eumetazoa;Bilateria;Protostomia;Spiralia;Lophotrochozoa;Mollusca;Bivalvia;Autobranchia;Pteriomorphia;Ostreida;Ostreoidea;Ostreidae;Crassostrea;Crassostrea virginica
UdeM00000018305 6596 427.2 UdeM00000018305 XP_045201850.1 29.1 1103 695 37 67 1123 7 1068 6.90E-116 427.2 6596 cellular organisms;Eukaryota;Opisthokonta;Metazoa;Eumetazoa;Bilateria;Protostomia;Spiralia;Lophotrochozoa;Mollusca;Bivalvia;Autobranchia;Heteroconchia;Euheterodonta;Imparidentia;Venerida;Veneroidea;Veneridae;Mercenaria;Mercenaria mercenaria
UdeM00000018306 6573 414.1 UdeM00000018306 XP_021349077.1 40.5 560 322 3 11 560 96 654 2.90E-112 414.1 6573 cellular organisms;Eukaryota;Opisthokonta;Metazoa;Eumetazoa;Bilateria;Protostomia;Spiralia;Lophotrochozoa;Mollusca;Bivalvia;Autobranchia;Pteriomorphia;Pectinida;Pectinoidea;Pectinidae;Mizuhopecten;Mizuhopecten yessoensis
UdeM00000018307 6573 413.3 UdeM00000018307 XP_021371609.1 47.4 454 224 1 12 450 14 467 4.00E-112 413.3 6573 cellular organisms;Eukaryota;Opisthokonta;Metazoa;Eumetazoa;Bilateria;Protostomia;Spiralia;Lophotrochozoa;Mollusca;Bivalvia;Autobranchia;Pteriomorphia;Pectinida;Pectinoidea;Pectinidae;Mizuhopecten;Mizuhopecten yessoensis
UdeM00000018308 6573 206.5 UdeM00000018308 XP_021349207.1 36.3 325 189 9 68 380 101 419 6.30E-50 206.5 6573 cellular organisms;Eukaryota;Opisthokonta;Metazoa;Eumetazoa;Bilateria;Protostomia;Spiralia;Lophotrochozoa;Mollusca;Bivalvia;Autobranchia;Pteriomorphia;Pectinida;Pectinoidea;Pectinidae;Mizuhopecten;Mizuhopecten yessoensis
UdeM00000018309 6573 108.2 UdeM00000018309 XP_021371608.1 54.3 105 48 0 12 116 14 118 7.10E-21 108.2 6573 cellular organisms;Eukaryota;Opisthokonta;Metazoa;Eumetazoa;Bilateria;Protostomia;Spiralia;Lophotrochozoa;Mollusca;Bivalvia;Autobranchia;Pteriomorphia;Pectinida;Pectinoidea;Pectinidae;Mizuhopecten;Mizuhopecten yessoensis
UdeM00000018310 29159 207.2 UdeM00000018310 XP_034332822.1 39 310 163 8 100 400 119 411 4.00E-50 207.2 29159 cellular organisms;Eukaryota;Opisthokonta;Metazoa;Eumetazoa;Bilateria;Protostomia;

Spiralia;Lophotrochozoa;Mollusca;Bivalvia;Autobranchia;Pteriomorphia;Ostreida;Ostreoidea;Ostreidae;Crassostrea;Crassostrea gigas
UdeM00000018311 1735272 323.9 UdeM00000018311 XP_041374187.1 48.1 349 158 9 1 335 1 340 2.40E-85 323.9 1735272 cellular organisms;Eukaryota;Opisthokonta;Metazoa;Eumetazoa;Bilateria;Protostomia;Spiralia;Lophotrochozoa;Mollusca;Gastropoda;Neomphalina;Neomphaloidea;Peltospiridae;Gigantopelta;Gigantopelta aegis
UdeM00000018312 6579 164.1 UdeM00000018312 XP_033747958.1 55.9 127 56 0 8 134 18 144 1.40E-37 164.1 6579 cellular organisms;Eukaryota;Opisthokonta;Metazoa;Eumetazoa;Bilateria;Protostomia;Spiralia;Lophotrochozoa;Mollusca;Bivalvia;Autobranchia;Pteriomorphia;Pectinida;Pectinoidea;Pectinidae;Pecten;Pecten maximus
UdeM00000018313 6579 164.1 UdeM00000018313 XP_033747958.1 55.9 127 56 0 24 150 18 144 1.60E-37 164.1 6579 cellular organisms;Eukaryota;Opisthokonta;Metazoa;Eumetazoa;Bilateria;Protostomia;Spiralia;Lophotrochozoa;Mollusca;Bivalvia;Autobranchia;Pteriomorphia;Pectinida;Pectinoidea;Pectinidae;Pecten;Pecten maximus
UdeM00000018314 6565 205.7 UdeM00000018314 XP_022294078.1 39.9 296 168 6 100 388 147 439 1.10E-49 205.7 6565 cellular organisms;Eukaryota;Opisthokonta;Metazoa;Eumetazoa;Bilateria;Protostomia;Spiralia;Lophotrochozoa;Mollusca;Bivalvia;Autobranchia;Pteriomorphia;Ostreida;Ostreoidea;Ostreidae;Crassostrea;Crassostrea virginica
UdeM00000018315 6596 479.2 UdeM00000018315 XP_045201087.1 53 432 197 5 1 430 1 428 5.60E-132 479.2 6596 cellular organisms;Eukaryota;Opisthokonta;Metazoa;Eumetazoa;Bilateria;Protostomia;Spiralia;Lophotrochozoa;Mollusca;Bivalvia;Autobranchia;Heteroconchia;Euheterodonta;Imparidentia;Venerida;Veneroidea;Veneridae;Mercenaria;Mercenaria mercenaria
UdeM00000018316 6596 410.6 UdeM00000018316 XP_045202300.1 35 738 454 12 1 726 1 724 4.10E-111 410.6 6596 cellular organisms;Eukaryota;Opisthokonta;Metazoa;Eumetazoa;Bilateria;Protostomia;Spiralia;Lophotrochozoa;Mollusca;Bivalvia;Autobranchia;Heteroconchia;Euheterodonta;Imparidentia;Venerida;Veneroidea;Veneridae;Mercenaria;Mercenaria mercenaria
UdeM00000018317 29159 212.6 UdeM00000018317 XP_034329879.1 62.3 167 59 2 1 167 3 165 3.80E-52 212.6 29159 cellular organisms;Eukaryota;Opisthokonta;Metazoa;Eumetazoa;Bilateria;Protostomia;Spiralia;Lophotrochozoa;Mollusca;Bivalvia;Autobranchia;Pteriomorphia;Ostreida;Ostreoidea;Ostreidae;Crassostrea;Crassostrea gigas
UdeM00000018318 6596 544.3 UdeM00000018318 XP_045200175.1 52.9 1052 361 24 1 1010 1 959 3.40E-151 544.3 6596 cellular organisms;Eukaryota;Opisthokonta;Metazoa;Eumetazoa;Bilateria;Protostomia;Spiralia;Lophotrochozoa;Mollusca;Bivalvia;Autobranchia;Heteroconchia;Euheterodonta;Imparidentia;Venerida;Veneroidea;Veneridae;Mercenaria;Mercenaria mercenaria
UdeM00000018319 6596 278.9 UdeM00000018319 XP_045200175.1 58.9 246 84 7 12 245 964 1204 6.40E-72 278.9 6596 cellular organisms;Eukaryota;Opisthokonta;Metazoa;Eumetazoa;Bilateria;Protostomia;Spiralia;Lophotrochozoa;Mollusca;Bivalvia;Autobranchia;Heteroconchia;Euheterodonta;Imparidentia;Venerida;Veneroidea;Veneridae;Mercenaria;Mercenaria mercenaria
UdeM00000018320 6579 435.3 UdeM00000018320 XP_033759836.1 45.6 509 265 4 24 523 108 613 1.10E-118 435.3 6579 cellular

organisms;Eukaryota;Opisthokonta;Metazoa;Eumetazoa;Bilateria;Protostomia;
Spiralia;Lophotrochozoa;Mollusca;Bivalvia;Autobranchia;Pteriomorphia;Pect
inida;Pectinoidea;Pectinidae;Pecten;Pecten maximus
UdeM00000018321 32264 112.8 UdeM00000018321 XP_015783371.1 26.6 364
227 15 224 570 367 707 1.60E-21 112.8 32264 cellular
organisms;Eukaryota;Opisthokonta;Metazoa;Eumetazoa;Bilateria;Protostomia;
Ecdysozoa;Panarthropoda;Arthropoda;Chelicerata;Arachnida;Acari;Acariforme
s;Trombidiformes;Prostigmata;Eleutherengona;Raphignathae;Tetranychoidea;T
etranychidae;Tetranychus;Tetranychus urticae
UdeM00000018322 6579 79 UdeM00000018322 XP_033762031.1 32.4 142
94 2 5 144 384 525 1.00E-11 79 6579 cellular
organisms;Eukaryota;Opisthokonta;Metazoa;Eumetazoa;Bilateria;Protostomia;
Spiralia;Lophotrochozoa;Mollusca;Bivalvia;Autobranchia;Pteriomorphia;Pect
inida;Pectinoidea;Pectinidae;Pecten;Pecten maximus
UdeM00000018323 6596 212.6 UdeM00000018323 XP_045182620.1 39.5 299
170 6 97 389 90 383 9.10E-52 212.6 6596 cellular
organisms;Eukaryota;Opisthokonta;Metazoa;Eumetazoa;Bilateria;Protostomia;
Spiralia;Lophotrochozoa;Mollusca;Bivalvia;Autobranchia;Heteroconchia;Euhe
terodonta;Imparidentia;Venerida;Veneroidea;Veneridae;Mercenaria;Mercenari
a mercenaria
UdeM00000018326 6596 403.7 UdeM00000018326 XP_045170750.1 65.7 283
90 2 7 289 6 281 2.00E-109 403.7 6596 cellular
organisms;Eukaryota;Opisthokonta;Metazoa;Eumetazoa;Bilateria;Protostomia;
Spiralia;Lophotrochozoa;Mollusca;Bivalvia;Autobranchia;Heteroconchia;Euhe
terodonta;Imparidentia;Venerida;Veneroidea;Veneridae;Mercenaria;Mercenari
a mercenaria
UdeM00000018325 6596 512.7 UdeM00000018325 XP_045170750.1 66.1 366
116 3 7 372 6 363 4.00E-142 512.7 6596 cellular
organisms;Eukaryota;Opisthokonta;Metazoa;Eumetazoa;Bilateria;Protostomia;
Spiralia;Lophotrochozoa;Mollusca;Bivalvia;Autobranchia;Heteroconchia;Euhe
terodonta;Imparidentia;Venerida;Veneroidea;Veneridae;Mercenaria;Mercenari
a mercenaria
UdeM00000018327 6596 213 UdeM00000018327 XP_045192227.1 39.7 290
162 6 67 354 159 437 6.20E-52 213 6596 cellular
organisms;Eukaryota;Opisthokonta;Metazoa;Eumetazoa;Bilateria;Protostomia;
Spiralia;Lophotrochozoa;Mollusca;Bivalvia;Autobranchia;Heteroconchia;Euhe
terodonta;Imparidentia;Venerida;Veneroidea;Veneridae;Mercenaria;Mercenari
a mercenaria
UdeM00000018328 6596 204.5 UdeM00000018328 XP_045170750.1 66.7 135
45 0 1 135 143 277 9.30E-50 204.5 6596 cellular
organisms;Eukaryota;Opisthokonta;Metazoa;Eumetazoa;Bilateria;Protostomia;
Spiralia;Lophotrochozoa;Mollusca;Bivalvia;Autobranchia;Heteroconchia;Euhe
terodonta;Imparidentia;Venerida;Veneroidea;Veneridae;Mercenaria;Mercenari
a mercenaria
UdeM00000018329 6596 499.6 UdeM00000018329 XP_045170750.1 63.9 371
119 3 9 379 8 363 3.50E-138 499.6 6596 cellular
organisms;Eukaryota;Opisthokonta;Metazoa;Eumetazoa;Bilateria;Protostomia;
Spiralia;Lophotrochozoa;Mollusca;Bivalvia;Autobranchia;Heteroconchia;Euhe
terodonta;Imparidentia;Venerida;Veneroidea;Veneridae;Mercenaria;Mercenari
a mercenaria
UdeM00000018330 400727 176.4 UdeM00000018330 XP_025113942.1 73
111 30 0 1 111 1 111 2.10E-41 176.4 400727
cellular
organisms;Eukaryota;Opisthokonta;Metazoa;Eumetazoa;Bilateria;Protostomia;

Spiralia;Lophotrochozoa;Mollusca;Gastropoda;Caenogastropoda;Architaenioglossa;Ampullarioidea;Ampullariidae;Pomacea;Pomacea canaliculata
UdeM00000018333 2652724 68.2 UdeM00000018333 XP_020900815.1 54.7 53 24 0 1 53 698 750 9.70E-09 68.2 2652724 cellular organisms;Eukaryota;Opisthokonta;Metazoa;Eumetazoa;Cnidaria;Anthozoa;Hexacorallia;Actiniaria;Aiptasiidae;Exaiptasia;Exaiptasia diaphana
UdeM00000018335 6596 382.1 UdeM00000018335 XP_045201453.1 50.6 389 171 11 23 403 7 382 8.80E-103 382.1 6596 cellular organisms;Eukaryota;Opisthokonta;Metazoa;Eumetazoa;Bilateria;Protostomia;Spiralia;Lophotrochozoa;Mollusca;Bivalvia;Autobranchia;Heteroconchia;Euheterodonta;Imparidentia;Venerida;Veneroidea;Veneridae;Mercenaria;Mercenaria mercenaria
UdeM00000018334 6596 381.7 UdeM00000018334 XP_045201453.1 50.6 389 171 11 28 408 7 382 1.20E-102 381.7 6596 cellular organisms;Eukaryota;Opisthokonta;Metazoa;Eumetazoa;Bilateria;Protostomia;Spiralia;Lophotrochozoa;Mollusca;Bivalvia;Autobranchia;Heteroconchia;Euheterodonta;Imparidentia;Venerida;Veneroidea;Veneridae;Mercenaria;Mercenaria mercenaria
UdeM00000018336 6596 414.8 UdeM00000018336 XP_045199319.1 80.5 226 44 0 1567 1792 1533 1758 5.40E-112 414.8 6596 cellular organisms;Eukaryota;Opisthokonta;Metazoa;Eumetazoa;Bilateria;Protostomia;Spiralia;Lophotrochozoa;Mollusca;Bivalvia;Autobranchia;Heteroconchia;Euheterodonta;Imparidentia;Venerida;Veneroidea;Veneridae;Mercenaria;Mercenaria mercenaria
UdeM00000018338 1735272 197.2 UdeM00000018338 XP_041362472.1 41.4 251 141 3 263 507 68 318 5.70E-47 197.2 1735272 cellular organisms;Eukaryota;Opisthokonta;Metazoa;Eumetazoa;Bilateria;Protostomia;Spiralia;Lophotrochozoa;Mollusca;Gastropoda;Neomphalina;Neomphaloidea;Peltospiridae;Gigantopelta;Gigantopelta aegis
UdeM00000018339 6596 395.2 UdeM00000018339 XP_045199317.1 78.9 223 47 0 100 322 1539 1761 8.00E-107 395.2 6596 cellular organisms;Eukaryota;Opisthokonta;Metazoa;Eumetazoa;Bilateria;Protostomia;Spiralia;Lophotrochozoa;Mollusca;Bivalvia;Autobranchia;Heteroconchia;Euheterodonta;Imparidentia;Venerida;Veneroidea;Veneridae;Mercenaria;Mercenaria mercenaria
UdeM00000018340 29159 118.6 UdeM00000018340 XP_011419183.2 30.5 200 126 2 26 221 77 267 2.00E-23 118.6 29159 cellular organisms;Eukaryota;Opisthokonta;Metazoa;Eumetazoa;Bilateria;Protostomia;Spiralia;Lophotrochozoa;Mollusca;Bivalvia;Autobranchia;Pteriomorphia;Ostreida;Ostreoidea;Ostreidae;Crassostrea;Crassostrea gigas
UdeM00000018341 6579 134.8 UdeM00000018341 XP_033750238.1 29.5 217 141 3 4 219 52 257 1.30E-28 134.8 6579 cellular organisms;Eukaryota;Opisthokonta;Metazoa;Eumetazoa;Bilateria;Protostomia;Spiralia;Lophotrochozoa;Mollusca;Bivalvia;Autobranchia;Pteriomorphia;Pectinida;Pectinoidea;Pectinidae;Pecten;Pecten maximus
UdeM00000018342 6579 139.4 UdeM00000018342 XP_033750238.1 30.5 220 141 3 1 219 49 257 5.40E-30 139.4 6579 cellular organisms;Eukaryota;Opisthokonta;Metazoa;Eumetazoa;Bilateria;Protostomia;Spiralia;Lophotrochozoa;Mollusca;Bivalvia;Autobranchia;Pteriomorphia;Pectinida;Pectinoidea;Pectinidae;Pecten;Pecten maximus
UdeM00000018343 6579 143.3 UdeM00000018343 XP_033750238.1 30.9 223 142 3 45 266 46 257 4.50E-31 143.3 6579 cellular

organisms;Eukaryota;Opisthokonta;Metazoa;Eumetazoa;Bilateria;Protostomia;Spiralia;Lophotrochozoa;Mollusca;Bivalvia;Autobranchia;Pteriomorphia;Pectinida;Pectinoidea;Pectinidae;Pecten;Pecten maximus
UdeM00000018347 6596 377.1 UdeM00000018347 XP_045201156.1 31.3 860
524 16 1 836 32 848 5.80E-101 377.1 6596 cellular
organisms;Eukaryota;Opisthokonta;Metazoa;Eumetazoa;Bilateria;Protostomia;Spiralia;Lophotrochozoa;Mollusca;Bivalvia;Autobranchia;Heteroconchia;Euheterodonta;Imparidentia;Venerida;Veneroidea;Veneridae;Mercenaria;Mercenaria mercenaria
UdeM00000018348 6596 1453 UdeM00000018348 XP_045202557.1 52.7 1422
556 23 1 1379 1 1348 0.00E+00 1453 6596 cellular
organisms;Eukaryota;Opisthokonta;Metazoa;Eumetazoa;Bilateria;Protostomia;Spiralia;Lophotrochozoa;Mollusca;Bivalvia;Autobranchia;Heteroconchia;Euheterodonta;Imparidentia;Venerida;Veneroidea;Veneridae;Mercenaria;Mercenaria mercenaria
UdeM00000018349 6706 150.2 UdeM00000018349 XP_042235765.1 43.3 157
89 0 1 157 1 157 2.20E-33 150.2 6706 cellular
organisms;Eukaryota;Opisthokonta;Metazoa;Eumetazoa;Bilateria;Protostomia;Ecdysozoa;Panarthropoda;Arthropoda;Mandibulata;Pancrustacea;Crustacea;Multicrustacea;Malacostraca;Eumalacostraca;Eucarida;Decapoda;Pleocyemata;Astacidea;Nephropoidea;Nephropidae;Homarus;Homarus americanus
UdeM00000018350 6573 356.3 UdeM00000018350 XP_021359188.1 64.4 253
87 1 1 253 1 250 3.50E-95 356.3 6573 cellular
organisms;Eukaryota;Opisthokonta;Metazoa;Eumetazoa;Bilateria;Protostomia;Spiralia;Lophotrochozoa;Mollusca;Bivalvia;Autobranchia;Pteriomorphia;Pectinida;Pectinoidea;Pectinidae;Mizuhopecten;Mizuhopecten yessoensis
UdeM00000018351 2607531 543.5 UdeM00000018351 XP_029642003.1 50.2
528 258 4 1 526 112 636 3.00E-151 543.5 2607531
cellular
organisms;Eukaryota;Opisthokonta;Metazoa;Eumetazoa;Bilateria;Protostomia;Spiralia;Lophotrochozoa;Mollusca;Cephalopoda;Coleoidea;Octopodiformes;Octopoda;Incirrata;Octopodidae;Octopus;Octopus sinensis
UdeM00000044396 6573 98.6 UdeM00000044396 XP_021364275.1 50.4 121
52 3 161 277 279 395 1.30E-17 98.6 6573 cellular
organisms;Eukaryota;Opisthokonta;Metazoa;Eumetazoa;Bilateria;Protostomia;Spiralia;Lophotrochozoa;Mollusca;Bivalvia;Autobranchia;Pteriomorphia;Pectinida;Pectinoidea;Pectinidae;Mizuhopecten;Mizuhopecten yessoensis
UdeM00000019423 29159 70.9 UdeM00000019423 XP_011412814.1 40.2 102
57 2 5 105 9 107 1.10E-09 70.9 29159 cellular
organisms;Eukaryota;Opisthokonta;Metazoa;Eumetazoa;Bilateria;Protostomia;Spiralia;Lophotrochozoa;Mollusca;Bivalvia;Autobranchia;Pteriomorphia;Ostreida;Ostreoidea;Ostreidae;Crassostrea;Crassostrea gigas
UdeM00000019424 29159 316.6 UdeM00000019424 XP_034317111.1 55.8 283
124 1 2 283 171 453 3.20E-83 316.6 29159 cellular
organisms;Eukaryota;Opisthokonta;Metazoa;Eumetazoa;Bilateria;Protostomia;Spiralia;Lophotrochozoa;Mollusca;Bivalvia;Autobranchia;Pteriomorphia;Ostreida;Ostreoidea;Ostreidae;Crassostrea;Crassostrea gigas
UdeM00000019425 2652724 58.9 UdeM00000019425 XP_020895639.1 29.2
89 63 0 79 167 73 161 7.20E-06 58.9 2652724
cellular
organisms;Eukaryota;Opisthokonta;Metazoa;Eumetazoa;Cnidaria;Anthozoa;Hexacorallia;Actiniaria;Aiptasiidae;Exaiptasia;Exaiptasia diaphana
UdeM00000019426 7574 234.6 UdeM00000019426 XP_013384628.1 47.5 276
119 4 1 260 33 298 1.50E-58 234.6 7574 cellular

organisms;Eukaryota;Opisthokonta;Metazoa;Eumetazoa;Bilateria;Protostomia;Spiralia;Lophotrochozoa;Brachiopoda;Linguliformea;Lingulata;Lingulida;Linguloidea;Lingulidae;Lingula;Lingula anatina
UdeM00000019427 7574 604.7 UdeM00000019427 XP_013382110.1 48.4 603 286 8 1 602 37 615 1.20E-169 604.7 7574 cellular organisms;Eukaryota;Opisthokonta;Metazoa;Eumetazoa;Bilateria;Protostomia;Spiralia;Lophotrochozoa;Brachiopoda;Linguliformea;Lingulata;Lingulida;Linguloidea;Lingulidae;Lingula;Lingula anatina
UdeM00000019428 8218 66.6 UdeM00000019428 XP_034081603.1 32.6 138 84 3 63 195 158 291 4.30E-08 66.6 8218 cellular organisms;Eukaryota;Opisthokonta;Metazoa;Eumetazoa;Bilateria;Deuterostomia;Chordata;Craniata;Vertebrata;Gnathostomata;Teleostomi;Euteleostomi;Actinopterygii;Actinopteri;Neopterygii;Teleostei;Osteoglossocephalai;Clupeocephala;Euteleosteomorpha;Neoteleostei;Eurypterygia;Ctenosquamata;Acanthomorphata;Euacanthomorphacea;Percomorphaceae;Eupercaria;Perciformes;Notothenioidei;Bathydraconidae;Gymnodraco;Gymnodraco acuticeps
UdeM00000019429 7830 90.1 UdeM00000019429 XP_038646175.1 35.2 125 79 1 1 125 410 532 2.20E-15 90.1 7830 cellular organisms;Eukaryota;Opisthokonta;Metazoa;Eumetazoa;Bilateria;Deuterostomia;Chordata;Craniata;Vertebrata;Gnathostomata;Chondrichthyes;Elasmobranchii;Selachii;Galeomorphii;Galeoidea;Carcharhiniformes;Scyliorhinidae;Scyliorhinus;Scyliorhinus canicula
UdeM00000019430 7830 63.2 UdeM00000019430 XP_038646175.1 30.4 102 69 1 3 104 433 532 2.40E-07 63.2 7830 cellular organisms;Eukaryota;Opisthokonta;Metazoa;Eumetazoa;Bilateria;Deuterostomia;Chordata;Craniata;Vertebrata;Gnathostomata;Chondrichthyes;Elasmobranchii;Selachii;Galeomorphii;Galeoidea;Carcharhiniformes;Scyliorhinidae;Scyliorhinus;Scyliorhinus canicula
UdeM00000019431 45264 430.3 UdeM00000019431 XP_044179186.1 54.4 386 173 2 36 421 87 469 2.90E-117 430.3 45264 cellular organisms;Eukaryota;Opisthokonta;Metazoa;Eumetazoa;Cnidaria;Anthozoa;Hexacorallia;Scleractinia;Astrocoeniina;Acroporidae;Acropora;Acropora millepora
UdeM00000019432 6596 1145.6 UdeM00000019432 XP_045162681.1 63.5 937 293 11 7 932 8 906 0.00E+00 1145.6 6596 cellular organisms;Eukaryota;Opisthokonta;Metazoa;Eumetazoa;Bilateria;Protostomia;Spiralia;Lophotrochozoa;Mollusca;Bivalvia;Autobranchia;Heteroconchia;Euheterodonta;Imparidentia;Venerida;Veneroidea;Veneridae;Mercenaria;Mercenaria mercenaria
UdeM00000019433 6596 1132.5 UdeM00000019433 XP_045162681.1 61.9 961 293 12 7 956 8 906 0.00E+00 1132.5 6596 cellular organisms;Eukaryota;Opisthokonta;Metazoa;Eumetazoa;Bilateria;Protostomia;Spiralia;Lophotrochozoa;Mollusca;Bivalvia;Autobranchia;Heteroconchia;Euheterodonta;Imparidentia;Venerida;Veneroidea;Veneridae;Mercenaria;Mercenaria mercenaria
UdeM00000019434 6596 295.4 UdeM00000019434 XP_045163570.1 73.7 281 69 2 20 300 111 386 1.00E-76 295.4 6596 cellular organisms;Eukaryota;Opisthokonta;Metazoa;Eumetazoa;Bilateria;Protostomia;Spiralia;Lophotrochozoa;Mollusca;Bivalvia;Autobranchia;Heteroconchia;Euheterodonta;Imparidentia;Venerida;Veneroidea;Veneridae;Mercenaria;Mercenaria mercenaria

UdeM00000019435 6596 714.1 UdeM00000019435 XP_045161386.1 53.7 1302
452 28 30 1278 160 1363 3.10E-202 714.1 6596 cellular
organisms;Eukaryota;Opisthokonta;Metazoa;Eumetazoa;Bilateria;Protostomia;
Spiralia;Lophotrochozoa;Mollusca;Bivalvia;Autobranchia;Heteroconchia;Euhe
terodonta;Imparidentia;Venerida;Veneroidea;Veneridae;Mercenaria;Mercenari
a mercenaria
UdeM00000019436 6596 725.7 UdeM00000019436 XP_045161387.1 54.2 1293
402 27 30 1255 160 1329 1.00E-205 725.7 6596 cellular
organisms;Eukaryota;Opisthokonta;Metazoa;Eumetazoa;Bilateria;Protostomia;
Spiralia;Lophotrochozoa;Mollusca;Bivalvia;Autobranchia;Heteroconchia;Euhe
terodonta;Imparidentia;Venerida;Veneroidea;Veneridae;Mercenaria;Mercenari
a mercenaria
UdeM00000019437 6579 72 UdeM00000019437 XP_033730355.1 30.8 182
114 7 1 172 1 180 9.00E-10 72 6579 cellular
organisms;Eukaryota;Opisthokonta;Metazoa;Eumetazoa;Bilateria;Protostomia;
Spiralia;Lophotrochozoa;Mollusca;Bivalvia;Autobranchia;Pteriomorphia;Pect
inida;Pectinoidea;Pectinidae;Pecten;Pecten maximus
UdeM00000019440 6596 115.9 UdeM00000019440 XP_045177372.1 29.5 275
177 9 159 425 273 538 1.20E-22 115.9 6596 cellular
organisms;Eukaryota;Opisthokonta;Metazoa;Eumetazoa;Bilateria;Protostomia;
Spiralia;Lophotrochozoa;Mollusca;Bivalvia;Autobranchia;Heteroconchia;Euhe
terodonta;Imparidentia;Venerida;Veneroidea;Veneridae;Mercenaria;Mercenari
a mercenaria
UdeM00000019441 6596 130.2 UdeM00000019441 XP_045181277.1 34.1 205
129 5 31 232 124 325 3.60E-27 130.2 6596 cellular
organisms;Eukaryota;Opisthokonta;Metazoa;Eumetazoa;Bilateria;Protostomia;
Spiralia;Lophotrochozoa;Mollusca;Bivalvia;Autobranchia;Heteroconchia;Euhe
terodonta;Imparidentia;Venerida;Veneroidea;Veneridae;Mercenaria;Mercenari
a mercenaria
UdeM00000019442 6596 109.4 UdeM00000019442 XP_045181277.1 30.9 188
110 6 438 615 142 319 1.70E-20 109.4 6596 cellular
organisms;Eukaryota;Opisthokonta;Metazoa;Eumetazoa;Bilateria;Protostomia;
Spiralia;Lophotrochozoa;Mollusca;Bivalvia;Autobranchia;Heteroconchia;Euhe
terodonta;Imparidentia;Venerida;Veneroidea;Veneridae;Mercenaria;Mercenari
a mercenaria
UdeM00000019443 6596 145.6 UdeM00000019443 XP_045181277.1 31.9 238
142 6 4 231 92 319 8.10E-32 145.6 6596 cellular
organisms;Eukaryota;Opisthokonta;Metazoa;Eumetazoa;Bilateria;Protostomia;
Spiralia;Lophotrochozoa;Mollusca;Bivalvia;Autobranchia;Heteroconchia;Euhe
terodonta;Imparidentia;Venerida;Veneroidea;Veneridae;Mercenaria;Mercenari
a mercenaria
UdeM00000019445 7739 72.8 UdeM00000019445 XP_035659254.1 37.3 126
74 2 2 124 649 772 3.50E-10 72.8 7739 cellular
organisms;Eukaryota;Opisthokonta;Metazoa;Eumetazoa;Bilateria;Deuterostomi
a;Chordata;Cephalochordata;Leptocardii;Amphioxiformes;Branchiostomidae;Br
anchiostoma;Branchiostoma floridae
UdeM00000019447 6579 112.5 UdeM00000019447 XP_033728927.1 35.9 156
88 2 2 156 29 173 1.10E-21 112.5 6579 cellular
organisms;Eukaryota;Opisthokonta;Metazoa;Eumetazoa;Bilateria;Protostomia;
Spiralia;Lophotrochozoa;Mollusca;Bivalvia;Autobranchia;Pteriomorphia;Pect
inida;Pectinoidea;Pectinidae;Pecten;Pecten maximus
UdeM00000019448 6573 241.5 UdeM00000019448 XP_021363050.1 50.2 237
108 4 16 243 63 298 1.20E-60 241.5 6573 cellular
organisms;Eukaryota;Opisthokonta;Metazoa;Eumetazoa;Bilateria;Protostomia;

Spiralia;Lophotrochozoa;Mollusca;Bivalvia;Autobranchia;Pteriomorphia;Pectinida;Pectinoidea;Pectinidae;Mizuhopecten;Mizuhopecten yessoensis
UdeM00000019449 6596 806.2 UdeM00000019449 XP_045162989.1 54.1 706 314 5 3 700 102 805 3.30E-230 806.2 6596 cellular organisms;Eukaryota;Opisthokonta;Metazoa;Eumetazoa;Bilateria;Protostomia;Spiralia;Lophotrochozoa;Mollusca;Bivalvia;Autobranchia;Heteroconchia;Euheterodonta;Imparidentia;Venerida;Veneroidea;Veneridae;Mercenaria;Mercenaria mercenaria
UdeM00000019450 400727 90.1 UdeM00000019450 XP_025081733.1 59.8 82 30 3 1 81 13 92 2.30E-15 90.1 400727 cellular organisms;Eukaryota;Opisthokonta;Metazoa;Eumetazoa;Bilateria;Protostomia;Spiralia;Lophotrochozoa;Mollusca;Gastropoda;Caenogastropoda;Architaenioglossa;Ampullarioidea;Ampullariidae;Pomacea;Pomacea canaliculata
UdeM00000019451 29159 368.6 UdeM00000019451 XP_034338513.1 80.3 218 42 1 1 218 1 217 5.80E-99 368.6 29159 cellular organisms;Eukaryota;Opisthokonta;Metazoa;Eumetazoa;Bilateria;Protostomia;Spiralia;Lophotrochozoa;Mollusca;Bivalvia;Autobranchia;Pteriomorphia;Ostreida;Ostreoidea;Ostreidae;Crassostrea;Crassostrea gigas
UdeM00000019452 6596 506.1 UdeM00000019452 XP_045163117.1 55.2 435 179 4 2 427 94 521 4.40E-140 506.1 6596 cellular organisms;Eukaryota;Opisthokonta;Metazoa;Eumetazoa;Bilateria;Protostomia;Spiralia;Lophotrochozoa;Mollusca;Bivalvia;Autobranchia;Heteroconchia;Euheterodonta;Imparidentia;Venerida;Veneroidea;Veneridae;Mercenaria;Mercenaria mercenaria
UdeM00000019453 400727 145.2 UdeM00000019453 XP_025082559.1 42.5 181 91 6 38 205 40 220 1.10E-31 145.2 400727 cellular organisms;Eukaryota;Opisthokonta;Metazoa;Eumetazoa;Bilateria;Protostomia;Spiralia;Lophotrochozoa;Mollusca;Gastropoda;Caenogastropoda;Architaenioglossa;Ampullarioidea;Ampullariidae;Pomacea;Pomacea canaliculata
UdeM00000019454 29159 517.7 UdeM00000019454 XP_011418417.1 65.3 461 144 5 1 449 1 457 1.50E-143 517.7 29159 cellular organisms;Eukaryota;Opisthokonta;Metazoa;Eumetazoa;Bilateria;Protostomia;Spiralia;Lophotrochozoa;Mollusca;Bivalvia;Autobranchia;Pteriomorphia;Ostreida;Ostreoidea;Ostreidae;Crassostrea;Crassostrea gigas
UdeM00000019455 13686 375.2 UdeM00000019455 XP_011170408.3 41.3 494 265 9 97 573 125 610 1.50E-100 375.2 13686 cellular organisms;Eukaryota;Opisthokonta;Metazoa;Eumetazoa;Bilateria;Protostomia;Ecdysozoa;Panarthropoda;Arthropoda;Mandibulata;Pancrustacea;Hexapoda;Insecta;Dicondylia;Pterygota;Neoptera;Endopterygota;Hymenoptera;Apocrita;Aculeata;Formicoidea;Formicidae;Myrmicinae;Solenopsidini;Solenopsis;Solenopsis invicta
UdeM00000019456 6565 469.9 UdeM00000019456 XP_022286359.1 47.6 468 236 2 3 461 5 472 3.70E-129 469.9 6565 cellular organisms;Eukaryota;Opisthokonta;Metazoa;Eumetazoa;Bilateria;Protostomia;Spiralia;Lophotrochozoa;Mollusca;Bivalvia;Autobranchia;Pteriomorphia;Ostreida;Ostreoidea;Ostreidae;Crassostrea;Crassostrea virginica
UdeM00000019457 6596 196.8 UdeM00000019457 XP_045176388.1 27.6 446 311 7 23 464 13 450 6.00E-47 196.8 6596 cellular organisms;Eukaryota;Opisthokonta;Metazoa;Eumetazoa;Bilateria;Protostomia;Spiralia;Lophotrochozoa;Mollusca;Bivalvia;Autobranchia;Heteroconchia;Euheterodonta;Imparidentia;Venerida;Veneroidea;Veneridae;Mercenaria;Mercenaria mercenaria

UdeM00000019458 6596 217.2 UdeM00000019458 XP_045164036.1 31.6 459
299 9 14 462 9 462 4.30E-53 217.2 6596 cellular
organisms;Eukaryota;Opisthokonta;Metazoa;Eumetazoa;Bilateria;Protostomia;
Spiralia;Lophotrochozoa;Mollusca;Bivalvia;Autobranchia;Heteroconchia;Euhe
terodonta;Imparidentia;Venerida;Veneroidea;Veneridae;Mercenaria;Mercenari
a mercenaria
UdeM00000019459 6596 306.6 UdeM00000019459 XP_045161550.1 42.7 522
230 19 1 477 1 498 5.50E-80 306.6 6596 cellular
organisms;Eukaryota;Opisthokonta;Metazoa;Eumetazoa;Bilateria;Protostomia;
Spiralia;Lophotrochozoa;Mollusca;Bivalvia;Autobranchia;Heteroconchia;Euhe
terodonta;Imparidentia;Venerida;Veneroidea;Veneridae;Mercenaria;Mercenari
a mercenaria
UdeM00000019460 29159 471.1 UdeM00000019460 XP_011445975.1 81.1 270
48 2 17 285 17 284 1.00E-129 471.1 29159 cellular
organisms;Eukaryota;Opisthokonta;Metazoa;Eumetazoa;Bilateria;Protostomia;
Spiralia;Lophotrochozoa;Mollusca;Bivalvia;Autobranchia;Pteriomorphia;Ostr
eida;Ostreoidea;Ostreidae;Crassostrea;Crassostrea gigas
UdeM00000019461 6596 166.8 UdeM00000019461 XP_045163253.1 48.9 190
86 7 3 189 6 187 2.70E-38 166.8 6596 cellular
organisms;Eukaryota;Opisthokonta;Metazoa;Eumetazoa;Bilateria;Protostomia;
Spiralia;Lophotrochozoa;Mollusca;Bivalvia;Autobranchia;Heteroconchia;Euhe
terodonta;Imparidentia;Venerida;Veneroidea;Veneridae;Mercenaria;Mercenari
a mercenaria
UdeM00000019462 6596 157.9 UdeM00000019462 XP_045163901.1 40.8 201
118 1 1 201 20 219 1.40E-35 157.9 6596 cellular
organisms;Eukaryota;Opisthokonta;Metazoa;Eumetazoa;Bilateria;Protostomia;
Spiralia;Lophotrochozoa;Mollusca;Bivalvia;Autobranchia;Heteroconchia;Euhe
terodonta;Imparidentia;Venerida;Veneroidea;Veneridae;Mercenaria;Mercenari
a mercenaria
UdeM00000019463 225164 292.4 UdeM00000019463 XP_009063653.1 30.6
627 389 17 3 620 8 597 1.40E-75 292.4 225164
cellular
organisms;Eukaryota;Opisthokonta;Metazoa;Eumetazoa;Bilateria;Protostomia;
Spiralia;Lophotrochozoa;Mollusca;Gastropoda;Patellogastropoda;Lottioidea;
Lottiidae;Lottia;Lottia gigantea
UdeM00000019464 45264 464.2 UdeM00000019464 XP_044165535.1 49.1 450
223 2 175 620 123 570 3.60E-127 464.2 45264 cellular
organisms;Eukaryota;Opisthokonta;Metazoa;Eumetazoa;Cnidaria;Anthozoa;Hexa
corallia;Scleractinia;Astrocoeniina;Acroporidae;Acropora;Acropora
millepora
UdeM00000019465 6565 181.8 UdeM00000019465 XP_022344733.1 60.7 150
59 0 4 153 218 367 7.60E-43 181.8 6565 cellular
organisms;Eukaryota;Opisthokonta;Metazoa;Eumetazoa;Bilateria;Protostomia;
Spiralia;Lophotrochozoa;Mollusca;Bivalvia;Autobranchia;Pteriomorphia;Ostr
eida;Ostreoidea;Ostreidae;Crassostrea;Crassostrea virginica
UdeM00000019466 6573 717.2 UdeM00000019466 XP_021366468.1 82.3 423
73 2 410 830 364 786 2.40E-203 717.2 6573 cellular
organisms;Eukaryota;Opisthokonta;Metazoa;Eumetazoa;Bilateria;Protostomia;
Spiralia;Lophotrochozoa;Mollusca;Bivalvia;Autobranchia;Pteriomorphia;Pect
inida;Pectinoidea;Pectinidae;Mizuhopecten;Mizuhopecten yessoensis
UdeM00000019467 94827 142.1 UdeM00000019467 XP_010214492.1 35.7 221
140 2 1 220 616 835 1.10E-30 142.1 94827 cellular
organisms;Eukaryota;Opisthokonta;Metazoa;Eumetazoa;Bilateria;Deuterostomi
a;Chordata;Craniata;Vertebrata;Gnathostomata;Teleostomi;Euteleostomi;Sarc

opterygii;Dipnotetrapodomorpha;Tetrapoda;Amniota;Sauropsida;Sauria;Archelosauria;Archosauria;Dinosauria;Saurischia;Theropoda;Coelurosauria;Aves;Palaeognathae;Tinamiformes;Tinamidae;Tinamus;Tinamus guttatus
UdeM00000019468 143995 83.2 UdeM00000019468 XP_012136805.1 32.1 159 98 3 81 239 56 204 1.10E-12 83.2 143995 cellular organisms;Eukaryota;Opisthokonta;Metazoa;Eumetazoa;Bilateria;Protostomia;Ecdysozoa;Panarthropoda;Arthropoda;Mandibulata;Pancrustacea;Hexapoda;Insecta;Dicondylia;Pterygota;Neoptera;Endopterygota;Hymenoptera;Apocrita;Aculeata;Apoidea;Megachilidae;Megachilinae;Megachilini;Megachile;Megachile rotundata
UdeM00000019469 7460 132.9 UdeM00000019469 XP_026301736.1 27.7 430 255 15 1 393 740 1150 1.00E-27 132.9 7460 cellular organisms;Eukaryota;Opisthokonta;Metazoa;Eumetazoa;Bilateria;Protostomia;Ecdysozoa;Panarthropoda;Arthropoda;Mandibulata;Pancrustacea;Hexapoda;Insecta;Dicondylia;Pterygota;Neoptera;Endopterygota;Hymenoptera;Apocrita;Aculeata;Apoidea;Apidae;Apinae;Apini;Apis;Apis mellifera
UdeM00000019470 6565 306.6 UdeM00000019470 XP_022335580.1 53.6 265 122 1 21 285 39 302 3.30E-80 306.6 6565 cellular organisms;Eukaryota;Opisthokonta;Metazoa;Eumetazoa;Bilateria;Protostomia;Spiralia;Lophotrochozoa;Mollusca;Bivalvia;Autobranchia;Pteriomorphia;Ostreida;Ostreoidea;Ostreidae;Crassostrea;Crassostrea virginica
UdeM00000019471 6579 523.5 UdeM00000019471 XP_033724839.1 64.5 409 142 2 190 595 5 413 3.60E-145 523.5 6579 cellular organisms;Eukaryota;Opisthokonta;Metazoa;Eumetazoa;Bilateria;Protostomia;Spiralia;Lophotrochozoa;Mollusca;Bivalvia;Autobranchia;Pteriomorphia;Pectinida;Pectinoidea;Pectinidae;Pecten;Pecten maximus
UdeM00000019472 6565 72.8 UdeM00000019472 XP_022297447.1 50 66 33 0 1 66 301 366 1.90E-10 72.8 6565 cellular organisms;Eukaryota;Opisthokonta;Metazoa;Eumetazoa;Bilateria;Protostomia;Spiralia;Lophotrochozoa;Mollusca;Bivalvia;Autobranchia;Pteriomorphia;Ostreida;Ostreoidea;Ostreidae;Crassostrea;Crassostrea virginica
UdeM00000019473 6087 88.6 UdeM00000019473 XP_012558072.1 33.6 146 85 3 40 185 70 203 9.20E-15 88.6 6087 cellular organisms;Eukaryota;Opisthokonta;Metazoa;Eumetazoa;Cnidaria;Hydrozoa;Hydroidolina;Anthoathecata;Aplanulata;Hydridae;Hydra;Hydra vulgaris
UdeM00000019474 352472 59.7 UdeM00000019474 XP_640087.1 28.1 121 72 3 1 109 236 353 2.70E-06 59.7 352472 cellular organisms;Eukaryota;Amoebozoa;Evosea;Eumycetozoa;Dictyostelia;Dictyosteliales;Dictyosteliaceae;Dictyostelium;Dictyostelium discoideum;Dictyostelium discoideum AX4
UdeM00000019475 29159 253.8 UdeM00000019475 XP_011419833.1 69.5 187 51 2 1 185 230 412 1.70E-64 253.8 29159 cellular organisms;Eukaryota;Opisthokonta;Metazoa;Eumetazoa;Bilateria;Protostomia;Spiralia;Lophotrochozoa;Mollusca;Bivalvia;Autobranchia;Pteriomorphia;Ostreida;Ostreoidea;Ostreidae;Crassostrea;Crassostrea gigas
UdeM00000019476 29159 287 UdeM00000019476 XP_011419833.1 70.5 207 55 2 1 205 210 412 2.00E-74 287 29159 cellular organisms;Eukaryota;Opisthokonta;Metazoa;Eumetazoa;Bilateria;Protostomia;Spiralia;Lophotrochozoa;Mollusca;Bivalvia;Autobranchia;Pteriomorphia;Ostreida;Ostreoidea;Ostreidae;Crassostrea;Crassostrea gigas
UdeM00000019477 6500 168.3 UdeM00000019477 XP_005089155.2 61.7 141 43 3 42 176 64 199 9.70E-39 168.3 6500 cellular

organisms;Eukaryota;Opisthokonta;Metazoa;Eumetazoa;Bilateria;Protostomia;Spiralia;Lophotrochozoa;Mollusca;Gastropoda;Heterobranchia;Euthyneura;Tectipleura;Aplysiida;Aplysioidea;Aplysiidae;Aplysia;Aplysia californica
UdeM00000019478 151771 356.3 UdeM00000019478 XP_028406951.1 37.6 591 326 11 47 606 88 666 7.70E-95 356.3 151771 cellular organisms;Eukaryota;Opisthokonta;Metazoa;Eumetazoa;Cnidaria;Anthozoa;Octocorallia;Alcyonacea;Alcyoniina;Nephtheidae;Dendronephthya;Dendronephthya gigantea
UdeM00000019481 6945 62.4 UdeM00000019481 XP_040078187.1 29.9 117 81 1 76 191 38 154 1.30E-06 62.4 6945 cellular organisms;Eukaryota;Opisthokonta;Metazoa;Eumetazoa;Bilateria;Protostomia;Ecdysozoa;Panarthropoda;Arthropoda;Chelicerata;Arachnida;Acari;Parasitiformes;Ixodida;Ixodoidea;Ixodidae;Ixodinae;Ixodes;Ixodes scapularis
UdeM00000019482 6945 62.4 UdeM00000019482 XP_040078187.1 29.9 117 81 1 55 170 38 154 1.20E-06 62.4 6945 cellular organisms;Eukaryota;Opisthokonta;Metazoa;Eumetazoa;Bilateria;Protostomia;Ecdysozoa;Panarthropoda;Arthropoda;Chelicerata;Arachnida;Acari;Parasitiformes;Ixodida;Ixodoidea;Ixodidae;Ixodinae;Ixodes;Ixodes scapularis
UdeM00000019483 6596 102.4 UdeM00000019483 XP_045161259.1 41.9 124 64 4 25 143 26 146 5.70E-19 102.4 6596 cellular organisms;Eukaryota;Opisthokonta;Metazoa;Eumetazoa;Bilateria;Protostomia;Spiralia;Lophotrochozoa;Mollusca;Bivalvia;Autobranchia;Heteroconchia;Euheterodonta;Imparidentia;Venerida;Veneroidea;Veneridae;Mercenaria;Mercenaria mercenaria
UdeM00000019484 6596 102.4 UdeM00000019484 XP_045161259.1 38.3 149 77 5 14 153 26 168 5.30E-19 102.4 6596 cellular organisms;Eukaryota;Opisthokonta;Metazoa;Eumetazoa;Bilateria;Protostomia;Spiralia;Lophotrochozoa;Mollusca;Bivalvia;Autobranchia;Heteroconchia;Euheterodonta;Imparidentia;Venerida;Veneroidea;Veneridae;Mercenaria;Mercenaria mercenaria
UdeM00000019485 6596 104.8 UdeM00000019485 XP_045161260.1 37.2 137 79 3 17 149 15 148 1.20E-19 104.8 6596 cellular organisms;Eukaryota;Opisthokonta;Metazoa;Eumetazoa;Bilateria;Protostomia;Spiralia;Lophotrochozoa;Mollusca;Bivalvia;Autobranchia;Heteroconchia;Euheterodonta;Imparidentia;Venerida;Veneroidea;Veneridae;Mercenaria;Mercenaria mercenaria
UdeM00000019487 6596 111.3 UdeM00000019487 XP_045161259.1 39.7 146 79 4 25 164 26 168 1.20E-21 111.3 6596 cellular organisms;Eukaryota;Opisthokonta;Metazoa;Eumetazoa;Bilateria;Protostomia;Spiralia;Lophotrochozoa;Mollusca;Bivalvia;Autobranchia;Heteroconchia;Euheterodonta;Imparidentia;Venerida;Veneroidea;Veneridae;Mercenaria;Mercenaria mercenaria
UdeM00000019488 6596 554.3 UdeM00000019488 XP_045162509.1 64.8 423 143 3 1 420 1 420 1.30E-154 554.3 6596 cellular organisms;Eukaryota;Opisthokonta;Metazoa;Eumetazoa;Bilateria;Protostomia;Spiralia;Lophotrochozoa;Mollusca;Bivalvia;Autobranchia;Heteroconchia;Euheterodonta;Imparidentia;Venerida;Veneroidea;Veneridae;Mercenaria;Mercenaria mercenaria
UdeM00000019489 6596 670.2 UdeM00000019489 XP_045161143.1 69.7 446 135 0 35 480 237 682 1.90E-189 670.2 6596 cellular organisms;Eukaryota;Opisthokonta;Metazoa;Eumetazoa;Bilateria;Protostomia;Spiralia;Lophotrochozoa;Mollusca;Bivalvia;Autobranchia;Heteroconchia;Euhe

terodonta;Imparidentia;Venerida;Veneroidea;Veneridae;Mercenaria;Mercenaria mercenaria
UdeM00000019490 6596 393.7 UdeM00000019490 XP_045161143.1 48 444
80 2 1 293 239 682 2.10E-106 393.7 6596 cellular organisms;Eukaryota;Opisthokonta;Metazoa;Eumetazoa;Bilateria;Protostomia;Spiralia;Lophotrochozoa;Mollusca;Bivalvia;Autobranchia;Heteroconchia;Euheterodonta;Imparidentia;Venerida;Veneroidea;Veneridae;Mercenaria;Mercenaria mercenaria
UdeM00000019491 6596 786.6 UdeM00000019491 XP_045161143.1 61 616
211 3 5 594 70 682 2.30E-224 786.6 6596 cellular organisms;Eukaryota;Opisthokonta;Metazoa;Eumetazoa;Bilateria;Protostomia;Spiralia;Lophotrochozoa;Mollusca;Bivalvia;Autobranchia;Heteroconchia;Euheterodonta;Imparidentia;Venerida;Veneroidea;Veneridae;Mercenaria;Mercenaria mercenaria
UdeM00000019492 6596 636.7 UdeM00000019492 XP_045161143.1 66.4 444
147 1 1 442 239 682 2.20E-179 636.7 6596 cellular organisms;Eukaryota;Opisthokonta;Metazoa;Eumetazoa;Bilateria;Protostomia;Spiralia;Lophotrochozoa;Mollusca;Bivalvia;Autobranchia;Heteroconchia;Euheterodonta;Imparidentia;Venerida;Veneroidea;Veneridae;Mercenaria;Mercenaria mercenaria
UdeM00000019493 6565 102.4 UdeM00000019493 XP_022338490.1 36.3 135
84 2 1 134 4 137 5.00E-19 102.4 6565 cellular organisms;Eukaryota;Opisthokonta;Metazoa;Eumetazoa;Bilateria;Protostomia;Spiralia;Lophotrochozoa;Mollusca;Bivalvia;Autobranchia;Pteriomorphia;Ostreida;Ostreoidea;Ostreidae;Crassostrea;Crassostrea virginica
UdeM00000019494 6596 689.9 UdeM00000019494 XP_045161143.1 71.2 444
128 0 1 444 239 682 2.20E-195 689.9 6596 cellular organisms;Eukaryota;Opisthokonta;Metazoa;Eumetazoa;Bilateria;Protostomia;Spiralia;Lophotrochozoa;Mollusca;Bivalvia;Autobranchia;Heteroconchia;Euheterodonta;Imparidentia;Venerida;Veneroidea;Veneridae;Mercenaria;Mercenaria mercenaria
UdeM00000019495 6596 105.1 UdeM00000019495 XP_045161143.1 43.6 110
60 1 6 115 70 177 7.30E-20 105.1 6596 cellular organisms;Eukaryota;Opisthokonta;Metazoa;Eumetazoa;Bilateria;Protostomia;Spiralia;Lophotrochozoa;Mollusca;Bivalvia;Autobranchia;Heteroconchia;Euheterodonta;Imparidentia;Venerida;Veneroidea;Veneridae;Mercenaria;Mercenaria mercenaria
UdeM00000019496 6573 758.8 UdeM00000019496 XP_021363497.1 62 566
210 3 4 565 15 579 4.80E-216 758.8 6573 cellular organisms;Eukaryota;Opisthokonta;Metazoa;Eumetazoa;Bilateria;Protostomia;Spiralia;Lophotrochozoa;Mollusca;Bivalvia;Autobranchia;Pteriomorphia;Pectinida;Pectinoidea;Pectinidae;Mizuhopecten;Mizuhopecten yessoensis
UdeM00000019497 6565 719.9 UdeM00000019497 XP_022336153.1 60 565
221 5 5 567 8 569 2.50E-204 719.9 6565 cellular organisms;Eukaryota;Opisthokonta;Metazoa;Eumetazoa;Bilateria;Protostomia;Spiralia;Lophotrochozoa;Mollusca;Bivalvia;Autobranchia;Pteriomorphia;Ostreida;Ostreoidea;Ostreidae;Crassostrea;Crassostrea virginica
UdeM00000019498 6596 862.4 UdeM00000019498 XP_045162475.1 71.2 556
158 2 1 554 41 596 3.00E-247 862.4 6596 cellular organisms;Eukaryota;Opisthokonta;Metazoa;Eumetazoa;Bilateria;Protostomia;Spiralia;Lophotrochozoa;Mollusca;Bivalvia;Autobranchia;Heteroconchia;Euheterodonta;Imparidentia;Venerida;Veneroidea;Veneridae;Mercenaria;Mercenaria mercenaria

UdeM00000019499	6596	109.8	UdeM00000019499	XP_045208478.1	42.1	133
74	2	151	281	213	344	5.90E-21	109.8	6596	cellular
organisms;Eukaryota;Opisthokonta;Metazoa;Eumetazoa;Bilateria;Protostomia;
Spiralia;Lophotrochozoa;Mollusca;Bivalvia;Autobranchia;Heteroconchia;Euhe
terodonta;Imparidentia;Venerida;Veneroidea;Veneridae;Mercenaria;Mercenari
a mercenaria
UdeM00000019500	6596	621.3	UdeM00000019500	XP_045160149.1	31.3	1703
722	35	6	1311	3	1654	2.80E-174	621.3	6596	cellular
organisms;Eukaryota;Opisthokonta;Metazoa;Eumetazoa;Bilateria;Protostomia;
Spiralia;Lophotrochozoa;Mollusca;Bivalvia;Autobranchia;Heteroconchia;Euhe
terodonta;Imparidentia;Venerida;Veneroidea;Veneridae;Mercenaria;Mercenari
a mercenaria
UdeM00000019501	151771	248.1	UdeM00000019501	XP_028406951.1	31.7
492	215	7	1	396	119	585	2.00E-62	248.1	151771
cellular
organisms;Eukaryota;Opisthokonta;Metazoa;Eumetazoa;Cnidaria;Anthozoa;Octo
corallia;Alcyonacea;Alcyoniina;Nephtheidae;Dendronephthya;Dendronephthya
gigantea
UdeM00000019503	6579	232.3	UdeM00000019503	XP_033764002.1	54.5	202
91	1	6	206	12	213	5.80E-58	232.3	6579	cellular
organisms;Eukaryota;Opisthokonta;Metazoa;Eumetazoa;Bilateria;Protostomia;
Spiralia;Lophotrochozoa;Mollusca;Bivalvia;Autobranchia;Pteriomorphia;Pect
inida;Pectinoidea;Pectinidae;Pecten;Pecten maximus
UdeM00000019504	6596	323.2	UdeM00000019504	XP_045162910.1	65.8	231
77	2	4	234	60	288	2.90E-85	323.2	6596	cellular
organisms;Eukaryota;Opisthokonta;Metazoa;Eumetazoa;Bilateria;Protostomia;
Spiralia;Lophotrochozoa;Mollusca;Bivalvia;Autobranchia;Heteroconchia;Euhe
terodonta;Imparidentia;Venerida;Veneroidea;Veneridae;Mercenaria;Mercenari
a mercenaria
UdeM00000019507	6573	84	UdeM00000019507	XP_021360986.1	70.4	54
16	0	9	62	11	64	1.20E-13	84	6573	cellular
organisms;Eukaryota;Opisthokonta;Metazoa;Eumetazoa;Bilateria;Protostomia;
Spiralia;Lophotrochozoa;Mollusca;Bivalvia;Autobranchia;Pteriomorphia;Pect
inida;Pectinoidea;Pectinidae;Mizuhopecten;Mizuhopecten yessoensis
UdeM00000019508	6573	156.4	UdeM00000019508	XP_021360982.1	75.9	112
26	1	4	115	3	113	2.20E-35	156.4	6573	cellular
organisms;Eukaryota;Opisthokonta;Metazoa;Eumetazoa;Bilateria;Protostomia;
Spiralia;Lophotrochozoa;Mollusca;Bivalvia;Autobranchia;Pteriomorphia;Pect
inida;Pectinoidea;Pectinidae;Mizuhopecten;Mizuhopecten yessoensis
UdeM00000019509	6573	1409.8	UdeM00000019509	XP_021360979.1	63.7
2065	681	14	55	2095	422	2442	0.00E+00	1409.8	6573
cellular
organisms;Eukaryota;Opisthokonta;Metazoa;Eumetazoa;Bilateria;Protostomia;
Spiralia;Lophotrochozoa;Mollusca;Bivalvia;Autobranchia;Pteriomorphia;Pect
inida;Pectinoidea;Pectinidae;Mizuhopecten;Mizuhopecten yessoensis
UdeM00000019510	6565	3369.7	UdeM00000019510	XP_022339234.1	73.4
2253	566	13	1	2234	1	2238	0.00E+00	3369.7	6565
cellular
organisms;Eukaryota;Opisthokonta;Metazoa;Eumetazoa;Bilateria;Protostomia;
Spiralia;Lophotrochozoa;Mollusca;Bivalvia;Autobranchia;Pteriomorphia;Ostr
eida;Ostreoidea;Ostreidae;Crassostrea;Crassostrea virginica
UdeM00000019511	6596	212.6	UdeM00000019511	XP_045163028.1	37.3	354
209	6	19	372	26	366	9.30E-52	212.6	6596	cellular
organisms;Eukaryota;Opisthokonta;Metazoa;Eumetazoa;Bilateria;Protostomia;

Spiralia;Lophotrochozoa;Mollusca;Bivalvia;Autobranchia;Heteroconchia;Euheterodonta;Imparidentia;Venerida;Veneroidea;Veneridae;Mercenaria;Mercenaria mercenaria
UdeM00000019512 6596 125.2 UdeM00000019512 XP_045163028.1 46.8 154 77 3 95 247 73 222 1.50E-25 125.2 6596 cellular organisms;Eukaryota;Opisthokonta;Metazoa;Eumetazoa;Bilateria;Protostomia;Spiralia;Lophotrochozoa;Mollusca;Bivalvia;Autobranchia;Heteroconchia;Euheterodonta;Imparidentia;Venerida;Veneroidea;Veneridae;Mercenaria;Mercenaria mercenaria
UdeM00000019513 143950 128.3 UdeM00000019513 XP_025407409.1 44.5 137 71 2 1 132 70 206 7.50E-27 128.3 143950 cellular organisms;Eukaryota;Opisthokonta;Metazoa;Eumetazoa;Bilateria;Protostomia;Ecdysozoa;Panarthropoda;Arthropoda;Mandibulata;Pancrustacea;Hexapoda;Insecta;Dicondylia;Pterygota;Neoptera;Paraneoptera;Hemiptera;Sternorrhyncha;Aphidomorpha;Aphidoidea;Aphididae;Chaitophorinae;Sipha;Sipha flava
UdeM00000019514 143950 111.7 UdeM00000019514 XP_025407409.1 44.8 116 60 1 1 112 70 185 6.30E-22 111.7 143950 cellular organisms;Eukaryota;Opisthokonta;Metazoa;Eumetazoa;Bilateria;Protostomia;Ecdysozoa;Panarthropoda;Arthropoda;Mandibulata;Pancrustacea;Hexapoda;Insecta;Dicondylia;Pterygota;Neoptera;Paraneoptera;Hemiptera;Sternorrhyncha;Aphidomorpha;Aphidoidea;Aphididae;Chaitophorinae;Sipha;Sipha flava
UdeM00000019515 6596 237.7 UdeM00000019515 XP_045163028.1 40.9 372 208 7 1 371 8 368 2.70E-59 237.7 6596 cellular organisms;Eukaryota;Opisthokonta;Metazoa;Eumetazoa;Bilateria;Protostomia;Spiralia;Lophotrochozoa;Mollusca;Bivalvia;Autobranchia;Heteroconchia;Euheterodonta;Imparidentia;Venerida;Veneroidea;Veneridae;Mercenaria;Mercenaria mercenaria
UdeM00000019516 6596 245.7 UdeM00000019516 XP_045163028.1 41 356 197 4 17 372 26 368 1.00E-61 245.7 6596 cellular organisms;Eukaryota;Opisthokonta;Metazoa;Eumetazoa;Bilateria;Protostomia;Spiralia;Lophotrochozoa;Mollusca;Bivalvia;Autobranchia;Heteroconchia;Euheterodonta;Imparidentia;Venerida;Veneroidea;Veneridae;Mercenaria;Mercenaria mercenaria
UdeM00000019517 6596 229.2 UdeM00000019517 XP_045163028.1 37.6 356 218 3 5 360 15 366 9.30E-57 229.2 6596 cellular organisms;Eukaryota;Opisthokonta;Metazoa;Eumetazoa;Bilateria;Protostomia;Spiralia;Lophotrochozoa;Mollusca;Bivalvia;Autobranchia;Heteroconchia;Euheterodonta;Imparidentia;Venerida;Veneroidea;Veneridae;Mercenaria;Mercenaria mercenaria
UdeM00000019519 8090 100.9 UdeM00000019519 XP_023821816.1 66.7 66 22 0 4 69 675 740 7.30E-19 100.9 8090 cellular organisms;Eukaryota;Opisthokonta;Metazoa;Eumetazoa;Bilateria;Deuterostomia;Chordata;Craniata;Vertebrata;Gnathostomata;Teleostomi;Euteleostomi;Actinopterygii;Actinopteri;Neopterygii;Teleostei;Osteoglossocephalai;Clupeocephala;Euteleosteomorpha;Neoteleostei;Eurypterygia;Ctenosquamata;Acanthomorphata;Euacanthomorphacea;Percomorphaceae;Ovalentaria;Atherinomorphae;Beloniformes;Adrianichthyoidei;Adrianichthyidae;Oryziinae;Oryzias;Oryzias latipes
UdeM00000019521 6596 149.8 UdeM00000019521 XP_045181277.1 34.4 294 175 10 20 303 120 405 5.70E-33 149.8 6596 cellular organisms;Eukaryota;Opisthokonta;Metazoa;Eumetazoa;Bilateria;Protostomia;Spiralia;Lophotrochozoa;Mollusca;Bivalvia;Autobranchia;Heteroconchia;Euhe

terodonta;Imparidentia;Venerida;Veneroidea;Veneridae;Mercenaria;Mercenari
a mercenaria
UdeM00000019522 6596 92.4 UdeM00000019522 XP_045181277.1 26 269
131 6 165 368 55 320 1.30E-15 92.4 6596 cellular
organisms;Eukaryota;Opisthokonta;Metazoa;Eumetazoa;Bilateria;Protostomia;
Spiralia;Lophotrochozoa;Mollusca;Bivalvia;Autobranchia;Heteroconchia;Euhe
terodonta;Imparidentia;Venerida;Veneroidea;Veneridae;Mercenaria;Mercenari
a mercenaria
UdeM00000019523 6596 92.4 UdeM00000019523 XP_045181277.1 26 269
131 6 43 246 55 320 9.00E-16 92.4 6596 cellular
organisms;Eukaryota;Opisthokonta;Metazoa;Eumetazoa;Bilateria;Protostomia;
Spiralia;Lophotrochozoa;Mollusca;Bivalvia;Autobranchia;Heteroconchia;Euhe
terodonta;Imparidentia;Venerida;Veneroidea;Veneridae;Mercenaria;Mercenari
a mercenaria
UdeM00000019524 6596 72.8 UdeM00000019524 XP_045177153.1 29.6 186
109 8 46 214 22 202 6.20E-10 72.8 6596 cellular
organisms;Eukaryota;Opisthokonta;Metazoa;Eumetazoa;Bilateria;Protostomia;
Spiralia;Lophotrochozoa;Mollusca;Bivalvia;Autobranchia;Heteroconchia;Euhe
terodonta;Imparidentia;Venerida;Veneroidea;Veneridae;Mercenaria;Mercenari
a mercenaria
UdeM00000019525 6596 76.3 UdeM00000019525 XP_045177153.1 31.7 183
107 8 60 227 23 202 5.90E-11 76.3 6596 cellular
organisms;Eukaryota;Opisthokonta;Metazoa;Eumetazoa;Bilateria;Protostomia;
Spiralia;Lophotrochozoa;Mollusca;Bivalvia;Autobranchia;Heteroconchia;Euhe
terodonta;Imparidentia;Venerida;Veneroidea;Veneridae;Mercenaria;Mercenari
a mercenaria
UdeM00000019527 6596 71.2 UdeM00000019527 XP_045163252.1 30.3 198
115 7 74 257 263 451 2.10E-09 71.2 6596 cellular
organisms;Eukaryota;Opisthokonta;Metazoa;Eumetazoa;Bilateria;Protostomia;
Spiralia;Lophotrochozoa;Mollusca;Bivalvia;Autobranchia;Heteroconchia;Euhe
terodonta;Imparidentia;Venerida;Veneroidea;Veneridae;Mercenaria;Mercenari
a mercenaria
UdeM00000019526 6596 71.2 UdeM00000019526 XP_045163252.1 30.3 198
115 7 109 292 263 451 2.40E-09 71.2 6596 cellular
organisms;Eukaryota;Opisthokonta;Metazoa;Eumetazoa;Bilateria;Protostomia;
Spiralia;Lophotrochozoa;Mollusca;Bivalvia;Autobranchia;Heteroconchia;Euhe
terodonta;Imparidentia;Venerida;Veneroidea;Veneridae;Mercenaria;Mercenari
a mercenaria
UdeM00000019528 6596 71.2 UdeM00000019528 XP_045163252.1 30.3 198
115 7 182 365 263 451 3.00E-09 71.2 6596 cellular
organisms;Eukaryota;Opisthokonta;Metazoa;Eumetazoa;Bilateria;Protostomia;
Spiralia;Lophotrochozoa;Mollusca;Bivalvia;Autobranchia;Heteroconchia;Euhe
terodonta;Imparidentia;Venerida;Veneroidea;Veneridae;Mercenaria;Mercenari
a mercenaria
UdeM00000019529 29159 112.8 UdeM00000019529 XP_011453536.2 46.7 105
56 0 9 113 13 117 2.90E-22 112.8 29159 cellular
organisms;Eukaryota;Opisthokonta;Metazoa;Eumetazoa;Bilateria;Protostomia;
Spiralia;Lophotrochozoa;Mollusca;Bivalvia;Autobranchia;Pteriomorphia;Ostr
eida;Ostreoidea;Ostreidae;Crassostrea;Crassostrea gigas
UdeM00000019530 6596 288.5 UdeM00000019530 XP_045175151.1 37.4 457
252 9 28 468 38 476 1.50E-74 288.5 6596 cellular
organisms;Eukaryota;Opisthokonta;Metazoa;Eumetazoa;Bilateria;Protostomia;
Spiralia;Lophotrochozoa;Mollusca;Bivalvia;Autobranchia;Heteroconchia;Euhe

terodonta;Imparidentia;Venerida;Veneroidea;Veneridae;Mercenaria;Mercenari
a mercenaria
UdeM00000019531 6596 290.4 UdeM00000019531 XP_045175151.1 37.1 458
257 8 23 468 38 476 4.00E-75 290.4 6596 cellular
organisms;Eukaryota;Opisthokonta;Metazoa;Eumetazoa;Bilateria;Protostomia;
Spiralia;Lophotrochozoa;Mollusca;Bivalvia;Autobranchia;Heteroconchia;Euhe
terodonta;Imparidentia;Venerida;Veneroidea;Veneridae;Mercenaria;Mercenari
a mercenaria
UdeM00000019532 400727 145.2 UdeM00000019532 XP_025082564.1 31.2
560 308 21 26 544 59 582 3.10E-31 145.2 400727
cellular
organisms;Eukaryota;Opisthokonta;Metazoa;Eumetazoa;Bilateria;Protostomia;
Spiralia;Lophotrochozoa;Mollusca;Gastropoda;Caenogastropoda;Architaeniogl
ossa;Ampullarioidea;Ampullariidae;Pomacea;Pomacea canaliculata
UdeM00000019534 6579 375.6 UdeM00000019534 XP_033764119.1 76 229
54 1 1 228 1 229 4.70E-101 375.6 6579 cellular
organisms;Eukaryota;Opisthokonta;Metazoa;Eumetazoa;Bilateria;Protostomia;
Spiralia;Lophotrochozoa;Mollusca;Bivalvia;Autobranchia;Pteriomorphia;Pect
inida;Pectinoidea;Pectinidae;Pecten;Pecten maximus
UdeM00000019535 225164 581.6 UdeM00000019535 XP_009060418.1 80.1
361 71 1 216 576 215 574 1.10E-162 581.6 225164
cellular
organisms;Eukaryota;Opisthokonta;Metazoa;Eumetazoa;Bilateria;Protostomia;
Spiralia;Lophotrochozoa;Mollusca;Gastropoda;Patellogastropoda;Lottioidea;
Lottiidae;Lottia;Lottia gigantea
UdeM00000019536 6596 904 UdeM00000019536 XP_045163494.1 69.8 692
206 2 1 690 1 691 1.10E-259 904 6596 cellular
organisms;Eukaryota;Opisthokonta;Metazoa;Eumetazoa;Bilateria;Protostomia;
Spiralia;Lophotrochozoa;Mollusca;Bivalvia;Autobranchia;Heteroconchia;Euhe
terodonta;Imparidentia;Venerida;Veneroidea;Veneridae;Mercenaria;Mercenari
a mercenaria
UdeM00000019537 6565 225.3 UdeM00000019537 XP_022342749.1 48.8 256
120 4 3 247 4 259 1.20E-55 225.3 6565 cellular
organisms;Eukaryota;Opisthokonta;Metazoa;Eumetazoa;Bilateria;Protostomia;
Spiralia;Lophotrochozoa;Mollusca;Bivalvia;Autobranchia;Pteriomorphia;Ostr
eida;Ostreoidea;Ostreidae;Crassostrea;Crassostrea virginica
UdeM00000019538 6596 253.4 UdeM00000019538 XP_045162729.1 63.4 202
73 1 1 202 1 201 2.40E-64 253.4 6596 cellular
organisms;Eukaryota;Opisthokonta;Metazoa;Eumetazoa;Bilateria;Protostomia;
Spiralia;Lophotrochozoa;Mollusca;Bivalvia;Autobranchia;Heteroconchia;Euhe
terodonta;Imparidentia;Venerida;Veneroidea;Veneridae;Mercenaria;Mercenari
a mercenaria
UdeM00000019539 6596 565.1 UdeM00000019539 XP_045162684.1 57.1 452
189 1 5 451 30 481 8.10E-158 565.1 6596 cellular
organisms;Eukaryota;Opisthokonta;Metazoa;Eumetazoa;Bilateria;Protostomia;
Spiralia;Lophotrochozoa;Mollusca;Bivalvia;Autobranchia;Heteroconchia;Euhe
terodonta;Imparidentia;Venerida;Veneroidea;Veneridae;Mercenaria;Mercenari
a mercenaria
UdeM00000019540 6573 409.1 UdeM00000019540 XP_021340170.1 55.5 366
161 2 63 427 109 473 7.10E-111 409.1 6573 cellular
organisms;Eukaryota;Opisthokonta;Metazoa;Eumetazoa;Bilateria;Protostomia;
Spiralia;Lophotrochozoa;Mollusca;Bivalvia;Autobranchia;Pteriomorphia;Pect
inida;Pectinoidea;Pectinidae;Mizuhopecten;Mizuhopecten yessoensis

UdeM00000019541 1735272 442.2 UdeM00000019541 XP_041377515.1 48.7
415 202 4 26 434 154 563 2.60E-120 442.2 1735272
cellular
organisms;Eukaryota;Opisthokonta;Metazoa;Eumetazoa;Bilateria;Protostomia;
Spiralia;Lophotrochozoa;Mollusca;Gastropoda;Neomphalina;Neomphaloidea;Pel
tospiridae;Gigantopelta;Gigantopelta aegis
UdeM00000019542 1735272 442.2 UdeM00000019542 XP_041377515.1 48.7
415 202 4 26 434 154 563 2.60E-120 442.2 1735272
cellular
organisms;Eukaryota;Opisthokonta;Metazoa;Eumetazoa;Bilateria;Protostomia;
Spiralia;Lophotrochozoa;Mollusca;Gastropoda;Neomphalina;Neomphaloidea;Pel
tospiridae;Gigantopelta;Gigantopelta aegis
UdeM00000019543 6596 113.2 UdeM00000019543 XP_045177735.1 60.8 74
29 0 32 105 76 149 2.00E-22 113.2 6596 cellular
organisms;Eukaryota;Opisthokonta;Metazoa;Eumetazoa;Bilateria;Protostomia;
Spiralia;Lophotrochozoa;Mollusca;Bivalvia;Autobranchia;Heteroconchia;Euhe
terodonta;Imparidentia;Venerida;Veneroidea;Veneridae;Mercenaria;Mercenari
a mercenaria
UdeM00000019544 400727 100.1 UdeM00000019544 XP_025080280.1 64.2
67 24 0 27 93 7 73 1.50E-18 100.1 400727
cellular
organisms;Eukaryota;Opisthokonta;Metazoa;Eumetazoa;Bilateria;Protostomia;
Spiralia;Lophotrochozoa;Mollusca;Gastropoda;Caenogastropoda;Architaeniogl
ossa;Ampullarioidea;Ampullariidae;Pomacea;Pomacea canaliculata
UdeM00000019545 1735272 1879 UdeM00000019545 XP_041358414.1 63.1
1459 515 9 1 1446 7 1454 0.00E+00 1879 1735272
cellular
organisms;Eukaryota;Opisthokonta;Metazoa;Eumetazoa;Bilateria;Protostomia;
Spiralia;Lophotrochozoa;Mollusca;Gastropoda;Neomphalina;Neomphaloidea;Pel
tospiridae;Gigantopelta;Gigantopelta aegis
UdeM00000019546 6565 170.2 UdeM00000019546 XP_022299727.1 47.4 171
89 1 1 171 106 275 2.60E-39 170.2 6565 cellular
organisms;Eukaryota;Opisthokonta;Metazoa;Eumetazoa;Bilateria;Protostomia;
Spiralia;Lophotrochozoa;Mollusca;Bivalvia;Autobranchia;Pteriomorphia;Ostr
eida;Ostreoidea;Ostreidae;Crassostrea;Crassostrea virginica
UdeM00000019547 6579 685.3 UdeM00000019547 XP_033760107.1 76.9 433
100 0 1 433 1451 1883 5.20E-194 685.3 6579 cellular
organisms;Eukaryota;Opisthokonta;Metazoa;Eumetazoa;Bilateria;Protostomia;
Spiralia;Lophotrochozoa;Mollusca;Bivalvia;Autobranchia;Pteriomorphia;Pect
inida;Pectinoidea;Pectinidae;Pecten;Pecten maximus
UdeM00000019548 6596 523.1 UdeM00000019548 XP_045161770.1 87.8 278
33 1 1 277 1 278 2.20E-145 523.1 6596 cellular
organisms;Eukaryota;Opisthokonta;Metazoa;Eumetazoa;Bilateria;Protostomia;
Spiralia;Lophotrochozoa;Mollusca;Bivalvia;Autobranchia;Heteroconchia;Euhe
terodonta;Imparidentia;Venerida;Veneroidea;Veneridae;Mercenaria;Mercenari
a mercenaria
UdeM00000019549 29159 84.3 UdeM00000019549 XP_034338503.1 68.4 95
30 0 55 149 2 96 1.80E-13 84.3 29159 cellular
organisms;Eukaryota;Opisthokonta;Metazoa;Eumetazoa;Bilateria;Protostomia;
Spiralia;Lophotrochozoa;Mollusca;Bivalvia;Autobranchia;Pteriomorphia;Ostr
eida;Ostreoidea;Ostreidae;Crassostrea;Crassostrea gigas
UdeM00000019550 6596 300.8 UdeM00000019550 XP_045162257.1 49.7 300
148 2 1 297 1 300 1.90E-78 300.8 6596 cellular
organisms;Eukaryota;Opisthokonta;Metazoa;Eumetazoa;Bilateria;Protostomia;

Spiralia;Lophotrochozoa;Mollusca;Bivalvia;Autobranchia;Heteroconchia;Euheterodonta;Imparidentia;Venerida;Veneroidea;Veneridae;Mercenaria;Mercenaria mercenaria
UdeM00000019551 37621 146 UdeM00000019551 XP_014678940.1 75.3 85 21 0 21 105 484 568 4.00E-32 146 37621 cellular organisms;Eukaryota;Opisthokonta;Metazoa;Eumetazoa;Bilateria;Protostomia;Ecdysozoa;Scalidophora;Priapulida;Priapulimorpha;Priapulimorphida;Priapulidae;Priapulus;Priapulus caudatus
UdeM00000019552 29159 77.4 UdeM00000019552 XP_034339168.1 80 45 9 0 1 45 883 927 5.20E-12 77.4 29159 cellular organisms;Eukaryota;Opisthokonta;Metazoa;Eumetazoa;Bilateria;Protostomia;Spiralia;Lophotrochozoa;Mollusca;Bivalvia;Autobranchia;Pteriomorphia;Ostreida;Ostreoidea;Ostreidae;Crassostrea;Crassostrea gigas
UdeM00000019553 29159 103.6 UdeM00000019553 XP_034339168.1 46.5 129 40 1 1 100 883 1011 1.50E-19 103.6 29159 cellular organisms;Eukaryota;Opisthokonta;Metazoa;Eumetazoa;Bilateria;Protostomia;Spiralia;Lophotrochozoa;Mollusca;Bivalvia;Autobranchia;Pteriomorphia;Ostreida;Ostreoidea;Ostreidae;Crassostrea;Crassostrea gigas
UdeM00000019554 29159 160.6 UdeM00000019554 XP_034338151.1 40.5 220 58 2 3 149 131 350 1.70E-36 160.6 29159 cellular organisms;Eukaryota;Opisthokonta;Metazoa;Eumetazoa;Bilateria;Protostomia;Spiralia;Lophotrochozoa;Mollusca;Bivalvia;Autobranchia;Pteriomorphia;Ostreida;Ostreoidea;Ostreidae;Crassostrea;Crassostrea gigas
UdeM00000019556 6579 957.6 UdeM00000019556 XP_033760558.1 26.6 2212 1521 20 20 2202 730 2867 4.70E-275 957.6 6579 cellular organisms;Eukaryota;Opisthokonta;Metazoa;Eumetazoa;Bilateria;Protostomia;Spiralia;Lophotrochozoa;Mollusca;Bivalvia;Autobranchia;Pteriomorphia;Pectinida;Pectinoidea;Pectinidae;Pecten;Pecten maximus
UdeM00000019557 6596 265.4 UdeM00000019557 XP_045162858.1 51.2 256 121 3 1 255 1449 1701 1.10E-67 265.4 6596 cellular organisms;Eukaryota;Opisthokonta;Metazoa;Eumetazoa;Bilateria;Protostomia;Spiralia;Lophotrochozoa;Mollusca;Bivalvia;Autobranchia;Heteroconchia;Euheterodonta;Imparidentia;Venerida;Veneroidea;Veneridae;Mercenaria;Mercenaria mercenaria
UdeM00000019558 6596 67.4 UdeM00000019558 XP_045162855.1 40.5 84 46 3 43 125 34 114 1.90E-08 67.4 6596 cellular organisms;Eukaryota;Opisthokonta;Metazoa;Eumetazoa;Bilateria;Protostomia;Spiralia;Lophotrochozoa;Mollusca;Bivalvia;Autobranchia;Heteroconchia;Euheterodonta;Imparidentia;Venerida;Veneroidea;Veneridae;Mercenaria;Mercenaria mercenaria
UdeM00000019559 1735272 229.9 UdeM00000019559 XP_041357458.1 51 247 62 4 1 188 197 443 2.60E-57 229.9 1735272 cellular organisms;Eukaryota;Opisthokonta;Metazoa;Eumetazoa;Bilateria;Protostomia;Spiralia;Lophotrochozoa;Mollusca;Gastropoda;Neomphalina;Neomphaloidea;Peltospiridae;Gigantopelta;Gigantopelta aegis
UdeM00000019560 6579 776.5 UdeM00000019560 XP_033760558.1 25.6 2102 1422 28 3 2016 441 2489 7.90E-221 776.5 6579 cellular organisms;Eukaryota;Opisthokonta;Metazoa;Eumetazoa;Bilateria;Protostomia;Spiralia;Lophotrochozoa;Mollusca;Bivalvia;Autobranchia;Pteriomorphia;Pectinida;Pectinoidea;Pectinidae;Pecten;Pecten maximus
UdeM00000019561 6596 244.6 UdeM00000019561 XP_045162858.1 51.3 236 105 4 22 251 1480 1711 2.00E-61 244.6 6596 cellular organisms;Eukaryota;Opisthokonta;Metazoa;Eumetazoa;Bilateria;Protostomia;

Spiralia;Lophotrochozoa;Mollusca;Bivalvia;Autobranchia;Heteroconchia;Euheterodonta;Imparidentia;Venerida;Veneroidea;Veneridae;Mercenaria;Mercenaria mercenaria
UdeM00000019562 6579 289.7 UdeM00000019562 XP_033763324.1 31.6 629 362 13 576 1193 507 1078 1.70E-74 289.7 6579 cellular organisms;Eukaryota;Opisthokonta;Metazoa;Eumetazoa;Bilateria;Protostomia;Spiralia;Lophotrochozoa;Mollusca;Bivalvia;Autobranchia;Pteriomorphia;Pectinida;Pectinoidea;Pectinidae;Pecten;Pecten maximus
UdeM00000019563 6596 167.2 UdeM00000019563 XP_045162858.1 52.5 158 69 3 6 162 1549 1701 3.60E-38 167.2 6596 cellular organisms;Eukaryota;Opisthokonta;Metazoa;Eumetazoa;Bilateria;Protostomia;Spiralia;Lophotrochozoa;Mollusca;Bivalvia;Autobranchia;Heteroconchia;Euheterodonta;Imparidentia;Venerida;Veneroidea;Veneridae;Mercenaria;Mercenaria mercenaria
UdeM00000019564 6596 234.2 UdeM00000019564 XP_045162858.1 50.4 230 108 3 13 241 1480 1704 2.40E-58 234.2 6596 cellular organisms;Eukaryota;Opisthokonta;Metazoa;Eumetazoa;Bilateria;Protostomia;Spiralia;Lophotrochozoa;Mollusca;Bivalvia;Autobranchia;Heteroconchia;Euheterodonta;Imparidentia;Venerida;Veneroidea;Veneridae;Mercenaria;Mercenaria mercenaria
UdeM00000019565 6565 75.5 UdeM00000019565 XP_022339292.1 58.1 62 25 1 5 66 982 1042 2.90E-11 75.5 6565 cellular organisms;Eukaryota;Opisthokonta;Metazoa;Eumetazoa;Bilateria;Protostomia;Spiralia;Lophotrochozoa;Mollusca;Bivalvia;Autobranchia;Pteriomorphia;Ostreida;Ostreoidea;Ostreidae;Crassostrea;Crassostrea virginica
UdeM00000019566 6565 61.6 UdeM00000019566 XP_022339292.1 31.2 112 68 2 7 114 870 976 8.50E-07 61.6 6565 cellular organisms;Eukaryota;Opisthokonta;Metazoa;Eumetazoa;Bilateria;Protostomia;Spiralia;Lophotrochozoa;Mollusca;Bivalvia;Autobranchia;Pteriomorphia;Ostreida;Ostreoidea;Ostreidae;Crassostrea;Crassostrea virginica
UdeM00000019567 6579 499.2 UdeM00000019567 XP_033763324.1 37.4 819 455 18 223 1018 521 1304 1.90E-137 499.2 6579 cellular organisms;Eukaryota;Opisthokonta;Metazoa;Eumetazoa;Bilateria;Protostomia;Spiralia;Lophotrochozoa;Mollusca;Bivalvia;Autobranchia;Pteriomorphia;Pectinida;Pectinoidea;Pectinidae;Pecten;Pecten maximus
UdeM00000019569 6579 481.9 UdeM00000019569 XP_033763324.1 36.7 825 473 15 286 1089 521 1317 2.40E-132 481.9 6579 cellular organisms;Eukaryota;Opisthokonta;Metazoa;Eumetazoa;Bilateria;Protostomia;Spiralia;Lophotrochozoa;Mollusca;Bivalvia;Autobranchia;Pteriomorphia;Pectinida;Pectinoidea;Pectinidae;Pecten;Pecten maximus
UdeM00000019570 6579 483.8 UdeM00000019570 XP_033763324.1 37.9 780 451 16 132 885 521 1293 5.40E-133 483.8 6579 cellular organisms;Eukaryota;Opisthokonta;Metazoa;Eumetazoa;Bilateria;Protostomia;Spiralia;Lophotrochozoa;Mollusca;Bivalvia;Autobranchia;Pteriomorphia;Pectinida;Pectinoidea;Pectinidae;Pecten;Pecten maximus
UdeM00000019572 6565 169.1 UdeM00000019572 XP_022345276.1 44.7 190 52 1 47 183 212 401 7.00E-39 169.1 6565 cellular organisms;Eukaryota;Opisthokonta;Metazoa;Eumetazoa;Bilateria;Protostomia;Spiralia;Lophotrochozoa;Mollusca;Bivalvia;Autobranchia;Pteriomorphia;Ostreida;Ostreoidea;Ostreidae;Crassostrea;Crassostrea virginica
UdeM00000019573 6565 760.8 UdeM00000019573 XP_022336997.1 58.1 604 242 5 9 611 74 667 1.40E-216 760.8 6565 cellular organisms;Eukaryota;Opisthokonta;Metazoa;Eumetazoa;Bilateria;Protostomia;

Spiralia;Lophotrochozoa;Mollusca;Bivalvia;Autobranchia;Pteriomorphia;Ostreida;Ostreoidea;Ostreidae;Crassostrea;Crassostrea virginica
UdeM00000019575 2607531 563.1 UdeM00000019575 XP_029643579.1 39 698 413 5 55 749 51 738 5.10E-157 563.1 2607531 cellular organisms;Eukaryota;Opisthokonta;Metazoa;Eumetazoa;Bilateria;Protostomia;Spiralia;Lophotrochozoa;Mollusca;Cephalopoda;Coleoidea;Octopodiformes;Octopoda;Incirrata;Octopodidae;Octopus;Octopus sinensis
UdeM00000019576 225164 265.8 UdeM00000019576 XP_009049950.1 34.2 377 238 2 57 433 48 414 1.00E-67 265.8 225164 cellular organisms;Eukaryota;Opisthokonta;Metazoa;Eumetazoa;Bilateria;Protostomia;Spiralia;Lophotrochozoa;Mollusca;Gastropoda;Patellogastropoda;Lottioidea;Lottiidae;Lottia;Lottia gigantea
UdeM00000019577 6596 1495.3 UdeM00000019577 XP_045161724.1 76.5 925 204 5 1 920 1 917 0.00E+00 1495.3 6596 cellular organisms;Eukaryota;Opisthokonta;Metazoa;Eumetazoa;Bilateria;Protostomia;Spiralia;Lophotrochozoa;Mollusca;Bivalvia;Autobranchia;Heteroconchia;Euheterodonta;Imparidentia;Venerida;Veneroidea;Veneridae;Mercenaria;Mercenaria mercenaria
UdeM00000019578 6596 902.5 UdeM00000019578 XP_045161732.1 75.8 782 153 10 1 755 4 776 3.60E-259 902.5 6596 cellular organisms;Eukaryota;Opisthokonta;Metazoa;Eumetazoa;Bilateria;Protostomia;Spiralia;Lophotrochozoa;Mollusca;Bivalvia;Autobranchia;Heteroconchia;Euheterodonta;Imparidentia;Venerida;Veneroidea;Veneridae;Mercenaria;Mercenaria mercenaria
UdeM00000019579 6596 917.5 UdeM00000019579 XP_045161732.1 76.1 788 152 10 1 761 4 782 1.10E-263 917.5 6596 cellular organisms;Eukaryota;Opisthokonta;Metazoa;Eumetazoa;Bilateria;Protostomia;Spiralia;Lophotrochozoa;Mollusca;Bivalvia;Autobranchia;Heteroconchia;Euheterodonta;Imparidentia;Venerida;Veneroidea;Veneridae;Mercenaria;Mercenaria mercenaria
UdeM00000019591 6579 84 UdeM00000019591 XP_033728927.1 32.4 136 78 3 45 166 3 138 2.10E-13 84 6579 cellular organisms;Eukaryota;Opisthokonta;Metazoa;Eumetazoa;Bilateria;Protostomia;Spiralia;Lophotrochozoa;Mollusca;Bivalvia;Autobranchia;Pteriomorphia;Pectinida;Pectinoidea;Pectinidae;Pecten;Pecten maximus
UdeM00000019592 50429 68.2 UdeM00000019592 XP_022804186.1 43.1 72 41 0 1 72 430 501 5.50E-09 68.2 50429 cellular organisms;Eukaryota;Opisthokonta;Metazoa;Eumetazoa;Cnidaria;Anthozoa;Hexacorallia;Scleractinia;Astrocoeniina;Pocilloporidae;Stylophora;Stylophora pistillata
UdeM00000019594 6596 475.3 UdeM00000019594 XP_045163816.1 44.6 561 308 2 25 583 36 595 1.10E-130 475.3 6596 cellular organisms;Eukaryota;Opisthokonta;Metazoa;Eumetazoa;Bilateria;Protostomia;Spiralia;Lophotrochozoa;Mollusca;Bivalvia;Autobranchia;Heteroconchia;Euheterodonta;Imparidentia;Venerida;Veneroidea;Veneridae;Mercenaria;Mercenaria mercenaria
UdeM00000019595 6596 469.9 UdeM00000019595 XP_045163816.1 44.1 567 314 2 25 589 36 601 4.70E-129 469.9 6596 cellular organisms;Eukaryota;Opisthokonta;Metazoa;Eumetazoa;Bilateria;Protostomia;Spiralia;Lophotrochozoa;Mollusca;Bivalvia;Autobranchia;Heteroconchia;Euhe

terodonta;Imparidentia;Venerida;Veneroidea;Veneridae;Mercenaria;Mercenaria mercenaria
UdeM00000019596 6596 143.3 UdeM00000019596 XP_045163816.1 37 216
134 2 7 221 21 235 4.70E-31 143.3 6596 cellular organisms;Eukaryota;Opisthokonta;Metazoa;Eumetazoa;Bilateria;Protostomia;Spiralia;Lophotrochozoa;Mollusca;Bivalvia;Autobranchia;Heteroconchia;Euheterodonta;Imparidentia;Venerida;Veneroidea;Veneridae;Mercenaria;Mercenaria mercenaria
UdeM00000019598 6596 476.5 UdeM00000019598 XP_045163816.1 44.1 567
314 2 25 589 36 601 5.00E-131 476.5 6596 cellular organisms;Eukaryota;Opisthokonta;Metazoa;Eumetazoa;Bilateria;Protostomia;Spiralia;Lophotrochozoa;Mollusca;Bivalvia;Autobranchia;Heteroconchia;Euheterodonta;Imparidentia;Venerida;Veneroidea;Veneridae;Mercenaria;Mercenaria mercenaria
UdeM00000019599 6579 104.8 UdeM00000019599 XP_033761495.1 65.8 73
25 0 36 108 24 96 9.50E-20 104.8 6579 cellular organisms;Eukaryota;Opisthokonta;Metazoa;Eumetazoa;Bilateria;Protostomia;Spiralia;Lophotrochozoa;Mollusca;Bivalvia;Autobranchia;Pteriomorphia;Pectinida;Pectinoidea;Pectinidae;Pecten;Pecten maximus
UdeM00000019600 6579 331.6 UdeM00000019600 XP_033761495.1 83.6 183
30 0 1 183 177 359 1.50E-87 331.6 6579 cellular organisms;Eukaryota;Opisthokonta;Metazoa;Eumetazoa;Bilateria;Protostomia;Spiralia;Lophotrochozoa;Mollusca;Bivalvia;Autobranchia;Pteriomorphia;Pectinida;Pectinoidea;Pectinidae;Pecten;Pecten maximus
UdeM00000019601 1735272 82.4 UdeM00000019601 XP_041362151.1 37.2
113 70 1 9 120 563 675 4.30E-13 82.4 1735272
cellular organisms;Eukaryota;Opisthokonta;Metazoa;Eumetazoa;Bilateria;Protostomia;Spiralia;Lophotrochozoa;Mollusca;Gastropoda;Neomphalina;Neomphaloidea;Peltospiridae;Gigantopelta;Gigantopelta aegis
UdeM00000019602 6579 990.7 UdeM00000019602 XP_033744655.1 75.9 636
153 0 1 636 1 636 8.60E-286 990.7 6579 cellular organisms;Eukaryota;Opisthokonta;Metazoa;Eumetazoa;Bilateria;Protostomia;Spiralia;Lophotrochozoa;Mollusca;Bivalvia;Autobranchia;Pteriomorphia;Pectinida;Pectinoidea;Pectinidae;Pecten;Pecten maximus
UdeM00000019603 7574 68.9 UdeM00000019603 XP_013408145.1 26.2 229
113 10 233 451 2275 2457 1.90E-08 68.9 7574 cellular organisms;Eukaryota;Opisthokonta;Metazoa;Eumetazoa;Bilateria;Protostomia;Spiralia;Lophotrochozoa;Brachiopoda;Linguliformea;Lingulata;Lingulida;Linguloidea;Lingulidae;Lingula;Lingula anatina
UdeM00000019605 6596 229.9 UdeM00000019605 XP_045209202.1 28 507
258 9 27 433 1217 1716 6.10E-57 229.9 6596 cellular organisms;Eukaryota;Opisthokonta;Metazoa;Eumetazoa;Bilateria;Protostomia;Spiralia;Lophotrochozoa;Mollusca;Bivalvia;Autobranchia;Heteroconchia;Euheterodonta;Imparidentia;Venerida;Veneroidea;Veneridae;Mercenaria;Mercenaria mercenaria
UdeM00000019606 6526 120.9 UdeM00000019606 XP_013068176.1 45.5 121
64 2 10 129 677 796 1.30E-24 120.9 6526 cellular organisms;Eukaryota;Opisthokonta;Metazoa;Eumetazoa;Bilateria;Protostomia;Spiralia;Lophotrochozoa;Mollusca;Gastropoda;Heterobranchia;Euthyneura;Panpulmonata;Hygrophila;Lymnaeoidea;Planorbidae;Biomphalaria;Biomphalaria glabrata
UdeM00000019607 1735272 141.4 UdeM00000019607 XP_041361861.1 45.7
151 53 3 1 122 71 221 8.10E-31 141.4 1735272

cellular organisms;Eukaryota;Opisthokonta;Metazoa;Eumetazoa;Bilateria;Protostomia;Spiralia;Lophotrochozoa;Mollusca;Gastropoda;Neomphalina;Neomphaloidea;Peltospiridae;Gigantopelta;Gigantopelta aegis
UdeM00000019608 29159 83.2 UdeM00000019608 XP_034317111.1 60.7 61 24 0 2 62 171 231 1.70E-13 83.2 29159 cellular organisms;Eukaryota;Opisthokonta;Metazoa;Eumetazoa;Bilateria;Protostomia;Spiralia;Lophotrochozoa;Mollusca;Bivalvia;Autobranchia;Pteriomorphia;Ostreida;Ostreoidea;Ostreidae;Crassostrea;Crassostrea gigas
UdeM00000019609 6565 62 UdeM00000019609 XP_022299838.1 48.8 41 21 0 61 101 67 107 5.10E-07 62 6565 cellular organisms;Eukaryota;Opisthokonta;Metazoa;Eumetazoa;Bilateria;Protostomia;Spiralia;Lophotrochozoa;Mollusca;Bivalvia;Autobranchia;Pteriomorphia;Ostreida;Ostreoidea;Ostreidae;Crassostrea;Crassostrea virginica
UdeM00000019611 6579 199.1 UdeM00000019611 XP_033748473.1 51.3 193 90 3 202 392 1 191 1.00E-47 199.1 6579 cellular organisms;Eukaryota;Opisthokonta;Metazoa;Eumetazoa;Bilateria;Protostomia;Spiralia;Lophotrochozoa;Mollusca;Bivalvia;Autobranchia;Pteriomorphia;Pectinida;Pectinoidea;Pectinidae;Pecten;Pecten maximus
UdeM00000019612 50390 193.7 UdeM00000019612 XP_028127913.1 43.3 231 130 1 1 231 1 230 2.60E-46 193.7 50390 cellular organisms;Eukaryota;Opisthokonta;Metazoa;Eumetazoa;Bilateria;Protostomia;Ecdysozoa;Panarthropoda;Arthropoda;Mandibulata;Pancrustacea;Hexapoda;Insecta;Dicondylia;Pterygota;Neoptera;Endopterygota;Coleoptera;Polyphaga;Cucujiformia;Chrysomeloidea;Chrysomelidae;Galerucinae;Luperini;Diabroticina;Diabroticites;Diabrotica;Diabrotica virgifera;Diabrotica virgifera virgifera
UdeM00000019613 6596 479.9 UdeM00000019613 XP_045163816.1 44.4 567 312 2 25 589 36 601 4.50E-132 479.9 6596 cellular organisms;Eukaryota;Opisthokonta;Metazoa;Eumetazoa;Bilateria;Protostomia;Spiralia;Lophotrochozoa;Mollusca;Bivalvia;Autobranchia;Heteroconchia;Euheterodonta;Imparidentia;Venerida;Veneroidea;Veneridae;Mercenaria;Mercenaria mercenaria
UdeM00000019615 6596 340.9 UdeM00000019615 XP_045163816.1 46.9 375 198 1 20 394 228 601 2.20E-90 340.9 6596 cellular organisms;Eukaryota;Opisthokonta;Metazoa;Eumetazoa;Bilateria;Protostomia;Spiralia;Lophotrochozoa;Mollusca;Bivalvia;Autobranchia;Heteroconchia;Euheterodonta;Imparidentia;Venerida;Veneroidea;Veneridae;Mercenaria;Mercenaria mercenaria
UdeM00000019616 6596 139.4 UdeM00000019616 XP_045163816.1 38.4 198 120 1 25 220 36 233 6.90E-30 139.4 6596 cellular organisms;Eukaryota;Opisthokonta;Metazoa;Eumetazoa;Bilateria;Protostomia;Spiralia;Lophotrochozoa;Mollusca;Bivalvia;Autobranchia;Heteroconchia;Euheterodonta;Imparidentia;Venerida;Veneroidea;Veneridae;Mercenaria;Mercenaria mercenaria
UdeM00000019617 7604 157.5 UdeM00000019617 XP_033638534.1 43.2 190 100 2 314 495 1 190 5.80E-35 157.5 7604 cellular organisms;Eukaryota;Opisthokonta;Metazoa;Eumetazoa;Bilateria;Deuterostomia;Echinodermata;Eleutherozoa;Asterozoa;Asteroidea;Forcipulatacea;Forcipulatida;Asteriidae;Asterias;Asterias rubens
UdeM00000019619 6087 77.8 UdeM00000019619 XP_012561779.1 44.9 89 46 1 29 114 686 774 3.00E-11 77.8 6087 cellular organisms;Eukaryota;Opisthokonta;Metazoa;Eumetazoa;Cnidaria;Hydrozoa;Hydroidolina;Anthoathecata;Aplanulata;Hydridae;Hydra;Hydra vulgaris

UdeM00000019623 1735272 154.5 UdeM00000019623 XP_041365752.1 33.9 286 173 4 25 303 165 441 2.30E-34 154.5 1735272 cellular organisms;Eukaryota;Opisthokonta;Metazoa;Eumetazoa;Bilateria;Protostomia;Spiralia;Lophotrochozoa;Mollusca;Gastropoda;Neomphalina;Neomphaloidea;Peltospiridae;Gigantopelta;Gigantopelta aegis
UdeM00000019625 6565 94 UdeM00000019625 XP_022322825.1 41.8 110 63 1 4 112 343 452 1.40E-16 94 6565 cellular organisms;Eukaryota;Opisthokonta;Metazoa;Eumetazoa;Bilateria;Protostomia;Spiralia;Lophotrochozoa;Mollusca;Bivalvia;Autobranchia;Pteriomorphia;Ostreida;Ostreoidea;Ostreidae;Crassostrea;Crassostrea virginica
UdeM00000019626 29159 104.4 UdeM00000019626 XP_011453536.2 37 165 54 5 7 126 14 173 2.20E-19 104.4 29159 cellular organisms;Eukaryota;Opisthokonta;Metazoa;Eumetazoa;Bilateria;Protostomia;Spiralia;Lophotrochozoa;Mollusca;Bivalvia;Autobranchia;Pteriomorphia;Ostreida;Ostreoidea;Ostreidae;Crassostrea;Crassostrea gigas
UdeM00000019630 6579 86.7 UdeM00000019630 XP_033753277.1 43.4 113 63 1 20 131 43 155 4.40E-14 86.7 6579 cellular organisms;Eukaryota;Opisthokonta;Metazoa;Eumetazoa;Bilateria;Protostomia;Spiralia;Lophotrochozoa;Mollusca;Bivalvia;Autobranchia;Pteriomorphia;Pectinida;Pectinoidea;Pectinidae;Pecten;Pecten maximus
UdeM00000019631 6565 115.2 UdeM00000019631 XP_022322825.1 45.5 132 70 2 2 132 377 507 7.00E-23 115.2 6565 cellular organisms;Eukaryota;Opisthokonta;Metazoa;Eumetazoa;Bilateria;Protostomia;Spiralia;Lophotrochozoa;Mollusca;Bivalvia;Autobranchia;Pteriomorphia;Ostreida;Ostreoidea;Ostreidae;Crassostrea;Crassostrea virginica
UdeM00000019632 6596 161.8 UdeM00000019632 XP_045162108.1 54.7 128 55 3 2 129 52 176 1.20E-36 161.8 6596 cellular organisms;Eukaryota;Opisthokonta;Metazoa;Eumetazoa;Bilateria;Protostomia;Spiralia;Lophotrochozoa;Mollusca;Bivalvia;Autobranchia;Heteroconchia;Euheterodonta;Imparidentia;Venerida;Veneroidea;Veneridae;Mercenaria;Mercenaria mercenaria
UdeM00000019633 6596 91.7 UdeM00000019633 XP_045163252.1 34.4 195 111 5 220 403 263 451 2.40E-15 91.7 6596 cellular organisms;Eukaryota;Opisthokonta;Metazoa;Eumetazoa;Bilateria;Protostomia;Spiralia;Lophotrochozoa;Mollusca;Bivalvia;Autobranchia;Heteroconchia;Euheterodonta;Imparidentia;Venerida;Veneroidea;Veneridae;Mercenaria;Mercenaria mercenaria
UdeM00000019634 6596 75.1 UdeM00000019634 XP_045177153.1 31.5 181 102 8 37 200 27 202 1.20E-10 75.1 6596 cellular organisms;Eukaryota;Opisthokonta;Metazoa;Eumetazoa;Bilateria;Protostomia;Spiralia;Lophotrochozoa;Mollusca;Bivalvia;Autobranchia;Heteroconchia;Euheterodonta;Imparidentia;Venerida;Veneroidea;Veneridae;Mercenaria;Mercenaria mercenaria
UdeM00000019635 6596 73.2 UdeM00000019635 XP_045177153.1 31.6 158 89 5 6 147 48 202 3.30E-10 73.2 6596 cellular organisms;Eukaryota;Opisthokonta;Metazoa;Eumetazoa;Bilateria;Protostomia;Spiralia;Lophotrochozoa;Mollusca;Bivalvia;Autobranchia;Heteroconchia;Euheterodonta;Imparidentia;Venerida;Veneroidea;Veneridae;Mercenaria;Mercenaria mercenaria
UdeM00000019636 6596 397.1 UdeM00000019636 XP_045163074.1 51 439 184 10 72 486 243 674 3.60E-107 397.1 6596 cellular organisms;Eukaryota;Opisthokonta;Metazoa;Eumetazoa;Bilateria;Protostomia;Spiralia;Lophotrochozoa;Mollusca;Bivalvia;Autobranchia;Heteroconchia;Euhe

terodonta;Imparidentia;Venerida;Veneroidea;Veneridae;Mercenaria;Mercenaria mercenaria
UdeM00000019637 29159 622.1 UdeM00000019637 XP_034299214.1 49.4 708
324 9 30 704 889 1595 8.80E-175 622.1 29159 cellular organisms;Eukaryota;Opisthokonta;Metazoa;Eumetazoa;Bilateria;Protostomia;Spiralia;Lophotrochozoa;Mollusca;Bivalvia;Autobranchia;Pteriomorphia;Ostreida;Ostreoidea;Ostreidae;Crassostrea;Crassostrea gigas
UdeM00000019638 6596 750.7 UdeM00000019638 XP_045163568.1 50.1 875
383 12 88 921 733 1594 2.10E-213 750.7 6596 cellular organisms;Eukaryota;Opisthokonta;Metazoa;Eumetazoa;Bilateria;Protostomia;Spiralia;Lophotrochozoa;Mollusca;Bivalvia;Autobranchia;Heteroconchia;Euheterodonta;Imparidentia;Venerida;Veneroidea;Veneridae;Mercenaria;Mercenaria mercenaria
UdeM00000019639 75366 186 UdeM00000019639 XP_016148261.1 47.1 242
107 6 11 245 757 984 6.10E-44 186 75366 cellular organisms;Eukaryota;Opisthokonta;Metazoa;Eumetazoa;Bilateria;Deuterostomia;Chordata;Craniata;Vertebrata;Gnathostomata;Teleostomi;Euteleostomi;Actinopterygii;Actinopteri;Neopterygii;Teleostei;Osteoglossocephalai;Clupeocephala;Otomorpha;Ostariophysi;Otophysi;Cypriniphysae;Cypriniformes;Cyprinoidei;Cyprinidae;Cyprininae;Sinocyclocheilus;Sinocyclocheilus grahami
UdeM00000019641 45264 392.9 UdeM00000019641 XP_044165535.1 49.6 367
181 1 1 363 158 524 4.90E-106 392.9 45264 cellular organisms;Eukaryota;Opisthokonta;Metazoa;Eumetazoa;Cnidaria;Anthozoa;Hexacorallia;Scleractinia;Astrocoeniina;Acroporidae;Acropora;Acropora millepora
UdeM00000019642 6596 790.8 UdeM00000019642 XP_045163568.1 54.5 888
361 15 6 858 715 1594 1.70E-225 790.8 6596 cellular organisms;Eukaryota;Opisthokonta;Metazoa;Eumetazoa;Bilateria;Protostomia;Spiralia;Lophotrochozoa;Mollusca;Bivalvia;Autobranchia;Heteroconchia;Euheterodonta;Imparidentia;Venerida;Veneroidea;Veneridae;Mercenaria;Mercenaria mercenaria
UdeM00000019643 6596 501.1 UdeM00000019643 XP_045163568.1 56.1 611
229 13 10 589 733 1335 1.90E-138 501.1 6596 cellular organisms;Eukaryota;Opisthokonta;Metazoa;Eumetazoa;Bilateria;Protostomia;Spiralia;Lophotrochozoa;Mollusca;Bivalvia;Autobranchia;Heteroconchia;Euheterodonta;Imparidentia;Venerida;Veneroidea;Veneridae;Mercenaria;Mercenaria mercenaria
UdeM00000019644 29159 299.3 UdeM00000019644 XP_019929897.2 57.9 252
105 1 1 252 1360 1610 4.70E-78 299.3 29159 cellular organisms;Eukaryota;Opisthokonta;Metazoa;Eumetazoa;Bilateria;Protostomia;Spiralia;Lophotrochozoa;Mollusca;Bivalvia;Autobranchia;Pteriomorphia;Ostreida;Ostreoidea;Ostreidae;Crassostrea;Crassostrea gigas
UdeM00000019645 6579 69.7 UdeM00000019645 XP_033763913.1 61.7 60
22 1 1 60 1565 1623 1.50E-09 69.7 6579 cellular organisms;Eukaryota;Opisthokonta;Metazoa;Eumetazoa;Bilateria;Protostomia;Spiralia;Lophotrochozoa;Mollusca;Bivalvia;Autobranchia;Pteriomorphia;Pectinida;Pectinoidea;Pectinidae;Pecten;Pecten maximus
UdeM00000019646 6565 246.1 UdeM00000019646 XP_022336762.1 38.6 396
191 9 5 348 1068 1463 6.60E-62 246.1 6565 cellular organisms;Eukaryota;Opisthokonta;Metazoa;Eumetazoa;Bilateria;Protostomia;Spiralia;Lophotrochozoa;Mollusca;Bivalvia;Autobranchia;Pteriomorphia;Ostreida;Ostreoidea;Ostreidae;Crassostrea;Crassostrea virginica
UdeM00000019647 6596 103.2 UdeM00000019647 XP_045163568.1 79.7 64
13 0 1 64 977 1040 1.50E-19 103.2 6596 cellular

organisms;Eukaryota;Opisthokonta;Metazoa;Eumetazoa;Bilateria;Protostomia;Spiralia;Lophotrochozoa;Mollusca;Bivalvia;Autobranchia;Heteroconchia;Euheterodonta;Imparidentia;Venerida;Veneroidea;Veneridae;Mercenaria;Mercenaria mercenaria
UdeM00000019648 6596 208.4 UdeM00000019648 XP_045163568.1 55.5 218 64 4 12 196 733 950 8.50E-51 208.4 6596 cellular organisms;Eukaryota;Opisthokonta;Metazoa;Eumetazoa;Bilateria;Protostomia;Spiralia;Lophotrochozoa;Mollusca;Bivalvia;Autobranchia;Heteroconchia;Euheterodonta;Imparidentia;Venerida;Veneroidea;Veneridae;Mercenaria;Mercenaria mercenaria
UdeM00000019649 6596 878.2 UdeM00000019649 XP_045163568.1 53.2 977 343 19 1106 1978 628 1594 1.90E-251 878.2 6596 cellular organisms;Eukaryota;Opisthokonta;Metazoa;Eumetazoa;Bilateria;Protostomia;Spiralia;Lophotrochozoa;Mollusca;Bivalvia;Autobranchia;Heteroconchia;Euheterodonta;Imparidentia;Venerida;Veneroidea;Veneridae;Mercenaria;Mercenaria mercenaria
UdeM00000019650 6596 941 UdeM00000019650 XP_045163568.1 58.6 977 355 17 728 1665 628 1594 2.00E-270 941 6596 cellular organisms;Eukaryota;Opisthokonta;Metazoa;Eumetazoa;Bilateria;Protostomia;Spiralia;Lophotrochozoa;Mollusca;Bivalvia;Autobranchia;Heteroconchia;Euheterodonta;Imparidentia;Venerida;Veneroidea;Veneridae;Mercenaria;Mercenaria mercenaria
UdeM00000019651 6573 100.5 UdeM00000019651 XP_021365625.1 54.1 85 39 0 76 160 22 106 2.80E-18 100.5 6573 cellular organisms;Eukaryota;Opisthokonta;Metazoa;Eumetazoa;Bilateria;Protostomia;Spiralia;Lophotrochozoa;Mollusca;Bivalvia;Autobranchia;Pteriomorphia;Pectinida;Pectinoidea;Pectinidae;Mizuhopecten;Mizuhopecten yessoensis
UdeM00000019652 6596 426 UdeM00000019652 XP_045163568.1 62.1 398 125 8 432 809 628 1019 1.20E-115 426 6596 cellular organisms;Eukaryota;Opisthokonta;Metazoa;Eumetazoa;Bilateria;Protostomia;Spiralia;Lophotrochozoa;Mollusca;Bivalvia;Autobranchia;Heteroconchia;Euheterodonta;Imparidentia;Venerida;Veneroidea;Veneridae;Mercenaria;Mercenaria mercenaria
UdeM00000019653 6596 186.8 UdeM00000019653 XP_045163568.1 53.9 204 70 6 432 616 628 826 8.30E-44 186.8 6596 cellular organisms;Eukaryota;Opisthokonta;Metazoa;Eumetazoa;Bilateria;Protostomia;Spiralia;Lophotrochozoa;Mollusca;Bivalvia;Autobranchia;Heteroconchia;Euheterodonta;Imparidentia;Venerida;Veneroidea;Veneridae;Mercenaria;Mercenaria mercenaria
UdeM00000019655 6579 128.3 UdeM00000019655 XP_033761495.1 59.4 101 40 1 36 136 24 123 7.70E-27 128.3 6579 cellular organisms;Eukaryota;Opisthokonta;Metazoa;Eumetazoa;Bilateria;Protostomia;Spiralia;Lophotrochozoa;Mollusca;Bivalvia;Autobranchia;Pteriomorphia;Pectinida;Pectinoidea;Pectinidae;Pecten;Pecten maximus
UdeM00000019656 1735272 87.4 UdeM00000019656 XP_041362151.1 39.8 113 67 1 9 120 563 675 1.50E-14 87.4 1735272 cellular organisms;Eukaryota;Opisthokonta;Metazoa;Eumetazoa;Bilateria;Protostomia;Spiralia;Lophotrochozoa;Mollusca;Gastropoda;Neomphalina;Neomphaloidea;Peltospiridae;Gigantopelta;Gigantopelta aegis
UdeM00000019657 6596 293.1 UdeM00000019657 XP_045161225.1 62.8 239 81 2 1 238 64 295 3.40E-76 293.1 6596 cellular organisms;Eukaryota;Opisthokonta;Metazoa;Eumetazoa;Bilateria;Protostomia;Spiralia;Lophotrochozoa;Mollusca;Bivalvia;Autobranchia;Heteroconchia;Euhe

terodonta;Imparidentia;Venerida;Veneroidea;Veneridae;Mercenaria;Mercenaria mercenaria
UdeM00000019658 6579 954.5 UdeM00000019658 XP_033763909.1 65 695 238 1 197 891 103 792 9.40E-275 954.5 6579 cellular organisms;Eukaryota;Opisthokonta;Metazoa;Eumetazoa;Bilateria;Protostomia;Spiralia;Lophotrochozoa;Mollusca;Bivalvia;Autobranchia;Pteriomorphia;Pectinida;Pectinoidea;Pectinidae;Pecten;Pecten maximus
UdeM00000019659 400727 303.1 UdeM00000019659 XP_025080913.1 65.3 216 72 1 3 215 16 231 2.80E-79 303.1 400727 cellular organisms;Eukaryota;Opisthokonta;Metazoa;Eumetazoa;Bilateria;Protostomia;Spiralia;Lophotrochozoa;Mollusca;Gastropoda;Caenogastropoda;Architaenioglossa;Ampullarioidea;Ampullariidae;Pomacea;Pomacea canaliculata
UdeM00000019660 2607531 282.7 UdeM00000019660 XP_029638331.1 38.3 384 230 6 1 382 161 539 6.90E-73 282.7 2607531 cellular organisms;Eukaryota;Opisthokonta;Metazoa;Eumetazoa;Bilateria;Protostomia;Spiralia;Lophotrochozoa;Mollusca;Cephalopoda;Coleoidea;Octopodiformes;Octopoda;Incirrata;Octopodidae;Octopus;Octopus sinensis
UdeM00000019661 6573 143.7 UdeM00000019661 XP_021368946.1 31.9 263 165 3 112 367 1 256 5.60E-31 143.7 6573 cellular organisms;Eukaryota;Opisthokonta;Metazoa;Eumetazoa;Bilateria;Protostomia;Spiralia;Lophotrochozoa;Mollusca;Bivalvia;Autobranchia;Pteriomorphia;Pectinida;Pectinoidea;Pectinidae;Mizuhopecten;Mizuhopecten yessoensis
UdeM00000019662 6596 266.2 UdeM00000019662 XP_045161679.1 59 222 76 5 10 219 4 222 4.20E-68 266.2 6596 cellular organisms;Eukaryota;Opisthokonta;Metazoa;Eumetazoa;Bilateria;Protostomia;Spiralia;Lophotrochozoa;Mollusca;Bivalvia;Autobranchia;Heteroconchia;Euheterodonta;Imparidentia;Venerida;Veneroidea;Veneridae;Mercenaria;Mercenaria mercenaria
UdeM00000019664 158441 291.6 UdeM00000019664 XP_021955985.1 93.2 147 10 0 1 147 45 191 5.70E-76 291.6 158441 cellular organisms;Eukaryota;Opisthokonta;Metazoa;Eumetazoa;Bilateria;Protostomia;Ecdysozoa;Panarthropoda;Arthropoda;Mandibulata;Pancrustacea;Hexapoda;Collembola;Entomobryomorpha;Isotomoidea;Isotomidae;Proisotominae;Folsomia;Folsomia candida
UdeM00000019663 158441 379.8 UdeM00000019663 XP_021955985.1 94.8 191 10 0 1 191 1 191 2.10E-102 379.8 158441 cellular organisms;Eukaryota;Opisthokonta;Metazoa;Eumetazoa;Bilateria;Protostomia;Ecdysozoa;Panarthropoda;Arthropoda;Mandibulata;Pancrustacea;Hexapoda;Collembola;Entomobryomorpha;Isotomoidea;Isotomidae;Proisotominae;Folsomia;Folsomia candida
UdeM00000044490 6596 67.8 UdeM00000044490 XP_045198465.1 42.2 83 47 1 14 95 25 107 1.10E-08 67.8 6596 cellular organisms;Eukaryota;Opisthokonta;Metazoa;Eumetazoa;Bilateria;Protostomia;Spiralia;Lophotrochozoa;Mollusca;Bivalvia;Autobranchia;Heteroconchia;Euheterodonta;Imparidentia;Venerida;Veneroidea;Veneridae;Mercenaria;Mercenaria mercenaria
UdeM00000044213 50429 174.5 UdeM00000044213 XP_022795673.1 59.9 142 56 1 1 141 196 337 9.90E-41 174.5 50429 cellular organisms;Eukaryota;Opisthokonta;Metazoa;Eumetazoa;Cnidaria;Anthozoa;Hexa

corallia;Scleractinia;Astrocoeniina;Pocilloporidae;Stylophora;Stylophora pistillata
UdeM00000016568 6596 355.9 UdeM00000016568 XP_045177659.1 76.7 210 49 0 1 210 1 210 3.70E-95 355.9 6596 cellular organisms;Eukaryota;Opisthokonta;Metazoa;Eumetazoa;Bilateria;Protostomia;Spiralia;Lophotrochozoa;Mollusca;Bivalvia;Autobranchia;Heteroconchia;Euheterodonta;Imparidentia;Venerida;Veneroidea;Veneridae;Mercenaria;Mercenaria mercenaria
UdeM00000016569 6596 633.6 UdeM00000016569 XP_045177659.1 70 434 118 2 1 433 1 423 1.80E-178 633.6 6596 cellular organisms;Eukaryota;Opisthokonta;Metazoa;Eumetazoa;Bilateria;Protostomia;Spiralia;Lophotrochozoa;Mollusca;Bivalvia;Autobranchia;Heteroconchia;Euheterodonta;Imparidentia;Venerida;Veneroidea;Veneridae;Mercenaria;Mercenaria mercenaria
UdeM00000016570 6596 166 UdeM00000016570 XP_045179875.1 56.2 169 70 3 9 175 148 314 4.50E-38 166 6596 cellular organisms;Eukaryota;Opisthokonta;Metazoa;Eumetazoa;Bilateria;Protostomia;Spiralia;Lophotrochozoa;Mollusca;Bivalvia;Autobranchia;Heteroconchia;Euheterodonta;Imparidentia;Venerida;Veneroidea;Veneridae;Mercenaria;Mercenaria mercenaria
UdeM00000016571 6596 162.2 UdeM00000016571 XP_045179875.1 56.5 161 66 3 113 271 156 314 9.80E-37 162.2 6596 cellular organisms;Eukaryota;Opisthokonta;Metazoa;Eumetazoa;Bilateria;Protostomia;Spiralia;Lophotrochozoa;Mollusca;Bivalvia;Autobranchia;Heteroconchia;Euheterodonta;Imparidentia;Venerida;Veneroidea;Veneridae;Mercenaria;Mercenaria mercenaria
UdeM00000016572 6596 150.2 UdeM00000016572 XP_045179875.1 77.1 96 22 0 1 96 1 96 1.80E-33 150.2 6596 cellular organisms;Eukaryota;Opisthokonta;Metazoa;Eumetazoa;Bilateria;Protostomia;Spiralia;Lophotrochozoa;Mollusca;Bivalvia;Autobranchia;Heteroconchia;Euheterodonta;Imparidentia;Venerida;Veneroidea;Veneridae;Mercenaria;Mercenaria mercenaria
UdeM00000016573 6565 526.6 UdeM00000016573 XP_022308439.1 48.3 580 286 7 298 874 604 1172 6.50E-146 526.6 6565 cellular organisms;Eukaryota;Opisthokonta;Metazoa;Eumetazoa;Bilateria;Protostomia;Spiralia;Lophotrochozoa;Mollusca;Bivalvia;Autobranchia;Pteriomorphia;Ostreida;Ostreoidea;Ostreidae;Crassostrea;Crassostrea virginica
UdeM00000016574 6565 580.5 UdeM00000016574 XP_022308439.1 46.3 698 339 10 298 980 604 1280 4.10E-162 580.5 6565 cellular organisms;Eukaryota;Opisthokonta;Metazoa;Eumetazoa;Bilateria;Protostomia;Spiralia;Lophotrochozoa;Mollusca;Bivalvia;Autobranchia;Pteriomorphia;Ostreida;Ostreoidea;Ostreidae;Crassostrea;Crassostrea virginica
UdeM00000016576 6573 226.1 UdeM00000016576 XP_021368945.1 47.7 281 142 2 26 302 45 324 6.00E-56 226.1 6573 cellular organisms;Eukaryota;Opisthokonta;Metazoa;Eumetazoa;Bilateria;Protostomia;Spiralia;Lophotrochozoa;Mollusca;Bivalvia;Autobranchia;Pteriomorphia;Pectinida;Pectinoidea;Pectinidae;Mizuhopecten;Mizuhopecten yessoensis
UdeM00000016578 6579 498.4 UdeM00000016578 XP_033763088.1 48.9 1052 518 4 1 1036 2 1049 2.20E-137 498.4 6579 cellular organisms;Eukaryota;Opisthokonta;Metazoa;Eumetazoa;Bilateria;Protostomia;Spiralia;Lophotrochozoa;Mollusca;Bivalvia;Autobranchia;Pteriomorphia;Pectinida;Pectinoidea;Pectinidae;Pecten;Pecten maximus
UdeM00000016579 6579 491.5 UdeM00000016579 XP_033763088.1 48.5 1048 524 3 1 1032 2 1049 2.60E-135 491.5 6579 cellular

organisms;Eukaryota;Opisthokonta;Metazoa;Eumetazoa;Bilateria;Protostomia;Spiralia;Lophotrochozoa;Mollusca;Bivalvia;Autobranchia;Pteriomorphia;Pectinida;Pectinoidea;Pectinidae;Pecten;Pecten maximus
UdeM00000016580 6596 194.9 UdeM00000016580 XP_045161683.1 44.3 282 151 3 2 279 3 282 1.40E-46 194.9 6596 cellular organisms;Eukaryota;Opisthokonta;Metazoa;Eumetazoa;Bilateria;Protostomia;Spiralia;Lophotrochozoa;Mollusca;Bivalvia;Autobranchia;Heteroconchia;Euheterodonta;Imparidentia;Venerida;Veneroidea;Veneridae;Mercenaria;Mercenaria mercenaria
UdeM00000016582 6596 768.1 UdeM00000016582 XP_045162259.1 39.5 1247 663 26 261 1452 139 1348 2.00E-218 768.1 6596 cellular organisms;Eukaryota;Opisthokonta;Metazoa;Eumetazoa;Bilateria;Protostomia;Spiralia;Lophotrochozoa;Mollusca;Bivalvia;Autobranchia;Heteroconchia;Euheterodonta;Imparidentia;Venerida;Veneroidea;Veneridae;Mercenaria;Mercenaria mercenaria
UdeM00000016583 6596 458 UdeM00000016583 XP_045162261.1 35.3 781 450 15 1 739 177 944 3.10E-125 458 6596 cellular organisms;Eukaryota;Opisthokonta;Metazoa;Eumetazoa;Bilateria;Protostomia;Spiralia;Lophotrochozoa;Mollusca;Bivalvia;Autobranchia;Heteroconchia;Euheterodonta;Imparidentia;Venerida;Veneroidea;Veneridae;Mercenaria;Mercenaria mercenaria
UdeM00000016584 6596 455.7 UdeM00000016584 XP_045162261.1 35.3 771 445 14 20 749 187 944 1.50E-124 455.7 6596 cellular organisms;Eukaryota;Opisthokonta;Metazoa;Eumetazoa;Bilateria;Protostomia;Spiralia;Lophotrochozoa;Mollusca;Bivalvia;Autobranchia;Heteroconchia;Euheterodonta;Imparidentia;Venerida;Veneroidea;Veneridae;Mercenaria;Mercenaria mercenaria
UdeM00000016585 6596 458 UdeM00000016585 XP_045162261.1 35.3 781 450 15 1 739 177 944 3.10E-125 458 6596 cellular organisms;Eukaryota;Opisthokonta;Metazoa;Eumetazoa;Bilateria;Protostomia;Spiralia;Lophotrochozoa;Mollusca;Bivalvia;Autobranchia;Heteroconchia;Euheterodonta;Imparidentia;Venerida;Veneroidea;Veneridae;Mercenaria;Mercenaria mercenaria
UdeM00000016586 6596 455.7 UdeM00000016586 XP_045162261.1 35.3 771 445 14 20 749 187 944 1.50E-124 455.7 6596 cellular organisms;Eukaryota;Opisthokonta;Metazoa;Eumetazoa;Bilateria;Protostomia;Spiralia;Lophotrochozoa;Mollusca;Bivalvia;Autobranchia;Heteroconchia;Euheterodonta;Imparidentia;Venerida;Veneroidea;Veneridae;Mercenaria;Mercenaria mercenaria
UdeM00000016587 6596 422.2 UdeM00000016587 XP_045162261.1 29 1225 672 31 17 1061 123 1329 2.00E-114 422.2 6596 cellular organisms;Eukaryota;Opisthokonta;Metazoa;Eumetazoa;Bilateria;Protostomia;Spiralia;Lophotrochozoa;Mollusca;Bivalvia;Autobranchia;Heteroconchia;Euheterodonta;Imparidentia;Venerida;Veneroidea;Veneridae;Mercenaria;Mercenaria mercenaria
UdeM00000016588 6596 448.7 UdeM00000016588 XP_045162261.1 33.3 807 473 16 67 820 177 971 2.00E-122 448.7 6596 cellular organisms;Eukaryota;Opisthokonta;Metazoa;Eumetazoa;Bilateria;Protostomia;Spiralia;Lophotrochozoa;Mollusca;Bivalvia;Autobranchia;Heteroconchia;Euheterodonta;Imparidentia;Venerida;Veneroidea;Veneridae;Mercenaria;Mercenaria mercenaria
UdeM00000016589 6596 471.1 UdeM00000016589 XP_045162261.1 34.4 826 491 16 32 818 177 990 3.70E-129 471.1 6596 cellular organisms;Eukaryota;Opisthokonta;Metazoa;Eumetazoa;Bilateria;Protostomia;

Spiralia;Lophotrochozoa;Mollusca;Bivalvia;Autobranchia;Heteroconchia;Euheterodonta;Imparidentia;Venerida;Veneroidea;Veneridae;Mercenaria;Mercenaria mercenaria
UdeM00000016590 6596 473.4 UdeM00000016590 XP_045162261.1 34.3 826 499 14 32 825 177 990 7.40E-130 473.4 6596 cellular organisms;Eukaryota;Opisthokonta;Metazoa;Eumetazoa;Bilateria;Protostomia;Spiralia;Lophotrochozoa;Mollusca;Bivalvia;Autobranchia;Heteroconchia;Euheterodonta;Imparidentia;Venerida;Veneroidea;Veneridae;Mercenaria;Mercenaria mercenaria
UdeM00000016591 6596 195.3 UdeM00000016591 XP_045173241.1 33.2 467 265 14 186 631 433 873 2.60E-46 195.3 6596 cellular organisms;Eukaryota;Opisthokonta;Metazoa;Eumetazoa;Bilateria;Protostomia;Spiralia;Lophotrochozoa;Mollusca;Bivalvia;Autobranchia;Heteroconchia;Euheterodonta;Imparidentia;Venerida;Veneroidea;Veneridae;Mercenaria;Mercenaria mercenaria
UdeM00000016593 6596 158.3 UdeM00000016593 XP_045162256.1 35 592 304 14 19 544 64 640 2.80E-35 158.3 6596 cellular organisms;Eukaryota;Opisthokonta;Metazoa;Eumetazoa;Bilateria;Protostomia;Spiralia;Lophotrochozoa;Mollusca;Bivalvia;Autobranchia;Heteroconchia;Euheterodonta;Imparidentia;Venerida;Veneroidea;Veneridae;Mercenaria;Mercenaria mercenaria
UdeM00000016594 6579 282 UdeM00000016594 XP_033763324.1 32.2 597 354 15 545 1132 524 1078 3.50E-72 282 6579 cellular organisms;Eukaryota;Opisthokonta;Metazoa;Eumetazoa;Bilateria;Protostomia;Spiralia;Lophotrochozoa;Mollusca;Bivalvia;Autobranchia;Pteriomorphia;Pectinida;Pectinoidea;Pectinidae;Pecten;Pecten maximus
UdeM00000016595 6596 258.8 UdeM00000016595 XP_045162858.1 52.3 239 108 3 9 246 1480 1713 1.30E-65 258.8 6596 cellular organisms;Eukaryota;Opisthokonta;Metazoa;Eumetazoa;Bilateria;Protostomia;Spiralia;Lophotrochozoa;Mollusca;Bivalvia;Autobranchia;Heteroconchia;Euheterodonta;Imparidentia;Venerida;Veneroidea;Veneridae;Mercenaria;Mercenaria mercenaria
UdeM00000016596 6565 216.1 UdeM00000016596 XP_022297447.1 53.2 201 78 2 8 192 81 281 5.00E-53 216.1 6565 cellular organisms;Eukaryota;Opisthokonta;Metazoa;Eumetazoa;Bilateria;Protostomia;Spiralia;Lophotrochozoa;Mollusca;Bivalvia;Autobranchia;Pteriomorphia;Ostreida;Ostreoidea;Ostreidae;Crassostrea;Crassostrea virginica
UdeM00000016597 45264 167.2 UdeM00000016597 XP_044166166.1 49.3 150 75 1 1 149 667 816 2.20E-38 167.2 45264 cellular organisms;Eukaryota;Opisthokonta;Metazoa;Eumetazoa;Cnidaria;Anthozoa;Hexacorallia;Scleractinia;Astrocoeniina;Acroporidae;Acropora;Acropora millepora
UdeM00000016598 6596 547 UdeM00000016598 XP_045191668.1 30.2 1262 737 43 527 1687 81 1299 9.20E-152 547 6596 cellular organisms;Eukaryota;Opisthokonta;Metazoa;Eumetazoa;Bilateria;Protostomia;Spiralia;Lophotrochozoa;Mollusca;Bivalvia;Autobranchia;Heteroconchia;Euheterodonta;Imparidentia;Venerida;Veneroidea;Veneridae;Mercenaria;Mercenaria mercenaria
UdeM00000016599 6565 61.6 UdeM00000016599 XP_022343311.1 35.4 99 57 3 3 100 103 195 7.30E-07 61.6 6565 cellular organisms;Eukaryota;Opisthokonta;Metazoa;Eumetazoa;Bilateria;Protostomia;Spiralia;Lophotrochozoa;Mollusca;Bivalvia;Autobranchia;Pteriomorphia;Ostreida;Ostreoidea;Ostreidae;Crassostrea;Crassostrea virginica

UdeM00000016602 225164 99.4 UdeM00000016602 XP_009062103.1 27.2
195 139 2 1 194 1 193 6.00E-18 99.4 225164
cellular
organisms;Eukaryota;Opisthokonta;Metazoa;Eumetazoa;Bilateria;Protostomia;
Spiralia;Lophotrochozoa;Mollusca;Gastropoda;Patellogastropoda;Lottioidea;
Lottiidae;Lottia;Lottia gigantea
UdeM00000016605 6596 59.7 UdeM00000016605 XP_045163980.1 36.5 126
68 3 75 192 49 170 4.90E-06 59.7 6596 cellular
organisms;Eukaryota;Opisthokonta;Metazoa;Eumetazoa;Bilateria;Protostomia;
Spiralia;Lophotrochozoa;Mollusca;Bivalvia;Autobranchia;Heteroconchia;Euhe
terodonta;Imparidentia;Venerida;Veneroidea;Veneridae;Mercenaria;Mercenari
a mercenaria
UdeM00000016606 6596 202.6 UdeM00000016606 XP_045167035.1 46.8 186
98 1 192 377 155 339 9.00E-49 202.6 6596 cellular
organisms;Eukaryota;Opisthokonta;Metazoa;Eumetazoa;Bilateria;Protostomia;
Spiralia;Lophotrochozoa;Mollusca;Bivalvia;Autobranchia;Heteroconchia;Euhe
terodonta;Imparidentia;Venerida;Veneroidea;Veneridae;Mercenaria;Mercenari
a mercenaria
UdeM00000016607 6565 223.4 UdeM00000016607 XP_022286598.1 44.3 323
149 9 1 310 1 305 4.00E-55 223.4 6565 cellular
organisms;Eukaryota;Opisthokonta;Metazoa;Eumetazoa;Bilateria;Protostomia;
Spiralia;Lophotrochozoa;Mollusca;Bivalvia;Autobranchia;Pteriomorphia;Ostr
eida;Ostreoidea;Ostreidae;Crassostrea;Crassostrea virginica
UdeM00000016609 6565 404.4 UdeM00000016609 XP_022320668.1 69.6 270
82 0 20 289 441 710 1.20E-109 404.4 6565 cellular
organisms;Eukaryota;Opisthokonta;Metazoa;Eumetazoa;Bilateria;Protostomia;
Spiralia;Lophotrochozoa;Mollusca;Bivalvia;Autobranchia;Pteriomorphia;Ostr
eida;Ostreoidea;Ostreidae;Crassostrea;Crassostrea virginica
UdeM00000016610 6596 190.3 UdeM00000016610 XP_045167035.1 44.1 186
103 1 218 403 155 339 4.90E-45 190.3 6596 cellular
organisms;Eukaryota;Opisthokonta;Metazoa;Eumetazoa;Bilateria;Protostomia;
Spiralia;Lophotrochozoa;Mollusca;Bivalvia;Autobranchia;Heteroconchia;Euhe
terodonta;Imparidentia;Venerida;Veneroidea;Veneridae;Mercenaria;Mercenari
a mercenaria
UdeM00000016611 6596 202.6 UdeM00000016611 XP_045167035.1 41.3 242
117 4 190 408 100 339 9.70E-49 202.6 6596 cellular
organisms;Eukaryota;Opisthokonta;Metazoa;Eumetazoa;Bilateria;Protostomia;
Spiralia;Lophotrochozoa;Mollusca;Bivalvia;Autobranchia;Heteroconchia;Euhe
terodonta;Imparidentia;Venerida;Veneroidea;Veneridae;Mercenaria;Mercenari
a mercenaria
UdeM00000016613 6596 863.2 UdeM00000016613 XP_045161992.1 40.2 1178
597 23 8 1116 16 1155 3.60E-247 863.2 6596 cellular
organisms;Eukaryota;Opisthokonta;Metazoa;Eumetazoa;Bilateria;Protostomia;
Spiralia;Lophotrochozoa;Mollusca;Bivalvia;Autobranchia;Heteroconchia;Euhe
terodonta;Imparidentia;Venerida;Veneroidea;Veneridae;Mercenaria;Mercenari
a mercenaria
UdeM00000016614 6596 781.6 UdeM00000016614 XP_045161992.1 37.4 1226
506 19 8 1002 16 1211 1.20E-222 781.6 6596 cellular
organisms;Eukaryota;Opisthokonta;Metazoa;Eumetazoa;Bilateria;Protostomia;
Spiralia;Lophotrochozoa;Mollusca;Bivalvia;Autobranchia;Heteroconchia;Euhe
terodonta;Imparidentia;Venerida;Veneroidea;Veneridae;Mercenaria;Mercenari
a mercenaria
UdeM00000016615 6596 199.1 UdeM00000016615 XP_045161738.1 89.9 109
11 0 1 109 1 109 6.60E-48 199.1 6596 cellular

organisms;Eukaryota;Opisthokonta;Metazoa;Eumetazoa;Bilateria;Protostomia;Spiralia;Lophotrochozoa;Mollusca;Bivalvia;Autobranchia;Heteroconchia;Euheterodonta;Imparidentia;Venerida;Veneroidea;Veneridae;Mercenaria;Mercenaria mercenaria
UdeM00000016616 6579 320.9 UdeM00000016616 XP_033764146.1 64.5 228
80 1 1 227 1 228 1.30E-84 320.9 6579 cellular
organisms;Eukaryota;Opisthokonta;Metazoa;Eumetazoa;Bilateria;Protostomia;Spiralia;Lophotrochozoa;Mollusca;Bivalvia;Autobranchia;Pteriomorphia;Pectinida;Pectinoidea;Pectinidae;Pecten;Pecten maximus
UdeM00000016620 6596 1208.4 UdeM00000016620 XP_045162575.1 80
734 146 1 2 734 122 855 0.00E+00 1208.4 6596
cellular
organisms;Eukaryota;Opisthokonta;Metazoa;Eumetazoa;Bilateria;Protostomia;Spiralia;Lophotrochozoa;Mollusca;Bivalvia;Autobranchia;Heteroconchia;Euheterodonta;Imparidentia;Venerida;Veneroidea;Veneridae;Mercenaria;Mercenaria mercenaria
UdeM00000016621 2607531 310.1 UdeM00000016621 XP_029657462.1 56.9
276 119 0 92 367 7 282 3.80E-81 310.1 2607531
cellular
organisms;Eukaryota;Opisthokonta;Metazoa;Eumetazoa;Bilateria;Protostomia;Spiralia;Lophotrochozoa;Mollusca;Cephalopoda;Coleoidea;Octopodiformes;Octopoda;Incirrata;Octopodidae;Octopus;Octopus sinensis
UdeM00000016622 29159 84 UdeM00000016622 XP_011452539.2 43.6 117
59 4 1 111 1 116 1.40E-13 84 29159 cellular
organisms;Eukaryota;Opisthokonta;Metazoa;Eumetazoa;Bilateria;Protostomia;Spiralia;Lophotrochozoa;Mollusca;Bivalvia;Autobranchia;Pteriomorphia;Ostreida;Ostreoidea;Ostreidae;Crassostrea;Crassostrea gigas
UdeM00000016623 6596 141 UdeM00000016623 XP_045215532.1 32.4 262
162 8 16 269 2 256 2.80E-30 141 6596 cellular
organisms;Eukaryota;Opisthokonta;Metazoa;Eumetazoa;Bilateria;Protostomia;Spiralia;Lophotrochozoa;Mollusca;Bivalvia;Autobranchia;Heteroconchia;Euheterodonta;Imparidentia;Venerida;Veneroidea;Veneridae;Mercenaria;Mercenaria mercenaria
UdeM00000016624 6596 141 UdeM00000016624 XP_045215532.1 32.4 262
162 8 6 259 2 256 2.70E-30 141 6596 cellular
organisms;Eukaryota;Opisthokonta;Metazoa;Eumetazoa;Bilateria;Protostomia;Spiralia;Lophotrochozoa;Mollusca;Bivalvia;Autobranchia;Heteroconchia;Euheterodonta;Imparidentia;Venerida;Veneroidea;Veneridae;Mercenaria;Mercenaria mercenaria
UdeM00000016625 189513 217.6 UdeM00000016625 XP_044740614.1 67.8
152 48 1 3 153 5 156 1.10E-53 217.6 189513
cellular
organisms;Eukaryota;Opisthokonta;Metazoa;Eumetazoa;Bilateria;Protostomia;Ecdysozoa;Panarthropoda;Arthropoda;Mandibulata;Pancrustacea;Hexapoda;Insecta;Dicondylia;Pterygota;Neoptera;Endopterygota;Neuropterida;Neuroptera;Hemerobiiformia;Chrysopidae;Chrysopinae;Chrysoperla;Chrysoperla carnea
UdeM00000016626 400727 2246.9 UdeM00000016626 XP_025081588.1
80 1810 315 12 118 1893 77 1873 0.00E+00 2246.9
400727 cellular
organisms;Eukaryota;Opisthokonta;Metazoa;Eumetazoa;Bilateria;Protostomia;Spiralia;Lophotrochozoa;Mollusca;Gastropoda;Caenogastropoda;Architaenioglossa;Ampullarioidea;Ampullariidae;Pomacea;Pomacea canaliculata
UdeM00000016627 6596 255 UdeM00000016627 XP_045162558.1 81 142
27 0 1 142 2 143 5.80E-65 255 6596 cellular

organisms;Eukaryota;Opisthokonta;Metazoa;Eumetazoa;Bilateria;Protostomia;Spiralia;Lophotrochozoa;Mollusca;Bivalvia;Autobranchia;Heteroconchia;Euheterodonta;Imparidentia;Venerida;Veneroidea;Veneridae;Mercenaria;Mercenaria mercenaria
UdeM00000016628 6573 254.6 UdeM00000016628 XP_021355436.1 73.1 175 45 1 1 173 171 345 9.10E-65 254.6 6573 cellular organisms;Eukaryota;Opisthokonta;Metazoa;Eumetazoa;Bilateria;Protostomia;Spiralia;Lophotrochozoa;Mollusca;Bivalvia;Autobranchia;Pteriomorphia;Pectinida;Pectinoidea;Pectinidae;Mizuhopecten;Mizuhopecten yessoensis
UdeM00000016629 6579 157.1 UdeM00000016629 XP_033764276.1 42.3 194 105 2 1 194 14 200 2.30E-35 157.1 6579 cellular organisms;Eukaryota;Opisthokonta;Metazoa;Eumetazoa;Bilateria;Protostomia;Spiralia;Lophotrochozoa;Mollusca;Bivalvia;Autobranchia;Pteriomorphia;Pectinida;Pectinoidea;Pectinidae;Pecten;Pecten maximus
UdeM00000016630 6596 220.3 UdeM00000016630 XP_045163995.1 47.6 225 117 1 211 434 16 240 4.70E-54 220.3 6596 cellular organisms;Eukaryota;Opisthokonta;Metazoa;Eumetazoa;Bilateria;Protostomia;Spiralia;Lophotrochozoa;Mollusca;Bivalvia;Autobranchia;Heteroconchia;Euheterodonta;Imparidentia;Venerida;Veneroidea;Veneridae;Mercenaria;Mercenaria mercenaria
UdeM00000016631 6596 220.7 UdeM00000016631 XP_045163995.1 47.2 218 114 1 167 383 23 240 3.20E-54 220.7 6596 cellular organisms;Eukaryota;Opisthokonta;Metazoa;Eumetazoa;Bilateria;Protostomia;Spiralia;Lophotrochozoa;Mollusca;Bivalvia;Autobranchia;Heteroconchia;Euheterodonta;Imparidentia;Venerida;Veneroidea;Veneridae;Mercenaria;Mercenaria mercenaria
UdeM00000016632 6596 245 UdeM00000016632 XP_045163995.1 55.1 214 95 1 27 239 27 240 9.90E-62 245 6596 cellular organisms;Eukaryota;Opisthokonta;Metazoa;Eumetazoa;Bilateria;Protostomia;Spiralia;Lophotrochozoa;Mollusca;Bivalvia;Autobranchia;Heteroconchia;Euheterodonta;Imparidentia;Venerida;Veneroidea;Veneridae;Mercenaria;Mercenaria mercenaria
UdeM00000016634 6565 231.1 UdeM00000016634 XP_022286723.1 39.9 311 175 6 7 317 4 302 2.00E-57 231.1 6565 cellular organisms;Eukaryota;Opisthokonta;Metazoa;Eumetazoa;Bilateria;Protostomia;Spiralia;Lophotrochozoa;Mollusca;Bivalvia;Autobranchia;Pteriomorphia;Ostreida;Ostreoidea;Ostreidae;Crassostrea;Crassostrea virginica
UdeM00000016635 45264 395.2 UdeM00000016635 XP_044165535.1 49.6 371 183 1 1 367 158 528 9.90E-107 395.2 45264 cellular organisms;Eukaryota;Opisthokonta;Metazoa;Eumetazoa;Cnidaria;Anthozoa;Hexacorallia;Scleractinia;Astrocoeniina;Acroporidae;Acropora;Acropora millepora
UdeM00000016636 6565 276.2 UdeM00000016636 XP_022339386.1 95.9 145 6 0 1 145 1 145 2.50E-71 276.2 6565 cellular organisms;Eukaryota;Opisthokonta;Metazoa;Eumetazoa;Bilateria;Protostomia;Spiralia;Lophotrochozoa;Mollusca;Bivalvia;Autobranchia;Pteriomorphia;Ostreida;Ostreoidea;Ostreidae;Crassostrea;Crassostrea virginica
UdeM00000016637 6596 194.9 UdeM00000016637 XP_045176664.1 34.7 346 212 9 20 356 227 567 2.50E-46 194.9 6596 cellular organisms;Eukaryota;Opisthokonta;Metazoa;Eumetazoa;Bilateria;Protostomia;Spiralia;Lophotrochozoa;Mollusca;Bivalvia;Autobranchia;Heteroconchia;Euheterodonta;Imparidentia;Venerida;Veneroidea;Veneridae;Mercenaria;Mercenaria mercenaria

UdeM00000016638 6596 112.5 UdeM00000016638 XP_045163991.1 35.9 220
114 4 41 260 56 248 8.50E-22 112.5 6596 cellular
organisms;Eukaryota;Opisthokonta;Metazoa;Eumetazoa;Bilateria;Protostomia;
Spiralia;Lophotrochozoa;Mollusca;Bivalvia;Autobranchia;Heteroconchia;Euhe
terodonta;Imparidentia;Venerida;Veneroidea;Veneridae;Mercenaria;Mercenari
a mercenaria
UdeM00000016639 6596 112.5 UdeM00000016639 XP_045163991.1 35.9 220
114 4 42 261 56 248 8.50E-22 112.5 6596 cellular
organisms;Eukaryota;Opisthokonta;Metazoa;Eumetazoa;Bilateria;Protostomia;
Spiralia;Lophotrochozoa;Mollusca;Bivalvia;Autobranchia;Heteroconchia;Euhe
terodonta;Imparidentia;Venerida;Veneroidea;Veneridae;Mercenaria;Mercenari
a mercenaria
UdeM00000016640 6596 550.8 UdeM00000016640 XP_045163440.1 70.2 372
111 0 1 372 50 421 1.30E-153 550.8 6596 cellular
organisms;Eukaryota;Opisthokonta;Metazoa;Eumetazoa;Bilateria;Protostomia;
Spiralia;Lophotrochozoa;Mollusca;Bivalvia;Autobranchia;Heteroconchia;Euhe
terodonta;Imparidentia;Venerida;Veneroidea;Veneridae;Mercenaria;Mercenari
a mercenaria
UdeM00000016641 6596 595.9 UdeM00000016641 XP_045163440.1 67.2 421
138 0 1 421 1 421 4.10E-167 595.9 6596 cellular
organisms;Eukaryota;Opisthokonta;Metazoa;Eumetazoa;Bilateria;Protostomia;
Spiralia;Lophotrochozoa;Mollusca;Bivalvia;Autobranchia;Heteroconchia;Euhe
terodonta;Imparidentia;Venerida;Veneroidea;Veneridae;Mercenaria;Mercenari
a mercenaria
UdeM00000016642 6565 186.4 UdeM00000016642 XP_022335889.1 71.5 123
34 1 11 133 12 133 2.90E-44 186.4 6565 cellular
organisms;Eukaryota;Opisthokonta;Metazoa;Eumetazoa;Bilateria;Protostomia;
Spiralia;Lophotrochozoa;Mollusca;Bivalvia;Autobranchia;Pteriomorphia;Ostr
eida;Ostreoidea;Ostreidae;Crassostrea;Crassostrea virginica
UdeM00000016643 6596 1039.3 UdeM00000016643 XP_045161343.1 68
731 222 5 1 726 213 936 2.40E-300 1039.3 6596
cellular
organisms;Eukaryota;Opisthokonta;Metazoa;Eumetazoa;Bilateria;Protostomia;
Spiralia;Lophotrochozoa;Mollusca;Bivalvia;Autobranchia;Heteroconchia;Euhe
terodonta;Imparidentia;Venerida;Veneroidea;Veneridae;Mercenaria;Mercenari
a mercenaria
UdeM00000016644 29159 224.2 UdeM00000016644 XP_011432760.1 60.7 178
69 1 6 182 8 185 1.40E-55 224.2 29159 cellular
organisms;Eukaryota;Opisthokonta;Metazoa;Eumetazoa;Bilateria;Protostomia;
Spiralia;Lophotrochozoa;Mollusca;Bivalvia;Autobranchia;Pteriomorphia;Ostr
eida;Ostreoidea;Ostreidae;Crassostrea;Crassostrea gigas
UdeM00000016645 1735272 198.4 UdeM00000016645 XP_041377218.1 64.8
142 50 0 1 142 1 142 6.30E-48 198.4 1735272
cellular
organisms;Eukaryota;Opisthokonta;Metazoa;Eumetazoa;Bilateria;Protostomia;
Spiralia;Lophotrochozoa;Mollusca;Gastropoda;Neomphalina;Neomphaloidea;Pel
tospiridae;Gigantopelta;Gigantopelta aegis
UdeM00000016646 6596 194.1 UdeM00000016646 XP_045162566.1 66.9 133
44 0 11 143 10 142 1.20E-46 194.1 6596 cellular
organisms;Eukaryota;Opisthokonta;Metazoa;Eumetazoa;Bilateria;Protostomia;
Spiralia;Lophotrochozoa;Mollusca;Bivalvia;Autobranchia;Heteroconchia;Euhe
terodonta;Imparidentia;Venerida;Veneroidea;Veneridae;Mercenaria;Mercenari
a mercenaria

UdeM00000016647 29159 236.1 UdeM00000016647 XP_034335856.1 35.9 463 246 8 1 446 89 517 1.10E-58 236.1 29159 cellular organisms;Eukaryota;Opisthokonta;Metazoa;Eumetazoa;Bilateria;Protostomia;Spiralia;Lophotrochozoa;Mollusca;Bivalvia;Autobranchia;Pteriomorphia;Ostreida;Ostreoidea;Ostreidae;Crassostrea;Crassostrea gigas
UdeM00000016648 6596 491.1 UdeM00000016648 XP_045162705.1 74.8 301 75 1 1 301 1 300 1.00E-135 491.1 6596 cellular organisms;Eukaryota;Opisthokonta;Metazoa;Eumetazoa;Bilateria;Protostomia;Spiralia;Lophotrochozoa;Mollusca;Bivalvia;Autobranchia;Heteroconchia;Euheterodonta;Imparidentia;Venerida;Veneroidea;Veneridae;Mercenaria;Mercenaria mercenaria
UdeM00000016649 6596 129.8 UdeM00000016649 XP_045209459.1 41.2 165 94 1 77 241 32 193 4.70E-27 129.8 6596 cellular organisms;Eukaryota;Opisthokonta;Metazoa;Eumetazoa;Bilateria;Protostomia;Spiralia;Lophotrochozoa;Mollusca;Bivalvia;Autobranchia;Heteroconchia;Euheterodonta;Imparidentia;Venerida;Veneroidea;Veneridae;Mercenaria;Mercenaria mercenaria
UdeM00000016650 6596 237.7 UdeM00000016650 XP_045163027.1 90.9 143 11 1 1 143 1 141 9.50E-60 237.7 6596 cellular organisms;Eukaryota;Opisthokonta;Metazoa;Eumetazoa;Bilateria;Protostomia;Spiralia;Lophotrochozoa;Mollusca;Bivalvia;Autobranchia;Heteroconchia;Euheterodonta;Imparidentia;Venerida;Veneroidea;Veneridae;Mercenaria;Mercenaria mercenaria
UdeM00000016651 6596 612.8 UdeM00000016651 XP_045162994.1 63.7 499 170 4 20 508 32 529 3.90E-172 612.8 6596 cellular organisms;Eukaryota;Opisthokonta;Metazoa;Eumetazoa;Bilateria;Protostomia;Spiralia;Lophotrochozoa;Mollusca;Bivalvia;Autobranchia;Heteroconchia;Euheterodonta;Imparidentia;Venerida;Veneroidea;Veneridae;Mercenaria;Mercenaria mercenaria
UdeM00000016652 225164 328.2 UdeM00000016652 XP_009048161.1 48.6 385 113 2 35 334 83 467 1.20E-86 328.2 225164 cellular organisms;Eukaryota;Opisthokonta;Metazoa;Eumetazoa;Bilateria;Protostomia;Spiralia;Lophotrochozoa;Mollusca;Gastropoda;Patellogastropoda;Lottioidea;Lottiidae;Lottia;Lottia gigantea
UdeM00000016653 6596 493.8 UdeM00000016653 XP_045161412.1 71.4 322 64 1 1 294 39 360 1.50E-136 493.8 6596 cellular organisms;Eukaryota;Opisthokonta;Metazoa;Eumetazoa;Bilateria;Protostomia;Spiralia;Lophotrochozoa;Mollusca;Bivalvia;Autobranchia;Heteroconchia;Euheterodonta;Imparidentia;Venerida;Veneroidea;Veneridae;Mercenaria;Mercenaria mercenaria
UdeM00000016654 6596 431.8 UdeM00000016654 XP_045161409.1 66.2 296 97 1 2 294 54 349 7.70E-118 431.8 6596 cellular organisms;Eukaryota;Opisthokonta;Metazoa;Eumetazoa;Bilateria;Protostomia;Spiralia;Lophotrochozoa;Mollusca;Bivalvia;Autobranchia;Heteroconchia;Euheterodonta;Imparidentia;Venerida;Veneroidea;Veneridae;Mercenaria;Mercenaria mercenaria
UdeM00000016655 6596 444.1 UdeM00000016655 XP_045161409.1 66 306 101 1 1 303 44 349 1.50E-121 444.1 6596 cellular organisms;Eukaryota;Opisthokonta;Metazoa;Eumetazoa;Bilateria;Protostomia;Spiralia;Lophotrochozoa;Mollusca;Bivalvia;Autobranchia;Heteroconchia;Euheterodonta;Imparidentia;Venerida;Veneroidea;Veneridae;Mercenaria;Mercenaria mercenaria

UdeM00000016656	6596	106.7	UdeM00000016656	XP_045200636.1	28.2	238	155	7	215	448	117	342	1.20E-19	106.7	6596	cellular organisms;Eukaryota;Opisthokonta;Metazoa;Eumetazoa;Bilateria;Protostomia;Spiralia;Lophotrochozoa;Mollusca;Bivalvia;Autobranchia;Heteroconchia;Euheterodonta;Imparidentia;Venerida;Veneroidea;Veneridae;Mercenaria;Mercenaria mercenaria
UdeM00000016657	6596	496.9	UdeM00000016657	XP_045161310.1	49	598	259	7	23	590	3	584	3.60E-137	496.9	6596	cellular organisms;Eukaryota;Opisthokonta;Metazoa;Eumetazoa;Bilateria;Protostomia;Spiralia;Lophotrochozoa;Mollusca;Bivalvia;Autobranchia;Heteroconchia;Euheterodonta;Imparidentia;Venerida;Veneroidea;Veneridae;Mercenaria;Mercenaria mercenaria
UdeM00000016658	225164	445.3	UdeM00000016658	XP_009064943.1	53.2	412	175	3	30	423	2	413	8.80E-122	445.3	225164	cellular organisms;Eukaryota;Opisthokonta;Metazoa;Eumetazoa;Bilateria;Protostomia;Spiralia;Lophotrochozoa;Mollusca;Gastropoda;Patellogastropoda;Lottioidea;Lottiidae;Lottia;Lottia gigantea
UdeM00000016659	6579	102.1	UdeM00000016659	XP_033764037.1	46	126	60	1	20	137	18	143	6.90E-19	102.1	6579	cellular organisms;Eukaryota;Opisthokonta;Metazoa;Eumetazoa;Bilateria;Protostomia;Spiralia;Lophotrochozoa;Mollusca;Bivalvia;Autobranchia;Pteriomorphia;Pectinida;Pectinoidea;Pectinidae;Pecten;Pecten maximus
UdeM00000016661	6573	543.1	UdeM00000016661	XP_021353256.1	89	292	32	0	1	292	1	292	2.50E-151	543.1	6573	cellular organisms;Eukaryota;Opisthokonta;Metazoa;Eumetazoa;Bilateria;Protostomia;Spiralia;Lophotrochozoa;Mollusca;Bivalvia;Autobranchia;Pteriomorphia;Pectinida;Pectinoidea;Pectinidae;Mizuhopecten;Mizuhopecten yessoensis
UdeM00000016662	1735272	849.7	UdeM00000016662	XP_041377555.1	71.9	559	146	2	1	555	119	670	2.20E-243	849.7	1735272	cellular organisms;Eukaryota;Opisthokonta;Metazoa;Eumetazoa;Bilateria;Protostomia;Spiralia;Lophotrochozoa;Mollusca;Gastropoda;Neomphalina;Neomphaloidea;Peltospiridae;Gigantopelta;Gigantopelta aegis
UdeM00000016663	1735272	374.4	UdeM00000016663	XP_041355564.1	52.9	306	143	1	25	329	17	322	1.50E-100	374.4	1735272	cellular organisms;Eukaryota;Opisthokonta;Metazoa;Eumetazoa;Bilateria;Protostomia;Spiralia;Lophotrochozoa;Mollusca;Gastropoda;Neomphalina;Neomphaloidea;Peltospiridae;Gigantopelta;Gigantopelta aegis
UdeM00000016664	6573	919.8	UdeM00000016664	XP_021366576.1	70.7	628	175	4	6	628	20	643	1.80E-264	919.8	6573	cellular organisms;Eukaryota;Opisthokonta;Metazoa;Eumetazoa;Bilateria;Protostomia;Spiralia;Lophotrochozoa;Mollusca;Bivalvia;Autobranchia;Pteriomorphia;Pectinida;Pectinoidea;Pectinidae;Mizuhopecten;Mizuhopecten yessoensis
UdeM00000016665	6573	928.7	UdeM00000016665	XP_021366576.1	70.2	642	182	4	8	644	6	643	4.00E-267	928.7	6573	cellular organisms;Eukaryota;Opisthokonta;Metazoa;Eumetazoa;Bilateria;Protostomia;Spiralia;Lophotrochozoa;Mollusca;Bivalvia;Autobranchia;Pteriomorphia;Pectinida;Pectinoidea;Pectinidae;Mizuhopecten;Mizuhopecten yessoensis
UdeM00000016666	6579	924.5	UdeM00000016666	XP_033763798.1	70.6	630	175	5	5	626	4	631	7.60E-266	924.5	6579	cellular organisms;Eukaryota;Opisthokonta;Metazoa;Eumetazoa;Bilateria;Protostomia;

Spiralia;Lophotrochozoa;Mollusca;Bivalvia;Autobranchia;Pteriomorphia;Pectinida;Pectinoidea;Pectinidae;Pecten;Pecten maximus
UdeM00000016667 6579 884.8 UdeM00000016667 XP_033763798.1 69.1 605 181 3 42 642 43 645 6.70E-254 884.8 6579 cellular organisms;Eukaryota;Opisthokonta;Metazoa;Eumetazoa;Bilateria;Protostomia;Spiralia;Lophotrochozoa;Mollusca;Bivalvia;Autobranchia;Pteriomorphia;Pectinida;Pectinoidea;Pectinidae;Pecten;Pecten maximus
UdeM00000016668 6596 610.1 UdeM00000016668 XP_045161926.1 69.4 405 123 1 3 407 265 668 2.00E-171 610.1 6596 cellular organisms;Eukaryota;Opisthokonta;Metazoa;Eumetazoa;Bilateria;Protostomia;Spiralia;Lophotrochozoa;Mollusca;Bivalvia;Autobranchia;Heteroconchia;Euheterodonta;Imparidentia;Venerida;Veneroidea;Veneridae;Mercenaria;Mercenaria mercenaria
UdeM00000016669 6579 273.9 UdeM00000016669 XP_033763798.1 66.7 192 60 2 28 215 42 233 1.80E-70 273.9 6579 cellular organisms;Eukaryota;Opisthokonta;Metazoa;Eumetazoa;Bilateria;Protostomia;Spiralia;Lophotrochozoa;Mollusca;Bivalvia;Autobranchia;Pteriomorphia;Pectinida;Pectinoidea;Pectinidae;Pecten;Pecten maximus
UdeM00000016670 1415580 646.7 UdeM00000016670 XP_030075314.1 52.2 642 287 4 1 639 1 625 3.00E-182 646.7 1415580 cellular organisms;Eukaryota;Opisthokonta;Metazoa;Eumetazoa;Bilateria;Deuterostomia;Chordata;Craniata;Vertebrata;Gnathostomata;Teleostomi;Euteleostomi;Sarcopterygii;Dipnotetrapodomorpha;Tetrapoda;Amphibia;Gymnophiona;Siphonopidae;Microcaecilia;Microcaecilia unicolor
UdeM00000016671 6579 916.4 UdeM00000016671 XP_033763798.1 70.1 648 174 8 5 640 6 645 2.10E-263 916.4 6579 cellular organisms;Eukaryota;Opisthokonta;Metazoa;Eumetazoa;Bilateria;Protostomia;Spiralia;Lophotrochozoa;Mollusca;Bivalvia;Autobranchia;Pteriomorphia;Pectinida;Pectinoidea;Pectinidae;Pecten;Pecten maximus
UdeM00000016672 6596 375.6 UdeM00000016672 XP_045161926.1 64.8 267 92 2 1 267 406 670 5.80E-101 375.6 6596 cellular organisms;Eukaryota;Opisthokonta;Metazoa;Eumetazoa;Bilateria;Protostomia;Spiralia;Lophotrochozoa;Mollusca;Bivalvia;Autobranchia;Heteroconchia;Euheterodonta;Imparidentia;Venerida;Veneroidea;Veneridae;Mercenaria;Mercenaria mercenaria
UdeM00000016673 6579 339.7 UdeM00000016673 XP_033763798.1 64.7 255 82 5 24 271 28 281 3.40E-90 339.7 6579 cellular organisms;Eukaryota;Opisthokonta;Metazoa;Eumetazoa;Bilateria;Protostomia;Spiralia;Lophotrochozoa;Mollusca;Bivalvia;Autobranchia;Pteriomorphia;Pectinida;Pectinoidea;Pectinidae;Pecten;Pecten maximus
UdeM00000016674 6573 848.6 UdeM00000016674 XP_021366576.1 64.7 646 210 8 5 640 6 643 5.30E-243 848.6 6573 cellular organisms;Eukaryota;Opisthokonta;Metazoa;Eumetazoa;Bilateria;Protostomia;Spiralia;Lophotrochozoa;Mollusca;Bivalvia;Autobranchia;Pteriomorphia;Pectinida;Pectinoidea;Pectinidae;Mizuhopecten;Mizuhopecten yessoensis
UdeM00000016676 6596 67.8 UdeM00000016676 XP_045161926.1 61.7 47 18 0 22 68 457 503 7.50E-09 67.8 6596 cellular organisms;Eukaryota;Opisthokonta;Metazoa;Eumetazoa;Bilateria;Protostomia;Spiralia;Lophotrochozoa;Mollusca;Bivalvia;Autobranchia;Heteroconchia;Euheterodonta;Imparidentia;Venerida;Veneroidea;Veneridae;Mercenaria;Mercenaria mercenaria
UdeM00000016675 6596 67.4 UdeM00000016675 XP_045161926.1 63 46 17 0 30 75 458 503 1.10E-08 67.4 6596 cellular

organisms;Eukaryota;Opisthokonta;Metazoa;Eumetazoa;Bilateria;Protostomia;
Spiralia;Lophotrochozoa;Mollusca;Bivalvia;Autobranchia;Heteroconchia;Euheterodonta;Imparidentia;Venerida;Veneroidea;Veneridae;Mercenaria;Mercenaria mercenaria
UdeM00000016678 6579 827.4 UdeM00000016678 XP_033763798.1 62.4 644 230 8 64 700 7 645 1.40E-236 827.4 6579 cellular organisms;Eukaryota;Opisthokonta;Metazoa;Eumetazoa;Bilateria;Protostomia;Spiralia;Lophotrochozoa;Mollusca;Bivalvia;Autobranchia;Pteriomorphia;Pectinida;Pectinoidea;Pectinidae;Pecten;Pecten maximus
UdeM00000016680 6579 870.9 UdeM00000016680 XP_033763798.1 65.1 645 211 9 5 640 6 645 1.00E-249 870.9 6579 cellular organisms;Eukaryota;Opisthokonta;Metazoa;Eumetazoa;Bilateria;Protostomia;Spiralia;Lophotrochozoa;Mollusca;Bivalvia;Autobranchia;Pteriomorphia;Pectinida;Pectinoidea;Pectinidae;Pecten;Pecten maximus
UdeM00000016679 6579 869.4 UdeM00000016679 XP_033763798.1 65 645 212 9 5 640 6 645 2.90E-249 869.4 6579 cellular organisms;Eukaryota;Opisthokonta;Metazoa;Eumetazoa;Bilateria;Protostomia;Spiralia;Lophotrochozoa;Mollusca;Bivalvia;Autobranchia;Pteriomorphia;Pectinida;Pectinoidea;Pectinidae;Pecten;Pecten maximus
UdeM00000016681 29159 141.7 UdeM00000016681 XP_011436201.2 47.9 188 79 6 304 483 552 728 2.40E-30 141.7 29159 cellular organisms;Eukaryota;Opisthokonta;Metazoa;Eumetazoa;Bilateria;Protostomia;Spiralia;Lophotrochozoa;Mollusca;Bivalvia;Autobranchia;Pteriomorphia;Ostreida;Ostreoidea;Ostreidae;Crassostrea;Crassostrea gigas
UdeM00000016682 6579 409.8 UdeM00000016682 XP_033764060.1 57.4 319 136 0 5 323 5 323 3.20E-111 409.8 6579 cellular organisms;Eukaryota;Opisthokonta;Metazoa;Eumetazoa;Bilateria;Protostomia;Spiralia;Lophotrochozoa;Mollusca;Bivalvia;Autobranchia;Pteriomorphia;Pectinida;Pectinoidea;Pectinidae;Pecten;Pecten maximus
UdeM00000016683 6579 350.1 UdeM00000016683 XP_033764060.1 55.4 280 125 0 5 284 5 284 2.80E-93 350.1 6579 cellular organisms;Eukaryota;Opisthokonta;Metazoa;Eumetazoa;Bilateria;Protostomia;Spiralia;Lophotrochozoa;Mollusca;Bivalvia;Autobranchia;Pteriomorphia;Pectinida;Pectinoidea;Pectinidae;Pecten;Pecten maximus
UdeM00000016684 6579 350.5 UdeM00000016684 XP_033764060.1 55.4 280 125 0 5 284 5 284 2.10E-93 350.5 6579 cellular organisms;Eukaryota;Opisthokonta;Metazoa;Eumetazoa;Bilateria;Protostomia;Spiralia;Lophotrochozoa;Mollusca;Bivalvia;Autobranchia;Pteriomorphia;Pectinida;Pectinoidea;Pectinidae;Pecten;Pecten maximus
UdeM00000016685 6596 454.9 UdeM00000016685 XP_045163575.1 73.1 301 81 0 1 301 338 638 8.30E-125 454.9 6596 cellular organisms;Eukaryota;Opisthokonta;Metazoa;Eumetazoa;Bilateria;Protostomia;Spiralia;Lophotrochozoa;Mollusca;Bivalvia;Autobranchia;Heteroconchia;Euheterodonta;Imparidentia;Venerida;Veneroidea;Veneridae;Mercenaria;Mercenaria mercenaria
UdeM00000016687 6596 828.9 UdeM00000016687 XP_045161994.1 47 945 412 11 3 888 4 918 5.90E-237 828.9 6596 cellular organisms;Eukaryota;Opisthokonta;Metazoa;Eumetazoa;Bilateria;Protostomia;Spiralia;Lophotrochozoa;Mollusca;Bivalvia;Autobranchia;Heteroconchia;Euheterodonta;Imparidentia;Venerida;Veneroidea;Veneridae;Mercenaria;Mercenaria mercenaria
UdeM00000016688 29159 633.6 UdeM00000016688 XP_011424866.2 38.2 895 523 11 27 899 1 887 3.70E-178 633.6 29159 cellular organisms;Eukaryota;Opisthokonta;Metazoa;Eumetazoa;Bilateria;Protostomia;

Spiralia;Lophotrochozoa;Mollusca;Bivalvia;Autobranchia;Pteriomorphia;Ostreida;Ostreoidea;Ostreidae;Crassostrea;Crassostrea gigas
UdeM00000016689 29159 569.7 UdeM00000016689 XP_034335858.1 39.6 685 391 6 1 671 120 795 4.90E-159 569.7 29159 cellular organisms;Eukaryota;Opisthokonta;Metazoa;Eumetazoa;Bilateria;Protostomia;Spiralia;Lophotrochozoa;Mollusca;Bivalvia;Autobranchia;Pteriomorphia;Ostreida;Ostreoidea;Ostreidae;Crassostrea;Crassostrea gigas
UdeM00000016690 29159 574.3 UdeM00000016690 XP_034335858.1 39.6 684 391 5 1 670 120 795 2.00E-160 574.3 29159 cellular organisms;Eukaryota;Opisthokonta;Metazoa;Eumetazoa;Bilateria;Protostomia;Spiralia;Lophotrochozoa;Mollusca;Bivalvia;Autobranchia;Pteriomorphia;Ostreida;Ostreoidea;Ostreidae;Crassostrea;Crassostrea gigas
UdeM00000016691 6579 201.4 UdeM00000016691 XP_033763903.1 39.7 209 125 1 14 221 552 760 1.40E-48 201.4 6579 cellular organisms;Eukaryota;Opisthokonta;Metazoa;Eumetazoa;Bilateria;Protostomia;Spiralia;Lophotrochozoa;Mollusca;Bivalvia;Autobranchia;Pteriomorphia;Pectinida;Pectinoidea;Pectinidae;Pecten;Pecten maximus
UdeM00000016693 29159 348.2 UdeM00000016693 XP_034335858.1 44.7 342 184 2 1 337 197 538 1.20E-92 348.2 29159 cellular organisms;Eukaryota;Opisthokonta;Metazoa;Eumetazoa;Bilateria;Protostomia;Spiralia;Lophotrochozoa;Mollusca;Bivalvia;Autobranchia;Pteriomorphia;Ostreida;Ostreoidea;Ostreidae;Crassostrea;Crassostrea gigas
UdeM00000016692 6565 503.8 UdeM00000016692 XP_022336349.1 44.9 506 274 2 15 515 23 528 2.60E-139 503.8 6565 cellular organisms;Eukaryota;Opisthokonta;Metazoa;Eumetazoa;Bilateria;Protostomia;Spiralia;Lophotrochozoa;Mollusca;Bivalvia;Autobranchia;Pteriomorphia;Ostreida;Ostreoidea;Ostreidae;Crassostrea;Crassostrea virginica
UdeM00000016694 6565 166.4 UdeM00000016694 XP_022336349.1 47.5 158 83 0 15 172 23 180 3.20E-38 166.4 6565 cellular organisms;Eukaryota;Opisthokonta;Metazoa;Eumetazoa;Bilateria;Protostomia;Spiralia;Lophotrochozoa;Mollusca;Bivalvia;Autobranchia;Pteriomorphia;Ostreida;Ostreoidea;Ostreidae;Crassostrea;Crassostrea virginica
UdeM00000016695 6579 501.9 UdeM00000016695 XP_033763903.1 40.8 566 318 5 1 557 224 781 1.10E-138 501.9 6579 cellular organisms;Eukaryota;Opisthokonta;Metazoa;Eumetazoa;Bilateria;Protostomia;Spiralia;Lophotrochozoa;Mollusca;Bivalvia;Autobranchia;Pteriomorphia;Pectinida;Pectinoidea;Pectinidae;Pecten;Pecten maximus
UdeM00000016696 6565 145.2 UdeM00000016696 XP_022336349.1 41.8 158 92 0 241 398 23 180 1.80E-31 145.2 6565 cellular organisms;Eukaryota;Opisthokonta;Metazoa;Eumetazoa;Bilateria;Protostomia;Spiralia;Lophotrochozoa;Mollusca;Bivalvia;Autobranchia;Pteriomorphia;Ostreida;Ostreoidea;Ostreidae;Crassostrea;Crassostrea virginica
UdeM00000016698 6596 312 UdeM00000016698 XP_045179858.1 32.1 652 167 7 2 390 199 837 1.40E-81 312 6596 cellular organisms;Eukaryota;Opisthokonta;Metazoa;Eumetazoa;Bilateria;Protostomia;Spiralia;Lophotrochozoa;Mollusca;Bivalvia;Autobranchia;Heteroconchia;Euheterodonta;Imparidentia;Venerida;Veneroidea;Veneridae;Mercenaria;Mercenaria mercenaria
UdeM00000016699 6573 203.8 UdeM00000016699 XP_021366586.1 46 211 109 1 3 213 1 206 2.50E-49 203.8 6573 cellular organisms;Eukaryota;Opisthokonta;Metazoa;Eumetazoa;Bilateria;Protostomia;Spiralia;Lophotrochozoa;Mollusca;Bivalvia;Autobranchia;Pteriomorphia;Pectinida;Pectinoidea;Pectinidae;Mizuhopecten;Mizuhopecten yessoensis

UdeM00000016700 37653 299.7 UdeM00000016700 XP_014771786.1 36.4 626
317 12 78 658 48 637 9.40E-78 299.7 37653 cellular
organisms;Eukaryota;Opisthokonta;Metazoa;Eumetazoa;Bilateria;Protostomia;
Spiralia;Lophotrochozoa;Mollusca;Cephalopoda;Coleoidea;Octopodiformes;Oct
opoda;Incirrata;Octopodidae;Octopus;Octopus bimaculoides
UdeM00000016702 29159 372.1 UdeM00000016702 XP_011434453.2 27.4 1342
612 32 74 1355 33 1072 3.70E-99 372.1 29159 cellular
organisms;Eukaryota;Opisthokonta;Metazoa;Eumetazoa;Bilateria;Protostomia;
Spiralia;Lophotrochozoa;Mollusca;Bivalvia;Autobranchia;Pteriomorphia;Ostr
eida;Ostreoidea;Ostreidae;Crassostrea;Crassostrea gigas
UdeM00000016703 6596 621.7 UdeM00000016703 XP_045163414.1 69.8 411
124 0 1 411 1 411 6.70E-175 621.7 6596 cellular
organisms;Eukaryota;Opisthokonta;Metazoa;Eumetazoa;Bilateria;Protostomia;
Spiralia;Lophotrochozoa;Mollusca;Bivalvia;Autobranchia;Heteroconchia;Euhe
terodonta;Imparidentia;Venerida;Veneroidea;Veneridae;Mercenaria;Mercenari
a mercenaria
UdeM00000016704 6596 1565.1 UdeM00000016704 XP_045163414.1 70.7
1045 304 2 1 1043 1 1045 0.00E+00 1565.1 6596
cellular
organisms;Eukaryota;Opisthokonta;Metazoa;Eumetazoa;Bilateria;Protostomia;
Spiralia;Lophotrochozoa;Mollusca;Bivalvia;Autobranchia;Heteroconchia;Euhe
terodonta;Imparidentia;Venerida;Veneroidea;Veneridae;Mercenaria;Mercenari
a mercenaria
UdeM00000016705 6596 948.7 UdeM00000016705 XP_045163414.1 71.3 635
180 2 6 638 411 1045 3.70E-273 948.7 6596 cellular
organisms;Eukaryota;Opisthokonta;Metazoa;Eumetazoa;Bilateria;Protostomia;
Spiralia;Lophotrochozoa;Mollusca;Bivalvia;Autobranchia;Heteroconchia;Euhe
terodonta;Imparidentia;Venerida;Veneroidea;Veneridae;Mercenaria;Mercenari
a mercenaria
UdeM00000016706 409849 122.9 UdeM00000016706 XP_033839884.1 34
194 128 0 50 243 23 216 6.50E-25 122.9 409849
cellular
organisms;Eukaryota;Opisthokonta;Metazoa;Eumetazoa;Bilateria;Deuterostomi
a;Chordata;Craniata;Vertebrata;Gnathostomata;Teleostomi;Euteleostomi;Acti
nopterygii;Actinopteri;Neopterygii;Teleostei;Osteoglossocephalai;Clupeoce
phala;Euteleosteomorpha;Neoteleostei;Eurypterygia;Ctenosquamata;Acanthomo
rphata;Euacanthomorphacea;Percomorphaceae;Gobiaria;Gobiiformes;Gobioidei;
Gobiidae;Oxudercinae;Periophthalmus;Periophthalmus magnuspinnatus
UdeM00000016707 1735272 598.6 UdeM00000016707 XP_041377444.1 40.2
809 451 12 41 826 250 1048 1.20E-167 598.6 1735272
cellular
organisms;Eukaryota;Opisthokonta;Metazoa;Eumetazoa;Bilateria;Protostomia;
Spiralia;Lophotrochozoa;Mollusca;Gastropoda;Neomphalina;Neomphaloidea;Pel
tospiridae;Gigantopelta;Gigantopelta aegis
UdeM00000016708 210632 651.4 UdeM00000016708 XP_028283034.1 32.3
1195 629 14 1 1169 26 1066 2.20E-183 651.4 210632
cellular
organisms;Eukaryota;Opisthokonta;Metazoa;Eumetazoa;Bilateria;Deuterostomi
a;Chordata;Craniata;Vertebrata;Gnathostomata;Teleostomi;Euteleostomi;Acti
nopterygii;Actinopteri;Neopterygii;Teleostei;Osteoglossocephalai;Clupeoce
phala;Euteleosteomorpha;Neoteleostei;Eurypterygia;Ctenosquamata;Acanthomo
rphata;Euacanthomorphacea;Percomorphaceae;Ovalentaria;Ovalentaria
incertae sedis;Ambassidae;Parambassis;Parambassis ranga

UdeM00000016709 210632 651.4 UdeM00000016709 XP_028283034.1 32.8
1201 614 17 1 1168 26 1066 2.20E-183 651.4 210632
cellular
organisms;Eukaryota;Opisthokonta;Metazoa;Eumetazoa;Bilateria;Deuterostomia;Chordata;Craniata;Vertebrata;Gnathostomata;Teleostomi;Euteleostomi;Actinopterygii;Actinopteri;Neopterygii;Teleostei;Osteoglossocephalai;Clupeocephala;Euteleosteomorpha;Neoteleostei;Eurypterygia;Ctenosquamata;Acanthomorphata;Euacanthomorphacea;Percomorphaceae;Ovalentaria;Ovalentaria incertae sedis;Ambassidae;Parambassis;Parambassis ranga
UdeM00000016710 6573 554.7 UdeM00000016710 XP_021340202.1 58.2 452
188 1 35 486 62 512 1.20E-154 554.7 6573 cellular
organisms;Eukaryota;Opisthokonta;Metazoa;Eumetazoa;Bilateria;Protostomia;Spiralia;Lophotrochozoa;Mollusca;Bivalvia;Autobranchia;Pteriomorphia;Pectinida;Pectinoidea;Pectinidae;Mizuhopecten;Mizuhopecten yessoensis
UdeM00000042474 29159 67.4 UdeM00000042474 XP_034319609.1 55.1 49
22 0 35 83 906 954 1.00E-08 67.4 29159 cellular
organisms;Eukaryota;Opisthokonta;Metazoa;Eumetazoa;Bilateria;Protostomia;Spiralia;Lophotrochozoa;Mollusca;Bivalvia;Autobranchia;Pteriomorphia;Ostreida;Ostreoidea;Ostreidae;Crassostrea;Crassostrea gigas
UdeM00000042477 6565 214.5 UdeM00000042477 XP_022306954.1 64.6 144
51 0 1 144 367 510 9.10E-53 214.5 6565 cellular
organisms;Eukaryota;Opisthokonta;Metazoa;Eumetazoa;Bilateria;Protostomia;Spiralia;Lophotrochozoa;Mollusca;Bivalvia;Autobranchia;Pteriomorphia;Ostreida;Ostreoidea;Ostreidae;Crassostrea;Crassostrea virginica
UdeM00000042478 29159 93.2 UdeM00000042478 XP_034319609.1 39.7 116
46 1 1 92 839 954 1.90E-16 93.2 29159 cellular
organisms;Eukaryota;Opisthokonta;Metazoa;Eumetazoa;Bilateria;Protostomia;Spiralia;Lophotrochozoa;Mollusca;Bivalvia;Autobranchia;Pteriomorphia;Ostreida;Ostreoidea;Ostreidae;Crassostrea;Crassostrea gigas
UdeM00000042479 6596 164.1 UdeM00000042479 XP_045177053.1 34.7 291
168 11 4 281 150 431 2.60E-37 164.1 6596 cellular
organisms;Eukaryota;Opisthokonta;Metazoa;Eumetazoa;Bilateria;Protostomia;Spiralia;Lophotrochozoa;Mollusca;Bivalvia;Autobranchia;Heteroconchia;Euheterodonta;Imparidentia;Venerida;Veneroidea;Veneridae;Mercenaria;Mercenaria mercenaria
UdeM00000042482 29159 73.2 UdeM00000042482 XP_034321668.1 73.9 46
12 0 1 46 483 528 2.10E-10 73.2 29159 cellular
organisms;Eukaryota;Opisthokonta;Metazoa;Eumetazoa;Bilateria;Protostomia;Spiralia;Lophotrochozoa;Mollusca;Bivalvia;Autobranchia;Pteriomorphia;Ostreida;Ostreoidea;Ostreidae;Crassostrea;Crassostrea gigas
UdeM00000042484 6565 108.2 UdeM00000042484 XP_022307853.1 34 212
125 8 18 225 376 576 1.50E-20 108.2 6565 cellular
organisms;Eukaryota;Opisthokonta;Metazoa;Eumetazoa;Bilateria;Protostomia;Spiralia;Lophotrochozoa;Mollusca;Bivalvia;Autobranchia;Pteriomorphia;Ostreida;Ostreoidea;Ostreidae;Crassostrea;Crassostrea virginica
UdeM00000042485 6596 153.3 UdeM00000042485 XP_045177053.1 34.2 257
156 7 3 250 124 376 4.20E-34 153.3 6596 cellular
organisms;Eukaryota;Opisthokonta;Metazoa;Eumetazoa;Bilateria;Protostomia;Spiralia;Lophotrochozoa;Mollusca;Bivalvia;Autobranchia;Heteroconchia;Euheterodonta;Imparidentia;Venerida;Veneroidea;Veneridae;Mercenaria;Mercenaria mercenaria
UdeM00000042488 7741 60.1 UdeM00000042488 XP_019620205.1 29.7 138
85 5 89 223 748 876 4.30E-06 60.1 7741 cellular
organisms;Eukaryota;Opisthokonta;Metazoa;Eumetazoa;Bilateria;Deuterostomi

a;Chordata;Cephalochordata;Leptocardii;Amphioxiformes;Branchiostomidae;Branchiostoma;Branchiostoma belcheri
UdeM00000042489 7741 60.1 UdeM00000042489 XP_019620205.1 29.7 138 85 5 13 147 748 876 2.90E-06 60.1 7741 cellular organisms;Eukaryota;Opisthokonta;Metazoa;Eumetazoa;Bilateria;Deuterostomia;Chordata;Cephalochordata;Leptocardii;Amphioxiformes;Branchiostomidae;Branchiostoma;Branchiostoma belcheri
UdeM00000042490 45351 88.2 UdeM00000042490 XP_032239173.1 28.1 256 159 6 79 324 100 340 3.50E-14 88.2 45351 cellular organisms;Eukaryota;Opisthokonta;Metazoa;Eumetazoa;Cnidaria;Anthozoa;Hexacorallia;Actiniaria;Edwardsiidae;Nematostella;Nematostella vectensis
UdeM00000042492 29159 246.1 UdeM00000042492 XP_011424053.2 77.6 147 33 0 1 147 1 147 2.70E-62 246.1 29159 cellular organisms;Eukaryota;Opisthokonta;Metazoa;Eumetazoa;Bilateria;Protostomia;Spiralia;Lophotrochozoa;Mollusca;Bivalvia;Autobranchia;Pteriomorphia;Ostreida;Ostreoidea;Ostreidae;Crassostrea;Crassostrea gigas
UdeM00000042493 6579 220.3 UdeM00000042493 XP_033758968.1 26.2 679 450 17 33 684 15 669 7.60E-54 220.3 6579 cellular organisms;Eukaryota;Opisthokonta;Metazoa;Eumetazoa;Bilateria;Protostomia;Spiralia;Lophotrochozoa;Mollusca;Bivalvia;Autobranchia;Pteriomorphia;Pectinida;Pectinoidea;Pectinidae;Pecten;Pecten maximus
UdeM00000042494 6579 218.4 UdeM00000042494 XP_033758968.1 26.3 681 452 17 23 675 11 669 2.90E-53 218.4 6579 cellular organisms;Eukaryota;Opisthokonta;Metazoa;Eumetazoa;Bilateria;Protostomia;Spiralia;Lophotrochozoa;Mollusca;Bivalvia;Autobranchia;Pteriomorphia;Pectinida;Pectinoidea;Pectinidae;Pecten;Pecten maximus
UdeM00000042495 6573 211.5 UdeM00000042495 XP_021341731.1 24.7 675 474 13 27 681 15 675 3.50E-51 211.5 6573 cellular organisms;Eukaryota;Opisthokonta;Metazoa;Eumetazoa;Bilateria;Protostomia;Spiralia;Lophotrochozoa;Mollusca;Bivalvia;Autobranchia;Pteriomorphia;Pectinida;Pectinoidea;Pectinidae;Mizuhopecten;Mizuhopecten yessoensis
UdeM00000042497 6596 64.3 UdeM00000042497 XP_045177458.1 30.8 107 73 1 366 471 77 183 6.70E-07 64.3 6596 cellular organisms;Eukaryota;Opisthokonta;Metazoa;Eumetazoa;Bilateria;Protostomia;Spiralia;Lophotrochozoa;Mollusca;Bivalvia;Autobranchia;Heteroconchia;Euheterodonta;Imparidentia;Venerida;Veneroidea;Veneridae;Mercenaria;Mercenaria mercenaria
UdeM00000042498 6565 84.7 UdeM00000042498 XP_022330482.1 26.1 234 156 7 686 916 406 625 6.80E-13 84.7 6565 cellular organisms;Eukaryota;Opisthokonta;Metazoa;Eumetazoa;Bilateria;Protostomia;Spiralia;Lophotrochozoa;Mollusca;Bivalvia;Autobranchia;Pteriomorphia;Ostreida;Ostreoidea;Ostreidae;Crassostrea;Crassostrea virginica
UdeM00000042499 7741 201.4 UdeM00000042499 XP_019646171.1 45.7 208 109 2 31 238 255 458 1.30E-48 201.4 7741 cellular organisms;Eukaryota;Opisthokonta;Metazoa;Eumetazoa;Bilateria;Deuterostomia;Chordata;Cephalochordata;Leptocardii;Amphioxiformes;Branchiostomidae;Branchiostoma;Branchiostoma belcheri
UdeM00000042500 7574 141 UdeM00000042500 XP_013397768.1 27.6 424 243 14 1 364 103 522 3.20E-30 141 7574 cellular organisms;Eukaryota;Opisthokonta;Metazoa;Eumetazoa;Bilateria;Protostomia;Spiralia;Lophotrochozoa;Brachiopoda;Linguliformea;Lingulata;Lingulida;Linguloidea;Lingulidae;Lingula;Lingula anatina
UdeM00000042501 29159 98.2 UdeM00000042501 XP_034310917.1 34.1 167 100 5 2 160 572 736 1.00E-17 98.2 29159 cellular

organisms;Eukaryota;Opisthokonta;Metazoa;Eumetazoa;Bilateria;Protostomia;Spiralia;Lophotrochozoa;Mollusca;Bivalvia;Autobranchia;Pteriomorphia;Ostreida;Ostreoidea;Ostreidae;Crassostrea;Crassostrea gigas
UdeM00000042502 29159 130.6 UdeM00000042502 XP_034333290.1 35.6 239 139 8 1 228 1910 2144 5.50E-27 130.6 29159 cellular organisms;Eukaryota;Opisthokonta;Metazoa;Eumetazoa;Bilateria;Protostomia;Spiralia;Lophotrochozoa;Mollusca;Bivalvia;Autobranchia;Pteriomorphia;Ostreida;Ostreoidea;Ostreidae;Crassostrea;Crassostrea gigas
UdeM00000042504 6579 151 UdeM00000042504 XP_033738785.1 45.5 189 98 4 64 248 252 439 3.00E-33 151 6579 cellular organisms;Eukaryota;Opisthokonta;Metazoa;Eumetazoa;Bilateria;Protostomia;Spiralia;Lophotrochozoa;Mollusca;Bivalvia;Autobranchia;Pteriomorphia;Pectinida;Pectinoidea;Pectinidae;Pecten;Pecten maximus
UdeM00000042505 6579 151 UdeM00000042505 XP_033738785.1 45.5 189 98 4 64 248 252 439 3.00E-33 151 6579 cellular organisms;Eukaryota;Opisthokonta;Metazoa;Eumetazoa;Bilateria;Protostomia;Spiralia;Lophotrochozoa;Mollusca;Bivalvia;Autobranchia;Pteriomorphia;Pectinida;Pectinoidea;Pectinidae;Pecten;Pecten maximus
UdeM00000042506 70779 70.1 UdeM00000042506 XP_015777172.1 38.8 80 49 0 7 86 58 137 1.80E-09 70.1 70779 cellular organisms;Eukaryota;Opisthokonta;Metazoa;Eumetazoa;Cnidaria;Anthozoa;Hexacorallia;Scleractinia;Astrocoeniina;Acroporidae;Acropora;Acropora digitifera
UdeM00000042507 6596 251.5 UdeM00000042507 XP_045180379.1 69.1 175 52 1 1 173 1 175 7.70E-64 251.5 6596 cellular organisms;Eukaryota;Opisthokonta;Metazoa;Eumetazoa;Bilateria;Protostomia;Spiralia;Lophotrochozoa;Mollusca;Bivalvia;Autobranchia;Heteroconchia;Euheterodonta;Imparidentia;Venerida;Veneroidea;Veneridae;Mercenaria;Mercenaria mercenaria
UdeM00000042509 37621 154.1 UdeM00000042509 XP_014673981.1 43.2 206 77 5 6 174 16 218 1.80E-34 154.1 37621 cellular organisms;Eukaryota;Opisthokonta;Metazoa;Eumetazoa;Bilateria;Protostomia;Ecdysozoa;Scalidophora;Priapulida;Priapulimorpha;Priapulimorphida;Priapulidae;Priapulus;Priapulus caudatus
UdeM00000042510 37621 213.4 UdeM00000042510 XP_014672567.1 52.3 174 83 0 10 183 345 518 2.90E-52 213.4 37621 cellular organisms;Eukaryota;Opisthokonta;Metazoa;Eumetazoa;Bilateria;Protostomia;Ecdysozoa;Scalidophora;Priapulida;Priapulimorpha;Priapulimorphida;Priapulidae;Priapulus;Priapulus caudatus
UdeM00000042511 7668 119.8 UdeM00000042511 XP_011671223.1 47.6 124 63 2 8 131 10 131 3.40E-24 119.8 7668 cellular organisms;Eukaryota;Opisthokonta;Metazoa;Eumetazoa;Bilateria;Deuterostomia;Echinodermata;Eleutherozoa;Echinozoa;Echinoidea;Euechinoidea;Echinacea;Camarodonta;Echinidea;Strongylocentrotidae;Strongylocentrotus;Strongylocentrotus purpuratus
UdeM00000042512 6596 85.9 UdeM00000042512 XP_045209828.1 68.8 64 19 1 107 169 99 162 5.50E-14 85.9 6596 cellular organisms;Eukaryota;Opisthokonta;Metazoa;Eumetazoa;Bilateria;Protostomia;Spiralia;Lophotrochozoa;Mollusca;Bivalvia;Autobranchia;Heteroconchia;Euheterodonta;Imparidentia;Venerida;Veneroidea;Veneridae;Mercenaria;Mercenaria mercenaria
UdeM00000042513 6596 1330.1 UdeM00000042513 XP_045209751.1 86.8 775 100 2 1 774 1 774 0.00E+00 1330.1 6596 cellular

organisms;Eukaryota;Opisthokonta;Metazoa;Eumetazoa;Bilateria;Protostomia;Spiralia;Lophotrochozoa;Mollusca;Bivalvia;Autobranchia;Heteroconchia;Euheterodonta;Imparidentia;Venerida;Veneroidea;Veneridae;Mercenaria;Mercenaria mercenaria
UdeM00000042514 6596 239.6 UdeM00000042514 XP_045201323.1 44.3 325 171 5 50 371 106 423 7.30E-60 239.6 6596 cellular organisms;Eukaryota;Opisthokonta;Metazoa;Eumetazoa;Bilateria;Protostomia;Spiralia;Lophotrochozoa;Mollusca;Bivalvia;Autobranchia;Heteroconchia;Euheterodonta;Imparidentia;Venerida;Veneroidea;Veneridae;Mercenaria;Mercenaria mercenaria
UdeM00000042515 6596 222.6 UdeM00000042515 XP_045201320.1 41 315 178 4 59 371 119 427 9.60E-55 222.6 6596 cellular organisms;Eukaryota;Opisthokonta;Metazoa;Eumetazoa;Bilateria;Protostomia;Spiralia;Lophotrochozoa;Mollusca;Bivalvia;Autobranchia;Heteroconchia;Euheterodonta;Imparidentia;Venerida;Veneroidea;Veneridae;Mercenaria;Mercenaria mercenaria
UdeM00000042516 400727 66.2 UdeM00000042516 XP_025110088.1 36.1 83 53 0 15 97 89 171 4.00E-08 66.2 400727 cellular organisms;Eukaryota;Opisthokonta;Metazoa;Eumetazoa;Bilateria;Protostomia;Spiralia;Lophotrochozoa;Mollusca;Gastropoda;Caenogastropoda;Architaenioglossa;Ampullarioidea;Ampullariidae;Pomacea;Pomacea canaliculata
UdeM00000042517 400727 66.2 UdeM00000042517 XP_025110088.1 36.1 83 53 0 15 97 89 171 4.00E-08 66.2 400727 cellular organisms;Eukaryota;Opisthokonta;Metazoa;Eumetazoa;Bilateria;Protostomia;Spiralia;Lophotrochozoa;Mollusca;Gastropoda;Caenogastropoda;Architaenioglossa;Ampullarioidea;Ampullariidae;Pomacea;Pomacea canaliculata
UdeM00000042518 6596 222.6 UdeM00000042518 XP_045201320.1 41 315 178 4 59 371 119 427 9.60E-55 222.6 6596 cellular organisms;Eukaryota;Opisthokonta;Metazoa;Eumetazoa;Bilateria;Protostomia;Spiralia;Lophotrochozoa;Mollusca;Bivalvia;Autobranchia;Heteroconchia;Euheterodonta;Imparidentia;Venerida;Veneroidea;Veneridae;Mercenaria;Mercenaria mercenaria
UdeM00000042520 6596 94.4 UdeM00000042520 XP_045201323.1 39.5 177 87 2 3 159 40 216 1.40E-16 94.4 6596 cellular organisms;Eukaryota;Opisthokonta;Metazoa;Eumetazoa;Bilateria;Protostomia;Spiralia;Lophotrochozoa;Mollusca;Bivalvia;Autobranchia;Heteroconchia;Euheterodonta;Imparidentia;Venerida;Veneroidea;Veneridae;Mercenaria;Mercenaria mercenaria
UdeM00000042521 7719 101.3 UdeM00000042521 NP_001027676.1 86.2 58 8 0 80 137 11 68 1.00E-18 101.3 7719 cellular organisms;Eukaryota;Opisthokonta;Metazoa;Eumetazoa;Bilateria;Deuterostomia;Chordata;Tunicata;Ascidiacea;Phlebobranchia;Cionidae;Ciona;Ciona intestinalis
UdeM00000042522 6579 129.4 UdeM00000042522 XP_033733095.1 50 126 56 3 8 127 9 133 3.80E-27 129.4 6579 cellular organisms;Eukaryota;Opisthokonta;Metazoa;Eumetazoa;Bilateria;Protostomia;Spiralia;Lophotrochozoa;Mollusca;Bivalvia;Autobranchia;Pteriomorphia;Pectinida;Pectinoidea;Pectinidae;Pecten;Pecten maximus
UdeM00000044290 6565 866.3 UdeM00000044290 XP_022308715.1 77.3 516 116 1 8 523 6 520 2.00E-248 866.3 6565 cellular organisms;Eukaryota;Opisthokonta;Metazoa;Eumetazoa;Bilateria;Protostomia;

Spiralia;Lophotrochozoa;Mollusca;Bivalvia;Autobranchia;Pteriomorphia;Ostreida;Ostreoidea;Ostreidae;Crassostrea;Crassostrea virginica
UdeM00000019257 6500 108.2 UdeM00000019257 XP_035824657.1 61.4 88 34 0 31 118 22 109 2.00E-20 108.2 6500 cellular organisms;Eukaryota;Opisthokonta;Metazoa;Eumetazoa;Bilateria;Protostomia;Spiralia;Lophotrochozoa;Mollusca;Gastropoda;Heterobranchia;Euthyneura;Tectipleura;Aplysiida;Aplysioidea;Aplysiidae;Aplysia;Aplysia californica
UdeM00000019258 6596 112.8 UdeM00000019258 XP_045165187.1 33.6 295 171 6 47 328 14 296 8.20E-22 112.8 6596 cellular organisms;Eukaryota;Opisthokonta;Metazoa;Eumetazoa;Bilateria;Protostomia;Spiralia;Lophotrochozoa;Mollusca;Bivalvia;Autobranchia;Heteroconchia;Euheterodonta;Imparidentia;Venerida;Veneroidea;Veneridae;Mercenaria;Mercenaria mercenaria
UdeM00000019259 6596 162.5 UdeM00000019259 XP_045165957.1 42.8 187 100 4 11 193 8 191 5.70E-37 162.5 6596 cellular organisms;Eukaryota;Opisthokonta;Metazoa;Eumetazoa;Bilateria;Protostomia;Spiralia;Lophotrochozoa;Mollusca;Bivalvia;Autobranchia;Heteroconchia;Euheterodonta;Imparidentia;Venerida;Veneroidea;Veneridae;Mercenaria;Mercenaria mercenaria
UdeM00000019260 6596 772.7 UdeM00000019260 XP_045166343.1 65.2 595 186 7 1 591 18 595 3.50E-220 772.7 6596 cellular organisms;Eukaryota;Opisthokonta;Metazoa;Eumetazoa;Bilateria;Protostomia;Spiralia;Lophotrochozoa;Mollusca;Bivalvia;Autobranchia;Heteroconchia;Euheterodonta;Imparidentia;Venerida;Veneroidea;Veneridae;Mercenaria;Mercenaria mercenaria
UdeM00000019261 6596 706.8 UdeM00000019261 XP_045164785.1 61.7 595 205 5 2 580 6 593 2.40E-200 706.8 6596 cellular organisms;Eukaryota;Opisthokonta;Metazoa;Eumetazoa;Bilateria;Protostomia;Spiralia;Lophotrochozoa;Mollusca;Bivalvia;Autobranchia;Heteroconchia;Euheterodonta;Imparidentia;Venerida;Veneroidea;Veneridae;Mercenaria;Mercenaria mercenaria
UdeM00000019263 6596 721.1 UdeM00000019263 XP_045164785.1 64.1 579 183 4 19 584 17 583 1.30E-204 721.1 6596 cellular organisms;Eukaryota;Opisthokonta;Metazoa;Eumetazoa;Bilateria;Protostomia;Spiralia;Lophotrochozoa;Mollusca;Bivalvia;Autobranchia;Heteroconchia;Euheterodonta;Imparidentia;Venerida;Veneroidea;Veneridae;Mercenaria;Mercenaria mercenaria
UdeM00000019262 6596 721.1 UdeM00000019262 XP_045164785.1 64.1 579 183 4 19 584 17 583 1.30E-204 721.1 6596 cellular organisms;Eukaryota;Opisthokonta;Metazoa;Eumetazoa;Bilateria;Protostomia;Spiralia;Lophotrochozoa;Mollusca;Bivalvia;Autobranchia;Heteroconchia;Euheterodonta;Imparidentia;Venerida;Veneroidea;Veneridae;Mercenaria;Mercenaria mercenaria
UdeM00000019264 6596 354.8 UdeM00000019264 XP_045207182.1 57.8 296 113 3 61 352 5 292 1.70E-94 354.8 6596 cellular organisms;Eukaryota;Opisthokonta;Metazoa;Eumetazoa;Bilateria;Protostomia;Spiralia;Lophotrochozoa;Mollusca;Bivalvia;Autobranchia;Heteroconchia;Euheterodonta;Imparidentia;Venerida;Veneroidea;Veneridae;Mercenaria;Mercenaria mercenaria
UdeM00000019265 6596 320.1 UdeM00000019265 XP_045165300.1 52.2 335 153 2 24 354 23 354 3.60E-84 320.1 6596 cellular organisms;Eukaryota;Opisthokonta;Metazoa;Eumetazoa;Bilateria;Protostomia;Spiralia;Lophotrochozoa;Mollusca;Bivalvia;Autobranchia;Heteroconchia;Euhe

terodonta;Imparidentia;Venerida;Veneroidea;Veneridae;Mercenaria;Mercenaria mercenaria
UdeM00000019266 6596 316.6 UdeM00000019266 XP_045165300.1 52.3 331 151 2 2 328 27 354 3.70E-83 316.6 6596 cellular organisms;Eukaryota;Opisthokonta;Metazoa;Eumetazoa;Bilateria;Protostomia;Spiralia;Lophotrochozoa;Mollusca;Bivalvia;Autobranchia;Heteroconchia;Euheterodonta;Imparidentia;Venerida;Veneroidea;Veneridae;Mercenaria;Mercenaria mercenaria
UdeM00000019267 1735272 284.3 UdeM00000019267 XP_041349062.1 62.9 229 83 1 27 255 37 263 1.60E-73 284.3 1735272 cellular organisms;Eukaryota;Opisthokonta;Metazoa;Eumetazoa;Bilateria;Protostomia;Spiralia;Lophotrochozoa;Mollusca;Gastropoda;Neomphalina;Neomphaloidea;Peltospiridae;Gigantopelta;Gigantopelta aegis
UdeM00000019268 1735272 284.3 UdeM00000019268 XP_041349062.1 62.9 229 83 1 25 253 37 263 1.60E-73 284.3 1735272 cellular organisms;Eukaryota;Opisthokonta;Metazoa;Eumetazoa;Bilateria;Protostomia;Spiralia;Lophotrochozoa;Mollusca;Gastropoda;Neomphalina;Neomphaloidea;Peltospiridae;Gigantopelta;Gigantopelta aegis
UdeM00000019269 2607531 203.8 UdeM00000019269 XP_036362952.1 36.6 309 188 4 51 352 80 387 3.70E-49 203.8 2607531 cellular organisms;Eukaryota;Opisthokonta;Metazoa;Eumetazoa;Bilateria;Protostomia;Spiralia;Lophotrochozoa;Mollusca;Cephalopoda;Coleoidea;Octopodiformes;Octopoda;Incirrata;Octopodidae;Octopus;Octopus sinensis
UdeM00000019270 6565 903.3 UdeM00000019270 XP_022300211.1 42.6 1137 609 21 12 1135 9 1114 3.30E-259 903.3 6565 cellular organisms;Eukaryota;Opisthokonta;Metazoa;Eumetazoa;Bilateria;Protostomia;Spiralia;Lophotrochozoa;Mollusca;Bivalvia;Autobranchia;Pteriomorphia;Ostreida;Ostreoidea;Ostreidae;Crassostrea;Crassostrea virginica
UdeM00000019271 6596 75.9 UdeM00000019271 XP_045197826.1 33.5 173 89 3 28 199 46 193 6.80E-11 75.9 6596 cellular organisms;Eukaryota;Opisthokonta;Metazoa;Eumetazoa;Bilateria;Protostomia;Spiralia;Lophotrochozoa;Mollusca;Bivalvia;Autobranchia;Heteroconchia;Euheterodonta;Imparidentia;Venerida;Veneroidea;Veneridae;Mercenaria;Mercenaria mercenaria
UdeM00000019272 6596 451.4 UdeM00000019272 XP_045166715.1 67.7 356 105 5 1 354 1 348 1.00E-123 451.4 6596 cellular organisms;Eukaryota;Opisthokonta;Metazoa;Eumetazoa;Bilateria;Protostomia;Spiralia;Lophotrochozoa;Mollusca;Bivalvia;Autobranchia;Heteroconchia;Euheterodonta;Imparidentia;Venerida;Veneroidea;Veneridae;Mercenaria;Mercenaria mercenaria
UdeM00000019273 6596 407.1 UdeM00000019273 XP_045166716.1 44.4 453 243 6 1 451 34 479 2.80E-110 407.1 6596 cellular organisms;Eukaryota;Opisthokonta;Metazoa;Eumetazoa;Bilateria;Protostomia;Spiralia;Lophotrochozoa;Mollusca;Bivalvia;Autobranchia;Heteroconchia;Euheterodonta;Imparidentia;Venerida;Veneroidea;Veneridae;Mercenaria;Mercenaria mercenaria
UdeM00000019274 6596 576.6 UdeM00000019274 XP_045174038.1 54.7 530 238 2 5 532 46 575 3.20E-161 576.6 6596 cellular organisms;Eukaryota;Opisthokonta;Metazoa;Eumetazoa;Bilateria;Protostomia;Spiralia;Lophotrochozoa;Mollusca;Bivalvia;Autobranchia;Heteroconchia;Euhe

terodonta;Imparidentia;Venerida;Veneroidea;Veneridae;Mercenaria;Mercenaria mercenaria
UdeM00000019275 29159 92.4 UdeM00000019275 XP_011427550.2 84.6 52 8 0 1 52 391 442 1.80E-16 92.4 29159 cellular organisms;Eukaryota;Opisthokonta;Metazoa;Eumetazoa;Bilateria;Protostomia;Spiralia;Lophotrochozoa;Mollusca;Bivalvia;Autobranchia;Pteriomorphia;Ostreida;Ostreoidea;Ostreidae;Crassostrea;Crassostrea gigas
UdeM00000019276 6565 582.4 UdeM00000019276 XP_022296668.1 83 323 55 0 64 386 113 435 4.20E-163 582.4 6565 cellular organisms;Eukaryota;Opisthokonta;Metazoa;Eumetazoa;Bilateria;Protostomia;Spiralia;Lophotrochozoa;Mollusca;Bivalvia;Autobranchia;Pteriomorphia;Ostreida;Ostreoidea;Ostreidae;Crassostrea;Crassostrea virginica
UdeM00000019277 6579 211.1 UdeM00000019277 XP_033745913.1 78.2 119 26 0 1 119 1 119 8.50E-52 211.1 6579 cellular organisms;Eukaryota;Opisthokonta;Metazoa;Eumetazoa;Bilateria;Protostomia;Spiralia;Lophotrochozoa;Mollusca;Bivalvia;Autobranchia;Pteriomorphia;Pectinida;Pectinoidea;Pectinidae;Pecten;Pecten maximus
UdeM00000019278 6596 275.8 UdeM00000019278 XP_045166365.1 75.3 178 44 0 1 178 1 178 3.90E-71 275.8 6596 cellular organisms;Eukaryota;Opisthokonta;Metazoa;Eumetazoa;Bilateria;Protostomia;Spiralia;Lophotrochozoa;Mollusca;Bivalvia;Autobranchia;Heteroconchia;Euheterodonta;Imparidentia;Venerida;Veneroidea;Veneridae;Mercenaria;Mercenaria mercenaria
UdeM00000019279 6596 158.3 UdeM00000019279 XP_045166365.1 67.9 112 36 0 1 112 67 178 5.70E-36 158.3 6596 cellular organisms;Eukaryota;Opisthokonta;Metazoa;Eumetazoa;Bilateria;Protostomia;Spiralia;Lophotrochozoa;Mollusca;Bivalvia;Autobranchia;Heteroconchia;Euheterodonta;Imparidentia;Venerida;Veneroidea;Veneridae;Mercenaria;Mercenaria mercenaria
UdeM00000019280 6596 809.7 UdeM00000019280 XP_045166400.1 45.4 923 456 14 115 1013 815 1713 4.40E-231 809.7 6596 cellular organisms;Eukaryota;Opisthokonta;Metazoa;Eumetazoa;Bilateria;Protostomia;Spiralia;Lophotrochozoa;Mollusca;Bivalvia;Autobranchia;Heteroconchia;Euheterodonta;Imparidentia;Venerida;Veneroidea;Veneridae;Mercenaria;Mercenaria mercenaria
UdeM00000019281 6596 357.5 UdeM00000019281 XP_045166400.1 56 573 195 9 1 568 30 550 3.30E-95 357.5 6596 cellular organisms;Eukaryota;Opisthokonta;Metazoa;Eumetazoa;Bilateria;Protostomia;Spiralia;Lophotrochozoa;Mollusca;Bivalvia;Autobranchia;Heteroconchia;Euheterodonta;Imparidentia;Venerida;Veneroidea;Veneridae;Mercenaria;Mercenaria mercenaria
UdeM00000019282 6596 614.4 UdeM00000019282 XP_045164743.1 62.1 459 173 1 1 459 56 513 1.20E-172 614.4 6596 cellular organisms;Eukaryota;Opisthokonta;Metazoa;Eumetazoa;Bilateria;Protostomia;Spiralia;Lophotrochozoa;Mollusca;Bivalvia;Autobranchia;Heteroconchia;Euheterodonta;Imparidentia;Venerida;Veneroidea;Veneridae;Mercenaria;Mercenaria mercenaria
UdeM00000019283 1735272 126.3 UdeM00000019283 XP_041348272.1 66.7 96 29 2 1 93 1 96 5.60E-26 126.3 1735272 cellular organisms;Eukaryota;Opisthokonta;Metazoa;Eumetazoa;Bilateria;Protostomia;Spiralia;Lophotrochozoa;Mollusca;Gastropoda;Neomphalina;Neomphaloidea;Peltospiridae;Gigantopelta;Gigantopelta aegis

UdeM00000019284 6573 572 UdeM00000019284 XP_021357770.1 60 477
187 2 1 473 1 477 7.00E-160 572 6573 cellular
organisms;Eukaryota;Opisthokonta;Metazoa;Eumetazoa;Bilateria;Protostomia;
Spiralia;Lophotrochozoa;Mollusca;Bivalvia;Autobranchia;Pteriomorphia;Pect
inida;Pectinoidea;Pectinidae;Mizuhopecten;Mizuhopecten yessoensis
UdeM00000019285 400727 288.9 UdeM00000019285 XP_025107315.1 56.7
254 103 2 4 251 3 255 6.30E-75 288.9 400727
cellular
organisms;Eukaryota;Opisthokonta;Metazoa;Eumetazoa;Bilateria;Protostomia;
Spiralia;Lophotrochozoa;Mollusca;Gastropoda;Caenogastropoda;Architaeniogl
ossa;Ampullarioidea;Ampullariidae;Pomacea;Pomacea canaliculata
UdeM00000019286 6579 176.8 UdeM00000019286 XP_033744861.1 69 113
35 0 4 116 2 114 1.60E-41 176.8 6579 cellular
organisms;Eukaryota;Opisthokonta;Metazoa;Eumetazoa;Bilateria;Protostomia;
Spiralia;Lophotrochozoa;Mollusca;Bivalvia;Autobranchia;Pteriomorphia;Pect
inida;Pectinoidea;Pectinidae;Pecten;Pecten maximus
UdeM00000019287 6573 558.9 UdeM00000019287 XP_021370987.1 47.6 694
208 14 203 751 88 770 9.80E-156 558.9 6573 cellular
organisms;Eukaryota;Opisthokonta;Metazoa;Eumetazoa;Bilateria;Protostomia;
Spiralia;Lophotrochozoa;Mollusca;Bivalvia;Autobranchia;Pteriomorphia;Pect
inida;Pectinoidea;Pectinidae;Mizuhopecten;Mizuhopecten yessoensis
UdeM00000019288 1735272 424.1 UdeM00000019288 XP_041356266.1 51.1
407 189 4 51 454 209 608 2.30E-115 424.1 1735272
cellular
organisms;Eukaryota;Opisthokonta;Metazoa;Eumetazoa;Bilateria;Protostomia;
Spiralia;Lophotrochozoa;Mollusca;Gastropoda;Neomphalina;Neomphaloidea;Pel
tospiridae;Gigantopelta;Gigantopelta aegis
UdeM00000019289 6596 154.5 UdeM00000019289 XP_045185048.1 35 260
129 5 7 250 8 243 2.10E-34 154.5 6596 cellular
organisms;Eukaryota;Opisthokonta;Metazoa;Eumetazoa;Bilateria;Protostomia;
Spiralia;Lophotrochozoa;Mollusca;Bivalvia;Autobranchia;Heteroconchia;Euhe
terodonta;Imparidentia;Venerida;Veneroidea;Veneridae;Mercenaria;Mercenari
a mercenaria
UdeM00000019290 6596 573.5 UdeM00000019290 XP_045164236.1 72.7 396
108 0 50 445 33 428 2.30E-160 573.5 6596 cellular
organisms;Eukaryota;Opisthokonta;Metazoa;Eumetazoa;Bilateria;Protostomia;
Spiralia;Lophotrochozoa;Mollusca;Bivalvia;Autobranchia;Heteroconchia;Euhe
terodonta;Imparidentia;Venerida;Veneroidea;Veneridae;Mercenaria;Mercenari
a mercenaria
UdeM00000019291 6565 350.1 UdeM00000019291 XP_022338762.1 36.9 572
309 9 6 554 23 565 5.20E-93 350.1 6565 cellular
organisms;Eukaryota;Opisthokonta;Metazoa;Eumetazoa;Bilateria;Protostomia;
Spiralia;Lophotrochozoa;Mollusca;Bivalvia;Autobranchia;Pteriomorphia;Ostr
eida;Ostreoidea;Ostreidae;Crassostrea;Crassostrea virginica
UdeM00000019292 218467 108.6 UdeM00000019292 XP_023212519.1 55.4
101 44 1 1 101 1 100 4.70E-21 108.6 218467
cellular
organisms;Eukaryota;Opisthokonta;Metazoa;Eumetazoa;Bilateria;Protostomia;
Ecdysozoa;Panarthropoda;Arthropoda;Chelicerata;Arachnida;Scorpiones;Buthi
da;Buthoidea;Buthidae;Centruroides;Centruroides sculpturatus
UdeM00000019293 6596 71.2 UdeM00000019293 XP_045164744.1 33 224
115 11 31 242 50 250 3.00E-09 71.2 6596 cellular
organisms;Eukaryota;Opisthokonta;Metazoa;Eumetazoa;Bilateria;Protostomia;
Spiralia;Lophotrochozoa;Mollusca;Bivalvia;Autobranchia;Heteroconchia;Euhe

terodonta;Imparidentia;Venerida;Veneroidea;Veneridae;Mercenaria;Mercenari
a mercenaria
UdeM00000019294 6596 370.2 UdeM00000019294 XP_045165546.1 63.8 268
96 1 4 270 1 268 2.30E-99 370.2 6596 cellular
organisms;Eukaryota;Opisthokonta;Metazoa;Eumetazoa;Bilateria;Protostomia;
Spiralia;Lophotrochozoa;Mollusca;Bivalvia;Autobranchia;Heteroconchia;Euhe
terodonta;Imparidentia;Venerida;Veneroidea;Veneridae;Mercenaria;Mercenari
a mercenaria
UdeM00000019295 6596 175.3 UdeM00000019295 XP_045165545.1 59 139
55 1 1 139 1 137 5.70E-41 175.3 6596 cellular
organisms;Eukaryota;Opisthokonta;Metazoa;Eumetazoa;Bilateria;Protostomia;
Spiralia;Lophotrochozoa;Mollusca;Bivalvia;Autobranchia;Heteroconchia;Euhe
terodonta;Imparidentia;Venerida;Veneroidea;Veneridae;Mercenaria;Mercenari
a mercenaria
UdeM00000019296 6596 593.2 UdeM00000019296 XP_045165544.1 64.6 441
149 4 17 457 3 436 2.80E-166 593.2 6596 cellular
organisms;Eukaryota;Opisthokonta;Metazoa;Eumetazoa;Bilateria;Protostomia;
Spiralia;Lophotrochozoa;Mollusca;Bivalvia;Autobranchia;Heteroconchia;Euhe
terodonta;Imparidentia;Venerida;Veneroidea;Veneridae;Mercenaria;Mercenari
a mercenaria
UdeM00000019297 6596 4773 UdeM00000019297 XP_045165547.1 61.2 3589
1323 20 7 3573 10 3549 0.00E+00 4773 6596 cellular
organisms;Eukaryota;Opisthokonta;Metazoa;Eumetazoa;Bilateria;Protostomia;
Spiralia;Lophotrochozoa;Mollusca;Bivalvia;Autobranchia;Heteroconchia;Euhe
terodonta;Imparidentia;Venerida;Veneroidea;Veneridae;Mercenaria;Mercenari
a mercenaria
UdeM00000019298 6596 1888.6 UdeM00000019298 XP_045165547.1 59.3
1432 547 10 17 1437 2143 3549 0.00E+00 1888.6 6596
cellular
organisms;Eukaryota;Opisthokonta;Metazoa;Eumetazoa;Bilateria;Protostomia;
Spiralia;Lophotrochozoa;Mollusca;Bivalvia;Autobranchia;Heteroconchia;Euhe
terodonta;Imparidentia;Venerida;Veneroidea;Veneridae;Mercenaria;Mercenari
a mercenaria
UdeM00000019299 6579 669.1 UdeM00000019299 XP_033745766.1 47.9 1034
420 31 1 972 1 977 8.60E-189 669.1 6579 cellular
organisms;Eukaryota;Opisthokonta;Metazoa;Eumetazoa;Bilateria;Protostomia;
Spiralia;Lophotrochozoa;Mollusca;Bivalvia;Autobranchia;Pteriomorphia;Pect
inida;Pectinoidea;Pectinidae;Pecten;Pecten maximus
UdeM00000019300 6596 713.4 UdeM00000019300 XP_045164368.1 70.7 488
141 2 3 489 57 543 2.00E-202 713.4 6596 cellular
organisms;Eukaryota;Opisthokonta;Metazoa;Eumetazoa;Bilateria;Protostomia;
Spiralia;Lophotrochozoa;Mollusca;Bivalvia;Autobranchia;Heteroconchia;Euhe
terodonta;Imparidentia;Venerida;Veneroidea;Veneridae;Mercenaria;Mercenari
a mercenaria
UdeM00000019301 7994 334 UdeM00000019301 XP_007244151.1 91.1 180
16 0 1 180 1 180 1.20E-88 334 7994 cellular
organisms;Eukaryota;Opisthokonta;Metazoa;Eumetazoa;Bilateria;Deuterostomi
a;Chordata;Craniata;Vertebrata;Gnathostomata;Teleostomi;Euteleostomi;Acti
nopterygii;Actinopteri;Neopterygii;Teleostei;Osteoglossocephalai;Clupeoce
phala;Otomorpha;Ostariophysi;Otophysi;Characiphysae;Characiformes;Characo
idei;Characidae;Stethaprioninae;Astyanax;Astyanax mexicanus
UdeM00000019302 6596 236.1 UdeM00000019302 XP_045164259.1 63.9 166
60 0 3 168 2 167 3.50E-59 236.1 6596 cellular
organisms;Eukaryota;Opisthokonta;Metazoa;Eumetazoa;Bilateria;Protostomia;

Spiralia;Lophotrochozoa;Mollusca;Bivalvia;Autobranchia;Heteroconchia;Euheterodonta;Imparidentia;Venerida;Veneroidea;Veneridae;Mercenaria;Mercenaria mercenaria
UdeM00000019303 6596 1107 UdeM00000019303 XP_045164272.1 67.3 795 240 5 3 778 2 795 0.00E+00 1107 6596 cellular organisms;Eukaryota;Opisthokonta;Metazoa;Eumetazoa;Bilateria;Protostomia;Spiralia;Lophotrochozoa;Mollusca;Bivalvia;Autobranchia;Heteroconchia;Euheterodonta;Imparidentia;Venerida;Veneroidea;Veneridae;Mercenaria;Mercenaria mercenaria
UdeM00000019304 37653 279.6 UdeM00000019304 XP_014767589.1 55 222 98 1 288 507 278 499 9.50E-72 279.6 37653 cellular organisms;Eukaryota;Opisthokonta;Metazoa;Eumetazoa;Bilateria;Protostomia;Spiralia;Lophotrochozoa;Mollusca;Cephalopoda;Coleoidea;Octopodiformes;Octopoda;Incirrata;Octopodidae;Octopus;Octopus bimaculoides
UdeM00000019305 6573 274.6 UdeM00000019305 XP_021374318.1 91.5 142 12 0 34 175 1 142 8.60E-71 274.6 6573 cellular organisms;Eukaryota;Opisthokonta;Metazoa;Eumetazoa;Bilateria;Protostomia;Spiralia;Lophotrochozoa;Mollusca;Bivalvia;Autobranchia;Pteriomorphia;Pectinida;Pectinoidea;Pectinidae;Mizuhopecten;Mizuhopecten yessoensis
UdeM00000019306 6565 299.7 UdeM00000019306 XP_022296870.1 42.5 468 214 15 1 456 10 434 6.40E-78 299.7 6565 cellular organisms;Eukaryota;Opisthokonta;Metazoa;Eumetazoa;Bilateria;Protostomia;Spiralia;Lophotrochozoa;Mollusca;Bivalvia;Autobranchia;Pteriomorphia;Ostreida;Ostreoidea;Ostreidae;Crassostrea;Crassostrea virginica
UdeM00000019307 6596 214.9 UdeM00000019307 XP_045165897.1 58.5 159 66 0 298 456 172 330 2.10E-52 214.9 6596 cellular organisms;Eukaryota;Opisthokonta;Metazoa;Eumetazoa;Bilateria;Protostomia;Spiralia;Lophotrochozoa;Mollusca;Bivalvia;Autobranchia;Heteroconchia;Euheterodonta;Imparidentia;Venerida;Veneroidea;Veneridae;Mercenaria;Mercenaria mercenaria
UdeM00000019308 6596 218 UdeM00000019308 XP_045165897.1 53.1 177 83 0 11 187 154 330 1.10E-53 218 6596 cellular organisms;Eukaryota;Opisthokonta;Metazoa;Eumetazoa;Bilateria;Protostomia;Spiralia;Lophotrochozoa;Mollusca;Bivalvia;Autobranchia;Heteroconchia;Euheterodonta;Imparidentia;Venerida;Veneroidea;Veneridae;Mercenaria;Mercenaria mercenaria
UdeM00000019309 6579 191.4 UdeM00000019309 XP_033744636.1 50.6 176 84 3 3 177 9 182 9.60E-46 191.4 6579 cellular organisms;Eukaryota;Opisthokonta;Metazoa;Eumetazoa;Bilateria;Protostomia;Spiralia;Lophotrochozoa;Mollusca;Bivalvia;Autobranchia;Pteriomorphia;Pectinida;Pectinoidea;Pectinidae;Pecten;Pecten maximus
UdeM00000019310 6596 2083.1 UdeM00000019310 XP_045164400.1 61.1 1785 633 19 1 1739 1 1770 0.00E+00 2083.1 6596 cellular organisms;Eukaryota;Opisthokonta;Metazoa;Eumetazoa;Bilateria;Protostomia;Spiralia;Lophotrochozoa;Mollusca;Bivalvia;Autobranchia;Heteroconchia;Euheterodonta;Imparidentia;Venerida;Veneroidea;Veneridae;Mercenaria;Mercenaria mercenaria
UdeM00000019311 6573 646 UdeM00000019311 XP_021367542.1 69.6 624 185 4 5 623 1 624 5.00E-182 646 6573 cellular organisms;Eukaryota;Opisthokonta;Metazoa;Eumetazoa;Bilateria;Protostomia;Spiralia;Lophotrochozoa;Mollusca;Bivalvia;Autobranchia;Pteriomorphia;Pectinida;Pectinoidea;Pectinidae;Mizuhopecten;Mizuhopecten yessoensis

UdeM00000019312 6596 202.6 UdeM00000019312 XP_045165451.1 71.7 127
36 0 3 129 580 706 3.30E-49 202.6 6596 cellular
organisms;Eukaryota;Opisthokonta;Metazoa;Eumetazoa;Bilateria;Protostomia;
Spiralia;Lophotrochozoa;Mollusca;Bivalvia;Autobranchia;Heteroconchia;Euhe
terodonta;Imparidentia;Venerida;Veneroidea;Veneridae;Mercenaria;Mercenari
a mercenaria
UdeM00000019313 6596 622.1 UdeM00000019313 XP_045165452.1 51.6 591
276 4 6 594 3 585 7.70E-175 622.1 6596 cellular
organisms;Eukaryota;Opisthokonta;Metazoa;Eumetazoa;Bilateria;Protostomia;
Spiralia;Lophotrochozoa;Mollusca;Bivalvia;Autobranchia;Heteroconchia;Euhe
terodonta;Imparidentia;Venerida;Veneroidea;Veneridae;Mercenaria;Mercenari
a mercenaria
UdeM00000019317 6596 3854.3 UdeM00000019317 XP_045167257.1 51.9
3885 974 36 1 3809 50 3114 0.00E+00 3854.3 6596
cellular
organisms;Eukaryota;Opisthokonta;Metazoa;Eumetazoa;Bilateria;Protostomia;
Spiralia;Lophotrochozoa;Mollusca;Bivalvia;Autobranchia;Heteroconchia;Euhe
terodonta;Imparidentia;Venerida;Veneroidea;Veneridae;Mercenaria;Mercenari
a mercenaria
UdeM00000019318 6579 653.3 UdeM00000019318 XP_033744745.1 81 389
74 0 1 389 1 389 2.00E-184 653.3 6579 cellular
organisms;Eukaryota;Opisthokonta;Metazoa;Eumetazoa;Bilateria;Protostomia;
Spiralia;Lophotrochozoa;Mollusca;Bivalvia;Autobranchia;Pteriomorphia;Pect
inida;Pectinoidea;Pectinidae;Pecten;Pecten maximus
UdeM00000019319 6565 207.2 UdeM00000019319 XP_022299270.1 82 122
22 0 3 124 2 123 1.20E-50 207.2 6565 cellular
organisms;Eukaryota;Opisthokonta;Metazoa;Eumetazoa;Bilateria;Protostomia;
Spiralia;Lophotrochozoa;Mollusca;Bivalvia;Autobranchia;Pteriomorphia;Ostr
eida;Ostreoidea;Ostreidae;Crassostrea;Crassostrea virginica
UdeM00000019320 29159 253.8 UdeM00000019320 XP_011412152.2 55 218
93 4 25 242 31 243 2.20E-64 253.8 29159 cellular
organisms;Eukaryota;Opisthokonta;Metazoa;Eumetazoa;Bilateria;Protostomia;
Spiralia;Lophotrochozoa;Mollusca;Bivalvia;Autobranchia;Pteriomorphia;Ostr
eida;Ostreoidea;Ostreidae;Crassostrea;Crassostrea gigas
UdeM00000019321 6565 513.1 UdeM00000019321 XP_022299276.1 56.6 495
206 4 19 506 236 728 4.10E-142 513.1 6565 cellular
organisms;Eukaryota;Opisthokonta;Metazoa;Eumetazoa;Bilateria;Protostomia;
Spiralia;Lophotrochozoa;Mollusca;Bivalvia;Autobranchia;Pteriomorphia;Ostr
eida;Ostreoidea;Ostreidae;Crassostrea;Crassostrea virginica
UdeM00000019322 6596 600.1 UdeM00000019322 XP_045167238.1 43.8 950
442 17 1 899 1 909 4.60E-168 600.1 6596 cellular
organisms;Eukaryota;Opisthokonta;Metazoa;Eumetazoa;Bilateria;Protostomia;
Spiralia;Lophotrochozoa;Mollusca;Bivalvia;Autobranchia;Heteroconchia;Euhe
terodonta;Imparidentia;Venerida;Veneroidea;Veneridae;Mercenaria;Mercenari
a mercenaria
UdeM00000019323 6596 733.4 UdeM00000019323 XP_045166452.1 42.4 945
523 13 71 1000 73 1011 3.80E-208 733.4 6596 cellular
organisms;Eukaryota;Opisthokonta;Metazoa;Eumetazoa;Bilateria;Protostomia;
Spiralia;Lophotrochozoa;Mollusca;Bivalvia;Autobranchia;Heteroconchia;Euhe
terodonta;Imparidentia;Venerida;Veneroidea;Veneridae;Mercenaria;Mercenari
a mercenaria
UdeM00000019325 6573 140.6 UdeM00000019325 XP_021375759.1 37.8 241
117 4 1 241 1 208 3.00E-30 140.6 6573 cellular
organisms;Eukaryota;Opisthokonta;Metazoa;Eumetazoa;Bilateria;Protostomia;

Spiralia;Lophotrochozoa;Mollusca;Bivalvia;Autobranchia;Pteriomorphia;Pectinida;Pectinoidea;Pectinidae;Mizuhopecten;Mizuhopecten yessoensis
UdeM00000019326 6596 1999.6 UdeM00000019326 XP_045164400.1 60.6 1710 603 16 44 1724 103 1770 0.00E+00 1999.6 6596 cellular organisms;Eukaryota;Opisthokonta;Metazoa;Eumetazoa;Bilateria;Protostomia;Spiralia;Lophotrochozoa;Mollusca;Bivalvia;Autobranchia;Heteroconchia;Euheterodonta;Imparidentia;Venerida;Veneroidea;Veneridae;Mercenaria;Mercenaria mercenaria
UdeM00000019327 6596 1898.6 UdeM00000019327 XP_045164400.1 62.2 1588 522 17 1 1561 220 1756 0.00E+00 1898.6 6596 cellular organisms;Eukaryota;Opisthokonta;Metazoa;Eumetazoa;Bilateria;Protostomia;Spiralia;Lophotrochozoa;Mollusca;Bivalvia;Autobranchia;Heteroconchia;Euheterodonta;Imparidentia;Venerida;Veneroidea;Veneridae;Mercenaria;Mercenaria mercenaria
UdeM00000019328 6596 164.1 UdeM00000019328 XP_045164400.1 65.3 124 43 0 1 124 272 395 1.20E-37 164.1 6596 cellular organisms;Eukaryota;Opisthokonta;Metazoa;Eumetazoa;Bilateria;Protostomia;Spiralia;Lophotrochozoa;Mollusca;Bivalvia;Autobranchia;Heteroconchia;Euheterodonta;Imparidentia;Venerida;Veneroidea;Veneridae;Mercenaria;Mercenaria mercenaria
UdeM00000019329 6565 128.3 UdeM00000019329 XP_022298399.1 57.6 99 37 1 1 94 1354 1452 7.70E-27 128.3 6565 cellular organisms;Eukaryota;Opisthokonta;Metazoa;Eumetazoa;Bilateria;Protostomia;Spiralia;Lophotrochozoa;Mollusca;Bivalvia;Autobranchia;Pteriomorphia;Ostreida;Ostreoidea;Ostreidae;Crassostrea;Crassostrea virginica
UdeM00000019330 6596 1023.5 UdeM00000019330 XP_045164400.1 62.3 848 284 10 2 826 931 1765 1.50E-295 1023.5 6596 cellular organisms;Eukaryota;Opisthokonta;Metazoa;Eumetazoa;Bilateria;Protostomia;Spiralia;Lophotrochozoa;Mollusca;Bivalvia;Autobranchia;Heteroconchia;Euheterodonta;Imparidentia;Venerida;Veneroidea;Veneridae;Mercenaria;Mercenaria mercenaria
UdeM00000019331 6579 117.5 UdeM00000019331 XP_033746445.1 50.4 139 62 2 1 132 735 873 1.80E-23 117.5 6579 cellular organisms;Eukaryota;Opisthokonta;Metazoa;Eumetazoa;Bilateria;Protostomia;Spiralia;Lophotrochozoa;Mollusca;Bivalvia;Autobranchia;Pteriomorphia;Pectinida;Pectinoidea;Pectinidae;Pecten;Pecten maximus
UdeM00000019332 6596 682.2 UdeM00000019332 XP_045164400.1 72.4 468 117 2 1 457 271 737 5.50E-193 682.2 6596 cellular organisms;Eukaryota;Opisthokonta;Metazoa;Eumetazoa;Bilateria;Protostomia;Spiralia;Lophotrochozoa;Mollusca;Bivalvia;Autobranchia;Heteroconchia;Euheterodonta;Imparidentia;Venerida;Veneroidea;Veneridae;Mercenaria;Mercenaria mercenaria
UdeM00000019333 6596 228.8 UdeM00000019333 XP_045164400.1 65.4 179 60 1 1 177 220 398 6.90E-57 228.8 6596 cellular organisms;Eukaryota;Opisthokonta;Metazoa;Eumetazoa;Bilateria;Protostomia;Spiralia;Lophotrochozoa;Mollusca;Bivalvia;Autobranchia;Heteroconchia;Euheterodonta;Imparidentia;Venerida;Veneroidea;Veneridae;Mercenaria;Mercenaria mercenaria
UdeM00000019334 6596 72.8 UdeM00000019334 XP_045164400.1 59.4 69 24 1 27 91 201 269 2.70E-10 72.8 6596 cellular organisms;Eukaryota;Opisthokonta;Metazoa;Eumetazoa;Bilateria;Protostomia;

Spiralia;Lophotrochozoa;Mollusca;Bivalvia;Autobranchia;Heteroconchia;Euheterodonta;Imparidentia;Venerida;Veneroidea;Veneridae;Mercenaria;Mercenaria mercenaria
UdeM00000019335 6565 152.9 UdeM00000019335 XP_022298399.1 60.9 115 40 1 1 110 1354 1468 2.80E-34 152.9 6565 cellular organisms;Eukaryota;Opisthokonta;Metazoa;Eumetazoa;Bilateria;Protostomia;Spiralia;Lophotrochozoa;Mollusca;Bivalvia;Autobranchia;Pteriomorphia;Ostreida;Ostreoidea;Ostreidae;Crassostrea;Crassostrea virginica
UdeM00000019336 6596 2066.6 UdeM00000019336 XP_045164400.1 60.5 1808 627 24 1 1772 1 1756 0.00E+00 2066.6 6596 cellular organisms;Eukaryota;Opisthokonta;Metazoa;Eumetazoa;Bilateria;Protostomia;Spiralia;Lophotrochozoa;Mollusca;Bivalvia;Autobranchia;Heteroconchia;Euheterodonta;Imparidentia;Venerida;Veneroidea;Veneridae;Mercenaria;Mercenaria mercenaria
UdeM00000019337 6565 132.1 UdeM00000019337 XP_022299305.1 90.3 72 7 0 6 77 13 84 3.00E-28 132.1 6565 cellular organisms;Eukaryota;Opisthokonta;Metazoa;Eumetazoa;Bilateria;Protostomia;Spiralia;Lophotrochozoa;Mollusca;Bivalvia;Autobranchia;Pteriomorphia;Ostreida;Ostreoidea;Ostreidae;Crassostrea;Crassostrea virginica
UdeM00000019338 6596 215.7 UdeM00000019338 XP_045166851.1 56.3 174 73 2 1 171 1 174 5.30E-53 215.7 6596 cellular organisms;Eukaryota;Opisthokonta;Metazoa;Eumetazoa;Bilateria;Protostomia;Spiralia;Lophotrochozoa;Mollusca;Bivalvia;Autobranchia;Heteroconchia;Euheterodonta;Imparidentia;Venerida;Veneroidea;Veneridae;Mercenaria;Mercenaria mercenaria
UdeM00000019339 6596 233 UdeM00000019339 XP_045167134.1 44.7 331 97 6 1 327 1 249 5.50E-58 233 6596 cellular organisms;Eukaryota;Opisthokonta;Metazoa;Eumetazoa;Bilateria;Protostomia;Spiralia;Lophotrochozoa;Mollusca;Bivalvia;Autobranchia;Heteroconchia;Euheterodonta;Imparidentia;Venerida;Veneroidea;Veneridae;Mercenaria;Mercenaria mercenaria
UdeM00000019340 6596 146.7 UdeM00000019340 XP_045167134.1 34.8 382 186 10 40 377 353 715 7.70E-32 146.7 6596 cellular organisms;Eukaryota;Opisthokonta;Metazoa;Eumetazoa;Bilateria;Protostomia;Spiralia;Lophotrochozoa;Mollusca;Bivalvia;Autobranchia;Heteroconchia;Euheterodonta;Imparidentia;Venerida;Veneroidea;Veneridae;Mercenaria;Mercenaria mercenaria
UdeM00000019341 6596 494.2 UdeM00000019341 XP_045167134.1 63.1 398 139 5 8 401 851 1244 1.60E-136 494.2 6596 cellular organisms;Eukaryota;Opisthokonta;Metazoa;Eumetazoa;Bilateria;Protostomia;Spiralia;Lophotrochozoa;Mollusca;Bivalvia;Autobranchia;Heteroconchia;Euheterodonta;Imparidentia;Venerida;Veneroidea;Veneridae;Mercenaria;Mercenaria mercenaria
UdeM00000019342 6596 1221.8 UdeM00000019342 XP_045166252.1 67.1 916 273 6 19 908 13 926 0.00E+00 1221.8 6596 cellular organisms;Eukaryota;Opisthokonta;Metazoa;Eumetazoa;Bilateria;Protostomia;Spiralia;Lophotrochozoa;Mollusca;Bivalvia;Autobranchia;Heteroconchia;Euheterodonta;Imparidentia;Venerida;Veneroidea;Veneridae;Mercenaria;Mercenaria mercenaria
UdeM00000019343 6596 233.4 UdeM00000019343 XP_045165769.1 75.3 146 36 0 13 158 27 172 2.20E-58 233.4 6596 cellular organisms;Eukaryota;Opisthokonta;Metazoa;Eumetazoa;Bilateria;Protostomia;

Spiralia;Lophotrochozoa;Mollusca;Bivalvia;Autobranchia;Heteroconchia;Euheterodonta;Imparidentia;Venerida;Veneroidea;Veneridae;Mercenaria;Mercenaria mercenaria
UdeM00000019344 6596 964.9 UdeM00000019344 XP_045165769.1 63.3 739 250 5 1 723 204 937 5.70E-278 964.9 6596 cellular organisms;Eukaryota;Opisthokonta;Metazoa;Eumetazoa;Bilateria;Protostomia;Spiralia;Lophotrochozoa;Mollusca;Bivalvia;Autobranchia;Heteroconchia;Euheterodonta;Imparidentia;Venerida;Veneroidea;Veneridae;Mercenaria;Mercenaria mercenaria
UdeM00000019348 29159 79 UdeM00000019348 XP_034305523.1 41.7 96 48 2 20 108 90 184 4.40E-12 79 29159 cellular organisms;Eukaryota;Opisthokonta;Metazoa;Eumetazoa;Bilateria;Protostomia;Spiralia;Lophotrochozoa;Mollusca;Bivalvia;Autobranchia;Pteriomorphia;Ostreida;Ostreoidea;Ostreidae;Crassostrea;Crassostrea gigas
UdeM00000019350 29159 201.8 UdeM00000019350 XP_034305523.1 54.2 166 76 0 1 166 737 902 6.80E-49 201.8 29159 cellular organisms;Eukaryota;Opisthokonta;Metazoa;Eumetazoa;Bilateria;Protostomia;Spiralia;Lophotrochozoa;Mollusca;Bivalvia;Autobranchia;Pteriomorphia;Ostreida;Ostreoidea;Ostreidae;Crassostrea;Crassostrea gigas
UdeM00000019351 6596 780.8 UdeM00000019351 XP_045167316.1 47 815 414 4 6 816 5 805 1.70E-222 780.8 6596 cellular organisms;Eukaryota;Opisthokonta;Metazoa;Eumetazoa;Bilateria;Protostomia;Spiralia;Lophotrochozoa;Mollusca;Bivalvia;Autobranchia;Heteroconchia;Euheterodonta;Imparidentia;Venerida;Veneroidea;Veneridae;Mercenaria;Mercenaria mercenaria
UdeM00000019352 6596 367.5 UdeM00000019352 XP_045165397.1 49.2 317 152 2 3 315 4 315 1.70E-98 367.5 6596 cellular organisms;Eukaryota;Opisthokonta;Metazoa;Eumetazoa;Bilateria;Protostomia;Spiralia;Lophotrochozoa;Mollusca;Bivalvia;Autobranchia;Heteroconchia;Euheterodonta;Imparidentia;Venerida;Veneroidea;Veneridae;Mercenaria;Mercenaria mercenaria
UdeM00000019353 6596 370.2 UdeM00000019353 XP_045165397.1 49.4 318 152 2 3 316 4 316 2.70E-99 370.2 6596 cellular organisms;Eukaryota;Opisthokonta;Metazoa;Eumetazoa;Bilateria;Protostomia;Spiralia;Lophotrochozoa;Mollusca;Bivalvia;Autobranchia;Heteroconchia;Euheterodonta;Imparidentia;Venerida;Veneroidea;Veneridae;Mercenaria;Mercenaria mercenaria
UdeM00000019354 6596 369 UdeM00000019354 XP_045165397.1 53.4 311 142 3 11 320 8 316 6.10E-99 369 6596 cellular organisms;Eukaryota;Opisthokonta;Metazoa;Eumetazoa;Bilateria;Protostomia;Spiralia;Lophotrochozoa;Mollusca;Bivalvia;Autobranchia;Heteroconchia;Euheterodonta;Imparidentia;Venerida;Veneroidea;Veneridae;Mercenaria;Mercenaria mercenaria
UdeM00000019355 6596 337.4 UdeM00000019355 XP_045165397.1 52.6 291 137 1 3 293 27 316 1.80E-89 337.4 6596 cellular organisms;Eukaryota;Opisthokonta;Metazoa;Eumetazoa;Bilateria;Protostomia;Spiralia;Lophotrochozoa;Mollusca;Bivalvia;Autobranchia;Heteroconchia;Euheterodonta;Imparidentia;Venerida;Veneroidea;Veneridae;Mercenaria;Mercenaria mercenaria
UdeM00000019356 6596 397.5 UdeM00000019356 XP_045165397.1 55 309 138 1 7 315 8 315 1.60E-107 397.5 6596 cellular organisms;Eukaryota;Opisthokonta;Metazoa;Eumetazoa;Bilateria;Protostomia;Spiralia;Lophotrochozoa;Mollusca;Bivalvia;Autobranchia;Heteroconchia;Euhe

terodonta;Imparidentia;Venerida;Veneroidea;Veneridae;Mercenaria;Mercenaria mercenaria
UdeM00000019357 6596 157.9 UdeM00000019357 XP_045165397.1 45 151 82 1 29 179 92 241 1.20E-35 157.9 6596 cellular organisms;Eukaryota;Opisthokonta;Metazoa;Eumetazoa;Bilateria;Protostomia;Spiralia;Lophotrochozoa;Mollusca;Bivalvia;Autobranchia;Heteroconchia;Euheterodonta;Imparidentia;Venerida;Veneroidea;Veneridae;Mercenaria;Mercenaria mercenaria
UdeM00000019358 6596 322 UdeM00000019358 XP_045165424.1 38.1 528 298 11 24 534 87 602 1.80E-84 322 6596 cellular organisms;Eukaryota;Opisthokonta;Metazoa;Eumetazoa;Bilateria;Protostomia;Spiralia;Lophotrochozoa;Mollusca;Bivalvia;Autobranchia;Heteroconchia;Euheterodonta;Imparidentia;Venerida;Veneroidea;Veneridae;Mercenaria;Mercenaria mercenaria
UdeM00000019359 6579 473 UdeM00000019359 XP_033744665.1 62.5 381 137 3 1 375 1 381 3.50E-130 473 6579 cellular organisms;Eukaryota;Opisthokonta;Metazoa;Eumetazoa;Bilateria;Protostomia;Spiralia;Lophotrochozoa;Mollusca;Bivalvia;Autobranchia;Pteriomorphia;Pectinida;Pectinoidea;Pectinidae;Pecten;Pecten maximus
UdeM00000019360 6565 102.4 UdeM00000019360 XP_022301665.1 30.9 265 153 7 9 250 27 284 8.50E-19 102.4 6565 cellular organisms;Eukaryota;Opisthokonta;Metazoa;Eumetazoa;Bilateria;Protostomia;Spiralia;Lophotrochozoa;Mollusca;Bivalvia;Autobranchia;Pteriomorphia;Ostreida;Ostreoidea;Ostreidae;Crassostrea;Crassostrea virginica
UdeM00000019361 6596 547.4 UdeM00000019361 XP_045166763.1 63.5 430 154 2 1 427 1 430 1.70E-152 547.4 6596 cellular organisms;Eukaryota;Opisthokonta;Metazoa;Eumetazoa;Bilateria;Protostomia;Spiralia;Lophotrochozoa;Mollusca;Bivalvia;Autobranchia;Heteroconchia;Euheterodonta;Imparidentia;Venerida;Veneroidea;Veneridae;Mercenaria;Mercenaria mercenaria
UdeM00000019362 6596 319.7 UdeM00000019362 XP_045166331.1 52.6 369 151 8 1 366 1 348 4.80E-84 319.7 6596 cellular organisms;Eukaryota;Opisthokonta;Metazoa;Eumetazoa;Bilateria;Protostomia;Spiralia;Lophotrochozoa;Mollusca;Bivalvia;Autobranchia;Heteroconchia;Euheterodonta;Imparidentia;Venerida;Veneroidea;Veneridae;Mercenaria;Mercenaria mercenaria
UdeM00000019363 6596 595.1 UdeM00000019363 XP_045166333.1 75.3 392 90 5 3 388 350 740 6.30E-167 595.1 6596 cellular organisms;Eukaryota;Opisthokonta;Metazoa;Eumetazoa;Bilateria;Protostomia;Spiralia;Lophotrochozoa;Mollusca;Bivalvia;Autobranchia;Heteroconchia;Euheterodonta;Imparidentia;Venerida;Veneroidea;Veneridae;Mercenaria;Mercenaria mercenaria
UdeM00000019368 6596 200.3 UdeM00000019368 XP_045165360.1 57.1 154 66 0 1 154 1 154 1.80E-48 200.3 6596 cellular organisms;Eukaryota;Opisthokonta;Metazoa;Eumetazoa;Bilateria;Protostomia;Spiralia;Lophotrochozoa;Mollusca;Bivalvia;Autobranchia;Heteroconchia;Euheterodonta;Imparidentia;Venerida;Veneroidea;Veneridae;Mercenaria;Mercenaria mercenaria
UdeM00000019367 6596 202.6 UdeM00000019367 XP_045165360.1 58.8 153 63 0 9 161 2 154 3.80E-49 202.6 6596 cellular organisms;Eukaryota;Opisthokonta;Metazoa;Eumetazoa;Bilateria;Protostomia;Spiralia;Lophotrochozoa;Mollusca;Bivalvia;Autobranchia;Heteroconchia;Euheterodonta;Imparidentia;Venerida;Veneroidea;Veneridae;Mercenaria;Mercenaria mercenaria

UdeM00000019369 6596 191.8 UdeM00000019369 XP_045164846.1 56.6 159
68 1 3 161 2 159 6.70E-46 191.8 6596 cellular
organisms;Eukaryota;Opisthokonta;Metazoa;Eumetazoa;Bilateria;Protostomia;Spiralia;Lophotrochozoa;Mollusca;Bivalvia;Autobranchia;Heteroconchia;Euheterodonta;Imparidentia;Venerida;Veneroidea;Veneridae;Mercenaria;Mercenaria mercenaria
UdeM00000019370 6596 238.8 UdeM00000019370 XP_045164850.1 74.1 158
40 1 1 157 1 158 4.70E-60 238.8 6596 cellular
organisms;Eukaryota;Opisthokonta;Metazoa;Eumetazoa;Bilateria;Protostomia;Spiralia;Lophotrochozoa;Mollusca;Bivalvia;Autobranchia;Heteroconchia;Euheterodonta;Imparidentia;Venerida;Veneroidea;Veneridae;Mercenaria;Mercenaria mercenaria
UdeM00000019371 29159 140.2 UdeM00000019371 XP_034317709.1 42.4 191
100 6 6 193 303 486 2.90E-30 140.2 29159 cellular
organisms;Eukaryota;Opisthokonta;Metazoa;Eumetazoa;Bilateria;Protostomia;Spiralia;Lophotrochozoa;Mollusca;Bivalvia;Autobranchia;Pteriomorphia;Ostreida;Ostreoidea;Ostreidae;Crassostrea;Crassostrea gigas
UdeM00000019372 29159 95.1 UdeM00000019372 XP_034317709.1 33 218
116 7 14 230 202 390 1.70E-16 95.1 29159 cellular
organisms;Eukaryota;Opisthokonta;Metazoa;Eumetazoa;Bilateria;Protostomia;Spiralia;Lophotrochozoa;Mollusca;Bivalvia;Autobranchia;Pteriomorphia;Ostreida;Ostreoidea;Ostreidae;Crassostrea;Crassostrea gigas
UdeM00000019373 6573 177.2 UdeM00000019373 XP_021356984.1 52.3 176
81 3 44 216 32 207 2.40E-41 177.2 6573 cellular
organisms;Eukaryota;Opisthokonta;Metazoa;Eumetazoa;Bilateria;Protostomia;Spiralia;Lophotrochozoa;Mollusca;Bivalvia;Autobranchia;Pteriomorphia;Pectinida;Pectinoidea;Pectinidae;Mizuhopecten;Mizuhopecten yessoensis
UdeM00000019374 6596 414.1 UdeM00000019374 XP_045164956.1 49.8 402
195 3 10 408 6 403 2.10E-112 414.1 6596 cellular
organisms;Eukaryota;Opisthokonta;Metazoa;Eumetazoa;Bilateria;Protostomia;Spiralia;Lophotrochozoa;Mollusca;Bivalvia;Autobranchia;Heteroconchia;Euheterodonta;Imparidentia;Venerida;Veneroidea;Veneridae;Mercenaria;Mercenaria mercenaria
UdeM00000019376 6565 1289.2 UdeM00000019376 XP_022297721.1 67.8
1390 411 12 166 1535 86 1459 0.00E+00 1289.2 6565
cellular
organisms;Eukaryota;Opisthokonta;Metazoa;Eumetazoa;Bilateria;Protostomia;Spiralia;Lophotrochozoa;Mollusca;Bivalvia;Autobranchia;Pteriomorphia;Ostreida;Ostreoidea;Ostreidae;Crassostrea;Crassostrea virginica
UdeM00000019377 6596 883.6 UdeM00000019377 XP_045165907.1 58.8 687
268 7 9 684 8 690 1.60E-253 883.6 6596 cellular
organisms;Eukaryota;Opisthokonta;Metazoa;Eumetazoa;Bilateria;Protostomia;Spiralia;Lophotrochozoa;Mollusca;Bivalvia;Autobranchia;Heteroconchia;Euheterodonta;Imparidentia;Venerida;Veneroidea;Veneridae;Mercenaria;Mercenaria mercenaria
UdeM00000019378 6596 912.1 UdeM00000019378 XP_045165907.1 58 712
283 6 8 707 7 714 4.30E-262 912.1 6596 cellular
organisms;Eukaryota;Opisthokonta;Metazoa;Eumetazoa;Bilateria;Protostomia;Spiralia;Lophotrochozoa;Mollusca;Bivalvia;Autobranchia;Heteroconchia;Euheterodonta;Imparidentia;Venerida;Veneroidea;Veneridae;Mercenaria;Mercenaria mercenaria
UdeM00000019379 7574 105.5 UdeM00000019379 XP_013396733.1 35.1 194
89 2 108 264 55 248 1.00E-19 105.5 7574 cellular
organisms;Eukaryota;Opisthokonta;Metazoa;Eumetazoa;Bilateria;Protostomia;

Spiralia;Lophotrochozoa;Brachiopoda;Linguliformea;Lingulata;Lingulida;Linguloidea;Lingulidae;Lingula;Lingula anatina
UdeM00000019380 6596 120.2 UdeM00000019380 XP_045216733.1 42.2 135 71 1 5 139 385 512 2.20E-24 120.2 6596 cellular organisms;Eukaryota;Opisthokonta;Metazoa;Eumetazoa;Bilateria;Protostomia;Spiralia;Lophotrochozoa;Mollusca;Bivalvia;Autobranchia;Heteroconchia;Euheterodonta;Imparidentia;Venerida;Veneroidea;Veneridae;Mercenaria;Mercenaria mercenaria
UdeM00000019381 6596 238 UdeM00000019381 XP_045216732.1 44.5 272 130 3 7 272 248 504 1.60E-59 238 6596 cellular organisms;Eukaryota;Opisthokonta;Metazoa;Eumetazoa;Bilateria;Protostomia;Spiralia;Lophotrochozoa;Mollusca;Bivalvia;Autobranchia;Heteroconchia;Euheterodonta;Imparidentia;Venerida;Veneroidea;Veneridae;Mercenaria;Mercenaria mercenaria
UdeM00000019382 6596 170.6 UdeM00000019382 XP_045216732.1 52 150 71 1 96 245 234 382 2.50E-39 170.6 6596 cellular organisms;Eukaryota;Opisthokonta;Metazoa;Eumetazoa;Bilateria;Protostomia;Spiralia;Lophotrochozoa;Mollusca;Bivalvia;Autobranchia;Heteroconchia;Euheterodonta;Imparidentia;Venerida;Veneroidea;Veneridae;Mercenaria;Mercenaria mercenaria
UdeM00000019383 6596 66.2 UdeM00000019383 XP_045216733.1 35.6 87 49 1 43 129 433 512 3.60E-08 66.2 6596 cellular organisms;Eukaryota;Opisthokonta;Metazoa;Eumetazoa;Bilateria;Protostomia;Spiralia;Lophotrochozoa;Mollusca;Bivalvia;Autobranchia;Heteroconchia;Euheterodonta;Imparidentia;Venerida;Veneroidea;Veneridae;Mercenaria;Mercenaria mercenaria
UdeM00000019385 6573 88.2 UdeM00000019385 XP_021370045.1 26.7 243 158 6 105 333 730 966 2.90E-14 88.2 6573 cellular organisms;Eukaryota;Opisthokonta;Metazoa;Eumetazoa;Bilateria;Protostomia;Spiralia;Lophotrochozoa;Mollusca;Bivalvia;Autobranchia;Pteriomorphia;Pectinida;Pectinoidea;Pectinidae;Mizuhopecten;Mizuhopecten yessoensis
UdeM00000019384 6573 88.2 UdeM00000019384 XP_021370045.1 26.7 243 158 6 182 410 730 966 3.40E-14 88.2 6573 cellular organisms;Eukaryota;Opisthokonta;Metazoa;Eumetazoa;Bilateria;Protostomia;Spiralia;Lophotrochozoa;Mollusca;Bivalvia;Autobranchia;Pteriomorphia;Pectinida;Pectinoidea;Pectinidae;Mizuhopecten;Mizuhopecten yessoensis
UdeM00000019386 29159 371.3 UdeM00000019386 XP_011415226.1 61.8 296 112 1 1 295 1 296 1.30E-99 371.3 29159 cellular organisms;Eukaryota;Opisthokonta;Metazoa;Eumetazoa;Bilateria;Protostomia;Spiralia;Lophotrochozoa;Mollusca;Bivalvia;Autobranchia;Pteriomorphia;Ostreida;Ostreoidea;Ostreidae;Crassostrea;Crassostrea gigas
UdeM00000019388 6573 380.9 UdeM00000019388 XP_021351964.1 56 316 128 5 13 321 15 326 1.60E-102 380.9 6573 cellular organisms;Eukaryota;Opisthokonta;Metazoa;Eumetazoa;Bilateria;Protostomia;Spiralia;Lophotrochozoa;Mollusca;Bivalvia;Autobranchia;Pteriomorphia;Pectinida;Pectinoidea;Pectinidae;Mizuhopecten;Mizuhopecten yessoensis
UdeM00000019387 6573 503.4 UdeM00000019387 XP_021351964.1 57.5 407 162 5 13 412 15 417 2.80E-139 503.4 6573 cellular organisms;Eukaryota;Opisthokonta;Metazoa;Eumetazoa;Bilateria;Protostomia;Spiralia;Lophotrochozoa;Mollusca;Bivalvia;Autobranchia;Pteriomorphia;Pectinida;Pectinoidea;Pectinidae;Mizuhopecten;Mizuhopecten yessoensis
UdeM00000019389 6596 588.2 UdeM00000019389 XP_045164321.1 43.3 781 418 14 10 778 36 803 1.60E-164 588.2 6596 cellular organisms;Eukaryota;Opisthokonta;Metazoa;Eumetazoa;Bilateria;Protostomia;

Spiralia;Lophotrochozoa;Mollusca;Bivalvia;Autobranchia;Heteroconchia;Euheterodonta;Imparidentia;Venerida;Veneroidea;Veneridae;Mercenaria;Mercenaria mercenaria
UdeM00000019390 6596 580.9 UdeM00000019390 XP_045164321.1 44 702 379 7 5 698 36 731 2.50E-162 580.9 6596 cellular organisms;Eukaryota;Opisthokonta;Metazoa;Eumetazoa;Bilateria;Protostomia;Spiralia;Lophotrochozoa;Mollusca;Bivalvia;Autobranchia;Heteroconchia;Euheterodonta;Imparidentia;Venerida;Veneroidea;Veneridae;Mercenaria;Mercenaria mercenaria
UdeM00000019391 143950 61.2 UdeM00000019391 XP_025407278.1 35 123 67 4 272 390 117 230 3.30E-06 61.2 143950 cellular organisms;Eukaryota;Opisthokonta;Metazoa;Eumetazoa;Bilateria;Protostomia;Ecdysozoa;Panarthropoda;Arthropoda;Mandibulata;Pancrustacea;Hexapoda;Insecta;Dicondylia;Pterygota;Neoptera;Paraneoptera;Hemiptera;Sternorrhyncha;Aphidomorpha;Aphidoidea;Aphididae;Chaitophorinae;Sipha;Sipha flava
UdeM00000019392 6412 144.4 UdeM00000019392 XP_009015452.1 47.3 146 77 0 1 146 1 146 1.10E-31 144.4 6412 cellular organisms;Eukaryota;Opisthokonta;Metazoa;Eumetazoa;Bilateria;Protostomia;Spiralia;Lophotrochozoa;Annelida;Clitellata;Hirudinea;Rhynchobdellida;Glossiphoniidae;Helobdella;Helobdella robusta
UdeM00000019393 7719 204.5 UdeM00000019393 XP_004227365.1 54.7 161 73 0 6 166 2 162 1.00E-49 204.5 7719 cellular organisms;Eukaryota;Opisthokonta;Metazoa;Eumetazoa;Bilateria;Deuterostomia;Chordata;Tunicata;Ascidiacea;Phlebobranchia;Cionidae;Ciona;Ciona intestinalis
UdeM00000019394 6573 109.8 UdeM00000019394 XP_021368922.1 57.1 98 39 2 5 100 21 117 6.90E-21 109.8 6573 cellular organisms;Eukaryota;Opisthokonta;Metazoa;Eumetazoa;Bilateria;Protostomia;Spiralia;Lophotrochozoa;Mollusca;Bivalvia;Autobranchia;Pteriomorphia;Pectinida;Pectinoidea;Pectinidae;Mizuhopecten;Mizuhopecten yessoensis
UdeM00000019395 6596 432.6 UdeM00000019395 XP_045167166.1 78.7 268 56 1 37 304 223 489 4.30E-118 432.6 6596 cellular organisms;Eukaryota;Opisthokonta;Metazoa;Eumetazoa;Bilateria;Protostomia;Spiralia;Lophotrochozoa;Mollusca;Bivalvia;Autobranchia;Heteroconchia;Euheterodonta;Imparidentia;Venerida;Veneroidea;Veneridae;Mercenaria;Mercenaria mercenaria
UdeM00000019396 6596 429.9 UdeM00000019396 XP_045167166.1 81.7 252 46 0 12 263 238 489 2.40E-117 429.9 6596 cellular organisms;Eukaryota;Opisthokonta;Metazoa;Eumetazoa;Bilateria;Protostomia;Spiralia;Lophotrochozoa;Mollusca;Bivalvia;Autobranchia;Heteroconchia;Euheterodonta;Imparidentia;Venerida;Veneroidea;Veneridae;Mercenaria;Mercenaria mercenaria
UdeM00000019397 211228 164.5 UdeM00000019397 XP_015592401.1 32.4 315 184 5 15 312 3 305 2.50E-37 164.5 211228 cellular organisms;Eukaryota;Opisthokonta;Metazoa;Eumetazoa;Bilateria;Protostomia;Ecdysozoa;Panarthropoda;Arthropoda;Mandibulata;Pancrustacea;Hexapoda;Insecta;Dicondylia;Pterygota;Neoptera;Endopterygota;Hymenoptera;Cephoidea;Cephidae;Cephus;Cephus cinctus
UdeM00000019398 29159 236.5 UdeM00000019398 XP_034306524.1 53.7 246 104 3 1 245 1 237 3.60E-59 236.5 29159 cellular organisms;Eukaryota;Opisthokonta;Metazoa;Eumetazoa;Bilateria;Protostomia;

Spiralia;Lophotrochozoa;Mollusca;Bivalvia;Autobranchia;Pteriomorphia;Ostreida;Ostreoidea;Ostreidae;Crassostrea;Crassostrea gigas
UdeM00000019399 6596 1168.3 UdeM00000019399 XP_045166623.1 79.1 818 165 3 1 812 1 818 0.00E+00 1168.3 6596 cellular organisms;Eukaryota;Opisthokonta;Metazoa;Eumetazoa;Bilateria;Protostomia;Spiralia;Lophotrochozoa;Mollusca;Bivalvia;Autobranchia;Heteroconchia;Euheterodonta;Imparidentia;Venerida;Veneroidea;Veneridae;Mercenaria;Mercenaria mercenaria
UdeM00000019400 6596 1196 UdeM00000019400 XP_045166623.1 83.4 821 133 2 1 821 1 818 0.00E+00 1196 6596 cellular organisms;Eukaryota;Opisthokonta;Metazoa;Eumetazoa;Bilateria;Protostomia;Spiralia;Lophotrochozoa;Mollusca;Bivalvia;Autobranchia;Heteroconchia;Euheterodonta;Imparidentia;Venerida;Veneroidea;Veneridae;Mercenaria;Mercenaria mercenaria
UdeM00000019401 6596 641 UdeM00000019401 XP_045166493.1 61.5 592 200 6 471 1059 529 1095 2.70E-180 641 6596 cellular organisms;Eukaryota;Opisthokonta;Metazoa;Eumetazoa;Bilateria;Protostomia;Spiralia;Lophotrochozoa;Mollusca;Bivalvia;Autobranchia;Heteroconchia;Euheterodonta;Imparidentia;Venerida;Veneroidea;Veneridae;Mercenaria;Mercenaria mercenaria
UdeM00000019402 6596 272.3 UdeM00000019402 XP_045166493.1 54.2 347 124 3 47 361 1096 1439 9.70E-70 272.3 6596 cellular organisms;Eukaryota;Opisthokonta;Metazoa;Eumetazoa;Bilateria;Protostomia;Spiralia;Lophotrochozoa;Mollusca;Bivalvia;Autobranchia;Heteroconchia;Euheterodonta;Imparidentia;Venerida;Veneroidea;Veneridae;Mercenaria;Mercenaria mercenaria
UdeM00000019403 29159 591.7 UdeM00000019403 XP_011444116.2 65.5 490 146 7 5 490 26 496 8.80E-166 591.7 29159 cellular organisms;Eukaryota;Opisthokonta;Metazoa;Eumetazoa;Bilateria;Protostomia;Spiralia;Lophotrochozoa;Mollusca;Bivalvia;Autobranchia;Pteriomorphia;Ostreida;Ostreoidea;Ostreidae;Crassostrea;Crassostrea gigas
UdeM00000019404 29159 554.7 UdeM00000019404 XP_011444116.2 65.8 453 132 7 5 453 26 459 1.20E-154 554.7 29159 cellular organisms;Eukaryota;Opisthokonta;Metazoa;Eumetazoa;Bilateria;Protostomia;Spiralia;Lophotrochozoa;Mollusca;Bivalvia;Autobranchia;Pteriomorphia;Ostreida;Ostreoidea;Ostreidae;Crassostrea;Crassostrea gigas
UdeM00000019407 29159 74.3 UdeM00000019407 XP_034307878.1 32.3 99 67 0 1 99 92 190 2.90E-10 74.3 29159 cellular organisms;Eukaryota;Opisthokonta;Metazoa;Eumetazoa;Bilateria;Protostomia;Spiralia;Lophotrochozoa;Mollusca;Bivalvia;Autobranchia;Pteriomorphia;Ostreida;Ostreoidea;Ostreidae;Crassostrea;Crassostrea gigas
UdeM00000019408 29159 212.2 UdeM00000019408 XP_034312908.1 28 649 343 21 9 564 6 623 1.70E-51 212.2 29159 cellular organisms;Eukaryota;Opisthokonta;Metazoa;Eumetazoa;Bilateria;Protostomia;Spiralia;Lophotrochozoa;Mollusca;Bivalvia;Autobranchia;Pteriomorphia;Ostreida;Ostreoidea;Ostreidae;Crassostrea;Crassostrea gigas
UdeM00000019409 6579 76.3 UdeM00000019409 XP_033744435.1 47.3 93 46 3 10 101 462 552 2.80E-11 76.3 6579 cellular organisms;Eukaryota;Opisthokonta;Metazoa;Eumetazoa;Bilateria;Protostomia;Spiralia;Lophotrochozoa;Mollusca;Bivalvia;Autobranchia;Pteriomorphia;Pectinida;Pectinoidea;Pectinidae;Pecten;Pecten maximus
UdeM00000019410 6573 67.4 UdeM00000019410 XP_021345511.1 67.9 53 17 0 4 56 43 95 1.30E-08 67.4 6573 cellular

organisms;Eukaryota;Opisthokonta;Metazoa;Eumetazoa;Bilateria;Protostomia;Spiralia;Lophotrochozoa;Mollusca;Bivalvia;Autobranchia;Pteriomorphia;Pectinida;Pectinoidea;Pectinidae;Mizuhopecten;Mizuhopecten yessoensis
UdeM00000019411 6596 747.7 UdeM00000019411 XP_045167219.1 68.9 556 144 5 1 555 87 614 1.10E-212 747.7 6596 cellular organisms;Eukaryota;Opisthokonta;Metazoa;Eumetazoa;Bilateria;Protostomia;Spiralia;Lophotrochozoa;Mollusca;Bivalvia;Autobranchia;Heteroconchia;Euheterodonta;Imparidentia;Venerida;Veneroidea;Veneridae;Mercenaria;Mercenaria mercenaria
UdeM00000019412 6596 808.5 UdeM00000019412 XP_045165346.1 48.3 1095 414 28 612 1636 652 1664 1.50E-230 808.5 6596 cellular organisms;Eukaryota;Opisthokonta;Metazoa;Eumetazoa;Bilateria;Protostomia;Spiralia;Lophotrochozoa;Mollusca;Bivalvia;Autobranchia;Heteroconchia;Euheterodonta;Imparidentia;Venerida;Veneroidea;Veneridae;Mercenaria;Mercenaria mercenaria
UdeM00000019413 1735272 134 UdeM00000019413 XP_041352779.1 43.6 156 81 3 2 155 55 205 2.60E-28 134 1735272 cellular organisms;Eukaryota;Opisthokonta;Metazoa;Eumetazoa;Bilateria;Protostomia;Spiralia;Lophotrochozoa;Mollusca;Gastropoda;Neomphalina;Neomphaloidea;Peltospiridae;Gigantopelta;Gigantopelta aegis
UdeM00000019414 1735272 131.3 UdeM00000019414 XP_041352779.1 43.2 155 81 3 2 154 55 204 1.00E-27 131.3 1735272 cellular organisms;Eukaryota;Opisthokonta;Metazoa;Eumetazoa;Bilateria;Protostomia;Spiralia;Lophotrochozoa;Mollusca;Gastropoda;Neomphalina;Neomphaloidea;Peltospiridae;Gigantopelta;Gigantopelta aegis
UdeM00000019416 6596 1120.5 UdeM00000019416 XP_045166222.1 61.1 906 333 9 17 908 396 1296 0.00E+00 1120.5 6596 cellular organisms;Eukaryota;Opisthokonta;Metazoa;Eumetazoa;Bilateria;Protostomia;Spiralia;Lophotrochozoa;Mollusca;Bivalvia;Autobranchia;Heteroconchia;Euheterodonta;Imparidentia;Venerida;Veneroidea;Veneridae;Mercenaria;Mercenaria mercenaria
UdeM00000019418 6500 98.6 UdeM00000019418 XP_012938236.1 34.6 156 95 2 13 161 19 174 7.70E-18 98.6 6500 cellular organisms;Eukaryota;Opisthokonta;Metazoa;Eumetazoa;Bilateria;Protostomia;Spiralia;Lophotrochozoa;Mollusca;Gastropoda;Heterobranchia;Euthyneura;Tectipleura;Aplysiida;Aplysioidea;Aplysiidae;Aplysia;Aplysia californica
UdeM00000019417 6500 98.6 UdeM00000019417 XP_012938236.1 33.5 167 101 3 7 166 11 174 8.00E-18 98.6 6500 cellular organisms;Eukaryota;Opisthokonta;Metazoa;Eumetazoa;Bilateria;Protostomia;Spiralia;Lophotrochozoa;Mollusca;Gastropoda;Heterobranchia;Euthyneura;Tectipleura;Aplysiida;Aplysioidea;Aplysiidae;Aplysia;Aplysia californica
UdeM00000019419 29159 231.5 UdeM00000019419 XP_034317976.1 59 178 72 1 1 177 484 661 8.40E-58 231.5 29159 cellular organisms;Eukaryota;Opisthokonta;Metazoa;Eumetazoa;Bilateria;Protostomia;Spiralia;Lophotrochozoa;Mollusca;Bivalvia;Autobranchia;Pteriomorphia;Ostreida;Ostreoidea;Ostreidae;Crassostrea;Crassostrea gigas
UdeM00000019420 29159 599.7 UdeM00000019420 XP_034317976.1 48.5 655 304 8 2 626 10 661 4.10E-168 599.7 29159 cellular organisms;Eukaryota;Opisthokonta;Metazoa;Eumetazoa;Bilateria;Protostomia;Spiralia;Lophotrochozoa;Mollusca;Bivalvia;Autobranchia;Pteriomorphia;Ostreida;Ostreoidea;Ostreidae;Crassostrea;Crassostrea gigas

UdeM00000019421 6596 441.4 UdeM00000019421 XP_045164186.1 62.6 356
126 2 1 350 1 355 1.10E-120 441.4 6596 cellular
organisms;Eukaryota;Opisthokonta;Metazoa;Eumetazoa;Bilateria;Protostomia;Spiralia;Lophotrochozoa;Mollusca;Bivalvia;Autobranchia;Heteroconchia;Euheterodonta;Imparidentia;Venerida;Veneroidea;Veneridae;Mercenaria;Mercenaria mercenaria
UdeM00000019422 6596 1166.8 UdeM00000019422 XP_045164511.1 75.6
766 181 3 39 798 29 794 0.00E+00 1166.8 6596
cellular
organisms;Eukaryota;Opisthokonta;Metazoa;Eumetazoa;Bilateria;Protostomia;Spiralia;Lophotrochozoa;Mollusca;Bivalvia;Autobranchia;Heteroconchia;Euheterodonta;Imparidentia;Venerida;Veneroidea;Veneridae;Mercenaria;Mercenaria mercenaria
UdeM00000044476 441366 170.2 UdeM00000044476 XP_028296753.1 38.4
284 158 9 6 285 32 302 3.70E-39 170.2 441366
cellular
organisms;Eukaryota;Opisthokonta;Metazoa;Eumetazoa;Bilateria;Deuterostomia;Chordata;Craniata;Vertebrata;Gnathostomata;Teleostomi;Euteleostomi;Actinopterygii;Actinopteri;Neopterygii;Teleostei;Osteoglossocephalai;Clupeocephala;Euteleosteomorpha;Neoteleostei;Eurypterygia;Ctenosquamata;Acanthomorphata;Euacanthomorphacea;Percomorphaceae;Ovalentaria;Blenniimorphae;Blenniiformes;Gobiesocoidei;Gobiesocidae;Gobiesocinae;Gouania;Gouania willdenowi
UdeM00000044477 441366 132.9 UdeM00000044477 XP_028326620.1 40.6
197 107 5 1 194 48 237 4.50E-28 132.9 441366
cellular
organisms;Eukaryota;Opisthokonta;Metazoa;Eumetazoa;Bilateria;Deuterostomia;Chordata;Craniata;Vertebrata;Gnathostomata;Teleostomi;Euteleostomi;Actinopterygii;Actinopteri;Neopterygii;Teleostei;Osteoglossocephalai;Clupeocephala;Euteleosteomorpha;Neoteleostei;Eurypterygia;Ctenosquamata;Acanthomorphata;Euacanthomorphacea;Percomorphaceae;Ovalentaria;Blenniimorphae;Blenniiformes;Gobiesocoidei;Gobiesocidae;Gobiesocinae;Gouania;Gouania willdenowi
UdeM00000044478 6573 235.3 UdeM00000044478 XP_021355947.1 52.4 267
104 7 4 258 3 258 9.00E-59 235.3 6573 cellular
organisms;Eukaryota;Opisthokonta;Metazoa;Eumetazoa;Bilateria;Protostomia;Spiralia;Lophotrochozoa;Mollusca;Bivalvia;Autobranchia;Pteriomorphia;Pectinida;Pectinoidea;Pectinidae;Mizuhopecten;Mizuhopecten yessoensis
UdeM00000029713 6565 80.9 UdeM00000029713 XP_022291345.1 59.6 52
21 0 613 664 555 606 6.90E-12 80.9 6565 cellular
organisms;Eukaryota;Opisthokonta;Metazoa;Eumetazoa;Bilateria;Protostomia;Spiralia;Lophotrochozoa;Mollusca;Bivalvia;Autobranchia;Pteriomorphia;Ostreida;Ostreoidea;Ostreidae;Crassostrea;Crassostrea virginica
UdeM00000029714 6579 72.8 UdeM00000029714 XP_033742581.1 38.7 93
54 1 10 99 95 187 3.10E-10 72.8 6579 cellular
organisms;Eukaryota;Opisthokonta;Metazoa;Eumetazoa;Bilateria;Protostomia;Spiralia;Lophotrochozoa;Mollusca;Bivalvia;Autobranchia;Pteriomorphia;Pectinida;Pectinoidea;Pectinidae;Pecten;Pecten maximus
UdeM00000029715 6565 98.2 UdeM00000029715 XP_022302274.1 37.9 145
54 2 154 263 159 302 1.60E-17 98.2 6565 cellular
organisms;Eukaryota;Opisthokonta;Metazoa;Eumetazoa;Bilateria;Protostomia;Spiralia;Lophotrochozoa;Mollusca;Bivalvia;Autobranchia;Pteriomorphia;Ostreida;Ostreoidea;Ostreidae;Crassostrea;Crassostrea virginica

UdeM00000029716	6596	116.3	UdeM00000029716	XP_045199965.1	42.3	137
	77	1	2223	2357	1245	1381	5.20E-22	116.3	6596	cellular
organisms;Eukaryota;Opisthokonta;Metazoa;Eumetazoa;Bilateria;Protostomia;
Spiralia;Lophotrochozoa;Mollusca;Bivalvia;Autobranchia;Heteroconchia;Euhe
terodonta;Imparidentia;Venerida;Veneroidea;Veneridae;Mercenaria;Mercenari
a mercenaria
UdeM00000029718	6579	127.9	UdeM00000029718	XP_033736443.1	64.1	92
	33	0	1	92	194	285	7.30E-27	127.9	6579	cellular
organisms;Eukaryota;Opisthokonta;Metazoa;Eumetazoa;Bilateria;Protostomia;
Spiralia;Lophotrochozoa;Mollusca;Bivalvia;Autobranchia;Pteriomorphia;Pect
inida;Pectinoidea;Pectinidae;Pecten;Pecten maximus
UdeM00000029719	6596	254.2	UdeM00000029719	XP_045197663.1	31.9	448
	189	6	107	547	1149	1487	8.30E-64	254.2	6596	cellular
organisms;Eukaryota;Opisthokonta;Metazoa;Eumetazoa;Bilateria;Protostomia;
Spiralia;Lophotrochozoa;Mollusca;Bivalvia;Autobranchia;Heteroconchia;Euhe
terodonta;Imparidentia;Venerida;Veneroidea;Veneridae;Mercenaria;Mercenari
a mercenaria
UdeM00000029720	6573	64.7	UdeM00000029720	XP_021362785.1	40.3	77
	42	2	293	366	1146	1221	2.80E-07	64.7	6573	cellular
organisms;Eukaryota;Opisthokonta;Metazoa;Eumetazoa;Bilateria;Protostomia;
Spiralia;Lophotrochozoa;Mollusca;Bivalvia;Autobranchia;Pteriomorphia;Pect
inida;Pectinoidea;Pectinidae;Mizuhopecten;Mizuhopecten yessoensis
UdeM00000029722	6596	371.3	UdeM00000029722	XP_045197606.1	34.6	624
	300	12	411	1004	1835	2380	3.80E-99	371.3	6596	cellular
organisms;Eukaryota;Opisthokonta;Metazoa;Eumetazoa;Bilateria;Protostomia;
Spiralia;Lophotrochozoa;Mollusca;Bivalvia;Autobranchia;Heteroconchia;Euhe
terodonta;Imparidentia;Venerida;Veneroidea;Veneridae;Mercenaria;Mercenari
a mercenaria
UdeM00000029721	6596	371.3	UdeM00000029721	XP_045197606.1	34.6	624
	300	12	589	1182	1835	2380	4.50E-99	371.3	6596	cellular
organisms;Eukaryota;Opisthokonta;Metazoa;Eumetazoa;Bilateria;Protostomia;
Spiralia;Lophotrochozoa;Mollusca;Bivalvia;Autobranchia;Heteroconchia;Euhe
terodonta;Imparidentia;Venerida;Veneroidea;Veneridae;Mercenaria;Mercenari
a mercenaria
UdeM00000029724	6596	359.4	UdeM00000029724	XP_045197598.1	37.2	521
	227	7	585	1065	1524	1984	1.60E-95	359.4	6596	cellular
organisms;Eukaryota;Opisthokonta;Metazoa;Eumetazoa;Bilateria;Protostomia;
Spiralia;Lophotrochozoa;Mollusca;Bivalvia;Autobranchia;Heteroconchia;Euhe
terodonta;Imparidentia;Venerida;Veneroidea;Veneridae;Mercenaria;Mercenari
a mercenaria
UdeM00000029728	6596	360.9	UdeM00000029728	XP_045197598.1	37.5	526
	225	6	525	1011	1524	1984	5.20E-96	360.9	6596	cellular
organisms;Eukaryota;Opisthokonta;Metazoa;Eumetazoa;Bilateria;Protostomia;
Spiralia;Lophotrochozoa;Mollusca;Bivalvia;Autobranchia;Heteroconchia;Euhe
terodonta;Imparidentia;Venerida;Veneroidea;Veneridae;Mercenaria;Mercenari
a mercenaria
UdeM00000029730	6579	207.6	UdeM00000029730	XP_033741736.1	53.6	196
	89	1	1	194	671	866	1.50E-50	207.6	6579	cellular
organisms;Eukaryota;Opisthokonta;Metazoa;Eumetazoa;Bilateria;Protostomia;
Spiralia;Lophotrochozoa;Mollusca;Bivalvia;Autobranchia;Pteriomorphia;Pect
inida;Pectinoidea;Pectinidae;Pecten;Pecten maximus
UdeM00000029733	1415580	80.9	UdeM00000029733	XP_030066010.1	27.2
	246	133	8	9	217	247	483	2.30E-12	80.9	1415580
	cellular

organisms;Eukaryota;Opisthokonta;Metazoa;Eumetazoa;Bilateria;Deuterostomia;Chordata;Craniata;Vertebrata;Gnathostomata;Teleostomi;Euteleostomi;Sarcopterygii;Dipnotetrapodomorpha;Tetrapoda;Amphibia;Gymnophiona;Siphonopidae;Microcaecilia;Microcaecilia unicolor
UdeM00000029734 6596 139.8 UdeM00000029734 XP_045197598.1 36.1 219 97 2 63 243 1773 1986 4.60E-30 139.8 6596 cellular organisms;Eukaryota;Opisthokonta;Metazoa;Eumetazoa;Bilateria;Protostomia;Spiralia;Lophotrochozoa;Mollusca;Bivalvia;Autobranchia;Heteroconchia;Euheterodonta;Imparidentia;Venerida;Veneroidea;Veneridae;Mercenaria;Mercenaria mercenaria
UdeM00000029735 6596 215.3 UdeM00000029735 XP_045197621.1 35.1 345 160 8 175 516 61 344 1.80E-52 215.3 6596 cellular organisms;Eukaryota;Opisthokonta;Metazoa;Eumetazoa;Bilateria;Protostomia;Spiralia;Lophotrochozoa;Mollusca;Bivalvia;Autobranchia;Heteroconchia;Euheterodonta;Imparidentia;Venerida;Veneroidea;Veneridae;Mercenaria;Mercenaria mercenaria
UdeM00000029739 6596 355.1 UdeM00000029739 XP_045197606.1 37.5 573 239 8 660 1188 1883 2380 3.40E-94 355.1 6596 cellular organisms;Eukaryota;Opisthokonta;Metazoa;Eumetazoa;Bilateria;Protostomia;Spiralia;Lophotrochozoa;Mollusca;Bivalvia;Autobranchia;Heteroconchia;Euheterodonta;Imparidentia;Venerida;Veneroidea;Veneridae;Mercenaria;Mercenaria mercenaria
UdeM00000029741 6596 66.6 UdeM00000029741 XP_045195775.1 32.7 110 69 3 19 126 190 296 2.60E-08 66.6 6596 cellular organisms;Eukaryota;Opisthokonta;Metazoa;Eumetazoa;Bilateria;Protostomia;Spiralia;Lophotrochozoa;Mollusca;Bivalvia;Autobranchia;Heteroconchia;Euheterodonta;Imparidentia;Venerida;Veneroidea;Veneridae;Mercenaria;Mercenaria mercenaria
UdeM00000029742 6596 351.3 UdeM00000029742 XP_045197598.1 35.5 547 235 5 860 1368 1520 1986 5.60E-93 351.3 6596 cellular organisms;Eukaryota;Opisthokonta;Metazoa;Eumetazoa;Bilateria;Protostomia;Spiralia;Lophotrochozoa;Mollusca;Bivalvia;Autobranchia;Heteroconchia;Euheterodonta;Imparidentia;Venerida;Veneroidea;Veneridae;Mercenaria;Mercenaria mercenaria
UdeM00000029743 10224 68.9 UdeM00000029743 XP_006820186.1 50.8 59 29 0 29 87 175 233 3.60E-09 68.9 10224 cellular organisms;Eukaryota;Opisthokonta;Metazoa;Eumetazoa;Bilateria;Deuterostomia;Hemichordata;Enteropneusta;Harrimaniidae;Saccoglossus;Saccoglossus kowalevskii
UdeM00000029744 151771 132.1 UdeM00000029744 XP_028415423.1 30.4 369 210 11 4 334 7 366 1.40E-27 132.1 151771 cellular organisms;Eukaryota;Opisthokonta;Metazoa;Eumetazoa;Cnidaria;Anthozoa;Octocorallia;Alcyonacea;Alcyoniina;Nephtheidae;Dendronephthya;Dendronephthya gigantea
UdeM00000029745 6596 164.5 UdeM00000029745 XP_045197621.1 30.4 335 199 6 564 892 7 313 7.40E-37 164.5 6596 cellular organisms;Eukaryota;Opisthokonta;Metazoa;Eumetazoa;Bilateria;Protostomia;Spiralia;Lophotrochozoa;Mollusca;Bivalvia;Autobranchia;Heteroconchia;Euheterodonta;Imparidentia;Venerida;Veneroidea;Veneridae;Mercenaria;Mercenaria mercenaria
UdeM00000029747 6596 357.1 UdeM00000029747 XP_045197598.1 36.8 519 231 6 596 1075 1524 1984 8.00E-95 357.1 6596 cellular organisms;Eukaryota;Opisthokonta;Metazoa;Eumetazoa;Bilateria;Protostomia;

Spiralia;Lophotrochozoa;Mollusca;Bivalvia;Autobranchia;Heteroconchia;Euheterodonta;Imparidentia;Venerida;Veneroidea;Veneridae;Mercenaria;Mercenaria mercenaria
UdeM00000029750 27794 87.4 UdeM00000029750 XP_043351520.1 30.6 206 118 7 13 211 150 337 2.40E-14 87.4 27794 cellular organisms;Eukaryota;Opisthokonta;Metazoa;Eumetazoa;Bilateria;Deuterostomia;Chordata;Craniata;Vertebrata;Gnathostomata;Teleostomi;Euteleostomi;Sarcopterygii;Dipnotetrapodomorpha;Tetrapoda;Amniota;Sauropsida;Sauria;Archelosauria;Testudinata;Testudines;Cryptodira;Durocryptodira;Americhelydia;Chelonioidea;Dermochelyidae;Dermochelys;Dermochelys coriacea
UdeM00000029751 6596 352.4 UdeM00000029751 XP_045197598.1 36.1 548 230 6 1166 1674 1520 1986 3.10E-93 352.4 6596 cellular organisms;Eukaryota;Opisthokonta;Metazoa;Eumetazoa;Bilateria;Protostomia;Spiralia;Lophotrochozoa;Mollusca;Bivalvia;Autobranchia;Heteroconchia;Euheterodonta;Imparidentia;Venerida;Veneroidea;Veneridae;Mercenaria;Mercenaria mercenaria
UdeM00000029752 6565 90.5 UdeM00000029752 XP_022318775.1 46.4 110 55 3 1 107 1 109 1.70E-15 90.5 6565 cellular organisms;Eukaryota;Opisthokonta;Metazoa;Eumetazoa;Bilateria;Protostomia;Spiralia;Lophotrochozoa;Mollusca;Bivalvia;Autobranchia;Pteriomorphia;Ostreida;Ostreoidea;Ostreidae;Crassostrea;Crassostrea virginica
UdeM00000029753 37621 253.4 UdeM00000029753 XP_014673981.1 48.9 278 124 7 47 308 37 312 3.70E-64 253.4 37621 cellular organisms;Eukaryota;Opisthokonta;Metazoa;Eumetazoa;Bilateria;Protostomia;Ecdysozoa;Scalidophora;Priapulida;Priapulimorpha;Priapulimorphida;Priapulidae;Priapulus;Priapulus caudatus
UdeM00000029755 6596 167.2 UdeM00000029755 XP_045197621.1 34.5 255 138 4 454 705 84 312 9.80E-38 167.2 6596 cellular organisms;Eukaryota;Opisthokonta;Metazoa;Eumetazoa;Bilateria;Protostomia;Spiralia;Lophotrochozoa;Mollusca;Bivalvia;Autobranchia;Heteroconchia;Euheterodonta;Imparidentia;Venerida;Veneroidea;Veneridae;Mercenaria;Mercenaria mercenaria
UdeM00000029756 50429 73.2 UdeM00000029756 XP_022790680.1 46.2 80 37 3 3 80 40 115 4.40E-10 73.2 50429 cellular organisms;Eukaryota;Opisthokonta;Metazoa;Eumetazoa;Cnidaria;Anthozoa;Hexacorallia;Scleractinia;Astrocoeniina;Pocilloporidae;Stylophora;Stylophora pistillata
UdeM00000029757 2652724 119 UdeM00000029757 XP_028515960.1 30.9 262 89 5 3 173 4 264 6.20E-24 119 2652724 cellular organisms;Eukaryota;Opisthokonta;Metazoa;Eumetazoa;Cnidaria;Anthozoa;Hexacorallia;Actiniaria;Aiptasiidae;Exaiptasia;Exaiptasia diaphana
UdeM00000029758 6596 365.9 UdeM00000029758 XP_045197598.1 37.3 531 228 7 676 1167 1520 1984 1.90E-97 365.9 6596 cellular organisms;Eukaryota;Opisthokonta;Metazoa;Eumetazoa;Bilateria;Protostomia;Spiralia;Lophotrochozoa;Mollusca;Bivalvia;Autobranchia;Heteroconchia;Euheterodonta;Imparidentia;Venerida;Veneroidea;Veneridae;Mercenaria;Mercenaria mercenaria
UdeM00000029760 1234273 78.6 UdeM00000029760 XP_027000537.1 29.6 135 91 2 18 152 70 200 9.10E-12 78.6 1234273 cellular organisms;Eukaryota;Opisthokonta;Metazoa;Eumetazoa;Bilateria;Deuterostomia;Chordata;Craniata;Vertebrata;Gnathostomata;Teleostomi;Euteleostomi;Actinopterygii;Actinopteri;Neopterygii;Teleostei;Osteoglossocephalai;Clupeoce

phala;Otomorpha;Ostariophysi;Otophysi;Characiphysae;Siluriformes;Siluroidei;Bagridae;Tachysurus;Tachysurus fulvidraco
UdeM00000029759 1234273 78.6 UdeM00000029759 XP_027000537.1 29.6 135 91 2 18 152 70 200 9.80E-12 78.6 1234273 cellular organisms;Eukaryota;Opisthokonta;Metazoa;Eumetazoa;Bilateria;Deuterostomia;Chordata;Craniata;Vertebrata;Gnathostomata;Teleostomi;Euteleostomi;Actinopterygii;Actinopteri;Neopterygii;Teleostei;Osteoglossocephalai;Clupeocephala;Otomorpha;Ostariophysi;Otophysi;Characiphysae;Siluriformes;Siluroidei;Bagridae;Tachysurus;Tachysurus fulvidraco
UdeM00000029762 6596 207.6 UdeM00000029762 XP_045196404.1 28.4 633 370 18 11 591 188 789 4.30E-50 207.6 6596 cellular organisms;Eukaryota;Opisthokonta;Metazoa;Eumetazoa;Bilateria;Protostomia;Spiralia;Lophotrochozoa;Mollusca;Bivalvia;Autobranchia;Heteroconchia;Euheterodonta;Imparidentia;Venerida;Veneroidea;Veneridae;Mercenaria;Mercenaria mercenaria
UdeM00000029763 6596 1331.2 UdeM00000029763 XP_045178231.1 51.8 1428 576 9 10 1420 217 1548 0.00E+00 1331.2 6596 cellular organisms;Eukaryota;Opisthokonta;Metazoa;Eumetazoa;Bilateria;Protostomia;Spiralia;Lophotrochozoa;Mollusca;Bivalvia;Autobranchia;Heteroconchia;Euheterodonta;Imparidentia;Venerida;Veneroidea;Veneridae;Mercenaria;Mercenaria mercenaria
UdeM00000029764 6596 1428.3 UdeM00000029764 XP_045178231.1 50.4 1572 671 12 51 1611 74 1548 0.00E+00 1428.3 6596 cellular organisms;Eukaryota;Opisthokonta;Metazoa;Eumetazoa;Bilateria;Protostomia;Spiralia;Lophotrochozoa;Mollusca;Bivalvia;Autobranchia;Heteroconchia;Euheterodonta;Imparidentia;Venerida;Veneroidea;Veneridae;Mercenaria;Mercenaria mercenaria
UdeM00000029765 6596 379.4 UdeM00000029765 XP_045195620.1 58.5 301 124 1 29 329 40 339 4.60E-102 379.4 6596 cellular organisms;Eukaryota;Opisthokonta;Metazoa;Eumetazoa;Bilateria;Protostomia;Spiralia;Lophotrochozoa;Mollusca;Bivalvia;Autobranchia;Heteroconchia;Euheterodonta;Imparidentia;Venerida;Veneroidea;Veneridae;Mercenaria;Mercenaria mercenaria
UdeM00000029766 1735272 503.4 UdeM00000029766 XP_041374187.1 53.9 488 197 9 1 480 1 468 3.10E-139 503.4 1735272 cellular organisms;Eukaryota;Opisthokonta;Metazoa;Eumetazoa;Bilateria;Protostomia;Spiralia;Lophotrochozoa;Mollusca;Gastropoda;Neomphalina;Neomphaloidea;Peltospiridae;Gigantopelta;Gigantopelta aegis
UdeM00000029767 6579 265.8 UdeM00000029767 XP_033764274.1 49.2 236 115 2 1 233 361 594 5.30E-68 265.8 6579 cellular organisms;Eukaryota;Opisthokonta;Metazoa;Eumetazoa;Bilateria;Protostomia;Spiralia;Lophotrochozoa;Mollusca;Bivalvia;Autobranchia;Pteriomorphia;Pectinida;Pectinoidea;Pectinidae;Pecten;Pecten maximus
UdeM00000029768 6596 239.6 UdeM00000029768 XP_045173143.1 54.5 224 94 4 1 220 89 308 3.80E-60 239.6 6596 cellular organisms;Eukaryota;Opisthokonta;Metazoa;Eumetazoa;Bilateria;Protostomia;Spiralia;Lophotrochozoa;Mollusca;Bivalvia;Autobranchia;Heteroconchia;Euheterodonta;Imparidentia;Venerida;Veneroidea;Veneridae;Mercenaria;Mercenaria mercenaria

UdeM00000029769 6596 273.5 UdeM00000029769 XP_045173143.1 53.6 252
109 4 3 250 61 308 2.70E-70 273.5 6596 cellular
organisms;Eukaryota;Opisthokonta;Metazoa;Eumetazoa;Bilateria;Protostomia;
Spiralia;Lophotrochozoa;Mollusca;Bivalvia;Autobranchia;Heteroconchia;Euhe
terodonta;Imparidentia;Venerida;Veneroidea;Veneridae;Mercenaria;Mercenari
a mercenaria
UdeM00000029770 6596 95.9 UdeM00000029770 XP_045179943.1 29.9 174
107 2 32 190 10 183 8.40E-17 95.9 6596 cellular
organisms;Eukaryota;Opisthokonta;Metazoa;Eumetazoa;Bilateria;Protostomia;
Spiralia;Lophotrochozoa;Mollusca;Bivalvia;Autobranchia;Heteroconchia;Euhe
terodonta;Imparidentia;Venerida;Veneroidea;Veneridae;Mercenaria;Mercenari
a mercenaria
UdeM00000029771 6565 555.8 UdeM00000029771 XP_022287588.1 63.3 392
144 0 6 397 2 393 4.40E-155 555.8 6565 cellular
organisms;Eukaryota;Opisthokonta;Metazoa;Eumetazoa;Bilateria;Protostomia;
Spiralia;Lophotrochozoa;Mollusca;Bivalvia;Autobranchia;Pteriomorphia;Ostr
eida;Ostreoidea;Ostreidae;Crassostrea;Crassostrea virginica
UdeM00000029772 6579 968.8 UdeM00000029772 XP_033741161.1 67.4 733
214 4 1 725 1 716 3.90E-279 968.8 6579 cellular
organisms;Eukaryota;Opisthokonta;Metazoa;Eumetazoa;Bilateria;Protostomia;
Spiralia;Lophotrochozoa;Mollusca;Bivalvia;Autobranchia;Pteriomorphia;Pect
inida;Pectinoidea;Pectinidae;Pecten;Pecten maximus
UdeM00000029773 6596 265 UdeM00000029773 XP_045197939.1 66.7 186
57 1 9 189 33 218 7.80E-68 265 6596 cellular
organisms;Eukaryota;Opisthokonta;Metazoa;Eumetazoa;Bilateria;Protostomia;
Spiralia;Lophotrochozoa;Mollusca;Bivalvia;Autobranchia;Heteroconchia;Euhe
terodonta;Imparidentia;Venerida;Veneroidea;Veneridae;Mercenaria;Mercenari
a mercenaria
UdeM00000029774 7604 303.5 UdeM00000029774 XP_033633146.1 41 390
212 3 262 633 29 418 6.40E-79 303.5 7604 cellular
organisms;Eukaryota;Opisthokonta;Metazoa;Eumetazoa;Bilateria;Deuterostomi
a;Echinodermata;Eleutherozoa;Asterozoa;Asteroidea;Forcipulatacea;Forcipul
atida;Asteriidae;Asterias;Asterias rubens
UdeM00000029775 6596 451.4 UdeM00000029775 XP_045197939.1 67.4 322
102 2 46 366 220 539 1.10E-123 451.4 6596 cellular
organisms;Eukaryota;Opisthokonta;Metazoa;Eumetazoa;Bilateria;Protostomia;
Spiralia;Lophotrochozoa;Mollusca;Bivalvia;Autobranchia;Heteroconchia;Euhe
terodonta;Imparidentia;Venerida;Veneroidea;Veneridae;Mercenaria;Mercenari
a mercenaria
UdeM00000029776 29159 163.3 UdeM00000029776 XP_034319761.1 39.1 207
116 4 595 800 1556 1753 1.70E-36 163.3 29159 cellular
organisms;Eukaryota;Opisthokonta;Metazoa;Eumetazoa;Bilateria;Protostomia;
Spiralia;Lophotrochozoa;Mollusca;Bivalvia;Autobranchia;Pteriomorphia;Ostr
eida;Ostreoidea;Ostreidae;Crassostrea;Crassostrea gigas
UdeM00000029777 6565 72.8 UdeM00000029777 XP_022294141.1 48.5 68
34 1 75 142 27 93 4.00E-10 72.8 6565 cellular
organisms;Eukaryota;Opisthokonta;Metazoa;Eumetazoa;Bilateria;Protostomia;
Spiralia;Lophotrochozoa;Mollusca;Bivalvia;Autobranchia;Pteriomorphia;Ostr
eida;Ostreoidea;Ostreidae;Crassostrea;Crassostrea virginica
UdeM00000029778 6596 347.4 UdeM00000029778 XP_045196451.1 47.1 448
196 14 8 433 91 519 2.60E-92 347.4 6596 cellular
organisms;Eukaryota;Opisthokonta;Metazoa;Eumetazoa;Bilateria;Protostomia;
Spiralia;Lophotrochozoa;Mollusca;Bivalvia;Autobranchia;Heteroconchia;Euhe

terodonta;Imparidentia;Venerida;Veneroidea;Veneridae;Mercenaria;Mercenaria mercenaria
UdeM00000029779 6596 793.5 UdeM00000029779 XP_045197470.1 52.6 734 345 3 1 732 1 733 2.30E-226 793.5 6596 cellular organisms;Eukaryota;Opisthokonta;Metazoa;Eumetazoa;Bilateria;Protostomia;Spiralia;Lophotrochozoa;Mollusca;Bivalvia;Autobranchia;Heteroconchia;Euheterodonta;Imparidentia;Venerida;Veneroidea;Veneridae;Mercenaria;Mercenaria mercenaria
UdeM00000029780 1735272 337.8 UdeM00000029780 XP_041368620.1 51.1 331 145 3 8 338 7 320 1.60E-89 337.8 1735272 cellular organisms;Eukaryota;Opisthokonta;Metazoa;Eumetazoa;Bilateria;Protostomia;Spiralia;Lophotrochozoa;Mollusca;Gastropoda;Neomphalina;Neomphaloidea;Peltospiridae;Gigantopelta;Gigantopelta aegis
UdeM00000029781 6596 447.2 UdeM00000029781 XP_045178889.1 28.2 1350 770 50 73 1286 126 1412 7.10E-122 447.2 6596 cellular organisms;Eukaryota;Opisthokonta;Metazoa;Eumetazoa;Bilateria;Protostomia;Spiralia;Lophotrochozoa;Mollusca;Bivalvia;Autobranchia;Heteroconchia;Euheterodonta;Imparidentia;Venerida;Veneroidea;Veneridae;Mercenaria;Mercenaria mercenaria
UdeM00000029782 6596 1149.8 UdeM00000029782 XP_045197475.1 55.8 1014 430 9 7 1013 30 1032 0.00E+00 1149.8 6596 cellular organisms;Eukaryota;Opisthokonta;Metazoa;Eumetazoa;Bilateria;Protostomia;Spiralia;Lophotrochozoa;Mollusca;Bivalvia;Autobranchia;Heteroconchia;Euheterodonta;Imparidentia;Venerida;Veneroidea;Veneridae;Mercenaria;Mercenaria mercenaria
UdeM00000029783 6596 927.5 UdeM00000029783 XP_045159593.1 67.1 689 220 3 1 689 1 682 9.50E-267 927.5 6596 cellular organisms;Eukaryota;Opisthokonta;Metazoa;Eumetazoa;Bilateria;Protostomia;Spiralia;Lophotrochozoa;Mollusca;Bivalvia;Autobranchia;Heteroconchia;Euheterodonta;Imparidentia;Venerida;Veneroidea;Veneridae;Mercenaria;Mercenaria mercenaria
UdeM00000029784 6596 343.6 UdeM00000029784 XP_045195330.1 38.9 447 243 6 4 424 50 492 3.60E-91 343.6 6596 cellular organisms;Eukaryota;Opisthokonta;Metazoa;Eumetazoa;Bilateria;Protostomia;Spiralia;Lophotrochozoa;Mollusca;Bivalvia;Autobranchia;Heteroconchia;Euheterodonta;Imparidentia;Venerida;Veneroidea;Veneridae;Mercenaria;Mercenaria mercenaria
UdeM00000029786 6579 379 UdeM00000029786 XP_033749291.1 37.1 626 380 7 11 626 823 1444 1.20E-101 379 6579 cellular organisms;Eukaryota;Opisthokonta;Metazoa;Eumetazoa;Bilateria;Protostomia;Spiralia;Lophotrochozoa;Mollusca;Bivalvia;Autobranchia;Pteriomorphia;Pectinida;Pectinoidea;Pectinidae;Pecten;Pecten maximus
UdeM00000029787 222816 183.3 UdeM00000029787 XP_012285318.1 35.5 355 212 5 1 338 289 643 5.10E-43 183.3 222816 cellular organisms;Eukaryota;Opisthokonta;Metazoa;Eumetazoa;Bilateria;Protostomia;Ecdysozoa;Panarthropoda;Arthropoda;Mandibulata;Pancrustacea;Hexapoda;Insecta;Dicondylia;Pterygota;Neoptera;Endopterygota;Hymenoptera;Orussoidea;Orussidae;Orussus;Orussus abietinus
UdeM00000029788 6596 151 UdeM00000029788 XP_045216663.1 41.7 168 98 0 16 183 2 169 1.50E-33 151 6596 cellular organisms;Eukaryota;Opisthokonta;Metazoa;Eumetazoa;Bilateria;Protostomia;

Spiralia;Lophotrochozoa;Mollusca;Bivalvia;Autobranchia;Heteroconchia;Euheterodonta;Imparidentia;Venerida;Veneroidea;Veneridae;Mercenaria;Mercenaria mercenaria
UdeM00000029790 6565 313.9 UdeM00000029790 XP_022329040.1 36 519 319 6 11 520 14 528 4.00E-82 313.9 6565 cellular organisms;Eukaryota;Opisthokonta;Metazoa;Eumetazoa;Bilateria;Protostomia;Spiralia;Lophotrochozoa;Mollusca;Bivalvia;Autobranchia;Pteriomorphia;Ostreida;Ostreoidea;Ostreidae;Crassostrea;Crassostrea virginica
UdeM00000029792 202533 316.2 UdeM00000029792 XP_035214221.1 38.1 507 291 6 9 499 19 518 7.70E-83 316.2 202533 cellular organisms;Eukaryota;Opisthokonta;Metazoa;Eumetazoa;Bilateria;Protostomia;Ecdysozoa;Panarthropoda;Arthropoda;Chelicerata;Arachnida;Araneae;Araneomorphae;Entelegynae;Eresoidea;Eresidae;Stegodyphus;Stegodyphus dumicola
UdeM00000029794 136037 384.8 UdeM00000029794 XP_021919438.1 36.7 633 384 7 8 626 5 634 2.30E-103 384.8 136037 cellular organisms;Eukaryota;Opisthokonta;Metazoa;Eumetazoa;Bilateria;Protostomia;Ecdysozoa;Panarthropoda;Arthropoda;Mandibulata;Pancrustacea;Hexapoda;Insecta;Dicondylia;Pterygota;Neoptera;Polyneoptera;Dictyoptera;Blattodea;Blattoidea;Termitoidae;Termopsidae;Termopsinae;Termopsini;Zootermopsis;Zootermopsis nevadensis
UdeM00000029796 136037 392.1 UdeM00000029796 XP_021919438.1 36.8 633 383 7 8 626 5 634 1.30E-105 392.1 136037 cellular organisms;Eukaryota;Opisthokonta;Metazoa;Eumetazoa;Bilateria;Protostomia;Ecdysozoa;Panarthropoda;Arthropoda;Mandibulata;Pancrustacea;Hexapoda;Insecta;Dicondylia;Pterygota;Neoptera;Polyneoptera;Dictyoptera;Blattodea;Blattoidea;Termitoidae;Termopsidae;Termopsinae;Termopsini;Zootermopsis;Zootermopsis nevadensis
UdeM00000029797 8673 118.6 UdeM00000029797 XP_026565175.1 38.8 178 104 3 10 183 515 691 8.60E-24 118.6 8673 cellular organisms;Eukaryota;Opisthokonta;Metazoa;Eumetazoa;Bilateria;Deuterostomia;Chordata;Craniata;Vertebrata;Gnathostomata;Teleostomi;Euteleostomi;Sarcopterygii;Dipnotetrapodomorpha;Tetrapoda;Amniota;Sauropsida;Sauria;Lepidosauria;Squamata;Bifurcata;Unidentata;Episquamata;Toxicofera;Serpentes;Colubroidea;Elapidae;Acanthophiinae;Pseudonaja;Pseudonaja textilis
UdeM00000029798 202533 271.6 UdeM00000029798 XP_035214221.1 43.3 335 186 3 9 342 19 350 1.40E-69 271.6 202533 cellular organisms;Eukaryota;Opisthokonta;Metazoa;Eumetazoa;Bilateria;Protostomia;Ecdysozoa;Panarthropoda;Arthropoda;Chelicerata;Arachnida;Araneae;Araneomorphae;Entelegynae;Eresoidea;Eresidae;Stegodyphus;Stegodyphus dumicola
UdeM00000029799 6579 374.8 UdeM00000029799 XP_033749291.1 36.7 616 376 7 11 616 823 1434 2.10E-100 374.8 6579 cellular organisms;Eukaryota;Opisthokonta;Metazoa;Eumetazoa;Bilateria;Protostomia;Spiralia;Lophotrochozoa;Mollusca;Bivalvia;Autobranchia;Pteriomorphia;Pectinida;Pectinoidea;Pectinidae;Pecten;Pecten maximus
UdeM00000029800 136037 385.6 UdeM00000029800 XP_021919438.1 36.5 633 385 7 8 626 5 634 1.20E-103 385.6 136037 cellular organisms;Eukaryota;Opisthokonta;Metazoa;Eumetazoa;Bilateria;Protostomia;Ecdysozoa;Panarthropoda;Arthropoda;Mandibulata;Pancrustacea;Hexapoda;Insecta;Dicondylia;Pterygota;Neoptera;Polyneoptera;Dictyoptera;Blattodea;Blat

toidea;Termitoidae;Termopsidae;Termopsinae;Termopsini;Zootermopsis;Zootermopsis nevadensis
UdeM00000029801 136037 384 UdeM00000029801 XP_021919437.1 36.8 634 383 8 8 626 5 635 3.60E-103 384 136037 cellular organisms;Eukaryota;Opisthokonta;Metazoa;Eumetazoa;Bilateria;Protostomia;Ecdysozoa;Panarthropoda;Arthropoda;Mandibulata;Pancrustacea;Hexapoda;Insecta;Dicondylia;Pterygota;Neoptera;Polyneoptera;Dictyoptera;Blattodea;Blattoidea;Termitoidae;Termopsidae;Termopsinae;Termopsini;Zootermopsis;Zootermopsis nevadensis
UdeM00000029802 136037 383.6 UdeM00000029802 XP_021919438.1 36.3 633 386 7 8 626 5 634 4.70E-103 383.6 136037 cellular organisms;Eukaryota;Opisthokonta;Metazoa;Eumetazoa;Bilateria;Protostomia;Ecdysozoa;Panarthropoda;Arthropoda;Mandibulata;Pancrustacea;Hexapoda;Insecta;Dicondylia;Pterygota;Neoptera;Polyneoptera;Dictyoptera;Blattodea;Blattoidea;Termitoidae;Termopsidae;Termopsinae;Termopsini;Zootermopsis;Zootermopsis nevadensis
UdeM00000029804 136037 383.3 UdeM00000029804 XP_021919438.1 36.7 633 384 7 8 626 5 634 6.20E-103 383.3 136037 cellular organisms;Eukaryota;Opisthokonta;Metazoa;Eumetazoa;Bilateria;Protostomia;Ecdysozoa;Panarthropoda;Arthropoda;Mandibulata;Pancrustacea;Hexapoda;Insecta;Dicondylia;Pterygota;Neoptera;Polyneoptera;Dictyoptera;Blattodea;Blattoidea;Termitoidae;Termopsidae;Termopsinae;Termopsini;Zootermopsis;Zootermopsis nevadensis
UdeM00000029806 136037 391 UdeM00000029806 XP_021919438.1 36.7 633 384 7 8 626 5 634 3.00E-105 391 136037 cellular organisms;Eukaryota;Opisthokonta;Metazoa;Eumetazoa;Bilateria;Protostomia;Ecdysozoa;Panarthropoda;Arthropoda;Mandibulata;Pancrustacea;Hexapoda;Insecta;Dicondylia;Pterygota;Neoptera;Polyneoptera;Dictyoptera;Blattodea;Blattoidea;Termitoidae;Termopsidae;Termopsinae;Termopsini;Zootermopsis;Zootermopsis nevadensis
UdeM00000029807 6596 386.7 UdeM00000029807 XP_045216663.1 35.8 626 390 5 11 626 13 636 5.60E-104 386.7 6596 cellular organisms;Eukaryota;Opisthokonta;Metazoa;Eumetazoa;Bilateria;Protostomia;Spiralia;Lophotrochozoa;Mollusca;Bivalvia;Autobranchia;Heteroconchia;Euheterodonta;Imparidentia;Venerida;Veneroidea;Veneridae;Mercenaria;Mercenaria mercenaria
UdeM00000029808 136037 383.3 UdeM00000029808 XP_021919437.1 36.6 634 384 8 8 626 5 635 6.20E-103 383.3 136037 cellular organisms;Eukaryota;Opisthokonta;Metazoa;Eumetazoa;Bilateria;Protostomia;Ecdysozoa;Panarthropoda;Arthropoda;Mandibulata;Pancrustacea;Hexapoda;Insecta;Dicondylia;Pterygota;Neoptera;Polyneoptera;Dictyoptera;Blattodea;Blattoidea;Termitoidae;Termopsidae;Termopsinae;Termopsini;Zootermopsis;Zootermopsis nevadensis
UdeM00000029809 118141 204.9 UdeM00000029809 XP_036393933.1 32.7 364 242 3 1 363 127 488 1.90E-49 204.9 118141 cellular organisms;Eukaryota;Opisthokonta;Metazoa;Eumetazoa;Bilateria;Deuterostomia;Chordata;Craniata;Vertebrata;Gnathostomata;Teleostomi;Euteleostomi;Acti

nopterygii;Actinopteri;Neopterygii;Teleostei;Elopocephalai;Elopocephala;Elopomorpha;Elopiformes;Megalopidae;Megalops;Megalops cyprinoides
UdeM00000029810 6596 285 UdeM00000029810 XP_045195422.1 37.3 408 232 7 77 476 125 516 1.70E-73 285 6596 cellular organisms;Eukaryota;Opisthokonta;Metazoa;Eumetazoa;Bilateria;Protostomia;Spiralia;Lophotrochozoa;Mollusca;Bivalvia;Autobranchia;Heteroconchia;Euheterodonta;Imparidentia;Venerida;Veneroidea;Veneridae;Mercenaria;Mercenaria mercenaria
UdeM00000029811 6596 284.6 UdeM00000029811 XP_045195422.1 37 416 222 6 8 407 125 516 1.90E-73 284.6 6596 cellular organisms;Eukaryota;Opisthokonta;Metazoa;Eumetazoa;Bilateria;Protostomia;Spiralia;Lophotrochozoa;Mollusca;Bivalvia;Autobranchia;Heteroconchia;Euheterodonta;Imparidentia;Venerida;Veneroidea;Veneridae;Mercenaria;Mercenaria mercenaria
UdeM00000029812 400727 939.1 UdeM00000029812 XP_025112799.1 75.5 612 149 1 76 686 37 648 3.20E-270 939.1 400727 cellular organisms;Eukaryota;Opisthokonta;Metazoa;Eumetazoa;Bilateria;Protostomia;Spiralia;Lophotrochozoa;Mollusca;Gastropoda;Caenogastropoda;Architaenioglossa;Ampullarioidea;Ampullariidae;Pomacea;Pomacea canaliculata
UdeM00000029813 6596 1112.1 UdeM00000029813 XP_045196540.1 49.7 1400 612 37 12 1387 211 1542 0.00E+00 1112.1 6596 cellular organisms;Eukaryota;Opisthokonta;Metazoa;Eumetazoa;Bilateria;Protostomia;Spiralia;Lophotrochozoa;Mollusca;Bivalvia;Autobranchia;Heteroconchia;Euheterodonta;Imparidentia;Venerida;Veneroidea;Veneridae;Mercenaria;Mercenaria mercenaria
UdeM00000029814 6579 1375.9 UdeM00000029814 XP_033741190.1 60.8 1212 447 19 110 1310 578 1772 0.00E+00 1375.9 6579 cellular organisms;Eukaryota;Opisthokonta;Metazoa;Eumetazoa;Bilateria;Protostomia;Spiralia;Lophotrochozoa;Mollusca;Bivalvia;Autobranchia;Pteriomorphia;Pectinida;Pectinoidea;Pectinidae;Pecten;Pecten maximus
UdeM00000029815 6596 385.2 UdeM00000029815 XP_045196390.1 44.7 573 224 14 12 571 32 524 1.50E-103 385.2 6596 cellular organisms;Eukaryota;Opisthokonta;Metazoa;Eumetazoa;Bilateria;Protostomia;Spiralia;Lophotrochozoa;Mollusca;Bivalvia;Autobranchia;Heteroconchia;Euheterodonta;Imparidentia;Venerida;Veneroidea;Veneridae;Mercenaria;Mercenaria mercenaria
UdeM00000029816 6596 502.3 UdeM00000029816 XP_045198045.1 61.2 400 155 0 1 400 144 543 6.00E-139 502.3 6596 cellular organisms;Eukaryota;Opisthokonta;Metazoa;Eumetazoa;Bilateria;Protostomia;Spiralia;Lophotrochozoa;Mollusca;Bivalvia;Autobranchia;Heteroconchia;Euheterodonta;Imparidentia;Venerida;Veneroidea;Veneridae;Mercenaria;Mercenaria mercenaria
UdeM00000029817 6596 177.6 UdeM00000029817 XP_045198045.1 62.8 129 46 2 1 128 1 128 1.10E-41 177.6 6596 cellular organisms;Eukaryota;Opisthokonta;Metazoa;Eumetazoa;Bilateria;Protostomia;Spiralia;Lophotrochozoa;Mollusca;Bivalvia;Autobranchia;Heteroconchia;Euheterodonta;Imparidentia;Venerida;Veneroidea;Veneridae;Mercenaria;Mercenaria mercenaria
UdeM00000029818 6596 131 UdeM00000029818 XP_045198045.1 49.2 126 51 1 26 138 418 543 1.40E-27 131 6596 cellular organisms;Eukaryota;Opisthokonta;Metazoa;Eumetazoa;Bilateria;Protostomia;

Spiralia;Lophotrochozoa;Mollusca;Bivalvia;Autobranchia;Heteroconchia;Euheterodonta;Imparidentia;Venerida;Veneroidea;Veneridae;Mercenaria;Mercenaria mercenaria
UdeM00000029819 6573 91.3 UdeM00000029819 XP_021380018.1 59.4 69 28 0 1 69 173 241 5.40E-16 91.3 6573 cellular organisms;Eukaryota;Opisthokonta;Metazoa;Eumetazoa;Bilateria;Protostomia;Spiralia;Lophotrochozoa;Mollusca;Bivalvia;Autobranchia;Pteriomorphia;Pectinida;Pectinoidea;Pectinidae;Mizuhopecten;Mizuhopecten yessoensis
UdeM00000029820 6596 736.9 UdeM00000029820 XP_045198045.1 64.9 544 189 2 1 543 1 543 1.90E-209 736.9 6596 cellular organisms;Eukaryota;Opisthokonta;Metazoa;Eumetazoa;Bilateria;Protostomia;Spiralia;Lophotrochozoa;Mollusca;Bivalvia;Autobranchia;Heteroconchia;Euheterodonta;Imparidentia;Venerida;Veneroidea;Veneridae;Mercenaria;Mercenaria mercenaria
UdeM00000029821 6579 59.7 UdeM00000029821 XP_033761099.1 22.7 321 213 10 79 377 5 312 9.60E-06 59.7 6579 cellular organisms;Eukaryota;Opisthokonta;Metazoa;Eumetazoa;Bilateria;Protostomia;Spiralia;Lophotrochozoa;Mollusca;Bivalvia;Autobranchia;Pteriomorphia;Pectinida;Pectinoidea;Pectinidae;Pecten;Pecten maximus
UdeM00000029822 6596 715.7 UdeM00000029822 XP_045198045.1 63.8 544 192 3 1 540 1 543 4.60E-203 715.7 6596 cellular organisms;Eukaryota;Opisthokonta;Metazoa;Eumetazoa;Bilateria;Protostomia;Spiralia;Lophotrochozoa;Mollusca;Bivalvia;Autobranchia;Heteroconchia;Euheterodonta;Imparidentia;Venerida;Veneroidea;Veneridae;Mercenaria;Mercenaria mercenaria
UdeM00000029823 6579 540 UdeM00000029823 XP_033739920.1 49.7 537 239 5 1 507 1 536 3.30E-150 540 6579 cellular organisms;Eukaryota;Opisthokonta;Metazoa;Eumetazoa;Bilateria;Protostomia;Spiralia;Lophotrochozoa;Mollusca;Bivalvia;Autobranchia;Pteriomorphia;Pectinida;Pectinoidea;Pectinidae;Pecten;Pecten maximus
UdeM00000029824 6596 97.4 UdeM00000029824 XP_045198045.1 57.5 73 31 0 1 73 471 543 9.80E-18 97.4 6596 cellular organisms;Eukaryota;Opisthokonta;Metazoa;Eumetazoa;Bilateria;Protostomia;Spiralia;Lophotrochozoa;Mollusca;Bivalvia;Autobranchia;Heteroconchia;Euheterodonta;Imparidentia;Venerida;Veneroidea;Veneridae;Mercenaria;Mercenaria mercenaria
UdeM00000029825 6596 548.5 UdeM00000029825 XP_045198045.1 63.1 425 154 2 1 423 1 424 7.70E-153 548.5 6596 cellular organisms;Eukaryota;Opisthokonta;Metazoa;Eumetazoa;Bilateria;Protostomia;Spiralia;Lophotrochozoa;Mollusca;Bivalvia;Autobranchia;Heteroconchia;Euheterodonta;Imparidentia;Venerida;Veneroidea;Veneridae;Mercenaria;Mercenaria mercenaria
UdeM00000029826 29159 548.1 UdeM00000029826 XP_011428614.2 50 550 243 5 1 524 1 544 1.20E-152 548.1 29159 cellular organisms;Eukaryota;Opisthokonta;Metazoa;Eumetazoa;Bilateria;Protostomia;Spiralia;Lophotrochozoa;Mollusca;Bivalvia;Autobranchia;Pteriomorphia;Ostreida;Ostreoidea;Ostreidae;Crassostrea;Crassostrea gigas
UdeM00000029827 6596 274.2 UdeM00000029827 XP_045196350.1 64.4 208 71 3 52 259 53 257 1.70E-70 274.2 6596 cellular organisms;Eukaryota;Opisthokonta;Metazoa;Eumetazoa;Bilateria;Protostomia;Spiralia;Lophotrochozoa;Mollusca;Bivalvia;Autobranchia;Heteroconchia;Euheterodonta;Imparidentia;Venerida;Veneroidea;Veneridae;Mercenaria;Mercenaria mercenaria

UdeM00000029828 6573 1037.7 UdeM00000029828 XP_021354033.1 47.5
1188 558 17 1 1172 1 1138 1.10E-299 1037.7 6573
cellular
organisms;Eukaryota;Opisthokonta;Metazoa;Eumetazoa;Bilateria;Protostomia;
Spiralia;Lophotrochozoa;Mollusca;Bivalvia;Autobranchia;Pteriomorphia;Pect
inida;Pectinoidea;Pectinidae;Mizuhopecten;Mizuhopecten yessoensis
UdeM00000029829 6596 272.7 UdeM00000029829 XP_045196345.1 59.8 219
85 1 1 219 34 249 4.10E-70 272.7 6596 cellular
organisms;Eukaryota;Opisthokonta;Metazoa;Eumetazoa;Bilateria;Protostomia;
Spiralia;Lophotrochozoa;Mollusca;Bivalvia;Autobranchia;Heteroconchia;Euhe
terodonta;Imparidentia;Venerida;Veneroidea;Veneridae;Mercenaria;Mercenari
a mercenaria
UdeM00000029830 400727 332.8 UdeM00000029830 XP_025097668.1 86.7
180 24 0 1 180 1 180 2.70E-88 332.8 400727
cellular
organisms;Eukaryota;Opisthokonta;Metazoa;Eumetazoa;Bilateria;Protostomia;
Spiralia;Lophotrochozoa;Mollusca;Gastropoda;Caenogastropoda;Architaenioglossa;Ampullarioidea;Ampullariidae;Pomacea;Pomacea canaliculata
UdeM00000029831 6573 624.8 UdeM00000029831 XP_021359228.1 33.9 1108
698 15 134 1218 24 1120 2.30E-175 624.8 6573 cellular
organisms;Eukaryota;Opisthokonta;Metazoa;Eumetazoa;Bilateria;Protostomia;
Spiralia;Lophotrochozoa;Mollusca;Bivalvia;Autobranchia;Pteriomorphia;Pect
inida;Pectinoidea;Pectinidae;Mizuhopecten;Mizuhopecten yessoensis
UdeM00000029832 37621 131.7 UdeM00000029832 XP_014676916.1 29.7 350
174 10 34 327 7 340 2.20E-27 131.7 37621 cellular
organisms;Eukaryota;Opisthokonta;Metazoa;Eumetazoa;Bilateria;Protostomia;
Ecdysozoa;Scalidophora;Priapulida;Priapulimorpha;Priapulimorphida;Priapul
idae;Priapulus;Priapulus caudatus
UdeM00000029833 1735272 243 UdeM00000029833 XP_041353997.1 83.2
137 23 0 18 154 44 180 2.40E-61 243 1735272
cellular
organisms;Eukaryota;Opisthokonta;Metazoa;Eumetazoa;Bilateria;Protostomia;
Spiralia;Lophotrochozoa;Mollusca;Gastropoda;Neomphalina;Neomphaloidea;Pel
tospiridae;Gigantopelta;Gigantopelta aegis
UdeM00000029834 6573 614.8 UdeM00000029834 XP_021359228.1 33.1 1111
703 15 122 1206 24 1120 2.40E-172 614.8 6573 cellular
organisms;Eukaryota;Opisthokonta;Metazoa;Eumetazoa;Bilateria;Protostomia;
Spiralia;Lophotrochozoa;Mollusca;Bivalvia;Autobranchia;Pteriomorphia;Pect
inida;Pectinoidea;Pectinidae;Mizuhopecten;Mizuhopecten yessoensis
UdeM00000029835 6573 501.9 UdeM00000029835 XP_021359228.1 30.7 1218
770 24 118 1317 26 1187 2.50E-138 501.9 6573 cellular
organisms;Eukaryota;Opisthokonta;Metazoa;Eumetazoa;Bilateria;Protostomia;
Spiralia;Lophotrochozoa;Mollusca;Bivalvia;Autobranchia;Pteriomorphia;Pect
inida;Pectinoidea;Pectinidae;Mizuhopecten;Mizuhopecten yessoensis
UdeM00000029836 6565 293.1 UdeM00000029836 XP_022291971.1 63.6 236
75 2 2 227 3 237 3.10E-76 293.1 6565 cellular
organisms;Eukaryota;Opisthokonta;Metazoa;Eumetazoa;Bilateria;Protostomia;
Spiralia;Lophotrochozoa;Mollusca;Bivalvia;Autobranchia;Pteriomorphia;Ostr
eida;Ostreoidea;Ostreidae;Crassostrea;Crassostrea virginica
UdeM00000029837 6596 601.3 UdeM00000029837 XP_045197593.1 51.9 626
262 10 22 621 287 899 1.40E-168 601.3 6596 cellular
organisms;Eukaryota;Opisthokonta;Metazoa;Eumetazoa;Bilateria;Protostomia;
Spiralia;Lophotrochozoa;Mollusca;Bivalvia;Autobranchia;Heteroconchia;Euhe

terodonta;Imparidentia;Venerida;Veneroidea;Veneridae;Mercenaria;Mercenaria mercenaria
UdeM00000029838 7741 278.5 UdeM00000029838 XP_019622111.1 50.5 281 134 4 5 283 1 278 9.60E-72 278.5 7741 cellular organisms;Eukaryota;Opisthokonta;Metazoa;Eumetazoa;Bilateria;Deuterostomia;Chordata;Cephalochordata;Leptocardii;Amphioxiformes;Branchiostomidae;Branchiostoma;Branchiostoma belcheri
UdeM00000029840 6596 479.6 UdeM00000029840 XP_045197069.1 41.3 659 293 10 8 661 100 669 6.60E-132 479.6 6596 cellular organisms;Eukaryota;Opisthokonta;Metazoa;Eumetazoa;Bilateria;Protostomia;Spiralia;Lophotrochozoa;Mollusca;Bivalvia;Autobranchia;Heteroconchia;Euheterodonta;Imparidentia;Venerida;Veneroidea;Veneridae;Mercenaria;Mercenaria mercenaria
UdeM00000029839 6596 596.3 UdeM00000029839 XP_045197069.1 43 758 337 11 1 752 1 669 5.50E-167 596.3 6596 cellular organisms;Eukaryota;Opisthokonta;Metazoa;Eumetazoa;Bilateria;Protostomia;Spiralia;Lophotrochozoa;Mollusca;Bivalvia;Autobranchia;Heteroconchia;Euheterodonta;Imparidentia;Venerida;Veneroidea;Veneridae;Mercenaria;Mercenaria mercenaria
UdeM00000029841 6596 101.3 UdeM00000029841 XP_045197068.1 52 98 44 2 1 98 468 562 7.40E-19 101.3 6596 cellular organisms;Eukaryota;Opisthokonta;Metazoa;Eumetazoa;Bilateria;Protostomia;Spiralia;Lophotrochozoa;Mollusca;Bivalvia;Autobranchia;Heteroconchia;Euheterodonta;Imparidentia;Venerida;Veneroidea;Veneridae;Mercenaria;Mercenaria mercenaria
UdeM00000029842 6596 77 UdeM00000029842 XP_045201878.1 56.7 67 25 1 1 63 1 67 1.50E-11 77 6596 cellular organisms;Eukaryota;Opisthokonta;Metazoa;Eumetazoa;Bilateria;Protostomia;Spiralia;Lophotrochozoa;Mollusca;Bivalvia;Autobranchia;Heteroconchia;Euheterodonta;Imparidentia;Venerida;Veneroidea;Veneridae;Mercenaria;Mercenaria mercenaria
UdeM00000029843 6596 272.7 UdeM00000029843 XP_045197050.1 61.6 198 75 1 66 262 710 907 8.50E-70 272.7 6596 cellular organisms;Eukaryota;Opisthokonta;Metazoa;Eumetazoa;Bilateria;Protostomia;Spiralia;Lophotrochozoa;Mollusca;Bivalvia;Autobranchia;Heteroconchia;Euheterodonta;Imparidentia;Venerida;Veneroidea;Veneridae;Mercenaria;Mercenaria mercenaria
UdeM00000029844 6596 725.3 UdeM00000029844 XP_045197050.1 52.6 701 302 9 23 707 24 710 7.50E-206 725.3 6596 cellular organisms;Eukaryota;Opisthokonta;Metazoa;Eumetazoa;Bilateria;Protostomia;Spiralia;Lophotrochozoa;Mollusca;Bivalvia;Autobranchia;Heteroconchia;Euheterodonta;Imparidentia;Venerida;Veneroidea;Veneridae;Mercenaria;Mercenaria mercenaria
UdeM00000029845 1735272 151.4 UdeM00000029845 XP_041351901.1 61.5 122 45 1 1 120 1 122 7.50E-34 151.4 1735272 cellular organisms;Eukaryota;Opisthokonta;Metazoa;Eumetazoa;Bilateria;Protostomia;Spiralia;Lophotrochozoa;Mollusca;Gastropoda;Neomphalina;Neomphaloidea;Peltospiridae;Gigantopelta;Gigantopelta aegis
UdeM00000029846 6596 496.1 UdeM00000029846 XP_045197821.1 47.4 546 270 9 20 557 9 545 5.80E-137 496.1 6596 cellular organisms;Eukaryota;Opisthokonta;Metazoa;Eumetazoa;Bilateria;Protostomia;Spiralia;Lophotrochozoa;Mollusca;Bivalvia;Autobranchia;Heteroconchia;Euhe

terodonta;Imparidentia;Venerida;Veneroidea;Veneridae;Mercenaria;Mercenaria mercenaria
UdeM00000029847 6596 581.3 UdeM00000029847 XP_045195290.1 40 821 456 11 5 820 27 815 2.00E-162 581.3 6596 cellular organisms;Eukaryota;Opisthokonta;Metazoa;Eumetazoa;Bilateria;Protostomia;Spiralia;Lophotrochozoa;Mollusca;Bivalvia;Autobranchia;Heteroconchia;Euheterodonta;Imparidentia;Venerida;Veneroidea;Veneridae;Mercenaria;Mercenaria mercenaria
UdeM00000029849 6596 96.3 UdeM00000029849 XP_045195284.1 31.3 201 118 7 1 198 7 190 4.80E-17 96.3 6596 cellular organisms;Eukaryota;Opisthokonta;Metazoa;Eumetazoa;Bilateria;Protostomia;Spiralia;Lophotrochozoa;Mollusca;Bivalvia;Autobranchia;Heteroconchia;Euheterodonta;Imparidentia;Venerida;Veneroidea;Veneridae;Mercenaria;Mercenaria mercenaria
UdeM00000029850 6596 394.8 UdeM00000029850 XP_045195290.1 39.6 553 291 4 1 553 233 742 3.00E-106 394.8 6596 cellular organisms;Eukaryota;Opisthokonta;Metazoa;Eumetazoa;Bilateria;Protostomia;Spiralia;Lophotrochozoa;Mollusca;Bivalvia;Autobranchia;Heteroconchia;Euheterodonta;Imparidentia;Venerida;Veneroidea;Veneridae;Mercenaria;Mercenaria mercenaria
UdeM00000029851 6596 194.9 UdeM00000029851 XP_045195297.1 33.9 363 222 3 377 739 97 441 9.10E-46 194.9 6596 cellular organisms;Eukaryota;Opisthokonta;Metazoa;Eumetazoa;Bilateria;Protostomia;Spiralia;Lophotrochozoa;Mollusca;Bivalvia;Autobranchia;Heteroconchia;Euheterodonta;Imparidentia;Venerida;Veneroidea;Veneridae;Mercenaria;Mercenaria mercenaria
UdeM00000029852 6596 198.4 UdeM00000029852 XP_045195290.1 33.7 365 223 4 308 672 472 817 7.90E-47 198.4 6596 cellular organisms;Eukaryota;Opisthokonta;Metazoa;Eumetazoa;Bilateria;Protostomia;Spiralia;Lophotrochozoa;Mollusca;Bivalvia;Autobranchia;Heteroconchia;Euheterodonta;Imparidentia;Venerida;Veneroidea;Veneridae;Mercenaria;Mercenaria mercenaria
UdeM00000029853 6596 194.5 UdeM00000029853 XP_045195297.1 33.9 363 222 3 686 1048 97 441 1.10E-45 194.5 6596 cellular organisms;Eukaryota;Opisthokonta;Metazoa;Eumetazoa;Bilateria;Protostomia;Spiralia;Lophotrochozoa;Mollusca;Bivalvia;Autobranchia;Heteroconchia;Euheterodonta;Imparidentia;Venerida;Veneroidea;Veneridae;Mercenaria;Mercenaria mercenaria
UdeM00000029854 6596 553.5 UdeM00000029854 XP_045195290.1 39 820 465 9 5 820 27 815 4.50E-154 553.5 6596 cellular organisms;Eukaryota;Opisthokonta;Metazoa;Eumetazoa;Bilateria;Protostomia;Spiralia;Lophotrochozoa;Mollusca;Bivalvia;Autobranchia;Heteroconchia;Euheterodonta;Imparidentia;Venerida;Veneroidea;Veneridae;Mercenaria;Mercenaria mercenaria
UdeM00000029856 6596 117.1 UdeM00000029856 XP_045195290.1 47.5 122 64 0 1 122 195 316 1.70E-23 117.1 6596 cellular organisms;Eukaryota;Opisthokonta;Metazoa;Eumetazoa;Bilateria;Protostomia;Spiralia;Lophotrochozoa;Mollusca;Bivalvia;Autobranchia;Heteroconchia;Euheterodonta;Imparidentia;Venerida;Veneroidea;Veneridae;Mercenaria;Mercenaria mercenaria
UdeM00000029857 6596 288.1 UdeM00000029857 XP_045195290.1 34.4 508 283 7 1 477 327 815 2.10E-74 288.1 6596 cellular organisms;Eukaryota;Opisthokonta;Metazoa;Eumetazoa;Bilateria;Protostomia;Spiralia;Lophotrochozoa;Mollusca;Bivalvia;Autobranchia;Heteroconchia;Euhe

terodonta;Imparidentia;Venerida;Veneroidea;Veneridae;Mercenaria;Mercenaria mercenaria
UdeM00000029858 6596 564.7 UdeM00000029858 XP_045195290.1 39 821 464 11 5 820 27 815 1.90E-157 564.7 6596 cellular organisms;Eukaryota;Opisthokonta;Metazoa;Eumetazoa;Bilateria;Protostomia;Spiralia;Lophotrochozoa;Mollusca;Bivalvia;Autobranchia;Heteroconchia;Euheterodonta;Imparidentia;Venerida;Veneroidea;Veneridae;Mercenaria;Mercenaria mercenaria
UdeM00000029859 6596 400.2 UdeM00000029859 XP_045195290.1 37.8 624 357 7 1 599 195 812 4.70E-108 400.2 6596 cellular organisms;Eukaryota;Opisthokonta;Metazoa;Eumetazoa;Bilateria;Protostomia;Spiralia;Lophotrochozoa;Mollusca;Bivalvia;Autobranchia;Heteroconchia;Euheterodonta;Imparidentia;Venerida;Veneroidea;Veneridae;Mercenaria;Mercenaria mercenaria
UdeM00000029860 6596 549.7 UdeM00000029860 XP_045195290.1 38.5 820 469 8 5 820 27 815 6.50E-153 549.7 6596 cellular organisms;Eukaryota;Opisthokonta;Metazoa;Eumetazoa;Bilateria;Protostomia;Spiralia;Lophotrochozoa;Mollusca;Bivalvia;Autobranchia;Heteroconchia;Euheterodonta;Imparidentia;Venerida;Veneroidea;Veneridae;Mercenaria;Mercenaria mercenaria
UdeM00000029862 6596 520.8 UdeM00000029862 XP_045195290.1 40.1 735 405 9 7 736 29 733 2.90E-144 520.8 6596 cellular organisms;Eukaryota;Opisthokonta;Metazoa;Eumetazoa;Bilateria;Protostomia;Spiralia;Lophotrochozoa;Mollusca;Bivalvia;Autobranchia;Heteroconchia;Euheterodonta;Imparidentia;Venerida;Veneroidea;Veneridae;Mercenaria;Mercenaria mercenaria
UdeM00000029864 6596 560.8 UdeM00000029864 XP_045195290.1 39.2 821 462 11 5 820 27 815 2.80E-156 560.8 6596 cellular organisms;Eukaryota;Opisthokonta;Metazoa;Eumetazoa;Bilateria;Protostomia;Spiralia;Lophotrochozoa;Mollusca;Bivalvia;Autobranchia;Heteroconchia;Euheterodonta;Imparidentia;Venerida;Veneroidea;Veneridae;Mercenaria;Mercenaria mercenaria
UdeM00000029866 6565 62.4 UdeM00000029866 XP_022334943.1 43.3 67 38 0 2 68 112 178 3.10E-07 62.4 6565 cellular organisms;Eukaryota;Opisthokonta;Metazoa;Eumetazoa;Bilateria;Protostomia;Spiralia;Lophotrochozoa;Mollusca;Bivalvia;Autobranchia;Pteriomorphia;Ostreida;Ostreoidea;Ostreidae;Crassostrea;Crassostrea virginica
UdeM00000029868 6596 570.9 UdeM00000029868 XP_045197246.1 33.3 1300 536 31 7 991 754 2037 3.30E-159 570.9 6596 cellular organisms;Eukaryota;Opisthokonta;Metazoa;Eumetazoa;Bilateria;Protostomia;Spiralia;Lophotrochozoa;Mollusca;Bivalvia;Autobranchia;Heteroconchia;Euheterodonta;Imparidentia;Venerida;Veneroidea;Veneridae;Mercenaria;Mercenaria mercenaria
UdeM00000029869 6596 391 UdeM00000029869 XP_045170511.1 31.7 773 445 22 264 975 179 929 5.30E-105 391 6596 cellular organisms;Eukaryota;Opisthokonta;Metazoa;Eumetazoa;Bilateria;Protostomia;Spiralia;Lophotrochozoa;Mollusca;Bivalvia;Autobranchia;Heteroconchia;Euheterodonta;Imparidentia;Venerida;Veneroidea;Veneridae;Mercenaria;Mercenaria mercenaria
UdeM00000029870 6596 109.4 UdeM00000029870 XP_045196093.1 37.9 190 112 5 10 196 290 476 5.40E-21 109.4 6596 cellular organisms;Eukaryota;Opisthokonta;Metazoa;Eumetazoa;Bilateria;Protostomia;Spiralia;Lophotrochozoa;Mollusca;Bivalvia;Autobranchia;Heteroconchia;Euhe

terodonta;Imparidentia;Venerida;Veneroidea;Veneridae;Mercenaria;Mercenari
a mercenaria
UdeM00000029872 6596 325.1 UdeM00000029872 XP_045197245.1 33.2 707
425 22 128 804 664 1353 2.60E-85 325.1 6596 cellular
organisms;Eukaryota;Opisthokonta;Metazoa;Eumetazoa;Bilateria;Protostomia;
Spiralia;Lophotrochozoa;Mollusca;Bivalvia;Autobranchia;Heteroconchia;Euhe
terodonta;Imparidentia;Venerida;Veneroidea;Veneridae;Mercenaria;Mercenari
a mercenaria
UdeM00000029873 6596 122.9 UdeM00000029873 XP_045197368.1 32.1 243
160 4 19 259 163 402 6.40E-25 122.9 6596 cellular
organisms;Eukaryota;Opisthokonta;Metazoa;Eumetazoa;Bilateria;Protostomia;
Spiralia;Lophotrochozoa;Mollusca;Bivalvia;Autobranchia;Heteroconchia;Euhe
terodonta;Imparidentia;Venerida;Veneroidea;Veneridae;Mercenaria;Mercenari
a mercenaria
UdeM00000029874 6573 188.7 UdeM00000029874 XP_021358159.1 37.2 298
174 7 6 293 366 660 1.60E-44 188.7 6573 cellular
organisms;Eukaryota;Opisthokonta;Metazoa;Eumetazoa;Bilateria;Protostomia;
Spiralia;Lophotrochozoa;Mollusca;Bivalvia;Autobranchia;Pteriomorphia;Pect
inida;Pectinoidea;Pectinidae;Mizuhopecten;Mizuhopecten yessoensis
UdeM00000029875 6596 189.5 UdeM00000029875 XP_045197249.1 42.3 253
137 4 220 468 438 685 1.10E-44 189.5 6596 cellular
organisms;Eukaryota;Opisthokonta;Metazoa;Eumetazoa;Bilateria;Protostomia;
Spiralia;Lophotrochozoa;Mollusca;Bivalvia;Autobranchia;Heteroconchia;Euhe
terodonta;Imparidentia;Venerida;Veneroidea;Veneridae;Mercenaria;Mercenari
a mercenaria
UdeM00000029876 6596 77.4 UdeM00000029876 XP_045197385.1 28.9 204
131 6 2 201 379 572 2.50E-11 77.4 6596 cellular
organisms;Eukaryota;Opisthokonta;Metazoa;Eumetazoa;Bilateria;Protostomia;
Spiralia;Lophotrochozoa;Mollusca;Bivalvia;Autobranchia;Heteroconchia;Euhe
terodonta;Imparidentia;Venerida;Veneroidea;Veneridae;Mercenaria;Mercenari
a mercenaria
UdeM00000029877 6596 67.4 UdeM00000029877 XP_045197368.1 38 100
60 2 4 101 336 435 1.30E-08 67.4 6596 cellular
organisms;Eukaryota;Opisthokonta;Metazoa;Eumetazoa;Bilateria;Protostomia;
Spiralia;Lophotrochozoa;Mollusca;Bivalvia;Autobranchia;Heteroconchia;Euhe
terodonta;Imparidentia;Venerida;Veneroidea;Veneridae;Mercenaria;Mercenari
a mercenaria
UdeM00000029880 6573 448.4 UdeM00000029880 XP_021344066.1 60.1 343
135 1 69 411 150 490 1.10E-122 448.4 6573 cellular
organisms;Eukaryota;Opisthokonta;Metazoa;Eumetazoa;Bilateria;Protostomia;
Spiralia;Lophotrochozoa;Mollusca;Bivalvia;Autobranchia;Pteriomorphia;Pect
inida;Pectinoidea;Pectinidae;Mizuhopecten;Mizuhopecten yessoensis
UdeM00000029881 441366 391.7 UdeM00000029881 XP_028296753.1 39.3
596 332 13 6 593 32 605 1.60E-105 391.7 441366
cellular
organisms;Eukaryota;Opisthokonta;Metazoa;Eumetazoa;Bilateria;Deuterostomi
a;Chordata;Craniata;Vertebrata;Gnathostomata;Teleostomi;Euteleostomi;Acti
nopterygii;Actinopteri;Neopterygii;Teleostei;Osteoglossocephalai;Clupeoce
phala;Euteleosteomorpha;Neoteleostei;Eurypterygia;Ctenosquamata;Acanthomo
rphata;Euacanthomorphacea;Percomorphaceae;Ovalentaria;Blenniimorphae;Blen
niiformes;Gobiesocoidei;Gobiesocidae;Gobiesocinae;Gouania;Gouania
willdenowi
UdeM00000029882 6596 513.5 UdeM00000029882 XP_045197246.1 39.6 826
357 14 7 704 1226 2037 4.40E-142 513.5 6596 cellular

organisms;Eukaryota;Opisthokonta;Metazoa;Eumetazoa;Bilateria;Protostomia;Spiralia;Lophotrochozoa;Mollusca;Bivalvia;Autobranchia;Heteroconchia;Euheterodonta;Imparidentia;Venerida;Veneroidea;Veneridae;Mercenaria;Mercenaria mercenaria
UdeM00000029883 29159 329.7 UdeM00000029883 XP_034332444.1 48.9 350 163 5 378 720 1779 2119 9.20E-87 329.7 29159 cellular organisms;Eukaryota;Opisthokonta;Metazoa;Eumetazoa;Bilateria;Protostomia;Spiralia;Lophotrochozoa;Mollusca;Bivalvia;Autobranchia;Pteriomorphia;Ostreida;Ostreoidea;Ostreidae;Crassostrea;Crassostrea gigas
UdeM00000029884 29159 407.9 UdeM00000029884 XP_034332444.1 60 320 119 3 7 317 1792 2111 1.20E-110 407.9 29159 cellular organisms;Eukaryota;Opisthokonta;Metazoa;Eumetazoa;Bilateria;Protostomia;Spiralia;Lophotrochozoa;Mollusca;Bivalvia;Autobranchia;Pteriomorphia;Ostreida;Ostreoidea;Ostreidae;Crassostrea;Crassostrea gigas
UdeM00000029885 29159 405.2 UdeM00000029885 XP_034332444.1 57.9 337 127 4 7 337 1792 2119 8.10E-110 405.2 29159 cellular organisms;Eukaryota;Opisthokonta;Metazoa;Eumetazoa;Bilateria;Protostomia;Spiralia;Lophotrochozoa;Mollusca;Bivalvia;Autobranchia;Pteriomorphia;Ostreida;Ostreoidea;Ostreidae;Crassostrea;Crassostrea gigas
UdeM00000029886 6596 408.7 UdeM00000029886 XP_045197249.1 36.8 684 397 20 29 685 438 1113 1.50E-110 408.7 6596 cellular organisms;Eukaryota;Opisthokonta;Metazoa;Eumetazoa;Bilateria;Protostomia;Spiralia;Lophotrochozoa;Mollusca;Bivalvia;Autobranchia;Heteroconchia;Euheterodonta;Imparidentia;Venerida;Veneroidea;Veneridae;Mercenaria;Mercenaria mercenaria
UdeM00000029887 29159 215.3 UdeM00000029887 XP_034332444.1 53.5 187 78 3 51 229 1926 2111 8.80E-53 215.3 29159 cellular organisms;Eukaryota;Opisthokonta;Metazoa;Eumetazoa;Bilateria;Protostomia;Spiralia;Lophotrochozoa;Mollusca;Bivalvia;Autobranchia;Pteriomorphia;Ostreida;Ostreoidea;Ostreidae;Crassostrea;Crassostrea gigas
UdeM00000029888 29159 212.2 UdeM00000029888 XP_034332444.1 53.5 187 77 3 51 228 1926 2111 7.40E-52 212.2 29159 cellular organisms;Eukaryota;Opisthokonta;Metazoa;Eumetazoa;Bilateria;Protostomia;Spiralia;Lophotrochozoa;Mollusca;Bivalvia;Autobranchia;Pteriomorphia;Ostreida;Ostreoidea;Ostreidae;Crassostrea;Crassostrea gigas
UdeM00000029890 6596 381.7 UdeM00000029890 XP_045197249.1 25.8 1471 498 36 123 1010 500 1959 3.00E-102 381.7 6596 cellular organisms;Eukaryota;Opisthokonta;Metazoa;Eumetazoa;Bilateria;Protostomia;Spiralia;Lophotrochozoa;Mollusca;Bivalvia;Autobranchia;Heteroconchia;Euheterodonta;Imparidentia;Venerida;Veneroidea;Veneridae;Mercenaria;Mercenaria mercenaria
UdeM00000029891 6596 201.8 UdeM00000029891 XP_045197245.1 31 630 357 18 634 1248 441 1007 5.00E-48 201.8 6596 cellular organisms;Eukaryota;Opisthokonta;Metazoa;Eumetazoa;Bilateria;Protostomia;Spiralia;Lophotrochozoa;Mollusca;Bivalvia;Autobranchia;Heteroconchia;Euheterodonta;Imparidentia;Venerida;Veneroidea;Veneridae;Mercenaria;Mercenaria mercenaria
UdeM00000029892 29159 411.4 UdeM00000029892 XP_034332444.1 55.8 346 143 4 6 341 1766 2111 1.20E-111 411.4 29159 cellular organisms;Eukaryota;Opisthokonta;Metazoa;Eumetazoa;Bilateria;Protostomia;Spiralia;Lophotrochozoa;Mollusca;Bivalvia;Autobranchia;Pteriomorphia;Ostreida;Ostreoidea;Ostreidae;Crassostrea;Crassostrea gigas
UdeM00000029893 6596 599.4 UdeM00000029893 XP_045196094.1 35.1 1151 598 30 38 1096 268 1361 9.50E-168 599.4 6596 cellular

organisms;Eukaryota;Opisthokonta;Metazoa;Eumetazoa;Bilateria;Protostomia;
Spiralia;Lophotrochozoa;Mollusca;Bivalvia;Autobranchia;Heteroconchia;Euheterodonta;Imparidentia;Venerida;Veneroidea;Veneridae;Mercenaria;Mercenaria mercenaria
UdeM00000029894 6596 221.9 UdeM00000029894 XP_045195374.1 52.4 208
97 1 22 227 19 226 8.60E-55 221.9 6596 cellular
organisms;Eukaryota;Opisthokonta;Metazoa;Eumetazoa;Bilateria;Protostomia;
Spiralia;Lophotrochozoa;Mollusca;Bivalvia;Autobranchia;Heteroconchia;Euheterodonta;Imparidentia;Venerida;Veneroidea;Veneridae;Mercenaria;Mercenaria mercenaria
UdeM00000029895 6573 526.9 UdeM00000029895 XP_021360724.1 57.5 699
274 8 4 695 10 692 3.80E-146 526.9 6573 cellular
organisms;Eukaryota;Opisthokonta;Metazoa;Eumetazoa;Bilateria;Protostomia;
Spiralia;Lophotrochozoa;Mollusca;Bivalvia;Autobranchia;Pteriomorphia;Pectinida;Pectinoidea;Pectinidae;Mizuhopecten;Mizuhopecten yessoensis
UdeM00000029896 6573 519.6 UdeM00000029896 XP_021360724.1 55.5 722
275 8 4 718 10 692 6.30E-144 519.6 6573 cellular
organisms;Eukaryota;Opisthokonta;Metazoa;Eumetazoa;Bilateria;Protostomia;
Spiralia;Lophotrochozoa;Mollusca;Bivalvia;Autobranchia;Pteriomorphia;Pectinida;Pectinoidea;Pectinidae;Mizuhopecten;Mizuhopecten yessoensis
UdeM00000029898 6596 173.7 UdeM00000029898 XP_045197682.1 54.2 203
82 5 1 201 4 197 2.50E-40 173.7 6596 cellular
organisms;Eukaryota;Opisthokonta;Metazoa;Eumetazoa;Bilateria;Protostomia;
Spiralia;Lophotrochozoa;Mollusca;Bivalvia;Autobranchia;Heteroconchia;Euheterodonta;Imparidentia;Venerida;Veneroidea;Veneridae;Mercenaria;Mercenaria mercenaria
UdeM00000029899 6596 320.1 UdeM00000029899 XP_045196432.1 47.3 336
142 3 43 343 26 361 3.50E-84 320.1 6596 cellular
organisms;Eukaryota;Opisthokonta;Metazoa;Eumetazoa;Bilateria;Protostomia;
Spiralia;Lophotrochozoa;Mollusca;Bivalvia;Autobranchia;Heteroconchia;Euheterodonta;Imparidentia;Venerida;Veneroidea;Veneridae;Mercenaria;Mercenaria mercenaria
UdeM00000029900 6596 425.6 UdeM00000029900 XP_045196429.1 74 262
68 0 62 323 83 344 5.50E-116 425.6 6596 cellular
organisms;Eukaryota;Opisthokonta;Metazoa;Eumetazoa;Bilateria;Protostomia;
Spiralia;Lophotrochozoa;Mollusca;Bivalvia;Autobranchia;Heteroconchia;Euheterodonta;Imparidentia;Venerida;Veneroidea;Veneridae;Mercenaria;Mercenaria mercenaria
UdeM00000029901 6596 1147.9 UdeM00000029901 XP_045197475.1 55.4
1014 434 10 7 1013 30 1032 0.00E+00 1147.9 6596
cellular
organisms;Eukaryota;Opisthokonta;Metazoa;Eumetazoa;Bilateria;Protostomia;
Spiralia;Lophotrochozoa;Mollusca;Bivalvia;Autobranchia;Heteroconchia;Euheterodonta;Imparidentia;Venerida;Veneroidea;Veneridae;Mercenaria;Mercenaria mercenaria
UdeM00000029902 6596 1120.5 UdeM00000029902 XP_045197475.1 54.6
1007 431 9 7 991 30 1032 0.00E+00 1120.5 6596
cellular
organisms;Eukaryota;Opisthokonta;Metazoa;Eumetazoa;Bilateria;Protostomia;
Spiralia;Lophotrochozoa;Mollusca;Bivalvia;Autobranchia;Heteroconchia;Euheterodonta;Imparidentia;Venerida;Veneroidea;Veneridae;Mercenaria;Mercenaria mercenaria
UdeM00000029903 6596 1115.5 UdeM00000029903 XP_045197475.1 54.4
1007 433 9 7 991 30 1032 0.00E+00 1115.5 6596

cellular organisms;Eukaryota;Opisthokonta;Metazoa;Eumetazoa;Bilateria;Protostomia;Spiralia;Lophotrochozoa;Mollusca;Bivalvia;Autobranchia;Heteroconchia;Euheterodonta;Imparidentia;Venerida;Veneroidea;Veneridae;Mercenaria;Mercenaria mercenaria
UdeM00000029904 6596 1143.3 UdeM00000029904 XP_045197475.1 55.2 1014 436 10 7 1013 30 1032 0.00E+00 1143.3 6596 cellular organisms;Eukaryota;Opisthokonta;Metazoa;Eumetazoa;Bilateria;Protostomia;Spiralia;Lophotrochozoa;Mollusca;Bivalvia;Autobranchia;Heteroconchia;Euheterodonta;Imparidentia;Venerida;Veneroidea;Veneridae;Mercenaria;Mercenaria mercenaria
UdeM00000029905 6579 505 UdeM00000029905 XP_033738649.1 63.2 383 139 1 1 383 1 381 8.60E-140 505 6579 cellular organisms;Eukaryota;Opisthokonta;Metazoa;Eumetazoa;Bilateria;Protostomia;Spiralia;Lophotrochozoa;Mollusca;Bivalvia;Autobranchia;Pteriomorphia;Pectinida;Pectinoidea;Pectinidae;Pecten;Pecten maximus
UdeM00000029906 6579 619 UdeM00000029906 XP_033738649.1 64.7 453 157 2 1 453 1 450 5.10E-174 619 6579 cellular organisms;Eukaryota;Opisthokonta;Metazoa;Eumetazoa;Bilateria;Protostomia;Spiralia;Lophotrochozoa;Mollusca;Bivalvia;Autobranchia;Pteriomorphia;Pectinida;Pectinoidea;Pectinidae;Pecten;Pecten maximus
UdeM00000029908 29159 79.3 UdeM00000029908 XP_011427293.2 35.1 154 83 4 1 138 45 197 4.20E-12 79.3 29159 cellular organisms;Eukaryota;Opisthokonta;Metazoa;Eumetazoa;Bilateria;Protostomia;Spiralia;Lophotrochozoa;Mollusca;Bivalvia;Autobranchia;Pteriomorphia;Ostreida;Ostreoidea;Ostreidae;Crassostrea;Crassostrea gigas
UdeM00000029907 6596 110.5 UdeM00000029907 XP_045209482.1 44.2 147 79 1 25 168 20 166 2.50E-21 110.5 6596 cellular organisms;Eukaryota;Opisthokonta;Metazoa;Eumetazoa;Bilateria;Protostomia;Spiralia;Lophotrochozoa;Mollusca;Bivalvia;Autobranchia;Heteroconchia;Euheterodonta;Imparidentia;Venerida;Veneroidea;Veneridae;Mercenaria;Mercenaria mercenaria
UdeM00000029911 6596 76.6 UdeM00000029911 XP_045209482.1 36.4 132 81 1 1 129 59 190 2.80E-11 76.6 6596 cellular organisms;Eukaryota;Opisthokonta;Metazoa;Eumetazoa;Bilateria;Protostomia;Spiralia;Lophotrochozoa;Mollusca;Bivalvia;Autobranchia;Heteroconchia;Euheterodonta;Imparidentia;Venerida;Veneroidea;Veneridae;Mercenaria;Mercenaria mercenaria
UdeM00000029910 6596 111.3 UdeM00000029910 XP_045169739.1 36.9 198 122 1 5 202 7 201 1.50E-21 111.3 6596 cellular organisms;Eukaryota;Opisthokonta;Metazoa;Eumetazoa;Bilateria;Protostomia;Spiralia;Lophotrochozoa;Mollusca;Bivalvia;Autobranchia;Heteroconchia;Euheterodonta;Imparidentia;Venerida;Veneroidea;Veneridae;Mercenaria;Mercenaria mercenaria
UdeM00000029914 6579 602.8 UdeM00000029914 XP_033738649.1 62.5 464 160 3 1 464 1 450 3.90E-169 602.8 6579 cellular organisms;Eukaryota;Opisthokonta;Metazoa;Eumetazoa;Bilateria;Protostomia;Spiralia;Lophotrochozoa;Mollusca;Bivalvia;Autobranchia;Pteriomorphia;Pectinida;Pectinoidea;Pectinidae;Pecten;Pecten maximus
UdeM00000029915 6596 80.5 UdeM00000029915 XP_045187509.1 31.9 141 95 1 1 141 65 204 2.10E-12 80.5 6596 cellular organisms;Eukaryota;Opisthokonta;Metazoa;Eumetazoa;Bilateria;Protostomia;Spiralia;Lophotrochozoa;Mollusca;Bivalvia;Autobranchia;Heteroconchia;Euhe

terodonta;Imparidentia;Venerida;Veneroidea;Veneridae;Mercenaria;Mercenaria mercenaria
UdeM00000029916 6596 112.8 UdeM00000029916 XP_045209482.1 46.2 143 74 1 25 164 20 162 5.40E-22 112.8 6596 cellular organisms;Eukaryota;Opisthokonta;Metazoa;Eumetazoa;Bilateria;Protostomia;Spiralia;Lophotrochozoa;Mollusca;Bivalvia;Autobranchia;Heteroconchia;Euheterodonta;Imparidentia;Venerida;Veneroidea;Veneridae;Mercenaria;Mercenaria mercenaria
UdeM00000029917 6596 72 UdeM00000029917 XP_045185101.1 30.6 144 84 3 34 164 23 163 7.90E-10 72 6596 cellular organisms;Eukaryota;Opisthokonta;Metazoa;Eumetazoa;Bilateria;Protostomia;Spiralia;Lophotrochozoa;Mollusca;Bivalvia;Autobranchia;Heteroconchia;Euheterodonta;Imparidentia;Venerida;Veneroidea;Veneridae;Mercenaria;Mercenaria mercenaria
UdeM00000029918 6596 107.8 UdeM00000029918 XP_045209482.1 43.8 144 75 2 1 144 1 138 1.80E-20 107.8 6596 cellular organisms;Eukaryota;Opisthokonta;Metazoa;Eumetazoa;Bilateria;Protostomia;Spiralia;Lophotrochozoa;Mollusca;Bivalvia;Autobranchia;Heteroconchia;Euheterodonta;Imparidentia;Venerida;Veneroidea;Veneridae;Mercenaria;Mercenaria mercenaria
UdeM00000029919 1735272 109.4 UdeM00000029919 XP_041374221.1 35.8 187 110 3 20 202 12 192 5.50E-21 109.4 1735272 cellular organisms;Eukaryota;Opisthokonta;Metazoa;Eumetazoa;Bilateria;Protostomia;Spiralia;Lophotrochozoa;Mollusca;Gastropoda;Neomphalina;Neomphaloidea;Peltospiridae;Gigantopelta;Gigantopelta aegis
UdeM00000029920 1735272 111.3 UdeM00000029920 XP_041357433.1 42.1 152 85 2 39 189 14 163 1.60E-21 111.3 1735272 cellular organisms;Eukaryota;Opisthokonta;Metazoa;Eumetazoa;Bilateria;Protostomia;Spiralia;Lophotrochozoa;Mollusca;Gastropoda;Neomphalina;Neomphaloidea;Peltospiridae;Gigantopelta;Gigantopelta aegis
UdeM00000029921 747089 85.5 UdeM00000029921 XP_025175728.1 27.2 305 191 10 10 290 532 829 2.50E-13 85.5 747089 cellular organisms;Eukaryota;Opisthokonta;Fungi;Fungi incertae sedis;Mucoromycota;Glomeromycotina;Glomeromycetes;Glomerales;Glomeraceae;Rhizophagus;Rhizophagus irregularis;Rhizophagus irregularis DAOM 181602=DAOM 197198
UdeM00000029922 747089 95.9 UdeM00000029922 XP_025174809.1 28 311 196 10 10 299 504 807 1.80E-16 95.9 747089 cellular organisms;Eukaryota;Opisthokonta;Fungi;Fungi incertae sedis;Mucoromycota;Glomeromycotina;Glomeromycetes;Glomerales;Glomeraceae;Rhizophagus;Rhizophagus irregularis;Rhizophagus irregularis DAOM 181602=DAOM 197198
UdeM00000029926 1735272 110.2 UdeM00000029926 XP_041374221.1 36.2 185 110 3 19 199 12 192 3.20E-21 110.2 1735272 cellular organisms;Eukaryota;Opisthokonta;Metazoa;Eumetazoa;Bilateria;Protostomia;Spiralia;Lophotrochozoa;Mollusca;Gastropoda;Neomphalina;Neomphaloidea;Peltospiridae;Gigantopelta;Gigantopelta aegis
UdeM00000029927 225164 108.2 UdeM00000029927 XP_009058199.1 49.2 118 52 3 1 117 1 111 7.10E-21 108.2 225164 cellular organisms;Eukaryota;Opisthokonta;Metazoa;Eumetazoa;Bilateria;Protostomia;

Spiralia;Lophotrochozoa;Mollusca;Gastropoda;Patellogastropoda;Lottioidea;
Lottiidae;Lottia;Lottia gigantea
UdeM00000029928 6579 216.5 UdeM00000029928 XP_033752673.1 56 200
70 1 1 182 453 652 2.90E-53 216.5 6579 cellular
organisms;Eukaryota;Opisthokonta;Metazoa;Eumetazoa;Bilateria;Protostomia;
Spiralia;Lophotrochozoa;Mollusca;Bivalvia;Autobranchia;Pteriomorphia;Pect
inida;Pectinoidea;Pectinidae;Pecten;Pecten maximus
UdeM00000029929 225164 121.7 UdeM00000029929 XP_009058200.1 50.9
108 53 0 4 111 108 215 5.90E-25 121.7 225164
cellular
organisms;Eukaryota;Opisthokonta;Metazoa;Eumetazoa;Bilateria;Protostomia;
Spiralia;Lophotrochozoa;Mollusca;Gastropoda;Patellogastropoda;Lottioidea;
Lottiidae;Lottia;Lottia gigantea
UdeM00000029930 8175 234.2 UdeM00000029930 XP_030292627.1 57.5 181
77 0 9 189 11 191 1.40E-58 234.2 8175 cellular
organisms;Eukaryota;Opisthokonta;Metazoa;Eumetazoa;Bilateria;Deuterostomi
a;Chordata;Craniata;Vertebrata;Gnathostomata;Teleostomi;Euteleostomi;Acti
nopterygii;Actinopteri;Neopterygii;Teleostei;Osteoglossocephalai;Clupeoce
phala;Euteleosteomorpha;Neoteleostei;Eurypterygia;Ctenosquamata;Acanthomo
rphata;Euacanthomorphacea;Percomorphaceae;Eupercaria;Spariformes;Sparidae
;Sparus;Sparus aurata
UdeM00000029931 6596 112.1 UdeM00000029931 XP_045209482.1 46 139
72 1 29 164 24 162 8.80E-22 112.1 6596 cellular
organisms;Eukaryota;Opisthokonta;Metazoa;Eumetazoa;Bilateria;Protostomia;
Spiralia;Lophotrochozoa;Mollusca;Bivalvia;Autobranchia;Heteroconchia;Euhe
terodonta;Imparidentia;Venerida;Veneroidea;Veneridae;Mercenaria;Mercenari
a mercenaria
UdeM00000029934 6596 107.5 UdeM00000029934 XP_045209482.1 43.8 144
75 2 1 144 1 138 2.30E-20 107.5 6596 cellular
organisms;Eukaryota;Opisthokonta;Metazoa;Eumetazoa;Bilateria;Protostomia;
Spiralia;Lophotrochozoa;Mollusca;Bivalvia;Autobranchia;Heteroconchia;Euhe
terodonta;Imparidentia;Venerida;Veneroidea;Veneridae;Mercenaria;Mercenari
a mercenaria
UdeM00000029935 1735272 112.8 UdeM00000029935 XP_041374221.1 37.7
167 96 1 1 167 34 192 4.20E-22 112.8 1735272
cellular
organisms;Eukaryota;Opisthokonta;Metazoa;Eumetazoa;Bilateria;Protostomia;
Spiralia;Lophotrochozoa;Mollusca;Gastropoda;Neomphalina;Neomphaloidea;Pel
tospiridae;Gigantopelta;Gigantopelta aegis
UdeM00000029936 1735272 117.9 UdeM00000029936 XP_041374221.1 36.8
190 107 3 27 212 12 192 1.60E-23 117.9 1735272
cellular
organisms;Eukaryota;Opisthokonta;Metazoa;Eumetazoa;Bilateria;Protostomia;
Spiralia;Lophotrochozoa;Mollusca;Gastropoda;Neomphalina;Neomphaloidea;Pel
tospiridae;Gigantopelta;Gigantopelta aegis
UdeM00000029937 6596 105.9 UdeM00000029937 XP_045209482.1 40.7 167
84 3 13 175 8 163 6.50E-20 105.9 6596 cellular
organisms;Eukaryota;Opisthokonta;Metazoa;Eumetazoa;Bilateria;Protostomia;
Spiralia;Lophotrochozoa;Mollusca;Bivalvia;Autobranchia;Heteroconchia;Euhe
terodonta;Imparidentia;Venerida;Veneroidea;Veneridae;Mercenaria;Mercenari
a mercenaria
UdeM00000029938 6596 99.8 UdeM00000029938 XP_045183938.1 36.7 177
93 4 5 177 14 175 4.70E-18 99.8 6596 cellular
organisms;Eukaryota;Opisthokonta;Metazoa;Eumetazoa;Bilateria;Protostomia;

Spiralia;Lophotrochozoa;Mollusca;Bivalvia;Autobranchia;Heteroconchia;Euheterodonta;Imparidentia;Venerida;Veneroidea;Veneridae;Mercenaria;Mercenaria mercenaria
UdeM00000029939 6596 114 UdeM00000029939 XP_045209482.1 44 159 79 3 24 177 14 167 2.30E-22 114 6596 cellular organisms;Eukaryota;Opisthokonta;Metazoa;Eumetazoa;Bilateria;Protostomia;Spiralia;Lophotrochozoa;Mollusca;Bivalvia;Autobranchia;Heteroconchia;Euheterodonta;Imparidentia;Venerida;Veneroidea;Veneridae;Mercenaria;Mercenaria mercenaria
UdeM00000029940 747089 95.9 UdeM00000029940 XP_025174809.1 28.9 315 188 12 10 299 504 807 1.80E-16 95.9 747089 cellular organisms;Eukaryota;Opisthokonta;Fungi;Fungi incertae sedis;Mucoromycota;Glomeromycotina;Glomeromycetes;Glomerales;Glomeraceae;Rhizophagus;Rhizophagus irregularis;Rhizophagus irregularis DAOM 181602=DAOM 197198
UdeM00000029941 1735272 105.5 UdeM00000029941 XP_041374221.1 38 163 96 2 1 162 34 192 6.40E-20 105.5 1735272 cellular organisms;Eukaryota;Opisthokonta;Metazoa;Eumetazoa;Bilateria;Protostomia;Spiralia;Lophotrochozoa;Mollusca;Gastropoda;Neomphalina;Neomphaloidea;Peltospiridae;Gigantopelta;Gigantopelta aegis
UdeM00000029942 225164 107.1 UdeM00000029942 XP_009058199.1 48.3 118 53 3 1 117 1 111 1.60E-20 107.1 225164 cellular organisms;Eukaryota;Opisthokonta;Metazoa;Eumetazoa;Bilateria;Protostomia;Spiralia;Lophotrochozoa;Mollusca;Gastropoda;Patellogastropoda;Lottioidea;Lottiidae;Lottia;Lottia gigantea
UdeM00000029943 6579 265.4 UdeM00000029943 XP_033752673.1 63.5 200 73 0 1 200 453 652 5.90E-68 265.4 6579 cellular organisms;Eukaryota;Opisthokonta;Metazoa;Eumetazoa;Bilateria;Protostomia;Spiralia;Lophotrochozoa;Mollusca;Bivalvia;Autobranchia;Pteriomorphia;Pectinida;Pectinoidea;Pectinidae;Pecten;Pecten maximus
UdeM00000029944 205130 82.8 UdeM00000029944 XP_026180020.1 50.8 65 32 0 1 65 197 261 2.10E-13 82.8 205130 cellular organisms;Eukaryota;Opisthokonta;Metazoa;Eumetazoa;Bilateria;Deuterostomia;Chordata;Craniata;Vertebrata;Gnathostomata;Teleostomi;Euteleostomi;Actinopterygii;Actinopteri;Neopterygii;Teleostei;Osteoglossocephalai;Clupeocephala;Euteleosteomorpha;Neoteleostei;Eurypterygia;Ctenosquamata;Acanthomorphata;Euacanthomorphacea;Percomorphaceae;Anabantaria;Synbranchiformes;Mastacembeloidei;Mastacembelidae;Mastacembelus;Mastacembelus armatus
UdeM00000029945 8175 231.9 UdeM00000029945 XP_030292627.1 56.9 181 78 0 9 189 11 191 6.90E-58 231.9 8175 cellular organisms;Eukaryota;Opisthokonta;Metazoa;Eumetazoa;Bilateria;Deuterostomia;Chordata;Craniata;Vertebrata;Gnathostomata;Teleostomi;Euteleostomi;Actinopterygii;Actinopteri;Neopterygii;Teleostei;Osteoglossocephalai;Clupeocephala;Euteleosteomorpha;Neoteleostei;Eurypterygia;Ctenosquamata;Acanthomorphata;Euacanthomorphacea;Percomorphaceae;Eupercaria;Spariformes;Sparidae;Sparus;Sparus aurata
UdeM00000029946 6596 112.1 UdeM00000029946 XP_045209482.1 46.8 139 71 1 29 164 24 162 8.80E-22 112.1 6596 cellular organisms;Eukaryota;Opisthokonta;Metazoa;Eumetazoa;Bilateria;Protostomia;Spiralia;Lophotrochozoa;Mollusca;Bivalvia;Autobranchia;Heteroconchia;Euhe

terodonta;Imparidentia;Venerida;Veneroidea;Veneridae;Mercenaria;Mercenaria mercenaria
UdeM00000029948 6596 107.8 UdeM00000029948 XP_045209482.1 43.8 144 75 2 1 144 1 138 1.50E-20 107.8 6596 cellular organisms;Eukaryota;Opisthokonta;Metazoa;Eumetazoa;Bilateria;Protostomia;Spiralia;Lophotrochozoa;Mollusca;Bivalvia;Autobranchia;Heteroconchia;Euheterodonta;Imparidentia;Venerida;Veneroidea;Veneridae;Mercenaria;Mercenaria mercenaria
UdeM00000029950 1735272 116.7 UdeM00000029950 XP_041374221.1 36.3 190 108 3 22 207 12 192 3.60E-23 116.7 1735272 cellular organisms;Eukaryota;Opisthokonta;Metazoa;Eumetazoa;Bilateria;Protostomia;Spiralia;Lophotrochozoa;Mollusca;Gastropoda;Neomphalina;Neomphaloidea;Peltospiridae;Gigantopelta;Gigantopelta aegis
UdeM00000029951 1735272 107.5 UdeM00000029951 XP_041357433.1 35.6 188 113 5 48 231 11 194 2.40E-20 107.5 1735272 cellular organisms;Eukaryota;Opisthokonta;Metazoa;Eumetazoa;Bilateria;Protostomia;Spiralia;Lophotrochozoa;Mollusca;Gastropoda;Neomphalina;Neomphaloidea;Peltospiridae;Gigantopelta;Gigantopelta aegis
UdeM00000029952 6412 79.7 UdeM00000029952 XP_009017487.1 61.4 57 22 0 1 57 151 207 3.20E-12 79.7 6412 cellular organisms;Eukaryota;Opisthokonta;Metazoa;Eumetazoa;Bilateria;Protostomia;Spiralia;Lophotrochozoa;Annelida;Clitellata;Hirudinea;Rhynchobdellida;Glossiphoniidae;Helobdella;Helobdella robusta
UdeM00000029954 1735272 101.3 UdeM00000029954 XP_041370461.1 37 189 104 5 64 248 13 190 1.90E-18 101.3 1735272 cellular organisms;Eukaryota;Opisthokonta;Metazoa;Eumetazoa;Bilateria;Protostomia;Spiralia;Lophotrochozoa;Mollusca;Gastropoda;Neomphalina;Neomphaloidea;Peltospiridae;Gigantopelta;Gigantopelta aegis
UdeM00000029955 6596 97.8 UdeM00000029955 XP_045169739.1 38.1 155 78 1 33 187 5 141 2.20E-17 97.8 6596 cellular organisms;Eukaryota;Opisthokonta;Metazoa;Eumetazoa;Bilateria;Protostomia;Spiralia;Lophotrochozoa;Mollusca;Bivalvia;Autobranchia;Heteroconchia;Euheterodonta;Imparidentia;Venerida;Veneroidea;Veneridae;Mercenaria;Mercenaria mercenaria
UdeM00000029956 6850 67.8 UdeM00000029956 XP_013781880.1 37.5 80 50 0 2 81 218 297 7.80E-09 67.8 6850 cellular organisms;Eukaryota;Opisthokonta;Metazoa;Eumetazoa;Bilateria;Protostomia;Ecdysozoa;Panarthropoda;Arthropoda;Chelicerata;Merostomata;Xiphosura;Limulidae;Limulus;Limulus polyphemus
UdeM00000029957 6850 68.6 UdeM00000029957 XP_013778022.1 39.5 76 46 0 3 78 138 213 5.30E-09 68.6 6850 cellular organisms;Eukaryota;Opisthokonta;Metazoa;Eumetazoa;Bilateria;Protostomia;Ecdysozoa;Panarthropoda;Arthropoda;Chelicerata;Merostomata;Xiphosura;Limulidae;Limulus;Limulus polyphemus
UdeM00000029960 6596 602.1 UdeM00000029960 XP_045197169.1 75.4 394 80 4 2 393 79 457 5.20E-169 602.1 6596 cellular organisms;Eukaryota;Opisthokonta;Metazoa;Eumetazoa;Bilateria;Protostomia;Spiralia;Lophotrochozoa;Mollusca;Bivalvia;Autobranchia;Heteroconchia;Euheterodonta;Imparidentia;Venerida;Veneroidea;Veneridae;Mercenaria;Mercenaria mercenaria

UdeM00000029961 6579 190.3 UdeM00000029961 XP_033739677.1 84.2 101
16 0 44 144 376 476 1.70E-45 190.3 6579 cellular
organisms;Eukaryota;Opisthokonta;Metazoa;Eumetazoa;Bilateria;Protostomia;
Spiralia;Lophotrochozoa;Mollusca;Bivalvia;Autobranchia;Pteriomorphia;Pect
inida;Pectinoidea;Pectinidae;Pecten;Pecten maximus
UdeM00000029962 6579 654.8 UdeM00000029962 XP_033739684.1 65.8 570
160 15 1 551 1 554 9.50E-185 654.8 6579 cellular
organisms;Eukaryota;Opisthokonta;Metazoa;Eumetazoa;Bilateria;Protostomia;
Spiralia;Lophotrochozoa;Mollusca;Bivalvia;Autobranchia;Pteriomorphia;Pect
inida;Pectinoidea;Pectinidae;Pecten;Pecten maximus
UdeM00000029963 6596 362.1 UdeM00000029963 XP_045199074.1 62.2 270
101 1 1 269 1 270 6.20E-97 362.1 6596 cellular
organisms;Eukaryota;Opisthokonta;Metazoa;Eumetazoa;Bilateria;Protostomia;
Spiralia;Lophotrochozoa;Mollusca;Bivalvia;Autobranchia;Heteroconchia;Euhe
terodonta;Imparidentia;Venerida;Veneroidea;Veneridae;Mercenaria;Mercenari
a mercenaria
UdeM00000029964 6579 634 UdeM00000029964 XP_033741164.1 78.1 401
81 4 11 405 61 460 1.30E-178 634 6579 cellular
organisms;Eukaryota;Opisthokonta;Metazoa;Eumetazoa;Bilateria;Protostomia;
Spiralia;Lophotrochozoa;Mollusca;Bivalvia;Autobranchia;Pteriomorphia;Pect
inida;Pectinoidea;Pectinidae;Pecten;Pecten maximus
UdeM00000029966 225164 60.5 UdeM00000029966 XP_009062033.1 40.4
89 43 4 22 105 83 166 1.70E-06 60.5 225164
cellular
organisms;Eukaryota;Opisthokonta;Metazoa;Eumetazoa;Bilateria;Protostomia;
Spiralia;Lophotrochozoa;Mollusca;Gastropoda;Patellogastropoda;Lottioidea;
Lottiidae;Lottia;Lottia gigantea
UdeM00000029968 6596 316.2 UdeM00000029968 XP_045169256.1 31.3 633
393 14 1 620 96 699 9.20E-83 316.2 6596 cellular
organisms;Eukaryota;Opisthokonta;Metazoa;Eumetazoa;Bilateria;Protostomia;
Spiralia;Lophotrochozoa;Mollusca;Bivalvia;Autobranchia;Heteroconchia;Euhe
terodonta;Imparidentia;Venerida;Veneroidea;Veneridae;Mercenaria;Mercenari
a mercenaria
UdeM00000029969 225164 73.9 UdeM00000029969 XP_009063181.1 32.7
205 109 4 1 182 1 199 2.40E-10 73.9 225164
cellular
organisms;Eukaryota;Opisthokonta;Metazoa;Eumetazoa;Bilateria;Protostomia;
Spiralia;Lophotrochozoa;Mollusca;Gastropoda;Patellogastropoda;Lottioidea;
Lottiidae;Lottia;Lottia gigantea
UdeM00000029970 8218 107.1 UdeM00000029970 XP_034071052.1 46.7 105
55 1 3 106 137 241 1.50E-20 107.1 8218 cellular
organisms;Eukaryota;Opisthokonta;Metazoa;Eumetazoa;Bilateria;Deuterostomi
a;Chordata;Craniata;Vertebrata;Gnathostomata;Teleostomi;Euteleostomi;Acti
nopterygii;Actinopteri;Neopterygii;Teleostei;Osteoglossocephalai;Clupeoce
phala;Euteleosteomorpha;Neoteleostei;Eurypterygia;Ctenosquamata;Acanthomo
rphata;Euacanthomorphacea;Percomorphaceae;Eupercaria;Perciformes;Notothen
ioidei;Bathydraconidae;Gymnodraco;Gymnodraco acuticeps
UdeM00000029971 6596 65.9 UdeM00000029971 XP_045211214.1 27.3 172
118 2 35 203 529 696 7.40E-08 65.9 6596 cellular
organisms;Eukaryota;Opisthokonta;Metazoa;Eumetazoa;Bilateria;Protostomia;
Spiralia;Lophotrochozoa;Mollusca;Bivalvia;Autobranchia;Heteroconchia;Euhe
terodonta;Imparidentia;Venerida;Veneroidea;Veneridae;Mercenaria;Mercenari
a mercenaria

UdeM00000029972 6596 595.9 UdeM00000029972 XP_045198007.1 81.2 346
65 0 29 374 28 373 3.60E-167 595.9 6596 cellular
organisms;Eukaryota;Opisthokonta;Metazoa;Eumetazoa;Bilateria;Protostomia;
Spiralia;Lophotrochozoa;Mollusca;Bivalvia;Autobranchia;Heteroconchia;Euhe
terodonta;Imparidentia;Venerida;Veneroidea;Veneridae;Mercenaria;Mercenari
a mercenaria
UdeM00000029974 6573 115.5 UdeM00000029974 XP_021362122.1 36.6 183
88 3 3 157 2 184 7.70E-23 115.5 6573 cellular
organisms;Eukaryota;Opisthokonta;Metazoa;Eumetazoa;Bilateria;Protostomia;
Spiralia;Lophotrochozoa;Mollusca;Bivalvia;Autobranchia;Pteriomorphia;Pect
inida;Pectinoidea;Pectinidae;Mizuhopecten;Mizuhopecten yessoensis
UdeM00000029975 225164 540.8 UdeM00000029975 XP_009055944.1 51
576 215 8 1 563 1 522 2.10E-150 540.8 225164
cellular
organisms;Eukaryota;Opisthokonta;Metazoa;Eumetazoa;Bilateria;Protostomia;
Spiralia;Lophotrochozoa;Mollusca;Gastropoda;Patellogastropoda;Lottioidea;
Lottiidae;Lottia;Lottia gigantea
UdeM00000029976 10224 114 UdeM00000029976 XP_006811529.1 49.1 116
50 2 59 165 5 120 1.80E-22 114 10224 cellular
organisms;Eukaryota;Opisthokonta;Metazoa;Eumetazoa;Bilateria;Deuterostomi
a;Hemichordata;Enteropneusta;Harrimaniidae;Saccoglossus;Saccoglossus
kowalevskii
UdeM00000029977 2607531 455.7 UdeM00000029977 XP_029645990.1 60.5
702 226 8 1 654 1 699 2.00E-124 455.7 2607531
cellular
organisms;Eukaryota;Opisthokonta;Metazoa;Eumetazoa;Bilateria;Protostomia;
Spiralia;Lophotrochozoa;Mollusca;Cephalopoda;Coleoidea;Octopodiformes;Oct
opoda;Incirrata;Octopodidae;Octopus;Octopus sinensis
UdeM00000029978 6579 493.8 UdeM00000029978 XP_033740915.1 61.2 358
135 3 101 454 110 467 2.30E-136 493.8 6579 cellular
organisms;Eukaryota;Opisthokonta;Metazoa;Eumetazoa;Bilateria;Protostomia;
Spiralia;Lophotrochozoa;Mollusca;Bivalvia;Autobranchia;Pteriomorphia;Pect
inida;Pectinoidea;Pectinidae;Pecten;Pecten maximus
UdeM00000029979 6579 493.8 UdeM00000029979 XP_033740915.1 61.2 358
135 3 87 440 110 467 2.30E-136 493.8 6579 cellular
organisms;Eukaryota;Opisthokonta;Metazoa;Eumetazoa;Bilateria;Protostomia;
Spiralia;Lophotrochozoa;Mollusca;Bivalvia;Autobranchia;Pteriomorphia;Pect
inida;Pectinoidea;Pectinidae;Pecten;Pecten maximus
UdeM00000029980 6596 214.2 UdeM00000029980 XP_045204166.1 35.3 300
190 4 22 321 43 338 2.60E-52 214.2 6596 cellular
organisms;Eukaryota;Opisthokonta;Metazoa;Eumetazoa;Bilateria;Protostomia;
Spiralia;Lophotrochozoa;Mollusca;Bivalvia;Autobranchia;Heteroconchia;Euhe
terodonta;Imparidentia;Venerida;Veneroidea;Veneridae;Mercenaria;Mercenari
a mercenaria
UdeM00000029982 6596 216.1 UdeM00000029982 XP_045204166.1 34.1 337
214 6 18 354 10 338 7.50E-53 216.1 6596 cellular
organisms;Eukaryota;Opisthokonta;Metazoa;Eumetazoa;Bilateria;Protostomia;
Spiralia;Lophotrochozoa;Mollusca;Bivalvia;Autobranchia;Heteroconchia;Euhe
terodonta;Imparidentia;Venerida;Veneroidea;Veneridae;Mercenaria;Mercenari
a mercenaria
UdeM00000029985 6596 100.5 UdeM00000029985 XP_045204166.1 32.2 152
101 2 18 169 189 338 2.30E-18 100.5 6596 cellular
organisms;Eukaryota;Opisthokonta;Metazoa;Eumetazoa;Bilateria;Protostomia;
Spiralia;Lophotrochozoa;Mollusca;Bivalvia;Autobranchia;Heteroconchia;Euhe

terodonta;Imparidentia;Venerida;Veneroidea;Veneridae;Mercenaria;Mercenaria mercenaria
UdeM00000029984 6596 120.6 UdeM00000029984 XP_045204166.1 33.3 201 128 4 21 221 144 338 2.70E-24 120.6 6596 cellular organisms;Eukaryota;Opisthokonta;Metazoa;Eumetazoa;Bilateria;Protostomia;Spiralia;Lophotrochozoa;Mollusca;Bivalvia;Autobranchia;Heteroconchia;Euheterodonta;Imparidentia;Venerida;Veneroidea;Veneridae;Mercenaria;Mercenaria mercenaria
UdeM00000029988 6596 224.2 UdeM00000029988 XP_045204166.1 36.4 302 188 4 43 344 32 329 2.70E-55 224.2 6596 cellular organisms;Eukaryota;Opisthokonta;Metazoa;Eumetazoa;Bilateria;Protostomia;Spiralia;Lophotrochozoa;Mollusca;Bivalvia;Autobranchia;Heteroconchia;Euheterodonta;Imparidentia;Venerida;Veneroidea;Veneridae;Mercenaria;Mercenaria mercenaria
UdeM00000029991 6596 225.7 UdeM00000029991 XP_045204166.1 35.6 334 202 6 18 351 16 336 9.40E-56 225.7 6596 cellular organisms;Eukaryota;Opisthokonta;Metazoa;Eumetazoa;Bilateria;Protostomia;Spiralia;Lophotrochozoa;Mollusca;Bivalvia;Autobranchia;Heteroconchia;Euheterodonta;Imparidentia;Venerida;Veneroidea;Veneridae;Mercenaria;Mercenaria mercenaria
UdeM00000029992 6596 225.3 UdeM00000029992 XP_045204166.1 33.2 316 184 3 42 357 29 317 1.30E-55 225.3 6596 cellular organisms;Eukaryota;Opisthokonta;Metazoa;Eumetazoa;Bilateria;Protostomia;Spiralia;Lophotrochozoa;Mollusca;Bivalvia;Autobranchia;Heteroconchia;Euheterodonta;Imparidentia;Venerida;Veneroidea;Veneridae;Mercenaria;Mercenaria mercenaria
UdeM00000029993 1735272 246.9 UdeM00000029993 XP_041354081.1 46.4 461 217 11 1 450 116 557 4.90E-62 246.9 1735272 cellular organisms;Eukaryota;Opisthokonta;Metazoa;Eumetazoa;Bilateria;Protostomia;Spiralia;Lophotrochozoa;Mollusca;Gastropoda;Neomphalina;Neomphaloidea;Peltospiridae;Gigantopelta;Gigantopelta aegis
UdeM00000029994 1735272 278.5 UdeM00000029994 XP_041354081.1 46.3 525 245 16 1 514 59 557 1.70E-71 278.5 1735272 cellular organisms;Eukaryota;Opisthokonta;Metazoa;Eumetazoa;Bilateria;Protostomia;Spiralia;Lophotrochozoa;Mollusca;Gastropoda;Neomphalina;Neomphaloidea;Peltospiridae;Gigantopelta;Gigantopelta aegis
UdeM00000029995 7574 186 UdeM00000029995 XP_013384628.1 59 161 57 2 1 161 33 184 3.80E-44 186 7574 cellular organisms;Eukaryota;Opisthokonta;Metazoa;Eumetazoa;Bilateria;Protostomia;Spiralia;Lophotrochozoa;Brachiopoda;Linguliformea;Lingulata;Lingulida;Linguloidea;Lingulidae;Lingula;Lingula anatina
UdeM00000029996 6596 6917.4 UdeM00000029996 XP_045195814.1 81.2 4213 780 6 1 4206 1 4208 0.00E+00 6917.4 6596 cellular organisms;Eukaryota;Opisthokonta;Metazoa;Eumetazoa;Bilateria;Protostomia;Spiralia;Lophotrochozoa;Mollusca;Bivalvia;Autobranchia;Heteroconchia;Euheterodonta;Imparidentia;Venerida;Veneroidea;Veneridae;Mercenaria;Mercenaria mercenaria
UdeM00000029997 6596 6714.4 UdeM00000029997 XP_045195814.1 81.1 4106 762 7 1 4099 1 4100 0.00E+00 6714.4 6596 cellular organisms;Eukaryota;Opisthokonta;Metazoa;Eumetazoa;Bilateria;Protostomia;

Spiralia;Lophotrochozoa;Mollusca;Bivalvia;Autobranchia;Heteroconchia;Euheterodonta;Imparidentia;Venerida;Veneroidea;Veneridae;Mercenaria;Mercenaria mercenaria
UdeM00000029998 6596 198.4 UdeM00000029998 XP_045197627.1 34.4 393 245 8 1 387 10 395 2.00E-47 198.4 6596 cellular organisms;Eukaryota;Opisthokonta;Metazoa;Eumetazoa;Bilateria;Protostomia;Spiralia;Lophotrochozoa;Mollusca;Bivalvia;Autobranchia;Heteroconchia;Euheterodonta;Imparidentia;Venerida;Veneroidea;Veneridae;Mercenaria;Mercenaria mercenaria
UdeM00000029999 6596 132.5 UdeM00000029999 XP_045196362.1 31.6 351 207 14 57 391 48 381 1.30E-27 132.5 6596 cellular organisms;Eukaryota;Opisthokonta;Metazoa;Eumetazoa;Bilateria;Protostomia;Spiralia;Lophotrochozoa;Mollusca;Bivalvia;Autobranchia;Heteroconchia;Euheterodonta;Imparidentia;Venerida;Veneroidea;Veneridae;Mercenaria;Mercenaria mercenaria
UdeM00000030000 29159 68.9 UdeM00000030000 XP_011438591.2 31.9 163 100 6 19 174 18 176 2.70E-08 68.9 29159 cellular organisms;Eukaryota;Opisthokonta;Metazoa;Eumetazoa;Bilateria;Protostomia;Spiralia;Lophotrochozoa;Mollusca;Bivalvia;Autobranchia;Pteriomorphia;Ostreida;Ostreoidea;Ostreidae;Crassostrea;Crassostrea gigas
UdeM00000030001 6573 124.8 UdeM00000030001 XP_021345507.1 47.4 135 67 2 4 138 5 135 8.60E-26 124.8 6573 cellular organisms;Eukaryota;Opisthokonta;Metazoa;Eumetazoa;Bilateria;Protostomia;Spiralia;Lophotrochozoa;Mollusca;Bivalvia;Autobranchia;Pteriomorphia;Pectinida;Pectinoidea;Pectinidae;Mizuhopecten;Mizuhopecten yessoensis
UdeM00000030002 6596 103.2 UdeM00000030002 XP_045169370.1 38.6 153 83 4 4 154 15 158 3.00E-19 103.2 6596 cellular organisms;Eukaryota;Opisthokonta;Metazoa;Eumetazoa;Bilateria;Protostomia;Spiralia;Lophotrochozoa;Mollusca;Bivalvia;Autobranchia;Heteroconchia;Euheterodonta;Imparidentia;Venerida;Veneroidea;Veneridae;Mercenaria;Mercenaria mercenaria
UdeM00000030003 6573 165.2 UdeM00000030003 XP_021347733.1 30.9 320 206 6 1 316 332 640 1.40E-37 165.2 6573 cellular organisms;Eukaryota;Opisthokonta;Metazoa;Eumetazoa;Bilateria;Protostomia;Spiralia;Lophotrochozoa;Mollusca;Bivalvia;Autobranchia;Pteriomorphia;Pectinida;Pectinoidea;Pectinidae;Mizuhopecten;Mizuhopecten yessoensis
UdeM00000030004 6579 440.7 UdeM00000030004 XP_033740238.1 37 646 386 8 20 653 20 656 3.40E-120 440.7 6579 cellular organisms;Eukaryota;Opisthokonta;Metazoa;Eumetazoa;Bilateria;Protostomia;Spiralia;Lophotrochozoa;Mollusca;Bivalvia;Autobranchia;Pteriomorphia;Pectinida;Pectinoidea;Pectinidae;Pecten;Pecten maximus
UdeM00000030005 6573 439.9 UdeM00000030005 XP_021347733.1 36.8 639 384 8 7 636 5 632 5.70E-120 439.9 6573 cellular organisms;Eukaryota;Opisthokonta;Metazoa;Eumetazoa;Bilateria;Protostomia;Spiralia;Lophotrochozoa;Mollusca;Bivalvia;Autobranchia;Pteriomorphia;Pectinida;Pectinoidea;Pectinidae;Mizuhopecten;Mizuhopecten yessoensis
UdeM00000030006 6596 261.2 UdeM00000030006 XP_045169370.1 44.4 295 151 5 18 310 27 310 1.80E-66 261.2 6596 cellular organisms;Eukaryota;Opisthokonta;Metazoa;Eumetazoa;Bilateria;Protostomia;Spiralia;Lophotrochozoa;Mollusca;Bivalvia;Autobranchia;Heteroconchia;Euheterodonta;Imparidentia;Venerida;Veneroidea;Veneridae;Mercenaria;Mercenaria mercenaria
UdeM00000030008 6573 441.8 UdeM00000030008 XP_021347733.1 36.1 640 387 8 7 636 5 632 1.50E-120 441.8 6573 cellular

organisms;Eukaryota;Opisthokonta;Metazoa;Eumetazoa;Bilateria;Protostomia;
Spiralia;Lophotrochozoa;Mollusca;Bivalvia;Autobranchia;Pteriomorphia;Pect
inida;Pectinoidea;Pectinidae;Mizuhopecten;Mizuhopecten yessoensis
UdeM00000030009 6596 956.1 UdeM00000030009 XP_045197367.1 49.5 1058
440 18 3 1030 2 995 3.80E-275 956.1 6596 cellular
organisms;Eukaryota;Opisthokonta;Metazoa;Eumetazoa;Bilateria;Protostomia;
Spiralia;Lophotrochozoa;Mollusca;Bivalvia;Autobranchia;Heteroconchia;Euhe
terodonta;Imparidentia;Venerida;Veneroidea;Veneridae;Mercenaria;Mercenari
a mercenaria
UdeM00000030010 6596 955.3 UdeM00000030010 XP_045197367.1 49.6 1058
445 18 3 1036 2 995 6.40E-275 955.3 6596 cellular
organisms;Eukaryota;Opisthokonta;Metazoa;Eumetazoa;Bilateria;Protostomia;
Spiralia;Lophotrochozoa;Mollusca;Bivalvia;Autobranchia;Heteroconchia;Euhe
terodonta;Imparidentia;Venerida;Veneroidea;Veneridae;Mercenaria;Mercenari
a mercenaria
UdeM00000030011 6579 278.1 UdeM00000030011 XP_033741736.1 54.9 244
108 1 1 242 614 857 1.10E-71 278.1 6579 cellular
organisms;Eukaryota;Opisthokonta;Metazoa;Eumetazoa;Bilateria;Protostomia;
Spiralia;Lophotrochozoa;Mollusca;Bivalvia;Autobranchia;Pteriomorphia;Pect
inida;Pectinoidea;Pectinidae;Pecten;Pecten maximus
UdeM00000030012 109280 110.5 UdeM00000030012 XP_019729826.1 32.1
212 105 3 3 175 7 218 2.20E-21 110.5 109280
cellular
organisms;Eukaryota;Opisthokonta;Metazoa;Eumetazoa;Bilateria;Deuterostomi
a;Chordata;Craniata;Vertebrata;Gnathostomata;Teleostomi;Euteleostomi;Acti
nopterygii;Actinopteri;Neopterygii;Teleostei;Osteoglossocephalai;Clupeoce
phala;Euteleosteomorpha;Neoteleostei;Eurypterygia;Ctenosquamata;Acanthomo
rphata;Euacanthomorphacea;Percomorphaceae;Syngnathiaria;Syngnathiformes;S
yngnathoidei;Syngnathidae;Hippocampinae;Hippocampus;Hippocampus comes
UdeM00000030014 6526 71.2 UdeM00000030014 XP_013078803.1 37.6 109
60 3 1 109 18 118 1.00E-09 71.2 6526 cellular
organisms;Eukaryota;Opisthokonta;Metazoa;Eumetazoa;Bilateria;Protostomia;
Spiralia;Lophotrochozoa;Mollusca;Gastropoda;Heterobranchia;Euthyneura;Pan
pulmonata;Hygrophila;Lymnaeoidea;Planorbidae;Biomphalaria;Biomphalaria
glabrata
UdeM00000030015 70779 135.6 UdeM00000030015 XP_015755194.1 68.4 98
30 1 5 101 52 149 3.60E-29 135.6 70779 cellular
organisms;Eukaryota;Opisthokonta;Metazoa;Eumetazoa;Cnidaria;Anthozoa;Hexa
corallia;Scleractinia;Astrocoeniina;Acroporidae;Acropora;Acropora
digitifera
UdeM00000030016 70779 135.2 UdeM00000030016 XP_015755194.1 68.4 98
30 1 16 112 52 149 5.20E-29 135.2 70779 cellular
organisms;Eukaryota;Opisthokonta;Metazoa;Eumetazoa;Cnidaria;Anthozoa;Hexa
corallia;Scleractinia;Astrocoeniina;Acroporidae;Acropora;Acropora
digitifera
UdeM00000030017 6596 70.9 UdeM00000030017 XP_045197368.1 34.1 129
82 3 110 237 434 560 3.40E-09 70.9 6596 cellular
organisms;Eukaryota;Opisthokonta;Metazoa;Eumetazoa;Bilateria;Protostomia;
Spiralia;Lophotrochozoa;Mollusca;Bivalvia;Autobranchia;Heteroconchia;Euhe
terodonta;Imparidentia;Venerida;Veneroidea;Veneridae;Mercenaria;Mercenari
a mercenaria
UdeM00000030019 7574 83.6 UdeM00000030019 XP_013413319.1 33.6 125
70 4 3 127 522 633 6.00E-13 83.6 7574 cellular
organisms;Eukaryota;Opisthokonta;Metazoa;Eumetazoa;Bilateria;Protostomia;

Spiralia;Lophotrochozoa;Brachiopoda;Linguliformea;Lingulata;Lingulida;Linguloidea;Lingulidae;Lingula;Lingula anatina
UdeM00000030020 6596 273.9 UdeM00000030020 XP_045168024.1 46.1 308
152 4 40 346 13 307 3.00E-70 273.9 6596 cellular organisms;Eukaryota;Opisthokonta;Metazoa;Eumetazoa;Bilateria;Protostomia;Spiralia;Lophotrochozoa;Mollusca;Bivalvia;Autobranchia;Heteroconchia;Euheterodonta;Imparidentia;Venerida;Veneroidea;Veneridae;Mercenaria;Mercenaria mercenaria
UdeM00000030021 6596 274.2 UdeM00000030021 XP_045168024.1 46.1 308
152 4 40 346 13 307 2.30E-70 274.2 6596 cellular organisms;Eukaryota;Opisthokonta;Metazoa;Eumetazoa;Bilateria;Protostomia;Spiralia;Lophotrochozoa;Mollusca;Bivalvia;Autobranchia;Heteroconchia;Euheterodonta;Imparidentia;Venerida;Veneroidea;Veneridae;Mercenaria;Mercenaria mercenaria
UdeM00000030022 6596 74.3 UdeM00000030022 XP_045197390.1 35.7 129
80 3 48 175 1 127 2.50E-10 74.3 6596 cellular organisms;Eukaryota;Opisthokonta;Metazoa;Eumetazoa;Bilateria;Protostomia;Spiralia;Lophotrochozoa;Mollusca;Bivalvia;Autobranchia;Heteroconchia;Euheterodonta;Imparidentia;Venerida;Veneroidea;Veneridae;Mercenaria;Mercenaria mercenaria
UdeM00000030023 70779 91.3 UdeM00000030023 XP_015755194.1 63.4 71
25 1 16 85 52 122 6.70E-16 91.3 70779 cellular organisms;Eukaryota;Opisthokonta;Metazoa;Eumetazoa;Cnidaria;Anthozoa;Hexacorallia;Scleractinia;Astrocoeniina;Acroporidae;Acropora;Acropora digitifera
UdeM00000030024 6596 67.8 UdeM00000030024 XP_045205019.1 29.9 167
110 6 73 237 221 382 2.80E-08 67.8 6596 cellular organisms;Eukaryota;Opisthokonta;Metazoa;Eumetazoa;Bilateria;Protostomia;Spiralia;Lophotrochozoa;Mollusca;Bivalvia;Autobranchia;Heteroconchia;Euheterodonta;Imparidentia;Venerida;Veneroidea;Veneridae;Mercenaria;Mercenaria mercenaria
UdeM00000030025 70779 177.2 UdeM00000030025 XP_015755194.1 61.5 148
53 2 4 147 2 149 1.60E-41 177.2 70779 cellular organisms;Eukaryota;Opisthokonta;Metazoa;Eumetazoa;Cnidaria;Anthozoa;Hexacorallia;Scleractinia;Astrocoeniina;Acroporidae;Acropora;Acropora digitifera
UdeM00000030027 6596 394.8 UdeM00000030027 XP_045169235.1 38.6 596
316 15 20 603 22 579 2.00E-106 394.8 6596 cellular organisms;Eukaryota;Opisthokonta;Metazoa;Eumetazoa;Bilateria;Protostomia;Spiralia;Lophotrochozoa;Mollusca;Bivalvia;Autobranchia;Heteroconchia;Euheterodonta;Imparidentia;Venerida;Veneroidea;Veneridae;Mercenaria;Mercenaria mercenaria
UdeM00000030029 6596 393.3 UdeM00000030029 XP_045195510.1 38.2 597
330 11 2 588 5 572 5.70E-106 393.3 6596 cellular organisms;Eukaryota;Opisthokonta;Metazoa;Eumetazoa;Bilateria;Protostomia;Spiralia;Lophotrochozoa;Mollusca;Bivalvia;Autobranchia;Heteroconchia;Euheterodonta;Imparidentia;Venerida;Veneroidea;Veneridae;Mercenaria;Mercenaria mercenaria
UdeM00000030030 29159 2068.1 UdeM00000030030 XP_034333968.1 87.5
1139 137 1 1 1134 1 1139 0.00E+00 2068.1
29159 cellular organisms;Eukaryota;Opisthokonta;Metazoa;Eumetazoa;Bilateria;Protostomia;Spiralia;Lophotrochozoa;Mollusca;Bivalvia;Autobranchia;Pteriomorphia;Ostreida;Ostreoidea;Ostreidae;Crassostrea;Crassostrea gigas

UdeM00000030031 6596 2113.2 UdeM00000030031 XP_045198448.1 58.4
2517 936 12 1 2503 1 2419 0.00E+00 2113.2 6596
cellular
organisms;Eukaryota;Opisthokonta;Metazoa;Eumetazoa;Bilateria;Protostomia;
Spiralia;Lophotrochozoa;Mollusca;Bivalvia;Autobranchia;Heteroconchia;Euheterodonta;Imparidentia;Venerida;Veneroidea;Veneridae;Mercenaria;Mercenaria mercenaria
UdeM00000030032 1735272 232.3 UdeM00000030032 XP_041354771.1 40.7
290 172 0 1 290 175 464 8.10E-58 232.3 1735272
cellular
organisms;Eukaryota;Opisthokonta;Metazoa;Eumetazoa;Bilateria;Protostomia;
Spiralia;Lophotrochozoa;Mollusca;Gastropoda;Neomphalina;Neomphaloidea;Peltospiridae;Gigantopelta;Gigantopelta aegis
UdeM00000030033 1735272 231.9 UdeM00000030033 XP_041354771.1 41.1
287 169 0 1 287 178 464 1.10E-57 231.9 1735272
cellular
organisms;Eukaryota;Opisthokonta;Metazoa;Eumetazoa;Bilateria;Protostomia;
Spiralia;Lophotrochozoa;Mollusca;Gastropoda;Neomphalina;Neomphaloidea;Peltospiridae;Gigantopelta;Gigantopelta aegis
UdeM00000030034 29159 879.8 UdeM00000030034 XP_019925622.2 85 533
77 1 1 533 1 530 1.80E-252 879.8 29159 cellular
organisms;Eukaryota;Opisthokonta;Metazoa;Eumetazoa;Bilateria;Protostomia;
Spiralia;Lophotrochozoa;Mollusca;Bivalvia;Autobranchia;Pteriomorphia;Ostreida;Ostreoidea;Ostreidae;Crassostrea;Crassostrea gigas
UdeM00000030035 400727 193.7 UdeM00000030035 XP_025098134.1 54.7
161 72 1 7 166 5 165 1.80E-46 193.7 400727
cellular
organisms;Eukaryota;Opisthokonta;Metazoa;Eumetazoa;Bilateria;Protostomia;
Spiralia;Lophotrochozoa;Mollusca;Gastropoda;Caenogastropoda;Architaenioglossa;Ampullarioidea;Ampullariidae;Pomacea;Pomacea canaliculata
UdeM00000030036 6596 789.6 UdeM00000030036 XP_045198229.1 76 508
111 3 11 512 63 565 2.30E-225 789.6 6596 cellular
organisms;Eukaryota;Opisthokonta;Metazoa;Eumetazoa;Bilateria;Protostomia;
Spiralia;Lophotrochozoa;Mollusca;Bivalvia;Autobranchia;Heteroconchia;Euheterodonta;Imparidentia;Venerida;Veneroidea;Veneridae;Mercenaria;Mercenaria mercenaria
UdeM00000030037 6596 78.2 UdeM00000030037 XP_045158584.1 44.2 95
50 2 15 109 50 141 8.90E-12 78.2 6596 cellular
organisms;Eukaryota;Opisthokonta;Metazoa;Eumetazoa;Bilateria;Protostomia;
Spiralia;Lophotrochozoa;Mollusca;Bivalvia;Autobranchia;Heteroconchia;Euheterodonta;Imparidentia;Venerida;Veneroidea;Veneridae;Mercenaria;Mercenaria mercenaria
UdeM00000030038 6573 152.1 UdeM00000030038 XP_021357585.1 56.2 128
54 2 26 151 3 130 9.40E-34 152.1 6573 cellular
organisms;Eukaryota;Opisthokonta;Metazoa;Eumetazoa;Bilateria;Protostomia;
Spiralia;Lophotrochozoa;Mollusca;Bivalvia;Autobranchia;Pteriomorphia;Pectinida;Pectinoidea;Pectinidae;Mizuhopecten;Mizuhopecten yessoensis
UdeM00000030040 6579 469.2 UdeM00000030040 XP_033740037.1 36.9 821
456 14 11 811 1473 2251 1.20E-128 469.2 6579 cellular
organisms;Eukaryota;Opisthokonta;Metazoa;Eumetazoa;Bilateria;Protostomia;
Spiralia;Lophotrochozoa;Mollusca;Bivalvia;Autobranchia;Pteriomorphia;Pectinida;Pectinoidea;Pectinidae;Pecten;Pecten maximus
UdeM00000030041 6573 438.7 UdeM00000030041 XP_021359425.1 27.9 1217
761 24 4 1116 253 1456 2.20E-119 438.7 6573 cellular

organisms;Eukaryota;Opisthokonta;Metazoa;Eumetazoa;Bilateria;Protostomia;Spiralia;Lophotrochozoa;Mollusca;Bivalvia;Autobranchia;Pteriomorphia;Pectinida;Pectinoidea;Pectinidae;Mizuhopecten;Mizuhopecten yessoensis
UdeM00000030042 400727 257.7 UdeM00000030042 XP_025099514.1 78.8 198 41 1 1 197 3 200 1.20E-65 257.7 400727 cellular organisms;Eukaryota;Opisthokonta;Metazoa;Eumetazoa;Bilateria;Protostomia;Spiralia;Lophotrochozoa;Mollusca;Gastropoda;Caenogastropoda;Architaenioglossa;Ampullarioidea;Ampullariidae;Pomacea;Pomacea canaliculata
UdeM00000030043 6579 370.5 UdeM00000030043 XP_033740146.1 39.3 552 284 9 2 505 3 551 3.30E-99 370.5 6579 cellular organisms;Eukaryota;Opisthokonta;Metazoa;Eumetazoa;Bilateria;Protostomia;Spiralia;Lophotrochozoa;Mollusca;Bivalvia;Autobranchia;Pteriomorphia;Pectinida;Pectinoidea;Pectinidae;Pecten;Pecten maximus
UdeM00000030044 6565 72.8 UdeM00000030044 XP_022290295.1 29.6 199 130 5 369 562 5 198 2.70E-09 72.8 6565 cellular organisms;Eukaryota;Opisthokonta;Metazoa;Eumetazoa;Bilateria;Protostomia;Spiralia;Lophotrochozoa;Mollusca;Bivalvia;Autobranchia;Pteriomorphia;Ostreida;Ostreoidea;Ostreidae;Crassostrea;Crassostrea virginica
UdeM00000030045 29159 420.2 UdeM00000030045 XP_011418174.2 40.9 580 301 9 3 541 4 582 3.90E-114 420.2 29159 cellular organisms;Eukaryota;Opisthokonta;Metazoa;Eumetazoa;Bilateria;Protostomia;Spiralia;Lophotrochozoa;Mollusca;Bivalvia;Autobranchia;Pteriomorphia;Ostreida;Ostreoidea;Ostreidae;Crassostrea;Crassostrea gigas
UdeM00000030046 1735272 287.3 UdeM00000030046 XP_041373141.1 36.6 434 252 7 7 425 12 437 3.30E-74 287.3 1735272 cellular organisms;Eukaryota;Opisthokonta;Metazoa;Eumetazoa;Bilateria;Protostomia;Spiralia;Lophotrochozoa;Mollusca;Gastropoda;Neomphalina;Neomphaloidea;Peltospiridae;Gigantopelta;Gigantopelta aegis
UdeM00000030047 6573 388.7 UdeM00000030047 XP_021349278.1 41.7 636 299 17 45 642 59 660 1.50E-104 388.7 6573 cellular organisms;Eukaryota;Opisthokonta;Metazoa;Eumetazoa;Bilateria;Protostomia;Spiralia;Lophotrochozoa;Mollusca;Bivalvia;Autobranchia;Pteriomorphia;Pectinida;Pectinoidea;Pectinidae;Mizuhopecten;Mizuhopecten yessoensis
UdeM00000030048 6579 365.2 UdeM00000030048 XP_033740146.1 38.8 551 277 10 2 494 3 551 1.40E-97 365.2 6579 cellular organisms;Eukaryota;Opisthokonta;Metazoa;Eumetazoa;Bilateria;Protostomia;Spiralia;Lophotrochozoa;Mollusca;Bivalvia;Autobranchia;Pteriomorphia;Pectinida;Pectinoidea;Pectinidae;Pecten;Pecten maximus
UdeM00000030049 6579 429.5 UdeM00000030049 XP_033740146.1 42.7 553 305 7 1 543 1 551 6.40E-117 429.5 6579 cellular organisms;Eukaryota;Opisthokonta;Metazoa;Eumetazoa;Bilateria;Protostomia;Spiralia;Lophotrochozoa;Mollusca;Bivalvia;Autobranchia;Pteriomorphia;Pectinida;Pectinoidea;Pectinidae;Pecten;Pecten maximus
UdeM00000030050 6596 395.6 UdeM00000030050 XP_045165662.1 61 310 116 3 3 312 8 312 5.90E-107 395.6 6596 cellular organisms;Eukaryota;Opisthokonta;Metazoa;Eumetazoa;Bilateria;Protostomia;Spiralia;Lophotrochozoa;Mollusca;Bivalvia;Autobranchia;Heteroconchia;Euheterodonta;Imparidentia;Venerida;Veneroidea;Veneridae;Mercenaria;Mercenaria mercenaria
UdeM00000030053 7574 376.7 UdeM00000030053 XP_013397625.1 46.8 387 199 4 4 386 5 388 3.50E-101 376.7 7574 cellular organisms;Eukaryota;Opisthokonta;Metazoa;Eumetazoa;Bilateria;Protostomia;

Spiralia;Lophotrochozoa;Brachiopoda;Linguliformea;Lingulata;Lingulida;Linguloidea;Lingulidae;Lingula;Lingula anatina
UdeM00000030051 7574 379.8 UdeM00000030051 XP_013397625.1 45.9 401 207 5 4 397 5 402 4.90E-102 379.8 7574 cellular organisms;Eukaryota;Opisthokonta;Metazoa;Eumetazoa;Bilateria;Protostomia;Spiralia;Lophotrochozoa;Brachiopoda;Linguliformea;Lingulata;Lingulida;Linguloidea;Lingulidae;Lingula;Lingula anatina
UdeM00000030052 7574 571.2 UdeM00000030052 XP_013397625.1 48.7 579 289 5 4 577 5 580 1.50E-159 571.2 7574 cellular organisms;Eukaryota;Opisthokonta;Metazoa;Eumetazoa;Bilateria;Protostomia;Spiralia;Lophotrochozoa;Brachiopoda;Linguliformea;Lingulata;Lingulida;Linguloidea;Lingulidae;Lingula;Lingula anatina
UdeM00000030054 7574 205.7 UdeM00000030054 XP_023933057.1 50.2 201 99 1 20 219 185 385 6.20E-50 205.7 7574 cellular organisms;Eukaryota;Opisthokonta;Metazoa;Eumetazoa;Bilateria;Protostomia;Spiralia;Lophotrochozoa;Brachiopoda;Linguliformea;Lingulata;Lingulida;Linguloidea;Lingulidae;Lingula;Lingula anatina
UdeM00000030056 7574 434.5 UdeM00000030056 XP_013397625.1 47.9 447 227 3 4 447 5 448 1.70E-118 434.5 7574 cellular organisms;Eukaryota;Opisthokonta;Metazoa;Eumetazoa;Bilateria;Protostomia;Spiralia;Lophotrochozoa;Brachiopoda;Linguliformea;Lingulata;Lingulida;Linguloidea;Lingulidae;Lingula;Lingula anatina
UdeM00000030055 7574 545.4 UdeM00000030055 XP_013397625.1 47.3 584 300 5 4 582 5 585 8.60E-152 545.4 7574 cellular organisms;Eukaryota;Opisthokonta;Metazoa;Eumetazoa;Bilateria;Protostomia;Spiralia;Lophotrochozoa;Brachiopoda;Linguliformea;Lingulata;Lingulida;Linguloidea;Lingulidae;Lingula;Lingula anatina
UdeM00000030057 7574 109.4 UdeM00000030057 XP_013391382.1 45.6 125 67 1 1 124 459 583 3.40E-21 109.4 7574 cellular organisms;Eukaryota;Opisthokonta;Metazoa;Eumetazoa;Bilateria;Protostomia;Spiralia;Lophotrochozoa;Brachiopoda;Linguliformea;Lingulata;Lingulida;Linguloidea;Lingulidae;Lingula;Lingula anatina
UdeM00000030058 1735272 324.3 UdeM00000030058 XP_041351917.1 74.4 203 51 1 1 203 1 202 1.10E-85 324.3 1735272 cellular organisms;Eukaryota;Opisthokonta;Metazoa;Eumetazoa;Bilateria;Protostomia;Spiralia;Lophotrochozoa;Mollusca;Gastropoda;Neomphalina;Neomphaloidea;Peltospiridae;Gigantopelta;Gigantopelta aegis
UdeM00000030059 6596 184.5 UdeM00000030059 XP_045196404.1 35.1 276 170 5 160 430 518 789 2.90E-43 184.5 6596 cellular organisms;Eukaryota;Opisthokonta;Metazoa;Eumetazoa;Bilateria;Protostomia;Spiralia;Lophotrochozoa;Mollusca;Bivalvia;Autobranchia;Heteroconchia;Euheterodonta;Imparidentia;Venerida;Veneroidea;Veneridae;Mercenaria;Mercenaria mercenaria
UdeM00000030060 6596 183.7 UdeM00000030060 XP_045186528.1 35.9 276 148 4 162 437 415 661 4.90E-43 183.7 6596 cellular organisms;Eukaryota;Opisthokonta;Metazoa;Eumetazoa;Bilateria;Protostomia;Spiralia;Lophotrochozoa;Mollusca;Bivalvia;Autobranchia;Heteroconchia;Euheterodonta;Imparidentia;Venerida;Veneroidea;Veneridae;Mercenaria;Mercenaria mercenaria
UdeM00000030061 6596 362.1 UdeM00000030061 XP_045199078.1 35.1 596 338 14 8 580 10 579 1.50E-96 362.1 6596 cellular organisms;Eukaryota;Opisthokonta;Metazoa;Eumetazoa;Bilateria;Protostomia;Spiralia;Lophotrochozoa;Mollusca;Bivalvia;Autobranchia;Heteroconchia;Euhe

terodonta;Imparidentia;Venerida;Veneroidea;Veneridae;Mercenaria;Mercenaria mercenaria
UdeM00000030062 6596 186.4 UdeM00000030062 XP_045176508.1 36.6 273 149 5 159 430 249 498 7.50E-44 186.4 6596 cellular organisms;Eukaryota;Opisthokonta;Metazoa;Eumetazoa;Bilateria;Protostomia;Spiralia;Lophotrochozoa;Mollusca;Bivalvia;Autobranchia;Heteroconchia;Euheterodonta;Imparidentia;Venerida;Veneroidea;Veneridae;Mercenaria;Mercenaria mercenaria
UdeM00000030063 6579 689.5 UdeM00000030063 XP_033746880.1 52.1 674 295 7 1 646 815 1488 4.10E-195 689.5 6579 cellular organisms;Eukaryota;Opisthokonta;Metazoa;Eumetazoa;Bilateria;Protostomia;Spiralia;Lophotrochozoa;Mollusca;Bivalvia;Autobranchia;Pteriomorphia;Pectinida;Pectinoidea;Pectinidae;Pecten;Pecten maximus
UdeM00000030064 6565 158.7 UdeM00000030064 XP_022328252.1 44.1 211 100 5 1 193 1105 1315 7.50E-36 158.7 6565 cellular organisms;Eukaryota;Opisthokonta;Metazoa;Eumetazoa;Bilateria;Protostomia;Spiralia;Lophotrochozoa;Mollusca;Bivalvia;Autobranchia;Pteriomorphia;Ostreida;Ostreoidea;Ostreidae;Crassostrea;Crassostrea virginica
UdeM00000030066 6596 71.2 UdeM00000030066 XP_045215232.1 50 56 27 1 4 59 7 61 2.00E-09 71.2 6596 cellular organisms;Eukaryota;Opisthokonta;Metazoa;Eumetazoa;Bilateria;Protostomia;Spiralia;Lophotrochozoa;Mollusca;Bivalvia;Autobranchia;Heteroconchia;Euheterodonta;Imparidentia;Venerida;Veneroidea;Veneridae;Mercenaria;Mercenaria mercenaria
UdeM00000030065 6596 71.2 UdeM00000030065 XP_045215232.1 50 56 27 1 4 59 7 61 2.30E-09 71.2 6596 cellular organisms;Eukaryota;Opisthokonta;Metazoa;Eumetazoa;Bilateria;Protostomia;Spiralia;Lophotrochozoa;Mollusca;Bivalvia;Autobranchia;Heteroconchia;Euheterodonta;Imparidentia;Venerida;Veneroidea;Veneridae;Mercenaria;Mercenaria mercenaria
UdeM00000030067 6596 188 UdeM00000030067 XP_045176508.1 36.8 280 154 5 184 463 242 498 2.80E-44 188 6596 cellular organisms;Eukaryota;Opisthokonta;Metazoa;Eumetazoa;Bilateria;Protostomia;Spiralia;Lophotrochozoa;Mollusca;Bivalvia;Autobranchia;Heteroconchia;Euheterodonta;Imparidentia;Venerida;Veneroidea;Veneridae;Mercenaria;Mercenaria mercenaria
UdeM00000030068 6596 188.3 UdeM00000030068 XP_045176508.1 36.9 279 154 5 184 462 242 498 2.10E-44 188.3 6596 cellular organisms;Eukaryota;Opisthokonta;Metazoa;Eumetazoa;Bilateria;Protostomia;Spiralia;Lophotrochozoa;Mollusca;Bivalvia;Autobranchia;Heteroconchia;Euheterodonta;Imparidentia;Venerida;Veneroidea;Veneridae;Mercenaria;Mercenaria mercenaria
UdeM00000030069 6596 188.7 UdeM00000030069 XP_045176508.1 36.3 278 146 6 155 429 249 498 1.50E-44 188.7 6596 cellular organisms;Eukaryota;Opisthokonta;Metazoa;Eumetazoa;Bilateria;Protostomia;Spiralia;Lophotrochozoa;Mollusca;Bivalvia;Autobranchia;Heteroconchia;Euheterodonta;Imparidentia;Venerida;Veneroidea;Veneridae;Mercenaria;Mercenaria mercenaria
UdeM00000030070 6579 250 UdeM00000030070 XP_033740793.1 38.1 367 190 7 539 871 531 894 1.10E-62 250 6579 cellular organisms;Eukaryota;Opisthokonta;Metazoa;Eumetazoa;Bilateria;Protostomia;Spiralia;Lophotrochozoa;Mollusca;Bivalvia;Autobranchia;Pteriomorphia;Pectinida;Pectinoidea;Pectinidae;Pecten;Pecten maximus

UdeM00000030071 29159 62.4 UdeM00000030071 XP_034315424.1 37.6 109
58 4 12 116 213 315 1.00E-06 62.4 29159 cellular
organisms;Eukaryota;Opisthokonta;Metazoa;Eumetazoa;Bilateria;Protostomia;
Spiralia;Lophotrochozoa;Mollusca;Bivalvia;Autobranchia;Pteriomorphia;Ostr
eida;Ostreoidea;Ostreidae;Crassostrea;Crassostrea gigas
UdeM00000030072 6596 191.4 UdeM00000030072 XP_045195920.1 62.8 145
53 1 2 145 722 866 7.90E-46 191.4 6596 cellular
organisms;Eukaryota;Opisthokonta;Metazoa;Eumetazoa;Bilateria;Protostomia;
Spiralia;Lophotrochozoa;Mollusca;Bivalvia;Autobranchia;Heteroconchia;Euhe
terodonta;Imparidentia;Venerida;Veneroidea;Veneridae;Mercenaria;Mercenari
a mercenaria
UdeM00000030073 7574 360.1 UdeM00000030073 XP_013381040.1 67.2 256
78 3 16 270 56 306 2.40E-96 360.1 7574 cellular
organisms;Eukaryota;Opisthokonta;Metazoa;Eumetazoa;Bilateria;Protostomia;
Spiralia;Lophotrochozoa;Brachiopoda;Linguliformea;Lingulata;Lingulida;Lin
guloidea;Lingulidae;Lingula;Lingula anatina
UdeM00000030074 400727 474.2 UdeM00000030074 XP_025081071.1 41.9
640 341 8 85 715 108 725 3.00E-130 474.2 400727
cellular
organisms;Eukaryota;Opisthokonta;Metazoa;Eumetazoa;Bilateria;Protostomia;
Spiralia;Lophotrochozoa;Mollusca;Gastropoda;Caenogastropoda;Architaeniogl
ossa;Ampullarioidea;Ampullariidae;Pomacea;Pomacea canaliculata
UdeM00000030075 6596 807 UdeM00000030075 XP_045196641.1 69.7 792
212 2 3 794 4 767 2.20E-230 807 6596 cellular
organisms;Eukaryota;Opisthokonta;Metazoa;Eumetazoa;Bilateria;Protostomia;
Spiralia;Lophotrochozoa;Mollusca;Bivalvia;Autobranchia;Heteroconchia;Euhe
terodonta;Imparidentia;Venerida;Veneroidea;Veneridae;Mercenaria;Mercenari
a mercenaria
UdeM00000030076 6596 679.5 UdeM00000030076 XP_045196646.1 66.7 558
141 15 67 611 62 587 4.00E-192 679.5 6596 cellular
organisms;Eukaryota;Opisthokonta;Metazoa;Eumetazoa;Bilateria;Protostomia;
Spiralia;Lophotrochozoa;Mollusca;Bivalvia;Autobranchia;Heteroconchia;Euhe
terodonta;Imparidentia;Venerida;Veneroidea;Veneridae;Mercenaria;Mercenari
a mercenaria
UdeM00000030077 6596 677.2 UdeM00000030077 XP_045196646.1 66.5 558
141 15 67 610 62 587 2.00E-191 677.2 6596 cellular
organisms;Eukaryota;Opisthokonta;Metazoa;Eumetazoa;Bilateria;Protostomia;
Spiralia;Lophotrochozoa;Mollusca;Bivalvia;Autobranchia;Heteroconchia;Euhe
terodonta;Imparidentia;Venerida;Veneroidea;Veneridae;Mercenaria;Mercenari
a mercenaria
UdeM00000030078 6596 681.4 UdeM00000030078 XP_045196646.1 67 557
139 15 67 609 62 587 1.10E-192 681.4 6596 cellular
organisms;Eukaryota;Opisthokonta;Metazoa;Eumetazoa;Bilateria;Protostomia;
Spiralia;Lophotrochozoa;Mollusca;Bivalvia;Autobranchia;Heteroconchia;Euhe
terodonta;Imparidentia;Venerida;Veneroidea;Veneridae;Mercenaria;Mercenari
a mercenaria
UdeM00000030079 6596 232.6 UdeM00000030079 XP_045195277.1 89.7 126
13 0 63 188 2026 2151 4.00E-58 232.6 6596 cellular
organisms;Eukaryota;Opisthokonta;Metazoa;Eumetazoa;Bilateria;Protostomia;
Spiralia;Lophotrochozoa;Mollusca;Bivalvia;Autobranchia;Heteroconchia;Euhe
terodonta;Imparidentia;Venerida;Veneroidea;Veneridae;Mercenaria;Mercenari
a mercenaria
UdeM00000030080 37653 288.1 UdeM00000030080 XP_014784607.1 67 206
65 1 1 203 141 346 8.70E-75 288.1 37653 cellular

organisms;Eukaryota;Opisthokonta;Metazoa;Eumetazoa;Bilateria;Protostomia;
Spiralia;Lophotrochozoa;Mollusca;Cephalopoda;Coleoidea;Octopodiformes;Oct
opoda;Incirrata;Octopodidae;Octopus;Octopus bimaculoides
UdeM00000030081 6596 82.4 UdeM00000030081 XP_045195285.1 24.9 422
272 12 17 429 35 420 1.60E-12 82.4 6596 cellular
organisms;Eukaryota;Opisthokonta;Metazoa;Eumetazoa;Bilateria;Protostomia;
Spiralia;Lophotrochozoa;Mollusca;Bivalvia;Autobranchia;Heteroconchia;Euhe
terodonta;Imparidentia;Venerida;Veneroidea;Veneridae;Mercenaria;Mercenari
a mercenaria
UdeM00000030082 6596 81.6 UdeM00000030082 XP_045195285.1 24.9 422
272 12 17 429 35 420 4.70E-12 81.6 6596 cellular
organisms;Eukaryota;Opisthokonta;Metazoa;Eumetazoa;Bilateria;Protostomia;
Spiralia;Lophotrochozoa;Mollusca;Bivalvia;Autobranchia;Heteroconchia;Euhe
terodonta;Imparidentia;Venerida;Veneroidea;Veneridae;Mercenaria;Mercenari
a mercenaria
UdeM00000030083 6579 61.2 UdeM00000030083 XP_033741021.1 28.1 228
135 6 11 222 41 255 2.00E-06 61.2 6579 cellular
organisms;Eukaryota;Opisthokonta;Metazoa;Eumetazoa;Bilateria;Protostomia;
Spiralia;Lophotrochozoa;Mollusca;Bivalvia;Autobranchia;Pteriomorphia;Pect
inida;Pectinoidea;Pectinidae;Pecten;Pecten maximus
UdeM00000030085 6596 184.9 UdeM00000030085 XP_045195291.1 32.6 347
223 7 10 353 8 346 2.10E-43 184.9 6596 cellular
organisms;Eukaryota;Opisthokonta;Metazoa;Eumetazoa;Bilateria;Protostomia;
Spiralia;Lophotrochozoa;Mollusca;Bivalvia;Autobranchia;Heteroconchia;Euhe
terodonta;Imparidentia;Venerida;Veneroidea;Veneridae;Mercenaria;Mercenari
a mercenaria
UdeM00000030086 6596 183.7 UdeM00000030086 XP_045195291.1 32.3 347
224 7 10 353 8 346 4.70E-43 183.7 6596 cellular
organisms;Eukaryota;Opisthokonta;Metazoa;Eumetazoa;Bilateria;Protostomia;
Spiralia;Lophotrochozoa;Mollusca;Bivalvia;Autobranchia;Heteroconchia;Euhe
terodonta;Imparidentia;Venerida;Veneroidea;Veneridae;Mercenaria;Mercenari
a mercenaria
UdeM00000030087 6596 180.6 UdeM00000030087 XP_045195291.1 32.4 346
223 7 3 345 9 346 3.90E-42 180.6 6596 cellular
organisms;Eukaryota;Opisthokonta;Metazoa;Eumetazoa;Bilateria;Protostomia;
Spiralia;Lophotrochozoa;Mollusca;Bivalvia;Autobranchia;Heteroconchia;Euhe
terodonta;Imparidentia;Venerida;Veneroidea;Veneridae;Mercenaria;Mercenari
a mercenaria
UdeM00000030088 6596 958.4 UdeM00000030088 XP_045196538.1 44.8 1508
670 36 1 1433 1 1421 1.20E-275 958.4 6596 cellular
organisms;Eukaryota;Opisthokonta;Metazoa;Eumetazoa;Bilateria;Protostomia;
Spiralia;Lophotrochozoa;Mollusca;Bivalvia;Autobranchia;Heteroconchia;Euhe
terodonta;Imparidentia;Venerida;Veneroidea;Veneridae;Mercenaria;Mercenari
a mercenaria
UdeM00000030089 6596 692.2 UdeM00000030089 XP_045197843.1 58.6 534
212 5 5 535 65 592 5.60E-196 692.2 6596 cellular
organisms;Eukaryota;Opisthokonta;Metazoa;Eumetazoa;Bilateria;Protostomia;
Spiralia;Lophotrochozoa;Mollusca;Bivalvia;Autobranchia;Heteroconchia;Euhe
terodonta;Imparidentia;Venerida;Veneroidea;Veneridae;Mercenaria;Mercenari
a mercenaria
UdeM00000030090 37653 520.4 UdeM00000030090 XP_014784610.1 63 408
133 5 1 391 1 407 2.00E-144 520.4 37653 cellular
organisms;Eukaryota;Opisthokonta;Metazoa;Eumetazoa;Bilateria;Protostomia;

Spiralia;Lophotrochozoa;Mollusca;Cephalopoda;Coleoidea;Octopodiformes;Octopoda;Incirrata;Octopodidae;Octopus;Octopus bimaculoides
UdeM00000030092 6573 360.5 UdeM00000030092 XP_021380096.1 40.3 588 281 15 131 662 77 650 4.60E-96 360.5 6573 cellular organisms;Eukaryota;Opisthokonta;Metazoa;Eumetazoa;Bilateria;Protostomia;Spiralia;Lophotrochozoa;Mollusca;Bivalvia;Autobranchia;Pteriomorphia;Pectinida;Pectinoidea;Pectinidae;Mizuhopecten;Mizuhopecten yessoensis
UdeM00000030093 6596 216.9 UdeM00000030093 XP_045195162.1 65.4 162 56 0 4 165 1 162 2.00E-53 216.9 6596 cellular organisms;Eukaryota;Opisthokonta;Metazoa;Eumetazoa;Bilateria;Protostomia;Spiralia;Lophotrochozoa;Mollusca;Bivalvia;Autobranchia;Heteroconchia;Euheterodonta;Imparidentia;Venerida;Veneroidea;Veneridae;Mercenaria;Mercenaria mercenaria
UdeM00000030094 6596 476.5 UdeM00000030094 XP_045195164.1 87.2 337 43 0 1 337 1 337 3.40E-131 476.5 6596 cellular organisms;Eukaryota;Opisthokonta;Metazoa;Eumetazoa;Bilateria;Protostomia;Spiralia;Lophotrochozoa;Mollusca;Bivalvia;Autobranchia;Heteroconchia;Euheterodonta;Imparidentia;Venerida;Veneroidea;Veneridae;Mercenaria;Mercenaria mercenaria
UdeM00000030095 29159 306.2 UdeM00000030095 XP_011424929.3 40 448 216 9 8 442 27 434 6.80E-80 306.2 29159 cellular organisms;Eukaryota;Opisthokonta;Metazoa;Eumetazoa;Bilateria;Protostomia;Spiralia;Lophotrochozoa;Mollusca;Bivalvia;Autobranchia;Pteriomorphia;Ostreida;Ostreoidea;Ostreidae;Crassostrea;Crassostrea gigas
UdeM00000030096 6579 477.2 UdeM00000030096 XP_033739932.1 66.6 341 108 1 1 335 1 341 1.70E-131 477.2 6579 cellular organisms;Eukaryota;Opisthokonta;Metazoa;Eumetazoa;Bilateria;Protostomia;Spiralia;Lophotrochozoa;Mollusca;Bivalvia;Autobranchia;Pteriomorphia;Pectinida;Pectinoidea;Pectinidae;Pecten;Pecten maximus
UdeM00000030097 37653 1667.1 UdeM00000030097 XP_014779463.1 62.8 1450 486 14 45 1472 17 1434 0.00E+00 1667.1 37653 cellular organisms;Eukaryota;Opisthokonta;Metazoa;Eumetazoa;Bilateria;Protostomia;Spiralia;Lophotrochozoa;Mollusca;Cephalopoda;Coleoidea;Octopodiformes;Octopoda;Incirrata;Octopodidae;Octopus;Octopus bimaculoides
UdeM00000030098 37653 1670.2 UdeM00000030098 XP_014779463.1 62.7 1449 487 13 45 1471 17 1434 0.00E+00 1670.2 37653 cellular organisms;Eukaryota;Opisthokonta;Metazoa;Eumetazoa;Bilateria;Protostomia;Spiralia;Lophotrochozoa;Mollusca;Cephalopoda;Coleoidea;Octopodiformes;Octopoda;Incirrata;Octopodidae;Octopus;Octopus bimaculoides
UdeM00000030099 6596 7445.1 UdeM00000030099 XP_045195277.1 81.4 4552 832 3 63 4602 70 4619 0.00E+00 7445.1 6596 cellular organisms;Eukaryota;Opisthokonta;Metazoa;Eumetazoa;Bilateria;Protostomia;Spiralia;Lophotrochozoa;Mollusca;Bivalvia;Autobranchia;Heteroconchia;Euheterodonta;Imparidentia;Venerida;Veneroidea;Veneridae;Mercenaria;Mercenaria mercenaria
UdeM00000030100 6596 100.5 UdeM00000030100 XP_045195265.1 41.3 167 84 6 22 177 13 176 2.40E-18 100.5 6596 cellular organisms;Eukaryota;Opisthokonta;Metazoa;Eumetazoa;Bilateria;Protostomia;Spiralia;Lophotrochozoa;Mollusca;Bivalvia;Autobranchia;Heteroconchia;Euheterodonta;Imparidentia;Venerida;Veneroidea;Veneridae;Mercenaria;Mercenaria mercenaria

UdeM00000030101 225164 123.6 UdeM00000030101 XP_009060400.1 28
446 271 11 12 426 1 427 6.60E-25 123.6 225164
cellular
organisms;Eukaryota;Opisthokonta;Metazoa;Eumetazoa;Bilateria;Protostomia;
Spiralia;Lophotrochozoa;Mollusca;Gastropoda;Patellogastropoda;Lottioidea;
Lottiidae;Lottia;Lottia gigantea
UdeM00000030102 6565 531.9 UdeM00000030102 XP_022286442.1 80.2 318
63 0 1 318 1 318 5.40E-148 531.9 6565 cellular
organisms;Eukaryota;Opisthokonta;Metazoa;Eumetazoa;Bilateria;Protostomia;
Spiralia;Lophotrochozoa;Mollusca;Bivalvia;Autobranchia;Pteriomorphia;Ostr
eida;Ostreoidea;Ostreidae;Crassostrea;Crassostrea virginica
UdeM00000030103 6500 85.1 UdeM00000030103 XP_005091192.1 51.4 74
36 0 20 93 7 80 5.10E-14 85.1 6500 cellular
organisms;Eukaryota;Opisthokonta;Metazoa;Eumetazoa;Bilateria;Protostomia;
Spiralia;Lophotrochozoa;Mollusca;Gastropoda;Heterobranchia;Euthyneura;Tec
tipleura;Aplysiida;Aplysioidea;Aplysiidae;Aplysia;Aplysia californica
UdeM00000030105 1735272 84.7 UdeM00000030105 XP_041351986.1 49.4
85 41 1 5 87 1 85 6.30E-14 84.7 1735272
cellular
organisms;Eukaryota;Opisthokonta;Metazoa;Eumetazoa;Bilateria;Protostomia;
Spiralia;Lophotrochozoa;Mollusca;Gastropoda;Neomphalina;Neomphaloidea;Pel
tospiridae;Gigantopelta;Gigantopelta aegis
UdeM00000030104 225164 61.6 UdeM00000030104 XP_009060322.1 47.5
59 31 0 5 63 1 59 5.90E-07 61.6 225164
cellular
organisms;Eukaryota;Opisthokonta;Metazoa;Eumetazoa;Bilateria;Protostomia;
Spiralia;Lophotrochozoa;Mollusca;Gastropoda;Patellogastropoda;Lottioidea;
Lottiidae;Lottia;Lottia gigantea
UdeM00000030106 6596 238.4 UdeM00000030106 XP_045182143.1 55.9 195
85 1 17 211 21 214 8.20E-60 238.4 6596 cellular
organisms;Eukaryota;Opisthokonta;Metazoa;Eumetazoa;Bilateria;Protostomia;
Spiralia;Lophotrochozoa;Mollusca;Bivalvia;Autobranchia;Heteroconchia;Euhe
terodonta;Imparidentia;Venerida;Veneroidea;Veneridae;Mercenaria;Mercenari
a mercenaria
UdeM00000030107 6105 304.3 UdeM00000030107 XP_031572471.1 42.5 369
200 7 6 366 9 373 2.40E-79 304.3 6105 cellular
organisms;Eukaryota;Opisthokonta;Metazoa;Eumetazoa;Cnidaria;Anthozoa;Hexa
corallia;Actiniaria;Actiniidae;Actinia;Actinia tenebrosa
UdeM00000030108 46731 315.1 UdeM00000030108 XP_027048652.1 43.4 371
195 8 6 364 10 377 1.60E-82 315.1 46731 cellular
organisms;Eukaryota;Opisthokonta;Metazoa;Eumetazoa;Cnidaria;Anthozoa;Hexa
corallia;Scleractinia;Astrocoeniina;Pocilloporidae;Pocillopora;Pocillopor
a damicornis
UdeM00000030109 7574 357.5 UdeM00000030109 XP_023932609.1 37.4 548
304 16 1 517 13 552 3.10E-95 357.5 7574 cellular
organisms;Eukaryota;Opisthokonta;Metazoa;Eumetazoa;Bilateria;Protostomia;
Spiralia;Lophotrochozoa;Brachiopoda;Linguliformea;Lingulata;Lingulida;Lin
guloidea;Lingulidae;Lingula;Lingula anatina
UdeM00000030110 6596 115.2 UdeM00000030110 XP_045182143.1 41.4 152
59 2 5 127 64 214 6.30E-23 115.2 6596 cellular
organisms;Eukaryota;Opisthokonta;Metazoa;Eumetazoa;Bilateria;Protostomia;
Spiralia;Lophotrochozoa;Mollusca;Bivalvia;Autobranchia;Heteroconchia;Euhe
terodonta;Imparidentia;Venerida;Veneroidea;Veneridae;Mercenaria;Mercenari
a mercenaria

UdeM00000030111 48498 254.2 UdeM00000030111 XP_020620504.1 54.5 189
85 1 9 196 576 764 1.90E-64 254.2 48498 cellular
organisms;Eukaryota;Opisthokonta;Metazoa;Eumetazoa;Cnidaria;Anthozoa;Hexacorallia;Scleractinia;Faviina;Merulinidae;Orbicella;Orbicella faveolata
UdeM00000030113 6105 322.8 UdeM00000030113 XP_031572471.1 46.3 356
180 7 10 358 22 373 7.90E-85 322.8 6105 cellular
organisms;Eukaryota;Opisthokonta;Metazoa;Eumetazoa;Cnidaria;Anthozoa;Hexacorallia;Actiniaria;Actiniidae;Actinia;Actinia tenebrosa
UdeM00000030115 6573 327.4 UdeM00000030115 XP_021362608.1 36.8 494
279 12 1 472 16 498 3.20E-86 327.4 6573 cellular
organisms;Eukaryota;Opisthokonta;Metazoa;Eumetazoa;Bilateria;Protostomia;Spiralia;Lophotrochozoa;Mollusca;Bivalvia;Autobranchia;Pteriomorphia;Pectinida;Pectinoidea;Pectinidae;Mizuhopecten;Mizuhopecten yessoensis
UdeM00000030114 6573 327.4 UdeM00000030114 XP_021362608.1 36.8 494
279 12 1 472 16 498 3.50E-86 327.4 6573 cellular
organisms;Eukaryota;Opisthokonta;Metazoa;Eumetazoa;Bilateria;Protostomia;Spiralia;Lophotrochozoa;Mollusca;Bivalvia;Autobranchia;Pteriomorphia;Pectinida;Pectinoidea;Pectinidae;Mizuhopecten;Mizuhopecten yessoensis
UdeM00000030118 6573 322.8 UdeM00000030118 XP_021362608.1 35.9 501
286 13 2 479 9 497 8.00E-85 322.8 6573 cellular
organisms;Eukaryota;Opisthokonta;Metazoa;Eumetazoa;Bilateria;Protostomia;Spiralia;Lophotrochozoa;Mollusca;Bivalvia;Autobranchia;Pteriomorphia;Pectinida;Pectinoidea;Pectinidae;Mizuhopecten;Mizuhopecten yessoensis
UdeM00000030117 6573 322.8 UdeM00000030117 XP_021362608.1 35.9 501
286 13 2 479 9 497 8.80E-85 322.8 6573 cellular
organisms;Eukaryota;Opisthokonta;Metazoa;Eumetazoa;Bilateria;Protostomia;Spiralia;Lophotrochozoa;Mollusca;Bivalvia;Autobranchia;Pteriomorphia;Pectinida;Pectinoidea;Pectinidae;Mizuhopecten;Mizuhopecten yessoensis
UdeM00000030119 7574 413.3 UdeM00000030119 XP_023932609.1 39.2 584
322 14 1 557 13 590 5.60E-112 413.3 7574 cellular
organisms;Eukaryota;Opisthokonta;Metazoa;Eumetazoa;Bilateria;Protostomia;Spiralia;Lophotrochozoa;Brachiopoda;Linguliformea;Lingulata;Lingulida;Linguloidea;Lingulidae;Lingula;Lingula anatina
UdeM00000030120 7574 413.3 UdeM00000030120 XP_023932609.1 39.2 584
322 14 1 557 13 590 5.60E-112 413.3 7574 cellular
organisms;Eukaryota;Opisthokonta;Metazoa;Eumetazoa;Bilateria;Protostomia;Spiralia;Lophotrochozoa;Brachiopoda;Linguliformea;Lingulata;Lingulida;Linguloidea;Lingulidae;Lingula;Lingula anatina
UdeM00000030121 6573 326.6 UdeM00000030121 XP_021362608.1 36.2 505
288 12 2 484 9 501 5.20E-86 326.6 6573 cellular
organisms;Eukaryota;Opisthokonta;Metazoa;Eumetazoa;Bilateria;Protostomia;Spiralia;Lophotrochozoa;Mollusca;Bivalvia;Autobranchia;Pteriomorphia;Pectinida;Pectinoidea;Pectinidae;Mizuhopecten;Mizuhopecten yessoensis
UdeM00000030122 6105 319.7 UdeM00000030122 XP_031572471.1 44.4 369
194 7 6 367 9 373 5.60E-84 319.7 6105 cellular
organisms;Eukaryota;Opisthokonta;Metazoa;Eumetazoa;Cnidaria;Anthozoa;Hexacorallia;Actiniaria;Actiniidae;Actinia;Actinia tenebrosa
UdeM00000030123 1735272 327.4 UdeM00000030123 XP_041361403.1 46.2
357 181 7 17 366 21 373 3.20E-86 327.4 1735272
cellular
organisms;Eukaryota;Opisthokonta;Metazoa;Eumetazoa;Bilateria;Protostomia;Spiralia;Lophotrochozoa;Mollusca;Gastropoda;Neomphalina;Neomphaloidea;Peltospiridae;Gigantopelta;Gigantopelta aegis

UdeM00000030124	6596	75.1	UdeM00000030124	XP_045173548.1	47.4	57
	28	1	95	151	613	667	8.60E-11	75.1	6596	cellular
organisms;Eukaryota;Opisthokonta;Metazoa;Eumetazoa;Bilateria;Protostomia;Spiralia;Lophotrochozoa;Mollusca;Bivalvia;Autobranchia;Heteroconchia;Euheterodonta;Imparidentia;Venerida;Veneroidea;Veneridae;Mercenaria;Mercenaria mercenaria
UdeM00000030125	48498	112.1	UdeM00000030125	XP_020620494.1	42.5	134
	62	2	131	260	728	850	1.20E-21	112.1	48498	cellular
organisms;Eukaryota;Opisthokonta;Metazoa;Eumetazoa;Cnidaria;Anthozoa;Hexacorallia;Scleractinia;Faviina;Merulinidae;Orbicella;Orbicella faveolata
UdeM00000030126	45351	122.9	UdeM00000030126	XP_032224987.1	47.5	122
	63	1	32	152	94	215	3.80E-25	122.9	45351	cellular
organisms;Eukaryota;Opisthokonta;Metazoa;Eumetazoa;Cnidaria;Anthozoa;Hexacorallia;Actiniaria;Edwardsiidae;Nematostella;Nematostella vectensis
UdeM00000030127	7574	578.9	UdeM00000030127	XP_023932609.1	41.8	717
	385	14	16	707	33	742	9.60E-162	578.9	7574	cellular
organisms;Eukaryota;Opisthokonta;Metazoa;Eumetazoa;Bilateria;Protostomia;Spiralia;Lophotrochozoa;Brachiopoda;Linguliformea;Lingulata;Lingulida;Linguloidea;Lingulidae;Lingula;Lingula anatina
UdeM00000030129	29159	3488.7	UdeM00000030129	XP_034333407.1	75.3
	2258	541	11	75	2327	103	2349	0.00E+00	3488.7
	29159	cellular
organisms;Eukaryota;Opisthokonta;Metazoa;Eumetazoa;Bilateria;Protostomia;Spiralia;Lophotrochozoa;Mollusca;Bivalvia;Autobranchia;Pteriomorphia;Ostreida;Ostreoidea;Ostreidae;Crassostrea;Crassostrea gigas
UdeM00000030130	6596	306.2	UdeM00000030130	XP_045187105.1	48.3	333
	160	5	11	343	5	325	5.20E-80	306.2	6596	cellular
organisms;Eukaryota;Opisthokonta;Metazoa;Eumetazoa;Bilateria;Protostomia;Spiralia;Lophotrochozoa;Mollusca;Bivalvia;Autobranchia;Heteroconchia;Euheterodonta;Imparidentia;Venerida;Veneroidea;Veneridae;Mercenaria;Mercenaria mercenaria
UdeM00000030131	6596	620.5	UdeM00000030131	XP_045212567.1	90	329
	33	0	20	348	443	771	1.30E-174	620.5	6596	cellular
organisms;Eukaryota;Opisthokonta;Metazoa;Eumetazoa;Bilateria;Protostomia;Spiralia;Lophotrochozoa;Mollusca;Bivalvia;Autobranchia;Heteroconchia;Euheterodonta;Imparidentia;Venerida;Veneroidea;Veneridae;Mercenaria;Mercenaria mercenaria
UdeM00000030132	6596	1197.2	UdeM00000030132	XP_045212567.1	81.5
	728	82	1	67	794	97	771	0.00E+00	1197.2	6596
	cellular
organisms;Eukaryota;Opisthokonta;Metazoa;Eumetazoa;Bilateria;Protostomia;Spiralia;Lophotrochozoa;Mollusca;Bivalvia;Autobranchia;Heteroconchia;Euheterodonta;Imparidentia;Venerida;Veneroidea;Veneridae;Mercenaria;Mercenaria mercenaria
UdeM00000030133	6596	370.9	UdeM00000030133	XP_045198089.1	39.7	774
	409	21	1	752	37	774	3.80E-99	370.9	6596	cellular
organisms;Eukaryota;Opisthokonta;Metazoa;Eumetazoa;Bilateria;Protostomia;Spiralia;Lophotrochozoa;Mollusca;Bivalvia;Autobranchia;Heteroconchia;Euheterodonta;Imparidentia;Venerida;Veneroidea;Veneridae;Mercenaria;Mercenaria mercenaria
UdeM00000030134	6596	86.3	UdeM00000030134	XP_045196666.1	33	194
	87	6	70	248	143	308	6.10E-14	86.3	6596	cellular
organisms;Eukaryota;Opisthokonta;Metazoa;Eumetazoa;Bilateria;Protostomia;Spiralia;Lophotrochozoa;Mollusca;Bivalvia;Autobranchia;Heteroconchia;Euhe

terodonta;Imparidentia;Venerida;Veneroidea;Veneridae;Mercenaria;Mercenaria mercenaria
UdeM00000030135 6596 159.8 UdeM00000030135 XP_045198458.1 73.7 99
26 0 1 99 1 99 1.80E-36 159.8 6596 cellular
organisms;Eukaryota;Opisthokonta;Metazoa;Eumetazoa;Bilateria;Protostomia;Spiralia;Lophotrochozoa;Mollusca;Bivalvia;Autobranchia;Heteroconchia;Euheterodonta;Imparidentia;Venerida;Veneroidea;Veneridae;Mercenaria;Mercenaria mercenaria
UdeM00000030136 225164 123.6 UdeM00000030136 XP_009062162.1 63.8
127 34 2 8 127 102 223 1.80E-25 123.6 225164
cellular
organisms;Eukaryota;Opisthokonta;Metazoa;Eumetazoa;Bilateria;Protostomia;Spiralia;Lophotrochozoa;Mollusca;Gastropoda;Patellogastropoda;Lottioidea;Lottiidae;Lottia;Lottia gigantea
UdeM00000030137 6596 1437.2 UdeM00000030137 XP_045198458.1 46.4
2766 819 55 1 2674 128 2322 0.00E+00 1437.2 6596
cellular
organisms;Eukaryota;Opisthokonta;Metazoa;Eumetazoa;Bilateria;Protostomia;Spiralia;Lophotrochozoa;Mollusca;Bivalvia;Autobranchia;Heteroconchia;Euheterodonta;Imparidentia;Venerida;Veneroidea;Veneridae;Mercenaria;Mercenaria mercenaria
UdeM00000030138 6596 266.5 UdeM00000030138 XP_045167863.1 61.8 199
73 2 169 367 106 301 4.90E-68 266.5 6596 cellular
organisms;Eukaryota;Opisthokonta;Metazoa;Eumetazoa;Bilateria;Protostomia;Spiralia;Lophotrochozoa;Mollusca;Bivalvia;Autobranchia;Heteroconchia;Euheterodonta;Imparidentia;Venerida;Veneroidea;Veneridae;Mercenaria;Mercenaria mercenaria
UdeM00000030139 6596 1279.2 UdeM00000030139 XP_045196090.1 85.3
843 116 4 1 838 1 840 0.00E+00 1279.2 6596
cellular
organisms;Eukaryota;Opisthokonta;Metazoa;Eumetazoa;Bilateria;Protostomia;Spiralia;Lophotrochozoa;Mollusca;Bivalvia;Autobranchia;Heteroconchia;Euheterodonta;Imparidentia;Venerida;Veneroidea;Veneridae;Mercenaria;Mercenaria mercenaria
UdeM00000030140 6579 315.1 UdeM00000030140 XP_033740054.1 41.7 446
248 7 9 446 6 447 1.50E-82 315.1 6579 cellular
organisms;Eukaryota;Opisthokonta;Metazoa;Eumetazoa;Bilateria;Protostomia;Spiralia;Lophotrochozoa;Mollusca;Bivalvia;Autobranchia;Pteriomorphia;Pectinida;Pectinoidea;Pectinidae;Pecten;Pecten maximus
UdeM00000030141 6573 193.4 UdeM00000030141 XP_021377366.1 75.8 128
27 2 1 127 1 125 1.90E-46 193.4 6573 cellular
organisms;Eukaryota;Opisthokonta;Metazoa;Eumetazoa;Bilateria;Protostomia;Spiralia;Lophotrochozoa;Mollusca;Bivalvia;Autobranchia;Pteriomorphia;Pectinida;Pectinoidea;Pectinidae;Mizuhopecten;Mizuhopecten yessoensis
UdeM00000030142 37621 113.6 UdeM00000030142 XP_014672134.1 41.6 137
77 1 2 135 139 275 2.10E-22 113.6 37621 cellular
organisms;Eukaryota;Opisthokonta;Metazoa;Eumetazoa;Bilateria;Protostomia;Ecdysozoa;Scalidophora;Priapulida;Priapulimorpha;Priapulimorphida;Priapulidae;Priapulus;Priapulus caudatus
UdeM00000030147 6579 234.2 UdeM00000030147 XP_033740844.1 49.4 344
138 16 1 319 130 462 2.40E-58 234.2 6579 cellular
organisms;Eukaryota;Opisthokonta;Metazoa;Eumetazoa;Bilateria;Protostomia;Spiralia;Lophotrochozoa;Mollusca;Bivalvia;Autobranchia;Pteriomorphia;Pectinida;Pectinoidea;Pectinidae;Pecten;Pecten maximus

UdeM00000030146	6579	263.1	UdeM00000030146	XP_033740844.1	45	556
	229	22	1	507	130	657	7.40E-67	263.1	6579	cellular
organisms;Eukaryota;Opisthokonta;Metazoa;Eumetazoa;Bilateria;Protostomia;
Spiralia;Lophotrochozoa;Mollusca;Bivalvia;Autobranchia;Pteriomorphia;Pect
inida;Pectinoidea;Pectinidae;Pecten;Pecten maximus
UdeM00000030148	6596	69.3	UdeM00000030148	XP_045181585.1	35.9	92
	57	2	1	91	1	91	1.70E-08	69.3	6596	cellular
organisms;Eukaryota;Opisthokonta;Metazoa;Eumetazoa;Bilateria;Protostomia;
Spiralia;Lophotrochozoa;Mollusca;Bivalvia;Autobranchia;Heteroconchia;Euhe
terodonta;Imparidentia;Venerida;Veneroidea;Veneridae;Mercenaria;Mercenari
a mercenaria
UdeM00000030149	6565	81.3	UdeM00000030149	XP_022329491.1	39.8	98
	58	1	1	97	1	98	5.80E-12	81.3	6565	cellular
organisms;Eukaryota;Opisthokonta;Metazoa;Eumetazoa;Bilateria;Protostomia;
Spiralia;Lophotrochozoa;Mollusca;Bivalvia;Autobranchia;Pteriomorphia;Ostr
eida;Ostreoidea;Ostreidae;Crassostrea;Crassostrea virginica
UdeM00000030150	29159	173.3	UdeM00000030150	XP_034323800.1	23.9	750
	448	22	15	737	27	680	1.10E-39	173.3	29159	cellular
organisms;Eukaryota;Opisthokonta;Metazoa;Eumetazoa;Bilateria;Protostomia;
Spiralia;Lophotrochozoa;Mollusca;Bivalvia;Autobranchia;Pteriomorphia;Ostr
eida;Ostreoidea;Ostreidae;Crassostrea;Crassostrea gigas
UdeM00000030152	6596	4008.4	UdeM00000030152	XP_045195413.1	46.9
	4656	2006	68	1	4255	1077	5665	0.00E+00	4008.4	6596
	cellular
organisms;Eukaryota;Opisthokonta;Metazoa;Eumetazoa;Bilateria;Protostomia;
Spiralia;Lophotrochozoa;Mollusca;Bivalvia;Autobranchia;Heteroconchia;Euhe
terodonta;Imparidentia;Venerida;Veneroidea;Veneridae;Mercenaria;Mercenari
a mercenaria
UdeM00000030154	6596	114.8	UdeM00000030154	XP_045198336.1	42.6	122
	69	1	5	125	579	700	8.70E-23	114.8	6596	cellular
organisms;Eukaryota;Opisthokonta;Metazoa;Eumetazoa;Bilateria;Protostomia;
Spiralia;Lophotrochozoa;Mollusca;Bivalvia;Autobranchia;Heteroconchia;Euhe
terodonta;Imparidentia;Venerida;Veneroidea;Veneridae;Mercenaria;Mercenari
a mercenaria
UdeM00000030153	6596	246.9	UdeM00000030153	XP_045195418.1	32.1	455
	289	8	575	1019	736	1180	1.10E-61	246.9	6596	cellular
organisms;Eukaryota;Opisthokonta;Metazoa;Eumetazoa;Bilateria;Protostomia;
Spiralia;Lophotrochozoa;Mollusca;Bivalvia;Autobranchia;Heteroconchia;Euhe
terodonta;Imparidentia;Venerida;Veneroidea;Veneridae;Mercenaria;Mercenari
a mercenaria
UdeM00000030155	6596	854.4	UdeM00000030155	XP_045195413.1	48.6	933
	446	10	698	1603	4740	5665	2.40E-244	854.4	6596	cellular
organisms;Eukaryota;Opisthokonta;Metazoa;Eumetazoa;Bilateria;Protostomia;
Spiralia;Lophotrochozoa;Mollusca;Bivalvia;Autobranchia;Heteroconchia;Euhe
terodonta;Imparidentia;Venerida;Veneroidea;Veneridae;Mercenaria;Mercenari
a mercenaria
UdeM00000030155	6596	444.5	UdeM00000030155	XP_045195413.1	42.4	568
	315	6	165	724	1230	1793	5.70E-121	444.5	6596	cellular
organisms;Eukaryota;Opisthokonta;Metazoa;Eumetazoa;Bilateria;Protostomia;
Spiralia;Lophotrochozoa;Mollusca;Bivalvia;Autobranchia;Heteroconchia;Euhe
terodonta;Imparidentia;Venerida;Veneroidea;Veneridae;Mercenaria;Mercenari
a mercenaria
UdeM00000010343	6579	250.8	UdeM00000010343	XP_033757472.1	53.6	401
	142	5	1	401	1	357	3.00E-63	250.8	6579	cellular

organisms;Eukaryota;Opisthokonta;Metazoa;Eumetazoa;Bilateria;Protostomia;Spiralia;Lophotrochozoa;Mollusca;Bivalvia;Autobranchia;Pteriomorphia;Pectinida;Pectinoidea;Pectinidae;Pecten;Pecten maximus
UdeM00000010345 7574 236.1 UdeM00000010345 XP_013405909.1 72.6 146 40 0 1 146 1 146 5.80E-59 236.1 7574 cellular organisms;Eukaryota;Opisthokonta;Metazoa;Eumetazoa;Bilateria;Protostomia;Spiralia;Lophotrochozoa;Brachiopoda;Linguliformea;Lingulata;Lingulida;Linguloidea;Lingulidae;Lingula;Lingula anatina
UdeM00000010344 31138 65.9 UdeM00000010344 XP_034648626.1 30.7 114 65 3 4 103 206 319 3.80E-08 65.9 31138 cellular organisms;Eukaryota;Opisthokonta;Metazoa;Eumetazoa;Bilateria;Deuterostomia;Chordata;Craniata;Vertebrata;Gnathostomata;Teleostomi;Euteleostomi;Sarcopterygii;Dipnotetrapodomorpha;Tetrapoda;Amniota;Sauropsida;Sauria;Archelosauria;Testudinata;Testudines;Cryptodira;Durocryptodira;Testudinoidea;Emydidae;Trachemys;Trachemys scripta;Trachemys scripta elegans
UdeM00000010346 6596 850.5 UdeM00000010346 XP_045188151.1 49.7 845 349 13 1 773 85 925 1.70E-243 850.5 6596 cellular organisms;Eukaryota;Opisthokonta;Metazoa;Eumetazoa;Bilateria;Protostomia;Spiralia;Lophotrochozoa;Mollusca;Bivalvia;Autobranchia;Heteroconchia;Euheterodonta;Imparidentia;Venerida;Veneroidea;Veneridae;Mercenaria;Mercenaria mercenaria
UdeM00000010347 225164 320.5 UdeM00000010347 XP_009051864.1 36.2 464 284 7 43 499 41 499 3.90E-84 320.5 225164 cellular organisms;Eukaryota;Opisthokonta;Metazoa;Eumetazoa;Bilateria;Protostomia;Spiralia;Lophotrochozoa;Mollusca;Gastropoda;Patellogastropoda;Lottioidea;Lottiidae;Lottia;Lottia gigantea
UdeM00000010348 1735272 392.1 UdeM00000010348 XP_041368965.1 46.2 487 210 10 19 497 16 458 1.10E-105 392.1 1735272 cellular organisms;Eukaryota;Opisthokonta;Metazoa;Eumetazoa;Bilateria;Protostomia;Spiralia;Lophotrochozoa;Mollusca;Gastropoda;Neomphalina;Neomphaloidea;Peltospiridae;Gigantopelta;Gigantopelta aegis
UdeM00000010349 6596 244.6 UdeM00000010349 XP_045189419.1 75.2 157 39 0 63 219 4 160 1.20E-61 244.6 6596 cellular organisms;Eukaryota;Opisthokonta;Metazoa;Eumetazoa;Bilateria;Protostomia;Spiralia;Lophotrochozoa;Mollusca;Bivalvia;Autobranchia;Heteroconchia;Euheterodonta;Imparidentia;Venerida;Veneroidea;Veneridae;Mercenaria;Mercenaria mercenaria
UdeM00000010350 6596 569.3 UdeM00000010350 XP_045189999.1 77.6 375 83 1 24 397 15 389 3.80E-159 569.3 6596 cellular organisms;Eukaryota;Opisthokonta;Metazoa;Eumetazoa;Bilateria;Protostomia;Spiralia;Lophotrochozoa;Mollusca;Bivalvia;Autobranchia;Heteroconchia;Euheterodonta;Imparidentia;Venerida;Veneroidea;Veneridae;Mercenaria;Mercenaria mercenaria
UdeM00000010351 6579 708.4 UdeM00000010351 XP_033756445.1 72.2 474 127 2 172 640 203 676 8.40E-201 708.4 6579 cellular organisms;Eukaryota;Opisthokonta;Metazoa;Eumetazoa;Bilateria;Protostomia;Spiralia;Lophotrochozoa;Mollusca;Bivalvia;Autobranchia;Pteriomorphia;Pectinida;Pectinoidea;Pectinidae;Pecten;Pecten maximus
UdeM00000010352 6579 201.1 UdeM00000010352 XP_033756445.1 69.9 146 42 1 1 144 222 367 9.90E-49 201.1 6579 cellular organisms;Eukaryota;Opisthokonta;Metazoa;Eumetazoa;Bilateria;Protostomia;

Spiralia;Lophotrochozoa;Mollusca;Bivalvia;Autobranchia;Pteriomorphia;Pectinida;Pectinoidea;Pectinidae;Pecten;Pecten maximus
UdeM00000010353 6596 240.7 UdeM00000010353 XP_045189995.1 61 210 80 2 1 209 1 209 1.60E-60 240.7 6596 cellular organisms;Eukaryota;Opisthokonta;Metazoa;Eumetazoa;Bilateria;Protostomia;Spiralia;Lophotrochozoa;Mollusca;Bivalvia;Autobranchia;Heteroconchia;Euheterodonta;Imparidentia;Venerida;Veneroidea;Veneridae;Mercenaria;Mercenaria mercenaria
UdeM00000010354 6596 586.6 UdeM00000010354 XP_045189708.1 55 540 236 4 8 547 10 542 3.20E-164 586.6 6596 cellular organisms;Eukaryota;Opisthokonta;Metazoa;Eumetazoa;Bilateria;Protostomia;Spiralia;Lophotrochozoa;Mollusca;Bivalvia;Autobranchia;Heteroconchia;Euheterodonta;Imparidentia;Venerida;Veneroidea;Veneridae;Mercenaria;Mercenaria mercenaria
UdeM00000010355 6596 269.2 UdeM00000010355 XP_045189377.1 68.9 196 49 3 1 196 1 184 4.00E-69 269.2 6596 cellular organisms;Eukaryota;Opisthokonta;Metazoa;Eumetazoa;Bilateria;Protostomia;Spiralia;Lophotrochozoa;Mollusca;Bivalvia;Autobranchia;Heteroconchia;Euheterodonta;Imparidentia;Venerida;Veneroidea;Veneridae;Mercenaria;Mercenaria mercenaria
UdeM00000010356 29159 123.6 UdeM00000010356 XP_034327695.1 65.3 75 26 0 12 86 11 85 1.20E-25 123.6 29159 cellular organisms;Eukaryota;Opisthokonta;Metazoa;Eumetazoa;Bilateria;Protostomia;Spiralia;Lophotrochozoa;Mollusca;Bivalvia;Autobranchia;Pteriomorphia;Ostreida;Ostreoidea;Ostreidae;Crassostrea;Crassostrea gigas
UdeM00000010357 29159 216.1 UdeM00000010357 XP_034327699.1 52.5 221 76 4 11 222 49 249 4.60E-53 216.1 29159 cellular organisms;Eukaryota;Opisthokonta;Metazoa;Eumetazoa;Bilateria;Protostomia;Spiralia;Lophotrochozoa;Mollusca;Bivalvia;Autobranchia;Pteriomorphia;Ostreida;Ostreoidea;Ostreidae;Crassostrea;Crassostrea gigas
UdeM00000010358 6565 132.9 UdeM00000010358 XP_022344103.1 48.7 156 68 2 1 144 117 272 3.30E-28 132.9 6565 cellular organisms;Eukaryota;Opisthokonta;Metazoa;Eumetazoa;Bilateria;Protostomia;Spiralia;Lophotrochozoa;Mollusca;Bivalvia;Autobranchia;Pteriomorphia;Ostreida;Ostreoidea;Ostreidae;Crassostrea;Crassostrea virginica
UdeM00000010360 6573 564.3 UdeM00000010360 XP_021371958.1 79.2 389 58 4 8 395 18 384 1.20E-157 564.3 6573 cellular organisms;Eukaryota;Opisthokonta;Metazoa;Eumetazoa;Bilateria;Protostomia;Spiralia;Lophotrochozoa;Mollusca;Bivalvia;Autobranchia;Pteriomorphia;Pectinida;Pectinoidea;Pectinidae;Mizuhopecten;Mizuhopecten yessoensis
UdeM00000010359 6579 553.9 UdeM00000010359 XP_033756090.1 79.4 383 50 5 7 388 15 369 1.60E-154 553.9 6579 cellular organisms;Eukaryota;Opisthokonta;Metazoa;Eumetazoa;Bilateria;Protostomia;Spiralia;Lophotrochozoa;Mollusca;Bivalvia;Autobranchia;Pteriomorphia;Pectinida;Pectinoidea;Pectinidae;Pecten;Pecten maximus
UdeM00000010361 6596 613.6 UdeM00000010361 XP_045187267.1 51.1 569 261 5 6 571 4 558 2.50E-172 613.6 6596 cellular organisms;Eukaryota;Opisthokonta;Metazoa;Eumetazoa;Bilateria;Protostomia;Spiralia;Lophotrochozoa;Mollusca;Bivalvia;Autobranchia;Heteroconchia;Euheterodonta;Imparidentia;Venerida;Veneroidea;Veneridae;Mercenaria;Mercenaria mercenaria
UdeM00000010362 6596 505 UdeM00000010362 XP_045187267.1 50.3 481 230 4 1 478 84 558 1.10E-139 505 6596 cellular organisms;Eukaryota;Opisthokonta;Metazoa;Eumetazoa;Bilateria;Protostomia;

Spiralia;Lophotrochozoa;Mollusca;Bivalvia;Autobranchia;Heteroconchia;Euheterodonta;Imparidentia;Venerida;Veneroidea;Veneridae;Mercenaria;Mercenaria mercenaria
UdeM00000010363 6579 69.3 UdeM00000010363 XP_033752062.1 33.9 109 72 0 40 148 57 165 7.10E-09 69.3 6579 cellular organisms;Eukaryota;Opisthokonta;Metazoa;Eumetazoa;Bilateria;Protostomia;Spiralia;Lophotrochozoa;Mollusca;Bivalvia;Autobranchia;Pteriomorphia;Pectinida;Pectinoidea;Pectinidae;Pecten;Pecten maximus
UdeM00000010364 6596 184.1 UdeM00000010364 XP_045176664.1 35.9 290 176 7 9 293 267 551 2.70E-43 184.1 6596 cellular organisms;Eukaryota;Opisthokonta;Metazoa;Eumetazoa;Bilateria;Protostomia;Spiralia;Lophotrochozoa;Mollusca;Bivalvia;Autobranchia;Heteroconchia;Euheterodonta;Imparidentia;Venerida;Veneroidea;Veneridae;Mercenaria;Mercenaria mercenaria
UdeM00000010365 6596 90.1 UdeM00000010365 XP_045177372.1 29.5 193 112 5 36 205 364 555 3.50E-15 90.1 6596 cellular organisms;Eukaryota;Opisthokonta;Metazoa;Eumetazoa;Bilateria;Protostomia;Spiralia;Lophotrochozoa;Mollusca;Bivalvia;Autobranchia;Heteroconchia;Euheterodonta;Imparidentia;Venerida;Veneroidea;Veneridae;Mercenaria;Mercenaria mercenaria
UdeM00000010366 6596 133.7 UdeM00000010366 XP_045176094.1 40.8 174 98 4 36 208 83 252 3.10E-28 133.7 6596 cellular organisms;Eukaryota;Opisthokonta;Metazoa;Eumetazoa;Bilateria;Protostomia;Spiralia;Lophotrochozoa;Mollusca;Bivalvia;Autobranchia;Heteroconchia;Euheterodonta;Imparidentia;Venerida;Veneroidea;Veneridae;Mercenaria;Mercenaria mercenaria
UdeM00000010367 6596 85.9 UdeM00000010367 XP_045177372.1 27.7 184 87 3 11 148 372 555 4.80E-14 85.9 6596 cellular organisms;Eukaryota;Opisthokonta;Metazoa;Eumetazoa;Bilateria;Protostomia;Spiralia;Lophotrochozoa;Mollusca;Bivalvia;Autobranchia;Heteroconchia;Euheterodonta;Imparidentia;Venerida;Veneroidea;Veneridae;Mercenaria;Mercenaria mercenaria
UdeM00000010368 6596 108.6 UdeM00000010368 XP_045176094.1 42 138 76 3 17 153 115 249 7.10E-21 108.6 6596 cellular organisms;Eukaryota;Opisthokonta;Metazoa;Eumetazoa;Bilateria;Protostomia;Spiralia;Lophotrochozoa;Mollusca;Bivalvia;Autobranchia;Heteroconchia;Euheterodonta;Imparidentia;Venerida;Veneroidea;Veneridae;Mercenaria;Mercenaria mercenaria
UdeM00000010370 6596 82.4 UdeM00000010370 XP_045176094.1 39.8 113 64 3 1 112 140 249 4.00E-13 82.4 6596 cellular organisms;Eukaryota;Opisthokonta;Metazoa;Eumetazoa;Bilateria;Protostomia;Spiralia;Lophotrochozoa;Mollusca;Bivalvia;Autobranchia;Heteroconchia;Euheterodonta;Imparidentia;Venerida;Veneroidea;Veneridae;Mercenaria;Mercenaria mercenaria
UdeM00000010369 6596 108.6 UdeM00000010369 XP_045176094.1 42 138 76 3 17 153 115 249 7.10E-21 108.6 6596 cellular organisms;Eukaryota;Opisthokonta;Metazoa;Eumetazoa;Bilateria;Protostomia;Spiralia;Lophotrochozoa;Mollusca;Bivalvia;Autobranchia;Heteroconchia;Euheterodonta;Imparidentia;Venerida;Veneroidea;Veneridae;Mercenaria;Mercenaria mercenaria
UdeM00000010371 6596 117.9 UdeM00000010371 XP_045176094.1 43.5 138 74 3 6 142 115 249 1.10E-23 117.9 6596 cellular organisms;Eukaryota;Opisthokonta;Metazoa;Eumetazoa;Bilateria;Protostomia;Spiralia;Lophotrochozoa;Mollusca;Bivalvia;Autobranchia;Heteroconchia;Euhe

terodonta;Imparidentia;Venerida;Veneroidea;Veneridae;Mercenaria;Mercenaria mercenaria
UdeM00000010373 6596 236.5 UdeM00000010373 XP_045177373.1 30.2 569 346 21 9 561 21 554 8.30E-59 236.5 6596 cellular organisms;Eukaryota;Opisthokonta;Metazoa;Eumetazoa;Bilateria;Protostomia;Spiralia;Lophotrochozoa;Mollusca;Bivalvia;Autobranchia;Heteroconchia;Euheterodonta;Imparidentia;Venerida;Veneroidea;Veneridae;Mercenaria;Mercenaria mercenaria
UdeM00000010375 6596 230.7 UdeM00000010375 XP_045187272.1 52.7 205 91 1 2 206 231 429 2.00E-57 230.7 6596 cellular organisms;Eukaryota;Opisthokonta;Metazoa;Eumetazoa;Bilateria;Protostomia;Spiralia;Lophotrochozoa;Mollusca;Bivalvia;Autobranchia;Heteroconchia;Euheterodonta;Imparidentia;Venerida;Veneroidea;Veneridae;Mercenaria;Mercenaria mercenaria
UdeM00000010374 6596 234.6 UdeM00000010374 XP_045187272.1 52.9 210 93 1 3 212 226 429 1.40E-58 234.6 6596 cellular organisms;Eukaryota;Opisthokonta;Metazoa;Eumetazoa;Bilateria;Protostomia;Spiralia;Lophotrochozoa;Mollusca;Bivalvia;Autobranchia;Heteroconchia;Euheterodonta;Imparidentia;Venerida;Veneroidea;Veneridae;Mercenaria;Mercenaria mercenaria
UdeM00000010376 6579 213.4 UdeM00000010376 XP_033736263.1 47.6 208 108 1 106 312 367 574 4.20E-52 213.4 6579 cellular organisms;Eukaryota;Opisthokonta;Metazoa;Eumetazoa;Bilateria;Protostomia;Spiralia;Lophotrochozoa;Mollusca;Bivalvia;Autobranchia;Pteriomorphia;Pectinida;Pectinoidea;Pectinidae;Pecten;Pecten maximus
UdeM00000010377 6596 211.8 UdeM00000010377 XP_045176664.1 29 496 325 10 23 500 35 521 2.10E-51 211.8 6596 cellular organisms;Eukaryota;Opisthokonta;Metazoa;Eumetazoa;Bilateria;Protostomia;Spiralia;Lophotrochozoa;Mollusca;Bivalvia;Autobranchia;Heteroconchia;Euheterodonta;Imparidentia;Venerida;Veneroidea;Veneridae;Mercenaria;Mercenaria mercenaria
UdeM00000010378 6596 127.9 UdeM00000010378 XP_045176094.1 39.2 171 100 3 27 196 95 262 1.80E-26 127.9 6596 cellular organisms;Eukaryota;Opisthokonta;Metazoa;Eumetazoa;Bilateria;Protostomia;Spiralia;Lophotrochozoa;Mollusca;Bivalvia;Autobranchia;Heteroconchia;Euheterodonta;Imparidentia;Venerida;Veneroidea;Veneridae;Mercenaria;Mercenaria mercenaria
UdeM00000010379 6596 191.8 UdeM00000010379 XP_045177373.1 29.3 540 269 23 8 455 20 538 2.00E-45 191.8 6596 cellular organisms;Eukaryota;Opisthokonta;Metazoa;Eumetazoa;Bilateria;Protostomia;Spiralia;Lophotrochozoa;Mollusca;Bivalvia;Autobranchia;Heteroconchia;Euheterodonta;Imparidentia;Venerida;Veneroidea;Veneridae;Mercenaria;Mercenaria mercenaria
UdeM00000010380 6596 471.1 UdeM00000010380 XP_045187267.1 48.2 481 240 4 1 478 84 558 1.70E-129 471.1 6596 cellular organisms;Eukaryota;Opisthokonta;Metazoa;Eumetazoa;Bilateria;Protostomia;Spiralia;Lophotrochozoa;Mollusca;Bivalvia;Autobranchia;Heteroconchia;Euheterodonta;Imparidentia;Venerida;Veneroidea;Veneridae;Mercenaria;Mercenaria mercenaria
UdeM00000010382 6579 128.3 UdeM00000010382 XP_033736263.1 51.8 112 53 1 20 130 463 574 7.40E-27 128.3 6579 cellular organisms;Eukaryota;Opisthokonta;Metazoa;Eumetazoa;Bilateria;Protostomia;Spiralia;Lophotrochozoa;Mollusca;Bivalvia;Autobranchia;Pteriomorphia;Pectinida;Pectinoidea;Pectinidae;Pecten;Pecten maximus

UdeM00000010383 6596 156.4 UdeM00000010383 XP_045214418.1 36.9 236
140 5 104 332 22 255 6.60E-35 156.4 6596 cellular
organisms;Eukaryota;Opisthokonta;Metazoa;Eumetazoa;Bilateria;Protostomia;
Spiralia;Lophotrochozoa;Mollusca;Bivalvia;Autobranchia;Heteroconchia;Euhe
terodonta;Imparidentia;Venerida;Veneroidea;Veneridae;Mercenaria;Mercenari
a mercenaria
UdeM00000010384 225164 127.5 UdeM00000010384 XP_009046505.1 47.2
142 47 1 1 114 241 382 1.10E-26 127.5 225164
cellular
organisms;Eukaryota;Opisthokonta;Metazoa;Eumetazoa;Bilateria;Protostomia;
Spiralia;Lophotrochozoa;Mollusca;Gastropoda;Patellogastropoda;Lottioidea;
Lottiidae;Lottia;Lottia gigantea
UdeM00000010385 6596 177.2 UdeM00000010385 XP_045177373.1 28.2 503
290 19 1 452 22 504 4.90E-41 177.2 6596 cellular
organisms;Eukaryota;Opisthokonta;Metazoa;Eumetazoa;Bilateria;Protostomia;
Spiralia;Lophotrochozoa;Mollusca;Bivalvia;Autobranchia;Heteroconchia;Euhe
terodonta;Imparidentia;Venerida;Veneroidea;Veneridae;Mercenaria;Mercenari
a mercenaria
UdeM00000010386 6596 227.3 UdeM00000010386 XP_045177373.1 31 545
323 23 1 520 22 538 4.80E-56 227.3 6596 cellular
organisms;Eukaryota;Opisthokonta;Metazoa;Eumetazoa;Bilateria;Protostomia;
Spiralia;Lophotrochozoa;Mollusca;Bivalvia;Autobranchia;Heteroconchia;Euhe
terodonta;Imparidentia;Venerida;Veneroidea;Veneridae;Mercenaria;Mercenari
a mercenaria
UdeM00000010388 6596 102.8 UdeM00000010388 XP_045214418.1 37.7 146
79 3 18 152 129 273 3.90E-19 102.8 6596 cellular
organisms;Eukaryota;Opisthokonta;Metazoa;Eumetazoa;Bilateria;Protostomia;
Spiralia;Lophotrochozoa;Mollusca;Bivalvia;Autobranchia;Heteroconchia;Euhe
terodonta;Imparidentia;Venerida;Veneroidea;Veneridae;Mercenaria;Mercenari
a mercenaria
UdeM00000010389 6596 219.2 UdeM00000010389 XP_045176664.1 29.3 559
340 15 9 540 21 551 1.40E-53 219.2 6596 cellular
organisms;Eukaryota;Opisthokonta;Metazoa;Eumetazoa;Bilateria;Protostomia;
Spiralia;Lophotrochozoa;Mollusca;Bivalvia;Autobranchia;Heteroconchia;Euhe
terodonta;Imparidentia;Venerida;Veneroidea;Veneridae;Mercenaria;Mercenari
a mercenaria
UdeM00000010390 6596 191 UdeM00000010390 XP_045181277.1 36.7 283
168 7 27 302 127 405 2.30E-45 191 6596 cellular
organisms;Eukaryota;Opisthokonta;Metazoa;Eumetazoa;Bilateria;Protostomia;
Spiralia;Lophotrochozoa;Mollusca;Bivalvia;Autobranchia;Heteroconchia;Euhe
terodonta;Imparidentia;Venerida;Veneroidea;Veneridae;Mercenaria;Mercenari
a mercenaria
UdeM00000010391 6596 112.8 UdeM00000010391 XP_045187267.1 51.1 88
43 0 44 131 359 446 3.30E-22 112.8 6596 cellular
organisms;Eukaryota;Opisthokonta;Metazoa;Eumetazoa;Bilateria;Protostomia;
Spiralia;Lophotrochozoa;Mollusca;Bivalvia;Autobranchia;Heteroconchia;Euhe
terodonta;Imparidentia;Venerida;Veneroidea;Veneridae;Mercenaria;Mercenari
a mercenaria
UdeM00000010392 225164 173.3 UdeM00000010392 XP_009058769.1 37.6
263 139 5 144 406 531 768 6.20E-40 173.3 225164
cellular
organisms;Eukaryota;Opisthokonta;Metazoa;Eumetazoa;Bilateria;Protostomia;
Spiralia;Lophotrochozoa;Mollusca;Gastropoda;Patellogastropoda;Lottioidea;
Lottiidae;Lottia;Lottia gigantea

UdeM00000010393 6596 74.3 UdeM00000010393 XP_045176094.1 41.2 80
47 0 1 80 140 219 8.70E-11 74.3 6596 cellular
organisms;Eukaryota;Opisthokonta;Metazoa;Eumetazoa;Bilateria;Protostomia;
Spiralia;Lophotrochozoa;Mollusca;Bivalvia;Autobranchia;Heteroconchia;Euhe
terodonta;Imparidentia;Venerida;Veneroidea;Veneridae;Mercenaria;Mercenari
a mercenaria
UdeM00000010394 1735272 80.9 UdeM00000010394 XP_041352309.1 62.1
58 22 0 2 59 301 358 6.20E-13 80.9 1735272
cellular
organisms;Eukaryota;Opisthokonta;Metazoa;Eumetazoa;Bilateria;Protostomia;
Spiralia;Lophotrochozoa;Mollusca;Gastropoda;Neomphalina;Neomphaloidea;Pel
tospiridae;Gigantopelta;Gigantopelta aegis
UdeM00000010395 225164 123.2 UdeM00000010395 XP_009046505.1 53.6
97 45 0 2 98 411 507 1.90E-25 123.2 225164
cellular
organisms;Eukaryota;Opisthokonta;Metazoa;Eumetazoa;Bilateria;Protostomia;
Spiralia;Lophotrochozoa;Mollusca;Gastropoda;Patellogastropoda;Lottioidea;
Lottiidae;Lottia;Lottia gigantea
UdeM00000010396 7054 98.6 UdeM00000010396 XP_031337031.1 33.1 166
64 2 1 119 302 467 5.70E-18 98.6 7054 cellular
organisms;Eukaryota;Opisthokonta;Metazoa;Eumetazoa;Bilateria;Protostomia;
Ecdysozoa;Panarthropoda;Arthropoda;Mandibulata;Pancrustacea;Hexapoda;Inse
cta;Dicondylia;Pterygota;Neoptera;Endopterygota;Coleoptera;Polyphaga;Elat
eriformia;Elateroidea;Lampyridae;Lampyrinae;Photinus;Photinus pyralis
UdeM00000010397 202533 190.3 UdeM00000010397 XP_035231949.1 35
331 99 4 1 217 115 443 2.70E-45 190.3 202533
cellular
organisms;Eukaryota;Opisthokonta;Metazoa;Eumetazoa;Bilateria;Protostomia;
Ecdysozoa;Panarthropoda;Arthropoda;Chelicerata;Arachnida;Araneae;Araneomo
rphae;Entelegynae;Eresoidea;Eresidae;Stegodyphus;Stegodyphus dumicola
UdeM00000010398 6596 177.6 UdeM00000010398 XP_045177372.1 26.5 563
336 19 1 530 22 539 4.40E-41 177.6 6596 cellular
organisms;Eukaryota;Opisthokonta;Metazoa;Eumetazoa;Bilateria;Protostomia;
Spiralia;Lophotrochozoa;Mollusca;Bivalvia;Autobranchia;Heteroconchia;Euhe
terodonta;Imparidentia;Venerida;Veneroidea;Veneridae;Mercenaria;Mercenari
a mercenaria
UdeM00000010399 6596 154.8 UdeM00000010399 XP_045177373.1 26.5 490
276 15 9 420 21 504 2.60E-34 154.8 6596 cellular
organisms;Eukaryota;Opisthokonta;Metazoa;Eumetazoa;Bilateria;Protostomia;
Spiralia;Lophotrochozoa;Mollusca;Bivalvia;Autobranchia;Heteroconchia;Euhe
terodonta;Imparidentia;Venerida;Veneroidea;Veneridae;Mercenaria;Mercenari
a mercenaria
UdeM00000010400 6596 88.6 UdeM00000010400 XP_045174482.1 24.5 323
206 8 9 305 21 331 1.70E-14 88.6 6596 cellular
organisms;Eukaryota;Opisthokonta;Metazoa;Eumetazoa;Bilateria;Protostomia;
Spiralia;Lophotrochozoa;Mollusca;Bivalvia;Autobranchia;Heteroconchia;Euhe
terodonta;Imparidentia;Venerida;Veneroidea;Veneridae;Mercenaria;Mercenari
a mercenaria
UdeM00000010401 6596 184.9 UdeM00000010401 XP_045176664.1 26.5 543
309 14 32 496 35 565 2.50E-43 184.9 6596 cellular
organisms;Eukaryota;Opisthokonta;Metazoa;Eumetazoa;Bilateria;Protostomia;
Spiralia;Lophotrochozoa;Mollusca;Bivalvia;Autobranchia;Heteroconchia;Euhe
terodonta;Imparidentia;Venerida;Veneroidea;Veneridae;Mercenaria;Mercenari
a mercenaria

UdeM00000010402 6579 63.2 UdeM00000010402 XP_033736263.1 45.2 62
33 1 6 66 464 525 1.60E-07 63.2 6579 cellular
organisms;Eukaryota;Opisthokonta;Metazoa;Eumetazoa;Bilateria;Protostomia;
Spiralia;Lophotrochozoa;Mollusca;Bivalvia;Autobranchia;Pteriomorphia;Pect
inida;Pectinoidea;Pectinidae;Pecten;Pecten maximus
UdeM00000010403 6596 162.2 UdeM00000010403 XP_045177373.1 26.9 506
275 16 1 487 78 507 1.70E-36 162.2 6596 cellular
organisms;Eukaryota;Opisthokonta;Metazoa;Eumetazoa;Bilateria;Protostomia;
Spiralia;Lophotrochozoa;Mollusca;Bivalvia;Autobranchia;Heteroconchia;Euhe
terodonta;Imparidentia;Venerida;Veneroidea;Veneridae;Mercenaria;Mercenari
a mercenaria
UdeM00000010404 6565 71.6 UdeM00000010404 XP_022322825.1 36.8 114
61 3 5 107 344 457 6.80E-10 71.6 6565 cellular
organisms;Eukaryota;Opisthokonta;Metazoa;Eumetazoa;Bilateria;Protostomia;
Spiralia;Lophotrochozoa;Mollusca;Bivalvia;Autobranchia;Pteriomorphia;Ostr
eida;Ostreoidea;Ostreidae;Crassostrea;Crassostrea virginica
UdeM00000010405 29159 74.3 UdeM00000010405 XP_034322772.1 54.2 59
27 0 1 59 1 59 1.70E-10 74.3 29159 cellular
organisms;Eukaryota;Opisthokonta;Metazoa;Eumetazoa;Bilateria;Protostomia;
Spiralia;Lophotrochozoa;Mollusca;Bivalvia;Autobranchia;Pteriomorphia;Ostr
eida;Ostreoidea;Ostreidae;Crassostrea;Crassostrea gigas
UdeM00000010406 6596 540.8 UdeM00000010406 XP_045188551.1 29.1 1613
1010 51 398 1958 1947 3477 8.10E-150 540.8 6596 cellular
organisms;Eukaryota;Opisthokonta;Metazoa;Eumetazoa;Bilateria;Protostomia;
Spiralia;Lophotrochozoa;Mollusca;Bivalvia;Autobranchia;Heteroconchia;Euhe
terodonta;Imparidentia;Venerida;Veneroidea;Veneridae;Mercenaria;Mercenari
a mercenaria
UdeM00000010407 6573 286.6 UdeM00000010407 XP_021355424.1 26 1267
670 44 11 1060 428 1643 1.30E-73 286.6 6573 cellular
organisms;Eukaryota;Opisthokonta;Metazoa;Eumetazoa;Bilateria;Protostomia;
Spiralia;Lophotrochozoa;Mollusca;Bivalvia;Autobranchia;Pteriomorphia;Pect
inida;Pectinoidea;Pectinidae;Mizuhopecten;Mizuhopecten yessoensis
UdeM00000010411 29159 125.9 UdeM00000010411 XP_034329356.1 59.5 116
46 1 276 390 117 232 1.10E-25 125.9 29159 cellular
organisms;Eukaryota;Opisthokonta;Metazoa;Eumetazoa;Bilateria;Protostomia;
Spiralia;Lophotrochozoa;Mollusca;Bivalvia;Autobranchia;Pteriomorphia;Ostr
eida;Ostreoidea;Ostreidae;Crassostrea;Crassostrea gigas
UdeM00000010412 29159 156 UdeM00000010412 XP_034329176.1 32.1 315
180 6 63 359 1217 1515 1.60E-34 156 29159 cellular
organisms;Eukaryota;Opisthokonta;Metazoa;Eumetazoa;Bilateria;Protostomia;
Spiralia;Lophotrochozoa;Mollusca;Bivalvia;Autobranchia;Pteriomorphia;Ostr
eida;Ostreoidea;Ostreidae;Crassostrea;Crassostrea gigas
UdeM00000010413 29159 112.8 UdeM00000010413 XP_034317326.1 44.3 131
70 2 20 148 680 809 3.70E-22 112.8 29159 cellular
organisms;Eukaryota;Opisthokonta;Metazoa;Eumetazoa;Bilateria;Protostomia;
Spiralia;Lophotrochozoa;Mollusca;Bivalvia;Autobranchia;Pteriomorphia;Ostr
eida;Ostreoidea;Ostreidae;Crassostrea;Crassostrea gigas
UdeM00000010414 6596 104.4 UdeM00000010414 XP_045190830.1 28.1 306
191 10 1 293 889 1178 3.30E-19 104.4 6596 cellular
organisms;Eukaryota;Opisthokonta;Metazoa;Eumetazoa;Bilateria;Protostomia;
Spiralia;Lophotrochozoa;Mollusca;Bivalvia;Autobranchia;Heteroconchia;Euhe
terodonta;Imparidentia;Venerida;Veneroidea;Veneridae;Mercenaria;Mercenari
a mercenaria

UdeM00000010416 45264 309.3 UdeM00000010416 XP_044165143.1 66.7 210
70 0 1 210 217 426 4.30E-81 309.3 45264 cellular
organisms;Eukaryota;Opisthokonta;Metazoa;Eumetazoa;Cnidaria;Anthozoa;Hexacorallia;Scleractinia;Astrocoeniina;Acroporidae;Acropora;Acropora millepora
UdeM00000010417 151771 220.3 UdeM00000010417 XP_028406951.1 27.9
613 301 9 2 516 97 666 5.60E-54 220.3 151771
cellular
organisms;Eukaryota;Opisthokonta;Metazoa;Eumetazoa;Cnidaria;Anthozoa;Octocorallia;Alcyonacea;Alcyoniina;Nephtheidae;Dendronephthya;Dendronephthya gigantea
UdeM00000010418 6579 449.5 UdeM00000010418 XP_033731448.1 27.3 1662
772 44 9 1623 244 1515 1.80E-122 449.5 6579 cellular
organisms;Eukaryota;Opisthokonta;Metazoa;Eumetazoa;Bilateria;Protostomia;Spiralia;Lophotrochozoa;Mollusca;Bivalvia;Autobranchia;Pteriomorphia;Pectinida;Pectinoidea;Pectinidae;Pecten;Pecten maximus
UdeM00000010419 6579 253.1 UdeM00000010419 XP_033731448.1 25.2 1300
715 45 9 1080 244 1514 1.60E-63 253.1 6579 cellular
organisms;Eukaryota;Opisthokonta;Metazoa;Eumetazoa;Bilateria;Protostomia;Spiralia;Lophotrochozoa;Mollusca;Bivalvia;Autobranchia;Pteriomorphia;Pectinida;Pectinoidea;Pectinidae;Pecten;Pecten maximus
UdeM00000010420 6596 473.4 UdeM00000010420 XP_045172093.1 32 1054
614 24 569 1567 720 1725 1.20E-129 473.4 6596 cellular
organisms;Eukaryota;Opisthokonta;Metazoa;Eumetazoa;Bilateria;Protostomia;Spiralia;Lophotrochozoa;Mollusca;Bivalvia;Autobranchia;Heteroconchia;Euheterodonta;Imparidentia;Venerida;Veneroidea;Veneridae;Mercenaria;Mercenaria mercenaria
UdeM00000010421 6596 198.7 UdeM00000010421 XP_045188486.1 52.4 187
78 4 11 187 2365 2550 6.40E-48 198.7 6596 cellular
organisms;Eukaryota;Opisthokonta;Metazoa;Eumetazoa;Bilateria;Protostomia;Spiralia;Lophotrochozoa;Mollusca;Bivalvia;Autobranchia;Heteroconchia;Euheterodonta;Imparidentia;Venerida;Veneroidea;Veneridae;Mercenaria;Mercenaria mercenaria
UdeM00000039089 6596 120.9 UdeM00000039089 XP_045162656.1 28.7 334
201 9 8 314 402 725 2.80E-24 120.9 6596 cellular
organisms;Eukaryota;Opisthokonta;Metazoa;Eumetazoa;Bilateria;Protostomia;Spiralia;Lophotrochozoa;Mollusca;Bivalvia;Autobranchia;Heteroconchia;Euheterodonta;Imparidentia;Venerida;Veneroidea;Veneridae;Mercenaria;Mercenaria mercenaria
UdeM00000039090 6596 98.6 UdeM00000039090 XP_045162610.1 30.5 210
119 5 5 197 546 745 9.50E-18 98.6 6596 cellular
organisms;Eukaryota;Opisthokonta;Metazoa;Eumetazoa;Bilateria;Protostomia;Spiralia;Lophotrochozoa;Mollusca;Bivalvia;Autobranchia;Heteroconchia;Euheterodonta;Imparidentia;Venerida;Veneroidea;Veneridae;Mercenaria;Mercenaria mercenaria
UdeM00000039091 6579 73.2 UdeM00000039091 XP_033759012.1 38.1 97
56 2 10 106 709 801 2.40E-10 73.2 6579 cellular
organisms;Eukaryota;Opisthokonta;Metazoa;Eumetazoa;Bilateria;Protostomia;Spiralia;Lophotrochozoa;Mollusca;Bivalvia;Autobranchia;Pteriomorphia;Pectinida;Pectinoidea;Pectinidae;Pecten;Pecten maximus
UdeM00000039092 45264 132.9 UdeM00000039092 XP_044184031.1 47.2 142
68 2 1 139 300 437 3.40E-28 132.9 45264 cellular
organisms;Eukaryota;Opisthokonta;Metazoa;Eumetazoa;Cnidaria;Anthozoa;Hexa

corallia;Scleractinia;Astrocoeniina;Acroporidae;Acropora;Acropora millepora
UdeM00000039093 151771 325.5 UdeM00000039093 XP_028419301.1 58.2 263 109 1 1 262 217 479 6.40E-86 325.5 151771 cellular organisms;Eukaryota;Opisthokonta;Metazoa;Eumetazoa;Cnidaria;Anthozoa;Octocorallia;Alcyonacea;Alcyoniina;Nephtheidae;Dendronephthya;Dendronephthya gigantea
UdeM00000039094 45264 138.7 UdeM00000039094 XP_044184028.1 47.6 166 82 3 42 206 68 229 8.60E-30 138.7 45264 cellular organisms;Eukaryota;Opisthokonta;Metazoa;Eumetazoa;Cnidaria;Anthozoa;Hexacorallia;Scleractinia;Astrocoeniina;Acroporidae;Acropora;Acropora millepora
UdeM00000039097 29159 365.9 UdeM00000039097 XP_034299474.1 43.2 461 249 6 1 456 178 630 7.40E-98 365.9 29159 cellular organisms;Eukaryota;Opisthokonta;Metazoa;Eumetazoa;Bilateria;Protostomia;Spiralia;Lophotrochozoa;Mollusca;Bivalvia;Autobranchia;Pteriomorphia;Ostreida;Ostreoidea;Ostreidae;Crassostrea;Crassostrea gigas
UdeM00000039098 6596 132.5 UdeM00000039098 XP_045163314.1 32.7 284 179 6 30 301 4 287 9.50E-28 132.5 6596 cellular organisms;Eukaryota;Opisthokonta;Metazoa;Eumetazoa;Bilateria;Protostomia;Spiralia;Lophotrochozoa;Mollusca;Bivalvia;Autobranchia;Heteroconchia;Euheterodonta;Imparidentia;Venerida;Veneroidea;Veneridae;Mercenaria;Mercenaria mercenaria
UdeM00000039099 48701 74.7 UdeM00000039099 XP_014842330.1 25.5 278 158 13 75 348 195 427 2.70E-10 74.7 48701 cellular organisms;Eukaryota;Opisthokonta;Metazoa;Eumetazoa;Bilateria;Deuterostomia;Chordata;Craniata;Vertebrata;Gnathostomata;Teleostomi;Euteleostomi;Actinopterygii;Actinopteri;Neopterygii;Teleostei;Osteoglossocephalai;Clupeocephala;Euteleosteomorpha;Neoteleostei;Eurypterygia;Ctenosquamata;Acanthomorphata;Euacanthomorphacea;Percomorphaceae;Ovalentaria;Atherinomorphae;Cyprinodontiformes;Cyprinodontoidei;Poeciliidae;Poeciliinae;Poecilia;Poecilia mexicana
UdeM00000039100 6565 66.2 UdeM00000039100 XP_022316733.1 67.5 40 13 0 1 40 188 227 1.90E-08 66.2 6565 cellular organisms;Eukaryota;Opisthokonta;Metazoa;Eumetazoa;Bilateria;Protostomia;Spiralia;Lophotrochozoa;Mollusca;Bivalvia;Autobranchia;Pteriomorphia;Ostreida;Ostreoidea;Ostreidae;Crassostrea;Crassostrea virginica
UdeM00000039101 6596 74.3 UdeM00000039101 XP_045208720.1 33 185 91 6 9 167 1115 1292 1.70E-10 74.3 6596 cellular organisms;Eukaryota;Opisthokonta;Metazoa;Eumetazoa;Bilateria;Protostomia;Spiralia;Lophotrochozoa;Mollusca;Bivalvia;Autobranchia;Heteroconchia;Euheterodonta;Imparidentia;Venerida;Veneroidea;Veneridae;Mercenaria;Mercenaria mercenaria
UdeM00000039102 6596 71.6 UdeM00000039102 XP_045192251.1 44.7 85 46 1 61 145 337 420 9.40E-10 71.6 6596 cellular organisms;Eukaryota;Opisthokonta;Metazoa;Eumetazoa;Bilateria;Protostomia;Spiralia;Lophotrochozoa;Mollusca;Bivalvia;Autobranchia;Heteroconchia;Euheterodonta;Imparidentia;Venerida;Veneroidea;Veneridae;Mercenaria;Mercenaria mercenaria
UdeM00000039103 6596 303.1 UdeM00000039103 XP_045162129.1 30 826 499 26 8 773 119 925 1.10E-78 303.1 6596 cellular organisms;Eukaryota;Opisthokonta;Metazoa;Eumetazoa;Bilateria;Protostomia;Spiralia;Lophotrochozoa;Mollusca;Bivalvia;Autobranchia;Heteroconchia;Euhe

terodonta;Imparidentia;Venerida;Veneroidea;Veneridae;Mercenaria;Mercenaria mercenaria
UdeM00000039105 6596 301.2 UdeM00000039105 XP_045162129.1 30.3 813 488 26 8 760 119 912 3.80E-78 301.2 6596 cellular organisms;Eukaryota;Opisthokonta;Metazoa;Eumetazoa;Bilateria;Protostomia;Spiralia;Lophotrochozoa;Mollusca;Bivalvia;Autobranchia;Heteroconchia;Euheterodonta;Imparidentia;Venerida;Veneroidea;Veneridae;Mercenaria;Mercenaria mercenaria
UdeM00000039106 114398 427.6 UdeM00000039106 XP_042903861.1 39.1 609 360 6 1 605 1 602 2.70E-116 427.6 114398 cellular organisms;Eukaryota;Opisthokonta;Metazoa;Eumetazoa;Bilateria;Protostomia;Ecdysozoa;Panarthropoda;Arthropoda;Chelicerata;Arachnida;Araneae;Araneomorphae;Entelegynae;Orbiculariae;Araneoidea;Theridiidae;Parasteatoda;Parasteatoda tepidariorum
UdeM00000039107 8187 73.2 UdeM00000039107 XP_018528006.1 27.3 209 109 11 133 336 1432 1602 7.90E-10 73.2 8187 cellular organisms;Eukaryota;Opisthokonta;Metazoa;Eumetazoa;Bilateria;Deuterostomia;Chordata;Craniata;Vertebrata;Gnathostomata;Teleostomi;Euteleostomi;Actinopterygii;Actinopteri;Neopterygii;Teleostei;Osteoglossocephalai;Clupeocephala;Euteleosteomorpha;Neoteleostei;Eurypterygia;Ctenosquamata;Acanthomorphata;Euacanthomorphacea;Percomorphaceae;Carangaria;Carangaria incertae sedis;Centropomidae;Lates;Lates calcarifer
UdeM00000039108 7054 79.3 UdeM00000039108 XP_031350480.1 46.2 78 41 1 2 78 390 467 2.40E-12 79.3 7054 cellular organisms;Eukaryota;Opisthokonta;Metazoa;Eumetazoa;Bilateria;Protostomia;Ecdysozoa;Panarthropoda;Arthropoda;Mandibulata;Pancrustacea;Hexapoda;Insecta;Dicondylia;Pterygota;Neoptera;Endopterygota;Coleoptera;Polyphaga;Elateriformia;Elateroidea;Lampyridae;Lampyrinae;Photinus;Photinus pyralis
UdeM00000039109 1234273 99 UdeM00000039109 XP_027006834.1 46 100 53 1 3 101 290 389 3.80E-18 99 1234273 cellular organisms;Eukaryota;Opisthokonta;Metazoa;Eumetazoa;Bilateria;Deuterostomia;Chordata;Craniata;Vertebrata;Gnathostomata;Teleostomi;Euteleostomi;Actinopterygii;Actinopteri;Neopterygii;Teleostei;Osteoglossocephalai;Clupeocephala;Otomorpha;Ostariophysi;Otophysi;Characiphysae;Siluriformes;Siluroidei;Bagridae;Tachysurus;Tachysurus fulvidraco
UdeM00000039110 6573 102.8 UdeM00000039110 XP_021347744.1 44.7 114 63 0 1 114 6 119 2.90E-19 102.8 6573 cellular organisms;Eukaryota;Opisthokonta;Metazoa;Eumetazoa;Bilateria;Protostomia;Spiralia;Lophotrochozoa;Mollusca;Bivalvia;Autobranchia;Pteriomorphia;Pectinida;Pectinoidea;Pectinidae;Mizuhopecten;Mizuhopecten yessoensis
UdeM00000039111 6596 99 UdeM00000039111 XP_045163314.1 36.1 180 106 5 30 200 4 183 7.40E-18 99 6596 cellular organisms;Eukaryota;Opisthokonta;Metazoa;Eumetazoa;Bilateria;Protostomia;Spiralia;Lophotrochozoa;Mollusca;Bivalvia;Autobranchia;Heteroconchia;Euheterodonta;Imparidentia;Venerida;Veneroidea;Veneridae;Mercenaria;Mercenaria mercenaria
UdeM00000039112 6596 128.3 UdeM00000039112 XP_045162610.1 25.3 407 256 11 72 458 264 642 2.60E-26 128.3 6596 cellular organisms;Eukaryota;Opisthokonta;Metazoa;Eumetazoa;Bilateria;Protostomia;Spiralia;Lophotrochozoa;Mollusca;Bivalvia;Autobranchia;Heteroconchia;Euheterodonta;Imparidentia;Venerida;Veneroidea;Veneridae;Mercenaria;Mercenaria mercenaria

UdeM00000039113 6596 133.3 UdeM00000039113 XP_045162610.1 26.6 384
252 11 72 430 264 642 7.50E-28 133.3 6596 cellular
organisms;Eukaryota;Opisthokonta;Metazoa;Eumetazoa;Bilateria;Protostomia;
Spiralia;Lophotrochozoa;Mollusca;Bivalvia;Autobranchia;Heteroconchia;Euhe
terodonta;Imparidentia;Venerida;Veneroidea;Veneridae;Mercenaria;Mercenari
a mercenaria
UdeM00000039116 6596 236.9 UdeM00000039116 XP_045162129.1 29.2 705
439 22 246 902 139 831 1.20E-58 236.9 6596 cellular
organisms;Eukaryota;Opisthokonta;Metazoa;Eumetazoa;Bilateria;Protostomia;
Spiralia;Lophotrochozoa;Mollusca;Bivalvia;Autobranchia;Heteroconchia;Euhe
terodonta;Imparidentia;Venerida;Veneroidea;Veneridae;Mercenaria;Mercenari
a mercenaria
UdeM00000039118 29159 66.2 UdeM00000039118 XP_034310431.1 65.3 49
16 1 24 71 74 122 1.90E-08 66.2 29159 cellular
organisms;Eukaryota;Opisthokonta;Metazoa;Eumetazoa;Bilateria;Protostomia;
Spiralia;Lophotrochozoa;Mollusca;Bivalvia;Autobranchia;Pteriomorphia;Ostr
eida;Ostreoidea;Ostreidae;Crassostrea;Crassostrea gigas
UdeM00000039120 6573 181.8 UdeM00000039120 XP_021358159.1 35.3 303
183 7 1 293 361 660 2.00E-42 181.8 6573 cellular
organisms;Eukaryota;Opisthokonta;Metazoa;Eumetazoa;Bilateria;Protostomia;
Spiralia;Lophotrochozoa;Mollusca;Bivalvia;Autobranchia;Pteriomorphia;Pect
inida;Pectinoidea;Pectinidae;Mizuhopecten;Mizuhopecten yessoensis
UdeM00000039121 6565 68.6 UdeM00000039121 XP_022299838.1 46.9 49
26 0 152 200 59 107 1.10E-08 68.6 6565 cellular
organisms;Eukaryota;Opisthokonta;Metazoa;Eumetazoa;Bilateria;Protostomia;
Spiralia;Lophotrochozoa;Mollusca;Bivalvia;Autobranchia;Pteriomorphia;Ostr
eida;Ostreoidea;Ostreidae;Crassostrea;Crassostrea virginica
UdeM00000039128 202533 84.7 UdeM00000039128 XP_035216523.1 32.8
125 84 0 1 125 1 125 1.30E-13 84.7 202533
cellular
organisms;Eukaryota;Opisthokonta;Metazoa;Eumetazoa;Bilateria;Protostomia;
Ecdysozoa;Panarthropoda;Arthropoda;Chelicerata;Arachnida;Araneae;Araneomo
rphae;Entelegynae;Eresoidea;Eresidae;Stegodyphus;Stegodyphus dumicola
UdeM00000039130 50429 271.2 UdeM00000039130 XP_022799197.1 29.6 597
375 13 11 580 14 592 3.20E-69 271.2 50429 cellular
organisms;Eukaryota;Opisthokonta;Metazoa;Eumetazoa;Cnidaria;Anthozoa;Hexa
corallia;Scleractinia;Astrocoeniina;Pocilloporidae;Stylophora;Stylophora
pistillata
UdeM00000039131 50429 214.9 UdeM00000039131 XP_022799197.1 29.9 418
275 7 4 414 5 411 2.00E-52 214.9 50429 cellular
organisms;Eukaryota;Opisthokonta;Metazoa;Eumetazoa;Cnidaria;Anthozoa;Hexa
corallia;Scleractinia;Astrocoeniina;Pocilloporidae;Stylophora;Stylophora
pistillata
UdeM00000039133 1529436 192.2 UdeM00000039133 XP_033123577.1 46.4
224 113 5 1 222 339 557 7.90E-46 192.2 1529436
cellular
organisms;Eukaryota;Opisthokonta;Metazoa;Eumetazoa;Bilateria;Deuterostomi
a;Echinodermata;Pelmatozoa;Crinoidea;Articulata;Comatulida;Comatulidae;Co
matulinae;Anneissia;Anneissia japonica
UdeM00000039134 6596 204.9 UdeM00000039134 XP_045177776.1 40.8 260
150 2 11 269 695 951 2.40E-49 204.9 6596 cellular
organisms;Eukaryota;Opisthokonta;Metazoa;Eumetazoa;Bilateria;Protostomia;
Spiralia;Lophotrochozoa;Mollusca;Bivalvia;Autobranchia;Heteroconchia;Euhe

terodonta;Imparidentia;Venerida;Veneroidea;Veneridae;Mercenaria;Mercenari
a mercenaria
UdeM00000039135 6596 293.9 UdeM00000039135 XP_045177776.1 37.3 418
249 6 11 425 695 1102 4.90E-76 293.9 6596 cellular
organisms;Eukaryota;Opisthokonta;Metazoa;Eumetazoa;Bilateria;Protostomia;
Spiralia;Lophotrochozoa;Mollusca;Bivalvia;Autobranchia;Heteroconchia;Euhe
terodonta;Imparidentia;Venerida;Veneroidea;Veneridae;Mercenaria;Mercenari
a mercenaria
UdeM00000039139 7741 233 UdeM00000039139 XP_019640655.1 51.1 221
107 1 39 258 1 221 4.20E-58 233 7741 cellular
organisms;Eukaryota;Opisthokonta;Metazoa;Eumetazoa;Bilateria;Deuterostomi
a;Chordata;Cephalochordata;Leptocardii;Amphioxiformes;Branchiostomidae;Br
anchiostoma;Branchiostoma belcheri
UdeM00000039140 7739 475.7 UdeM00000039140 XP_035696678.1 51.8 450
176 2 2 410 423 872 5.90E-131 475.7 7739 cellular
organisms;Eukaryota;Opisthokonta;Metazoa;Eumetazoa;Bilateria;Deuterostomi
a;Chordata;Cephalochordata;Leptocardii;Amphioxiformes;Branchiostomidae;Br
anchiostoma;Branchiostoma floridae
UdeM00000039141 6596 141 UdeM00000039141 XP_045163314.1 34.3 283
174 6 7 277 4 286 3.50E-30 141 6596 cellular
organisms;Eukaryota;Opisthokonta;Metazoa;Eumetazoa;Bilateria;Protostomia;
Spiralia;Lophotrochozoa;Mollusca;Bivalvia;Autobranchia;Heteroconchia;Euhe
terodonta;Imparidentia;Venerida;Veneroidea;Veneridae;Mercenaria;Mercenari
a mercenaria
UdeM00000039142 6596 99.4 UdeM00000039142 XP_045162663.1 33 200
126 7 27 220 337 534 6.20E-18 99.4 6596 cellular
organisms;Eukaryota;Opisthokonta;Metazoa;Eumetazoa;Bilateria;Protostomia;
Spiralia;Lophotrochozoa;Mollusca;Bivalvia;Autobranchia;Heteroconchia;Euhe
terodonta;Imparidentia;Venerida;Veneroidea;Veneridae;Mercenaria;Mercenari
a mercenaria
UdeM00000039143 34506 93.6 UdeM00000039143 XP_024499529.1 39.2 166
88 9 295 457 241 396 7.40E-16 93.6 34506 cellular
organisms;Eukaryota;Opisthokonta;Metazoa;Eumetazoa;Bilateria;Protostomia;
Ecdysozoa;Nematoda;Chromadorea;Rhabditida;Tylenchina;Panagrolaimomorpha;S
trongyloidoidea;Strongyloididae;Strongyloides;Strongyloides ratti
UdeM00000039144 34506 93.6 UdeM00000039144 XP_024499529.1 39.2 166
88 9 295 457 241 396 7.40E-16 93.6 34506 cellular
organisms;Eukaryota;Opisthokonta;Metazoa;Eumetazoa;Bilateria;Protostomia;
Ecdysozoa;Nematoda;Chromadorea;Rhabditida;Tylenchina;Panagrolaimomorpha;S
trongyloidoidea;Strongyloididae;Strongyloides;Strongyloides ratti
UdeM00000039145 6573 124 UdeM00000039145 XP_021354853.1 65.5 84
29 0 1 84 532 615 3.20E-25 124 6573 cellular
organisms;Eukaryota;Opisthokonta;Metazoa;Eumetazoa;Bilateria;Protostomia;
Spiralia;Lophotrochozoa;Mollusca;Bivalvia;Autobranchia;Pteriomorphia;Pect
inida;Pectinoidea;Pectinidae;Mizuhopecten;Mizuhopecten yessoensis
UdeM00000039147 6596 144.1 UdeM00000039147 XP_045188256.1 39.8 259
129 6 91 341 101 340 3.40E-31 144.1 6596 cellular
organisms;Eukaryota;Opisthokonta;Metazoa;Eumetazoa;Bilateria;Protostomia;
Spiralia;Lophotrochozoa;Mollusca;Bivalvia;Autobranchia;Heteroconchia;Euhe
terodonta;Imparidentia;Venerida;Veneroidea;Veneridae;Mercenaria;Mercenari
a mercenaria
UdeM00000039148 6596 151.8 UdeM00000039148 XP_045177915.1 61.8 110
41 1 1 110 234 342 5.30E-34 151.8 6596 cellular
organisms;Eukaryota;Opisthokonta;Metazoa;Eumetazoa;Bilateria;Protostomia;

Spiralia;Lophotrochozoa;Mollusca;Bivalvia;Autobranchia;Heteroconchia;Euheterodonta;Imparidentia;Venerida;Veneroidea;Veneridae;Mercenaria;Mercenaria mercenaria
UdeM00000039149 6596 275.4 UdeM00000039149 XP_045201099.1 65 200 70 0 1 200 101 300 6.00E-71 275.4 6596 cellular organisms;Eukaryota;Opisthokonta;Metazoa;Eumetazoa;Bilateria;Protostomia;Spiralia;Lophotrochozoa;Mollusca;Bivalvia;Autobranchia;Heteroconchia;Euheterodonta;Imparidentia;Venerida;Veneroidea;Veneridae;Mercenaria;Mercenaria mercenaria
UdeM00000039150 7897 76.6 UdeM00000039150 XP_014349537.1 50.7 67 33 0 28 94 52 118 1.80E-11 76.6 7897 cellular organisms;Eukaryota;Opisthokonta;Metazoa;Eumetazoa;Bilateria;Deuterostomia;Chordata;Craniata;Vertebrata;Gnathostomata;Teleostomi;Euteleostomi;Sarcopterygii;Coelacanthimorpha;Coelacanthiformes;Coelacanthidae;Latimeria;Latimeria chalumnae
UdeM00000039154 6596 211.1 UdeM00000039154 XP_045209246.1 27 708 440 29 33 688 38 720 4.60E-51 211.1 6596 cellular organisms;Eukaryota;Opisthokonta;Metazoa;Eumetazoa;Bilateria;Protostomia;Spiralia;Lophotrochozoa;Mollusca;Bivalvia;Autobranchia;Heteroconchia;Euheterodonta;Imparidentia;Venerida;Veneroidea;Veneridae;Mercenaria;Mercenaria mercenaria
UdeM00000039155 6573 485.3 UdeM00000039155 XP_021375728.1 49.7 531 189 9 1 461 1 523 8.40E-134 485.3 6573 cellular organisms;Eukaryota;Opisthokonta;Metazoa;Eumetazoa;Bilateria;Protostomia;Spiralia;Lophotrochozoa;Mollusca;Bivalvia;Autobranchia;Pteriomorphia;Pectinida;Pectinoidea;Pectinidae;Mizuhopecten;Mizuhopecten yessoensis
UdeM00000039156 6579 218.4 UdeM00000039156 XP_033739209.1 48.3 236 106 2 9 239 2 226 1.00E-53 218.4 6579 cellular organisms;Eukaryota;Opisthokonta;Metazoa;Eumetazoa;Bilateria;Protostomia;Spiralia;Lophotrochozoa;Mollusca;Bivalvia;Autobranchia;Pteriomorphia;Pectinida;Pectinoidea;Pectinidae;Pecten;Pecten maximus
UdeM00000025492 8168 89 UdeM00000025492 XP_039662215.1 31.9 160 100 4 114 270 311 464 1.20E-14 89 8168 cellular organisms;Eukaryota;Opisthokonta;Metazoa;Eumetazoa;Bilateria;Deuterostomia;Chordata;Craniata;Vertebrata;Gnathostomata;Teleostomi;Euteleostomi;Actinopterygii;Actinopteri;Neopterygii;Teleostei;Osteoglossocephalai;Clupeocephala;Euteleosteomorpha;Neoteleostei;Eurypterygia;Ctenosquamata;Acanthomorphata;Euacanthomorphacea;Percomorphaceae;Eupercaria;Perciformes;Percoidei;Percidae;Percinae;Perca;Perca fluviatilis
UdeM00000025493 303518 69.7 UdeM00000025493 XP_013763448.1 30.7 150 89 3 20 159 176 320 4.20E-09 69.7 303518 cellular organisms;Eukaryota;Opisthokonta;Metazoa;Eumetazoa;Bilateria;Deuterostomia;Chordata;Craniata;Vertebrata;Gnathostomata;Teleostomi;Euteleostomi;Actinopterygii;Actinopteri;Neopterygii;Teleostei;Osteoglossocephalai;Clupeocephala;Euteleosteomorpha;Neoteleostei;Eurypterygia;Ctenosquamata;Acanthomorphata;Euacanthomorphacea;Percomorphaceae;Ovalentaria;Cichlomorphae;Cichliformes;Cichlidae;African cichlids;Pseudocrenilabrinae;Haplochromini;Pundamilia;Pundamilia nyererei
UdeM00000025494 6596 85.1 UdeM00000025494 XP_045208720.1 33.7 258 126 11 4 228 1044 1289 1.30E-13 85.1 6596 cellular organisms;Eukaryota;Opisthokonta;Metazoa;Eumetazoa;Bilateria;Protostomia;Spiralia;Lophotrochozoa;Mollusca;Bivalvia;Autobranchia;Heteroconchia;Euhe

terodonta;Imparidentia;Venerida;Veneroidea;Veneridae;Mercenaria;Mercenaria mercenaria
UdeM00000025495 158441 174.9 UdeM00000025495 XP_021944704.1 26.7 618 378 20 11 575 229 824 3.00E-40 174.9 158441 cellular organisms;Eukaryota;Opisthokonta;Metazoa;Eumetazoa;Bilateria;Protostomia;Ecdysozoa;Panarthropoda;Arthropoda;Mandibulata;Pancrustacea;Hexapoda;Collembola;Entomobryomorpha;Isotomoidea;Isotomidae;Proisotominae;Folsomia;Folsomia candida
UdeM00000025496 158441 186.8 UdeM00000025496 XP_021944704.1 27.3 619 383 20 11 585 229 824 7.80E-44 186.8 158441 cellular organisms;Eukaryota;Opisthokonta;Metazoa;Eumetazoa;Bilateria;Protostomia;Ecdysozoa;Panarthropoda;Arthropoda;Mandibulata;Pancrustacea;Hexapoda;Collembola;Entomobryomorpha;Isotomoidea;Isotomidae;Proisotominae;Folsomia;Folsomia candida
UdeM00000025497 1735272 171.4 UdeM00000025497 XP_041378242.1 43.3 224 124 3 64 286 435 656 1.70E-39 171.4 1735272 cellular organisms;Eukaryota;Opisthokonta;Metazoa;Eumetazoa;Bilateria;Protostomia;Spiralia;Lophotrochozoa;Mollusca;Gastropoda;Neomphalina;Neomphaloidea;Peltospiridae;Gigantopelta;Gigantopelta aegis
UdeM00000025498 6573 133.7 UdeM00000025498 XP_021361564.1 33.5 272 158 6 270 535 5 259 7.30E-28 133.7 6573 cellular organisms;Eukaryota;Opisthokonta;Metazoa;Eumetazoa;Bilateria;Protostomia;Spiralia;Lophotrochozoa;Mollusca;Bivalvia;Autobranchia;Pteriomorphia;Pectinida;Pectinoidea;Pectinidae;Mizuhopecten;Mizuhopecten yessoensis
UdeM00000025499 6596 75.5 UdeM00000025499 XP_045198179.1 27.2 202 136 3 5 201 883 1078 8.80E-11 75.5 6596 cellular organisms;Eukaryota;Opisthokonta;Metazoa;Eumetazoa;Bilateria;Protostomia;Spiralia;Lophotrochozoa;Mollusca;Bivalvia;Autobranchia;Heteroconchia;Euheterodonta;Imparidentia;Venerida;Veneroidea;Veneridae;Mercenaria;Mercenaria mercenaria
UdeM00000025500 10224 355.1 UdeM00000025500 XP_006820181.1 41.9 477 258 6 1 465 164 633 2.00E-94 355.1 10224 cellular organisms;Eukaryota;Opisthokonta;Metazoa;Eumetazoa;Bilateria;Deuterostomia;Hemichordata;Enteropneusta;Harrimaniidae;Saccoglossus;Saccoglossus kowalevskii
UdeM00000025501 29159 209.1 UdeM00000025501 XP_019924327.2 24.6 708 223 9 1 408 360 1056 1.00E-50 209.1 29159 cellular organisms;Eukaryota;Opisthokonta;Metazoa;Eumetazoa;Bilateria;Protostomia;Spiralia;Lophotrochozoa;Mollusca;Bivalvia;Autobranchia;Pteriomorphia;Ostreida;Ostreoidea;Ostreidae;Crassostrea;Crassostrea gigas
UdeM00000025504 29159 395.6 UdeM00000025504 XP_019926412.2 45 496 201 8 1 435 1 485 8.60E-107 395.6 29159 cellular organisms;Eukaryota;Opisthokonta;Metazoa;Eumetazoa;Bilateria;Protostomia;Spiralia;Lophotrochozoa;Mollusca;Bivalvia;Autobranchia;Pteriomorphia;Ostreida;Ostreoidea;Ostreidae;Crassostrea;Crassostrea gigas
UdeM00000025508 202533 184.1 UdeM00000025508 XP_035216523.1 33.3 321 206 5 1 314 1 320 3.10E-43 184.1 202533 cellular organisms;Eukaryota;Opisthokonta;Metazoa;Eumetazoa;Bilateria;Protostomia;Ecdysozoa;Panarthropoda;Arthropoda;Chelicerata;Arachnida;Araneae;Araneomorphae;Entelegynae;Eresoidea;Eresidae;Stegodyphus;Stegodyphus dumicola

UdeM00000025509 50429 266.5 UdeM00000025509 XP_022799197.1 29.6 594
377 13 11 579 14 591 7.80E-68 266.5 50429 cellular
organisms;Eukaryota;Opisthokonta;Metazoa;Eumetazoa;Cnidaria;Anthozoa;Hexa
corallia;Scleractinia;Astrocoeniina;Pocilloporidae;Stylophora;Stylophora
pistillata
UdeM00000025510 114398 84.7 UdeM00000025510 XP_042913023.1 55.4
74 33 0 1 74 122 195 5.30E-14 84.7 114398
cellular
organisms;Eukaryota;Opisthokonta;Metazoa;Eumetazoa;Bilateria;Protostomia;
Ecdysozoa;Panarthropoda;Arthropoda;Chelicerata;Arachnida;Araneae;Araneomo
rphae;Entelegynae;Orbiculariae;Araneoidea;Theridiidae;Parasteatoda;Parast
eatoda tepidariorum
UdeM00000025511 1735272 62.8 UdeM00000025511 XP_041355798.1 32.4
105 70 1 20 124 52 155 5.00E-07 62.8 1735272
cellular
organisms;Eukaryota;Opisthokonta;Metazoa;Eumetazoa;Bilateria;Protostomia;
Spiralia;Lophotrochozoa;Mollusca;Gastropoda;Neomphalina;Neomphaloidea;Pel
tospiridae;Gigantopelta;Gigantopelta aegis
UdeM00000025512 1735272 96.7 UdeM00000025512 XP_041355798.1 35.4
144 91 2 20 163 52 193 3.00E-17 96.7 1735272
cellular
organisms;Eukaryota;Opisthokonta;Metazoa;Eumetazoa;Bilateria;Protostomia;
Spiralia;Lophotrochozoa;Mollusca;Gastropoda;Neomphalina;Neomphaloidea;Pel
tospiridae;Gigantopelta;Gigantopelta aegis
UdeM00000025513 50429 256.1 UdeM00000025513 XP_022799197.1 29 601
381 13 4 571 5 592 1.00E-64 256.1 50429 cellular
organisms;Eukaryota;Opisthokonta;Metazoa;Eumetazoa;Cnidaria;Anthozoa;Hexa
corallia;Scleractinia;Astrocoeniina;Pocilloporidae;Stylophora;Stylophora
pistillata
UdeM00000025514 50429 253.4 UdeM00000025514 XP_022799197.1 28.7 592
378 12 1 561 14 592 6.60E-64 253.4 50429 cellular
organisms;Eukaryota;Opisthokonta;Metazoa;Eumetazoa;Cnidaria;Anthozoa;Hexa
corallia;Scleractinia;Astrocoeniina;Pocilloporidae;Stylophora;Stylophora
pistillata
UdeM00000025517 7574 161 UdeM00000025517 XP_013401097.1 48.6 185
94 1 6 190 544 727 1.50E-36 161 7574 cellular
organisms;Eukaryota;Opisthokonta;Metazoa;Eumetazoa;Bilateria;Protostomia;
Spiralia;Lophotrochozoa;Brachiopoda;Linguliformea;Lingulata;Lingulida;Lin
guloidea;Lingulidae;Lingula;Lingula anatina
UdeM00000025518 6500 59.7 UdeM00000025518 XP_035828412.1 30.4 102
65 2 24 119 33 134 5.30E-06 59.7 6500 cellular
organisms;Eukaryota;Opisthokonta;Metazoa;Eumetazoa;Bilateria;Protostomia;
Spiralia;Lophotrochozoa;Mollusca;Gastropoda;Heterobranchia;Euthyneura;Tec
tipleura;Aplysiida;Aplysioidea;Aplysiidae;Aplysia;Aplysia californica
UdeM00000025519 6596 121.3 UdeM00000025519 XP_045180427.1 34.7 274
146 5 1 242 1 273 1.80E-24 121.3 6596 cellular
organisms;Eukaryota;Opisthokonta;Metazoa;Eumetazoa;Bilateria;Protostomia;
Spiralia;Lophotrochozoa;Mollusca;Bivalvia;Autobranchia;Heteroconchia;Euhe
terodonta;Imparidentia;Venerida;Veneroidea;Veneridae;Mercenaria;Mercenari
a mercenaria
UdeM00000025520 6596 286.2 UdeM00000025520 XP_045208720.1 33.2 584
336 17 166 734 190 734 1.20E-73 286.2 6596 cellular
organisms;Eukaryota;Opisthokonta;Metazoa;Eumetazoa;Bilateria;Protostomia;
Spiralia;Lophotrochozoa;Mollusca;Bivalvia;Autobranchia;Heteroconchia;Euhe

terodonta;Imparidentia;Venerida;Veneroidea;Veneridae;Mercenaria;Mercenaria mercenaria
UdeM00000025523 29159 485.7 UdeM00000025523 XP_034317101.1 48.8 494 227 8 30 515 116 591 7.20E-134 485.7 29159 cellular organisms;Eukaryota;Opisthokonta;Metazoa;Eumetazoa;Bilateria;Protostomia;Spiralia;Lophotrochozoa;Mollusca;Bivalvia;Autobranchia;Pteriomorphia;Ostreida;Ostreoidea;Ostreidae;Crassostrea;Crassostrea gigas
UdeM00000025524 37621 96.7 UdeM00000025524 XP_014672570.1 58 81 34 0 11 91 40 120 2.00E-17 96.7 37621 cellular organisms;Eukaryota;Opisthokonta;Metazoa;Eumetazoa;Bilateria;Protostomia;Ecdysozoa;Scalidophora;Priapulida;Priapulimorpha;Priapulimorphida;Priapulidae;Priapulus;Priapulus caudatus
UdeM00000025525 37621 104.4 UdeM00000025525 XP_014673981.1 59.1 88 36 0 1 88 76 163 1.10E-19 104.4 37621 cellular organisms;Eukaryota;Opisthokonta;Metazoa;Eumetazoa;Bilateria;Protostomia;Ecdysozoa;Scalidophora;Priapulida;Priapulimorpha;Priapulimorphida;Priapulidae;Priapulus;Priapulus caudatus
UdeM00000025526 37621 128.6 UdeM00000025526 XP_014672567.1 52.3 111 48 2 5 114 370 476 5.30E-27 128.6 37621 cellular organisms;Eukaryota;Opisthokonta;Metazoa;Eumetazoa;Bilateria;Protostomia;Ecdysozoa;Scalidophora;Priapulida;Priapulimorpha;Priapulimorphida;Priapulidae;Priapulus;Priapulus caudatus
UdeM00000025528 6596 144.8 UdeM00000025528 XP_045157297.1 53.3 122 54 2 47 168 907 1025 3.00E-31 144.8 6596 cellular organisms;Eukaryota;Opisthokonta;Metazoa;Eumetazoa;Bilateria;Protostomia;Spiralia;Lophotrochozoa;Mollusca;Bivalvia;Autobranchia;Heteroconchia;Euheterodonta;Imparidentia;Venerida;Veneroidea;Veneridae;Mercenaria;Mercenaria mercenaria
UdeM00000025530 7913 181 UdeM00000025530 XP_041082893.1 64.4 135 48 0 20 154 33 167 1.10E-42 181 7913 cellular organisms;Eukaryota;Opisthokonta;Metazoa;Eumetazoa;Bilateria;Deuterostomia;Chordata;Craniata;Vertebrata;Gnathostomata;Teleostomi;Euteleostomi;Actinopterygii;Actinopteri;Chondrostei;Acipenseriformes;Acipenseroidei;Polyodontidae;Polyodontinae;Polyodontini;Polyodon;Polyodon spathula
UdeM00000025534 29159 117.9 UdeM00000025534 XP_011451530.1 43 135 70 2 70 203 78 206 1.60E-23 117.9 29159 cellular organisms;Eukaryota;Opisthokonta;Metazoa;Eumetazoa;Bilateria;Protostomia;Spiralia;Lophotrochozoa;Mollusca;Bivalvia;Autobranchia;Pteriomorphia;Ostreida;Ostreoidea;Ostreidae;Crassostrea;Crassostrea gigas
UdeM00000025535 29159 128.6 UdeM00000025535 XP_034316164.1 56.6 99 38 2 1 97 1 96 4.70E-27 128.6 29159 cellular organisms;Eukaryota;Opisthokonta;Metazoa;Eumetazoa;Bilateria;Protostomia;Spiralia;Lophotrochozoa;Mollusca;Bivalvia;Autobranchia;Pteriomorphia;Ostreida;Ostreoidea;Ostreidae;Crassostrea;Crassostrea gigas
UdeM00000025536 6565 112.1 UdeM00000025536 XP_022312527.1 47 115 60 1 69 182 70 184 7.70E-22 112.1 6565 cellular organisms;Eukaryota;Opisthokonta;Metazoa;Eumetazoa;Bilateria;Protostomia;Spiralia;Lophotrochozoa;Mollusca;Bivalvia;Autobranchia;Pteriomorphia;Ostreida;Ostreoidea;Ostreidae;Crassostrea;Crassostrea virginica
UdeM00000025537 10224 63.5 UdeM00000025537 XP_006819348.1 40.5 74 44 0 93 166 369 442 2.80E-07 63.5 10224 cellular organisms;Eukaryota;Opisthokonta;Metazoa;Eumetazoa;Bilateria;Deuterostomia;Hemichordata;Enteropneusta;Harrimaniidae;Saccoglossus;Saccoglossus kowalevskii

UdeM00000025538	10224	63.2	UdeM00000025538	XP_006819348.1	40.5	74
44	0	55	128	369	442	2.90E-07	63.2	10224	cellular organisms;Eukaryota;Opisthokonta;Metazoa;Eumetazoa;Bilateria;Deuterostomia;Hemichordata;Enteropneusta;Harrimaniidae;Saccoglossus;Saccoglossus kowalevskii
UdeM00000025539	6565	90.1	UdeM00000025539	XP_022290516.1	50.5	103
37	2	3	91	254	356	1.60E-15	90.1	6565	cellular organisms;Eukaryota;Opisthokonta;Metazoa;Eumetazoa;Bilateria;Protostomia;Spiralia;Lophotrochozoa;Mollusca;Bivalvia;Autobranchia;Pteriomorphia;Ostreida;Ostreoidea;Ostreidae;Crassostrea;Crassostrea virginica
UdeM00000025540	6573	182.6	UdeM00000025540	XP_021356047.1	52.4	164
77	1	47	209	148	311	5.40E-43	182.6	6573	cellular organisms;Eukaryota;Opisthokonta;Metazoa;Eumetazoa;Bilateria;Protostomia;Spiralia;Lophotrochozoa;Mollusca;Bivalvia;Autobranchia;Pteriomorphia;Pectinida;Pectinoidea;Pectinidae;Mizuhopecten;Mizuhopecten yessoensis
UdeM00000025541	1735272	84.7	UdeM00000025541	XP_041349488.1	47.3
112	46	4	1	111	1	100	9.10E-14	84.7	1735272
cellular
organisms;Eukaryota;Opisthokonta;Metazoa;Eumetazoa;Bilateria;Protostomia;Spiralia;Lophotrochozoa;Mollusca;Gastropoda;Neomphalina;Neomphaloidea;Peltospiridae;Gigantopelta;Gigantopelta aegis
UdeM00000025542	6573	102.8	UdeM00000025542	XP_021351151.1	59.5	74
28	1	21	92	975	1048	6.00E-19	102.8	6573	cellular organisms;Eukaryota;Opisthokonta;Metazoa;Eumetazoa;Bilateria;Protostomia;Spiralia;Lophotrochozoa;Mollusca;Bivalvia;Autobranchia;Pteriomorphia;Pectinida;Pectinoidea;Pectinidae;Mizuhopecten;Mizuhopecten yessoensis
UdeM00000025543	6596	82.8	UdeM00000025543	XP_045208696.1	25.3	356
199	10	2	306	51	390	8.30E-13	82.8	6596	cellular organisms;Eukaryota;Opisthokonta;Metazoa;Eumetazoa;Bilateria;Protostomia;Spiralia;Lophotrochozoa;Mollusca;Bivalvia;Autobranchia;Heteroconchia;Euheterodonta;Imparidentia;Venerida;Veneroidea;Veneridae;Mercenaria;Mercenaria mercenaria
UdeM00000025544	6573	263.5	UdeM00000025544	XP_021352851.1	31.1	707
417	28	92	763	132	803	8.90E-67	263.5	6573	cellular organisms;Eukaryota;Opisthokonta;Metazoa;Eumetazoa;Bilateria;Protostomia;Spiralia;Lophotrochozoa;Mollusca;Bivalvia;Autobranchia;Pteriomorphia;Pectinida;Pectinoidea;Pectinidae;Mizuhopecten;Mizuhopecten yessoensis
UdeM00000025545	29159	211.8	UdeM00000025545	XP_034313077.1	64.4	160
57	0	2	161	107	266	6.40E-52	211.8	29159	cellular organisms;Eukaryota;Opisthokonta;Metazoa;Eumetazoa;Bilateria;Protostomia;Spiralia;Lophotrochozoa;Mollusca;Bivalvia;Autobranchia;Pteriomorphia;Ostreida;Ostreoidea;Ostreidae;Crassostrea;Crassostrea gigas
UdeM00000025547	6565	132.9	UdeM00000025547	XP_022295065.1	55	109
49	0	1	109	187	295	2.50E-28	132.9	6565	cellular organisms;Eukaryota;Opisthokonta;Metazoa;Eumetazoa;Bilateria;Protostomia;Spiralia;Lophotrochozoa;Mollusca;Bivalvia;Autobranchia;Pteriomorphia;Ostreida;Ostreoidea;Ostreidae;Crassostrea;Crassostrea virginica
UdeM00000025549	6565	252.7	UdeM00000025549	XP_022297447.1	51.2	248
105	2	1	232	117	364	4.70E-64	252.7	6565	cellular organisms;Eukaryota;Opisthokonta;Metazoa;Eumetazoa;Bilateria;Protostomia;Spiralia;Lophotrochozoa;Mollusca;Bivalvia;Autobranchia;Pteriomorphia;Ostreida;Ostreoidea;Ostreidae;Crassostrea;Crassostrea virginica
UdeM00000025550	70779	120.6	UdeM00000025550	XP_015764363.1	31.4	229
137	4	1	210	21	248	2.50E-24	120.6	70779	cellular

organisms;Eukaryota;Opisthokonta;Metazoa;Eumetazoa;Cnidaria;Anthozoa;Hexacorallia;Scleractinia;Astrocoeniina;Acroporidae;Acropora;Acropora digitifera
UdeM00000025554 202533 184.9 UdeM00000025554 XP_035216523.1 33.3 321 206 5 1 314 1 320 1.80E-43 184.9 202533 cellular organisms;Eukaryota;Opisthokonta;Metazoa;Eumetazoa;Bilateria;Protostomia;Ecdysozoa;Panarthropoda;Arthropoda;Chelicerata;Arachnida;Araneae;Araneomorphae;Entelegynae;Eresoidea;Eresidae;Stegodyphus;Stegodyphus dumicola
UdeM00000025555 50429 266.2 UdeM00000025555 XP_022799197.1 29.6 594 377 13 11 579 14 591 1.00E-67 266.2 50429 cellular organisms;Eukaryota;Opisthokonta;Metazoa;Eumetazoa;Cnidaria;Anthozoa;Hexacorallia;Scleractinia;Astrocoeniina;Pocilloporidae;Stylophora;Stylophora pistillata
UdeM00000025556 1735272 62.8 UdeM00000025556 XP_041355798.1 32.4 105 70 1 20 124 52 155 5.00E-07 62.8 1735272 cellular organisms;Eukaryota;Opisthokonta;Metazoa;Eumetazoa;Bilateria;Protostomia;Spiralia;Lophotrochozoa;Mollusca;Gastropoda;Neomphalina;Neomphaloidea;Peltospiridae;Gigantopelta;Gigantopelta aegis
UdeM00000025557 1735272 96.7 UdeM00000025557 XP_041355798.1 35.4 144 91 2 20 163 52 193 3.00E-17 96.7 1735272 cellular organisms;Eukaryota;Opisthokonta;Metazoa;Eumetazoa;Bilateria;Protostomia;Spiralia;Lophotrochozoa;Mollusca;Gastropoda;Neomphalina;Neomphaloidea;Peltospiridae;Gigantopelta;Gigantopelta aegis
UdeM00000025558 50429 256.5 UdeM00000025558 XP_022799197.1 28.8 601 382 13 4 571 5 592 8.00E-65 256.5 50429 cellular organisms;Eukaryota;Opisthokonta;Metazoa;Eumetazoa;Cnidaria;Anthozoa;Hexacorallia;Scleractinia;Astrocoeniina;Pocilloporidae;Stylophora;Stylophora pistillata
UdeM00000025559 50429 253.4 UdeM00000025559 XP_022799197.1 28.5 592 379 12 1 561 14 592 6.60E-64 253.4 50429 cellular organisms;Eukaryota;Opisthokonta;Metazoa;Eumetazoa;Cnidaria;Anthozoa;Hexacorallia;Scleractinia;Astrocoeniina;Pocilloporidae;Stylophora;Stylophora pistillata
UdeM00000025562 6565 126.7 UdeM00000025562 XP_022335449.1 72.5 80 22 0 189 268 7 86 1.00E-25 126.7 6565 cellular organisms;Eukaryota;Opisthokonta;Metazoa;Eumetazoa;Bilateria;Protostomia;Spiralia;Lophotrochozoa;Mollusca;Bivalvia;Autobranchia;Pteriomorphia;Ostreida;Ostreoidea;Ostreidae;Crassostrea;Crassostrea virginica
UdeM00000025563 6500 59.7 UdeM00000025563 XP_035828412.1 30.4 102 65 2 24 119 33 134 3.90E-06 59.7 6500 cellular organisms;Eukaryota;Opisthokonta;Metazoa;Eumetazoa;Bilateria;Protostomia;Spiralia;Lophotrochozoa;Mollusca;Gastropoda;Heterobranchia;Euthyneura;Tectipleura;Aplysiida;Aplysioidea;Aplysiidae;Aplysia;Aplysia californica
UdeM00000025564 6596 198 UdeM00000025564 XP_045181571.1 28.9 551 347 16 61 576 342 882 3.40E-47 198 6596 cellular organisms;Eukaryota;Opisthokonta;Metazoa;Eumetazoa;Bilateria;Protostomia;Spiralia;Lophotrochozoa;Mollusca;Bivalvia;Autobranchia;Heteroconchia;Euheterodonta;Imparidentia;Venerida;Veneroidea;Veneridae;Mercenaria;Mercenaria mercenaria
UdeM00000025565 37293 202.2 UdeM00000025565 XP_021527228.1 69.3 150 19 1 13 135 11 160 4.20E-49 202.2 37293 cellular

organisms;Eukaryota;Opisthokonta;Metazoa;Eumetazoa;Bilateria;Deuterostomia;Chordata;Craniata;Vertebrata;Gnathostomata;Teleostomi;Euteleostomi;Sarcopterygii;Dipnotetrapodomorpha;Tetrapoda;Amniota;Mammalia;Theria;Eutheria;Boreoeutheria;Euarchontoglires;Primates;Haplorrhini;Simiiformes;Platyrrhini;Aotidae;Aotus;Aotus nancymaae
UdeM00000025566 1735272 115.2 UdeM00000025566 XP_041379632.1 33.2 262 161 6 59 317 243 493 1.70E-22 115.2 1735272 cellular organisms;Eukaryota;Opisthokonta;Metazoa;Eumetazoa;Bilateria;Protostomia;Spiralia;Lophotrochozoa;Mollusca;Gastropoda;Neomphalina;Neomphaloidea;Peltospiridae;Gigantopelta;Gigantopelta aegis
UdeM00000030591 6596 228 UdeM00000030591 XP_045194118.1 39 372 202 10 41 399 1544 1903 2.10E-56 228 6596 cellular organisms;Eukaryota;Opisthokonta;Metazoa;Eumetazoa;Bilateria;Protostomia;Spiralia;Lophotrochozoa;Mollusca;Bivalvia;Autobranchia;Heteroconchia;Euheterodonta;Imparidentia;Venerida;Veneroidea;Veneridae;Mercenaria;Mercenaria mercenaria
UdeM00000030592 176946 66.2 UdeM00000030592 XP_015745591.2 41.2 85 43 2 1 78 2514 2598 2.90E-08 66.2 176946 cellular organisms;Eukaryota;Opisthokonta;Metazoa;Eumetazoa;Bilateria;Deuterostomia;Chordata;Craniata;Vertebrata;Gnathostomata;Teleostomi;Euteleostomi;Sarcopterygii;Dipnotetrapodomorpha;Tetrapoda;Amniota;Sauropsida;Sauria;Lepidosauria;Squamata;Bifurcata;Unidentata;Episquamata;Toxicofera;Serpentes;Henophidia;Pythonidae;Python;Python bivittatus
UdeM00000039606 6596 191.8 UdeM00000039606 XP_045161174.1 61.1 149 54 2 46 193 39 184 8.20E-46 191.8 6596 cellular organisms;Eukaryota;Opisthokonta;Metazoa;Eumetazoa;Bilateria;Protostomia;Spiralia;Lophotrochozoa;Mollusca;Bivalvia;Autobranchia;Heteroconchia;Euheterodonta;Imparidentia;Venerida;Veneroidea;Veneridae;Mercenaria;Mercenaria mercenaria
UdeM00000039607 6596 169.5 UdeM00000039607 XP_045162518.1 71.8 124 34 1 25 148 199 321 3.30E-39 169.5 6596 cellular organisms;Eukaryota;Opisthokonta;Metazoa;Eumetazoa;Bilateria;Protostomia;Spiralia;Lophotrochozoa;Mollusca;Bivalvia;Autobranchia;Heteroconchia;Euheterodonta;Imparidentia;Venerida;Veneroidea;Veneridae;Mercenaria;Mercenaria mercenaria
UdeM00000039608 6596 191.8 UdeM00000039608 XP_045161174.1 61.1 149 54 2 46 193 39 184 8.20E-46 191.8 6596 cellular organisms;Eukaryota;Opisthokonta;Metazoa;Eumetazoa;Bilateria;Protostomia;Spiralia;Lophotrochozoa;Mollusca;Bivalvia;Autobranchia;Heteroconchia;Euheterodonta;Imparidentia;Venerida;Veneroidea;Veneridae;Mercenaria;Mercenaria mercenaria
UdeM00000039609 6596 169.5 UdeM00000039609 XP_045162518.1 71.8 124 34 1 25 148 199 321 3.30E-39 169.5 6596 cellular organisms;Eukaryota;Opisthokonta;Metazoa;Eumetazoa;Bilateria;Protostomia;Spiralia;Lophotrochozoa;Mollusca;Bivalvia;Autobranchia;Heteroconchia;Euheterodonta;Imparidentia;Venerida;Veneroidea;Veneridae;Mercenaria;Mercenaria mercenaria
UdeM00000039610 6596 191.8 UdeM00000039610 XP_045161174.1 61.1 149 54 2 46 193 39 184 8.20E-46 191.8 6596 cellular organisms;Eukaryota;Opisthokonta;Metazoa;Eumetazoa;Bilateria;Protostomia;Spiralia;Lophotrochozoa;Mollusca;Bivalvia;Autobranchia;Heteroconchia;Euhe

terodonta;Imparidentia;Venerida;Veneroidea;Veneridae;Mercenaria;Mercenaria mercenaria
UdeM00000039611 6596 169.5 UdeM00000039611 XP_045162518.1 71.8 124 34 1 25 148 199 321 3.30E-39 169.5 6596 cellular organisms;Eukaryota;Opisthokonta;Metazoa;Eumetazoa;Bilateria;Protostomia;Spiralia;Lophotrochozoa;Mollusca;Bivalvia;Autobranchia;Heteroconchia;Euheterodonta;Imparidentia;Venerida;Veneroidea;Veneridae;Mercenaria;Mercenaria mercenaria
UdeM00000039612 6596 191.8 UdeM00000039612 XP_045161174.1 61.1 149 54 2 46 193 39 184 8.20E-46 191.8 6596 cellular organisms;Eukaryota;Opisthokonta;Metazoa;Eumetazoa;Bilateria;Protostomia;Spiralia;Lophotrochozoa;Mollusca;Bivalvia;Autobranchia;Heteroconchia;Euheterodonta;Imparidentia;Venerida;Veneroidea;Veneridae;Mercenaria;Mercenaria mercenaria
UdeM00000039613 6596 169.5 UdeM00000039613 XP_045162518.1 71.8 124 34 1 25 148 199 321 3.30E-39 169.5 6596 cellular organisms;Eukaryota;Opisthokonta;Metazoa;Eumetazoa;Bilateria;Protostomia;Spiralia;Lophotrochozoa;Mollusca;Bivalvia;Autobranchia;Heteroconchia;Euheterodonta;Imparidentia;Venerida;Veneroidea;Veneridae;Mercenaria;Mercenaria mercenaria
UdeM00000039614 6596 191.8 UdeM00000039614 XP_045161174.1 61.1 149 54 2 46 193 39 184 8.20E-46 191.8 6596 cellular organisms;Eukaryota;Opisthokonta;Metazoa;Eumetazoa;Bilateria;Protostomia;Spiralia;Lophotrochozoa;Mollusca;Bivalvia;Autobranchia;Heteroconchia;Euheterodonta;Imparidentia;Venerida;Veneroidea;Veneridae;Mercenaria;Mercenaria mercenaria
UdeM00000039615 6596 169.5 UdeM00000039615 XP_045162518.1 71.8 124 34 1 25 148 199 321 3.30E-39 169.5 6596 cellular organisms;Eukaryota;Opisthokonta;Metazoa;Eumetazoa;Bilateria;Protostomia;Spiralia;Lophotrochozoa;Mollusca;Bivalvia;Autobranchia;Heteroconchia;Euheterodonta;Imparidentia;Venerida;Veneroidea;Veneridae;Mercenaria;Mercenaria mercenaria
UdeM00000039616 6596 191.8 UdeM00000039616 XP_045161174.1 61.1 149 54 2 46 193 39 184 8.20E-46 191.8 6596 cellular organisms;Eukaryota;Opisthokonta;Metazoa;Eumetazoa;Bilateria;Protostomia;Spiralia;Lophotrochozoa;Mollusca;Bivalvia;Autobranchia;Heteroconchia;Euheterodonta;Imparidentia;Venerida;Veneroidea;Veneridae;Mercenaria;Mercenaria mercenaria
UdeM00000039617 6596 169.5 UdeM00000039617 XP_045162518.1 67.4 135 33 1 25 148 199 333 3.30E-39 169.5 6596 cellular organisms;Eukaryota;Opisthokonta;Metazoa;Eumetazoa;Bilateria;Protostomia;Spiralia;Lophotrochozoa;Mollusca;Bivalvia;Autobranchia;Heteroconchia;Euheterodonta;Imparidentia;Venerida;Veneroidea;Veneridae;Mercenaria;Mercenaria mercenaria
UdeM00000039618 6596 191.8 UdeM00000039618 XP_045161174.1 61.1 149 54 2 46 193 39 184 8.20E-46 191.8 6596 cellular organisms;Eukaryota;Opisthokonta;Metazoa;Eumetazoa;Bilateria;Protostomia;Spiralia;Lophotrochozoa;Mollusca;Bivalvia;Autobranchia;Heteroconchia;Euheterodonta;Imparidentia;Venerida;Veneroidea;Veneridae;Mercenaria;Mercenaria mercenaria
UdeM00000039619 6596 169.5 UdeM00000039619 XP_045162518.1 71.8 124 34 1 25 148 199 321 3.30E-39 169.5 6596 cellular organisms;Eukaryota;Opisthokonta;Metazoa;Eumetazoa;Bilateria;Protostomia;Spiralia;Lophotrochozoa;Mollusca;Bivalvia;Autobranchia;Heteroconchia;Euhe

terodonta;Imparidentia;Venerida;Veneroidea;Veneridae;Mercenaria;Mercenari
a mercenaria
UdeM00000039620 6596 191.8 UdeM00000039620 XP_045161174.1 61.1 149
54 2 46 193 39 184 8.20E-46 191.8 6596 cellular
organisms;Eukaryota;Opisthokonta;Metazoa;Eumetazoa;Bilateria;Protostomia;
Spiralia;Lophotrochozoa;Mollusca;Bivalvia;Autobranchia;Heteroconchia;Euhe
terodonta;Imparidentia;Venerida;Veneroidea;Veneridae;Mercenaria;Mercenari
a mercenaria
UdeM00000039621 6596 169.5 UdeM00000039621 XP_045162518.1 71.8 124
34 1 25 148 199 321 3.30E-39 169.5 6596 cellular
organisms;Eukaryota;Opisthokonta;Metazoa;Eumetazoa;Bilateria;Protostomia;
Spiralia;Lophotrochozoa;Mollusca;Bivalvia;Autobranchia;Heteroconchia;Euhe
terodonta;Imparidentia;Venerida;Veneroidea;Veneridae;Mercenaria;Mercenari
a mercenaria
UdeM00000039622 6596 191.8 UdeM00000039622 XP_045161174.1 61.1 149
54 2 46 193 39 184 8.20E-46 191.8 6596 cellular
organisms;Eukaryota;Opisthokonta;Metazoa;Eumetazoa;Bilateria;Protostomia;
Spiralia;Lophotrochozoa;Mollusca;Bivalvia;Autobranchia;Heteroconchia;Euhe
terodonta;Imparidentia;Venerida;Veneroidea;Veneridae;Mercenaria;Mercenari
a mercenaria
UdeM00000039623 6596 169.5 UdeM00000039623 XP_045162518.1 71.8 124
34 1 25 148 199 321 3.30E-39 169.5 6596 cellular
organisms;Eukaryota;Opisthokonta;Metazoa;Eumetazoa;Bilateria;Protostomia;
Spiralia;Lophotrochozoa;Mollusca;Bivalvia;Autobranchia;Heteroconchia;Euhe
terodonta;Imparidentia;Venerida;Veneroidea;Veneridae;Mercenaria;Mercenari
a mercenaria
UdeM00000039624 6596 191.4 UdeM00000039624 XP_045161175.1 66.2 133
44 1 62 193 12 144 1.10E-45 191.4 6596 cellular
organisms;Eukaryota;Opisthokonta;Metazoa;Eumetazoa;Bilateria;Protostomia;
Spiralia;Lophotrochozoa;Mollusca;Bivalvia;Autobranchia;Heteroconchia;Euhe
terodonta;Imparidentia;Venerida;Veneroidea;Veneridae;Mercenaria;Mercenari
a mercenaria
UdeM00000039625 6596 169.5 UdeM00000039625 XP_045162518.1 71.8 124
34 1 25 148 199 321 3.30E-39 169.5 6596 cellular
organisms;Eukaryota;Opisthokonta;Metazoa;Eumetazoa;Bilateria;Protostomia;
Spiralia;Lophotrochozoa;Mollusca;Bivalvia;Autobranchia;Heteroconchia;Euhe
terodonta;Imparidentia;Venerida;Veneroidea;Veneridae;Mercenaria;Mercenari
a mercenaria
UdeM00000039626 6596 191.8 UdeM00000039626 XP_045161174.1 61.1 149
54 2 46 193 39 184 8.20E-46 191.8 6596 cellular
organisms;Eukaryota;Opisthokonta;Metazoa;Eumetazoa;Bilateria;Protostomia;
Spiralia;Lophotrochozoa;Mollusca;Bivalvia;Autobranchia;Heteroconchia;Euhe
terodonta;Imparidentia;Venerida;Veneroidea;Veneridae;Mercenaria;Mercenari
a mercenaria
UdeM00000039627 6596 169.5 UdeM00000039627 XP_045162518.1 71.8 124
34 1 25 148 199 321 3.30E-39 169.5 6596 cellular
organisms;Eukaryota;Opisthokonta;Metazoa;Eumetazoa;Bilateria;Protostomia;
Spiralia;Lophotrochozoa;Mollusca;Bivalvia;Autobranchia;Heteroconchia;Euhe
terodonta;Imparidentia;Venerida;Veneroidea;Veneridae;Mercenaria;Mercenari
a mercenaria
UdeM00000039628 6596 191.8 UdeM00000039628 XP_045161174.1 61.1 149
54 2 46 193 39 184 8.20E-46 191.8 6596 cellular
organisms;Eukaryota;Opisthokonta;Metazoa;Eumetazoa;Bilateria;Protostomia;
Spiralia;Lophotrochozoa;Mollusca;Bivalvia;Autobranchia;Heteroconchia;Euhe

terodonta;Imparidentia;Venerida;Veneroidea;Veneridae;Mercenaria;Mercenaria mercenaria
UdeM00000039629 6596 169.5 UdeM00000039629 XP_045162518.1 71.8 124 34 1 25 148 199 321 3.30E-39 169.5 6596 cellular organisms;Eukaryota;Opisthokonta;Metazoa;Eumetazoa;Bilateria;Protostomia;Spiralia;Lophotrochozoa;Mollusca;Bivalvia;Autobranchia;Heteroconchia;Euheterodonta;Imparidentia;Venerida;Veneroidea;Veneridae;Mercenaria;Mercenaria mercenaria
UdeM00000039630 6596 191.8 UdeM00000039630 XP_045161174.1 61.1 149 54 2 46 193 39 184 8.20E-46 191.8 6596 cellular organisms;Eukaryota;Opisthokonta;Metazoa;Eumetazoa;Bilateria;Protostomia;Spiralia;Lophotrochozoa;Mollusca;Bivalvia;Autobranchia;Heteroconchia;Euheterodonta;Imparidentia;Venerida;Veneroidea;Veneridae;Mercenaria;Mercenaria mercenaria
UdeM00000039631 6596 169.1 UdeM00000039631 XP_045162518.1 71.8 124 34 1 386 509 199 321 1.50E-38 169.1 6596 cellular organisms;Eukaryota;Opisthokonta;Metazoa;Eumetazoa;Bilateria;Protostomia;Spiralia;Lophotrochozoa;Mollusca;Bivalvia;Autobranchia;Heteroconchia;Euheterodonta;Imparidentia;Venerida;Veneroidea;Veneridae;Mercenaria;Mercenaria mercenaria
UdeM00000039632 6596 191.8 UdeM00000039632 XP_045161174.1 61.1 149 54 2 46 193 39 184 8.20E-46 191.8 6596 cellular organisms;Eukaryota;Opisthokonta;Metazoa;Eumetazoa;Bilateria;Protostomia;Spiralia;Lophotrochozoa;Mollusca;Bivalvia;Autobranchia;Heteroconchia;Euheterodonta;Imparidentia;Venerida;Veneroidea;Veneridae;Mercenaria;Mercenaria mercenaria
UdeM00000039633 6596 169.5 UdeM00000039633 XP_045162518.1 71.8 124 34 1 25 148 199 321 3.30E-39 169.5 6596 cellular organisms;Eukaryota;Opisthokonta;Metazoa;Eumetazoa;Bilateria;Protostomia;Spiralia;Lophotrochozoa;Mollusca;Bivalvia;Autobranchia;Heteroconchia;Euheterodonta;Imparidentia;Venerida;Veneroidea;Veneridae;Mercenaria;Mercenaria mercenaria
UdeM00000039634 6596 191.8 UdeM00000039634 XP_045161174.1 61.1 149 54 2 46 193 39 184 8.20E-46 191.8 6596 cellular organisms;Eukaryota;Opisthokonta;Metazoa;Eumetazoa;Bilateria;Protostomia;Spiralia;Lophotrochozoa;Mollusca;Bivalvia;Autobranchia;Heteroconchia;Euheterodonta;Imparidentia;Venerida;Veneroidea;Veneridae;Mercenaria;Mercenaria mercenaria
UdeM00000039635 6596 169.5 UdeM00000039635 XP_045162518.1 71.8 124 34 1 25 148 199 321 3.30E-39 169.5 6596 cellular organisms;Eukaryota;Opisthokonta;Metazoa;Eumetazoa;Bilateria;Protostomia;Spiralia;Lophotrochozoa;Mollusca;Bivalvia;Autobranchia;Heteroconchia;Euheterodonta;Imparidentia;Venerida;Veneroidea;Veneridae;Mercenaria;Mercenaria mercenaria
UdeM00000019967 6596 127.5 UdeM00000019967 XP_045161434.1 50.8 130 64 0 64 193 98 227 2.00E-26 127.5 6596 cellular organisms;Eukaryota;Opisthokonta;Metazoa;Eumetazoa;Bilateria;Protostomia;Spiralia;Lophotrochozoa;Mollusca;Bivalvia;Autobranchia;Heteroconchia;Euheterodonta;Imparidentia;Venerida;Veneroidea;Veneridae;Mercenaria;Mercenaria mercenaria
UdeM00000019968 6596 649.8 UdeM00000019968 XP_045160149.1 31.8 1588 644 29 6 1197 3 1547 6.80E-183 649.8 6596 cellular organisms;Eukaryota;Opisthokonta;Metazoa;Eumetazoa;Bilateria;Protostomia;Spiralia;Lophotrochozoa;Mollusca;Bivalvia;Autobranchia;Heteroconchia;Euhe

terodonta;Imparidentia;Venerida;Veneroidea;Veneridae;Mercenaria;Mercenaria mercenaria
UdeM00000019969 29159 310.5 UdeM00000019969 XP_034332465.1 47.1 363 180 5 24 375 204 565 3.90E-81 310.5 29159 cellular organisms;Eukaryota;Opisthokonta;Metazoa;Eumetazoa;Bilateria;Protostomia;Spiralia;Lophotrochozoa;Mollusca;Bivalvia;Autobranchia;Pteriomorphia;Ostreida;Ostreoidea;Ostreidae;Crassostrea;Crassostrea gigas
UdeM00000019970 29159 131 UdeM00000019970 XP_034332465.1 45.1 144 73 2 77 219 74 212 1.90E-27 131 29159 cellular organisms;Eukaryota;Opisthokonta;Metazoa;Eumetazoa;Bilateria;Protostomia;Spiralia;Lophotrochozoa;Mollusca;Bivalvia;Autobranchia;Pteriomorphia;Ostreida;Ostreoidea;Ostreidae;Crassostrea;Crassostrea gigas
UdeM00000019971 29159 444.5 UdeM00000019971 XP_034332465.1 49.3 475 226 6 5 470 74 542 2.00E-121 444.5 29159 cellular organisms;Eukaryota;Opisthokonta;Metazoa;Eumetazoa;Bilateria;Protostomia;Spiralia;Lophotrochozoa;Mollusca;Bivalvia;Autobranchia;Pteriomorphia;Ostreida;Ostreoidea;Ostreidae;Crassostrea;Crassostrea gigas
UdeM00000019973 29159 442.6 UdeM00000019973 XP_034332465.1 48 498 241 7 77 562 74 565 8.80E-121 442.6 29159 cellular organisms;Eukaryota;Opisthokonta;Metazoa;Eumetazoa;Bilateria;Protostomia;Spiralia;Lophotrochozoa;Mollusca;Bivalvia;Autobranchia;Pteriomorphia;Ostreida;Ostreoidea;Ostreidae;Crassostrea;Crassostrea gigas
UdeM00000019975 29159 424.9 UdeM00000019975 XP_034332465.1 48.8 475 228 7 77 542 74 542 2.00E-115 424.9 29159 cellular organisms;Eukaryota;Opisthokonta;Metazoa;Eumetazoa;Bilateria;Protostomia;Spiralia;Lophotrochozoa;Mollusca;Bivalvia;Autobranchia;Pteriomorphia;Ostreida;Ostreoidea;Ostreidae;Crassostrea;Crassostrea gigas
UdeM00000019976 29159 437.6 UdeM00000019976 XP_034332465.1 48.1 493 237 7 77 560 74 556 3.10E-119 437.6 29159 cellular organisms;Eukaryota;Opisthokonta;Metazoa;Eumetazoa;Bilateria;Protostomia;Spiralia;Lophotrochozoa;Mollusca;Bivalvia;Autobranchia;Pteriomorphia;Ostreida;Ostreoidea;Ostreidae;Crassostrea;Crassostrea gigas
UdeM00000019977 6596 164.1 UdeM00000019977 XP_045160157.1 38.2 267 137 5 6 249 3 264 2.40E-37 164.1 6596 cellular organisms;Eukaryota;Opisthokonta;Metazoa;Eumetazoa;Bilateria;Protostomia;Spiralia;Lophotrochozoa;Mollusca;Bivalvia;Autobranchia;Heteroconchia;Euheterodonta;Imparidentia;Venerida;Veneroidea;Veneridae;Mercenaria;Mercenaria mercenaria
UdeM00000019980 29159 352.1 UdeM00000019980 XP_034332465.1 51.7 362 166 5 14 367 182 542 1.10E-93 352.1 29159 cellular organisms;Eukaryota;Opisthokonta;Metazoa;Eumetazoa;Bilateria;Protostomia;Spiralia;Lophotrochozoa;Mollusca;Bivalvia;Autobranchia;Pteriomorphia;Ostreida;Ostreoidea;Ostreidae;Crassostrea;Crassostrea gigas
UdeM00000019981 37621 497.7 UdeM00000019981 XP_014678940.1 43.5 591 312 8 93 663 367 955 2.40E-137 497.7 37621 cellular organisms;Eukaryota;Opisthokonta;Metazoa;Eumetazoa;Bilateria;Protostomia;Ecdysozoa;Scalidophora;Priapulida;Priapulimorpha;Priapulimorphida;Priapulidae;Priapulus;Priapulus caudatus
UdeM00000019983 37621 500.4 UdeM00000019983 XP_014678940.1 43.7 591 311 8 93 663 367 955 3.70E-138 500.4 37621 cellular organisms;Eukaryota;Opisthokonta;Metazoa;Eumetazoa;Bilateria;Protostomia;Ecdysozoa;Scalidophora;Priapulida;Priapulimorpha;Priapulimorphida;Priapulidae;Priapulus;Priapulus caudatus

UdeM00000019984 29159 109.8 UdeM00000019984 XP_011440881.2 43.2 148 80 1 27 170 8 155 3.80E-21 109.8 29159 cellular organisms;Eukaryota;Opisthokonta;Metazoa;Eumetazoa;Bilateria;Protostomia;Spiralia;Lophotrochozoa;Mollusca;Bivalvia;Autobranchia;Pteriomorphia;Ostreida;Ostreoidea;Ostreidae;Crassostrea;Crassostrea gigas
UdeM00000019985 29159 99 UdeM00000019985 XP_011440881.2 48 123 63 1 48 169 33 155 7.20E-18 99 29159 cellular organisms;Eukaryota;Opisthokonta;Metazoa;Eumetazoa;Bilateria;Protostomia;Spiralia;Lophotrochozoa;Mollusca;Bivalvia;Autobranchia;Pteriomorphia;Ostreida;Ostreoidea;Ostreidae;Crassostrea;Crassostrea gigas
UdeM00000019986 6596 79.7 UdeM00000019986 XP_045161434.1 36 125 80 0 46 170 100 224 4.10E-12 79.7 6596 cellular organisms;Eukaryota;Opisthokonta;Metazoa;Eumetazoa;Bilateria;Protostomia;Spiralia;Lophotrochozoa;Mollusca;Bivalvia;Autobranchia;Heteroconchia;Euheterodonta;Imparidentia;Venerida;Veneroidea;Veneridae;Mercenaria;Mercenaria mercenaria
UdeM00000019987 6596 98.6 UdeM00000019987 XP_045161434.1 42.5 146 80 2 38 181 94 237 8.80E-18 98.6 6596 cellular organisms;Eukaryota;Opisthokonta;Metazoa;Eumetazoa;Bilateria;Protostomia;Spiralia;Lophotrochozoa;Mollusca;Bivalvia;Autobranchia;Heteroconchia;Euheterodonta;Imparidentia;Venerida;Veneroidea;Veneridae;Mercenaria;Mercenaria mercenaria
UdeM00000019988 150288 166 UdeM00000019988 XP_020788097.1 66 106 36 0 2 107 383 488 2.60E-38 166 150288 cellular organisms;Eukaryota;Opisthokonta;Metazoa;Eumetazoa;Bilateria;Deuterostomia;Chordata;Craniata;Vertebrata;Gnathostomata;Teleostomi;Euteleostomi;Actinopterygii;Actinopteri;Neopterygii;Teleostei;Osteoglossocephalai;Clupeocephala;Euteleosteomorpha;Neoteleostei;Eurypterygia;Ctenosquamata;Acanthomorphata;Euacanthomorphacea;Percomorphaceae;Gobiaria;Gobiiformes;Gobioidei;Gobiidae;Oxudercinae;Boleophthalmus;Boleophthalmus pectinirostris
UdeM00000019989 50390 319.7 UdeM00000019989 XP_028142380.1 57.3 234 99 1 2 235 170 402 3.10E-84 319.7 50390 cellular organisms;Eukaryota;Opisthokonta;Metazoa;Eumetazoa;Bilateria;Protostomia;Ecdysozoa;Panarthropoda;Arthropoda;Mandibulata;Pancrustacea;Hexapoda;Insecta;Dicondylia;Pterygota;Neoptera;Endopterygota;Coleoptera;Polyphaga;Cucujiformia;Chrysomeloidea;Chrysomelidae;Galerucinae;Luperini;Diabroticina;Diabroticites;Diabrotica;Diabrotica virgifera;Diabrotica virgifera virgifera
UdeM00000019990 29159 72 UdeM00000019990 XP_011440881.2 37.2 129 80 1 43 171 28 155 1.00E-09 72 29159 cellular organisms;Eukaryota;Opisthokonta;Metazoa;Eumetazoa;Bilateria;Protostomia;Spiralia;Lophotrochozoa;Mollusca;Bivalvia;Autobranchia;Pteriomorphia;Ostreida;Ostreoidea;Ostreidae;Crassostrea;Crassostrea gigas
UdeM00000019992 29159 79.7 UdeM00000019992 XP_011440881.2 35.1 134 87 0 41 174 26 159 4.20E-12 79.7 29159 cellular organisms;Eukaryota;Opisthokonta;Metazoa;Eumetazoa;Bilateria;Protostomia;Spiralia;Lophotrochozoa;Mollusca;Bivalvia;Autobranchia;Pteriomorphia;Ostreida;Ostreoidea;Ostreidae;Crassostrea;Crassostrea gigas
UdeM00000019993 29159 72.8 UdeM00000019993 XP_011440881.2 33.6 134 89 0 41 174 26 159 5.20E-10 72.8 29159 cellular organisms;Eukaryota;Opisthokonta;Metazoa;Eumetazoa;Bilateria;Protostomia;Spiralia;Lophotrochozoa;Mollusca;Bivalvia;Autobranchia;Pteriomorphia;Ostreida;Ostreoidea;Ostreidae;Crassostrea;Crassostrea gigas

UdeM00000019994	29159	105.9	UdeM00000019994	XP_011440881.2	54.9	113
51	0	61	173	46	158	5.50E-20	105.9	29159	cellular organisms;Eukaryota;Opisthokonta;Metazoa;Eumetazoa;Bilateria;Protostomia;Spiralia;Lophotrochozoa;Mollusca;Bivalvia;Autobranchia;Pteriomorphia;Ostreida;Ostreoidea;Ostreidae;Crassostrea;Crassostrea gigas
UdeM00000019995	6573	476.9	UdeM00000019995	XP_021368737.1	59.7	1346
445	27	90	1377	1	1307	8.90E-131	476.9	6573	cellular organisms;Eukaryota;Opisthokonta;Metazoa;Eumetazoa;Bilateria;Protostomia;Spiralia;Lophotrochozoa;Mollusca;Bivalvia;Autobranchia;Pteriomorphia;Pectinida;Pectinoidea;Pectinidae;Mizuhopecten;Mizuhopecten yessoensis
UdeM00000019996	6596	481.5	UdeM00000019996	XP_045162753.1	57.3	405
162	4	40	443	34	428	1.20E-132	481.5	6596	cellular organisms;Eukaryota;Opisthokonta;Metazoa;Eumetazoa;Bilateria;Protostomia;Spiralia;Lophotrochozoa;Mollusca;Bivalvia;Autobranchia;Heteroconchia;Euheterodonta;Imparidentia;Venerida;Veneroidea;Veneridae;Mercenaria;Mercenaria mercenaria
UdeM00000019997	29159	128.6	UdeM00000019997	XP_034307878.1	32.6	178
120	0	70	247	6	183	1.20E-26	128.6	29159	cellular organisms;Eukaryota;Opisthokonta;Metazoa;Eumetazoa;Bilateria;Protostomia;Spiralia;Lophotrochozoa;Mollusca;Bivalvia;Autobranchia;Pteriomorphia;Ostreida;Ostreoidea;Ostreidae;Crassostrea;Crassostrea gigas
UdeM00000020000	6596	106.7	UdeM00000020000	XP_045161226.1	38.5	143
72	5	7	143	86	218	3.20E-20	106.7	6596	cellular organisms;Eukaryota;Opisthokonta;Metazoa;Eumetazoa;Bilateria;Protostomia;Spiralia;Lophotrochozoa;Mollusca;Bivalvia;Autobranchia;Heteroconchia;Euheterodonta;Imparidentia;Venerida;Veneroidea;Veneridae;Mercenaria;Mercenaria mercenaria
UdeM00000020001	6579	275.4	UdeM00000020001	XP_033732603.1	50.5	279
137	1	9	287	8	285	8.90E-71	275.4	6579	cellular organisms;Eukaryota;Opisthokonta;Metazoa;Eumetazoa;Bilateria;Protostomia;Spiralia;Lophotrochozoa;Mollusca;Bivalvia;Autobranchia;Pteriomorphia;Pectinida;Pectinoidea;Pectinidae;Pecten;Pecten maximus
UdeM00000020002	6596	341.3	UdeM00000020002	XP_045163591.1	63.7	256
91	2	1	255	10	264	1.10E-90	341.3	6596	cellular organisms;Eukaryota;Opisthokonta;Metazoa;Eumetazoa;Bilateria;Protostomia;Spiralia;Lophotrochozoa;Mollusca;Bivalvia;Autobranchia;Heteroconchia;Euheterodonta;Imparidentia;Venerida;Veneroidea;Veneridae;Mercenaria;Mercenaria mercenaria
UdeM00000020003	6596	303.9	UdeM00000020003	XP_045163591.1	67.9	212
67	1	2	213	54	264	1.60E-79	303.9	6596	cellular organisms;Eukaryota;Opisthokonta;Metazoa;Eumetazoa;Bilateria;Protostomia;Spiralia;Lophotrochozoa;Mollusca;Bivalvia;Autobranchia;Heteroconchia;Euheterodonta;Imparidentia;Venerida;Veneroidea;Veneridae;Mercenaria;Mercenaria mercenaria
UdeM00000020004	6565	209.9	UdeM00000020004	XP_022317300.1	33.6	387
247	4	17	393	25	411	5.80E-51	209.9	6565	cellular organisms;Eukaryota;Opisthokonta;Metazoa;Eumetazoa;Bilateria;Protostomia;Spiralia;Lophotrochozoa;Mollusca;Bivalvia;Autobranchia;Pteriomorphia;Ostreida;Ostreoidea;Ostreidae;Crassostrea;Crassostrea virginica
UdeM00000020005	6573	209.5	UdeM00000020005	XP_021363743.1	34.4	299
192	2	2	297	16	313	7.30E-51	209.5	6573	cellular organisms;Eukaryota;Opisthokonta;Metazoa;Eumetazoa;Bilateria;Protostomia;Spiralia;Lophotrochozoa;Mollusca;Bivalvia;Autobranchia;Pteriomorphia;Pectinida;Pectinoidea;Pectinidae;Mizuhopecten;Mizuhopecten yessoensis

UdeM00000020006 6596 136.7 UdeM00000020006 XP_045208720.1 30.9 307
194 7 106 401 432 731 8.90E-29 136.7 6596 cellular
organisms;Eukaryota;Opisthokonta;Metazoa;Eumetazoa;Bilateria;Protostomia;
Spiralia;Lophotrochozoa;Mollusca;Bivalvia;Autobranchia;Heteroconchia;Euhe
terodonta;Imparidentia;Venerida;Veneroidea;Veneridae;Mercenaria;Mercenari
a mercenaria
UdeM00000020007 6596 212.2 UdeM00000020007 XP_045159519.1 31 394
261 5 4 389 16 406 1.20E-51 212.2 6596 cellular
organisms;Eukaryota;Opisthokonta;Metazoa;Eumetazoa;Bilateria;Protostomia;
Spiralia;Lophotrochozoa;Mollusca;Bivalvia;Autobranchia;Heteroconchia;Euhe
terodonta;Imparidentia;Venerida;Veneroidea;Veneridae;Mercenaria;Mercenari
a mercenaria
UdeM00000020008 6596 637.1 UdeM00000020008 XP_045161661.1 75.4 394
96 1 1 394 1 393 1.50E-179 637.1 6596 cellular
organisms;Eukaryota;Opisthokonta;Metazoa;Eumetazoa;Bilateria;Protostomia;
Spiralia;Lophotrochozoa;Mollusca;Bivalvia;Autobranchia;Heteroconchia;Euhe
terodonta;Imparidentia;Venerida;Veneroidea;Veneridae;Mercenaria;Mercenari
a mercenaria
UdeM00000020009 6573 332.8 UdeM00000020009 XP_021374258.1 46.6 393
195 4 30 421 12 390 6.30E-88 332.8 6573 cellular
organisms;Eukaryota;Opisthokonta;Metazoa;Eumetazoa;Bilateria;Protostomia;
Spiralia;Lophotrochozoa;Mollusca;Bivalvia;Autobranchia;Pteriomorphia;Pect
inida;Pectinoidea;Pectinidae;Mizuhopecten;Mizuhopecten yessoensis
UdeM00000020010 6596 97.8 UdeM00000020010 XP_045163443.1 46.7 210
90 6 122 326 2562 2754 2.70E-17 97.8 6596 cellular
organisms;Eukaryota;Opisthokonta;Metazoa;Eumetazoa;Bilateria;Protostomia;
Spiralia;Lophotrochozoa;Mollusca;Bivalvia;Autobranchia;Heteroconchia;Euhe
terodonta;Imparidentia;Venerida;Veneroidea;Veneridae;Mercenaria;Mercenari
a mercenaria
UdeM00000020011 6596 89.4 UdeM00000020011 XP_045163443.1 25.3 451
276 8 1425 1826 1904 2342 5.50E-14 89.4 6596 cellular
organisms;Eukaryota;Opisthokonta;Metazoa;Eumetazoa;Bilateria;Protostomia;
Spiralia;Lophotrochozoa;Mollusca;Bivalvia;Autobranchia;Heteroconchia;Euhe
terodonta;Imparidentia;Venerida;Veneroidea;Veneridae;Mercenaria;Mercenari
a mercenaria
UdeM00000020012 6579 742.7 UdeM00000020012 XP_033763361.1 47.3 1132
405 18 19 1133 12 968 7.10E-211 742.7 6579 cellular
organisms;Eukaryota;Opisthokonta;Metazoa;Eumetazoa;Bilateria;Protostomia;
Spiralia;Lophotrochozoa;Mollusca;Bivalvia;Autobranchia;Pteriomorphia;Pect
inida;Pectinoidea;Pectinidae;Pecten;Pecten maximus
UdeM00000020013 13397 472.2 UdeM00000020013 XP_041068243.1 58.6 367
150 1 2 366 222 588 5.90E-130 472.2 13397 cellular
organisms;Eukaryota;Opisthokonta;Metazoa;Eumetazoa;Bilateria;Deuterostomi
a;Chordata;Craniata;Vertebrata;Gnathostomata;Chondrichthyes;Elasmobranchi
i;Selachii;Galeomorphii;Galeoidea;Lamniformes;Alopiidae;Lamninae;Carcharo
don;Carcharodon carcharias
UdeM00000020015 13397 583.6 UdeM00000020015 XP_041068243.1 59.4 429
174 0 2 430 166 594 2.10E-163 583.6 13397 cellular
organisms;Eukaryota;Opisthokonta;Metazoa;Eumetazoa;Bilateria;Deuterostomi
a;Chordata;Craniata;Vertebrata;Gnathostomata;Chondrichthyes;Elasmobranchi
i;Selachii;Galeomorphii;Galeoidea;Lamniformes;Alopiidae;Lamninae;Carcharo
don;Carcharodon carcharias
UdeM00000020016 7830 596.3 UdeM00000020016 XP_038673199.1 59.6 450
180 1 1 448 186 635 3.30E-167 596.3 7830 cellular

organisms;Eukaryota;Opisthokonta;Metazoa;Eumetazoa;Bilateria;Deuterostomia;Chordata;Craniata;Vertebrata;Gnathostomata;Chondrichthyes;Elasmobranchii;Selachii;Galeomorphii;Galeoidea;Carcharhiniformes;Scyliorhinidae;Scyliorhinus;Scyliorhinus canicula
UdeM00000020017 259920 278.1 UdeM00000020017 XP_020376696.1 61.4 207 76 2 2 208 185 387 9.30E-72 278.1 259920 cellular organisms;Eukaryota;Opisthokonta;Metazoa;Eumetazoa;Bilateria;Deuterostomia;Chordata;Craniata;Vertebrata;Gnathostomata;Chondrichthyes;Elasmobranchii;Selachii;Galeomorphii;Galeoidea;Orectolobiformes;Rhincodontidae;Rhincodon;Rhincodon typus
UdeM00000020018 6565 305.8 UdeM00000020018 XP_022297447.1 53.2 284 117 2 8 275 81 364 5.50E-80 305.8 6565 cellular organisms;Eukaryota;Opisthokonta;Metazoa;Eumetazoa;Bilateria;Protostomia;Spiralia;Lophotrochozoa;Mollusca;Bivalvia;Autobranchia;Pteriomorphia;Ostreida;Ostreoidea;Ostreidae;Crassostrea;Crassostrea virginica
UdeM00000020019 6565 253.8 UdeM00000020019 XP_022297447.1 51.2 248 105 2 1 232 117 364 2.10E-64 253.8 6565 cellular organisms;Eukaryota;Opisthokonta;Metazoa;Eumetazoa;Bilateria;Protostomia;Spiralia;Lophotrochozoa;Mollusca;Bivalvia;Autobranchia;Pteriomorphia;Ostreida;Ostreoidea;Ostreidae;Crassostrea;Crassostrea virginica
UdeM00000020020 13397 366.3 UdeM00000020020 XP_041068243.1 61.9 260 99 0 1 260 111 370 3.20E-98 366.3 13397 cellular organisms;Eukaryota;Opisthokonta;Metazoa;Eumetazoa;Bilateria;Deuterostomia;Chordata;Craniata;Vertebrata;Gnathostomata;Chondrichthyes;Elasmobranchii;Selachii;Galeomorphii;Galeoidea;Lamniformes;Alopiidae;Lamninae;Carcharodon;Carcharodon carcharias
UdeM00000020021 105023 335.5 UdeM00000020021 XP_015796544.1 59.1 235 96 0 1 235 254 488 5.80E-89 335.5 105023 cellular organisms;Eukaryota;Opisthokonta;Metazoa;Eumetazoa;Bilateria;Deuterostomia;Chordata;Craniata;Vertebrata;Gnathostomata;Teleostomi;Euteleostomi;Actinopterygii;Actinopteri;Neopterygii;Teleostei;Osteoglossocephalai;Clupeocephala;Euteleosteomorpha;Neoteleostei;Eurypterygia;Ctenosquamata;Acanthomorphata;Euacanthomorphacea;Percomorphaceae;Ovalentaria;Atherinomorphae;Cyprinodontiformes;Aplocheiloidei;Nothobranchiidae;Nothobranchius;Nothobranchius furzeri
UdeM00000020022 7830 798.5 UdeM00000020022 XP_038673199.1 57.8 618 253 1 1 610 130 747 5.90E-228 798.5 7830 cellular organisms;Eukaryota;Opisthokonta;Metazoa;Eumetazoa;Bilateria;Deuterostomia;Chordata;Craniata;Vertebrata;Gnathostomata;Chondrichthyes;Elasmobranchii;Selachii;Galeomorphii;Galeoidea;Carcharhiniformes;Scyliorhinidae;Scyliorhinus;Scyliorhinus canicula
UdeM00000020023 13397 202.2 UdeM00000020023 XP_041068243.1 59.4 155 55 1 2 148 238 392 4.60E-49 202.2 13397 cellular organisms;Eukaryota;Opisthokonta;Metazoa;Eumetazoa;Bilateria;Deuterostomia;Chordata;Craniata;Vertebrata;Gnathostomata;Chondrichthyes;Elasmobranchii;Selachii;Galeomorphii;Galeoidea;Lamniformes;Alopiidae;Lamninae;Carcharodon;Carcharodon carcharias
UdeM00000020024 37621 142.9 UdeM00000020024 XP_014679100.1 37.9 232 130 8 768 994 78 300 2.20E-30 142.9 37621 cellular organisms;Eukaryota;Opisthokonta;Metazoa;Eumetazoa;Bilateria;Protostomia;Ecdysozoa;Scalidophora;Priapulida;Priapulimorpha;Priapulimorphida;Priapulidae;Priapulus;Priapulus caudatus

UdeM00000020025 50390 385.2 UdeM00000020025 XP_028142380.1 57 263
113 0 3 265 30 292 6.80E-104 385.2 50390 cellular
organisms;Eukaryota;Opisthokonta;Metazoa;Eumetazoa;Bilateria;Protostomia;
Ecdysozoa;Panarthropoda;Arthropoda;Mandibulata;Pancrustacea;Hexapoda;Inse
cta;Dicondylia;Pterygota;Neoptera;Endopterygota;Coleoptera;Polyphaga;Cucu
jiformia;Chrysomeloidea;Chrysomelidae;Galerucinae;Luperini;Diabroticina;D
iabroticites;Diabrotica;Diabrotica virgifera;Diabrotica virgifera
virgifera
UdeM00000020026 50390 236.5 UdeM00000020026 XP_028154228.1 55.2 174
76 1 1 174 126 297 2.80E-59 236.5 50390 cellular
organisms;Eukaryota;Opisthokonta;Metazoa;Eumetazoa;Bilateria;Protostomia;
Ecdysozoa;Panarthropoda;Arthropoda;Mandibulata;Pancrustacea;Hexapoda;Inse
cta;Dicondylia;Pterygota;Neoptera;Endopterygota;Coleoptera;Polyphaga;Cucu
jiformia;Chrysomeloidea;Chrysomelidae;Galerucinae;Luperini;Diabroticina;D
iabroticites;Diabrotica;Diabrotica virgifera;Diabrotica virgifera
virgifera
UdeM00000020027 50390 232.3 UdeM00000020027 XP_028154228.1 55.2 174
76 1 1 174 126 297 5.30E-58 232.3 50390 cellular
organisms;Eukaryota;Opisthokonta;Metazoa;Eumetazoa;Bilateria;Protostomia;
Ecdysozoa;Panarthropoda;Arthropoda;Mandibulata;Pancrustacea;Hexapoda;Inse
cta;Dicondylia;Pterygota;Neoptera;Endopterygota;Coleoptera;Polyphaga;Cucu
jiformia;Chrysomeloidea;Chrysomelidae;Galerucinae;Luperini;Diabroticina;D
iabroticites;Diabrotica;Diabrotica virgifera;Diabrotica virgifera
virgifera
UdeM00000020028 51655 116.3 UdeM00000020028 XP_037966588.1 56.5 92
40 0 1 92 673 764 2.10E-23 116.3 51655 cellular
organisms;Eukaryota;Opisthokonta;Metazoa;Eumetazoa;Bilateria;Protostomia;
Ecdysozoa;Panarthropoda;Arthropoda;Mandibulata;Pancrustacea;Hexapoda;Inse
cta;Dicondylia;Pterygota;Neoptera;Endopterygota;Amphiesmenoptera;Lepidopt
era;Glossata;Neolepidoptera;Heteroneura;Ditrysia;Yponomeutoidea;Plutellid
ae;Plutella;Plutella xylostella
UdeM00000020029 6596 1008.4 UdeM00000020029 XP_045161992.1 44.7
1187 605 21 125 1291 56 1211 8.00E-291 1008.4 6596
cellular
organisms;Eukaryota;Opisthokonta;Metazoa;Eumetazoa;Bilateria;Protostomia;
Spiralia;Lophotrochozoa;Mollusca;Bivalvia;Autobranchia;Heteroconchia;Euhe
terodonta;Imparidentia;Venerida;Veneroidea;Veneridae;Mercenaria;Mercenari
a mercenaria
UdeM00000020030 6596 988 UdeM00000020030 XP_045161992.1 43.9 1185
591 19 125 1264 56 1211 1.10E-284 988 6596 cellular
organisms;Eukaryota;Opisthokonta;Metazoa;Eumetazoa;Bilateria;Protostomia;
Spiralia;Lophotrochozoa;Mollusca;Bivalvia;Autobranchia;Heteroconchia;Euhe
terodonta;Imparidentia;Venerida;Veneroidea;Veneridae;Mercenaria;Mercenari
a mercenaria
UdeM00000020031 6596 1027.7 UdeM00000020031 XP_045161992.1 44.9
1208 590 21 29 1189 33 1211 1.20E-296 1027.7 6596
cellular
organisms;Eukaryota;Opisthokonta;Metazoa;Eumetazoa;Bilateria;Protostomia;
Spiralia;Lophotrochozoa;Mollusca;Bivalvia;Autobranchia;Heteroconchia;Euhe
terodonta;Imparidentia;Venerida;Veneroidea;Veneridae;Mercenaria;Mercenari
a mercenaria
UdeM00000020032 6565 129.8 UdeM00000020032 XP_022321863.1 33.2 217
135 5 180 389 324 537 7.60E-27 129.8 6565 cellular
organisms;Eukaryota;Opisthokonta;Metazoa;Eumetazoa;Bilateria;Protostomia;

Spiralia;Lophotrochozoa;Mollusca;Bivalvia;Autobranchia;Pteriomorphia;Ostreida;Ostreoidea;Ostreidae;Crassostrea;Crassostrea virginica
UdeM00000020033 6596 1046.6 UdeM00000020033 XP_045161992.1 45.9 1216 597 20 27 1216 31 1211 2.50E-302 1046.6 6596 cellular organisms;Eukaryota;Opisthokonta;Metazoa;Eumetazoa;Bilateria;Protostomia;Spiralia;Lophotrochozoa;Mollusca;Bivalvia;Autobranchia;Heteroconchia;Euheterodonta;Imparidentia;Venerida;Veneroidea;Veneridae;Mercenaria;Mercenaria mercenaria
UdeM00000020034 6596 910.2 UdeM00000020034 XP_045161992.1 42.1 1230 574 26 27 1167 31 1211 2.70E-261 910.2 6596 cellular organisms;Eukaryota;Opisthokonta;Metazoa;Eumetazoa;Bilateria;Protostomia;Spiralia;Lophotrochozoa;Mollusca;Bivalvia;Autobranchia;Heteroconchia;Euheterodonta;Imparidentia;Venerida;Veneroidea;Veneridae;Mercenaria;Mercenaria mercenaria
UdeM00000020035 6596 933.3 UdeM00000020035 XP_045161992.1 42.8 1232 590 27 27 1194 31 1211 3.00E-268 933.3 6596 cellular organisms;Eukaryota;Opisthokonta;Metazoa;Eumetazoa;Bilateria;Protostomia;Spiralia;Lophotrochozoa;Mollusca;Bivalvia;Autobranchia;Heteroconchia;Euheterodonta;Imparidentia;Venerida;Veneroidea;Veneridae;Mercenaria;Mercenaria mercenaria
UdeM00000020036 6596 1047.3 UdeM00000020036 XP_045161992.1 45.9 1219 592 22 27 1216 31 1211 1.50E-302 1047.3 6596 cellular organisms;Eukaryota;Opisthokonta;Metazoa;Eumetazoa;Bilateria;Protostomia;Spiralia;Lophotrochozoa;Mollusca;Bivalvia;Autobranchia;Heteroconchia;Euheterodonta;Imparidentia;Venerida;Veneroidea;Veneridae;Mercenaria;Mercenaria mercenaria
UdeM00000020037 6573 107.5 UdeM00000020037 XP_021365372.1 39 141 78 3 84 219 19 156 2.30E-20 107.5 6573 cellular organisms;Eukaryota;Opisthokonta;Metazoa;Eumetazoa;Bilateria;Protostomia;Spiralia;Lophotrochozoa;Mollusca;Bivalvia;Autobranchia;Pteriomorphia;Pectinida;Pectinoidea;Pectinidae;Mizuhopecten;Mizuhopecten yessoensis
UdeM00000020038 6596 1037.3 UdeM00000020038 XP_045158027.1 38 1655 900 38 83 1666 184 1783 2.10E-299 1037.3 6596 cellular organisms;Eukaryota;Opisthokonta;Metazoa;Eumetazoa;Bilateria;Protostomia;Spiralia;Lophotrochozoa;Mollusca;Bivalvia;Autobranchia;Heteroconchia;Euheterodonta;Imparidentia;Venerida;Veneroidea;Veneridae;Mercenaria;Mercenaria mercenaria
UdeM00000020039 6565 354.4 UdeM00000020039 XP_022344976.1 57.1 336 134 4 22 350 23 355 2.00E-94 354.4 6565 cellular organisms;Eukaryota;Opisthokonta;Metazoa;Eumetazoa;Bilateria;Protostomia;Spiralia;Lophotrochozoa;Mollusca;Bivalvia;Autobranchia;Pteriomorphia;Ostreida;Ostreoidea;Ostreidae;Crassostrea;Crassostrea virginica
UdeM00000020040 6596 145.6 UdeM00000020040 XP_045204064.1 50.4 139 69 0 25 163 26 164 5.70E-32 145.6 6596 cellular organisms;Eukaryota;Opisthokonta;Metazoa;Eumetazoa;Bilateria;Protostomia;Spiralia;Lophotrochozoa;Mollusca;Bivalvia;Autobranchia;Heteroconchia;Euheterodonta;Imparidentia;Venerida;Veneroidea;Veneridae;Mercenaria;Mercenaria mercenaria
UdeM00000020041 6596 586.3 UdeM00000020041 XP_045204064.1 61.8 566 195 3 25 570 26 590 5.50E-164 586.3 6596 cellular organisms;Eukaryota;Opisthokonta;Metazoa;Eumetazoa;Bilateria;Protostomia;

Spiralia;Lophotrochozoa;Mollusca;Bivalvia;Autobranchia;Heteroconchia;Euheterodonta;Imparidentia;Venerida;Veneroidea;Veneridae;Mercenaria;Mercenaria mercenaria
UdeM00000020042 6596 98.2 UdeM00000020042 XP_045160441.1 30.8 182 123 2 11 192 7 185 1.20E-17 98.2 6596 cellular organisms;Eukaryota;Opisthokonta;Metazoa;Eumetazoa;Bilateria;Protostomia;Spiralia;Lophotrochozoa;Mollusca;Bivalvia;Autobranchia;Heteroconchia;Euheterodonta;Imparidentia;Venerida;Veneroidea;Veneridae;Mercenaria;Mercenaria mercenaria
UdeM00000020043 6596 341.3 UdeM00000020043 XP_045160441.1 28.5 861 555 24 32 855 192 1028 3.70E-90 341.3 6596 cellular organisms;Eukaryota;Opisthokonta;Metazoa;Eumetazoa;Bilateria;Protostomia;Spiralia;Lophotrochozoa;Mollusca;Bivalvia;Autobranchia;Heteroconchia;Euheterodonta;Imparidentia;Venerida;Veneroidea;Veneridae;Mercenaria;Mercenaria mercenaria
UdeM00000020044 6596 354 UdeM00000020044 XP_045160441.1 26.7 1054 676 28 11 999 7 1028 6.40E-94 354 6596 cellular organisms;Eukaryota;Opisthokonta;Metazoa;Eumetazoa;Bilateria;Protostomia;Spiralia;Lophotrochozoa;Mollusca;Bivalvia;Autobranchia;Heteroconchia;Euheterodonta;Imparidentia;Venerida;Veneroidea;Veneridae;Mercenaria;Mercenaria mercenaria
UdeM00000020045 6596 444.9 UdeM00000020045 XP_045173249.1 34.1 787 450 15 24 753 32 806 2.00E-121 444.9 6596 cellular organisms;Eukaryota;Opisthokonta;Metazoa;Eumetazoa;Bilateria;Protostomia;Spiralia;Lophotrochozoa;Mollusca;Bivalvia;Autobranchia;Heteroconchia;Euheterodonta;Imparidentia;Venerida;Veneroidea;Veneridae;Mercenaria;Mercenaria mercenaria
UdeM00000020046 6596 451.1 UdeM00000020046 XP_045173249.1 34.3 788 448 16 13 743 32 806 2.80E-123 451.1 6596 cellular organisms;Eukaryota;Opisthokonta;Metazoa;Eumetazoa;Bilateria;Protostomia;Spiralia;Lophotrochozoa;Mollusca;Bivalvia;Autobranchia;Heteroconchia;Euheterodonta;Imparidentia;Venerida;Veneroidea;Veneridae;Mercenaria;Mercenaria mercenaria
UdeM00000020048 6596 202.2 UdeM00000020048 XP_045173249.1 31.2 385 202 7 653 977 16 397 3.00E-48 202.2 6596 cellular organisms;Eukaryota;Opisthokonta;Metazoa;Eumetazoa;Bilateria;Protostomia;Spiralia;Lophotrochozoa;Mollusca;Bivalvia;Autobranchia;Heteroconchia;Euheterodonta;Imparidentia;Venerida;Veneroidea;Veneridae;Mercenaria;Mercenaria mercenaria
UdeM00000020047 6596 202.2 UdeM00000020047 XP_045173249.1 31.2 385 202 7 651 975 16 397 3.00E-48 202.2 6596 cellular organisms;Eukaryota;Opisthokonta;Metazoa;Eumetazoa;Bilateria;Protostomia;Spiralia;Lophotrochozoa;Mollusca;Bivalvia;Autobranchia;Heteroconchia;Euheterodonta;Imparidentia;Venerida;Veneroidea;Veneridae;Mercenaria;Mercenaria mercenaria
UdeM00000020049 6596 436 UdeM00000020049 XP_045173249.1 33.2 788 456 14 23 753 32 806 9.50E-119 436 6596 cellular organisms;Eukaryota;Opisthokonta;Metazoa;Eumetazoa;Bilateria;Protostomia;Spiralia;Lophotrochozoa;Mollusca;Bivalvia;Autobranchia;Heteroconchia;Euheterodonta;Imparidentia;Venerida;Veneroidea;Veneridae;Mercenaria;Mercenaria mercenaria
UdeM00000020050 6596 217.6 UdeM00000020050 XP_045173249.1 32.7 388 218 8 624 973 16 398 6.90E-53 217.6 6596 cellular organisms;Eukaryota;Opisthokonta;Metazoa;Eumetazoa;Bilateria;Protostomia;

Spiralia;Lophotrochozoa;Mollusca;Bivalvia;Autobranchia;Heteroconchia;Euheterodonta;Imparidentia;Venerida;Veneroidea;Veneridae;Mercenaria;Mercenaria mercenaria
UdeM00000020051 6596 633.3 UdeM00000020051 XP_045162684.1 69.6 437 126 2 8 438 32 467 2.50E-178 633.3 6596 cellular organisms;Eukaryota;Opisthokonta;Metazoa;Eumetazoa;Bilateria;Protostomia;Spiralia;Lophotrochozoa;Mollusca;Bivalvia;Autobranchia;Heteroconchia;Euheterodonta;Imparidentia;Venerida;Veneroidea;Veneridae;Mercenaria;Mercenaria mercenaria
UdeM00000020052 6579 689.1 UdeM00000020052 XP_033764361.1 75.7 445 108 0 1 445 1 445 3.70E-195 689.1 6579 cellular organisms;Eukaryota;Opisthokonta;Metazoa;Eumetazoa;Bilateria;Protostomia;Spiralia;Lophotrochozoa;Mollusca;Bivalvia;Autobranchia;Pteriomorphia;Pectinida;Pectinoidea;Pectinidae;Pecten;Pecten maximus
UdeM00000020054 6596 568.5 UdeM00000020054 XP_045163865.1 66.8 458 131 6 1 439 1 456 7.20E-159 568.5 6596 cellular organisms;Eukaryota;Opisthokonta;Metazoa;Eumetazoa;Bilateria;Protostomia;Spiralia;Lophotrochozoa;Mollusca;Bivalvia;Autobranchia;Heteroconchia;Euheterodonta;Imparidentia;Venerida;Veneroidea;Veneridae;Mercenaria;Mercenaria mercenaria
UdeM00000020055 6596 347.1 UdeM00000020055 XP_045161198.1 45.7 431 203 7 1 404 1 427 3.10E-92 347.1 6596 cellular organisms;Eukaryota;Opisthokonta;Metazoa;Eumetazoa;Bilateria;Protostomia;Spiralia;Lophotrochozoa;Mollusca;Bivalvia;Autobranchia;Heteroconchia;Euheterodonta;Imparidentia;Venerida;Veneroidea;Veneridae;Mercenaria;Mercenaria mercenaria
UdeM00000020056 6596 370.2 UdeM00000020056 XP_045161198.1 45.6 432 220 7 1 422 1 427 3.60E-99 370.2 6596 cellular organisms;Eukaryota;Opisthokonta;Metazoa;Eumetazoa;Bilateria;Protostomia;Spiralia;Lophotrochozoa;Mollusca;Bivalvia;Autobranchia;Heteroconchia;Euheterodonta;Imparidentia;Venerida;Veneroidea;Veneridae;Mercenaria;Mercenaria mercenaria
UdeM00000020057 6573 260.4 UdeM00000020057 XP_021366537.1 33.6 645 364 22 13 625 12 624 6.00E-66 260.4 6573 cellular organisms;Eukaryota;Opisthokonta;Metazoa;Eumetazoa;Bilateria;Protostomia;Spiralia;Lophotrochozoa;Mollusca;Bivalvia;Autobranchia;Pteriomorphia;Pectinida;Pectinoidea;Pectinidae;Mizuhopecten;Mizuhopecten yessoensis
UdeM00000020058 8175 126.7 UdeM00000020058 XP_030274546.1 26.7 442 267 11 589 990 27 451 1.60E-25 126.7 8175 cellular organisms;Eukaryota;Opisthokonta;Metazoa;Eumetazoa;Bilateria;Deuterostomia;Chordata;Craniata;Vertebrata;Gnathostomata;Teleostomi;Euteleostomi;Actinopterygii;Actinopteri;Neopterygii;Teleostei;Osteoglossocephalai;Clupeocephala;Euteleosteomorpha;Neoteleostei;Eurypterygia;Ctenosquamata;Acanthomorphata;Euacanthomorphacea;Percomorphaceae;Eupercaria;Spariformes;Sparidae;Sparus;Sparus aurata
UdeM00000020059 6596 700.7 UdeM00000020059 XP_045157300.1 36.1 1001 606 15 2 989 317 1296 2.70E-198 700.7 6596 cellular organisms;Eukaryota;Opisthokonta;Metazoa;Eumetazoa;Bilateria;Protostomia;Spiralia;Lophotrochozoa;Mollusca;Bivalvia;Autobranchia;Heteroconchia;Euheterodonta;Imparidentia;Venerida;Veneroidea;Veneridae;Mercenaria;Mercenaria mercenaria
UdeM00000020061 6596 172.9 UdeM00000020061 XP_045157647.1 28.2 308 206 4 462 761 2210 2510 1.50E-39 172.9 6596 cellular organisms;Eukaryota;Opisthokonta;Metazoa;Eumetazoa;Bilateria;Protostomia;

Spiralia;Lophotrochozoa;Mollusca;Bivalvia;Autobranchia;Heteroconchia;Euheterodonta;Imparidentia;Venerida;Veneroidea;Veneridae;Mercenaria;Mercenaria mercenaria
UdeM00000020060 6596 172.9 UdeM00000020060 XP_045157647.1 28.2 308 206 4 624 923 2210 2510 1.80E-39 172.9 6596 cellular organisms;Eukaryota;Opisthokonta;Metazoa;Eumetazoa;Bilateria;Protostomia;Spiralia;Lophotrochozoa;Mollusca;Bivalvia;Autobranchia;Heteroconchia;Euheterodonta;Imparidentia;Venerida;Veneroidea;Veneridae;Mercenaria;Mercenaria mercenaria
UdeM00000020064 1203425 94.7 UdeM00000020064 XP_034551711.1 29.6 318 179 10 577 863 139 442 6.00E-16 94.7 1203425 cellular organisms;Eukaryota;Opisthokonta;Metazoa;Eumetazoa;Bilateria;Deuterostomia;Chordata;Craniata;Vertebrata;Gnathostomata;Teleostomi;Euteleostomi;Actinopterygii;Actinopteri;Neopterygii;Teleostei;Osteoglossocephalai;Clupeocephala;Euteleosteomorpha;Neoteleostei;Eurypterygia;Ctenosquamata;Acanthomorphata;Euacanthomorphacea;Percomorphaceae;Eupercaria;Labriformes;Labridae;Notolabrus;Notolabrus celidotus
UdeM00000020065 6596 478 UdeM00000020065 XP_045157300.1 33.5 777 466 7 2 734 317 1086 2.50E-131 478 6596 cellular organisms;Eukaryota;Opisthokonta;Metazoa;Eumetazoa;Bilateria;Protostomia;Spiralia;Lophotrochozoa;Mollusca;Bivalvia;Autobranchia;Heteroconchia;Euheterodonta;Imparidentia;Venerida;Veneroidea;Veneridae;Mercenaria;Mercenaria mercenaria
UdeM00000020066 6596 199.1 UdeM00000020066 XP_045157647.1 23.7 751 460 30 149 863 1837 2510 2.30E-47 199.1 6596 cellular organisms;Eukaryota;Opisthokonta;Metazoa;Eumetazoa;Bilateria;Protostomia;Spiralia;Lophotrochozoa;Mollusca;Bivalvia;Autobranchia;Heteroconchia;Euheterodonta;Imparidentia;Venerida;Veneroidea;Veneridae;Mercenaria;Mercenaria mercenaria
UdeM00000020067 215358 86.3 UdeM00000020067 XP_027138606.1 33.3 174 56 1 1 114 596 769 2.90E-14 86.3 215358 cellular organisms;Eukaryota;Opisthokonta;Metazoa;Eumetazoa;Bilateria;Deuterostomia;Chordata;Craniata;Vertebrata;Gnathostomata;Teleostomi;Euteleostomi;Actinopterygii;Actinopteri;Neopterygii;Teleostei;Osteoglossocephalai;Clupeocephala;Euteleosteomorpha;Neoteleostei;Eurypterygia;Ctenosquamata;Acanthomorphata;Euacanthomorphacea;Percomorphaceae;Eupercaria;Eupercaria incertae sedis;Sciaenidae;Larimichthys;Larimichthys crocea
UdeM00000020070 6596 146.4 UdeM00000020070 XP_045162258.1 37.4 206 101 2 5 182 13 218 3.90E-32 146.4 6596 cellular organisms;Eukaryota;Opisthokonta;Metazoa;Eumetazoa;Bilateria;Protostomia;Spiralia;Lophotrochozoa;Mollusca;Bivalvia;Autobranchia;Heteroconchia;Euheterodonta;Imparidentia;Venerida;Veneroidea;Veneridae;Mercenaria;Mercenaria mercenaria
UdeM00000020072 37621 191.8 UdeM00000020072 XP_014678940.1 65.4 133 45 1 1 132 510 642 5.60E-46 191.8 37621 cellular organisms;Eukaryota;Opisthokonta;Metazoa;Eumetazoa;Bilateria;Protostomia;Ecdysozoa;Scalidophora;Priapulida;Priapulimorpha;Priapulimorphida;Priapulidae;Priapulus;Priapulus caudatus
UdeM00000020073 29159 84.3 UdeM00000020073 XP_034339168.1 71.2 59 17 0 1 59 879 937 6.50E-14 84.3 29159 cellular organisms;Eukaryota;Opisthokonta;Metazoa;Eumetazoa;Bilateria;Protostomia;

Spiralia;Lophotrochozoa;Mollusca;Bivalvia;Autobranchia;Pteriomorphia;Ostreida;Ostreoidea;Ostreidae;Crassostrea;Crassostrea gigas
UdeM00000020074 29159 104.8 UdeM00000020074 XP_034338151.1 69.8 63 19 0 1 63 285 347 5.60E-20 104.8 29159 cellular organisms;Eukaryota;Opisthokonta;Metazoa;Eumetazoa;Bilateria;Protostomia;Spiralia;Lophotrochozoa;Mollusca;Bivalvia;Autobranchia;Pteriomorphia;Ostreida;Ostreoidea;Ostreidae;Crassostrea;Crassostrea gigas
UdeM00000020075 45351 81.6 UdeM00000020075 XP_032227196.1 35.1 151 90 5 2 146 10 158 1.50E-12 81.6 45351 cellular organisms;Eukaryota;Opisthokonta;Metazoa;Eumetazoa;Cnidaria;Anthozoa;Hexacorallia;Actiniaria;Edwardsiidae;Nematostella;Nematostella vectensis
UdeM00000020076 50429 75.9 UdeM00000020076 XP_022804128.1 30 223 140 8 4 222 12 222 8.60E-11 75.9 50429 cellular organisms;Eukaryota;Opisthokonta;Metazoa;Eumetazoa;Cnidaria;Anthozoa;Hexacorallia;Scleractinia;Astrocoeniina;Pocilloporidae;Stylophora;Stylophora pistillata
UdeM00000020077 6573 719.2 UdeM00000020077 XP_021376771.1 26.5 1590 1084 12 201 1740 58 1612 1.30E-203 719.2 6573 cellular organisms;Eukaryota;Opisthokonta;Metazoa;Eumetazoa;Bilateria;Protostomia;Spiralia;Lophotrochozoa;Mollusca;Bivalvia;Autobranchia;Pteriomorphia;Pectinida;Pectinoidea;Pectinidae;Mizuhopecten;Mizuhopecten yessoensis
UdeM00000020078 6565 169.1 UdeM00000020078 XP_022339292.1 36.9 279 94 5 27 227 982 1256 1.00E-38 169.1 6565 cellular organisms;Eukaryota;Opisthokonta;Metazoa;Eumetazoa;Bilateria;Protostomia;Spiralia;Lophotrochozoa;Mollusca;Bivalvia;Autobranchia;Pteriomorphia;Ostreida;Ostreoidea;Ostreidae;Crassostrea;Crassostrea virginica
UdeM00000020080 6596 342.4 UdeM00000020080 XP_045162770.1 86.6 179 24 0 1 179 34 212 3.40E-91 342.4 6596 cellular organisms;Eukaryota;Opisthokonta;Metazoa;Eumetazoa;Bilateria;Protostomia;Spiralia;Lophotrochozoa;Mollusca;Bivalvia;Autobranchia;Heteroconchia;Euheterodonta;Imparidentia;Venerida;Veneroidea;Veneridae;Mercenaria;Mercenaria mercenaria
UdeM00000020082 29159 113.2 UdeM00000020082 XP_019922882.2 36.3 182 103 2 154 335 240 408 6.30E-22 113.2 29159 cellular organisms;Eukaryota;Opisthokonta;Metazoa;Eumetazoa;Bilateria;Protostomia;Spiralia;Lophotrochozoa;Mollusca;Bivalvia;Autobranchia;Pteriomorphia;Ostreida;Ostreoidea;Ostreidae;Crassostrea;Crassostrea gigas
UdeM00000020084 29159 67.4 UdeM00000020084 XP_034307878.1 28.2 131 92 2 1 131 92 220 2.10E-08 67.4 29159 cellular organisms;Eukaryota;Opisthokonta;Metazoa;Eumetazoa;Bilateria;Protostomia;Spiralia;Lophotrochozoa;Mollusca;Bivalvia;Autobranchia;Pteriomorphia;Ostreida;Ostreoidea;Ostreidae;Crassostrea;Crassostrea gigas
UdeM00000020085 29159 132.5 UdeM00000020085 XP_034312908.1 30.5 338 179 15 9 328 6 305 1.10E-27 132.5 29159 cellular organisms;Eukaryota;Opisthokonta;Metazoa;Eumetazoa;Bilateria;Protostomia;Spiralia;Lophotrochozoa;Mollusca;Bivalvia;Autobranchia;Pteriomorphia;Ostreida;Ostreoidea;Ostreidae;Crassostrea;Crassostrea gigas
UdeM00000020086 29159 102.4 UdeM00000020086 XP_034312908.1 38.1 155 91 3 1 153 472 623 5.20E-19 102.4 29159 cellular organisms;Eukaryota;Opisthokonta;Metazoa;Eumetazoa;Bilateria;Protostomia;Spiralia;Lophotrochozoa;Mollusca;Bivalvia;Autobranchia;Pteriomorphia;Ostreida;Ostreoidea;Ostreidae;Crassostrea;Crassostrea gigas
UdeM00000020087 6573 188.3 UdeM00000020087 XP_021366593.1 40.6 286 140 5 1 260 174 455 1.20E-44 188.3 6573 cellular

organisms;Eukaryota;Opisthokonta;Metazoa;Eumetazoa;Bilateria;Protostomia;Spiralia;Lophotrochozoa;Mollusca;Bivalvia;Autobranchia;Pteriomorphia;Pectinida;Pectinoidea;Pectinidae;Mizuhopecten;Mizuhopecten yessoensis
UdeM00000020088 6596 197.2 UdeM00000020088 XP_045162038.1 52.9 204 95 1 1 203 144 347 2.10E-47 197.2 6596 cellular organisms;Eukaryota;Opisthokonta;Metazoa;Eumetazoa;Bilateria;Protostomia;Spiralia;Lophotrochozoa;Mollusca;Bivalvia;Autobranchia;Heteroconchia;Euheterodonta;Imparidentia;Venerida;Veneroidea;Veneridae;Mercenaria;Mercenaria mercenaria
UdeM00000020089 6596 377.1 UdeM00000020089 XP_045162794.1 41.8 534 246 13 4 497 2 510 3.90E-101 377.1 6596 cellular organisms;Eukaryota;Opisthokonta;Metazoa;Eumetazoa;Bilateria;Protostomia;Spiralia;Lophotrochozoa;Mollusca;Bivalvia;Autobranchia;Heteroconchia;Euheterodonta;Imparidentia;Venerida;Veneroidea;Veneridae;Mercenaria;Mercenaria mercenaria
UdeM00000020090 6573 727.6 UdeM00000020090 XP_021372834.1 44.6 984 454 23 21 960 6 942 2.00E-206 727.6 6573 cellular organisms;Eukaryota;Opisthokonta;Metazoa;Eumetazoa;Bilateria;Protostomia;Spiralia;Lophotrochozoa;Mollusca;Bivalvia;Autobranchia;Pteriomorphia;Pectinida;Pectinoidea;Pectinidae;Mizuhopecten;Mizuhopecten yessoensis
UdeM00000020091 1415580 143.3 UdeM00000020091 XP_030075314.1 43 214 99 4 1 206 427 625 3.50E-31 143.3 1415580 cellular organisms;Eukaryota;Opisthokonta;Metazoa;Eumetazoa;Bilateria;Deuterostomia;Chordata;Craniata;Vertebrata;Gnathostomata;Teleostomi;Euteleostomi;Sarcopterygii;Dipnotetrapodomorpha;Tetrapoda;Amphibia;Gymnophiona;Siphonopidae;Microcaecilia;Microcaecilia unicolor
UdeM00000020092 7757 282.7 UdeM00000020092 XP_032835197.1 77 183 42 0 2 184 149 331 3.40E-73 282.7 7757 cellular organisms;Eukaryota;Opisthokonta;Metazoa;Eumetazoa;Bilateria;Deuterostomia;Chordata;Craniata;Vertebrata;Cyclostomata;Hyperoartia;Petromyzontiformes;Petromyzontidae;Petromyzon;Petromyzon marinus
UdeM00000020093 7757 125.2 UdeM00000020093 XP_032835197.1 86.4 66 9 0 1 66 52 117 3.20E-26 125.2 7757 cellular organisms;Eukaryota;Opisthokonta;Metazoa;Eumetazoa;Bilateria;Deuterostomia;Chordata;Craniata;Vertebrata;Cyclostomata;Hyperoartia;Petromyzontiformes;Petromyzontidae;Petromyzon;Petromyzon marinus
UdeM00000020095 6579 1099.3 UdeM00000020095 XP_033739131.1 54.8 971 402 6 1 942 408 1370 0.00E+00 1099.3 6579 cellular organisms;Eukaryota;Opisthokonta;Metazoa;Eumetazoa;Bilateria;Protostomia;Spiralia;Lophotrochozoa;Mollusca;Bivalvia;Autobranchia;Pteriomorphia;Pectinida;Pectinoidea;Pectinidae;Pecten;Pecten maximus
UdeM00000020098 6565 532.3 UdeM00000020098 XP_022341603.1 72.7 377 89 9 9 376 92 463 4.90E-148 532.3 6565 cellular organisms;Eukaryota;Opisthokonta;Metazoa;Eumetazoa;Bilateria;Protostomia;Spiralia;Lophotrochozoa;Mollusca;Bivalvia;Autobranchia;Pteriomorphia;Ostreida;Ostreoidea;Ostreidae;Crassostrea;Crassostrea virginica
UdeM00000020100 37653 299.7 UdeM00000020100 XP_014769083.1 45.4 403 203 4 486 878 1 396 1.20E-77 299.7 37653 cellular organisms;Eukaryota;Opisthokonta;Metazoa;Eumetazoa;Bilateria;Protostomia;Spiralia;Lophotrochozoa;Mollusca;Cephalopoda;Coleoidea;Octopodiformes;Octopoda;Incirrata;Octopodidae;Octopus;Octopus bimaculoides

UdeM00000020099	37653	299.7	UdeM00000020099	XP_014769083.1	45.4	403
	203	4	497	889	1	396	1.30E-77	299.7	37653	cellular
organisms;Eukaryota;Opisthokonta;Metazoa;Eumetazoa;Bilateria;Protostomia;
Spiralia;Lophotrochozoa;Mollusca;Cephalopoda;Coleoidea;Octopodiformes;Oct
opoda;Incirrata;Octopodidae;Octopus;Octopus bimaculoides
UdeM00000020101	29159	245.7	UdeM00000020101	XP_011446387.1	61.9	226
	63	3	1	226	250	452	5.50E-62	245.7	29159	cellular
organisms;Eukaryota;Opisthokonta;Metazoa;Eumetazoa;Bilateria;Protostomia;
Spiralia;Lophotrochozoa;Mollusca;Bivalvia;Autobranchia;Pteriomorphia;Ostr
eida;Ostreoidea;Ostreidae;Crassostrea;Crassostrea gigas
UdeM00000020102	6596	258.8	UdeM00000020102	XP_045163405.1	56.5	253
	95	4	1	247	1	244	7.10E-66	258.8	6596	cellular
organisms;Eukaryota;Opisthokonta;Metazoa;Eumetazoa;Bilateria;Protostomia;
Spiralia;Lophotrochozoa;Mollusca;Bivalvia;Autobranchia;Heteroconchia;Euhe
terodonta;Imparidentia;Venerida;Veneroidea;Veneridae;Mercenaria;Mercenari
a mercenaria
UdeM00000020103	6596	266.2	UdeM00000020103	XP_045161589.1	53.1	254
	107	5	774	1017	26	277	1.80E-67	266.2	6596	cellular
organisms;Eukaryota;Opisthokonta;Metazoa;Eumetazoa;Bilateria;Protostomia;
Spiralia;Lophotrochozoa;Mollusca;Bivalvia;Autobranchia;Heteroconchia;Euhe
terodonta;Imparidentia;Venerida;Veneroidea;Veneridae;Mercenaria;Mercenari
a mercenaria
UdeM00000020104	6596	306.6	UdeM00000020104	XP_045162842.1	80.7	181
	35	0	44	224	44	224	2.60E-80	306.6	6596	cellular
organisms;Eukaryota;Opisthokonta;Metazoa;Eumetazoa;Bilateria;Protostomia;
Spiralia;Lophotrochozoa;Mollusca;Bivalvia;Autobranchia;Heteroconchia;Euhe
terodonta;Imparidentia;Venerida;Veneroidea;Veneridae;Mercenaria;Mercenari
a mercenaria
UdeM00000020105	6596	242.3	UdeM00000020105	XP_045162021.1	67.6	176
	57	0	1	176	1	176	5.20E-61	242.3	6596	cellular
organisms;Eukaryota;Opisthokonta;Metazoa;Eumetazoa;Bilateria;Protostomia;
Spiralia;Lophotrochozoa;Mollusca;Bivalvia;Autobranchia;Heteroconchia;Euhe
terodonta;Imparidentia;Venerida;Veneroidea;Veneridae;Mercenaria;Mercenari
a mercenaria
UdeM00000020106	6596	1137.5	UdeM00000020106	XP_045162021.1	68.6
	814	248	3	2	815	186	991	0.00E+00	1137.5	6596
	cellular
organisms;Eukaryota;Opisthokonta;Metazoa;Eumetazoa;Bilateria;Protostomia;
Spiralia;Lophotrochozoa;Mollusca;Bivalvia;Autobranchia;Heteroconchia;Euhe
terodonta;Imparidentia;Venerida;Veneroidea;Veneridae;Mercenaria;Mercenari
a mercenaria
UdeM00000020107	45264	429.5	UdeM00000020107	XP_044165535.1	49.8	402
	198	1	1	398	136	537	5.40E-117	429.5	45264	cellular
organisms;Eukaryota;Opisthokonta;Metazoa;Eumetazoa;Cnidaria;Anthozoa;Hexa
corallia;Scleractinia;Astrocoeniina;Acroporidae;Acropora;Acropora
millepora
UdeM00000020108	6573	415.6	UdeM00000020108	XP_021361079.1	70.4	270
	80	0	4	273	6	275	4.80E-113	415.6	6573	cellular
organisms;Eukaryota;Opisthokonta;Metazoa;Eumetazoa;Bilateria;Protostomia;
Spiralia;Lophotrochozoa;Mollusca;Bivalvia;Autobranchia;Pteriomorphia;Pect
inida;Pectinoidea;Pectinidae;Mizuhopecten;Mizuhopecten yessoensis
UdeM00000020109	6500	912.9	UdeM00000020109	XP_035829419.1	63.1	773
	259	8	1	770	1	750	2.70E-262	912.9	6500	cellular
organisms;Eukaryota;Opisthokonta;Metazoa;Eumetazoa;Bilateria;Protostomia;

Spiralia;Lophotrochozoa;Mollusca;Gastropoda;Heterobranchia;Euthyneura;Tec
tipleura;Aplysiida;Aplysioidea;Aplysiidae;Aplysia;Aplysia californica
UdeM00000020110 6500 1129 UdeM00000020110 XP_035829419.1 65.2 891
284 8 1 888 1 868 0.00E+00 1129 6500 cellular
organisms;Eukaryota;Opisthokonta;Metazoa;Eumetazoa;Bilateria;Protostomia;
Spiralia;Lophotrochozoa;Mollusca;Gastropoda;Heterobranchia;Euthyneura;Tec
tipleura;Aplysiida;Aplysioidea;Aplysiidae;Aplysia;Aplysia californica
UdeM00000020111 400727 198 UdeM00000020111 XP_025083756.1 73.5
132 35 0 1 132 1 132 7.90E-48 198 400727
cellular
organisms;Eukaryota;Opisthokonta;Metazoa;Eumetazoa;Bilateria;Protostomia;
Spiralia;Lophotrochozoa;Mollusca;Gastropoda;Caenogastropoda;Architaeniogl
ossa;Ampullarioidea;Ampullariidae;Pomacea;Pomacea canaliculata
UdeM00000020112 6565 105.5 UdeM00000020112 XP_022297447.1 53.6 97
43 2 1 96 150 245 3.80E-20 105.5 6565 cellular
organisms;Eukaryota;Opisthokonta;Metazoa;Eumetazoa;Bilateria;Protostomia;
Spiralia;Lophotrochozoa;Mollusca;Bivalvia;Autobranchia;Pteriomorphia;Ostr
eida;Ostreoidea;Ostreidae;Crassostrea;Crassostrea virginica
UdeM00000020113 400727 1527.3 UdeM00000020113 XP_025082598.1
84.1 863 135 1 1 861 1 863 0.00E+00 1527.3
400727 cellular
organisms;Eukaryota;Opisthokonta;Metazoa;Eumetazoa;Bilateria;Protostomia;
Spiralia;Lophotrochozoa;Mollusca;Gastropoda;Caenogastropoda;Architaeniogl
ossa;Ampullarioidea;Ampullariidae;Pomacea;Pomacea canaliculata
UdeM00000020114 6579 483.4 UdeM00000020114 XP_033731191.1 51.4 457
174 2 1 409 2 458 2.80E-133 483.4 6579 cellular
organisms;Eukaryota;Opisthokonta;Metazoa;Eumetazoa;Bilateria;Protostomia;
Spiralia;Lophotrochozoa;Mollusca;Bivalvia;Autobranchia;Pteriomorphia;Pect
inida;Pectinoidea;Pectinidae;Pecten;Pecten maximus
UdeM00000020115 6579 86.3 UdeM00000020115 XP_033735423.1 33 109
67 2 6 109 18 125 2.80E-14 86.3 6579 cellular
organisms;Eukaryota;Opisthokonta;Metazoa;Eumetazoa;Bilateria;Protostomia;
Spiralia;Lophotrochozoa;Mollusca;Bivalvia;Autobranchia;Pteriomorphia;Pect
inida;Pectinoidea;Pectinidae;Pecten;Pecten maximus
UdeM00000020116 6596 238.4 UdeM00000020116 XP_045161545.1 56.7 201
87 0 3 203 20 220 7.90E-60 238.4 6596 cellular
organisms;Eukaryota;Opisthokonta;Metazoa;Eumetazoa;Bilateria;Protostomia;
Spiralia;Lophotrochozoa;Mollusca;Bivalvia;Autobranchia;Heteroconchia;Euhe
terodonta;Imparidentia;Venerida;Veneroidea;Veneridae;Mercenaria;Mercenari
a mercenaria
UdeM00000042427 6596 97.1 UdeM00000042427 XP_045206069.1 23.6 501
281 16 426 914 236 646 1.30E-16 97.1 6596 cellular
organisms;Eukaryota;Opisthokonta;Metazoa;Eumetazoa;Bilateria;Protostomia;
Spiralia;Lophotrochozoa;Mollusca;Bivalvia;Autobranchia;Heteroconchia;Euhe
terodonta;Imparidentia;Venerida;Veneroidea;Veneridae;Mercenaria;Mercenari
a mercenaria
UdeM00000042428 6565 119 UdeM00000042428 XP_022324765.1 26.7 329
213 8 227 540 936 1251 1.90E-23 119 6565 cellular
organisms;Eukaryota;Opisthokonta;Metazoa;Eumetazoa;Bilateria;Protostomia;
Spiralia;Lophotrochozoa;Mollusca;Bivalvia;Autobranchia;Pteriomorphia;Ostr
eida;Ostreoidea;Ostreidae;Crassostrea;Crassostrea virginica
UdeM00000042429 6565 119 UdeM00000042429 XP_022324765.1 26.7 329
213 8 192 505 936 1251 1.80E-23 119 6565 cellular
organisms;Eukaryota;Opisthokonta;Metazoa;Eumetazoa;Bilateria;Protostomia;

Spiralia;Lophotrochozoa;Mollusca;Bivalvia;Autobranchia;Pteriomorphia;Ostreida;Ostreoidea;Ostreidae;Crassostrea;Crassostrea virginica
UdeM00000042431 6596 240 UdeM00000042431 XP_045206207.1 58.1 198 76 1 185 375 5 202 5.10E-60 240 6596 cellular organisms;Eukaryota;Opisthokonta;Metazoa;Eumetazoa;Bilateria;Protostomia;Spiralia;Lophotrochozoa;Mollusca;Bivalvia;Autobranchia;Heteroconchia;Euheterodonta;Imparidentia;Venerida;Veneroidea;Veneridae;Mercenaria;Mercenaria mercenaria
UdeM00000042432 6596 631.3 UdeM00000042432 XP_045204309.1 52.8 627 272 11 7 623 275 887 1.30E-177 631.3 6596 cellular organisms;Eukaryota;Opisthokonta;Metazoa;Eumetazoa;Bilateria;Protostomia;Spiralia;Lophotrochozoa;Mollusca;Bivalvia;Autobranchia;Heteroconchia;Euheterodonta;Imparidentia;Venerida;Veneroidea;Veneridae;Mercenaria;Mercenaria mercenaria
UdeM00000042433 6565 146 UdeM00000042433 XP_022326041.1 30.4 345 173 9 129 473 94 371 1.30E-31 146 6565 cellular organisms;Eukaryota;Opisthokonta;Metazoa;Eumetazoa;Bilateria;Protostomia;Spiralia;Lophotrochozoa;Mollusca;Bivalvia;Autobranchia;Pteriomorphia;Ostreida;Ostreoidea;Ostreidae;Crassostrea;Crassostrea virginica
UdeM00000042435 10224 134 UdeM00000042435 XP_006813844.1 51.7 116 56 0 44 159 247 362 1.70E-28 134 10224 cellular organisms;Eukaryota;Opisthokonta;Metazoa;Eumetazoa;Bilateria;Deuterostomia;Hemichordata;Enteropneusta;Harrimaniidae;Saccoglossus;Saccoglossus kowalevskii
UdeM00000042436 400727 168.3 UdeM00000042436 XP_025108466.1 41.2 250 135 3 2 251 305 542 1.30E-38 168.3 400727 cellular organisms;Eukaryota;Opisthokonta;Metazoa;Eumetazoa;Bilateria;Protostomia;Spiralia;Lophotrochozoa;Mollusca;Gastropoda;Caenogastropoda;Architaenioglossa;Ampullarioidea;Ampullariidae;Pomacea;Pomacea canaliculata
UdeM00000042437 29159 136.3 UdeM00000042437 XP_034300836.1 29.6 362 209 10 28 375 458 787 7.80E-29 136.3 29159 cellular organisms;Eukaryota;Opisthokonta;Metazoa;Eumetazoa;Bilateria;Protostomia;Spiralia;Lophotrochozoa;Mollusca;Bivalvia;Autobranchia;Pteriomorphia;Ostreida;Ostreoidea;Ostreidae;Crassostrea;Crassostrea gigas
UdeM00000042438 29159 62 UdeM00000042438 XP_034311339.1 27 159 105 5 25 173 179 336 2.10E-06 62 29159 cellular organisms;Eukaryota;Opisthokonta;Metazoa;Eumetazoa;Bilateria;Protostomia;Spiralia;Lophotrochozoa;Mollusca;Bivalvia;Autobranchia;Pteriomorphia;Ostreida;Ostreoidea;Ostreidae;Crassostrea;Crassostrea gigas
UdeM00000042439 6596 130.2 UdeM00000042439 XP_045206721.1 33.8 263 144 10 124 372 31 277 7.40E-27 130.2 6596 cellular organisms;Eukaryota;Opisthokonta;Metazoa;Eumetazoa;Bilateria;Protostomia;Spiralia;Lophotrochozoa;Mollusca;Bivalvia;Autobranchia;Heteroconchia;Euheterodonta;Imparidentia;Venerida;Veneroidea;Veneridae;Mercenaria;Mercenaria mercenaria
UdeM00000042440 6596 77.4 UdeM00000042440 XP_045206721.1 34 147 81 5 1 137 584 724 4.50E-11 77.4 6596 cellular organisms;Eukaryota;Opisthokonta;Metazoa;Eumetazoa;Bilateria;Protostomia;Spiralia;Lophotrochozoa;Mollusca;Bivalvia;Autobranchia;Heteroconchia;Euheterodonta;Imparidentia;Venerida;Veneroidea;Veneridae;Mercenaria;Mercenaria mercenaria
UdeM00000042441 225164 95.9 UdeM00000042441 XP_009051404.1 34.8 198 107 8 1 182 47 238 7.10E-17 95.9 225164

cellular
organisms;Eukaryota;Opisthokonta;Metazoa;Eumetazoa;Bilateria;Protostomia;
Spiralia;Lophotrochozoa;Mollusca;Gastropoda;Patellogastropoda;Lottioidea;
Lottiidae;Lottia;Lottia gigantea
UdeM00000042442 6596 143.7 UdeM00000042442 XP_045206721.1 22.2 747
435 22 128 759 54 769 1.10E-30 143.7 6596 cellular
organisms;Eukaryota;Opisthokonta;Metazoa;Eumetazoa;Bilateria;Protostomia;
Spiralia;Lophotrochozoa;Mollusca;Bivalvia;Autobranchia;Heteroconchia;Euhe
terodonta;Imparidentia;Venerida;Veneroidea;Veneridae;Mercenaria;Mercenari
a mercenaria
UdeM00000042444 1606681 70.5 UdeM00000042444 XP_043100876.1 34.7
144 76 7 18 148 162 300 3.40E-09 70.5 1606681
cellular
organisms;Eukaryota;Opisthokonta;Metazoa;Eumetazoa;Bilateria;Deuterostomi
a;Chordata;Craniata;Vertebrata;Gnathostomata;Teleostomi;Euteleostomi;Acti
nopterygii;Actinopteri;Neopterygii;Teleostei;Osteoglossocephalai;Clupeoce
phala;Otomorpha;Ostariophysi;Otophysi;Cypriniphysae;Cypriniformes;Cyprino
idei;Cyprinidae;Smiliogastrinae;Puntigrus;Puntigrus tetrazona
UdeM00000042448 6596 181.8 UdeM00000042448 XP_045182760.1 39 267
159 3 1 265 375 639 4.10E-42 181.8 6596 cellular
organisms;Eukaryota;Opisthokonta;Metazoa;Eumetazoa;Bilateria;Protostomia;
Spiralia;Lophotrochozoa;Mollusca;Bivalvia;Autobranchia;Heteroconchia;Euhe
terodonta;Imparidentia;Venerida;Veneroidea;Veneridae;Mercenaria;Mercenari
a mercenaria
UdeM00000042449 6596 96.3 UdeM00000042449 XP_045209795.1 36.3 179
109 3 56 230 1071 1248 8.80E-17 96.3 6596 cellular
organisms;Eukaryota;Opisthokonta;Metazoa;Eumetazoa;Bilateria;Protostomia;
Spiralia;Lophotrochozoa;Mollusca;Bivalvia;Autobranchia;Heteroconchia;Euhe
terodonta;Imparidentia;Venerida;Veneroidea;Veneridae;Mercenaria;Mercenari
a mercenaria
UdeM00000042450 6565 577.8 UdeM00000042450 XP_022315266.1 47.8 642
307 11 140 763 508 1139 2.10E-161 577.8 6565 cellular
organisms;Eukaryota;Opisthokonta;Metazoa;Eumetazoa;Bilateria;Protostomia;
Spiralia;Lophotrochozoa;Mollusca;Bivalvia;Autobranchia;Pteriomorphia;Ostr
eida;Ostreoidea;Ostreidae;Crassostrea;Crassostrea virginica
UdeM00000042451 37621 104 UdeM00000042451 XP_014675741.1 50.5 103
49 2 5 106 39 140 1.20E-19 104 37621 cellular
organisms;Eukaryota;Opisthokonta;Metazoa;Eumetazoa;Bilateria;Protostomia;
Ecdysozoa;Scalidophora;Priapulida;Priapulimorpha;Priapulimorphida;Priapul
idae;Priapulus;Priapulus caudatus
UdeM00000042452 6565 239.6 UdeM00000042452 XP_022295544.1 43.3 314
125 6 55 323 53 358 5.70E-60 239.6 6565 cellular
organisms;Eukaryota;Opisthokonta;Metazoa;Eumetazoa;Bilateria;Protostomia;
Spiralia;Lophotrochozoa;Mollusca;Bivalvia;Autobranchia;Pteriomorphia;Ostr
eida;Ostreoidea;Ostreidae;Crassostrea;Crassostrea virginica
UdeM00000042453 6596 801.2 UdeM00000042453 XP_045204149.1 75.1 555
135 3 124 676 105 658 1.00E-228 801.2 6596 cellular
organisms;Eukaryota;Opisthokonta;Metazoa;Eumetazoa;Bilateria;Protostomia;
Spiralia;Lophotrochozoa;Mollusca;Bivalvia;Autobranchia;Heteroconchia;Euhe
terodonta;Imparidentia;Venerida;Veneroidea;Veneridae;Mercenaria;Mercenari
a mercenaria
UdeM00000042454 6573 219.9 UdeM00000042454 XP_021339071.1 53.7 203
87 2 48 248 39 236 3.50E-54 219.9 6573 cellular
organisms;Eukaryota;Opisthokonta;Metazoa;Eumetazoa;Bilateria;Protostomia;

Spiralia;Lophotrochozoa;Mollusca;Bivalvia;Autobranchia;Pteriomorphia;Pectinida;Pectinoidea;Pectinidae;Mizuhopecten;Mizuhopecten yessoensis
UdeM00000042455 6596 371.7 UdeM00000042455 XP_045204146.1 81 221 42 0 1 221 1 221 6.50E-100 371.7 6596 cellular organisms;Eukaryota;Opisthokonta;Metazoa;Eumetazoa;Bilateria;Protostomia;Spiralia;Lophotrochozoa;Mollusca;Bivalvia;Autobranchia;Heteroconchia;Euheterodonta;Imparidentia;Venerida;Veneroidea;Veneridae;Mercenaria;Mercenaria mercenaria
UdeM00000042456 29159 285.4 UdeM00000042456 XP_034302273.1 76.2 185 43 1 1 184 1 185 5.10E-74 285.4 29159 cellular organisms;Eukaryota;Opisthokonta;Metazoa;Eumetazoa;Bilateria;Protostomia;Spiralia;Lophotrochozoa;Mollusca;Bivalvia;Autobranchia;Pteriomorphia;Ostreida;Ostreoidea;Ostreidae;Crassostrea;Crassostrea gigas
UdeM00000042457 6573 265.4 UdeM00000042457 XP_021341452.1 61 218 84 1 6 222 13 230 6.60E-68 265.4 6573 cellular organisms;Eukaryota;Opisthokonta;Metazoa;Eumetazoa;Bilateria;Protostomia;Spiralia;Lophotrochozoa;Mollusca;Bivalvia;Autobranchia;Pteriomorphia;Pectinida;Pectinoidea;Pectinidae;Mizuhopecten;Mizuhopecten yessoensis
UdeM00000042458 6596 625.5 UdeM00000042458 XP_045192044.1 47.9 658 327 7 108 752 108 762 9.20E-176 625.5 6596 cellular organisms;Eukaryota;Opisthokonta;Metazoa;Eumetazoa;Bilateria;Protostomia;Spiralia;Lophotrochozoa;Mollusca;Bivalvia;Autobranchia;Heteroconchia;Euheterodonta;Imparidentia;Venerida;Veneroidea;Veneridae;Mercenaria;Mercenaria mercenaria
UdeM00000042459 6596 443 UdeM00000042459 XP_045205754.1 36.9 907 474 22 1155 2054 1792 2607 2.10E-120 443 6596 cellular organisms;Eukaryota;Opisthokonta;Metazoa;Eumetazoa;Bilateria;Protostomia;Spiralia;Lophotrochozoa;Mollusca;Bivalvia;Autobranchia;Heteroconchia;Euheterodonta;Imparidentia;Venerida;Veneroidea;Veneridae;Mercenaria;Mercenaria mercenaria
UdeM00000042460 6500 254.2 UdeM00000042460 XP_005095954.2 71 169 46 1 1 166 1 169 1.10E-64 254.2 6500 cellular organisms;Eukaryota;Opisthokonta;Metazoa;Eumetazoa;Bilateria;Protostomia;Spiralia;Lophotrochozoa;Mollusca;Gastropoda;Heterobranchia;Euthyneura;Tectipleura;Aplysiida;Aplysioidea;Aplysiidae;Aplysia;Aplysia californica
UdeM00000042461 6596 1138.3 UdeM00000042461 XP_045205756.1 60.8 954 353 11 4 945 52 996 0.00E+00 1138.3 6596 cellular organisms;Eukaryota;Opisthokonta;Metazoa;Eumetazoa;Bilateria;Protostomia;Spiralia;Lophotrochozoa;Mollusca;Bivalvia;Autobranchia;Heteroconchia;Euheterodonta;Imparidentia;Venerida;Veneroidea;Veneridae;Mercenaria;Mercenaria mercenaria
UdeM00000042462 6596 915.6 UdeM00000042462 XP_045205756.1 58.8 786 304 10 4 777 52 829 4.30E-263 915.6 6596 cellular organisms;Eukaryota;Opisthokonta;Metazoa;Eumetazoa;Bilateria;Protostomia;Spiralia;Lophotrochozoa;Mollusca;Bivalvia;Autobranchia;Heteroconchia;Euheterodonta;Imparidentia;Venerida;Veneroidea;Veneridae;Mercenaria;Mercenaria mercenaria
UdeM00000042463 386614 110.2 UdeM00000042463 XP_032889954.1 29 272 156 8 7 272 6 246 6.40E-21 110.2 386614 cellular organisms;Eukaryota;Opisthokonta;Metazoa;Eumetazoa;Bilateria;Deuterostomia;Chordata;Craniata;Vertebrata;Gnathostomata;Chondrichthyes;Elasmobranchii;Batoidea;Rajiformes;Rajidae;Amblyraja;Amblyraja radiata

UdeM00000042464 386614 109 UdeM00000042464 XP_032889954.1 29 276 156 10 7 277 6 246 1.40E-20 109 386614 cellular organisms;Eukaryota;Opisthokonta;Metazoa;Eumetazoa;Bilateria;Deuterostomia;Chordata;Craniata;Vertebrata;Gnathostomata;Chondrichthyes;Elasmobranchii;Batoidea;Rajiformes;Rajidae;Amblyraja;Amblyraja radiata
UdeM00000042465 32507 67.4 UdeM00000042465 XP_006809938.1 32.1 112 69 1 59 170 51 155 2.00E-08 67.4 32507 cellular organisms;Eukaryota;Opisthokonta;Metazoa;Eumetazoa;Bilateria;Deuterostomia;Chordata;Craniata;Vertebrata;Gnathostomata;Teleostomi;Euteleostomi;Actinopterygii;Actinopteri;Neopterygii;Teleostei;Osteoglossocephalai;Clupeocephala;Euteleosteomorpha;Neoteleostei;Eurypterygia;Ctenosquamata;Acanthomorphata;Euacanthomorphacea;Percomorphaceae;Ovalentaria;Cichlomorphae;Cichliformes;Cichlidae;African cichlids;Pseudocrenilabrinae;Lamprologini;Neolamprologus;Neolamprologus brichardi
UdeM00000042466 6596 128.3 UdeM00000042466 XP_045186727.1 40.6 175 95 2 176 349 396 562 2.10E-26 128.3 6596 cellular organisms;Eukaryota;Opisthokonta;Metazoa;Eumetazoa;Bilateria;Protostomia;Spiralia;Lophotrochozoa;Mollusca;Bivalvia;Autobranchia;Heteroconchia;Euheterodonta;Imparidentia;Venerida;Veneroidea;Veneridae;Mercenaria;Mercenaria mercenaria
UdeM00000042467 386614 97.1 UdeM00000042467 XP_032889954.1 34.9 169 91 5 27 192 94 246 4.20E-17 97.1 386614 cellular organisms;Eukaryota;Opisthokonta;Metazoa;Eumetazoa;Bilateria;Deuterostomia;Chordata;Craniata;Vertebrata;Gnathostomata;Chondrichthyes;Elasmobranchii;Batoidea;Rajiformes;Rajidae;Amblyraja;Amblyraja radiata
UdeM00000042469 29159 226.5 UdeM00000042469 XP_034334367.1 46.5 254 130 4 6 253 126 379 4.10E-56 226.5 29159 cellular organisms;Eukaryota;Opisthokonta;Metazoa;Eumetazoa;Bilateria;Protostomia;Spiralia;Lophotrochozoa;Mollusca;Bivalvia;Autobranchia;Pteriomorphia;Ostreida;Ostreoidea;Ostreidae;Crassostrea;Crassostrea gigas
UdeM00000042470 386614 72 UdeM00000042470 XP_032889954.1 32.1 140 84 5 68 206 58 187 1.00E-09 72 386614 cellular organisms;Eukaryota;Opisthokonta;Metazoa;Eumetazoa;Bilateria;Deuterostomia;Chordata;Craniata;Vertebrata;Gnathostomata;Chondrichthyes;Elasmobranchii;Batoidea;Rajiformes;Rajidae;Amblyraja;Amblyraja radiata
UdeM00000042471 386614 115.9 UdeM00000042471 XP_032889954.1 29.6 270 156 10 4 267 6 247 1.20E-22 115.9 386614 cellular organisms;Eukaryota;Opisthokonta;Metazoa;Eumetazoa;Bilateria;Deuterostomia;Chordata;Craniata;Vertebrata;Gnathostomata;Chondrichthyes;Elasmobranchii;Batoidea;Rajiformes;Rajidae;Amblyraja;Amblyraja radiata
UdeM00000042473 32507 89.4 UdeM00000042473 XP_006809938.1 34.8 164 92 5 64 225 55 205 8.90E-15 89.4 32507 cellular organisms;Eukaryota;Opisthokonta;Metazoa;Eumetazoa;Bilateria;Deuterostomia;Chordata;Craniata;Vertebrata;Gnathostomata;Teleostomi;Euteleostomi;Actinopterygii;Actinopteri;Neopterygii;Teleostei;Osteoglossocephalai;Clupeocephala;Euteleosteomorpha;Neoteleostei;Eurypterygia;Ctenosquamata;Acanthomorphata;Euacanthomorphacea;Percomorphaceae;Ovalentaria;Cichlomorphae;Cichliformes;Cichlidae;African

cichlids;Pseudocrenilabrinae;Lamprologini;Neolamprologus;Neolamprologus brichardi
UdeM00000015884 6573 3919 UdeM00000015884 XP_021353290.1 39.1 5383 3029 77 1 5252 2963 8229 0.00E+00 3919 6573 cellular organisms;Eukaryota;Opisthokonta;Metazoa;Eumetazoa;Bilateria;Protostomia;Spiralia;Lophotrochozoa;Mollusca;Bivalvia;Autobranchia;Pteriomorphia;Pectinida;Pectinoidea;Pectinidae;Mizuhopecten;Mizuhopecten yessoensis
UdeM00000015885 6573 3892.4 UdeM00000015885 XP_021353290.1 39 5382 3005 77 1 5219 2963 8229 0.00E+00 3892.4 6573 cellular organisms;Eukaryota;Opisthokonta;Metazoa;Eumetazoa;Bilateria;Protostomia;Spiralia;Lophotrochozoa;Mollusca;Bivalvia;Autobranchia;Pteriomorphia;Pectinida;Pectinoidea;Pectinidae;Mizuhopecten;Mizuhopecten yessoensis
UdeM00000015886 6596 1173.3 UdeM00000015886 XP_045156859.1 31.5 2117 1351 39 26 2109 4307 6356 0.00E+00 1173.3 6596 cellular organisms;Eukaryota;Opisthokonta;Metazoa;Eumetazoa;Bilateria;Protostomia;Spiralia;Lophotrochozoa;Mollusca;Bivalvia;Autobranchia;Heteroconchia;Euheterodonta;Imparidentia;Venerida;Veneroidea;Veneridae;Mercenaria;Mercenaria mercenaria
UdeM00000015887 6596 214.9 UdeM00000015887 XP_045171866.1 25.6 793 557 13 9 795 14 779 3.70E-52 214.9 6596 cellular organisms;Eukaryota;Opisthokonta;Metazoa;Eumetazoa;Bilateria;Protostomia;Spiralia;Lophotrochozoa;Mollusca;Bivalvia;Autobranchia;Heteroconchia;Euheterodonta;Imparidentia;Venerida;Veneroidea;Veneridae;Mercenaria;Mercenaria mercenaria
UdeM00000015888 6565 2603.9 UdeM00000015888 XP_022341753.1 35.7 4161 2417 88 1 3997 362 4428 0.00E+00 2603.9 6565 cellular organisms;Eukaryota;Opisthokonta;Metazoa;Eumetazoa;Bilateria;Protostomia;Spiralia;Lophotrochozoa;Mollusca;Bivalvia;Autobranchia;Pteriomorphia;Ostreida;Ostreoidea;Ostreidae;Crassostrea;Crassostrea virginica
UdeM00000015889 6565 2548.5 UdeM00000015889 XP_022341753.1 35.4 4161 2412 88 1 3981 362 4428 0.00E+00 2548.5 6565 cellular organisms;Eukaryota;Opisthokonta;Metazoa;Eumetazoa;Bilateria;Protostomia;Spiralia;Lophotrochozoa;Mollusca;Bivalvia;Autobranchia;Pteriomorphia;Ostreida;Ostreoidea;Ostreidae;Crassostrea;Crassostrea virginica
UdeM00000015890 6573 353.2 UdeM00000015890 XP_021353372.1 52.2 347 157 6 11 356 13 351 3.90E-94 353.2 6573 cellular organisms;Eukaryota;Opisthokonta;Metazoa;Eumetazoa;Bilateria;Protostomia;Spiralia;Lophotrochozoa;Mollusca;Bivalvia;Autobranchia;Pteriomorphia;Pectinida;Pectinoidea;Pectinidae;Mizuhopecten;Mizuhopecten yessoensis
UdeM00000015891 6596 257.3 UdeM00000015891 XP_045189535.1 40.9 386 209 5 11 378 19 403 3.10E-65 257.3 6596 cellular organisms;Eukaryota;Opisthokonta;Metazoa;Eumetazoa;Bilateria;Protostomia;Spiralia;Lophotrochozoa;Mollusca;Bivalvia;Autobranchia;Heteroconchia;Euheterodonta;Imparidentia;Venerida;Veneroidea;Veneridae;Mercenaria;Mercenaria mercenaria
UdeM00000015892 6596 715.7 UdeM00000015892 XP_045163192.1 58.5 588 229 6 1 584 1 577 6.90E-203 715.7 6596 cellular organisms;Eukaryota;Opisthokonta;Metazoa;Eumetazoa;Bilateria;Protostomia;Spiralia;Lophotrochozoa;Mollusca;Bivalvia;Autobranchia;Heteroconchia;Euhe

terodonta;Imparidentia;Venerida;Veneroidea;Veneridae;Mercenaria;Mercenaria mercenaria
UdeM00000015893 6596 740.3 UdeM00000015893 XP_045163959.1 71.8 493 138 1 49 540 58 550 1.70E-210 740.3 6596 cellular organisms;Eukaryota;Opisthokonta;Metazoa;Eumetazoa;Bilateria;Protostomia;Spiralia;Lophotrochozoa;Mollusca;Bivalvia;Autobranchia;Heteroconchia;Euheterodonta;Imparidentia;Venerida;Veneroidea;Veneridae;Mercenaria;Mercenaria mercenaria
UdeM00000015894 6596 699.1 UdeM00000015894 XP_045163959.1 68.7 486 151 1 56 540 65 550 4.30E-198 699.1 6596 cellular organisms;Eukaryota;Opisthokonta;Metazoa;Eumetazoa;Bilateria;Protostomia;Spiralia;Lophotrochozoa;Mollusca;Bivalvia;Autobranchia;Heteroconchia;Euheterodonta;Imparidentia;Venerida;Veneroidea;Veneridae;Mercenaria;Mercenaria mercenaria
UdeM00000015895 6596 547.4 UdeM00000015895 XP_045163959.1 67.3 392 127 1 1 391 159 550 1.50E-152 547.4 6596 cellular organisms;Eukaryota;Opisthokonta;Metazoa;Eumetazoa;Bilateria;Protostomia;Spiralia;Lophotrochozoa;Mollusca;Bivalvia;Autobranchia;Heteroconchia;Euheterodonta;Imparidentia;Venerida;Veneroidea;Veneridae;Mercenaria;Mercenaria mercenaria
UdeM00000015896 6596 447.2 UdeM00000015896 XP_045198610.1 46.7 533 223 12 1 514 2278 2768 2.80E-122 447.2 6596 cellular organisms;Eukaryota;Opisthokonta;Metazoa;Eumetazoa;Bilateria;Protostomia;Spiralia;Lophotrochozoa;Mollusca;Bivalvia;Autobranchia;Heteroconchia;Euheterodonta;Imparidentia;Venerida;Veneroidea;Veneridae;Mercenaria;Mercenaria mercenaria
UdeM00000015897 6596 1512.3 UdeM00000015897 XP_045198610.1 44.8 1901 899 34 7 1823 445 2278 0.00E+00 1512.3 6596 cellular organisms;Eukaryota;Opisthokonta;Metazoa;Eumetazoa;Bilateria;Protostomia;Spiralia;Lophotrochozoa;Mollusca;Bivalvia;Autobranchia;Heteroconchia;Euheterodonta;Imparidentia;Venerida;Veneroidea;Veneridae;Mercenaria;Mercenaria mercenaria
UdeM00000015898 6579 699.9 UdeM00000015898 XP_033763693.1 51.9 775 314 23 1 733 138 895 3.80E-198 699.9 6579 cellular organisms;Eukaryota;Opisthokonta;Metazoa;Eumetazoa;Bilateria;Protostomia;Spiralia;Lophotrochozoa;Mollusca;Bivalvia;Autobranchia;Pteriomorphia;Pectinida;Pectinoidea;Pectinidae;Pecten;Pecten maximus
UdeM00000015899 6579 479.9 UdeM00000015899 XP_033763693.1 46.6 648 312 16 66 685 253 894 6.10E-132 479.9 6579 cellular organisms;Eukaryota;Opisthokonta;Metazoa;Eumetazoa;Bilateria;Protostomia;Spiralia;Lophotrochozoa;Mollusca;Bivalvia;Autobranchia;Pteriomorphia;Pectinida;Pectinoidea;Pectinidae;Pecten;Pecten maximus
UdeM00000015900 6238 78.2 UdeM00000015900 XP_002634025.1 23.8 235 140 7 33 233 27 256 2.90E-11 78.2 6238 cellular organisms;Eukaryota;Opisthokonta;Metazoa;Eumetazoa;Bilateria;Protostomia;Ecdysozoa;Nematoda;Chromadorea;Rhabditida;Rhabditina;Rhabditomorpha;Rhabditoidea;Rhabditidae;Peloderinae;Caenorhabditis;Caenorhabditis briggsae
UdeM00000015901 6579 415.2 UdeM00000015901 XP_033763967.1 66.6 323 99 4 1 317 1 320 9.10E-113 415.2 6579 cellular organisms;Eukaryota;Opisthokonta;Metazoa;Eumetazoa;Bilateria;Protostomia;Spiralia;Lophotrochozoa;Mollusca;Bivalvia;Autobranchia;Pteriomorphia;Pectinida;Pectinoidea;Pectinidae;Pecten;Pecten maximus

UdeM00000015902 6573 776.5 UdeM00000015902 XP_021364536.1 82.8 436
75 0 1 436 1 436 2.00E-221 776.5 6573 cellular
organisms;Eukaryota;Opisthokonta;Metazoa;Eumetazoa;Bilateria;Protostomia;
Spiralia;Lophotrochozoa;Mollusca;Bivalvia;Autobranchia;Pteriomorphia;Pect
inida;Pectinoidea;Pectinidae;Mizuhopecten;Mizuhopecten yessoensis
UdeM00000015903 6573 369.4 UdeM00000015903 XP_021372507.1 60.7 285
111 1 7 290 6 290 4.20E-99 369.4 6573 cellular
organisms;Eukaryota;Opisthokonta;Metazoa;Eumetazoa;Bilateria;Protostomia;
Spiralia;Lophotrochozoa;Mollusca;Bivalvia;Autobranchia;Pteriomorphia;Pect
inida;Pectinoidea;Pectinidae;Mizuhopecten;Mizuhopecten yessoensis
UdeM00000015904 6596 189.1 UdeM00000015904 XP_045161175.1 65.4 133
45 1 62 193 12 144 5.30E-45 189.1 6596 cellular
organisms;Eukaryota;Opisthokonta;Metazoa;Eumetazoa;Bilateria;Protostomia;
Spiralia;Lophotrochozoa;Mollusca;Bivalvia;Autobranchia;Heteroconchia;Euhe
terodonta;Imparidentia;Venerida;Veneroidea;Veneridae;Mercenaria;Mercenari
a mercenaria
UdeM00000015905 6596 157.9 UdeM00000015905 XP_045162518.1 71.6 116
32 1 1 116 207 321 7.80E-36 157.9 6596 cellular
organisms;Eukaryota;Opisthokonta;Metazoa;Eumetazoa;Bilateria;Protostomia;
Spiralia;Lophotrochozoa;Mollusca;Bivalvia;Autobranchia;Heteroconchia;Euhe
terodonta;Imparidentia;Venerida;Veneroidea;Veneridae;Mercenaria;Mercenari
a mercenaria
UdeM00000015906 6596 191.8 UdeM00000015906 XP_045161174.1 61.1 149
54 2 46 193 39 184 8.20E-46 191.8 6596 cellular
organisms;Eukaryota;Opisthokonta;Metazoa;Eumetazoa;Bilateria;Protostomia;
Spiralia;Lophotrochozoa;Mollusca;Bivalvia;Autobranchia;Heteroconchia;Euhe
terodonta;Imparidentia;Venerida;Veneroidea;Veneridae;Mercenaria;Mercenari
a mercenaria
UdeM00000015907 6596 169.5 UdeM00000015907 XP_045162518.1 71.8 124
34 1 25 148 199 321 3.30E-39 169.5 6596 cellular
organisms;Eukaryota;Opisthokonta;Metazoa;Eumetazoa;Bilateria;Protostomia;
Spiralia;Lophotrochozoa;Mollusca;Bivalvia;Autobranchia;Heteroconchia;Euhe
terodonta;Imparidentia;Venerida;Veneroidea;Veneridae;Mercenaria;Mercenari
a mercenaria
UdeM00000015908 6596 191.8 UdeM00000015908 XP_045161174.1 61.1 149
54 2 46 193 39 184 8.20E-46 191.8 6596 cellular
organisms;Eukaryota;Opisthokonta;Metazoa;Eumetazoa;Bilateria;Protostomia;
Spiralia;Lophotrochozoa;Mollusca;Bivalvia;Autobranchia;Heteroconchia;Euhe
terodonta;Imparidentia;Venerida;Veneroidea;Veneridae;Mercenaria;Mercenari
a mercenaria
UdeM00000015909 6596 169.5 UdeM00000015909 XP_045162518.1 71.8 124
34 1 25 148 199 321 3.30E-39 169.5 6596 cellular
organisms;Eukaryota;Opisthokonta;Metazoa;Eumetazoa;Bilateria;Protostomia;
Spiralia;Lophotrochozoa;Mollusca;Bivalvia;Autobranchia;Heteroconchia;Euhe
terodonta;Imparidentia;Venerida;Veneroidea;Veneridae;Mercenaria;Mercenari
a mercenaria
UdeM00000015910 6596 191.8 UdeM00000015910 XP_045161174.1 61.1 149
54 2 46 193 39 184 8.20E-46 191.8 6596 cellular
organisms;Eukaryota;Opisthokonta;Metazoa;Eumetazoa;Bilateria;Protostomia;
Spiralia;Lophotrochozoa;Mollusca;Bivalvia;Autobranchia;Heteroconchia;Euhe
terodonta;Imparidentia;Venerida;Veneroidea;Veneridae;Mercenaria;Mercenari
a mercenaria
UdeM00000015911 6596 169.5 UdeM00000015911 XP_045162518.1 71.8 124
34 1 25 148 199 321 3.30E-39 169.5 6596 cellular

organisms;Eukaryota;Opisthokonta;Metazoa;Eumetazoa;Bilateria;Protostomia;Spiralia;Lophotrochozoa;Mollusca;Bivalvia;Autobranchia;Heteroconchia;Euheterodonta;Imparidentia;Venerida;Veneroidea;Veneridae;Mercenaria;Mercenaria mercenaria
UdeM00000015912 6596 191.8 UdeM00000015912 XP_045161174.1 61.1 149 54 2 46 193 39 184 8.20E-46 191.8 6596 cellular organisms;Eukaryota;Opisthokonta;Metazoa;Eumetazoa;Bilateria;Protostomia;Spiralia;Lophotrochozoa;Mollusca;Bivalvia;Autobranchia;Heteroconchia;Euheterodonta;Imparidentia;Venerida;Veneroidea;Veneridae;Mercenaria;Mercenaria mercenaria
UdeM00000015913 6596 169.5 UdeM00000015913 XP_045162518.1 71.8 124 34 1 25 148 199 321 3.30E-39 169.5 6596 cellular organisms;Eukaryota;Opisthokonta;Metazoa;Eumetazoa;Bilateria;Protostomia;Spiralia;Lophotrochozoa;Mollusca;Bivalvia;Autobranchia;Heteroconchia;Euheterodonta;Imparidentia;Venerida;Veneroidea;Veneridae;Mercenaria;Mercenaria mercenaria
UdeM00000015914 6596 191.8 UdeM00000015914 XP_045161174.1 61.1 149 54 2 46 193 39 184 8.20E-46 191.8 6596 cellular organisms;Eukaryota;Opisthokonta;Metazoa;Eumetazoa;Bilateria;Protostomia;Spiralia;Lophotrochozoa;Mollusca;Bivalvia;Autobranchia;Heteroconchia;Euheterodonta;Imparidentia;Venerida;Veneroidea;Veneridae;Mercenaria;Mercenaria mercenaria
UdeM00000015915 6596 169.5 UdeM00000015915 XP_045162518.1 71.8 124 34 1 25 148 199 321 3.30E-39 169.5 6596 cellular organisms;Eukaryota;Opisthokonta;Metazoa;Eumetazoa;Bilateria;Protostomia;Spiralia;Lophotrochozoa;Mollusca;Bivalvia;Autobranchia;Heteroconchia;Euheterodonta;Imparidentia;Venerida;Veneroidea;Veneridae;Mercenaria;Mercenaria mercenaria
UdeM00000015916 6596 191.8 UdeM00000015916 XP_045161174.1 61.1 149 54 2 46 193 39 184 8.20E-46 191.8 6596 cellular organisms;Eukaryota;Opisthokonta;Metazoa;Eumetazoa;Bilateria;Protostomia;Spiralia;Lophotrochozoa;Mollusca;Bivalvia;Autobranchia;Heteroconchia;Euheterodonta;Imparidentia;Venerida;Veneroidea;Veneridae;Mercenaria;Mercenaria mercenaria
UdeM00000015917 6596 169.5 UdeM00000015917 XP_045162518.1 71.8 124 34 1 25 148 199 321 3.30E-39 169.5 6596 cellular organisms;Eukaryota;Opisthokonta;Metazoa;Eumetazoa;Bilateria;Protostomia;Spiralia;Lophotrochozoa;Mollusca;Bivalvia;Autobranchia;Heteroconchia;Euheterodonta;Imparidentia;Venerida;Veneroidea;Veneridae;Mercenaria;Mercenaria mercenaria
UdeM00000015918 6596 191.8 UdeM00000015918 XP_045161174.1 61.1 149 54 2 46 193 39 184 8.20E-46 191.8 6596 cellular organisms;Eukaryota;Opisthokonta;Metazoa;Eumetazoa;Bilateria;Protostomia;Spiralia;Lophotrochozoa;Mollusca;Bivalvia;Autobranchia;Heteroconchia;Euheterodonta;Imparidentia;Venerida;Veneroidea;Veneridae;Mercenaria;Mercenaria mercenaria
UdeM00000015919 6596 169.5 UdeM00000015919 XP_045162518.1 71.8 124 34 1 25 148 199 321 3.30E-39 169.5 6596 cellular organisms;Eukaryota;Opisthokonta;Metazoa;Eumetazoa;Bilateria;Protostomia;Spiralia;Lophotrochozoa;Mollusca;Bivalvia;Autobranchia;Heteroconchia;Euheterodonta;Imparidentia;Venerida;Veneroidea;Veneridae;Mercenaria;Mercenaria mercenaria
UdeM00000015920 6596 191.8 UdeM00000015920 XP_045161174.1 61.1 149 54 2 46 193 39 184 8.20E-46 191.8 6596 cellular

organisms;Eukaryota;Opisthokonta;Metazoa;Eumetazoa;Bilateria;Protostomia;Spiralia;Lophotrochozoa;Mollusca;Bivalvia;Autobranchia;Heteroconchia;Euheterodonta;Imparidentia;Venerida;Veneroidea;Veneridae;Mercenaria;Mercenaria mercenaria
UdeM00000015921 6596 169.5 UdeM00000015921 XP_045162518.1 71.8 124 34 1 25 148 199 321 3.30E-39 169.5 6596 cellular organisms;Eukaryota;Opisthokonta;Metazoa;Eumetazoa;Bilateria;Protostomia;Spiralia;Lophotrochozoa;Mollusca;Bivalvia;Autobranchia;Heteroconchia;Euheterodonta;Imparidentia;Venerida;Veneroidea;Veneridae;Mercenaria;Mercenaria mercenaria
UdeM00000015922 6596 191.8 UdeM00000015922 XP_045161174.1 61.1 149 54 2 46 193 39 184 8.20E-46 191.8 6596 cellular organisms;Eukaryota;Opisthokonta;Metazoa;Eumetazoa;Bilateria;Protostomia;Spiralia;Lophotrochozoa;Mollusca;Bivalvia;Autobranchia;Heteroconchia;Euheterodonta;Imparidentia;Venerida;Veneroidea;Veneridae;Mercenaria;Mercenaria mercenaria
UdeM00000015923 6596 169.5 UdeM00000015923 XP_045162518.1 71.8 124 34 1 25 148 199 321 3.30E-39 169.5 6596 cellular organisms;Eukaryota;Opisthokonta;Metazoa;Eumetazoa;Bilateria;Protostomia;Spiralia;Lophotrochozoa;Mollusca;Bivalvia;Autobranchia;Heteroconchia;Euheterodonta;Imparidentia;Venerida;Veneroidea;Veneridae;Mercenaria;Mercenaria mercenaria
UdeM00000015924 6596 191.8 UdeM00000015924 XP_045161174.1 61.1 149 54 2 46 193 39 184 8.20E-46 191.8 6596 cellular organisms;Eukaryota;Opisthokonta;Metazoa;Eumetazoa;Bilateria;Protostomia;Spiralia;Lophotrochozoa;Mollusca;Bivalvia;Autobranchia;Heteroconchia;Euheterodonta;Imparidentia;Venerida;Veneroidea;Veneridae;Mercenaria;Mercenaria mercenaria
UdeM00000015925 6596 169.5 UdeM00000015925 XP_045162518.1 71.8 124 34 1 25 148 199 321 3.30E-39 169.5 6596 cellular organisms;Eukaryota;Opisthokonta;Metazoa;Eumetazoa;Bilateria;Protostomia;Spiralia;Lophotrochozoa;Mollusca;Bivalvia;Autobranchia;Heteroconchia;Euheterodonta;Imparidentia;Venerida;Veneroidea;Veneridae;Mercenaria;Mercenaria mercenaria
UdeM00000015926 6596 191.8 UdeM00000015926 XP_045161174.1 61.1 149 54 2 46 193 39 184 8.20E-46 191.8 6596 cellular organisms;Eukaryota;Opisthokonta;Metazoa;Eumetazoa;Bilateria;Protostomia;Spiralia;Lophotrochozoa;Mollusca;Bivalvia;Autobranchia;Heteroconchia;Euheterodonta;Imparidentia;Venerida;Veneroidea;Veneridae;Mercenaria;Mercenaria mercenaria
UdeM00000015927 6596 169.5 UdeM00000015927 XP_045162518.1 71.8 124 34 1 25 148 199 321 3.30E-39 169.5 6596 cellular organisms;Eukaryota;Opisthokonta;Metazoa;Eumetazoa;Bilateria;Protostomia;Spiralia;Lophotrochozoa;Mollusca;Bivalvia;Autobranchia;Heteroconchia;Euheterodonta;Imparidentia;Venerida;Veneroidea;Veneridae;Mercenaria;Mercenaria mercenaria
UdeM00000015928 6596 191.8 UdeM00000015928 XP_045161174.1 61.1 149 54 2 46 193 39 184 8.20E-46 191.8 6596 cellular organisms;Eukaryota;Opisthokonta;Metazoa;Eumetazoa;Bilateria;Protostomia;Spiralia;Lophotrochozoa;Mollusca;Bivalvia;Autobranchia;Heteroconchia;Euheterodonta;Imparidentia;Venerida;Veneroidea;Veneridae;Mercenaria;Mercenaria mercenaria
UdeM00000015929 6596 169.5 UdeM00000015929 XP_045162518.1 71.8 124 34 1 25 148 199 321 3.30E-39 169.5 6596 cellular

organisms;Eukaryota;Opisthokonta;Metazoa;Eumetazoa;Bilateria;Protostomia;Spiralia;Lophotrochozoa;Mollusca;Bivalvia;Autobranchia;Heteroconchia;Euheterodonta;Imparidentia;Venerida;Veneroidea;Veneridae;Mercenaria;Mercenaria mercenaria
UdeM00000015930 6596 191.8 UdeM00000015930 XP_045161174.1 61.1 149 54 2 46 193 39 184 8.20E-46 191.8 6596 cellular organisms;Eukaryota;Opisthokonta;Metazoa;Eumetazoa;Bilateria;Protostomia;Spiralia;Lophotrochozoa;Mollusca;Bivalvia;Autobranchia;Heteroconchia;Euheterodonta;Imparidentia;Venerida;Veneroidea;Veneridae;Mercenaria;Mercenaria mercenaria
UdeM00000015931 6596 169.5 UdeM00000015931 XP_045162518.1 71.8 124 34 1 25 148 199 321 3.30E-39 169.5 6596 cellular organisms;Eukaryota;Opisthokonta;Metazoa;Eumetazoa;Bilateria;Protostomia;Spiralia;Lophotrochozoa;Mollusca;Bivalvia;Autobranchia;Heteroconchia;Euheterodonta;Imparidentia;Venerida;Veneroidea;Veneridae;Mercenaria;Mercenaria mercenaria
UdeM00000015932 6596 191.8 UdeM00000015932 XP_045161174.1 61.1 149 54 2 46 193 39 184 8.20E-46 191.8 6596 cellular organisms;Eukaryota;Opisthokonta;Metazoa;Eumetazoa;Bilateria;Protostomia;Spiralia;Lophotrochozoa;Mollusca;Bivalvia;Autobranchia;Heteroconchia;Euheterodonta;Imparidentia;Venerida;Veneroidea;Veneridae;Mercenaria;Mercenaria mercenaria
UdeM00000015933 6596 169.5 UdeM00000015933 XP_045162518.1 71.8 124 34 1 25 148 199 321 3.30E-39 169.5 6596 cellular organisms;Eukaryota;Opisthokonta;Metazoa;Eumetazoa;Bilateria;Protostomia;Spiralia;Lophotrochozoa;Mollusca;Bivalvia;Autobranchia;Heteroconchia;Euheterodonta;Imparidentia;Venerida;Veneroidea;Veneridae;Mercenaria;Mercenaria mercenaria
UdeM00000015934 6596 191.8 UdeM00000015934 XP_045161174.1 61.1 149 54 2 46 193 39 184 8.20E-46 191.8 6596 cellular organisms;Eukaryota;Opisthokonta;Metazoa;Eumetazoa;Bilateria;Protostomia;Spiralia;Lophotrochozoa;Mollusca;Bivalvia;Autobranchia;Heteroconchia;Euheterodonta;Imparidentia;Venerida;Veneroidea;Veneridae;Mercenaria;Mercenaria mercenaria
UdeM00000015935 6596 167.9 UdeM00000015935 XP_045162518.1 71 124 35 1 25 148 199 321 9.50E-39 167.9 6596 cellular organisms;Eukaryota;Opisthokonta;Metazoa;Eumetazoa;Bilateria;Protostomia;Spiralia;Lophotrochozoa;Mollusca;Bivalvia;Autobranchia;Heteroconchia;Euheterodonta;Imparidentia;Venerida;Veneroidea;Veneridae;Mercenaria;Mercenaria mercenaria
UdeM00000015936 6596 191.8 UdeM00000015936 XP_045161174.1 61.1 149 54 2 46 193 39 184 8.20E-46 191.8 6596 cellular organisms;Eukaryota;Opisthokonta;Metazoa;Eumetazoa;Bilateria;Protostomia;Spiralia;Lophotrochozoa;Mollusca;Bivalvia;Autobranchia;Heteroconchia;Euheterodonta;Imparidentia;Venerida;Veneroidea;Veneridae;Mercenaria;Mercenaria mercenaria
UdeM00000015937 6596 169.1 UdeM00000015937 XP_045162518.1 71.8 124 34 1 386 509 199 321 1.50E-38 169.1 6596 cellular organisms;Eukaryota;Opisthokonta;Metazoa;Eumetazoa;Bilateria;Protostomia;Spiralia;Lophotrochozoa;Mollusca;Bivalvia;Autobranchia;Heteroconchia;Euheterodonta;Imparidentia;Venerida;Veneroidea;Veneridae;Mercenaria;Mercenaria mercenaria
UdeM00000015938 6596 191.8 UdeM00000015938 XP_045161174.1 61.1 149 54 2 46 193 39 184 8.20E-46 191.8 6596 cellular

organisms;Eukaryota;Opisthokonta;Metazoa;Eumetazoa;Bilateria;Protostomia;Spiralia;Lophotrochozoa;Mollusca;Bivalvia;Autobranchia;Heteroconchia;Euheterodonta;Imparidentia;Venerida;Veneroidea;Veneridae;Mercenaria;Mercenaria mercenaria
UdeM00000015939 1735272 470.3 UdeM00000015939 XP_041377214.1 75.4 313 77 0 35 347 53 365 2.20E-129 470.3 1735272 cellular organisms;Eukaryota;Opisthokonta;Metazoa;Eumetazoa;Bilateria;Protostomia;Spiralia;Lophotrochozoa;Mollusca;Gastropoda;Neomphalina;Neomphaloidea;Peltospiridae;Gigantopelta;Gigantopelta aegis
UdeM00000015940 1735272 486.5 UdeM00000015940 XP_041377214.1 79.2 308 64 0 44 351 60 367 3.00E-134 486.5 1735272 cellular organisms;Eukaryota;Opisthokonta;Metazoa;Eumetazoa;Bilateria;Protostomia;Spiralia;Lophotrochozoa;Mollusca;Gastropoda;Neomphalina;Neomphaloidea;Peltospiridae;Gigantopelta;Gigantopelta aegis
UdeM00000015941 1735272 487.3 UdeM00000015941 XP_041377214.1 78.3 314 68 0 44 357 60 373 1.80E-134 487.3 1735272 cellular organisms;Eukaryota;Opisthokonta;Metazoa;Eumetazoa;Bilateria;Protostomia;Spiralia;Lophotrochozoa;Mollusca;Gastropoda;Neomphalina;Neomphaloidea;Peltospiridae;Gigantopelta;Gigantopelta aegis
UdeM00000015942 6565 1380.5 UdeM00000015942 XP_022342025.1 67.2 1013 324 6 119 1128 97 1104 0.00E+00 1380.5 6565 cellular organisms;Eukaryota;Opisthokonta;Metazoa;Eumetazoa;Bilateria;Protostomia;Spiralia;Lophotrochozoa;Mollusca;Bivalvia;Autobranchia;Pteriomorphia;Ostreida;Ostreoidea;Ostreidae;Crassostrea;Crassostrea virginica
UdeM00000015943 6596 265.4 UdeM00000015943 XP_045213493.1 47.2 254 124 3 2 246 137 389 7.80E-68 265.4 6596 cellular organisms;Eukaryota;Opisthokonta;Metazoa;Eumetazoa;Bilateria;Protostomia;Spiralia;Lophotrochozoa;Mollusca;Bivalvia;Autobranchia;Heteroconchia;Euheterodonta;Imparidentia;Venerida;Veneroidea;Veneridae;Mercenaria;Mercenaria mercenaria
UdeM00000015944 6596 172.2 UdeM00000015944 XP_045213493.1 46.5 159 78 2 5 157 232 389 5.90E-40 172.2 6596 cellular organisms;Eukaryota;Opisthokonta;Metazoa;Eumetazoa;Bilateria;Protostomia;Spiralia;Lophotrochozoa;Mollusca;Bivalvia;Autobranchia;Heteroconchia;Euheterodonta;Imparidentia;Venerida;Veneroidea;Veneridae;Mercenaria;Mercenaria mercenaria
UdeM00000015945 6596 337.4 UdeM00000015945 XP_045213493.1 47.2 343 168 6 59 393 37 374 2.60E-89 337.4 6596 cellular organisms;Eukaryota;Opisthokonta;Metazoa;Eumetazoa;Bilateria;Protostomia;Spiralia;Lophotrochozoa;Mollusca;Bivalvia;Autobranchia;Heteroconchia;Euheterodonta;Imparidentia;Venerida;Veneroidea;Veneridae;Mercenaria;Mercenaria mercenaria
UdeM00000015946 6596 324.3 UdeM00000015946 XP_045163858.1 45.3 327 173 2 6 326 65 391 1.80E-85 324.3 6596 cellular organisms;Eukaryota;Opisthokonta;Metazoa;Eumetazoa;Bilateria;Protostomia;Spiralia;Lophotrochozoa;Mollusca;Bivalvia;Autobranchia;Heteroconchia;Euheterodonta;Imparidentia;Venerida;Veneroidea;Veneridae;Mercenaria;Mercenaria mercenaria
UdeM00000015947 6500 291.6 UdeM00000015947 XP_005095133.1 56.1 237 102 2 9 244 1 236 9.40E-76 291.6 6500 cellular

organisms;Eukaryota;Opisthokonta;Metazoa;Eumetazoa;Bilateria;Protostomia;Spiralia;Lophotrochozoa;Mollusca;Gastropoda;Heterobranchia;Euthyneura;Tectipleura;Aplysiida;Aplysioidea;Aplysiidae;Aplysia;Aplysia californica
UdeM00000015948 6596 529.3 UdeM00000015948 XP_045163475.1 51.1 507 232 6 14 507 21 524 7.70E-147 529.3 6596 cellular organisms;Eukaryota;Opisthokonta;Metazoa;Eumetazoa;Bilateria;Protostomia;Spiralia;Lophotrochozoa;Mollusca;Bivalvia;Autobranchia;Heteroconchia;Euheterodonta;Imparidentia;Venerida;Veneroidea;Veneridae;Mercenaria;Mercenaria mercenaria
UdeM00000015950 6596 352.8 UdeM00000015950 XP_045162203.1 49.6 361 163 6 1 342 659 1019 5.20E-94 352.8 6596 cellular organisms;Eukaryota;Opisthokonta;Metazoa;Eumetazoa;Bilateria;Protostomia;Spiralia;Lophotrochozoa;Mollusca;Bivalvia;Autobranchia;Heteroconchia;Euheterodonta;Imparidentia;Venerida;Veneroidea;Veneridae;Mercenaria;Mercenaria mercenaria
UdeM00000015951 1735272 416.8 UdeM00000015951 XP_041356266.1 50.4 409 189 4 51 454 209 608 3.60E-113 416.8 1735272 cellular organisms;Eukaryota;Opisthokonta;Metazoa;Eumetazoa;Bilateria;Protostomia;Spiralia;Lophotrochozoa;Mollusca;Gastropoda;Neomphalina;Neomphaloidea;Peltospiridae;Gigantopelta;Gigantopelta aegis
UdeM00000015953 225164 75.5 UdeM00000015953 XP_009054454.1 39.7 121 68 3 2 121 56 172 6.10E-11 75.5 225164 cellular organisms;Eukaryota;Opisthokonta;Metazoa;Eumetazoa;Bilateria;Protostomia;Spiralia;Lophotrochozoa;Mollusca;Gastropoda;Patellogastropoda;Lottioidea;Lottiidae;Lottia;Lottia gigantea
UdeM00000015952 6596 142.5 UdeM00000015952 XP_045185048.1 39.3 178 92 2 7 168 8 185 5.60E-31 142.5 6596 cellular organisms;Eukaryota;Opisthokonta;Metazoa;Eumetazoa;Bilateria;Protostomia;Spiralia;Lophotrochozoa;Mollusca;Bivalvia;Autobranchia;Heteroconchia;Euheterodonta;Imparidentia;Venerida;Veneroidea;Veneridae;Mercenaria;Mercenaria mercenaria
UdeM00000015954 6596 751.1 UdeM00000015954 XP_045162203.1 43.8 938 434 19 11 863 1084 2013 1.50E-213 751.1 6596 cellular organisms;Eukaryota;Opisthokonta;Metazoa;Eumetazoa;Bilateria;Protostomia;Spiralia;Lophotrochozoa;Mollusca;Bivalvia;Autobranchia;Heteroconchia;Euheterodonta;Imparidentia;Venerida;Veneroidea;Veneridae;Mercenaria;Mercenaria mercenaria
UdeM00000015955 6596 746.9 UdeM00000015955 XP_045162203.1 43.9 922 425 18 6 843 1100 2013 2.80E-212 746.9 6596 cellular organisms;Eukaryota;Opisthokonta;Metazoa;Eumetazoa;Bilateria;Protostomia;Spiralia;Lophotrochozoa;Mollusca;Bivalvia;Autobranchia;Heteroconchia;Euheterodonta;Imparidentia;Venerida;Veneroidea;Veneridae;Mercenaria;Mercenaria mercenaria
UdeM00000015956 6596 751.5 UdeM00000015956 XP_045162203.1 43.6 920 428 16 5 840 1099 2011 1.20E-213 751.5 6596 cellular organisms;Eukaryota;Opisthokonta;Metazoa;Eumetazoa;Bilateria;Protostomia;Spiralia;Lophotrochozoa;Mollusca;Bivalvia;Autobranchia;Heteroconchia;Euheterodonta;Imparidentia;Venerida;Veneroidea;Veneridae;Mercenaria;Mercenaria mercenaria
UdeM00000015957 6596 548.5 UdeM00000015957 XP_045183751.1 49.7 527 248 7 3 520 23 541 9.10E-153 548.5 6596 cellular organisms;Eukaryota;Opisthokonta;Metazoa;Eumetazoa;Bilateria;Protostomia;

Spiralia;Lophotrochozoa;Mollusca;Bivalvia;Autobranchia;Heteroconchia;Euheterodonta;Imparidentia;Venerida;Veneroidea;Veneridae;Mercenaria;Mercenaria mercenaria
UdeM00000015960 6596 538.9 UdeM00000015960 XP_045162442.1 64.5 566 178 4 120 679 30 578 9.40E-150 538.9 6596 cellular organisms;Eukaryota;Opisthokonta;Metazoa;Eumetazoa;Bilateria;Protostomia;Spiralia;Lophotrochozoa;Mollusca;Bivalvia;Autobranchia;Heteroconchia;Euheterodonta;Imparidentia;Venerida;Veneroidea;Veneridae;Mercenaria;Mercenaria mercenaria
UdeM00000015961 6596 464.9 UdeM00000015961 XP_045162442.1 67.4 337 107 2 1 335 36 371 1.00E-127 464.9 6596 cellular organisms;Eukaryota;Opisthokonta;Metazoa;Eumetazoa;Bilateria;Protostomia;Spiralia;Lophotrochozoa;Mollusca;Bivalvia;Autobranchia;Heteroconchia;Euheterodonta;Imparidentia;Venerida;Veneroidea;Veneridae;Mercenaria;Mercenaria mercenaria
UdeM00000015962 6596 117.9 UdeM00000015962 XP_045162442.1 52.2 113 50 1 28 136 466 578 1.10E-23 117.9 6596 cellular organisms;Eukaryota;Opisthokonta;Metazoa;Eumetazoa;Bilateria;Protostomia;Spiralia;Lophotrochozoa;Mollusca;Bivalvia;Autobranchia;Heteroconchia;Euheterodonta;Imparidentia;Venerida;Veneroidea;Veneridae;Mercenaria;Mercenaria mercenaria
UdeM00000015963 6596 178.7 UdeM00000015963 XP_045162962.1 45.3 448 213 6 7 424 16 461 1.50E-41 178.7 6596 cellular organisms;Eukaryota;Opisthokonta;Metazoa;Eumetazoa;Bilateria;Protostomia;Spiralia;Lophotrochozoa;Mollusca;Bivalvia;Autobranchia;Heteroconchia;Euheterodonta;Imparidentia;Venerida;Veneroidea;Veneridae;Mercenaria;Mercenaria mercenaria
UdeM00000015964 6596 170.6 UdeM00000015964 XP_045178436.1 37.2 266 158 4 478 738 52 313 7.40E-39 170.6 6596 cellular organisms;Eukaryota;Opisthokonta;Metazoa;Eumetazoa;Bilateria;Protostomia;Spiralia;Lophotrochozoa;Mollusca;Bivalvia;Autobranchia;Heteroconchia;Euheterodonta;Imparidentia;Venerida;Veneroidea;Veneridae;Mercenaria;Mercenaria mercenaria
UdeM00000015965 6596 213.4 UdeM00000015965 XP_045178436.1 36.4 319 191 6 538 852 7 317 1.20E-51 213.4 6596 cellular organisms;Eukaryota;Opisthokonta;Metazoa;Eumetazoa;Bilateria;Protostomia;Spiralia;Lophotrochozoa;Mollusca;Bivalvia;Autobranchia;Heteroconchia;Euheterodonta;Imparidentia;Venerida;Veneroidea;Veneridae;Mercenaria;Mercenaria mercenaria
UdeM00000015966 6573 390.2 UdeM00000015966 XP_021369816.1 76.7 266 60 1 2 265 3 268 2.10E-105 390.2 6573 cellular organisms;Eukaryota;Opisthokonta;Metazoa;Eumetazoa;Bilateria;Protostomia;Spiralia;Lophotrochozoa;Mollusca;Bivalvia;Autobranchia;Pteriomorphia;Pectinida;Pectinoidea;Pectinidae;Mizuhopecten;Mizuhopecten yessoensis
UdeM00000015967 6579 704.1 UdeM00000015967 XP_033763222.1 66.7 510 130 3 1 505 1 475 1.30E-199 704.1 6579 cellular organisms;Eukaryota;Opisthokonta;Metazoa;Eumetazoa;Bilateria;Protostomia;Spiralia;Lophotrochozoa;Mollusca;Bivalvia;Autobranchia;Pteriomorphia;Pectinida;Pectinoidea;Pectinidae;Pecten;Pecten maximus
UdeM00000015968 215358 117.9 UdeM00000015968 XP_027132797.1 48.2 114 59 0 1 114 138 251 9.90E-24 117.9 215358 cellular organisms;Eukaryota;Opisthokonta;Metazoa;Eumetazoa;Bilateria;Deuterostomia;Chordata;Craniata;Vertebrata;Gnathostomata;Teleostomi;Euteleostomi;Acti

nopterygii;Actinopteri;Neopterygii;Teleostei;Osteoglossocephalai;Clupeocephala;Euteleosteomorpha;Neoteleostei;Eurypterygia;Ctenosquamata;Acanthomorphata;Euacanthomorphacea;Percomorphaceae;Eupercaria;Eupercaria incertae sedis;Sciaenidae;Larimichthys;Larimichthys crocea
UdeM00000015969 106582 90.5 UdeM00000015969 XP_012779827.2 53.6 69 32 0 1 69 293 361 9.10E-16 90.5 106582 cellular organisms;Eukaryota;Opisthokonta;Metazoa;Eumetazoa;Bilateria;Deuterostomia;Chordata;Craniata;Vertebrata;Gnathostomata;Teleostomi;Euteleostomi;Actinopterygii;Actinopteri;Neopterygii;Teleostei;Osteoglossocephalai;Clupeocephala;Euteleosteomorpha;Neoteleostei;Eurypterygia;Ctenosquamata;Acanthomorphata;Euacanthomorphacea;Percomorphaceae;Ovalentaria;Cichlomorphae;Cichliformes;Cichlidae;African cichlids;Pseudocrenilabrinae;Haplochromini;Maylandia;Maylandia zebra complex;Maylandia zebra
UdeM00000015970 51655 105.5 UdeM00000015970 XP_037976812.1 29.9 214 102 3 5 170 93 306 6.70E-20 105.5 51655 cellular organisms;Eukaryota;Opisthokonta;Metazoa;Eumetazoa;Bilateria;Protostomia;Ecdysozoa;Panarthropoda;Arthropoda;Mandibulata;Pancrustacea;Hexapoda;Insecta;Dicondylia;Pterygota;Neoptera;Endopterygota;Amphiesmenoptera;Lepidoptera;Glossata;Neolepidoptera;Heteroneura;Ditrysia;Yponomeutoidea;Plutellidae;Plutella;Plutella xylostella
UdeM00000015971 6565 531.9 UdeM00000015971 XP_022306197.1 46.1 698 293 14 9 654 16 682 1.10E-147 531.9 6565 cellular organisms;Eukaryota;Opisthokonta;Metazoa;Eumetazoa;Bilateria;Protostomia;Spiralia;Lophotrochozoa;Mollusca;Bivalvia;Autobranchia;Pteriomorphia;Ostreida;Ostreoidea;Ostreidae;Crassostrea;Crassostrea virginica
UdeM00000015972 6596 384.4 UdeM00000015972 XP_045163264.1 73.3 243 64 1 61 302 82 324 1.30E-103 384.4 6596 cellular organisms;Eukaryota;Opisthokonta;Metazoa;Eumetazoa;Bilateria;Protostomia;Spiralia;Lophotrochozoa;Mollusca;Bivalvia;Autobranchia;Heteroconchia;Euheterodonta;Imparidentia;Venerida;Veneroidea;Veneridae;Mercenaria;Mercenaria mercenaria
UdeM00000015973 6573 172.9 UdeM00000015973 XP_021372846.1 64.9 114 40 0 95 208 138 251 1.10E-39 172.9 6573 cellular organisms;Eukaryota;Opisthokonta;Metazoa;Eumetazoa;Bilateria;Protostomia;Spiralia;Lophotrochozoa;Mollusca;Bivalvia;Autobranchia;Pteriomorphia;Pectinida;Pectinoidea;Pectinidae;Mizuhopecten;Mizuhopecten yessoensis
UdeM00000015974 1735272 176.8 UdeM00000015974 XP_041377869.1 40 280 150 5 16 287 486 755 4.00E-41 176.8 1735272 cellular organisms;Eukaryota;Opisthokonta;Metazoa;Eumetazoa;Bilateria;Protostomia;Spiralia;Lophotrochozoa;Mollusca;Gastropoda;Neomphalina;Neomphaloidea;Peltospiridae;Gigantopelta;Gigantopelta aegis
UdeM00000015975 6596 1559.7 UdeM00000015975 XP_045177443.1 63.1 1293 447 11 3 1281 2 1278 0.00E+00 1559.7 6596 cellular organisms;Eukaryota;Opisthokonta;Metazoa;Eumetazoa;Bilateria;Protostomia;Spiralia;Lophotrochozoa;Mollusca;Bivalvia;Autobranchia;Heteroconchia;Euheterodonta;Imparidentia;Venerida;Veneroidea;Veneridae;Mercenaria;Mercenaria mercenaria
UdeM00000015978 6596 717.6 UdeM00000015978 XP_045171655.1 60.1 584 225 2 52 627 41 624 1.40E-203 717.6 6596 cellular organisms;Eukaryota;Opisthokonta;Metazoa;Eumetazoa;Bilateria;Protostomia;

Spiralia;Lophotrochozoa;Mollusca;Bivalvia;Autobranchia;Heteroconchia;Euheterodonta;Imparidentia;Venerida;Veneroidea;Veneridae;Mercenaria;Mercenaria mercenaria
UdeM00000015979 6596 612.5 UdeM00000015979 XP_045171655.1 60.7 484 186 1 2 481 141 624 4.70E-172 612.5 6596 cellular organisms;Eukaryota;Opisthokonta;Metazoa;Eumetazoa;Bilateria;Protostomia;Spiralia;Lophotrochozoa;Mollusca;Bivalvia;Autobranchia;Heteroconchia;Euheterodonta;Imparidentia;Venerida;Veneroidea;Veneridae;Mercenaria;Mercenaria mercenaria
UdeM00000015980 6500 295 UdeM00000015980 XP_005092455.1 57.5 221 94 0 3 223 8 228 1.00E-76 295 6500 cellular organisms;Eukaryota;Opisthokonta;Metazoa;Eumetazoa;Bilateria;Protostomia;Spiralia;Lophotrochozoa;Mollusca;Gastropoda;Heterobranchia;Euthyneura;Tectipleura;Aplysiida;Aplysioidea;Aplysiidae;Aplysia;Aplysia californica
UdeM00000015981 225164 412.9 UdeM00000015981 XP_009057084.1 45.3 470 248 6 35 499 43 508 5.80E-112 412.9 225164 cellular organisms;Eukaryota;Opisthokonta;Metazoa;Eumetazoa;Bilateria;Protostomia;Spiralia;Lophotrochozoa;Mollusca;Gastropoda;Patellogastropoda;Lottioidea;Lottiidae;Lottia;Lottia gigantea
UdeM00000015982 400727 226.1 UdeM00000015982 XP_025099511.1 48.5 202 102 1 20 221 93 292 4.50E-56 226.1 400727 cellular organisms;Eukaryota;Opisthokonta;Metazoa;Eumetazoa;Bilateria;Protostomia;Spiralia;Lophotrochozoa;Mollusca;Gastropoda;Caenogastropoda;Architaenioglossa;Ampullarioidea;Ampullariidae;Pomacea;Pomacea canaliculata
UdeM00000015983 225164 184.5 UdeM00000015983 XP_009057084.1 43.2 234 127 4 18 246 25 257 1.70E-43 184.5 225164 cellular organisms;Eukaryota;Opisthokonta;Metazoa;Eumetazoa;Bilateria;Protostomia;Spiralia;Lophotrochozoa;Mollusca;Gastropoda;Patellogastropoda;Lottioidea;Lottiidae;Lottia;Lottia gigantea
UdeM00000015984 6500 292.4 UdeM00000015984 XP_005092455.1 61.1 203 79 0 21 223 26 228 6.50E-76 292.4 6500 cellular organisms;Eukaryota;Opisthokonta;Metazoa;Eumetazoa;Bilateria;Protostomia;Spiralia;Lophotrochozoa;Mollusca;Gastropoda;Heterobranchia;Euthyneura;Tectipleura;Aplysiida;Aplysioidea;Aplysiidae;Aplysia;Aplysia californica
UdeM00000015985 6500 291.6 UdeM00000015985 XP_005092455.1 56.6 221 96 0 3 223 8 228 1.10E-75 291.6 6500 cellular organisms;Eukaryota;Opisthokonta;Metazoa;Eumetazoa;Bilateria;Protostomia;Spiralia;Lophotrochozoa;Mollusca;Gastropoda;Heterobranchia;Euthyneura;Tectipleura;Aplysiida;Aplysioidea;Aplysiidae;Aplysia;Aplysia californica
UdeM00000015986 225164 389.4 UdeM00000015986 XP_009065179.1 41.3 460 263 4 24 477 4 462 6.50E-105 389.4 225164 cellular organisms;Eukaryota;Opisthokonta;Metazoa;Eumetazoa;Bilateria;Protostomia;Spiralia;Lophotrochozoa;Mollusca;Gastropoda;Patellogastropoda;Lottioidea;Lottiidae;Lottia;Lottia gigantea
UdeM00000015987 6565 107.1 UdeM00000015987 XP_022292180.1 70.5 78 19 2 6 82 96 170 1.10E-20 107.1 6565 cellular organisms;Eukaryota;Opisthokonta;Metazoa;Eumetazoa;Bilateria;Protostomia;Spiralia;Lophotrochozoa;Mollusca;Bivalvia;Autobranchia;Pteriomorphia;Ostreida;Ostreoidea;Ostreidae;Crassostrea;Crassostrea virginica

UdeM00000015988 400727 226.9 UdeM00000015988 XP_025099511.1 43.4
244 132 3 21 261 52 292 3.10E-56 226.9 400727
cellular
organisms;Eukaryota;Opisthokonta;Metazoa;Eumetazoa;Bilateria;Protostomia;
Spiralia;Lophotrochozoa;Mollusca;Gastropoda;Caenogastropoda;Architaenioglossa;Ampullarioidea;Ampullariidae;Pomacea;Pomacea canaliculata
UdeM00000015989 225164 386.7 UdeM00000015989 XP_009065179.1 41.3
470 265 6 37 501 19 482 4.40E-104 386.7 225164
cellular
organisms;Eukaryota;Opisthokonta;Metazoa;Eumetazoa;Bilateria;Protostomia;
Spiralia;Lophotrochozoa;Mollusca;Gastropoda;Patellogastropoda;Lottioidea;
Lottiidae;Lottia;Lottia gigantea
UdeM00000015990 400727 296.6 UdeM00000015990 XP_025099197.1 45.3
322 173 2 7 327 189 508 3.90E-77 296.6 400727
cellular
organisms;Eukaryota;Opisthokonta;Metazoa;Eumetazoa;Bilateria;Protostomia;
Spiralia;Lophotrochozoa;Mollusca;Gastropoda;Caenogastropoda;Architaenioglossa;Ampullarioidea;Ampullariidae;Pomacea;Pomacea canaliculata
UdeM00000015991 225164 116.3 UdeM00000015991 XP_009057084.1 44
150 75 4 16 157 20 168 3.60E-23 116.3 225164
cellular
organisms;Eukaryota;Opisthokonta;Metazoa;Eumetazoa;Bilateria;Protostomia;
Spiralia;Lophotrochozoa;Mollusca;Gastropoda;Patellogastropoda;Lottioidea;
Lottiidae;Lottia;Lottia gigantea
UdeM00000015993 7574 74.3 UdeM00000015993 XP_013400906.1 41.1 95
55 1 18 112 226 319 1.20E-10 74.3 7574 cellular
organisms;Eukaryota;Opisthokonta;Metazoa;Eumetazoa;Bilateria;Protostomia;
Spiralia;Lophotrochozoa;Brachiopoda;Linguliformea;Lingulata;Lingulida;Linguloidea;Lingulidae;Lingula;Lingula anatina
UdeM00000015998 6596 223.8 UdeM00000015998 XP_045216699.1 66.3 169
55 2 27 193 4 172 8.30E-55 223.8 6596 cellular
organisms;Eukaryota;Opisthokonta;Metazoa;Eumetazoa;Bilateria;Protostomia;
Spiralia;Lophotrochozoa;Mollusca;Bivalvia;Autobranchia;Heteroconchia;Euheterodonta;Imparidentia;Venerida;Veneroidea;Veneridae;Mercenaria;Mercenaria mercenaria
UdeM00000016000 6596 151.4 UdeM00000016000 XP_045163791.1 27.8 540
290 14 1 504 21 496 3.20E-33 151.4 6596 cellular
organisms;Eukaryota;Opisthokonta;Metazoa;Eumetazoa;Bilateria;Protostomia;
Spiralia;Lophotrochozoa;Mollusca;Bivalvia;Autobranchia;Heteroconchia;Euheterodonta;Imparidentia;Venerida;Veneroidea;Veneridae;Mercenaria;Mercenaria mercenaria
UdeM00000016003 6565 778.1 UdeM00000016003 XP_022337369.1 55.6 773
304 12 4 764 693 1438 1.00E-221 778.1 6565 cellular
organisms;Eukaryota;Opisthokonta;Metazoa;Eumetazoa;Bilateria;Protostomia;
Spiralia;Lophotrochozoa;Mollusca;Bivalvia;Autobranchia;Pteriomorphia;Ostreida;Ostreoidea;Ostreidae;Crassostrea;Crassostrea virginica
UdeM00000016004 29159 64.3 UdeM00000016004 XP_034299542.1 32.7 110
66 2 38 144 31 135 2.90E-07 64.3 29159 cellular
organisms;Eukaryota;Opisthokonta;Metazoa;Eumetazoa;Bilateria;Protostomia;
Spiralia;Lophotrochozoa;Mollusca;Bivalvia;Autobranchia;Pteriomorphia;Ostreida;Ostreoidea;Ostreidae;Crassostrea;Crassostrea gigas
UdeM00000016005 29159 65.5 UdeM00000016005 XP_034299542.1 33 109
66 2 38 143 31 135 1.30E-07 65.5 29159 cellular
organisms;Eukaryota;Opisthokonta;Metazoa;Eumetazoa;Bilateria;Protostomia;

Spiralia;Lophotrochozoa;Mollusca;Bivalvia;Autobranchia;Pteriomorphia;Ostreida;Ostreoidea;Ostreidae;Crassostrea;Crassostrea gigas
UdeM00000016006 6573 126.3 UdeM00000016006 XP_021372906.1 47.9 144 70 3 24 166 19 158 3.80E-26 126.3 6573 cellular organisms;Eukaryota;Opisthokonta;Metazoa;Eumetazoa;Bilateria;Protostomia;Spiralia;Lophotrochozoa;Mollusca;Bivalvia;Autobranchia;Pteriomorphia;Pectinida;Pectinoidea;Pectinidae;Mizuhopecten;Mizuhopecten yessoensis
UdeM00000016007 6573 100.5 UdeM00000016007 XP_021372906.1 54.5 99 42 2 6 103 62 158 1.40E-18 100.5 6573 cellular organisms;Eukaryota;Opisthokonta;Metazoa;Eumetazoa;Bilateria;Protostomia;Spiralia;Lophotrochozoa;Mollusca;Bivalvia;Autobranchia;Pteriomorphia;Pectinida;Pectinoidea;Pectinidae;Mizuhopecten;Mizuhopecten yessoensis
UdeM00000016008 1735272 303.1 UdeM00000016008 XP_041377845.1 49 298 149 1 56 353 20 314 4.60E-79 303.1 1735272 cellular organisms;Eukaryota;Opisthokonta;Metazoa;Eumetazoa;Bilateria;Protostomia;Spiralia;Lophotrochozoa;Mollusca;Gastropoda;Neomphalina;Neomphaloidea;Peltospiridae;Gigantopelta;Gigantopelta aegis
UdeM00000016009 1735272 283.1 UdeM00000016009 XP_041377845.1 49.8 267 134 0 56 322 20 286 4.40E-73 283.1 1735272 cellular organisms;Eukaryota;Opisthokonta;Metazoa;Eumetazoa;Bilateria;Protostomia;Spiralia;Lophotrochozoa;Mollusca;Gastropoda;Neomphalina;Neomphaloidea;Peltospiridae;Gigantopelta;Gigantopelta aegis
UdeM00000016010 6596 356.7 UdeM00000016010 XP_045162975.1 71 217 63 0 1 217 1 217 2.20E-95 356.7 6596 cellular organisms;Eukaryota;Opisthokonta;Metazoa;Eumetazoa;Bilateria;Protostomia;Spiralia;Lophotrochozoa;Mollusca;Bivalvia;Autobranchia;Heteroconchia;Euheterodonta;Imparidentia;Venerida;Veneroidea;Veneridae;Mercenaria;Mercenaria mercenaria
UdeM00000016011 6596 848.6 UdeM00000016011 XP_045161432.1 55.6 772 321 6 4 768 10 766 6.30E-243 848.6 6596 cellular organisms;Eukaryota;Opisthokonta;Metazoa;Eumetazoa;Bilateria;Protostomia;Spiralia;Lophotrochozoa;Mollusca;Bivalvia;Autobranchia;Heteroconchia;Euheterodonta;Imparidentia;Venerida;Veneroidea;Veneridae;Mercenaria;Mercenaria mercenaria
UdeM00000016012 6596 682.9 UdeM00000016012 XP_045161433.1 78.9 422 88 1 80 500 96 517 3.00E-193 682.9 6596 cellular organisms;Eukaryota;Opisthokonta;Metazoa;Eumetazoa;Bilateria;Protostomia;Spiralia;Lophotrochozoa;Mollusca;Bivalvia;Autobranchia;Heteroconchia;Euheterodonta;Imparidentia;Venerida;Veneroidea;Veneridae;Mercenaria;Mercenaria mercenaria
UdeM00000016014 6579 158.3 UdeM00000016014 XP_033724604.1 28.8 490 259 13 1 461 1 429 2.40E-35 158.3 6579 cellular organisms;Eukaryota;Opisthokonta;Metazoa;Eumetazoa;Bilateria;Protostomia;Spiralia;Lophotrochozoa;Mollusca;Bivalvia;Autobranchia;Pteriomorphia;Pectinida;Pectinoidea;Pectinidae;Pecten;Pecten maximus
UdeM00000016015 6596 141 UdeM00000016015 XP_045162709.1 31.5 381 219 6 1 342 1 378 2.90E-30 141 6596 cellular organisms;Eukaryota;Opisthokonta;Metazoa;Eumetazoa;Bilateria;Protostomia;Spiralia;Lophotrochozoa;Mollusca;Bivalvia;Autobranchia;Heteroconchia;Euheterodonta;Imparidentia;Venerida;Veneroidea;Veneridae;Mercenaria;Mercenaria mercenaria

UdeM00000016016 6596 300.4 UdeM00000016016 XP_045213379.1 59.8 244
98 0 4 247 1 244 2.10E-78 300.4 6596 cellular
organisms;Eukaryota;Opisthokonta;Metazoa;Eumetazoa;Bilateria;Protostomia;
Spiralia;Lophotrochozoa;Mollusca;Bivalvia;Autobranchia;Heteroconchia;Euheterodonta;Imparidentia;Venerida;Veneroidea;Veneridae;Mercenaria;Mercenaria mercenaria
UdeM00000016018 6596 829.3 UdeM00000016018 XP_045162289.1 70.9 601
171 4 3 601 2 600 3.20E-237 829.3 6596 cellular
organisms;Eukaryota;Opisthokonta;Metazoa;Eumetazoa;Bilateria;Protostomia;
Spiralia;Lophotrochozoa;Mollusca;Bivalvia;Autobranchia;Heteroconchia;Euheterodonta;Imparidentia;Venerida;Veneroidea;Veneridae;Mercenaria;Mercenaria mercenaria
UdeM00000016017 6596 828.9 UdeM00000016017 XP_045162290.1 70.3 606
176 4 3 606 2 605 4.20E-237 828.9 6596 cellular
organisms;Eukaryota;Opisthokonta;Metazoa;Eumetazoa;Bilateria;Protostomia;
Spiralia;Lophotrochozoa;Mollusca;Bivalvia;Autobranchia;Heteroconchia;Euheterodonta;Imparidentia;Venerida;Veneroidea;Veneridae;Mercenaria;Mercenaria mercenaria
UdeM00000016019 6596 804.7 UdeM00000016019 XP_045162289.1 68.7 601
185 3 37 636 2 600 9.00E-230 804.7 6596 cellular
organisms;Eukaryota;Opisthokonta;Metazoa;Eumetazoa;Bilateria;Protostomia;
Spiralia;Lophotrochozoa;Mollusca;Bivalvia;Autobranchia;Heteroconchia;Euheterodonta;Imparidentia;Venerida;Veneroidea;Veneridae;Mercenaria;Mercenaria mercenaria
UdeM00000016020 6596 804.7 UdeM00000016020 XP_045162289.1 68.7 601
185 3 46 645 2 600 9.10E-230 804.7 6596 cellular
organisms;Eukaryota;Opisthokonta;Metazoa;Eumetazoa;Bilateria;Protostomia;
Spiralia;Lophotrochozoa;Mollusca;Bivalvia;Autobranchia;Heteroconchia;Euheterodonta;Imparidentia;Venerida;Veneroidea;Veneridae;Mercenaria;Mercenaria mercenaria
UdeM00000016021 6596 804.7 UdeM00000016021 XP_045162289.1 68.7 601
185 3 3 602 2 600 8.50E-230 804.7 6596 cellular
organisms;Eukaryota;Opisthokonta;Metazoa;Eumetazoa;Bilateria;Protostomia;
Spiralia;Lophotrochozoa;Mollusca;Bivalvia;Autobranchia;Heteroconchia;Euheterodonta;Imparidentia;Venerida;Veneroidea;Veneridae;Mercenaria;Mercenaria mercenaria
UdeM00000016025 6573 221.9 UdeM00000016025 XP_021362715.1 39.7 287
173 0 2 288 41 327 1.10E-54 221.9 6573 cellular
organisms;Eukaryota;Opisthokonta;Metazoa;Eumetazoa;Bilateria;Protostomia;
Spiralia;Lophotrochozoa;Mollusca;Bivalvia;Autobranchia;Pteriomorphia;Pectinida;Pectinoidea;Pectinidae;Mizuhopecten;Mizuhopecten yessoensis
UdeM00000016026 6573 198.7 UdeM00000016026 XP_021362715.1 40.6 254
151 0 1 254 74 327 9.20E-48 198.7 6573 cellular
organisms;Eukaryota;Opisthokonta;Metazoa;Eumetazoa;Bilateria;Protostomia;
Spiralia;Lophotrochozoa;Mollusca;Bivalvia;Autobranchia;Pteriomorphia;Pectinida;Pectinoidea;Pectinidae;Mizuhopecten;Mizuhopecten yessoensis
UdeM00000016027 6579 594.3 UdeM00000016027 XP_033763139.1 54 589
235 7 22 575 29 616 1.60E-166 594.3 6579 cellular
organisms;Eukaryota;Opisthokonta;Metazoa;Eumetazoa;Bilateria;Protostomia;
Spiralia;Lophotrochozoa;Mollusca;Bivalvia;Autobranchia;Pteriomorphia;Pectinida;Pectinoidea;Pectinidae;Pecten;Pecten maximus
UdeM00000016029 6579 103.6 UdeM00000016029 XP_033763846.1 40.5 148
87 1 12 158 11 158 2.40E-19 103.6 6579 cellular
organisms;Eukaryota;Opisthokonta;Metazoa;Eumetazoa;Bilateria;Protostomia;

Spiralia;Lophotrochozoa;Mollusca;Bivalvia;Autobranchia;Pteriomorphia;Pectinida;Pectinoidea;Pectinidae;Pecten;Pecten maximus
UdeM00000016032 1735272 139.4 UdeM00000016032 XP_041378082.1 64.8 91 29 1 1 91 67 154 2.60E-30 139.4 1735272 cellular organisms;Eukaryota;Opisthokonta;Metazoa;Eumetazoa;Bilateria;Protostomia;Spiralia;Lophotrochozoa;Mollusca;Gastropoda;Neomphalina;Neomphaloidea;Peltospiridae;Gigantopelta;Gigantopelta aegis
UdeM00000016033 6596 381.3 UdeM00000016033 XP_045163108.1 49.8 448 202 8 1 433 46 485 1.60E-102 381.3 6596 cellular organisms;Eukaryota;Opisthokonta;Metazoa;Eumetazoa;Bilateria;Protostomia;Spiralia;Lophotrochozoa;Mollusca;Bivalvia;Autobranchia;Heteroconchia;Euheterodonta;Imparidentia;Venerida;Veneroidea;Veneridae;Mercenaria;Mercenaria mercenaria
UdeM00000016034 6579 240.7 UdeM00000016034 XP_033763223.1 45.1 346 173 6 1 333 154 495 2.60E-60 240.7 6579 cellular organisms;Eukaryota;Opisthokonta;Metazoa;Eumetazoa;Bilateria;Protostomia;Spiralia;Lophotrochozoa;Mollusca;Bivalvia;Autobranchia;Pteriomorphia;Pectinida;Pectinoidea;Pectinidae;Pecten;Pecten maximus
UdeM00000016035 6596 406 UdeM00000016035 XP_045156248.1 37.1 626 298 9 4 565 7 600 7.90E-110 406 6596 cellular organisms;Eukaryota;Opisthokonta;Metazoa;Eumetazoa;Bilateria;Protostomia;Spiralia;Lophotrochozoa;Mollusca;Bivalvia;Autobranchia;Heteroconchia;Euheterodonta;Imparidentia;Venerida;Veneroidea;Veneridae;Mercenaria;Mercenaria mercenaria
UdeM00000016036 6596 258.1 UdeM00000016036 XP_045164043.1 46.6 341 133 9 1 334 1 299 1.80E-65 258.1 6596 cellular organisms;Eukaryota;Opisthokonta;Metazoa;Eumetazoa;Bilateria;Protostomia;Spiralia;Lophotrochozoa;Mollusca;Bivalvia;Autobranchia;Heteroconchia;Euheterodonta;Imparidentia;Venerida;Veneroidea;Veneridae;Mercenaria;Mercenaria mercenaria
UdeM00000016037 6596 360.5 UdeM00000016037 XP_045163895.1 52 429 188 7 29 440 34 461 3.00E-96 360.5 6596 cellular organisms;Eukaryota;Opisthokonta;Metazoa;Eumetazoa;Bilateria;Protostomia;Spiralia;Lophotrochozoa;Mollusca;Bivalvia;Autobranchia;Heteroconchia;Euheterodonta;Imparidentia;Venerida;Veneroidea;Veneridae;Mercenaria;Mercenaria mercenaria
UdeM00000016039 29159 246.9 UdeM00000016039 XP_034319278.1 60.6 198 63 4 8 196 7 198 2.20E-62 246.9 29159 cellular organisms;Eukaryota;Opisthokonta;Metazoa;Eumetazoa;Bilateria;Protostomia;Spiralia;Lophotrochozoa;Mollusca;Bivalvia;Autobranchia;Pteriomorphia;Ostreida;Ostreoidea;Ostreidae;Crassostrea;Crassostrea gigas
UdeM00000016040 6565 270.4 UdeM00000016040 XP_022329522.1 59.8 241 84 4 1 233 1 236 2.20E-69 270.4 6565 cellular organisms;Eukaryota;Opisthokonta;Metazoa;Eumetazoa;Bilateria;Protostomia;Spiralia;Lophotrochozoa;Mollusca;Bivalvia;Autobranchia;Pteriomorphia;Ostreida;Ostreoidea;Ostreidae;Crassostrea;Crassostrea virginica
UdeM00000016041 29159 116.7 UdeM00000016041 XP_034311349.1 58.1 105 39 2 2 101 25 129 1.70E-23 116.7 29159 cellular organisms;Eukaryota;Opisthokonta;Metazoa;Eumetazoa;Bilateria;Protostomia;Spiralia;Lophotrochozoa;Mollusca;Bivalvia;Autobranchia;Pteriomorphia;Ostreida;Ostreoidea;Ostreidae;Crassostrea;Crassostrea gigas
UdeM00000016045 6579 352.4 UdeM00000016045 XP_033725098.1 41.1 506 235 6 40 483 259 763 8.90E-94 352.4 6579 cellular

organisms;Eukaryota;Opisthokonta;Metazoa;Eumetazoa;Bilateria;Protostomia;
Spiralia;Lophotrochozoa;Mollusca;Bivalvia;Autobranchia;Pteriomorphia;Pect
inida;Pectinoidea;Pectinidae;Pecten;Pecten maximus
UdeM00000044183 29159 91.7 UdeM00000044183 XP_034315421.1 36.4 143
83 2 91 226 333 474 1.30E-15 91.7 29159 cellular
organisms;Eukaryota;Opisthokonta;Metazoa;Eumetazoa;Bilateria;Protostomia;
Spiralia;Lophotrochozoa;Mollusca;Bivalvia;Autobranchia;Pteriomorphia;Ostr
eida;Ostreoidea;Ostreidae;Crassostrea;Crassostrea gigas
UdeM00000044184 6238 108.2 UdeM00000044184 XP_045091372.1 31.9 226
127 7 2 202 4 227 1.30E-20 108.2 6238 cellular
organisms;Eukaryota;Opisthokonta;Metazoa;Eumetazoa;Bilateria;Protostomia;
Ecdysozoa;Nematoda;Chromadorea;Rhabditida;Rhabditina;Rhabditomorpha;Rhabd
itoidea;Rhabditidae;Peloderinae;Caenorhabditis;Caenorhabditis briggsae
UdeM00000012421 6579 788.1 UdeM00000012421 XP_033751572.1 44.7 1094
480 36 1 1027 1 1036 1.30E-224 788.1 6579 cellular
organisms;Eukaryota;Opisthokonta;Metazoa;Eumetazoa;Bilateria;Protostomia;
Spiralia;Lophotrochozoa;Mollusca;Bivalvia;Autobranchia;Pteriomorphia;Pect
inida;Pectinoidea;Pectinidae;Pecten;Pecten maximus
UdeM00000012422 6596 718.4 UdeM00000012422 XP_045175535.1 76.1 477
108 1 1 471 1 477 6.20E-204 718.4 6596 cellular
organisms;Eukaryota;Opisthokonta;Metazoa;Eumetazoa;Bilateria;Protostomia;
Spiralia;Lophotrochozoa;Mollusca;Bivalvia;Autobranchia;Heteroconchia;Euhe
terodonta;Imparidentia;Venerida;Veneroidea;Veneridae;Mercenaria;Mercenari
a mercenaria
UdeM00000012423 6579 391.3 UdeM00000012423 XP_033752662.1 57.2 594
221 11 7 588 666 1238 2.20E-105 391.3 6579 cellular
organisms;Eukaryota;Opisthokonta;Metazoa;Eumetazoa;Bilateria;Protostomia;
Spiralia;Lophotrochozoa;Mollusca;Bivalvia;Autobranchia;Pteriomorphia;Pect
inida;Pectinoidea;Pectinidae;Pecten;Pecten maximus
UdeM00000012424 6573 201.8 UdeM00000012424 XP_021347988.1 39.3 412
218 13 1 401 250 640 1.60E-48 201.8 6573 cellular
organisms;Eukaryota;Opisthokonta;Metazoa;Eumetazoa;Bilateria;Protostomia;
Spiralia;Lophotrochozoa;Mollusca;Bivalvia;Autobranchia;Pteriomorphia;Pect
inida;Pectinoidea;Pectinidae;Mizuhopecten;Mizuhopecten yessoensis
UdeM00000012425 6596 514.2 UdeM00000012425 XP_045175535.1 65.8 383
127 4 30 409 1294 1675 1.50E-142 514.2 6596 cellular
organisms;Eukaryota;Opisthokonta;Metazoa;Eumetazoa;Bilateria;Protostomia;
Spiralia;Lophotrochozoa;Mollusca;Bivalvia;Autobranchia;Heteroconchia;Euhe
terodonta;Imparidentia;Venerida;Veneroidea;Veneridae;Mercenaria;Mercenari
a mercenaria
UdeM00000012426 7741 177.6 UdeM00000012426 XP_019639753.1 52.9 155
70 1 2 153 29 183 1.30E-41 177.6 7741 cellular
organisms;Eukaryota;Opisthokonta;Metazoa;Eumetazoa;Bilateria;Deuterostomi
a;Chordata;Cephalochordata;Leptocardii;Amphioxiformes;Branchiostomidae;Br
anchiostoma;Branchiostoma belcheri
UdeM00000012427 29159 689.9 UdeM00000012427 XP_011434736.2 50.6 725
325 11 140 841 15 729 4.10E-195 689.9 29159 cellular
organisms;Eukaryota;Opisthokonta;Metazoa;Eumetazoa;Bilateria;Protostomia;
Spiralia;Lophotrochozoa;Mollusca;Bivalvia;Autobranchia;Pteriomorphia;Ostr
eida;Ostreoidea;Ostreidae;Crassostrea;Crassostrea gigas
UdeM00000012429 6596 793.1 UdeM00000012429 XP_045174290.1 56.5 774
171 3 92 863 95 704 3.50E-226 793.1 6596 cellular
organisms;Eukaryota;Opisthokonta;Metazoa;Eumetazoa;Bilateria;Protostomia;
Spiralia;Lophotrochozoa;Mollusca;Bivalvia;Autobranchia;Heteroconchia;Euhe

terodonta;Imparidentia;Venerida;Veneroidea;Veneridae;Mercenaria;Mercenaria mercenaria
UdeM00000012430 6596 90.9 UdeM00000012430 XP_045176774.1 28.4 264 150 6 47 287 297 544 2.90E-15 90.9 6596 cellular organisms;Eukaryota;Opisthokonta;Metazoa;Eumetazoa;Bilateria;Protostomia;Spiralia;Lophotrochozoa;Mollusca;Bivalvia;Autobranchia;Heteroconchia;Euheterodonta;Imparidentia;Venerida;Veneroidea;Veneridae;Mercenaria;Mercenaria mercenaria
UdeM00000012431 6596 217.6 UdeM00000012431 XP_045176774.1 46.7 255 128 6 13 264 39 288 2.40E-53 217.6 6596 cellular organisms;Eukaryota;Opisthokonta;Metazoa;Eumetazoa;Bilateria;Protostomia;Spiralia;Lophotrochozoa;Mollusca;Bivalvia;Autobranchia;Heteroconchia;Euheterodonta;Imparidentia;Venerida;Veneroidea;Veneridae;Mercenaria;Mercenaria mercenaria
UdeM00000012432 6573 339 UdeM00000012432 XP_021342594.1 67.5 231 74 1 17 247 7 236 5.30E-90 339 6573 cellular organisms;Eukaryota;Opisthokonta;Metazoa;Eumetazoa;Bilateria;Protostomia;Spiralia;Lophotrochozoa;Mollusca;Bivalvia;Autobranchia;Pteriomorphia;Pectinida;Pectinoidea;Pectinidae;Mizuhopecten;Mizuhopecten yessoensis
UdeM00000012433 1735272 1465.7 UdeM00000012433 XP_041367703.1 82.6 861 150 0 1 861 245 1105 0.00E+00 1465.7 1735272 cellular organisms;Eukaryota;Opisthokonta;Metazoa;Eumetazoa;Bilateria;Protostomia;Spiralia;Lophotrochozoa;Mollusca;Gastropoda;Neomphalina;Neomphaloidea;Peltospiridae;Gigantopelta;Gigantopelta aegis
UdeM00000012434 6596 109.8 UdeM00000012434 XP_045174713.1 64.4 87 30 1 17 103 394 479 4.00E-21 109.8 6596 cellular organisms;Eukaryota;Opisthokonta;Metazoa;Eumetazoa;Bilateria;Protostomia;Spiralia;Lophotrochozoa;Mollusca;Bivalvia;Autobranchia;Heteroconchia;Euheterodonta;Imparidentia;Venerida;Veneroidea;Veneridae;Mercenaria;Mercenaria mercenaria
UdeM00000012435 6596 81.6 UdeM00000012435 XP_045174280.1 37.6 109 64 2 18 123 27 134 8.20E-13 81.6 6596 cellular organisms;Eukaryota;Opisthokonta;Metazoa;Eumetazoa;Bilateria;Protostomia;Spiralia;Lophotrochozoa;Mollusca;Bivalvia;Autobranchia;Heteroconchia;Euheterodonta;Imparidentia;Venerida;Veneroidea;Veneridae;Mercenaria;Mercenaria mercenaria
UdeM00000012436 6596 74.3 UdeM00000012436 XP_045174280.1 38 92 54 1 2 90 43 134 9.90E-11 74.3 6596 cellular organisms;Eukaryota;Opisthokonta;Metazoa;Eumetazoa;Bilateria;Protostomia;Spiralia;Lophotrochozoa;Mollusca;Bivalvia;Autobranchia;Heteroconchia;Euheterodonta;Imparidentia;Venerida;Veneroidea;Veneridae;Mercenaria;Mercenaria mercenaria
UdeM00000012437 400727 335.1 UdeM00000012437 XP_025088253.1 68.5 235 71 2 183 417 230 461 1.30E-88 335.1 400727 cellular organisms;Eukaryota;Opisthokonta;Metazoa;Eumetazoa;Bilateria;Protostomia;Spiralia;Lophotrochozoa;Mollusca;Gastropoda;Caenogastropoda;Architaenioglossa;Ampullarioidea;Ampullariidae;Pomacea;Pomacea canaliculata
UdeM00000012438 6596 973 UdeM00000012438 XP_045174274.1 73.6 682 137 13 28 693 9 663 2.00E-280 973 6596 cellular organisms;Eukaryota;Opisthokonta;Metazoa;Eumetazoa;Bilateria;Protostomia;Spiralia;Lophotrochozoa;Mollusca;Bivalvia;Autobranchia;Heteroconchia;Euhe

terodonta;Imparidentia;Venerida;Veneroidea;Veneridae;Mercenaria;Mercenari a mercenaria
UdeM00000012439 6596 844.3 UdeM00000012439 XP_045174275.1 50 996 136 13 7 989 17 663 1.50E-241 844.3 6596 cellular organisms;Eukaryota;Opisthokonta;Metazoa;Eumetazoa;Bilateria;Protostomia;Spiralia;Lophotrochozoa;Mollusca;Bivalvia;Autobranchia;Heteroconchia;Euheterodonta;Imparidentia;Venerida;Veneroidea;Veneridae;Mercenaria;Mercenari a mercenaria
UdeM00000012440 6596 666.8 UdeM00000012440 XP_045174293.1 70.1 461 136 2 1 460 753 1212 3.00E-188 666.8 6596 cellular organisms;Eukaryota;Opisthokonta;Metazoa;Eumetazoa;Bilateria;Protostomia;Spiralia;Lophotrochozoa;Mollusca;Bivalvia;Autobranchia;Heteroconchia;Euheterodonta;Imparidentia;Venerida;Veneroidea;Veneridae;Mercenaria;Mercenari a mercenaria
UdeM00000012441 6596 788.9 UdeM00000012441 XP_045174293.1 77.8 505 112 0 38 542 34 538 4.20E-225 788.9 6596 cellular organisms;Eukaryota;Opisthokonta;Metazoa;Eumetazoa;Bilateria;Protostomia;Spiralia;Lophotrochozoa;Mollusca;Bivalvia;Autobranchia;Heteroconchia;Euheterodonta;Imparidentia;Venerida;Veneroidea;Veneridae;Mercenaria;Mercenari a mercenaria
UdeM00000012442 2607531 270 UdeM00000012442 XP_029642573.2 29.3 600 330 16 23 549 712 1290 7.00E-69 270 2607531 cellular organisms;Eukaryota;Opisthokonta;Metazoa;Eumetazoa;Bilateria;Protostomia;Spiralia;Lophotrochozoa;Mollusca;Cephalopoda;Coleoidea;Octopodiformes;Octopoda;Incirrata;Octopodidae;Octopus;Octopus sinensis
UdeM00000012447 6573 157.9 UdeM00000012447 XP_021368660.1 33.8 240 141 4 8 231 13 250 1.60E-35 157.9 6573 cellular organisms;Eukaryota;Opisthokonta;Metazoa;Eumetazoa;Bilateria;Protostomia;Spiralia;Lophotrochozoa;Mollusca;Bivalvia;Autobranchia;Pteriomorphia;Pectinida;Pectinoidea;Pectinidae;Mizuhopecten;Mizuhopecten yessoensis
UdeM00000012448 6596 496.9 UdeM00000012448 XP_045176042.1 44.6 518 277 6 191 703 50 562 4.30E-137 496.9 6596 cellular organisms;Eukaryota;Opisthokonta;Metazoa;Eumetazoa;Bilateria;Protostomia;Spiralia;Lophotrochozoa;Mollusca;Bivalvia;Autobranchia;Heteroconchia;Euheterodonta;Imparidentia;Venerida;Veneroidea;Veneridae;Mercenaria;Mercenari a mercenaria
UdeM00000012449 6596 139.4 UdeM00000012449 XP_045175306.1 39 213 115 3 1 211 24 223 5.30E-30 139.4 6596 cellular organisms;Eukaryota;Opisthokonta;Metazoa;Eumetazoa;Bilateria;Protostomia;Spiralia;Lophotrochozoa;Mollusca;Bivalvia;Autobranchia;Heteroconchia;Euheterodonta;Imparidentia;Venerida;Veneroidea;Veneridae;Mercenaria;Mercenari a mercenaria
UdeM00000012450 6573 84.3 UdeM00000012450 XP_021353116.1 30.2 252 135 9 13 241 38 271 2.60E-13 84.3 6573 cellular organisms;Eukaryota;Opisthokonta;Metazoa;Eumetazoa;Bilateria;Protostomia;Spiralia;Lophotrochozoa;Mollusca;Bivalvia;Autobranchia;Pteriomorphia;Pectinida;Pectinoidea;Pectinidae;Mizuhopecten;Mizuhopecten yessoensis
UdeM00000012451 6596 151.4 UdeM00000012451 XP_045175679.1 32.9 462 159 16 55 414 49 461 2.70E-33 151.4 6596 cellular organisms;Eukaryota;Opisthokonta;Metazoa;Eumetazoa;Bilateria;Protostomia;Spiralia;Lophotrochozoa;Mollusca;Bivalvia;Autobranchia;Heteroconchia;Euheterodonta;Imparidentia;Venerida;Veneroidea;Veneridae;Mercenaria;Mercenari a mercenaria

UdeM00000012452 6596 152.9 UdeM00000012452 XP_045175679.1 32.1 483
177 16 33 413 28 461 9.30E-34 152.9 6596 cellular
organisms;Eukaryota;Opisthokonta;Metazoa;Eumetazoa;Bilateria;Protostomia;Spiralia;Lophotrochozoa;Mollusca;Bivalvia;Autobranchia;Heteroconchia;Euheterodonta;Imparidentia;Venerida;Veneroidea;Veneridae;Mercenaria;Mercenaria mercenaria
UdeM00000012453 29159 429.1 UdeM00000012453 XP_011444138.2 49 527
249 7 30 541 3 524 8.40E-117 429.1 29159 cellular
organisms;Eukaryota;Opisthokonta;Metazoa;Eumetazoa;Bilateria;Protostomia;Spiralia;Lophotrochozoa;Mollusca;Bivalvia;Autobranchia;Pteriomorphia;Ostreida;Ostreoidea;Ostreidae;Crassostrea;Crassostrea gigas
UdeM00000012454 29159 282.3 UdeM00000012454 XP_011442603.1 75 164
41 0 1 164 1 164 3.80E-73 282.3 29159 cellular
organisms;Eukaryota;Opisthokonta;Metazoa;Eumetazoa;Bilateria;Protostomia;Spiralia;Lophotrochozoa;Mollusca;Bivalvia;Autobranchia;Pteriomorphia;Ostreida;Ostreoidea;Ostreidae;Crassostrea;Crassostrea gigas
UdeM00000012455 29159 273.1 UdeM00000012455 XP_011442603.1 72.6 164
45 0 1 164 1 164 2.30E-70 273.1 29159 cellular
organisms;Eukaryota;Opisthokonta;Metazoa;Eumetazoa;Bilateria;Protostomia;Spiralia;Lophotrochozoa;Mollusca;Bivalvia;Autobranchia;Pteriomorphia;Ostreida;Ostreoidea;Ostreidae;Crassostrea;Crassostrea gigas
UdeM00000012457 6573 772.3 UdeM00000012457 XP_021345779.1 45.6 1032
460 22 54 1007 178 1186 7.50E-220 772.3 6573 cellular
organisms;Eukaryota;Opisthokonta;Metazoa;Eumetazoa;Bilateria;Protostomia;Spiralia;Lophotrochozoa;Mollusca;Bivalvia;Autobranchia;Pteriomorphia;Pectinida;Pectinoidea;Pectinidae;Mizuhopecten;Mizuhopecten yessoensis
UdeM00000012458 6579 326.6 UdeM00000012458 XP_033753032.1 27 1385
772 50 21 1353 2 1199 1.50E-85 326.6 6579 cellular
organisms;Eukaryota;Opisthokonta;Metazoa;Eumetazoa;Bilateria;Protostomia;Spiralia;Lophotrochozoa;Mollusca;Bivalvia;Autobranchia;Pteriomorphia;Pectinida;Pectinoidea;Pectinidae;Pecten;Pecten maximus
UdeM00000012459 6596 97.1 UdeM00000012459 XP_045177102.1 48 127
55 3 36 152 34 159 2.20E-17 97.1 6596 cellular
organisms;Eukaryota;Opisthokonta;Metazoa;Eumetazoa;Bilateria;Protostomia;Spiralia;Lophotrochozoa;Mollusca;Bivalvia;Autobranchia;Heteroconchia;Euheterodonta;Imparidentia;Venerida;Veneroidea;Veneridae;Mercenaria;Mercenaria mercenaria
UdeM00000012460 6573 150.2 UdeM00000012460 XP_021356433.1 54 139
63 1 1 138 330 468 2.90E-33 150.2 6573 cellular
organisms;Eukaryota;Opisthokonta;Metazoa;Eumetazoa;Bilateria;Protostomia;Spiralia;Lophotrochozoa;Mollusca;Bivalvia;Autobranchia;Pteriomorphia;Pectinida;Pectinoidea;Pectinidae;Mizuhopecten;Mizuhopecten yessoensis
UdeM00000012461 6579 122.1 UdeM00000012461 XP_033751890.1 34.7 199
110 4 8 196 109 297 8.10E-25 122.1 6579 cellular
organisms;Eukaryota;Opisthokonta;Metazoa;Eumetazoa;Bilateria;Protostomia;Spiralia;Lophotrochozoa;Mollusca;Bivalvia;Autobranchia;Pteriomorphia;Pectinida;Pectinoidea;Pectinidae;Pecten;Pecten maximus
UdeM00000012463 6596 1290.8 UdeM00000012463 XP_045175283.1 77.5
835 184 3 1 834 165 996 0.00E+00 1290.8 6596
cellular
organisms;Eukaryota;Opisthokonta;Metazoa;Eumetazoa;Bilateria;Protostomia;Spiralia;Lophotrochozoa;Mollusca;Bivalvia;Autobranchia;Heteroconchia;Euheterodonta;Imparidentia;Venerida;Veneroidea;Veneridae;Mercenaria;Mercenaria mercenaria

UdeM00000012464 225164 75.9 UdeM00000012464 XP_009051402.1 32.5
154 89 5 15 159 215 362 3.60E-10 75.9 225164
cellular
organisms;Eukaryota;Opisthokonta;Metazoa;Eumetazoa;Bilateria;Protostomia;
Spiralia;Lophotrochozoa;Mollusca;Gastropoda;Patellogastropoda;Lottioidea;
Lottiidae;Lottia;Lottia gigantea
UdeM00000012465 6573 607.4 UdeM00000012465 XP_021368124.1 52.7 674
257 17 56 682 24 682 2.20E-170 607.4 6573 cellular
organisms;Eukaryota;Opisthokonta;Metazoa;Eumetazoa;Bilateria;Protostomia;
Spiralia;Lophotrochozoa;Mollusca;Bivalvia;Autobranchia;Pteriomorphia;Pect
inida;Pectinoidea;Pectinidae;Mizuhopecten;Mizuhopecten yessoensis
UdeM00000012466 6596 286.6 UdeM00000012466 XP_045174259.1 61.8 220
82 1 1 218 1831 2050 2.80E-74 286.6 6596 cellular
organisms;Eukaryota;Opisthokonta;Metazoa;Eumetazoa;Bilateria;Protostomia;
Spiralia;Lophotrochozoa;Mollusca;Bivalvia;Autobranchia;Heteroconchia;Euhe
terodonta;Imparidentia;Venerida;Veneroidea;Veneridae;Mercenaria;Mercenari
a mercenaria
UdeM00000012467 1735272 141 UdeM00000012467 XP_041367077.1 42
176 51 2 4 128 726 901 1.10E-30 141 1735272
cellular
organisms;Eukaryota;Opisthokonta;Metazoa;Eumetazoa;Bilateria;Protostomia;
Spiralia;Lophotrochozoa;Mollusca;Gastropoda;Neomphalina;Neomphaloidea;Pel
tospiridae;Gigantopelta;Gigantopelta aegis
UdeM00000012468 225164 147.9 UdeM00000012468 XP_009066022.1 26.3
577 286 12 5 477 115 656 3.30E-32 147.9 225164
cellular
organisms;Eukaryota;Opisthokonta;Metazoa;Eumetazoa;Bilateria;Protostomia;
Spiralia;Lophotrochozoa;Mollusca;Gastropoda;Patellogastropoda;Lottioidea;
Lottiidae;Lottia;Lottia gigantea
UdeM00000012469 6596 349.7 UdeM00000012469 XP_045174259.1 63.1 268
97 1 1 266 1783 2050 3.30E-93 349.7 6596 cellular
organisms;Eukaryota;Opisthokonta;Metazoa;Eumetazoa;Bilateria;Protostomia;
Spiralia;Lophotrochozoa;Mollusca;Bivalvia;Autobranchia;Heteroconchia;Euhe
terodonta;Imparidentia;Venerida;Veneroidea;Veneridae;Mercenaria;Mercenari
a mercenaria
UdeM00000012470 6596 1498.8 UdeM00000012470 XP_045174259.1 52.6
1382 620 9 158 1533 261 1613 0.00E+00 1498.8 6596
cellular
organisms;Eukaryota;Opisthokonta;Metazoa;Eumetazoa;Bilateria;Protostomia;
Spiralia;Lophotrochozoa;Mollusca;Bivalvia;Autobranchia;Heteroconchia;Euhe
terodonta;Imparidentia;Venerida;Veneroidea;Veneridae;Mercenaria;Mercenari
a mercenaria
UdeM00000012471 6596 2095.9 UdeM00000012471 XP_045174259.1 56.2
1819 761 10 158 1969 261 2050 0.00E+00 2095.9 6596
cellular
organisms;Eukaryota;Opisthokonta;Metazoa;Eumetazoa;Bilateria;Protostomia;
Spiralia;Lophotrochozoa;Mollusca;Bivalvia;Autobranchia;Heteroconchia;Euhe
terodonta;Imparidentia;Venerida;Veneroidea;Veneridae;Mercenaria;Mercenari
a mercenaria
UdeM00000012472 6596 2167.5 UdeM00000012472 XP_045174259.1 53
2075 910 19 1 2061 28 2050 0.00E+00 2167.5 6596
cellular
organisms;Eukaryota;Opisthokonta;Metazoa;Eumetazoa;Bilateria;Protostomia;
Spiralia;Lophotrochozoa;Mollusca;Bivalvia;Autobranchia;Heteroconchia;Euhe

terodonta;Imparidentia;Venerida;Veneroidea;Veneridae;Mercenaria;Mercenaria mercenaria
UdeM00000012473 6573 259.2 UdeM00000012473 XP_021368296.1 34.5 632 313 20 1 573 1 590 1.80E-65 259.2 6573 cellular organisms;Eukaryota;Opisthokonta;Metazoa;Eumetazoa;Bilateria;Protostomia;Spiralia;Lophotrochozoa;Mollusca;Bivalvia;Autobranchia;Pteriomorphia;Pectinida;Pectinoidea;Pectinidae;Mizuhopecten;Mizuhopecten yessoensis
UdeM00000012474 6579 198.7 UdeM00000012474 XP_033752953.1 40.2 264 143 3 3 266 560 808 9.30E-48 198.7 6579 cellular organisms;Eukaryota;Opisthokonta;Metazoa;Eumetazoa;Bilateria;Protostomia;Spiralia;Lophotrochozoa;Mollusca;Bivalvia;Autobranchia;Pteriomorphia;Pectinida;Pectinoidea;Pectinidae;Pecten;Pecten maximus
UdeM00000012475 6596 65.5 UdeM00000012475 XP_045176763.1 58.6 58 18 2 10 61 19 76 5.60E-08 65.5 6596 cellular organisms;Eukaryota;Opisthokonta;Metazoa;Eumetazoa;Bilateria;Protostomia;Spiralia;Lophotrochozoa;Mollusca;Bivalvia;Autobranchia;Heteroconchia;Euheterodonta;Imparidentia;Venerida;Veneroidea;Veneridae;Mercenaria;Mercenaria mercenaria
UdeM00000012476 1735272 522.7 UdeM00000012476 XP_041356796.1 50.8 543 252 8 83 619 59 592 6.40E-145 522.7 1735272 cellular organisms;Eukaryota;Opisthokonta;Metazoa;Eumetazoa;Bilateria;Protostomia;Spiralia;Lophotrochozoa;Mollusca;Gastropoda;Neomphalina;Neomphaloidea;Peltospiridae;Gigantopelta;Gigantopelta aegis
UdeM00000012477 6596 698.4 UdeM00000012477 XP_045176730.1 65.3 487 169 0 1 487 1 487 6.70E-198 698.4 6596 cellular organisms;Eukaryota;Opisthokonta;Metazoa;Eumetazoa;Bilateria;Protostomia;Spiralia;Lophotrochozoa;Mollusca;Bivalvia;Autobranchia;Heteroconchia;Euheterodonta;Imparidentia;Venerida;Veneroidea;Veneridae;Mercenaria;Mercenaria mercenaria
UdeM00000012478 6596 699.9 UdeM00000012478 XP_045176730.1 64.9 490 172 0 1 490 1 490 2.30E-198 699.9 6596 cellular organisms;Eukaryota;Opisthokonta;Metazoa;Eumetazoa;Bilateria;Protostomia;Spiralia;Lophotrochozoa;Mollusca;Bivalvia;Autobranchia;Heteroconchia;Euheterodonta;Imparidentia;Venerida;Veneroidea;Veneridae;Mercenaria;Mercenaria mercenaria
UdeM00000012479 6596 217.6 UdeM00000012479 XP_045176442.1 34.5 368 166 4 19 313 65 430 2.20E-53 217.6 6596 cellular organisms;Eukaryota;Opisthokonta;Metazoa;Eumetazoa;Bilateria;Protostomia;Spiralia;Lophotrochozoa;Mollusca;Bivalvia;Autobranchia;Heteroconchia;Euheterodonta;Imparidentia;Venerida;Veneroidea;Veneridae;Mercenaria;Mercenaria mercenaria
UdeM00000012480 46514 136.3 UdeM00000012480 XP_038053347.1 33.3 210 137 3 2 210 574 781 4.50E-29 136.3 46514 cellular organisms;Eukaryota;Opisthokonta;Metazoa;Eumetazoa;Bilateria;Deuterostomia;Echinodermata;Eleutherozoa;Asterozoa;Asteroidea;Valvatacea;Valvatida;Asterinidae;Patiria;Patiria miniata
UdeM00000012481 7998 590.9 UdeM00000012481 XP_017334791.1 66.6 422 141 0 1 422 1 422 2.10E-165 590.9 7998 cellular organisms;Eukaryota;Opisthokonta;Metazoa;Eumetazoa;Bilateria;Deuterostomia;Chordata;Craniata;Vertebrata;Gnathostomata;Teleostomi;Euteleostomi;Actinopterygii;Actinopteri;Neopterygii;Teleostei;Osteoglossocephalai;Clupeocephala;Otomorpha;Ostariophysi;Otophysi;Characiphysae;Siluriformes;Siluroidei;Ictaluridae;Ictalurus;Ictalurus punctatus

UdeM00000012482 6596 283.1 UdeM00000012482 XP_045174627.1 43.3 323
175 3 4 318 1511 1833 4.40E-73 283.1 6596 cellular
organisms;Eukaryota;Opisthokonta;Metazoa;Eumetazoa;Bilateria;Protostomia;
Spiralia;Lophotrochozoa;Mollusca;Bivalvia;Autobranchia;Heteroconchia;Euhe
terodonta;Imparidentia;Venerida;Veneroidea;Veneridae;Mercenaria;Mercenari
a mercenaria
UdeM00000012483 6579 136 UdeM00000012483 XP_033751294.1 63.6 88
32 0 3 90 1875 1962 2.80E-29 136 6579 cellular
organisms;Eukaryota;Opisthokonta;Metazoa;Eumetazoa;Bilateria;Protostomia;
Spiralia;Lophotrochozoa;Mollusca;Bivalvia;Autobranchia;Pteriomorphia;Pect
inida;Pectinoidea;Pectinidae;Pecten;Pecten maximus
UdeM00000012484 6579 447.2 UdeM00000012484 XP_033751294.1 43.6 518
225 8 3 455 1875 2390 2.60E-122 447.2 6579 cellular
organisms;Eukaryota;Opisthokonta;Metazoa;Eumetazoa;Bilateria;Protostomia;
Spiralia;Lophotrochozoa;Mollusca;Bivalvia;Autobranchia;Pteriomorphia;Pect
inida;Pectinoidea;Pectinidae;Pecten;Pecten maximus
UdeM00000012485 6579 326.6 UdeM00000012485 XP_033751294.1 41.7 412
173 8 4 350 1981 2390 3.90E-86 326.6 6579 cellular
organisms;Eukaryota;Opisthokonta;Metazoa;Eumetazoa;Bilateria;Protostomia;
Spiralia;Lophotrochozoa;Mollusca;Bivalvia;Autobranchia;Pteriomorphia;Pect
inida;Pectinoidea;Pectinidae;Pecten;Pecten maximus
UdeM00000009127 6565 193 UdeM00000009127 XP_022287659.1 42.1 247
136 4 11 253 8 251 5.50E-46 193 6565 cellular
organisms;Eukaryota;Opisthokonta;Metazoa;Eumetazoa;Bilateria;Protostomia;
Spiralia;Lophotrochozoa;Mollusca;Bivalvia;Autobranchia;Pteriomorphia;Ostr
eida;Ostreoidea;Ostreidae;Crassostrea;Crassostrea virginica
UdeM00000009128 29159 565.8 UdeM00000009128 XP_034330259.1 41.6 735
381 13 245 964 2 703 1.00E-157 565.8 29159 cellular
organisms;Eukaryota;Opisthokonta;Metazoa;Eumetazoa;Bilateria;Protostomia;
Spiralia;Lophotrochozoa;Mollusca;Bivalvia;Autobranchia;Pteriomorphia;Ostr
eida;Ostreoidea;Ostreidae;Crassostrea;Crassostrea gigas
UdeM00000009130 29159 135.6 UdeM00000009130 XP_034315421.1 35.7 258
107 5 40 242 221 474 8.60E-29 135.6 29159 cellular
organisms;Eukaryota;Opisthokonta;Metazoa;Eumetazoa;Bilateria;Protostomia;
Spiralia;Lophotrochozoa;Mollusca;Bivalvia;Autobranchia;Pteriomorphia;Ostr
eida;Ostreoidea;Ostreidae;Crassostrea;Crassostrea gigas
UdeM00000009132 29159 321.2 UdeM00000009132 XP_034318666.1 65.8 219
75 0 1 219 482 700 1.00E-84 321.2 29159 cellular
organisms;Eukaryota;Opisthokonta;Metazoa;Eumetazoa;Bilateria;Protostomia;
Spiralia;Lophotrochozoa;Mollusca;Bivalvia;Autobranchia;Pteriomorphia;Ostr
eida;Ostreoidea;Ostreidae;Crassostrea;Crassostrea gigas
UdeM00000009133 29159 351.3 UdeM00000009133 XP_034318948.1 41.6 473
160 3 1 357 481 953 1.50E-93 351.3 29159 cellular
organisms;Eukaryota;Opisthokonta;Metazoa;Eumetazoa;Bilateria;Protostomia;
Spiralia;Lophotrochozoa;Mollusca;Bivalvia;Autobranchia;Pteriomorphia;Ostr
eida;Ostreoidea;Ostreidae;Crassostrea;Crassostrea gigas
UdeM00000009134 6565 200.3 UdeM00000009134 XP_022306954.1 44.3 212
116 1 1 210 737 948 2.50E-48 200.3 6565 cellular
organisms;Eukaryota;Opisthokonta;Metazoa;Eumetazoa;Bilateria;Protostomia;
Spiralia;Lophotrochozoa;Mollusca;Bivalvia;Autobranchia;Pteriomorphia;Ostr
eida;Ostreoidea;Ostreidae;Crassostrea;Crassostrea virginica
UdeM00000009135 6706 151 UdeM00000009135 XP_042235765.1 43.9 157
88 0 1 157 1 157 1.30E-33 151 6706 cellular
organisms;Eukaryota;Opisthokonta;Metazoa;Eumetazoa;Bilateria;Protostomia;

Ecdysozoa;Panarthropoda;Arthropoda;Mandibulata;Pancrustacea;Crustacea;Multicrustacea;Malacostraca;Eumalacostraca;Eucarida;Decapoda;Pleocyemata;Astacidea;Nephropoidea;Nephropidae;Homarus;Homarus americanus
UdeM00000009136 7906 119 UdeM00000009136 XP_034783435.1 26.8 384 248 7 60 414 46 425 1.80E-23 119 7906 cellular organisms;Eukaryota;Opisthokonta;Metazoa;Eumetazoa;Bilateria;Deuterostomia;Chordata;Craniata;Vertebrata;Gnathostomata;Teleostomi;Euteleostomi;Actinopterygii;Actinopteri;Chondrostei;Acipenseriformes;Acipenseroidei;Acipenseridae;Acipenserinae;Acipenserini;Acipenser;Acipenser ruthenus
UdeM00000009137 6596 68.9 UdeM00000009137 XP_045208259.1 50.7 67 30 1 8 71 239 305 3.80E-09 68.9 6596 cellular organisms;Eukaryota;Opisthokonta;Metazoa;Eumetazoa;Bilateria;Protostomia;Spiralia;Lophotrochozoa;Mollusca;Bivalvia;Autobranchia;Heteroconchia;Euheterodonta;Imparidentia;Venerida;Veneroidea;Veneridae;Mercenaria;Mercenaria mercenaria
UdeM00000009143 6596 132.1 UdeM00000009143 XP_045181000.1 62.6 99 37 0 8 106 856 954 4.30E-28 132.1 6596 cellular organisms;Eukaryota;Opisthokonta;Metazoa;Eumetazoa;Bilateria;Protostomia;Spiralia;Lophotrochozoa;Mollusca;Bivalvia;Autobranchia;Heteroconchia;Euheterodonta;Imparidentia;Venerida;Veneroidea;Veneridae;Mercenaria;Mercenaria mercenaria
UdeM00000009144 6596 185.3 UdeM00000009144 XP_045182316.1 45.8 227 119 3 1 227 392 614 1.40E-43 185.3 6596 cellular organisms;Eukaryota;Opisthokonta;Metazoa;Eumetazoa;Bilateria;Protostomia;Spiralia;Lophotrochozoa;Mollusca;Bivalvia;Autobranchia;Heteroconchia;Euheterodonta;Imparidentia;Venerida;Veneroidea;Veneridae;Mercenaria;Mercenaria mercenaria
UdeM00000012032 7741 282.3 UdeM00000012032 XP_019622111.1 50.4 280 136 3 5 283 1 278 6.60E-73 282.3 7741 cellular organisms;Eukaryota;Opisthokonta;Metazoa;Eumetazoa;Bilateria;Deuterostomia;Chordata;Cephalochordata;Leptocardii;Amphioxiformes;Branchiostomidae;Branchiostoma;Branchiostoma belcheri
UdeM00000012033 6596 102.1 UdeM00000012033 XP_045181091.1 30.2 298 167 10 6 286 121 394 1.70E-18 102.1 6596 cellular organisms;Eukaryota;Opisthokonta;Metazoa;Eumetazoa;Bilateria;Protostomia;Spiralia;Lophotrochozoa;Mollusca;Bivalvia;Autobranchia;Heteroconchia;Euheterodonta;Imparidentia;Venerida;Veneroidea;Veneridae;Mercenaria;Mercenaria mercenaria
UdeM00000012034 6596 201.1 UdeM00000012034 XP_045181091.1 34.8 376 219 6 31 397 28 386 3.30E-48 201.1 6596 cellular organisms;Eukaryota;Opisthokonta;Metazoa;Eumetazoa;Bilateria;Protostomia;Spiralia;Lophotrochozoa;Mollusca;Bivalvia;Autobranchia;Heteroconchia;Euheterodonta;Imparidentia;Venerida;Veneroidea;Veneridae;Mercenaria;Mercenaria mercenaria
UdeM00000012035 6573 1653.6 UdeM00000012035 XP_021354423.1 65.2 1195 403 8 2 1186 3 1194 0.00E+00 1653.6 6573 cellular organisms;Eukaryota;Opisthokonta;Metazoa;Eumetazoa;Bilateria;Protostomia;Spiralia;Lophotrochozoa;Mollusca;Bivalvia;Autobranchia;Pteriomorphia;Pectinida;Pectinoidea;Pectinidae;Mizuhopecten;Mizuhopecten yessoensis
UdeM00000012036 6596 458.8 UdeM00000012036 XP_045182653.1 49.6 448 215 5 12 448 1180 1627 8.40E-126 458.8 6596 cellular organisms;Eukaryota;Opisthokonta;Metazoa;Eumetazoa;Bilateria;Protostomia;Spiralia;Lophotrochozoa;Mollusca;Bivalvia;Autobranchia;Heteroconchia;Euhe

terodonta;Imparidentia;Venerida;Veneroidea;Veneridae;Mercenaria;Mercenaria mercenaria
UdeM00000012037 6579 558.9 UdeM00000012037 XP_033726516.1 42.9 758 382 12 1 734 1 731 9.50E-156 558.9 6579 cellular organisms;Eukaryota;Opisthokonta;Metazoa;Eumetazoa;Bilateria;Protostomia;Spiralia;Lophotrochozoa;Mollusca;Bivalvia;Autobranchia;Pteriomorphia;Pectinida;Pectinoidea;Pectinidae;Pecten;Pecten maximus
UdeM00000012038 6596 461.5 UdeM00000012038 XP_045182653.1 59.9 416 158 7 1 409 1635 2048 1.10E-126 461.5 6596 cellular organisms;Eukaryota;Opisthokonta;Metazoa;Eumetazoa;Bilateria;Protostomia;Spiralia;Lophotrochozoa;Mollusca;Bivalvia;Autobranchia;Heteroconchia;Euheterodonta;Imparidentia;Venerida;Veneroidea;Veneridae;Mercenaria;Mercenaria mercenaria
UdeM00000012039 6596 469.5 UdeM00000012039 XP_045182653.1 60.3 416 158 6 1 411 1635 2048 4.20E-129 469.5 6596 cellular organisms;Eukaryota;Opisthokonta;Metazoa;Eumetazoa;Bilateria;Protostomia;Spiralia;Lophotrochozoa;Mollusca;Bivalvia;Autobranchia;Heteroconchia;Euheterodonta;Imparidentia;Venerida;Veneroidea;Veneridae;Mercenaria;Mercenaria mercenaria
UdeM00000012040 6596 350.1 UdeM00000012040 XP_045170643.1 49.6 357 166 4 31 379 32 382 3.50E-93 350.1 6596 cellular organisms;Eukaryota;Opisthokonta;Metazoa;Eumetazoa;Bilateria;Protostomia;Spiralia;Lophotrochozoa;Mollusca;Bivalvia;Autobranchia;Heteroconchia;Euheterodonta;Imparidentia;Venerida;Veneroidea;Veneridae;Mercenaria;Mercenaria mercenaria
UdeM00000012041 34638 72 UdeM00000012041 XP_003738265.1 53.5 71 30 1 9 76 10 80 4.50E-10 72 34638 cellular organisms;Eukaryota;Opisthokonta;Metazoa;Eumetazoa;Bilateria;Protostomia;Ecdysozoa;Panarthropoda;Arthropoda;Chelicerata;Arachnida;Acari;Parasitiformes;Mesostigmata;Monogynaspida;Gamasina;Phytoseioidea;Phytoseiidae;Typhlodrominae;Galendromus;Galendromus occidentalis
UdeM00000012042 6596 573.2 UdeM00000012042 XP_045182120.1 67.7 462 120 3 1 433 1 462 2.90E-160 573.2 6596 cellular organisms;Eukaryota;Opisthokonta;Metazoa;Eumetazoa;Bilateria;Protostomia;Spiralia;Lophotrochozoa;Mollusca;Bivalvia;Autobranchia;Heteroconchia;Euheterodonta;Imparidentia;Venerida;Veneroidea;Veneridae;Mercenaria;Mercenaria mercenaria
UdeM00000012043 6596 622.1 UdeM00000012043 XP_045182118.1 72.2 439 105 4 1 428 40 472 5.30E-175 622.1 6596 cellular organisms;Eukaryota;Opisthokonta;Metazoa;Eumetazoa;Bilateria;Protostomia;Spiralia;Lophotrochozoa;Mollusca;Bivalvia;Autobranchia;Heteroconchia;Euheterodonta;Imparidentia;Venerida;Veneroidea;Veneridae;Mercenaria;Mercenaria mercenaria
UdeM00000012044 6596 225.3 UdeM00000012044 XP_045172484.1 25.3 668 458 17 16 667 78 720 2.30E-55 225.3 6596 cellular organisms;Eukaryota;Opisthokonta;Metazoa;Eumetazoa;Bilateria;Protostomia;Spiralia;Lophotrochozoa;Mollusca;Bivalvia;Autobranchia;Heteroconchia;Euheterodonta;Imparidentia;Venerida;Veneroidea;Veneridae;Mercenaria;Mercenaria mercenaria
UdeM00000012045 6596 246.9 UdeM00000012045 XP_045182096.1 41.4 370 173 9 1 353 118 460 4.00E-62 246.9 6596 cellular organisms;Eukaryota;Opisthokonta;Metazoa;Eumetazoa;Bilateria;Protostomia;Spiralia;Lophotrochozoa;Mollusca;Bivalvia;Autobranchia;Heteroconchia;Euhe

terodonta;Imparidentia;Venerida;Veneroidea;Veneridae;Mercenaria;Mercenaria mercenaria
UdeM00000012047 6579 68.6 UdeM00000012047 XP_033755850.1 42.4 85 49 0 9 93 321 405 6.30E-09 68.6 6579 cellular organisms;Eukaryota;Opisthokonta;Metazoa;Eumetazoa;Bilateria;Protostomia;Spiralia;Lophotrochozoa;Mollusca;Bivalvia;Autobranchia;Pteriomorphia;Pectinida;Pectinoidea;Pectinidae;Pecten;Pecten maximus
UdeM00000012049 6596 283.9 UdeM00000012049 XP_045182305.1 46.1 323 166 3 2 318 3 323 3.30E-73 283.9 6596 cellular organisms;Eukaryota;Opisthokonta;Metazoa;Eumetazoa;Bilateria;Protostomia;Spiralia;Lophotrochozoa;Mollusca;Bivalvia;Autobranchia;Heteroconchia;Euheterodonta;Imparidentia;Venerida;Veneroidea;Veneridae;Mercenaria;Mercenaria mercenaria
UdeM00000012050 6596 880.2 UdeM00000012050 XP_045180223.1 65.2 741 217 13 2 708 3 736 2.10E-252 880.2 6596 cellular organisms;Eukaryota;Opisthokonta;Metazoa;Eumetazoa;Bilateria;Protostomia;Spiralia;Lophotrochozoa;Mollusca;Bivalvia;Autobranchia;Heteroconchia;Euheterodonta;Imparidentia;Venerida;Veneroidea;Veneridae;Mercenaria;Mercenaria mercenaria
UdeM00000012051 6573 773.9 UdeM00000012051 XP_021343413.1 69.3 534 164 0 3 536 5 538 1.40E-220 773.9 6573 cellular organisms;Eukaryota;Opisthokonta;Metazoa;Eumetazoa;Bilateria;Protostomia;Spiralia;Lophotrochozoa;Mollusca;Bivalvia;Autobranchia;Pteriomorphia;Pectinida;Pectinoidea;Pectinidae;Mizuhopecten;Mizuhopecten yessoensis
UdeM00000012052 29159 204.1 UdeM00000012052 XP_011445135.2 45.7 258 135 3 94 347 172 428 2.90E-49 204.1 29159 cellular organisms;Eukaryota;Opisthokonta;Metazoa;Eumetazoa;Bilateria;Protostomia;Spiralia;Lophotrochozoa;Mollusca;Bivalvia;Autobranchia;Pteriomorphia;Ostreida;Ostreoidea;Ostreidae;Crassostrea;Crassostrea gigas
UdeM00000012054 6565 213.4 UdeM00000012054 XP_022345146.1 29.8 413 287 2 5 414 761 1173 5.80E-52 213.4 6565 cellular organisms;Eukaryota;Opisthokonta;Metazoa;Eumetazoa;Bilateria;Protostomia;Spiralia;Lophotrochozoa;Mollusca;Bivalvia;Autobranchia;Pteriomorphia;Ostreida;Ostreoidea;Ostreidae;Crassostrea;Crassostrea virginica
UdeM00000012055 6565 285.4 UdeM00000012055 XP_022337165.1 65.3 216 66 1 8 223 6 212 6.20E-74 285.4 6565 cellular organisms;Eukaryota;Opisthokonta;Metazoa;Eumetazoa;Bilateria;Protostomia;Spiralia;Lophotrochozoa;Mollusca;Bivalvia;Autobranchia;Pteriomorphia;Ostreida;Ostreoidea;Ostreidae;Crassostrea;Crassostrea virginica
UdeM00000012056 6573 375.6 UdeM00000012056 XP_021371458.1 38.9 565 281 8 158 690 251 783 1.40E-100 375.6 6573 cellular organisms;Eukaryota;Opisthokonta;Metazoa;Eumetazoa;Bilateria;Protostomia;Spiralia;Lophotrochozoa;Mollusca;Bivalvia;Autobranchia;Pteriomorphia;Pectinida;Pectinoidea;Pectinidae;Mizuhopecten;Mizuhopecten yessoensis
UdeM00000012057 6596 323.6 UdeM00000012057 XP_045177781.1 36.2 541 283 7 1 498 97 618 6.60E-85 323.6 6596 cellular organisms;Eukaryota;Opisthokonta;Metazoa;Eumetazoa;Bilateria;Protostomia;Spiralia;Lophotrochozoa;Mollusca;Bivalvia;Autobranchia;Heteroconchia;Euheterodonta;Imparidentia;Venerida;Veneroidea;Veneridae;Mercenaria;Mercenaria mercenaria
UdeM00000012058 6596 374.4 UdeM00000012058 XP_045182203.1 38.1 598 334 10 114 691 187 768 3.10E-100 374.4 6596 cellular organisms;Eukaryota;Opisthokonta;Metazoa;Eumetazoa;Bilateria;Protostomia;Spiralia;Lophotrochozoa;Mollusca;Bivalvia;Autobranchia;Heteroconchia;Euhe

terodonta;Imparidentia;Venerida;Veneroidea;Veneridae;Mercenaria;Mercenaria mercenaria
UdeM00000012059 6596 367.5 UdeM00000012059 XP_045177776.1 40.5 524 293 4 1 520 405 913 2.90E-98 367.5 6596 cellular organisms;Eukaryota;Opisthokonta;Metazoa;Eumetazoa;Bilateria;Protostomia;Spiralia;Lophotrochozoa;Mollusca;Bivalvia;Autobranchia;Heteroconchia;Euheterodonta;Imparidentia;Venerida;Veneroidea;Veneridae;Mercenaria;Mercenaria mercenaria
UdeM00000012060 6565 111.3 UdeM00000012060 XP_022308598.1 34.1 220 136 5 31 246 15 229 1.80E-21 111.3 6565 cellular organisms;Eukaryota;Opisthokonta;Metazoa;Eumetazoa;Bilateria;Protostomia;Spiralia;Lophotrochozoa;Mollusca;Bivalvia;Autobranchia;Pteriomorphia;Ostreida;Ostreoidea;Ostreidae;Crassostrea;Crassostrea virginica
UdeM00000012061 6573 380.9 UdeM00000012061 XP_021371458.1 42.7 503 241 6 266 745 305 783 3.60E-102 380.9 6573 cellular organisms;Eukaryota;Opisthokonta;Metazoa;Eumetazoa;Bilateria;Protostomia;Spiralia;Lophotrochozoa;Mollusca;Bivalvia;Autobranchia;Pteriomorphia;Pectinida;Pectinoidea;Pectinidae;Mizuhopecten;Mizuhopecten yessoensis
UdeM00000012062 6596 429.9 UdeM00000012062 XP_045177776.1 41.8 577 295 11 360 929 393 935 1.10E-116 429.9 6596 cellular organisms;Eukaryota;Opisthokonta;Metazoa;Eumetazoa;Bilateria;Protostomia;Spiralia;Lophotrochozoa;Mollusca;Bivalvia;Autobranchia;Heteroconchia;Euheterodonta;Imparidentia;Venerida;Veneroidea;Veneridae;Mercenaria;Mercenaria mercenaria
UdeM00000012063 6596 435.6 UdeM00000012063 XP_045177776.1 41.9 575 295 10 360 927 393 935 1.90E-118 435.6 6596 cellular organisms;Eukaryota;Opisthokonta;Metazoa;Eumetazoa;Bilateria;Protostomia;Spiralia;Lophotrochozoa;Mollusca;Bivalvia;Autobranchia;Heteroconchia;Euheterodonta;Imparidentia;Venerida;Veneroidea;Veneridae;Mercenaria;Mercenaria mercenaria
UdeM00000012064 29159 115.2 UdeM00000012064 XP_011430296.2 38.4 164 81 1 7 150 8 171 7.50E-23 115.2 29159 cellular organisms;Eukaryota;Opisthokonta;Metazoa;Eumetazoa;Bilateria;Protostomia;Spiralia;Lophotrochozoa;Mollusca;Bivalvia;Autobranchia;Pteriomorphia;Ostreida;Ostreoidea;Ostreidae;Crassostrea;Crassostrea gigas
UdeM00000012065 1735272 140.6 UdeM00000012065 XP_041362167.1 40.5 173 99 2 7 175 9 181 1.90E-30 140.6 1735272 cellular organisms;Eukaryota;Opisthokonta;Metazoa;Eumetazoa;Bilateria;Protostomia;Spiralia;Lophotrochozoa;Mollusca;Gastropoda;Neomphalina;Neomphaloidea;Peltospiridae;Gigantopelta;Gigantopelta aegis
UdeM00000012066 6500 87 UdeM00000012066 XP_012946233.1 58.5 65 26 1 4 68 3 66 1.20E-14 87 6500 cellular organisms;Eukaryota;Opisthokonta;Metazoa;Eumetazoa;Bilateria;Protostomia;Spiralia;Lophotrochozoa;Mollusca;Gastropoda;Heterobranchia;Euthyneura;Tectipleura;Aplysiida;Aplysioidea;Aplysiidae;Aplysia;Aplysia californica
UdeM00000012073 1735272 288.1 UdeM00000012073 XP_041352907.1 55.6 248 106 2 45 289 90 336 1.30E-74 288.1 1735272 cellular organisms;Eukaryota;Opisthokonta;Metazoa;Eumetazoa;Bilateria;Protostomia;Spiralia;Lophotrochozoa;Mollusca;Gastropoda;Neomphalina;Neomphaloidea;Peltospiridae;Gigantopelta;Gigantopelta aegis
UdeM00000012074 7757 324.3 UdeM00000012074 XP_032815300.1 59.9 257 100 3 115 369 677 932 2.00E-85 324.3 7757 cellular

organisms;Eukaryota;Opisthokonta;Metazoa;Eumetazoa;Bilateria;Deuterostomia;Chordata;Craniata;Vertebrata;Cyclostomata;Hyperoartia;Petromyzontiformes;Petromyzontidae;Petromyzon;Petromyzon marinus
UdeM00000012075 6596 275.4 UdeM00000012075 XP_045180737.1 35.1 864 310 17 182 808 593 1442 2.30E-70 275.4 6596 cellular organisms;Eukaryota;Opisthokonta;Metazoa;Eumetazoa;Bilateria;Protostomia;Spiralia;Lophotrochozoa;Mollusca;Bivalvia;Autobranchia;Heteroconchia;Euheterodonta;Imparidentia;Venerida;Veneroidea;Veneridae;Mercenaria;Mercenaria mercenaria
UdeM00000012076 6596 110.5 UdeM00000012076 XP_045180737.1 49.2 130 59 2 216 344 584 707 6.10E-21 110.5 6596 cellular organisms;Eukaryota;Opisthokonta;Metazoa;Eumetazoa;Bilateria;Protostomia;Spiralia;Lophotrochozoa;Mollusca;Bivalvia;Autobranchia;Heteroconchia;Euheterodonta;Imparidentia;Venerida;Veneroidea;Veneridae;Mercenaria;Mercenaria mercenaria
UdeM00000012077 6596 118.6 UdeM00000012077 XP_045180737.1 58.7 109 43 1 13 119 14 122 7.30E-24 118.6 6596 cellular organisms;Eukaryota;Opisthokonta;Metazoa;Eumetazoa;Bilateria;Protostomia;Spiralia;Lophotrochozoa;Mollusca;Bivalvia;Autobranchia;Heteroconchia;Euheterodonta;Imparidentia;Venerida;Veneroidea;Veneridae;Mercenaria;Mercenaria mercenaria
UdeM00000012079 1735272 502.7 UdeM00000012079 XP_041361294.1 66 341 116 0 4 344 125 465 3.80E-139 502.7 1735272 cellular organisms;Eukaryota;Opisthokonta;Metazoa;Eumetazoa;Bilateria;Protostomia;Spiralia;Lophotrochozoa;Mollusca;Gastropoda;Neomphalina;Neomphaloidea;Peltospiridae;Gigantopelta;Gigantopelta aegis
UdeM00000012078 1735272 655.6 UdeM00000012078 XP_041361294.1 64.1 465 167 0 1 465 1 465 4.70E-185 655.6 1735272 cellular organisms;Eukaryota;Opisthokonta;Metazoa;Eumetazoa;Bilateria;Protostomia;Spiralia;Lophotrochozoa;Mollusca;Gastropoda;Neomphalina;Neomphaloidea;Peltospiridae;Gigantopelta;Gigantopelta aegis
UdeM00000012080 1735272 161 UdeM00000012080 XP_041361294.1 60.8 120 47 0 1 120 1 120 9.50E-37 161 1735272 cellular organisms;Eukaryota;Opisthokonta;Metazoa;Eumetazoa;Bilateria;Protostomia;Spiralia;Lophotrochozoa;Mollusca;Gastropoda;Neomphalina;Neomphaloidea;Peltospiridae;Gigantopelta;Gigantopelta aegis
UdeM00000012081 6596 2433.3 UdeM00000012081 XP_045193558.1 34.4 4508 2704 94 1 4405 41 4399 0.00E+00 2433.3 6596 cellular organisms;Eukaryota;Opisthokonta;Metazoa;Eumetazoa;Bilateria;Protostomia;Spiralia;Lophotrochozoa;Mollusca;Bivalvia;Autobranchia;Heteroconchia;Euheterodonta;Imparidentia;Venerida;Veneroidea;Veneridae;Mercenaria;Mercenaria mercenaria
UdeM00000012082 6573 94.4 UdeM00000012082 XP_021365625.1 52.9 87 41 0 8 94 40 126 1.90E-16 94.4 6573 cellular organisms;Eukaryota;Opisthokonta;Metazoa;Eumetazoa;Bilateria;Protostomia;Spiralia;Lophotrochozoa;Mollusca;Bivalvia;Autobranchia;Pteriomorphia;Pectinida;Pectinoidea;Pectinidae;Mizuhopecten;Mizuhopecten yessoensis
UdeM00000012083 29159 108.6 UdeM00000012083 XP_034313077.1 53.5 99 45 1 17 114 126 224 5.30E-21 108.6 29159 cellular organisms;Eukaryota;Opisthokonta;Metazoa;Eumetazoa;Bilateria;Protostomia;

Spiralia;Lophotrochozoa;Mollusca;Bivalvia;Autobranchia;Pteriomorphia;Ostreida;Ostreoidea;Ostreidae;Crassostrea;Crassostrea gigas
UdeM00000012084 6596 409.8 UdeM00000012084 XP_045182480.1 58.5 371 144 5 43 407 44 410 3.90E-111 409.8 6596 cellular organisms;Eukaryota;Opisthokonta;Metazoa;Eumetazoa;Bilateria;Protostomia;Spiralia;Lophotrochozoa;Mollusca;Bivalvia;Autobranchia;Heteroconchia;Euheterodonta;Imparidentia;Venerida;Veneroidea;Veneridae;Mercenaria;Mercenaria mercenaria
UdeM00000012085 1437191 80.9 UdeM00000012085 XP_029054816.1 42.3 78 44 1 2 78 386 463 8.10E-13 80.9 1437191 cellular organisms;Eukaryota;Opisthokonta;Metazoa;Eumetazoa;Bilateria;Protostomia;Ecdysozoa;Panarthropoda;Arthropoda;Mandibulata;Pancrustacea;Hexapoda;Insecta;Dicondylia;Pterygota;Neoptera;Endopterygota;Hymenoptera;Apocrita;Aculeata;Apoidea;Megachilidae;Megachilinae;Osmiini;Osmia;Osmia bicornis;Osmia bicornis bicornis
UdeM00000012086 45264 251.1 UdeM00000012086 XP_044177087.1 49.8 249 122 2 5 253 216 461 1.50E-63 251.1 45264 cellular organisms;Eukaryota;Opisthokonta;Metazoa;Eumetazoa;Cnidaria;Anthozoa;Hexacorallia;Scleractinia;Astrocoeniina;Acroporidae;Acropora;Acropora millepora
UdeM00000012087 400727 389.4 UdeM00000012087 XP_025091401.1 36.2 702 369 15 11 688 27 673 9.60E-105 389.4 400727 cellular organisms;Eukaryota;Opisthokonta;Metazoa;Eumetazoa;Bilateria;Protostomia;Spiralia;Lophotrochozoa;Mollusca;Gastropoda;Caenogastropoda;Architaenioglossa;Ampullarioidea;Ampullariidae;Pomacea;Pomacea canaliculata
UdeM00000012088 6596 342 UdeM00000012088 XP_045209667.1 45.4 326 177 1 85 410 191 515 1.00E-90 342 6596 cellular organisms;Eukaryota;Opisthokonta;Metazoa;Eumetazoa;Bilateria;Protostomia;Spiralia;Lophotrochozoa;Mollusca;Bivalvia;Autobranchia;Heteroconchia;Euheterodonta;Imparidentia;Venerida;Veneroidea;Veneridae;Mercenaria;Mercenaria mercenaria
UdeM00000012089 6596 342 UdeM00000012089 XP_045209667.1 45.4 326 177 1 123 448 191 515 1.10E-90 342 6596 cellular organisms;Eukaryota;Opisthokonta;Metazoa;Eumetazoa;Bilateria;Protostomia;Spiralia;Lophotrochozoa;Mollusca;Bivalvia;Autobranchia;Heteroconchia;Euheterodonta;Imparidentia;Venerida;Veneroidea;Veneridae;Mercenaria;Mercenaria mercenaria
UdeM00000012090 6596 343.6 UdeM00000012090 XP_045209667.1 45.1 324 177 1 93 416 191 513 3.60E-91 343.6 6596 cellular organisms;Eukaryota;Opisthokonta;Metazoa;Eumetazoa;Bilateria;Protostomia;Spiralia;Lophotrochozoa;Mollusca;Bivalvia;Autobranchia;Heteroconchia;Euheterodonta;Imparidentia;Venerida;Veneroidea;Veneridae;Mercenaria;Mercenaria mercenaria
UdeM00000012091 6565 158.3 UdeM00000012091 XP_022328252.1 44.1 211 100 5 1 193 1105 1315 9.80E-36 158.3 6565 cellular organisms;Eukaryota;Opisthokonta;Metazoa;Eumetazoa;Bilateria;Protostomia;Spiralia;Lophotrochozoa;Mollusca;Bivalvia;Autobranchia;Pteriomorphia;Ostreida;Ostreoidea;Ostreidae;Crassostrea;Crassostrea virginica
UdeM00000012092 6565 864 UdeM00000012092 XP_022308715.1 76.9 516 118 1 8 523 6 520 9.80E-248 864 6565 cellular organisms;Eukaryota;Opisthokonta;Metazoa;Eumetazoa;Bilateria;Protostomia;

Spiralia;Lophotrochozoa;Mollusca;Bivalvia;Autobranchia;Pteriomorphia;Ostreida;Ostreoidea;Ostreidae;Crassostrea;Crassostrea virginica
UdeM00000012093 6565 866.3 UdeM00000012093 XP_022308715.1 77.3 516 116 1 8 523 6 520 2.00E-248 866.3 6565 cellular organisms;Eukaryota;Opisthokonta;Metazoa;Eumetazoa;Bilateria;Protostomia;Spiralia;Lophotrochozoa;Mollusca;Bivalvia;Autobranchia;Pteriomorphia;Ostreida;Ostreoidea;Ostreidae;Crassostrea;Crassostrea virginica
UdeM00000012094 6596 206.1 UdeM00000012094 XP_045180504.1 52.5 198 90 3 10 203 17 214 4.40E-50 206.1 6596 cellular organisms;Eukaryota;Opisthokonta;Metazoa;Eumetazoa;Bilateria;Protostomia;Spiralia;Lophotrochozoa;Mollusca;Bivalvia;Autobranchia;Heteroconchia;Euheterodonta;Imparidentia;Venerida;Veneroidea;Veneridae;Mercenaria;Mercenaria mercenaria
UdeM00000012096 6596 102.1 UdeM00000012096 XP_045180504.1 51.2 84 41 0 24 107 214 297 6.50E-19 102.1 6596 cellular organisms;Eukaryota;Opisthokonta;Metazoa;Eumetazoa;Bilateria;Protostomia;Spiralia;Lophotrochozoa;Mollusca;Bivalvia;Autobranchia;Heteroconchia;Euheterodonta;Imparidentia;Venerida;Veneroidea;Veneridae;Mercenaria;Mercenaria mercenaria
UdeM00000012097 6596 122.9 UdeM00000012097 XP_045181496.1 43.9 155 84 1 95 246 345 499 5.90E-25 122.9 6596 cellular organisms;Eukaryota;Opisthokonta;Metazoa;Eumetazoa;Bilateria;Protostomia;Spiralia;Lophotrochozoa;Mollusca;Bivalvia;Autobranchia;Heteroconchia;Euheterodonta;Imparidentia;Venerida;Veneroidea;Veneridae;Mercenaria;Mercenaria mercenaria
UdeM00000012098 6596 237.3 UdeM00000012098 XP_045181496.1 43.4 286 158 2 95 376 345 630 3.20E-59 237.3 6596 cellular organisms;Eukaryota;Opisthokonta;Metazoa;Eumetazoa;Bilateria;Protostomia;Spiralia;Lophotrochozoa;Mollusca;Bivalvia;Autobranchia;Heteroconchia;Euheterodonta;Imparidentia;Venerida;Veneroidea;Veneridae;Mercenaria;Mercenaria mercenaria
UdeM00000012099 6596 644 UdeM00000012099 XP_045181496.1 47.4 645 334 3 17 656 83 727 2.10E-181 644 6596 cellular organisms;Eukaryota;Opisthokonta;Metazoa;Eumetazoa;Bilateria;Protostomia;Spiralia;Lophotrochozoa;Mollusca;Bivalvia;Autobranchia;Heteroconchia;Euheterodonta;Imparidentia;Venerida;Veneroidea;Veneridae;Mercenaria;Mercenaria mercenaria
UdeM00000012100 6596 851.7 UdeM00000012100 XP_045181496.1 60.1 704 260 6 15 702 17 715 7.00E-244 851.7 6596 cellular organisms;Eukaryota;Opisthokonta;Metazoa;Eumetazoa;Bilateria;Protostomia;Spiralia;Lophotrochozoa;Mollusca;Bivalvia;Autobranchia;Heteroconchia;Euheterodonta;Imparidentia;Venerida;Veneroidea;Veneridae;Mercenaria;Mercenaria mercenaria
UdeM00000012101 6573 212.2 UdeM00000012101 XP_021354348.1 50.9 289 129 3 1 289 1 276 1.30E-51 212.2 6573 cellular organisms;Eukaryota;Opisthokonta;Metazoa;Eumetazoa;Bilateria;Protostomia;Spiralia;Lophotrochozoa;Mollusca;Bivalvia;Autobranchia;Pteriomorphia;Pectinida;Pectinoidea;Pectinidae;Mizuhopecten;Mizuhopecten yessoensis
UdeM00000012102 6565 190.7 UdeM00000012102 XP_022336632.1 24.2 652 442 15 1 634 63 680 5.90E-45 190.7 6565 cellular organisms;Eukaryota;Opisthokonta;Metazoa;Eumetazoa;Bilateria;Protostomia;Spiralia;Lophotrochozoa;Mollusca;Bivalvia;Autobranchia;Pteriomorphia;Ostreida;Ostreoidea;Ostreidae;Crassostrea;Crassostrea virginica

UdeM00000012103 8078 126.3 UdeM00000012103 XP_035996634.1 57 93
40 0 1 93 214 306 2.40E-26 126.3 8078 cellular
organisms;Eukaryota;Opisthokonta;Metazoa;Eumetazoa;Bilateria;Deuterostomi
a;Chordata;Craniata;Vertebrata;Gnathostomata;Teleostomi;Euteleostomi;Acti
nopterygii;Actinopteri;Neopterygii;Teleostei;Osteoglossocephalai;Clupeoce
phala;Euteleosteomorpha;Neoteleostei;Eurypterygia;Ctenosquamata;Acanthomo
rphata;Euacanthomorphacea;Percomorphaceae;Ovalentaria;Atherinomorphae;Cyp
rinodontiformes;Cyprinodontoidei;Fundulidae;Fundulus;Fundulus
heteroclitus
UdeM00000012104 63155 147.9 UdeM00000012104 XP_030580630.1 53.5 129
60 0 14 142 8 136 9.80E-33 147.9 63155 cellular
organisms;Eukaryota;Opisthokonta;Metazoa;Eumetazoa;Bilateria;Deuterostomi
a;Chordata;Craniata;Vertebrata;Gnathostomata;Teleostomi;Euteleostomi;Acti
nopterygii;Actinopteri;Neopterygii;Teleostei;Osteoglossocephalai;Clupeoce
phala;Euteleosteomorpha;Neoteleostei;Eurypterygia;Ctenosquamata;Acanthomo
rphata;Euacanthomorphacea;Percomorphaceae;Ovalentaria;Cichlomorphae;Cichl
iformes;Cichlidae;New World
cichlids;Cichlasomatinae;Heroini;Archocentrus;Archocentrus centrarchus
UdeM00000012105 6596 661.8 UdeM00000012105 XP_045180584.1 52.3 583
271 4 51 633 95 670 9.00E-187 661.8 6596 cellular
organisms;Eukaryota;Opisthokonta;Metazoa;Eumetazoa;Bilateria;Protostomia;
Spiralia;Lophotrochozoa;Mollusca;Bivalvia;Autobranchia;Heteroconchia;Euhe
terodonta;Imparidentia;Venerida;Veneroidea;Veneridae;Mercenaria;Mercenari
a mercenaria
UdeM00000012106 29159 60.1 UdeM00000012106 XP_019918370.2 48.3 58
28 1 116 171 37 94 3.40E-06 60.1 29159 cellular
organisms;Eukaryota;Opisthokonta;Metazoa;Eumetazoa;Bilateria;Protostomia;
Spiralia;Lophotrochozoa;Mollusca;Bivalvia;Autobranchia;Pteriomorphia;Ostr
eida;Ostreoidea;Ostreidae;Crassostrea;Crassostrea gigas
UdeM00000012107 7739 449.5 UdeM00000012107 XP_035696678.1 57.5 374
156 1 13 383 530 903 4.60E-123 449.5 7739 cellular
organisms;Eukaryota;Opisthokonta;Metazoa;Eumetazoa;Bilateria;Deuterostomi
a;Chordata;Cephalochordata;Leptocardii;Amphioxiformes;Branchiostomidae;Br
anchiostoma;Branchiostoma floridae
UdeM00000012108 6596 142.1 UdeM00000012108 XP_045182223.1 26.2 362
250 10 36 384 30 387 1.50E-30 142.1 6596 cellular
organisms;Eukaryota;Opisthokonta;Metazoa;Eumetazoa;Bilateria;Protostomia;
Spiralia;Lophotrochozoa;Mollusca;Bivalvia;Autobranchia;Heteroconchia;Euhe
terodonta;Imparidentia;Venerida;Veneroidea;Veneridae;Mercenaria;Mercenari
a mercenaria
UdeM00000012109 6596 82 UdeM00000012109 XP_045182223.1 30.6 157
102 4 36 186 30 185 1.50E-12 82 6596 cellular
organisms;Eukaryota;Opisthokonta;Metazoa;Eumetazoa;Bilateria;Protostomia;
Spiralia;Lophotrochozoa;Mollusca;Bivalvia;Autobranchia;Heteroconchia;Euhe
terodonta;Imparidentia;Venerida;Veneroidea;Veneridae;Mercenaria;Mercenari
a mercenaria
UdeM00000012111 6579 123.6 UdeM00000012111 XP_033727138.1 46.3 121
63 1 476 594 563 683 9.40E-25 123.6 6579 cellular
organisms;Eukaryota;Opisthokonta;Metazoa;Eumetazoa;Bilateria;Protostomia;
Spiralia;Lophotrochozoa;Mollusca;Bivalvia;Autobranchia;Pteriomorphia;Pect
inida;Pectinoidea;Pectinidae;Pecten;Pecten maximus
UdeM00000012112 6596 209.1 UdeM00000012112 XP_045181411.1 22.6 780
424 12 27 798 677 1284 2.00E-50 209.1 6596 cellular
organisms;Eukaryota;Opisthokonta;Metazoa;Eumetazoa;Bilateria;Protostomia;

Spiralia;Lophotrochozoa;Mollusca;Bivalvia;Autobranchia;Heteroconchia;Euheterodonta;Imparidentia;Venerida;Veneroidea;Veneridae;Mercenaria;Mercenaria mercenaria
UdeM00000012113 6579 534.6 UdeM00000012113 XP_033726652.1 47.2 629 300 10 11 620 15 630 1.60E-148 534.6 6579 cellular organisms;Eukaryota;Opisthokonta;Metazoa;Eumetazoa;Bilateria;Protostomia;Spiralia;Lophotrochozoa;Mollusca;Bivalvia;Autobranchia;Pteriomorphia;Pectinida;Pectinoidea;Pectinidae;Pecten;Pecten maximus
UdeM00000012118 6596 368.2 UdeM00000012118 XP_045181639.1 79.2 212 44 0 1 212 1 212 6.90E-99 368.2 6596 cellular organisms;Eukaryota;Opisthokonta;Metazoa;Eumetazoa;Bilateria;Protostomia;Spiralia;Lophotrochozoa;Mollusca;Bivalvia;Autobranchia;Heteroconchia;Euheterodonta;Imparidentia;Venerida;Veneroidea;Veneridae;Mercenaria;Mercenaria mercenaria
UdeM00000012119 6596 403.3 UdeM00000012119 XP_045181259.1 45.6 579 217 18 6 547 5 522 5.00E-109 403.3 6596 cellular organisms;Eukaryota;Opisthokonta;Metazoa;Eumetazoa;Bilateria;Protostomia;Spiralia;Lophotrochozoa;Mollusca;Bivalvia;Autobranchia;Heteroconchia;Euheterodonta;Imparidentia;Venerida;Veneroidea;Veneridae;Mercenaria;Mercenaria mercenaria
UdeM00000012120 6573 235.3 UdeM00000012120 XP_021355947.1 52.4 267 104 7 4 258 3 258 9.00E-59 235.3 6573 cellular organisms;Eukaryota;Opisthokonta;Metazoa;Eumetazoa;Bilateria;Protostomia;Spiralia;Lophotrochozoa;Mollusca;Bivalvia;Autobranchia;Pteriomorphia;Pectinida;Pectinoidea;Pectinidae;Mizuhopecten;Mizuhopecten yessoensis
UdeM00000012122 441366 132.9 UdeM00000012122 XP_028326620.1 40.6 197 107 5 1 194 48 237 4.50E-28 132.9 441366 cellular organisms;Eukaryota;Opisthokonta;Metazoa;Eumetazoa;Bilateria;Deuterostomia;Chordata;Craniata;Vertebrata;Gnathostomata;Teleostomi;Euteleostomi;Actinopterygii;Actinopteri;Neopterygii;Teleostei;Osteoglossocephalai;Clupeocephala;Euteleosteomorpha;Neoteleostei;Eurypterygia;Ctenosquamata;Acanthomorphata;Euacanthomorphacea;Percomorphaceae;Ovalentaria;Blenniimorphae;Blenniiformes;Gobiesocoidei;Gobiesocidae;Gobiesocinae;Gouania;Gouania willdenowi
UdeM00000012121 441366 170.2 UdeM00000012121 XP_028296753.1 38.4 284 158 9 6 285 32 302 3.70E-39 170.2 441366 cellular organisms;Eukaryota;Opisthokonta;Metazoa;Eumetazoa;Bilateria;Deuterostomia;Chordata;Craniata;Vertebrata;Gnathostomata;Teleostomi;Euteleostomi;Actinopterygii;Actinopteri;Neopterygii;Teleostei;Osteoglossocephalai;Clupeocephala;Euteleosteomorpha;Neoteleostei;Eurypterygia;Ctenosquamata;Acanthomorphata;Euacanthomorphacea;Percomorphaceae;Ovalentaria;Blenniimorphae;Blenniiformes;Gobiesocoidei;Gobiesocidae;Gobiesocinae;Gouania;Gouania willdenowi
UdeM00000012123 6596 742.3 UdeM00000012123 XP_045182167.1 61 649 230 11 18 656 309 944 5.40E-211 742.3 6596 cellular organisms;Eukaryota;Opisthokonta;Metazoa;Eumetazoa;Bilateria;Protostomia;Spiralia;Lophotrochozoa;Mollusca;Bivalvia;Autobranchia;Heteroconchia;Euheterodonta;Imparidentia;Venerida;Veneroidea;Veneridae;Mercenaria;Mercenaria mercenaria
UdeM00000012124 29159 423.7 UdeM00000012124 XP_034316358.1 42.9 508 274 10 1 504 5 500 3.30E-115 423.7 29159 cellular organisms;Eukaryota;Opisthokonta;Metazoa;Eumetazoa;Bilateria;Protostomia;

Spiralia;Lophotrochozoa;Mollusca;Bivalvia;Autobranchia;Pteriomorphia;Ostreida;Ostreoidea;Ostreidae;Crassostrea;Crassostrea gigas
UdeM00000012125 29159 423.7 UdeM00000012125 XP_034316358.1 42.8 509
275 10 62 566 4 500 3.70E-115 423.7 29159 cellular organisms;Eukaryota;Opisthokonta;Metazoa;Eumetazoa;Bilateria;Protostomia;Spiralia;Lophotrochozoa;Mollusca;Bivalvia;Autobranchia;Pteriomorphia;Ostreida;Ostreoidea;Ostreidae;Crassostrea;Crassostrea gigas
UdeM00000012126 6565 202.6 UdeM00000012126 XP_022344978.1 34 430
215 11 1 398 1 393 9.40E-49 202.6 6565 cellular organisms;Eukaryota;Opisthokonta;Metazoa;Eumetazoa;Bilateria;Protostomia;Spiralia;Lophotrochozoa;Mollusca;Bivalvia;Autobranchia;Pteriomorphia;Ostreida;Ostreoidea;Ostreidae;Crassostrea;Crassostrea virginica
UdeM00000012131 6596 227.3 UdeM00000012131 XP_045181493.1 50.8 266
113 4 10 274 465 713 2.60E-56 227.3 6596 cellular organisms;Eukaryota;Opisthokonta;Metazoa;Eumetazoa;Bilateria;Protostomia;Spiralia;Lophotrochozoa;Mollusca;Bivalvia;Autobranchia;Heteroconchia;Euheterodonta;Imparidentia;Venerida;Veneroidea;Veneridae;Mercenaria;Mercenaria mercenaria
UdeM00000012134 1735272 124.4 UdeM00000012134 XP_041360768.1 51.7
116 56 0 9 124 6 121 1.10E-25 124.4 1735272
cellular organisms;Eukaryota;Opisthokonta;Metazoa;Eumetazoa;Bilateria;Protostomia;Spiralia;Lophotrochozoa;Mollusca;Gastropoda;Neomphalina;Neomphaloidea;Peltospiridae;Gigantopelta;Gigantopelta aegis
UdeM00000012136 29159 622.1 UdeM00000012136 XP_034323675.1 72.3 415
98 4 1 399 1 414 6.50E-175 622.1 29159 cellular organisms;Eukaryota;Opisthokonta;Metazoa;Eumetazoa;Bilateria;Protostomia;Spiralia;Lophotrochozoa;Mollusca;Bivalvia;Autobranchia;Pteriomorphia;Ostreida;Ostreoidea;Ostreidae;Crassostrea;Crassostrea gigas
UdeM00000012137 29159 600.9 UdeM00000012137 XP_034323675.1 65.5 458
98 5 1 442 1 414 1.70E-168 600.9 29159 cellular organisms;Eukaryota;Opisthokonta;Metazoa;Eumetazoa;Bilateria;Protostomia;Spiralia;Lophotrochozoa;Mollusca;Bivalvia;Autobranchia;Pteriomorphia;Ostreida;Ostreoidea;Ostreidae;Crassostrea;Crassostrea gigas
UdeM00000012138 6596 164.5 UdeM00000012138 XP_045171211.1 52 125
60 0 16 140 19 143 1.00E-37 164.5 6596 cellular organisms;Eukaryota;Opisthokonta;Metazoa;Eumetazoa;Bilateria;Protostomia;Spiralia;Lophotrochozoa;Mollusca;Bivalvia;Autobranchia;Heteroconchia;Euheterodonta;Imparidentia;Venerida;Veneroidea;Veneridae;Mercenaria;Mercenaria mercenaria
UdeM00000012139 6596 120.2 UdeM00000012139 XP_045180931.1 49.2 132
66 1 1 131 1 132 2.50E-24 120.2 6596 cellular organisms;Eukaryota;Opisthokonta;Metazoa;Eumetazoa;Bilateria;Protostomia;Spiralia;Lophotrochozoa;Mollusca;Bivalvia;Autobranchia;Heteroconchia;Euheterodonta;Imparidentia;Venerida;Veneroidea;Veneridae;Mercenaria;Mercenaria mercenaria
UdeM00000012140 6596 120.2 UdeM00000012140 XP_045180931.1 49.2 132
66 1 1 131 1 132 3.40E-24 120.2 6596 cellular organisms;Eukaryota;Opisthokonta;Metazoa;Eumetazoa;Bilateria;Protostomia;Spiralia;Lophotrochozoa;Mollusca;Bivalvia;Autobranchia;Heteroconchia;Euheterodonta;Imparidentia;Venerida;Veneroidea;Veneridae;Mercenaria;Mercenaria mercenaria
UdeM00000018533 6596 291.6 UdeM00000018533 XP_045201488.1 31.6 589
364 10 82 640 67 646 2.50E-75 291.6 6596 cellular

organisms;Eukaryota;Opisthokonta;Metazoa;Eumetazoa;Bilateria;Protostomia;Spiralia;Lophotrochozoa;Mollusca;Bivalvia;Autobranchia;Heteroconchia;Euheterodonta;Imparidentia;Venerida;Veneroidea;Veneridae;Mercenaria;Mercenaria mercenaria
UdeM00000018534 6596 386.3 UdeM00000018534 XP_045177776.1 41.4 543 287 11 13 539 396 923 9.20E-104 386.3 6596 cellular organisms;Eukaryota;Opisthokonta;Metazoa;Eumetazoa;Bilateria;Protostomia;Spiralia;Lophotrochozoa;Mollusca;Bivalvia;Autobranchia;Heteroconchia;Euheterodonta;Imparidentia;Venerida;Veneroidea;Veneridae;Mercenaria;Mercenaria mercenaria
UdeM00000018536 133434 98.2 UdeM00000018536 XP_022091439.1 37.3 161 83 3 3 146 524 683 9.40E-18 98.2 133434 cellular organisms;Eukaryota;Opisthokonta;Metazoa;Eumetazoa;Bilateria;Deuterostomia;Echinodermata;Eleutherozoa;Asterozoa;Asteroidea;Valvatacea;Valvatida;Acanthasteridae;Acanthaster;Acanthaster planci
UdeM00000018537 45351 83.6 UdeM00000018537 XP_001623471.2 31.4 188 102 4 10 181 12 188 3.40E-13 83.6 45351 cellular organisms;Eukaryota;Opisthokonta;Metazoa;Eumetazoa;Cnidaria;Anthozoa;Hexacorallia;Actiniaria;Edwardsiidae;Nematostella;Nematostella vectensis
UdeM00000018538 6596 376.3 UdeM00000018538 XP_045177776.1 41.2 532 288 10 1 519 405 924 7.00E-101 376.3 6596 cellular organisms;Eukaryota;Opisthokonta;Metazoa;Eumetazoa;Bilateria;Protostomia;Spiralia;Lophotrochozoa;Mollusca;Bivalvia;Autobranchia;Heteroconchia;Euheterodonta;Imparidentia;Venerida;Veneroidea;Veneridae;Mercenaria;Mercenaria mercenaria
UdeM00000018539 6596 250.8 UdeM00000018539 XP_045177776.1 42.4 335 183 6 62 394 549 875 3.00E-63 250.8 6596 cellular organisms;Eukaryota;Opisthokonta;Metazoa;Eumetazoa;Bilateria;Protostomia;Spiralia;Lophotrochozoa;Mollusca;Bivalvia;Autobranchia;Heteroconchia;Euheterodonta;Imparidentia;Venerida;Veneroidea;Veneridae;Mercenaria;Mercenaria mercenaria
UdeM00000018541 6596 728.4 UdeM00000018541 XP_045179300.1 65.9 555 119 5 22 575 22 507 7.20E-207 728.4 6596 cellular organisms;Eukaryota;Opisthokonta;Metazoa;Eumetazoa;Bilateria;Protostomia;Spiralia;Lophotrochozoa;Mollusca;Bivalvia;Autobranchia;Heteroconchia;Euheterodonta;Imparidentia;Venerida;Veneroidea;Veneridae;Mercenaria;Mercenaria mercenaria
UdeM00000018542 59861 176.4 UdeM00000018542 XP_041731387.1 45.6 193 101 2 1 189 13 205 3.40E-41 176.4 59861 cellular organisms;Eukaryota;Opisthokonta;Metazoa;Eumetazoa;Bilateria;Deuterostomia;Chordata;Craniata;Vertebrata;Gnathostomata;Teleostomi;Euteleostomi;Actinopterygii;Actinopteri;Neopterygii;Teleostei;Osteoglossocephalai;Clupeocephala;Euteleosteomorpha;Protacanthopterygii;Salmoniformes;Salmonidae;Coreginae;Coregonus;Coregonus clupeaformis
UdeM00000018543 7741 290 UdeM00000018543 XP_019622111.1 52.7 281 128 4 5 283 1 278 3.20E-75 290 7741 cellular organisms;Eukaryota;Opisthokonta;Metazoa;Eumetazoa;Bilateria;Deuterostomia;Chordata;Cephalochordata;Leptocardii;Amphioxiformes;Branchiostomidae;Branchiostoma;Branchiostoma belcheri
UdeM00000018544 6565 142.9 UdeM00000018544 XP_022286553.1 31.6 269 178 2 17 282 14 279 7.20E-31 142.9 6565 cellular organisms;Eukaryota;Opisthokonta;Metazoa;Eumetazoa;Bilateria;Protostomia;

Spiralia;Lophotrochozoa;Mollusca;Bivalvia;Autobranchia;Pteriomorphia;Ostreida;Ostreoidea;Ostreidae;Crassostrea;Crassostrea virginica
UdeM00000018545 6565 104.8 UdeM00000018545 XP_022336926.1 30.3 231 144 6 86 308 516 737 2.20E-19 104.8 6565 cellular organisms;Eukaryota;Opisthokonta;Metazoa;Eumetazoa;Bilateria;Protostomia;Spiralia;Lophotrochozoa;Mollusca;Bivalvia;Autobranchia;Pteriomorphia;Ostreida;Ostreoidea;Ostreidae;Crassostrea;Crassostrea virginica
UdeM00000018546 6596 387.9 UdeM00000018546 XP_045162172.1 25.8 1386 623 18 90 1433 36 1057 5.70E-104 387.9 6596 cellular organisms;Eukaryota;Opisthokonta;Metazoa;Eumetazoa;Bilateria;Protostomia;Spiralia;Lophotrochozoa;Mollusca;Bivalvia;Autobranchia;Heteroconchia;Euheterodonta;Imparidentia;Venerida;Veneroidea;Veneridae;Mercenaria;Mercenaria mercenaria
UdeM00000018547 6596 276.2 UdeM00000018547 XP_045177590.1 44.3 323 144 7 18 309 45 362 5.90E-71 276.2 6596 cellular organisms;Eukaryota;Opisthokonta;Metazoa;Eumetazoa;Bilateria;Protostomia;Spiralia;Lophotrochozoa;Mollusca;Bivalvia;Autobranchia;Heteroconchia;Euheterodonta;Imparidentia;Venerida;Veneroidea;Veneridae;Mercenaria;Mercenaria mercenaria
UdeM00000018548 6596 811.6 UdeM00000018548 XP_045177590.1 54.2 688 304 8 2 683 326 1008 7.80E-232 811.6 6596 cellular organisms;Eukaryota;Opisthokonta;Metazoa;Eumetazoa;Bilateria;Protostomia;Spiralia;Lophotrochozoa;Mollusca;Bivalvia;Autobranchia;Heteroconchia;Euheterodonta;Imparidentia;Venerida;Veneroidea;Veneridae;Mercenaria;Mercenaria mercenaria
UdeM00000018549 6596 511.5 UdeM00000018549 XP_045177590.1 61.6 476 133 13 5 465 1080 1520 1.10E-141 511.5 6596 cellular organisms;Eukaryota;Opisthokonta;Metazoa;Eumetazoa;Bilateria;Protostomia;Spiralia;Lophotrochozoa;Mollusca;Bivalvia;Autobranchia;Heteroconchia;Euheterodonta;Imparidentia;Venerida;Veneroidea;Veneridae;Mercenaria;Mercenaria mercenaria
UdeM00000018550 6596 452.6 UdeM00000018550 XP_045177655.1 50.5 442 213 3 1 436 1 442 5.80E-124 452.6 6596 cellular organisms;Eukaryota;Opisthokonta;Metazoa;Eumetazoa;Bilateria;Protostomia;Spiralia;Lophotrochozoa;Mollusca;Bivalvia;Autobranchia;Heteroconchia;Euheterodonta;Imparidentia;Venerida;Veneroidea;Veneridae;Mercenaria;Mercenaria mercenaria
UdeM00000018552 6596 347.1 UdeM00000018552 XP_045177776.1 38.8 536 280 10 1 505 405 923 4.40E-92 347.1 6596 cellular organisms;Eukaryota;Opisthokonta;Metazoa;Eumetazoa;Bilateria;Protostomia;Spiralia;Lophotrochozoa;Mollusca;Bivalvia;Autobranchia;Heteroconchia;Euheterodonta;Imparidentia;Venerida;Veneroidea;Veneridae;Mercenaria;Mercenaria mercenaria
UdeM00000018553 45351 87.4 UdeM00000018553 XP_001623471.2 31 200 109 5 10 192 12 199 2.20E-14 87.4 45351 cellular organisms;Eukaryota;Opisthokonta;Metazoa;Eumetazoa;Cnidaria;Anthozoa;Hexacorallia;Actiniaria;Edwardsiidae;Nematostella;Nematostella vectensis
UdeM00000018554 6596 278.1 UdeM00000018554 XP_045178146.1 37.6 415 207 4 22 384 18 432 2.00E-71 278.1 6596 cellular organisms;Eukaryota;Opisthokonta;Metazoa;Eumetazoa;Bilateria;Protostomia;Spiralia;Lophotrochozoa;Mollusca;Bivalvia;Autobranchia;Heteroconchia;Euheterodonta;Imparidentia;Venerida;Veneroidea;Veneridae;Mercenaria;Mercenaria mercenaria

UdeM00000018555 6596 81.6 UdeM00000018555 XP_045179680.1 34.3 181
100 7 223 391 201 374 2.40E-12 81.6 6596 cellular organisms;Eukaryota;Opisthokonta;Metazoa;Eumetazoa;Bilateria;Protostomia;Spiralia;Lophotrochozoa;Mollusca;Bivalvia;Autobranchia;Heteroconchia;Euheterodonta;Imparidentia;Venerida;Veneroidea;Veneridae;Mercenaria;Mercenaria mercenaria
UdeM00000018556 6573 110.9 UdeM00000018556 XP_021339818.1 40.3 144
82 3 191 331 40 182 4.00E-21 110.9 6573 cellular organisms;Eukaryota;Opisthokonta;Metazoa;Eumetazoa;Bilateria;Protostomia;Spiralia;Lophotrochozoa;Mollusca;Bivalvia;Autobranchia;Pteriomorphia;Pectinida;Pectinoidea;Pectinidae;Mizuhopecten;Mizuhopecten yessoensis
UdeM00000018557 6596 944.1 UdeM00000018557 XP_045177690.1 69.7 661
197 2 1 659 1 660 9.40E-272 944.1 6596 cellular organisms;Eukaryota;Opisthokonta;Metazoa;Eumetazoa;Bilateria;Protostomia;Spiralia;Lophotrochozoa;Mollusca;Bivalvia;Autobranchia;Heteroconchia;Euheterodonta;Imparidentia;Venerida;Veneroidea;Veneridae;Mercenaria;Mercenaria mercenaria
UdeM00000018558 6596 937.9 UdeM00000018558 XP_045177690.1 69.1 667
197 3 1 665 1 660 6.80E-270 937.9 6596 cellular organisms;Eukaryota;Opisthokonta;Metazoa;Eumetazoa;Bilateria;Protostomia;Spiralia;Lophotrochozoa;Mollusca;Bivalvia;Autobranchia;Heteroconchia;Euheterodonta;Imparidentia;Venerida;Veneroidea;Veneridae;Mercenaria;Mercenaria mercenaria
UdeM00000018559 6596 934.9 UdeM00000018559 XP_045177690.1 69.3 662
194 3 1 660 6 660 5.70E-269 934.9 6596 cellular organisms;Eukaryota;Opisthokonta;Metazoa;Eumetazoa;Bilateria;Protostomia;Spiralia;Lophotrochozoa;Mollusca;Bivalvia;Autobranchia;Heteroconchia;Euheterodonta;Imparidentia;Venerida;Veneroidea;Veneridae;Mercenaria;Mercenaria mercenaria
UdeM00000018560 6596 288.9 UdeM00000018560 XP_045179222.1 51.5 268
125 3 31 297 17 280 7.50E-75 288.9 6596 cellular organisms;Eukaryota;Opisthokonta;Metazoa;Eumetazoa;Bilateria;Protostomia;Spiralia;Lophotrochozoa;Mollusca;Bivalvia;Autobranchia;Heteroconchia;Euheterodonta;Imparidentia;Venerida;Veneroidea;Veneridae;Mercenaria;Mercenaria mercenaria
UdeM00000018561 29159 314.3 UdeM00000018561 XP_011432228.2 49.8 492
240 4 6 494 108 595 2.80E-82 314.3 29159 cellular organisms;Eukaryota;Opisthokonta;Metazoa;Eumetazoa;Bilateria;Protostomia;Spiralia;Lophotrochozoa;Mollusca;Bivalvia;Autobranchia;Pteriomorphia;Ostreida;Ostreoidea;Ostreidae;Crassostrea;Crassostrea gigas
UdeM00000018562 29159 322 UdeM00000018562 XP_011432228.2 50 496
244 3 46 541 104 595 1.40E-84 322 29159 cellular organisms;Eukaryota;Opisthokonta;Metazoa;Eumetazoa;Bilateria;Protostomia;Spiralia;Lophotrochozoa;Mollusca;Bivalvia;Autobranchia;Pteriomorphia;Ostreida;Ostreoidea;Ostreidae;Crassostrea;Crassostrea gigas
UdeM00000018563 6412 118.6 UdeM00000018563 XP_009014168.1 58.4 89
33 1 1 85 1 89 4.70E-24 118.6 6412 cellular organisms;Eukaryota;Opisthokonta;Metazoa;Eumetazoa;Bilateria;Protostomia;Spiralia;Lophotrochozoa;Annelida;Clitellata;Hirudinea;Rhynchobdellida;Glossiphoniidae;Helobdella;Helobdella robusta
UdeM00000018564 6596 667.2 UdeM00000018564 XP_045178501.1 74.1 409
101 2 1 409 1 404 1.40E-188 667.2 6596 cellular organisms;Eukaryota;Opisthokonta;Metazoa;Eumetazoa;Bilateria;Protostomia;Spiralia;Lophotrochozoa;Mollusca;Bivalvia;Autobranchia;Heteroconchia;Euhe

terodonta;Imparidentia;Venerida;Veneroidea;Veneridae;Mercenaria;Mercenaria mercenaria
UdeM00000018565 6596 276.2 UdeM00000018565 XP_045179389.1 48.5 295
125 4 25 301 33 318 5.10E-71 276.2 6596 cellular
organisms;Eukaryota;Opisthokonta;Metazoa;Eumetazoa;Bilateria;Protostomia;Spiralia;Lophotrochozoa;Mollusca;Bivalvia;Autobranchia;Heteroconchia;Euheterodonta;Imparidentia;Venerida;Veneroidea;Veneridae;Mercenaria;Mercenaria mercenaria
UdeM00000018566 225164 370.5 UdeM00000018566 XP_009062152.1 53.4
328 144 3 73 395 362 685 2.60E-99 370.5 225164
cellular
organisms;Eukaryota;Opisthokonta;Metazoa;Eumetazoa;Bilateria;Protostomia;Spiralia;Lophotrochozoa;Mollusca;Gastropoda;Patellogastropoda;Lottioidea;Lottiidae;Lottia;Lottia gigantea
UdeM00000018567 29159 266.5 UdeM00000018567 XP_034315989.1 67.5 191
62 0 6 196 31 221 2.60E-68 266.5 29159 cellular
organisms;Eukaryota;Opisthokonta;Metazoa;Eumetazoa;Bilateria;Protostomia;Spiralia;Lophotrochozoa;Mollusca;Bivalvia;Autobranchia;Pteriomorphia;Ostreida;Ostreoidea;Ostreidae;Crassostrea;Crassostrea gigas
UdeM00000018568 151771 59.7 UdeM00000018568 XP_028412199.1 37
92 55 2 2 92 3 92 2.50E-06 59.7 151771
cellular
organisms;Eukaryota;Opisthokonta;Metazoa;Eumetazoa;Cnidaria;Anthozoa;Octocorallia;Alcyonacea;Alcyoniina;Nephtheidae;Dendronephthya;Dendronephthya gigantea
UdeM00000018569 45351 82.8 UdeM00000018569 XP_001623471.2 32.6 172
95 4 3 155 6 175 5.80E-13 82.8 45351 cellular
organisms;Eukaryota;Opisthokonta;Metazoa;Eumetazoa;Cnidaria;Anthozoa;Hexacorallia;Actiniaria;Edwardsiidae;Nematostella;Nematostella vectensis
UdeM00000018570 6596 548.9 UdeM00000018570 XP_045178302.1 71.5 432
120 2 24 453 13 443 6.10E-153 548.9 6596 cellular
organisms;Eukaryota;Opisthokonta;Metazoa;Eumetazoa;Bilateria;Protostomia;Spiralia;Lophotrochozoa;Mollusca;Bivalvia;Autobranchia;Heteroconchia;Euheterodonta;Imparidentia;Venerida;Veneroidea;Veneridae;Mercenaria;Mercenaria mercenaria
UdeM00000018571 6573 91.7 UdeM00000018571 XP_021365625.1 51.7 87
42 0 53 139 40 126 9.20E-16 91.7 6573 cellular
organisms;Eukaryota;Opisthokonta;Metazoa;Eumetazoa;Bilateria;Protostomia;Spiralia;Lophotrochozoa;Mollusca;Bivalvia;Autobranchia;Pteriomorphia;Pectinida;Pectinoidea;Pectinidae;Mizuhopecten;Mizuhopecten yessoensis
UdeM00000018572 6596 783.9 UdeM00000018572 XP_045209346.1 41.1 1039
603 8 23 1052 35 1073 2.70E-223 783.9 6596 cellular
organisms;Eukaryota;Opisthokonta;Metazoa;Eumetazoa;Bilateria;Protostomia;Spiralia;Lophotrochozoa;Mollusca;Bivalvia;Autobranchia;Heteroconchia;Euheterodonta;Imparidentia;Venerida;Veneroidea;Veneridae;Mercenaria;Mercenaria mercenaria
UdeM00000018573 6596 787.7 UdeM00000018573 XP_045209346.1 40.8 1061
616 9 2 1051 14 1073 1.80E-224 787.7 6596 cellular
organisms;Eukaryota;Opisthokonta;Metazoa;Eumetazoa;Bilateria;Protostomia;Spiralia;Lophotrochozoa;Mollusca;Bivalvia;Autobranchia;Heteroconchia;Euheterodonta;Imparidentia;Venerida;Veneroidea;Veneridae;Mercenaria;Mercenaria mercenaria
UdeM00000018574 6596 125.9 UdeM00000018574 XP_045209346.1 41.3 150
87 1 2 150 924 1073 4.90E-26 125.9 6596 cellular

organisms;Eukaryota;Opisthokonta;Metazoa;Eumetazoa;Bilateria;Protostomia;Spiralia;Lophotrochozoa;Mollusca;Bivalvia;Autobranchia;Heteroconchia;Euheterodonta;Imparidentia;Venerida;Veneroidea;Veneridae;Mercenaria;Mercenaria mercenaria
UdeM00000018578 6596 377.9 UdeM00000018578 XP_045177776.1 40.8 534 289 10 1 522 405 923 3.20E-101 377.9 6596 cellular organisms;Eukaryota;Opisthokonta;Metazoa;Eumetazoa;Bilateria;Protostomia;Spiralia;Lophotrochozoa;Mollusca;Bivalvia;Autobranchia;Heteroconchia;Euheterodonta;Imparidentia;Venerida;Veneroidea;Veneridae;Mercenaria;Mercenaria mercenaria
UdeM00000018580 45351 83.6 UdeM00000018580 XP_001623471.2 32.5 197 100 6 3 180 6 188 3.50E-13 83.6 45351 cellular organisms;Eukaryota;Opisthokonta;Metazoa;Eumetazoa;Cnidaria;Anthozoa;Hexacorallia;Actiniaria;Edwardsiidae;Nematostella;Nematostella vectensis
UdeM00000018581 6596 166.8 UdeM00000018581 XP_045177695.1 48.6 177 89 2 15 190 172 347 2.80E-38 166.8 6596 cellular organisms;Eukaryota;Opisthokonta;Metazoa;Eumetazoa;Bilateria;Protostomia;Spiralia;Lophotrochozoa;Mollusca;Bivalvia;Autobranchia;Heteroconchia;Euheterodonta;Imparidentia;Venerida;Veneroidea;Veneridae;Mercenaria;Mercenaria mercenaria
UdeM00000018582 6579 78.2 UdeM00000018582 XP_033725557.1 67.4 43 14 0 1 43 59 101 9.30E-12 78.2 6579 cellular organisms;Eukaryota;Opisthokonta;Metazoa;Eumetazoa;Bilateria;Protostomia;Spiralia;Lophotrochozoa;Mollusca;Bivalvia;Autobranchia;Pteriomorphia;Pectinida;Pectinoidea;Pectinidae;Pecten;Pecten maximus
UdeM00000018583 6596 201.4 UdeM00000018583 XP_045177778.1 41.4 302 155 7 1 291 405 695 2.60E-48 201.4 6596 cellular organisms;Eukaryota;Opisthokonta;Metazoa;Eumetazoa;Bilateria;Protostomia;Spiralia;Lophotrochozoa;Mollusca;Bivalvia;Autobranchia;Heteroconchia;Euheterodonta;Imparidentia;Venerida;Veneroidea;Veneridae;Mercenaria;Mercenaria mercenaria
UdeM00000018584 1250792 153.3 UdeM00000018584 XP_041861569.1 37.5 224 101 5 76 265 449 667 4.30E-34 153.3 1250792 cellular organisms;Eukaryota;Opisthokonta;Metazoa;Eumetazoa;Bilateria;Deuterostomia;Chordata;Craniata;Vertebrata;Gnathostomata;Teleostomi;Euteleostomi;Actinopterygii;Actinopteri;Neopterygii;Teleostei;Osteoglossocephalai;Clupeocephala;Euteleosteomorpha;Neoteleostei;Eurypterygia;Ctenosquamata;Acanthomorphata;Euacanthomorphacea;Percomorphaceae;Ovalentaria;Atherinomorphae;Atheriniformes;Melanotaeniidae;Melanotaenia;Melanotaenia boesemani
UdeM00000018587 45351 86.7 UdeM00000018587 XP_001623471.2 32.8 189 98 5 10 181 12 188 4.00E-14 86.7 45351 cellular organisms;Eukaryota;Opisthokonta;Metazoa;Eumetazoa;Cnidaria;Anthozoa;Hexacorallia;Actiniaria;Edwardsiidae;Nematostella;Nematostella vectensis
UdeM00000018588 6596 152.1 UdeM00000018588 XP_045177590.1 68 100 32 0 125 224 826 925 9.50E-34 152.1 6596 cellular organisms;Eukaryota;Opisthokonta;Metazoa;Eumetazoa;Bilateria;Protostomia;Spiralia;Lophotrochozoa;Mollusca;Bivalvia;Autobranchia;Heteroconchia;Euheterodonta;Imparidentia;Venerida;Veneroidea;Veneridae;Mercenaria;Mercenaria mercenaria
UdeM00000018589 1735272 122.5 UdeM00000018589 XP_041356959.1 63.3 98 35 1 125 222 116 212 6.90E-25 122.5 1735272 cellular organisms;Eukaryota;Opisthokonta;Metazoa;Eumetazoa;Bilateria;Protostomia;

Spiralia;Lophotrochozoa;Mollusca;Gastropoda;Neomphalina;Neomphaloidea;Peltospiridae;Gigantopelta;Gigantopelta aegis
UdeM00000018590 6500 78.2 UdeM00000018590 XP_035825129.1 37.7 77 48 0 1 77 703 779 5.50E-12 78.2 6500 cellular organisms;Eukaryota;Opisthokonta;Metazoa;Eumetazoa;Bilateria;Protostomia;Spiralia;Lophotrochozoa;Mollusca;Gastropoda;Heterobranchia;Euthyneura;Tectipleura;Aplysiida;Aplysioidea;Aplysiidae;Aplysia;Aplysia californica
UdeM00000018591 7739 185.7 UdeM00000018591 XP_035662901.1 50.5 190 90 2 1 189 403 589 5.60E-44 185.7 7739 cellular organisms;Eukaryota;Opisthokonta;Metazoa;Eumetazoa;Bilateria;Deuterostomia;Chordata;Cephalochordata;Leptocardii;Amphioxiformes;Branchiostomidae;Branchiostoma;Branchiostoma floridae
UdeM00000018592 6596 540.4 UdeM00000018592 XP_045177590.1 50.6 478 230 6 14 488 249 723 2.60E-150 540.4 6596 cellular organisms;Eukaryota;Opisthokonta;Metazoa;Eumetazoa;Bilateria;Protostomia;Spiralia;Lophotrochozoa;Mollusca;Bivalvia;Autobranchia;Heteroconchia;Euheterodonta;Imparidentia;Venerida;Veneroidea;Veneridae;Mercenaria;Mercenaria mercenaria
UdeM00000018594 6596 166.4 UdeM00000018594 XP_045177590.1 41.5 217 112 5 4 211 45 255 4.50E-38 166.4 6596 cellular organisms;Eukaryota;Opisthokonta;Metazoa;Eumetazoa;Bilateria;Protostomia;Spiralia;Lophotrochozoa;Mollusca;Bivalvia;Autobranchia;Heteroconchia;Euheterodonta;Imparidentia;Venerida;Veneroidea;Veneridae;Mercenaria;Mercenaria mercenaria
UdeM00000018595 29159 108.2 UdeM00000018595 XP_034319609.1 26.1 268 106 2 1 176 687 954 1.10E-20 108.2 29159 cellular organisms;Eukaryota;Opisthokonta;Metazoa;Eumetazoa;Bilateria;Protostomia;Spiralia;Lophotrochozoa;Mollusca;Bivalvia;Autobranchia;Pteriomorphia;Ostreida;Ostreoidea;Ostreidae;Crassostrea;Crassostrea gigas
UdeM00000018596 29159 66.2 UdeM00000018596 XP_034322147.1 28.2 177 28 1 1 78 374 550 2.10E-08 66.2 29159 cellular organisms;Eukaryota;Opisthokonta;Metazoa;Eumetazoa;Bilateria;Protostomia;Spiralia;Lophotrochozoa;Mollusca;Bivalvia;Autobranchia;Pteriomorphia;Ostreida;Ostreoidea;Ostreidae;Crassostrea;Crassostrea gigas
UdeM00000018599 6596 187.6 UdeM00000018599 XP_045177781.1 43.6 220 121 3 49 267 401 618 4.40E-44 187.6 6596 cellular organisms;Eukaryota;Opisthokonta;Metazoa;Eumetazoa;Bilateria;Protostomia;Spiralia;Lophotrochozoa;Mollusca;Bivalvia;Autobranchia;Heteroconchia;Euheterodonta;Imparidentia;Venerida;Veneroidea;Veneridae;Mercenaria;Mercenaria mercenaria
UdeM00000018600 29159 201.8 UdeM00000018600 XP_011449140.2 50.9 163 80 0 8 170 107 269 7.00E-49 201.8 29159 cellular organisms;Eukaryota;Opisthokonta;Metazoa;Eumetazoa;Bilateria;Protostomia;Spiralia;Lophotrochozoa;Mollusca;Bivalvia;Autobranchia;Pteriomorphia;Ostreida;Ostreoidea;Ostreidae;Crassostrea;Crassostrea gigas
UdeM00000018601 6596 190.7 UdeM00000018601 XP_045177778.1 38.1 299 168 5 1 290 405 695 4.30E-45 190.7 6596 cellular organisms;Eukaryota;Opisthokonta;Metazoa;Eumetazoa;Bilateria;Protostomia;Spiralia;Lophotrochozoa;Mollusca;Bivalvia;Autobranchia;Heteroconchia;Euheterodonta;Imparidentia;Venerida;Veneroidea;Veneridae;Mercenaria;Mercenaria mercenaria
UdeM00000018603 1250792 139.8 UdeM00000018603 XP_041861569.1 43.7 167 86 4 3 168 508 667 3.20E-30 139.8 1250792 cellular

organisms;Eukaryota;Opisthokonta;Metazoa;Eumetazoa;Bilateria;Deuterostomia;Chordata;Craniata;Vertebrata;Gnathostomata;Teleostomi;Euteleostomi;Actinopterygii;Actinopteri;Neopterygii;Teleostei;Osteoglossocephalai;Clupeocephala;Euteleosteomorpha;Neoteleostei;Eurypterygia;Ctenosquamata;Acanthomorphata;Euacanthomorphacea;Percomorphaceae;Ovalentaria;Atherinomorphae;Atheriniformes;Melanotaeniidae;Melanotaenia;Melanotaenia boesemani
UdeM00000018604 7962 91.7 UdeM00000018604 XP_042606186.1 35.3 150 80 6 5 138 273 421 8.10E-16 91.7 7962 cellular organisms;Eukaryota;Opisthokonta;Metazoa;Eumetazoa;Bilateria;Deuterostomia;Chordata;Craniata;Vertebrata;Gnathostomata;Teleostomi;Euteleostomi;Actinopterygii;Actinopteri;Neopterygii;Teleostei;Osteoglossocephalai;Clupeocephala;Otomorpha;Ostariophysi;Otophysi;Cypriniphysae;Cypriniformes;Cyprinoidei;Cyprinidae;Cyprininae;Cyprinus;Cyprinus carpio
UdeM00000018605 6596 165.2 UdeM00000018605 XP_045200121.1 37.9 253 147 3 40 283 51 302 1.30E-37 165.2 6596 cellular organisms;Eukaryota;Opisthokonta;Metazoa;Eumetazoa;Bilateria;Protostomia;Spiralia;Lophotrochozoa;Mollusca;Bivalvia;Autobranchia;Heteroconchia;Euheterodonta;Imparidentia;Venerida;Veneroidea;Veneridae;Mercenaria;Mercenaria mercenaria
UdeM00000018606 6579 149.1 UdeM00000018606 XP_033732676.1 32.9 298 180 10 17 306 121 406 9.50E-33 149.1 6579 cellular organisms;Eukaryota;Opisthokonta;Metazoa;Eumetazoa;Bilateria;Protostomia;Spiralia;Lophotrochozoa;Mollusca;Bivalvia;Autobranchia;Pteriomorphia;Pectinida;Pectinoidea;Pectinidae;Pecten;Pecten maximus
UdeM00000018607 6596 197.2 UdeM00000018607 XP_045177776.1 43.2 234 128 3 11 243 696 925 5.70E-47 197.2 6596 cellular organisms;Eukaryota;Opisthokonta;Metazoa;Eumetazoa;Bilateria;Protostomia;Spiralia;Lophotrochozoa;Mollusca;Bivalvia;Autobranchia;Heteroconchia;Euheterodonta;Imparidentia;Venerida;Veneroidea;Veneridae;Mercenaria;Mercenaria mercenaria
UdeM00000018608 6565 63.9 UdeM00000018608 XP_022320668.1 30.1 166 82 6 5 138 627 790 1.80E-07 63.9 6565 cellular organisms;Eukaryota;Opisthokonta;Metazoa;Eumetazoa;Bilateria;Protostomia;Spiralia;Lophotrochozoa;Mollusca;Bivalvia;Autobranchia;Pteriomorphia;Ostreida;Ostreoidea;Ostreidae;Crassostrea;Crassostrea virginica
UdeM00000018609 6579 163.3 UdeM00000018609 XP_033725289.1 40.7 204 74 2 1 157 316 519 2.70E-37 163.3 6579 cellular organisms;Eukaryota;Opisthokonta;Metazoa;Eumetazoa;Bilateria;Protostomia;Spiralia;Lophotrochozoa;Mollusca;Bivalvia;Autobranchia;Pteriomorphia;Pectinida;Pectinoidea;Pectinidae;Pecten;Pecten maximus
UdeM00000018610 6596 214.5 UdeM00000018610 XP_045177778.1 41.1 319 166 7 386 693 388 695 5.00E-52 214.5 6596 cellular organisms;Eukaryota;Opisthokonta;Metazoa;Eumetazoa;Bilateria;Protostomia;Spiralia;Lophotrochozoa;Mollusca;Bivalvia;Autobranchia;Heteroconchia;Euheterodonta;Imparidentia;Venerida;Veneroidea;Veneridae;Mercenaria;Mercenaria mercenaria
UdeM00000018611 6596 400.6 UdeM00000018611 XP_045174460.1 33.6 679 342 10 6 659 5 599 3.90E-108 400.6 6596 cellular organisms;Eukaryota;Opisthokonta;Metazoa;Eumetazoa;Bilateria;Protostomia;Spiralia;Lophotrochozoa;Mollusca;Bivalvia;Autobranchia;Heteroconchia;Euheterodonta;Imparidentia;Venerida;Veneroidea;Veneridae;Mercenaria;Mercenaria mercenaria
UdeM00000018612 6573 172.9 UdeM00000018612 XP_021363425.1 52.7 150 71 0 2 151 4 153 3.90E-40 172.9 6573 cellular

organisms;Eukaryota;Opisthokonta;Metazoa;Eumetazoa;Bilateria;Protostomia;Spiralia;Lophotrochozoa;Mollusca;Bivalvia;Autobranchia;Pteriomorphia;Pectinida;Pectinoidea;Pectinidae;Mizuhopecten;Mizuhopecten yessoensis
UdeM00000018613 456900 70.9 UdeM00000018613 XP_018394401.1 32.8 137 84 2 17 152 15 144 1.60E-09 70.9 456900 cellular organisms;Eukaryota;Opisthokonta;Metazoa;Eumetazoa;Bilateria;Protostomia;Ecdysozoa;Panarthropoda;Arthropoda;Mandibulata;Pancrustacea;Hexapoda;Insecta;Dicondylia;Pterygota;Neoptera;Endopterygota;Hymenoptera;Apocrita;Aculeata;Formicoidea;Formicidae;Myrmicinae;Attini;Cyphomyrmex;Cyphomyrmex costatus
UdeM00000018614 6596 439.9 UdeM00000018614 XP_045178493.1 53.3 381 173 3 1 379 1 378 3.30E-120 439.9 6596 cellular organisms;Eukaryota;Opisthokonta;Metazoa;Eumetazoa;Bilateria;Protostomia;Spiralia;Lophotrochozoa;Mollusca;Bivalvia;Autobranchia;Heteroconchia;Euheterodonta;Imparidentia;Venerida;Veneroidea;Veneridae;Mercenaria;Mercenaria mercenaria
UdeM00000018615 6596 436 UdeM00000018615 XP_045178493.1 53.8 381 171 3 1 379 1 378 4.80E-119 436 6596 cellular organisms;Eukaryota;Opisthokonta;Metazoa;Eumetazoa;Bilateria;Protostomia;Spiralia;Lophotrochozoa;Mollusca;Bivalvia;Autobranchia;Heteroconchia;Euheterodonta;Imparidentia;Venerida;Veneroidea;Veneridae;Mercenaria;Mercenaria mercenaria
UdeM00000018616 6573 580.5 UdeM00000018616 XP_021367864.1 88.6 315 36 0 1 315 1 315 1.30E-162 580.5 6573 cellular organisms;Eukaryota;Opisthokonta;Metazoa;Eumetazoa;Bilateria;Protostomia;Spiralia;Lophotrochozoa;Mollusca;Bivalvia;Autobranchia;Pteriomorphia;Pectinida;Pectinoidea;Pectinidae;Mizuhopecten;Mizuhopecten yessoensis
UdeM00000018617 6526 244.2 UdeM00000018617 XP_013060793.1 52.4 225 107 0 56 280 1 225 2.00E-61 244.2 6526 cellular organisms;Eukaryota;Opisthokonta;Metazoa;Eumetazoa;Bilateria;Protostomia;Spiralia;Lophotrochozoa;Mollusca;Gastropoda;Heterobranchia;Euthyneura;Panpulmonata;Hygrophila;Lymnaeoidea;Planorbidae;Biomphalaria;Biomphalaria glabrata
UdeM00000018619 6573 142.9 UdeM00000018619 XP_021367172.1 37.8 336 179 6 51 363 394 722 8.00E-31 142.9 6573 cellular organisms;Eukaryota;Opisthokonta;Metazoa;Eumetazoa;Bilateria;Protostomia;Spiralia;Lophotrochozoa;Mollusca;Bivalvia;Autobranchia;Pteriomorphia;Pectinida;Pectinoidea;Pectinidae;Mizuhopecten;Mizuhopecten yessoensis
UdeM00000018618 6596 254.2 UdeM00000018618 XP_045178535.1 39.2 725 317 15 1 668 1 658 4.60E-64 254.2 6596 cellular organisms;Eukaryota;Opisthokonta;Metazoa;Eumetazoa;Bilateria;Protostomia;Spiralia;Lophotrochozoa;Mollusca;Bivalvia;Autobranchia;Heteroconchia;Euheterodonta;Imparidentia;Venerida;Veneroidea;Veneridae;Mercenaria;Mercenaria mercenaria
UdeM00000018620 6565 161.8 UdeM00000018620 XP_022324511.1 40.3 278 138 5 1 270 1 258 1.60E-36 161.8 6565 cellular organisms;Eukaryota;Opisthokonta;Metazoa;Eumetazoa;Bilateria;Protostomia;Spiralia;Lophotrochozoa;Mollusca;Bivalvia;Autobranchia;Pteriomorphia;Ostreida;Ostreoidea;Ostreidae;Crassostrea;Crassostrea virginica
UdeM00000018621 6596 280 UdeM00000018621 XP_045179111.1 33.3 670 179 15 13 425 70 728 5.10E-72 280 6596 cellular organisms;Eukaryota;Opisthokonta;Metazoa;Eumetazoa;Bilateria;Protostomia;Spiralia;Lophotrochozoa;Mollusca;Bivalvia;Autobranchia;Heteroconchia;Euhe

terodonta;Imparidentia;Venerida;Veneroidea;Veneridae;Mercenaria;Mercenari
a mercenaria
UdeM00000018622 400727 184.5 UdeM00000018622 XP_025089150.1 38.6
259 148 6 24 271 4 262 1.80E-43 184.5 400727
cellular
organisms;Eukaryota;Opisthokonta;Metazoa;Eumetazoa;Bilateria;Protostomia;
Spiralia;Lophotrochozoa;Mollusca;Gastropoda;Caenogastropoda;Architaeniogl
ossa;Ampullarioidea;Ampullariidae;Pomacea;Pomacea canaliculata
UdeM00000018624 6573 177.9 UdeM00000018624 XP_021371412.1 41.7 223
124 4 42 262 1118 1336 3.80E-41 177.9 6573 cellular
organisms;Eukaryota;Opisthokonta;Metazoa;Eumetazoa;Bilateria;Protostomia;
Spiralia;Lophotrochozoa;Mollusca;Bivalvia;Autobranchia;Pteriomorphia;Pect
inida;Pectinoidea;Pectinidae;Mizuhopecten;Mizuhopecten yessoensis
UdeM00000018625 70779 91.7 UdeM00000018625 XP_015772592.1 37.9 174
93 3 1 159 185 358 9.60E-16 91.7 70779 cellular
organisms;Eukaryota;Opisthokonta;Metazoa;Eumetazoa;Cnidaria;Anthozoa;Hexa
corallia;Scleractinia;Astrocoeniina;Acroporidae;Acropora;Acropora
digitifera
UdeM00000018626 1735272 101.7 UdeM00000018626 XP_041354133.1 39.3
145 73 4 25 169 1 130 1.00E-18 101.7 1735272
cellular
organisms;Eukaryota;Opisthokonta;Metazoa;Eumetazoa;Bilateria;Protostomia;
Spiralia;Lophotrochozoa;Mollusca;Gastropoda;Neomphalina;Neomphaloidea;Pel
tospiridae;Gigantopelta;Gigantopelta aegis
UdeM00000018627 6579 84.7 UdeM00000018627 XP_033727573.1 31.7 202
114 7 13 193 45 243 2.00E-13 84.7 6579 cellular
organisms;Eukaryota;Opisthokonta;Metazoa;Eumetazoa;Bilateria;Protostomia;
Spiralia;Lophotrochozoa;Mollusca;Bivalvia;Autobranchia;Pteriomorphia;Pect
inida;Pectinoidea;Pectinidae;Pecten;Pecten maximus
UdeM00000018628 225164 75.5 UdeM00000018628 XP_009057862.1 28
175 112 7 266 433 30 197 2.00E-10 75.5 225164
cellular
organisms;Eukaryota;Opisthokonta;Metazoa;Eumetazoa;Bilateria;Protostomia;
Spiralia;Lophotrochozoa;Mollusca;Gastropoda;Patellogastropoda;Lottioidea;
Lottiidae;Lottia;Lottia gigantea
UdeM00000018629 29159 155.6 UdeM00000018629 XP_011419444.2 40.4 213
123 3 10 220 21 231 7.50E-35 155.6 29159 cellular
organisms;Eukaryota;Opisthokonta;Metazoa;Eumetazoa;Bilateria;Protostomia;
Spiralia;Lophotrochozoa;Mollusca;Bivalvia;Autobranchia;Pteriomorphia;Ostr
eida;Ostreoidea;Ostreidae;Crassostrea;Crassostrea gigas
UdeM00000018630 6596 170.2 UdeM00000018630 XP_045158722.1 42.3 215
113 3 1 215 48 251 3.20E-39 170.2 6596 cellular
organisms;Eukaryota;Opisthokonta;Metazoa;Eumetazoa;Bilateria;Protostomia;
Spiralia;Lophotrochozoa;Mollusca;Bivalvia;Autobranchia;Heteroconchia;Euhe
terodonta;Imparidentia;Venerida;Veneroidea;Veneridae;Mercenaria;Mercenari
a mercenaria
UdeM00000018631 7054 67.4 UdeM00000018631 XP_031350403.1 46.7 60
31 1 4 62 225 284 9.50E-09 67.4 7054 cellular
organisms;Eukaryota;Opisthokonta;Metazoa;Eumetazoa;Bilateria;Protostomia;
Ecdysozoa;Panarthropoda;Arthropoda;Mandibulata;Pancrustacea;Hexapoda;Inse
cta;Dicondylia;Pterygota;Neoptera;Endopterygota;Coleoptera;Polyphaga;Elat
eriformia;Elateroidea;Lampyridae;Lampyrinae;Photinus;Photinus pyralis
UdeM00000018632 37621 84 UdeM00000018632 XP_014671072.1 47.4 78
39 1 24 101 106 181 1.20E-13 84 37621 cellular

organisms;Eukaryota;Opisthokonta;Metazoa;Eumetazoa;Bilateria;Protostomia;
Ecdysozoa;Scalidophora;Priapulida;Priapulimorpha;Priapulimorphida;Priapul
idae;Priapulus;Priapulus caudatus
UdeM00000018633 6596 161 UdeM00000018633 XP_045158722.1 45.6 182
88 3 3 184 75 245 1.80E-36 161 6596 cellular
organisms;Eukaryota;Opisthokonta;Metazoa;Eumetazoa;Bilateria;Protostomia;
Spiralia;Lophotrochozoa;Mollusca;Bivalvia;Autobranchia;Heteroconchia;Euhe
terodonta;Imparidentia;Venerida;Veneroidea;Veneridae;Mercenaria;Mercenari
a mercenaria
UdeM00000018634 6573 92.4 UdeM00000018634 XP_021354128.1 60.6 66
26 0 22 87 21 86 3.00E-16 92.4 6573 cellular
organisms;Eukaryota;Opisthokonta;Metazoa;Eumetazoa;Bilateria;Protostomia;
Spiralia;Lophotrochozoa;Mollusca;Bivalvia;Autobranchia;Pteriomorphia;Pect
inida;Pectinoidea;Pectinidae;Mizuhopecten;Mizuhopecten yessoensis
UdeM00000018635 6596 1231.1 UdeM00000018635 XP_045179723.1 63.9
917 326 1 1 912 100 1016 0.00E+00 1231.1 6596
cellular
organisms;Eukaryota;Opisthokonta;Metazoa;Eumetazoa;Bilateria;Protostomia;
Spiralia;Lophotrochozoa;Mollusca;Bivalvia;Autobranchia;Heteroconchia;Euhe
terodonta;Imparidentia;Venerida;Veneroidea;Veneridae;Mercenaria;Mercenari
a mercenaria
UdeM00000018636 400727 537 UdeM00000018636 XP_025092893.1 61.4
420 147 2 21 426 27 445 2.20E-149 537 400727
cellular
organisms;Eukaryota;Opisthokonta;Metazoa;Eumetazoa;Bilateria;Protostomia;
Spiralia;Lophotrochozoa;Mollusca;Gastropoda;Caenogastropoda;Architaeniogl
ossa;Ampullarioidea;Ampullariidae;Pomacea;Pomacea canaliculata
UdeM00000018637 400727 539.7 UdeM00000018637 XP_025092893.1 57.9
454 156 2 21 474 27 445 3.80E-150 539.7 400727
cellular
organisms;Eukaryota;Opisthokonta;Metazoa;Eumetazoa;Bilateria;Protostomia;
Spiralia;Lophotrochozoa;Mollusca;Gastropoda;Caenogastropoda;Architaeniogl
ossa;Ampullarioidea;Ampullariidae;Pomacea;Pomacea canaliculata
UdeM00000018638 6596 1280 UdeM00000018638 XP_045177686.1 67.5 944
267 5 1 907 1 941 0.00E+00 1280 6596 cellular
organisms;Eukaryota;Opisthokonta;Metazoa;Eumetazoa;Bilateria;Protostomia;
Spiralia;Lophotrochozoa;Mollusca;Bivalvia;Autobranchia;Heteroconchia;Euhe
terodonta;Imparidentia;Venerida;Veneroidea;Veneridae;Mercenaria;Mercenari
a mercenaria
UdeM00000018639 6596 721.1 UdeM00000018639 XP_045178731.1 55.3 573
251 4 40 610 120 689 1.20E-204 721.1 6596 cellular
organisms;Eukaryota;Opisthokonta;Metazoa;Eumetazoa;Bilateria;Protostomia;
Spiralia;Lophotrochozoa;Mollusca;Bivalvia;Autobranchia;Heteroconchia;Euhe
terodonta;Imparidentia;Venerida;Veneroidea;Veneridae;Mercenaria;Mercenari
a mercenaria
UdeM00000018640 29159 208.4 UdeM00000018640 XP_011447117.2 42 319
148 8 10 305 2 306 1.30E-50 208.4 29159 cellular
organisms;Eukaryota;Opisthokonta;Metazoa;Eumetazoa;Bilateria;Protostomia;
Spiralia;Lophotrochozoa;Mollusca;Bivalvia;Autobranchia;Pteriomorphia;Ostr
eida;Ostreoidea;Ostreidae;Crassostrea;Crassostrea gigas
UdeM00000018641 6579 194.9 UdeM00000018641 XP_033749282.1 43 270
129 8 30 282 54 315 1.40E-46 194.9 6579 cellular
organisms;Eukaryota;Opisthokonta;Metazoa;Eumetazoa;Bilateria;Protostomia;

Spiralia;Lophotrochozoa;Mollusca;Bivalvia;Autobranchia;Pteriomorphia;Pectinida;Pectinoidea;Pectinidae;Pecten;Pecten maximus
UdeM00000018642 6579 221.1 UdeM00000018642 XP_033749282.1 40.2 321 167 8 7 310 3 315 2.00E-54 221.1 6579 cellular organisms;Eukaryota;Opisthokonta;Metazoa;Eumetazoa;Bilateria;Protostomia;Spiralia;Lophotrochozoa;Mollusca;Bivalvia;Autobranchia;Pteriomorphia;Pectinida;Pectinoidea;Pectinidae;Pecten;Pecten maximus
UdeM00000018643 6596 297.7 UdeM00000018643 XP_045201862.1 31.3 707 428 19 5 669 4 694 3.60E-77 297.7 6596 cellular organisms;Eukaryota;Opisthokonta;Metazoa;Eumetazoa;Bilateria;Protostomia;Spiralia;Lophotrochozoa;Mollusca;Bivalvia;Autobranchia;Heteroconchia;Euheterodonta;Imparidentia;Venerida;Veneroidea;Veneridae;Mercenaria;Mercenaria mercenaria
UdeM00000018644 6596 179.5 UdeM00000018644 XP_045156925.1 35.2 307 181 7 6 297 5 308 6.40E-42 179.5 6596 cellular organisms;Eukaryota;Opisthokonta;Metazoa;Eumetazoa;Bilateria;Protostomia;Spiralia;Lophotrochozoa;Mollusca;Bivalvia;Autobranchia;Heteroconchia;Euheterodonta;Imparidentia;Venerida;Veneroidea;Veneridae;Mercenaria;Mercenaria mercenaria
UdeM00000018645 6596 100.5 UdeM00000018645 XP_045157494.1 26.4 368 226 12 14 349 347 701 4.50E-18 100.5 6596 cellular organisms;Eukaryota;Opisthokonta;Metazoa;Eumetazoa;Bilateria;Protostomia;Spiralia;Lophotrochozoa;Mollusca;Bivalvia;Autobranchia;Heteroconchia;Euheterodonta;Imparidentia;Venerida;Veneroidea;Veneridae;Mercenaria;Mercenaria mercenaria
UdeM00000018646 6596 288.5 UdeM00000018646 XP_045201862.1 30.1 700 434 17 5 658 4 694 2.20E-74 288.5 6596 cellular organisms;Eukaryota;Opisthokonta;Metazoa;Eumetazoa;Bilateria;Protostomia;Spiralia;Lophotrochozoa;Mollusca;Bivalvia;Autobranchia;Heteroconchia;Euheterodonta;Imparidentia;Venerida;Veneroidea;Veneridae;Mercenaria;Mercenaria mercenaria
UdeM00000018647 6596 233.8 UdeM00000018647 XP_045157409.1 32 466 289 7 6 444 5 469 4.30E-58 233.8 6596 cellular organisms;Eukaryota;Opisthokonta;Metazoa;Eumetazoa;Bilateria;Protostomia;Spiralia;Lophotrochozoa;Mollusca;Bivalvia;Autobranchia;Heteroconchia;Euheterodonta;Imparidentia;Venerida;Veneroidea;Veneridae;Mercenaria;Mercenaria mercenaria
UdeM00000018648 6596 61.6 UdeM00000018648 XP_045156697.1 29.2 137 94 2 41 174 571 707 1.20E-06 61.6 6596 cellular organisms;Eukaryota;Opisthokonta;Metazoa;Eumetazoa;Bilateria;Protostomia;Spiralia;Lophotrochozoa;Mollusca;Bivalvia;Autobranchia;Heteroconchia;Euheterodonta;Imparidentia;Venerida;Veneroidea;Veneridae;Mercenaria;Mercenaria mercenaria
UdeM00000018650 6596 293.5 UdeM00000018650 XP_045201862.1 30.1 710 432 18 5 669 4 694 6.90E-76 293.5 6596 cellular organisms;Eukaryota;Opisthokonta;Metazoa;Eumetazoa;Bilateria;Protostomia;Spiralia;Lophotrochozoa;Mollusca;Bivalvia;Autobranchia;Heteroconchia;Euheterodonta;Imparidentia;Venerida;Veneroidea;Veneridae;Mercenaria;Mercenaria mercenaria
UdeM00000018651 6596 791.6 UdeM00000018651 XP_045178731.1 55.9 621 256 4 16 621 72 689 7.40E-226 791.6 6596 cellular organisms;Eukaryota;Opisthokonta;Metazoa;Eumetazoa;Bilateria;Protostomia;Spiralia;Lophotrochozoa;Mollusca;Bivalvia;Autobranchia;Heteroconchia;Euhe

terodonta;Imparidentia;Venerida;Veneroidea;Veneridae;Mercenaria;Mercenaria mercenaria
UdeM00000018652 6596 405.2 UdeM00000018652 XP_045214637.1 49.7 384 189 3 9 392 14 393 9.40E-110 405.2 6596 cellular organisms;Eukaryota;Opisthokonta;Metazoa;Eumetazoa;Bilateria;Protostomia;Spiralia;Lophotrochozoa;Mollusca;Bivalvia;Autobranchia;Heteroconchia;Euheterodonta;Imparidentia;Venerida;Veneroidea;Veneridae;Mercenaria;Mercenaria mercenaria
UdeM00000018653 6596 370.5 UdeM00000018653 XP_045214637.1 49.6 343 170 2 11 353 16 355 2.30E-99 370.5 6596 cellular organisms;Eukaryota;Opisthokonta;Metazoa;Eumetazoa;Bilateria;Protostomia;Spiralia;Lophotrochozoa;Mollusca;Bivalvia;Autobranchia;Heteroconchia;Euheterodonta;Imparidentia;Venerida;Veneroidea;Veneridae;Mercenaria;Mercenaria mercenaria
UdeM00000018654 6596 424.9 UdeM00000018654 XP_045214637.1 51.2 383 184 2 9 391 14 393 1.10E-115 424.9 6596 cellular organisms;Eukaryota;Opisthokonta;Metazoa;Eumetazoa;Bilateria;Protostomia;Spiralia;Lophotrochozoa;Mollusca;Bivalvia;Autobranchia;Heteroconchia;Euheterodonta;Imparidentia;Venerida;Veneroidea;Veneridae;Mercenaria;Mercenaria mercenaria
UdeM00000018655 6596 402.9 UdeM00000018655 XP_045211006.1 51.2 383 184 2 19 401 11 390 4.80E-109 402.9 6596 cellular organisms;Eukaryota;Opisthokonta;Metazoa;Eumetazoa;Bilateria;Protostomia;Spiralia;Lophotrochozoa;Mollusca;Bivalvia;Autobranchia;Heteroconchia;Euheterodonta;Imparidentia;Venerida;Veneroidea;Veneridae;Mercenaria;Mercenaria mercenaria
UdeM00000018656 6596 302.8 UdeM00000018656 XP_045214637.1 49.5 293 145 2 1 293 106 395 4.90E-79 302.8 6596 cellular organisms;Eukaryota;Opisthokonta;Metazoa;Eumetazoa;Bilateria;Protostomia;Spiralia;Lophotrochozoa;Mollusca;Bivalvia;Autobranchia;Heteroconchia;Euheterodonta;Imparidentia;Venerida;Veneroidea;Veneridae;Mercenaria;Mercenaria mercenaria
UdeM00000018657 6596 397.1 UdeM00000018657 XP_045214637.1 50.9 373 180 2 7 379 26 395 2.50E-107 397.1 6596 cellular organisms;Eukaryota;Opisthokonta;Metazoa;Eumetazoa;Bilateria;Protostomia;Spiralia;Lophotrochozoa;Mollusca;Bivalvia;Autobranchia;Heteroconchia;Euheterodonta;Imparidentia;Venerida;Veneroidea;Veneridae;Mercenaria;Mercenaria mercenaria
UdeM00000018658 6596 392.1 UdeM00000018658 XP_045211006.1 50.5 374 182 2 4 377 20 390 7.90E-106 392.1 6596 cellular organisms;Eukaryota;Opisthokonta;Metazoa;Eumetazoa;Bilateria;Protostomia;Spiralia;Lophotrochozoa;Mollusca;Bivalvia;Autobranchia;Heteroconchia;Euheterodonta;Imparidentia;Venerida;Veneroidea;Veneridae;Mercenaria;Mercenaria mercenaria
UdeM00000018659 6596 414.8 UdeM00000018659 XP_045214637.1 51.7 373 177 2 7 379 26 395 1.10E-112 414.8 6596 cellular organisms;Eukaryota;Opisthokonta;Metazoa;Eumetazoa;Bilateria;Protostomia;Spiralia;Lophotrochozoa;Mollusca;Bivalvia;Autobranchia;Heteroconchia;Euheterodonta;Imparidentia;Venerida;Veneroidea;Veneridae;Mercenaria;Mercenaria mercenaria
UdeM00000018660 6596 421.4 UdeM00000018660 XP_045214637.1 51.2 385 185 2 19 403 14 395 1.30E-114 421.4 6596 cellular organisms;Eukaryota;Opisthokonta;Metazoa;Eumetazoa;Bilateria;Protostomia;Spiralia;Lophotrochozoa;Mollusca;Bivalvia;Autobranchia;Heteroconchia;Euhe

terodonta;Imparidentia;Venerida;Veneroidea;Veneridae;Mercenaria;Mercenaria mercenaria
UdeM00000018667 1735272 61.6 UdeM00000018667 XP_041355104.1 21.4 294 214 6 189 478 5 285 5.00E-06 61.6 1735272 cellular organisms;Eukaryota;Opisthokonta;Metazoa;Eumetazoa;Bilateria;Protostomia;Spiralia;Lophotrochozoa;Mollusca;Gastropoda;Neomphalina;Neomphaloidea;Peltospiridae;Gigantopelta;Gigantopelta aegis
UdeM00000018668 1735272 68.9 UdeM00000018668 XP_041355104.1 21 271 200 4 12 272 19 285 2.30E-08 68.9 1735272 cellular organisms;Eukaryota;Opisthokonta;Metazoa;Eumetazoa;Bilateria;Protostomia;Spiralia;Lophotrochozoa;Mollusca;Gastropoda;Neomphalina;Neomphaloidea;Peltospiridae;Gigantopelta;Gigantopelta aegis
UdeM00000018669 6596 73.9 UdeM00000018669 XP_045209248.1 20.1 463 341 12 156 602 137 586 9.20E-10 73.9 6596 cellular organisms;Eukaryota;Opisthokonta;Metazoa;Eumetazoa;Bilateria;Protostomia;Spiralia;Lophotrochozoa;Mollusca;Bivalvia;Autobranchia;Heteroconchia;Euheterodonta;Imparidentia;Venerida;Veneroidea;Veneridae;Mercenaria;Mercenaria mercenaria
UdeM00000018670 6596 403.7 UdeM00000018670 XP_045214637.1 49.7 382 190 1 9 390 14 393 2.70E-109 403.7 6596 cellular organisms;Eukaryota;Opisthokonta;Metazoa;Eumetazoa;Bilateria;Protostomia;Spiralia;Lophotrochozoa;Mollusca;Bivalvia;Autobranchia;Heteroconchia;Euheterodonta;Imparidentia;Venerida;Veneroidea;Veneridae;Mercenaria;Mercenaria mercenaria
UdeM00000018671 6596 390.6 UdeM00000018671 XP_045211006.1 48.7 382 194 1 19 400 11 390 2.40E-105 390.6 6596 cellular organisms;Eukaryota;Opisthokonta;Metazoa;Eumetazoa;Bilateria;Protostomia;Spiralia;Lophotrochozoa;Mollusca;Bivalvia;Autobranchia;Heteroconchia;Euheterodonta;Imparidentia;Venerida;Veneroidea;Veneridae;Mercenaria;Mercenaria mercenaria
UdeM00000018672 6596 1359.7 UdeM00000018672 XP_045177518.1 53.6 2591 910 33 1 2342 1 2549 0.00E+00 1359.7 6596 cellular organisms;Eukaryota;Opisthokonta;Metazoa;Eumetazoa;Bilateria;Protostomia;Spiralia;Lophotrochozoa;Mollusca;Bivalvia;Autobranchia;Heteroconchia;Euheterodonta;Imparidentia;Venerida;Veneroidea;Veneridae;Mercenaria;Mercenaria mercenaria
UdeM00000018673 1735272 379 UdeM00000018673 XP_041374422.1 41.6 846 377 18 2521 3343 938 1689 6.20E-101 379 1735272 cellular organisms;Eukaryota;Opisthokonta;Metazoa;Eumetazoa;Bilateria;Protostomia;Spiralia;Lophotrochozoa;Mollusca;Gastropoda;Neomphalina;Neomphaloidea;Peltospiridae;Gigantopelta;Gigantopelta aegis
UdeM00000018674 29159 236.1 UdeM00000018674 XP_034300416.1 67.7 195 56 2 102 296 759 946 5.70E-59 236.1 29159 cellular organisms;Eukaryota;Opisthokonta;Metazoa;Eumetazoa;Bilateria;Protostomia;Spiralia;Lophotrochozoa;Mollusca;Bivalvia;Autobranchia;Pteriomorphia;Ostreida;Ostreoidea;Ostreidae;Crassostrea;Crassostrea gigas
UdeM00000018675 6596 192.2 UdeM00000018675 XP_045177919.1 37.6 580 279 16 8 529 7 561 2.20E-45 192.2 6596 cellular organisms;Eukaryota;Opisthokonta;Metazoa;Eumetazoa;Bilateria;Protostomia;Spiralia;Lophotrochozoa;Mollusca;Bivalvia;Autobranchia;Heteroconchia;Euhe

terodonta;Imparidentia;Venerida;Veneroidea;Veneridae;Mercenaria;Mercenaria mercenaria
UdeM00000018676 6579 562 UdeM00000018676 XP_033741736.1 59.1 443 174 2 2 442 420 857 7.00E-157 562 6579 cellular organisms;Eukaryota;Opisthokonta;Metazoa;Eumetazoa;Bilateria;Protostomia;Spiralia;Lophotrochozoa;Mollusca;Bivalvia;Autobranchia;Pteriomorphia;Pectinida;Pectinoidea;Pectinidae;Pecten;Pecten maximus
UdeM00000018677 6579 277.7 UdeM00000018677 XP_033741736.1 54.9 244 108 1 1 242 614 857 1.50E-71 277.7 6579 cellular organisms;Eukaryota;Opisthokonta;Metazoa;Eumetazoa;Bilateria;Protostomia;Spiralia;Lophotrochozoa;Mollusca;Bivalvia;Autobranchia;Pteriomorphia;Pectinida;Pectinoidea;Pectinidae;Pecten;Pecten maximus
UdeM00000018678 6579 1265.8 UdeM00000018678 XP_033756411.1 70.6 867 254 1 1 867 69 934 0.00E+00 1265.8 6579 cellular organisms;Eukaryota;Opisthokonta;Metazoa;Eumetazoa;Bilateria;Protostomia;Spiralia;Lophotrochozoa;Mollusca;Bivalvia;Autobranchia;Pteriomorphia;Pectinida;Pectinoidea;Pectinidae;Pecten;Pecten maximus
UdeM00000018681 29159 63.9 UdeM00000018681 XP_034300301.1 35.9 103 61 2 35 133 7 108 6.80E-07 63.9 29159 cellular organisms;Eukaryota;Opisthokonta;Metazoa;Eumetazoa;Bilateria;Protostomia;Spiralia;Lophotrochozoa;Mollusca;Bivalvia;Autobranchia;Pteriomorphia;Ostreida;Ostreoidea;Ostreidae;Crassostrea;Crassostrea gigas
UdeM00000018682 6573 131 UdeM00000018682 XP_021352025.1 63.2 87 32 0 1 87 1 87 7.80E-28 131 6573 cellular organisms;Eukaryota;Opisthokonta;Metazoa;Eumetazoa;Bilateria;Protostomia;Spiralia;Lophotrochozoa;Mollusca;Bivalvia;Autobranchia;Pteriomorphia;Pectinida;Pectinoidea;Pectinidae;Mizuhopecten;Mizuhopecten yessoensis
UdeM00000018683 6565 1964.9 UdeM00000018683 XP_022325329.1 48 2034 905 26 56 1958 1648 3659 0.00E+00 1964.9 6565 cellular organisms;Eukaryota;Opisthokonta;Metazoa;Eumetazoa;Bilateria;Protostomia;Spiralia;Lophotrochozoa;Mollusca;Bivalvia;Autobranchia;Pteriomorphia;Ostreida;Ostreoidea;Ostreidae;Crassostrea;Crassostrea virginica
UdeM00000018684 6565 286.6 UdeM00000018684 XP_022325330.1 53 247 105 3 4 248 3645 3882 3.20E-74 286.6 6565 cellular organisms;Eukaryota;Opisthokonta;Metazoa;Eumetazoa;Bilateria;Protostomia;Spiralia;Lophotrochozoa;Mollusca;Bivalvia;Autobranchia;Pteriomorphia;Ostreida;Ostreoidea;Ostreidae;Crassostrea;Crassostrea virginica
UdeM00000018685 6596 1253.4 UdeM00000018685 XP_045179073.1 44.3 1517 772 18 157 1656 436 1896 0.00E+00 1253.4 6596 cellular organisms;Eukaryota;Opisthokonta;Metazoa;Eumetazoa;Bilateria;Protostomia;Spiralia;Lophotrochozoa;Mollusca;Bivalvia;Autobranchia;Heteroconchia;Euheterodonta;Imparidentia;Venerida;Veneroidea;Veneridae;Mercenaria;Mercenaria mercenaria
UdeM00000018686 1735272 825.5 UdeM00000018686 XP_041374928.1 76.7 558 125 2 16 572 954 1507 6.30E-236 825.5 1735272 cellular organisms;Eukaryota;Opisthokonta;Metazoa;Eumetazoa;Bilateria;Protostomia;Spiralia;Lophotrochozoa;Mollusca;Gastropoda;Neomphalina;Neomphaloidea;Peltospiridae;Gigantopelta;Gigantopelta aegis
UdeM00000018688 6579 592 UdeM00000018688 XP_033728944.1 56 611 196 11 6 553 68 668 7.60E-166 592 6579 cellular

organisms;Eukaryota;Opisthokonta;Metazoa;Eumetazoa;Bilateria;Protostomia;Spiralia;Lophotrochozoa;Mollusca;Bivalvia;Autobranchia;Pteriomorphia;Pectinida;Pectinoidea;Pectinidae;Pecten;Pecten maximus
UdeM00000018689 6596 214.5 UdeM00000018689 XP_045176664.1 29 556 336 15 25 543 13 546 3.40E-52 214.5 6596 cellular organisms;Eukaryota;Opisthokonta;Metazoa;Eumetazoa;Bilateria;Protostomia;Spiralia;Lophotrochozoa;Mollusca;Bivalvia;Autobranchia;Heteroconchia;Euheterodonta;Imparidentia;Venerida;Veneroidea;Veneridae;Mercenaria;Mercenaria mercenaria
UdeM00000018692 6596 122.1 UdeM00000018692 XP_045177550.1 55.6 117 51 1 1 117 1 116 4.80E-25 122.1 6596 cellular organisms;Eukaryota;Opisthokonta;Metazoa;Eumetazoa;Bilateria;Protostomia;Spiralia;Lophotrochozoa;Mollusca;Bivalvia;Autobranchia;Heteroconchia;Euheterodonta;Imparidentia;Venerida;Veneroidea;Veneridae;Mercenaria;Mercenaria mercenaria
UdeM00000018693 29159 659.4 UdeM00000018693 XP_034300049.1 64.6 531 178 5 1 531 1 521 4.20E-186 659.4 29159 cellular organisms;Eukaryota;Opisthokonta;Metazoa;Eumetazoa;Bilateria;Protostomia;Spiralia;Lophotrochozoa;Mollusca;Bivalvia;Autobranchia;Pteriomorphia;Ostreida;Ostreoidea;Ostreidae;Crassostrea;Crassostrea gigas
UdeM00000018694 6596 672.9 UdeM00000018694 XP_045177538.1 68.5 511 151 7 1 504 1 508 3.70E-190 672.9 6596 cellular organisms;Eukaryota;Opisthokonta;Metazoa;Eumetazoa;Bilateria;Protostomia;Spiralia;Lophotrochozoa;Mollusca;Bivalvia;Autobranchia;Heteroconchia;Euheterodonta;Imparidentia;Venerida;Veneroidea;Veneridae;Mercenaria;Mercenaria mercenaria
UdeM00000018695 6596 383.3 UdeM00000018695 XP_045179364.1 40 518 301 8 1 514 1 512 5.00E-103 383.3 6596 cellular organisms;Eukaryota;Opisthokonta;Metazoa;Eumetazoa;Bilateria;Protostomia;Spiralia;Lophotrochozoa;Mollusca;Bivalvia;Autobranchia;Heteroconchia;Euheterodonta;Imparidentia;Venerida;Veneroidea;Veneridae;Mercenaria;Mercenaria mercenaria
UdeM00000018696 6596 661.8 UdeM00000018696 XP_045191450.1 71.9 417 117 0 22 438 21 437 6.20E-187 661.8 6596 cellular organisms;Eukaryota;Opisthokonta;Metazoa;Eumetazoa;Bilateria;Protostomia;Spiralia;Lophotrochozoa;Mollusca;Bivalvia;Autobranchia;Heteroconchia;Euheterodonta;Imparidentia;Venerida;Veneroidea;Veneridae;Mercenaria;Mercenaria mercenaria
UdeM00000018697 6596 553.9 UdeM00000018697 XP_045178492.1 62.4 407 153 0 1 407 1 407 1.70E-154 553.9 6596 cellular organisms;Eukaryota;Opisthokonta;Metazoa;Eumetazoa;Bilateria;Protostomia;Spiralia;Lophotrochozoa;Mollusca;Bivalvia;Autobranchia;Heteroconchia;Euheterodonta;Imparidentia;Venerida;Veneroidea;Veneridae;Mercenaria;Mercenaria mercenaria
UdeM00000018698 1735272 84 UdeM00000018698 XP_041374001.1 29.1 179 124 1 13 188 367 545 3.30E-13 84 1735272 cellular organisms;Eukaryota;Opisthokonta;Metazoa;Eumetazoa;Bilateria;Protostomia;Spiralia;Lophotrochozoa;Mollusca;Gastropoda;Neomphalina;Neomphaloidea;Peltospiridae;Gigantopelta;Gigantopelta aegis
UdeM00000018699 1735272 83.6 UdeM00000018699 XP_041374001.1 30.2 205 119 7 21 214 367 558 5.00E-13 83.6 1735272 cellular organisms;Eukaryota;Opisthokonta;Metazoa;Eumetazoa;Bilateria;Protostomia;

Spiralia;Lophotrochozoa;Mollusca;Gastropoda;Neomphalina;Neomphaloidea;Peltospiridae;Gigantopelta;Gigantopelta aegis
UdeM00000018700 6596 282.7 UdeM00000018700 XP_045216733.1 45.4 317 157 5 129 442 209 512 8.00E-73 282.7 6596 cellular organisms;Eukaryota;Opisthokonta;Metazoa;Eumetazoa;Bilateria;Protostomia;Spiralia;Lophotrochozoa;Mollusca;Bivalvia;Autobranchia;Heteroconchia;Euheterodonta;Imparidentia;Venerida;Veneroidea;Veneridae;Mercenaria;Mercenaria mercenaria
UdeM00000018702 1735272 92.4 UdeM00000018702 XP_041374001.1 32.4 256 145 8 511 750 361 604 2.90E-15 92.4 1735272 cellular organisms;Eukaryota;Opisthokonta;Metazoa;Eumetazoa;Bilateria;Protostomia;Spiralia;Lophotrochozoa;Mollusca;Gastropoda;Neomphalina;Neomphaloidea;Peltospiridae;Gigantopelta;Gigantopelta aegis
UdeM00000018703 13397 72.8 UdeM00000018703 XP_041070135.1 38 108 57 4 131 233 129 231 1.50E-09 72.8 13397 cellular organisms;Eukaryota;Opisthokonta;Metazoa;Eumetazoa;Bilateria;Deuterostomia;Chordata;Craniata;Vertebrata;Gnathostomata;Chondrichthyes;Elasmobranchii;Selachii;Galeomorphii;Galeoidea;Lamniformes;Alopiidae;Lamninae;Carcharodon;Carcharodon carcharias
UdeM00000018704 6596 135.6 UdeM00000018704 XP_045181277.1 36 203 120 5 219 414 222 421 1.50E-28 135.6 6596 cellular organisms;Eukaryota;Opisthokonta;Metazoa;Eumetazoa;Bilateria;Protostomia;Spiralia;Lophotrochozoa;Mollusca;Bivalvia;Autobranchia;Heteroconchia;Euheterodonta;Imparidentia;Venerida;Veneroidea;Veneridae;Mercenaria;Mercenaria mercenaria
UdeM00000018706 6596 64.7 UdeM00000018706 XP_045179674.1 35.9 103 63 3 47 148 434 534 2.10E-07 64.7 6596 cellular organisms;Eukaryota;Opisthokonta;Metazoa;Eumetazoa;Bilateria;Protostomia;Spiralia;Lophotrochozoa;Mollusca;Bivalvia;Autobranchia;Heteroconchia;Euheterodonta;Imparidentia;Venerida;Veneroidea;Veneridae;Mercenaria;Mercenaria mercenaria
UdeM00000018707 6596 58.5 UdeM00000018707 XP_045189070.1 40.2 87 50 2 43 128 1 86 7.10E-06 58.5 6596 cellular organisms;Eukaryota;Opisthokonta;Metazoa;Eumetazoa;Bilateria;Protostomia;Spiralia;Lophotrochozoa;Mollusca;Bivalvia;Autobranchia;Heteroconchia;Euheterodonta;Imparidentia;Venerida;Veneroidea;Veneridae;Mercenaria;Mercenaria mercenaria
UdeM00000018708 6596 522.3 UdeM00000018708 XP_045177528.1 40.7 1024 425 32 119 1005 115 1093 1.30E-144 522.3 6596 cellular organisms;Eukaryota;Opisthokonta;Metazoa;Eumetazoa;Bilateria;Protostomia;Spiralia;Lophotrochozoa;Mollusca;Bivalvia;Autobranchia;Heteroconchia;Euheterodonta;Imparidentia;Venerida;Veneroidea;Veneridae;Mercenaria;Mercenaria mercenaria
UdeM00000018709 7515 90.1 UdeM00000018709 XP_026482378.1 21.7 474 289 14 1011 1447 113 541 2.50E-14 90.1 7515 cellular organisms;Eukaryota;Opisthokonta;Metazoa;Eumetazoa;Bilateria;Protostomia;Ecdysozoa;Panarthropoda;Arthropoda;Mandibulata;Pancrustacea;Hexapoda;Insecta;Dicondylia;Pterygota;Neoptera;Endopterygota;Siphonaptera;Pulicomorpha;Pulicoidea;Pulicidae;Archaeopsyllinae;Ctenocephalides;Ctenocephalides felis
UdeM00000018711 6565 58.2 UdeM00000018711 XP_022299727.1 37.8 82 47 2 1 80 46 125 5.80E-06 58.2 6565 cellular organisms;Eukaryota;Opisthokonta;Metazoa;Eumetazoa;Bilateria;Protostomia;

Spiralia;Lophotrochozoa;Mollusca;Bivalvia;Autobranchia;Pteriomorphia;Ostreida;Ostreoidea;Ostreidae;Crassostrea;Crassostrea virginica
UdeM00000018712 2607531 169.9 UdeM00000018712 XP_029635924.1 55.4 157 54 3 58 203 33 184 3.70E-39 169.9 2607531 cellular organisms;Eukaryota;Opisthokonta;Metazoa;Eumetazoa;Bilateria;Protostomia;Spiralia;Lophotrochozoa;Mollusca;Cephalopoda;Coleoidea;Octopodiformes;Octopoda;Incirrata;Octopodidae;Octopus;Octopus sinensis
UdeM00000018713 400727 1048.5 UdeM00000018713 XP_025080030.1 61.3 911 302 12 40 942 363 1230 5.10E-303 1048.5 400727 cellular organisms;Eukaryota;Opisthokonta;Metazoa;Eumetazoa;Bilateria;Protostomia;Spiralia;Lophotrochozoa;Mollusca;Gastropoda;Caenogastropoda;Architaenioglossa;Ampullarioidea;Ampullariidae;Pomacea;Pomacea canaliculata
UdeM00000018714 400727 1048.5 UdeM00000018714 XP_025080030.1 61.3 911 302 12 39 941 363 1230 5.10E-303 1048.5 400727 cellular organisms;Eukaryota;Opisthokonta;Metazoa;Eumetazoa;Bilateria;Protostomia;Spiralia;Lophotrochozoa;Mollusca;Gastropoda;Caenogastropoda;Architaenioglossa;Ampullarioidea;Ampullariidae;Pomacea;Pomacea canaliculata
UdeM00000018715 1529436 174.5 UdeM00000018715 XP_033095325.1 41.7 211 107 1 105 299 48 258 2.30E-40 174.5 1529436 cellular organisms;Eukaryota;Opisthokonta;Metazoa;Eumetazoa;Bilateria;Deuterostomia;Echinodermata;Pelmatozoa;Crinoidea;Articulata;Comatulida;Comatulidae;Comatulinae;Anneissia;Anneissia japonica
UdeM00000018716 6573 679.1 UdeM00000018716 XP_021363533.1 60.7 588 209 8 1 576 579 1156 5.80E-192 679.1 6573 cellular organisms;Eukaryota;Opisthokonta;Metazoa;Eumetazoa;Bilateria;Protostomia;Spiralia;Lophotrochozoa;Mollusca;Bivalvia;Autobranchia;Pteriomorphia;Pectinida;Pectinoidea;Pectinidae;Mizuhopecten;Mizuhopecten yessoensis
UdeM00000018717 6573 674.5 UdeM00000018717 XP_021363533.1 57.2 631 211 9 1 625 579 1156 1.50E-190 674.5 6573 cellular organisms;Eukaryota;Opisthokonta;Metazoa;Eumetazoa;Bilateria;Protostomia;Spiralia;Lophotrochozoa;Mollusca;Bivalvia;Autobranchia;Pteriomorphia;Pectinida;Pectinoidea;Pectinidae;Mizuhopecten;Mizuhopecten yessoensis
UdeM00000018718 2607531 469.5 UdeM00000018718 XP_029635924.1 59.9 397 146 6 162 552 86 475 5.70E-129 469.5 2607531 cellular organisms;Eukaryota;Opisthokonta;Metazoa;Eumetazoa;Bilateria;Protostomia;Spiralia;Lophotrochozoa;Mollusca;Cephalopoda;Coleoidea;Octopodiformes;Octopoda;Incirrata;Octopodidae;Octopus;Octopus sinensis
UdeM00000018720 1735272 144.8 UdeM00000018720 XP_041374691.1 46.8 154 82 0 1 154 85 238 9.60E-32 144.8 1735272 cellular organisms;Eukaryota;Opisthokonta;Metazoa;Eumetazoa;Bilateria;Protostomia;Spiralia;Lophotrochozoa;Mollusca;Gastropoda;Neomphalina;Neomphaloidea;Peltospiridae;Gigantopelta;Gigantopelta aegis
UdeM00000018719 1735272 200.3 UdeM00000018719 XP_041374691.1 44.1 238 131 2 3 239 2 238 2.90E-48 200.3 1735272 cellular organisms;Eukaryota;Opisthokonta;Metazoa;Eumetazoa;Bilateria;Protostomia;Spiralia;Lophotrochozoa;Mollusca;Gastropoda;Neomphalina;Neomphaloidea;Peltospiridae;Gigantopelta;Gigantopelta aegis

UdeM00000018721 6596 514.2 UdeM00000018721 XP_045179310.1 42.9 729
319 23 27 678 20 728 2.50E-142 514.2 6596 cellular
organisms;Eukaryota;Opisthokonta;Metazoa;Eumetazoa;Bilateria;Protostomia;
Spiralia;Lophotrochozoa;Mollusca;Bivalvia;Autobranchia;Heteroconchia;Euhe
terodonta;Imparidentia;Venerida;Veneroidea;Veneridae;Mercenaria;Mercenari
a mercenaria
UdeM00000018722 6596 581.6 UdeM00000018722 XP_045179013.1 76.4 415
91 6 11 419 22 435 1.00E-162 581.6 6596 cellular
organisms;Eukaryota;Opisthokonta;Metazoa;Eumetazoa;Bilateria;Protostomia;
Spiralia;Lophotrochozoa;Mollusca;Bivalvia;Autobranchia;Heteroconchia;Euhe
terodonta;Imparidentia;Venerida;Veneroidea;Veneridae;Mercenaria;Mercenari
a mercenaria
UdeM00000018723 6945 66.6 UdeM00000018723 XP_040065097.1 28.2 170
88 5 59 194 85 254 6.10E-08 66.6 6945 cellular
organisms;Eukaryota;Opisthokonta;Metazoa;Eumetazoa;Bilateria;Protostomia;
Ecdysozoa;Panarthropoda;Arthropoda;Chelicerata;Arachnida;Acari;Parasitifo
rmes;Ixodida;Ixodoidea;Ixodidae;Ixodinae;Ixodes;Ixodes scapularis
UdeM00000018724 6565 479.6 UdeM00000018724 XP_022320474.1 59.8 348
137 2 64 410 59 404 4.10E-132 479.6 6565 cellular
organisms;Eukaryota;Opisthokonta;Metazoa;Eumetazoa;Bilateria;Protostomia;
Spiralia;Lophotrochozoa;Mollusca;Bivalvia;Autobranchia;Pteriomorphia;Ostr
eida;Ostreoidea;Ostreidae;Crassostrea;Crassostrea virginica
UdeM00000018726 6596 314.7 UdeM00000018726 XP_045178771.1 81.6 179
33 0 1 179 1 179 7.60E-83 314.7 6596 cellular
organisms;Eukaryota;Opisthokonta;Metazoa;Eumetazoa;Bilateria;Protostomia;
Spiralia;Lophotrochozoa;Mollusca;Bivalvia;Autobranchia;Heteroconchia;Euhe
terodonta;Imparidentia;Venerida;Veneroidea;Veneridae;Mercenaria;Mercenari
a mercenaria
UdeM00000018727 6596 741.9 UdeM00000018727 XP_045178291.1 48.2 1094
431 33 1 1013 113 1151 1.10E-210 741.9 6596 cellular
organisms;Eukaryota;Opisthokonta;Metazoa;Eumetazoa;Bilateria;Protostomia;
Spiralia;Lophotrochozoa;Mollusca;Bivalvia;Autobranchia;Heteroconchia;Euhe
terodonta;Imparidentia;Venerida;Veneroidea;Veneridae;Mercenaria;Mercenari
a mercenaria
UdeM00000018728 1735272 171.4 UdeM00000018728 XP_041356145.1 53.6
140 64 1 33 172 48 186 1.10E-39 171.4 1735272
cellular
organisms;Eukaryota;Opisthokonta;Metazoa;Eumetazoa;Bilateria;Protostomia;
Spiralia;Lophotrochozoa;Mollusca;Gastropoda;Neomphalina;Neomphaloidea;Pel
tospiridae;Gigantopelta;Gigantopelta aegis
UdeM00000018729 114398 427.2 UdeM00000018729 XP_042903861.1 38.8
609 362 6 1 605 1 602 3.50E-116 427.2 114398
cellular
organisms;Eukaryota;Opisthokonta;Metazoa;Eumetazoa;Bilateria;Protostomia;
Ecdysozoa;Panarthropoda;Arthropoda;Chelicerata;Arachnida;Araneae;Araneomo
rphae;Entelegynae;Orbiculariae;Araneoidea;Theridiidae;Parasteatoda;Parast
eatoda tepidariorum
UdeM00000018730 1735272 167.5 UdeM00000018730 XP_041374781.1 43.3
245 116 5 35 269 97 328 2.30E-38 167.5 1735272
cellular
organisms;Eukaryota;Opisthokonta;Metazoa;Eumetazoa;Bilateria;Protostomia;
Spiralia;Lophotrochozoa;Mollusca;Gastropoda;Neomphalina;Neomphaloidea;Pel
tospiridae;Gigantopelta;Gigantopelta aegis

UdeM00000018733	6579	85.9	UdeM00000018733	XP_033761616.1	52.7	74
	34	1	2	75	167	239	2.50E-14	85.9	6579	cellular
organisms;Eukaryota;Opisthokonta;Metazoa;Eumetazoa;Bilateria;Protostomia;
Spiralia;Lophotrochozoa;Mollusca;Bivalvia;Autobranchia;Pteriomorphia;Pect
inida;Pectinoidea;Pectinidae;Pecten;Pecten maximus
UdeM00000018735	6565	89.4	UdeM00000018735	XP_022322655.1	47.4	95
	50	0	8	102	339	433	3.00E-15	89.4	6565	cellular
organisms;Eukaryota;Opisthokonta;Metazoa;Eumetazoa;Bilateria;Protostomia;
Spiralia;Lophotrochozoa;Mollusca;Bivalvia;Autobranchia;Pteriomorphia;Ostr
eida;Ostreoidea;Ostreidae;Crassostrea;Crassostrea virginica
UdeM00000018739	1735272	409.1	UdeM00000018739	XP_041374003.1	95.8
	213	9	0	1	213	1	213	3.50E-111	409.1	1735272
	cellular
organisms;Eukaryota;Opisthokonta;Metazoa;Eumetazoa;Bilateria;Protostomia;
Spiralia;Lophotrochozoa;Mollusca;Gastropoda;Neomphalina;Neomphaloidea;Pel
tospiridae;Gigantopelta;Gigantopelta aegis
UdeM00000018740	29159	849	UdeM00000018740	XP_011426045.1	68.4	602
	179	3	1	591	1	602	3.70E-243	849	29159	cellular
organisms;Eukaryota;Opisthokonta;Metazoa;Eumetazoa;Bilateria;Protostomia;
Spiralia;Lophotrochozoa;Mollusca;Bivalvia;Autobranchia;Pteriomorphia;Ostr
eida;Ostreoidea;Ostreidae;Crassostrea;Crassostrea gigas
UdeM00000018741	6596	609.8	UdeM00000018741	XP_045179322.1	45.1	809
	339	14	22	769	16	780	4.90E-171	609.8	6596	cellular
organisms;Eukaryota;Opisthokonta;Metazoa;Eumetazoa;Bilateria;Protostomia;
Spiralia;Lophotrochozoa;Mollusca;Bivalvia;Autobranchia;Heteroconchia;Euhe
terodonta;Imparidentia;Venerida;Veneroidea;Veneridae;Mercenaria;Mercenari
a mercenaria
UdeM00000018742	6596	1038.5	UdeM00000018742	XP_045178359.1	73.2
	657	172	3	1	656	1	654	3.60E-300	1038.5	6596
	cellular
organisms;Eukaryota;Opisthokonta;Metazoa;Eumetazoa;Bilateria;Protostomia;
Spiralia;Lophotrochozoa;Mollusca;Bivalvia;Autobranchia;Heteroconchia;Euhe
terodonta;Imparidentia;Venerida;Veneroidea;Veneridae;Mercenaria;Mercenari
a mercenaria
UdeM00000018743	6579	497.3	UdeM00000018743	XP_033728865.1	78	300
	66	0	1	300	3721	4020	1.40E-137	497.3	6579	cellular
organisms;Eukaryota;Opisthokonta;Metazoa;Eumetazoa;Bilateria;Protostomia;
Spiralia;Lophotrochozoa;Mollusca;Bivalvia;Autobranchia;Pteriomorphia;Pect
inida;Pectinoidea;Pectinidae;Pecten;Pecten maximus
UdeM00000018744	6596	6884.7	UdeM00000018744	XP_045178280.1	83.6
	4011	653	4	1	4010	1	4008	0.00E+00	6884.7	6596
	cellular
organisms;Eukaryota;Opisthokonta;Metazoa;Eumetazoa;Bilateria;Protostomia;
Spiralia;Lophotrochozoa;Mollusca;Bivalvia;Autobranchia;Heteroconchia;Euhe
terodonta;Imparidentia;Venerida;Veneroidea;Veneridae;Mercenaria;Mercenari
a mercenaria
UdeM00000018745	1735272	152.5	UdeM00000018745	XP_041374447.1	50.4
	139	69	0	6	144	5	143	4.10E-34	152.5	1735272
	cellular
organisms;Eukaryota;Opisthokonta;Metazoa;Eumetazoa;Bilateria;Protostomia;
Spiralia;Lophotrochozoa;Mollusca;Gastropoda;Neomphalina;Neomphaloidea;Pel
tospiridae;Gigantopelta;Gigantopelta aegis
UdeM00000018746	6565	264.2	UdeM00000018746	XP_022325420.1	42	283
	161	2	139	418	86	368	2.80E-67	264.2	6565	cellular

organisms;Eukaryota;Opisthokonta;Metazoa;Eumetazoa;Bilateria;Protostomia;Spiralia;Lophotrochozoa;Mollusca;Bivalvia;Autobranchia;Pteriomorphia;Ostreida;Ostreoidea;Ostreidae;Crassostrea;Crassostrea virginica
UdeM00000018747 6579 264.2 UdeM00000018747 XP_033742496.1 39.5 362 200 6 66 419 64 414 2.80E-67 264.2 6579 cellular organisms;Eukaryota;Opisthokonta;Metazoa;Eumetazoa;Bilateria;Protostomia;Spiralia;Lophotrochozoa;Mollusca;Bivalvia;Autobranchia;Pteriomorphia;Pectinida;Pectinoidea;Pectinidae;Pecten;Pecten maximus
UdeM00000018748 6579 265.4 UdeM00000018748 XP_033742496.1 39.5 362 200 6 66 419 64 414 1.30E-67 265.4 6579 cellular organisms;Eukaryota;Opisthokonta;Metazoa;Eumetazoa;Bilateria;Protostomia;Spiralia;Lophotrochozoa;Mollusca;Bivalvia;Autobranchia;Pteriomorphia;Pectinida;Pectinoidea;Pectinidae;Pecten;Pecten maximus
UdeM00000018749 37621 179.5 UdeM00000018749 XP_014677460.1 36.7 311 62 2 14 189 20 330 4.00E-42 179.5 37621 cellular organisms;Eukaryota;Opisthokonta;Metazoa;Eumetazoa;Bilateria;Protostomia;Ecdysozoa;Scalidophora;Priapulida;Priapulimorpha;Priapulimorphida;Priapulidae;Priapulus;Priapulus caudatus
UdeM00000018751 6565 279.3 UdeM00000018751 XP_022325420.1 44.9 285 153 2 202 482 84 368 9.60E-72 279.3 6565 cellular organisms;Eukaryota;Opisthokonta;Metazoa;Eumetazoa;Bilateria;Protostomia;Spiralia;Lophotrochozoa;Mollusca;Bivalvia;Autobranchia;Pteriomorphia;Ostreida;Ostreoidea;Ostreidae;Crassostrea;Crassostrea virginica
UdeM00000018752 6565 274.6 UdeM00000018752 XP_022325420.1 44 284 155 2 233 512 84 367 2.50E-70 274.6 6565 cellular organisms;Eukaryota;Opisthokonta;Metazoa;Eumetazoa;Bilateria;Protostomia;Spiralia;Lophotrochozoa;Mollusca;Bivalvia;Autobranchia;Pteriomorphia;Ostreida;Ostreoidea;Ostreidae;Crassostrea;Crassostrea virginica
UdeM00000018753 6579 133.7 UdeM00000018753 XP_033742496.1 45.8 142 66 1 89 219 100 241 3.70E-28 133.7 6579 cellular organisms;Eukaryota;Opisthokonta;Metazoa;Eumetazoa;Bilateria;Protostomia;Spiralia;Lophotrochozoa;Mollusca;Bivalvia;Autobranchia;Pteriomorphia;Pectinida;Pectinoidea;Pectinidae;Pecten;Pecten maximus
UdeM00000018754 6579 289.3 UdeM00000018754 XP_033742496.1 44.8 319 159 4 113 415 100 417 8.00E-75 289.3 6579 cellular organisms;Eukaryota;Opisthokonta;Metazoa;Eumetazoa;Bilateria;Protostomia;Spiralia;Lophotrochozoa;Mollusca;Bivalvia;Autobranchia;Pteriomorphia;Pectinida;Pectinoidea;Pectinidae;Pecten;Pecten maximus
UdeM00000018755 6565 278.1 UdeM00000018755 XP_022325420.1 43.3 289 160 2 161 445 84 372 2.00E-71 278.1 6565 cellular organisms;Eukaryota;Opisthokonta;Metazoa;Eumetazoa;Bilateria;Protostomia;Spiralia;Lophotrochozoa;Mollusca;Bivalvia;Autobranchia;Pteriomorphia;Ostreida;Ostreoidea;Ostreidae;Crassostrea;Crassostrea virginica
UdeM00000018756 29159 415.6 UdeM00000018756 XP_034331441.1 29.4 1039 604 25 97 1043 1 1001 1.80E-112 415.6 29159 cellular organisms;Eukaryota;Opisthokonta;Metazoa;Eumetazoa;Bilateria;Protostomia;Spiralia;Lophotrochozoa;Mollusca;Bivalvia;Autobranchia;Pteriomorphia;Ostreida;Ostreoidea;Ostreidae;Crassostrea;Crassostrea gigas
UdeM00000018757 6596 709.1 UdeM00000018757 XP_045179358.1 83.5 406 62 3 1 401 72 477 3.10E-201 709.1 6596 cellular organisms;Eukaryota;Opisthokonta;Metazoa;Eumetazoa;Bilateria;Protostomia;Spiralia;Lophotrochozoa;Mollusca;Bivalvia;Autobranchia;Heteroconchia;Euheterodonta;Imparidentia;Venerida;Veneroidea;Veneridae;Mercenaria;Mercenaria mercenaria

UdeM00000018758 37621 803.9 UdeM00000018758 XP_014679849.1 61 621
234 4 1 613 3 623 1.40E-229 803.9 37621 cellular
organisms;Eukaryota;Opisthokonta;Metazoa;Eumetazoa;Bilateria;Protostomia;
Ecdysozoa;Scalidophora;Priapulida;Priapulimorpha;Priapulimorphida;Priapul
idae;Priapulus;Priapulus caudatus
UdeM00000018759 37621 640.2 UdeM00000018759 XP_014679849.1 51.5 621
218 5 1 538 3 623 2.40E-180 640.2 37621 cellular
organisms;Eukaryota;Opisthokonta;Metazoa;Eumetazoa;Bilateria;Protostomia;
Ecdysozoa;Scalidophora;Priapulida;Priapulimorpha;Priapulimorphida;Priapul
idae;Priapulus;Priapulus caudatus
UdeM00000018760 37621 724.9 UdeM00000018760 XP_014679850.1 69.9 512
144 3 1 504 1 510 6.90E-206 724.9 37621 cellular
organisms;Eukaryota;Opisthokonta;Metazoa;Eumetazoa;Bilateria;Protostomia;
Ecdysozoa;Scalidophora;Priapulida;Priapulimorpha;Priapulimorphida;Priapul
idae;Priapulus;Priapulus caudatus
UdeM00000018761 6596 444.5 UdeM00000018761 XP_045177986.1 51 408
191 3 19 423 3 404 1.50E-121 444.5 6596 cellular
organisms;Eukaryota;Opisthokonta;Metazoa;Eumetazoa;Bilateria;Protostomia;
Spiralia;Lophotrochozoa;Mollusca;Bivalvia;Autobranchia;Heteroconchia;Euhe
terodonta;Imparidentia;Venerida;Veneroidea;Veneridae;Mercenaria;Mercenari
a mercenaria
UdeM00000018762 29159 355.5 UdeM00000018762 XP_011419561.2 41.7 424
235 5 1 416 1 420 9.10E-95 355.5 29159 cellular
organisms;Eukaryota;Opisthokonta;Metazoa;Eumetazoa;Bilateria;Protostomia;
Spiralia;Lophotrochozoa;Mollusca;Bivalvia;Autobranchia;Pteriomorphia;Ostr
eida;Ostreoidea;Ostreidae;Crassostrea;Crassostrea gigas
UdeM00000018763 6565 320.1 UdeM00000018763 XP_022322406.1 41.1 397
226 3 9 404 15 404 4.20E-84 320.1 6565 cellular
organisms;Eukaryota;Opisthokonta;Metazoa;Eumetazoa;Bilateria;Protostomia;
Spiralia;Lophotrochozoa;Mollusca;Bivalvia;Autobranchia;Pteriomorphia;Ostr
eida;Ostreoidea;Ostreidae;Crassostrea;Crassostrea virginica
UdeM00000018764 1735272 1504.2 UdeM00000018764 XP_041374427.1
61.1 1303 456 12 48 1346 1787 3042 0.00E+00 1504.2
1735272 cellular
organisms;Eukaryota;Opisthokonta;Metazoa;Eumetazoa;Bilateria;Protostomia;
Spiralia;Lophotrochozoa;Mollusca;Gastropoda;Neomphalina;Neomphaloidea;Pel
tospiridae;Gigantopelta;Gigantopelta aegis
UdeM00000018765 7741 287 UdeM00000018765 XP_019622111.1 51.3 275
132 2 5 278 1 274 2.70E-74 287 7741 cellular
organisms;Eukaryota;Opisthokonta;Metazoa;Eumetazoa;Bilateria;Deuterostomi
a;Chordata;Cephalochordata;Leptocardii;Amphioxiformes;Branchiostomidae;Br
anchiostoma;Branchiostoma belcheri
UdeM00000018766 6596 184.9 UdeM00000018766 XP_045178474.1 61.2 160
55 3 31 188 1632 1786 1.70E-43 184.9 6596 cellular
organisms;Eukaryota;Opisthokonta;Metazoa;Eumetazoa;Bilateria;Protostomia;
Spiralia;Lophotrochozoa;Mollusca;Bivalvia;Autobranchia;Heteroconchia;Euhe
terodonta;Imparidentia;Venerida;Veneroidea;Veneridae;Mercenaria;Mercenari
a mercenaria
UdeM00000018767 6596 1493 UdeM00000018767 XP_045178474.1 82.8 905
147 4 465 1365 713 1612 0.00E+00 1493 6596 cellular
organisms;Eukaryota;Opisthokonta;Metazoa;Eumetazoa;Bilateria;Protostomia;
Spiralia;Lophotrochozoa;Mollusca;Bivalvia;Autobranchia;Heteroconchia;Euhe
terodonta;Imparidentia;Venerida;Veneroidea;Veneridae;Mercenaria;Mercenari
a mercenaria

UdeM00000018768 6596 1185.2 UdeM00000018768 XP_045178474.1 86.4
691 93 1 13 703 23 712 0.00E+00 1185.2 6596
cellular
organisms;Eukaryota;Opisthokonta;Metazoa;Eumetazoa;Bilateria;Protostomia;
Spiralia;Lophotrochozoa;Mollusca;Bivalvia;Autobranchia;Heteroconchia;Euhe
terodonta;Imparidentia;Venerida;Veneroidea;Veneridae;Mercenaria;Mercenari
a mercenaria
UdeM00000018769 6573 180.3 UdeM00000018769 XP_021366307.1 96.7 90
3 0 1 90 1 90 1.10E-42 180.3 6573 cellular
organisms;Eukaryota;Opisthokonta;Metazoa;Eumetazoa;Bilateria;Protostomia;
Spiralia;Lophotrochozoa;Mollusca;Bivalvia;Autobranchia;Pteriomorphia;Pect
inida;Pectinoidea;Pectinidae;Mizuhopecten;Mizuhopecten yessoensis
UdeM00000018770 6596 69.7 UdeM00000018770 XP_045178533.1 68 50
13 1 487 533 638 687 1.30E-08 69.7 6596 cellular
organisms;Eukaryota;Opisthokonta;Metazoa;Eumetazoa;Bilateria;Protostomia;
Spiralia;Lophotrochozoa;Mollusca;Bivalvia;Autobranchia;Heteroconchia;Euhe
terodonta;Imparidentia;Venerida;Veneroidea;Veneridae;Mercenaria;Mercenari
a mercenaria
UdeM00000018771 6596 109 UdeM00000018771 XP_045178533.1 67.8 87
26 2 1 87 1 85 4.90E-20 109 6596 cellular
organisms;Eukaryota;Opisthokonta;Metazoa;Eumetazoa;Bilateria;Protostomia;
Spiralia;Lophotrochozoa;Mollusca;Bivalvia;Autobranchia;Heteroconchia;Euhe
terodonta;Imparidentia;Venerida;Veneroidea;Veneridae;Mercenaria;Mercenari
a mercenaria
UdeM00000018772 6579 118.6 UdeM00000018772 XP_033729096.1 57.9 107
39 2 6 112 5 105 5.10E-24 118.6 6579 cellular
organisms;Eukaryota;Opisthokonta;Metazoa;Eumetazoa;Bilateria;Protostomia;
Spiralia;Lophotrochozoa;Mollusca;Bivalvia;Autobranchia;Pteriomorphia;Pect
inida;Pectinoidea;Pectinidae;Pecten;Pecten maximus
UdeM00000018774 6596 458.4 UdeM00000018774 XP_045179542.1 63.8 345
122 2 16 359 17 359 8.50E-126 458.4 6596 cellular
organisms;Eukaryota;Opisthokonta;Metazoa;Eumetazoa;Bilateria;Protostomia;
Spiralia;Lophotrochozoa;Mollusca;Bivalvia;Autobranchia;Heteroconchia;Euhe
terodonta;Imparidentia;Venerida;Veneroidea;Veneridae;Mercenaria;Mercenari
a mercenaria
UdeM00000018776 6596 176 UdeM00000018776 XP_045179542.1 91.8 85
7 0 39 123 21 105 2.90E-41 176 6596 cellular
organisms;Eukaryota;Opisthokonta;Metazoa;Eumetazoa;Bilateria;Protostomia;
Spiralia;Lophotrochozoa;Mollusca;Bivalvia;Autobranchia;Heteroconchia;Euhe
terodonta;Imparidentia;Venerida;Veneroidea;Veneridae;Mercenaria;Mercenari
a mercenaria
UdeM00000018777 6596 203.8 UdeM00000018777 XP_045179542.1 49.8 207
101 2 2 207 155 359 2.20E-49 203.8 6596 cellular
organisms;Eukaryota;Opisthokonta;Metazoa;Eumetazoa;Bilateria;Protostomia;
Spiralia;Lophotrochozoa;Mollusca;Bivalvia;Autobranchia;Heteroconchia;Euhe
terodonta;Imparidentia;Venerida;Veneroidea;Veneridae;Mercenaria;Mercenari
a mercenaria
UdeM00000018779 6596 300.8 UdeM00000018779 XP_045179897.1 73.9 188
49 0 1 188 1 188 1.20E-78 300.8 6596 cellular
organisms;Eukaryota;Opisthokonta;Metazoa;Eumetazoa;Bilateria;Protostomia;
Spiralia;Lophotrochozoa;Mollusca;Bivalvia;Autobranchia;Heteroconchia;Euhe
terodonta;Imparidentia;Venerida;Veneroidea;Veneridae;Mercenaria;Mercenari
a mercenaria

UdeM00000018780	6579	367.5	UdeM00000018780	XP_033728412.1	50.8	366
	172	5	1	363	1	361	2.00E-98	367.5	6579	cellular
organisms;Eukaryota;Opisthokonta;Metazoa;Eumetazoa;Bilateria;Protostomia;
Spiralia;Lophotrochozoa;Mollusca;Bivalvia;Autobranchia;Pteriomorphia;Pect
inida;Pectinoidea;Pectinidae;Pecten;Pecten maximus
UdeM00000018781	46514	112.8	UdeM00000018781	XP_038065360.1	42.5	120
	69	0	4	123	626	745	4.50E-22	112.8	46514	cellular
organisms;Eukaryota;Opisthokonta;Metazoa;Eumetazoa;Bilateria;Deuterostomi
a;Echinodermata;Eleutherozoa;Asterozoa;Asteroidea;Valvatacea;Valvatida;As
terinidae;Patiria;Patiria miniata
UdeM00000018782	114398	137.5	UdeM00000018782	XP_042910816.1	43.8
	144	81	0	19	162	25	168	1.50E-29	137.5	114398
	cellular
organisms;Eukaryota;Opisthokonta;Metazoa;Eumetazoa;Bilateria;Protostomia;
Ecdysozoa;Panarthropoda;Arthropoda;Chelicerata;Arachnida;Araneae;Araneomo
rphae;Entelegynae;Orbiculariae;Araneoidea;Theridiidae;Parasteatoda;Parast
eatoda tepidariorum
UdeM00000018783	29159	115.2	UdeM00000018783	XP_011451530.1	47.2	108
	56	1	15	121	88	195	7.20E-23	115.2	29159	cellular
organisms;Eukaryota;Opisthokonta;Metazoa;Eumetazoa;Bilateria;Protostomia;
Spiralia;Lophotrochozoa;Mollusca;Bivalvia;Autobranchia;Pteriomorphia;Ostr
eida;Ostreoidea;Ostreidae;Crassostrea;Crassostrea gigas
UdeM00000018784	29159	527.3	UdeM00000018784	XP_034303860.1	53.9	458
	173	3	1	422	293	748	1.80E-146	527.3	29159	cellular
organisms;Eukaryota;Opisthokonta;Metazoa;Eumetazoa;Bilateria;Protostomia;
Spiralia;Lophotrochozoa;Mollusca;Bivalvia;Autobranchia;Pteriomorphia;Ostr
eida;Ostreoidea;Ostreidae;Crassostrea;Crassostrea gigas
UdeM00000018785	29159	131.3	UdeM00000018785	XP_034303860.1	43.5	200
	96	7	5	199	21	208	1.70E-27	131.3	29159	cellular
organisms;Eukaryota;Opisthokonta;Metazoa;Eumetazoa;Bilateria;Protostomia;
Spiralia;Lophotrochozoa;Mollusca;Bivalvia;Autobranchia;Pteriomorphia;Ostr
eida;Ostreoidea;Ostreidae;Crassostrea;Crassostrea gigas
UdeM00000018786	6573	198.4	UdeM00000018786	XP_021359218.1	60.7	150
	55	2	1	147	445	593	6.60E-48	198.4	6573	cellular
organisms;Eukaryota;Opisthokonta;Metazoa;Eumetazoa;Bilateria;Protostomia;
Spiralia;Lophotrochozoa;Mollusca;Bivalvia;Autobranchia;Pteriomorphia;Pect
inida;Pectinoidea;Pectinidae;Mizuhopecten;Mizuhopecten yessoensis
UdeM00000018787	5762	99.8	UdeM00000018787	XP_002670566.1	29.4	255
	163	7	32	270	80	333	6.60E-18	99.8	5762	cellular
organisms;Eukaryota;Discoba;Heterolobosea;Tetramitia;Eutetramitia;Vahlkam
pfiidae;Naegleria;Naegleria gruberi
UdeM00000018788	6596	403.3	UdeM00000018788	XP_045216525.1	41.1	604
	315	22	110	701	110	684	6.70E-109	403.3	6596	cellular
organisms;Eukaryota;Opisthokonta;Metazoa;Eumetazoa;Bilateria;Protostomia;
Spiralia;Lophotrochozoa;Mollusca;Bivalvia;Autobranchia;Heteroconchia;Euhe
terodonta;Imparidentia;Venerida;Veneroidea;Veneridae;Mercenaria;Mercenari
a mercenaria
UdeM00000018789	6596	458.4	UdeM00000018789	XP_045216525.1	42.4	651
	330	21	119	747	60	687	1.80E-125	458.4	6596	cellular
organisms;Eukaryota;Opisthokonta;Metazoa;Eumetazoa;Bilateria;Protostomia;
Spiralia;Lophotrochozoa;Mollusca;Bivalvia;Autobranchia;Heteroconchia;Euhe
terodonta;Imparidentia;Venerida;Veneroidea;Veneridae;Mercenaria;Mercenari
a mercenaria

UdeM00000018790 6596 471.5 UdeM00000018790 XP_045216525.1 42.6 707
357 25 26 708 6 687 2.00E-129 471.5 6596 cellular
organisms;Eukaryota;Opisthokonta;Metazoa;Eumetazoa;Bilateria;Protostomia;
Spiralia;Lophotrochozoa;Mollusca;Bivalvia;Autobranchia;Heteroconchia;Euhe
terodonta;Imparidentia;Venerida;Veneroidea;Veneridae;Mercenaria;Mercenari
a mercenaria
UdeM00000018791 6596 424.5 UdeM00000018791 XP_045216525.1 42.5 643
319 27 62 687 72 680 2.80E-115 424.5 6596 cellular
organisms;Eukaryota;Opisthokonta;Metazoa;Eumetazoa;Bilateria;Protostomia;
Spiralia;Lophotrochozoa;Mollusca;Bivalvia;Autobranchia;Heteroconchia;Euhe
terodonta;Imparidentia;Venerida;Veneroidea;Veneridae;Mercenaria;Mercenari
a mercenaria
UdeM00000018792 6596 437.2 UdeM00000018792 XP_045216525.1 42 643
322 26 61 686 72 680 4.10E-119 437.2 6596 cellular
organisms;Eukaryota;Opisthokonta;Metazoa;Eumetazoa;Bilateria;Protostomia;
Spiralia;Lophotrochozoa;Mollusca;Bivalvia;Autobranchia;Heteroconchia;Euhe
terodonta;Imparidentia;Venerida;Veneroidea;Veneridae;Mercenaria;Mercenari
a mercenaria
UdeM00000044341 77166 68.6 UdeM00000044341 XP_019769193.1 46.6 73
31 1 2 74 190 254 4.90E-09 68.6 77166 cellular
organisms;Eukaryota;Opisthokonta;Metazoa;Eumetazoa;Bilateria;Protostomia;
Ecdysozoa;Panarthropoda;Arthropoda;Mandibulata;Pancrustacea;Hexapoda;Inse
cta;Dicondylia;Pterygota;Neoptera;Endopterygota;Coleoptera;Polyphaga;Cucu
jiformia;Curculionoidea;Curculionidae;Scolytinae;Dendroctonus;Dendroctonu
s ponderosae
UdeM00000044342 37621 142.9 UdeM00000044342 XP_014679100.1 37.9 232
130 8 768 994 78 300 2.20E-30 142.9 37621 cellular
organisms;Eukaryota;Opisthokonta;Metazoa;Eumetazoa;Bilateria;Protostomia;
Ecdysozoa;Scalidophora;Priapulida;Priapulimorpha;Priapulimorphida;Priapul
idae;Priapulus;Priapulus caudatus
UdeM00000042523 6596 637.5 UdeM00000042523 XP_045171884.1 55 729
249 15 2 670 11 720 1.90E-179 637.5 6596 cellular
organisms;Eukaryota;Opisthokonta;Metazoa;Eumetazoa;Bilateria;Protostomia;
Spiralia;Lophotrochozoa;Mollusca;Bivalvia;Autobranchia;Heteroconchia;Euhe
terodonta;Imparidentia;Venerida;Veneroidea;Veneridae;Mercenaria;Mercenari
a mercenaria
UdeM00000042525 6579 238.8 UdeM00000042525 XP_033743128.1 35.5 422
258 4 4 412 7 427 1.20E-59 238.8 6579 cellular
organisms;Eukaryota;Opisthokonta;Metazoa;Eumetazoa;Bilateria;Protostomia;
Spiralia;Lophotrochozoa;Mollusca;Bivalvia;Autobranchia;Pteriomorphia;Pect
inida;Pectinoidea;Pectinidae;Pecten;Pecten maximus
UdeM00000042526 6596 172.9 UdeM00000042526 XP_045165713.1 41.5 212
123 1 16 226 4 215 4.70E-40 172.9 6596 cellular
organisms;Eukaryota;Opisthokonta;Metazoa;Eumetazoa;Bilateria;Protostomia;
Spiralia;Lophotrochozoa;Mollusca;Bivalvia;Autobranchia;Heteroconchia;Euhe
terodonta;Imparidentia;Venerida;Veneroidea;Veneridae;Mercenaria;Mercenari
a mercenaria
UdeM00000042527 6596 82 UdeM00000042527 XP_045182758.1 29.2 250
155 8 2 237 137 378 1.10E-12 82 6596 cellular
organisms;Eukaryota;Opisthokonta;Metazoa;Eumetazoa;Bilateria;Protostomia;
Spiralia;Lophotrochozoa;Mollusca;Bivalvia;Autobranchia;Heteroconchia;Euhe
terodonta;Imparidentia;Venerida;Veneroidea;Veneridae;Mercenaria;Mercenari
a mercenaria

UdeM00000042528	6579	232.3	UdeM00000042528	XP_033743128.1	35	397
235	2	5	387	29	416	1.10E-57	232.3	6579	cellular organisms;Eukaryota;Opisthokonta;Metazoa;Eumetazoa;Bilateria;Protostomia;Spiralia;Lophotrochozoa;Mollusca;Bivalvia;Autobranchia;Pteriomorphia;Pectinida;Pectinoidea;Pectinidae;Pecten;Pecten maximus
UdeM00000042529	6596	60.1	UdeM00000042529	XP_045165062.1	37.3	75
47	0	21	95	287	361	2.00E-06	60.1	6596	cellular organisms;Eukaryota;Opisthokonta;Metazoa;Eumetazoa;Bilateria;Protostomia;Spiralia;Lophotrochozoa;Mollusca;Bivalvia;Autobranchia;Heteroconchia;Euheterodonta;Imparidentia;Venerida;Veneroidea;Veneridae;Mercenaria;Mercenaria mercenaria
UdeM00000042530	6573	160.2	UdeM00000042530	XP_021372444.1	35.3	235
152	0	14	248	20	254	4.50E-36	160.2	6573	cellular organisms;Eukaryota;Opisthokonta;Metazoa;Eumetazoa;Bilateria;Protostomia;Spiralia;Lophotrochozoa;Mollusca;Bivalvia;Autobranchia;Pteriomorphia;Pectinida;Pectinoidea;Pectinidae;Mizuhopecten;Mizuhopecten yessoensis
UdeM00000042531	6579	226.5	UdeM00000042531	XP_033743128.1	34.7	401
256	2	1	396	17	416	6.10E-56	226.5	6579	cellular organisms;Eukaryota;Opisthokonta;Metazoa;Eumetazoa;Bilateria;Protostomia;Spiralia;Lophotrochozoa;Mollusca;Bivalvia;Autobranchia;Pteriomorphia;Pectinida;Pectinoidea;Pectinidae;Pecten;Pecten maximus
UdeM00000042532	6565	95.9	UdeM00000042532	XP_022315793.1	50.5	99
47	1	8	104	2	100	3.30E-17	95.9	6565	cellular organisms;Eukaryota;Opisthokonta;Metazoa;Eumetazoa;Bilateria;Protostomia;Spiralia;Lophotrochozoa;Mollusca;Bivalvia;Autobranchia;Pteriomorphia;Ostreida;Ostreoidea;Ostreidae;Crassostrea;Crassostrea virginica
UdeM00000042533	6565	96.3	UdeM00000042533	XP_022315793.1	50.5	99
47	1	4	100	2	100	2.40E-17	96.3	6565	cellular organisms;Eukaryota;Opisthokonta;Metazoa;Eumetazoa;Bilateria;Protostomia;Spiralia;Lophotrochozoa;Mollusca;Bivalvia;Autobranchia;Pteriomorphia;Ostreida;Ostreoidea;Ostreidae;Crassostrea;Crassostrea virginica
UdeM00000042534	6596	107.8	UdeM00000042534	XP_045187622.1	29.9	281
153	7	1	257	132	392	2.10E-20	107.8	6596	cellular organisms;Eukaryota;Opisthokonta;Metazoa;Eumetazoa;Bilateria;Protostomia;Spiralia;Lophotrochozoa;Mollusca;Bivalvia;Autobranchia;Heteroconchia;Euheterodonta;Imparidentia;Venerida;Veneroidea;Veneridae;Mercenaria;Mercenaria mercenaria
UdeM00000042535	6596	131.3	UdeM00000042535	XP_045165713.1	42.9	156
86	1	7	162	3	155	1.20E-27	131.3	6596	cellular organisms;Eukaryota;Opisthokonta;Metazoa;Eumetazoa;Bilateria;Protostomia;Spiralia;Lophotrochozoa;Mollusca;Bivalvia;Autobranchia;Heteroconchia;Euheterodonta;Imparidentia;Venerida;Veneroidea;Veneridae;Mercenaria;Mercenaria mercenaria
UdeM00000042536	6573	92.8	UdeM00000042536	XP_021372444.1	60.3	73
29	0	103	175	339	411	4.60E-16	92.8	6573	cellular organisms;Eukaryota;Opisthokonta;Metazoa;Eumetazoa;Bilateria;Protostomia;Spiralia;Lophotrochozoa;Mollusca;Bivalvia;Autobranchia;Pteriomorphia;Pectinida;Pectinoidea;Pectinidae;Mizuhopecten;Mizuhopecten yessoensis
UdeM00000042538	6579	176.8	UdeM00000042538	XP_033743128.1	32.3	387
225	2	20	371	33	417	5.30E-41	176.8	6579	cellular organisms;Eukaryota;Opisthokonta;Metazoa;Eumetazoa;Bilateria;Protostomia;Spiralia;Lophotrochozoa;Mollusca;Bivalvia;Autobranchia;Pteriomorphia;Pectinida;Pectinoidea;Pectinidae;Pecten;Pecten maximus

UdeM00000042539 6596 222.6 UdeM00000042539 XP_045165713.1 34.4 398
227 5 18 398 7 387 8.90E-55 222.6 6596 cellular
organisms;Eukaryota;Opisthokonta;Metazoa;Eumetazoa;Bilateria;Protostomia;
Spiralia;Lophotrochozoa;Mollusca;Bivalvia;Autobranchia;Heteroconchia;Euhe
terodonta;Imparidentia;Venerida;Veneroidea;Veneridae;Mercenaria;Mercenari
a mercenaria
UdeM00000042540 6579 91.7 UdeM00000042540 XP_033743128.1 46.7 105
55 1 94 198 324 427 1.20E-15 91.7 6579 cellular
organisms;Eukaryota;Opisthokonta;Metazoa;Eumetazoa;Bilateria;Protostomia;
Spiralia;Lophotrochozoa;Mollusca;Bivalvia;Autobranchia;Pteriomorphia;Pect
inida;Pectinoidea;Pectinidae;Pecten;Pecten maximus
UdeM00000042542 6579 120.9 UdeM00000042542 XP_033743128.1 34.3 268
162 4 1 255 154 420 2.60E-24 120.9 6579 cellular
organisms;Eukaryota;Opisthokonta;Metazoa;Eumetazoa;Bilateria;Protostomia;
Spiralia;Lophotrochozoa;Mollusca;Bivalvia;Autobranchia;Pteriomorphia;Pect
inida;Pectinoidea;Pectinidae;Pecten;Pecten maximus
UdeM00000042543 6596 166.8 UdeM00000042543 XP_045187622.1 31.6 348
216 5 15 348 8 347 5.10E-38 166.8 6596 cellular
organisms;Eukaryota;Opisthokonta;Metazoa;Eumetazoa;Bilateria;Protostomia;
Spiralia;Lophotrochozoa;Mollusca;Bivalvia;Autobranchia;Heteroconchia;Euhe
terodonta;Imparidentia;Venerida;Veneroidea;Veneridae;Mercenaria;Mercenari
a mercenaria
UdeM00000042548 6579 162.5 UdeM00000042548 XP_033743128.1 36.4 220
140 0 14 233 29 248 7.50E-37 162.5 6579 cellular
organisms;Eukaryota;Opisthokonta;Metazoa;Eumetazoa;Bilateria;Protostomia;
Spiralia;Lophotrochozoa;Mollusca;Bivalvia;Autobranchia;Pteriomorphia;Pect
inida;Pectinoidea;Pectinidae;Pecten;Pecten maximus
UdeM00000042550 6579 65.5 UdeM00000042550 XP_033743128.1 40 75
45 0 3 77 166 240 7.70E-08 65.5 6579 cellular
organisms;Eukaryota;Opisthokonta;Metazoa;Eumetazoa;Bilateria;Protostomia;
Spiralia;Lophotrochozoa;Mollusca;Bivalvia;Autobranchia;Pteriomorphia;Pect
inida;Pectinoidea;Pectinidae;Pecten;Pecten maximus
UdeM00000042551 6579 220.7 UdeM00000042551 XP_033743128.1 33.7 395
243 2 14 396 29 416 3.30E-54 220.7 6579 cellular
organisms;Eukaryota;Opisthokonta;Metazoa;Eumetazoa;Bilateria;Protostomia;
Spiralia;Lophotrochozoa;Mollusca;Bivalvia;Autobranchia;Pteriomorphia;Pect
inida;Pectinoidea;Pectinidae;Pecten;Pecten maximus
UdeM00000042552 6596 177.6 UdeM00000042552 XP_045165713.1 31.9 354
207 6 1 337 62 398 2.70E-41 177.6 6596 cellular
organisms;Eukaryota;Opisthokonta;Metazoa;Eumetazoa;Bilateria;Protostomia;
Spiralia;Lophotrochozoa;Mollusca;Bivalvia;Autobranchia;Heteroconchia;Euhe
terodonta;Imparidentia;Venerida;Veneroidea;Veneridae;Mercenaria;Mercenari
a mercenaria
UdeM00000042553 6596 207.6 UdeM00000042553 XP_045165713.1 33.3 405
223 4 15 393 3 386 3.00E-50 207.6 6596 cellular
organisms;Eukaryota;Opisthokonta;Metazoa;Eumetazoa;Bilateria;Protostomia;
Spiralia;Lophotrochozoa;Mollusca;Bivalvia;Autobranchia;Heteroconchia;Euhe
terodonta;Imparidentia;Venerida;Veneroidea;Veneridae;Mercenaria;Mercenari
a mercenaria
UdeM00000042554 6579 225.7 UdeM00000042554 XP_033743128.1 36.3 408
240 6 2 392 12 416 1.00E-55 225.7 6579 cellular
organisms;Eukaryota;Opisthokonta;Metazoa;Eumetazoa;Bilateria;Protostomia;
Spiralia;Lophotrochozoa;Mollusca;Bivalvia;Autobranchia;Pteriomorphia;Pect
inida;Pectinoidea;Pectinidae;Pecten;Pecten maximus

UdeM00000042555 6579 217.2 UdeM00000042555 XP_033743128.1 35.4 407
248 5 2 395 12 416 3.70E-53 217.2 6579 cellular
organisms;Eukaryota;Opisthokonta;Metazoa;Eumetazoa;Bilateria;Protostomia;
Spiralia;Lophotrochozoa;Mollusca;Bivalvia;Autobranchia;Pteriomorphia;Pect
inida;Pectinoidea;Pectinidae;Pecten;Pecten maximus
UdeM00000042556 6596 1030.4 UdeM00000042556 XP_045171157.1 48.6
1168 255 8 1 1166 28 852 1.90E-297 1030.4 6596
cellular
organisms;Eukaryota;Opisthokonta;Metazoa;Eumetazoa;Bilateria;Protostomia;
Spiralia;Lophotrochozoa;Mollusca;Bivalvia;Autobranchia;Heteroconchia;Euhe
terodonta;Imparidentia;Venerida;Veneroidea;Veneridae;Mercenaria;Mercenari
a mercenaria
UdeM00000042557 6596 1035 UdeM00000042557 XP_045171157.1 48.7 1167
255 7 1 1165 28 852 7.90E-299 1035 6596 cellular
organisms;Eukaryota;Opisthokonta;Metazoa;Eumetazoa;Bilateria;Protostomia;
Spiralia;Lophotrochozoa;Mollusca;Bivalvia;Autobranchia;Heteroconchia;Euhe
terodonta;Imparidentia;Venerida;Veneroidea;Veneridae;Mercenaria;Mercenari
a mercenaria
UdeM00000042558 6596 310.1 UdeM00000042558 XP_045171553.1 29 1053
630 26 12 1002 88 1084 1.10E-80 310.1 6596 cellular
organisms;Eukaryota;Opisthokonta;Metazoa;Eumetazoa;Bilateria;Protostomia;
Spiralia;Lophotrochozoa;Mollusca;Bivalvia;Autobranchia;Heteroconchia;Euhe
terodonta;Imparidentia;Venerida;Veneroidea;Veneridae;Mercenaria;Mercenari
a mercenaria
UdeM00000042559 6596 300.4 UdeM00000042559 XP_045171692.1 50.9 273
133 1 1 273 1 272 2.90E-78 300.4 6596 cellular
organisms;Eukaryota;Opisthokonta;Metazoa;Eumetazoa;Bilateria;Protostomia;
Spiralia;Lophotrochozoa;Mollusca;Bivalvia;Autobranchia;Heteroconchia;Euhe
terodonta;Imparidentia;Venerida;Veneroidea;Veneridae;Mercenaria;Mercenari
a mercenaria
UdeM00000042560 6565 634.4 UdeM00000042560 XP_022339184.1 80.2 388
77 0 26 413 28 415 1.00E-178 634.4 6565 cellular
organisms;Eukaryota;Opisthokonta;Metazoa;Eumetazoa;Bilateria;Protostomia;
Spiralia;Lophotrochozoa;Mollusca;Bivalvia;Autobranchia;Pteriomorphia;Ostr
eida;Ostreoidea;Ostreidae;Crassostrea;Crassostrea virginica
UdeM00000042561 29159 399.1 UdeM00000042561 XP_011455039.2 40.8 493
276 8 1 486 41 524 8.50E-108 399.1 29159 cellular
organisms;Eukaryota;Opisthokonta;Metazoa;Eumetazoa;Bilateria;Protostomia;
Spiralia;Lophotrochozoa;Mollusca;Bivalvia;Autobranchia;Pteriomorphia;Ostr
eida;Ostreoidea;Ostreidae;Crassostrea;Crassostrea gigas
UdeM00000042562 6573 407.1 UdeM00000042562 XP_021340121.1 51.1 378
180 3 73 446 326 702 2.90E-110 407.1 6573 cellular
organisms;Eukaryota;Opisthokonta;Metazoa;Eumetazoa;Bilateria;Protostomia;
Spiralia;Lophotrochozoa;Mollusca;Bivalvia;Autobranchia;Pteriomorphia;Pect
inida;Pectinoidea;Pectinidae;Mizuhopecten;Mizuhopecten yessoensis
UdeM00000042563 6573 386.7 UdeM00000042563 XP_021363050.1 48.5 379
188 4 71 444 322 698 4.00E-104 386.7 6573 cellular
organisms;Eukaryota;Opisthokonta;Metazoa;Eumetazoa;Bilateria;Protostomia;
Spiralia;Lophotrochozoa;Mollusca;Bivalvia;Autobranchia;Pteriomorphia;Pect
inida;Pectinoidea;Pectinidae;Mizuhopecten;Mizuhopecten yessoensis
UdeM00000042564 29159 165.6 UdeM00000042564 XP_011435143.2 33.7 270
168 4 2 264 117 382 8.60E-38 165.6 29159 cellular
organisms;Eukaryota;Opisthokonta;Metazoa;Eumetazoa;Bilateria;Protostomia;

Spiralia;Lophotrochozoa;Mollusca;Bivalvia;Autobranchia;Pteriomorphia;Ostreida;Ostreoidea;Ostreidae;Crassostrea;Crassostrea gigas
UdeM00000042566 6596 148.7 UdeM00000042566 XP_045202146.1 44.9 147 79 1 63 209 76 220 8.50E-33 148.7 6596 cellular organisms;Eukaryota;Opisthokonta;Metazoa;Eumetazoa;Bilateria;Protostomia;Spiralia;Lophotrochozoa;Mollusca;Bivalvia;Autobranchia;Heteroconchia;Euheterodonta;Imparidentia;Venerida;Veneroidea;Veneridae;Mercenaria;Mercenaria mercenaria
UdeM00000042565 6596 140.2 UdeM00000042565 XP_045202146.1 46.3 134 70 1 63 196 76 207 2.90E-30 140.2 6596 cellular organisms;Eukaryota;Opisthokonta;Metazoa;Eumetazoa;Bilateria;Protostomia;Spiralia;Lophotrochozoa;Mollusca;Bivalvia;Autobranchia;Heteroconchia;Euheterodonta;Imparidentia;Venerida;Veneroidea;Veneridae;Mercenaria;Mercenaria mercenaria
UdeM00000042567 6579 231.9 UdeM00000042567 XP_033751112.1 39.9 301 158 3 74 354 67 364 1.30E-57 231.9 6579 cellular organisms;Eukaryota;Opisthokonta;Metazoa;Eumetazoa;Bilateria;Protostomia;Spiralia;Lophotrochozoa;Mollusca;Bivalvia;Autobranchia;Pteriomorphia;Pectinida;Pectinoidea;Pectinidae;Pecten;Pecten maximus
UdeM00000042568 6526 173.3 UdeM00000042568 XP_013073210.1 35.9 256 161 3 57 312 105 357 4.90E-40 173.3 6526 cellular organisms;Eukaryota;Opisthokonta;Metazoa;Eumetazoa;Bilateria;Protostomia;Spiralia;Lophotrochozoa;Mollusca;Gastropoda;Heterobranchia;Euthyneura;Panpulmonata;Hygrophila;Lymnaeoidea;Planorbidae;Biomphalaria;Biomphalaria glabrata
UdeM00000042571 6596 295.8 UdeM00000042571 XP_045172292.1 52.2 270 124 1 20 289 10 274 5.90E-77 295.8 6596 cellular organisms;Eukaryota;Opisthokonta;Metazoa;Eumetazoa;Bilateria;Protostomia;Spiralia;Lophotrochozoa;Mollusca;Bivalvia;Autobranchia;Heteroconchia;Euheterodonta;Imparidentia;Venerida;Veneroidea;Veneridae;Mercenaria;Mercenaria mercenaria
UdeM00000042572 6596 131.3 UdeM00000042572 XP_045172978.1 74.7 79 20 0 46 124 179 257 8.60E-28 131.3 6596 cellular organisms;Eukaryota;Opisthokonta;Metazoa;Eumetazoa;Bilateria;Protostomia;Spiralia;Lophotrochozoa;Mollusca;Bivalvia;Autobranchia;Heteroconchia;Euheterodonta;Imparidentia;Venerida;Veneroidea;Veneridae;Mercenaria;Mercenaria mercenaria
UdeM00000042573 6596 726.9 UdeM00000042573 XP_045172978.1 55.4 673 269 13 46 710 179 828 2.60E-206 726.9 6596 cellular organisms;Eukaryota;Opisthokonta;Metazoa;Eumetazoa;Bilateria;Protostomia;Spiralia;Lophotrochozoa;Mollusca;Bivalvia;Autobranchia;Heteroconchia;Euheterodonta;Imparidentia;Venerida;Veneroidea;Veneridae;Mercenaria;Mercenaria mercenaria
UdeM00000042576 6579 419.9 UdeM00000042576 XP_033748360.1 57 388 147 6 39 426 111 478 4.00E-114 419.9 6579 cellular organisms;Eukaryota;Opisthokonta;Metazoa;Eumetazoa;Bilateria;Protostomia;Spiralia;Lophotrochozoa;Mollusca;Bivalvia;Autobranchia;Pteriomorphia;Pectinida;Pectinoidea;Pectinidae;Pecten;Pecten maximus
UdeM00000026946 7574 150.6 UdeM00000026946 XP_013396733.1 40.4 228 133 3 74 299 22 248 3.20E-33 150.6 7574 cellular organisms;Eukaryota;Opisthokonta;Metazoa;Eumetazoa;Bilateria;Protostomia;Spiralia;Lophotrochozoa;Brachiopoda;Linguliformea;Lingulata;Lingulida;Linguloidea;Lingulidae;Lingula;Lingula anatina

UdeM00000026947 29159 919.1 UdeM00000026947 XP_034302988.1 70.8 612
175 1 40 651 65 672 3.40E-264 919.1 29159 cellular
organisms;Eukaryota;Opisthokonta;Metazoa;Eumetazoa;Bilateria;Protostomia;
Spiralia;Lophotrochozoa;Mollusca;Bivalvia;Autobranchia;Pteriomorphia;Ostr
eida;Ostreoidea;Ostreidae;Crassostrea;Crassostrea gigas
UdeM00000026948 6565 236.5 UdeM00000026948 XP_022321940.1 24.8 674
471 14 12 661 16 677 9.90E-59 236.5 6565 cellular
organisms;Eukaryota;Opisthokonta;Metazoa;Eumetazoa;Bilateria;Protostomia;
Spiralia;Lophotrochozoa;Mollusca;Bivalvia;Autobranchia;Pteriomorphia;Ostr
eida;Ostreoidea;Ostreidae;Crassostrea;Crassostrea virginica
UdeM00000026949 6596 133.7 UdeM00000026949 XP_045206389.1 51.2 127
51 4 12 131 161 283 1.90E-28 133.7 6596 cellular
organisms;Eukaryota;Opisthokonta;Metazoa;Eumetazoa;Bilateria;Protostomia;
Spiralia;Lophotrochozoa;Mollusca;Bivalvia;Autobranchia;Heteroconchia;Euhe
terodonta;Imparidentia;Venerida;Veneroidea;Veneridae;Mercenaria;Mercenari
a mercenaria
UdeM00000026950 6596 969.1 UdeM00000026950 XP_045206940.1 57.9 907
258 19 37 869 1 857 3.70E-279 969.1 6596 cellular
organisms;Eukaryota;Opisthokonta;Metazoa;Eumetazoa;Bilateria;Protostomia;
Spiralia;Lophotrochozoa;Mollusca;Bivalvia;Autobranchia;Heteroconchia;Euhe
terodonta;Imparidentia;Venerida;Veneroidea;Veneridae;Mercenaria;Mercenari
a mercenaria
UdeM00000026951 6596 979.5 UdeM00000026951 XP_045206940.1 58.9 892
258 18 37 854 1 857 2.70E-282 979.5 6596 cellular
organisms;Eukaryota;Opisthokonta;Metazoa;Eumetazoa;Bilateria;Protostomia;
Spiralia;Lophotrochozoa;Mollusca;Bivalvia;Autobranchia;Heteroconchia;Euhe
terodonta;Imparidentia;Venerida;Veneroidea;Veneridae;Mercenaria;Mercenari
a mercenaria
UdeM00000026952 6850 77.8 UdeM00000026952 XP_013783731.1 32.9 146
91 2 5 150 846 984 1.30E-11 77.8 6850 cellular
organisms;Eukaryota;Opisthokonta;Metazoa;Eumetazoa;Bilateria;Protostomia;
Ecdysozoa;Panarthropoda;Arthropoda;Chelicerata;Merostomata;Xiphosura;Limu
lidae;Limulus;Limulus polyphemus
UdeM00000026953 6596 368.6 UdeM00000026953 XP_045206584.1 63.5 274
97 2 1 272 1 273 6.80E-99 368.6 6596 cellular
organisms;Eukaryota;Opisthokonta;Metazoa;Eumetazoa;Bilateria;Protostomia;
Spiralia;Lophotrochozoa;Mollusca;Bivalvia;Autobranchia;Heteroconchia;Euhe
terodonta;Imparidentia;Venerida;Veneroidea;Veneridae;Mercenaria;Mercenari
a mercenaria
UdeM00000026954 6565 471.9 UdeM00000026954 XP_022320194.1 55.5 476
196 6 1 476 1 460 1.70E-129 471.9 6565 cellular
organisms;Eukaryota;Opisthokonta;Metazoa;Eumetazoa;Bilateria;Protostomia;
Spiralia;Lophotrochozoa;Mollusca;Bivalvia;Autobranchia;Pteriomorphia;Ostr
eida;Ostreoidea;Ostreidae;Crassostrea;Crassostrea virginica
UdeM00000026955 6596 485.7 UdeM00000026955 XP_045206044.1 44.3 628
284 9 127 749 932 1498 1.10E-133 485.7 6596 cellular
organisms;Eukaryota;Opisthokonta;Metazoa;Eumetazoa;Bilateria;Protostomia;
Spiralia;Lophotrochozoa;Mollusca;Bivalvia;Autobranchia;Heteroconchia;Euhe
terodonta;Imparidentia;Venerida;Veneroidea;Veneridae;Mercenaria;Mercenari
a mercenaria
UdeM00000026956 6565 646 UdeM00000026956 XP_022325799.1 76.2 491
111 4 7 495 356 842 4.10E-182 646 6565 cellular
organisms;Eukaryota;Opisthokonta;Metazoa;Eumetazoa;Bilateria;Protostomia;

Spiralia;Lophotrochozoa;Mollusca;Bivalvia;Autobranchia;Pteriomorphia;Ostreida;Ostreoidea;Ostreidae;Crassostrea;Crassostrea virginica
UdeM00000026957 6596 302.8 UdeM00000026957 XP_045206044.1 48.4 395 141 13 12 367 8 378 6.20E-79 302.8 6596 cellular organisms;Eukaryota;Opisthokonta;Metazoa;Eumetazoa;Bilateria;Protostomia;Spiralia;Lophotrochozoa;Mollusca;Bivalvia;Autobranchia;Heteroconchia;Euheterodonta;Imparidentia;Venerida;Veneroidea;Veneridae;Mercenaria;Mercenaria mercenaria
UdeM00000026958 29159 103.2 UdeM00000026958 XP_034300565.1 55.6 99 43 1 14 112 468 565 1.30E-18 103.2 29159 cellular organisms;Eukaryota;Opisthokonta;Metazoa;Eumetazoa;Bilateria;Protostomia;Spiralia;Lophotrochozoa;Mollusca;Bivalvia;Autobranchia;Pteriomorphia;Ostreida;Ostreoidea;Ostreidae;Crassostrea;Crassostrea gigas
UdeM00000026959 400727 504.2 UdeM00000026959 XP_025113774.1 71.9 363 98 3 7 368 214 573 1.40E-139 504.2 400727 cellular organisms;Eukaryota;Opisthokonta;Metazoa;Eumetazoa;Bilateria;Protostomia;Spiralia;Lophotrochozoa;Mollusca;Gastropoda;Caenogastropoda;Architaenioglossa;Ampullarioidea;Ampullariidae;Pomacea;Pomacea canaliculata
UdeM00000026960 6596 354.8 UdeM00000026960 XP_045201815.1 34.5 730 428 18 68 769 76 783 3.00E-94 354.8 6596 cellular organisms;Eukaryota;Opisthokonta;Metazoa;Eumetazoa;Bilateria;Protostomia;Spiralia;Lophotrochozoa;Mollusca;Bivalvia;Autobranchia;Heteroconchia;Euheterodonta;Imparidentia;Venerida;Veneroidea;Veneridae;Mercenaria;Mercenaria mercenaria
UdeM00000026961 6596 339.3 UdeM00000026961 XP_045201815.1 33.4 718 427 16 17 703 18 715 1.20E-89 339.3 6596 cellular organisms;Eukaryota;Opisthokonta;Metazoa;Eumetazoa;Bilateria;Protostomia;Spiralia;Lophotrochozoa;Mollusca;Bivalvia;Autobranchia;Heteroconchia;Euheterodonta;Imparidentia;Venerida;Veneroidea;Veneridae;Mercenaria;Mercenaria mercenaria
UdeM00000026962 6596 332 UdeM00000026962 XP_045201815.1 31.8 824 486 19 11 798 9 792 2.10E-87 332 6596 cellular organisms;Eukaryota;Opisthokonta;Metazoa;Eumetazoa;Bilateria;Protostomia;Spiralia;Lophotrochozoa;Mollusca;Bivalvia;Autobranchia;Heteroconchia;Euheterodonta;Imparidentia;Venerida;Veneroidea;Veneridae;Mercenaria;Mercenaria mercenaria
UdeM00000026963 6596 1325.1 UdeM00000026963 XP_045203422.1 72 1005 238 12 1 994 32 1004 0.00E+00 1325.1 6596 cellular organisms;Eukaryota;Opisthokonta;Metazoa;Eumetazoa;Bilateria;Protostomia;Spiralia;Lophotrochozoa;Mollusca;Bivalvia;Autobranchia;Heteroconchia;Euheterodonta;Imparidentia;Venerida;Veneroidea;Veneridae;Mercenaria;Mercenaria mercenaria
UdeM00000026964 6579 272.3 UdeM00000026964 XP_033730189.1 48.3 294 104 5 1 251 1 289 6.30E-70 272.3 6579 cellular organisms;Eukaryota;Opisthokonta;Metazoa;Eumetazoa;Bilateria;Protostomia;Spiralia;Lophotrochozoa;Mollusca;Bivalvia;Autobranchia;Pteriomorphia;Pectinida;Pectinoidea;Pectinidae;Pecten;Pecten maximus
UdeM00000026965 6579 434.5 UdeM00000026965 XP_033730189.1 54.1 399 135 5 1 356 1 394 1.80E-118 434.5 6579 cellular organisms;Eukaryota;Opisthokonta;Metazoa;Eumetazoa;Bilateria;Protostomia;Spiralia;Lophotrochozoa;Mollusca;Bivalvia;Autobranchia;Pteriomorphia;Pectinida;Pectinoidea;Pectinidae;Pecten;Pecten maximus

UdeM00000026966 6596 123.6 UdeM00000026966 XP_045194560.1 39 136
72 1 102 237 21 145 3.30E-25 123.6 6596 cellular
organisms;Eukaryota;Opisthokonta;Metazoa;Eumetazoa;Bilateria;Protostomia;
Spiralia;Lophotrochozoa;Mollusca;Bivalvia;Autobranchia;Heteroconchia;Euhe
terodonta;Imparidentia;Venerida;Veneroidea;Veneridae;Mercenaria;Mercenari
a mercenaria
UdeM00000026967 6596 136.7 UdeM00000026967 XP_045194557.1 43 165
75 4 999 1152 746 902 1.80E-28 136.7 6596 cellular
organisms;Eukaryota;Opisthokonta;Metazoa;Eumetazoa;Bilateria;Protostomia;
Spiralia;Lophotrochozoa;Mollusca;Bivalvia;Autobranchia;Heteroconchia;Euhe
terodonta;Imparidentia;Venerida;Veneroidea;Veneridae;Mercenaria;Mercenari
a mercenaria
UdeM00000026968 6596 136.7 UdeM00000026968 XP_045194557.1 43 165
75 4 1001 1154 746 902 1.80E-28 136.7 6596 cellular
organisms;Eukaryota;Opisthokonta;Metazoa;Eumetazoa;Bilateria;Protostomia;
Spiralia;Lophotrochozoa;Mollusca;Bivalvia;Autobranchia;Heteroconchia;Euhe
terodonta;Imparidentia;Venerida;Veneroidea;Veneridae;Mercenaria;Mercenari
a mercenaria
UdeM00000026969 6565 148.3 UdeM00000026969 XP_022302274.1 47.9 146
74 2 154 299 159 302 1.60E-32 148.3 6565 cellular
organisms;Eukaryota;Opisthokonta;Metazoa;Eumetazoa;Bilateria;Protostomia;
Spiralia;Lophotrochozoa;Mollusca;Bivalvia;Autobranchia;Pteriomorphia;Ostr
eida;Ostreoidea;Ostreidae;Crassostrea;Crassostrea virginica
UdeM00000026970 6596 817.8 UdeM00000026970 XP_045194546.1 66.6 580
193 1 1 580 26 604 9.50E-234 817.8 6596 cellular
organisms;Eukaryota;Opisthokonta;Metazoa;Eumetazoa;Bilateria;Protostomia;
Spiralia;Lophotrochozoa;Mollusca;Bivalvia;Autobranchia;Heteroconchia;Euhe
terodonta;Imparidentia;Venerida;Veneroidea;Veneridae;Mercenaria;Mercenari
a mercenaria
UdeM00000026971 6596 639.4 UdeM00000026971 XP_045164241.1 69.3 436
134 0 4 439 2 437 3.30E-180 639.4 6596 cellular
organisms;Eukaryota;Opisthokonta;Metazoa;Eumetazoa;Bilateria;Protostomia;
Spiralia;Lophotrochozoa;Mollusca;Bivalvia;Autobranchia;Heteroconchia;Euhe
terodonta;Imparidentia;Venerida;Veneroidea;Veneridae;Mercenaria;Mercenari
a mercenaria
UdeM00000026972 225164 660.2 UdeM00000026972 XP_009065481.1 55.9
590 257 1 3 592 426 1012 2.40E-186 660.2 225164
cellular
organisms;Eukaryota;Opisthokonta;Metazoa;Eumetazoa;Bilateria;Protostomia;
Spiralia;Lophotrochozoa;Mollusca;Gastropoda;Patellogastropoda;Lottioidea;
Lottiidae;Lottia;Lottia gigantea
UdeM00000026973 1735272 1181.8 UdeM00000026973 XP_041369326.1
57.5 1011 427 2 7 1015 6 1015 0.00E+00 1181.8
1735272 cellular
organisms;Eukaryota;Opisthokonta;Metazoa;Eumetazoa;Bilateria;Protostomia;
Spiralia;Lophotrochozoa;Mollusca;Gastropoda;Neomphalina;Neomphaloidea;Pel
tospiridae;Gigantopelta;Gigantopelta aegis
UdeM00000026974 6596 912.5 UdeM00000026974 XP_045164241.1 61.1 712
257 2 8 714 6 702 3.40E-262 912.5 6596 cellular
organisms;Eukaryota;Opisthokonta;Metazoa;Eumetazoa;Bilateria;Protostomia;
Spiralia;Lophotrochozoa;Mollusca;Bivalvia;Autobranchia;Heteroconchia;Euhe
terodonta;Imparidentia;Venerida;Veneroidea;Veneridae;Mercenaria;Mercenari
a mercenaria

UdeM00000026975 6596 325.9 UdeM00000026975 XP_045186701.1 56.1 289
127 0 1 289 213 501 5.30E-86 325.9 6596 cellular
organisms;Eukaryota;Opisthokonta;Metazoa;Eumetazoa;Bilateria;Protostomia;
Spiralia;Lophotrochozoa;Mollusca;Bivalvia;Autobranchia;Heteroconchia;Euhe
terodonta;Imparidentia;Venerida;Veneroidea;Veneridae;Mercenaria;Mercenari
a mercenaria
UdeM00000026977 6596 198.4 UdeM00000026977 XP_045187823.1 45.3 192
103 2 1 192 1 190 1.20E-47 198.4 6596 cellular
organisms;Eukaryota;Opisthokonta;Metazoa;Eumetazoa;Bilateria;Protostomia;
Spiralia;Lophotrochozoa;Mollusca;Bivalvia;Autobranchia;Heteroconchia;Euhe
terodonta;Imparidentia;Venerida;Veneroidea;Veneridae;Mercenaria;Mercenari
a mercenaria
UdeM00000026976 6596 276.9 UdeM00000026976 XP_045187823.1 39.1 419
237 7 1 403 1 417 4.00E-71 276.9 6596 cellular
organisms;Eukaryota;Opisthokonta;Metazoa;Eumetazoa;Bilateria;Protostomia;
Spiralia;Lophotrochozoa;Mollusca;Bivalvia;Autobranchia;Heteroconchia;Euhe
terodonta;Imparidentia;Venerida;Veneroidea;Veneridae;Mercenaria;Mercenari
a mercenaria
UdeM00000026978 6596 610.1 UdeM00000026978 XP_045199866.1 70.9 381
106 2 2 377 36 416 1.90E-171 610.1 6596 cellular
organisms;Eukaryota;Opisthokonta;Metazoa;Eumetazoa;Bilateria;Protostomia;
Spiralia;Lophotrochozoa;Mollusca;Bivalvia;Autobranchia;Heteroconchia;Euhe
terodonta;Imparidentia;Venerida;Veneroidea;Veneridae;Mercenaria;Mercenari
a mercenaria
UdeM00000026979 6596 2640.9 UdeM00000026979 XP_045199866.1 58.3
2093 773 10 3 2089 404 2402 0.00E+00 2640.9 6596
cellular
organisms;Eukaryota;Opisthokonta;Metazoa;Eumetazoa;Bilateria;Protostomia;
Spiralia;Lophotrochozoa;Mollusca;Bivalvia;Autobranchia;Heteroconchia;Euhe
terodonta;Imparidentia;Venerida;Veneroidea;Veneridae;Mercenaria;Mercenari
a mercenaria
UdeM00000026980 1735272 130.6 UdeM00000026980 XP_041369835.1 46.5
142 69 4 4 141 9 147 1.70E-27 130.6 1735272
cellular
organisms;Eukaryota;Opisthokonta;Metazoa;Eumetazoa;Bilateria;Protostomia;
Spiralia;Lophotrochozoa;Mollusca;Gastropoda;Neomphalina;Neomphaloidea;Pel
tospiridae;Gigantopelta;Gigantopelta aegis
UdeM00000026981 6596 658.7 UdeM00000026981 XP_045209562.1 68.6 439
136 1 21 457 90 528 5.70E-186 658.7 6596 cellular
organisms;Eukaryota;Opisthokonta;Metazoa;Eumetazoa;Bilateria;Protostomia;
Spiralia;Lophotrochozoa;Mollusca;Bivalvia;Autobranchia;Heteroconchia;Euhe
terodonta;Imparidentia;Venerida;Veneroidea;Veneridae;Mercenaria;Mercenari
a mercenaria
UdeM00000026982 225164 267.3 UdeM00000026982 XP_009050560.1 86.4
147 20 0 1 147 1 147 1.20E-68 267.3 225164
cellular
organisms;Eukaryota;Opisthokonta;Metazoa;Eumetazoa;Bilateria;Protostomia;
Spiralia;Lophotrochozoa;Mollusca;Gastropoda;Patellogastropoda;Lottioidea;
Lottiidae;Lottia;Lottia gigantea
UdeM00000026983 6596 430.6 UdeM00000026983 XP_045198983.1 52.1 455
190 8 2 444 9 447 2.40E-117 430.6 6596 cellular
organisms;Eukaryota;Opisthokonta;Metazoa;Eumetazoa;Bilateria;Protostomia;
Spiralia;Lophotrochozoa;Mollusca;Bivalvia;Autobranchia;Heteroconchia;Euhe

terodonta;Imparidentia;Venerida;Veneroidea;Veneridae;Mercenaria;Mercenaria mercenaria
UdeM00000026984 29159 75.9 UdeM00000026984 XP_011457094.1 35.1 131 80 2 4 133 2 128 5.70E-11 75.9 29159 cellular organisms;Eukaryota;Opisthokonta;Metazoa;Eumetazoa;Bilateria;Protostomia;Spiralia;Lophotrochozoa;Mollusca;Bivalvia;Autobranchia;Pteriomorphia;Ostreida;Ostreoidea;Ostreidae;Crassostrea;Crassostrea gigas
UdeM00000026985 1735272 492.3 UdeM00000026985 XP_041369682.1 53.8 450 186 8 16 454 296 734 6.80E-136 492.3 1735272 cellular organisms;Eukaryota;Opisthokonta;Metazoa;Eumetazoa;Bilateria;Protostomia;Spiralia;Lophotrochozoa;Mollusca;Gastropoda;Neomphalina;Neomphaloidea;Peltospiridae;Gigantopelta;Gigantopelta aegis
UdeM00000026986 6596 284.6 UdeM00000026986 XP_045162844.1 50.2 263 129 1 11 273 44 304 1.40E-73 284.6 6596 cellular organisms;Eukaryota;Opisthokonta;Metazoa;Eumetazoa;Bilateria;Protostomia;Spiralia;Lophotrochozoa;Mollusca;Bivalvia;Autobranchia;Heteroconchia;Euheterodonta;Imparidentia;Venerida;Veneroidea;Veneridae;Mercenaria;Mercenaria mercenaria
UdeM00000026987 29159 94.4 UdeM00000026987 XP_011422012.2 39.4 142 70 5 6 141 5 136 1.30E-16 94.4 29159 cellular organisms;Eukaryota;Opisthokonta;Metazoa;Eumetazoa;Bilateria;Protostomia;Spiralia;Lophotrochozoa;Mollusca;Bivalvia;Autobranchia;Pteriomorphia;Ostreida;Ostreoidea;Ostreidae;Crassostrea;Crassostrea gigas
UdeM00000026988 29159 90.1 UdeM00000026988 XP_034300135.1 43.3 120 62 4 24 141 21 136 2.40E-15 90.1 29159 cellular organisms;Eukaryota;Opisthokonta;Metazoa;Eumetazoa;Bilateria;Protostomia;Spiralia;Lophotrochozoa;Mollusca;Bivalvia;Autobranchia;Pteriomorphia;Ostreida;Ostreoidea;Ostreidae;Crassostrea;Crassostrea gigas
UdeM00000026989 29159 110.5 UdeM00000026989 XP_034315424.1 31.6 253 151 6 43 284 73 314 6.00E-21 110.5 29159 cellular organisms;Eukaryota;Opisthokonta;Metazoa;Eumetazoa;Bilateria;Protostomia;Spiralia;Lophotrochozoa;Mollusca;Bivalvia;Autobranchia;Pteriomorphia;Ostreida;Ostreoidea;Ostreidae;Crassostrea;Crassostrea gigas
UdeM00000026991 50390 183 UdeM00000026991 XP_028127913.1 42.9 217 123 1 1 217 1 216 4.30E-43 183 50390 cellular organisms;Eukaryota;Opisthokonta;Metazoa;Eumetazoa;Bilateria;Protostomia;Ecdysozoa;Panarthropoda;Arthropoda;Mandibulata;Pancrustacea;Hexapoda;Insecta;Dicondylia;Pterygota;Neoptera;Endopterygota;Coleoptera;Polyphaga;Cucujiformia;Chrysomeloidea;Chrysomelidae;Galerucinae;Luperini;Diabroticina;Diabroticites;Diabrotica;Diabrotica virgifera;Diabrotica virgifera virgifera
UdeM00000026992 50390 149.1 UdeM00000026992 XP_028127913.1 43.9 164 91 1 1 164 1 163 5.30E-33 149.1 50390 cellular organisms;Eukaryota;Opisthokonta;Metazoa;Eumetazoa;Bilateria;Protostomia;Ecdysozoa;Panarthropoda;Arthropoda;Mandibulata;Pancrustacea;Hexapoda;Insecta;Dicondylia;Pterygota;Neoptera;Endopterygota;Coleoptera;Polyphaga;Cucujiformia;Chrysomeloidea;Chrysomelidae;Galerucinae;Luperini;Diabroticina;Diabroticites;Diabrotica;Diabrotica virgifera;Diabrotica virgifera virgifera
UdeM00000026993 50390 161.4 UdeM00000026993 XP_028127913.1 42.9 191 106 2 1 189 1 190 1.10E-36 161.4 50390 cellular organisms;Eukaryota;Opisthokonta;Metazoa;Eumetazoa;Bilateria;Protostomia;Ecdysozoa;Panarthropoda;Arthropoda;Mandibulata;Pancrustacea;Hexapoda;Inse

cta;Dicondylia;Pterygota;Neoptera;Endopterygota;Coleoptera;Polyphaga;Cucujiformia;Chrysomeloidea;Chrysomelidae;Galerucinae;Luperini;Diabroticina;Diabroticites;Diabrotica;Diabrotica virgifera;Diabrotica virgifera virgifera
UdeM00000026995 1735272 220.7 UdeM00000026995 XP_041369281.1 54.5 191 86 1 1 191 82 271 1.60E-54 220.7 1735272 cellular organisms;Eukaryota;Opisthokonta;Metazoa;Eumetazoa;Bilateria;Protostomia;Spiralia;Lophotrochozoa;Mollusca;Gastropoda;Neomphalina;Neomphaloidea;Peltospiridae;Gigantopelta;Gigantopelta aegis
UdeM00000026994 1735272 234.6 UdeM00000026994 XP_041369281.1 54.1 218 95 3 78 295 59 271 1.70E-58 234.6 1735272 cellular organisms;Eukaryota;Opisthokonta;Metazoa;Eumetazoa;Bilateria;Protostomia;Spiralia;Lophotrochozoa;Mollusca;Gastropoda;Neomphalina;Neomphaloidea;Peltospiridae;Gigantopelta;Gigantopelta aegis
UdeM00000026996 6573 170.6 UdeM00000026996 XP_021355484.1 64.7 119 42 0 83 201 106 224 2.00E-39 170.6 6573 cellular organisms;Eukaryota;Opisthokonta;Metazoa;Eumetazoa;Bilateria;Protostomia;Spiralia;Lophotrochozoa;Mollusca;Bivalvia;Autobranchia;Pteriomorphia;Pectinida;Pectinoidea;Pectinidae;Mizuhopecten;Mizuhopecten yessoensis
UdeM00000026997 6573 181 UdeM00000026997 XP_021355484.1 50 226 80 7 1 195 1 224 1.40E-42 181 6573 cellular organisms;Eukaryota;Opisthokonta;Metazoa;Eumetazoa;Bilateria;Protostomia;Spiralia;Lophotrochozoa;Mollusca;Bivalvia;Autobranchia;Pteriomorphia;Pectinida;Pectinoidea;Pectinidae;Mizuhopecten;Mizuhopecten yessoensis
UdeM00000026999 6579 551.6 UdeM00000026999 XP_033732865.1 50.9 603 263 12 9 597 35 618 1.50E-153 551.6 6579 cellular organisms;Eukaryota;Opisthokonta;Metazoa;Eumetazoa;Bilateria;Protostomia;Spiralia;Lophotrochozoa;Mollusca;Bivalvia;Autobranchia;Pteriomorphia;Pectinida;Pectinoidea;Pectinidae;Pecten;Pecten maximus
UdeM00000027000 6596 337.8 UdeM00000027000 XP_045203827.1 54.8 305 137 1 7 311 8 311 1.50E-89 337.8 6596 cellular organisms;Eukaryota;Opisthokonta;Metazoa;Eumetazoa;Bilateria;Protostomia;Spiralia;Lophotrochozoa;Mollusca;Bivalvia;Autobranchia;Heteroconchia;Euheterodonta;Imparidentia;Venerida;Veneroidea;Veneridae;Mercenaria;Mercenaria mercenaria
UdeM00000027001 1735272 85.1 UdeM00000027001 XP_041362151.1 38.1 113 69 1 9 120 563 675 7.20E-14 85.1 1735272 cellular organisms;Eukaryota;Opisthokonta;Metazoa;Eumetazoa;Bilateria;Protostomia;Spiralia;Lophotrochozoa;Mollusca;Gastropoda;Neomphalina;Neomphaloidea;Peltospiridae;Gigantopelta;Gigantopelta aegis
UdeM00000027002 6579 58.5 UdeM00000027002 XP_033761495.1 61.4 44 17 0 36 79 24 67 4.40E-06 58.5 6579 cellular organisms;Eukaryota;Opisthokonta;Metazoa;Eumetazoa;Bilateria;Protostomia;Spiralia;Lophotrochozoa;Mollusca;Bivalvia;Autobranchia;Pteriomorphia;Pectinida;Pectinoidea;Pectinidae;Pecten;Pecten maximus
UdeM00000027003 6596 377.5 UdeM00000027003 XP_045203827.1 57.7 310 131 0 3 312 4 313 1.70E-101 377.5 6596 cellular organisms;Eukaryota;Opisthokonta;Metazoa;Eumetazoa;Bilateria;Protostomia;Spiralia;Lophotrochozoa;Mollusca;Bivalvia;Autobranchia;Heteroconchia;Euheterodonta;Imparidentia;Venerida;Veneroidea;Veneridae;Mercenaria;Mercenaria mercenaria

UdeM00000027004 6596 500.7 UdeM00000027004 XP_045164893.1 66.3 365
122 1 4 368 3 366 1.50E-138 500.7 6596 cellular
organisms;Eukaryota;Opisthokonta;Metazoa;Eumetazoa;Bilateria;Protostomia;Spiralia;Lophotrochozoa;Mollusca;Bivalvia;Autobranchia;Heteroconchia;Euheterodonta;Imparidentia;Venerida;Veneroidea;Veneridae;Mercenaria;Mercenaria mercenaria
UdeM00000027005 6596 105.5 UdeM00000027005 XP_045208672.1 36.6 134
81 4 160 292 22 152 1.20E-19 105.5 6596 cellular
organisms;Eukaryota;Opisthokonta;Metazoa;Eumetazoa;Bilateria;Protostomia;Spiralia;Lophotrochozoa;Mollusca;Bivalvia;Autobranchia;Heteroconchia;Euheterodonta;Imparidentia;Venerida;Veneroidea;Veneridae;Mercenaria;Mercenaria mercenaria
UdeM00000027006 6596 105.1 UdeM00000027006 XP_045208672.1 36.6 134
81 4 223 355 22 152 1.80E-19 105.1 6596 cellular
organisms;Eukaryota;Opisthokonta;Metazoa;Eumetazoa;Bilateria;Protostomia;Spiralia;Lophotrochozoa;Mollusca;Bivalvia;Autobranchia;Heteroconchia;Euheterodonta;Imparidentia;Venerida;Veneroidea;Veneridae;Mercenaria;Mercenaria mercenaria
UdeM00000027008 6500 213.4 UdeM00000027008 XP_012939160.1 59.4 180
47 2 19 173 4 182 5.50E-52 213.4 6500 cellular
organisms;Eukaryota;Opisthokonta;Metazoa;Eumetazoa;Bilateria;Protostomia;Spiralia;Lophotrochozoa;Mollusca;Gastropoda;Heterobranchia;Euthyneura;Tectipleura;Aplysiida;Aplysioidea;Aplysiidae;Aplysia;Aplysia californica
UdeM00000027013 6596 105.5 UdeM00000027013 XP_045208672.1 36.6 134
81 4 160 292 22 152 1.20E-19 105.5 6596 cellular
organisms;Eukaryota;Opisthokonta;Metazoa;Eumetazoa;Bilateria;Protostomia;Spiralia;Lophotrochozoa;Mollusca;Bivalvia;Autobranchia;Heteroconchia;Euheterodonta;Imparidentia;Venerida;Veneroidea;Veneridae;Mercenaria;Mercenaria mercenaria
UdeM00000027012 6596 105.5 UdeM00000027012 XP_045208672.1 36.6 134
81 4 166 298 22 152 1.20E-19 105.5 6596 cellular
organisms;Eukaryota;Opisthokonta;Metazoa;Eumetazoa;Bilateria;Protostomia;Spiralia;Lophotrochozoa;Mollusca;Bivalvia;Autobranchia;Heteroconchia;Euheterodonta;Imparidentia;Venerida;Veneroidea;Veneridae;Mercenaria;Mercenaria mercenaria
UdeM00000027014 218467 78.2 UdeM00000027014 XP_023220707.1 22.8
364 215 14 13 356 92 409 2.40E-11 78.2 218467
cellular
organisms;Eukaryota;Opisthokonta;Metazoa;Eumetazoa;Bilateria;Protostomia;Ecdysozoa;Panarthropoda;Arthropoda;Chelicerata;Arachnida;Scorpiones;Buthida;Buthoidea;Buthidae;Centruroides;Centruroides sculpturatus
UdeM00000027015 6596 119.4 UdeM00000027015 XP_045157325.1 27.6 344
199 15 148 484 2286 2586 1.50E-23 119.4 6596 cellular
organisms;Eukaryota;Opisthokonta;Metazoa;Eumetazoa;Bilateria;Protostomia;Spiralia;Lophotrochozoa;Mollusca;Bivalvia;Autobranchia;Heteroconchia;Euheterodonta;Imparidentia;Venerida;Veneroidea;Veneridae;Mercenaria;Mercenaria mercenaria
UdeM00000027016 8236 62.4 UdeM00000027016 XP_044217658.1 26.2 168
100 7 56 218 200 348 8.60E-07 62.4 8236 cellular
organisms;Eukaryota;Opisthokonta;Metazoa;Eumetazoa;Bilateria;Deuterostomia;Chordata;Craniata;Vertebrata;Gnathostomata;Teleostomi;Euteleostomi;Actinopterygii;Actinopteri;Neopterygii;Teleostei;Osteoglossocephalai;Clupeocephala;Euteleosteomorpha;Neoteleostei;Eurypterygia;Ctenosquamata;Acanthomo

rphata;Euacanthomorphacea;Percomorphaceae;Pelagiaria;Scombriformes;Scombridae;Scombrinae;Thunnini;Thunnus;Thunnus albacares
UdeM00000027017 6596 100.1 UdeM00000027017 XP_045192613.1 26.8 366 220 18 7 349 992 1332 5.90E-18 100.1 6596 cellular organisms;Eukaryota;Opisthokonta;Metazoa;Eumetazoa;Bilateria;Protostomia;Spiralia;Lophotrochozoa;Mollusca;Bivalvia;Autobranchia;Heteroconchia;Euheterodonta;Imparidentia;Venerida;Veneroidea;Veneridae;Mercenaria;Mercenaria mercenaria
UdeM00000027018 37653 255 UdeM00000027018 XP_014773075.1 22.2 1915 1096 90 235 2045 1622 3247 8.40E-64 255 37653 cellular organisms;Eukaryota;Opisthokonta;Metazoa;Eumetazoa;Bilateria;Protostomia;Spiralia;Lophotrochozoa;Mollusca;Cephalopoda;Coleoidea;Octopodiformes;Octopoda;Incirrata;Octopodidae;Octopus;Octopus bimaculoides
UdeM00000027019 6596 187.2 UdeM00000027019 XP_045210928.1 63.2 144 53 0 1 144 4 147 2.00E-44 187.2 6596 cellular organisms;Eukaryota;Opisthokonta;Metazoa;Eumetazoa;Bilateria;Protostomia;Spiralia;Lophotrochozoa;Mollusca;Bivalvia;Autobranchia;Heteroconchia;Euheterodonta;Imparidentia;Venerida;Veneroidea;Veneridae;Mercenaria;Mercenaria mercenaria
UdeM00000027020 6596 226.5 UdeM00000027020 XP_045189940.1 63.4 175 61 2 1 173 1 174 2.60E-56 226.5 6596 cellular organisms;Eukaryota;Opisthokonta;Metazoa;Eumetazoa;Bilateria;Protostomia;Spiralia;Lophotrochozoa;Mollusca;Bivalvia;Autobranchia;Heteroconchia;Euheterodonta;Imparidentia;Venerida;Veneroidea;Veneridae;Mercenaria;Mercenaria mercenaria
UdeM00000027021 6573 343.2 UdeM00000027021 XP_021376136.1 44.1 476 211 15 1 435 1 462 4.90E-91 343.2 6573 cellular organisms;Eukaryota;Opisthokonta;Metazoa;Eumetazoa;Bilateria;Protostomia;Spiralia;Lophotrochozoa;Mollusca;Bivalvia;Autobranchia;Pteriomorphia;Pectinida;Pectinoidea;Pectinidae;Mizuhopecten;Mizuhopecten yessoensis
UdeM00000027022 6596 600.1 UdeM00000027022 XP_045204483.1 52.3 623 255 9 1 583 1 621 2.90E-168 600.1 6596 cellular organisms;Eukaryota;Opisthokonta;Metazoa;Eumetazoa;Bilateria;Protostomia;Spiralia;Lophotrochozoa;Mollusca;Bivalvia;Autobranchia;Heteroconchia;Euheterodonta;Imparidentia;Venerida;Veneroidea;Veneridae;Mercenaria;Mercenaria mercenaria
UdeM00000027023 6565 583.2 UdeM00000027023 XP_022321940.1 43.5 671 370 5 10 672 13 682 4.30E-163 583.2 6565 cellular organisms;Eukaryota;Opisthokonta;Metazoa;Eumetazoa;Bilateria;Protostomia;Spiralia;Lophotrochozoa;Mollusca;Bivalvia;Autobranchia;Pteriomorphia;Ostreida;Ostreoidea;Ostreidae;Crassostrea;Crassostrea virginica
UdeM00000027024 6596 130.2 UdeM00000027024 XP_045211321.1 55.3 103 46 0 3 105 66 168 1.60E-27 130.2 6596 cellular organisms;Eukaryota;Opisthokonta;Metazoa;Eumetazoa;Bilateria;Protostomia;Spiralia;Lophotrochozoa;Mollusca;Bivalvia;Autobranchia;Heteroconchia;Euheterodonta;Imparidentia;Venerida;Veneroidea;Veneridae;Mercenaria;Mercenaria mercenaria
UdeM00000027025 6573 107.8 UdeM00000027025 XP_021370619.1 44.1 118 66 0 10 127 9 126 1.20E-20 107.8 6573 cellular organisms;Eukaryota;Opisthokonta;Metazoa;Eumetazoa;Bilateria;Protostomia;Spiralia;Lophotrochozoa;Mollusca;Bivalvia;Autobranchia;Pteriomorphia;Pectinida;Pectinoidea;Pectinidae;Mizuhopecten;Mizuhopecten yessoensis
UdeM00000027026 6596 211.8 UdeM00000027026 XP_045211321.1 57.1 168 70 2 1 166 1 168 6.60E-52 211.8 6596 cellular

organisms;Eukaryota;Opisthokonta;Metazoa;Eumetazoa;Bilateria;Protostomia;Spiralia;Lophotrochozoa;Mollusca;Bivalvia;Autobranchia;Heteroconchia;Euheterodonta;Imparidentia;Venerida;Veneroidea;Veneridae;Mercenaria;Mercenaria mercenaria
UdeM00000027027 1735272 195.3 UdeM00000027027 XP_041371017.1 60 155 59 1 20 174 27 178 6.60E-47 195.3 1735272 cellular organisms;Eukaryota;Opisthokonta;Metazoa;Eumetazoa;Bilateria;Protostomia;Spiralia;Lophotrochozoa;Mollusca;Gastropoda;Neomphalina;Neomphaloidea;Peltospiridae;Gigantopelta;Gigantopelta aegis
UdeM00000027028 6573 134.8 UdeM00000027028 XP_021360339.1 46.8 156 75 3 9 157 4 158 1.10E-28 134.8 6573 cellular organisms;Eukaryota;Opisthokonta;Metazoa;Eumetazoa;Bilateria;Protostomia;Spiralia;Lophotrochozoa;Mollusca;Bivalvia;Autobranchia;Pteriomorphia;Pectinida;Pectinoidea;Pectinidae;Mizuhopecten;Mizuhopecten yessoensis
UdeM00000027030 6596 88.6 UdeM00000027030 XP_045194463.1 31.8 151 90 3 1 138 115 265 6.90E-15 88.6 6596 cellular organisms;Eukaryota;Opisthokonta;Metazoa;Eumetazoa;Bilateria;Protostomia;Spiralia;Lophotrochozoa;Mollusca;Bivalvia;Autobranchia;Heteroconchia;Euheterodonta;Imparidentia;Venerida;Veneroidea;Veneridae;Mercenaria;Mercenaria mercenaria
UdeM00000027031 6596 372.1 UdeM00000027031 XP_045196406.1 66.9 260 84 2 1 259 32 290 6.10E-100 372.1 6596 cellular organisms;Eukaryota;Opisthokonta;Metazoa;Eumetazoa;Bilateria;Protostomia;Spiralia;Lophotrochozoa;Mollusca;Bivalvia;Autobranchia;Heteroconchia;Euheterodonta;Imparidentia;Venerida;Veneroidea;Veneridae;Mercenaria;Mercenaria mercenaria
UdeM00000027033 6596 372.1 UdeM00000027033 XP_045196406.1 66.9 260 84 2 1 259 32 290 6.00E-100 372.1 6596 cellular organisms;Eukaryota;Opisthokonta;Metazoa;Eumetazoa;Bilateria;Protostomia;Spiralia;Lophotrochozoa;Mollusca;Bivalvia;Autobranchia;Heteroconchia;Euheterodonta;Imparidentia;Venerida;Veneroidea;Veneridae;Mercenaria;Mercenaria mercenaria
UdeM00000027035 225164 288.5 UdeM00000027035 XP_009067154.1 63.4 205 73 2 1 204 81 284 6.80E-75 288.5 225164 cellular organisms;Eukaryota;Opisthokonta;Metazoa;Eumetazoa;Bilateria;Protostomia;Spiralia;Lophotrochozoa;Mollusca;Gastropoda;Patellogastropoda;Lottioidea;Lottiidae;Lottia;Lottia gigantea
UdeM00000027036 6596 210.3 UdeM00000027036 XP_045196406.1 66.4 137 46 0 1 137 32 168 1.60E-51 210.3 6596 cellular organisms;Eukaryota;Opisthokonta;Metazoa;Eumetazoa;Bilateria;Protostomia;Spiralia;Lophotrochozoa;Mollusca;Bivalvia;Autobranchia;Heteroconchia;Euheterodonta;Imparidentia;Venerida;Veneroidea;Veneridae;Mercenaria;Mercenaria mercenaria
UdeM00000027037 225164 147.5 UdeM00000027037 XP_009067154.1 65.3 101 34 1 17 117 154 253 1.20E-32 147.5 225164 cellular organisms;Eukaryota;Opisthokonta;Metazoa;Eumetazoa;Bilateria;Protostomia;Spiralia;Lophotrochozoa;Mollusca;Gastropoda;Patellogastropoda;Lottioidea;Lottiidae;Lottia;Lottia gigantea
UdeM00000027038 6596 387.5 UdeM00000027038 XP_045196300.1 62.9 267 98 1 193 458 6 272 2.40E-104 387.5 6596 cellular organisms;Eukaryota;Opisthokonta;Metazoa;Eumetazoa;Bilateria;Protostomia;

Spiralia;Lophotrochozoa;Mollusca;Bivalvia;Autobranchia;Heteroconchia;Euheterodonta;Imparidentia;Venerida;Veneroidea;Veneridae;Mercenaria;Mercenaria mercenaria
UdeM00000027039 27687 377.5 UdeM00000027039 XP_028657207.1 40.6 545 270 11 4 508 194 724 2.70E-101 377.5 27687 cellular organisms;Eukaryota;Opisthokonta;Metazoa;Eumetazoa;Bilateria;Deuterostomia;Chordata;Craniata;Vertebrata;Gnathostomata;Teleostomi;Euteleostomi;Actinopterygii;Cladistia;Polypteriformes;Polypteridae;Erpetoichthys;Erpetoichthys calabaricus
UdeM00000027040 6596 315.1 UdeM00000027040 XP_045210908.1 71 200 58 0 20 219 36 235 1.10E-82 315.1 6596 cellular organisms;Eukaryota;Opisthokonta;Metazoa;Eumetazoa;Bilateria;Protostomia;Spiralia;Lophotrochozoa;Mollusca;Bivalvia;Autobranchia;Heteroconchia;Euheterodonta;Imparidentia;Venerida;Veneroidea;Veneridae;Mercenaria;Mercenaria mercenaria
UdeM00000027041 6596 388.3 UdeM00000027041 XP_045163613.1 52.6 346 155 6 1 338 1 345 1.00E-104 388.3 6596 cellular organisms;Eukaryota;Opisthokonta;Metazoa;Eumetazoa;Bilateria;Protostomia;Spiralia;Lophotrochozoa;Mollusca;Bivalvia;Autobranchia;Heteroconchia;Euheterodonta;Imparidentia;Venerida;Veneroidea;Veneridae;Mercenaria;Mercenaria mercenaria
UdeM00000027042 6596 387.5 UdeM00000027042 XP_045196300.1 59.9 284 113 1 8 290 6 289 1.50E-104 387.5 6596 cellular organisms;Eukaryota;Opisthokonta;Metazoa;Eumetazoa;Bilateria;Protostomia;Spiralia;Lophotrochozoa;Mollusca;Bivalvia;Autobranchia;Heteroconchia;Euheterodonta;Imparidentia;Venerida;Veneroidea;Veneridae;Mercenaria;Mercenaria mercenaria
UdeM00000027043 6596 236.5 UdeM00000027043 XP_045201450.1 57.1 226 93 3 1 225 1 223 3.40E-59 236.5 6596 cellular organisms;Eukaryota;Opisthokonta;Metazoa;Eumetazoa;Bilateria;Protostomia;Spiralia;Lophotrochozoa;Mollusca;Bivalvia;Autobranchia;Heteroconchia;Euheterodonta;Imparidentia;Venerida;Veneroidea;Veneridae;Mercenaria;Mercenaria mercenaria
UdeM00000027044 6596 538.1 UdeM00000027044 XP_045201406.1 49.3 574 291 0 15 588 17 590 1.40E-149 538.1 6596 cellular organisms;Eukaryota;Opisthokonta;Metazoa;Eumetazoa;Bilateria;Protostomia;Spiralia;Lophotrochozoa;Mollusca;Bivalvia;Autobranchia;Heteroconchia;Euheterodonta;Imparidentia;Venerida;Veneroidea;Veneridae;Mercenaria;Mercenaria mercenaria
UdeM00000027045 6579 119 UdeM00000027045 XP_033729601.1 68.4 114 36 0 1 114 1 114 4.00E-24 119 6579 cellular organisms;Eukaryota;Opisthokonta;Metazoa;Eumetazoa;Bilateria;Protostomia;Spiralia;Lophotrochozoa;Mollusca;Bivalvia;Autobranchia;Pteriomorphia;Pectinida;Pectinoidea;Pectinidae;Pecten;Pecten maximus
UdeM00000027046 45264 238.4 UdeM00000027046 XP_044165535.1 47.2 231 118 1 1 227 294 524 1.00E-59 238.4 45264 cellular organisms;Eukaryota;Opisthokonta;Metazoa;Eumetazoa;Cnidaria;Anthozoa;Hexacorallia;Scleractinia;Astrocoeniina;Acroporidae;Acropora;Acropora millepora
UdeM00000027047 6573 167.5 UdeM00000027047 XP_021376971.1 29.3 533 320 17 3 507 402 905 4.30E-38 167.5 6573 cellular organisms;Eukaryota;Opisthokonta;Metazoa;Eumetazoa;Bilateria;Protostomia;Spiralia;Lophotrochozoa;Mollusca;Bivalvia;Autobranchia;Pteriomorphia;Pectinida;Pectinoidea;Pectinidae;Mizuhopecten;Mizuhopecten yessoensis

UdeM00000041302 7998 246.5 UdeM00000041302 XP_017334791.1 68.5 162
51 0 1 162 62 223 2.30E-62 246.5 7998 cellular
organisms;Eukaryota;Opisthokonta;Metazoa;Eumetazoa;Bilateria;Deuterostomi
a;Chordata;Craniata;Vertebrata;Gnathostomata;Teleostomi;Euteleostomi;Acti
nopterygii;Actinopteri;Neopterygii;Teleostei;Osteoglossocephalai;Clupeoce
phala;Otomorpha;Ostariophysi;Otophysi;Characiphysae;Siluriformes;Siluroid
ei;Ictaluridae;Ictalurus;Ictalurus punctatus
UdeM00000041303 6579 218 UdeM00000041303 XP_033732173.1 64.8 159
51 5 1 156 1 157 8.60E-54 218 6579 cellular
organisms;Eukaryota;Opisthokonta;Metazoa;Eumetazoa;Bilateria;Protostomia;
Spiralia;Lophotrochozoa;Mollusca;Bivalvia;Autobranchia;Pteriomorphia;Pect
inida;Pectinoidea;Pectinidae;Pecten;Pecten maximus
UdeM00000041304 29159 229.6 UdeM00000041304 XP_011429223.2 59.2 211
81 3 1 210 153 359 3.80E-57 229.6 29159 cellular
organisms;Eukaryota;Opisthokonta;Metazoa;Eumetazoa;Bilateria;Protostomia;
Spiralia;Lophotrochozoa;Mollusca;Bivalvia;Autobranchia;Pteriomorphia;Ostr
eida;Ostreoidea;Ostreidae;Crassostrea;Crassostrea gigas
UdeM00000041305 29159 418.7 UdeM00000041305 XP_011429223.2 60 360
139 3 6 364 4 359 7.60E-114 418.7 29159 cellular
organisms;Eukaryota;Opisthokonta;Metazoa;Eumetazoa;Bilateria;Protostomia;
Spiralia;Lophotrochozoa;Mollusca;Bivalvia;Autobranchia;Pteriomorphia;Ostr
eida;Ostreoidea;Ostreidae;Crassostrea;Crassostrea gigas
UdeM00000041306 6565 139.4 UdeM00000041306 XP_022305948.1 65.6 90
31 0 1 90 103 192 2.20E-30 139.4 6565 cellular
organisms;Eukaryota;Opisthokonta;Metazoa;Eumetazoa;Bilateria;Protostomia;
Spiralia;Lophotrochozoa;Mollusca;Bivalvia;Autobranchia;Pteriomorphia;Ostr
eida;Ostreoidea;Ostreidae;Crassostrea;Crassostrea virginica
UdeM00000041307 6573 303.1 UdeM00000041307 XP_021345141.1 87 177
23 0 1 177 1 177 2.30E-79 303.1 6573 cellular
organisms;Eukaryota;Opisthokonta;Metazoa;Eumetazoa;Bilateria;Protostomia;
Spiralia;Lophotrochozoa;Mollusca;Bivalvia;Autobranchia;Pteriomorphia;Pect
inida;Pectinoidea;Pectinidae;Mizuhopecten;Mizuhopecten yessoensis
UdeM00000041308 6596 67.4 UdeM00000041308 XP_045211374.1 36.7 98
60 2 2 98 33 129 1.30E-08 67.4 6596 cellular
organisms;Eukaryota;Opisthokonta;Metazoa;Eumetazoa;Bilateria;Protostomia;
Spiralia;Lophotrochozoa;Mollusca;Bivalvia;Autobranchia;Heteroconchia;Euhe
terodonta;Imparidentia;Venerida;Veneroidea;Veneridae;Mercenaria;Mercenari
a mercenaria
UdeM00000041309 29159 368.6 UdeM00000041309 XP_019925641.2 57.1 352
141 3 44 390 33 379 1.20E-98 368.6 29159 cellular
organisms;Eukaryota;Opisthokonta;Metazoa;Eumetazoa;Bilateria;Protostomia;
Spiralia;Lophotrochozoa;Mollusca;Bivalvia;Autobranchia;Pteriomorphia;Ostr
eida;Ostreoidea;Ostreidae;Crassostrea;Crassostrea gigas
UdeM00000041310 6596 68.9 UdeM00000041310 XP_045210055.1 50 88
38 3 2 84 21 107 4.00E-08 68.9 6596 cellular
organisms;Eukaryota;Opisthokonta;Metazoa;Eumetazoa;Bilateria;Protostomia;
Spiralia;Lophotrochozoa;Mollusca;Bivalvia;Autobranchia;Heteroconchia;Euhe
terodonta;Imparidentia;Venerida;Veneroidea;Veneridae;Mercenaria;Mercenari
a mercenaria
UdeM00000041311 6596 109.4 UdeM00000041311 XP_045198590.1 34.4 209
123 7 265 466 431 632 1.30E-20 109.4 6596 cellular
organisms;Eukaryota;Opisthokonta;Metazoa;Eumetazoa;Bilateria;Protostomia;
Spiralia;Lophotrochozoa;Mollusca;Bivalvia;Autobranchia;Heteroconchia;Euhe

terodonta;Imparidentia;Venerida;Veneroidea;Veneridae;Mercenaria;Mercenaria mercenaria
UdeM00000041312 260615 159.5 UdeM00000041312 XP_039342393.1 67.4 144 21 2 5 122 382 525 2.80E-36 159.5 260615 cellular organisms;Eukaryota;Opisthokonta;Metazoa;Eumetazoa;Bilateria;Deuterostomia;Chordata;Craniata;Vertebrata;Gnathostomata;Teleostomi;Euteleostomi;Sarcopterygii;Dipnotetrapodomorpha;Tetrapoda;Amniota;Sauropsida;Sauria;Archelosauria;Testudinata;Testudines;Cryptodira;Durocryptodira;Testudinoidea;Geoemydidae;Geoemydinae;Mauremys;Mauremys reevesii
UdeM00000041313 260615 130.6 UdeM00000041313 XP_039342393.1 64.2 123 18 2 1 97 403 525 1.10E-27 130.6 260615 cellular organisms;Eukaryota;Opisthokonta;Metazoa;Eumetazoa;Bilateria;Deuterostomia;Chordata;Craniata;Vertebrata;Gnathostomata;Teleostomi;Euteleostomi;Sarcopterygii;Dipnotetrapodomorpha;Tetrapoda;Amniota;Sauropsida;Sauria;Archelosauria;Testudinata;Testudines;Cryptodira;Durocryptodira;Testudinoidea;Geoemydidae;Geoemydinae;Mauremys;Mauremys reevesii
UdeM00000041314 6573 396 UdeM00000041314 XP_021347801.1 69.8 404 109 6 1 400 1 395 2.10E-106 396 6573 cellular organisms;Eukaryota;Opisthokonta;Metazoa;Eumetazoa;Bilateria;Protostomia;Spiralia;Lophotrochozoa;Mollusca;Bivalvia;Autobranchia;Pteriomorphia;Pectinida;Pectinoidea;Pectinidae;Mizuhopecten;Mizuhopecten yessoensis
UdeM00000041315 6596 146.7 UdeM00000041315 XP_045192805.1 32.2 264 177 2 37 298 59 322 4.80E-32 146.7 6596 cellular organisms;Eukaryota;Opisthokonta;Metazoa;Eumetazoa;Bilateria;Protostomia;Spiralia;Lophotrochozoa;Mollusca;Bivalvia;Autobranchia;Heteroconchia;Euheterodonta;Imparidentia;Venerida;Veneroidea;Veneridae;Mercenaria;Mercenaria mercenaria
UdeM00000041316 29159 256.5 UdeM00000041316 XP_034316161.1 43.6 291 163 1 1 290 718 1008 4.00E-65 256.5 29159 cellular organisms;Eukaryota;Opisthokonta;Metazoa;Eumetazoa;Bilateria;Protostomia;Spiralia;Lophotrochozoa;Mollusca;Bivalvia;Autobranchia;Pteriomorphia;Ostreida;Ostreoidea;Ostreidae;Crassostrea;Crassostrea gigas
UdeM00000041319 1437191 144.4 UdeM00000041319 XP_029054816.1 43.2 162 89 2 1 159 302 463 1.30E-31 144.4 1437191 cellular organisms;Eukaryota;Opisthokonta;Metazoa;Eumetazoa;Bilateria;Protostomia;Ecdysozoa;Panarthropoda;Arthropoda;Mandibulata;Pancrustacea;Hexapoda;Insecta;Dicondylia;Pterygota;Neoptera;Endopterygota;Hymenoptera;Apocrita;Aculeata;Apoidea;Megachilidae;Megachilinae;Osmiini;Osmia;Osmia bicornis;Osmia bicornis bicornis
UdeM00000041320 1415580 143.7 UdeM00000041320 XP_030075314.1 46.4 153 81 1 1 153 1 152 2.00E-31 143.7 1415580 cellular organisms;Eukaryota;Opisthokonta;Metazoa;Eumetazoa;Bilateria;Deuterostomia;Chordata;Craniata;Vertebrata;Gnathostomata;Teleostomi;Euteleostomi;Sarcopterygii;Dipnotetrapodomorpha;Tetrapoda;Amphibia;Gymnophiona;Siphonopidae;Microcaecilia;Microcaecilia unicolor
UdeM00000041321 1415580 504.6 UdeM00000041321 XP_030075314.1 54.3 479 200 4 1 476 163 625 1.40E-139 504.6 1415580 cellular organisms;Eukaryota;Opisthokonta;Metazoa;Eumetazoa;Bilateria;Deuterostomia;Chordata;Craniata;Vertebrata;Gnathostomata;Teleostomi;Euteleostomi;Sarc

opterygii;Dipnotetrapodomorpha;Tetrapoda;Amphibia;Gymnophiona;Siphonopidae;Microcaecilia;Microcaecilia unicolor
UdeM00000041322 6596 152.1 UdeM00000041322 XP_045158316.1 48.3 145
66 6 40 182 177 314 6.70E-34 152.1 6596 cellular organisms;Eukaryota;Opisthokonta;Metazoa;Eumetazoa;Bilateria;Protostomia;Spiralia;Lophotrochozoa;Mollusca;Bivalvia;Autobranchia;Heteroconchia;Euheterodonta;Imparidentia;Venerida;Veneroidea;Veneridae;Mercenaria;Mercenaria mercenaria
UdeM00000041325 6565 1407.9 UdeM00000041325 XP_022329293.1 71.6
1117 291 8 1 1104 1 1104 0.00E+00 1407.9 6565
cellular organisms;Eukaryota;Opisthokonta;Metazoa;Eumetazoa;Bilateria;Protostomia;Spiralia;Lophotrochozoa;Mollusca;Bivalvia;Autobranchia;Pteriomorphia;Ostreida;Ostreoidea;Ostreidae;Crassostrea;Crassostrea virginica
UdeM00000041327 6596 90.9 UdeM00000041327 XP_045208010.1 70.4 81
18 2 1 80 1 76 8.50E-16 90.9 6596 cellular organisms;Eukaryota;Opisthokonta;Metazoa;Eumetazoa;Bilateria;Protostomia;Spiralia;Lophotrochozoa;Mollusca;Bivalvia;Autobranchia;Heteroconchia;Euheterodonta;Imparidentia;Venerida;Veneroidea;Veneridae;Mercenaria;Mercenaria mercenaria
UdeM00000041326 6573 282.3 UdeM00000041326 XP_021341993.1 76.2 214
45 2 1 212 1 210 5.00E-73 282.3 6573 cellular organisms;Eukaryota;Opisthokonta;Metazoa;Eumetazoa;Bilateria;Protostomia;Spiralia;Lophotrochozoa;Mollusca;Bivalvia;Autobranchia;Pteriomorphia;Pectinida;Pectinoidea;Pectinidae;Mizuhopecten;Mizuhopecten yessoensis
UdeM00000041328 29159 196.4 UdeM00000041328 XP_011450032.1 64.3 171
60 1 113 282 38 208 4.80E-47 196.4 29159 cellular organisms;Eukaryota;Opisthokonta;Metazoa;Eumetazoa;Bilateria;Protostomia;Spiralia;Lophotrochozoa;Mollusca;Bivalvia;Autobranchia;Pteriomorphia;Ostreida;Ostreoidea;Ostreidae;Crassostrea;Crassostrea gigas
UdeM00000041329 6596 1008.1 UdeM00000041329 XP_045209793.1 52.5
1060 487 8 2 1049 245 1299 1.60E-290 1008.1 6596
cellular organisms;Eukaryota;Opisthokonta;Metazoa;Eumetazoa;Bilateria;Protostomia;Spiralia;Lophotrochozoa;Mollusca;Bivalvia;Autobranchia;Heteroconchia;Euheterodonta;Imparidentia;Venerida;Veneroidea;Veneridae;Mercenaria;Mercenaria mercenaria
UdeM00000041332 6596 138.7 UdeM00000041332 XP_045209793.1 49.5 111
56 0 2 112 1188 1298 7.90E-30 138.7 6596 cellular organisms;Eukaryota;Opisthokonta;Metazoa;Eumetazoa;Bilateria;Protostomia;Spiralia;Lophotrochozoa;Mollusca;Bivalvia;Autobranchia;Heteroconchia;Euheterodonta;Imparidentia;Venerida;Veneroidea;Veneridae;Mercenaria;Mercenaria mercenaria
UdeM00000041333 6596 104.4 UdeM00000041333 XP_045209793.1 54.5 77
35 0 2 78 1188 1264 8.90E-20 104.4 6596 cellular organisms;Eukaryota;Opisthokonta;Metazoa;Eumetazoa;Bilateria;Protostomia;Spiralia;Lophotrochozoa;Mollusca;Bivalvia;Autobranchia;Heteroconchia;Euheterodonta;Imparidentia;Venerida;Veneroidea;Veneridae;Mercenaria;Mercenaria mercenaria
UdeM00000041334 6596 870.2 UdeM00000041334 XP_045209793.1 42 1149
547 14 26 1058 34 1179 2.80E-249 870.2 6596 cellular organisms;Eukaryota;Opisthokonta;Metazoa;Eumetazoa;Bilateria;Protostomia;Spiralia;Lophotrochozoa;Mollusca;Bivalvia;Autobranchia;Heteroconchia;Euhe

terodonta;Imparidentia;Venerida;Veneroidea;Veneridae;Mercenaria;Mercenaria mercenaria
UdeM00000041335 6596 1056.6 UdeM00000041335 XP_045209793.1 45.2 1285 636 14 79 1322 40 1297 2.60E-305 1056.6 6596 cellular organisms;Eukaryota;Opisthokonta;Metazoa;Eumetazoa;Bilateria;Protostomia;Spiralia;Lophotrochozoa;Mollusca;Bivalvia;Autobranchia;Heteroconchia;Euheterodonta;Imparidentia;Venerida;Veneroidea;Veneridae;Mercenaria;Mercenaria mercenaria
UdeM00000041336 6596 305.1 UdeM00000041336 XP_045208580.1 53.3 291 136 0 11 301 8 298 1.10E-79 305.1 6596 cellular organisms;Eukaryota;Opisthokonta;Metazoa;Eumetazoa;Bilateria;Protostomia;Spiralia;Lophotrochozoa;Mollusca;Bivalvia;Autobranchia;Heteroconchia;Euheterodonta;Imparidentia;Venerida;Veneroidea;Veneridae;Mercenaria;Mercenaria mercenaria
UdeM00000041337 6596 316.6 UdeM00000041337 XP_045208580.1 55.8 285 125 1 68 352 3 286 4.10E-83 316.6 6596 cellular organisms;Eukaryota;Opisthokonta;Metazoa;Eumetazoa;Bilateria;Protostomia;Spiralia;Lophotrochozoa;Mollusca;Bivalvia;Autobranchia;Heteroconchia;Euheterodonta;Imparidentia;Venerida;Veneroidea;Veneridae;Mercenaria;Mercenaria mercenaria
UdeM00000041338 6596 220.7 UdeM00000041338 XP_045193084.1 31.1 441 277 9 241 666 28 456 5.60E-54 220.7 6596 cellular organisms;Eukaryota;Opisthokonta;Metazoa;Eumetazoa;Bilateria;Protostomia;Spiralia;Lophotrochozoa;Mollusca;Bivalvia;Autobranchia;Heteroconchia;Euheterodonta;Imparidentia;Venerida;Veneroidea;Veneridae;Mercenaria;Mercenaria mercenaria
UdeM00000041339 29159 214.5 UdeM00000041339 XP_034312908.1 28.2 649 342 20 9 564 6 623 3.40E-52 214.5 29159 cellular organisms;Eukaryota;Opisthokonta;Metazoa;Eumetazoa;Bilateria;Protostomia;Spiralia;Lophotrochozoa;Mollusca;Bivalvia;Autobranchia;Pteriomorphia;Ostreida;Ostreoidea;Ostreidae;Crassostrea;Crassostrea gigas
UdeM00000041340 29159 73.9 UdeM00000041340 XP_034307878.1 32.3 99 67 0 1 99 92 190 3.70E-10 73.9 29159 cellular organisms;Eukaryota;Opisthokonta;Metazoa;Eumetazoa;Bilateria;Protostomia;Spiralia;Lophotrochozoa;Mollusca;Bivalvia;Autobranchia;Pteriomorphia;Ostreida;Ostreoidea;Ostreidae;Crassostrea;Crassostrea gigas
UdeM00000041343 7574 104.8 UdeM00000041343 XP_013418747.1 34 153 90 2 227 372 42 190 2.50E-19 104.8 7574 cellular organisms;Eukaryota;Opisthokonta;Metazoa;Eumetazoa;Bilateria;Protostomia;Spiralia;Lophotrochozoa;Brachiopoda;Linguliformea;Lingulata;Lingulida;Linguloidea;Lingulidae;Lingula;Lingula anatina
UdeM00000041345 7604 303.5 UdeM00000041345 XP_033633146.1 41 390 212 3 262 633 29 418 8.30E-79 303.5 7604 cellular organisms;Eukaryota;Opisthokonta;Metazoa;Eumetazoa;Bilateria;Deuterostomia;Echinodermata;Eleutherozoa;Asterozoa;Asteroidea;Forcipulatacea;Forcipulatida;Asteriidae;Asterias;Asterias rubens
UdeM00000041346 400727 92.8 UdeM00000041346 XP_025078952.1 24.1 493 340 13 40 512 66 544 1.40E-15 92.8 400727 cellular organisms;Eukaryota;Opisthokonta;Metazoa;Eumetazoa;Bilateria;Protostomia;Spiralia;Lophotrochozoa;Mollusca;Gastropoda;Caenogastropoda;Architaenioglossa;Ampullarioidea;Ampullariidae;Pomacea;Pomacea canaliculata

UdeM00000041347 400727 92 UdeM00000041347 XP_025078952.1 25.1
434 293 12 52 467 125 544 2.10E-15 92 400727
cellular
organisms;Eukaryota;Opisthokonta;Metazoa;Eumetazoa;Bilateria;Protostomia;Spiralia;Lophotrochozoa;Mollusca;Gastropoda;Caenogastropoda;Architaenioglossa;Ampullarioidea;Ampullariidae;Pomacea;Pomacea canaliculata
UdeM00000041349 6596 357.1 UdeM00000041349 XP_045201596.1 39.2 546
300 9 4 533 8 537 4.00E-95 357.1 6596 cellular
organisms;Eukaryota;Opisthokonta;Metazoa;Eumetazoa;Bilateria;Protostomia;Spiralia;Lophotrochozoa;Mollusca;Bivalvia;Autobranchia;Heteroconchia;Euheterodonta;Imparidentia;Venerida;Veneroidea;Veneridae;Mercenaria;Mercenaria mercenaria
UdeM00000041350 6596 330.1 UdeM00000041350 XP_045200182.1 64.1 231
83 0 1 231 16 246 2.40E-87 330.1 6596 cellular
organisms;Eukaryota;Opisthokonta;Metazoa;Eumetazoa;Bilateria;Protostomia;Spiralia;Lophotrochozoa;Mollusca;Bivalvia;Autobranchia;Heteroconchia;Euheterodonta;Imparidentia;Venerida;Veneroidea;Veneridae;Mercenaria;Mercenaria mercenaria
UdeM00000041351 6596 1094.7 UdeM00000041351 XP_045200187.1 64.5
857 271 9 6 839 7 853 0.00E+00 1094.7 6596
cellular
organisms;Eukaryota;Opisthokonta;Metazoa;Eumetazoa;Bilateria;Protostomia;Spiralia;Lophotrochozoa;Mollusca;Bivalvia;Autobranchia;Heteroconchia;Euheterodonta;Imparidentia;Venerida;Veneroidea;Veneridae;Mercenaria;Mercenaria mercenaria
UdeM00000041352 6596 172.9 UdeM00000041352 XP_045200185.1 62.2 156
51 4 1 155 733 881 3.10E-40 172.9 6596 cellular
organisms;Eukaryota;Opisthokonta;Metazoa;Eumetazoa;Bilateria;Protostomia;Spiralia;Lophotrochozoa;Mollusca;Bivalvia;Autobranchia;Heteroconchia;Euheterodonta;Imparidentia;Venerida;Veneroidea;Veneridae;Mercenaria;Mercenaria mercenaria
UdeM00000041353 6573 133.3 UdeM00000041353 XP_021356128.1 79.1 86
16 2 95 178 349 434 6.10E-28 133.3 6573 cellular
organisms;Eukaryota;Opisthokonta;Metazoa;Eumetazoa;Bilateria;Protostomia;Spiralia;Lophotrochozoa;Mollusca;Bivalvia;Autobranchia;Pteriomorphia;Pectinida;Pectinoidea;Pectinidae;Mizuhopecten;Mizuhopecten yessoensis
UdeM00000041354 105023 261.9 UdeM00000041354 XP_015828284.1 35.8
430 251 8 15 428 142 562 1.50E-66 261.9 105023
cellular
organisms;Eukaryota;Opisthokonta;Metazoa;Eumetazoa;Bilateria;Deuterostomia;Chordata;Craniata;Vertebrata;Gnathostomata;Teleostomi;Euteleostomi;Actinopterygii;Actinopteri;Neopterygii;Teleostei;Osteoglossocephalai;Clupeocephala;Euteleosteomorpha;Neoteleostei;Eurypterygia;Ctenosquamata;Acanthomorphata;Euacanthomorphacea;Percomorphaceae;Ovalentaria;Atherinomorphae;Cyprinodontiformes;Aplocheiloidei;Nothobranchiidae;Nothobranchius;Nothobranchius furzeri
UdeM00000041355 6565 250 UdeM00000041355 XP_022286673.1 58.5 234
75 7 1 225 1 221 3.10E-63 250 6565 cellular
organisms;Eukaryota;Opisthokonta;Metazoa;Eumetazoa;Bilateria;Protostomia;Spiralia;Lophotrochozoa;Mollusca;Bivalvia;Autobranchia;Pteriomorphia;Ostreida;Ostreoidea;Ostreidae;Crassostrea;Crassostrea virginica
UdeM00000041356 6596 594 UdeM00000041356 XP_045200058.1 65.2 494
122 11 1 474 1 464 1.90E-166 594 6596 cellular
organisms;Eukaryota;Opisthokonta;Metazoa;Eumetazoa;Bilateria;Protostomia;

Spiralia;Lophotrochozoa;Mollusca;Bivalvia;Autobranchia;Heteroconchia;Euheterodonta;Imparidentia;Venerida;Veneroidea;Veneridae;Mercenaria;Mercenaria mercenaria
UdeM00000041358 6573 175.3 UdeM00000041358 XP_021376567.1 93.3 89 6 0 1 89 476 564 4.20E-41 175.3 6573 cellular organisms;Eukaryota;Opisthokonta;Metazoa;Eumetazoa;Bilateria;Protostomia;Spiralia;Lophotrochozoa;Mollusca;Bivalvia;Autobranchia;Pteriomorphia;Pectinida;Pectinoidea;Pectinidae;Mizuhopecten;Mizuhopecten yessoensis
UdeM00000041359 6565 316.6 UdeM00000041359 XP_022286087.1 48.7 427 190 8 1 423 557 958 4.70E-83 316.6 6565 cellular organisms;Eukaryota;Opisthokonta;Metazoa;Eumetazoa;Bilateria;Protostomia;Spiralia;Lophotrochozoa;Mollusca;Bivalvia;Autobranchia;Pteriomorphia;Ostreida;Ostreoidea;Ostreidae;Crassostrea;Crassostrea virginica
UdeM00000041360 6573 140.6 UdeM00000041360 XP_021340634.1 47.1 170 74 3 4 157 2 171 1.70E-30 140.6 6573 cellular organisms;Eukaryota;Opisthokonta;Metazoa;Eumetazoa;Bilateria;Protostomia;Spiralia;Lophotrochozoa;Mollusca;Bivalvia;Autobranchia;Pteriomorphia;Pectinida;Pectinoidea;Pectinidae;Mizuhopecten;Mizuhopecten yessoensis
UdeM00000041361 6596 682.9 UdeM00000041361 XP_045201737.1 80.4 387 62 2 6 378 10 396 2.20E-193 682.9 6596 cellular organisms;Eukaryota;Opisthokonta;Metazoa;Eumetazoa;Bilateria;Protostomia;Spiralia;Lophotrochozoa;Mollusca;Bivalvia;Autobranchia;Heteroconchia;Euheterodonta;Imparidentia;Venerida;Veneroidea;Veneridae;Mercenaria;Mercenaria mercenaria
UdeM00000041362 6573 261.9 UdeM00000041362 XP_021365720.1 47.2 248 129 1 36 281 25 272 9.60E-67 261.9 6573 cellular organisms;Eukaryota;Opisthokonta;Metazoa;Eumetazoa;Bilateria;Protostomia;Spiralia;Lophotrochozoa;Mollusca;Bivalvia;Autobranchia;Pteriomorphia;Pectinida;Pectinoidea;Pectinidae;Mizuhopecten;Mizuhopecten yessoensis
UdeM00000041363 6596 570.1 UdeM00000041363 XP_045199403.1 61.7 515 156 7 12 493 111 617 2.80E-159 570.1 6596 cellular organisms;Eukaryota;Opisthokonta;Metazoa;Eumetazoa;Bilateria;Protostomia;Spiralia;Lophotrochozoa;Mollusca;Bivalvia;Autobranchia;Heteroconchia;Euheterodonta;Imparidentia;Venerida;Veneroidea;Veneridae;Mercenaria;Mercenaria mercenaria
UdeM00000041364 2607531 409.5 UdeM00000041364 XP_029648101.2 66.7 309 93 3 35 341 7 307 5.00E-111 409.5 2607531 cellular organisms;Eukaryota;Opisthokonta;Metazoa;Eumetazoa;Bilateria;Protostomia;Spiralia;Lophotrochozoa;Mollusca;Cephalopoda;Coleoidea;Octopodiformes;Octopoda;Incirrata;Octopodidae;Octopus;Octopus sinensis
UdeM00000041365 6596 1860.5 UdeM00000041365 XP_045201270.1 67.6 1417 425 13 88 1488 91 1489 0.00E+00 1860.5 6596 cellular organisms;Eukaryota;Opisthokonta;Metazoa;Eumetazoa;Bilateria;Protostomia;Spiralia;Lophotrochozoa;Mollusca;Bivalvia;Autobranchia;Heteroconchia;Euheterodonta;Imparidentia;Venerida;Veneroidea;Veneridae;Mercenaria;Mercenaria mercenaria
UdeM00000041366 7654 904.8 UdeM00000041366 XP_041454726.1 99.3 446 3 0 1 446 1 446 4.30E-260 904.8 7654 cellular organisms;Eukaryota;Opisthokonta;Metazoa;Eumetazoa;Bilateria;Deuterostomia;Echinodermata;Eleutherozoa;Echinozoa;Echinoidea;Euechinoidea;Echinacea;Temnopleuroida;Toxopneustidae;Lytechinus;Lytechinus variegatus

UdeM00000041367 7668 906 UdeM00000041367 XP_030840691.1 99.1 447
4 0 1 447 1 447 1.90E-260 906 7668 cellular
organisms;Eukaryota;Opisthokonta;Metazoa;Eumetazoa;Bilateria;Deuterostomi
a;Echinodermata;Eleutherozoa;Echinozoa;Echinoidea;Euechinoidea;Echinacea;
Camarodonta;Echinidea;Strongylocentrotidae;Strongylocentrotus;Strongyloce
ntrotus purpuratus
UdeM00000041368 400727 905.6 UdeM00000041368 XP_025114802.1 99.3
445 3 0 1 445 1 445 2.50E-260 905.6 400727
cellular
organisms;Eukaryota;Opisthokonta;Metazoa;Eumetazoa;Bilateria;Protostomia;
Spiralia;Lophotrochozoa;Mollusca;Gastropoda;Caenogastropoda;Architaeniogl
ossa;Ampullarioidea;Ampullariidae;Pomacea;Pomacea canaliculata
UdeM00000041369 7654 904.4 UdeM00000041369 XP_041454726.1 99.3 446
3 0 1 446 1 446 5.60E-260 904.4 7654 cellular
organisms;Eukaryota;Opisthokonta;Metazoa;Eumetazoa;Bilateria;Deuterostomi
a;Echinodermata;Eleutherozoa;Echinozoa;Echinoidea;Euechinoidea;Echinacea;
Temnopleuroida;Toxopneustidae;Lytechinus;Lytechinus variegatus
UdeM00000041370 7654 904.4 UdeM00000041370 XP_041454726.1 99.3 446
3 0 1 446 1 446 5.60E-260 904.4 7654 cellular
organisms;Eukaryota;Opisthokonta;Metazoa;Eumetazoa;Bilateria;Deuterostomi
a;Echinodermata;Eleutherozoa;Echinozoa;Echinoidea;Euechinoidea;Echinacea;
Temnopleuroida;Toxopneustidae;Lytechinus;Lytechinus variegatus
UdeM00000041371 6579 199.9 UdeM00000041371 XP_033761077.1 42.1 311
144 6 135 425 73 367 6.50E-48 199.9 6579 cellular
organisms;Eukaryota;Opisthokonta;Metazoa;Eumetazoa;Bilateria;Protostomia;
Spiralia;Lophotrochozoa;Mollusca;Bivalvia;Autobranchia;Pteriomorphia;Pect
inida;Pectinoidea;Pectinidae;Pecten;Pecten maximus
UdeM00000041374 33528 169.1 UdeM00000041374 XP_043972167.1 52.9 157
69 1 1 152 1 157 4.60E-39 169.1 33528 cellular
organisms;Eukaryota;Opisthokonta;Metazoa;Eumetazoa;Bilateria;Deuterostomi
a;Chordata;Craniata;Vertebrata;Gnathostomata;Teleostomi;Euteleostomi;Acti
nopterygii;Actinopteri;Neopterygii;Teleostei;Osteoglossocephalai;Clupeoce
phala;Euteleosteomorpha;Neoteleostei;Eurypterygia;Ctenosquamata;Acanthomo
rphata;Euacanthomorphacea;Percomorphaceae;Ovalentaria;Atherinomorphae;Cyp
rinodontiformes;Cyprinodontoidei;Poeciliidae;Poeciliinae;Gambusia;Gambusi
a affinis
UdeM00000041373 6596 482.3 UdeM00000041373 XP_045184035.1 48.3 476
233 4 1 464 1 475 7.20E-133 482.3 6596 cellular
organisms;Eukaryota;Opisthokonta;Metazoa;Eumetazoa;Bilateria;Protostomia;
Spiralia;Lophotrochozoa;Mollusca;Bivalvia;Autobranchia;Heteroconchia;Euhe
terodonta;Imparidentia;Venerida;Veneroidea;Veneridae;Mercenaria;Mercenari
a mercenaria
UdeM00000041375 6596 278.1 UdeM00000041375 XP_045184035.1 50.6 253
120 2 2 250 224 475 1.10E-71 278.1 6596 cellular
organisms;Eukaryota;Opisthokonta;Metazoa;Eumetazoa;Bilateria;Protostomia;
Spiralia;Lophotrochozoa;Mollusca;Bivalvia;Autobranchia;Heteroconchia;Euhe
terodonta;Imparidentia;Venerida;Veneroidea;Veneridae;Mercenaria;Mercenari
a mercenaria
UdeM00000041376 6579 136.7 UdeM00000041376 XP_033761231.1 54.2 107
48 1 14 119 515 621 2.00E-29 136.7 6579 cellular
organisms;Eukaryota;Opisthokonta;Metazoa;Eumetazoa;Bilateria;Protostomia;
Spiralia;Lophotrochozoa;Mollusca;Bivalvia;Autobranchia;Pteriomorphia;Pect
inida;Pectinoidea;Pectinidae;Pecten;Pecten maximus

UdeM00000041377 6596 720.3 UdeM00000041377 XP_045201172.1 65.4 503
170 3 11 513 13 511 1.70E-204 720.3 6596 cellular organisms;Eukaryota;Opisthokonta;Metazoa;Eumetazoa;Bilateria;Protostomia;Spiralia;Lophotrochozoa;Mollusca;Bivalvia;Autobranchia;Heteroconchia;Euheterodonta;Imparidentia;Venerida;Veneroidea;Veneridae;Mercenaria;Mercenaria mercenaria
UdeM00000041378 8090 235 UdeM00000041378 XP_023809151.1 42.6 345
160 10 50 377 15 338 1.60E-58 235 8090 cellular organisms;Eukaryota;Opisthokonta;Metazoa;Eumetazoa;Bilateria;Deuterostomia;Chordata;Craniata;Vertebrata;Gnathostomata;Teleostomi;Euteleostomi;Actinopterygii;Actinopteri;Neopterygii;Teleostei;Osteoglossocephalai;Clupeocephala;Euteleosteomorpha;Neoteleostei;Eurypterygia;Ctenosquamata;Acanthomorphata;Euacanthomorphacea;Percomorphaceae;Ovalentaria;Atherinomorphae;Beloniformes;Adrianichthyoidei;Adrianichthyidae;Oryziinae;Oryzias;Oryzias latipes
UdeM00000041379 1735272 201.1 UdeM00000041379 XP_041359434.1 89.7
136 10 1 1 132 1 136 9.10E-49 201.1 1735272
cellular organisms;Eukaryota;Opisthokonta;Metazoa;Eumetazoa;Bilateria;Protostomia;Spiralia;Lophotrochozoa;Mollusca;Gastropoda;Neomphalina;Neomphaloidea;Peltospiridae;Gigantopelta;Gigantopelta aegis
UdeM00000041380 6596 1053.1 UdeM00000041380 XP_045201777.1 36.5
1816 989 43 468 2161 1 1773 4.70E-304 1053.1 6596
cellular organisms;Eukaryota;Opisthokonta;Metazoa;Eumetazoa;Bilateria;Protostomia;Spiralia;Lophotrochozoa;Mollusca;Bivalvia;Autobranchia;Heteroconchia;Euheterodonta;Imparidentia;Venerida;Veneroidea;Veneridae;Mercenaria;Mercenaria mercenaria
UdeM00000041382 6596 862.8 UdeM00000041382 XP_045202682.1 37 1386
791 22 6 1366 238 1566 5.70E-247 862.8 6596 cellular organisms;Eukaryota;Opisthokonta;Metazoa;Eumetazoa;Bilateria;Protostomia;Spiralia;Lophotrochozoa;Mollusca;Bivalvia;Autobranchia;Heteroconchia;Euheterodonta;Imparidentia;Venerida;Veneroidea;Veneridae;Mercenaria;Mercenaria mercenaria
UdeM00000041383 6573 885.9 UdeM00000041383 XP_021377155.1 64.8 648
226 2 1 648 1 646 3.00E-254 885.9 6573 cellular organisms;Eukaryota;Opisthokonta;Metazoa;Eumetazoa;Bilateria;Protostomia;Spiralia;Lophotrochozoa;Mollusca;Bivalvia;Autobranchia;Pteriomorphia;Pectinida;Pectinoidea;Pectinidae;Mizuhopecten;Mizuhopecten yessoensis
UdeM00000041384 6565 134.4 UdeM00000041384 XP_022344344.1 34.7 219
130 8 642 856 159 368 6.80E-28 134.4 6565 cellular organisms;Eukaryota;Opisthokonta;Metazoa;Eumetazoa;Bilateria;Protostomia;Spiralia;Lophotrochozoa;Mollusca;Bivalvia;Autobranchia;Pteriomorphia;Ostreida;Ostreoidea;Ostreidae;Crassostrea;Crassostrea virginica
UdeM00000041385 6579 788.5 UdeM00000041385 XP_033759707.1 80.4 759
126 4 4 759 39 777 7.60E-225 788.5 6579 cellular organisms;Eukaryota;Opisthokonta;Metazoa;Eumetazoa;Bilateria;Protostomia;Spiralia;Lophotrochozoa;Mollusca;Bivalvia;Autobranchia;Pteriomorphia;Pectinida;Pectinoidea;Pectinidae;Pecten;Pecten maximus
UdeM00000041386 6596 1294.6 UdeM00000041386 XP_045201515.1 82.4
888 153 1 1 885 1 888 0.00E+00 1294.6 6596
cellular organisms;Eukaryota;Opisthokonta;Metazoa;Eumetazoa;Bilateria;Protostomia;Spiralia;Lophotrochozoa;Mollusca;Bivalvia;Autobranchia;Heteroconchia;Euhe

terodonta;Imparidentia;Venerida;Veneroidea;Veneridae;Mercenaria;Mercenaria mercenaria
UdeM00000041387 6579 85.1 UdeM00000041387 XP_033759354.1 32.8 122 72 1 10 131 7 118 1.60E-13 85.1 6579 cellular organisms;Eukaryota;Opisthokonta;Metazoa;Eumetazoa;Bilateria;Protostomia;Spiralia;Lophotrochozoa;Mollusca;Bivalvia;Autobranchia;Pteriomorphia;Pectinida;Pectinoidea;Pectinidae;Pecten;Pecten maximus
UdeM00000041388 6596 291.6 UdeM00000041388 XP_045199971.1 59.1 237 97 0 1 237 1 237 9.30E-76 291.6 6596 cellular organisms;Eukaryota;Opisthokonta;Metazoa;Eumetazoa;Bilateria;Protostomia;Spiralia;Lophotrochozoa;Mollusca;Bivalvia;Autobranchia;Heteroconchia;Euheterodonta;Imparidentia;Venerida;Veneroidea;Veneridae;Mercenaria;Mercenaria mercenaria
UdeM00000041389 6579 419.5 UdeM00000041389 XP_033760370.1 67.2 290 90 2 1 285 78 367 3.50E-114 419.5 6579 cellular organisms;Eukaryota;Opisthokonta;Metazoa;Eumetazoa;Bilateria;Protostomia;Spiralia;Lophotrochozoa;Mollusca;Bivalvia;Autobranchia;Pteriomorphia;Pectinida;Pectinoidea;Pectinidae;Pecten;Pecten maximus
UdeM00000041390 6596 154.5 UdeM00000041390 XP_045195108.1 48.8 160 76 2 1 155 231 389 3.20E-34 154.5 6596 cellular organisms;Eukaryota;Opisthokonta;Metazoa;Eumetazoa;Bilateria;Protostomia;Spiralia;Lophotrochozoa;Mollusca;Bivalvia;Autobranchia;Heteroconchia;Euheterodonta;Imparidentia;Venerida;Veneroidea;Veneridae;Mercenaria;Mercenaria mercenaria
UdeM00000041392 6596 547.4 UdeM00000041392 XP_045200407.1 68 394 125 1 1 394 2221 2613 1.50E-152 547.4 6596 cellular organisms;Eukaryota;Opisthokonta;Metazoa;Eumetazoa;Bilateria;Protostomia;Spiralia;Lophotrochozoa;Mollusca;Bivalvia;Autobranchia;Heteroconchia;Euheterodonta;Imparidentia;Venerida;Veneroidea;Veneridae;Mercenaria;Mercenaria mercenaria
UdeM00000041393 6596 1749.2 UdeM00000041393 XP_045200407.1 61.7 1472 475 11 1 1395 754 2213 0.00E+00 1749.2 6596 cellular organisms;Eukaryota;Opisthokonta;Metazoa;Eumetazoa;Bilateria;Protostomia;Spiralia;Lophotrochozoa;Mollusca;Bivalvia;Autobranchia;Heteroconchia;Euheterodonta;Imparidentia;Venerida;Veneroidea;Veneridae;Mercenaria;Mercenaria mercenaria
UdeM00000041394 6579 272.3 UdeM00000041394 XP_033730119.1 66.3 190 63 1 1 189 471 660 4.60E-70 272.3 6579 cellular organisms;Eukaryota;Opisthokonta;Metazoa;Eumetazoa;Bilateria;Protostomia;Spiralia;Lophotrochozoa;Mollusca;Bivalvia;Autobranchia;Pteriomorphia;Pectinida;Pectinoidea;Pectinidae;Pecten;Pecten maximus
UdeM00000041395 114398 432.2 UdeM00000041395 XP_042903861.1 39 605 358 6 5 605 5 602 1.10E-117 432.2 114398 cellular organisms;Eukaryota;Opisthokonta;Metazoa;Eumetazoa;Bilateria;Protostomia;Ecdysozoa;Panarthropoda;Arthropoda;Chelicerata;Arachnida;Araneae;Araneomorphae;Entelegynae;Orbiculariae;Araneoidea;Theridiidae;Parasteatoda;Parasteatoda tepidariorum
UdeM00000041396 6596 767.3 UdeM00000041396 XP_045200407.1 73 519 131 1 3 512 4 522 1.20E-218 767.3 6596 cellular organisms;Eukaryota;Opisthokonta;Metazoa;Eumetazoa;Bilateria;Protostomia;Spiralia;Lophotrochozoa;Mollusca;Bivalvia;Autobranchia;Heteroconchia;Euhe

terodonta;Imparidentia;Venerida;Veneroidea;Veneridae;Mercenaria;Mercenari
a mercenaria
UdeM00000041397 6596 79.7 UdeM00000041397 XP_045199380.1 46.6 88
46 1 29 115 1108 1195 2.70E-12 79.7 6596 cellular
organisms;Eukaryota;Opisthokonta;Metazoa;Eumetazoa;Bilateria;Protostomia;
Spiralia;Lophotrochozoa;Mollusca;Bivalvia;Autobranchia;Heteroconchia;Euhe
terodonta;Imparidentia;Venerida;Veneroidea;Veneridae;Mercenaria;Mercenari
a mercenaria
UdeM00000041398 6596 74.7 UdeM00000041398 XP_045199380.1 39.5 124
73 2 18 139 1072 1195 1.00E-10 74.7 6596 cellular
organisms;Eukaryota;Opisthokonta;Metazoa;Eumetazoa;Bilateria;Protostomia;
Spiralia;Lophotrochozoa;Mollusca;Bivalvia;Autobranchia;Heteroconchia;Euhe
terodonta;Imparidentia;Venerida;Veneroidea;Veneridae;Mercenaria;Mercenari
a mercenaria
UdeM00000041399 6596 74.7 UdeM00000041399 XP_045199380.1 39.5 124
73 2 18 139 1072 1195 1.00E-10 74.7 6596 cellular
organisms;Eukaryota;Opisthokonta;Metazoa;Eumetazoa;Bilateria;Protostomia;
Spiralia;Lophotrochozoa;Mollusca;Bivalvia;Autobranchia;Heteroconchia;Euhe
terodonta;Imparidentia;Venerida;Veneroidea;Veneridae;Mercenaria;Mercenari
a mercenaria
UdeM00000041400 6596 74.7 UdeM00000041400 XP_045199380.1 39.5 124
73 2 18 139 1072 1195 1.00E-10 74.7 6596 cellular
organisms;Eukaryota;Opisthokonta;Metazoa;Eumetazoa;Bilateria;Protostomia;
Spiralia;Lophotrochozoa;Mollusca;Bivalvia;Autobranchia;Heteroconchia;Euhe
terodonta;Imparidentia;Venerida;Veneroidea;Veneridae;Mercenaria;Mercenari
a mercenaria
UdeM00000041401 29159 274.6 UdeM00000041401 XP_011438294.2 59.9 207
82 1 1 206 1 207 1.20E-69 274.6 29159 cellular
organisms;Eukaryota;Opisthokonta;Metazoa;Eumetazoa;Bilateria;Protostomia;
Spiralia;Lophotrochozoa;Mollusca;Bivalvia;Autobranchia;Pteriomorphia;Ostr
eida;Ostreoidea;Ostreidae;Crassostrea;Crassostrea gigas
UdeM00000041403 6573 209.1 UdeM00000041403 XP_021340400.1 44.2 231
123 3 1 231 493 717 6.80E-51 209.1 6573 cellular
organisms;Eukaryota;Opisthokonta;Metazoa;Eumetazoa;Bilateria;Protostomia;
Spiralia;Lophotrochozoa;Mollusca;Bivalvia;Autobranchia;Pteriomorphia;Pect
inida;Pectinoidea;Pectinidae;Mizuhopecten;Mizuhopecten yessoensis
UdeM00000041405 6579 283.9 UdeM00000041405 XP_033759954.1 39.8 405
230 7 2 400 77 473 3.40E-73 283.9 6579 cellular
organisms;Eukaryota;Opisthokonta;Metazoa;Eumetazoa;Bilateria;Protostomia;
Spiralia;Lophotrochozoa;Mollusca;Bivalvia;Autobranchia;Pteriomorphia;Pect
inida;Pectinoidea;Pectinidae;Pecten;Pecten maximus
UdeM00000041407 6573 600.5 UdeM00000041407 XP_021340400.1 38.2 814
477 13 34 837 35 832 3.60E-168 600.5 6573 cellular
organisms;Eukaryota;Opisthokonta;Metazoa;Eumetazoa;Bilateria;Protostomia;
Spiralia;Lophotrochozoa;Mollusca;Bivalvia;Autobranchia;Pteriomorphia;Pect
inida;Pectinoidea;Pectinidae;Mizuhopecten;Mizuhopecten yessoensis
UdeM00000041408 6573 600.5 UdeM00000041408 XP_021340400.1 38.2 814
477 13 34 837 35 832 3.60E-168 600.5 6573 cellular
organisms;Eukaryota;Opisthokonta;Metazoa;Eumetazoa;Bilateria;Protostomia;
Spiralia;Lophotrochozoa;Mollusca;Bivalvia;Autobranchia;Pteriomorphia;Pect
inida;Pectinoidea;Pectinidae;Mizuhopecten;Mizuhopecten yessoensis
UdeM00000041409 6573 608.2 UdeM00000041409 XP_021340400.1 38.6 819
470 15 34 840 35 832 1.70E-170 608.2 6573 cellular
organisms;Eukaryota;Opisthokonta;Metazoa;Eumetazoa;Bilateria;Protostomia;

Spiralia;Lophotrochozoa;Mollusca;Bivalvia;Autobranchia;Pteriomorphia;Pectinida;Pectinoidea;Pectinidae;Mizuhopecten;Mizuhopecten yessoensis
UdeM00000041410 6573 325.9 UdeM00000041410 XP_021376733.1 40.9 484 266 11 1 475 466 938 1.10E-85 325.9 6573 cellular organisms;Eukaryota;Opisthokonta;Metazoa;Eumetazoa;Bilateria;Protostomia;Spiralia;Lophotrochozoa;Mollusca;Bivalvia;Autobranchia;Pteriomorphia;Pectinida;Pectinoidea;Pectinidae;Mizuhopecten;Mizuhopecten yessoensis
UdeM00000041411 6573 149.4 UdeM00000041411 XP_021340400.1 34.5 275 155 8 36 301 41 299 7.10E-33 149.4 6573 cellular organisms;Eukaryota;Opisthokonta;Metazoa;Eumetazoa;Bilateria;Protostomia;Spiralia;Lophotrochozoa;Mollusca;Bivalvia;Autobranchia;Pteriomorphia;Pectinida;Pectinoidea;Pectinidae;Mizuhopecten;Mizuhopecten yessoensis
UdeM00000041412 6573 557.8 UdeM00000041412 XP_021340400.1 38.7 856 448 21 30 837 36 862 3.00E-155 557.8 6573 cellular organisms;Eukaryota;Opisthokonta;Metazoa;Eumetazoa;Bilateria;Protostomia;Spiralia;Lophotrochozoa;Mollusca;Bivalvia;Autobranchia;Pteriomorphia;Pectinida;Pectinoidea;Pectinidae;Mizuhopecten;Mizuhopecten yessoensis
UdeM00000041413 6573 614.8 UdeM00000041413 XP_021340400.1 40.8 856 466 20 30 873 36 862 2.10E-172 614.8 6573 cellular organisms;Eukaryota;Opisthokonta;Metazoa;Eumetazoa;Bilateria;Protostomia;Spiralia;Lophotrochozoa;Mollusca;Bivalvia;Autobranchia;Pteriomorphia;Pectinida;Pectinoidea;Pectinidae;Mizuhopecten;Mizuhopecten yessoensis
UdeM00000041414 6573 320.9 UdeM00000041414 XP_021376735.1 43 395 194 8 18 396 553 932 4.30E-84 320.9 6573 cellular organisms;Eukaryota;Opisthokonta;Metazoa;Eumetazoa;Bilateria;Protostomia;Spiralia;Lophotrochozoa;Mollusca;Bivalvia;Autobranchia;Pteriomorphia;Pectinida;Pectinoidea;Pectinidae;Mizuhopecten;Mizuhopecten yessoensis
UdeM00000041415 6573 371.3 UdeM00000041415 XP_021340400.1 38.2 568 319 13 14 568 14 562 2.20E-99 371.3 6573 cellular organisms;Eukaryota;Opisthokonta;Metazoa;Eumetazoa;Bilateria;Protostomia;Spiralia;Lophotrochozoa;Mollusca;Bivalvia;Autobranchia;Pteriomorphia;Pectinida;Pectinoidea;Pectinidae;Mizuhopecten;Mizuhopecten yessoensis
UdeM00000041416 6596 145.6 UdeM00000041416 XP_045201682.1 68.4 98 27 1 12 105 4 101 3.80E-32 145.6 6596 cellular organisms;Eukaryota;Opisthokonta;Metazoa;Eumetazoa;Bilateria;Protostomia;Spiralia;Lophotrochozoa;Mollusca;Bivalvia;Autobranchia;Heteroconchia;Euheterodonta;Imparidentia;Venerida;Veneroidea;Veneridae;Mercenaria;Mercenaria mercenaria
UdeM00000041417 6596 145.6 UdeM00000041417 XP_045201682.1 68.4 98 27 1 11 104 4 101 3.80E-32 145.6 6596 cellular organisms;Eukaryota;Opisthokonta;Metazoa;Eumetazoa;Bilateria;Protostomia;Spiralia;Lophotrochozoa;Mollusca;Bivalvia;Autobranchia;Heteroconchia;Euheterodonta;Imparidentia;Venerida;Veneroidea;Veneridae;Mercenaria;Mercenaria mercenaria
UdeM00000041418 45264 85.9 UdeM00000041418 XP_044167111.1 57.9 57 24 0 5 61 6 62 3.70E-14 85.9 45264 cellular organisms;Eukaryota;Opisthokonta;Metazoa;Eumetazoa;Cnidaria;Anthozoa;Hexacorallia;Scleractinia;Astrocoeniina;Acroporidae;Acropora;Acropora millepora
UdeM00000041419 6573 1049.3 UdeM00000041419 XP_021351493.1 56.4 1060 375 22 1 1030 164 1166 3.20E-303 1049.3 6573 cellular organisms;Eukaryota;Opisthokonta;Metazoa;Eumetazoa;Bilateria;Protostomia;

Spiralia;Lophotrochozoa;Mollusca;Bivalvia;Autobranchia;Pteriomorphia;Pectinida;Pectinoidea;Pectinidae;Mizuhopecten;Mizuhopecten yessoensis
UdeM00000041420 29159 723.8 UdeM00000041420 XP_011422754.3 55.9 637 152 4 1 632 70 582 1.90E-205 723.8 29159 cellular organisms;Eukaryota;Opisthokonta;Metazoa;Eumetazoa;Bilateria;Protostomia;Spiralia;Lophotrochozoa;Mollusca;Bivalvia;Autobranchia;Pteriomorphia;Ostreida;Ostreoidea;Ostreidae;Crassostrea;Crassostrea gigas
UdeM00000041421 6596 209.1 UdeM00000041421 XP_045201068.1 42.6 272 133 9 1 262 1 259 6.60E-51 209.1 6596 cellular organisms;Eukaryota;Opisthokonta;Metazoa;Eumetazoa;Bilateria;Protostomia;Spiralia;Lophotrochozoa;Mollusca;Bivalvia;Autobranchia;Heteroconchia;Euheterodonta;Imparidentia;Venerida;Veneroidea;Veneridae;Mercenaria;Mercenaria mercenaria
UdeM00000041422 6565 134.4 UdeM00000041422 XP_022299727.1 31 248 128 4 1 207 460 705 1.60E-28 134.4 6565 cellular organisms;Eukaryota;Opisthokonta;Metazoa;Eumetazoa;Bilateria;Protostomia;Spiralia;Lophotrochozoa;Mollusca;Bivalvia;Autobranchia;Pteriomorphia;Ostreida;Ostreoidea;Ostreidae;Crassostrea;Crassostrea virginica
UdeM00000041423 6579 157.5 UdeM00000041423 XP_033726657.1 57.8 128 53 1 1 127 270 397 1.10E-35 157.5 6579 cellular organisms;Eukaryota;Opisthokonta;Metazoa;Eumetazoa;Bilateria;Protostomia;Spiralia;Lophotrochozoa;Mollusca;Bivalvia;Autobranchia;Pteriomorphia;Pectinida;Pectinoidea;Pectinidae;Pecten;Pecten maximus
UdeM00000041424 6565 142.1 UdeM00000041424 XP_022299727.1 44.9 147 81 0 1 147 106 252 5.60E-31 142.1 6565 cellular organisms;Eukaryota;Opisthokonta;Metazoa;Eumetazoa;Bilateria;Protostomia;Spiralia;Lophotrochozoa;Mollusca;Bivalvia;Autobranchia;Pteriomorphia;Ostreida;Ostreoidea;Ostreidae;Crassostrea;Crassostrea virginica
UdeM00000041425 6596 283.9 UdeM00000041425 XP_045200285.1 28 703 441 16 5 657 19 706 5.50E-73 283.9 6596 cellular organisms;Eukaryota;Opisthokonta;Metazoa;Eumetazoa;Bilateria;Protostomia;Spiralia;Lophotrochozoa;Mollusca;Bivalvia;Autobranchia;Heteroconchia;Euheterodonta;Imparidentia;Venerida;Veneroidea;Veneridae;Mercenaria;Mercenaria mercenaria
UdeM00000041426 6565 112.8 UdeM00000041426 XP_022328252.1 37.9 211 78 5 1 158 1105 1315 3.90E-22 112.8 6565 cellular organisms;Eukaryota;Opisthokonta;Metazoa;Eumetazoa;Bilateria;Protostomia;Spiralia;Lophotrochozoa;Mollusca;Bivalvia;Autobranchia;Pteriomorphia;Ostreida;Ostreoidea;Ostreidae;Crassostrea;Crassostrea virginica
UdeM00000041427 6596 174.5 UdeM00000041427 XP_045200267.1 38 274 149 7 7 277 445 700 2.00E-40 174.5 6596 cellular organisms;Eukaryota;Opisthokonta;Metazoa;Eumetazoa;Bilateria;Protostomia;Spiralia;Lophotrochozoa;Mollusca;Bivalvia;Autobranchia;Heteroconchia;Euheterodonta;Imparidentia;Venerida;Veneroidea;Veneridae;Mercenaria;Mercenaria mercenaria
UdeM00000041428 6596 223.8 UdeM00000041428 XP_045200267.1 36.4 341 202 7 2 334 15 348 3.40E-55 223.8 6596 cellular organisms;Eukaryota;Opisthokonta;Metazoa;Eumetazoa;Bilateria;Protostomia;Spiralia;Lophotrochozoa;Mollusca;Bivalvia;Autobranchia;Heteroconchia;Euheterodonta;Imparidentia;Venerida;Veneroidea;Veneridae;Mercenaria;Mercenaria mercenaria
UdeM00000041429 6596 406 UdeM00000041429 XP_045200267.1 35.5 713 416 17 2 697 15 700 9.90E-110 406 6596 cellular organisms;Eukaryota;Opisthokonta;Metazoa;Eumetazoa;Bilateria;Protostomia;

Spiralia;Lophotrochozoa;Mollusca;Bivalvia;Autobranchia;Heteroconchia;Euheterodonta;Imparidentia;Venerida;Veneroidea;Veneridae;Mercenaria;Mercenaria mercenaria
UdeM00000041430 7654 154.8 UdeM00000041430 XP_041463467.1 34.4 308 137 8 21 265 137 442 1.60E-34 154.8 7654 cellular organisms;Eukaryota;Opisthokonta;Metazoa;Eumetazoa;Bilateria;Deuterostomia;Echinodermata;Eleutherozoa;Echinozoa;Echinoidea;Euechinoidea;Echinacea;Temnopleuroida;Toxopneustidae;Lytechinus;Lytechinus variegatus
UdeM00000041431 50429 76.3 UdeM00000041431 XP_022790680.1 47.2 72 37 1 3 74 40 110 4.30E-11 76.3 50429 cellular organisms;Eukaryota;Opisthokonta;Metazoa;Eumetazoa;Cnidaria;Anthozoa;Hexacorallia;Scleractinia;Astrocoeniina;Pocilloporidae;Stylophora;Stylophora pistillata
UdeM00000041432 6596 378.6 UdeM00000041432 XP_045200267.1 34 711 424 18 9 701 22 705 1.70E-101 378.6 6596 cellular organisms;Eukaryota;Opisthokonta;Metazoa;Eumetazoa;Bilateria;Protostomia;Spiralia;Lophotrochozoa;Mollusca;Bivalvia;Autobranchia;Heteroconchia;Euheterodonta;Imparidentia;Venerida;Veneroidea;Veneridae;Mercenaria;Mercenaria mercenaria
UdeM00000041433 6596 291.6 UdeM00000041433 XP_045200285.1 28.8 715 435 21 9 675 19 707 2.70E-75 291.6 6596 cellular organisms;Eukaryota;Opisthokonta;Metazoa;Eumetazoa;Bilateria;Protostomia;Spiralia;Lophotrochozoa;Mollusca;Bivalvia;Autobranchia;Heteroconchia;Euheterodonta;Imparidentia;Venerida;Veneroidea;Veneridae;Mercenaria;Mercenaria mercenaria
UdeM00000041434 6596 77.4 UdeM00000041434 XP_045200280.1 31.9 138 90 3 7 143 539 673 1.90E-11 77.4 6596 cellular organisms;Eukaryota;Opisthokonta;Metazoa;Eumetazoa;Bilateria;Protostomia;Spiralia;Lophotrochozoa;Mollusca;Bivalvia;Autobranchia;Heteroconchia;Euheterodonta;Imparidentia;Venerida;Veneroidea;Veneridae;Mercenaria;Mercenaria mercenaria
UdeM00000041435 6596 376.7 UdeM00000041435 XP_045200267.1 33.9 711 425 18 9 701 22 705 6.40E-101 376.7 6596 cellular organisms;Eukaryota;Opisthokonta;Metazoa;Eumetazoa;Bilateria;Protostomia;Spiralia;Lophotrochozoa;Mollusca;Bivalvia;Autobranchia;Heteroconchia;Euheterodonta;Imparidentia;Venerida;Veneroidea;Veneridae;Mercenaria;Mercenaria mercenaria
UdeM00000041436 6596 289.7 UdeM00000041436 XP_045200285.1 28.6 717 436 21 9 677 19 707 1.00E-74 289.7 6596 cellular organisms;Eukaryota;Opisthokonta;Metazoa;Eumetazoa;Bilateria;Protostomia;Spiralia;Lophotrochozoa;Mollusca;Bivalvia;Autobranchia;Heteroconchia;Euheterodonta;Imparidentia;Venerida;Veneroidea;Veneridae;Mercenaria;Mercenaria mercenaria
UdeM00000041437 6596 77.4 UdeM00000041437 XP_045200280.1 31.9 138 90 3 7 143 539 673 1.90E-11 77.4 6596 cellular organisms;Eukaryota;Opisthokonta;Metazoa;Eumetazoa;Bilateria;Protostomia;Spiralia;Lophotrochozoa;Mollusca;Bivalvia;Autobranchia;Heteroconchia;Euheterodonta;Imparidentia;Venerida;Veneroidea;Veneridae;Mercenaria;Mercenaria mercenaria
UdeM00000041438 6596 375.9 UdeM00000041438 XP_045200267.1 33.3 709 432 17 9 701 22 705 1.10E-100 375.9 6596 cellular organisms;Eukaryota;Opisthokonta;Metazoa;Eumetazoa;Bilateria;Protostomia;Spiralia;Lophotrochozoa;Mollusca;Bivalvia;Autobranchia;Heteroconchia;Euhe

terodonta;Imparidentia;Venerida;Veneroidea;Veneridae;Mercenaria;Mercenaria mercenaria
UdeM00000041440 6596 295 UdeM00000041440 XP_045200285.1 27.7 725
436 20 9 681 19 707 2.50E-76 295 6596 cellular organisms;Eukaryota;Opisthokonta;Metazoa;Eumetazoa;Bilateria;Protostomia;Spiralia;Lophotrochozoa;Mollusca;Bivalvia;Autobranchia;Heteroconchia;Euheterodonta;Imparidentia;Venerida;Veneroidea;Veneridae;Mercenaria;Mercenaria mercenaria
UdeM00000041441 6596 379.8 UdeM00000041441 XP_045200267.1 35 703
410 18 9 690 24 700 7.50E-102 379.8 6596 cellular organisms;Eukaryota;Opisthokonta;Metazoa;Eumetazoa;Bilateria;Protostomia;Spiralia;Lophotrochozoa;Mollusca;Bivalvia;Autobranchia;Heteroconchia;Euheterodonta;Imparidentia;Venerida;Veneroidea;Veneridae;Mercenaria;Mercenaria mercenaria
UdeM00000041442 6596 386.7 UdeM00000041442 XP_045200267.1 34.9 702
412 18 9 690 24 700 6.20E-104 386.7 6596 cellular organisms;Eukaryota;Opisthokonta;Metazoa;Eumetazoa;Bilateria;Protostomia;Spiralia;Lophotrochozoa;Mollusca;Bivalvia;Autobranchia;Heteroconchia;Euheterodonta;Imparidentia;Venerida;Veneroidea;Veneridae;Mercenaria;Mercenaria mercenaria
UdeM00000041444 6596 498.4 UdeM00000041444 XP_045199728.1 63.1 366
134 1 225 590 49 413 1.30E-137 498.4 6596 cellular organisms;Eukaryota;Opisthokonta;Metazoa;Eumetazoa;Bilateria;Protostomia;Spiralia;Lophotrochozoa;Mollusca;Bivalvia;Autobranchia;Heteroconchia;Euheterodonta;Imparidentia;Venerida;Veneroidea;Veneridae;Mercenaria;Mercenaria mercenaria
UdeM00000041445 29159 534.6 UdeM00000041445 XP_034329719.1 74.1 347
89 1 3 348 620 966 9.10E-149 534.6 29159 cellular organisms;Eukaryota;Opisthokonta;Metazoa;Eumetazoa;Bilateria;Protostomia;Spiralia;Lophotrochozoa;Mollusca;Bivalvia;Autobranchia;Pteriomorphia;Ostreida;Ostreoidea;Ostreidae;Crassostrea;Crassostrea gigas
UdeM00000041446 6573 280 UdeM00000041446 XP_021375565.1 68.2 201
59 2 59 257 395 592 3.20E-72 280 6573 cellular organisms;Eukaryota;Opisthokonta;Metazoa;Eumetazoa;Bilateria;Protostomia;Spiralia;Lophotrochozoa;Mollusca;Bivalvia;Autobranchia;Pteriomorphia;Pectinida;Pectinoidea;Pectinidae;Mizuhopecten;Mizuhopecten yessoensis
UdeM00000041447 6596 534.6 UdeM00000041447 XP_045202556.1 76.5 340
60 2 14 333 57 396 9.50E-149 534.6 6596 cellular organisms;Eukaryota;Opisthokonta;Metazoa;Eumetazoa;Bilateria;Protostomia;Spiralia;Lophotrochozoa;Mollusca;Bivalvia;Autobranchia;Heteroconchia;Euheterodonta;Imparidentia;Venerida;Veneroidea;Veneridae;Mercenaria;Mercenaria mercenaria
UdeM00000041448 59861 199.9 UdeM00000041448 XP_045070405.1 50 208
102 2 2 207 47 254 4.90E-48 199.9 59861 cellular organisms;Eukaryota;Opisthokonta;Metazoa;Eumetazoa;Bilateria;Deuterostomia;Chordata;Craniata;Vertebrata;Gnathostomata;Teleostomi;Euteleostomi;Actinopterygii;Actinopteri;Neopterygii;Teleostei;Osteoglossocephalai;Clupeocephala;Euteleosteomorpha;Protacanthopterygii;Salmoniformes;Salmonidae;Coreguninae;Coregonus;Coregonus clupeaformis
UdeM00000041449 6596 1376.7 UdeM00000041449 XP_045199869.1 42.9
1687 942 14 34 1714 269 1940 0.00E+00 1376.7 6596
cellular organisms;Eukaryota;Opisthokonta;Metazoa;Eumetazoa;Bilateria;Protostomia;Spiralia;Lophotrochozoa;Mollusca;Bivalvia;Autobranchia;Heteroconchia;Euhe

terodonta;Imparidentia;Venerida;Veneroidea;Veneridae;Mercenaria;Mercenaria mercenaria
UdeM00000041450 6596 185.7 UdeM00000041450 XP_045199869.1 41.1 287 168 1 32 317 269 555 9.90E-44 185.7 6596 cellular organisms;Eukaryota;Opisthokonta;Metazoa;Eumetazoa;Bilateria;Protostomia;Spiralia;Lophotrochozoa;Mollusca;Bivalvia;Autobranchia;Heteroconchia;Euheterodonta;Imparidentia;Venerida;Veneroidea;Veneridae;Mercenaria;Mercenaria mercenaria
UdeM00000041451 6596 1159.4 UdeM00000041451 XP_045199869.1 42.8 1373 764 11 1 1366 582 1940 0.00E+00 1159.4 6596 cellular organisms;Eukaryota;Opisthokonta;Metazoa;Eumetazoa;Bilateria;Protostomia;Spiralia;Lophotrochozoa;Mollusca;Bivalvia;Autobranchia;Heteroconchia;Euheterodonta;Imparidentia;Venerida;Veneroidea;Veneridae;Mercenaria;Mercenaria mercenaria
UdeM00000041452 6596 1349.3 UdeM00000041452 XP_045199869.1 42 1686 956 12 32 1709 269 1940 0.00E+00 1349.3 6596 cellular organisms;Eukaryota;Opisthokonta;Metazoa;Eumetazoa;Bilateria;Protostomia;Spiralia;Lophotrochozoa;Mollusca;Bivalvia;Autobranchia;Heteroconchia;Euheterodonta;Imparidentia;Venerida;Veneroidea;Veneridae;Mercenaria;Mercenaria mercenaria
UdeM00000041453 6596 1341.6 UdeM00000041453 XP_045199869.1 42 1686 956 12 34 1711 269 1940 0.00E+00 1341.6 6596 cellular organisms;Eukaryota;Opisthokonta;Metazoa;Eumetazoa;Bilateria;Protostomia;Spiralia;Lophotrochozoa;Mollusca;Bivalvia;Autobranchia;Heteroconchia;Euheterodonta;Imparidentia;Venerida;Veneroidea;Veneridae;Mercenaria;Mercenaria mercenaria
UdeM00000041455 6596 1639 UdeM00000041455 XP_045199760.1 51.6 1823 830 21 103 1898 151 1947 0.00E+00 1639 6596 cellular organisms;Eukaryota;Opisthokonta;Metazoa;Eumetazoa;Bilateria;Protostomia;Spiralia;Lophotrochozoa;Mollusca;Bivalvia;Autobranchia;Heteroconchia;Euheterodonta;Imparidentia;Venerida;Veneroidea;Veneridae;Mercenaria;Mercenaria mercenaria
UdeM00000041456 6596 767.3 UdeM00000041456 XP_045200553.1 67.6 521 169 0 17 537 11 531 1.30E-218 767.3 6596 cellular organisms;Eukaryota;Opisthokonta;Metazoa;Eumetazoa;Bilateria;Protostomia;Spiralia;Lophotrochozoa;Mollusca;Bivalvia;Autobranchia;Heteroconchia;Euheterodonta;Imparidentia;Venerida;Veneroidea;Veneridae;Mercenaria;Mercenaria mercenaria
UdeM00000041457 6500 202.6 UdeM00000041457 XP_035827795.1 77.5 120 27 0 4 123 14 133 2.90E-49 202.6 6500 cellular organisms;Eukaryota;Opisthokonta;Metazoa;Eumetazoa;Bilateria;Protostomia;Spiralia;Lophotrochozoa;Mollusca;Gastropoda;Heterobranchia;Euthyneura;Tectipleura;Aplysiida;Aplysioidea;Aplysiidae;Aplysia;Aplysia californica
UdeM00000041461 6596 117.9 UdeM00000041461 XP_045158309.1 47.4 135 69 2 16 150 76 208 1.30E-23 117.9 6596 cellular organisms;Eukaryota;Opisthokonta;Metazoa;Eumetazoa;Bilateria;Protostomia;Spiralia;Lophotrochozoa;Mollusca;Bivalvia;Autobranchia;Heteroconchia;Euheterodonta;Imparidentia;Venerida;Veneroidea;Veneridae;Mercenaria;Mercenaria mercenaria
UdeM00000041462 6596 223 UdeM00000041462 XP_045197203.1 32.6 387 241 7 1 372 81 462 6.40E-55 223 6596 cellular

organisms;Eukaryota;Opisthokonta;Metazoa;Eumetazoa;Bilateria;Protostomia;Spiralia;Lophotrochozoa;Mollusca;Bivalvia;Autobranchia;Heteroconchia;Euheterodonta;Imparidentia;Venerida;Veneroidea;Veneridae;Mercenaria;Mercenaria mercenaria
UdeM00000041463 6596 413.3 UdeM00000041463 XP_045199078.1 39.4 594 332 10 6 587 11 588 5.20E-112 413.3 6596 cellular organisms;Eukaryota;Opisthokonta;Metazoa;Eumetazoa;Bilateria;Protostomia;Spiralia;Lophotrochozoa;Mollusca;Bivalvia;Autobranchia;Heteroconchia;Euheterodonta;Imparidentia;Venerida;Veneroidea;Veneridae;Mercenaria;Mercenaria mercenaria
UdeM00000041464 6573 610.1 UdeM00000041464 XP_021375393.1 59.8 502 200 1 1 502 36 535 2.50E-171 610.1 6573 cellular organisms;Eukaryota;Opisthokonta;Metazoa;Eumetazoa;Bilateria;Protostomia;Spiralia;Lophotrochozoa;Mollusca;Bivalvia;Autobranchia;Pteriomorphia;Pectinida;Pectinoidea;Pectinidae;Mizuhopecten;Mizuhopecten yessoensis
UdeM00000041465 6579 280.4 UdeM00000041465 XP_033735066.1 33.5 502 295 10 73 542 65 559 4.90E-72 280.4 6579 cellular organisms;Eukaryota;Opisthokonta;Metazoa;Eumetazoa;Bilateria;Protostomia;Spiralia;Lophotrochozoa;Mollusca;Bivalvia;Autobranchia;Pteriomorphia;Pectinida;Pectinoidea;Pectinidae;Pecten;Pecten maximus
UdeM00000041467 6579 288.1 UdeM00000041467 XP_033735066.1 32.3 548 328 12 29 544 23 559 2.40E-74 288.1 6579 cellular organisms;Eukaryota;Opisthokonta;Metazoa;Eumetazoa;Bilateria;Protostomia;Spiralia;Lophotrochozoa;Mollusca;Bivalvia;Autobranchia;Pteriomorphia;Pectinida;Pectinoidea;Pectinidae;Pecten;Pecten maximus
UdeM00000041468 6573 733.4 UdeM00000041468 XP_021373272.1 66.5 552 182 1 1 549 1 552 2.10E-208 733.4 6573 cellular organisms;Eukaryota;Opisthokonta;Metazoa;Eumetazoa;Bilateria;Protostomia;Spiralia;Lophotrochozoa;Mollusca;Bivalvia;Autobranchia;Pteriomorphia;Pectinida;Pectinoidea;Pectinidae;Mizuhopecten;Mizuhopecten yessoensis
UdeM00000041469 6596 339 UdeM00000041469 XP_045201857.1 40.1 436 250 2 6 432 5 438 9.50E-90 339 6596 cellular organisms;Eukaryota;Opisthokonta;Metazoa;Eumetazoa;Bilateria;Protostomia;Spiralia;Lophotrochozoa;Mollusca;Bivalvia;Autobranchia;Heteroconchia;Euheterodonta;Imparidentia;Venerida;Veneroidea;Veneridae;Mercenaria;Mercenaria mercenaria
UdeM00000041470 6596 298.9 UdeM00000041470 XP_045201949.1 46.2 372 159 5 12 343 9 379 8.60E-78 298.9 6596 cellular organisms;Eukaryota;Opisthokonta;Metazoa;Eumetazoa;Bilateria;Protostomia;Spiralia;Lophotrochozoa;Mollusca;Bivalvia;Autobranchia;Heteroconchia;Euheterodonta;Imparidentia;Venerida;Veneroidea;Veneridae;Mercenaria;Mercenaria mercenaria
UdeM00000041471 29159 603.6 UdeM00000041471 XP_034318263.1 77 374 86 0 3 376 4 377 1.70E-169 603.6 29159 cellular organisms;Eukaryota;Opisthokonta;Metazoa;Eumetazoa;Bilateria;Protostomia;Spiralia;Lophotrochozoa;Mollusca;Bivalvia;Autobranchia;Pteriomorphia;Ostreida;Ostreoidea;Ostreidae;Crassostrea;Crassostrea gigas
UdeM00000041472 59729 142.5 UdeM00000041472 XP_030144625.3 35.4 206 125 4 305 505 123 325 1.50E-30 142.5 59729 cellular organisms;Eukaryota;Opisthokonta;Metazoa;Eumetazoa;Bilateria;Deuterostomia;Chordata;Craniata;Vertebrata;Gnathostomata;Teleostomi;Euteleostomi;Sarcopterygii;Dipnotetrapodomorpha;Tetrapoda;Amniota;Sauropsida;Sauria;Archelosauria;Archosauria;Dinosauria;Saurischia;Theropoda;Coelurosauria;Aves;Ne

ognathae;Passeriformes;Passeroidea;Estrildidae;Estrildinae;Taeniopygia;Taeniopygia guttata
UdeM00000041475 6565 125.6 UdeM00000041475 XP_022287303.1 29.8 228 128 7 2 198 522 748 7.30E-26 125.6 6565 cellular organisms;Eukaryota;Opisthokonta;Metazoa;Eumetazoa;Bilateria;Protostomia;Spiralia;Lophotrochozoa;Mollusca;Bivalvia;Autobranchia;Pteriomorphia;Ostreida;Ostreoidea;Ostreidae;Crassostrea;Crassostrea virginica
UdeM00000041477 6565 92 UdeM00000041477 XP_022287303.1 42.9 105 58 2 5 108 591 694 5.30E-16 92 6565 cellular organisms;Eukaryota;Opisthokonta;Metazoa;Eumetazoa;Bilateria;Protostomia;Spiralia;Lophotrochozoa;Mollusca;Bivalvia;Autobranchia;Pteriomorphia;Ostreida;Ostreoidea;Ostreidae;Crassostrea;Crassostrea virginica
UdeM00000041476 6565 92.4 UdeM00000041476 XP_022287303.1 42.9 105 58 2 5 108 591 694 4.00E-16 92.4 6565 cellular organisms;Eukaryota;Opisthokonta;Metazoa;Eumetazoa;Bilateria;Protostomia;Spiralia;Lophotrochozoa;Mollusca;Bivalvia;Autobranchia;Pteriomorphia;Ostreida;Ostreoidea;Ostreidae;Crassostrea;Crassostrea virginica
UdeM00000041478 400727 478.8 UdeM00000041478 XP_025080098.1 87.7 260 30 1 1 258 1 260 4.40E-132 478.8 400727 cellular organisms;Eukaryota;Opisthokonta;Metazoa;Eumetazoa;Bilateria;Protostomia;Spiralia;Lophotrochozoa;Mollusca;Gastropoda;Caenogastropoda;Architaenioglossa;Ampullarioidea;Ampullariidae;Pomacea;Pomacea canaliculata
UdeM00000041479 400727 448.4 UdeM00000041479 XP_025080098.1 83.8 260 29 2 1 247 1 260 6.10E-123 448.4 400727 cellular organisms;Eukaryota;Opisthokonta;Metazoa;Eumetazoa;Bilateria;Protostomia;Spiralia;Lophotrochozoa;Mollusca;Gastropoda;Caenogastropoda;Architaenioglossa;Ampullarioidea;Ampullariidae;Pomacea;Pomacea canaliculata
UdeM00000041480 6579 821.2 UdeM00000041480 XP_033759680.1 74.5 596 131 8 257 834 192 784 1.20E-234 821.2 6579 cellular organisms;Eukaryota;Opisthokonta;Metazoa;Eumetazoa;Bilateria;Protostomia;Spiralia;Lophotrochozoa;Mollusca;Bivalvia;Autobranchia;Pteriomorphia;Pectinida;Pectinoidea;Pectinidae;Pecten;Pecten maximus
UdeM00000041481 6573 285.8 UdeM00000041481 XP_021368303.1 50.3 326 107 10 1 306 3 293 6.50E-74 285.8 6573 cellular organisms;Eukaryota;Opisthokonta;Metazoa;Eumetazoa;Bilateria;Protostomia;Spiralia;Lophotrochozoa;Mollusca;Bivalvia;Autobranchia;Pteriomorphia;Pectinida;Pectinoidea;Pectinidae;Mizuhopecten;Mizuhopecten yessoensis
UdeM00000041482 6596 63.9 UdeM00000041482 XP_045199535.1 52.9 51 24 0 216 266 162 212 3.50E-07 63.9 6596 cellular organisms;Eukaryota;Opisthokonta;Metazoa;Eumetazoa;Bilateria;Protostomia;Spiralia;Lophotrochozoa;Mollusca;Bivalvia;Autobranchia;Heteroconchia;Euheterodonta;Imparidentia;Venerida;Veneroidea;Veneridae;Mercenaria;Mercenaria mercenaria
UdeM00000041483 6596 851.3 UdeM00000041483 XP_045199534.1 62.7 611 228 0 1 611 61 671 7.70E-244 851.3 6596 cellular organisms;Eukaryota;Opisthokonta;Metazoa;Eumetazoa;Bilateria;Protostomia;Spiralia;Lophotrochozoa;Mollusca;Bivalvia;Autobranchia;Heteroconchia;Euheterodonta;Imparidentia;Venerida;Veneroidea;Veneridae;Mercenaria;Mercenaria mercenaria
UdeM00000041484 6579 75.1 UdeM00000041484 XP_033760834.1 41.1 112 58 2 7 111 1 111 7.00E-11 75.1 6579 cellular organisms;Eukaryota;Opisthokonta;Metazoa;Eumetazoa;Bilateria;Protostomia;

Spiralia;Lophotrochozoa;Mollusca;Bivalvia;Autobranchia;Pteriomorphia;Pectinida;Pectinoidea;Pectinidae;Pecten;Pecten maximus
UdeM00000041485 6596 232.3 UdeM00000041485 XP_045202502.1 50.3 348 167 4 1547 1892 1534 1877 5.20E-57 232.3 6596 cellular organisms;Eukaryota;Opisthokonta;Metazoa;Eumetazoa;Bilateria;Protostomia;Spiralia;Lophotrochozoa;Mollusca;Bivalvia;Autobranchia;Heteroconchia;Euheterodonta;Imparidentia;Venerida;Veneroidea;Veneridae;Mercenaria;Mercenaria mercenaria
UdeM00000041486 10224 85.9 UdeM00000041486 XP_002740528.1 37.3 142 59 4 2 121 106 239 4.30E-14 85.9 10224 cellular organisms;Eukaryota;Opisthokonta;Metazoa;Eumetazoa;Bilateria;Deuterostomia;Hemichordata;Enteropneusta;Harrimaniidae;Saccoglossus;Saccoglossus kowalevskii
UdeM00000041487 10224 184.1 UdeM00000041487 XP_006814936.1 42 245 134 4 111 350 64 305 3.10E-43 184.1 10224 cellular organisms;Eukaryota;Opisthokonta;Metazoa;Eumetazoa;Bilateria;Deuterostomia;Hemichordata;Enteropneusta;Harrimaniidae;Saccoglossus;Saccoglossus kowalevskii
UdeM00000041488 6596 157.1 UdeM00000041488 XP_045202502.1 77.8 108 23 1 1 108 1908 2014 1.20E-35 157.1 6596 cellular organisms;Eukaryota;Opisthokonta;Metazoa;Eumetazoa;Bilateria;Protostomia;Spiralia;Lophotrochozoa;Mollusca;Bivalvia;Autobranchia;Heteroconchia;Euheterodonta;Imparidentia;Venerida;Veneroidea;Veneridae;Mercenaria;Mercenaria mercenaria
UdeM00000041489 6596 320.1 UdeM00000041489 XP_045201857.1 39.6 412 241 2 6 409 7 418 4.70E-84 320.1 6596 cellular organisms;Eukaryota;Opisthokonta;Metazoa;Eumetazoa;Bilateria;Protostomia;Spiralia;Lophotrochozoa;Mollusca;Bivalvia;Autobranchia;Heteroconchia;Euheterodonta;Imparidentia;Venerida;Veneroidea;Veneridae;Mercenaria;Mercenaria mercenaria
UdeM00000041490 29159 141 UdeM00000041490 XP_034335613.1 30.1 362 183 9 9 366 498 793 3.10E-30 141 29159 cellular organisms;Eukaryota;Opisthokonta;Metazoa;Eumetazoa;Bilateria;Protostomia;Spiralia;Lophotrochozoa;Mollusca;Bivalvia;Autobranchia;Pteriomorphia;Ostreida;Ostreoidea;Ostreidae;Crassostrea;Crassostrea gigas
UdeM00000041491 6596 106.3 UdeM00000041491 XP_045197109.1 26.3 369 197 7 671 975 450 807 2.20E-19 106.3 6596 cellular organisms;Eukaryota;Opisthokonta;Metazoa;Eumetazoa;Bilateria;Protostomia;Spiralia;Lophotrochozoa;Mollusca;Bivalvia;Autobranchia;Heteroconchia;Euheterodonta;Imparidentia;Venerida;Veneroidea;Veneridae;Mercenaria;Mercenaria mercenaria
UdeM00000041494 6596 202.6 UdeM00000041494 XP_045197408.1 30 497 268 11 544 1009 952 1399 2.80E-48 202.6 6596 cellular organisms;Eukaryota;Opisthokonta;Metazoa;Eumetazoa;Bilateria;Protostomia;Spiralia;Lophotrochozoa;Mollusca;Bivalvia;Autobranchia;Heteroconchia;Euheterodonta;Imparidentia;Venerida;Veneroidea;Veneridae;Mercenaria;Mercenaria mercenaria
UdeM00000041495 6573 315.5 UdeM00000041495 XP_021372035.1 36.4 530 314 9 55 570 90 610 1.50E-82 315.5 6573 cellular organisms;Eukaryota;Opisthokonta;Metazoa;Eumetazoa;Bilateria;Protostomia;Spiralia;Lophotrochozoa;Mollusca;Bivalvia;Autobranchia;Pteriomorphia;Pectinida;Pectinoidea;Pectinidae;Mizuhopecten;Mizuhopecten yessoensis
UdeM00000041496 6579 114.4 UdeM00000041496 XP_033732935.1 37.6 173 101 4 21 188 35 205 1.60E-22 114.4 6579 cellular

organisms;Eukaryota;Opisthokonta;Metazoa;Eumetazoa;Bilateria;Protostomia;
Spiralia;Lophotrochozoa;Mollusca;Bivalvia;Autobranchia;Pteriomorphia;Pect
inida;Pectinoidea;Pectinidae;Pecten;Pecten maximus
UdeM00000041497 6565 136.7 UdeM00000041497 XP_022296914.1 31 287
148 2 1 287 174 410 6.10E-29 136.7 6565 cellular
organisms;Eukaryota;Opisthokonta;Metazoa;Eumetazoa;Bilateria;Protostomia;
Spiralia;Lophotrochozoa;Mollusca;Bivalvia;Autobranchia;Pteriomorphia;Ostr
eida;Ostreoidea;Ostreidae;Crassostrea;Crassostrea virginica
UdeM00000041498 6565 317 UdeM00000041498 XP_022298933.1 31 671
397 13 207 834 89 736 7.30E-83 317 6565 cellular
organisms;Eukaryota;Opisthokonta;Metazoa;Eumetazoa;Bilateria;Protostomia;
Spiralia;Lophotrochozoa;Mollusca;Bivalvia;Autobranchia;Pteriomorphia;Ostr
eida;Ostreoidea;Ostreidae;Crassostrea;Crassostrea virginica
UdeM00000041499 6579 115.5 UdeM00000041499 XP_033732935.1 38.2 173
100 4 21 188 35 205 7.20E-23 115.5 6579 cellular
organisms;Eukaryota;Opisthokonta;Metazoa;Eumetazoa;Bilateria;Protostomia;
Spiralia;Lophotrochozoa;Mollusca;Bivalvia;Autobranchia;Pteriomorphia;Pect
inida;Pectinoidea;Pectinidae;Pecten;Pecten maximus
UdeM00000041500 6573 319.7 UdeM00000041500 XP_021372035.1 36.8 527
310 9 58 570 93 610 7.70E-84 319.7 6573 cellular
organisms;Eukaryota;Opisthokonta;Metazoa;Eumetazoa;Bilateria;Protostomia;
Spiralia;Lophotrochozoa;Mollusca;Bivalvia;Autobranchia;Pteriomorphia;Pect
inida;Pectinoidea;Pectinidae;Mizuhopecten;Mizuhopecten yessoensis
UdeM00000041501 6596 60.1 UdeM00000041501 XP_045169188.1 44.8 67
33 2 1 67 859 921 1.30E-06 60.1 6596 cellular
organisms;Eukaryota;Opisthokonta;Metazoa;Eumetazoa;Bilateria;Protostomia;
Spiralia;Lophotrochozoa;Mollusca;Bivalvia;Autobranchia;Heteroconchia;Euhe
terodonta;Imparidentia;Venerida;Veneroidea;Veneridae;Mercenaria;Mercenari
a mercenaria
UdeM00000041502 6573 345.9 UdeM00000041502 XP_021346851.1 29.8 801
487 15 26 813 29 767 1.40E-91 345.9 6573 cellular
organisms;Eukaryota;Opisthokonta;Metazoa;Eumetazoa;Bilateria;Protostomia;
Spiralia;Lophotrochozoa;Mollusca;Bivalvia;Autobranchia;Pteriomorphia;Pect
inida;Pectinoidea;Pectinidae;Mizuhopecten;Mizuhopecten yessoensis
UdeM00000041503 6596 60.1 UdeM00000041503 XP_045169188.1 44.8 67
33 2 1 67 859 921 1.30E-06 60.1 6596 cellular
organisms;Eukaryota;Opisthokonta;Metazoa;Eumetazoa;Bilateria;Protostomia;
Spiralia;Lophotrochozoa;Mollusca;Bivalvia;Autobranchia;Heteroconchia;Euhe
terodonta;Imparidentia;Venerida;Veneroidea;Veneridae;Mercenaria;Mercenari
a mercenaria
UdeM00000041504 6565 311.6 UdeM00000041504 XP_022301129.1 38.3 418
250 5 157 567 320 736 2.10E-81 311.6 6565 cellular
organisms;Eukaryota;Opisthokonta;Metazoa;Eumetazoa;Bilateria;Protostomia;
Spiralia;Lophotrochozoa;Mollusca;Bivalvia;Autobranchia;Pteriomorphia;Ostr
eida;Ostreoidea;Ostreidae;Crassostrea;Crassostrea virginica
UdeM00000041505 29159 169.5 UdeM00000041505 XP_034331441.1 28.8 473
238 12 10 388 61 528 8.60E-39 169.5 29159 cellular
organisms;Eukaryota;Opisthokonta;Metazoa;Eumetazoa;Bilateria;Protostomia;
Spiralia;Lophotrochozoa;Mollusca;Bivalvia;Autobranchia;Pteriomorphia;Ostr
eida;Ostreoidea;Ostreidae;Crassostrea;Crassostrea gigas
UdeM00000041506 6573 463.4 UdeM00000041506 XP_021346851.1 33.4 874
512 13 26 886 29 845 6.70E-127 463.4 6573 cellular
organisms;Eukaryota;Opisthokonta;Metazoa;Eumetazoa;Bilateria;Protostomia;

Spiralia;Lophotrochozoa;Mollusca;Bivalvia;Autobranchia;Pteriomorphia;Pectinida;Pectinoidea;Pectinidae;Mizuhopecten;Mizuhopecten yessoensis
UdeM00000041507 1735272 296.2 UdeM00000041507 XP_041347002.1 60.8 265 98 3 269 531 194 454 8.30E-77 296.2 1735272 cellular organisms;Eukaryota;Opisthokonta;Metazoa;Eumetazoa;Bilateria;Protostomia;Spiralia;Lophotrochozoa;Mollusca;Gastropoda;Neomphalina;Neomphaloidea;Peltospiridae;Gigantopelta;Gigantopelta aegis
UdeM00000041508 1735272 296.2 UdeM00000041508 XP_041347002.1 60.8 265 98 3 238 500 194 454 7.80E-77 296.2 1735272 cellular organisms;Eukaryota;Opisthokonta;Metazoa;Eumetazoa;Bilateria;Protostomia;Spiralia;Lophotrochozoa;Mollusca;Gastropoda;Neomphalina;Neomphaloidea;Peltospiridae;Gigantopelta;Gigantopelta aegis
UdeM00000041512 225164 196.1 UdeM00000041512 XP_009062741.1 32.2 307 197 5 54 352 55 358 8.00E-47 196.1 225164 cellular organisms;Eukaryota;Opisthokonta;Metazoa;Eumetazoa;Bilateria;Protostomia;Spiralia;Lophotrochozoa;Mollusca;Gastropoda;Patellogastropoda;Lottioidea;Lottiidae;Lottia;Lottia gigantea
UdeM00000042378 6596 562.8 UdeM00000042378 XP_045178786.1 32.8 2235 983 52 50 1954 100 2144 1.80E-156 562.8 6596 cellular organisms;Eukaryota;Opisthokonta;Metazoa;Eumetazoa;Bilateria;Protostomia;Spiralia;Lophotrochozoa;Mollusca;Bivalvia;Autobranchia;Heteroconchia;Euheterodonta;Imparidentia;Venerida;Veneroidea;Veneridae;Mercenaria;Mercenaria mercenaria
UdeM00000042379 45351 93.2 UdeM00000042379 XP_001623471.2 31.3 211 118 5 3 197 6 205 4.50E-16 93.2 45351 cellular organisms;Eukaryota;Opisthokonta;Metazoa;Eumetazoa;Cnidaria;Anthozoa;Hexacorallia;Actiniaria;Edwardsiidae;Nematostella;Nematostella vectensis
UdeM00000042380 6596 193.4 UdeM00000042380 XP_045177779.1 44.2 231 125 4 56 285 398 625 4.60E-46 193.4 6596 cellular organisms;Eukaryota;Opisthokonta;Metazoa;Eumetazoa;Bilateria;Protostomia;Spiralia;Lophotrochozoa;Mollusca;Bivalvia;Autobranchia;Heteroconchia;Euheterodonta;Imparidentia;Venerida;Veneroidea;Veneridae;Mercenaria;Mercenaria mercenaria
UdeM00000042381 6596 116.7 UdeM00000042381 XP_045177779.1 44.3 122 67 1 3 123 401 522 2.10E-23 116.7 6596 cellular organisms;Eukaryota;Opisthokonta;Metazoa;Eumetazoa;Bilateria;Protostomia;Spiralia;Lophotrochozoa;Mollusca;Bivalvia;Autobranchia;Heteroconchia;Euheterodonta;Imparidentia;Venerida;Veneroidea;Veneridae;Mercenaria;Mercenaria mercenaria
UdeM00000042382 6596 189.9 UdeM00000042382 XP_045177778.1 39.8 299 162 6 1 289 405 695 8.50E-45 189.9 6596 cellular organisms;Eukaryota;Opisthokonta;Metazoa;Eumetazoa;Bilateria;Protostomia;Spiralia;Lophotrochozoa;Mollusca;Bivalvia;Autobranchia;Heteroconchia;Euheterodonta;Imparidentia;Venerida;Veneroidea;Veneridae;Mercenaria;Mercenaria mercenaria
UdeM00000042384 72036 303.1 UdeM00000042384 XP_040576246.1 45 349 184 6 7 351 117 461 4.50E-79 303.1 72036 cellular organisms;Eukaryota;Opisthokonta;Metazoa;Eumetazoa;Bilateria;Protostomia;Ecdysozoa;Panarthropoda;Arthropoda;Mandibulata;Pancrustacea;Crustacea;Multicrustacea;Hexanauplia;Copepoda;Neocopepoda;Podoplea;Siphonostomatoida;Caligidae;Lepeophtheirus;Lepeophtheirus salmonis

UdeM00000042386	29159	241.9	UdeM00000042386	XP_034302488.1	47	328
	155	5	1463	1785	7	320	6.30E-60	241.9	29159	cellular
organisms;Eukaryota;Opisthokonta;Metazoa;Eumetazoa;Bilateria;Protostomia;
Spiralia;Lophotrochozoa;Mollusca;Bivalvia;Autobranchia;Pteriomorphia;Ostr
eida;Ostreoidea;Ostreidae;Crassostrea;Crassostrea gigas
UdeM00000042387	6596	324.3	UdeM00000042387	XP_045199354.1	28.8	852
	485	22	1	758	65	888	4.10E-85	324.3	6596	cellular
organisms;Eukaryota;Opisthokonta;Metazoa;Eumetazoa;Bilateria;Protostomia;
Spiralia;Lophotrochozoa;Mollusca;Bivalvia;Autobranchia;Heteroconchia;Euhe
terodonta;Imparidentia;Venerida;Veneroidea;Veneridae;Mercenaria;Mercenari
a mercenaria
UdeM00000042388	6565	167.9	UdeM00000042388	XP_022297037.1	43.1	225
	121	3	6	225	11	233	1.40E-38	167.9	6565	cellular
organisms;Eukaryota;Opisthokonta;Metazoa;Eumetazoa;Bilateria;Protostomia;
Spiralia;Lophotrochozoa;Mollusca;Bivalvia;Autobranchia;Pteriomorphia;Ostr
eida;Ostreoidea;Ostreidae;Crassostrea;Crassostrea virginica
UdeM00000042389	6596	395.2	UdeM00000042389	XP_045199354.1	29.9	941
	545	25	1	864	65	967	2.10E-106	395.2	6596	cellular
organisms;Eukaryota;Opisthokonta;Metazoa;Eumetazoa;Bilateria;Protostomia;
Spiralia;Lophotrochozoa;Mollusca;Bivalvia;Autobranchia;Heteroconchia;Euhe
terodonta;Imparidentia;Venerida;Veneroidea;Veneridae;Mercenaria;Mercenari
a mercenaria
UdeM00000042390	6565	79.7	UdeM00000042390	XP_022297037.1	36	136
	80	3	6	136	11	144	3.20E-12	79.7	6565	cellular
organisms;Eukaryota;Opisthokonta;Metazoa;Eumetazoa;Bilateria;Protostomia;
Spiralia;Lophotrochozoa;Mollusca;Bivalvia;Autobranchia;Pteriomorphia;Ostr
eida;Ostreoidea;Ostreidae;Crassostrea;Crassostrea virginica
UdeM00000042391	6565	72	UdeM00000042391	XP_022297037.1	65.4	52
	18	0	1	52	183	234	1.30E-09	72	6565	cellular
organisms;Eukaryota;Opisthokonta;Metazoa;Eumetazoa;Bilateria;Protostomia;
Spiralia;Lophotrochozoa;Mollusca;Bivalvia;Autobranchia;Pteriomorphia;Ostr
eida;Ostreoidea;Ostreidae;Crassostrea;Crassostrea virginica
UdeM00000042392	7574	63.5	UdeM00000042392	XP_013389170.1	29.9	97
	65	2	9	104	338	432	1.80E-07	63.5	7574	cellular
organisms;Eukaryota;Opisthokonta;Metazoa;Eumetazoa;Bilateria;Protostomia;
Spiralia;Lophotrochozoa;Brachiopoda;Linguliformea;Lingulata;Lingulida;Lin
guloidea;Lingulidae;Lingula;Lingula anatina
UdeM00000042394	6596	370.9	UdeM00000042394	XP_045179669.1	53.2	391
	163	4	6	394	29	401	2.00E-99	370.9	6596	cellular
organisms;Eukaryota;Opisthokonta;Metazoa;Eumetazoa;Bilateria;Protostomia;
Spiralia;Lophotrochozoa;Mollusca;Bivalvia;Autobranchia;Heteroconchia;Euhe
terodonta;Imparidentia;Venerida;Veneroidea;Veneridae;Mercenaria;Mercenari
a mercenaria
UdeM00000042395	6596	186.8	UdeM00000042395	XP_045187052.1	50	188
	89	3	2	188	145	328	3.40E-44	186.8	6596	cellular
organisms;Eukaryota;Opisthokonta;Metazoa;Eumetazoa;Bilateria;Protostomia;
Spiralia;Lophotrochozoa;Mollusca;Bivalvia;Autobranchia;Heteroconchia;Euhe
terodonta;Imparidentia;Venerida;Veneroidea;Veneridae;Mercenaria;Mercenari
a mercenaria
UdeM00000042396	6596	186	UdeM00000042396	XP_045187052.1	49.7	187
	89	3	2	187	145	327	4.40E-44	186	6596	cellular
organisms;Eukaryota;Opisthokonta;Metazoa;Eumetazoa;Bilateria;Protostomia;
Spiralia;Lophotrochozoa;Mollusca;Bivalvia;Autobranchia;Heteroconchia;Euhe

terodonta;Imparidentia;Venerida;Veneroidea;Veneridae;Mercenaria;Mercenari
a mercenaria
UdeM00000042397 6596 99.8 UdeM00000042397 XP_045202346.1 40.5 131
74 2 6 135 16 143 2.90E-18 99.8 6596 cellular
organisms;Eukaryota;Opisthokonta;Metazoa;Eumetazoa;Bilateria;Protostomia;
Spiralia;Lophotrochozoa;Mollusca;Bivalvia;Autobranchia;Heteroconchia;Euhe
terodonta;Imparidentia;Venerida;Veneroidea;Veneridae;Mercenaria;Mercenari
a mercenaria
UdeM00000042398 6596 1781.1 UdeM00000042398 XP_045179914.1 59
1836 633 20 35 1814 1235 3006 0.00E+00 1781.1 6596
cellular
organisms;Eukaryota;Opisthokonta;Metazoa;Eumetazoa;Bilateria;Protostomia;
Spiralia;Lophotrochozoa;Mollusca;Bivalvia;Autobranchia;Heteroconchia;Euhe
terodonta;Imparidentia;Venerida;Veneroidea;Veneridae;Mercenaria;Mercenari
a mercenaria
UdeM00000042399 6565 1232.6 UdeM00000042399 XP_022322319.1 46
1290 673 14 1 1275 192 1472 0.00E+00 1232.6 6565
cellular
organisms;Eukaryota;Opisthokonta;Metazoa;Eumetazoa;Bilateria;Protostomia;
Spiralia;Lophotrochozoa;Mollusca;Bivalvia;Autobranchia;Pteriomorphia;Ostr
eida;Ostreoidea;Ostreidae;Crassostrea;Crassostrea virginica
UdeM00000042400 29159 204.5 UdeM00000042400 XP_034300594.1 54.2 177
77 1 1 177 61 233 1.20E-49 204.5 29159 cellular
organisms;Eukaryota;Opisthokonta;Metazoa;Eumetazoa;Bilateria;Protostomia;
Spiralia;Lophotrochozoa;Mollusca;Bivalvia;Autobranchia;Pteriomorphia;Ostr
eida;Ostreoidea;Ostreidae;Crassostrea;Crassostrea gigas
UdeM00000042402 6596 167.9 UdeM00000042402 XP_045204388.1 35.4 347
187 11 6 333 10 338 3.10E-38 167.9 6596 cellular
organisms;Eukaryota;Opisthokonta;Metazoa;Eumetazoa;Bilateria;Protostomia;
Spiralia;Lophotrochozoa;Mollusca;Bivalvia;Autobranchia;Heteroconchia;Euhe
terodonta;Imparidentia;Venerida;Veneroidea;Veneridae;Mercenaria;Mercenari
a mercenaria
UdeM00000042403 6579 216.1 UdeM00000042403 XP_033731739.1 54.7 179
79 1 1 179 202 378 3.70E-53 216.1 6579 cellular
organisms;Eukaryota;Opisthokonta;Metazoa;Eumetazoa;Bilateria;Protostomia;
Spiralia;Lophotrochozoa;Mollusca;Bivalvia;Autobranchia;Pteriomorphia;Pect
inida;Pectinoidea;Pectinidae;Pecten;Pecten maximus
UdeM00000042405 6573 252.7 UdeM00000042405 XP_021350148.1 45.3 311
161 5 2 306 33 340 6.50E-64 252.7 6573 cellular
organisms;Eukaryota;Opisthokonta;Metazoa;Eumetazoa;Bilateria;Protostomia;
Spiralia;Lophotrochozoa;Mollusca;Bivalvia;Autobranchia;Pteriomorphia;Pect
inida;Pectinoidea;Pectinidae;Mizuhopecten;Mizuhopecten yessoensis
UdeM00000042406 6565 371.7 UdeM00000042406 XP_022320666.1 53.8 379
165 7 3 377 3886 4258 1.30E-99 371.7 6565 cellular
organisms;Eukaryota;Opisthokonta;Metazoa;Eumetazoa;Bilateria;Protostomia;
Spiralia;Lophotrochozoa;Mollusca;Bivalvia;Autobranchia;Pteriomorphia;Ostr
eida;Ostreoidea;Ostreidae;Crassostrea;Crassostrea virginica
UdeM00000042407 29159 1053.9 UdeM00000042407 XP_034301274.1 59.1
815 328 4 3 814 2823 3635 1.50E-304 1053.9
29159 cellular
organisms;Eukaryota;Opisthokonta;Metazoa;Eumetazoa;Bilateria;Protostomia;
Spiralia;Lophotrochozoa;Mollusca;Bivalvia;Autobranchia;Pteriomorphia;Ostr
eida;Ostreoidea;Ostreidae;Crassostrea;Crassostrea gigas

UdeM00000042408 6526 3037.7 UdeM00000042408 XP_013077212.1 48.6
2990 1485 15 1 2976 231 3181 0.00E+00 3037.7 6526
cellular
organisms;Eukaryota;Opisthokonta;Metazoa;Eumetazoa;Bilateria;Protostomia;
Spiralia;Lophotrochozoa;Mollusca;Gastropoda;Heterobranchia;Euthyneura;Pan
pulmonata;Hygrophila;Lymnaeoidea;Planorbidae;Biomphalaria;Biomphalaria
glabrata
UdeM00000042409 1735272 212.2 UdeM00000042409 XP_041374759.1 45.9
209 110 2 3 209 19 226 6.30E-52 212.2 1735272
cellular
organisms;Eukaryota;Opisthokonta;Metazoa;Eumetazoa;Bilateria;Protostomia;
Spiralia;Lophotrochozoa;Mollusca;Gastropoda;Neomphalina;Neomphaloidea;Pel
tospiridae;Gigantopelta;Gigantopelta aegis
UdeM00000042410 6596 261.9 UdeM00000042410 XP_045178329.1 62.3 204
77 0 1 204 1 204 6.80E-67 261.9 6596 cellular
organisms;Eukaryota;Opisthokonta;Metazoa;Eumetazoa;Bilateria;Protostomia;
Spiralia;Lophotrochozoa;Mollusca;Bivalvia;Autobranchia;Heteroconchia;Euhe
terodonta;Imparidentia;Venerida;Veneroidea;Veneridae;Mercenaria;Mercenari
a mercenaria
UdeM00000042411 6565 841.6 UdeM00000042411 XP_022344846.1 72.2 543
137 2 1 543 2 530 5.40E-241 841.6 6565 cellular
organisms;Eukaryota;Opisthokonta;Metazoa;Eumetazoa;Bilateria;Protostomia;
Spiralia;Lophotrochozoa;Mollusca;Bivalvia;Autobranchia;Pteriomorphia;Ostr
eida;Ostreoidea;Ostreidae;Crassostrea;Crassostrea virginica
UdeM00000042412 7574 609.4 UdeM00000042412 XP_013382110.1 49.2 589
280 7 14 601 45 615 5.00E-171 609.4 7574 cellular
organisms;Eukaryota;Opisthokonta;Metazoa;Eumetazoa;Bilateria;Protostomia;
Spiralia;Lophotrochozoa;Brachiopoda;Linguliformea;Lingulata;Lingulida;Lin
guloidea;Lingulidae;Lingula;Lingula anatina
UdeM00000042413 7574 236.5 UdeM00000042413 XP_013409676.1 50.7 268
115 5 1 261 1 258 3.90E-59 236.5 7574 cellular
organisms;Eukaryota;Opisthokonta;Metazoa;Eumetazoa;Bilateria;Protostomia;
Spiralia;Lophotrochozoa;Brachiopoda;Linguliformea;Lingulata;Lingulida;Lin
guloidea;Lingulidae;Lingula;Lingula anatina
UdeM00000042414 6500 70.1 UdeM00000042414 XP_005096263.1 42.8 152
67 5 6 151 8 145 2.80E-09 70.1 6500 cellular
organisms;Eukaryota;Opisthokonta;Metazoa;Eumetazoa;Bilateria;Protostomia;
Spiralia;Lophotrochozoa;Mollusca;Gastropoda;Heterobranchia;Euthyneura;Tec
tipleura;Aplysiida;Aplysioidea;Aplysiidae;Aplysia;Aplysia californica
UdeM00000042415 6596 829.3 UdeM00000042415 XP_045179606.1 81.5 492
89 1 6 497 149 638 3.10E-237 829.3 6596 cellular
organisms;Eukaryota;Opisthokonta;Metazoa;Eumetazoa;Bilateria;Protostomia;
Spiralia;Lophotrochozoa;Mollusca;Bivalvia;Autobranchia;Heteroconchia;Euhe
terodonta;Imparidentia;Venerida;Veneroidea;Veneridae;Mercenaria;Mercenari
a mercenaria
UdeM00000042416 451745 110.2 UdeM00000042416 XP_037531102.1 28.2
209 144 3 7 215 184 386 3.50E-21 110.2 451745
cellular
organisms;Eukaryota;Opisthokonta;Metazoa;Eumetazoa;Bilateria;Deuterostomi
a;Chordata;Craniata;Vertebrata;Gnathostomata;Teleostomi;Euteleostomi;Acti
nopterygii;Actinopteri;Neopterygii;Teleostei;Osteoglossocephalai;Clupeoce
phala;Euteleosteomorpha;Neoteleostei;Eurypterygia;Ctenosquamata;Acanthomo
rphata;Euacanthomorphacea;Percomorphaceae;Ovalentaria;Atherinomorphae;Cyp
rinodontiformes;Aplocheiloidei;Rivulidae;Nematolebias;Nematolebias whitei

UdeM00000039991 441366 326.6 UdeM00000039991 XP_028296753.1 40.5
459 258 8 6 463 32 476 5.30E-86 326.6 441366
cellular
organisms;Eukaryota;Opisthokonta;Metazoa;Eumetazoa;Bilateria;Deuterostomia;Chordata;Craniata;Vertebrata;Gnathostomata;Teleostomi;Euteleostomi;Actinopterygii;Actinopteri;Neopterygii;Teleostei;Osteoglossocephalai;Clupeocephala;Euteleosteomorpha;Neoteleostei;Eurypterygia;Ctenosquamata;Acanthomorphata;Euacanthomorphacea;Percomorphaceae;Ovalentaria;Blenniimorphae;Blenniiformes;Gobiesocoidei;Gobiesocidae;Gobiesocinae;Gouania;Gouania willdenowi
UdeM00000039992 6573 104.4 UdeM00000039992 XP_021369796.1 34.4 151
98 1 165 315 4176 4325 2.90E-19 104.4 6573 cellular
organisms;Eukaryota;Opisthokonta;Metazoa;Eumetazoa;Bilateria;Protostomia;Spiralia;Lophotrochozoa;Mollusca;Bivalvia;Autobranchia;Pteriomorphia;Pectinida;Pectinoidea;Pectinidae;Mizuhopecten;Mizuhopecten yessoensis
UdeM00000039994 6596 412.5 UdeM00000039994 XP_045190136.1 26.2 1179
784 27 477 1600 10 1157 2.40E-111 412.5 6596 cellular
organisms;Eukaryota;Opisthokonta;Metazoa;Eumetazoa;Bilateria;Protostomia;Spiralia;Lophotrochozoa;Mollusca;Bivalvia;Autobranchia;Heteroconchia;Euheterodonta;Imparidentia;Venerida;Veneroidea;Veneridae;Mercenaria;Mercenaria mercenaria
UdeM00000039995 6596 445.3 UdeM00000039995 XP_045190136.1 27.1 1178
792 26 477 1617 10 1157 3.40E-121 445.3 6596 cellular
organisms;Eukaryota;Opisthokonta;Metazoa;Eumetazoa;Bilateria;Protostomia;Spiralia;Lophotrochozoa;Mollusca;Bivalvia;Autobranchia;Heteroconchia;Euheterodonta;Imparidentia;Venerida;Veneroidea;Veneridae;Mercenaria;Mercenaria mercenaria
UdeM00000021990 6596 191 UdeM00000021990 XP_045191793.1 39 310
160 7 1 307 258 541 2.40E-45 191 6596 cellular
organisms;Eukaryota;Opisthokonta;Metazoa;Eumetazoa;Bilateria;Protostomia;Spiralia;Lophotrochozoa;Mollusca;Bivalvia;Autobranchia;Heteroconchia;Euheterodonta;Imparidentia;Venerida;Veneroidea;Veneridae;Mercenaria;Mercenaria mercenaria
UdeM00000021991 6596 153.7 UdeM00000021991 XP_045177779.1 41.1 209
117 5 38 244 372 576 3.20E-34 153.7 6596 cellular
organisms;Eukaryota;Opisthokonta;Metazoa;Eumetazoa;Bilateria;Protostomia;Spiralia;Lophotrochozoa;Mollusca;Bivalvia;Autobranchia;Heteroconchia;Euheterodonta;Imparidentia;Venerida;Veneroidea;Veneridae;Mercenaria;Mercenaria mercenaria
UdeM00000021992 29159 106.3 UdeM00000021992 XP_034305547.1 29.3 222
137 6 79 284 21 238 6.60E-20 106.3 29159 cellular
organisms;Eukaryota;Opisthokonta;Metazoa;Eumetazoa;Bilateria;Protostomia;Spiralia;Lophotrochozoa;Mollusca;Bivalvia;Autobranchia;Pteriomorphia;Ostreida;Ostreoidea;Ostreidae;Crassostrea;Crassostrea gigas
UdeM00000021993 8154 73.9 UdeM00000021993 XP_026015163.1 52.1 73
35 0 1 73 1 73 1.30E-10 73.9 8154 cellular
organisms;Eukaryota;Opisthokonta;Metazoa;Eumetazoa;Bilateria;Deuterostomia;Chordata;Craniata;Vertebrata;Gnathostomata;Teleostomi;Euteleostomi;Actinopterygii;Actinopteri;Neopterygii;Teleostei;Osteoglossocephalai;Clupeocephala;Euteleosteomorpha;Neoteleostei;Eurypterygia;Ctenosquamata;Acanthomorphata;Euacanthomorphacea;Percomorphaceae;Ovalentaria;Cichlomorphae;Cichliformes;Cichlidae;African
cichlids;Pseudocrenilabrinae;Haplochromini;Astatotilapia;Astatotilapia calliptera

UdeM00000021994 6596 174.5 UdeM00000021994 XP_045182316.1 45.1 213
114 2 106 318 407 616 2.40E-40 174.5 6596 cellular
organisms;Eukaryota;Opisthokonta;Metazoa;Eumetazoa;Bilateria;Protostomia;
Spiralia;Lophotrochozoa;Mollusca;Bivalvia;Autobranchia;Heteroconchia;Euhe
terodonta;Imparidentia;Venerida;Veneroidea;Veneridae;Mercenaria;Mercenari
a mercenaria
UdeM00000021996 6596 109.4 UdeM00000021996 XP_045162187.1 68.8 77
24 0 1 77 36 112 3.40E-21 109.4 6596 cellular
organisms;Eukaryota;Opisthokonta;Metazoa;Eumetazoa;Bilateria;Protostomia;
Spiralia;Lophotrochozoa;Mollusca;Bivalvia;Autobranchia;Heteroconchia;Euhe
terodonta;Imparidentia;Venerida;Veneroidea;Veneridae;Mercenaria;Mercenari
a mercenaria
UdeM00000021997 6596 303.5 UdeM00000021997 XP_045177776.1 41.9 372
209 4 129 496 602 970 7.00E-79 303.5 6596 cellular
organisms;Eukaryota;Opisthokonta;Metazoa;Eumetazoa;Bilateria;Protostomia;
Spiralia;Lophotrochozoa;Mollusca;Bivalvia;Autobranchia;Heteroconchia;Euhe
terodonta;Imparidentia;Venerida;Veneroidea;Veneridae;Mercenaria;Mercenari
a mercenaria
UdeM00000021998 6596 394 UdeM00000021998 XP_045177779.1 37.4 562
338 9 73 631 247 797 4.70E-106 394 6596 cellular
organisms;Eukaryota;Opisthokonta;Metazoa;Eumetazoa;Bilateria;Protostomia;
Spiralia;Lophotrochozoa;Mollusca;Bivalvia;Autobranchia;Heteroconchia;Euhe
terodonta;Imparidentia;Venerida;Veneroidea;Veneridae;Mercenaria;Mercenari
a mercenaria
UdeM00000021999 45351 80.9 UdeM00000021999 XP_032226414.1 37.1 151
78 3 37 172 11 159 2.30E-12 80.9 45351 cellular
organisms;Eukaryota;Opisthokonta;Metazoa;Eumetazoa;Cnidaria;Anthozoa;Hexa
corallia;Actiniaria;Edwardsiidae;Nematostella;Nematostella vectensis
UdeM00000022000 6573 164.5 UdeM00000022000 XP_021358159.1 38.1 260
148 7 3 251 394 651 3.10E-37 164.5 6573 cellular
organisms;Eukaryota;Opisthokonta;Metazoa;Eumetazoa;Bilateria;Protostomia;
Spiralia;Lophotrochozoa;Mollusca;Bivalvia;Autobranchia;Pteriomorphia;Pect
inida;Pectinoidea;Pectinidae;Mizuhopecten;Mizuhopecten yessoensis
UdeM00000022002 29159 95.5 UdeM00000022002 XP_034330187.1 34.5 171
89 6 164 334 9 156 1.40E-16 95.5 29159 cellular
organisms;Eukaryota;Opisthokonta;Metazoa;Eumetazoa;Bilateria;Protostomia;
Spiralia;Lophotrochozoa;Mollusca;Bivalvia;Autobranchia;Pteriomorphia;Ostr
eida;Ostreoidea;Ostreidae;Crassostrea;Crassostrea gigas
UdeM00000022004 50429 92 UdeM00000022004 XP_022799946.1 31.8 157
103 3 312 468 6 158 2.20E-15 92 50429 cellular
organisms;Eukaryota;Opisthokonta;Metazoa;Eumetazoa;Cnidaria;Anthozoa;Hexa
corallia;Scleractinia;Astrocoeniina;Pocilloporidae;Stylophora;Stylophora
pistillata
UdeM00000022006 6596 115.5 UdeM00000022006 XP_045177776.1 29.5 244
157 5 133 365 803 1042 2.30E-22 115.5 6596 cellular
organisms;Eukaryota;Opisthokonta;Metazoa;Eumetazoa;Bilateria;Protostomia;
Spiralia;Lophotrochozoa;Mollusca;Bivalvia;Autobranchia;Heteroconchia;Euhe
terodonta;Imparidentia;Venerida;Veneroidea;Veneridae;Mercenaria;Mercenari
a mercenaria
UdeM00000022011 45351 91.3 UdeM00000022011 XP_032236999.1 35.8 151
81 2 9 144 11 160 1.50E-15 91.3 45351 cellular
organisms;Eukaryota;Opisthokonta;Metazoa;Eumetazoa;Cnidaria;Anthozoa;Hexa
corallia;Actiniaria;Edwardsiidae;Nematostella;Nematostella vectensis

UdeM00000022012	6596	125.6 UdeM00000022012	XP_045204184.1	41	166
92	3	2	164	60	222	6.30E-26	125.6 6596	cellular organisms;Eukaryota;Opisthokonta;Metazoa;Eumetazoa;Bilateria;Protostomia;Spiralia;Lophotrochozoa;Mollusca;Bivalvia;Autobranchia;Heteroconchia;Euheterodonta;Imparidentia;Venerida;Veneroidea;Veneridae;Mercenaria;Mercenaria mercenaria
UdeM00000022015	29159 96.7	UdeM00000022015	XP_034330187.1	33.7	172
97	5	240	409	9	165	7.70E-17	96.7	29159 cellular organisms;Eukaryota;Opisthokonta;Metazoa;Eumetazoa;Bilateria;Protostomia;Spiralia;Lophotrochozoa;Mollusca;Bivalvia;Autobranchia;Pteriomorphia;Ostreida;Ostreoidea;Ostreidae;Crassostrea;Crassostrea gigas
UdeM00000022017	6596	380.9 UdeM00000022017	XP_045177776.1	40.5	568
310	11	1	561	405	951	3.80E-102	380.9 6596	cellular organisms;Eukaryota;Opisthokonta;Metazoa;Eumetazoa;Bilateria;Protostomia;Spiralia;Lophotrochozoa;Mollusca;Bivalvia;Autobranchia;Heteroconchia;Euheterodonta;Imparidentia;Venerida;Veneroidea;Veneridae;Mercenaria;Mercenaria mercenaria
UdeM00000022018	6596	82.8	UdeM00000022018	XP_045161680.1	34.1	138
81	2	131	258	97	234	1.20E-12	82.8	6596	cellular organisms;Eukaryota;Opisthokonta;Metazoa;Eumetazoa;Bilateria;Protostomia;Spiralia;Lophotrochozoa;Mollusca;Bivalvia;Autobranchia;Heteroconchia;Euheterodonta;Imparidentia;Venerida;Veneroidea;Veneridae;Mercenaria;Mercenaria mercenaria
UdeM00000022019	6579	230.3 UdeM00000022019	XP_033728503.1	89.1	119
13	0	1	119	716	834	1.30E-57	230.3 6579	cellular organisms;Eukaryota;Opisthokonta;Metazoa;Eumetazoa;Bilateria;Protostomia;Spiralia;Lophotrochozoa;Mollusca;Bivalvia;Autobranchia;Pteriomorphia;Pectinida;Pectinoidea;Pectinidae;Pecten;Pecten maximus
UdeM00000022020	6573	201.4 UdeM00000022020	XP_021368509.1	84.2	114
13	1	1	109	859	972	6.30E-49	201.4 6573	cellular organisms;Eukaryota;Opisthokonta;Metazoa;Eumetazoa;Bilateria;Protostomia;Spiralia;Lophotrochozoa;Mollusca;Bivalvia;Autobranchia;Pteriomorphia;Pectinida;Pectinoidea;Pectinidae;Mizuhopecten;Mizuhopecten yessoensis
UdeM00000022021	6579	597.4 UdeM00000022021	XP_033728503.1	57.3	562
65	2	16	402	1014	1575	1.30E-167	597.4 6579	cellular organisms;Eukaryota;Opisthokonta;Metazoa;Eumetazoa;Bilateria;Protostomia;Spiralia;Lophotrochozoa;Mollusca;Bivalvia;Autobranchia;Pteriomorphia;Pectinida;Pectinoidea;Pectinidae;Pecten;Pecten maximus
UdeM00000022024	6596	60.5	UdeM00000022024	XP_045180775.1	29.9	107
66	1	85	182	1448	1554	2.80E-06	60.5	6596	cellular organisms;Eukaryota;Opisthokonta;Metazoa;Eumetazoa;Bilateria;Protostomia;Spiralia;Lophotrochozoa;Mollusca;Bivalvia;Autobranchia;Heteroconchia;Euheterodonta;Imparidentia;Venerida;Veneroidea;Veneridae;Mercenaria;Mercenaria mercenaria
UdeM00000022025	29159 99.4	UdeM00000022025	XP_034330187.1	37.4	171
84	6	164	334	9	156	9.90E-18	99.4	29159 cellular organisms;Eukaryota;Opisthokonta;Metazoa;Eumetazoa;Bilateria;Protostomia;Spiralia;Lophotrochozoa;Mollusca;Bivalvia;Autobranchia;Pteriomorphia;Ostreida;Ostreoidea;Ostreidae;Crassostrea;Crassostrea gigas
UdeM00000022026	29159 99.4	UdeM00000022026	XP_034330187.1	36.3	171
86	6	164	334	9	156	9.90E-18	99.4	29159 cellular organisms;Eukaryota;Opisthokonta;Metazoa;Eumetazoa;Bilateria;Protostomia;Spiralia;Lophotrochozoa;Mollusca;Bivalvia;Autobranchia;Pteriomorphia;Ostreida;Ostreoidea;Ostreidae;Crassostrea;Crassostrea gigas

UdeM00000022027 7574 79 UdeM00000022027 XP_023932360.1 24.3 210
131 6 30 237 1652 1835 2.30E-11 79 7574 cellular
organisms;Eukaryota;Opisthokonta;Metazoa;Eumetazoa;Bilateria;Protostomia;
Spiralia;Lophotrochozoa;Brachiopoda;Linguliformea;Lingulata;Lingulida;Lin
guloidea;Lingulidae;Lingula;Lingula anatina
UdeM00000022028 7574 79 UdeM00000022028 XP_023932360.1 24.3 210
131 6 30 237 1652 1835 2.50E-11 79 7574 cellular
organisms;Eukaryota;Opisthokonta;Metazoa;Eumetazoa;Bilateria;Protostomia;
Spiralia;Lophotrochozoa;Brachiopoda;Linguliformea;Lingulata;Lingulida;Lin
guloidea;Lingulidae;Lingula;Lingula anatina
UdeM00000022029 6596 318.5 UdeM00000022029 XP_045178416.1 47 372
145 3 110 429 4 375 1.30E-83 318.5 6596 cellular
organisms;Eukaryota;Opisthokonta;Metazoa;Eumetazoa;Bilateria;Protostomia;
Spiralia;Lophotrochozoa;Mollusca;Bivalvia;Autobranchia;Heteroconchia;Euhe
terodonta;Imparidentia;Venerida;Veneroidea;Veneridae;Mercenaria;Mercenari
a mercenaria
UdeM00000022030 1735272 275.4 UdeM00000022030 XP_041374011.1 58.3
206 84 1 1 204 512 717 5.90E-71 275.4 1735272
cellular
organisms;Eukaryota;Opisthokonta;Metazoa;Eumetazoa;Bilateria;Protostomia;
Spiralia;Lophotrochozoa;Mollusca;Gastropoda;Neomphalina;Neomphaloidea;Pel
tospiridae;Gigantopelta;Gigantopelta aegis
UdeM00000022031 1735272 292.7 UdeM00000022031 XP_041374011.1 57.1
224 94 1 1 222 494 717 3.90E-76 292.7 1735272
cellular
organisms;Eukaryota;Opisthokonta;Metazoa;Eumetazoa;Bilateria;Protostomia;
Spiralia;Lophotrochozoa;Mollusca;Gastropoda;Neomphalina;Neomphaloidea;Pel
tospiridae;Gigantopelta;Gigantopelta aegis
UdeM00000022032 1735272 414.8 UdeM00000022032 XP_041374011.1 43.2
486 270 4 12 494 10 492 1.50E-112 414.8 1735272
cellular
organisms;Eukaryota;Opisthokonta;Metazoa;Eumetazoa;Bilateria;Protostomia;
Spiralia;Lophotrochozoa;Mollusca;Gastropoda;Neomphalina;Neomphaloidea;Pel
tospiridae;Gigantopelta;Gigantopelta aegis
UdeM00000022033 6573 580.1 UdeM00000022033 XP_021341356.1 76.4 365
82 1 5 369 103 463 2.00E-162 580.1 6573 cellular
organisms;Eukaryota;Opisthokonta;Metazoa;Eumetazoa;Bilateria;Protostomia;
Spiralia;Lophotrochozoa;Mollusca;Bivalvia;Autobranchia;Pteriomorphia;Pect
inida;Pectinoidea;Pectinidae;Mizuhopecten;Mizuhopecten yessoensis
UdeM00000022035 400727 427.9 UdeM00000022035 XP_025104943.1 79.2
269 56 0 2 270 3 271 9.40E-117 427.9 400727
cellular
organisms;Eukaryota;Opisthokonta;Metazoa;Eumetazoa;Bilateria;Protostomia;
Spiralia;Lophotrochozoa;Mollusca;Gastropoda;Caenogastropoda;Architaeniogl
ossa;Ampullarioidea;Ampullariidae;Pomacea;Pomacea canaliculata
UdeM00000022036 6500 298.5 UdeM00000022036 XP_012945148.1 60.5 309
117 5 829 1134 25 331 3.80E-77 298.5 6500 cellular
organisms;Eukaryota;Opisthokonta;Metazoa;Eumetazoa;Bilateria;Protostomia;
Spiralia;Lophotrochozoa;Mollusca;Gastropoda;Heterobranchia;Euthyneura;Tec
tipleura;Aplysiida;Aplysioidea;Aplysiidae;Aplysia;Aplysia californica
UdeM00000022037 6579 1048.5 UdeM00000022037 XP_033728946.1 45.8
1236 600 16 1 1208 31 1224 1.30E-302 1048.5 6579
cellular
organisms;Eukaryota;Opisthokonta;Metazoa;Eumetazoa;Bilateria;Protostomia;

Spiralia;Lophotrochozoa;Mollusca;Bivalvia;Autobranchia;Pteriomorphia;Pectinida;Pectinoidea;Pectinidae;Pecten;Pecten maximus
UdeM00000022037 6579 108.6 UdeM00000022037 XP_033728946.1 34.3 178 100 4 2184 2360 2596 2757 1.10E-19 108.6 6579 cellular organisms;Eukaryota;Opisthokonta;Metazoa;Eumetazoa;Bilateria;Protostomia;Spiralia;Lophotrochozoa;Mollusca;Bivalvia;Autobranchia;Pteriomorphia;Pectinida;Pectinoidea;Pectinidae;Pecten;Pecten maximus
UdeM00000022039 6596 691.4 UdeM00000022039 XP_045178671.1 64.6 495 171 2 2 492 175 669 8.20E-196 691.4 6596 cellular organisms;Eukaryota;Opisthokonta;Metazoa;Eumetazoa;Bilateria;Protostomia;Spiralia;Lophotrochozoa;Mollusca;Bivalvia;Autobranchia;Heteroconchia;Euheterodonta;Imparidentia;Venerida;Veneroidea;Veneridae;Mercenaria;Mercenaria mercenaria
UdeM00000022040 6596 310.5 UdeM00000022040 XP_045178671.1 70 203 61 0 2 204 467 669 1.60E-81 310.5 6596 cellular organisms;Eukaryota;Opisthokonta;Metazoa;Eumetazoa;Bilateria;Protostomia;Spiralia;Lophotrochozoa;Mollusca;Bivalvia;Autobranchia;Heteroconchia;Euheterodonta;Imparidentia;Venerida;Veneroidea;Veneridae;Mercenaria;Mercenaria mercenaria
UdeM00000022041 6596 312.4 UdeM00000022041 XP_045178671.1 70.4 203 60 0 2 204 467 669 4.30E-82 312.4 6596 cellular organisms;Eukaryota;Opisthokonta;Metazoa;Eumetazoa;Bilateria;Protostomia;Spiralia;Lophotrochozoa;Mollusca;Bivalvia;Autobranchia;Heteroconchia;Euheterodonta;Imparidentia;Venerida;Veneroidea;Veneridae;Mercenaria;Mercenaria mercenaria
UdeM00000022042 29159 389 UdeM00000022042 XP_011428534.2 38.1 586 340 11 23 596 296 870 1.10E-104 389 29159 cellular organisms;Eukaryota;Opisthokonta;Metazoa;Eumetazoa;Bilateria;Protostomia;Spiralia;Lophotrochozoa;Mollusca;Bivalvia;Autobranchia;Pteriomorphia;Ostreida;Ostreoidea;Ostreidae;Crassostrea;Crassostrea gigas
UdeM00000007799 6573 76.6 UdeM00000007799 XP_021347744.1 43.9 82 46 0 6 87 7 88 2.20E-11 76.6 6573 cellular organisms;Eukaryota;Opisthokonta;Metazoa;Eumetazoa;Bilateria;Protostomia;Spiralia;Lophotrochozoa;Mollusca;Bivalvia;Autobranchia;Pteriomorphia;Pectinida;Pectinoidea;Pectinidae;Mizuhopecten;Mizuhopecten yessoensis
UdeM00000007801 6579 122.1 UdeM00000007801 XP_033739131.1 51.8 112 53 1 3 114 1260 1370 4.70E-25 122.1 6579 cellular organisms;Eukaryota;Opisthokonta;Metazoa;Eumetazoa;Bilateria;Protostomia;Spiralia;Lophotrochozoa;Mollusca;Bivalvia;Autobranchia;Pteriomorphia;Pectinida;Pectinoidea;Pectinidae;Pecten;Pecten maximus
UdeM00000007802 400727 493.4 UdeM00000007802 XP_025103991.1 71.2 479 119 4 2 471 3 471 3.10E-136 493.4 400727 cellular organisms;Eukaryota;Opisthokonta;Metazoa;Eumetazoa;Bilateria;Protostomia;Spiralia;Lophotrochozoa;Mollusca;Gastropoda;Caenogastropoda;Architaenioglossa;Ampullarioidea;Ampullariidae;Pomacea;Pomacea canaliculata
UdeM00000007803 7515 124.4 UdeM00000007803 XP_026479132.1 43.4 143 80 1 1 143 105 246 1.20E-25 124.4 7515 cellular organisms;Eukaryota;Opisthokonta;Metazoa;Eumetazoa;Bilateria;Protostomia;Ecdysozoa;Panarthropoda;Arthropoda;Mandibulata;Pancrustacea;Hexapoda;Insecta;Dicondylia;Pterygota;Neoptera;Endopterygota;Siphonaptera;Pulicomorpha;Pulicoidea;Pulicidae;Archaeopsyllinae;Ctenocephalides;Ctenocephalides felis

UdeM00000007804 7515 154.8 UdeM00000007804 XP_026479132.1 38.9 198
119 2 37 233 50 246 1.30E-34 154.8 7515 cellular
organisms;Eukaryota;Opisthokonta;Metazoa;Eumetazoa;Bilateria;Protostomia;
Ecdysozoa;Panarthropoda;Arthropoda;Mandibulata;Pancrustacea;Hexapoda;Inse
cta;Dicondylia;Pterygota;Neoptera;Endopterygota;Siphonaptera;Pulicomorpha
;Pulicoidea;Pulicidae;Archaeopsyllinae;Ctenocephalides;Ctenocephalides
felis
UdeM00000007805 6596 392.5 UdeM00000007805 XP_045161160.1 59.9 324
125 2 1 319 1 324 5.70E-106 392.5 6596 cellular
organisms;Eukaryota;Opisthokonta;Metazoa;Eumetazoa;Bilateria;Protostomia;
Spiralia;Lophotrochozoa;Mollusca;Bivalvia;Autobranchia;Heteroconchia;Euhe
terodonta;Imparidentia;Venerida;Veneroidea;Veneridae;Mercenaria;Mercenari
a mercenaria
UdeM00000007806 6579 320.5 UdeM00000007806 XP_033734733.1 32.5 1101
611 37 1 1067 1 1003 8.20E-84 320.5 6579 cellular
organisms;Eukaryota;Opisthokonta;Metazoa;Eumetazoa;Bilateria;Protostomia;
Spiralia;Lophotrochozoa;Mollusca;Bivalvia;Autobranchia;Pteriomorphia;Pect
inida;Pectinoidea;Pectinidae;Pecten;Pecten maximus
UdeM00000007807 6596 1614 UdeM00000007807 XP_045187153.1 89.9 884
89 0 4 887 7 890 0.00E+00 1614 6596 cellular
organisms;Eukaryota;Opisthokonta;Metazoa;Eumetazoa;Bilateria;Protostomia;
Spiralia;Lophotrochozoa;Mollusca;Bivalvia;Autobranchia;Heteroconchia;Euhe
terodonta;Imparidentia;Venerida;Veneroidea;Veneridae;Mercenaria;Mercenari
a mercenaria
UdeM00000007808 6596 62 UdeM00000007808 XP_045167285.1 36.8 87
53 1 7 91 159 245 5.00E-07 62 6596 cellular
organisms;Eukaryota;Opisthokonta;Metazoa;Eumetazoa;Bilateria;Protostomia;
Spiralia;Lophotrochozoa;Mollusca;Bivalvia;Autobranchia;Heteroconchia;Euhe
terodonta;Imparidentia;Venerida;Veneroidea;Veneridae;Mercenaria;Mercenari
a mercenaria
UdeM00000007809 36176 76.3 UdeM00000007809 XP_043552272.1 59.4 64
26 0 1 64 1 64 2.10E-11 76.3 36176 cellular
organisms;Eukaryota;Opisthokonta;Metazoa;Eumetazoa;Bilateria;Deuterostomi
a;Chordata;Craniata;Vertebrata;Gnathostomata;Chondrichthyes;Elasmobranchi
i;Selachii;Galeomorphii;Galeoidea;Orectolobiformes;Hemiscylliidae;Chilosc
yllium;Chiloscyllium plagiosum
UdeM00000007810 1415580 87.4 UdeM00000007810 XP_030075314.1 41.1
107 62 1 1 107 1 106 1.20E-14 87.4 1415580
cellular
organisms;Eukaryota;Opisthokonta;Metazoa;Eumetazoa;Bilateria;Deuterostomi
a;Chordata;Craniata;Vertebrata;Gnathostomata;Teleostomi;Euteleostomi;Sarc
opterygii;Dipnotetrapodomorpha;Tetrapoda;Amphibia;Gymnophiona;Siphonopida
e;Microcaecilia;Microcaecilia unicolor
UdeM00000007811 7757 488 UdeM00000007811 XP_032835197.1 79.5 308
62 1 1 308 52 358 1.20E-134 488 7757 cellular
organisms;Eukaryota;Opisthokonta;Metazoa;Eumetazoa;Bilateria;Deuterostomi
a;Chordata;Craniata;Vertebrata;Cyclostomata;Hyperoartia;Petromyzontiforme
s;Petromyzontidae;Petromyzon;Petromyzon marinus
UdeM00000007812 1415580 109 UdeM00000007812 XP_030075314.1 72.2
72 20 0 2 73 554 625 2.60E-21 109 1415580
cellular
organisms;Eukaryota;Opisthokonta;Metazoa;Eumetazoa;Bilateria;Deuterostomi
a;Chordata;Craniata;Vertebrata;Gnathostomata;Teleostomi;Euteleostomi;Sarc

opterygii;Dipnotetrapodomorpha;Tetrapoda;Amphibia;Gymnophiona;Siphonopida e;Microcaecilia;Microcaecilia unicolor
UdeM00000007813 6596 400.6 UdeM00000007813 XP_045167193.1 38.6 694 337 14 6 673 4 634 4.00E-108 400.6 6596 cellular organisms;Eukaryota;Opisthokonta;Metazoa;Eumetazoa;Bilateria;Protostomia; Spiralia;Lophotrochozoa;Mollusca;Bivalvia;Autobranchia;Heteroconchia;Euhe terodonta;Imparidentia;Venerida;Veneroidea;Veneridae;Mercenaria;Mercenari a mercenaria
UdeM00000007814 6596 736.9 UdeM00000007814 XP_045167204.1 56.6 831 301 18 1 780 1 822 4.10E-209 736.9 6596 cellular organisms;Eukaryota;Opisthokonta;Metazoa;Eumetazoa;Bilateria;Protostomia; Spiralia;Lophotrochozoa;Mollusca;Bivalvia;Autobranchia;Heteroconchia;Euhe terodonta;Imparidentia;Venerida;Veneroidea;Veneridae;Mercenaria;Mercenari a mercenaria
UdeM00000007815 6565 528.9 UdeM00000007815 XP_022332113.1 83.8 320 17 1 1 285 113 432 4.80E-147 528.9 6565 cellular organisms;Eukaryota;Opisthokonta;Metazoa;Eumetazoa;Bilateria;Protostomia; Spiralia;Lophotrochozoa;Mollusca;Bivalvia;Autobranchia;Pteriomorphia;Ostr eida;Ostreoidea;Ostreidae;Crassostrea;Crassostrea virginica
UdeM00000007816 6500 179.1 UdeM00000007816 XP_005102107.1 66.2 142 45 2 3 144 5 143 4.30E-42 179.1 6500 cellular organisms;Eukaryota;Opisthokonta;Metazoa;Eumetazoa;Bilateria;Protostomia; Spiralia;Lophotrochozoa;Mollusca;Gastropoda;Heterobranchia;Euthyneura;Tec tipleura;Aplysiida;Aplysioidea;Aplysiidae;Aplysia;Aplysia californica
UdeM00000007817 1410327 151 UdeM00000007817 XP_012759400.1 41.1 185 103 3 47 231 618 796 4.50E-33 151 1410327 cellular organisms;Eukaryota;Amoebozoa;Evosea;Eumycetozoa;Dictyostelia;Acytostelia les;Acytosteliaceae;Acytostelium;Acytostelium subglobosum;Acytostelium subglobosum LB1
UdeM00000007818 6596 718.4 UdeM00000007818 XP_045167260.1 57.2 671 197 5 6 673 4 587 8.60E-204 718.4 6596 cellular organisms;Eukaryota;Opisthokonta;Metazoa;Eumetazoa;Bilateria;Protostomia; Spiralia;Lophotrochozoa;Mollusca;Bivalvia;Autobranchia;Heteroconchia;Euhe terodonta;Imparidentia;Venerida;Veneroidea;Veneridae;Mercenaria;Mercenari a mercenaria
UdeM00000007819 6596 744.6 UdeM00000007819 XP_045167260.1 63.8 602 197 5 6 604 4 587 1.00E-211 744.6 6596 cellular organisms;Eukaryota;Opisthokonta;Metazoa;Eumetazoa;Bilateria;Protostomia; Spiralia;Lophotrochozoa;Mollusca;Bivalvia;Autobranchia;Heteroconchia;Euhe terodonta;Imparidentia;Venerida;Veneroidea;Veneridae;Mercenaria;Mercenari a mercenaria
UdeM00000007820 6579 541.6 UdeM00000007820 XP_033735477.1 58.9 462 177 6 27 476 37 497 1.00E-150 541.6 6579 cellular organisms;Eukaryota;Opisthokonta;Metazoa;Eumetazoa;Bilateria;Protostomia; Spiralia;Lophotrochozoa;Mollusca;Bivalvia;Autobranchia;Pteriomorphia;Pect inida;Pectinoidea;Pectinidae;Pecten;Pecten maximus
UdeM00000007821 6579 548.1 UdeM00000007821 XP_033735477.1 59.7 464 172 7 26 476 36 497 1.10E-152 548.1 6579 cellular organisms;Eukaryota;Opisthokonta;Metazoa;Eumetazoa;Bilateria;Protostomia; Spiralia;Lophotrochozoa;Mollusca;Bivalvia;Autobranchia;Pteriomorphia;Pect inida;Pectinoidea;Pectinidae;Pecten;Pecten maximus
UdeM00000007822 6596 257.7 UdeM00000007822 XP_045167298.1 49.1 273 123 2 109 368 119 388 2.50E-65 257.7 6596 cellular

organisms;Eukaryota;Opisthokonta;Metazoa;Eumetazoa;Bilateria;Protostomia;Spiralia;Lophotrochozoa;Mollusca;Bivalvia;Autobranchia;Heteroconchia;Euheterodonta;Imparidentia;Venerida;Veneroidea;Veneridae;Mercenaria;Mercenaria mercenaria
UdeM00000007823 6596 340.1 UdeM00000007823 XP_045191221.1 41.9 415
225 4 13 426 12 411 4.10E-90 340.1 6596 cellular
organisms;Eukaryota;Opisthokonta;Metazoa;Eumetazoa;Bilateria;Protostomia;Spiralia;Lophotrochozoa;Mollusca;Bivalvia;Autobranchia;Heteroconchia;Euheterodonta;Imparidentia;Venerida;Veneroidea;Veneridae;Mercenaria;Mercenaria mercenaria
UdeM00000007824 6596 369.4 UdeM00000007824 XP_045191221.1 45.6 410
218 5 9 416 7 413 6.10E-99 369.4 6596 cellular
organisms;Eukaryota;Opisthokonta;Metazoa;Eumetazoa;Bilateria;Protostomia;Spiralia;Lophotrochozoa;Mollusca;Bivalvia;Autobranchia;Heteroconchia;Euheterodonta;Imparidentia;Venerida;Veneroidea;Veneridae;Mercenaria;Mercenaria mercenaria
UdeM00000007825 6596 369.4 UdeM00000007825 XP_045191221.1 45.6 410
218 5 9 416 7 413 6.10E-99 369.4 6596 cellular
organisms;Eukaryota;Opisthokonta;Metazoa;Eumetazoa;Bilateria;Protostomia;Spiralia;Lophotrochozoa;Mollusca;Bivalvia;Autobranchia;Heteroconchia;Euheterodonta;Imparidentia;Venerida;Veneroidea;Veneridae;Mercenaria;Mercenaria mercenaria
UdeM00000007826 6596 369.4 UdeM00000007826 XP_045191221.1 45.6 410
218 5 9 416 7 413 6.10E-99 369.4 6596 cellular
organisms;Eukaryota;Opisthokonta;Metazoa;Eumetazoa;Bilateria;Protostomia;Spiralia;Lophotrochozoa;Mollusca;Bivalvia;Autobranchia;Heteroconchia;Euheterodonta;Imparidentia;Venerida;Veneroidea;Veneridae;Mercenaria;Mercenaria mercenaria
UdeM00000007827 6573 307.8 UdeM00000007827 XP_021347271.1 54 359
135 10 1 342 1 346 1.90E-80 307.8 6573 cellular
organisms;Eukaryota;Opisthokonta;Metazoa;Eumetazoa;Bilateria;Protostomia;Spiralia;Lophotrochozoa;Mollusca;Bivalvia;Autobranchia;Pteriomorphia;Pectinida;Pectinoidea;Pectinidae;Mizuhopecten;Mizuhopecten yessoensis
UdeM00000007828 6579 351.7 UdeM00000007828 XP_033735481.1 60.1 376
121 11 1 364 1 359 1.10E-93 351.7 6579 cellular
organisms;Eukaryota;Opisthokonta;Metazoa;Eumetazoa;Bilateria;Protostomia;Spiralia;Lophotrochozoa;Mollusca;Bivalvia;Autobranchia;Pteriomorphia;Pectinida;Pectinoidea;Pectinidae;Pecten;Pecten maximus
UdeM00000007830 29159 401.4 UdeM00000007830 XP_019926412.2 54.5 398
165 6 1 393 1 387 1.40E-108 401.4 29159 cellular
organisms;Eukaryota;Opisthokonta;Metazoa;Eumetazoa;Bilateria;Protostomia;Spiralia;Lophotrochozoa;Mollusca;Bivalvia;Autobranchia;Pteriomorphia;Ostreida;Ostreoidea;Ostreidae;Crassostrea;Crassostrea gigas
UdeM00000007831 6579 371.3 UdeM00000007831 XP_033735483.1 56.2 381
143 10 2 367 9 380 1.40E-99 371.3 6579 cellular
organisms;Eukaryota;Opisthokonta;Metazoa;Eumetazoa;Bilateria;Protostomia;Spiralia;Lophotrochozoa;Mollusca;Bivalvia;Autobranchia;Pteriomorphia;Pectinida;Pectinoidea;Pectinidae;Pecten;Pecten maximus
UdeM00000007832 667725 178.7 UdeM00000007832 XP_014153486.1 54.4
149 67 1 1 148 1 149 5.40E-42 178.7 667725
cellular
organisms;Eukaryota;Opisthokonta;Ichthyosporea;Ichthyophonida;Sphaeroforma;Sphaeroforma arctica;Sphaeroforma arctica JP610

UdeM00000007833 6573 171.8 UdeM00000007833 XP_021360372.1 57.9 140
57 1 1101 1240 620 757 5.60E-39 171.8 6573 cellular organisms;Eukaryota;Opisthokonta;Metazoa;Eumetazoa;Bilateria;Protostomia;Spiralia;Lophotrochozoa;Mollusca;Bivalvia;Autobranchia;Pteriomorphia;Pectinida;Pectinoidea;Pectinidae;Mizuhopecten;Mizuhopecten yessoensis
UdeM00000007835 6596 347.1 UdeM00000007835 XP_045157154.1 40.7 450
262 3 26 472 113 560 4.50E-92 347.1 6596 cellular organisms;Eukaryota;Opisthokonta;Metazoa;Eumetazoa;Bilateria;Protostomia;Spiralia;Lophotrochozoa;Mollusca;Bivalvia;Autobranchia;Heteroconchia;Euheterodonta;Imparidentia;Venerida;Veneroidea;Veneridae;Mercenaria;Mercenaria mercenaria
UdeM00000007836 6596 318.2 UdeM00000007836 XP_045187665.1 60.3 252
98 2 8 259 11 260 1.10E-83 318.2 6596 cellular organisms;Eukaryota;Opisthokonta;Metazoa;Eumetazoa;Bilateria;Protostomia;Spiralia;Lophotrochozoa;Mollusca;Bivalvia;Autobranchia;Heteroconchia;Euheterodonta;Imparidentia;Venerida;Veneroidea;Veneridae;Mercenaria;Mercenaria mercenaria
UdeM00000007837 6573 96.3 UdeM00000007837 XP_021367970.1 31 155
107 0 126 280 82 236 7.00E-17 96.3 6573 cellular organisms;Eukaryota;Opisthokonta;Metazoa;Eumetazoa;Bilateria;Protostomia;Spiralia;Lophotrochozoa;Mollusca;Bivalvia;Autobranchia;Pteriomorphia;Pectinida;Pectinoidea;Pectinidae;Mizuhopecten;Mizuhopecten yessoensis
UdeM00000007838 6579 473.8 UdeM00000007838 XP_033735451.1 41.5 776
392 14 1 748 1 742 4.20E-130 473.8 6579 cellular organisms;Eukaryota;Opisthokonta;Metazoa;Eumetazoa;Bilateria;Protostomia;Spiralia;Lophotrochozoa;Mollusca;Bivalvia;Autobranchia;Pteriomorphia;Pectinida;Pectinoidea;Pectinidae;Pecten;Pecten maximus
UdeM00000007839 6596 445.3 UdeM00000007839 XP_045167688.1 59.6 364
145 2 7 368 8 371 9.80E-122 445.3 6596 cellular organisms;Eukaryota;Opisthokonta;Metazoa;Eumetazoa;Bilateria;Protostomia;Spiralia;Lophotrochozoa;Mollusca;Bivalvia;Autobranchia;Heteroconchia;Euheterodonta;Imparidentia;Venerida;Veneroidea;Veneridae;Mercenaria;Mercenaria mercenaria
UdeM00000007840 10224 122.1 UdeM00000007840 XP_006813844.1 41.5 147
86 0 2 148 126 272 6.40E-25 122.1 10224 cellular organisms;Eukaryota;Opisthokonta;Metazoa;Eumetazoa;Bilateria;Deuterostomia;Hemichordata;Enteropneusta;Harrimaniidae;Saccoglossus;Saccoglossus kowalevskii
UdeM00000007841 10224 120.2 UdeM00000007841 XP_006813844.1 41.4 145
85 0 1 145 128 272 2.40E-24 120.2 10224 cellular organisms;Eukaryota;Opisthokonta;Metazoa;Eumetazoa;Bilateria;Deuterostomia;Hemichordata;Enteropneusta;Harrimaniidae;Saccoglossus;Saccoglossus kowalevskii
UdeM00000007842 6596 151 UdeM00000007842 XP_045167688.1 72.5 102
28 0 252 353 371 472 2.90E-33 151 6596 cellular organisms;Eukaryota;Opisthokonta;Metazoa;Eumetazoa;Bilateria;Protostomia;Spiralia;Lophotrochozoa;Mollusca;Bivalvia;Autobranchia;Heteroconchia;Euheterodonta;Imparidentia;Venerida;Veneroidea;Veneridae;Mercenaria;Mercenaria mercenaria
UdeM00000007845 29159 400.2 UdeM00000007845 XP_034311318.1 36.3 681
348 16 157 768 1 664 6.50E-108 400.2 29159 cellular organisms;Eukaryota;Opisthokonta;Metazoa;Eumetazoa;Bilateria;Protostomia;Spiralia;Lophotrochozoa;Mollusca;Bivalvia;Autobranchia;Pteriomorphia;Ostreida;Ostreoidea;Ostreidae;Crassostrea;Crassostrea gigas

UdeM00000007846 6579 198.4 UdeM00000007846 XP_033736448.1 39 272
155 5 5 267 10 279 2.20E-47 198.4 6579 cellular
organisms;Eukaryota;Opisthokonta;Metazoa;Eumetazoa;Bilateria;Protostomia;
Spiralia;Lophotrochozoa;Mollusca;Bivalvia;Autobranchia;Pteriomorphia;Pect
inida;Pectinoidea;Pectinidae;Pecten;Pecten maximus
UdeM00000007847 225164 320.1 UdeM00000007847 XP_009061546.1 75.5
196 48 0 1 196 1 196 2.00E-84 320.1 225164
cellular
organisms;Eukaryota;Opisthokonta;Metazoa;Eumetazoa;Bilateria;Protostomia;
Spiralia;Lophotrochozoa;Mollusca;Gastropoda;Patellogastropoda;Lottioidea;
Lottiidae;Lottia;Lottia gigantea
UdeM00000007848 6573 914.1 UdeM00000007848 XP_021343879.1 40.1 1305
711 20 4 1265 5 1281 2.00E-262 914.1 6573 cellular
organisms;Eukaryota;Opisthokonta;Metazoa;Eumetazoa;Bilateria;Protostomia;
Spiralia;Lophotrochozoa;Mollusca;Bivalvia;Autobranchia;Pteriomorphia;Pect
inida;Pectinoidea;Pectinidae;Mizuhopecten;Mizuhopecten yessoensis
UdeM00000007849 6526 68.9 UdeM00000007849 XP_013069545.1 28.4 176
107 5 46 218 1099 1258 9.70E-09 68.9 6526 cellular
organisms;Eukaryota;Opisthokonta;Metazoa;Eumetazoa;Bilateria;Protostomia;
Spiralia;Lophotrochozoa;Mollusca;Gastropoda;Heterobranchia;Euthyneura;Pan
pulmonata;Hygrophila;Lymnaeoidea;Planorbidae;Biomphalaria;Biomphalaria
glabrata
UdeM00000007850 6596 837.4 UdeM00000007850 XP_045167527.1 53.9 798
343 4 6 802 134 907 1.50E-239 837.4 6596 cellular
organisms;Eukaryota;Opisthokonta;Metazoa;Eumetazoa;Bilateria;Protostomia;
Spiralia;Lophotrochozoa;Mollusca;Bivalvia;Autobranchia;Heteroconchia;Euhe
terodonta;Imparidentia;Venerida;Veneroidea;Veneridae;Mercenaria;Mercenari
a mercenaria
UdeM00000007852 6565 731.9 UdeM00000007852 XP_022334489.1 55 645
282 7 5 644 12 653 7.50E-208 731.9 6565 cellular
organisms;Eukaryota;Opisthokonta;Metazoa;Eumetazoa;Bilateria;Protostomia;
Spiralia;Lophotrochozoa;Mollusca;Bivalvia;Autobranchia;Pteriomorphia;Ostr
eida;Ostreoidea;Ostreidae;Crassostrea;Crassostrea virginica
UdeM00000007853 29159 454.5 UdeM00000007853 XP_034332465.1 49.2 496
240 8 80 565 72 565 2.20E-124 454.5 29159 cellular
organisms;Eukaryota;Opisthokonta;Metazoa;Eumetazoa;Bilateria;Protostomia;
Spiralia;Lophotrochozoa;Mollusca;Bivalvia;Autobranchia;Pteriomorphia;Ostr
eida;Ostreoidea;Ostreidae;Crassostrea;Crassostrea gigas
UdeM00000007854 6596 121.3 UdeM00000007854 XP_045182392.1 42.5 153
84 3 1 153 432 580 1.50E-24 121.3 6596 cellular
organisms;Eukaryota;Opisthokonta;Metazoa;Eumetazoa;Bilateria;Protostomia;
Spiralia;Lophotrochozoa;Mollusca;Bivalvia;Autobranchia;Heteroconchia;Euhe
terodonta;Imparidentia;Venerida;Veneroidea;Veneridae;Mercenaria;Mercenari
a mercenaria
UdeM00000007855 29159 448.4 UdeM00000007855 XP_034332465.1 49.1 499
237 7 19 505 72 565 1.40E-122 448.4 29159 cellular
organisms;Eukaryota;Opisthokonta;Metazoa;Eumetazoa;Bilateria;Protostomia;
Spiralia;Lophotrochozoa;Mollusca;Bivalvia;Autobranchia;Pteriomorphia;Ostr
eida;Ostreoidea;Ostreidae;Crassostrea;Crassostrea gigas
UdeM00000007856 6565 416.4 UdeM00000007856 XP_022333610.1 59.7 347
136 2 2 347 469 812 4.80E-113 416.4 6565 cellular
organisms;Eukaryota;Opisthokonta;Metazoa;Eumetazoa;Bilateria;Protostomia;
Spiralia;Lophotrochozoa;Mollusca;Bivalvia;Autobranchia;Pteriomorphia;Ostr
eida;Ostreoidea;Ostreidae;Crassostrea;Crassostrea virginica

UdeM00000007857 29159 421.4 UdeM00000007857 XP_011442695.2 43.9 558
289 8 23 562 819 1370 1.90E-114 421.4 29159 cellular
organisms;Eukaryota;Opisthokonta;Metazoa;Eumetazoa;Bilateria;Protostomia;
Spiralia;Lophotrochozoa;Mollusca;Bivalvia;Autobranchia;Pteriomorphia;Ostr
eida;Ostreoidea;Ostreidae;Crassostrea;Crassostrea gigas
UdeM00000007858 6596 718.8 UdeM00000007858 XP_045187532.1 42.5 1060
382 17 1 852 1 1041 8.30E-204 718.8 6596 cellular
organisms;Eukaryota;Opisthokonta;Metazoa;Eumetazoa;Bilateria;Protostomia;
Spiralia;Lophotrochozoa;Mollusca;Bivalvia;Autobranchia;Heteroconchia;Euhe
terodonta;Imparidentia;Venerida;Veneroidea;Veneridae;Mercenaria;Mercenari
a mercenaria
UdeM00000007859 6596 357.8 UdeM00000007859 XP_045187532.1 46.6 455
186 10 1 413 1 440 1.80E-95 357.8 6596 cellular
organisms;Eukaryota;Opisthokonta;Metazoa;Eumetazoa;Bilateria;Protostomia;
Spiralia;Lophotrochozoa;Mollusca;Bivalvia;Autobranchia;Heteroconchia;Euhe
terodonta;Imparidentia;Venerida;Veneroidea;Veneridae;Mercenaria;Mercenari
a mercenaria
UdeM00000007860 6596 656.4 UdeM00000007860 XP_045187532.1 39.7 1056
392 15 1 826 1 1041 4.90E-185 656.4 6596 cellular
organisms;Eukaryota;Opisthokonta;Metazoa;Eumetazoa;Bilateria;Protostomia;
Spiralia;Lophotrochozoa;Mollusca;Bivalvia;Autobranchia;Heteroconchia;Euhe
terodonta;Imparidentia;Venerida;Veneroidea;Veneridae;Mercenaria;Mercenari
a mercenaria
UdeM00000007862 6596 139 UdeM00000007862 XP_045162402.1 45.3 148
81 0 50 197 412 559 6.30E-30 139 6596 cellular
organisms;Eukaryota;Opisthokonta;Metazoa;Eumetazoa;Bilateria;Protostomia;
Spiralia;Lophotrochozoa;Mollusca;Bivalvia;Autobranchia;Heteroconchia;Euhe
terodonta;Imparidentia;Venerida;Veneroidea;Veneridae;Mercenaria;Mercenari
a mercenaria
UdeM00000007863 7654 224.2 UdeM00000007863 XP_041477967.1 60.5 177
70 0 2 178 6 182 1.70E-55 224.2 7654 cellular
organisms;Eukaryota;Opisthokonta;Metazoa;Eumetazoa;Bilateria;Deuterostomi
a;Echinodermata;Eleutherozoa;Echinozoa;Echinoidea;Euechinoidea;Echinacea;
Temnopleuroida;Toxopneustidae;Lytechinus;Lytechinus variegatus
UdeM00000007864 6579 224.9 UdeM00000007864 XP_033737260.1 50.2 221
101 2 1 221 1 212 1.00E-55 224.9 6579 cellular
organisms;Eukaryota;Opisthokonta;Metazoa;Eumetazoa;Bilateria;Protostomia;
Spiralia;Lophotrochozoa;Mollusca;Bivalvia;Autobranchia;Pteriomorphia;Pect
inida;Pectinoidea;Pectinidae;Pecten;Pecten maximus
UdeM00000007866 6596 133.7 UdeM00000007866 XP_045161061.1 52 123
59 0 1 123 79 201 1.70E-28 133.7 6596 cellular
organisms;Eukaryota;Opisthokonta;Metazoa;Eumetazoa;Bilateria;Protostomia;
Spiralia;Lophotrochozoa;Mollusca;Bivalvia;Autobranchia;Heteroconchia;Euhe
terodonta;Imparidentia;Venerida;Veneroidea;Veneridae;Mercenaria;Mercenari
a mercenaria
UdeM00000007865 6596 237.7 UdeM00000007865 XP_045161061.1 57.2 201
85 1 1 200 1 201 1.30E-59 237.7 6596 cellular
organisms;Eukaryota;Opisthokonta;Metazoa;Eumetazoa;Bilateria;Protostomia;
Spiralia;Lophotrochozoa;Mollusca;Bivalvia;Autobranchia;Heteroconchia;Euhe
terodonta;Imparidentia;Venerida;Veneroidea;Veneridae;Mercenaria;Mercenari
a mercenaria
UdeM00000007867 6573 383.6 UdeM00000007867 XP_021340111.1 50.2 426
184 7 22 427 41 458 3.40E-103 383.6 6573 cellular
organisms;Eukaryota;Opisthokonta;Metazoa;Eumetazoa;Bilateria;Protostomia;

Spiralia;Lophotrochozoa;Mollusca;Bivalvia;Autobranchia;Pteriomorphia;Pectinida;Pectinoidea;Pectinidae;Mizuhopecten;Mizuhopecten yessoensis
UdeM00000007868 6579 731.5 UdeM00000007868 XP_033735883.1 47.9 771 367 12 58 803 14 774 1.20E-207 731.5 6579 cellular organisms;Eukaryota;Opisthokonta;Metazoa;Eumetazoa;Bilateria;Protostomia;Spiralia;Lophotrochozoa;Mollusca;Bivalvia;Autobranchia;Pteriomorphia;Pectinida;Pectinoidea;Pectinidae;Pecten;Pecten maximus
UdeM00000007869 6596 124.8 UdeM00000007869 XP_045178837.1 44.6 139 71 3 145 279 77 213 1.70E-25 124.8 6596 cellular organisms;Eukaryota;Opisthokonta;Metazoa;Eumetazoa;Bilateria;Protostomia;Spiralia;Lophotrochozoa;Mollusca;Bivalvia;Autobranchia;Heteroconchia;Euheterodonta;Imparidentia;Venerida;Veneroidea;Veneridae;Mercenaria;Mercenaria mercenaria
UdeM00000007870 7725 141.7 UdeM00000007870 XP_039250069.1 70.2 104 30 1 1 104 313 415 6.30E-31 141.7 7725 cellular organisms;Eukaryota;Opisthokonta;Metazoa;Eumetazoa;Bilateria;Deuterostomia;Chordata;Tunicata;Ascidiacea;Stolidobranchia;Styelidae;Styela;Styela clava
UdeM00000007871 6596 149.1 UdeM00000007871 XP_045173814.1 40.7 302 142 14 89 359 29 324 1.10E-32 149.1 6596 cellular organisms;Eukaryota;Opisthokonta;Metazoa;Eumetazoa;Bilateria;Protostomia;Spiralia;Lophotrochozoa;Mollusca;Bivalvia;Autobranchia;Heteroconchia;Euheterodonta;Imparidentia;Venerida;Veneroidea;Veneridae;Mercenaria;Mercenaria mercenaria
UdeM00000007872 6596 495.7 UdeM00000007872 XP_045173803.1 48.6 541 248 7 5 544 3 514 2.10E-136 495.7 6596 cellular organisms;Eukaryota;Opisthokonta;Metazoa;Eumetazoa;Bilateria;Protostomia;Spiralia;Lophotrochozoa;Mollusca;Bivalvia;Autobranchia;Heteroconchia;Euheterodonta;Imparidentia;Venerida;Veneroidea;Veneridae;Mercenaria;Mercenaria mercenaria
UdeM00000007873 6596 501.9 UdeM00000007873 XP_045188256.1 51.2 502 218 8 3 496 122 604 1.20E-138 501.9 6596 cellular organisms;Eukaryota;Opisthokonta;Metazoa;Eumetazoa;Bilateria;Protostomia;Spiralia;Lophotrochozoa;Mollusca;Bivalvia;Autobranchia;Heteroconchia;Euheterodonta;Imparidentia;Venerida;Veneroidea;Veneridae;Mercenaria;Mercenaria mercenaria
UdeM00000007874 6596 184.5 UdeM00000007874 XP_045202545.1 49 202 77 5 10 186 10 210 1.20E-43 184.5 6596 cellular organisms;Eukaryota;Opisthokonta;Metazoa;Eumetazoa;Bilateria;Protostomia;Spiralia;Lophotrochozoa;Mollusca;Bivalvia;Autobranchia;Heteroconchia;Euheterodonta;Imparidentia;Venerida;Veneroidea;Veneridae;Mercenaria;Mercenaria mercenaria
UdeM00000007875 6579 198.7 UdeM00000007875 XP_033741736.1 53.5 187 85 1 1 185 671 857 6.80E-48 198.7 6579 cellular organisms;Eukaryota;Opisthokonta;Metazoa;Eumetazoa;Bilateria;Protostomia;Spiralia;Lophotrochozoa;Mollusca;Bivalvia;Autobranchia;Pteriomorphia;Pectinida;Pectinoidea;Pectinidae;Pecten;Pecten maximus
UdeM00000007876 6596 340.1 UdeM00000007876 XP_045162348.1 81.1 206 36 2 115 318 54 258 3.80E-90 340.1 6596 cellular organisms;Eukaryota;Opisthokonta;Metazoa;Eumetazoa;Bilateria;Protostomia;Spiralia;Lophotrochozoa;Mollusca;Bivalvia;Autobranchia;Heteroconchia;Euheterodonta;Imparidentia;Venerida;Veneroidea;Veneridae;Mercenaria;Mercenaria mercenaria

UdeM00000007877 6579 217.6 UdeM00000007877 XP_033734184.1 37.3 373
206 9 3 356 20 383 2.60E-53 217.6 6579 cellular
organisms;Eukaryota;Opisthokonta;Metazoa;Eumetazoa;Bilateria;Protostomia;
Spiralia;Lophotrochozoa;Mollusca;Bivalvia;Autobranchia;Pteriomorphia;Pect
inida;Pectinoidea;Pectinidae;Pecten;Pecten maximus
UdeM00000007878 29159 223 UdeM00000007878 XP_034326637.1 37.9 359
200 8 8 351 6 356 6.00E-55 223 29159 cellular
organisms;Eukaryota;Opisthokonta;Metazoa;Eumetazoa;Bilateria;Protostomia;
Spiralia;Lophotrochozoa;Mollusca;Bivalvia;Autobranchia;Pteriomorphia;Ostr
eida;Ostreoidea;Ostreidae;Crassostrea;Crassostrea gigas
UdeM00000007879 6565 253.1 UdeM00000007879 XP_022334079.1 67.6 182
56 2 1 180 1 181 3.00E-64 253.1 6565 cellular
organisms;Eukaryota;Opisthokonta;Metazoa;Eumetazoa;Bilateria;Protostomia;
Spiralia;Lophotrochozoa;Mollusca;Bivalvia;Autobranchia;Pteriomorphia;Ostr
eida;Ostreoidea;Ostreidae;Crassostrea;Crassostrea virginica
UdeM00000007880 6579 496.5 UdeM00000007880 XP_033734608.1 44.6 583
295 7 44 613 2 569 4.80E-137 496.5 6579 cellular
organisms;Eukaryota;Opisthokonta;Metazoa;Eumetazoa;Bilateria;Protostomia;
Spiralia;Lophotrochozoa;Mollusca;Bivalvia;Autobranchia;Pteriomorphia;Pect
inida;Pectinoidea;Pectinidae;Pecten;Pecten maximus
UdeM00000007881 6579 85.1 UdeM00000007881 XP_033733680.1 30.9 220
127 5 17 214 11 227 1.20E-13 85.1 6579 cellular
organisms;Eukaryota;Opisthokonta;Metazoa;Eumetazoa;Bilateria;Protostomia;
Spiralia;Lophotrochozoa;Mollusca;Bivalvia;Autobranchia;Pteriomorphia;Pect
inida;Pectinoidea;Pectinidae;Pecten;Pecten maximus
UdeM00000007882 6565 158.7 UdeM00000007882 XP_022328708.1 48.5 169
86 1 1 168 1 169 7.10E-36 158.7 6565 cellular
organisms;Eukaryota;Opisthokonta;Metazoa;Eumetazoa;Bilateria;Protostomia;
Spiralia;Lophotrochozoa;Mollusca;Bivalvia;Autobranchia;Pteriomorphia;Ostr
eida;Ostreoidea;Ostreidae;Crassostrea;Crassostrea virginica
UdeM00000007883 6596 3191.4 UdeM00000007883 XP_045213071.1 47.8
3797 1696 61 1 3626 104 3785 0.00E+00 3191.4 6596
cellular
organisms;Eukaryota;Opisthokonta;Metazoa;Eumetazoa;Bilateria;Protostomia;
Spiralia;Lophotrochozoa;Mollusca;Bivalvia;Autobranchia;Heteroconchia;Euhe
terodonta;Imparidentia;Venerida;Veneroidea;Veneridae;Mercenaria;Mercenari
a mercenaria
UdeM00000007885 6596 694.5 UdeM00000007885 XP_045175481.1 71.3 471
130 2 4 469 16 486 9.40E-197 694.5 6596 cellular
organisms;Eukaryota;Opisthokonta;Metazoa;Eumetazoa;Bilateria;Protostomia;
Spiralia;Lophotrochozoa;Mollusca;Bivalvia;Autobranchia;Heteroconchia;Euhe
terodonta;Imparidentia;Venerida;Veneroidea;Veneridae;Mercenaria;Mercenari
a mercenaria
UdeM00000007886 6596 360.1 UdeM00000007886 XP_045187095.1 86.4 206
28 0 1 206 1 206 1.80E-96 360.1 6596 cellular
organisms;Eukaryota;Opisthokonta;Metazoa;Eumetazoa;Bilateria;Protostomia;
Spiralia;Lophotrochozoa;Mollusca;Bivalvia;Autobranchia;Heteroconchia;Euhe
terodonta;Imparidentia;Venerida;Veneroidea;Veneridae;Mercenaria;Mercenari
a mercenaria
UdeM00000007887 6579 349.4 UdeM00000007887 XP_033733793.1 64.9 268
92 1 18 285 571 836 4.40E-93 349.4 6579 cellular
organisms;Eukaryota;Opisthokonta;Metazoa;Eumetazoa;Bilateria;Protostomia;
Spiralia;Lophotrochozoa;Mollusca;Bivalvia;Autobranchia;Pteriomorphia;Pect
inida;Pectinoidea;Pectinidae;Pecten;Pecten maximus

UdeM00000007888 151771 104.4 UdeM00000007888 XP_028408207.1 38.9
157 95 1 84 240 138 293 2.10E-19 104.4 151771
cellular
organisms;Eukaryota;Opisthokonta;Metazoa;Eumetazoa;Cnidaria;Anthozoa;Octo
corallia;Alcyonacea;Alcyoniina;Nephtheidae;Dendronephthya;Dendronephthya
gigantea
UdeM00000007889 70779 102.4 UdeM00000007889 XP_015764363.1 36 214
125 6 27 239 73 275 9.20E-19 102.4 70779 cellular
organisms;Eukaryota;Opisthokonta;Metazoa;Eumetazoa;Cnidaria;Anthozoa;Hexa
corallia;Scleractinia;Astrocoeniina;Acroporidae;Acropora;Acropora
digitifera
UdeM00000007890 6596 659.4 UdeM00000007890 XP_045215580.1 66.1 528
166 5 41 557 141 666 3.90E-186 659.4 6596 cellular
organisms;Eukaryota;Opisthokonta;Metazoa;Eumetazoa;Bilateria;Protostomia;
Spiralia;Lophotrochozoa;Mollusca;Bivalvia;Autobranchia;Heteroconchia;Euhe
terodonta;Imparidentia;Venerida;Veneroidea;Veneridae;Mercenaria;Mercenari
a mercenaria
UdeM00000007891 6596 721.8 UdeM00000007891 XP_045182724.1 54.4 1578
690 13 217 1791 1 1551 2.60E-204 721.8 6596 cellular
organisms;Eukaryota;Opisthokonta;Metazoa;Eumetazoa;Bilateria;Protostomia;
Spiralia;Lophotrochozoa;Mollusca;Bivalvia;Autobranchia;Heteroconchia;Euhe
terodonta;Imparidentia;Venerida;Veneroidea;Veneridae;Mercenaria;Mercenari
a mercenaria
UdeM00000007892 6596 721.8 UdeM00000007892 XP_045182724.1 54.4 1578
690 13 546 2120 1 1551 3.00E-204 721.8 6596 cellular
organisms;Eukaryota;Opisthokonta;Metazoa;Eumetazoa;Bilateria;Protostomia;
Spiralia;Lophotrochozoa;Mollusca;Bivalvia;Autobranchia;Heteroconchia;Euhe
terodonta;Imparidentia;Venerida;Veneroidea;Veneridae;Mercenaria;Mercenari
a mercenaria
UdeM00000007893 6596 1094.7 UdeM00000007893 XP_045182791.1 69.6
853 239 6 7 851 32 872 0.00E+00 1094.7 6596
cellular
organisms;Eukaryota;Opisthokonta;Metazoa;Eumetazoa;Bilateria;Protostomia;
Spiralia;Lophotrochozoa;Mollusca;Bivalvia;Autobranchia;Heteroconchia;Euhe
terodonta;Imparidentia;Venerida;Veneroidea;Veneridae;Mercenaria;Mercenari
a mercenaria
UdeM00000007894 6579 243.4 UdeM00000007894 XP_033740907.1 68.7 163
51 0 9 171 11 173 2.10E-61 243.4 6579 cellular
organisms;Eukaryota;Opisthokonta;Metazoa;Eumetazoa;Bilateria;Protostomia;
Spiralia;Lophotrochozoa;Mollusca;Bivalvia;Autobranchia;Pteriomorphia;Pect
inida;Pectinoidea;Pectinidae;Pecten;Pecten maximus
UdeM00000007895 6596 332 UdeM00000007895 XP_045183083.1 77.2 202
46 0 5 206 2 203 5.30E-88 332 6596 cellular
organisms;Eukaryota;Opisthokonta;Metazoa;Eumetazoa;Bilateria;Protostomia;
Spiralia;Lophotrochozoa;Mollusca;Bivalvia;Autobranchia;Heteroconchia;Euhe
terodonta;Imparidentia;Venerida;Veneroidea;Veneridae;Mercenaria;Mercenari
a mercenaria
UdeM00000007896 6596 217.2 UdeM00000007896 XP_045168484.1 52.3 199
91 1 1 195 1 199 1.90E-53 217.2 6596 cellular
organisms;Eukaryota;Opisthokonta;Metazoa;Eumetazoa;Bilateria;Protostomia;
Spiralia;Lophotrochozoa;Mollusca;Bivalvia;Autobranchia;Heteroconchia;Euhe
terodonta;Imparidentia;Venerida;Veneroidea;Veneridae;Mercenaria;Mercenari
a mercenaria

UdeM00000007898 6596 225.3 UdeM00000007898 XP_045168484.1 55.8 199
84 1 1 195 1 199 6.60E-56 225.3 6596 cellular
organisms;Eukaryota;Opisthokonta;Metazoa;Eumetazoa;Bilateria;Protostomia;
Spiralia;Lophotrochozoa;Mollusca;Bivalvia;Autobranchia;Heteroconchia;Euhe
terodonta;Imparidentia;Venerida;Veneroidea;Veneridae;Mercenaria;Mercenari
a mercenaria
UdeM00000007899 6596 170.6 UdeM00000007899 XP_045168484.1 48.9 178
89 1 1 178 24 199 1.80E-39 170.6 6596 cellular
organisms;Eukaryota;Opisthokonta;Metazoa;Eumetazoa;Bilateria;Protostomia;
Spiralia;Lophotrochozoa;Mollusca;Bivalvia;Autobranchia;Heteroconchia;Euhe
terodonta;Imparidentia;Venerida;Veneroidea;Veneridae;Mercenaria;Mercenari
a mercenaria
UdeM00000007900 6596 184.9 UdeM00000007900 XP_045168484.1 46.7 195
102 1 4 198 7 199 1.00E-43 184.9 6596 cellular
organisms;Eukaryota;Opisthokonta;Metazoa;Eumetazoa;Bilateria;Protostomia;
Spiralia;Lophotrochozoa;Mollusca;Bivalvia;Autobranchia;Heteroconchia;Euhe
terodonta;Imparidentia;Venerida;Veneroidea;Veneridae;Mercenaria;Mercenari
a mercenaria
UdeM00000007901 6596 193.7 UdeM00000007901 XP_045168484.1 50.8 191
93 1 4 194 7 196 2.20E-46 193.7 6596 cellular
organisms;Eukaryota;Opisthokonta;Metazoa;Eumetazoa;Bilateria;Protostomia;
Spiralia;Lophotrochozoa;Mollusca;Bivalvia;Autobranchia;Heteroconchia;Euhe
terodonta;Imparidentia;Venerida;Veneroidea;Veneridae;Mercenaria;Mercenari
a mercenaria
UdeM00000007902 6596 216.9 UdeM00000007902 XP_045157162.1 55.6 196
80 4 1 195 1 190 2.40E-53 216.9 6596 cellular
organisms;Eukaryota;Opisthokonta;Metazoa;Eumetazoa;Bilateria;Protostomia;
Spiralia;Lophotrochozoa;Mollusca;Bivalvia;Autobranchia;Heteroconchia;Euhe
terodonta;Imparidentia;Venerida;Veneroidea;Veneridae;Mercenaria;Mercenari
a mercenaria
UdeM00000007903 6596 251.9 UdeM00000007903 XP_045168484.1 59.8 199
76 1 1 195 1 199 6.60E-64 251.9 6596 cellular
organisms;Eukaryota;Opisthokonta;Metazoa;Eumetazoa;Bilateria;Protostomia;
Spiralia;Lophotrochozoa;Mollusca;Bivalvia;Autobranchia;Heteroconchia;Euhe
terodonta;Imparidentia;Venerida;Veneroidea;Veneridae;Mercenaria;Mercenari
a mercenaria
UdeM00000007904 6596 196.4 UdeM00000007904 XP_045168484.1 61.9 147
56 0 17 163 53 199 2.80E-47 196.4 6596 cellular
organisms;Eukaryota;Opisthokonta;Metazoa;Eumetazoa;Bilateria;Protostomia;
Spiralia;Lophotrochozoa;Mollusca;Bivalvia;Autobranchia;Heteroconchia;Euhe
terodonta;Imparidentia;Venerida;Veneroidea;Veneridae;Mercenaria;Mercenari
a mercenaria
UdeM00000007905 6596 159.8 UdeM00000007905 XP_045168484.1 42.3 189
108 1 7 194 11 199 3.40E-36 159.8 6596 cellular
organisms;Eukaryota;Opisthokonta;Metazoa;Eumetazoa;Bilateria;Protostomia;
Spiralia;Lophotrochozoa;Mollusca;Bivalvia;Autobranchia;Heteroconchia;Euhe
terodonta;Imparidentia;Venerida;Veneroidea;Veneridae;Mercenaria;Mercenari
a mercenaria
UdeM00000007906 6596 218.8 UdeM00000007906 XP_045168484.1 54 202
86 2 1 198 1 199 6.40E-54 218.8 6596 cellular
organisms;Eukaryota;Opisthokonta;Metazoa;Eumetazoa;Bilateria;Protostomia;
Spiralia;Lophotrochozoa;Mollusca;Bivalvia;Autobranchia;Heteroconchia;Euhe
terodonta;Imparidentia;Venerida;Veneroidea;Veneridae;Mercenaria;Mercenari
a mercenaria

UdeM00000007907 6500 107.8 UdeM00000007907 XP_035825169.1 33.3 243
122 5 24 243 330 555 1.90E-20 107.8 6500 cellular
organisms;Eukaryota;Opisthokonta;Metazoa;Eumetazoa;Bilateria;Protostomia;
Spiralia;Lophotrochozoa;Mollusca;Gastropoda;Heterobranchia;Euthyneura;Tec
tipleura;Aplysiida;Aplysioidea;Aplysiidae;Aplysia;Aplysia californica
UdeM00000007908 6596 219.2 UdeM00000007908 XP_045157162.1 58.2 196
75 4 1 195 1 190 4.70E-54 219.2 6596 cellular
organisms;Eukaryota;Opisthokonta;Metazoa;Eumetazoa;Bilateria;Protostomia;
Spiralia;Lophotrochozoa;Mollusca;Bivalvia;Autobranchia;Heteroconchia;Euhe
terodonta;Imparidentia;Venerida;Veneroidea;Veneridae;Mercenaria;Mercenari
a mercenaria
UdeM00000007909 6596 220.7 UdeM00000007909 XP_045157162.1 58.7 196
74 4 1 195 1 190 1.60E-54 220.7 6596 cellular
organisms;Eukaryota;Opisthokonta;Metazoa;Eumetazoa;Bilateria;Protostomia;
Spiralia;Lophotrochozoa;Mollusca;Bivalvia;Autobranchia;Heteroconchia;Euhe
terodonta;Imparidentia;Venerida;Veneroidea;Veneridae;Mercenaria;Mercenari
a mercenaria
UdeM00000007910 6596 220.7 UdeM00000007910 XP_045157162.1 58.7 196
74 4 1 195 1 190 1.60E-54 220.7 6596 cellular
organisms;Eukaryota;Opisthokonta;Metazoa;Eumetazoa;Bilateria;Protostomia;
Spiralia;Lophotrochozoa;Mollusca;Bivalvia;Autobranchia;Heteroconchia;Euhe
terodonta;Imparidentia;Venerida;Veneroidea;Veneridae;Mercenaria;Mercenari
a mercenaria
UdeM00000007911 6596 220.7 UdeM00000007911 XP_045157162.1 58.7 196
74 4 1 195 1 190 1.60E-54 220.7 6596 cellular
organisms;Eukaryota;Opisthokonta;Metazoa;Eumetazoa;Bilateria;Protostomia;
Spiralia;Lophotrochozoa;Mollusca;Bivalvia;Autobranchia;Heteroconchia;Euhe
terodonta;Imparidentia;Venerida;Veneroidea;Veneridae;Mercenaria;Mercenari
a mercenaria
UdeM00000007912 6596 222.2 UdeM00000007912 XP_045157162.1 58.7 196
74 4 1 195 1 190 5.60E-55 222.2 6596 cellular
organisms;Eukaryota;Opisthokonta;Metazoa;Eumetazoa;Bilateria;Protostomia;
Spiralia;Lophotrochozoa;Mollusca;Bivalvia;Autobranchia;Heteroconchia;Euhe
terodonta;Imparidentia;Venerida;Veneroidea;Veneridae;Mercenaria;Mercenari
a mercenaria
UdeM00000007913 6596 220.3 UdeM00000007913 XP_045157162.1 58.2 196
75 4 1 195 1 190 2.10E-54 220.3 6596 cellular
organisms;Eukaryota;Opisthokonta;Metazoa;Eumetazoa;Bilateria;Protostomia;
Spiralia;Lophotrochozoa;Mollusca;Bivalvia;Autobranchia;Heteroconchia;Euhe
terodonta;Imparidentia;Venerida;Veneroidea;Veneridae;Mercenaria;Mercenari
a mercenaria
UdeM00000007914 6596 211.5 UdeM00000007914 XP_045168484.1 54.7 190
84 1 4 193 9 196 1.00E-51 211.5 6596 cellular
organisms;Eukaryota;Opisthokonta;Metazoa;Eumetazoa;Bilateria;Protostomia;
Spiralia;Lophotrochozoa;Mollusca;Bivalvia;Autobranchia;Heteroconchia;Euhe
terodonta;Imparidentia;Venerida;Veneroidea;Veneridae;Mercenaria;Mercenari
a mercenaria
UdeM00000007915 27687 111.3 UdeM00000007915 XP_028656852.1 41.5 135
76 3 4 136 316 449 9.80E-22 111.3 27687 cellular
organisms;Eukaryota;Opisthokonta;Metazoa;Eumetazoa;Bilateria;Deuterostomi
a;Chordata;Craniata;Vertebrata;Gnathostomata;Teleostomi;Euteleostomi;Acti
nopterygii;Cladistia;Polypteriformes;Polypteridae;Erpetoichthys;Erpetoich
thys calabaricus

UdeM00000007916 6596 176 UdeM00000007916 XP_045168484.1 51.7 176
82 2 1 176 24 196 4.30E-41 176 6596 cellular
organisms;Eukaryota;Opisthokonta;Metazoa;Eumetazoa;Bilateria;Protostomia;
Spiralia;Lophotrochozoa;Mollusca;Bivalvia;Autobranchia;Heteroconchia;Euhe
terodonta;Imparidentia;Venerida;Veneroidea;Veneridae;Mercenaria;Mercenari
a mercenaria
UdeM00000007918 6596 201.4 UdeM00000007918 XP_045168484.1 55.5 182
79 1 9 190 14 193 1.00E-48 201.4 6596 cellular
organisms;Eukaryota;Opisthokonta;Metazoa;Eumetazoa;Bilateria;Protostomia;
Spiralia;Lophotrochozoa;Mollusca;Bivalvia;Autobranchia;Heteroconchia;Euhe
terodonta;Imparidentia;Venerida;Veneroidea;Veneridae;Mercenaria;Mercenari
a mercenaria
UdeM00000007919 6596 199.1 UdeM00000007919 XP_045168484.1 55.7 183
78 2 9 191 14 193 5.00E-48 199.1 6596 cellular
organisms;Eukaryota;Opisthokonta;Metazoa;Eumetazoa;Bilateria;Protostomia;
Spiralia;Lophotrochozoa;Mollusca;Bivalvia;Autobranchia;Heteroconchia;Euhe
terodonta;Imparidentia;Venerida;Veneroidea;Veneridae;Mercenaria;Mercenari
a mercenaria
UdeM00000007920 6579 218.8 UdeM00000007920 XP_033734651.1 53.5 187
86 1 1 186 1 187 6.50E-54 218.8 6579 cellular
organisms;Eukaryota;Opisthokonta;Metazoa;Eumetazoa;Bilateria;Protostomia;
Spiralia;Lophotrochozoa;Mollusca;Bivalvia;Autobranchia;Pteriomorphia;Pect
inida;Pectinoidea;Pectinidae;Pecten;Pecten maximus
UdeM00000044298 400727 148.7 UdeM00000044298 XP_025095647.1 51.2
127 58 1 42 168 5 127 7.00E-33 148.7 400727
cellular
organisms;Eukaryota;Opisthokonta;Metazoa;Eumetazoa;Bilateria;Protostomia;
Spiralia;Lophotrochozoa;Mollusca;Gastropoda;Caenogastropoda;Architaeniogl
ossa;Ampullarioidea;Ampullariidae;Pomacea;Pomacea canaliculata
UdeM00000044299 400727 108.6 UdeM00000044299 XP_025093452.1 65.3
72 25 0 30 101 135 206 4.70E-21 108.6 400727
cellular
organisms;Eukaryota;Opisthokonta;Metazoa;Eumetazoa;Bilateria;Protostomia;
Spiralia;Lophotrochozoa;Mollusca;Gastropoda;Caenogastropoda;Architaeniogl
ossa;Ampullarioidea;Ampullariidae;Pomacea;Pomacea canaliculata
UdeM00000044300 6565 600.9 UdeM00000044300 XP_022287481.1 50.5 622
295 8 2 620 1522 2133 1.80E-168 600.9 6565 cellular
organisms;Eukaryota;Opisthokonta;Metazoa;Eumetazoa;Bilateria;Protostomia;
Spiralia;Lophotrochozoa;Mollusca;Bivalvia;Autobranchia;Pteriomorphia;Ostr
eida;Ostreoidea;Ostreidae;Crassostrea;Crassostrea virginica
UdeM00000044301 6596 114.8 UdeM00000044301 XP_045210599.1 43.7 151
70 3 87 224 160 308 1.50E-22 114.8 6596 cellular
organisms;Eukaryota;Opisthokonta;Metazoa;Eumetazoa;Bilateria;Protostomia;
Spiralia;Lophotrochozoa;Mollusca;Bivalvia;Autobranchia;Heteroconchia;Euhe
terodonta;Imparidentia;Venerida;Veneroidea;Veneridae;Mercenaria;Mercenari
a mercenaria
UdeM00000044302 7654 74.7 UdeM00000044302 XP_041473734.1 42.4 92
49 2 8 97 652 741 7.20E-11 74.7 7654 cellular
organisms;Eukaryota;Opisthokonta;Metazoa;Eumetazoa;Bilateria;Deuterostomi
a;Echinodermata;Eleutherozoa;Echinozoa;Echinoidea;Euechinoidea;Echinacea;
Temnopleuroida;Toxopneustidae;Lytechinus;Lytechinus variegatus
UdeM00000044352 7741 58.5 UdeM00000044352 XP_019641549.1 32.8 119
75 4 10 127 57 171 7.10E-06 58.5 7741 cellular
organisms;Eukaryota;Opisthokonta;Metazoa;Eumetazoa;Bilateria;Deuterostomi

a;Chordata;Cephalochordata;Leptocardii;Amphioxiformes;Branchiostomidae;Branchiostoma;Branchiostoma belcheri
UdeM00000044351 45264 67.4 UdeM00000044351 XP_044168285.1 25.4 169 121 3 44 212 396 559 2.50E-08 67.4 45264 cellular organisms;Eukaryota;Opisthokonta;Metazoa;Eumetazoa;Cnidaria;Anthozoa;Hexacorallia;Scleractinia;Astrocoeniina;Acroporidae;Acropora;Acropora millepora
UdeM00000001818 6565 105.1 UdeM00000001818 XP_022312780.1 63.5 74 27 0 1 74 5 78 4.00E-20 105.1 6565 cellular organisms;Eukaryota;Opisthokonta;Metazoa;Eumetazoa;Bilateria;Protostomia;Spiralia;Lophotrochozoa;Mollusca;Bivalvia;Autobranchia;Pteriomorphia;Ostreida;Ostreoidea;Ostreidae;Crassostrea;Crassostrea virginica
UdeM00000001819 29159 474.2 UdeM00000001819 XP_034303860.1 40 675 263 15 42 590 35 693 2.50E-130 474.2 29159 cellular organisms;Eukaryota;Opisthokonta;Metazoa;Eumetazoa;Bilateria;Protostomia;Spiralia;Lophotrochozoa;Mollusca;Bivalvia;Autobranchia;Pteriomorphia;Ostreida;Ostreoidea;Ostreidae;Crassostrea;Crassostrea gigas
UdeM00000001820 6565 103.2 UdeM00000001820 XP_022312780.1 62.2 74 28 0 1 74 5 78 1.50E-19 103.2 6565 cellular organisms;Eukaryota;Opisthokonta;Metazoa;Eumetazoa;Bilateria;Protostomia;Spiralia;Lophotrochozoa;Mollusca;Bivalvia;Autobranchia;Pteriomorphia;Ostreida;Ostreoidea;Ostreidae;Crassostrea;Crassostrea virginica
UdeM00000001821 29159 93.2 UdeM00000001821 XP_034310427.1 58 69 29 0 1 69 602 670 1.40E-16 93.2 29159 cellular organisms;Eukaryota;Opisthokonta;Metazoa;Eumetazoa;Bilateria;Protostomia;Spiralia;Lophotrochozoa;Mollusca;Bivalvia;Autobranchia;Pteriomorphia;Ostreida;Ostreoidea;Ostreidae;Crassostrea;Crassostrea gigas
UdeM00000001822 29159 338.2 UdeM00000001822 XP_034303860.1 34.4 596 233 13 27 480 29 608 1.70E-89 338.2 29159 cellular organisms;Eukaryota;Opisthokonta;Metazoa;Eumetazoa;Bilateria;Protostomia;Spiralia;Lophotrochozoa;Mollusca;Bivalvia;Autobranchia;Pteriomorphia;Ostreida;Ostreoidea;Ostreidae;Crassostrea;Crassostrea gigas
UdeM00000001823 29159 188.7 UdeM00000001823 XP_034303860.1 29.3 416 92 5 9 222 278 693 7.90E-45 188.7 29159 cellular organisms;Eukaryota;Opisthokonta;Metazoa;Eumetazoa;Bilateria;Protostomia;Spiralia;Lophotrochozoa;Mollusca;Bivalvia;Autobranchia;Pteriomorphia;Ostreida;Ostreoidea;Ostreidae;Crassostrea;Crassostrea gigas
UdeM00000001824 29159 110.9 UdeM00000001824 XP_034303860.1 39.2 189 98 7 79 262 32 208 2.50E-21 110.9 29159 cellular organisms;Eukaryota;Opisthokonta;Metazoa;Eumetazoa;Bilateria;Protostomia;Spiralia;Lophotrochozoa;Mollusca;Bivalvia;Autobranchia;Pteriomorphia;Ostreida;Ostreoidea;Ostreidae;Crassostrea;Crassostrea gigas
UdeM00000001825 6579 357.5 UdeM00000001825 XP_033752673.1 54.5 323 108 1 28 311 328 650 1.80E-95 357.5 6579 cellular organisms;Eukaryota;Opisthokonta;Metazoa;Eumetazoa;Bilateria;Protostomia;Spiralia;Lophotrochozoa;Mollusca;Bivalvia;Autobranchia;Pteriomorphia;Pectinida;Pectinoidea;Pectinidae;Pecten;Pecten maximus
UdeM00000001826 6500 188 UdeM00000001826 XP_012938994.2 46.3 188 101 0 34 221 69 256 1.40E-44 188 6500 cellular organisms;Eukaryota;Opisthokonta;Metazoa;Eumetazoa;Bilateria;Protostomia;Spiralia;Lophotrochozoa;Mollusca;Gastropoda;Heterobranchia;Euthyneura;Tectipleura;Aplysiida;Aplysioidea;Aplysiidae;Aplysia;Aplysia californica
UdeM00000001827 6596 424.1 UdeM00000001827 XP_045196634.1 54.8 343 153 1 13 355 7 347 1.80E-115 424.1 6596 cellular

organisms;Eukaryota;Opisthokonta;Metazoa;Eumetazoa;Bilateria;Protostomia;Spiralia;Lophotrochozoa;Mollusca;Bivalvia;Autobranchia;Heteroconchia;Euheterodonta;Imparidentia;Venerida;Veneroidea;Veneridae;Mercenaria;Mercenaria mercenaria
UdeM00000001829 6579 586.3 UdeM00000001829 XP_033741429.1 58.6 514 190 5 1 510 1 495 4.00E-164 586.3 6579 cellular organisms;Eukaryota;Opisthokonta;Metazoa;Eumetazoa;Bilateria;Protostomia;Spiralia;Lophotrochozoa;Mollusca;Bivalvia;Autobranchia;Pteriomorphia;Pectinida;Pectinoidea;Pectinidae;Pecten;Pecten maximus
UdeM00000001830 400727 97.4 UdeM00000001830 XP_025097146.1 61.8 68 26 0 1 68 1 68 8.00E-18 97.4 400727 cellular organisms;Eukaryota;Opisthokonta;Metazoa;Eumetazoa;Bilateria;Protostomia;Spiralia;Lophotrochozoa;Mollusca;Gastropoda;Caenogastropoda;Architaenioglossa;Ampullarioidea;Ampullariidae;Pomacea;Pomacea canaliculata
UdeM00000001831 6596 695.3 UdeM00000001831 XP_045196582.1 33.9 2077 1063 47 84 2108 62 1881 2.40E-196 695.3 6596 cellular organisms;Eukaryota;Opisthokonta;Metazoa;Eumetazoa;Bilateria;Protostomia;Spiralia;Lophotrochozoa;Mollusca;Bivalvia;Autobranchia;Heteroconchia;Euheterodonta;Imparidentia;Venerida;Veneroidea;Veneridae;Mercenaria;Mercenaria mercenaria
UdeM00000001833 6579 90.1 UdeM00000001833 XP_033758230.1 69.5 59 18 0 23 81 582 640 1.40E-15 90.1 6579 cellular organisms;Eukaryota;Opisthokonta;Metazoa;Eumetazoa;Bilateria;Protostomia;Spiralia;Lophotrochozoa;Mollusca;Bivalvia;Autobranchia;Pteriomorphia;Pectinida;Pectinoidea;Pectinidae;Pecten;Pecten maximus
UdeM00000001834 6579 183 UdeM00000001834 XP_033759640.1 47.9 190 92 2 22 204 117 306 3.90E-43 183 6579 cellular organisms;Eukaryota;Opisthokonta;Metazoa;Eumetazoa;Bilateria;Protostomia;Spiralia;Lophotrochozoa;Mollusca;Bivalvia;Autobranchia;Pteriomorphia;Pectinida;Pectinoidea;Pectinidae;Pecten;Pecten maximus
UdeM00000001835 6573 138.3 UdeM00000001835 XP_021362776.1 37.1 202 97 3 33 234 35 206 1.30E-29 138.3 6573 cellular organisms;Eukaryota;Opisthokonta;Metazoa;Eumetazoa;Bilateria;Protostomia;Spiralia;Lophotrochozoa;Mollusca;Bivalvia;Autobranchia;Pteriomorphia;Pectinida;Pectinoidea;Pectinidae;Mizuhopecten;Mizuhopecten yessoensis
UdeM00000001836 6596 361.7 UdeM00000001836 XP_045196737.1 48.3 474 185 18 1 422 1 466 1.30E-96 361.7 6596 cellular organisms;Eukaryota;Opisthokonta;Metazoa;Eumetazoa;Bilateria;Protostomia;Spiralia;Lophotrochozoa;Mollusca;Bivalvia;Autobranchia;Heteroconchia;Euheterodonta;Imparidentia;Venerida;Veneroidea;Veneridae;Mercenaria;Mercenaria mercenaria
UdeM00000001837 6565 869 UdeM00000001837 XP_022292411.1 72.4 566 134 4 2 567 695 1238 3.30E-249 869 6565 cellular organisms;Eukaryota;Opisthokonta;Metazoa;Eumetazoa;Bilateria;Protostomia;Spiralia;Lophotrochozoa;Mollusca;Bivalvia;Autobranchia;Pteriomorphia;Ostreida;Ostreoidea;Ostreidae;Crassostrea;Crassostrea virginica
UdeM00000001838 29159 1038.5 UdeM00000001838 XP_011455128.2 79.6 641 128 3 35 674 80 718 3.70E-300 1038.5 29159 cellular organisms;Eukaryota;Opisthokonta;Metazoa;Eumetazoa;Bilateria;Protostomia;Spiralia;Lophotrochozoa;Mollusca;Bivalvia;Autobranchia;Pteriomorphia;Ostreida;Ostreoidea;Ostreidae;Crassostrea;Crassostrea gigas

UdeM00000001839	2607531	127.1	UdeM00000001839	XP_029646202.1	83.3	72	12	0	15	86	57	128	1.50E-26	127.1	2607531	cellular organisms;Eukaryota;Opisthokonta;Metazoa;Eumetazoa;Bilateria;Protostomia;Spiralia;Lophotrochozoa;Mollusca;Cephalopoda;Coleoidea;Octopodiformes;Octopoda;Incirrata;Octopodidae;Octopus;Octopus sinensis
UdeM00000001840	6596	151.4	UdeM00000001840	XP_045199821.1	54.9	133	58	2	10	141	11	142	8.80E-34	151.4	6596	cellular organisms;Eukaryota;Opisthokonta;Metazoa;Eumetazoa;Bilateria;Protostomia;Spiralia;Lophotrochozoa;Mollusca;Bivalvia;Autobranchia;Heteroconchia;Euheterodonta;Imparidentia;Venerida;Veneroidea;Veneridae;Mercenaria;Mercenaria mercenaria
UdeM00000001841	6596	135.6	UdeM00000001841	XP_045199821.1	44.4	126	70	0	41	166	16	141	6.00E-29	135.6	6596	cellular organisms;Eukaryota;Opisthokonta;Metazoa;Eumetazoa;Bilateria;Protostomia;Spiralia;Lophotrochozoa;Mollusca;Bivalvia;Autobranchia;Heteroconchia;Euheterodonta;Imparidentia;Venerida;Veneroidea;Veneridae;Mercenaria;Mercenaria mercenaria
UdeM00000001842	6596	314.3	UdeM00000001842	XP_045196365.1	52.6	308	146	0	13	320	157	464	1.80E-82	314.3	6596	cellular organisms;Eukaryota;Opisthokonta;Metazoa;Eumetazoa;Bilateria;Protostomia;Spiralia;Lophotrochozoa;Mollusca;Bivalvia;Autobranchia;Heteroconchia;Euheterodonta;Imparidentia;Venerida;Veneroidea;Veneridae;Mercenaria;Mercenaria mercenaria
UdeM00000001843	114398	63.5	UdeM00000001843	XP_015918496.1	49.2	65	32	1	32	96	15	78	3.60E-06	63.5	114398	cellular organisms;Eukaryota;Opisthokonta;Metazoa;Eumetazoa;Bilateria;Protostomia;Ecdysozoa;Panarthropoda;Arthropoda;Chelicerata;Arachnida;Araneae;Araneomorphae;Entelegynae;Orbiculariae;Araneoidea;Theridiidae;Parasteatoda;Parasteatoda tepidariorum
UdeM00000001844	1735272	491.9	UdeM00000001844	XP_041354220.1	67.5	314	102	0	1	314	1	314	6.10E-136	491.9	1735272	cellular organisms;Eukaryota;Opisthokonta;Metazoa;Eumetazoa;Bilateria;Protostomia;Spiralia;Lophotrochozoa;Mollusca;Gastropoda;Neomphalina;Neomphaloidea;Peltospiridae;Gigantopelta;Gigantopelta aegis
UdeM00000001845	6596	583.9	UdeM00000001845	XP_045196481.1	45	671	358	6	17	682	6	670	2.60E-163	583.9	6596	cellular organisms;Eukaryota;Opisthokonta;Metazoa;Eumetazoa;Bilateria;Protostomia;Spiralia;Lophotrochozoa;Mollusca;Bivalvia;Autobranchia;Heteroconchia;Euheterodonta;Imparidentia;Venerida;Veneroidea;Veneridae;Mercenaria;Mercenaria mercenaria
UdeM00000001846	6596	325.5	UdeM00000001846	XP_045195385.1	54.1	331	138	5	4	324	133	459	7.80E-86	325.5	6596	cellular organisms;Eukaryota;Opisthokonta;Metazoa;Eumetazoa;Bilateria;Protostomia;Spiralia;Lophotrochozoa;Mollusca;Bivalvia;Autobranchia;Heteroconchia;Euheterodonta;Imparidentia;Venerida;Veneroidea;Veneridae;Mercenaria;Mercenaria mercenaria
UdeM00000001847	6596	527.3	UdeM00000001847	XP_045198506.1	35.7	990	502	26	34	932	1783	2728	3.90E-146	527.3	6596	cellular organisms;Eukaryota;Opisthokonta;Metazoa;Eumetazoa;Bilateria;Protostomia;Spiralia;Lophotrochozoa;Mollusca;Bivalvia;Autobranchia;Heteroconchia;Euhe

terodonta;Imparidentia;Venerida;Veneroidea;Veneridae;Mercenaria;Mercenaria mercenaria
UdeM00000001849 6596 240.4 UdeM00000001849 XP_045198695.1 34.9 499 172 5 5 354 2452 2946 3.60E-60 240.4 6596 cellular organisms;Eukaryota;Opisthokonta;Metazoa;Eumetazoa;Bilateria;Protostomia;Spiralia;Lophotrochozoa;Mollusca;Bivalvia;Autobranchia;Heteroconchia;Euheterodonta;Imparidentia;Venerida;Veneroidea;Veneridae;Mercenaria;Mercenaria mercenaria
UdeM00000001850 6596 602.8 UdeM00000001850 XP_045197975.1 36 1107 570 24 281 1273 907 1989 1.10E-168 602.8 6596 cellular organisms;Eukaryota;Opisthokonta;Metazoa;Eumetazoa;Bilateria;Protostomia;Spiralia;Lophotrochozoa;Mollusca;Bivalvia;Autobranchia;Heteroconchia;Euheterodonta;Imparidentia;Venerida;Veneroidea;Veneridae;Mercenaria;Mercenaria mercenaria
UdeM00000001851 6596 441 UdeM00000001851 XP_045198506.1 39.1 662 370 14 3 640 2043 2695 2.60E-120 441 6596 cellular organisms;Eukaryota;Opisthokonta;Metazoa;Eumetazoa;Bilateria;Protostomia;Spiralia;Lophotrochozoa;Mollusca;Bivalvia;Autobranchia;Heteroconchia;Euheterodonta;Imparidentia;Venerida;Veneroidea;Veneridae;Mercenaria;Mercenaria mercenaria
UdeM00000001852 6596 495.7 UdeM00000001852 XP_045198506.1 38.6 761 432 15 34 770 1946 2695 1.10E-136 495.7 6596 cellular organisms;Eukaryota;Opisthokonta;Metazoa;Eumetazoa;Bilateria;Protostomia;Spiralia;Lophotrochozoa;Mollusca;Bivalvia;Autobranchia;Heteroconchia;Euheterodonta;Imparidentia;Venerida;Veneroidea;Veneridae;Mercenaria;Mercenaria mercenaria
UdeM00000001853 6596 86.7 UdeM00000001853 XP_045198505.1 25.7 331 203 13 56 367 219 525 8.10E-14 86.7 6596 cellular organisms;Eukaryota;Opisthokonta;Metazoa;Eumetazoa;Bilateria;Protostomia;Spiralia;Lophotrochozoa;Mollusca;Bivalvia;Autobranchia;Heteroconchia;Euheterodonta;Imparidentia;Venerida;Veneroidea;Veneridae;Mercenaria;Mercenaria mercenaria
UdeM00000001854 6596 575.5 UdeM00000001854 XP_045198506.1 27.4 1962 1110 60 157 1928 892 2728 2.60E-160 575.5 6596 cellular organisms;Eukaryota;Opisthokonta;Metazoa;Eumetazoa;Bilateria;Protostomia;Spiralia;Lophotrochozoa;Mollusca;Bivalvia;Autobranchia;Heteroconchia;Euheterodonta;Imparidentia;Venerida;Veneroidea;Veneridae;Mercenaria;Mercenaria mercenaria
UdeM00000001855 6596 632.1 UdeM00000001855 XP_045198506.1 40.7 923 464 20 194 1083 1856 2728 1.30E-177 632.1 6596 cellular organisms;Eukaryota;Opisthokonta;Metazoa;Eumetazoa;Bilateria;Protostomia;Spiralia;Lophotrochozoa;Mollusca;Bivalvia;Autobranchia;Heteroconchia;Euheterodonta;Imparidentia;Venerida;Veneroidea;Veneridae;Mercenaria;Mercenaria mercenaria
UdeM00000001856 390379 224.9 UdeM00000001856 XP_034020940.1 24.7 1006 628 37 75 1036 3319 4238 4.70E-55 224.9 390379 cellular organisms;Eukaryota;Opisthokonta;Metazoa;Eumetazoa;Bilateria;Deuterostomia;Chordata;Craniata;Vertebrata;Gnathostomata;Teleostomi;Euteleostomi;Actinopterygii;Actinopteri;Neopterygii;Teleostei;Osteoglossocephalai;Clupeocephala;Euteleosteomorpha;Neoteleostei;Eurypterygia;Ctenosquamata;Acanthomorphata;Euacanthomorphacea;Percomorphaceae;Batrachoidaria;Batrachoidiformes;Batrachoididae;Thalassophyrininae;Thalassophryne;Thalassophryne amazonica

UdeM00000001858 260615 80.1 UdeM00000001858 XP_039385216.1 34.4 180 8 2 1 70 347 526 1.20E-12 80.1 260615 cellular organisms;Eukaryota;Opisthokonta;Metazoa;Eumetazoa;Bilateria;Deuterostomia;Chordata;Craniata;Vertebrata;Gnathostomata;Teleostomi;Euteleostomi;Sarcopterygii;Dipnotetrapodomorpha;Tetrapoda;Amniota;Sauropsida;Sauria;Archelosauria;Testudinata;Testudines;Cryptodira;Durocryptodira;Testudinoidea;Geoemydidae;Geoemydinae;Mauremys;Mauremys reevesii
UdeM00000001859 260615 205.7 UdeM00000001859 XP_039386738.1 70.6 180 9 3 1 136 347 526 3.80E-50 205.7 260615 cellular organisms;Eukaryota;Opisthokonta;Metazoa;Eumetazoa;Bilateria;Deuterostomia;Chordata;Craniata;Vertebrata;Gnathostomata;Teleostomi;Euteleostomi;Sarcopterygii;Dipnotetrapodomorpha;Tetrapoda;Amniota;Sauropsida;Sauria;Archelosauria;Testudinata;Testudines;Cryptodira;Durocryptodira;Testudinoidea;Geoemydidae;Geoemydinae;Mauremys;Mauremys reevesii
UdeM00000001860 29159 57.4 UdeM00000001860 XP_034315725.1 55.3 47 18 1 1 47 502 545 6.60E-06 57.4 29159 cellular organisms;Eukaryota;Opisthokonta;Metazoa;Eumetazoa;Bilateria;Protostomia;Spiralia;Lophotrochozoa;Mollusca;Bivalvia;Autobranchia;Pteriomorphia;Ostreida;Ostreoidea;Ostreidae;Crassostrea;Crassostrea gigas
UdeM00000001861 29159 134 UdeM00000001861 XP_034315725.1 55.6 117 50 1 1 117 559 673 1.30E-28 134 29159 cellular organisms;Eukaryota;Opisthokonta;Metazoa;Eumetazoa;Bilateria;Protostomia;Spiralia;Lophotrochozoa;Mollusca;Bivalvia;Autobranchia;Pteriomorphia;Ostreida;Ostreoidea;Ostreidae;Crassostrea;Crassostrea gigas
UdeM00000001866 6565 286.6 UdeM00000001866 XP_022286598.1 46.1 360 170 7 2 361 135 470 4.70E-74 286.6 6565 cellular organisms;Eukaryota;Opisthokonta;Metazoa;Eumetazoa;Bilateria;Protostomia;Spiralia;Lophotrochozoa;Mollusca;Bivalvia;Autobranchia;Pteriomorphia;Ostreida;Ostreoidea;Ostreidae;Crassostrea;Crassostrea virginica
UdeM00000001867 6565 247.7 UdeM00000001867 XP_022320668.1 68.8 160 50 0 1 160 527 686 1.00E-62 247.7 6565 cellular organisms;Eukaryota;Opisthokonta;Metazoa;Eumetazoa;Bilateria;Protostomia;Spiralia;Lophotrochozoa;Mollusca;Bivalvia;Autobranchia;Pteriomorphia;Ostreida;Ostreoidea;Ostreidae;Crassostrea;Crassostrea virginica
UdeM00000001870 6596 197.6 UdeM00000001870 XP_045195979.1 36.4 316 181 5 88 403 282 577 3.40E-47 197.6 6596 cellular organisms;Eukaryota;Opisthokonta;Metazoa;Eumetazoa;Bilateria;Protostomia;Spiralia;Lophotrochozoa;Mollusca;Bivalvia;Autobranchia;Heteroconchia;Euheterodonta;Imparidentia;Venerida;Veneroidea;Veneridae;Mercenaria;Mercenaria mercenaria
UdeM00000001875 6596 302 UdeM00000001875 XP_045196404.1 37.9 467 238 10 256 707 360 789 2.00E-78 302 6596 cellular organisms;Eukaryota;Opisthokonta;Metazoa;Eumetazoa;Bilateria;Protostomia;Spiralia;Lophotrochozoa;Mollusca;Bivalvia;Autobranchia;Heteroconchia;Euheterodonta;Imparidentia;Venerida;Veneroidea;Veneridae;Mercenaria;Mercenaria mercenaria
UdeM00000001878 6579 701 UdeM00000001878 XP_033728194.1 50.9 693 327 5 1 684 282 970 1.50E-198 701 6579 cellular organisms;Eukaryota;Opisthokonta;Metazoa;Eumetazoa;Bilateria;Protostomia;Spiralia;Lophotrochozoa;Mollusca;Bivalvia;Autobranchia;Pteriomorphia;Pectinida;Pectinoidea;Pectinidae;Pecten;Pecten maximus

UdeM00000001880 29159 219.9 UdeM00000001880 XP_034339165.1 38.3 334
192 8 1 331 186 508 4.70E-54 219.9 29159 cellular
organisms;Eukaryota;Opisthokonta;Metazoa;Eumetazoa;Bilateria;Protostomia;
Spiralia;Lophotrochozoa;Mollusca;Bivalvia;Autobranchia;Pteriomorphia;Ostr
eida;Ostreoidea;Ostreidae;Crassostrea;Crassostrea gigas
UdeM00000001881 29159 496.5 UdeM00000001881 XP_034299474.1 43.4 611
322 8 66 665 158 755 5.20E-137 496.5 29159 cellular
organisms;Eukaryota;Opisthokonta;Metazoa;Eumetazoa;Bilateria;Protostomia;
Spiralia;Lophotrochozoa;Mollusca;Bivalvia;Autobranchia;Pteriomorphia;Ostr
eida;Ostreoidea;Ostreidae;Crassostrea;Crassostrea gigas
UdeM00000001884 29159 424.5 UdeM00000001884 XP_034299474.1 44.1 526
279 7 3 522 239 755 2.00E-115 424.5 29159 cellular
organisms;Eukaryota;Opisthokonta;Metazoa;Eumetazoa;Bilateria;Protostomia;
Spiralia;Lophotrochozoa;Mollusca;Bivalvia;Autobranchia;Pteriomorphia;Ostr
eida;Ostreoidea;Ostreidae;Crassostrea;Crassostrea gigas
UdeM00000001885 29159 317 UdeM00000001885 XP_034330911.1 39.3 489
265 12 31 505 13 483 4.40E-83 317 29159 cellular
organisms;Eukaryota;Opisthokonta;Metazoa;Eumetazoa;Bilateria;Protostomia;
Spiralia;Lophotrochozoa;Mollusca;Bivalvia;Autobranchia;Pteriomorphia;Ostr
eida;Ostreoidea;Ostreidae;Crassostrea;Crassostrea gigas
UdeM00000001886 46731 101.7 UdeM00000001886 XP_027046473.1 43.8 112
63 0 74 185 19 130 1.10E-18 101.7 46731 cellular
organisms;Eukaryota;Opisthokonta;Metazoa;Eumetazoa;Cnidaria;Anthozoa;Hexa
corallia;Scleractinia;Astrocoeniina;Pocilloporidae;Pocillopora;Pocillopor
a damicornis
UdeM00000001890 6596 537 UdeM00000001890 XP_045178231.1 50.6 559
267 5 7 557 9 566 3.00E-149 537 6596 cellular
organisms;Eukaryota;Opisthokonta;Metazoa;Eumetazoa;Bilateria;Protostomia;
Spiralia;Lophotrochozoa;Mollusca;Bivalvia;Autobranchia;Heteroconchia;Euhe
terodonta;Imparidentia;Venerida;Veneroidea;Veneridae;Mercenaria;Mercenari
a mercenaria
UdeM00000001891 6596 125.9 UdeM00000001891 XP_045180427.1 33.6 274
127 4 1 220 1 273 6.60E-26 125.9 6596 cellular
organisms;Eukaryota;Opisthokonta;Metazoa;Eumetazoa;Bilateria;Protostomia;
Spiralia;Lophotrochozoa;Mollusca;Bivalvia;Autobranchia;Heteroconchia;Euhe
terodonta;Imparidentia;Venerida;Veneroidea;Veneridae;Mercenaria;Mercenari
a mercenaria
UdeM00000001892 6596 1186 UdeM00000001892 XP_045178231.1 64.1 957
329 5 1 952 602 1548 0.00E+00 1186 6596 cellular
organisms;Eukaryota;Opisthokonta;Metazoa;Eumetazoa;Bilateria;Protostomia;
Spiralia;Lophotrochozoa;Mollusca;Bivalvia;Autobranchia;Heteroconchia;Euhe
terodonta;Imparidentia;Venerida;Veneroidea;Veneridae;Mercenaria;Mercenari
a mercenaria
UdeM00000001894 6596 177.6 UdeM00000001894 XP_045195643.1 41.1 248
119 10 3 237 66 299 2.30E-41 177.6 6596 cellular
organisms;Eukaryota;Opisthokonta;Metazoa;Eumetazoa;Bilateria;Protostomia;
Spiralia;Lophotrochozoa;Mollusca;Bivalvia;Autobranchia;Heteroconchia;Euhe
terodonta;Imparidentia;Venerida;Veneroidea;Veneridae;Mercenaria;Mercenari
a mercenaria
UdeM00000001895 6579 345.1 UdeM00000001895 XP_033740923.1 36.6 558
314 15 5 552 7 534 1.60E-91 345.1 6579 cellular
organisms;Eukaryota;Opisthokonta;Metazoa;Eumetazoa;Bilateria;Protostomia;
Spiralia;Lophotrochozoa;Mollusca;Bivalvia;Autobranchia;Pteriomorphia;Pect
inida;Pectinoidea;Pectinidae;Pecten;Pecten maximus

UdeM00000001896 225164 407.5 UdeM00000001896 XP_009064346.1 41.6 538 272 12 21 544 16 525 2.60E-110 407.5 225164 cellular organisms;Eukaryota;Opisthokonta;Metazoa;Eumetazoa;Bilateria;Protostomia;Spiralia;Lophotrochozoa;Mollusca;Gastropoda;Patellogastropoda;Lottioidea;Lottiidae;Lottia;Lottia gigantea
UdeM00000001897 29159 433 UdeM00000001897 XP_011420306.2 83.9 261 38 2 12 272 1 257 2.90E-118 433 29159 cellular organisms;Eukaryota;Opisthokonta;Metazoa;Eumetazoa;Bilateria;Protostomia;Spiralia;Lophotrochozoa;Mollusca;Bivalvia;Autobranchia;Pteriomorphia;Ostreida;Ostreoidea;Ostreidae;Crassostrea;Crassostrea gigas
UdeM00000001898 6596 300.8 UdeM00000001898 XP_045195631.1 51 298 138 4 55 348 41 334 2.50E-78 300.8 6596 cellular organisms;Eukaryota;Opisthokonta;Metazoa;Eumetazoa;Bilateria;Protostomia;Spiralia;Lophotrochozoa;Mollusca;Bivalvia;Autobranchia;Heteroconchia;Euheterodonta;Imparidentia;Venerida;Veneroidea;Veneridae;Mercenaria;Mercenaria mercenaria
UdeM00000001899 8128 67 UdeM00000001899 XP_019206542.2 32 125 75 3 85 204 28 147 3.20E-08 67 8128 cellular organisms;Eukaryota;Opisthokonta;Metazoa;Eumetazoa;Bilateria;Deuterostomia;Chordata;Craniata;Vertebrata;Gnathostomata;Teleostomi;Euteleostomi;Actinopterygii;Actinopteri;Neopterygii;Teleostei;Osteoglossocephalai;Clupeocephala;Euteleosteomorpha;Neoteleostei;Eurypterygia;Ctenosquamata;Acanthomorphata;Euacanthomorphacea;Percomorphaceae;Ovalentaria;Cichlomorphae;Cichliformes;Cichlidae;African cichlids;Pseudocrenilabrinae;Oreochromini;Oreochromis;Oreochromis niloticus
UdeM00000001900 7906 243.8 UdeM00000001900 XP_034780548.1 63.6 184 67 0 2 185 193 376 1.70E-61 243.8 7906 cellular organisms;Eukaryota;Opisthokonta;Metazoa;Eumetazoa;Bilateria;Deuterostomia;Chordata;Craniata;Vertebrata;Gnathostomata;Teleostomi;Euteleostomi;Actinopterygii;Actinopteri;Chondrostei;Acipenseriformes;Acipenseroidei;Acipenseridae;Acipenserinae;Acipenserini;Acipenser;Acipenser ruthenus
UdeM00000001901 50429 75.5 UdeM00000001901 XP_022807755.1 44.9 89 47 1 2 90 89 175 4.20E-11 75.5 50429 cellular organisms;Eukaryota;Opisthokonta;Metazoa;Eumetazoa;Cnidaria;Anthozoa;Hexacorallia;Scleractinia;Astrocoeniina;Pocilloporidae;Stylophora;Stylophora pistillata
UdeM00000001902 481459 97.4 UdeM00000001902 XP_040031014.1 41.7 103 58 1 1 103 311 411 1.10E-17 97.4 481459 cellular organisms;Eukaryota;Opisthokonta;Metazoa;Eumetazoa;Bilateria;Deuterostomia;Chordata;Craniata;Vertebrata;Gnathostomata;Teleostomi;Euteleostomi;Actinopterygii;Actinopteri;Neopterygii;Teleostei;Osteoglossocephalai;Clupeocephala;Euteleosteomorpha;Neoteleostei;Eurypterygia;Ctenosquamata;Acanthomorphata;Euacanthomorphacea;Percomorphaceae;Eupercaria;Perciformes;Cottioidei;Gasterosteales;Gasterosteidae;Gasterosteus;Gasterosteus aculeatus;Gasterosteus aculeatus aculeatus
UdeM00000001903 29159 171 UdeM00000001903 XP_034335595.1 37.8 286 125 6 52 284 294 579 2.20E-39 171 29159 cellular organisms;Eukaryota;Opisthokonta;Metazoa;Eumetazoa;Bilateria;Protostomia;Spiralia;Lophotrochozoa;Mollusca;Bivalvia;Autobranchia;Pteriomorphia;Ostreida;Ostreoidea;Ostreidae;Crassostrea;Crassostrea gigas

UdeM00000001905 6565 492.7 UdeM00000001905 XP_022297037.1 50 534
238 6 6 532 11 522 6.10E-136 492.7 6565 cellular
organisms;Eukaryota;Opisthokonta;Metazoa;Eumetazoa;Bilateria;Protostomia;
Spiralia;Lophotrochozoa;Mollusca;Bivalvia;Autobranchia;Pteriomorphia;Ostr
eida;Ostreoidea;Ostreidae;Crassostrea;Crassostrea virginica
UdeM00000001912 29159 164.1 UdeM00000001912 XP_034335621.1 36.1 294
156 4 109 378 294 579 3.50E-37 164.1 29159 cellular
organisms;Eukaryota;Opisthokonta;Metazoa;Eumetazoa;Bilateria;Protostomia;
Spiralia;Lophotrochozoa;Mollusca;Bivalvia;Autobranchia;Pteriomorphia;Ostr
eida;Ostreoidea;Ostreidae;Crassostrea;Crassostrea gigas
UdeM00000001916 6573 155.6 UdeM00000001916 XP_021348626.1 35.3 269
142 6 14 250 257 525 8.30E-35 155.6 6573 cellular
organisms;Eukaryota;Opisthokonta;Metazoa;Eumetazoa;Bilateria;Protostomia;
Spiralia;Lophotrochozoa;Mollusca;Bivalvia;Autobranchia;Pteriomorphia;Pect
inida;Pectinoidea;Pectinidae;Mizuhopecten;Mizuhopecten yessoensis
UdeM00000001917 6596 253.1 UdeM00000001917 XP_045215628.1 48 246
123 3 25 268 86 328 4.10E-64 253.1 6596 cellular
organisms;Eukaryota;Opisthokonta;Metazoa;Eumetazoa;Bilateria;Protostomia;
Spiralia;Lophotrochozoa;Mollusca;Bivalvia;Autobranchia;Heteroconchia;Euhe
terodonta;Imparidentia;Venerida;Veneroidea;Veneridae;Mercenaria;Mercenari
a mercenaria
UdeM00000001918 6596 65.1 UdeM00000001918 XP_045185858.1 33.3 126
75 3 26 148 42 161 2.20E-07 65.1 6596 cellular
organisms;Eukaryota;Opisthokonta;Metazoa;Eumetazoa;Bilateria;Protostomia;
Spiralia;Lophotrochozoa;Mollusca;Bivalvia;Autobranchia;Heteroconchia;Euhe
terodonta;Imparidentia;Venerida;Veneroidea;Veneridae;Mercenaria;Mercenari
a mercenaria
UdeM00000001919 6596 65.1 UdeM00000001919 XP_045185858.1 33.3 126
75 3 26 148 42 161 2.30E-07 65.1 6596 cellular
organisms;Eukaryota;Opisthokonta;Metazoa;Eumetazoa;Bilateria;Protostomia;
Spiralia;Lophotrochozoa;Mollusca;Bivalvia;Autobranchia;Heteroconchia;Euhe
terodonta;Imparidentia;Venerida;Veneroidea;Veneridae;Mercenaria;Mercenari
a mercenaria
UdeM00000001920 6573 71.6 UdeM00000001920 XP_021358586.1 30.6 134
89 2 225 357 116 246 2.20E-09 71.6 6573 cellular
organisms;Eukaryota;Opisthokonta;Metazoa;Eumetazoa;Bilateria;Protostomia;
Spiralia;Lophotrochozoa;Mollusca;Bivalvia;Autobranchia;Pteriomorphia;Pect
inida;Pectinoidea;Pectinidae;Mizuhopecten;Mizuhopecten yessoensis
UdeM00000001921 6573 71.6 UdeM00000001921 XP_021358586.1 30.6 134
89 2 224 356 116 246 2.20E-09 71.6 6573 cellular
organisms;Eukaryota;Opisthokonta;Metazoa;Eumetazoa;Bilateria;Protostomia;
Spiralia;Lophotrochozoa;Mollusca;Bivalvia;Autobranchia;Pteriomorphia;Pect
inida;Pectinoidea;Pectinidae;Mizuhopecten;Mizuhopecten yessoensis
UdeM00000001922 6565 84.3 UdeM00000001922 XP_022345219.1 40 100
58 1 2 99 284 383 9.70E-14 84.3 6565 cellular
organisms;Eukaryota;Opisthokonta;Metazoa;Eumetazoa;Bilateria;Protostomia;
Spiralia;Lophotrochozoa;Mollusca;Bivalvia;Autobranchia;Pteriomorphia;Ostr
eida;Ostreoidea;Ostreidae;Crassostrea;Crassostrea virginica
UdeM00000001923 6565 327 UdeM00000001923 XP_022287686.1 56.1 262
114 1 1 262 1 261 2.20E-86 327 6565 cellular
organisms;Eukaryota;Opisthokonta;Metazoa;Eumetazoa;Bilateria;Protostomia;
Spiralia;Lophotrochozoa;Mollusca;Bivalvia;Autobranchia;Pteriomorphia;Ostr
eida;Ostreoidea;Ostreidae;Crassostrea;Crassostrea virginica

UdeM00000001924 6596 161.8 UdeM00000001924 XP_045215643.1 28.1 416
171 8 1 288 1 416 1.30E-36 161.8 6596 cellular organisms;Eukaryota;Opisthokonta;Metazoa;Eumetazoa;Bilateria;Protostomia;Spiralia;Lophotrochozoa;Mollusca;Bivalvia;Autobranchia;Heteroconchia;Euheterodonta;Imparidentia;Venerida;Veneroidea;Veneridae;Mercenaria;Mercenaria mercenaria
UdeM00000001925 6579 159.5 UdeM00000001925 XP_033744154.1 33.1 314
174 8 60 363 808 1095 8.30E-36 159.5 6579 cellular organisms;Eukaryota;Opisthokonta;Metazoa;Eumetazoa;Bilateria;Protostomia;Spiralia;Lophotrochozoa;Mollusca;Bivalvia;Autobranchia;Pteriomorphia;Pectinida;Pectinoidea;Pectinidae;Pecten;Pecten maximus
UdeM00000001926 29159 301.2 UdeM00000001926 XP_034322046.1 49.7 322
77 5 3 242 333 651 1.20E-78 301.2 29159 cellular organisms;Eukaryota;Opisthokonta;Metazoa;Eumetazoa;Bilateria;Protostomia;Spiralia;Lophotrochozoa;Mollusca;Bivalvia;Autobranchia;Pteriomorphia;Ostreida;Ostreoidea;Ostreidae;Crassostrea;Crassostrea gigas
UdeM00000001927 29159 65.1 UdeM00000001927 XP_034322639.1 43.5 92
48 2 77 164 18 109 9.80E-08 65.1 29159 cellular organisms;Eukaryota;Opisthokonta;Metazoa;Eumetazoa;Bilateria;Protostomia;Spiralia;Lophotrochozoa;Mollusca;Bivalvia;Autobranchia;Pteriomorphia;Ostreida;Ostreoidea;Ostreidae;Crassostrea;Crassostrea gigas
UdeM00000001928 6565 100.5 UdeM00000001928 XP_022290906.1 38.1 139
83 1 63 201 329 464 2.60E-18 100.5 6565 cellular organisms;Eukaryota;Opisthokonta;Metazoa;Eumetazoa;Bilateria;Protostomia;Spiralia;Lophotrochozoa;Mollusca;Bivalvia;Autobranchia;Pteriomorphia;Ostreida;Ostreoidea;Ostreidae;Crassostrea;Crassostrea virginica
UdeM00000001929 6579 103.2 UdeM00000001929 XP_033739518.1 35.4 181
90 4 50 203 522 702 4.20E-19 103.2 6579 cellular organisms;Eukaryota;Opisthokonta;Metazoa;Eumetazoa;Bilateria;Protostomia;Spiralia;Lophotrochozoa;Mollusca;Bivalvia;Autobranchia;Pteriomorphia;Pectinida;Pectinoidea;Pectinidae;Pecten;Pecten maximus
UdeM00000001930 29159 162.2 UdeM00000001930 XP_034334597.1 32.8 317
167 8 49 319 719 1035 1.10E-36 162.2 29159 cellular organisms;Eukaryota;Opisthokonta;Metazoa;Eumetazoa;Bilateria;Protostomia;Spiralia;Lophotrochozoa;Mollusca;Bivalvia;Autobranchia;Pteriomorphia;Ostreida;Ostreoidea;Ostreidae;Crassostrea;Crassostrea gigas
UdeM00000001931 6565 164.1 UdeM00000001931 XP_022288933.1 32.7 297
176 5 73 346 293 588 3.20E-37 164.1 6565 cellular organisms;Eukaryota;Opisthokonta;Metazoa;Eumetazoa;Bilateria;Protostomia;Spiralia;Lophotrochozoa;Mollusca;Bivalvia;Autobranchia;Pteriomorphia;Ostreida;Ostreoidea;Ostreidae;Crassostrea;Crassostrea virginica
UdeM00000001933 6596 84 UdeM00000001933 XP_045195973.1 36.8 152
76 4 16 164 308 442 2.10E-13 84 6596 cellular organisms;Eukaryota;Opisthokonta;Metazoa;Eumetazoa;Bilateria;Protostomia;Spiralia;Lophotrochozoa;Mollusca;Bivalvia;Autobranchia;Heteroconchia;Euheterodonta;Imparidentia;Venerida;Veneroidea;Veneridae;Mercenaria;Mercenaria mercenaria
UdeM00000001934 6565 171.8 UdeM00000001934 XP_022288931.1 34.1 305
172 5 41 318 290 592 1.40E-39 171.8 6565 cellular organisms;Eukaryota;Opisthokonta;Metazoa;Eumetazoa;Bilateria;Protostomia;Spiralia;Lophotrochozoa;Mollusca;Bivalvia;Autobranchia;Pteriomorphia;Ostreida;Ostreoidea;Ostreidae;Crassostrea;Crassostrea virginica
UdeM00000001939 6596 183.3 UdeM00000001939 XP_045195891.1 28.7 679
399 19 46 664 19 672 9.80E-43 183.3 6596 cellular

organisms;Eukaryota;Opisthokonta;Metazoa;Eumetazoa;Bilateria;Protostomia;Spiralia;Lophotrochozoa;Mollusca;Bivalvia;Autobranchia;Heteroconchia;Euheterodonta;Imparidentia;Venerida;Veneroidea;Veneridae;Mercenaria;Mercenaria mercenaria
UdeM00000001940 6596 151.8 UdeM00000001940 XP_045195892.1 40.4 250 132 6 529 776 623 857 3.70E-33 151.8 6596 cellular organisms;Eukaryota;Opisthokonta;Metazoa;Eumetazoa;Bilateria;Protostomia;Spiralia;Lophotrochozoa;Mollusca;Bivalvia;Autobranchia;Heteroconchia;Euheterodonta;Imparidentia;Venerida;Veneroidea;Veneridae;Mercenaria;Mercenaria mercenaria
UdeM00000001941 6579 470.7 UdeM00000001941 XP_033739698.1 72.3 311 86 0 11 321 11 321 1.50E-129 470.7 6579 cellular organisms;Eukaryota;Opisthokonta;Metazoa;Eumetazoa;Bilateria;Protostomia;Spiralia;Lophotrochozoa;Mollusca;Bivalvia;Autobranchia;Pteriomorphia;Pectinida;Pectinoidea;Pectinidae;Pecten;Pecten maximus
UdeM00000001943 13164 73.6 UdeM00000001943 XP_022167387.1 39.5 76 46 0 6 81 94 169 1.40E-10 73.6 13164 cellular organisms;Eukaryota;Opisthokonta;Metazoa;Eumetazoa;Bilateria;Protostomia;Ecdysozoa;Panarthropoda;Arthropoda;Mandibulata;Pancrustacea;Hexapoda;Insecta;Dicondylia;Pterygota;Neoptera;Paraneoptera;Hemiptera;Sternorrhyncha;Aphidomorpha;Aphidoidea;Aphididae;Aphidinae;Macrosiphini;Myzus;Myzus persicae
UdeM00000001944 48498 432.6 UdeM00000001944 XP_020611075.1 63.5 315 115 0 11 325 2 316 4.60E-118 432.6 48498 cellular organisms;Eukaryota;Opisthokonta;Metazoa;Eumetazoa;Cnidaria;Anthozoa;Hexacorallia;Scleractinia;Faviina;Merulinidae;Orbicella;Orbicella faveolata
UdeM00000001945 7741 453.8 UdeM00000001945 XP_019617300.1 65.9 320 109 0 13 332 29 348 2.00E-124 453.8 7741 cellular organisms;Eukaryota;Opisthokonta;Metazoa;Eumetazoa;Bilateria;Deuterostomia;Chordata;Cephalochordata;Leptocardii;Amphioxiformes;Branchiostomidae;Branchiostoma;Branchiostoma belcheri
UdeM00000001946 6579 186 UdeM00000001946 XP_033743002.1 43.8 194 109 0 115 308 10 203 7.60E-44 186 6579 cellular organisms;Eukaryota;Opisthokonta;Metazoa;Eumetazoa;Bilateria;Protostomia;Spiralia;Lophotrochozoa;Mollusca;Bivalvia;Autobranchia;Pteriomorphia;Pectinida;Pectinoidea;Pectinidae;Pecten;Pecten maximus
UdeM00000001947 6579 370.5 UdeM00000001947 XP_033740259.1 47.4 390 198 5 6 392 4 389 2.60E-99 370.5 6579 cellular organisms;Eukaryota;Opisthokonta;Metazoa;Eumetazoa;Bilateria;Protostomia;Spiralia;Lophotrochozoa;Mollusca;Bivalvia;Autobranchia;Pteriomorphia;Pectinida;Pectinoidea;Pectinidae;Pecten;Pecten maximus
UdeM00000001948 6596 106.7 UdeM00000001948 XP_045196692.1 31.7 306 174 11 30 310 27 322 6.70E-20 106.7 6596 cellular organisms;Eukaryota;Opisthokonta;Metazoa;Eumetazoa;Bilateria;Protostomia;Spiralia;Lophotrochozoa;Mollusca;Bivalvia;Autobranchia;Heteroconchia;Euheterodonta;Imparidentia;Venerida;Veneroidea;Veneridae;Mercenaria;Mercenaria mercenaria
UdeM00000001950 6105 646 UdeM00000001950 XP_031557674.1 33.9 1062 655 21 4 1032 5 1052 8.30E-182 646 6105 cellular organisms;Eukaryota;Opisthokonta;Metazoa;Eumetazoa;Cnidaria;Anthozoa;Hexacorallia;Actiniaria;Actiniidae;Actinia;Actinia tenebrosa
UdeM00000001951 6596 89.4 UdeM00000001951 XP_045197768.1 45 111 39 2 1 89 180 290 4.10E-15 89.4 6596 cellular organisms;Eukaryota;Opisthokonta;Metazoa;Eumetazoa;Bilateria;Protostomia;

Spiralia;Lophotrochozoa;Mollusca;Bivalvia;Autobranchia;Heteroconchia;Euheterodonta;Imparidentia;Venerida;Veneroidea;Veneridae;Mercenaria;Mercenaria mercenaria
UdeM00000001952 6105 637.5 UdeM00000001952 XP_031557674.1 34.2 1041 653 16 7 1024 21 1052 2.90E-179 637.5 6105 cellular organisms;Eukaryota;Opisthokonta;Metazoa;Eumetazoa;Cnidaria;Anthozoa;Hexacorallia;Actiniaria;Actiniidae;Actinia;Actinia tenebrosa
UdeM00000001953 6105 646.4 UdeM00000001953 XP_031557674.1 34.2 1056 658 17 2 1029 6 1052 6.30E-182 646.4 6105 cellular organisms;Eukaryota;Opisthokonta;Metazoa;Eumetazoa;Cnidaria;Anthozoa;Hexacorallia;Actiniaria;Actiniidae;Actinia;Actinia tenebrosa
UdeM00000001954 6105 618.6 UdeM00000001954 XP_031557674.1 33.8 1053 660 16 5 1027 7 1052 1.40E-173 618.6 6105 cellular organisms;Eukaryota;Opisthokonta;Metazoa;Eumetazoa;Cnidaria;Anthozoa;Hexacorallia;Actiniaria;Actiniidae;Actinia;Actinia tenebrosa
UdeM00000001955 6105 618.6 UdeM00000001955 XP_031557674.1 33.8 1053 660 16 10 1032 7 1052 1.40E-173 618.6 6105 cellular organisms;Eukaryota;Opisthokonta;Metazoa;Eumetazoa;Cnidaria;Anthozoa;Hexacorallia;Actiniaria;Actiniidae;Actinia;Actinia tenebrosa
UdeM00000001956 6596 447.6 UdeM00000001956 XP_045198784.1 49.7 431 216 1 3 432 103 533 1.80E-122 447.6 6596 cellular organisms;Eukaryota;Opisthokonta;Metazoa;Eumetazoa;Bilateria;Protostomia;Spiralia;Lophotrochozoa;Mollusca;Bivalvia;Autobranchia;Heteroconchia;Euheterodonta;Imparidentia;Venerida;Veneroidea;Veneridae;Mercenaria;Mercenaria mercenaria
UdeM00000001957 6596 387.9 UdeM00000001957 XP_045198127.1 46 448 231 7 35 479 54 493 2.10E-104 387.9 6596 cellular organisms;Eukaryota;Opisthokonta;Metazoa;Eumetazoa;Bilateria;Protostomia;Spiralia;Lophotrochozoa;Mollusca;Bivalvia;Autobranchia;Heteroconchia;Euheterodonta;Imparidentia;Venerida;Veneroidea;Veneridae;Mercenaria;Mercenaria mercenaria
UdeM00000001958 6105 625.9 UdeM00000001958 XP_031557674.1 33.9 1056 655 18 5 1027 7 1052 8.80E-176 625.9 6105 cellular organisms;Eukaryota;Opisthokonta;Metazoa;Eumetazoa;Cnidaria;Anthozoa;Hexacorallia;Actiniaria;Actiniidae;Actinia;Actinia tenebrosa
UdeM00000001959 6105 626.3 UdeM00000001959 XP_031557674.1 34 1053 658 17 5 1027 7 1052 6.80E-176 626.3 6105 cellular organisms;Eukaryota;Opisthokonta;Metazoa;Eumetazoa;Cnidaria;Anthozoa;Hexacorallia;Actiniaria;Actiniidae;Actinia;Actinia tenebrosa
UdeM00000001960 6573 443 UdeM00000001960 XP_021349183.1 68.1 383 121 1 1 383 81 462 4.00E-121 443 6573 cellular organisms;Eukaryota;Opisthokonta;Metazoa;Eumetazoa;Bilateria;Protostomia;Spiralia;Lophotrochozoa;Mollusca;Bivalvia;Autobranchia;Pteriomorphia;Pectinida;Pectinoidea;Pectinidae;Mizuhopecten;Mizuhopecten yessoensis
UdeM00000001961 6573 578.6 UdeM00000001961 XP_021349183.1 70.8 463 134 1 1 463 1 462 7.30E-162 578.6 6573 cellular organisms;Eukaryota;Opisthokonta;Metazoa;Eumetazoa;Bilateria;Protostomia;Spiralia;Lophotrochozoa;Mollusca;Bivalvia;Autobranchia;Pteriomorphia;Pectinida;Pectinoidea;Pectinidae;Mizuhopecten;Mizuhopecten yessoensis
UdeM00000001962 218467 112.8 UdeM00000001962 XP_023219748.1 53 115 53 1 15 128 84 198 3.20E-22 112.8 218467 cellular organisms;Eukaryota;Opisthokonta;Metazoa;Eumetazoa;Bilateria;Protostomia;

Ecdysozoa;Panarthropoda;Arthropoda;Chelicerata;Arachnida;Scorpiones;Buthida;Buthoidea;Buthidae;Centruroides;Centruroides sculpturatus
UdeM00000001963 1735272 228.8 UdeM00000001963 XP_041354581.1 47.4 230 117 3 24 250 91 319 9.80E-57 228.8 1735272 cellular organisms;Eukaryota;Opisthokonta;Metazoa;Eumetazoa;Bilateria;Protostomia;Spiralia;Lophotrochozoa;Mollusca;Gastropoda;Neomphalina;Neomphaloidea;Peltospiridae;Gigantopelta;Gigantopelta aegis
UdeM00000001964 1735272 211.1 UdeM00000001964 XP_041354581.1 47.6 210 106 3 48 253 121 330 1.70E-51 211.1 1735272 cellular organisms;Eukaryota;Opisthokonta;Metazoa;Eumetazoa;Bilateria;Protostomia;Spiralia;Lophotrochozoa;Mollusca;Gastropoda;Neomphalina;Neomphaloidea;Peltospiridae;Gigantopelta;Gigantopelta aegis
UdeM00000001965 6596 1946.4 UdeM00000001965 XP_045198705.1 52 1958 830 23 1 1869 407 2343 0.00E+00 1946.4 6596 cellular organisms;Eukaryota;Opisthokonta;Metazoa;Eumetazoa;Bilateria;Protostomia;Spiralia;Lophotrochozoa;Mollusca;Bivalvia;Autobranchia;Heteroconchia;Euheterodonta;Imparidentia;Venerida;Veneroidea;Veneridae;Mercenaria;Mercenaria mercenaria
UdeM00000001967 6596 629.4 UdeM00000001967 XP_045198706.1 38.8 981 550 15 1 969 760 1702 8.30E-177 629.4 6596 cellular organisms;Eukaryota;Opisthokonta;Metazoa;Eumetazoa;Bilateria;Protostomia;Spiralia;Lophotrochozoa;Mollusca;Bivalvia;Autobranchia;Heteroconchia;Euheterodonta;Imparidentia;Venerida;Veneroidea;Veneridae;Mercenaria;Mercenaria mercenaria
UdeM00000001968 6596 593.2 UdeM00000001968 XP_045198706.1 52.7 584 271 5 20 601 178 758 4.00E-166 593.2 6596 cellular organisms;Eukaryota;Opisthokonta;Metazoa;Eumetazoa;Bilateria;Protostomia;Spiralia;Lophotrochozoa;Mollusca;Bivalvia;Autobranchia;Heteroconchia;Euheterodonta;Imparidentia;Venerida;Veneroidea;Veneridae;Mercenaria;Mercenaria mercenaria
UdeM00000001969 1529436 60.1 UdeM00000001969 XP_033124707.1 30.2 106 62 2 27 120 503 608 2.30E-06 60.1 1529436 cellular organisms;Eukaryota;Opisthokonta;Metazoa;Eumetazoa;Bilateria;Deuterostomia;Echinodermata;Pelmatozoa;Crinoidea;Articulata;Comatulida;Comatulidae;Comatulinae;Anneissia;Anneissia japonica
UdeM00000001970 48498 151.4 UdeM00000001970 XP_020611646.1 30 293 197 4 4 288 1178 1470 1.80E-33 151.4 48498 cellular organisms;Eukaryota;Opisthokonta;Metazoa;Eumetazoa;Cnidaria;Anthozoa;Hexacorallia;Scleractinia;Faviina;Merulinidae;Orbicella;Orbicella faveolata
UdeM00000001971 7741 559.7 UdeM00000001971 XP_019618826.1 56.8 477 205 1 1 476 47 523 3.60E-156 559.7 7741 cellular organisms;Eukaryota;Opisthokonta;Metazoa;Eumetazoa;Bilateria;Deuterostomia;Chordata;Cephalochordata;Leptocardii;Amphioxiformes;Branchiostomidae;Branchiostoma;Branchiostoma belcheri
UdeM00000001972 1529436 117.5 UdeM00000001972 XP_033124707.1 33.3 231 136 6 2 224 388 608 2.30E-23 117.5 1529436 cellular organisms;Eukaryota;Opisthokonta;Metazoa;Eumetazoa;Bilateria;Deuterostomia;Echinodermata;Pelmatozoa;Crinoidea;Articulata;Comatulida;Comatulidae;Comatulinae;Anneissia;Anneissia japonica

UdeM00000001973 29159 132.1 UdeM00000001973 XP_034319761.1 26.9 275
177 4 108 365 19 286 1.50E-27 132.1 29159 cellular
organisms;Eukaryota;Opisthokonta;Metazoa;Eumetazoa;Bilateria;Protostomia;
Spiralia;Lophotrochozoa;Mollusca;Bivalvia;Autobranchia;Pteriomorphia;Ostr
eida;Ostreoidea;Ostreidae;Crassostrea;Crassostrea gigas
UdeM00000001974 1529436 60.8 UdeM00000001974 XP_033124707.1 28.8
111 70 2 5 107 499 608 1.20E-06 60.8 1529436
cellular
organisms;Eukaryota;Opisthokonta;Metazoa;Eumetazoa;Bilateria;Deuterostomi
a;Echinodermata;Pelmatozoa;Crinoidea;Articulata;Comatulida;Comatulidae;Co
matulinae;Anneissia;Anneissia japonica
UdeM00000001975 29159 362.5 UdeM00000001975 XP_034319761.1 27.9 746
532 4 68 811 10 751 1.40E-96 362.5 29159 cellular
organisms;Eukaryota;Opisthokonta;Metazoa;Eumetazoa;Bilateria;Protostomia;
Spiralia;Lophotrochozoa;Mollusca;Bivalvia;Autobranchia;Pteriomorphia;Ostr
eida;Ostreoidea;Ostreidae;Crassostrea;Crassostrea gigas
UdeM00000001978 6596 137.9 UdeM00000001978 XP_045198856.1 38.7 204
99 6 43 229 65 259 1.80E-29 137.9 6596 cellular
organisms;Eukaryota;Opisthokonta;Metazoa;Eumetazoa;Bilateria;Protostomia;
Spiralia;Lophotrochozoa;Mollusca;Bivalvia;Autobranchia;Heteroconchia;Euhe
terodonta;Imparidentia;Venerida;Veneroidea;Veneridae;Mercenaria;Mercenari
a mercenaria
UdeM00000001979 37653 215.7 UdeM00000001979 XP_014776580.1 89.3 121
13 0 1 121 1 121 3.30E-53 215.7 37653 cellular
organisms;Eukaryota;Opisthokonta;Metazoa;Eumetazoa;Bilateria;Protostomia;
Spiralia;Lophotrochozoa;Mollusca;Cephalopoda;Coleoidea;Octopodiformes;Oct
opoda;Incirrata;Octopodidae;Octopus;Octopus bimaculoides
UdeM00000001980 6526 59.7 UdeM00000001980 XP_013089460.1 37.5 128
64 6 7 134 5 116 3.50E-06 59.7 6526 cellular
organisms;Eukaryota;Opisthokonta;Metazoa;Eumetazoa;Bilateria;Protostomia;
Spiralia;Lophotrochozoa;Mollusca;Gastropoda;Heterobranchia;Euthyneura;Pan
pulmonata;Hygrophila;Lymnaeoidea;Planorbidae;Biomphalaria;Biomphalaria
glabrata
UdeM00000001982 6596 1251.1 UdeM00000001982 XP_045198596.1 49.8
1358 545 20 28 1333 16 1288 0.00E+00 1251.1 6596
cellular
organisms;Eukaryota;Opisthokonta;Metazoa;Eumetazoa;Bilateria;Protostomia;
Spiralia;Lophotrochozoa;Mollusca;Bivalvia;Autobranchia;Heteroconchia;Euhe
terodonta;Imparidentia;Venerida;Veneroidea;Veneridae;Mercenaria;Mercenari
a mercenaria
UdeM00000001984 6596 145.2 UdeM00000001984 XP_045195551.1 36.4 231
132 6 189 417 821 1038 2.20E-31 145.2 6596 cellular
organisms;Eukaryota;Opisthokonta;Metazoa;Eumetazoa;Bilateria;Protostomia;
Spiralia;Lophotrochozoa;Mollusca;Bivalvia;Autobranchia;Heteroconchia;Euhe
terodonta;Imparidentia;Venerida;Veneroidea;Veneridae;Mercenaria;Mercenari
a mercenaria
UdeM00000001986 6573 77.8 UdeM00000001986 XP_021376553.1 47.4 76
38 1 1 76 3 76 7.80E-12 77.8 6573 cellular
organisms;Eukaryota;Opisthokonta;Metazoa;Eumetazoa;Bilateria;Protostomia;
Spiralia;Lophotrochozoa;Mollusca;Bivalvia;Autobranchia;Pteriomorphia;Pect
inida;Pectinoidea;Pectinidae;Mizuhopecten;Mizuhopecten yessoensis
UdeM00000001989 6596 131.3 UdeM00000001989 XP_045195551.1 40.7 162
89 3 34 193 836 992 1.60E-27 131.3 6596 cellular
organisms;Eukaryota;Opisthokonta;Metazoa;Eumetazoa;Bilateria;Protostomia;

Spiralia;Lophotrochozoa;Mollusca;Bivalvia;Autobranchia;Heteroconchia;Euheterodonta;Imparidentia;Venerida;Veneroidea;Veneridae;Mercenaria;Mercenaria mercenaria
UdeM00000001990 6596 171.4 UdeM00000001990 XP_045187536.1 52.2 161 73 1 11 171 50 206 1.00E-39 171.4 6596 cellular organisms;Eukaryota;Opisthokonta;Metazoa;Eumetazoa;Bilateria;Protostomia;Spiralia;Lophotrochozoa;Mollusca;Bivalvia;Autobranchia;Heteroconchia;Euheterodonta;Imparidentia;Venerida;Veneroidea;Veneridae;Mercenaria;Mercenaria mercenaria
UdeM00000001991 6596 344.4 UdeM00000001991 XP_045187536.1 74.1 232 59 1 2 233 228 458 1.20E-91 344.4 6596 cellular organisms;Eukaryota;Opisthokonta;Metazoa;Eumetazoa;Bilateria;Protostomia;Spiralia;Lophotrochozoa;Mollusca;Bivalvia;Autobranchia;Heteroconchia;Euheterodonta;Imparidentia;Venerida;Veneroidea;Veneridae;Mercenaria;Mercenaria mercenaria
UdeM00000001992 6596 102.4 UdeM00000001992 XP_045187536.1 74.6 63 16 0 1 63 396 458 2.40E-19 102.4 6596 cellular organisms;Eukaryota;Opisthokonta;Metazoa;Eumetazoa;Bilateria;Protostomia;Spiralia;Lophotrochozoa;Mollusca;Bivalvia;Autobranchia;Heteroconchia;Euheterodonta;Imparidentia;Venerida;Veneroidea;Veneridae;Mercenaria;Mercenaria mercenaria
UdeM00000001993 6596 123.6 UdeM00000001993 XP_045187536.1 54.1 111 49 1 1 111 496 604 2.40E-25 123.6 6596 cellular organisms;Eukaryota;Opisthokonta;Metazoa;Eumetazoa;Bilateria;Protostomia;Spiralia;Lophotrochozoa;Mollusca;Bivalvia;Autobranchia;Heteroconchia;Euheterodonta;Imparidentia;Venerida;Veneroidea;Veneridae;Mercenaria;Mercenaria mercenaria
UdeM00000001994 6596 498 UdeM00000001994 XP_045195551.1 34.7 1124 577 23 336 1348 391 1468 3.70E-137 498 6596 cellular organisms;Eukaryota;Opisthokonta;Metazoa;Eumetazoa;Bilateria;Protostomia;Spiralia;Lophotrochozoa;Mollusca;Bivalvia;Autobranchia;Heteroconchia;Euheterodonta;Imparidentia;Venerida;Veneroidea;Veneridae;Mercenaria;Mercenaria mercenaria
UdeM00000001997 6579 146.7 UdeM00000001997 XP_033741098.1 50.4 137 67 1 2 137 4 140 2.10E-32 146.7 6579 cellular organisms;Eukaryota;Opisthokonta;Metazoa;Eumetazoa;Bilateria;Protostomia;Spiralia;Lophotrochozoa;Mollusca;Bivalvia;Autobranchia;Pteriomorphia;Pectinida;Pectinoidea;Pectinidae;Pecten;Pecten maximus
UdeM00000001998 6579 146.7 UdeM00000001998 XP_033741098.1 50.4 137 67 1 7 142 4 140 2.20E-32 146.7 6579 cellular organisms;Eukaryota;Opisthokonta;Metazoa;Eumetazoa;Bilateria;Protostomia;Spiralia;Lophotrochozoa;Mollusca;Bivalvia;Autobranchia;Pteriomorphia;Pectinida;Pectinoidea;Pectinidae;Pecten;Pecten maximus
UdeM00000001999 6596 748.4 UdeM00000001999 XP_045195549.1 77.4 446 97 2 1 445 1 443 5.20E-213 748.4 6596 cellular organisms;Eukaryota;Opisthokonta;Metazoa;Eumetazoa;Bilateria;Protostomia;Spiralia;Lophotrochozoa;Mollusca;Bivalvia;Autobranchia;Heteroconchia;Euheterodonta;Imparidentia;Venerida;Veneroidea;Veneridae;Mercenaria;Mercenaria mercenaria
UdeM00000002000 6596 840.1 UdeM00000002000 XP_045195108.1 49.5 1187 469 27 1 1149 1 1095 3.30E-240 840.1 6596 cellular organisms;Eukaryota;Opisthokonta;Metazoa;Eumetazoa;Bilateria;Protostomia;Spiralia;Lophotrochozoa;Mollusca;Bivalvia;Autobranchia;Heteroconchia;Euhe

terodonta;Imparidentia;Venerida;Veneroidea;Veneridae;Mercenaria;Mercenaria mercenaria
UdeM00000002001 6565 134 UdeM00000002001 XP_022299727.1 50 134 66 1 29 162 145 277 1.90E-28 134 6565 cellular organisms;Eukaryota;Opisthokonta;Metazoa;Eumetazoa;Bilateria;Protostomia;Spiralia;Lophotrochozoa;Mollusca;Bivalvia;Autobranchia;Pteriomorphia;Ostreida;Ostreoidea;Ostreidae;Crassostrea;Crassostrea virginica
UdeM00000002002 6596 458.4 UdeM00000002002 XP_045190515.1 32.6 973 534 28 281 1226 1100 1977 2.90E-125 458.4 6596 cellular organisms;Eukaryota;Opisthokonta;Metazoa;Eumetazoa;Bilateria;Protostomia;Spiralia;Lophotrochozoa;Mollusca;Bivalvia;Autobranchia;Heteroconchia;Euheterodonta;Imparidentia;Venerida;Veneroidea;Veneridae;Mercenaria;Mercenaria mercenaria
UdeM00000002003 6596 602.4 UdeM00000002003 XP_045190527.1 48.7 622 313 3 1 621 36 652 6.40E-169 602.4 6596 cellular organisms;Eukaryota;Opisthokonta;Metazoa;Eumetazoa;Bilateria;Protostomia;Spiralia;Lophotrochozoa;Mollusca;Bivalvia;Autobranchia;Heteroconchia;Euheterodonta;Imparidentia;Venerida;Veneroidea;Veneridae;Mercenaria;Mercenaria mercenaria
UdeM00000002004 29159 243 UdeM00000002004 XP_034322429.1 29 637 211 7 4 403 1 633 1.20E-60 243 29159 cellular organisms;Eukaryota;Opisthokonta;Metazoa;Eumetazoa;Bilateria;Protostomia;Spiralia;Lophotrochozoa;Mollusca;Bivalvia;Autobranchia;Pteriomorphia;Ostreida;Ostreoidea;Ostreidae;Crassostrea;Crassostrea gigas
UdeM00000002005 6596 226.9 UdeM00000002005 XP_045204261.1 41.3 276 150 6 6 276 39 307 3.30E-56 226.9 6596 cellular organisms;Eukaryota;Opisthokonta;Metazoa;Eumetazoa;Bilateria;Protostomia;Spiralia;Lophotrochozoa;Mollusca;Bivalvia;Autobranchia;Heteroconchia;Euheterodonta;Imparidentia;Venerida;Veneroidea;Veneridae;Mercenaria;Mercenaria mercenaria
UdeM00000002006 6596 232.3 UdeM00000002006 XP_045202146.1 46.7 242 112 3 45 272 77 315 7.70E-58 232.3 6596 cellular organisms;Eukaryota;Opisthokonta;Metazoa;Eumetazoa;Bilateria;Protostomia;Spiralia;Lophotrochozoa;Mollusca;Bivalvia;Autobranchia;Heteroconchia;Euheterodonta;Imparidentia;Venerida;Veneroidea;Veneridae;Mercenaria;Mercenaria mercenaria
UdeM00000002007 6596 232.3 UdeM00000002007 XP_045202146.1 46.7 242 112 3 46 273 77 315 7.80E-58 232.3 6596 cellular organisms;Eukaryota;Opisthokonta;Metazoa;Eumetazoa;Bilateria;Protostomia;Spiralia;Lophotrochozoa;Mollusca;Bivalvia;Autobranchia;Heteroconchia;Euheterodonta;Imparidentia;Venerida;Veneroidea;Veneridae;Mercenaria;Mercenaria mercenaria
UdeM00000002008 6579 856.3 UdeM00000002008 XP_033756903.1 76.1 876 209 0 16 891 1 876 3.50E-245 856.3 6579 cellular organisms;Eukaryota;Opisthokonta;Metazoa;Eumetazoa;Bilateria;Protostomia;Spiralia;Lophotrochozoa;Mollusca;Bivalvia;Autobranchia;Pteriomorphia;Pectinida;Pectinoidea;Pectinidae;Pecten;Pecten maximus
UdeM00000002009 400727 984.2 UdeM00000002009 XP_025095839.1 78.5 873 188 0 6 878 3 875 1.10E-283 984.2 400727 cellular organisms;Eukaryota;Opisthokonta;Metazoa;Eumetazoa;Bilateria;Protostomia;Spiralia;Lophotrochozoa;Mollusca;Gastropoda;Caenogastropoda;Architaenioglossa;Ampullarioidea;Ampullariidae;Pomacea;Pomacea canaliculata

UdeM00000002010 400727 986.5 UdeM00000002010 XP_025095839.1 78.6
877 186 1 1 877 1 875 2.20E-284 986.5 400727
cellular
organisms;Eukaryota;Opisthokonta;Metazoa;Eumetazoa;Bilateria;Protostomia;
Spiralia;Lophotrochozoa;Mollusca;Gastropoda;Caenogastropoda;Architaenioglossa;Ampullarioidea;Ampullariidae;Pomacea;Pomacea canaliculata
UdeM00000002012 6596 213.8 UdeM00000002012 XP_045189306.1 66.4 152
51 0 5 156 6 157 1.70E-52 213.8 6596 cellular
organisms;Eukaryota;Opisthokonta;Metazoa;Eumetazoa;Bilateria;Protostomia;
Spiralia;Lophotrochozoa;Mollusca;Bivalvia;Autobranchia;Heteroconchia;Euheterodonta;Imparidentia;Venerida;Veneroidea;Veneridae;Mercenaria;Mercenaria mercenaria
UdeM00000002013 6596 197.2 UdeM00000002013 XP_045189304.1 53.8 169
76 2 1 167 1 169 1.70E-47 197.2 6596 cellular
organisms;Eukaryota;Opisthokonta;Metazoa;Eumetazoa;Bilateria;Protostomia;
Spiralia;Lophotrochozoa;Mollusca;Bivalvia;Autobranchia;Heteroconchia;Euheterodonta;Imparidentia;Venerida;Veneroidea;Veneridae;Mercenaria;Mercenaria mercenaria
UdeM00000002014 6596 287.7 UdeM00000002014 XP_045173426.1 51.1 266
120 4 7 264 6 269 1.50E-74 287.7 6596 cellular
organisms;Eukaryota;Opisthokonta;Metazoa;Eumetazoa;Bilateria;Protostomia;
Spiralia;Lophotrochozoa;Mollusca;Bivalvia;Autobranchia;Heteroconchia;Euheterodonta;Imparidentia;Venerida;Veneroidea;Veneridae;Mercenaria;Mercenaria mercenaria
UdeM00000002015 6596 270.4 UdeM00000002015 XP_045173426.1 48.4 248
122 3 19 260 22 269 2.40E-69 270.4 6596 cellular
organisms;Eukaryota;Opisthokonta;Metazoa;Eumetazoa;Bilateria;Protostomia;
Spiralia;Lophotrochozoa;Mollusca;Bivalvia;Autobranchia;Heteroconchia;Euheterodonta;Imparidentia;Venerida;Veneroidea;Veneridae;Mercenaria;Mercenaria mercenaria
UdeM00000002016 6565 363.2 UdeM00000002016 XP_022289852.1 43.1 501
256 11 11 497 8 493 5.30E-97 363.2 6565 cellular
organisms;Eukaryota;Opisthokonta;Metazoa;Eumetazoa;Bilateria;Protostomia;
Spiralia;Lophotrochozoa;Mollusca;Bivalvia;Autobranchia;Pteriomorphia;Ostreida;Ostreoidea;Ostreidae;Crassostrea;Crassostrea virginica
UdeM00000002017 1735272 387.5 UdeM00000002017 XP_041368165.1 48.1
1102 567 2 3 1099 2 1103 5.70E-104 387.5 1735272
cellular
organisms;Eukaryota;Opisthokonta;Metazoa;Eumetazoa;Bilateria;Protostomia;
Spiralia;Lophotrochozoa;Mollusca;Gastropoda;Neomphalina;Neomphaloidea;Peltospiridae;Gigantopelta;Gigantopelta aegis
UdeM00000002018 6565 144.1 UdeM00000002018 XP_022343804.1 53 217
101 1 14 229 25 241 2.30E-31 144.1 6565 cellular
organisms;Eukaryota;Opisthokonta;Metazoa;Eumetazoa;Bilateria;Protostomia;
Spiralia;Lophotrochozoa;Mollusca;Bivalvia;Autobranchia;Pteriomorphia;Ostreida;Ostreoidea;Ostreidae;Crassostrea;Crassostrea virginica
UdeM00000002019 27405 75.1 UdeM00000002019 XP_042874304.1 34.4 122
64 3 153 260 199 318 1.50E-10 75.1 27405 cellular
organisms;Eukaryota;Opisthokonta;Metazoa;Eumetazoa;Bilateria;Protostomia;
Ecdysozoa;Panarthropoda;Arthropoda;Mandibulata;Pancrustacea;Crustacea;Multicrustacea;Malacostraca;Eumalacostraca;Eucarida;Decapoda;Dendrobranchiata;Penaeoidea;Penaeidae;Penaeus;Penaeus japonicus
UdeM00000002021 6596 687.2 UdeM00000002021 XP_045177521.1 62.4 651
202 6 62 670 454 1103 2.10E-194 687.2 6596 cellular

organisms;Eukaryota;Opisthokonta;Metazoa;Eumetazoa;Bilateria;Protostomia;Spiralia;Lophotrochozoa;Mollusca;Bivalvia;Autobranchia;Heteroconchia;Euheterodonta;Imparidentia;Venerida;Veneroidea;Veneridae;Mercenaria;Mercenaria mercenaria
UdeM00000002020 6596 1233 UdeM00000002020 XP_045177521.1 61.4 1116 369 11 1 1067 1 1103 0.00E+00 1233 6596 cellular organisms;Eukaryota;Opisthokonta;Metazoa;Eumetazoa;Bilateria;Protostomia;Spiralia;Lophotrochozoa;Mollusca;Bivalvia;Autobranchia;Heteroconchia;Euheterodonta;Imparidentia;Venerida;Veneroidea;Veneridae;Mercenaria;Mercenaria mercenaria
UdeM00000002022 6596 953.4 UdeM00000002022 XP_045177521.1 60.7 898 291 11 1 849 1 885 2.00E-274 953.4 6596 cellular organisms;Eukaryota;Opisthokonta;Metazoa;Eumetazoa;Bilateria;Protostomia;Spiralia;Lophotrochozoa;Mollusca;Bivalvia;Autobranchia;Heteroconchia;Euheterodonta;Imparidentia;Venerida;Veneroidea;Veneridae;Mercenaria;Mercenaria mercenaria
UdeM00000002023 6579 191.8 UdeM00000002023 XP_033757264.1 34.7 430 252 14 5 419 18 433 2.10E-45 191.8 6579 cellular organisms;Eukaryota;Opisthokonta;Metazoa;Eumetazoa;Bilateria;Protostomia;Spiralia;Lophotrochozoa;Mollusca;Bivalvia;Autobranchia;Pteriomorphia;Pectinida;Pectinoidea;Pectinidae;Pecten;Pecten maximus
UdeM00000002024 6579 450.7 UdeM00000002024 XP_033757412.1 53.3 448 208 1 21 468 6 452 3.70E-123 450.7 6579 cellular organisms;Eukaryota;Opisthokonta;Metazoa;Eumetazoa;Bilateria;Protostomia;Spiralia;Lophotrochozoa;Mollusca;Bivalvia;Autobranchia;Pteriomorphia;Pectinida;Pectinoidea;Pectinidae;Pecten;Pecten maximus
UdeM00000002025 6596 2055.8 UdeM00000002025 XP_045188105.1 58.9 1984 684 13 365 2232 1039 3007 0.00E+00 2055.8 6596 cellular organisms;Eukaryota;Opisthokonta;Metazoa;Eumetazoa;Bilateria;Protostomia;Spiralia;Lophotrochozoa;Mollusca;Bivalvia;Autobranchia;Heteroconchia;Euheterodonta;Imparidentia;Venerida;Veneroidea;Veneridae;Mercenaria;Mercenaria mercenaria
UdeM00000002026 6596 957.2 UdeM00000002026 XP_045188105.1 63.4 796 197 8 1 716 256 1037 1.20E-275 957.2 6596 cellular organisms;Eukaryota;Opisthokonta;Metazoa;Eumetazoa;Bilateria;Protostomia;Spiralia;Lophotrochozoa;Mollusca;Bivalvia;Autobranchia;Heteroconchia;Euheterodonta;Imparidentia;Venerida;Veneroidea;Veneridae;Mercenaria;Mercenaria mercenaria
UdeM00000002027 6596 466.1 UdeM00000002027 XP_045188105.1 74.5 310 69 3 1 309 1 301 3.50E-128 466.1 6596 cellular organisms;Eukaryota;Opisthokonta;Metazoa;Eumetazoa;Bilateria;Protostomia;Spiralia;Lophotrochozoa;Mollusca;Bivalvia;Autobranchia;Heteroconchia;Euheterodonta;Imparidentia;Venerida;Veneroidea;Veneridae;Mercenaria;Mercenaria mercenaria
UdeM00000002028 6596 427.9 UdeM00000002028 XP_045188105.1 80.4 255 49 1 1 255 1 254 8.80E-117 427.9 6596 cellular organisms;Eukaryota;Opisthokonta;Metazoa;Eumetazoa;Bilateria;Protostomia;Spiralia;Lophotrochozoa;Mollusca;Bivalvia;Autobranchia;Heteroconchia;Euheterodonta;Imparidentia;Venerida;Veneroidea;Veneridae;Mercenaria;Mercenaria mercenaria
UdeM00000002029 6579 302.4 UdeM00000002029 XP_033756003.1 61.3 248 95 1 23 269 1 248 5.90E-79 302.4 6579 cellular organisms;Eukaryota;Opisthokonta;Metazoa;Eumetazoa;Bilateria;Protostomia;

Spiralia;Lophotrochozoa;Mollusca;Bivalvia;Autobranchia;Pteriomorphia;Pectinida;Pectinoidea;Pectinidae;Pecten;Pecten maximus
UdeM00000002030 29159 258.8 UdeM00000002030 XP_034328347.1 42 348 184 5 1 346 1 332 1.10E-65 258.8 29159 cellular organisms;Eukaryota;Opisthokonta;Metazoa;Eumetazoa;Bilateria;Protostomia;Spiralia;Lophotrochozoa;Mollusca;Bivalvia;Autobranchia;Pteriomorphia;Ostreida;Ostreoidea;Ostreidae;Crassostrea;Crassostrea gigas
UdeM00000002031 29159 267.7 UdeM00000002031 XP_034328346.1 41.1 336 187 2 1 336 1 325 2.20E-68 267.7 29159 cellular organisms;Eukaryota;Opisthokonta;Metazoa;Eumetazoa;Bilateria;Protostomia;Spiralia;Lophotrochozoa;Mollusca;Bivalvia;Autobranchia;Pteriomorphia;Ostreida;Ostreoidea;Ostreidae;Crassostrea;Crassostrea gigas
UdeM00000002032 29159 515 UdeM00000002032 XP_011426140.1 72.3 382 93 5 1 379 1 372 8.40E-143 515 29159 cellular organisms;Eukaryota;Opisthokonta;Metazoa;Eumetazoa;Bilateria;Protostomia;Spiralia;Lophotrochozoa;Mollusca;Bivalvia;Autobranchia;Pteriomorphia;Ostreida;Ostreoidea;Ostreidae;Crassostrea;Crassostrea gigas
UdeM00000002033 1735272 257.3 UdeM00000002033 XP_041369094.1 27.5 724 421 15 1 719 1 625 6.00E-65 257.3 1735272 cellular organisms;Eukaryota;Opisthokonta;Metazoa;Eumetazoa;Bilateria;Protostomia;Spiralia;Lophotrochozoa;Mollusca;Gastropoda;Neomphalina;Neomphaloidea;Peltospiridae;Gigantopelta;Gigantopelta aegis
UdeM00000002034 6596 100.9 UdeM00000002034 XP_045189611.1 44.7 114 60 2 454 566 629 740 5.50E-18 100.9 6596 cellular organisms;Eukaryota;Opisthokonta;Metazoa;Eumetazoa;Bilateria;Protostomia;Spiralia;Lophotrochozoa;Mollusca;Bivalvia;Autobranchia;Heteroconchia;Euheterodonta;Imparidentia;Venerida;Veneroidea;Veneridae;Mercenaria;Mercenaria mercenaria
UdeM00000002035 6596 100.9 UdeM00000002035 XP_045189611.1 44.7 114 60 2 420 532 629 740 5.10E-18 100.9 6596 cellular organisms;Eukaryota;Opisthokonta;Metazoa;Eumetazoa;Bilateria;Protostomia;Spiralia;Lophotrochozoa;Mollusca;Bivalvia;Autobranchia;Heteroconchia;Euheterodonta;Imparidentia;Venerida;Veneroidea;Veneridae;Mercenaria;Mercenaria mercenaria
UdeM00000002038 6596 90.1 UdeM00000002038 XP_045189611.1 49.4 77 37 1 6 80 8 84 1.60E-15 90.1 6596 cellular organisms;Eukaryota;Opisthokonta;Metazoa;Eumetazoa;Bilateria;Protostomia;Spiralia;Lophotrochozoa;Mollusca;Bivalvia;Autobranchia;Heteroconchia;Euheterodonta;Imparidentia;Venerida;Veneroidea;Veneridae;Mercenaria;Mercenaria mercenaria
UdeM00000002039 6565 211.5 UdeM00000002039 XP_022321940.1 22.9 686 480 11 4 646 6 685 3.30E-51 211.5 6565 cellular organisms;Eukaryota;Opisthokonta;Metazoa;Eumetazoa;Bilateria;Protostomia;Spiralia;Lophotrochozoa;Mollusca;Bivalvia;Autobranchia;Pteriomorphia;Ostreida;Ostreoidea;Ostreidae;Crassostrea;Crassostrea virginica
UdeM00000002040 6596 65.9 UdeM00000002040 XP_045206071.1 39.2 74 44 1 52 125 689 761 4.80E-08 65.9 6596 cellular organisms;Eukaryota;Opisthokonta;Metazoa;Eumetazoa;Bilateria;Protostomia;Spiralia;Lophotrochozoa;Mollusca;Bivalvia;Autobranchia;Heteroconchia;Euheterodonta;Imparidentia;Venerida;Veneroidea;Veneridae;Mercenaria;Mercenaria mercenaria
UdeM00000002041 29159 143.7 UdeM00000002041 XP_034317706.1 31.2 250 160 4 12 261 18 255 6.20E-31 143.7 29159 cellular

organisms;Eukaryota;Opisthokonta;Metazoa;Eumetazoa;Bilateria;Protostomia;Spiralia;Lophotrochozoa;Mollusca;Bivalvia;Autobranchia;Pteriomorphia;Ostreida;Ostreoidea;Ostreidae;Crassostrea;Crassostrea gigas
UdeM00000002042 6565 232.6 UdeM00000002042 XP_022343730.1 49.7 288 121 10 2 272 38 318 6.10E-58 232.6 6565 cellular organisms;Eukaryota;Opisthokonta;Metazoa;Eumetazoa;Bilateria;Protostomia;Spiralia;Lophotrochozoa;Mollusca;Bivalvia;Autobranchia;Pteriomorphia;Ostreida;Ostreoidea;Ostreidae;Crassostrea;Crassostrea virginica
UdeM00000002043 7574 727.6 UdeM00000002043 XP_013409497.1 42 1010 566 8 1 995 791 1795 2.10E-206 727.6 7574 cellular organisms;Eukaryota;Opisthokonta;Metazoa;Eumetazoa;Bilateria;Protostomia;Spiralia;Lophotrochozoa;Brachiopoda;Linguliformea;Lingulata;Lingulida;Linguloidea;Lingulidae;Lingula;Lingula anatina
UdeM00000002044 7574 118.6 UdeM00000002044 XP_013409495.1 40.8 142 84 0 15 156 879 1020 7.00E-24 118.6 7574 cellular organisms;Eukaryota;Opisthokonta;Metazoa;Eumetazoa;Bilateria;Protostomia;Spiralia;Lophotrochozoa;Brachiopoda;Linguliformea;Lingulata;Lingulida;Linguloidea;Lingulidae;Lingula;Lingula anatina
UdeM00000002045 1735272 413.3 UdeM00000002045 XP_041370390.1 61.7 347 128 3 96 439 1465 1809 3.90E-112 413.3 1735272 cellular organisms;Eukaryota;Opisthokonta;Metazoa;Eumetazoa;Bilateria;Protostomia;Spiralia;Lophotrochozoa;Mollusca;Gastropoda;Neomphalina;Neomphaloidea;Peltospiridae;Gigantopelta;Gigantopelta aegis
UdeM00000002046 70779 85.9 UdeM00000002046 XP_015752579.1 45.8 83 42 1 47 126 18 100 4.40E-14 85.9 70779 cellular organisms;Eukaryota;Opisthokonta;Metazoa;Eumetazoa;Cnidaria;Anthozoa;Hexacorallia;Scleractinia;Astrocoeniina;Acroporidae;Acropora;Acropora digitifera
UdeM00000002047 29159 82 UdeM00000002047 XP_011425447.2 48.6 72 36 1 13 84 158 228 4.20E-13 82 29159 cellular organisms;Eukaryota;Opisthokonta;Metazoa;Eumetazoa;Bilateria;Protostomia;Spiralia;Lophotrochozoa;Mollusca;Bivalvia;Autobranchia;Pteriomorphia;Ostreida;Ostreoidea;Ostreidae;Crassostrea;Crassostrea gigas
UdeM00000002048 400682 201.1 UdeM00000002048 XP_019849765.1 47.8 184 83 3 71 243 533 714 1.70E-48 201.1 400682 cellular organisms;Eukaryota;Opisthokonta;Metazoa;Porifera;Demospongiae;Heteroscleromorpha;Haplosclerida;Niphatidae;Amphimedon;Amphimedon queenslandica
UdeM00000002049 400682 177.6 UdeM00000002049 XP_019849765.1 44.4 178 87 2 535 702 537 712 5.80E-41 177.6 400682 cellular organisms;Eukaryota;Opisthokonta;Metazoa;Porifera;Demospongiae;Heteroscleromorpha;Haplosclerida;Niphatidae;Amphimedon;Amphimedon queenslandica
UdeM00000002050 48498 153.7 UdeM00000002050 XP_020611730.1 61.2 103 40 0 48 150 31 133 2.00E-34 153.7 48498 cellular organisms;Eukaryota;Opisthokonta;Metazoa;Eumetazoa;Cnidaria;Anthozoa;Hexacorallia;Scleractinia;Faviina;Merulinidae;Orbicella;Orbicella faveolata
UdeM00000002052 7725 149.4 UdeM00000002052 XP_039258617.1 53.5 127 55 2 6 128 174 300 3.10E-33 149.4 7725 cellular organisms;Eukaryota;Opisthokonta;Metazoa;Eumetazoa;Bilateria;Deuterostomia;Chordata;Tunicata;Ascidiacea;Stolidobranchia;Styelidae;Styela;Styela clava

UdeM00000002051 146911 183.7 UdeM00000002051 XP_015282965.1 36.4
297 146 11 12 279 7 289 3.30E-43 183.7 146911
cellular
organisms;Eukaryota;Opisthokonta;Metazoa;Eumetazoa;Bilateria;Deuterostomia;Chordata;Craniata;Vertebrata;Gnathostomata;Teleostomi;Euteleostomi;Sarcopterygii;Dipnotetrapodomorpha;Tetrapoda;Amniota;Sauropsida;Sauria;Lepidosauria;Squamata;Bifurcata;Gekkota;Gekkonidae;Gekkoninae;Gekko;Gekko japonicus
UdeM00000002053 51298 90.5 UdeM00000002053 XP_036178578.1 55.4 74
32 1 1 73 399 472 9.90E-16 90.5 51298 cellular
organisms;Eukaryota;Opisthokonta;Metazoa;Eumetazoa;Bilateria;Deuterostomia;Chordata;Craniata;Vertebrata;Gnathostomata;Teleostomi;Euteleostomi;Sarcopterygii;Dipnotetrapodomorpha;Tetrapoda;Amniota;Mammalia;Theria;Eutheria;Boreoeutheria;Laurasiatheria;Chiroptera;Microchiroptera;Vespertilionidae;Myotis;Myotis myotis
UdeM00000002055 6573 456.8 UdeM00000002055 XP_021358892.1 32.5 822
532 12 124 926 205 1022 7.20E-125 456.8 6573 cellular
organisms;Eukaryota;Opisthokonta;Metazoa;Eumetazoa;Bilateria;Protostomia;Spiralia;Lophotrochozoa;Mollusca;Bivalvia;Autobranchia;Pteriomorphia;Pectinida;Pectinoidea;Pectinidae;Mizuhopecten;Mizuhopecten yessoensis
UdeM00000002056 400727 267.7 UdeM00000002056 XP_025094638.1 32.6
576 304 13 118 615 258 827 4.90E-68 267.7 400727
cellular
organisms;Eukaryota;Opisthokonta;Metazoa;Eumetazoa;Bilateria;Protostomia;Spiralia;Lophotrochozoa;Mollusca;Gastropoda;Caenogastropoda;Architaenioglossa;Ampullarioidea;Ampullariidae;Pomacea;Pomacea canaliculata
UdeM00000002056 400727 196.1 UdeM00000002056 XP_025094638.1 50.5
200 94 3 623 820 2196 2392 1.80E-46 196.1 400727
cellular
organisms;Eukaryota;Opisthokonta;Metazoa;Eumetazoa;Bilateria;Protostomia;Spiralia;Lophotrochozoa;Mollusca;Gastropoda;Caenogastropoda;Architaenioglossa;Ampullarioidea;Ampullariidae;Pomacea;Pomacea canaliculata
UdeM00000002058 6565 771.9 UdeM00000002058 XP_022335825.1 34 1367
851 23 395 1743 271 1604 1.70E-219 771.9 6565 cellular
organisms;Eukaryota;Opisthokonta;Metazoa;Eumetazoa;Bilateria;Protostomia;Spiralia;Lophotrochozoa;Mollusca;Bivalvia;Autobranchia;Pteriomorphia;Ostreida;Ostreoidea;Ostreidae;Crassostrea;Crassostrea virginica
UdeM00000002059 6565 762.7 UdeM00000002059 XP_022335825.1 34.1 1339
834 21 412 1733 298 1604 1.00E-216 762.7 6565 cellular
organisms;Eukaryota;Opisthokonta;Metazoa;Eumetazoa;Bilateria;Protostomia;Spiralia;Lophotrochozoa;Mollusca;Bivalvia;Autobranchia;Pteriomorphia;Ostreida;Ostreoidea;Ostreidae;Crassostrea;Crassostrea virginica
UdeM00000002060 6573 216.9 UdeM00000002060 XP_021358892.1 30.4 477
238 11 1 386 1301 1774 4.70E-53 216.9 6573 cellular
organisms;Eukaryota;Opisthokonta;Metazoa;Eumetazoa;Bilateria;Protostomia;Spiralia;Lophotrochozoa;Mollusca;Bivalvia;Autobranchia;Pteriomorphia;Pectinida;Pectinoidea;Pectinidae;Mizuhopecten;Mizuhopecten yessoensis
UdeM00000002061 6573 215.3 UdeM00000002061 XP_021358892.1 30.5 472
234 11 1 381 1306 1774 1.40E-52 215.3 6573 cellular
organisms;Eukaryota;Opisthokonta;Metazoa;Eumetazoa;Bilateria;Protostomia;Spiralia;Lophotrochozoa;Mollusca;Bivalvia;Autobranchia;Pteriomorphia;Pectinida;Pectinoidea;Pectinidae;Mizuhopecten;Mizuhopecten yessoensis
UdeM00000002062 6596 103.6 UdeM00000002062 XP_045190676.1 45.8 131
69 2 1 130 643 772 2.20E-19 103.6 6596 cellular

organisms;Eukaryota;Opisthokonta;Metazoa;Eumetazoa;Bilateria;Protostomia;Spiralia;Lophotrochozoa;Mollusca;Bivalvia;Autobranchia;Heteroconchia;Euheterodonta;Imparidentia;Venerida;Veneroidea;Veneridae;Mercenaria;Mercenaria mercenaria
UdeM00000002063 6596 444.1 UdeM00000002063 XP_045190678.1 43.4 539 290 8 1 527 601 1136 2.40E-121 444.1 6596 cellular organisms;Eukaryota;Opisthokonta;Metazoa;Eumetazoa;Bilateria;Protostomia;Spiralia;Lophotrochozoa;Mollusca;Bivalvia;Autobranchia;Heteroconchia;Euheterodonta;Imparidentia;Venerida;Veneroidea;Veneridae;Mercenaria;Mercenaria mercenaria
UdeM00000002064 6596 184.5 UdeM00000002064 XP_045202545.1 49 202 77 5 10 186 10 210 1.20E-43 184.5 6596 cellular organisms;Eukaryota;Opisthokonta;Metazoa;Eumetazoa;Bilateria;Protostomia;Spiralia;Lophotrochozoa;Mollusca;Bivalvia;Autobranchia;Heteroconchia;Euheterodonta;Imparidentia;Venerida;Veneroidea;Veneridae;Mercenaria;Mercenaria mercenaria
UdeM00000002065 6596 225.3 UdeM00000002065 XP_045188256.1 34.5 438 178 9 8 355 122 540 1.60E-55 225.3 6596 cellular organisms;Eukaryota;Opisthokonta;Metazoa;Eumetazoa;Bilateria;Protostomia;Spiralia;Lophotrochozoa;Mollusca;Bivalvia;Autobranchia;Heteroconchia;Euheterodonta;Imparidentia;Venerida;Veneroidea;Veneridae;Mercenaria;Mercenaria mercenaria
UdeM00000002066 7897 100.5 UdeM00000002066 XP_014349537.1 45.3 106 57 1 7 112 32 136 1.40E-18 100.5 7897 cellular organisms;Eukaryota;Opisthokonta;Metazoa;Eumetazoa;Bilateria;Deuterostomia;Chordata;Craniata;Vertebrata;Gnathostomata;Teleostomi;Euteleostomi;Sarcopterygii;Coelacanthimorpha;Coelacanthiformes;Coelacanthidae;Latimeria;Latimeria chalumnae
UdeM00000002067 6596 542.7 UdeM00000002067 XP_045188256.1 51.3 546 239 8 3 540 78 604 6.80E-151 542.7 6596 cellular organisms;Eukaryota;Opisthokonta;Metazoa;Eumetazoa;Bilateria;Protostomia;Spiralia;Lophotrochozoa;Mollusca;Bivalvia;Autobranchia;Heteroconchia;Euheterodonta;Imparidentia;Venerida;Veneroidea;Veneridae;Mercenaria;Mercenaria mercenaria
UdeM00000002070 6565 70.1 UdeM00000002070 XP_022337731.1 47 83 43 1 56 138 128 209 2.50E-09 70.1 6565 cellular organisms;Eukaryota;Opisthokonta;Metazoa;Eumetazoa;Bilateria;Protostomia;Spiralia;Lophotrochozoa;Mollusca;Bivalvia;Autobranchia;Pteriomorphia;Ostreida;Ostreoidea;Ostreidae;Crassostrea;Crassostrea virginica
UdeM00000002072 6596 263.8 UdeM00000002072 XP_045190673.1 40.9 413 215 9 475 877 408 801 7.60E-67 263.8 6596 cellular organisms;Eukaryota;Opisthokonta;Metazoa;Eumetazoa;Bilateria;Protostomia;Spiralia;Lophotrochozoa;Mollusca;Bivalvia;Autobranchia;Heteroconchia;Euheterodonta;Imparidentia;Venerida;Veneroidea;Veneridae;Mercenaria;Mercenaria mercenaria
UdeM00000002073 6565 726.1 UdeM00000002073 XP_022339595.1 33.2 1300 771 21 14 1235 607 1886 7.70E-206 726.1 6565 cellular organisms;Eukaryota;Opisthokonta;Metazoa;Eumetazoa;Bilateria;Protostomia;Spiralia;Lophotrochozoa;Mollusca;Bivalvia;Autobranchia;Pteriomorphia;Ostreida;Ostreoidea;Ostreidae;Crassostrea;Crassostrea virginica
UdeM00000002074 6596 510 UdeM00000002074 XP_045190678.1 46.1 601 306 9 18 610 546 1136 4.20E-141 510 6596 cellular organisms;Eukaryota;Opisthokonta;Metazoa;Eumetazoa;Bilateria;Protostomia;Spiralia;Lophotrochozoa;Mollusca;Bivalvia;Autobranchia;Heteroconchia;Euhe

terodonta;Imparidentia;Venerida;Veneroidea;Veneridae;Mercenaria;Mercenaria mercenaria
UdeM00000002076 7668 95.5 UdeM00000002076 XP_030845845.1 54.1 74
34 0 1 74 168 241 4.00E-17 95.5 7668 cellular organisms;Eukaryota;Opisthokonta;Metazoa;Eumetazoa;Bilateria;Deuterostomia;Echinodermata;Eleutherozoa;Echinozoa;Echinoidea;Euechinoidea;Echinacea;Camarodonta;Echinidea;Strongylocentrotidae;Strongylocentrotus;Strongylocentrotus purpuratus
UdeM00000002077 6596 520.8 UdeM00000002077 XP_045190515.1 30.1 1420
801 45 17 1382 690 1972 5.40E-144 520.8 6596 cellular organisms;Eukaryota;Opisthokonta;Metazoa;Eumetazoa;Bilateria;Protostomia;Spiralia;Lophotrochozoa;Mollusca;Bivalvia;Autobranchia;Heteroconchia;Euheterodonta;Imparidentia;Venerida;Veneroidea;Veneridae;Mercenaria;Mercenaria mercenaria
UdeM00000002078 7574 116.3 UdeM00000002078 XP_013388573.1 39.6 182
92 6 82 262 349 513 6.60E-23 116.3 7574 cellular organisms;Eukaryota;Opisthokonta;Metazoa;Eumetazoa;Bilateria;Protostomia;Spiralia;Lophotrochozoa;Brachiopoda;Linguliformea;Lingulata;Lingulida;Linguloidea;Lingulidae;Lingula;Lingula anatina
UdeM00000002079 7574 226.1 UdeM00000002079 XP_013388573.1 40.4 314
184 2 4 317 629 939 6.40E-56 226.1 7574 cellular organisms;Eukaryota;Opisthokonta;Metazoa;Eumetazoa;Bilateria;Protostomia;Spiralia;Lophotrochozoa;Brachiopoda;Linguliformea;Lingulata;Lingulida;Linguloidea;Lingulidae;Lingula;Lingula anatina
UdeM00000002080 6596 436.8 UdeM00000002080 XP_045188729.1 32.5 947
534 26 245 1168 870 1734 8.70E-119 436.8 6596 cellular organisms;Eukaryota;Opisthokonta;Metazoa;Eumetazoa;Bilateria;Protostomia;Spiralia;Lophotrochozoa;Mollusca;Bivalvia;Autobranchia;Heteroconchia;Euheterodonta;Imparidentia;Venerida;Veneroidea;Veneridae;Mercenaria;Mercenaria mercenaria
UdeM00000002083 1735272 87 UdeM00000002083 XP_041347204.1 33.8
160 89 7 1 157 346 491 2.40E-14 87 1735272
cellular organisms;Eukaryota;Opisthokonta;Metazoa;Eumetazoa;Bilateria;Protostomia;Spiralia;Lophotrochozoa;Mollusca;Gastropoda;Neomphalina;Neomphaloidea;Peltospiridae;Gigantopelta;Gigantopelta aegis
UdeM00000002082 1735272 135.6 UdeM00000002082 XP_041347204.1 31.7
300 159 12 2 270 207 491 9.90E-29 135.6 1735272
cellular organisms;Eukaryota;Opisthokonta;Metazoa;Eumetazoa;Bilateria;Protostomia;Spiralia;Lophotrochozoa;Mollusca;Gastropoda;Neomphalina;Neomphaloidea;Peltospiridae;Gigantopelta;Gigantopelta aegis
UdeM00000002084 6945 62.4 UdeM00000002084 XP_040078187.1 28.7 122
86 1 51 171 33 154 2.00E-06 62.4 6945 cellular organisms;Eukaryota;Opisthokonta;Metazoa;Eumetazoa;Bilateria;Protostomia;Ecdysozoa;Panarthropoda;Arthropoda;Chelicerata;Arachnida;Acari;Parasitiformes;Ixodida;Ixodoidea;Ixodidae;Ixodinae;Ixodes;Ixodes scapularis
UdeM00000002085 6945 62.4 UdeM00000002085 XP_040078187.1 28.7 122
86 1 51 171 33 154 2.00E-06 62.4 6945 cellular organisms;Eukaryota;Opisthokonta;Metazoa;Eumetazoa;Bilateria;Protostomia;Ecdysozoa;Panarthropoda;Arthropoda;Chelicerata;Arachnida;Acari;Parasitiformes;Ixodida;Ixodoidea;Ixodidae;Ixodinae;Ixodes;Ixodes scapularis
UdeM00000043667 6596 69.3 UdeM00000043667 XP_045183837.1 39.4 104
58 2 46 145 239 341 8.00E-09 69.3 6596 cellular

organisms;Eukaryota;Opisthokonta;Metazoa;Eumetazoa;Bilateria;Protostomia;Spiralia;Lophotrochozoa;Mollusca;Bivalvia;Autobranchia;Heteroconchia;Euheterodonta;Imparidentia;Venerida;Veneroidea;Veneridae;Mercenaria;Mercenaria mercenaria
UdeM00000043670 6596 70.1 UdeM00000043670 XP_045183837.1 38.5 104 59 2 46 145 239 341 3.50E-09 70.1 6596 cellular organisms;Eukaryota;Opisthokonta;Metazoa;Eumetazoa;Bilateria;Protostomia;Spiralia;Lophotrochozoa;Mollusca;Bivalvia;Autobranchia;Heteroconchia;Euheterodonta;Imparidentia;Venerida;Veneroidea;Veneridae;Mercenaria;Mercenaria mercenaria
UdeM00000030989 6596 364.4 UdeM00000030989 XP_045161155.1 49.4 336 167 1 31 363 123 458 1.70E-97 364.4 6596 cellular organisms;Eukaryota;Opisthokonta;Metazoa;Eumetazoa;Bilateria;Protostomia;Spiralia;Lophotrochozoa;Mollusca;Bivalvia;Autobranchia;Heteroconchia;Euheterodonta;Imparidentia;Venerida;Veneroidea;Veneridae;Mercenaria;Mercenaria mercenaria
UdeM00000030990 6596 170.2 UdeM00000030990 XP_045163223.1 72.6 113 27 1 1 113 1 109 1.50E-39 170.2 6596 cellular organisms;Eukaryota;Opisthokonta;Metazoa;Eumetazoa;Bilateria;Protostomia;Spiralia;Lophotrochozoa;Mollusca;Bivalvia;Autobranchia;Heteroconchia;Euheterodonta;Imparidentia;Venerida;Veneroidea;Veneridae;Mercenaria;Mercenaria mercenaria
UdeM00000030991 6596 69.7 UdeM00000030991 XP_045162944.1 47.6 82 27 3 159 231 141 215 5.50E-09 69.7 6596 cellular organisms;Eukaryota;Opisthokonta;Metazoa;Eumetazoa;Bilateria;Protostomia;Spiralia;Lophotrochozoa;Mollusca;Bivalvia;Autobranchia;Heteroconchia;Euheterodonta;Imparidentia;Venerida;Veneroidea;Veneridae;Mercenaria;Mercenaria mercenaria
UdeM00000030992 6596 1066.2 UdeM00000030992 XP_045163002.1 53 972 439 10 17 983 17 975 2.40E-308 1066.2 6596 cellular organisms;Eukaryota;Opisthokonta;Metazoa;Eumetazoa;Bilateria;Protostomia;Spiralia;Lophotrochozoa;Mollusca;Bivalvia;Autobranchia;Heteroconchia;Euheterodonta;Imparidentia;Venerida;Veneroidea;Veneridae;Mercenaria;Mercenaria mercenaria
UdeM00000030993 6579 135.6 UdeM00000030993 XP_033763141.1 42.6 195 108 2 14 205 10 203 7.50E-29 135.6 6579 cellular organisms;Eukaryota;Opisthokonta;Metazoa;Eumetazoa;Bilateria;Protostomia;Spiralia;Lophotrochozoa;Mollusca;Bivalvia;Autobranchia;Pteriomorphia;Pectinida;Pectinoidea;Pectinidae;Pecten;Pecten maximus
UdeM00000030994 6596 204.1 UdeM00000030994 XP_045173066.1 35.9 312 189 7 44 348 46 353 2.80E-49 204.1 6596 cellular organisms;Eukaryota;Opisthokonta;Metazoa;Eumetazoa;Bilateria;Protostomia;Spiralia;Lophotrochozoa;Mollusca;Bivalvia;Autobranchia;Heteroconchia;Euheterodonta;Imparidentia;Venerida;Veneroidea;Veneridae;Mercenaria;Mercenaria mercenaria
UdeM00000030995 6579 1055.4 UdeM00000030995 XP_033763055.1 51 1224 493 28 1 1148 1 1193 5.00E-305 1055.4 6579 cellular organisms;Eukaryota;Opisthokonta;Metazoa;Eumetazoa;Bilateria;Protostomia;Spiralia;Lophotrochozoa;Mollusca;Bivalvia;Autobranchia;Pteriomorphia;Pectinida;Pectinoidea;Pectinidae;Pecten;Pecten maximus
UdeM00000030996 6573 318.5 UdeM00000030996 XP_021366944.1 51.7 294 137 4 8 297 7 299 8.80E-84 318.5 6573 cellular

organisms;Eukaryota;Opisthokonta;Metazoa;Eumetazoa;Bilateria;Protostomia;
Spiralia;Lophotrochozoa;Mollusca;Bivalvia;Autobranchia;Pteriomorphia;Pect
inida;Pectinoidea;Pectinidae;Mizuhopecten;Mizuhopecten yessoensis
UdeM00000030997 6596 682.2 UdeM00000030997 XP_045164027.1 53.8 613
271 5 45 651 27 633 6.60E-193 682.2 6596 cellular
organisms;Eukaryota;Opisthokonta;Metazoa;Eumetazoa;Bilateria;Protostomia;
Spiralia;Lophotrochozoa;Mollusca;Bivalvia;Autobranchia;Heteroconchia;Euhe
terodonta;Imparidentia;Venerida;Veneroidea;Veneridae;Mercenaria;Mercenari
a mercenaria
UdeM00000030999 7719 197.6 UdeM00000030999 XP_004227365.1 52.8 161
76 0 4 164 2 162 1.20E-47 197.6 7719 cellular
organisms;Eukaryota;Opisthokonta;Metazoa;Eumetazoa;Bilateria;Deuterostomi
a;Chordata;Tunicata;Ascidiacea;Phlebobranchia;Cionidae;Ciona;Ciona
intestinalis
UdeM00000031000 7719 197.6 UdeM00000031000 XP_004227365.1 52.8 161
76 0 6 166 2 162 1.30E-47 197.6 7719 cellular
organisms;Eukaryota;Opisthokonta;Metazoa;Eumetazoa;Bilateria;Deuterostomi
a;Chordata;Tunicata;Ascidiacea;Phlebobranchia;Cionidae;Ciona;Ciona
intestinalis
UdeM00000031001 6596 463.4 UdeM00000031001 XP_045200982.1 70.2 299
87 1 4 300 6 304 2.80E-127 463.4 6596 cellular
organisms;Eukaryota;Opisthokonta;Metazoa;Eumetazoa;Bilateria;Protostomia;
Spiralia;Lophotrochozoa;Mollusca;Bivalvia;Autobranchia;Heteroconchia;Euhe
terodonta;Imparidentia;Venerida;Veneroidea;Veneridae;Mercenaria;Mercenari
a mercenaria
UdeM00000031002 6596 901.4 UdeM00000031002 XP_045200982.1 49.6 932
460 7 4 930 6 932 1.00E-258 901.4 6596 cellular
organisms;Eukaryota;Opisthokonta;Metazoa;Eumetazoa;Bilateria;Protostomia;
Spiralia;Lophotrochozoa;Mollusca;Bivalvia;Autobranchia;Heteroconchia;Euhe
terodonta;Imparidentia;Venerida;Veneroidea;Veneridae;Mercenaria;Mercenari
a mercenaria
UdeM00000031003 6579 80.5 UdeM00000031003 XP_033754238.1 63.2 57
21 0 59 115 72 128 1.60E-12 80.5 6579 cellular
organisms;Eukaryota;Opisthokonta;Metazoa;Eumetazoa;Bilateria;Protostomia;
Spiralia;Lophotrochozoa;Mollusca;Bivalvia;Autobranchia;Pteriomorphia;Pect
inida;Pectinoidea;Pectinidae;Pecten;Pecten maximus
UdeM00000031004 29159 84 UdeM00000031004 XP_034326245.1 25.8 236
161 4 262 484 32 266 6.20E-13 84 29159 cellular
organisms;Eukaryota;Opisthokonta;Metazoa;Eumetazoa;Bilateria;Protostomia;
Spiralia;Lophotrochozoa;Mollusca;Bivalvia;Autobranchia;Pteriomorphia;Ostr
eida;Ostreoidea;Ostreidae;Crassostrea;Crassostrea gigas
UdeM00000031005 6565 123.6 UdeM00000031005 XP_022329668.1 32 375
208 8 14 347 7 375 5.00E-25 123.6 6565 cellular
organisms;Eukaryota;Opisthokonta;Metazoa;Eumetazoa;Bilateria;Protostomia;
Spiralia;Lophotrochozoa;Mollusca;Bivalvia;Autobranchia;Pteriomorphia;Ostr
eida;Ostreoidea;Ostreidae;Crassostrea;Crassostrea virginica
UdeM00000031006 6565 123.6 UdeM00000031006 XP_022329668.1 32.5 375
205 9 14 346 7 375 5.00E-25 123.6 6565 cellular
organisms;Eukaryota;Opisthokonta;Metazoa;Eumetazoa;Bilateria;Protostomia;
Spiralia;Lophotrochozoa;Mollusca;Bivalvia;Autobranchia;Pteriomorphia;Ostr
eida;Ostreoidea;Ostreidae;Crassostrea;Crassostrea virginica
UdeM00000031007 218467 103.2 UdeM00000031007 XP_023212519.1 53.6
97 44 1 1 97 1 96 1.90E-19 103.2 218467
cellular

organisms;Eukaryota;Opisthokonta;Metazoa;Eumetazoa;Bilateria;Protostomia;Ecdysozoa;Panarthropoda;Arthropoda;Chelicerata;Arachnida;Scorpiones;Buthida;Buthoidea;Buthidae;Centruroides;Centruroides sculpturatus
UdeM00000031008 1529436 442.6 UdeM00000031008 XP_033120141.1 58.1 389 152 5 41 427 1154 1533 5.80E-121 442.6 1529436 cellular organisms;Eukaryota;Opisthokonta;Metazoa;Eumetazoa;Bilateria;Deuterostomia;Echinodermata;Pelmatozoa;Crinoidea;Articulata;Comatulida;Comatulidae;Comatulinae;Anneissia;Anneissia japonica
UdeM00000031009 6573 102.1 UdeM00000031009 XP_021365717.1 32.4 173 111 6 28 198 182 350 8.80E-19 102.1 6573 cellular organisms;Eukaryota;Opisthokonta;Metazoa;Eumetazoa;Bilateria;Protostomia;Spiralia;Lophotrochozoa;Mollusca;Bivalvia;Autobranchia;Pteriomorphia;Pectinida;Pectinoidea;Pectinidae;Mizuhopecten;Mizuhopecten yessoensis
UdeM00000031010 41139 126.3 UdeM00000031010 XP_044759447.1 31.4 277 180 6 92 361 13 286 8.00E-26 126.3 41139 cellular organisms;Eukaryota;Opisthokonta;Metazoa;Eumetazoa;Bilateria;Protostomia;Ecdysozoa;Panarthropoda;Arthropoda;Mandibulata;Pancrustacea;Hexapoda;Insecta;Dicondylia;Pterygota;Neoptera;Endopterygota;Coleoptera;Polyphaga;Cucujiformia;Cucujoidea;Coccinellidae;Coccinellinae;Coccinellini;Coccinella;Coccinella septempunctata
UdeM00000031014 6596 599.4 UdeM00000031014 XP_045162694.1 85 346 51 1 1 345 1 346 3.10E-168 599.4 6596 cellular organisms;Eukaryota;Opisthokonta;Metazoa;Eumetazoa;Bilateria;Protostomia;Spiralia;Lophotrochozoa;Mollusca;Bivalvia;Autobranchia;Heteroconchia;Euheterodonta;Imparidentia;Venerida;Veneroidea;Veneridae;Mercenaria;Mercenaria mercenaria
UdeM00000031015 6596 101.3 UdeM00000031015 XP_045181277.1 30.9 207 111 5 111 315 143 319 2.40E-18 101.3 6596 cellular organisms;Eukaryota;Opisthokonta;Metazoa;Eumetazoa;Bilateria;Protostomia;Spiralia;Lophotrochozoa;Mollusca;Bivalvia;Autobranchia;Heteroconchia;Euheterodonta;Imparidentia;Venerida;Veneroidea;Veneridae;Mercenaria;Mercenaria mercenaria
UdeM00000031016 6596 112.5 UdeM00000031016 XP_045176094.1 40.9 154 82 6 62 211 119 267 6.90E-22 112.5 6596 cellular organisms;Eukaryota;Opisthokonta;Metazoa;Eumetazoa;Bilateria;Protostomia;Spiralia;Lophotrochozoa;Mollusca;Bivalvia;Autobranchia;Heteroconchia;Euheterodonta;Imparidentia;Venerida;Veneroidea;Veneridae;Mercenaria;Mercenaria mercenaria
UdeM00000031017 6596 580.9 UdeM00000031017 XP_045199719.1 42.2 733 406 11 3 727 67 789 2.80E-162 580.9 6596 cellular organisms;Eukaryota;Opisthokonta;Metazoa;Eumetazoa;Bilateria;Protostomia;Spiralia;Lophotrochozoa;Mollusca;Bivalvia;Autobranchia;Heteroconchia;Euheterodonta;Imparidentia;Venerida;Veneroidea;Veneridae;Mercenaria;Mercenaria mercenaria
UdeM00000031018 6579 227.3 UdeM00000031018 XP_033729693.1 54.4 193 85 3 1 190 68 260 2.00E-56 227.3 6579 cellular organisms;Eukaryota;Opisthokonta;Metazoa;Eumetazoa;Bilateria;Protostomia;Spiralia;Lophotrochozoa;Mollusca;Bivalvia;Autobranchia;Pteriomorphia;Pectinida;Pectinoidea;Pectinidae;Pecten;Pecten maximus
UdeM00000031019 6596 229.2 UdeM00000031019 XP_045172944.1 49.8 263 106 5 1 257 1 243 6.00E-57 229.2 6596 cellular organisms;Eukaryota;Opisthokonta;Metazoa;Eumetazoa;Bilateria;Protostomia;Spiralia;Lophotrochozoa;Mollusca;Bivalvia;Autobranchia;Heteroconchia;Euhe

terodonta;Imparidentia;Venerida;Veneroidea;Veneridae;Mercenaria;Mercenaria mercenaria
UdeM00000031020 50429 168.3 UdeM00000031020 XP_022802642.1 75.8 99 24 0 1 99 1 99 8.10E-39 168.3 50429 cellular organisms;Eukaryota;Opisthokonta;Metazoa;Eumetazoa;Cnidaria;Anthozoa;Hexacorallia;Scleractinia;Astrocoeniina;Pocilloporidae;Stylophora;Stylophora pistillata
UdeM00000031021 6596 627.1 UdeM00000031021 XP_045199719.1 41.7 809 451 14 25 824 24 820 3.20E-176 627.1 6596 cellular organisms;Eukaryota;Opisthokonta;Metazoa;Eumetazoa;Bilateria;Protostomia;Spiralia;Lophotrochozoa;Mollusca;Bivalvia;Autobranchia;Heteroconchia;Euheterodonta;Imparidentia;Venerida;Veneroidea;Veneridae;Mercenaria;Mercenaria mercenaria
UdeM00000031023 6596 543.5 UdeM00000031023 XP_045199719.1 39.9 737 432 9 21 753 28 757 4.30E-151 543.5 6596 cellular organisms;Eukaryota;Opisthokonta;Metazoa;Eumetazoa;Bilateria;Protostomia;Spiralia;Lophotrochozoa;Mollusca;Bivalvia;Autobranchia;Heteroconchia;Euheterodonta;Imparidentia;Venerida;Veneroidea;Veneridae;Mercenaria;Mercenaria mercenaria
UdeM00000031024 6596 668.3 UdeM00000031024 XP_045199719.1 42 833 450 11 25 845 24 835 1.30E-188 668.3 6596 cellular organisms;Eukaryota;Opisthokonta;Metazoa;Eumetazoa;Bilateria;Protostomia;Spiralia;Lophotrochozoa;Mollusca;Bivalvia;Autobranchia;Heteroconchia;Euheterodonta;Imparidentia;Venerida;Veneroidea;Veneridae;Mercenaria;Mercenaria mercenaria
UdeM00000031025 116150 114.4 UdeM00000031025 XP_039753516.1 41.1 163 82 3 16 171 26 181 1.90E-22 114.4 116150 cellular organisms;Eukaryota;Opisthokonta;Metazoa;Eumetazoa;Bilateria;Protostomia;Ecdysozoa;Panarthropoda;Arthropoda;Mandibulata;Pancrustacea;Hexapoda;Insecta;Dicondylia;Pterygota;Neoptera;Endopterygota;Amphiesmenoptera;Lepidoptera;Glossata;Neolepidoptera;Heteroneura;Ditrysia;Obtectomera;Papilionoidea;Nymphalidae;Satyrinae;Satyrini;Parargina;Pararge;Pararge aegeria
UdeM00000031026 6579 117.9 UdeM00000031026 XP_033764248.1 39 182 85 4 15 192 19 178 1.70E-23 117.9 6579 cellular organisms;Eukaryota;Opisthokonta;Metazoa;Eumetazoa;Bilateria;Protostomia;Spiralia;Lophotrochozoa;Mollusca;Bivalvia;Autobranchia;Pteriomorphia;Pectinida;Pectinoidea;Pectinidae;Pecten;Pecten maximus
UdeM00000031027 6596 127.1 UdeM00000031027 XP_045163831.1 34.7 268 150 8 3 259 20 273 3.30E-26 127.1 6596 cellular organisms;Eukaryota;Opisthokonta;Metazoa;Eumetazoa;Bilateria;Protostomia;Spiralia;Lophotrochozoa;Mollusca;Bivalvia;Autobranchia;Heteroconchia;Euheterodonta;Imparidentia;Venerida;Veneroidea;Veneridae;Mercenaria;Mercenaria mercenaria
UdeM00000031028 6573 272.7 UdeM00000031028 XP_021366482.1 63.6 217 79 0 1 217 1 217 4.20E-70 272.7 6573 cellular organisms;Eukaryota;Opisthokonta;Metazoa;Eumetazoa;Bilateria;Protostomia;Spiralia;Lophotrochozoa;Mollusca;Bivalvia;Autobranchia;Pteriomorphia;Pectinida;Pectinoidea;Pectinidae;Mizuhopecten;Mizuhopecten yessoensis
UdeM00000031029 6500 180.6 UdeM00000031029 XP_005103007.1 61.2 129 50 0 44 172 40 168 1.80E-42 180.6 6500 cellular organisms;Eukaryota;Opisthokonta;Metazoa;Eumetazoa;Bilateria;Protostomia;Spiralia;Lophotrochozoa;Mollusca;Gastropoda;Heterobranchia;Euthyneura;Tectipleura;Aplysiida;Aplysioidea;Aplysiidae;Aplysia;Aplysia californica

UdeM00000031030 29159 177.6 UdeM00000031030 XP_011427968.2 55.9 152
63 3 1 148 1 152 1.20E-41 177.6 29159 cellular
organisms;Eukaryota;Opisthokonta;Metazoa;Eumetazoa;Bilateria;Protostomia;Spiralia;Lophotrochozoa;Mollusca;Bivalvia;Autobranchia;Pteriomorphia;Ostreida;Ostreoidea;Ostreidae;Crassostrea;Crassostrea gigas
UdeM00000031032 9371 59.7 UdeM00000031032 XP_045155162.1 49 51
24 1 273 321 124 174 7.90E-06 59.7 9371 cellular
organisms;Eukaryota;Opisthokonta;Metazoa;Eumetazoa;Bilateria;Deuterostomia;Chordata;Craniata;Vertebrata;Gnathostomata;Teleostomi;Euteleostomi;Sarcopterygii;Dipnotetrapodomorpha;Tetrapoda;Amniota;Mammalia;Theria;Eutheria;Afrotheria;Tenrecidae;Tenrecinae;Echinops;Echinops telfairi
UdeM00000031033 9371 59.7 UdeM00000031033 XP_045155162.1 49 51
24 1 322 370 124 174 9.10E-06 59.7 9371 cellular
organisms;Eukaryota;Opisthokonta;Metazoa;Eumetazoa;Bilateria;Deuterostomia;Chordata;Craniata;Vertebrata;Gnathostomata;Teleostomi;Euteleostomi;Sarcopterygii;Dipnotetrapodomorpha;Tetrapoda;Amniota;Mammalia;Theria;Eutheria;Afrotheria;Tenrecidae;Tenrecinae;Echinops;Echinops telfairi
UdeM00000031034 6596 893.3 UdeM00000031034 XP_045161992.1 43.1 1147
576 20 18 1116 33 1150 3.30E-256 893.3 6596 cellular
organisms;Eukaryota;Opisthokonta;Metazoa;Eumetazoa;Bilateria;Protostomia;Spiralia;Lophotrochozoa;Mollusca;Bivalvia;Autobranchia;Heteroconchia;Euheterodonta;Imparidentia;Venerida;Veneroidea;Veneridae;Mercenaria;Mercenaria mercenaria
UdeM00000031035 6596 912.5 UdeM00000031035 XP_045161992.1 43.8 1149
592 21 18 1143 33 1150 5.40E-262 912.5 6596 cellular
organisms;Eukaryota;Opisthokonta;Metazoa;Eumetazoa;Bilateria;Protostomia;Spiralia;Lophotrochozoa;Mollusca;Bivalvia;Autobranchia;Heteroconchia;Euheterodonta;Imparidentia;Venerida;Veneroidea;Veneridae;Mercenaria;Mercenaria mercenaria
UdeM00000031036 6596 1176.4 UdeM00000031036 XP_045161096.1 52.9
1192 444 11 18 1106 1 1177 0.00E+00 1176.4 6596
cellular
organisms;Eukaryota;Opisthokonta;Metazoa;Eumetazoa;Bilateria;Protostomia;Spiralia;Lophotrochozoa;Mollusca;Bivalvia;Autobranchia;Heteroconchia;Euheterodonta;Imparidentia;Venerida;Veneroidea;Veneridae;Mercenaria;Mercenaria mercenaria
UdeM00000031037 6596 1170.2 UdeM00000031037 XP_045161096.1 52.8
1192 444 12 18 1105 1 1177 0.00E+00 1170.2 6596
cellular
organisms;Eukaryota;Opisthokonta;Metazoa;Eumetazoa;Bilateria;Protostomia;Spiralia;Lophotrochozoa;Mollusca;Bivalvia;Autobranchia;Heteroconchia;Euheterodonta;Imparidentia;Venerida;Veneroidea;Veneridae;Mercenaria;Mercenaria mercenaria
UdeM00000031038 1735272 406.8 UdeM00000031038 XP_041377672.1 48
508 205 9 76 577 77 531 4.80E-110 406.8 1735272
cellular
organisms;Eukaryota;Opisthokonta;Metazoa;Eumetazoa;Bilateria;Protostomia;Spiralia;Lophotrochozoa;Mollusca;Gastropoda;Neomphalina;Neomphaloidea;Peltospiridae;Gigantopelta;Gigantopelta aegis
UdeM00000031039 1735272 715.7 UdeM00000031039 XP_041377672.1 68.4
506 159 1 228 733 532 1036 6.20E-203 715.7 1735272
cellular
organisms;Eukaryota;Opisthokonta;Metazoa;Eumetazoa;Bilateria;Protostomia;

Spiralia;Lophotrochozoa;Mollusca;Gastropoda;Neomphalina;Neomphaloidea;Peltospiridae;Gigantopelta;Gigantopelta aegis
UdeM00000031040 94885 105.1 UdeM00000031040 XP_034274523.1 62.1 87 30 1 6 89 61 147 4.60E-20 105.1 94885 cellular organisms;Eukaryota;Opisthokonta;Metazoa;Eumetazoa;Bilateria;Deuterostomia;Chordata;Craniata;Vertebrata;Gnathostomata;Teleostomi;Euteleostomi;Sarcopterygii;Dipnotetrapodomorpha;Tetrapoda;Amniota;Sauropsida;Sauria;Lepidosauria;Squamata;Bifurcata;Unidentata;Episquamata;Toxicofera;Serpentes;Colubroidea;Colubridae;Colubrinae;Pantherophis;Pantherophis guttatus
UdeM00000031041 37653 439.5 UdeM00000031041 XP_014789344.1 37.3 684 413 7 3 676 4 681 7.80E-120 439.5 37653 cellular organisms;Eukaryota;Opisthokonta;Metazoa;Eumetazoa;Bilateria;Protostomia;Spiralia;Lophotrochozoa;Mollusca;Cephalopoda;Coleoidea;Octopodiformes;Octopoda;Incirrata;Octopodidae;Octopus;Octopus bimaculoides
UdeM00000033685 7574 131.3 UdeM00000033685 XP_013418753.1 63.9 97 33 1 156 250 66 162 1.70E-27 131.3 7574 cellular organisms;Eukaryota;Opisthokonta;Metazoa;Eumetazoa;Bilateria;Protostomia;Spiralia;Lophotrochozoa;Brachiopoda;Linguliformea;Lingulata;Lingulida;Linguloidea;Lingulidae;Lingula;Lingula anatina
UdeM00000033686 6579 1133.2 UdeM00000033686 XP_033727362.1 60.7 937 364 2 1 934 1 936 0.00E+00 1133.2 6579 cellular organisms;Eukaryota;Opisthokonta;Metazoa;Eumetazoa;Bilateria;Protostomia;Spiralia;Lophotrochozoa;Mollusca;Bivalvia;Autobranchia;Pteriomorphia;Pectinida;Pectinoidea;Pectinidae;Pecten;Pecten maximus
UdeM00000033687 29159 626.7 UdeM00000033687 XP_011446826.2 62.7 453 169 0 37 489 37 489 2.60E-176 626.7 29159 cellular organisms;Eukaryota;Opisthokonta;Metazoa;Eumetazoa;Bilateria;Protostomia;Spiralia;Lophotrochozoa;Mollusca;Bivalvia;Autobranchia;Pteriomorphia;Ostreida;Ostreoidea;Ostreidae;Crassostrea;Crassostrea gigas
UdeM00000033688 6596 561.6 UdeM00000033688 XP_045205741.1 52.8 631 270 4 2 631 29 632 1.30E-156 561.6 6596 cellular organisms;Eukaryota;Opisthokonta;Metazoa;Eumetazoa;Bilateria;Protostomia;Spiralia;Lophotrochozoa;Mollusca;Bivalvia;Autobranchia;Heteroconchia;Euheterodonta;Imparidentia;Venerida;Veneroidea;Veneridae;Mercenaria;Mercenaria mercenaria
UdeM00000033689 6565 341.3 UdeM00000033689 XP_022306197.1 53.8 320 104 2 98 373 363 682 1.60E-90 341.3 6565 cellular organisms;Eukaryota;Opisthokonta;Metazoa;Eumetazoa;Bilateria;Protostomia;Spiralia;Lophotrochozoa;Mollusca;Bivalvia;Autobranchia;Pteriomorphia;Ostreida;Ostreoidea;Ostreidae;Crassostrea;Crassostrea virginica
UdeM00000033690 6596 560.5 UdeM00000033690 XP_045205741.1 53.3 615 275 4 2 615 29 632 2.70E-156 560.5 6596 cellular organisms;Eukaryota;Opisthokonta;Metazoa;Eumetazoa;Bilateria;Protostomia;Spiralia;Lophotrochozoa;Mollusca;Bivalvia;Autobranchia;Heteroconchia;Euheterodonta;Imparidentia;Venerida;Veneroidea;Veneridae;Mercenaria;Mercenaria mercenaria
UdeM00000033691 6596 568.2 UdeM00000033691 XP_045205741.1 54.2 611 267 5 6 615 34 632 1.30E-158 568.2 6596 cellular organisms;Eukaryota;Opisthokonta;Metazoa;Eumetazoa;Bilateria;Protostomia;Spiralia;Lophotrochozoa;Mollusca;Bivalvia;Autobranchia;Heteroconchia;Euheterodonta;Imparidentia;Venerida;Veneroidea;Veneridae;Mercenaria;Mercenaria mercenaria

UdeM00000033692 6565 78.6 UdeM00000033692 XP_022323426.1 32.3 161
101 3 19 177 19 173 9.10E-12 78.6 6565 cellular
organisms;Eukaryota;Opisthokonta;Metazoa;Eumetazoa;Bilateria;Protostomia;
Spiralia;Lophotrochozoa;Mollusca;Bivalvia;Autobranchia;Pteriomorphia;Ostr
eida;Ostreoidea;Ostreidae;Crassostrea;Crassostrea virginica
UdeM00000033693 6565 89 UdeM00000033693 XP_022323426.1 31.8 173
116 2 1 171 1 173 6.50E-15 89 6565 cellular
organisms;Eukaryota;Opisthokonta;Metazoa;Eumetazoa;Bilateria;Protostomia;
Spiralia;Lophotrochozoa;Mollusca;Bivalvia;Autobranchia;Pteriomorphia;Ostr
eida;Ostreoidea;Ostreidae;Crassostrea;Crassostrea virginica
UdeM00000033694 6596 93.2 UdeM00000033694 XP_045182675.1 29.8 168
116 2 6 171 5 172 3.40E-16 93.2 6596 cellular
organisms;Eukaryota;Opisthokonta;Metazoa;Eumetazoa;Bilateria;Protostomia;
Spiralia;Lophotrochozoa;Mollusca;Bivalvia;Autobranchia;Heteroconchia;Euhe
terodonta;Imparidentia;Venerida;Veneroidea;Veneridae;Mercenaria;Mercenari
a mercenaria
UdeM00000033695 6596 213.4 UdeM00000033695 XP_045181502.1 96.4 112
4 0 1 112 1 112 1.60E-52 213.4 6596 cellular
organisms;Eukaryota;Opisthokonta;Metazoa;Eumetazoa;Bilateria;Protostomia;
Spiralia;Lophotrochozoa;Mollusca;Bivalvia;Autobranchia;Heteroconchia;Euhe
terodonta;Imparidentia;Venerida;Veneroidea;Veneridae;Mercenaria;Mercenari
a mercenaria
UdeM00000033696 29159 933.3 UdeM00000033696 XP_034323329.1 67.2 725
220 8 1 715 1 717 1.80E-268 933.3 29159 cellular
organisms;Eukaryota;Opisthokonta;Metazoa;Eumetazoa;Bilateria;Protostomia;
Spiralia;Lophotrochozoa;Mollusca;Bivalvia;Autobranchia;Pteriomorphia;Ostr
eida;Ostreoidea;Ostreidae;Crassostrea;Crassostrea gigas
UdeM00000033697 400727 145.6 UdeM00000033697 XP_025089755.1 41.4
181 102 1 15 195 17 193 7.00E-32 145.6 400727
cellular
organisms;Eukaryota;Opisthokonta;Metazoa;Eumetazoa;Bilateria;Protostomia;
Spiralia;Lophotrochozoa;Mollusca;Gastropoda;Caenogastropoda;Architaenioglossa;Ampullarioidea;Ampullariidae;Pomacea;Pomacea canaliculata
UdeM00000033699 125878 156 UdeM00000033699 XP_018411046.1 69.5
105 31 1 19 122 27 131 3.10E-35 156 125878
cellular
organisms;Eukaryota;Opisthokonta;Metazoa;Eumetazoa;Bilateria;Deuterostomi
a;Chordata;Craniata;Vertebrata;Gnathostomata;Teleostomi;Euteleostomi;Sarc
opterygii;Dipnotetrapodomorpha;Tetrapoda;Amphibia;Batrachia;Anura;Neobatr
achia;Ranoidea;Dicroglossidae;Dicroglossinae;Nanorana;Nanorana parkeri
UdeM00000033698 125878 156 UdeM00000033698 XP_018411046.1 69.5
105 31 1 42 145 27 131 3.70E-35 156 125878
cellular
organisms;Eukaryota;Opisthokonta;Metazoa;Eumetazoa;Bilateria;Deuterostomi
a;Chordata;Craniata;Vertebrata;Gnathostomata;Teleostomi;Euteleostomi;Sarc
opterygii;Dipnotetrapodomorpha;Tetrapoda;Amphibia;Batrachia;Anura;Neobatr
achia;Ranoidea;Dicroglossidae;Dicroglossinae;Nanorana;Nanorana parkeri
UdeM00000033700 7739 845.9 UdeM00000033700 XP_035659512.1 88.5 462
52 1 1 462 1 461 2.40E-242 845.9 7739 cellular
organisms;Eukaryota;Opisthokonta;Metazoa;Eumetazoa;Bilateria;Deuterostomi
a;Chordata;Cephalochordata;Leptocardii;Amphioxiformes;Branchiostomidae;Br
anchiostoma;Branchiostoma floridae
UdeM00000033702 6596 612.5 UdeM00000033702 XP_045161437.1 76.1 377
90 0 43 419 43 419 4.80E-172 612.5 6596 cellular

organisms;Eukaryota;Opisthokonta;Metazoa;Eumetazoa;Bilateria;Protostomia;Spiralia;Lophotrochozoa;Mollusca;Bivalvia;Autobranchia;Heteroconchia;Euheterodonta;Imparidentia;Venerida;Veneroidea;Veneridae;Mercenaria;Mercenaria mercenaria
UdeM00000033704 6573 439.1 UdeM00000033704 XP_021360119.1 43.1 552 297 7 14 550 3 552 8.20E-120 439.1 6573 cellular organisms;Eukaryota;Opisthokonta;Metazoa;Eumetazoa;Bilateria;Protostomia;Spiralia;Lophotrochozoa;Mollusca;Bivalvia;Autobranchia;Pteriomorphia;Pectinida;Pectinoidea;Pectinidae;Mizuhopecten;Mizuhopecten yessoensis
UdeM00000033705 6596 212.2 UdeM00000033705 XP_045181873.1 87.8 115 14 0 2 116 3 117 4.00E-52 212.2 6596 cellular organisms;Eukaryota;Opisthokonta;Metazoa;Eumetazoa;Bilateria;Protostomia;Spiralia;Lophotrochozoa;Mollusca;Bivalvia;Autobranchia;Heteroconchia;Euheterodonta;Imparidentia;Venerida;Veneroidea;Veneridae;Mercenaria;Mercenaria mercenaria
UdeM00000033706 6573 233.8 UdeM00000033706 XP_021365243.1 67.3 223 60 7 1 213 1 220 2.00E-58 233.8 6573 cellular organisms;Eukaryota;Opisthokonta;Metazoa;Eumetazoa;Bilateria;Protostomia;Spiralia;Lophotrochozoa;Mollusca;Bivalvia;Autobranchia;Pteriomorphia;Pectinida;Pectinoidea;Pectinidae;Mizuhopecten;Mizuhopecten yessoensis
UdeM00000033707 46514 157.1 UdeM00000033707 XP_038073561.1 51.5 134 65 0 2 135 17 150 1.50E-35 157.1 46514 cellular organisms;Eukaryota;Opisthokonta;Metazoa;Eumetazoa;Bilateria;Deuterostomia;Echinodermata;Eleutherozoa;Asterozoa;Asteroidea;Valvatacea;Valvatida;Asterinidae;Patiria;Patiria miniata
UdeM00000033708 6579 606.3 UdeM00000033708 XP_033739131.1 57.3 508 197 4 1 489 864 1370 3.50E-170 606.3 6579 cellular organisms;Eukaryota;Opisthokonta;Metazoa;Eumetazoa;Bilateria;Protostomia;Spiralia;Lophotrochozoa;Mollusca;Bivalvia;Autobranchia;Pteriomorphia;Pectinida;Pectinoidea;Pectinidae;Pecten;Pecten maximus
UdeM00000033710 6596 261.9 UdeM00000033710 XP_045182024.1 61.3 212 78 2 2 210 3 213 7.50E-67 261.9 6596 cellular organisms;Eukaryota;Opisthokonta;Metazoa;Eumetazoa;Bilateria;Protostomia;Spiralia;Lophotrochozoa;Mollusca;Bivalvia;Autobranchia;Heteroconchia;Euheterodonta;Imparidentia;Venerida;Veneroidea;Veneridae;Mercenaria;Mercenaria mercenaria
UdeM00000033711 6596 301.6 UdeM00000033711 XP_045182024.1 64.3 235 72 2 48 271 257 490 1.00E-78 301.6 6596 cellular organisms;Eukaryota;Opisthokonta;Metazoa;Eumetazoa;Bilateria;Protostomia;Spiralia;Lophotrochozoa;Mollusca;Bivalvia;Autobranchia;Heteroconchia;Euheterodonta;Imparidentia;Venerida;Veneroidea;Veneridae;Mercenaria;Mercenaria mercenaria
UdeM00000033712 202533 261.2 UdeM00000033712 XP_035218957.1 48.9 276 140 1 179 454 107 381 2.50E-66 261.2 202533 cellular organisms;Eukaryota;Opisthokonta;Metazoa;Eumetazoa;Bilateria;Protostomia;Ecdysozoa;Panarthropoda;Arthropoda;Chelicerata;Arachnida;Araneae;Araneomorphae;Entelegynae;Eresoidea;Eresidae;Stegodyphus;Stegodyphus dumicola
UdeM00000033713 29159 244.2 UdeM00000033713 XP_011447953.2 78 150 33 0 1 150 1 150 1.10E-61 244.2 29159 cellular organisms;Eukaryota;Opisthokonta;Metazoa;Eumetazoa;Bilateria;Protostomia;Spiralia;Lophotrochozoa;Mollusca;Bivalvia;Autobranchia;Pteriomorphia;Ostreida;Ostreoidea;Ostreidae;Crassostrea;Crassostrea gigas

UdeM00000033714 29159 115.5 UdeM00000033714 XP_011447959.2 29 359
216 13 3 351 33 362 1.40E-22 115.5 29159 cellular organisms;Eukaryota;Opisthokonta;Metazoa;Eumetazoa;Bilateria;Protostomia;Spiralia;Lophotrochozoa;Mollusca;Bivalvia;Autobranchia;Pteriomorphia;Ostreida;Ostreoidea;Ostreidae;Crassostrea;Crassostrea gigas
UdeM00000033715 6596 753.8 UdeM00000033715 XP_045182686.1 44.1 1123
424 37 102 1113 20 1049 3.00E-214 753.8 6596 cellular organisms;Eukaryota;Opisthokonta;Metazoa;Eumetazoa;Bilateria;Protostomia;Spiralia;Lophotrochozoa;Mollusca;Bivalvia;Autobranchia;Heteroconchia;Euheterodonta;Imparidentia;Venerida;Veneroidea;Veneridae;Mercenaria;Mercenaria mercenaria
UdeM00000033717 6596 114.4 UdeM00000033717 XP_045188183.1 35.1 174
94 2 42 215 89 243 3.50E-22 114.4 6596 cellular organisms;Eukaryota;Opisthokonta;Metazoa;Eumetazoa;Bilateria;Protostomia;Spiralia;Lophotrochozoa;Mollusca;Bivalvia;Autobranchia;Heteroconchia;Euheterodonta;Imparidentia;Venerida;Veneroidea;Veneridae;Mercenaria;Mercenaria mercenaria
UdeM00000033718 6573 255 UdeM00000033718 XP_021377872.1 27.9 681
439 21 1 665 1 645 2.70E-64 255 6573 cellular organisms;Eukaryota;Opisthokonta;Metazoa;Eumetazoa;Bilateria;Protostomia;Spiralia;Lophotrochozoa;Mollusca;Bivalvia;Autobranchia;Pteriomorphia;Pectinida;Pectinoidea;Pectinidae;Mizuhopecten;Mizuhopecten yessoensis
UdeM00000033719 6573 140.2 UdeM00000033719 XP_021354369.1 50 132
61 2 308 436 460 589 6.40E-30 140.2 6573 cellular organisms;Eukaryota;Opisthokonta;Metazoa;Eumetazoa;Bilateria;Protostomia;Spiralia;Lophotrochozoa;Mollusca;Bivalvia;Autobranchia;Pteriomorphia;Pectinida;Pectinoidea;Pectinidae;Mizuhopecten;Mizuhopecten yessoensis
UdeM00000033720 6573 139.8 UdeM00000033720 XP_021354369.1 50 132
61 2 308 436 460 589 8.30E-30 139.8 6573 cellular organisms;Eukaryota;Opisthokonta;Metazoa;Eumetazoa;Bilateria;Protostomia;Spiralia;Lophotrochozoa;Mollusca;Bivalvia;Autobranchia;Pteriomorphia;Pectinida;Pectinoidea;Pectinidae;Mizuhopecten;Mizuhopecten yessoensis
UdeM00000033721 6565 204.1 UdeM00000033721 XP_022287001.1 55.4 175
76 1 156 328 69 243 2.90E-49 204.1 6565 cellular organisms;Eukaryota;Opisthokonta;Metazoa;Eumetazoa;Bilateria;Protostomia;Spiralia;Lophotrochozoa;Mollusca;Bivalvia;Autobranchia;Pteriomorphia;Ostreida;Ostreoidea;Ostreidae;Crassostrea;Crassostrea virginica
UdeM00000033722 6565 222.6 UdeM00000033722 XP_022287001.1 51 208
99 2 156 361 69 275 7.90E-55 222.6 6565 cellular organisms;Eukaryota;Opisthokonta;Metazoa;Eumetazoa;Bilateria;Protostomia;Spiralia;Lophotrochozoa;Mollusca;Bivalvia;Autobranchia;Pteriomorphia;Ostreida;Ostreoidea;Ostreidae;Crassostrea;Crassostrea virginica
UdeM00000033723 6596 290 UdeM00000033723 XP_045177776.1 30.7 641
259 9 1 626 405 875 7.00E-75 290 6596 cellular organisms;Eukaryota;Opisthokonta;Metazoa;Eumetazoa;Bilateria;Protostomia;Spiralia;Lophotrochozoa;Mollusca;Bivalvia;Autobranchia;Heteroconchia;Euheterodonta;Imparidentia;Venerida;Veneroidea;Veneridae;Mercenaria;Mercenaria mercenaria
UdeM00000033724 6596 90.5 UdeM00000033724 XP_045191531.1 37.6 125
71 2 1 120 807 929 4.70E-15 90.5 6596 cellular organisms;Eukaryota;Opisthokonta;Metazoa;Eumetazoa;Bilateria;Protostomia;Spiralia;Lophotrochozoa;Mollusca;Bivalvia;Autobranchia;Heteroconchia;Euheterodonta;Imparidentia;Venerida;Veneroidea;Veneridae;Mercenaria;Mercenaria mercenaria

UdeM00000033725	150288	210.7	UdeM00000033725	XP_020788097.1	69.3
	127	39	0	2	128	439	565	1.10E-51	210.7	150288
	cellular
organisms;Eukaryota;Opisthokonta;Metazoa;Eumetazoa;Bilateria;Deuterostomia;Chordata;Craniata;Vertebrata;Gnathostomata;Teleostomi;Euteleostomi;Actinopterygii;Actinopteri;Neopterygii;Teleostei;Osteoglossocephalai;Clupeocephala;Euteleosteomorpha;Neoteleostei;Eurypterygia;Ctenosquamata;Acanthomorphata;Euacanthomorphacea;Percomorphaceae;Gobiaria;Gobiiformes;Gobioidei;Gobiidae;Oxudercinae;Boleophthalmus;Boleophthalmus pectinirostris
UdeM00000033726	6596	135.6	UdeM00000033726	XP_045216906.1	30.5	282
	103	5	4	192	175	456	7.00E-29	135.6	6596	cellular
organisms;Eukaryota;Opisthokonta;Metazoa;Eumetazoa;Bilateria;Protostomia;Spiralia;Lophotrochozoa;Mollusca;Bivalvia;Autobranchia;Heteroconchia;Euheterodonta;Imparidentia;Venerida;Veneroidea;Veneridae;Mercenaria;Mercenaria mercenaria
UdeM00000033727	29159	150.2	UdeM00000033727	XP_034318217.1	55.8	120
	52	1	1	119	755	874	1.70E-33	150.2	29159	cellular
organisms;Eukaryota;Opisthokonta;Metazoa;Eumetazoa;Bilateria;Protostomia;Spiralia;Lophotrochozoa;Mollusca;Bivalvia;Autobranchia;Pteriomorphia;Ostreida;Ostreoidea;Ostreidae;Crassostrea;Crassostrea gigas
UdeM00000033728	6596	363.6	UdeM00000033728	XP_045177776.1	36.3	571
	286	8	1	532	405	936	6.10E-97	363.6	6596	cellular
organisms;Eukaryota;Opisthokonta;Metazoa;Eumetazoa;Bilateria;Protostomia;Spiralia;Lophotrochozoa;Mollusca;Bivalvia;Autobranchia;Heteroconchia;Euheterodonta;Imparidentia;Venerida;Veneroidea;Veneridae;Mercenaria;Mercenaria mercenaria
UdeM00000044422	3055	364	UdeM00000044422	XP_001702748.1	56	323
	141	1	1	323	29	350	2.00E-97	364	3055	cellular
organisms;Eukaryota;Viridiplantae;Chlorophyta;core
chlorophytes;Chlorophyceae;CS
clade;Chlamydomonadales;Chlamydomonadaceae;Chlamydomonas;Chlamydomonas
reinhardtii
UdeM00000044423	69820	615.9	UdeM00000044423	XP_022836162.1	61.3	478
	179	3	5	479	5	479	4.30E-173	615.9	69820	cellular
organisms;Eukaryota;Opisthokonta;Metazoa;Eumetazoa;Bilateria;Protostomia;Ecdysozoa;Panarthropoda;Arthropoda;Mandibulata;Pancrustacea;Hexapoda;Insecta;Dicondylia;Pterygota;Neoptera;Endopterygota;Amphiesmenoptera;Lepidoptera;Glossata;Neolepidoptera;Heteroneura;Ditrysia;Obtectomera;Noctuoidea;Noctuidae;Amphipyrinae;Spodoptera;Spodoptera litura
UdeM00000044424	294128	331.6	UdeM00000044424	XP_018027785.1	55.7
	314	136	2	4	314	25	338	1.10E-87	331.6	294128
	cellular
organisms;Eukaryota;Opisthokonta;Metazoa;Eumetazoa;Bilateria;Protostomia;Ecdysozoa;Panarthropoda;Arthropoda;Mandibulata;Pancrustacea;Crustacea;Multicrustacea;Malacostraca;Eumalacostraca;Peracarida;Amphipoda;Senticaudata;Talitrida;Talitroidea;Hyalellidae;Hyalella;Hyalella azteca
UdeM00000044343	6596	89.7	UdeM00000044343	XP_045186357.1	65.1	63
	22	0	17	79	523	585	1.80E-15	89.7	6596	cellular
organisms;Eukaryota;Opisthokonta;Metazoa;Eumetazoa;Bilateria;Protostomia;Spiralia;Lophotrochozoa;Mollusca;Bivalvia;Autobranchia;Heteroconchia;Euheterodonta;Imparidentia;Venerida;Veneroidea;Veneridae;Mercenaria;Mercenaria mercenaria
UdeM00000044344	6565	181.8	UdeM00000044344	XP_022337131.1	44	250
	113	5	28	277	2	224	1.20E-42	181.8	6565	cellular

organisms;Eukaryota;Opisthokonta;Metazoa;Eumetazoa;Bilateria;Protostomia;Spiralia;Lophotrochozoa;Mollusca;Bivalvia;Autobranchia;Pteriomorphia;Ostreida;Ostreoidea;Ostreidae;Crassostrea;Crassostrea virginica
UdeM00000043257 29159 63.2 UdeM00000043257 XP_034305617.1 44.3 61 32 1 3 63 97 155 1.90E-07 63.2 29159 cellular organisms;Eukaryota;Opisthokonta;Metazoa;Eumetazoa;Bilateria;Protostomia;Spiralia;Lophotrochozoa;Mollusca;Bivalvia;Autobranchia;Pteriomorphia;Ostreida;Ostreoidea;Ostreidae;Crassostrea;Crassostrea gigas
UdeM00000043258 6579 153.3 UdeM00000043258 XP_033728059.1 52.3 149 68 1 1 149 1066 1211 2.50E-34 153.3 6579 cellular organisms;Eukaryota;Opisthokonta;Metazoa;Eumetazoa;Bilateria;Protostomia;Spiralia;Lophotrochozoa;Mollusca;Bivalvia;Autobranchia;Pteriomorphia;Pectinida;Pectinoidea;Pectinidae;Pecten;Pecten maximus
UdeM00000043259 6596 187.2 UdeM00000043259 XP_045177776.1 36.6 333 181 9 1 329 405 711 3.90E-44 187.2 6596 cellular organisms;Eukaryota;Opisthokonta;Metazoa;Eumetazoa;Bilateria;Protostomia;Spiralia;Lophotrochozoa;Mollusca;Bivalvia;Autobranchia;Heteroconchia;Euheterodonta;Imparidentia;Venerida;Veneroidea;Veneridae;Mercenaria;Mercenaria mercenaria
UdeM00000043260 6596 181.4 UdeM00000043260 XP_045188183.1 41.3 235 133 4 6 238 3 234 5.10E-42 181.4 6596 cellular organisms;Eukaryota;Opisthokonta;Metazoa;Eumetazoa;Bilateria;Protostomia;Spiralia;Lophotrochozoa;Mollusca;Bivalvia;Autobranchia;Heteroconchia;Euheterodonta;Imparidentia;Venerida;Veneroidea;Veneridae;Mercenaria;Mercenaria mercenaria
UdeM00000043261 6596 289.7 UdeM00000043261 XP_045177779.1 36.3 416 249 9 4 415 394 797 1.50E-74 289.7 6596 cellular organisms;Eukaryota;Opisthokonta;Metazoa;Eumetazoa;Bilateria;Protostomia;Spiralia;Lophotrochozoa;Mollusca;Bivalvia;Autobranchia;Heteroconchia;Euheterodonta;Imparidentia;Venerida;Veneroidea;Veneridae;Mercenaria;Mercenaria mercenaria
UdeM00000043262 7054 221.9 UdeM00000043262 XP_031333698.1 37.8 336 140 5 63 337 321 648 1.30E-54 221.9 7054 cellular organisms;Eukaryota;Opisthokonta;Metazoa;Eumetazoa;Bilateria;Protostomia;Ecdysozoa;Panarthropoda;Arthropoda;Mandibulata;Pancrustacea;Hexapoda;Insecta;Dicondylia;Pterygota;Neoptera;Endopterygota;Coleoptera;Polyphaga;Elateriformia;Elateroidea;Lampyridae;Lampyrinae;Photinus;Photinus pyralis
UdeM00000043263 46514 67.4 UdeM00000043263 XP_038065173.1 49.4 77 35 2 5 77 8 84 9.20E-09 67.4 46514 cellular organisms;Eukaryota;Opisthokonta;Metazoa;Eumetazoa;Bilateria;Deuterostomia;Echinodermata;Eleutherozoa;Asterozoa;Asteroidea;Valvatacea;Valvatida;Asterinidae;Patiria;Patiria miniata
UdeM00000043265 6596 190.7 UdeM00000043265 XP_045182317.1 35.1 370 178 6 16 326 261 627 3.40E-45 190.7 6596 cellular organisms;Eukaryota;Opisthokonta;Metazoa;Eumetazoa;Bilateria;Protostomia;Spiralia;Lophotrochozoa;Mollusca;Bivalvia;Autobranchia;Heteroconchia;Euheterodonta;Imparidentia;Venerida;Veneroidea;Veneridae;Mercenaria;Mercenaria mercenaria
UdeM00000043267 6596 188.7 UdeM00000043267 XP_045191793.1 35 357 198 8 1 349 258 588 1.50E-44 188.7 6596 cellular organisms;Eukaryota;Opisthokonta;Metazoa;Eumetazoa;Bilateria;Protostomia;Spiralia;Lophotrochozoa;Mollusca;Bivalvia;Autobranchia;Heteroconchia;Euheterodonta;Imparidentia;Venerida;Veneroidea;Veneridae;Mercenaria;Mercenaria mercenaria

UdeM00000017129 189513 102.8 UdeM00000017129 XP_044736929.1 29.7
249 136 11 414 659 135 347 1.90E-18 102.8 189513
cellular
organisms;Eukaryota;Opisthokonta;Metazoa;Eumetazoa;Bilateria;Protostomia;
Ecdysozoa;Panarthropoda;Arthropoda;Mandibulata;Pancrustacea;Hexapoda;Inse
cta;Dicondylia;Pterygota;Neoptera;Endopterygota;Neuropterida;Neuroptera;H
emerobiiformia;Chrysopidae;Chrysopinae;Chrysoperla;Chrysoperla carnea
UdeM00000017130 6573 1508.8 UdeM00000017130 XP_021341468.1 51.7
1544 712 12 5 1525 17 1550 0.00E+00 1508.8 6573
cellular
organisms;Eukaryota;Opisthokonta;Metazoa;Eumetazoa;Bilateria;Protostomia;
Spiralia;Lophotrochozoa;Mollusca;Bivalvia;Autobranchia;Pteriomorphia;Pect
inida;Pectinoidea;Pectinidae;Mizuhopecten;Mizuhopecten yessoensis
UdeM00000017131 6596 680.6 UdeM00000017131 XP_045207510.1 60.2 583
228 2 5 584 639 1220 1.70E-192 680.6 6596 cellular
organisms;Eukaryota;Opisthokonta;Metazoa;Eumetazoa;Bilateria;Protostomia;
Spiralia;Lophotrochozoa;Mollusca;Bivalvia;Autobranchia;Heteroconchia;Euhe
terodonta;Imparidentia;Venerida;Veneroidea;Veneridae;Mercenaria;Mercenari
a mercenaria
UdeM00000017132 6573 797.3 UdeM00000017132 XP_021341468.1 47.5 886
442 8 7 870 19 903 1.90E-227 797.3 6573 cellular
organisms;Eukaryota;Opisthokonta;Metazoa;Eumetazoa;Bilateria;Protostomia;
Spiralia;Lophotrochozoa;Mollusca;Bivalvia;Autobranchia;Pteriomorphia;Pect
inida;Pectinoidea;Pectinidae;Mizuhopecten;Mizuhopecten yessoensis
UdeM00000017133 6596 85.5 UdeM00000017133 XP_045196953.1 21.8 774
483 33 19 717 20 746 3.00E-13 85.5 6596 cellular
organisms;Eukaryota;Opisthokonta;Metazoa;Eumetazoa;Bilateria;Protostomia;
Spiralia;Lophotrochozoa;Mollusca;Bivalvia;Autobranchia;Heteroconchia;Euhe
terodonta;Imparidentia;Venerida;Veneroidea;Veneridae;Mercenaria;Mercenari
a mercenaria
UdeM00000017134 6596 85.5 UdeM00000017134 XP_045196953.1 21.8 774
483 33 30 728 20 746 3.10E-13 85.5 6596 cellular
organisms;Eukaryota;Opisthokonta;Metazoa;Eumetazoa;Bilateria;Protostomia;
Spiralia;Lophotrochozoa;Mollusca;Bivalvia;Autobranchia;Heteroconchia;Euhe
terodonta;Imparidentia;Venerida;Veneroidea;Veneridae;Mercenaria;Mercenari
a mercenaria
UdeM00000017136 6565 88.2 UdeM00000017136 XP_022313678.1 25.3 379
227 16 335 683 145 497 4.70E-14 88.2 6565 cellular
organisms;Eukaryota;Opisthokonta;Metazoa;Eumetazoa;Bilateria;Protostomia;
Spiralia;Lophotrochozoa;Mollusca;Bivalvia;Autobranchia;Pteriomorphia;Ostr
eida;Ostreoidea;Ostreidae;Crassostrea;Crassostrea virginica
UdeM00000017135 6565 88.2 UdeM00000017135 XP_022313678.1 25.3 379
227 16 338 686 145 497 4.70E-14 88.2 6565 cellular
organisms;Eukaryota;Opisthokonta;Metazoa;Eumetazoa;Bilateria;Protostomia;
Spiralia;Lophotrochozoa;Mollusca;Bivalvia;Autobranchia;Pteriomorphia;Ostr
eida;Ostreoidea;Ostreidae;Crassostrea;Crassostrea virginica
UdeM00000017137 6565 474.6 UdeM00000017137 XP_022337291.1 39.9 711
401 13 2 701 58 753 2.40E-130 474.6 6565 cellular
organisms;Eukaryota;Opisthokonta;Metazoa;Eumetazoa;Bilateria;Protostomia;
Spiralia;Lophotrochozoa;Mollusca;Bivalvia;Autobranchia;Pteriomorphia;Ostr
eida;Ostreoidea;Ostreidae;Crassostrea;Crassostrea virginica
UdeM00000017139 6565 557.4 UdeM00000017139 XP_022337291.1 41.1 791
438 13 34 814 58 830 3.40E-155 557.4 6565 cellular
organisms;Eukaryota;Opisthokonta;Metazoa;Eumetazoa;Bilateria;Protostomia;

Spiralia;Lophotrochozoa;Mollusca;Bivalvia;Autobranchia;Pteriomorphia;Ostreida;Ostreoidea;Ostreidae;Crassostrea;Crassostrea virginica
UdeM00000017140 6596 532.3 UdeM00000017140 XP_045167151.1 44.5 721 380 11 24 734 84 794 1.00E-147 532.3 6596 cellular organisms;Eukaryota;Opisthokonta;Metazoa;Eumetazoa;Bilateria;Protostomia;Spiralia;Lophotrochozoa;Mollusca;Bivalvia;Autobranchia;Heteroconchia;Euheterodonta;Imparidentia;Venerida;Veneroidea;Veneridae;Mercenaria;Mercenaria mercenaria
UdeM00000017141 6596 715.7 UdeM00000017141 XP_045163747.1 64.4 564 183 4 46 606 88 636 5.00E-203 715.7 6596 cellular organisms;Eukaryota;Opisthokonta;Metazoa;Eumetazoa;Bilateria;Protostomia;Spiralia;Lophotrochozoa;Mollusca;Bivalvia;Autobranchia;Heteroconchia;Euheterodonta;Imparidentia;Venerida;Veneroidea;Veneridae;Mercenaria;Mercenaria mercenaria
UdeM00000017142 6596 715.7 UdeM00000017142 XP_045163747.1 64.4 564 183 4 62 622 88 636 5.20E-203 715.7 6596 cellular organisms;Eukaryota;Opisthokonta;Metazoa;Eumetazoa;Bilateria;Protostomia;Spiralia;Lophotrochozoa;Mollusca;Bivalvia;Autobranchia;Heteroconchia;Euheterodonta;Imparidentia;Venerida;Veneroidea;Veneridae;Mercenaria;Mercenaria mercenaria
UdeM00000017143 6596 98.6 UdeM00000017143 XP_045196953.1 20.9 791 504 28 23 754 18 745 3.60E-17 98.6 6596 cellular organisms;Eukaryota;Opisthokonta;Metazoa;Eumetazoa;Bilateria;Protostomia;Spiralia;Lophotrochozoa;Mollusca;Bivalvia;Autobranchia;Heteroconchia;Euheterodonta;Imparidentia;Venerida;Veneroidea;Veneridae;Mercenaria;Mercenaria mercenaria
UdeM00000017144 6596 84 UdeM00000017144 XP_045193866.1 23.2 725 442 30 71 737 22 689 9.60E-13 84 6596 cellular organisms;Eukaryota;Opisthokonta;Metazoa;Eumetazoa;Bilateria;Protostomia;Spiralia;Lophotrochozoa;Mollusca;Bivalvia;Autobranchia;Heteroconchia;Euheterodonta;Imparidentia;Venerida;Veneroidea;Veneridae;Mercenaria;Mercenaria mercenaria
UdeM00000017145 6596 84 UdeM00000017145 XP_045193866.1 32.1 262 140 11 86 324 384 630 5.20E-13 84 6596 cellular organisms;Eukaryota;Opisthokonta;Metazoa;Eumetazoa;Bilateria;Protostomia;Spiralia;Lophotrochozoa;Mollusca;Bivalvia;Autobranchia;Heteroconchia;Euheterodonta;Imparidentia;Venerida;Veneroidea;Veneridae;Mercenaria;Mercenaria mercenaria
UdeM00000017148 225164 125.6 UdeM00000017148 XP_009051919.1 58.6 111 43 2 4 111 34 144 4.40E-26 125.6 225164 cellular organisms;Eukaryota;Opisthokonta;Metazoa;Eumetazoa;Bilateria;Protostomia;Spiralia;Lophotrochozoa;Mollusca;Gastropoda;Patellogastropoda;Lottioidea;Lottiidae;Lottia;Lottia gigantea
UdeM00000017147 225164 186 UdeM00000017147 XP_009051919.1 61.8 144 54 1 14 156 1 144 3.80E-44 186 225164 cellular organisms;Eukaryota;Opisthokonta;Metazoa;Eumetazoa;Bilateria;Protostomia;Spiralia;Lophotrochozoa;Mollusca;Gastropoda;Patellogastropoda;Lottioidea;Lottiidae;Lottia;Lottia gigantea
UdeM00000017149 6596 133.7 UdeM00000017149 XP_045196953.1 23.7 807 477 30 17 763 18 745 1.00E-27 133.7 6596 cellular organisms;Eukaryota;Opisthokonta;Metazoa;Eumetazoa;Bilateria;Protostomia;Spiralia;Lophotrochozoa;Mollusca;Bivalvia;Autobranchia;Heteroconchia;Euhe

terodonta;Imparidentia;Venerida;Veneroidea;Veneridae;Mercenaria;Mercenaria mercenaria
UdeM00000017150 6573 171 UdeM00000017150 XP_021368552.1 52.1 169 81 0 1 169 1 169 1.30E-39 171 6573 cellular organisms;Eukaryota;Opisthokonta;Metazoa;Eumetazoa;Bilateria;Protostomia;Spiralia;Lophotrochozoa;Mollusca;Bivalvia;Autobranchia;Pteriomorphia;Pectinida;Pectinoidea;Pectinidae;Mizuhopecten;Mizuhopecten yessoensis
UdeM00000017151 6596 262.7 UdeM00000017151 XP_045185723.1 43.7 325 167 4 7 331 455 763 6.30E-67 262.7 6596 cellular organisms;Eukaryota;Opisthokonta;Metazoa;Eumetazoa;Bilateria;Protostomia;Spiralia;Lophotrochozoa;Mollusca;Bivalvia;Autobranchia;Heteroconchia;Euheterodonta;Imparidentia;Venerida;Veneroidea;Veneridae;Mercenaria;Mercenaria mercenaria
UdeM00000017154 6579 652.9 UdeM00000017154 XP_033748227.1 73.7 419 109 1 22 439 55 473 2.90E-184 652.9 6579 cellular organisms;Eukaryota;Opisthokonta;Metazoa;Eumetazoa;Bilateria;Protostomia;Spiralia;Lophotrochozoa;Mollusca;Bivalvia;Autobranchia;Pteriomorphia;Pectinida;Pectinoidea;Pectinidae;Pecten;Pecten maximus
UdeM00000017155 29159 251.5 UdeM00000017155 XP_011418327.2 67.3 171 56 0 1 171 1 171 7.60E-64 251.5 29159 cellular organisms;Eukaryota;Opisthokonta;Metazoa;Eumetazoa;Bilateria;Protostomia;Spiralia;Lophotrochozoa;Mollusca;Bivalvia;Autobranchia;Pteriomorphia;Ostreida;Ostreoidea;Ostreidae;Crassostrea;Crassostrea gigas
UdeM00000017156 6565 335.9 UdeM00000017156 XP_022342034.1 52.9 361 144 7 3 353 2 346 9.00E-89 335.9 6565 cellular organisms;Eukaryota;Opisthokonta;Metazoa;Eumetazoa;Bilateria;Protostomia;Spiralia;Lophotrochozoa;Mollusca;Bivalvia;Autobranchia;Pteriomorphia;Ostreida;Ostreoidea;Ostreidae;Crassostrea;Crassostrea virginica
UdeM00000017157 6596 194.1 UdeM00000017157 XP_045172235.1 56.7 208 79 3 12 219 2 198 1.80E-46 194.1 6596 cellular organisms;Eukaryota;Opisthokonta;Metazoa;Eumetazoa;Bilateria;Protostomia;Spiralia;Lophotrochozoa;Mollusca;Bivalvia;Autobranchia;Heteroconchia;Euheterodonta;Imparidentia;Venerida;Veneroidea;Veneridae;Mercenaria;Mercenaria mercenaria
UdeM00000017158 6596 173.7 UdeM00000017158 XP_045172235.1 56.7 187 70 3 12 198 2 177 2.40E-40 173.7 6596 cellular organisms;Eukaryota;Opisthokonta;Metazoa;Eumetazoa;Bilateria;Protostomia;Spiralia;Lophotrochozoa;Mollusca;Bivalvia;Autobranchia;Heteroconchia;Euheterodonta;Imparidentia;Venerida;Veneroidea;Veneridae;Mercenaria;Mercenaria mercenaria
UdeM00000017159 6596 344.7 UdeM00000017159 XP_045172778.1 77.7 215 48 0 40 254 5 219 9.80E-92 344.7 6596 cellular organisms;Eukaryota;Opisthokonta;Metazoa;Eumetazoa;Bilateria;Protostomia;Spiralia;Lophotrochozoa;Mollusca;Bivalvia;Autobranchia;Heteroconchia;Euheterodonta;Imparidentia;Venerida;Veneroidea;Veneridae;Mercenaria;Mercenaria mercenaria
UdeM00000017160 6596 344 UdeM00000017160 XP_045172778.1 78 214 47 0 1 214 6 219 1.40E-91 344 6596 cellular organisms;Eukaryota;Opisthokonta;Metazoa;Eumetazoa;Bilateria;Protostomia;Spiralia;Lophotrochozoa;Mollusca;Bivalvia;Autobranchia;Heteroconchia;Euheterodonta;Imparidentia;Venerida;Veneroidea;Veneridae;Mercenaria;Mercenaria mercenaria
UdeM00000017161 386614 201.1 UdeM00000017161 XP_032906139.1 37.8 291 174 1 12 302 15 298 2.10E-48 201.1 386614

cellular organisms;Eukaryota;Opisthokonta;Metazoa;Eumetazoa;Bilateria;Deuterostomia;Chordata;Craniata;Vertebrata;Gnathostomata;Chondrichthyes;Elasmobranchii;Batoidea;Rajiformes;Rajidae;Amblyraja;Amblyraja radiata
UdeM00000017162 29159 72.4 UdeM00000017162 XP_034330259.1 47.9 71 35 1 1 71 213 281 2.90E-10 72.4 29159 cellular organisms;Eukaryota;Opisthokonta;Metazoa;Eumetazoa;Bilateria;Protostomia;Spiralia;Lophotrochozoa;Mollusca;Bivalvia;Autobranchia;Pteriomorphia;Ostreida;Ostreoidea;Ostreidae;Crassostrea;Crassostrea gigas
UdeM00000017163 6596 270.4 UdeM00000017163 XP_045159778.1 50.6 253 123 2 11 262 4 255 2.50E-69 270.4 6596 cellular organisms;Eukaryota;Opisthokonta;Metazoa;Eumetazoa;Bilateria;Protostomia;Spiralia;Lophotrochozoa;Mollusca;Bivalvia;Autobranchia;Heteroconchia;Euheterodonta;Imparidentia;Venerida;Veneroidea;Veneridae;Mercenaria;Mercenaria mercenaria
UdeM00000017164 6596 444.9 UdeM00000017164 XP_045159777.1 57.9 366 147 2 1 366 52 410 1.00E-121 444.9 6596 cellular organisms;Eukaryota;Opisthokonta;Metazoa;Eumetazoa;Bilateria;Protostomia;Spiralia;Lophotrochozoa;Mollusca;Bivalvia;Autobranchia;Heteroconchia;Euheterodonta;Imparidentia;Venerida;Veneroidea;Veneridae;Mercenaria;Mercenaria mercenaria
UdeM00000017165 6596 133.7 UdeM00000017165 XP_045159778.1 62.7 102 38 0 43 144 266 367 2.00E-28 133.7 6596 cellular organisms;Eukaryota;Opisthokonta;Metazoa;Eumetazoa;Bilateria;Protostomia;Spiralia;Lophotrochozoa;Mollusca;Bivalvia;Autobranchia;Heteroconchia;Euheterodonta;Imparidentia;Venerida;Veneroidea;Veneridae;Mercenaria;Mercenaria mercenaria
UdeM00000017167 400727 146 UdeM00000017167 XP_025115998.1 29.9 364 233 8 8 357 505 860 9.40E-32 146 400727 cellular organisms;Eukaryota;Opisthokonta;Metazoa;Eumetazoa;Bilateria;Protostomia;Spiralia;Lophotrochozoa;Mollusca;Gastropoda;Caenogastropoda;Architaenioglossa;Ampullarioidea;Ampullariidae;Pomacea;Pomacea canaliculata
UdeM00000017173 6596 77.4 UdeM00000017173 XP_045202427.1 28.4 190 112 6 69 246 539 716 2.80E-11 77.4 6596 cellular organisms;Eukaryota;Opisthokonta;Metazoa;Eumetazoa;Bilateria;Protostomia;Spiralia;Lophotrochozoa;Mollusca;Bivalvia;Autobranchia;Heteroconchia;Euheterodonta;Imparidentia;Venerida;Veneroidea;Veneridae;Mercenaria;Mercenaria mercenaria
UdeM00000017174 6573 133.7 UdeM00000017174 XP_021362994.1 29.5 332 211 9 52 372 55 374 5.20E-28 133.7 6573 cellular organisms;Eukaryota;Opisthokonta;Metazoa;Eumetazoa;Bilateria;Protostomia;Spiralia;Lophotrochozoa;Mollusca;Bivalvia;Autobranchia;Pteriomorphia;Pectinida;Pectinoidea;Pectinidae;Mizuhopecten;Mizuhopecten yessoensis
UdeM00000017175 6412 412.9 UdeM00000017175 XP_009012646.1 56 348 150 2 1 346 1 347 4.20E-112 412.9 6412 cellular organisms;Eukaryota;Opisthokonta;Metazoa;Eumetazoa;Bilateria;Protostomia;Spiralia;Lophotrochozoa;Annelida;Clitellata;Hirudinea;Rhynchobdellida;Glossiphoniidae;Helobdella;Helobdella robusta
UdeM00000017178 10036 68.6 UdeM00000017178 XP_040602731.1 61.5 52 20 0 8 59 5 56 3.80E-09 68.6 10036 cellular organisms;Eukaryota;Opisthokonta;Metazoa;Eumetazoa;Bilateria;Deuterostomia;Chordata;Craniata;Vertebrata;Gnathostomata;Teleostomi;Euteleostomi;Sarcopterygii;Dipnotetrapodomorpha;Tetrapoda;Amniota;Mammalia;Theria;Eutheria

;Boreoeutheria;Euarchontoglires;Glires;Rodentia;Myomorpha;Muroidea;Cricetidae;Cricetinae;Mesocricetus;Mesocricetus auratus
UdeM00000017179 6579 440.7 UdeM00000017179 XP_033748232.1 33.1 1161 606 30 1801 2895 1251 2306 1.50E-119 440.7 6579 cellular organisms;Eukaryota;Opisthokonta;Metazoa;Eumetazoa;Bilateria;Protostomia;Spiralia;Lophotrochozoa;Mollusca;Bivalvia;Autobranchia;Pteriomorphia;Pectinida;Pectinoidea;Pectinidae;Pecten;Pecten maximus
UdeM00000017179 6579 58.5 UdeM00000017179 XP_033748232.1 60 45 18 0 43 87 7 51 1.60E-04 58.5 6579 cellular organisms;Eukaryota;Opisthokonta;Metazoa;Eumetazoa;Bilateria;Protostomia;Spiralia;Lophotrochozoa;Mollusca;Bivalvia;Autobranchia;Pteriomorphia;Pectinida;Pectinoidea;Pectinidae;Pecten;Pecten maximus
UdeM00000017180 6596 374.8 UdeM00000017180 XP_045207388.1 34.8 909 513 28 326 1182 9 889 4.10E-100 374.8 6596 cellular organisms;Eukaryota;Opisthokonta;Metazoa;Eumetazoa;Bilateria;Protostomia;Spiralia;Lophotrochozoa;Mollusca;Bivalvia;Autobranchia;Heteroconchia;Euheterodonta;Imparidentia;Venerida;Veneroidea;Veneridae;Mercenaria;Mercenaria mercenaria
UdeM00000017181 6596 374.8 UdeM00000017181 XP_045207388.1 34.8 909 513 28 422 1278 9 889 4.50E-100 374.8 6596 cellular organisms;Eukaryota;Opisthokonta;Metazoa;Eumetazoa;Bilateria;Protostomia;Spiralia;Lophotrochozoa;Mollusca;Bivalvia;Autobranchia;Heteroconchia;Euheterodonta;Imparidentia;Venerida;Veneroidea;Veneridae;Mercenaria;Mercenaria mercenaria
UdeM00000017182 6596 130.6 UdeM00000017182 XP_045207393.1 41.2 160 87 4 38 192 21 178 2.20E-27 130.6 6596 cellular organisms;Eukaryota;Opisthokonta;Metazoa;Eumetazoa;Bilateria;Protostomia;Spiralia;Lophotrochozoa;Mollusca;Bivalvia;Autobranchia;Heteroconchia;Euheterodonta;Imparidentia;Venerida;Veneroidea;Veneridae;Mercenaria;Mercenaria mercenaria
UdeM00000017183 6596 276.9 UdeM00000017183 XP_045171339.1 51.9 237 112 2 1 236 117 352 2.40E-71 276.9 6596 cellular organisms;Eukaryota;Opisthokonta;Metazoa;Eumetazoa;Bilateria;Protostomia;Spiralia;Lophotrochozoa;Mollusca;Bivalvia;Autobranchia;Heteroconchia;Euheterodonta;Imparidentia;Venerida;Veneroidea;Veneridae;Mercenaria;Mercenaria mercenaria
UdeM00000017184 29159 282.3 UdeM00000017184 XP_011422571.2 74.7 178 45 0 1 178 282 459 4.20E-73 282.3 29159 cellular organisms;Eukaryota;Opisthokonta;Metazoa;Eumetazoa;Bilateria;Protostomia;Spiralia;Lophotrochozoa;Mollusca;Bivalvia;Autobranchia;Pteriomorphia;Ostreida;Ostreoidea;Ostreidae;Crassostrea;Crassostrea gigas
UdeM00000017186 42526 157.9 UdeM00000017186 XP_036443704.1 68.2 107 34 0 2 108 44 150 7.40E-36 157.9 42526 cellular organisms;Eukaryota;Opisthokonta;Metazoa;Eumetazoa;Bilateria;Deuterostomia;Chordata;Craniata;Vertebrata;Gnathostomata;Teleostomi;Euteleostomi;Actinopterygii;Actinopteri;Neopterygii;Teleostei;Osteoglossocephalai;Clupeocephala;Otomorpha;Ostariophysi;Otophysi;Characiphysae;Characiformes;Characoidei;Serrasalmidae;Colossoma;Colossoma macropomum
UdeM00000021373 6596 574.7 UdeM00000021373 XP_045216588.1 53 566 231 6 1 549 2171 2718 1.70E-160 574.7 6596 cellular organisms;Eukaryota;Opisthokonta;Metazoa;Eumetazoa;Bilateria;Protostomia;Spiralia;Lophotrochozoa;Mollusca;Bivalvia;Autobranchia;Heteroconchia;Euheterodonta;Imparidentia;Venerida;Veneroidea;Veneridae;Mercenaria;Mercenaria mercenaria

UdeM00000021374 6596 1718.7 UdeM00000021374 XP_045216588.1 48.1 1808 885 20 18 1802 348 2125 0.00E+00 1718.7 6596 cellular organisms;Eukaryota;Opisthokonta;Metazoa;Eumetazoa;Bilateria;Protostomia;Spiralia;Lophotrochozoa;Mollusca;Bivalvia;Autobranchia;Heteroconchia;Euheterodonta;Imparidentia;Venerida;Veneroidea;Veneridae;Mercenaria;Mercenaria mercenaria
UdeM00000021375 6596 176.4 UdeM00000021375 XP_045215148.1 30.1 342 207 9 119 438 1 332 7.90E-41 176.4 6596 cellular organisms;Eukaryota;Opisthokonta;Metazoa;Eumetazoa;Bilateria;Protostomia;Spiralia;Lophotrochozoa;Mollusca;Bivalvia;Autobranchia;Heteroconchia;Euheterodonta;Imparidentia;Venerida;Veneroidea;Veneridae;Mercenaria;Mercenaria mercenaria
UdeM00000021376 6596 549.7 UdeM00000021376 XP_045216685.1 67.3 425 135 1 118 538 112 536 4.20E-153 549.7 6596 cellular organisms;Eukaryota;Opisthokonta;Metazoa;Eumetazoa;Bilateria;Protostomia;Spiralia;Lophotrochozoa;Mollusca;Bivalvia;Autobranchia;Heteroconchia;Euheterodonta;Imparidentia;Venerida;Veneroidea;Veneridae;Mercenaria;Mercenaria mercenaria
UdeM00000021378 6573 353.6 UdeM00000021378 XP_021360512.1 74.8 234 59 0 1 234 3 236 2.00E-94 353.6 6573 cellular organisms;Eukaryota;Opisthokonta;Metazoa;Eumetazoa;Bilateria;Protostomia;Spiralia;Lophotrochozoa;Mollusca;Bivalvia;Autobranchia;Pteriomorphia;Pectinida;Pectinoidea;Pectinidae;Mizuhopecten;Mizuhopecten yessoensis
UdeM00000021379 6579 375.6 UdeM00000021379 XP_033749419.1 74.9 247 62 0 1 247 3 249 5.10E-101 375.6 6579 cellular organisms;Eukaryota;Opisthokonta;Metazoa;Eumetazoa;Bilateria;Protostomia;Spiralia;Lophotrochozoa;Mollusca;Bivalvia;Autobranchia;Pteriomorphia;Pectinida;Pectinoidea;Pectinidae;Pecten;Pecten maximus
UdeM00000021380 6596 91.3 UdeM00000021380 XP_045207772.1 40.2 132 72 5 33 158 11 141 1.80E-15 91.3 6596 cellular organisms;Eukaryota;Opisthokonta;Metazoa;Eumetazoa;Bilateria;Protostomia;Spiralia;Lophotrochozoa;Mollusca;Bivalvia;Autobranchia;Heteroconchia;Euheterodonta;Imparidentia;Venerida;Veneroidea;Veneridae;Mercenaria;Mercenaria mercenaria
UdeM00000021381 6596 90.9 UdeM00000021381 XP_045207772.1 40.2 132 72 5 7 132 11 141 2.00E-15 90.9 6596 cellular organisms;Eukaryota;Opisthokonta;Metazoa;Eumetazoa;Bilateria;Protostomia;Spiralia;Lophotrochozoa;Mollusca;Bivalvia;Autobranchia;Heteroconchia;Euheterodonta;Imparidentia;Venerida;Veneroidea;Veneridae;Mercenaria;Mercenaria mercenaria
UdeM00000021382 6596 107.1 UdeM00000021382 XP_045181980.1 53.6 97 42 2 303 399 285 378 5.60E-20 107.1 6596 cellular organisms;Eukaryota;Opisthokonta;Metazoa;Eumetazoa;Bilateria;Protostomia;Spiralia;Lophotrochozoa;Mollusca;Bivalvia;Autobranchia;Heteroconchia;Euheterodonta;Imparidentia;Venerida;Veneroidea;Veneridae;Mercenaria;Mercenaria mercenaria
UdeM00000021383 6596 107.1 UdeM00000021383 XP_045181980.1 53.6 97 42 2 297 393 285 378 5.50E-20 107.1 6596 cellular organisms;Eukaryota;Opisthokonta;Metazoa;Eumetazoa;Bilateria;Protostomia;Spiralia;Lophotrochozoa;Mollusca;Bivalvia;Autobranchia;Heteroconchia;Euheterodonta;Imparidentia;Venerida;Veneroidea;Veneridae;Mercenaria;Mercenaria mercenaria

UdeM00000021384 6579 82 UdeM00000021384 XP_033750328.1 27.3 422
232 14 23 427 2 365 2.00E-12 82 6579 cellular
organisms;Eukaryota;Opisthokonta;Metazoa;Eumetazoa;Bilateria;Protostomia;
Spiralia;Lophotrochozoa;Mollusca;Bivalvia;Autobranchia;Pteriomorphia;Pect
inida;Pectinoidea;Pectinidae;Pecten;Pecten maximus
UdeM00000021385 400727 275.8 UdeM00000021385 XP_025077216.1 42.9
359 201 2 14 369 53 410 8.10E-71 275.8 400727
cellular
organisms;Eukaryota;Opisthokonta;Metazoa;Eumetazoa;Bilateria;Protostomia;
Spiralia;Lophotrochozoa;Mollusca;Gastropoda;Caenogastropoda;Architaeniogl
ossa;Ampullarioidea;Ampullariidae;Pomacea;Pomacea canaliculata
UdeM00000021386 6579 1639.8 UdeM00000021386 XP_033749503.1 61.9
1348 497 9 1 1333 1 1347 0.00E+00 1639.8 6579
cellular
organisms;Eukaryota;Opisthokonta;Metazoa;Eumetazoa;Bilateria;Protostomia;
Spiralia;Lophotrochozoa;Mollusca;Bivalvia;Autobranchia;Pteriomorphia;Pect
inida;Pectinoidea;Pectinidae;Pecten;Pecten maximus
UdeM00000021388 6573 146.7 UdeM00000021388 XP_021346093.1 40.5 215
77 1 10 224 13 176 3.70E-32 146.7 6573 cellular
organisms;Eukaryota;Opisthokonta;Metazoa;Eumetazoa;Bilateria;Protostomia;
Spiralia;Lophotrochozoa;Mollusca;Bivalvia;Autobranchia;Pteriomorphia;Pect
inida;Pectinoidea;Pectinidae;Mizuhopecten;Mizuhopecten yessoensis
UdeM00000021387 6573 146 UdeM00000021387 XP_021346093.1 40.5 215
77 1 11 225 13 176 6.30E-32 146 6573 cellular
organisms;Eukaryota;Opisthokonta;Metazoa;Eumetazoa;Bilateria;Protostomia;
Spiralia;Lophotrochozoa;Mollusca;Bivalvia;Autobranchia;Pteriomorphia;Pect
inida;Pectinoidea;Pectinidae;Mizuhopecten;Mizuhopecten yessoensis
UdeM00000021389 37621 145.6 UdeM00000021389 XP_014673981.1 46.8 171
57 3 114 250 48 218 9.10E-32 145.6 37621 cellular
organisms;Eukaryota;Opisthokonta;Metazoa;Eumetazoa;Bilateria;Protostomia;
Ecdysozoa;Scalidophora;Priapulida;Priapulimorpha;Priapulimorphida;Priapul
idae;Priapulus;Priapulus caudatus
UdeM00000021390 37621 211.8 UdeM00000021390 XP_014672567.1 51.7 174
84 0 10 183 345 518 9.00E-52 211.8 37621 cellular
organisms;Eukaryota;Opisthokonta;Metazoa;Eumetazoa;Bilateria;Protostomia;
Ecdysozoa;Scalidophora;Priapulida;Priapulimorpha;Priapulimorphida;Priapul
idae;Priapulus;Priapulus caudatus
UdeM00000021391 6573 498.4 UdeM00000021391 XP_021346091.1 45.3 656
223 9 3 627 84 634 1.30E-137 498.4 6573 cellular
organisms;Eukaryota;Opisthokonta;Metazoa;Eumetazoa;Bilateria;Protostomia;
Spiralia;Lophotrochozoa;Mollusca;Bivalvia;Autobranchia;Pteriomorphia;Pect
inida;Pectinoidea;Pectinidae;Mizuhopecten;Mizuhopecten yessoensis
UdeM00000021394 6565 62 UdeM00000021394 XP_022287521.1 39.7 73
44 0 2 74 313 385 4.90E-07 62 6565 cellular
organisms;Eukaryota;Opisthokonta;Metazoa;Eumetazoa;Bilateria;Protostomia;
Spiralia;Lophotrochozoa;Mollusca;Bivalvia;Autobranchia;Pteriomorphia;Ostr
eida;Ostreoidea;Ostreidae;Crassostrea;Crassostrea virginica
UdeM00000021395 6579 242.7 UdeM00000021395 XP_033751024.1 38.7 426
218 12 388 801 521 915 1.70E-60 242.7 6579 cellular
organisms;Eukaryota;Opisthokonta;Metazoa;Eumetazoa;Bilateria;Protostomia;
Spiralia;Lophotrochozoa;Mollusca;Bivalvia;Autobranchia;Pteriomorphia;Pect
inida;Pectinoidea;Pectinidae;Pecten;Pecten maximus
UdeM00000021396 6596 181 UdeM00000021396 XP_045156271.1 45.8 201
99 2 23 213 25 225 1.60E-42 181 6596 cellular

organisms;Eukaryota;Opisthokonta;Metazoa;Eumetazoa;Bilateria;Protostomia;Spiralia;Lophotrochozoa;Mollusca;Bivalvia;Autobranchia;Heteroconchia;Euheterodonta;Imparidentia;Venerida;Veneroidea;Veneridae;Mercenaria;Mercenaria mercenaria
UdeM00000021397 6596 295 UdeM00000021397 XP_045157060.1 43.5 331 177 3 65 389 46 372 1.50E-76 295 6596 cellular organisms;Eukaryota;Opisthokonta;Metazoa;Eumetazoa;Bilateria;Protostomia;Spiralia;Lophotrochozoa;Mollusca;Bivalvia;Autobranchia;Heteroconchia;Euheterodonta;Imparidentia;Venerida;Veneroidea;Veneridae;Mercenaria;Mercenaria mercenaria
UdeM00000021398 6596 215.7 UdeM00000021398 XP_045156445.1 53.3 199 92 1 4 201 70 268 6.30E-53 215.7 6596 cellular organisms;Eukaryota;Opisthokonta;Metazoa;Eumetazoa;Bilateria;Protostomia;Spiralia;Lophotrochozoa;Mollusca;Bivalvia;Autobranchia;Heteroconchia;Euheterodonta;Imparidentia;Venerida;Veneroidea;Veneridae;Mercenaria;Mercenaria mercenaria
UdeM00000021399 7574 105.9 UdeM00000021399 XP_013401097.1 53.6 112 51 1 6 117 544 654 3.60E-20 105.9 7574 cellular organisms;Eukaryota;Opisthokonta;Metazoa;Eumetazoa;Bilateria;Protostomia;Spiralia;Lophotrochozoa;Brachiopoda;Linguliformea;Lingulata;Lingulida;Linguloidea;Lingulidae;Lingula;Lingula anatina
UdeM00000021400 6596 65.5 UdeM00000021400 XP_045202346.1 33 109 69 2 6 113 16 121 5.10E-08 65.5 6596 cellular organisms;Eukaryota;Opisthokonta;Metazoa;Eumetazoa;Bilateria;Protostomia;Spiralia;Lophotrochozoa;Mollusca;Bivalvia;Autobranchia;Heteroconchia;Euheterodonta;Imparidentia;Venerida;Veneroidea;Veneridae;Mercenaria;Mercenaria mercenaria
UdeM00000021401 6596 253.1 UdeM00000021401 XP_045183440.1 52.9 240 108 3 2 240 145 380 5.20E-64 253.1 6596 cellular organisms;Eukaryota;Opisthokonta;Metazoa;Eumetazoa;Bilateria;Protostomia;Spiralia;Lophotrochozoa;Mollusca;Bivalvia;Autobranchia;Heteroconchia;Euheterodonta;Imparidentia;Venerida;Veneroidea;Veneridae;Mercenaria;Mercenaria mercenaria
UdeM00000021402 114398 228.8 UdeM00000021402 XP_042903861.1 27.7 607 252 7 1 425 1 602 1.30E-56 228.8 114398 cellular organisms;Eukaryota;Opisthokonta;Metazoa;Eumetazoa;Bilateria;Protostomia;Ecdysozoa;Panarthropoda;Arthropoda;Chelicerata;Arachnida;Araneae;Araneomorphae;Entelegynae;Orbiculariae;Araneoidea;Theridiidae;Parasteatoda;Parasteatoda tepidariorum
UdeM00000021403 6596 231.9 UdeM00000021403 XP_045195710.1 51.4 220 107 0 43 262 36 255 1.10E-57 231.9 6596 cellular organisms;Eukaryota;Opisthokonta;Metazoa;Eumetazoa;Bilateria;Protostomia;Spiralia;Lophotrochozoa;Mollusca;Bivalvia;Autobranchia;Heteroconchia;Euheterodonta;Imparidentia;Venerida;Veneroidea;Veneridae;Mercenaria;Mercenaria mercenaria
UdeM00000021404 6579 282.3 UdeM00000021404 XP_033748359.1 45.8 319 170 2 13 328 378 696 1.10E-72 282.3 6579 cellular organisms;Eukaryota;Opisthokonta;Metazoa;Eumetazoa;Bilateria;Protostomia;Spiralia;Lophotrochozoa;Mollusca;Bivalvia;Autobranchia;Pteriomorphia;Pectinida;Pectinoidea;Pectinidae;Pecten;Pecten maximus
UdeM00000021405 90988 171.4 UdeM00000021405 XP_039535177.1 25 608 393 16 43 600 28 622 3.50E-39 171.4 90988 cellular organisms;Eukaryota;Opisthokonta;Metazoa;Eumetazoa;Bilateria;Deuterostomi

a;Chordata;Craniata;Vertebrata;Gnathostomata;Teleostomi;Euteleostomi;Actinopterygii;Actinopteri;Neopterygii;Teleostei;Osteoglossocephalai;Clupeocephala;Otomorpha;Ostariophysi;Otophysi;Cypriniphysae;Cypriniformes;Cyprinoidei;Leuciscidae;Pogonichthyinae;Pimephales;Pimephales promelas
UdeM00000021407 6573 783.1 UdeM00000021407 XP_021364536.1 83.6 432 71 0 1 432 1 432 2.10E-223 783.1 6573 cellular organisms;Eukaryota;Opisthokonta;Metazoa;Eumetazoa;Bilateria;Protostomia;Spiralia;Lophotrochozoa;Mollusca;Bivalvia;Autobranchia;Pteriomorphia;Pectinida;Pectinoidea;Pectinidae;Mizuhopecten;Mizuhopecten yessoensis
UdeM00000021409 6596 58.9 UdeM00000021409 XP_045195711.1 43.3 67 36 1 15 81 1 65 3.50E-06 58.9 6596 cellular organisms;Eukaryota;Opisthokonta;Metazoa;Eumetazoa;Bilateria;Protostomia;Spiralia;Lophotrochozoa;Mollusca;Bivalvia;Autobranchia;Heteroconchia;Euheterodonta;Imparidentia;Venerida;Veneroidea;Veneridae;Mercenaria;Mercenaria mercenaria
UdeM00000021408 6596 281.6 UdeM00000021408 XP_045195711.1 51.1 278 134 1 15 292 1 276 1.20E-72 281.6 6596 cellular organisms;Eukaryota;Opisthokonta;Metazoa;Eumetazoa;Bilateria;Protostomia;Spiralia;Lophotrochozoa;Mollusca;Bivalvia;Autobranchia;Heteroconchia;Euheterodonta;Imparidentia;Venerida;Veneroidea;Veneridae;Mercenaria;Mercenaria mercenaria
UdeM00000021411 6596 215.3 UdeM00000021411 XP_045195710.1 50 214 107 0 3 216 71 284 7.80E-53 215.3 6596 cellular organisms;Eukaryota;Opisthokonta;Metazoa;Eumetazoa;Bilateria;Protostomia;Spiralia;Lophotrochozoa;Mollusca;Bivalvia;Autobranchia;Heteroconchia;Euheterodonta;Imparidentia;Venerida;Veneroidea;Veneridae;Mercenaria;Mercenaria mercenaria
UdeM00000021410 6596 267.3 UdeM00000021410 XP_045195711.1 48.9 272 131 1 104 367 7 278 2.90E-68 267.3 6596 cellular organisms;Eukaryota;Opisthokonta;Metazoa;Eumetazoa;Bilateria;Protostomia;Spiralia;Lophotrochozoa;Mollusca;Bivalvia;Autobranchia;Heteroconchia;Euheterodonta;Imparidentia;Venerida;Veneroidea;Veneridae;Mercenaria;Mercenaria mercenaria
UdeM00000021412 6596 255.8 UdeM00000021412 XP_045195710.1 55.1 225 94 1 15 232 35 259 6.20E-65 255.8 6596 cellular organisms;Eukaryota;Opisthokonta;Metazoa;Eumetazoa;Bilateria;Protostomia;Spiralia;Lophotrochozoa;Mollusca;Bivalvia;Autobranchia;Heteroconchia;Euheterodonta;Imparidentia;Venerida;Veneroidea;Veneridae;Mercenaria;Mercenaria mercenaria
UdeM00000021413 6565 155.6 UdeM00000021413 XP_022320952.1 47.3 186 72 3 12 171 321 506 7.30E-35 155.6 6565 cellular organisms;Eukaryota;Opisthokonta;Metazoa;Eumetazoa;Bilateria;Protostomia;Spiralia;Lophotrochozoa;Mollusca;Bivalvia;Autobranchia;Pteriomorphia;Ostreida;Ostreoidea;Ostreidae;Crassostrea;Crassostrea virginica
UdeM00000021414 37653 98.2 UdeM00000021414 XP_014784355.1 60 80 31 1 34 112 429 508 9.60E-18 98.2 37653 cellular organisms;Eukaryota;Opisthokonta;Metazoa;Eumetazoa;Bilateria;Protostomia;Spiralia;Lophotrochozoa;Mollusca;Cephalopoda;Coleoidea;Octopodiformes;Octopoda;Incirrata;Octopodidae;Octopus;Octopus bimaculoides
UdeM00000021415 37653 97.8 UdeM00000021415 XP_014784355.1 60 80 31 1 33 111 429 508 1.20E-17 97.8 37653 cellular organisms;Eukaryota;Opisthokonta;Metazoa;Eumetazoa;Bilateria;Protostomia;Spiralia;Lophotrochozoa;Mollusca;Cephalopoda;Coleoidea;Octopodiformes;Octopoda;Incirrata;Octopodidae;Octopus;Octopus bimaculoides

UdeM00000021416 29159 115.9 UdeM00000021416 XP_034322541.1 25.6 433
242 19 58 453 203 592 1.40E-22 115.9 29159 cellular
organisms;Eukaryota;Opisthokonta;Metazoa;Eumetazoa;Bilateria;Protostomia;Spiralia;Lophotrochozoa;Mollusca;Bivalvia;Autobranchia;Pteriomorphia;Ostreida;Ostreoidea;Ostreidae;Crassostrea;Crassostrea gigas
UdeM00000021419 225164 123.6 UdeM00000021419 XP_009061770.1 40.6
155 38 1 23 123 99 253 1.70E-25 123.6 225164
cellular
organisms;Eukaryota;Opisthokonta;Metazoa;Eumetazoa;Bilateria;Protostomia;Spiralia;Lophotrochozoa;Mollusca;Gastropoda;Patellogastropoda;Lottioidea;Lottiidae;Lottia;Lottia gigantea
UdeM00000021420 7462 78.6 UdeM00000021420 XP_031368538.1 31.2 144
97 1 14 155 468 611 8.00E-12 78.6 7462 cellular
organisms;Eukaryota;Opisthokonta;Metazoa;Eumetazoa;Bilateria;Protostomia;Ecdysozoa;Panarthropoda;Arthropoda;Mandibulata;Pancrustacea;Hexapoda;Insecta;Dicondylia;Pterygota;Neoptera;Endopterygota;Hymenoptera;Apocrita;Aculeata;Apoidea;Apidae;Apinae;Apini;Apis;Apis dorsata
UdeM00000021422 6596 412.9 UdeM00000021422 XP_045180006.1 52.1 436
177 3 2 408 1 433 4.80E-112 412.9 6596 cellular
organisms;Eukaryota;Opisthokonta;Metazoa;Eumetazoa;Bilateria;Protostomia;Spiralia;Lophotrochozoa;Mollusca;Bivalvia;Autobranchia;Heteroconchia;Euheterodonta;Imparidentia;Venerida;Veneroidea;Veneridae;Mercenaria;Mercenaria mercenaria
UdeM00000021423 6596 362.5 UdeM00000021423 XP_045156265.1 41.9 511
245 7 5 509 11 475 1.00E-96 362.5 6596 cellular
organisms;Eukaryota;Opisthokonta;Metazoa;Eumetazoa;Bilateria;Protostomia;Spiralia;Lophotrochozoa;Mollusca;Bivalvia;Autobranchia;Heteroconchia;Euheterodonta;Imparidentia;Venerida;Veneroidea;Veneridae;Mercenaria;Mercenaria mercenaria
UdeM00000021424 6596 89 UdeM00000021424 XP_045156267.1 58 88
32 3 71 154 100 186 6.00E-15 89 6596 cellular
organisms;Eukaryota;Opisthokonta;Metazoa;Eumetazoa;Bilateria;Protostomia;Spiralia;Lophotrochozoa;Mollusca;Bivalvia;Autobranchia;Heteroconchia;Euheterodonta;Imparidentia;Venerida;Veneroidea;Veneridae;Mercenaria;Mercenaria mercenaria
UdeM00000021425 6596 511.1 UdeM00000021425 XP_045216984.1 81.9 287
52 0 1 287 1 287 9.40E-142 511.1 6596 cellular
organisms;Eukaryota;Opisthokonta;Metazoa;Eumetazoa;Bilateria;Protostomia;Spiralia;Lophotrochozoa;Mollusca;Bivalvia;Autobranchia;Heteroconchia;Euheterodonta;Imparidentia;Venerida;Veneroidea;Veneridae;Mercenaria;Mercenaria mercenaria
UdeM00000021426 7936 71.2 UdeM00000021426 XP_035277210.1 70.8 48
13 1 3 50 2 48 8.00E-10 71.2 7936 cellular
organisms;Eukaryota;Opisthokonta;Metazoa;Eumetazoa;Bilateria;Deuterostomia;Chordata;Craniata;Vertebrata;Gnathostomata;Teleostomi;Euteleostomi;Actinopterygii;Actinopteri;Neopterygii;Teleostei;Elopocephalai;Elopocephala;Elopomorpha;Anguilliformes;Anguillidae;Anguilla;Anguilla anguilla
UdeM00000021427 45351 248.4 UdeM00000021427 XP_001631596.1 57.8 192
81 0 18 209 64 255 7.80E-63 248.4 45351 cellular
organisms;Eukaryota;Opisthokonta;Metazoa;Eumetazoa;Cnidaria;Anthozoa;Hexacorallia;Actiniaria;Edwardsiidae;Nematostella;Nematostella vectensis
UdeM00000021429 6596 119 UdeM00000021429 XP_045216977.1 74.7 75
14 1 17 86 2 76 3.20E-24 119 6596 cellular
organisms;Eukaryota;Opisthokonta;Metazoa;Eumetazoa;Bilateria;Protostomia;

Spiralia;Lophotrochozoa;Mollusca;Bivalvia;Autobranchia;Heteroconchia;Euheterodonta;Imparidentia;Venerida;Veneroidea;Veneridae;Mercenaria;Mercenaria mercenaria
UdeM00000021428 6596 148.3 UdeM00000021428 XP_045216977.1 88.2 76 9 0 1 76 1 76 4.40E-33 148.3 6596 cellular organisms;Eukaryota;Opisthokonta;Metazoa;Eumetazoa;Bilateria;Protostomia;Spiralia;Lophotrochozoa;Mollusca;Bivalvia;Autobranchia;Heteroconchia;Euheterodonta;Imparidentia;Venerida;Veneroidea;Veneridae;Mercenaria;Mercenaria mercenaria
UdeM00000021430 6596 562.4 UdeM00000021430 XP_045215956.1 53.3 598 152 12 1 589 1 480 6.90E-157 562.4 6596 cellular organisms;Eukaryota;Opisthokonta;Metazoa;Eumetazoa;Bilateria;Protostomia;Spiralia;Lophotrochozoa;Mollusca;Bivalvia;Autobranchia;Heteroconchia;Euheterodonta;Imparidentia;Venerida;Veneroidea;Veneridae;Mercenaria;Mercenaria mercenaria
UdeM00000021431 7574 235.3 UdeM00000021431 XP_013415139.1 49.2 254 116 4 1 241 1 254 7.90E-59 235.3 7574 cellular organisms;Eukaryota;Opisthokonta;Metazoa;Eumetazoa;Bilateria;Protostomia;Spiralia;Lophotrochozoa;Brachiopoda;Linguliformea;Lingulata;Lingulida;Linguloidea;Lingulidae;Lingula;Lingula anatina
UdeM00000021432 130825 62.4 UdeM00000021432 XP_040846503.1 52.3 44 21 0 3 46 1 44 2.60E-07 62.4 130825 cellular organisms;Eukaryota;Opisthokonta;Metazoa;Eumetazoa;Bilateria;Deuterostomia;Chordata;Craniata;Vertebrata;Gnathostomata;Teleostomi;Euteleostomi;Sarcopterygii;Dipnotetrapodomorpha;Tetrapoda;Amniota;Mammalia;Theria;Eutheria;Boreoeutheria;Euarchontoglires;Glires;Lagomorpha;Ochotonidae;Ochotona;Ochotona curzoniae
UdeM00000021433 6579 378.3 UdeM00000021433 XP_033750909.1 43.5 451 233 6 28 458 29 477 1.50E-101 378.3 6579 cellular organisms;Eukaryota;Opisthokonta;Metazoa;Eumetazoa;Bilateria;Protostomia;Spiralia;Lophotrochozoa;Mollusca;Bivalvia;Autobranchia;Pteriomorphia;Pectinida;Pectinoidea;Pectinidae;Pecten;Pecten maximus
UdeM00000021434 6573 1753.4 UdeM00000021434 XP_021340399.1 70.4 1462 407 15 140 1577 130 1589 0.00E+00 1753.4 6573 cellular organisms;Eukaryota;Opisthokonta;Metazoa;Eumetazoa;Bilateria;Protostomia;Spiralia;Lophotrochozoa;Mollusca;Bivalvia;Autobranchia;Pteriomorphia;Pectinida;Pectinoidea;Pectinidae;Mizuhopecten;Mizuhopecten yessoensis
UdeM00000021436 6500 222.6 UdeM00000021436 XP_035824079.1 41.7 276 149 4 1 264 106 381 8.20E-55 222.6 6500 cellular organisms;Eukaryota;Opisthokonta;Metazoa;Eumetazoa;Bilateria;Protostomia;Spiralia;Lophotrochozoa;Mollusca;Gastropoda;Heterobranchia;Euthyneura;Tectipleura;Aplysiida;Aplysioidea;Aplysiidae;Aplysia;Aplysia californica
UdeM00000021439 6579 163.3 UdeM00000021439 XP_033749843.1 30 526 285 16 22 496 28 521 7.90E-37 163.3 6579 cellular organisms;Eukaryota;Opisthokonta;Metazoa;Eumetazoa;Bilateria;Protostomia;Spiralia;Lophotrochozoa;Mollusca;Bivalvia;Autobranchia;Pteriomorphia;Pectinida;Pectinoidea;Pectinidae;Pecten;Pecten maximus
UdeM00000021440 6596 141.4 UdeM00000021440 XP_045215567.1 61.6 99 38 0 9 107 261 359 1.20E-30 141.4 6596 cellular organisms;Eukaryota;Opisthokonta;Metazoa;Eumetazoa;Bilateria;Protostomia;Spiralia;Lophotrochozoa;Mollusca;Bivalvia;Autobranchia;Heteroconchia;Euhe

terodonta;Imparidentia;Venerida;Veneroidea;Veneridae;Mercenaria;Mercenari
a mercenaria
UdeM00000021441 6596 138.7 UdeM00000021441 XP_045215434.1 34.1 211
139 0 73 283 15 225 1.30E-29 138.7 6596 cellular
organisms;Eukaryota;Opisthokonta;Metazoa;Eumetazoa;Bilateria;Protostomia;
Spiralia;Lophotrochozoa;Mollusca;Bivalvia;Autobranchia;Heteroconchia;Euhe
terodonta;Imparidentia;Venerida;Veneroidea;Veneridae;Mercenaria;Mercenari
a mercenaria
UdeM00000021442 6596 145.2 UdeM00000021442 XP_045215567.1 52.7 131
60 1 5 135 136 264 8.70E-32 145.2 6596 cellular
organisms;Eukaryota;Opisthokonta;Metazoa;Eumetazoa;Bilateria;Protostomia;
Spiralia;Lophotrochozoa;Mollusca;Bivalvia;Autobranchia;Heteroconchia;Euhe
terodonta;Imparidentia;Venerida;Veneroidea;Veneridae;Mercenaria;Mercenari
a mercenaria
UdeM00000021443 6596 163.7 UdeM00000021443 XP_045215567.1 54.1 135
62 0 3 137 1 135 1.70E-37 163.7 6596 cellular
organisms;Eukaryota;Opisthokonta;Metazoa;Eumetazoa;Bilateria;Protostomia;
Spiralia;Lophotrochozoa;Mollusca;Bivalvia;Autobranchia;Heteroconchia;Euhe
terodonta;Imparidentia;Venerida;Veneroidea;Veneridae;Mercenaria;Mercenari
a mercenaria
UdeM00000021444 6596 148.3 UdeM00000021444 XP_045216867.1 39.1 235
120 6 11 232 35 259 1.20E-32 148.3 6596 cellular
organisms;Eukaryota;Opisthokonta;Metazoa;Eumetazoa;Bilateria;Protostomia;
Spiralia;Lophotrochozoa;Mollusca;Bivalvia;Autobranchia;Heteroconchia;Euhe
terodonta;Imparidentia;Venerida;Veneroidea;Veneridae;Mercenaria;Mercenari
a mercenaria
UdeM00000021448 6596 716.1 UdeM00000021448 XP_045216892.1 68.7 504
157 1 3 506 415 917 3.20E-203 716.1 6596 cellular
organisms;Eukaryota;Opisthokonta;Metazoa;Eumetazoa;Bilateria;Protostomia;
Spiralia;Lophotrochozoa;Mollusca;Bivalvia;Autobranchia;Heteroconchia;Euhe
terodonta;Imparidentia;Venerida;Veneroidea;Veneridae;Mercenaria;Mercenari
a mercenaria
UdeM00000021447 6596 870.5 UdeM00000021447 XP_045216892.1 65.3 660
198 3 79 737 288 917 1.50E-249 870.5 6596 cellular
organisms;Eukaryota;Opisthokonta;Metazoa;Eumetazoa;Bilateria;Protostomia;
Spiralia;Lophotrochozoa;Mollusca;Bivalvia;Autobranchia;Heteroconchia;Euhe
terodonta;Imparidentia;Venerida;Veneroidea;Veneridae;Mercenaria;Mercenari
a mercenaria
UdeM00000021449 6565 156.4 UdeM00000021449 XP_022333402.1 80.9 94
18 0 2 95 773 866 2.10E-35 156.4 6565 cellular
organisms;Eukaryota;Opisthokonta;Metazoa;Eumetazoa;Bilateria;Protostomia;
Spiralia;Lophotrochozoa;Mollusca;Bivalvia;Autobranchia;Pteriomorphia;Ostr
eida;Ostreoidea;Ostreidae;Crassostrea;Crassostrea virginica
UdeM00000021450 6565 169.9 UdeM00000021450 XP_022297037.1 43.6 218
116 3 6 218 11 226 3.70E-39 169.9 6565 cellular
organisms;Eukaryota;Opisthokonta;Metazoa;Eumetazoa;Bilateria;Protostomia;
Spiralia;Lophotrochozoa;Mollusca;Bivalvia;Autobranchia;Pteriomorphia;Ostr
eida;Ostreoidea;Ostreidae;Crassostrea;Crassostrea virginica
UdeM00000021451 6596 186.4 UdeM00000021451 XP_045216892.1 52.4 185
25 1 36 157 103 287 2.70E-44 186.4 6596 cellular
organisms;Eukaryota;Opisthokonta;Metazoa;Eumetazoa;Bilateria;Protostomia;
Spiralia;Lophotrochozoa;Mollusca;Bivalvia;Autobranchia;Heteroconchia;Euhe
terodonta;Imparidentia;Venerida;Veneroidea;Veneridae;Mercenaria;Mercenari
a mercenaria

UdeM00000021452 6596 127.9 UdeM00000021452 XP_045216209.1 43.6 179
99 2 1 179 1 177 2.20E-26 127.9 6596 cellular
organisms;Eukaryota;Opisthokonta;Metazoa;Eumetazoa;Bilateria;Protostomia;
Spiralia;Lophotrochozoa;Mollusca;Bivalvia;Autobranchia;Heteroconchia;Euhe
terodonta;Imparidentia;Venerida;Veneroidea;Veneridae;Mercenaria;Mercenari
a mercenaria
UdeM00000021454 6565 159.1 UdeM00000021454 XP_022328252.1 44.5 211
99 5 1 193 1105 1315 5.80E-36 159.1 6565 cellular
organisms;Eukaryota;Opisthokonta;Metazoa;Eumetazoa;Bilateria;Protostomia;
Spiralia;Lophotrochozoa;Mollusca;Bivalvia;Autobranchia;Pteriomorphia;Ostr
eida;Ostreoidea;Ostreidae;Crassostrea;Crassostrea virginica
UdeM00000021455 29159 1224.9 UdeM00000021455 XP_034322339.1 52.2
1183 519 15 1 1144 142 1316 0.00E+00 1224.9
29159 cellular
organisms;Eukaryota;Opisthokonta;Metazoa;Eumetazoa;Bilateria;Protostomia;
Spiralia;Lophotrochozoa;Mollusca;Bivalvia;Autobranchia;Pteriomorphia;Ostr
eida;Ostreoidea;Ostreidae;Crassostrea;Crassostrea gigas
UdeM00000021456 6596 120.6 UdeM00000021456 XP_045216209.1 42.9 175
98 2 1 175 1 173 3.60E-24 120.6 6596 cellular
organisms;Eukaryota;Opisthokonta;Metazoa;Eumetazoa;Bilateria;Protostomia;
Spiralia;Lophotrochozoa;Mollusca;Bivalvia;Autobranchia;Heteroconchia;Euhe
terodonta;Imparidentia;Venerida;Veneroidea;Veneridae;Mercenaria;Mercenari
a mercenaria
UdeM00000021457 6596 118.2 UdeM00000021457 XP_045216209.1 35.9 220
135 4 1 218 1 216 1.80E-23 118.2 6596 cellular
organisms;Eukaryota;Opisthokonta;Metazoa;Eumetazoa;Bilateria;Protostomia;
Spiralia;Lophotrochozoa;Mollusca;Bivalvia;Autobranchia;Heteroconchia;Euhe
terodonta;Imparidentia;Venerida;Veneroidea;Veneridae;Mercenaria;Mercenari
a mercenaria
UdeM00000021458 42526 71.6 UdeM00000021458 XP_036431265.1 49.3 67
33 1 13 79 87 152 7.60E-10 71.6 42526 cellular
organisms;Eukaryota;Opisthokonta;Metazoa;Eumetazoa;Bilateria;Deuterostomi
a;Chordata;Craniata;Vertebrata;Gnathostomata;Teleostomi;Euteleostomi;Acti
nopterygii;Actinopteri;Neopterygii;Teleostei;Osteoglossocephalai;Clupeoce
phala;Otomorpha;Ostariophysi;Otophysi;Characiphysae;Characiformes;Characo
idei;Serrasalmidae;Colossoma;Colossoma macropomum
UdeM00000021459 6573 157.5 UdeM00000021459 XP_021368610.1 66.1 115
39 0 11 125 12 126 1.60E-35 157.5 6573 cellular
organisms;Eukaryota;Opisthokonta;Metazoa;Eumetazoa;Bilateria;Protostomia;
Spiralia;Lophotrochozoa;Mollusca;Bivalvia;Autobranchia;Pteriomorphia;Pect
inida;Pectinoidea;Pectinidae;Mizuhopecten;Mizuhopecten yessoensis
UdeM00000021460 6565 401.7 UdeM00000021460 XP_022322482.1 47.5 438
206 4 37 463 12 436 1.40E-108 401.7 6565 cellular
organisms;Eukaryota;Opisthokonta;Metazoa;Eumetazoa;Bilateria;Protostomia;
Spiralia;Lophotrochozoa;Mollusca;Bivalvia;Autobranchia;Pteriomorphia;Ostr
eida;Ostreoidea;Ostreidae;Crassostrea;Crassostrea virginica
UdeM00000021461 6565 132.5 UdeM00000021461 XP_022299727.1 49.6 133
66 1 4 136 140 271 4.20E-28 132.5 6565 cellular
organisms;Eukaryota;Opisthokonta;Metazoa;Eumetazoa;Bilateria;Protostomia;
Spiralia;Lophotrochozoa;Mollusca;Bivalvia;Autobranchia;Pteriomorphia;Ostr
eida;Ostreoidea;Ostreidae;Crassostrea;Crassostrea virginica
UdeM00000021462 133901 162.2 UdeM00000021462 XP_026279040.1 59.8
127 51 0 29 155 448 574 5.50E-37 162.2 133901
cellular

organisms;Eukaryota;Opisthokonta;Metazoa;Eumetazoa;Bilateria;Protostomia;Ecdysozoa;Panarthropoda;Arthropoda;Mandibulata;Pancrustacea;Hexapoda;Insecta;Dicondylia;Pterygota;Neoptera;Paraneoptera;Thysanoptera;Terebrantia;Thripoidea;Thripidae;Thripinae;Frankliniella;Frankliniella occidentalis
UdeM00000021463 29159 888.3 UdeM00000021463 XP_034300520.1 53.1 892 397 5 34 922 443 1316 8.90E-255 888.3 29159 cellular organisms;Eukaryota;Opisthokonta;Metazoa;Eumetazoa;Bilateria;Protostomia;Spiralia;Lophotrochozoa;Mollusca;Bivalvia;Autobranchia;Pteriomorphia;Ostreida;Ostreoidea;Ostreidae;Crassostrea;Crassostrea gigas
UdeM00000021464 6573 287.7 UdeM00000021464 XP_021362287.1 41.8 335 185 5 25 356 30 357 2.00E-74 287.7 6573 cellular organisms;Eukaryota;Opisthokonta;Metazoa;Eumetazoa;Bilateria;Protostomia;Spiralia;Lophotrochozoa;Mollusca;Bivalvia;Autobranchia;Pteriomorphia;Pectinida;Pectinoidea;Pectinidae;Mizuhopecten;Mizuhopecten yessoensis
UdeM00000021465 6573 139 UdeM00000021465 XP_021345906.1 41.2 250 122 5 101 343 113 344 1.10E-29 139 6573 cellular organisms;Eukaryota;Opisthokonta;Metazoa;Eumetazoa;Bilateria;Protostomia;Spiralia;Lophotrochozoa;Mollusca;Bivalvia;Autobranchia;Pteriomorphia;Pectinida;Pectinoidea;Pectinidae;Mizuhopecten;Mizuhopecten yessoensis
UdeM00000021466 1735272 583.6 UdeM00000021466 XP_041367912.1 57.9 451 188 2 41 489 112 562 2.40E-163 583.6 1735272 cellular organisms;Eukaryota;Opisthokonta;Metazoa;Eumetazoa;Bilateria;Protostomia;Spiralia;Lophotrochozoa;Mollusca;Gastropoda;Neomphalina;Neomphaloidea;Peltospiridae;Gigantopelta;Gigantopelta aegis
UdeM00000021468 6573 260.4 UdeM00000021468 XP_021348468.1 64.3 199 70 1 1 199 42 239 1.90E-66 260.4 6573 cellular organisms;Eukaryota;Opisthokonta;Metazoa;Eumetazoa;Bilateria;Protostomia;Spiralia;Lophotrochozoa;Mollusca;Bivalvia;Autobranchia;Pteriomorphia;Pectinida;Pectinoidea;Pectinidae;Mizuhopecten;Mizuhopecten yessoensis
UdeM00000021469 6573 321.6 UdeM00000021469 XP_021348468.1 65.4 240 82 1 1 240 1 239 8.40E-85 321.6 6573 cellular organisms;Eukaryota;Opisthokonta;Metazoa;Eumetazoa;Bilateria;Protostomia;Spiralia;Lophotrochozoa;Mollusca;Bivalvia;Autobranchia;Pteriomorphia;Pectinida;Pectinoidea;Pectinidae;Mizuhopecten;Mizuhopecten yessoensis
UdeM00000021470 6573 149.8 UdeM00000021470 XP_021365617.1 49.1 173 85 2 2 174 178 347 3.30E-33 149.8 6573 cellular organisms;Eukaryota;Opisthokonta;Metazoa;Eumetazoa;Bilateria;Protostomia;Spiralia;Lophotrochozoa;Mollusca;Bivalvia;Autobranchia;Pteriomorphia;Pectinida;Pectinoidea;Pectinidae;Mizuhopecten;Mizuhopecten yessoensis
UdeM00000021471 8128 63.2 UdeM00000021471 XP_019206542.2 41.5 82 41 2 59 133 106 187 3.20E-07 63.2 8128 cellular organisms;Eukaryota;Opisthokonta;Metazoa;Eumetazoa;Bilateria;Deuterostomia;Chordata;Craniata;Vertebrata;Gnathostomata;Teleostomi;Euteleostomi;Actinopterygii;Actinopteri;Neopterygii;Teleostei;Osteoglossocephalai;Clupeocephala;Euteleosteomorpha;Neoteleostei;Eurypterygia;Ctenosquamata;Acanthomorphata;Euacanthomorphacea;Percomorphaceae;Ovalentaria;Cichlomorphae;Cichliformes;Cichlidae;African cichlids;Pseudocrenilabrinae;Oreochromini;Oreochromis;Oreochromis niloticus
UdeM00000021474 6579 275.8 UdeM00000021474 XP_033741736.1 54.9 244 108 1 1 242 614 857 5.60E-71 275.8 6579 cellular organisms;Eukaryota;Opisthokonta;Metazoa;Eumetazoa;Bilateria;Protostomia;

Spiralia;Lophotrochozoa;Mollusca;Bivalvia;Autobranchia;Pteriomorphia;Pectinida;Pectinoidea;Pectinidae;Pecten;Pecten maximus
UdeM00000021473 6579 562 UdeM00000021473 XP_033741736.1 59.6 438 175 1 2 437 420 857 6.90E-157 562 6579 cellular organisms;Eukaryota;Opisthokonta;Metazoa;Eumetazoa;Bilateria;Protostomia;Spiralia;Lophotrochozoa;Mollusca;Bivalvia;Autobranchia;Pteriomorphia;Pectinida;Pectinoidea;Pectinidae;Pecten;Pecten maximus
UdeM00000021475 29159 241.1 UdeM00000021475 XP_019926412.2 53.4 238 100 3 1 235 1 230 1.40E-60 241.1 29159 cellular organisms;Eukaryota;Opisthokonta;Metazoa;Eumetazoa;Bilateria;Protostomia;Spiralia;Lophotrochozoa;Mollusca;Bivalvia;Autobranchia;Pteriomorphia;Ostreida;Ostreoidea;Ostreidae;Crassostrea;Crassostrea gigas
UdeM00000021476 6596 178.7 UdeM00000021476 XP_045216225.1 46 409 204 9 48 447 400 800 1.60E-41 178.7 6596 cellular organisms;Eukaryota;Opisthokonta;Metazoa;Eumetazoa;Bilateria;Protostomia;Spiralia;Lophotrochozoa;Mollusca;Bivalvia;Autobranchia;Heteroconchia;Euheterodonta;Imparidentia;Venerida;Veneroidea;Veneridae;Mercenaria;Mercenaria mercenaria
UdeM00000021477 6596 533.1 UdeM00000021477 XP_045216224.1 68.3 391 123 1 4 393 11 401 5.00E-148 533.1 6596 cellular organisms;Eukaryota;Opisthokonta;Metazoa;Eumetazoa;Bilateria;Protostomia;Spiralia;Lophotrochozoa;Mollusca;Bivalvia;Autobranchia;Heteroconchia;Euheterodonta;Imparidentia;Venerida;Veneroidea;Veneridae;Mercenaria;Mercenaria mercenaria
UdeM00000021478 6596 154.8 UdeM00000021478 XP_045215732.1 64.2 134 48 0 2 135 3 136 7.60E-35 154.8 6596 cellular organisms;Eukaryota;Opisthokonta;Metazoa;Eumetazoa;Bilateria;Protostomia;Spiralia;Lophotrochozoa;Mollusca;Bivalvia;Autobranchia;Heteroconchia;Euheterodonta;Imparidentia;Venerida;Veneroidea;Veneridae;Mercenaria;Mercenaria mercenaria
UdeM00000021479 6596 149.8 UdeM00000021479 XP_045215732.1 65.3 118 41 0 1 118 18 135 2.20E-33 149.8 6596 cellular organisms;Eukaryota;Opisthokonta;Metazoa;Eumetazoa;Bilateria;Protostomia;Spiralia;Lophotrochozoa;Mollusca;Bivalvia;Autobranchia;Heteroconchia;Euheterodonta;Imparidentia;Venerida;Veneroidea;Veneridae;Mercenaria;Mercenaria mercenaria
UdeM00000021481 6596 66.6 UdeM00000021481 XP_045215732.1 55.6 72 32 0 1 72 18 89 1.50E-08 66.6 6596 cellular organisms;Eukaryota;Opisthokonta;Metazoa;Eumetazoa;Bilateria;Protostomia;Spiralia;Lophotrochozoa;Mollusca;Bivalvia;Autobranchia;Heteroconchia;Euheterodonta;Imparidentia;Venerida;Veneroidea;Veneridae;Mercenaria;Mercenaria mercenaria
UdeM00000021482 6573 229.9 UdeM00000021482 XP_021362280.1 56.6 221 93 2 1 221 178 395 3.40E-57 229.9 6573 cellular organisms;Eukaryota;Opisthokonta;Metazoa;Eumetazoa;Bilateria;Protostomia;Spiralia;Lophotrochozoa;Mollusca;Bivalvia;Autobranchia;Pteriomorphia;Pectinida;Pectinoidea;Pectinidae;Mizuhopecten;Mizuhopecten yessoensis
UdeM00000021483 6596 189.1 UdeM00000021483 XP_045215725.1 66.7 138 45 1 31 167 24 161 4.50E-45 189.1 6596 cellular organisms;Eukaryota;Opisthokonta;Metazoa;Eumetazoa;Bilateria;Protostomia;Spiralia;Lophotrochozoa;Mollusca;Bivalvia;Autobranchia;Heteroconchia;Euheterodonta;Imparidentia;Venerida;Veneroidea;Veneridae;Mercenaria;Mercenaria mercenaria

UdeM00000021486 6596 3372.4 UdeM00000021486 XP_045215836.1 58.7 3113 1117 47 103 3172 337 3323 0.00E+00 3372.4 6596 cellular organisms;Eukaryota;Opisthokonta;Metazoa;Eumetazoa;Bilateria;Protostomia;Spiralia;Lophotrochozoa;Mollusca;Bivalvia;Autobranchia;Heteroconchia;Euheterodonta;Imparidentia;Venerida;Veneroidea;Veneridae;Mercenaria;Mercenaria mercenaria
UdeM00000021487 29159 519.2 UdeM00000021487 XP_034325757.1 72.9 340 85 3 1 339 1 334 3.90E-144 519.2 29159 cellular organisms;Eukaryota;Opisthokonta;Metazoa;Eumetazoa;Bilateria;Protostomia;Spiralia;Lophotrochozoa;Mollusca;Bivalvia;Autobranchia;Pteriomorphia;Ostreida;Ostreoidea;Ostreidae;Crassostrea;Crassostrea gigas
UdeM00000021488 151771 485.7 UdeM00000021488 XP_028419301.1 54 448 203 3 42 488 34 479 6.80E-134 485.7 151771 cellular organisms;Eukaryota;Opisthokonta;Metazoa;Eumetazoa;Cnidaria;Anthozoa;Octocorallia;Alcyonacea;Alcyoniina;Nephtheidae;Dendronephthya;Dendronephthya gigantea
UdeM00000021489 151771 65.9 UdeM00000021489 XP_028401094.1 37.5 88 50 1 47 134 88 170 4.60E-08 65.9 151771 cellular organisms;Eukaryota;Opisthokonta;Metazoa;Eumetazoa;Cnidaria;Anthozoa;Octocorallia;Alcyonacea;Alcyoniina;Nephtheidae;Dendronephthya;Dendronephthya gigantea
UdeM00000021490 10224 111.3 UdeM00000021490 XP_006814936.1 37.1 170 102 3 127 291 77 246 2.10E-21 111.3 10224 cellular organisms;Eukaryota;Opisthokonta;Metazoa;Eumetazoa;Bilateria;Deuterostomia;Hemichordata;Enteropneusta;Harrimaniidae;Saccoglossus;Saccoglossus kowalevskii
UdeM00000021492 1735272 253.4 UdeM00000021492 XP_041362467.1 54.2 264 107 6 1 255 1 259 3.00E-64 253.4 1735272 cellular organisms;Eukaryota;Opisthokonta;Metazoa;Eumetazoa;Bilateria;Protostomia;Spiralia;Lophotrochozoa;Mollusca;Gastropoda;Neomphalina;Neomphaloidea;Peltospiridae;Gigantopelta;Gigantopelta aegis
UdeM00000021493 6596 259.6 UdeM00000021493 XP_045156669.1 55.1 234 105 0 8 241 6 239 4.00E-66 259.6 6596 cellular organisms;Eukaryota;Opisthokonta;Metazoa;Eumetazoa;Bilateria;Protostomia;Spiralia;Lophotrochozoa;Mollusca;Bivalvia;Autobranchia;Heteroconchia;Euheterodonta;Imparidentia;Venerida;Veneroidea;Veneridae;Mercenaria;Mercenaria mercenaria
UdeM00000021494 400727 119.8 UdeM00000021494 XP_025076802.1 71.6 88 23 1 41 128 69 154 2.60E-24 119.8 400727 cellular organisms;Eukaryota;Opisthokonta;Metazoa;Eumetazoa;Bilateria;Protostomia;Spiralia;Lophotrochozoa;Mollusca;Gastropoda;Caenogastropoda;Architaenioglossa;Ampullarioidea;Ampullariidae;Pomacea;Pomacea canaliculata
UdeM00000021495 1735272 585.5 UdeM00000021495 XP_041368677.1 79.7 364 72 1 1 362 1 364 4.70E-164 585.5 1735272 cellular organisms;Eukaryota;Opisthokonta;Metazoa;Eumetazoa;Bilateria;Protostomia;Spiralia;Lophotrochozoa;Mollusca;Gastropoda;Neomphalina;Neomphaloidea;Peltospiridae;Gigantopelta;Gigantopelta aegis

UdeM00000021496 6573 158.3 UdeM00000021496 XP_021358522.1 97.5 79
2 0 1 79 1 79 4.10E-36 158.3 6573 cellular
organisms;Eukaryota;Opisthokonta;Metazoa;Eumetazoa;Bilateria;Protostomia;
Spiralia;Lophotrochozoa;Mollusca;Bivalvia;Autobranchia;Pteriomorphia;Pect
inida;Pectinoidea;Pectinidae;Mizuhopecten;Mizuhopecten yessoensis
UdeM00000021497 6596 1375.5 UdeM00000021497 XP_045156512.1 49.9
1518 621 31 7 1438 355 1818 0.00E+00 1375.5 6596
cellular
organisms;Eukaryota;Opisthokonta;Metazoa;Eumetazoa;Bilateria;Protostomia;
Spiralia;Lophotrochozoa;Mollusca;Bivalvia;Autobranchia;Heteroconchia;Euhe
terodonta;Imparidentia;Venerida;Veneroidea;Veneridae;Mercenaria;Mercenari
a mercenaria
UdeM00000021498 6596 219.5 UdeM00000021498 XP_045216916.1 51.7 205
97 2 71 273 60 264 5.10E-54 219.5 6596 cellular
organisms;Eukaryota;Opisthokonta;Metazoa;Eumetazoa;Bilateria;Protostomia;
Spiralia;Lophotrochozoa;Mollusca;Bivalvia;Autobranchia;Heteroconchia;Euhe
terodonta;Imparidentia;Venerida;Veneroidea;Veneridae;Mercenaria;Mercenari
a mercenaria
UdeM00000021499 6596 119.8 UdeM00000021499 XP_045204472.1 49.6 115
51 2 5 115 3 114 3.10E-24 119.8 6596 cellular
organisms;Eukaryota;Opisthokonta;Metazoa;Eumetazoa;Bilateria;Protostomia;
Spiralia;Lophotrochozoa;Mollusca;Bivalvia;Autobranchia;Heteroconchia;Euhe
terodonta;Imparidentia;Venerida;Veneroidea;Veneridae;Mercenaria;Mercenari
a mercenaria
UdeM00000021501 6526 198.4 UdeM00000021501 XP_013096252.1 60 160
63 1 11 169 16 175 7.50E-48 198.4 6526 cellular
organisms;Eukaryota;Opisthokonta;Metazoa;Eumetazoa;Bilateria;Protostomia;
Spiralia;Lophotrochozoa;Mollusca;Gastropoda;Heterobranchia;Euthyneura;Pan
pulmonata;Hygrophila;Lymnaeoidea;Planorbidae;Biomphalaria;Biomphalaria
glabrata
UdeM00000021500 29159 198.4 UdeM00000021500 XP_034322993.1 74 123
32 0 67 189 52 174 8.40E-48 198.4 29159 cellular
organisms;Eukaryota;Opisthokonta;Metazoa;Eumetazoa;Bilateria;Protostomia;
Spiralia;Lophotrochozoa;Mollusca;Bivalvia;Autobranchia;Pteriomorphia;Ostr
eida;Ostreoidea;Ostreidae;Crassostrea;Crassostrea gigas
UdeM00000021502 6596 510.4 UdeM00000021502 XP_045215251.1 46.3 557
278 9 30 577 17 561 3.00E-141 510.4 6596 cellular
organisms;Eukaryota;Opisthokonta;Metazoa;Eumetazoa;Bilateria;Protostomia;
Spiralia;Lophotrochozoa;Mollusca;Bivalvia;Autobranchia;Heteroconchia;Euhe
terodonta;Imparidentia;Venerida;Veneroidea;Veneridae;Mercenaria;Mercenari
a mercenaria
UdeM00000021503 6573 929.5 UdeM00000021503 XP_021377196.1 46.3 1090
561 12 5 1085 1 1075 4.00E-267 929.5 6573 cellular
organisms;Eukaryota;Opisthokonta;Metazoa;Eumetazoa;Bilateria;Protostomia;
Spiralia;Lophotrochozoa;Mollusca;Bivalvia;Autobranchia;Pteriomorphia;Pect
inida;Pectinoidea;Pectinidae;Mizuhopecten;Mizuhopecten yessoensis
UdeM00000021504 6596 970.7 UdeM00000021504 XP_045216905.1 40.4 1380
782 23 65 1420 2 1365 2.20E-279 970.7 6596 cellular
organisms;Eukaryota;Opisthokonta;Metazoa;Eumetazoa;Bilateria;Protostomia;
Spiralia;Lophotrochozoa;Mollusca;Bivalvia;Autobranchia;Heteroconchia;Euhe
terodonta;Imparidentia;Venerida;Veneroidea;Veneridae;Mercenaria;Mercenari
a mercenaria
UdeM00000021505 6596 995 UdeM00000021505 XP_045216905.1 40.8 1377
779 22 160 1517 2 1361 1.10E-286 995 6596 cellular

organisms;Eukaryota;Opisthokonta;Metazoa;Eumetazoa;Bilateria;Protostomia;Spiralia;Lophotrochozoa;Mollusca;Bivalvia;Autobranchia;Heteroconchia;Euheterodonta;Imparidentia;Venerida;Veneroidea;Veneridae;Mercenaria;Mercenaria mercenaria
UdeM00000021507 6596 351.3 UdeM00000021507 XP_045156624.1 48.1 372 175 4 30 390 26 390 1.70E-93 351.3 6596 cellular organisms;Eukaryota;Opisthokonta;Metazoa;Eumetazoa;Bilateria;Protostomia;Spiralia;Lophotrochozoa;Mollusca;Bivalvia;Autobranchia;Heteroconchia;Euheterodonta;Imparidentia;Venerida;Veneroidea;Veneridae;Mercenaria;Mercenaria mercenaria
UdeM00000021508 6596 351.7 UdeM00000021508 XP_045156624.1 55.1 301 135 0 30 330 26 326 1.20E-93 351.7 6596 cellular organisms;Eukaryota;Opisthokonta;Metazoa;Eumetazoa;Bilateria;Protostomia;Spiralia;Lophotrochozoa;Mollusca;Bivalvia;Autobranchia;Heteroconchia;Euheterodonta;Imparidentia;Venerida;Veneroidea;Veneridae;Mercenaria;Mercenaria mercenaria
UdeM00000021509 6596 349 UdeM00000021509 XP_045156624.1 49.2 366 168 5 30 384 26 384 8.00E-93 349 6596 cellular organisms;Eukaryota;Opisthokonta;Metazoa;Eumetazoa;Bilateria;Protostomia;Spiralia;Lophotrochozoa;Mollusca;Bivalvia;Autobranchia;Heteroconchia;Euheterodonta;Imparidentia;Venerida;Veneroidea;Veneridae;Mercenaria;Mercenaria mercenaria
UdeM00000021510 6596 297.4 UdeM00000021510 XP_045156624.1 54.8 263 118 1 1 262 59 321 1.90E-77 297.4 6596 cellular organisms;Eukaryota;Opisthokonta;Metazoa;Eumetazoa;Bilateria;Protostomia;Spiralia;Lophotrochozoa;Mollusca;Bivalvia;Autobranchia;Heteroconchia;Euheterodonta;Imparidentia;Venerida;Veneroidea;Veneridae;Mercenaria;Mercenaria mercenaria
UdeM00000021513 6596 635.6 UdeM00000021513 XP_045156574.1 67.5 477 145 2 2 473 377 848 5.30E-179 635.6 6596 cellular organisms;Eukaryota;Opisthokonta;Metazoa;Eumetazoa;Bilateria;Protostomia;Spiralia;Lophotrochozoa;Mollusca;Bivalvia;Autobranchia;Heteroconchia;Euheterodonta;Imparidentia;Venerida;Veneroidea;Veneridae;Mercenaria;Mercenaria mercenaria
UdeM00000021514 6596 133.3 UdeM00000021514 XP_045156574.1 56.6 106 46 0 18 123 207 312 3.00E-28 133.3 6596 cellular organisms;Eukaryota;Opisthokonta;Metazoa;Eumetazoa;Bilateria;Protostomia;Spiralia;Lophotrochozoa;Mollusca;Bivalvia;Autobranchia;Heteroconchia;Euheterodonta;Imparidentia;Venerida;Veneroidea;Veneridae;Mercenaria;Mercenaria mercenaria
UdeM00000021515 29159 139.8 UdeM00000021515 XP_034324132.1 62.4 101 35 1 3 100 17 117 1.90E-30 139.8 29159 cellular organisms;Eukaryota;Opisthokonta;Metazoa;Eumetazoa;Bilateria;Protostomia;Spiralia;Lophotrochozoa;Mollusca;Bivalvia;Autobranchia;Pteriomorphia;Ostreida;Ostreoidea;Ostreidae;Crassostrea;Crassostrea gigas
UdeM00000021516 6565 80.5 UdeM00000021516 XP_022330412.1 49.5 97 42 4 1 93 1 94 1.80E-12 80.5 6565 cellular organisms;Eukaryota;Opisthokonta;Metazoa;Eumetazoa;Bilateria;Protostomia;Spiralia;Lophotrochozoa;Mollusca;Bivalvia;Autobranchia;Pteriomorphia;Ostreida;Ostreoidea;Ostreidae;Crassostrea;Crassostrea virginica
UdeM00000021517 6500 198.4 UdeM00000021517 XP_005094757.1 64.2 137 49 0 5 141 7 143 6.60E-48 198.4 6500 cellular organisms;Eukaryota;Opisthokonta;Metazoa;Eumetazoa;Bilateria;Protostomia;

Spiralia;Lophotrochozoa;Mollusca;Gastropoda;Heterobranchia;Euthyneura;Tectipleura;Aplysiida;Aplysioidea;Aplysiidae;Aplysia;Aplysia californica
UdeM00000021518 6573 656.8 UdeM00000021518 XP_021360480.1 51.9 746
307 12 190 901 436 1163 4.10E-185 656.8 6573 cellular
organisms;Eukaryota;Opisthokonta;Metazoa;Eumetazoa;Bilateria;Protostomia;
Spiralia;Lophotrochozoa;Mollusca;Bivalvia;Autobranchia;Pteriomorphia;Pectinida;Pectinoidea;Pectinidae;Mizuhopecten;Mizuhopecten yessoensis
UdeM00000021519 6573 572 UdeM00000021519 XP_021371985.1 36.4 989
427 18 3 944 15 848 1.40E-159 572 6573 cellular
organisms;Eukaryota;Opisthokonta;Metazoa;Eumetazoa;Bilateria;Protostomia;
Spiralia;Lophotrochozoa;Mollusca;Bivalvia;Autobranchia;Pteriomorphia;Pectinida;Pectinoidea;Pectinidae;Mizuhopecten;Mizuhopecten yessoensis
UdeM00000021520 6579 438 UdeM00000021520 XP_033750349.1 44.1 555
197 5 68 620 5 448 2.10E-119 438 6579 cellular
organisms;Eukaryota;Opisthokonta;Metazoa;Eumetazoa;Bilateria;Protostomia;
Spiralia;Lophotrochozoa;Mollusca;Bivalvia;Autobranchia;Pteriomorphia;Pectinida;Pectinoidea;Pectinidae;Pecten;Pecten maximus
UdeM00000021521 6596 209.5 UdeM00000021521 XP_045216209.1 51.3 197
96 0 1 197 1 197 5.10E-51 209.5 6596 cellular
organisms;Eukaryota;Opisthokonta;Metazoa;Eumetazoa;Bilateria;Protostomia;
Spiralia;Lophotrochozoa;Mollusca;Bivalvia;Autobranchia;Heteroconchia;Euheterodonta;Imparidentia;Venerida;Veneroidea;Veneridae;Mercenaria;Mercenaria mercenaria
UdeM00000021524 6573 96.7 UdeM00000021524 XP_021371671.1 56 91
38 2 39 128 62 151 2.40E-17 96.7 6573 cellular
organisms;Eukaryota;Opisthokonta;Metazoa;Eumetazoa;Bilateria;Protostomia;
Spiralia;Lophotrochozoa;Mollusca;Bivalvia;Autobranchia;Pteriomorphia;Pectinida;Pectinoidea;Pectinidae;Mizuhopecten;Mizuhopecten yessoensis
UdeM00000021525 29159 1024.2 UdeM00000021525 XP_011449960.1 78.1
622 136 0 1 622 1 622 6.80E-296 1024.2
29159 cellular
organisms;Eukaryota;Opisthokonta;Metazoa;Eumetazoa;Bilateria;Protostomia;
Spiralia;Lophotrochozoa;Mollusca;Bivalvia;Autobranchia;Pteriomorphia;Ostreida;Ostreoidea;Ostreidae;Crassostrea;Crassostrea gigas
UdeM00000021526 6596 257.7 UdeM00000021526 XP_045172628.1 34.6 445
250 11 296 733 16 426 4.80E-65 257.7 6596 cellular
organisms;Eukaryota;Opisthokonta;Metazoa;Eumetazoa;Bilateria;Protostomia;
Spiralia;Lophotrochozoa;Mollusca;Bivalvia;Autobranchia;Heteroconchia;Euheterodonta;Imparidentia;Venerida;Veneroidea;Veneridae;Mercenaria;Mercenaria mercenaria
UdeM00000021528 6565 168.3 UdeM00000021528 XP_022294204.1 38 213
127 3 8 217 600 810 1.10E-38 168.3 6565 cellular
organisms;Eukaryota;Opisthokonta;Metazoa;Eumetazoa;Bilateria;Protostomia;
Spiralia;Lophotrochozoa;Mollusca;Bivalvia;Autobranchia;Pteriomorphia;Ostreida;Ostreoidea;Ostreidae;Crassostrea;Crassostrea virginica
UdeM00000021529 1735272 126.7 UdeM00000021529 XP_041368228.1 31.7
303 185 8 2 287 439 736 8.30E-26 126.7 1735272
cellular
organisms;Eukaryota;Opisthokonta;Metazoa;Eumetazoa;Bilateria;Protostomia;
Spiralia;Lophotrochozoa;Mollusca;Gastropoda;Neomphalina;Neomphaloidea;Peltospiridae;Gigantopelta;Gigantopelta aegis
UdeM00000021530 6579 735.3 UdeM00000021530 XP_033749673.1 78.6 579
104 3 167 745 151 709 8.10E-209 735.3 6579 cellular
organisms;Eukaryota;Opisthokonta;Metazoa;Eumetazoa;Bilateria;Protostomia;

Spiralia;Lophotrochozoa;Mollusca;Bivalvia;Autobranchia;Pteriomorphia;Pectinida;Pectinoidea;Pectinidae;Pecten;Pecten maximus
UdeM00000021531 6579 734.9 UdeM00000021531 XP_033749673.1 78.4 580 104 3 167 746 151 709 1.10E-208 734.9 6579 cellular organisms;Eukaryota;Opisthokonta;Metazoa;Eumetazoa;Bilateria;Protostomia;Spiralia;Lophotrochozoa;Mollusca;Bivalvia;Autobranchia;Pteriomorphia;Pectinida;Pectinoidea;Pectinidae;Pecten;Pecten maximus
UdeM00000021532 6596 924.5 UdeM00000021532 XP_045216318.1 56.4 904 353 8 1 887 1 880 1.30E-265 924.5 6596 cellular organisms;Eukaryota;Opisthokonta;Metazoa;Eumetazoa;Bilateria;Protostomia;Spiralia;Lophotrochozoa;Mollusca;Bivalvia;Autobranchia;Heteroconchia;Euheterodonta;Imparidentia;Venerida;Veneroidea;Veneridae;Mercenaria;Mercenaria mercenaria
UdeM00000021533 6579 263.1 UdeM00000021533 XP_033749210.1 70.6 180 48 1 2 176 1048 1227 2.70E-67 263.1 6579 cellular organisms;Eukaryota;Opisthokonta;Metazoa;Eumetazoa;Bilateria;Protostomia;Spiralia;Lophotrochozoa;Mollusca;Bivalvia;Autobranchia;Pteriomorphia;Pectinida;Pectinoidea;Pectinidae;Pecten;Pecten maximus
UdeM00000021534 1735272 342.4 UdeM00000021534 XP_041360632.1 41.4 500 256 13 1 490 1 473 9.40E-91 342.4 1735272 cellular organisms;Eukaryota;Opisthokonta;Metazoa;Eumetazoa;Bilateria;Protostomia;Spiralia;Lophotrochozoa;Mollusca;Gastropoda;Neomphalina;Neomphaloidea;Peltospiridae;Gigantopelta;Gigantopelta aegis
UdeM00000021535 6596 760.8 UdeM00000021535 XP_045216565.1 84.6 455 62 6 1 448 1 454 1.00E-216 760.8 6596 cellular organisms;Eukaryota;Opisthokonta;Metazoa;Eumetazoa;Bilateria;Protostomia;Spiralia;Lophotrochozoa;Mollusca;Bivalvia;Autobranchia;Heteroconchia;Euheterodonta;Imparidentia;Venerida;Veneroidea;Veneridae;Mercenaria;Mercenaria mercenaria
UdeM00000021536 6596 746.5 UdeM00000021536 XP_045216566.1 86 450 56 5 1 443 1 450 1.90E-212 746.5 6596 cellular organisms;Eukaryota;Opisthokonta;Metazoa;Eumetazoa;Bilateria;Protostomia;Spiralia;Lophotrochozoa;Mollusca;Bivalvia;Autobranchia;Heteroconchia;Euheterodonta;Imparidentia;Venerida;Veneroidea;Veneridae;Mercenaria;Mercenaria mercenaria
UdeM00000021537 6573 1137.5 UdeM00000021537 XP_021346116.1 69.8 1027 276 8 1 1001 1 1019 0.00E+00 1137.5 6573 cellular organisms;Eukaryota;Opisthokonta;Metazoa;Eumetazoa;Bilateria;Protostomia;Spiralia;Lophotrochozoa;Mollusca;Bivalvia;Autobranchia;Pteriomorphia;Pectinida;Pectinoidea;Pectinidae;Mizuhopecten;Mizuhopecten yessoensis
UdeM00000021539 6596 422.2 UdeM00000021539 XP_045156650.1 63.2 351 110 6 7 338 21 371 6.40E-115 422.2 6596 cellular organisms;Eukaryota;Opisthokonta;Metazoa;Eumetazoa;Bilateria;Protostomia;Spiralia;Lophotrochozoa;Mollusca;Bivalvia;Autobranchia;Heteroconchia;Euheterodonta;Imparidentia;Venerida;Veneroidea;Veneridae;Mercenaria;Mercenaria mercenaria
UdeM00000021540 6596 542 UdeM00000021540 XP_045156643.1 60 462 174 5 6 457 14 474 7.50E-151 542 6596 cellular organisms;Eukaryota;Opisthokonta;Metazoa;Eumetazoa;Bilateria;Protostomia;Spiralia;Lophotrochozoa;Mollusca;Bivalvia;Autobranchia;Heteroconchia;Euheterodonta;Imparidentia;Venerida;Veneroidea;Veneridae;Mercenaria;Mercenaria mercenaria

UdeM00000021541 6596 224.6 UdeM00000021541 XP_045156638.1 69.1 149
45 1 1 149 233 380 1.30E-55 224.6 6596 cellular
organisms;Eukaryota;Opisthokonta;Metazoa;Eumetazoa;Bilateria;Protostomia;
Spiralia;Lophotrochozoa;Mollusca;Bivalvia;Autobranchia;Heteroconchia;Euhe
terodonta;Imparidentia;Venerida;Veneroidea;Veneridae;Mercenaria;Mercenari
a mercenaria
UdeM00000021542 6596 530 UdeM00000021542 XP_045156643.1 74.1 332
85 1 30 361 31 361 2.80E-147 530 6596 cellular
organisms;Eukaryota;Opisthokonta;Metazoa;Eumetazoa;Bilateria;Protostomia;
Spiralia;Lophotrochozoa;Mollusca;Bivalvia;Autobranchia;Heteroconchia;Euhe
terodonta;Imparidentia;Venerida;Veneroidea;Veneridae;Mercenaria;Mercenari
a mercenaria
UdeM00000021544 6596 221.9 UdeM00000021544 XP_045177916.1 50.2 213
103 3 5 216 446 656 8.10E-55 221.9 6596 cellular
organisms;Eukaryota;Opisthokonta;Metazoa;Eumetazoa;Bilateria;Protostomia;
Spiralia;Lophotrochozoa;Mollusca;Bivalvia;Autobranchia;Heteroconchia;Euhe
terodonta;Imparidentia;Venerida;Veneroidea;Veneridae;Mercenaria;Mercenari
a mercenaria
UdeM00000040755 321614 217.2 UdeM00000040755 XP_001806620.1 47.7
243 121 5 16 253 116 357 2.40E-53 217.2 321614
cellular
organisms;Eukaryota;Opisthokonta;Fungi;Dikarya;Ascomycota;saccharomyceta;
Pezizomycotina;leotiomyceta;dothideomyceta;Dothideomycetes;Pleosporomycet
idae;Pleosporales;Pleosporineae;Phaeosphaeriaceae;Parastagonospora;Parast
agonospora nodorum;Parastagonospora nodorum SN15
UdeM00000043487 6596 500 UdeM00000043487 XP_045212781.1 61.9 436
144 7 1 416 18 451 3.00E-138 500 6596 cellular
organisms;Eukaryota;Opisthokonta;Metazoa;Eumetazoa;Bilateria;Protostomia;
Spiralia;Lophotrochozoa;Mollusca;Bivalvia;Autobranchia;Heteroconchia;Euhe
terodonta;Imparidentia;Venerida;Veneroidea;Veneridae;Mercenaria;Mercenari
a mercenaria
UdeM00000043488 6579 167.5 UdeM00000043488 XP_033731739.1 44.4 196
72 1 4 162 183 378 1.40E-38 167.5 6579 cellular
organisms;Eukaryota;Opisthokonta;Metazoa;Eumetazoa;Bilateria;Protostomia;
Spiralia;Lophotrochozoa;Mollusca;Bivalvia;Autobranchia;Pteriomorphia;Pect
inida;Pectinoidea;Pectinidae;Pecten;Pecten maximus
UdeM00000043490 6596 639.4 UdeM00000043490 XP_045203777.1 51.3 581
277 3 6 580 12 592 4.40E-180 639.4 6596 cellular
organisms;Eukaryota;Opisthokonta;Metazoa;Eumetazoa;Bilateria;Protostomia;
Spiralia;Lophotrochozoa;Mollusca;Bivalvia;Autobranchia;Heteroconchia;Euhe
terodonta;Imparidentia;Venerida;Veneroidea;Veneridae;Mercenaria;Mercenari
a mercenaria
UdeM00000043491 6573 110.5 UdeM00000043491 XP_021352053.1 55.1 89
38 1 1 87 113 201 1.10E-21 110.5 6573 cellular
organisms;Eukaryota;Opisthokonta;Metazoa;Eumetazoa;Bilateria;Protostomia;
Spiralia;Lophotrochozoa;Mollusca;Bivalvia;Autobranchia;Pteriomorphia;Pect
inida;Pectinoidea;Pectinidae;Mizuhopecten;Mizuhopecten yessoensis
UdeM00000043492 6596 585.9 UdeM00000043492 XP_045211712.1 77.2 369
80 1 1 369 1 365 3.80E-164 585.9 6596 cellular
organisms;Eukaryota;Opisthokonta;Metazoa;Eumetazoa;Bilateria;Protostomia;
Spiralia;Lophotrochozoa;Mollusca;Bivalvia;Autobranchia;Heteroconchia;Euhe
terodonta;Imparidentia;Venerida;Veneroidea;Veneridae;Mercenaria;Mercenari
a mercenaria

UdeM00000043495 6596 380.2 UdeM00000043495 XP_045214167.1 76.3 236
56 0 1 236 1 236 2.00E-102 380.2 6596 cellular
organisms;Eukaryota;Opisthokonta;Metazoa;Eumetazoa;Bilateria;Protostomia;
Spiralia;Lophotrochozoa;Mollusca;Bivalvia;Autobranchia;Heteroconchia;Euhe
terodonta;Imparidentia;Venerida;Veneroidea;Veneridae;Mercenaria;Mercenari
a mercenaria
UdeM00000044366 6596 317.8 UdeM00000044366 XP_045201815.1 31.5 759
443 18 77 795 64 785 4.00E-83 317.8 6596 cellular
organisms;Eukaryota;Opisthokonta;Metazoa;Eumetazoa;Bilateria;Protostomia;
Spiralia;Lophotrochozoa;Mollusca;Bivalvia;Autobranchia;Heteroconchia;Euhe
terodonta;Imparidentia;Venerida;Veneroidea;Veneridae;Mercenaria;Mercenari
a mercenaria
UdeM00000044367 6596 126.3 UdeM00000044367 XP_045192805.1 42.2 147
61 1 42 188 37 159 4.00E-26 126.3 6596 cellular
organisms;Eukaryota;Opisthokonta;Metazoa;Eumetazoa;Bilateria;Protostomia;
Spiralia;Lophotrochozoa;Mollusca;Bivalvia;Autobranchia;Heteroconchia;Euhe
terodonta;Imparidentia;Venerida;Veneroidea;Veneridae;Mercenaria;Mercenari
a mercenaria
UdeM00000044368 6596 134 UdeM00000044368 XP_045201815.1 31.5 327
180 8 1 314 4 299 3.20E-28 134 6596 cellular
organisms;Eukaryota;Opisthokonta;Metazoa;Eumetazoa;Bilateria;Protostomia;
Spiralia;Lophotrochozoa;Mollusca;Bivalvia;Autobranchia;Heteroconchia;Euhe
terodonta;Imparidentia;Venerida;Veneroidea;Veneridae;Mercenaria;Mercenari
a mercenaria
UdeM00000044211 1735272 431 UdeM00000044211 XP_041351670.1 58.8
354 142 2 22 375 20 369 1.50E-117 431 1735272
cellular
organisms;Eukaryota;Opisthokonta;Metazoa;Eumetazoa;Bilateria;Protostomia;
Spiralia;Lophotrochozoa;Mollusca;Gastropoda;Neomphalina;Neomphaloidea;Pel
tospiridae;Gigantopelta;Gigantopelta aegis
UdeM00000044212 6596 167.9 UdeM00000044212 XP_045196496.1 29.7 690
396 13 168 843 55 669 5.40E-38 167.9 6596 cellular
organisms;Eukaryota;Opisthokonta;Metazoa;Eumetazoa;Bilateria;Protostomia;
Spiralia;Lophotrochozoa;Mollusca;Bivalvia;Autobranchia;Heteroconchia;Euhe
terodonta;Imparidentia;Venerida;Veneroidea;Veneridae;Mercenaria;Mercenari
a mercenaria
UdeM00000040630 6596 530 UdeM00000040630 XP_045158043.1 45.8 618
324 7 134 746 170 781 4.80E-147 530 6596 cellular
organisms;Eukaryota;Opisthokonta;Metazoa;Eumetazoa;Bilateria;Protostomia;
Spiralia;Lophotrochozoa;Mollusca;Bivalvia;Autobranchia;Heteroconchia;Euhe
terodonta;Imparidentia;Venerida;Veneroidea;Veneridae;Mercenaria;Mercenari
a mercenaria
UdeM00000040631 29159 67 UdeM00000040631 XP_034315266.1 47.6 63
33 0 26 88 237 299 4.70E-08 67 29159 cellular
organisms;Eukaryota;Opisthokonta;Metazoa;Eumetazoa;Bilateria;Protostomia;
Spiralia;Lophotrochozoa;Mollusca;Bivalvia;Autobranchia;Pteriomorphia;Ostr
eida;Ostreoidea;Ostreidae;Crassostrea;Crassostrea gigas
UdeM00000040632 7906 203.4 UdeM00000040632 XP_034783111.1 58.9 168
69 0 61 228 88 255 3.20E-49 203.4 7906 cellular
organisms;Eukaryota;Opisthokonta;Metazoa;Eumetazoa;Bilateria;Deuterostomi
a;Chordata;Craniata;Vertebrata;Gnathostomata;Teleostomi;Euteleostomi;Acti
nopterygii;Actinopteri;Chondrostei;Acipenseriformes;Acipenseroidei;Acipen
seridae;Acipenserinae;Acipenserini;Acipenser;Acipenser ruthenus

UdeM00000040634 6596 208.8 UdeM00000040634 XP_045170374.1 47.2 235
118 2 2 230 279 513 7.70E-51 208.8 6596 cellular
organisms;Eukaryota;Opisthokonta;Metazoa;Eumetazoa;Bilateria;Protostomia;
Spiralia;Lophotrochozoa;Mollusca;Bivalvia;Autobranchia;Heteroconchia;Euhe
terodonta;Imparidentia;Venerida;Veneroidea;Veneridae;Mercenaria;Mercenari
a mercenaria
UdeM00000040635 37621 156.8 UdeM00000040635 XP_014677368.1 44.9 207
100 6 1 196 1 204 3.00E-35 156.8 37621 cellular
organisms;Eukaryota;Opisthokonta;Metazoa;Eumetazoa;Bilateria;Protostomia;
Ecdysozoa;Scalidophora;Priapulida;Priapulimorpha;Priapulimorphida;Priapul
idae;Priapulus;Priapulus caudatus
UdeM00000040636 7741 98.6 UdeM00000040636 XP_019633208.1 31.3 182
101 8 195 374 2715 2874 1.90E-17 98.6 7741 cellular
organisms;Eukaryota;Opisthokonta;Metazoa;Eumetazoa;Bilateria;Deuterostomi
a;Chordata;Cephalochordata;Leptocardii;Amphioxiformes;Branchiostomidae;Br
anchiostoma;Branchiostoma belcheri
UdeM00000040637 6596 119.8 UdeM00000040637 XP_045202346.1 38.8 152
89 2 6 156 16 164 3.20E-24 119.8 6596 cellular
organisms;Eukaryota;Opisthokonta;Metazoa;Eumetazoa;Bilateria;Protostomia;
Spiralia;Lophotrochozoa;Mollusca;Bivalvia;Autobranchia;Heteroconchia;Euhe
terodonta;Imparidentia;Venerida;Veneroidea;Veneridae;Mercenaria;Mercenari
a mercenaria
UdeM00000040638 6596 82 UdeM00000040638 XP_045201969.1 39.5 157
75 5 1 146 19 166 8.00E-13 82 6596 cellular
organisms;Eukaryota;Opisthokonta;Metazoa;Eumetazoa;Bilateria;Protostomia;
Spiralia;Lophotrochozoa;Mollusca;Bivalvia;Autobranchia;Heteroconchia;Euhe
terodonta;Imparidentia;Venerida;Veneroidea;Veneridae;Mercenaria;Mercenari
a mercenaria
UdeM00000040639 6596 1434.5 UdeM00000040639 XP_045160921.1 37.2
2089 1136 19 27 2099 114 2042 0.00E+00 1434.5 6596
cellular
organisms;Eukaryota;Opisthokonta;Metazoa;Eumetazoa;Bilateria;Protostomia;
Spiralia;Lophotrochozoa;Mollusca;Bivalvia;Autobranchia;Heteroconchia;Euhe
terodonta;Imparidentia;Venerida;Veneroidea;Veneridae;Mercenaria;Mercenari
a mercenaria
UdeM00000040640 6596 718 UdeM00000040640 XP_045160921.1 31.8 1333
787 11 1 1225 2849 4167 2.10E-203 718 6596 cellular
organisms;Eukaryota;Opisthokonta;Metazoa;Eumetazoa;Bilateria;Protostomia;
Spiralia;Lophotrochozoa;Mollusca;Bivalvia;Autobranchia;Heteroconchia;Euhe
terodonta;Imparidentia;Venerida;Veneroidea;Veneridae;Mercenaria;Mercenari
a mercenaria
UdeM00000040642 6596 401.4 UdeM00000040642 XP_045158043.1 49.4 419
208 3 4 421 366 781 1.50E-108 401.4 6596 cellular
organisms;Eukaryota;Opisthokonta;Metazoa;Eumetazoa;Bilateria;Protostomia;
Spiralia;Lophotrochozoa;Mollusca;Bivalvia;Autobranchia;Heteroconchia;Euhe
terodonta;Imparidentia;Venerida;Veneroidea;Veneridae;Mercenaria;Mercenari
a mercenaria
UdeM00000040641 6596 533.9 UdeM00000040641 XP_045158043.1 46.1 618
322 7 112 724 170 781 3.20E-148 533.9 6596 cellular
organisms;Eukaryota;Opisthokonta;Metazoa;Eumetazoa;Bilateria;Protostomia;
Spiralia;Lophotrochozoa;Mollusca;Bivalvia;Autobranchia;Heteroconchia;Euhe
terodonta;Imparidentia;Venerida;Veneroidea;Veneridae;Mercenaria;Mercenari
a mercenaria

UdeM00000040643 29159 69.3 UdeM00000040643 XP_019929678.1 36.2 105
58 2 48 152 200 295 9.40E-09 69.3 29159 cellular
organisms;Eukaryota;Opisthokonta;Metazoa;Eumetazoa;Bilateria;Protostomia;
Spiralia;Lophotrochozoa;Mollusca;Bivalvia;Autobranchia;Pteriomorphia;Ostr
eida;Ostreoidea;Ostreidae;Crassostrea;Crassostrea gigas
UdeM00000040644 29159 105.5 UdeM00000040644 XP_034312519.1 71.4 77
21 1 172 248 236 311 1.20E-19 105.5 29159 cellular
organisms;Eukaryota;Opisthokonta;Metazoa;Eumetazoa;Bilateria;Protostomia;
Spiralia;Lophotrochozoa;Mollusca;Bivalvia;Autobranchia;Pteriomorphia;Ostr
eida;Ostreoidea;Ostreidae;Crassostrea;Crassostrea gigas
UdeM00000040646 6596 93.6 UdeM00000040646 XP_045208792.1 45 131
64 4 1 125 1 129 2.30E-16 93.6 6596 cellular
organisms;Eukaryota;Opisthokonta;Metazoa;Eumetazoa;Bilateria;Protostomia;
Spiralia;Lophotrochozoa;Mollusca;Bivalvia;Autobranchia;Heteroconchia;Euhe
terodonta;Imparidentia;Venerida;Veneroidea;Veneridae;Mercenaria;Mercenari
a mercenaria
UdeM00000040647 6596 237.7 UdeM00000040647 XP_045160921.1 27.7 563
401 4 1 562 3003 3560 3.70E-59 237.7 6596 cellular
organisms;Eukaryota;Opisthokonta;Metazoa;Eumetazoa;Bilateria;Protostomia;
Spiralia;Lophotrochozoa;Mollusca;Bivalvia;Autobranchia;Heteroconchia;Euhe
terodonta;Imparidentia;Venerida;Veneroidea;Veneridae;Mercenaria;Mercenari
a mercenaria
UdeM00000040648 6596 515.4 UdeM00000040648 XP_045160921.1 36.3 780
369 12 4 675 3589 4348 1.10E-142 515.4 6596 cellular
organisms;Eukaryota;Opisthokonta;Metazoa;Eumetazoa;Bilateria;Protostomia;
Spiralia;Lophotrochozoa;Mollusca;Bivalvia;Autobranchia;Heteroconchia;Euhe
terodonta;Imparidentia;Venerida;Veneroidea;Veneridae;Mercenaria;Mercenari
a mercenaria
UdeM00000040649 6596 517.7 UdeM00000040649 XP_045158018.1 37.5 829
466 16 131 942 465 1258 3.10E-143 517.7 6596 cellular
organisms;Eukaryota;Opisthokonta;Metazoa;Eumetazoa;Bilateria;Protostomia;
Spiralia;Lophotrochozoa;Mollusca;Bivalvia;Autobranchia;Heteroconchia;Euhe
terodonta;Imparidentia;Venerida;Veneroidea;Veneridae;Mercenaria;Mercenari
a mercenaria
UdeM00000040650 7668 128.3 UdeM00000040650 XP_030851866.1 27.7 328
177 13 184 485 1959 2252 3.50E-26 128.3 7668 cellular
organisms;Eukaryota;Opisthokonta;Metazoa;Eumetazoa;Bilateria;Deuterostomi
a;Echinodermata;Eleutherozoa;Echinozoa;Echinoidea;Euechinoidea;Echinacea;
Camarodonta;Echinidea;Strongylocentrotidae;Strongylocentrotus;Strongyloce
ntrotus purpuratus
UdeM00000040651 7668 128.3 UdeM00000040651 XP_030851866.1 27.7 328
177 13 184 485 1959 2252 3.40E-26 128.3 7668 cellular
organisms;Eukaryota;Opisthokonta;Metazoa;Eumetazoa;Bilateria;Deuterostomi
a;Echinodermata;Eleutherozoa;Echinozoa;Echinoidea;Euechinoidea;Echinacea;
Camarodonta;Echinidea;Strongylocentrotidae;Strongylocentrotus;Strongyloce
ntrotus purpuratus
UdeM00000040652 1735272 124.8 UdeM00000040652 XP_041357850.1 50
118 58 1 1 118 158 274 8.40E-26 124.8 1735272
cellular
organisms;Eukaryota;Opisthokonta;Metazoa;Eumetazoa;Bilateria;Protostomia;
Spiralia;Lophotrochozoa;Mollusca;Gastropoda;Neomphalina;Neomphaloidea;Pel
tospiridae;Gigantopelta;Gigantopelta aegis
UdeM00000040653 6596 914.1 UdeM00000040653 XP_045160921.1 40.2 1201
705 10 1 1193 348 1543 1.90E-262 914.1 6596 cellular

organisms;Eukaryota;Opisthokonta;Metazoa;Eumetazoa;Bilateria;Protostomia;Spiralia;Lophotrochozoa;Mollusca;Bivalvia;Autobranchia;Heteroconchia;Euheterodonta;Imparidentia;Venerida;Veneroidea;Veneridae;Mercenaria;Mercenaria mercenaria
UdeM00000040654 6596 920.2 UdeM00000040654 XP_045160921.1 32 1844 1120 36 1 1807 1563 3309 4.00E-264 920.2 6596 cellular organisms;Eukaryota;Opisthokonta;Metazoa;Eumetazoa;Bilateria;Protostomia;Spiralia;Lophotrochozoa;Mollusca;Bivalvia;Autobranchia;Heteroconchia;Euheterodonta;Imparidentia;Venerida;Veneroidea;Veneridae;Mercenaria;Mercenaria mercenaria
UdeM00000040655 6596 608.6 UdeM00000040655 XP_045160921.1 34.4 978 516 11 12 881 3359 4318 1.30E-170 608.6 6596 cellular organisms;Eukaryota;Opisthokonta;Metazoa;Eumetazoa;Bilateria;Protostomia;Spiralia;Lophotrochozoa;Mollusca;Bivalvia;Autobranchia;Heteroconchia;Euheterodonta;Imparidentia;Venerida;Veneroidea;Veneridae;Mercenaria;Mercenaria mercenaria
UdeM00000040656 6596 613.2 UdeM00000040656 XP_045158018.1 32 1264 785 26 24 1244 27 1258 7.20E-172 613.2 6596 cellular organisms;Eukaryota;Opisthokonta;Metazoa;Eumetazoa;Bilateria;Protostomia;Spiralia;Lophotrochozoa;Mollusca;Bivalvia;Autobranchia;Heteroconchia;Euheterodonta;Imparidentia;Venerida;Veneroidea;Veneridae;Mercenaria;Mercenaria mercenaria
UdeM00000044452 151771 395.6 UdeM00000044452 XP_028419301.1 57.3 337 142 2 42 378 34 368 7.20E-107 395.6 151771 cellular organisms;Eukaryota;Opisthokonta;Metazoa;Eumetazoa;Cnidaria;Anthozoa;Octocorallia;Alcyonacea;Alcyoniina;Nephtheidae;Dendronephthya;Dendronephthya gigantea
UdeM00000035651 400727 500 UdeM00000035651 XP_025093876.1 73.9 318 83 0 1 318 6 323 2.30E-138 500 400727 cellular organisms;Eukaryota;Opisthokonta;Metazoa;Eumetazoa;Bilateria;Protostomia;Spiralia;Lophotrochozoa;Mollusca;Gastropoda;Caenogastropoda;Architaenioglossa;Ampullarioidea;Ampullariidae;Pomacea;Pomacea canaliculata
UdeM00000035652 6579 493.4 UdeM00000035652 XP_033756773.1 74.3 315 81 0 6 320 5 319 2.10E-136 493.4 6579 cellular organisms;Eukaryota;Opisthokonta;Metazoa;Eumetazoa;Bilateria;Protostomia;Spiralia;Lophotrochozoa;Mollusca;Bivalvia;Autobranchia;Pteriomorphia;Pectinida;Pectinoidea;Pectinidae;Pecten;Pecten maximus
UdeM00000035654 7574 101.3 UdeM00000035654 XP_013384015.1 49.2 120 59 2 6 125 186 303 1.50E-18 101.3 7574 cellular organisms;Eukaryota;Opisthokonta;Metazoa;Eumetazoa;Bilateria;Protostomia;Spiralia;Lophotrochozoa;Brachiopoda;Linguliformea;Lingulata;Lingulida;Linguloidea;Lingulidae;Lingula;Lingula anatina
UdeM00000035655 6596 77 UdeM00000035655 XP_045173729.1 44 75 42 0 52 126 101 175 2.10E-11 77 6596 cellular organisms;Eukaryota;Opisthokonta;Metazoa;Eumetazoa;Bilateria;Protostomia;Spiralia;Lophotrochozoa;Mollusca;Bivalvia;Autobranchia;Heteroconchia;Euheterodonta;Imparidentia;Venerida;Veneroidea;Veneridae;Mercenaria;Mercenaria mercenaria
UdeM00000035656 6596 80.9 UdeM00000035656 XP_045173729.1 46.8 77 40 1 52 128 101 176 1.40E-12 80.9 6596 cellular organisms;Eukaryota;Opisthokonta;Metazoa;Eumetazoa;Bilateria;Protostomia;Spiralia;Lophotrochozoa;Mollusca;Bivalvia;Autobranchia;Heteroconchia;Euhe

terodonta;Imparidentia;Venerida;Veneroidea;Veneridae;Mercenaria;Mercenaria mercenaria
UdeM00000035657 225164 80.1 UdeM00000035657 XP_009051888.1 30.9
152 92 5 28 176 28 169 3.30E-12 80.1 225164
cellular
organisms;Eukaryota;Opisthokonta;Metazoa;Eumetazoa;Bilateria;Protostomia;Spiralia;Lophotrochozoa;Mollusca;Gastropoda;Patellogastropoda;Lottioidea;Lottiidae;Lottia;Lottia gigantea
UdeM00000035658 6596 78.6 UdeM00000035658 XP_045173729.1 44 84
47 0 43 126 92 175 7.10E-12 78.6 6596 cellular
organisms;Eukaryota;Opisthokonta;Metazoa;Eumetazoa;Bilateria;Protostomia;Spiralia;Lophotrochozoa;Mollusca;Bivalvia;Autobranchia;Heteroconchia;Euheterodonta;Imparidentia;Venerida;Veneroidea;Veneridae;Mercenaria;Mercenaria mercenaria
UdeM00000035659 1735272 140.6 UdeM00000035659 XP_041369037.1 64.7
102 35 1 48 149 40 140 1.90E-30 140.6 1735272
cellular
organisms;Eukaryota;Opisthokonta;Metazoa;Eumetazoa;Bilateria;Protostomia;Spiralia;Lophotrochozoa;Mollusca;Gastropoda;Neomphalina;Neomphaloidea;Peltospiridae;Gigantopelta;Gigantopelta aegis
UdeM00000035660 1735272 833.9 UdeM00000035660 XP_041369037.1 68.8
600 168 4 1 591 162 751 1.30E-238 833.9 1735272
cellular
organisms;Eukaryota;Opisthokonta;Metazoa;Eumetazoa;Bilateria;Protostomia;Spiralia;Lophotrochozoa;Mollusca;Gastropoda;Neomphalina;Neomphaloidea;Peltospiridae;Gigantopelta;Gigantopelta aegis
UdeM00000035663 6573 232.3 UdeM00000035663 XP_021378568.1 62.4 165
58 2 634 794 490 654 2.50E-57 232.3 6573 cellular
organisms;Eukaryota;Opisthokonta;Metazoa;Eumetazoa;Bilateria;Protostomia;Spiralia;Lophotrochozoa;Mollusca;Bivalvia;Autobranchia;Pteriomorphia;Pectinida;Pectinoidea;Pectinidae;Mizuhopecten;Mizuhopecten yessoensis
UdeM00000035664 6596 464.5 UdeM00000035664 XP_045191127.1 45.8 522
256 5 1 497 5 524 1.70E-127 464.5 6596 cellular
organisms;Eukaryota;Opisthokonta;Metazoa;Eumetazoa;Bilateria;Protostomia;Spiralia;Lophotrochozoa;Mollusca;Bivalvia;Autobranchia;Heteroconchia;Euheterodonta;Imparidentia;Venerida;Veneroidea;Veneridae;Mercenaria;Mercenaria mercenaria
UdeM00000035665 6596 183.3 UdeM00000035665 XP_045190001.1 56.6 143
60 1 25 167 4 144 2.50E-43 183.3 6596 cellular
organisms;Eukaryota;Opisthokonta;Metazoa;Eumetazoa;Bilateria;Protostomia;Spiralia;Lophotrochozoa;Mollusca;Bivalvia;Autobranchia;Heteroconchia;Euheterodonta;Imparidentia;Venerida;Veneroidea;Veneridae;Mercenaria;Mercenaria mercenaria
UdeM00000035666 6579 151.4 UdeM00000035666 XP_033757506.1 43.4 143
80 1 14 155 14 156 1.00E-33 151.4 6579 cellular
organisms;Eukaryota;Opisthokonta;Metazoa;Eumetazoa;Bilateria;Protostomia;Spiralia;Lophotrochozoa;Mollusca;Bivalvia;Autobranchia;Pteriomorphia;Pectinida;Pectinoidea;Pectinidae;Pecten;Pecten maximus
UdeM00000035667 6596 364.8 UdeM00000035667 XP_045191095.1 60.5 258
101 1 24 280 50 307 1.00E-97 364.8 6596 cellular
organisms;Eukaryota;Opisthokonta;Metazoa;Eumetazoa;Bilateria;Protostomia;Spiralia;Lophotrochozoa;Mollusca;Bivalvia;Autobranchia;Heteroconchia;Euheterodonta;Imparidentia;Venerida;Veneroidea;Veneridae;Mercenaria;Mercenaria mercenaria

UdeM00000035668 6596 901.7 UdeM00000035668 XP_045189275.1 60.6 690
270 2 3 691 2 690 5.80E-259 901.7 6596 cellular
organisms;Eukaryota;Opisthokonta;Metazoa;Eumetazoa;Bilateria;Protostomia;
Spiralia;Lophotrochozoa;Mollusca;Bivalvia;Autobranchia;Heteroconchia;Euhe
terodonta;Imparidentia;Venerida;Veneroidea;Veneridae;Mercenaria;Mercenari
a mercenaria
UdeM00000035669 225164 325.1 UdeM00000035669 XP_009066648.1 32.2
652 383 12 4 648 28 627 2.00E-85 325.1 225164
cellular
organisms;Eukaryota;Opisthokonta;Metazoa;Eumetazoa;Bilateria;Protostomia;
Spiralia;Lophotrochozoa;Mollusca;Gastropoda;Patellogastropoda;Lottioidea;
Lottiidae;Lottia;Lottia gigantea
UdeM00000035670 6596 390.6 UdeM00000035670 XP_045188197.1 38.1 616
188 8 1 476 3 565 2.90E-105 390.6 6596 cellular
organisms;Eukaryota;Opisthokonta;Metazoa;Eumetazoa;Bilateria;Protostomia;
Spiralia;Lophotrochozoa;Mollusca;Bivalvia;Autobranchia;Heteroconchia;Euhe
terodonta;Imparidentia;Venerida;Veneroidea;Veneridae;Mercenaria;Mercenari
a mercenaria
UdeM00000035671 6596 360.1 UdeM00000035671 XP_045188197.1 36.3 615
167 7 1 442 3 565 3.90E-96 360.1 6596 cellular
organisms;Eukaryota;Opisthokonta;Metazoa;Eumetazoa;Bilateria;Protostomia;
Spiralia;Lophotrochozoa;Mollusca;Bivalvia;Autobranchia;Heteroconchia;Euhe
terodonta;Imparidentia;Venerida;Veneroidea;Veneridae;Mercenaria;Mercenari
a mercenaria
UdeM00000035673 6573 680.6 UdeM00000035673 XP_021369118.1 75.4 435
99 3 15 444 50 481 1.30E-192 680.6 6573 cellular
organisms;Eukaryota;Opisthokonta;Metazoa;Eumetazoa;Bilateria;Protostomia;
Spiralia;Lophotrochozoa;Mollusca;Bivalvia;Autobranchia;Pteriomorphia;Pect
inida;Pectinoidea;Pectinidae;Mizuhopecten;Mizuhopecten yessoensis
UdeM00000035674 6596 736.5 UdeM00000035674 XP_045188969.1 94.7 376
20 0 1 376 1 376 1.70E-209 736.5 6596 cellular
organisms;Eukaryota;Opisthokonta;Metazoa;Eumetazoa;Bilateria;Protostomia;
Spiralia;Lophotrochozoa;Mollusca;Bivalvia;Autobranchia;Heteroconchia;Euhe
terodonta;Imparidentia;Venerida;Veneroidea;Veneridae;Mercenaria;Mercenari
a mercenaria
UdeM00000035675 6596 1125.9 UdeM00000035675 XP_045189303.1 63.3
828 296 4 1 822 1 826 0.00E+00 1125.9 6596
cellular
organisms;Eukaryota;Opisthokonta;Metazoa;Eumetazoa;Bilateria;Protostomia;
Spiralia;Lophotrochozoa;Mollusca;Bivalvia;Autobranchia;Heteroconchia;Euhe
terodonta;Imparidentia;Venerida;Veneroidea;Veneridae;Mercenaria;Mercenari
a mercenaria
UdeM00000035676 6596 391.7 UdeM00000035676 XP_045189115.1 69.7 261
78 1 3 263 26 285 1.70E-105 391.7 6596 cellular
organisms;Eukaryota;Opisthokonta;Metazoa;Eumetazoa;Bilateria;Protostomia;
Spiralia;Lophotrochozoa;Mollusca;Bivalvia;Autobranchia;Heteroconchia;Euhe
terodonta;Imparidentia;Venerida;Veneroidea;Veneridae;Mercenaria;Mercenari
a mercenaria
UdeM00000035677 37621 141.7 UdeM00000035677 XP_014677462.1 52.1 144
58 2 2 141 30 166 8.90E-31 141.7 37621 cellular
organisms;Eukaryota;Opisthokonta;Metazoa;Eumetazoa;Bilateria;Protostomia;
Ecdysozoa;Scalidophora;Priapulida;Priapulimorpha;Priapulimorphida;Priapul
idae;Priapulus;Priapulus caudatus

UdeM00000035678	6573	632.5	UdeM00000035678	XP_021375729.1	49.8	626
289	6	1	601	1	626	5.70E-178	632.5	6573	cellular organisms;Eukaryota;Opisthokonta;Metazoa;Eumetazoa;Bilateria;Protostomia;Spiralia;Lophotrochozoa;Mollusca;Bivalvia;Autobranchia;Pteriomorphia;Pectinida;Pectinoidea;Pectinidae;Mizuhopecten;Mizuhopecten yessoensis
UdeM00000035679	6596	503.8	UdeM00000035679	XP_045189114.1	59.5	402
155	4	3	399	288	686	2.00E-139	503.8	6596	cellular organisms;Eukaryota;Opisthokonta;Metazoa;Eumetazoa;Bilateria;Protostomia;Spiralia;Lophotrochozoa;Mollusca;Bivalvia;Autobranchia;Heteroconchia;Euheterodonta;Imparidentia;Venerida;Veneroidea;Veneridae;Mercenaria;Mercenaria mercenaria
UdeM00000035680	6596	457.2	UdeM00000035680	XP_045189114.1	57.4	376
152	4	1	371	314	686	2.00E-125	457.2	6596	cellular organisms;Eukaryota;Opisthokonta;Metazoa;Eumetazoa;Bilateria;Protostomia;Spiralia;Lophotrochozoa;Mollusca;Bivalvia;Autobranchia;Heteroconchia;Euheterodonta;Imparidentia;Venerida;Veneroidea;Veneridae;Mercenaria;Mercenaria mercenaria
UdeM00000035681	6596	316.2	UdeM00000035681	XP_045190567.1	40.5	607
304	13	11	566	19	619	8.30E-83	316.2	6596	cellular organisms;Eukaryota;Opisthokonta;Metazoa;Eumetazoa;Bilateria;Protostomia;Spiralia;Lophotrochozoa;Mollusca;Bivalvia;Autobranchia;Heteroconchia;Euheterodonta;Imparidentia;Venerida;Veneroidea;Veneridae;Mercenaria;Mercenaria mercenaria
UdeM00000035683	6579	93.2	UdeM00000035683	XP_033725359.1	55.4	83
37	0	1	83	1	83	1.70E-16	93.2	6579	cellular organisms;Eukaryota;Opisthokonta;Metazoa;Eumetazoa;Bilateria;Protostomia;Spiralia;Lophotrochozoa;Mollusca;Bivalvia;Autobranchia;Pteriomorphia;Pectinida;Pectinoidea;Pectinidae;Pecten;Pecten maximus
UdeM00000035684	6596	243.4	UdeM00000035684	XP_045188426.1	31.8	538
275	12	2	531	29	482	6.60E-61	243.4	6596	cellular organisms;Eukaryota;Opisthokonta;Metazoa;Eumetazoa;Bilateria;Protostomia;Spiralia;Lophotrochozoa;Mollusca;Bivalvia;Autobranchia;Heteroconchia;Euheterodonta;Imparidentia;Venerida;Veneroidea;Veneridae;Mercenaria;Mercenaria mercenaria
UdeM00000035685	6596	283.1	UdeM00000035685	XP_045188426.1	37.4	478
293	3	49	523	10	484	7.30E-73	283.1	6596	cellular organisms;Eukaryota;Opisthokonta;Metazoa;Eumetazoa;Bilateria;Protostomia;Spiralia;Lophotrochozoa;Mollusca;Bivalvia;Autobranchia;Heteroconchia;Euheterodonta;Imparidentia;Venerida;Veneroidea;Veneridae;Mercenaria;Mercenaria mercenaria
UdeM00000035686	6596	1513	UdeM00000035686	XP_045190629.1	51.2	2403
888	38	360	2648	644	2876	0.00E+00	1513	6596	cellular organisms;Eukaryota;Opisthokonta;Metazoa;Eumetazoa;Bilateria;Protostomia;Spiralia;Lophotrochozoa;Mollusca;Bivalvia;Autobranchia;Heteroconchia;Euheterodonta;Imparidentia;Venerida;Veneroidea;Veneridae;Mercenaria;Mercenaria mercenaria
UdeM00000035687	6596	368.2	UdeM00000035687	XP_045190629.1	76.8	228
50	1	1	225	1	228	7.70E-99	368.2	6596	cellular organisms;Eukaryota;Opisthokonta;Metazoa;Eumetazoa;Bilateria;Protostomia;Spiralia;Lophotrochozoa;Mollusca;Bivalvia;Autobranchia;Heteroconchia;Euheterodonta;Imparidentia;Venerida;Veneroidea;Veneridae;Mercenaria;Mercenaria mercenaria
UdeM00000035688	6573	320.9	UdeM00000035688	XP_021363992.1	61.4	290
94	5	1	285	1	277	1.70E-84	320.9	6573	cellular

organisms;Eukaryota;Opisthokonta;Metazoa;Eumetazoa;Bilateria;Protostomia;Spiralia;Lophotrochozoa;Mollusca;Bivalvia;Autobranchia;Pteriomorphia;Pectinida;Pectinoidea;Pectinidae;Mizuhopecten;Mizuhopecten yessoensis
UdeM00000035689 6573 393.3 UdeM00000035689 XP_021369121.1 41 547 295 7 3 541 609 1135 5.10E-106 393.3 6573 cellular organisms;Eukaryota;Opisthokonta;Metazoa;Eumetazoa;Bilateria;Protostomia;Spiralia;Lophotrochozoa;Mollusca;Bivalvia;Autobranchia;Pteriomorphia;Pectinida;Pectinoidea;Pectinidae;Mizuhopecten;Mizuhopecten yessoensis
UdeM00000035690 6565 164.1 UdeM00000035690 XP_022288784.1 45.5 220 110 7 86 299 465 680 2.90E-37 164.1 6565 cellular organisms;Eukaryota;Opisthokonta;Metazoa;Eumetazoa;Bilateria;Protostomia;Spiralia;Lophotrochozoa;Mollusca;Bivalvia;Autobranchia;Pteriomorphia;Ostreida;Ostreoidea;Ostreidae;Crassostrea;Crassostrea virginica
UdeM00000035691 6565 164.5 UdeM00000035691 XP_022288784.1 45.9 220 107 8 86 297 465 680 2.20E-37 164.5 6565 cellular organisms;Eukaryota;Opisthokonta;Metazoa;Eumetazoa;Bilateria;Protostomia;Spiralia;Lophotrochozoa;Mollusca;Bivalvia;Autobranchia;Pteriomorphia;Ostreida;Ostreoidea;Ostreidae;Crassostrea;Crassostrea virginica
UdeM00000035693 6573 557.4 UdeM00000035693 XP_021369121.1 34 1144 671 26 12 1095 8 1127 4.10E-155 557.4 6573 cellular organisms;Eukaryota;Opisthokonta;Metazoa;Eumetazoa;Bilateria;Protostomia;Spiralia;Lophotrochozoa;Mollusca;Bivalvia;Autobranchia;Pteriomorphia;Pectinida;Pectinoidea;Pectinidae;Mizuhopecten;Mizuhopecten yessoensis
UdeM00000035695 6573 572.4 UdeM00000035695 XP_021369121.1 34.1 1186 676 24 3 1137 5 1135 1.30E-159 572.4 6573 cellular organisms;Eukaryota;Opisthokonta;Metazoa;Eumetazoa;Bilateria;Protostomia;Spiralia;Lophotrochozoa;Mollusca;Bivalvia;Autobranchia;Pteriomorphia;Pectinida;Pectinoidea;Pectinidae;Mizuhopecten;Mizuhopecten yessoensis
UdeM00000035696 6573 677.6 UdeM00000035696 XP_021369121.1 39.3 1151 605 28 14 1089 6 1137 2.70E-191 677.6 6573 cellular organisms;Eukaryota;Opisthokonta;Metazoa;Eumetazoa;Bilateria;Protostomia;Spiralia;Lophotrochozoa;Mollusca;Bivalvia;Autobranchia;Pteriomorphia;Pectinida;Pectinoidea;Pectinidae;Mizuhopecten;Mizuhopecten yessoensis
UdeM00000035697 6573 670.2 UdeM00000035697 XP_021369121.1 39 1160 605 29 14 1098 6 1137 4.40E-189 670.2 6573 cellular organisms;Eukaryota;Opisthokonta;Metazoa;Eumetazoa;Bilateria;Protostomia;Spiralia;Lophotrochozoa;Mollusca;Bivalvia;Autobranchia;Pteriomorphia;Pectinida;Pectinoidea;Pectinidae;Mizuhopecten;Mizuhopecten yessoensis
UdeM00000035700 400727 806.6 UdeM00000035700 XP_025093999.1 51.3 819 246 6 22 839 73 739 3.00E-230 806.6 400727 cellular organisms;Eukaryota;Opisthokonta;Metazoa;Eumetazoa;Bilateria;Protostomia;Spiralia;Lophotrochozoa;Mollusca;Gastropoda;Caenogastropoda;Architaenioglossa;Ampullarioidea;Ampullariidae;Pomacea;Pomacea canaliculata
UdeM00000035701 6565 301.6 UdeM00000035701 XP_022338358.1 38 492 265 11 37 526 12 465 2.00E-78 301.6 6565 cellular organisms;Eukaryota;Opisthokonta;Metazoa;Eumetazoa;Bilateria;Protostomia;Spiralia;Lophotrochozoa;Mollusca;Bivalvia;Autobranchia;Pteriomorphia;Ostreida;Ostreoidea;Ostreidae;Crassostrea;Crassostrea virginica
UdeM00000035702 6579 246.1 UdeM00000035702 XP_033739209.1 49.2 258 115 2 9 261 2 248 4.90E-62 246.1 6579 cellular organisms;Eukaryota;Opisthokonta;Metazoa;Eumetazoa;Bilateria;Protostomia;Spiralia;Lophotrochozoa;Mollusca;Bivalvia;Autobranchia;Pteriomorphia;Pectinida;Pectinoidea;Pectinidae;Pecten;Pecten maximus

UdeM00000035703 6565 265 UdeM00000035703 XP_022305704.1 32.8 485 232 6 103 523 3 457 2.00E-67 265 6565 cellular organisms;Eukaryota;Opisthokonta;Metazoa;Eumetazoa;Bilateria;Protostomia;Spiralia;Lophotrochozoa;Mollusca;Bivalvia;Autobranchia;Pteriomorphia;Ostreida;Ostreoidea;Ostreidae;Crassostrea;Crassostrea virginica
UdeM00000035704 7574 148.3 UdeM00000035704 XP_013405563.1 36.6 257 136 6 1 254 146 378 1.40E-32 148.3 7574 cellular organisms;Eukaryota;Opisthokonta;Metazoa;Eumetazoa;Bilateria;Protostomia;Spiralia;Lophotrochozoa;Brachiopoda;Linguliformea;Lingulata;Lingulida;Linguloidea;Lingulidae;Lingula;Lingula anatina
UdeM00000035705 6579 246.1 UdeM00000035705 XP_033739209.1 49.2 258 115 2 9 261 2 248 4.90E-62 246.1 6579 cellular organisms;Eukaryota;Opisthokonta;Metazoa;Eumetazoa;Bilateria;Protostomia;Spiralia;Lophotrochozoa;Mollusca;Bivalvia;Autobranchia;Pteriomorphia;Pectinida;Pectinoidea;Pectinidae;Pecten;Pecten maximus
UdeM00000035706 6565 265 UdeM00000035706 XP_022305704.1 32.8 485 232 6 103 523 3 457 2.00E-67 265 6565 cellular organisms;Eukaryota;Opisthokonta;Metazoa;Eumetazoa;Bilateria;Protostomia;Spiralia;Lophotrochozoa;Mollusca;Bivalvia;Autobranchia;Pteriomorphia;Ostreida;Ostreoidea;Ostreidae;Crassostrea;Crassostrea virginica
UdeM00000035707 7574 148.3 UdeM00000035707 XP_013405563.1 36.6 257 136 6 1 254 146 378 1.40E-32 148.3 7574 cellular organisms;Eukaryota;Opisthokonta;Metazoa;Eumetazoa;Bilateria;Protostomia;Spiralia;Lophotrochozoa;Brachiopoda;Linguliformea;Lingulata;Lingulida;Linguloidea;Lingulidae;Lingula;Lingula anatina
UdeM00000035708 6579 246.1 UdeM00000035708 XP_033739209.1 49.2 258 115 2 9 261 2 248 4.90E-62 246.1 6579 cellular organisms;Eukaryota;Opisthokonta;Metazoa;Eumetazoa;Bilateria;Protostomia;Spiralia;Lophotrochozoa;Mollusca;Bivalvia;Autobranchia;Pteriomorphia;Pectinida;Pectinoidea;Pectinidae;Pecten;Pecten maximus
UdeM00000035709 6565 265 UdeM00000035709 XP_022305704.1 32.8 485 232 6 103 523 3 457 2.00E-67 265 6565 cellular organisms;Eukaryota;Opisthokonta;Metazoa;Eumetazoa;Bilateria;Protostomia;Spiralia;Lophotrochozoa;Mollusca;Bivalvia;Autobranchia;Pteriomorphia;Ostreida;Ostreoidea;Ostreidae;Crassostrea;Crassostrea virginica
UdeM00000035710 6596 122.5 UdeM00000035710 XP_045215394.1 49.6 113 55 1 96 206 35 147 7.20E-25 122.5 6596 cellular organisms;Eukaryota;Opisthokonta;Metazoa;Eumetazoa;Bilateria;Protostomia;Spiralia;Lophotrochozoa;Mollusca;Bivalvia;Autobranchia;Heteroconchia;Euheterodonta;Imparidentia;Venerida;Veneroidea;Veneridae;Mercenaria;Mercenaria mercenaria
UdeM00000035711 1735272 435.3 UdeM00000035711 XP_041369772.1 59.5 380 145 2 21 392 13 391 9.30E-119 435.3 1735272 cellular organisms;Eukaryota;Opisthokonta;Metazoa;Eumetazoa;Bilateria;Protostomia;Spiralia;Lophotrochozoa;Mollusca;Gastropoda;Neomphalina;Neomphaloidea;Peltospiridae;Gigantopelta;Gigantopelta aegis
UdeM00000035712 6565 594.7 UdeM00000035712 XP_022333349.1 64.8 466 120 2 6 427 43 508 9.10E-167 594.7 6565 cellular organisms;Eukaryota;Opisthokonta;Metazoa;Eumetazoa;Bilateria;Protostomia;Spiralia;Lophotrochozoa;Mollusca;Bivalvia;Autobranchia;Pteriomorphia;Ostreida;Ostreoidea;Ostreidae;Crassostrea;Crassostrea virginica
UdeM00000035713 7757 71.2 UdeM00000035713 XP_032835784.1 60 60 24 0 3 62 4 63 7.80E-10 71.2 7757 cellular

organisms;Eukaryota;Opisthokonta;Metazoa;Eumetazoa;Bilateria;Deuterostomia;Chordata;Craniata;Vertebrata;Cyclostomata;Hyperoartia;Petromyzontiformes;Petromyzontidae;Petromyzon;Petromyzon marinus
UdeM00000035714 6579 250 UdeM00000035714 XP_033733679.1 63.4 186 68 0 3 188 2 187 2.50E-63 250 6579 cellular organisms;Eukaryota;Opisthokonta;Metazoa;Eumetazoa;Bilateria;Protostomia;Spiralia;Lophotrochozoa;Mollusca;Bivalvia;Autobranchia;Pteriomorphia;Pectinida;Pectinoidea;Pectinidae;Pecten;Pecten maximus
UdeM00000035715 1529436 151 UdeM00000035715 XP_033124485.1 83.3 78 11 1 1 78 1 76 6.50E-34 151 1529436 cellular organisms;Eukaryota;Opisthokonta;Metazoa;Eumetazoa;Bilateria;Deuterostomia;Echinodermata;Pelmatozoa;Crinoidea;Articulata;Comatulida;Comatulidae;Comatulinae;Anneissia;Anneissia japonica
UdeM00000035716 6573 130.6 UdeM00000035716 XP_021355033.1 40.3 144 85 1 2 144 4 147 2.70E-27 130.6 6573 cellular organisms;Eukaryota;Opisthokonta;Metazoa;Eumetazoa;Bilateria;Protostomia;Spiralia;Lophotrochozoa;Mollusca;Bivalvia;Autobranchia;Pteriomorphia;Pectinida;Pectinoidea;Pectinidae;Mizuhopecten;Mizuhopecten yessoensis
UdeM00000035717 6596 164.5 UdeM00000035717 XP_045207342.1 50.6 170 81 2 2 168 3 172 1.20E-37 164.5 6596 cellular organisms;Eukaryota;Opisthokonta;Metazoa;Eumetazoa;Bilateria;Protostomia;Spiralia;Lophotrochozoa;Mollusca;Bivalvia;Autobranchia;Heteroconchia;Euheterodonta;Imparidentia;Venerida;Veneroidea;Veneridae;Mercenaria;Mercenaria mercenaria
UdeM00000035718 6565 133.7 UdeM00000035718 XP_022321780.1 50.8 122 57 2 1 119 30 151 2.10E-28 133.7 6565 cellular organisms;Eukaryota;Opisthokonta;Metazoa;Eumetazoa;Bilateria;Protostomia;Spiralia;Lophotrochozoa;Mollusca;Bivalvia;Autobranchia;Pteriomorphia;Ostreida;Ostreoidea;Ostreidae;Crassostrea;Crassostrea virginica
UdeM00000035719 6573 87 UdeM00000035719 XP_021345893.1 30.3 152 83 2 77 227 20 149 3.40E-14 87 6573 cellular organisms;Eukaryota;Opisthokonta;Metazoa;Eumetazoa;Bilateria;Protostomia;Spiralia;Lophotrochozoa;Mollusca;Bivalvia;Autobranchia;Pteriomorphia;Pectinida;Pectinoidea;Pectinidae;Mizuhopecten;Mizuhopecten yessoensis
UdeM00000035721 6596 167.2 UdeM00000035721 XP_045207342.1 57 142 60 1 2 142 3 144 2.00E-38 167.2 6596 cellular organisms;Eukaryota;Opisthokonta;Metazoa;Eumetazoa;Bilateria;Protostomia;Spiralia;Lophotrochozoa;Mollusca;Bivalvia;Autobranchia;Heteroconchia;Euheterodonta;Imparidentia;Venerida;Veneroidea;Veneridae;Mercenaria;Mercenaria mercenaria
UdeM00000035722 6579 109 UdeM00000035722 XP_033750443.1 33.1 172 88 2 14 184 3 148 6.90E-21 109 6579 cellular organisms;Eukaryota;Opisthokonta;Metazoa;Eumetazoa;Bilateria;Protostomia;Spiralia;Lophotrochozoa;Mollusca;Bivalvia;Autobranchia;Pteriomorphia;Pectinida;Pectinoidea;Pectinidae;Pecten;Pecten maximus
UdeM00000035723 6565 109 UdeM00000035723 XP_022321782.1 39.8 176 77 5 13 186 2 150 7.40E-21 109 6565 cellular organisms;Eukaryota;Opisthokonta;Metazoa;Eumetazoa;Bilateria;Protostomia;Spiralia;Lophotrochozoa;Mollusca;Bivalvia;Autobranchia;Pteriomorphia;Ostreida;Ostreoidea;Ostreidae;Crassostrea;Crassostrea virginica
UdeM00000035724 6565 111.3 UdeM00000035724 XP_022321780.1 38.8 170 76 4 19 186 7 150 1.40E-21 111.3 6565 cellular organisms;Eukaryota;Opisthokonta;Metazoa;Eumetazoa;Bilateria;Protostomia;

Spiralia;Lophotrochozoa;Mollusca;Bivalvia;Autobranchia;Pteriomorphia;Ostr
eida;Ostreoidea;Ostreidae;Crassostrea;Crassostrea virginica
UdeM00000035725 7574 151.8 UdeM00000035725 XP_013421302.1 46.5 142
75 1 2 143 3 143 8.10E-34 151.8 7574 cellular
organisms;Eukaryota;Opisthokonta;Metazoa;Eumetazoa;Bilateria;Protostomia;
Spiralia;Lophotrochozoa;Brachiopoda;Linguliformea;Lingulata;Lingulida;Lin
guloidea;Lingulidae;Lingula;Lingula anatina
UdeM00000035726 6579 370.2 UdeM00000035726 XP_033728194.1 43.8 475
254 5 1 466 282 752 4.00E-99 370.2 6579 cellular
organisms;Eukaryota;Opisthokonta;Metazoa;Eumetazoa;Bilateria;Protostomia;
Spiralia;Lophotrochozoa;Mollusca;Bivalvia;Autobranchia;Pteriomorphia;Pect
inida;Pectinoidea;Pectinidae;Pecten;Pecten maximus
UdeM00000035727 6565 112.8 UdeM00000035727 XP_022321782.1 35 163
95 3 8 161 9 169 4.90E-22 112.8 6565 cellular
organisms;Eukaryota;Opisthokonta;Metazoa;Eumetazoa;Bilateria;Protostomia;
Spiralia;Lophotrochozoa;Mollusca;Bivalvia;Autobranchia;Pteriomorphia;Ostr
eida;Ostreoidea;Ostreidae;Crassostrea;Crassostrea virginica
UdeM00000035728 6565 507.3 UdeM00000035728 XP_022338256.1 61.4 438
155 6 16 445 56 487 2.00E-140 507.3 6565 cellular
organisms;Eukaryota;Opisthokonta;Metazoa;Eumetazoa;Bilateria;Protostomia;
Spiralia;Lophotrochozoa;Mollusca;Bivalvia;Autobranchia;Pteriomorphia;Ostr
eida;Ostreoidea;Ostreidae;Crassostrea;Crassostrea virginica
UdeM00000035729 6565 502.7 UdeM00000035729 XP_022338256.1 60.4 445
162 6 10 446 49 487 4.90E-139 502.7 6565 cellular
organisms;Eukaryota;Opisthokonta;Metazoa;Eumetazoa;Bilateria;Protostomia;
Spiralia;Lophotrochozoa;Mollusca;Bivalvia;Autobranchia;Pteriomorphia;Ostr
eida;Ostreoidea;Ostreidae;Crassostrea;Crassostrea virginica
UdeM00000035730 6565 502.3 UdeM00000035730 XP_022338258.1 63.9 416
135 7 7 413 72 481 5.90E-139 502.3 6565 cellular
organisms;Eukaryota;Opisthokonta;Metazoa;Eumetazoa;Bilateria;Protostomia;
Spiralia;Lophotrochozoa;Mollusca;Bivalvia;Autobranchia;Pteriomorphia;Ostr
eida;Ostreoidea;Ostreidae;Crassostrea;Crassostrea virginica
UdeM00000035732 6573 299.7 UdeM00000035732 XP_021374853.1 73.2 183
49 0 1 183 404 586 2.60E-78 299.7 6573 cellular
organisms;Eukaryota;Opisthokonta;Metazoa;Eumetazoa;Bilateria;Protostomia;
Spiralia;Lophotrochozoa;Mollusca;Bivalvia;Autobranchia;Pteriomorphia;Pect
inida;Pectinoidea;Pectinidae;Mizuhopecten;Mizuhopecten yessoensis
UdeM00000035733 6526 166.4 UdeM00000035733 XP_013091220.1 80.6 98
19 0 3 100 131 228 2.20E-38 166.4 6526 cellular
organisms;Eukaryota;Opisthokonta;Metazoa;Eumetazoa;Bilateria;Protostomia;
Spiralia;Lophotrochozoa;Mollusca;Gastropoda;Heterobranchia;Euthyneura;Pan
pulmonata;Hygrophila;Lymnaeoidea;Planorbidae;Biomphalaria;Biomphalaria
glabrata
UdeM00000035734 6526 166.4 UdeM00000035734 XP_013091220.1 80.6 98
19 0 3 100 131 228 2.20E-38 166.4 6526 cellular
organisms;Eukaryota;Opisthokonta;Metazoa;Eumetazoa;Bilateria;Protostomia;
Spiralia;Lophotrochozoa;Mollusca;Gastropoda;Heterobranchia;Euthyneura;Pan
pulmonata;Hygrophila;Lymnaeoidea;Planorbidae;Biomphalaria;Biomphalaria
glabrata
UdeM00000035735 6573 187.2 UdeM00000035735 XP_021358159.1 36 303
181 6 1 293 361 660 4.70E-44 187.2 6573 cellular
organisms;Eukaryota;Opisthokonta;Metazoa;Eumetazoa;Bilateria;Protostomia;
Spiralia;Lophotrochozoa;Mollusca;Bivalvia;Autobranchia;Pteriomorphia;Pect
inida;Pectinoidea;Pectinidae;Mizuhopecten;Mizuhopecten yessoensis

UdeM00000035736 6500 309.7 UdeM00000035736 XP_012936360.1 68.9 219
68 0 740 958 42 260 1.30E-80 309.7 6500 cellular
organisms;Eukaryota;Opisthokonta;Metazoa;Eumetazoa;Bilateria;Protostomia;
Spiralia;Lophotrochozoa;Mollusca;Gastropoda;Heterobranchia;Euthyneura;Tec
tipleura;Aplysiida;Aplysioidea;Aplysiidae;Aplysia;Aplysia californica
UdeM00000035737 6565 121.3 UdeM00000035737 XP_022344036.1 64.3 84
30 0 6 89 4 87 6.20E-25 121.3 6565 cellular
organisms;Eukaryota;Opisthokonta;Metazoa;Eumetazoa;Bilateria;Protostomia;
Spiralia;Lophotrochozoa;Mollusca;Bivalvia;Autobranchia;Pteriomorphia;Ostr
eida;Ostreoidea;Ostreidae;Crassostrea;Crassostrea virginica
UdeM00000035738 7574 860.1 UdeM00000035738 XP_013413356.1 63.9 682
223 10 1084 1758 388 1053 4.80E-246 860.1 7574 cellular
organisms;Eukaryota;Opisthokonta;Metazoa;Eumetazoa;Bilateria;Protostomia;
Spiralia;Lophotrochozoa;Brachiopoda;Linguliformea;Lingulata;Lingulida;Lin
guloidea;Lingulidae;Lingula;Lingula anatina
UdeM00000042334 400727 215.7 UdeM00000042334 XP_025089961.1 59.5
190 2 1 1 190 1 115 5.10E-53 215.7 400727
cellular
organisms;Eukaryota;Opisthokonta;Metazoa;Eumetazoa;Bilateria;Protostomia;
Spiralia;Lophotrochozoa;Mollusca;Gastropoda;Caenogastropoda;Architaeniogl
ossa;Ampullarioidea;Ampullariidae;Pomacea;Pomacea canaliculata
UdeM00000042335 1735272 301.2 UdeM00000042335 XP_041349021.1 50
286 143 0 5 290 43 328 1.40E-78 301.2 1735272
cellular
organisms;Eukaryota;Opisthokonta;Metazoa;Eumetazoa;Bilateria;Protostomia;
Spiralia;Lophotrochozoa;Mollusca;Gastropoda;Neomphalina;Neomphaloidea;Pel
tospiridae;Gigantopelta;Gigantopelta aegis
UdeM00000042336 7574 174.9 UdeM00000042336 XP_013416544.1 73.8 126
33 0 4 129 3 128 6.90E-41 174.9 7574 cellular
organisms;Eukaryota;Opisthokonta;Metazoa;Eumetazoa;Bilateria;Protostomia;
Spiralia;Lophotrochozoa;Brachiopoda;Linguliformea;Lingulata;Lingulida;Lin
guloidea;Lingulidae;Lingula;Lingula anatina
UdeM00000042337 6500 700.7 UdeM00000042337 XP_005090939.1 78.2 496
108 0 1 496 1 496 1.40E-198 700.7 6500 cellular
organisms;Eukaryota;Opisthokonta;Metazoa;Eumetazoa;Bilateria;Protostomia;
Spiralia;Lophotrochozoa;Mollusca;Gastropoda;Heterobranchia;Euthyneura;Tec
tipleura;Aplysiida;Aplysioidea;Aplysiidae;Aplysia;Aplysia californica
UdeM00000042338 6500 517.3 UdeM00000042338 XP_005090939.1 80.7 337
65 0 1 337 160 496 1.50E-143 517.3 6500 cellular
organisms;Eukaryota;Opisthokonta;Metazoa;Eumetazoa;Bilateria;Protostomia;
Spiralia;Lophotrochozoa;Mollusca;Gastropoda;Heterobranchia;Euthyneura;Tec
tipleura;Aplysiida;Aplysioidea;Aplysiidae;Aplysia;Aplysia californica
UdeM00000042340 29159 65.9 UdeM00000042340 XP_034307878.1 30.5 95
66 0 5 99 96 190 4.80E-08 65.9 29159 cellular
organisms;Eukaryota;Opisthokonta;Metazoa;Eumetazoa;Bilateria;Protostomia;
Spiralia;Lophotrochozoa;Mollusca;Bivalvia;Autobranchia;Pteriomorphia;Ostr
eida;Ostreoidea;Ostreidae;Crassostrea;Crassostrea gigas
UdeM00000042341 6573 189.5 UdeM00000042341 XP_021340815.1 47.9 188
86 2 2 187 3 180 4.00E-45 189.5 6573 cellular
organisms;Eukaryota;Opisthokonta;Metazoa;Eumetazoa;Bilateria;Protostomia;
Spiralia;Lophotrochozoa;Mollusca;Bivalvia;Autobranchia;Pteriomorphia;Pect
inida;Pectinoidea;Pectinidae;Mizuhopecten;Mizuhopecten yessoensis
UdeM00000042342 6596 468 UdeM00000042342 XP_045172771.1 46.4 534
268 7 1 524 1 526 1.60E-128 468 6596 cellular

organisms;Eukaryota;Opisthokonta;Metazoa;Eumetazoa;Bilateria;Protostomia;Spiralia;Lophotrochozoa;Mollusca;Bivalvia;Autobranchia;Heteroconchia;Euheterodonta;Imparidentia;Venerida;Veneroidea;Veneridae;Mercenaria;Mercenaria mercenaria
UdeM00000042343 6596 877.5 UdeM00000042343 XP_045171607.1 65.3 1010 336 5 1 1004 1 1002 1.60E-251 877.5 6596 cellular organisms;Eukaryota;Opisthokonta;Metazoa;Eumetazoa;Bilateria;Protostomia;Spiralia;Lophotrochozoa;Mollusca;Bivalvia;Autobranchia;Heteroconchia;Euheterodonta;Imparidentia;Venerida;Veneroidea;Veneridae;Mercenaria;Mercenaria mercenaria
UdeM00000042345 6596 1282.3 UdeM00000042345 XP_045173271.1 73.5 823 215 2 52 874 52 871 0.00E+00 1282.3 6596 cellular organisms;Eukaryota;Opisthokonta;Metazoa;Eumetazoa;Bilateria;Protostomia;Spiralia;Lophotrochozoa;Mollusca;Bivalvia;Autobranchia;Heteroconchia;Euheterodonta;Imparidentia;Venerida;Veneroidea;Veneridae;Mercenaria;Mercenaria mercenaria
UdeM00000042346 6565 290.4 UdeM00000042346 XP_022341463.1 67.1 207 64 2 53 259 53 255 2.40E-75 290.4 6565 cellular organisms;Eukaryota;Opisthokonta;Metazoa;Eumetazoa;Bilateria;Protostomia;Spiralia;Lophotrochozoa;Mollusca;Bivalvia;Autobranchia;Pteriomorphia;Ostreida;Ostreoidea;Ostreidae;Crassostrea;Crassostrea virginica
UdeM00000042347 6579 186.4 UdeM00000042347 XP_033747281.1 59.7 139 54 1 52 190 55 191 3.40E-44 186.4 6579 cellular organisms;Eukaryota;Opisthokonta;Metazoa;Eumetazoa;Bilateria;Protostomia;Spiralia;Lophotrochozoa;Mollusca;Bivalvia;Autobranchia;Pteriomorphia;Pectinida;Pectinoidea;Pectinidae;Pecten;Pecten maximus
UdeM00000042348 29159 429.1 UdeM00000042348 XP_034338151.1 53.4 416 174 4 20 416 35 449 6.40E-117 429.1 29159 cellular organisms;Eukaryota;Opisthokonta;Metazoa;Eumetazoa;Bilateria;Protostomia;Spiralia;Lophotrochozoa;Mollusca;Bivalvia;Autobranchia;Pteriomorphia;Ostreida;Ostreoidea;Ostreidae;Crassostrea;Crassostrea gigas
UdeM00000042349 6596 1330.9 UdeM00000042349 XP_045173270.1 76.3 823 192 2 52 874 52 871 0.00E+00 1330.9 6596 cellular organisms;Eukaryota;Opisthokonta;Metazoa;Eumetazoa;Bilateria;Protostomia;Spiralia;Lophotrochozoa;Mollusca;Bivalvia;Autobranchia;Heteroconchia;Euheterodonta;Imparidentia;Venerida;Veneroidea;Veneridae;Mercenaria;Mercenaria mercenaria
UdeM00000042350 6596 66.6 UdeM00000042350 XP_045172255.1 23.6 368 209 13 15 334 40 383 7.20E-08 66.6 6596 cellular organisms;Eukaryota;Opisthokonta;Metazoa;Eumetazoa;Bilateria;Protostomia;Spiralia;Lophotrochozoa;Mollusca;Bivalvia;Autobranchia;Heteroconchia;Euheterodonta;Imparidentia;Venerida;Veneroidea;Veneridae;Mercenaria;Mercenaria mercenaria
UdeM00000042351 6573 280.8 UdeM00000042351 XP_021371351.1 66.2 358 117 3 2 356 350 706 2.40E-72 280.8 6573 cellular organisms;Eukaryota;Opisthokonta;Metazoa;Eumetazoa;Bilateria;Protostomia;Spiralia;Lophotrochozoa;Mollusca;Bivalvia;Autobranchia;Pteriomorphia;Pectinida;Pectinoidea;Pectinidae;Mizuhopecten;Mizuhopecten yessoensis
UdeM00000042352 6579 735.3 UdeM00000042352 XP_033747709.1 64.1 701 248 3 6 703 7 706 7.10E-209 735.3 6579 cellular organisms;Eukaryota;Opisthokonta;Metazoa;Eumetazoa;Bilateria;Protostomia;

Spiralia;Lophotrochozoa;Mollusca;Bivalvia;Autobranchia;Pteriomorphia;Pectinida;Pectinoidea;Pectinidae;Pecten;Pecten maximus
UdeM00000042353 6596 515 UdeM00000042353 XP_045173758.1 52.5 465 213 3 35 499 19 475 1.10E-142 515 6596 cellular organisms;Eukaryota;Opisthokonta;Metazoa;Eumetazoa;Bilateria;Protostomia;Spiralia;Lophotrochozoa;Mollusca;Bivalvia;Autobranchia;Heteroconchia;Euheterodonta;Imparidentia;Venerida;Veneroidea;Veneridae;Mercenaria;Mercenaria mercenaria
UdeM00000042355 6579 268.9 UdeM00000042355 XP_033747711.1 64.9 211 73 1 1 210 803 1013 5.70E-69 268.9 6579 cellular organisms;Eukaryota;Opisthokonta;Metazoa;Eumetazoa;Bilateria;Protostomia;Spiralia;Lophotrochozoa;Mollusca;Bivalvia;Autobranchia;Pteriomorphia;Pectinida;Pectinoidea;Pectinidae;Pecten;Pecten maximus
UdeM00000042354 6579 270.4 UdeM00000042354 XP_033747711.1 65.1 212 73 1 2 212 802 1013 2.00E-69 270.4 6579 cellular organisms;Eukaryota;Opisthokonta;Metazoa;Eumetazoa;Bilateria;Protostomia;Spiralia;Lophotrochozoa;Mollusca;Bivalvia;Autobranchia;Pteriomorphia;Pectinida;Pectinoidea;Pectinidae;Pecten;Pecten maximus
UdeM00000042356 6596 194.5 UdeM00000042356 XP_045172776.1 40.2 358 170 14 11 357 435 759 2.30E-46 194.5 6596 cellular organisms;Eukaryota;Opisthokonta;Metazoa;Eumetazoa;Bilateria;Protostomia;Spiralia;Lophotrochozoa;Mollusca;Bivalvia;Autobranchia;Heteroconchia;Euheterodonta;Imparidentia;Venerida;Veneroidea;Veneridae;Mercenaria;Mercenaria mercenaria
UdeM00000042357 6596 493 UdeM00000042357 XP_045172777.1 67.5 421 112 6 1 417 1 400 4.30E-136 493 6596 cellular organisms;Eukaryota;Opisthokonta;Metazoa;Eumetazoa;Bilateria;Protostomia;Spiralia;Lophotrochozoa;Mollusca;Bivalvia;Autobranchia;Heteroconchia;Euheterodonta;Imparidentia;Venerida;Veneroidea;Veneridae;Mercenaria;Mercenaria mercenaria
UdeM00000042358 7668 75.5 UdeM00000042358 XP_030841875.1 50 64 31 1 3 66 71 133 5.80E-11 75.5 7668 cellular organisms;Eukaryota;Opisthokonta;Metazoa;Eumetazoa;Bilateria;Deuterostomia;Echinodermata;Eleutherozoa;Echinozoa;Echinoidea;Euechinoidea;Echinacea;Camarodonta;Echinidea;Strongylocentrotidae;Strongylocentrotus;Strongylocentrotus purpuratus
UdeM00000042359 6596 699.1 UdeM00000042359 XP_045172777.1 50.1 857 347 19 5 818 121 939 6.70E-198 699.1 6596 cellular organisms;Eukaryota;Opisthokonta;Metazoa;Eumetazoa;Bilateria;Protostomia;Spiralia;Lophotrochozoa;Mollusca;Bivalvia;Autobranchia;Heteroconchia;Euheterodonta;Imparidentia;Venerida;Veneroidea;Veneridae;Mercenaria;Mercenaria mercenaria
UdeM00000042360 1735272 109 UdeM00000042360 XP_041349948.1 70 80 22 1 1 80 1 78 1.10E-20 109 1735272 cellular organisms;Eukaryota;Opisthokonta;Metazoa;Eumetazoa;Bilateria;Protostomia;Spiralia;Lophotrochozoa;Mollusca;Gastropoda;Neomphalina;Neomphaloidea;Peltospiridae;Gigantopelta;Gigantopelta aegis
UdeM00000042361 6596 476.9 UdeM00000042361 XP_045173648.1 38.9 769 369 12 1 739 1 698 4.80E-131 476.9 6596 cellular organisms;Eukaryota;Opisthokonta;Metazoa;Eumetazoa;Bilateria;Protostomia;Spiralia;Lophotrochozoa;Mollusca;Bivalvia;Autobranchia;Heteroconchia;Euheterodonta;Imparidentia;Venerida;Veneroidea;Veneridae;Mercenaria;Mercenaria mercenaria

UdeM00000042362 6573 131.3 UdeM00000042362 XP_021349751.1 30.5 321
198 4 1 315 2 303 2.30E-27 131.3 6573 cellular organisms;Eukaryota;Opisthokonta;Metazoa;Eumetazoa;Bilateria;Protostomia;Spiralia;Lophotrochozoa;Mollusca;Bivalvia;Autobranchia;Pteriomorphia;Pectinida;Pectinoidea;Pectinidae;Mizuhopecten;Mizuhopecten yessoensis
UdeM00000042363 6579 589 UdeM00000042363 XP_033748230.1 60.9 463
167 3 2 451 144 605 5.30E-165 589 6579 cellular organisms;Eukaryota;Opisthokonta;Metazoa;Eumetazoa;Bilateria;Protostomia;Spiralia;Lophotrochozoa;Mollusca;Bivalvia;Autobranchia;Pteriomorphia;Pectinida;Pectinoidea;Pectinidae;Pecten;Pecten maximus
UdeM00000042364 2607531 193.4 UdeM00000042364 XP_036359789.1 82.5
103 18 0 6 108 27 129 1.70E-46 193.4 2607531
cellular organisms;Eukaryota;Opisthokonta;Metazoa;Eumetazoa;Bilateria;Protostomia;Spiralia;Lophotrochozoa;Mollusca;Cephalopoda;Coleoidea;Octopodiformes;Octopoda;Incirrata;Octopodidae;Octopus;Octopus sinensis
UdeM00000042365 6596 259.2 UdeM00000042365 XP_045171870.1 67.8 177
57 0 29 205 458 634 5.40E-66 259.2 6596 cellular organisms;Eukaryota;Opisthokonta;Metazoa;Eumetazoa;Bilateria;Protostomia;Spiralia;Lophotrochozoa;Mollusca;Bivalvia;Autobranchia;Heteroconchia;Euheterodonta;Imparidentia;Venerida;Veneroidea;Veneridae;Mercenaria;Mercenaria mercenaria
UdeM00000042366 6579 433.3 UdeM00000042366 XP_033747389.1 70.6 279
82 0 1 279 206 484 2.30E-118 433.3 6579 cellular organisms;Eukaryota;Opisthokonta;Metazoa;Eumetazoa;Bilateria;Protostomia;Spiralia;Lophotrochozoa;Mollusca;Bivalvia;Autobranchia;Pteriomorphia;Pectinida;Pectinoidea;Pectinidae;Pecten;Pecten maximus
UdeM00000042367 6565 97.4 UdeM00000042367 XP_022335714.1 49.1 110
39 4 47 154 96 190 1.70E-17 97.4 6565 cellular organisms;Eukaryota;Opisthokonta;Metazoa;Eumetazoa;Bilateria;Protostomia;Spiralia;Lophotrochozoa;Mollusca;Bivalvia;Autobranchia;Pteriomorphia;Ostreida;Ostreoidea;Ostreidae;Crassostrea;Crassostrea virginica
UdeM00000042368 6579 3042.3 UdeM00000042368 XP_033748694.1 92.1
1641 130 0 147 1787 15 1655 0.00E+00 3042.3 6579
cellular organisms;Eukaryota;Opisthokonta;Metazoa;Eumetazoa;Bilateria;Protostomia;Spiralia;Lophotrochozoa;Mollusca;Bivalvia;Autobranchia;Pteriomorphia;Pectinida;Pectinoidea;Pectinidae;Pecten;Pecten maximus
UdeM00000042369 37653 206.1 UdeM00000042369 XP_014789336.1 78.8 137
29 0 1 137 1 137 3.10E-50 206.1 37653 cellular organisms;Eukaryota;Opisthokonta;Metazoa;Eumetazoa;Bilateria;Protostomia;Spiralia;Lophotrochozoa;Mollusca;Cephalopoda;Coleoidea;Octopodiformes;Octopoda;Incirrata;Octopodidae;Octopus;Octopus bimaculoides
UdeM00000042370 6526 110.2 UdeM00000042370 XP_013067933.1 82.4 68
12 0 1 68 1 68 1.90E-21 110.2 6526 cellular organisms;Eukaryota;Opisthokonta;Metazoa;Eumetazoa;Bilateria;Protostomia;Spiralia;Lophotrochozoa;Mollusca;Gastropoda;Heterobranchia;Euthyneura;Panpulmonata;Hygrophila;Lymnaeoidea;Planorbidae;Biomphalaria;Biomphalaria glabrata
UdeM00000042371 6596 252.7 UdeM00000042371 XP_045173333.1 43.5 306
123 3 5 261 7 311 5.20E-64 252.7 6596 cellular organisms;Eukaryota;Opisthokonta;Metazoa;Eumetazoa;Bilateria;Protostomia;Spiralia;Lophotrochozoa;Mollusca;Bivalvia;Autobranchia;Heteroconchia;Euhe

terodonta;Imparidentia;Venerida;Veneroidea;Veneridae;Mercenaria;Mercenaria mercenaria
UdeM00000042372 6596 178.7 UdeM00000042372 XP_045181277.1 33.7 306
187 8 41 338 132 429 1.20E-41 178.7 6596 cellular
organisms;Eukaryota;Opisthokonta;Metazoa;Eumetazoa;Bilateria;Protostomia;Spiralia;Lophotrochozoa;Mollusca;Bivalvia;Autobranchia;Heteroconchia;Euheterodonta;Imparidentia;Venerida;Veneroidea;Veneridae;Mercenaria;Mercenaria mercenaria
UdeM00000042373 6596 153.7 UdeM00000042373 XP_045173647.1 38.2 207
126 2 1 205 226 432 2.60E-34 153.7 6596 cellular
organisms;Eukaryota;Opisthokonta;Metazoa;Eumetazoa;Bilateria;Protostomia;Spiralia;Lophotrochozoa;Mollusca;Bivalvia;Autobranchia;Heteroconchia;Euheterodonta;Imparidentia;Venerida;Veneroidea;Veneridae;Mercenaria;Mercenaria mercenaria
UdeM00000042374 6596 204.9 UdeM00000042374 XP_045181277.1 35.7 322
189 10 17 327 108 422 1.60E-49 204.9 6596 cellular
organisms;Eukaryota;Opisthokonta;Metazoa;Eumetazoa;Bilateria;Protostomia;Spiralia;Lophotrochozoa;Mollusca;Bivalvia;Autobranchia;Heteroconchia;Euheterodonta;Imparidentia;Venerida;Veneroidea;Veneridae;Mercenaria;Mercenaria mercenaria
UdeM00000042375 6596 63.5 UdeM00000042375 XP_045174485.1 31.5 127
82 4 30 155 651 773 3.90E-07 63.5 6596 cellular
organisms;Eukaryota;Opisthokonta;Metazoa;Eumetazoa;Bilateria;Protostomia;Spiralia;Lophotrochozoa;Mollusca;Bivalvia;Autobranchia;Heteroconchia;Euheterodonta;Imparidentia;Venerida;Veneroidea;Veneridae;Mercenaria;Mercenaria mercenaria
UdeM00000043537 29159 119 UdeM00000043537 XP_011449152.1 66.3 92
29 2 15 106 61 150 3.70E-24 119 29159 cellular
organisms;Eukaryota;Opisthokonta;Metazoa;Eumetazoa;Bilateria;Protostomia;Spiralia;Lophotrochozoa;Mollusca;Bivalvia;Autobranchia;Pteriomorphia;Ostreida;Ostreoidea;Ostreidae;Crassostrea;Crassostrea gigas
UdeM00000043538 6596 907.5 UdeM00000043538 XP_045199690.1 51.9 930
388 12 8 918 2 891 1.40E-260 907.5 6596 cellular
organisms;Eukaryota;Opisthokonta;Metazoa;Eumetazoa;Bilateria;Protostomia;Spiralia;Lophotrochozoa;Mollusca;Bivalvia;Autobranchia;Heteroconchia;Euheterodonta;Imparidentia;Venerida;Veneroidea;Veneridae;Mercenaria;Mercenaria mercenaria
UdeM00000043539 6596 704.5 UdeM00000043539 XP_045199690.1 50.6 769
322 13 1 748 160 891 1.40E-199 704.5 6596 cellular
organisms;Eukaryota;Opisthokonta;Metazoa;Eumetazoa;Bilateria;Protostomia;Spiralia;Lophotrochozoa;Mollusca;Bivalvia;Autobranchia;Heteroconchia;Euheterodonta;Imparidentia;Venerida;Veneroidea;Veneridae;Mercenaria;Mercenaria mercenaria
UdeM00000043540 6596 1349.7 UdeM00000043540 XP_045199705.1 66.7
1080 320 7 5 1048 301 1376 0.00E+00 1349.7 6596
cellular
organisms;Eukaryota;Opisthokonta;Metazoa;Eumetazoa;Bilateria;Protostomia;Spiralia;Lophotrochozoa;Mollusca;Bivalvia;Autobranchia;Heteroconchia;Euheterodonta;Imparidentia;Venerida;Veneroidea;Veneridae;Mercenaria;Mercenaria mercenaria
UdeM00000043541 37621 98.2 UdeM00000043541 XP_014678683.1 60 70
28 0 1 70 59 128 8.50E-18 98.2 37621 cellular
organisms;Eukaryota;Opisthokonta;Metazoa;Eumetazoa;Bilateria;Protostomia;

Ecdysozoa;Scalidophora;Priapulida;Priapulimorpha;Priapulimorphida;Priapulidae;Priapulus;Priapulus caudatus
UdeM00000043542 6596 461.8 UdeM00000043542 XP_045199705.1 80.4 275 54 0 1 275 27 301 5.90E-127 461.8 6596 cellular organisms;Eukaryota;Opisthokonta;Metazoa;Eumetazoa;Bilateria;Protostomia;Spiralia;Lophotrochozoa;Mollusca;Bivalvia;Autobranchia;Heteroconchia;Euheterodonta;Imparidentia;Venerida;Veneroidea;Veneridae;Mercenaria;Mercenaria mercenaria
UdeM00000043543 6573 715.3 UdeM00000043543 XP_021372077.1 80 444 76 4 1 440 1 435 5.50E-203 715.3 6573 cellular organisms;Eukaryota;Opisthokonta;Metazoa;Eumetazoa;Bilateria;Protostomia;Spiralia;Lophotrochozoa;Mollusca;Bivalvia;Autobranchia;Pteriomorphia;Pectinida;Pectinoidea;Pectinidae;Mizuhopecten;Mizuhopecten yessoensis
UdeM00000043544 6596 187.6 UdeM00000043544 XP_045177580.1 33.1 359 212 10 16 369 13 348 3.50E-44 187.6 6596 cellular organisms;Eukaryota;Opisthokonta;Metazoa;Eumetazoa;Bilateria;Protostomia;Spiralia;Lophotrochozoa;Mollusca;Bivalvia;Autobranchia;Heteroconchia;Euheterodonta;Imparidentia;Venerida;Veneroidea;Veneridae;Mercenaria;Mercenaria mercenaria
UdeM00000043545 7913 164.5 UdeM00000043545 XP_041090681.1 23.2 626 444 18 1 603 1809 2420 4.30E-37 164.5 7913 cellular organisms;Eukaryota;Opisthokonta;Metazoa;Eumetazoa;Bilateria;Deuterostomia;Chordata;Craniata;Vertebrata;Gnathostomata;Teleostomi;Euteleostomi;Actinopterygii;Actinopteri;Chondrostei;Acipenseriformes;Acipenseroidei;Polyodontidae;Polyodontinae;Polyodontini;Polyodon;Polyodon spathula
UdeM00000043546 6596 286.2 UdeM00000043546 XP_045200257.1 30.7 610 378 13 7 580 124 724 9.40E-74 286.2 6596 cellular organisms;Eukaryota;Opisthokonta;Metazoa;Eumetazoa;Bilateria;Protostomia;Spiralia;Lophotrochozoa;Mollusca;Bivalvia;Autobranchia;Heteroconchia;Euheterodonta;Imparidentia;Venerida;Veneroidea;Veneridae;Mercenaria;Mercenaria mercenaria
UdeM00000043547 6596 298.1 UdeM00000043547 XP_045200267.1 32.4 599 367 16 2 583 123 700 2.40E-77 298.1 6596 cellular organisms;Eukaryota;Opisthokonta;Metazoa;Eumetazoa;Bilateria;Protostomia;Spiralia;Lophotrochozoa;Mollusca;Bivalvia;Autobranchia;Heteroconchia;Euheterodonta;Imparidentia;Venerida;Veneroidea;Veneridae;Mercenaria;Mercenaria mercenaria
UdeM00000043549 6596 361.7 UdeM00000043549 XP_045202682.1 33.2 719 429 11 57 760 884 1566 2.30E-96 361.7 6596 cellular organisms;Eukaryota;Opisthokonta;Metazoa;Eumetazoa;Bilateria;Protostomia;Spiralia;Lophotrochozoa;Mollusca;Bivalvia;Autobranchia;Heteroconchia;Euheterodonta;Imparidentia;Venerida;Veneroidea;Veneridae;Mercenaria;Mercenaria mercenaria
UdeM00000043550 6596 361.7 UdeM00000043550 XP_045202682.1 33.2 719 429 11 57 760 884 1566 2.30E-96 361.7 6596 cellular organisms;Eukaryota;Opisthokonta;Metazoa;Eumetazoa;Bilateria;Protostomia;Spiralia;Lophotrochozoa;Mollusca;Bivalvia;Autobranchia;Heteroconchia;Euheterodonta;Imparidentia;Venerida;Veneroidea;Veneridae;Mercenaria;Mercenaria mercenaria
UdeM00000043551 6596 361.7 UdeM00000043551 XP_045202682.1 33.2 719 429 11 57 760 884 1566 2.30E-96 361.7 6596 cellular organisms;Eukaryota;Opisthokonta;Metazoa;Eumetazoa;Bilateria;Protostomia;Spiralia;Lophotrochozoa;Mollusca;Bivalvia;Autobranchia;Heteroconchia;Euhe

terodonta;Imparidentia;Venerida;Veneroidea;Veneridae;Mercenaria;Mercenaria mercenaria
UdeM00000043552 6596 361.7 UdeM00000043552 XP_045202682.1 33.2 719 429 11 57 760 884 1566 2.30E-96 361.7 6596 cellular organisms;Eukaryota;Opisthokonta;Metazoa;Eumetazoa;Bilateria;Protostomia;Spiralia;Lophotrochozoa;Mollusca;Bivalvia;Autobranchia;Heteroconchia;Euheterodonta;Imparidentia;Venerida;Veneroidea;Veneridae;Mercenaria;Mercenaria mercenaria
UdeM00000043553 6596 363.2 UdeM00000043553 XP_045202682.1 33.4 719 428 12 57 760 884 1566 7.90E-97 363.2 6596 cellular organisms;Eukaryota;Opisthokonta;Metazoa;Eumetazoa;Bilateria;Protostomia;Spiralia;Lophotrochozoa;Mollusca;Bivalvia;Autobranchia;Heteroconchia;Euheterodonta;Imparidentia;Venerida;Veneroidea;Veneridae;Mercenaria;Mercenaria mercenaria
UdeM00000043554 6596 221.1 UdeM00000043554 XP_045181277.1 40.4 285 157 7 189 466 127 405 3.10E-54 221.1 6596 cellular organisms;Eukaryota;Opisthokonta;Metazoa;Eumetazoa;Bilateria;Protostomia;Spiralia;Lophotrochozoa;Mollusca;Bivalvia;Autobranchia;Heteroconchia;Euheterodonta;Imparidentia;Venerida;Veneroidea;Veneridae;Mercenaria;Mercenaria mercenaria
UdeM00000043555 27687 562 UdeM00000043555 XP_028648107.1 26.5 1655 1060 44 163 1728 342 1929 2.60E-156 562 27687 cellular organisms;Eukaryota;Opisthokonta;Metazoa;Eumetazoa;Bilateria;Deuterostomia;Chordata;Craniata;Vertebrata;Gnathostomata;Teleostomi;Euteleostomi;Actinopterygii;Cladistia;Polypteriformes;Polypteridae;Erpetoichthys;Erpetoichthys calabaricus
UdeM00000043556 8384 156.8 UdeM00000043556 XP_040263820.1 30.6 372 234 12 12 375 47 402 5.60E-35 156.8 8384 cellular organisms;Eukaryota;Opisthokonta;Metazoa;Eumetazoa;Bilateria;Deuterostomia;Chordata;Craniata;Vertebrata;Gnathostomata;Teleostomi;Euteleostomi;Sarcopterygii;Dipnotetrapodomorpha;Tetrapoda;Amphibia;Batrachia;Anura;Neobatrachia;Hyloidea;Bufonidae;Bufo;Bufo bufo
UdeM00000043557 6850 147.9 UdeM00000043557 XP_013775720.2 29.4 320 209 8 109 425 171 476 3.00E-32 147.9 6850 cellular organisms;Eukaryota;Opisthokonta;Metazoa;Eumetazoa;Bilateria;Protostomia;Ecdysozoa;Panarthropoda;Arthropoda;Chelicerata;Merostomata;Xiphosura;Limulidae;Limulus;Limulus polyphemus
UdeM00000043558 6412 115.5 UdeM00000043558 XP_009031446.1 27.6 257 170 8 1 248 244 493 9.40E-23 115.5 6412 cellular organisms;Eukaryota;Opisthokonta;Metazoa;Eumetazoa;Bilateria;Protostomia;Spiralia;Lophotrochozoa;Annelida;Clitellata;Hirudinea;Rhynchobdellida;Glossiphoniidae;Helobdella;Helobdella robusta
UdeM00000043559 6412 164.1 UdeM00000043559 XP_009031446.1 28.2 365 241 11 83 433 136 493 4.00E-37 164.1 6412 cellular organisms;Eukaryota;Opisthokonta;Metazoa;Eumetazoa;Bilateria;Protostomia;Spiralia;Lophotrochozoa;Annelida;Clitellata;Hirudinea;Rhynchobdellida;Glossiphoniidae;Helobdella;Helobdella robusta
UdeM00000040657 6573 729.6 UdeM00000040657 XP_021345467.1 66.3 812 273 1 10 821 124 934 4.50E-207 729.6 6573 cellular organisms;Eukaryota;Opisthokonta;Metazoa;Eumetazoa;Bilateria;Protostomia;Spiralia;Lophotrochozoa;Mollusca;Bivalvia;Autobranchia;Pteriomorphia;Pectinida;Pectinoidea;Pectinidae;Mizuhopecten;Mizuhopecten yessoensis
UdeM00000040659 29159 99.8 UdeM00000040659 XP_011445731.2 39.8 123 72 2 17 139 227 347 3.40E-18 99.8 29159 cellular

organisms;Eukaryota;Opisthokonta;Metazoa;Eumetazoa;Bilateria;Protostomia;Spiralia;Lophotrochozoa;Mollusca;Bivalvia;Autobranchia;Pteriomorphia;Ostreida;Ostreoidea;Ostreidae;Crassostrea;Crassostrea gigas
UdeM00000040658 29159 102.8 UdeM00000040658 XP_011445731.2 36.8 144 87 3 245 388 208 347 1.00E-18 102.8 29159 cellular organisms;Eukaryota;Opisthokonta;Metazoa;Eumetazoa;Bilateria;Protostomia;Spiralia;Lophotrochozoa;Mollusca;Bivalvia;Autobranchia;Pteriomorphia;Ostreida;Ostreoidea;Ostreidae;Crassostrea;Crassostrea gigas
UdeM00000040660 29159 434.1 UdeM00000040660 XP_034307109.1 42.8 579 317 7 10 577 29 604 2.90E-118 434.1 29159 cellular organisms;Eukaryota;Opisthokonta;Metazoa;Eumetazoa;Bilateria;Protostomia;Spiralia;Lophotrochozoa;Mollusca;Bivalvia;Autobranchia;Pteriomorphia;Ostreida;Ostreoidea;Ostreidae;Crassostrea;Crassostrea gigas
UdeM00000040661 2652724 442.6 UdeM00000040661 XP_020903819.1 48.2 461 223 6 5 449 9 469 6.10E-121 442.6 2652724 cellular organisms;Eukaryota;Opisthokonta;Metazoa;Eumetazoa;Cnidaria;Anthozoa;Hexacorallia;Actiniaria;Aiptasiidae;Exaiptasia;Exaiptasia diaphana
UdeM00000040662 6596 252.7 UdeM00000040662 XP_045204432.1 48 248 126 2 10 254 105 352 5.00E-64 252.7 6596 cellular organisms;Eukaryota;Opisthokonta;Metazoa;Eumetazoa;Bilateria;Protostomia;Spiralia;Lophotrochozoa;Mollusca;Bivalvia;Autobranchia;Heteroconchia;Euheterodonta;Imparidentia;Venerida;Veneroidea;Veneridae;Mercenaria;Mercenaria mercenaria
UdeM00000040663 6573 491.1 UdeM00000040663 XP_021353682.1 44.8 585 311 5 12 584 39 623 1.90E-135 491.1 6573 cellular organisms;Eukaryota;Opisthokonta;Metazoa;Eumetazoa;Bilateria;Protostomia;Spiralia;Lophotrochozoa;Mollusca;Bivalvia;Autobranchia;Pteriomorphia;Pectinida;Pectinoidea;Pectinidae;Mizuhopecten;Mizuhopecten yessoensis
UdeM00000040664 6596 326.6 UdeM00000040664 XP_045204432.1 43.2 352 197 2 1 349 1 352 3.80E-86 326.6 6596 cellular organisms;Eukaryota;Opisthokonta;Metazoa;Eumetazoa;Bilateria;Protostomia;Spiralia;Lophotrochozoa;Mollusca;Bivalvia;Autobranchia;Heteroconchia;Euheterodonta;Imparidentia;Venerida;Veneroidea;Veneridae;Mercenaria;Mercenaria mercenaria
UdeM00000040666 6596 102.4 UdeM00000040666 XP_045204432.1 50 90 43 1 1 88 263 352 2.90E-19 102.4 6596 cellular organisms;Eukaryota;Opisthokonta;Metazoa;Eumetazoa;Bilateria;Protostomia;Spiralia;Lophotrochozoa;Mollusca;Bivalvia;Autobranchia;Heteroconchia;Euheterodonta;Imparidentia;Venerida;Veneroidea;Veneridae;Mercenaria;Mercenaria mercenaria
UdeM00000040665 6596 139.8 UdeM00000040665 XP_045204432.1 50.9 116 55 1 2 115 237 352 2.20E-30 139.8 6596 cellular organisms;Eukaryota;Opisthokonta;Metazoa;Eumetazoa;Bilateria;Protostomia;Spiralia;Lophotrochozoa;Mollusca;Bivalvia;Autobranchia;Heteroconchia;Euheterodonta;Imparidentia;Venerida;Veneroidea;Veneridae;Mercenaria;Mercenaria mercenaria
UdeM00000040667 6596 205.7 UdeM00000040667 XP_045204432.1 43.8 226 124 2 7 229 6 231 6.40E-50 205.7 6596 cellular organisms;Eukaryota;Opisthokonta;Metazoa;Eumetazoa;Bilateria;Protostomia;Spiralia;Lophotrochozoa;Mollusca;Bivalvia;Autobranchia;Heteroconchia;Euheterodonta;Imparidentia;Venerida;Veneroidea;Veneridae;Mercenaria;Mercenaria mercenaria

UdeM00000044439 83332 238 UdeM00000044439 NP_217959.1 93.1 130 9 0 393 522 22 151 2.60E-59 238 83332 cellular organisms;Bacteria;Terrabacteria group;Actinobacteria;Actinomycetia;Corynebacteriales;Mycobacteriaceae;Mycobacterium;Mycobacterium tuberculosis complex;Mycobacterium tuberculosis;Mycobacterium tuberculosis H37Rv
UdeM00000043682 6596 436 UdeM00000043682 XP_045187536.1 63.7 331 118 1 1 331 396 724 4.20E-119 436 6596 cellular organisms;Eukaryota;Opisthokonta;Metazoa;Eumetazoa;Bilateria;Protostomia;Spiralia;Lophotrochozoa;Mollusca;Bivalvia;Autobranchia;Heteroconchia;Euheterodonta;Imparidentia;Venerida;Veneroidea;Veneridae;Mercenaria;Mercenaria mercenaria
UdeM00000043683 6596 671 UdeM00000043683 XP_045187536.1 66.6 500 164 2 2 501 228 724 1.20E-189 671 6596 cellular organisms;Eukaryota;Opisthokonta;Metazoa;Eumetazoa;Bilateria;Protostomia;Spiralia;Lophotrochozoa;Mollusca;Bivalvia;Autobranchia;Heteroconchia;Euheterodonta;Imparidentia;Venerida;Veneroidea;Veneridae;Mercenaria;Mercenaria mercenaria
UdeM00000043685 1735272 88.2 UdeM00000043685 XP_041362151.1 38.9 113 68 1 9 120 563 675 8.50E-15 88.2 1735272 cellular organisms;Eukaryota;Opisthokonta;Metazoa;Eumetazoa;Bilateria;Protostomia;Spiralia;Lophotrochozoa;Mollusca;Gastropoda;Neomphalina;Neomphaloidea;Peltospiridae;Gigantopelta;Gigantopelta aegis
UdeM00000043687 6579 262.7 UdeM00000043687 XP_033761495.1 66.3 184 59 1 36 219 24 204 4.20E-67 262.7 6579 cellular organisms;Eukaryota;Opisthokonta;Metazoa;Eumetazoa;Bilateria;Protostomia;Spiralia;Lophotrochozoa;Mollusca;Bivalvia;Autobranchia;Pteriomorphia;Pectinida;Pectinoidea;Pectinidae;Pecten;Pecten maximus
UdeM00000043690 7574 258.8 UdeM00000043690 XP_013382110.1 59.1 208 79 1 1 208 37 238 5.80E-66 258.8 7574 cellular organisms;Eukaryota;Opisthokonta;Metazoa;Eumetazoa;Bilateria;Protostomia;Spiralia;Lophotrochozoa;Brachiopoda;Linguliformea;Lingulata;Lingulida;Linguloidea;Lingulidae;Lingula;Lingula anatina
UdeM00000043691 7574 259.2 UdeM00000043691 XP_013382110.1 61.3 194 75 0 14 207 45 238 4.40E-66 259.2 7574 cellular organisms;Eukaryota;Opisthokonta;Metazoa;Eumetazoa;Bilateria;Protostomia;Spiralia;Lophotrochozoa;Brachiopoda;Linguliformea;Lingulata;Lingulida;Linguloidea;Lingulidae;Lingula;Lingula anatina
UdeM00000043692 7574 255.8 UdeM00000043692 XP_013382110.1 47.4 234 119 2 3 236 386 615 5.50E-65 255.8 7574 cellular organisms;Eukaryota;Opisthokonta;Metazoa;Eumetazoa;Bilateria;Protostomia;Spiralia;Lophotrochozoa;Brachiopoda;Linguliformea;Lingulata;Lingulida;Linguloidea;Lingulidae;Lingula;Lingula anatina
UdeM00000043693 7574 188.7 UdeM00000043693 XP_013384628.1 58.3 163 59 2 1 163 33 186 6.50E-45 188.7 7574 cellular organisms;Eukaryota;Opisthokonta;Metazoa;Eumetazoa;Bilateria;Protostomia;Spiralia;Lophotrochozoa;Brachiopoda;Linguliformea;Lingulata;Lingulida;Linguloidea;Lingulidae;Lingula;Lingula anatina
UdeM00000043694 7574 221.9 UdeM00000043694 XP_013399039.1 48.2 247 116 4 3 243 4 244 9.10E-55 221.9 7574 cellular organisms;Eukaryota;Opisthokonta;Metazoa;Eumetazoa;Bilateria;Protostomia;Spiralia;Lophotrochozoa;Brachiopoda;Linguliformea;Lingulata;Lingulida;Linguloidea;Lingulidae;Lingula;Lingula anatina

UdeM00000043697 6596 276.9 UdeM00000043697 XP_045182316.1 40.9 399
192 6 1 398 392 747 7.30E-71 276.9 6596 cellular
organisms;Eukaryota;Opisthokonta;Metazoa;Eumetazoa;Bilateria;Protostomia;
Spiralia;Lophotrochozoa;Mollusca;Bivalvia;Autobranchia;Heteroconchia;Euhe
terodonta;Imparidentia;Venerida;Veneroidea;Veneridae;Mercenaria;Mercenari
a mercenaria
UdeM00000043698 6596 230.7 UdeM00000043698 XP_045182313.1 38.7 475
210 18 16 442 772 1213 3.60E-57 230.7 6596 cellular
organisms;Eukaryota;Opisthokonta;Metazoa;Eumetazoa;Bilateria;Protostomia;
Spiralia;Lophotrochozoa;Mollusca;Bivalvia;Autobranchia;Heteroconchia;Euhe
terodonta;Imparidentia;Venerida;Veneroidea;Veneridae;Mercenaria;Mercenari
a mercenaria
UdeM00000033322 6526 131.7 UdeM00000033322 XP_013095404.1 49.1 163
78 4 1 158 1 163 8.20E-28 131.7 6526 cellular
organisms;Eukaryota;Opisthokonta;Metazoa;Eumetazoa;Bilateria;Protostomia;
Spiralia;Lophotrochozoa;Mollusca;Gastropoda;Heterobranchia;Euthyneura;Pan
pulmonata;Hygrophila;Lymnaeoidea;Planorbidae;Biomphalaria;Biomphalaria
glabrata
UdeM00000033323 6596 102.4 UdeM00000033323 XP_045173241.1 30.5 311
131 11 17 257 554 849 1.20E-18 102.4 6596 cellular
organisms;Eukaryota;Opisthokonta;Metazoa;Eumetazoa;Bilateria;Protostomia;
Spiralia;Lophotrochozoa;Mollusca;Bivalvia;Autobranchia;Heteroconchia;Euhe
terodonta;Imparidentia;Venerida;Veneroidea;Veneridae;Mercenaria;Mercenari
a mercenaria
UdeM00000033324 46514 120.2 UdeM00000033324 XP_038073135.1 42.6 148
78 2 1 148 393 533 2.40E-24 120.2 46514 cellular
organisms;Eukaryota;Opisthokonta;Metazoa;Eumetazoa;Bilateria;Deuterostomi
a;Echinodermata;Eleutherozoa;Asterozoa;Asteroidea;Valvatacea;Valvatida;As
terinidae;Patiria;Patiria miniata
UdeM00000033326 46514 112.1 UdeM00000033326 XP_038073135.1 52.6 95
45 0 81 175 12 106 7.30E-22 112.1 46514 cellular
organisms;Eukaryota;Opisthokonta;Metazoa;Eumetazoa;Bilateria;Deuterostomi
a;Echinodermata;Eleutherozoa;Asterozoa;Asteroidea;Valvatacea;Valvatida;As
terinidae;Patiria;Patiria miniata
UdeM00000033327 6573 181 UdeM00000033327 XP_021359687.1 31.2 433
229 12 120 533 299 681 4.00E-42 181 6573 cellular
organisms;Eukaryota;Opisthokonta;Metazoa;Eumetazoa;Bilateria;Protostomia;
Spiralia;Lophotrochozoa;Mollusca;Bivalvia;Autobranchia;Pteriomorphia;Pect
inida;Pectinoidea;Pectinidae;Mizuhopecten;Mizuhopecten yessoensis
UdeM00000033328 6596 90.9 UdeM00000033328 XP_045214422.1 35.3 184
99 8 16 185 330 507 1.90E-15 90.9 6596 cellular
organisms;Eukaryota;Opisthokonta;Metazoa;Eumetazoa;Bilateria;Protostomia;
Spiralia;Lophotrochozoa;Mollusca;Bivalvia;Autobranchia;Heteroconchia;Euhe
terodonta;Imparidentia;Venerida;Veneroidea;Veneridae;Mercenaria;Mercenari
a mercenaria
UdeM00000033329 6596 90.9 UdeM00000033329 XP_045214422.1 35.3 184
99 8 20 189 330 507 2.00E-15 90.9 6596 cellular
organisms;Eukaryota;Opisthokonta;Metazoa;Eumetazoa;Bilateria;Protostomia;
Spiralia;Lophotrochozoa;Mollusca;Bivalvia;Autobranchia;Heteroconchia;Euhe
terodonta;Imparidentia;Venerida;Veneroidea;Veneridae;Mercenaria;Mercenari
a mercenaria
UdeM00000033330 6596 117.5 UdeM00000033330 XP_045214422.1 34.3 277
147 13 1 258 247 507 2.70E-23 117.5 6596 cellular
organisms;Eukaryota;Opisthokonta;Metazoa;Eumetazoa;Bilateria;Protostomia;

Spiralia;Lophotrochozoa;Mollusca;Bivalvia;Autobranchia;Heteroconchia;Euheterodonta;Imparidentia;Venerida;Veneroidea;Veneridae;Mercenaria;Mercenaria mercenaria
UdeM00000033331 6596 92.8 UdeM00000033331 XP_045158080.1 37.6 157 93 3 45 198 676 830 5.40E-16 92.8 6596 cellular organisms;Eukaryota;Opisthokonta;Metazoa;Eumetazoa;Bilateria;Protostomia;Spiralia;Lophotrochozoa;Mollusca;Bivalvia;Autobranchia;Heteroconchia;Euheterodonta;Imparidentia;Venerida;Veneroidea;Veneridae;Mercenaria;Mercenaria mercenaria
UdeM00000033332 6596 197.2 UdeM00000033332 XP_045214422.1 35.9 390 203 16 158 535 153 507 5.30E-47 197.2 6596 cellular organisms;Eukaryota;Opisthokonta;Metazoa;Eumetazoa;Bilateria;Protostomia;Spiralia;Lophotrochozoa;Mollusca;Bivalvia;Autobranchia;Heteroconchia;Euheterodonta;Imparidentia;Venerida;Veneroidea;Veneridae;Mercenaria;Mercenaria mercenaria
UdeM00000033333 29159 88.6 UdeM00000033333 XP_034320029.1 62.9 70 25 1 1 70 418 486 3.70E-15 88.6 29159 cellular organisms;Eukaryota;Opisthokonta;Metazoa;Eumetazoa;Bilateria;Protostomia;Spiralia;Lophotrochozoa;Mollusca;Bivalvia;Autobranchia;Pteriomorphia;Ostreida;Ostreoidea;Ostreidae;Crassostrea;Crassostrea gigas
UdeM00000033334 6596 195.7 UdeM00000033334 XP_045173241.1 30.2 507 316 15 28 530 377 849 1.50E-46 195.7 6596 cellular organisms;Eukaryota;Opisthokonta;Metazoa;Eumetazoa;Bilateria;Protostomia;Spiralia;Lophotrochozoa;Mollusca;Bivalvia;Autobranchia;Heteroconchia;Euheterodonta;Imparidentia;Venerida;Veneroidea;Veneridae;Mercenaria;Mercenaria mercenaria
UdeM00000033335 6596 345.5 UdeM00000033335 XP_045177776.1 35.8 559 327 8 23 573 393 927 2.80E-91 345.5 6596 cellular organisms;Eukaryota;Opisthokonta;Metazoa;Eumetazoa;Bilateria;Protostomia;Spiralia;Lophotrochozoa;Mollusca;Bivalvia;Autobranchia;Heteroconchia;Euheterodonta;Imparidentia;Venerida;Veneroidea;Veneridae;Mercenaria;Mercenaria mercenaria
UdeM00000033337 72036 90.1 UdeM00000033337 XP_040576246.1 42.2 90 52 0 3 92 253 342 1.80E-15 90.1 72036 cellular organisms;Eukaryota;Opisthokonta;Metazoa;Eumetazoa;Bilateria;Protostomia;Ecdysozoa;Panarthropoda;Arthropoda;Mandibulata;Pancrustacea;Crustacea;Multicrustacea;Hexanauplia;Copepoda;Neocopepoda;Podoplea;Siphonostomatoida;Caligidae;Lepeophtheirus;Lepeophtheirus salmonis
UdeM00000033340 6596 205.3 UdeM00000033340 XP_045173275.1 30.9 570 335 18 4 546 328 865 2.00E-49 205.3 6596 cellular organisms;Eukaryota;Opisthokonta;Metazoa;Eumetazoa;Bilateria;Protostomia;Spiralia;Lophotrochozoa;Mollusca;Bivalvia;Autobranchia;Heteroconchia;Euheterodonta;Imparidentia;Venerida;Veneroidea;Veneridae;Mercenaria;Mercenaria mercenaria
UdeM00000033341 6596 127.9 UdeM00000033341 XP_045177648.1 47.4 135 64 2 25 152 24 158 1.20E-26 127.9 6596 cellular organisms;Eukaryota;Opisthokonta;Metazoa;Eumetazoa;Bilateria;Protostomia;Spiralia;Lophotrochozoa;Mollusca;Bivalvia;Autobranchia;Heteroconchia;Euheterodonta;Imparidentia;Venerida;Veneroidea;Veneridae;Mercenaria;Mercenaria mercenaria
UdeM00000033343 6526 108.2 UdeM00000033343 XP_013089974.1 28.9 308 196 8 98 394 69 364 2.80E-20 108.2 6526 cellular organisms;Eukaryota;Opisthokonta;Metazoa;Eumetazoa;Bilateria;Protostomia;Spiralia;Lophotrochozoa;Mollusca;Gastropoda;Heterobranchia;Euthyneura;Pan

pulmonata;Hygrophila;Lymnaeoidea;Planorbidae;Biomphalaria;Biomphalaria glabrata
UdeM00000033344 6526 109.8 UdeM00000033344 XP_013089974.1 28.9 311 198 8 98 397 69 367 9.10E-21 109.8 6526 cellular organisms;Eukaryota;Opisthokonta;Metazoa;Eumetazoa;Bilateria;Protostomia;Spiralia;Lophotrochozoa;Mollusca;Gastropoda;Heterobranchia;Euthyneura;Panpulmonata;Hygrophila;Lymnaeoidea;Planorbidae;Biomphalaria;Biomphalaria glabrata
UdeM00000033345 6596 172.2 UdeM00000033345 XP_045182203.1 54.2 144 66 0 33 176 504 647 8.00E-40 172.2 6596 cellular organisms;Eukaryota;Opisthokonta;Metazoa;Eumetazoa;Bilateria;Protostomia;Spiralia;Lophotrochozoa;Mollusca;Bivalvia;Autobranchia;Heteroconchia;Euheterodonta;Imparidentia;Venerida;Veneroidea;Veneridae;Mercenaria;Mercenaria mercenaria
UdeM00000033347 400727 348.6 UdeM00000033347 XP_025089898.1 36.3 597 340 11 184 749 251 838 2.00E-92 348.6 400727 cellular organisms;Eukaryota;Opisthokonta;Metazoa;Eumetazoa;Bilateria;Protostomia;Spiralia;Lophotrochozoa;Mollusca;Gastropoda;Caenogastropoda;Architaenioglossa;Ampullarioidea;Ampullariidae;Pomacea;Pomacea canaliculata
UdeM00000033348 400727 348.6 UdeM00000033348 XP_025089898.1 36.3 597 340 11 149 714 251 838 1.90E-92 348.6 400727 cellular organisms;Eukaryota;Opisthokonta;Metazoa;Eumetazoa;Bilateria;Protostomia;Spiralia;Lophotrochozoa;Mollusca;Gastropoda;Caenogastropoda;Architaenioglossa;Ampullarioidea;Ampullariidae;Pomacea;Pomacea canaliculata
UdeM00000033349 29159 390.6 UdeM00000033349 XP_011414121.1 45.5 420 220 3 11 429 5 416 2.60E-105 390.6 29159 cellular organisms;Eukaryota;Opisthokonta;Metazoa;Eumetazoa;Bilateria;Protostomia;Spiralia;Lophotrochozoa;Mollusca;Bivalvia;Autobranchia;Pteriomorphia;Ostreida;Ostreoidea;Ostreidae;Crassostrea;Crassostrea gigas
UdeM00000033350 6579 396.4 UdeM00000033350 XP_033738649.1 45.7 431 225 4 12 438 5 430 4.90E-107 396.4 6579 cellular organisms;Eukaryota;Opisthokonta;Metazoa;Eumetazoa;Bilateria;Protostomia;Spiralia;Lophotrochozoa;Mollusca;Bivalvia;Autobranchia;Pteriomorphia;Pectinida;Pectinoidea;Pectinidae;Pecten;Pecten maximus
UdeM00000033351 218467 153.3 UdeM00000033351 XP_023213948.1 46.4 179 95 1 4 182 1458 1635 3.30E-34 153.3 218467 cellular organisms;Eukaryota;Opisthokonta;Metazoa;Eumetazoa;Bilateria;Protostomia;Ecdysozoa;Panarthropoda;Arthropoda;Chelicerata;Arachnida;Scorpiones;Buthida;Buthoidea;Buthidae;Centruroides;Centruroides sculpturatus
UdeM00000033352 50429 70.1 UdeM00000033352 XP_022804186.1 43.1 72 41 0 1 72 430 501 2.30E-09 70.1 50429 cellular organisms;Eukaryota;Opisthokonta;Metazoa;Eumetazoa;Cnidaria;Anthozoa;Hexacorallia;Scleractinia;Astrocoeniina;Pocilloporidae;Stylophora;Stylophora pistillata
UdeM00000033354 6565 63.2 UdeM00000033354 XP_022294688.1 46 63 33 1 51 112 43 105 3.20E-07 63.2 6565 cellular organisms;Eukaryota;Opisthokonta;Metazoa;Eumetazoa;Bilateria;Protostomia;Spiralia;Lophotrochozoa;Mollusca;Bivalvia;Autobranchia;Pteriomorphia;Ostreida;Ostreoidea;Ostreidae;Crassostrea;Crassostrea virginica
UdeM00000033355 6596 95.9 UdeM00000033355 XP_045157166.1 46.9 96 50 1 22 117 31 125 3.70E-17 95.9 6596 cellular

organisms;Eukaryota;Opisthokonta;Metazoa;Eumetazoa;Bilateria;Protostomia;Spiralia;Lophotrochozoa;Mollusca;Bivalvia;Autobranchia;Heteroconchia;Euheterodonta;Imparidentia;Venerida;Veneroidea;Veneridae;Mercenaria;Mercenaria mercenaria
UdeM00000033357 6596 105.1 UdeM00000033357 XP_045157166.1 50 96 47 1 18 113 31 125 6.00E-20 105.1 6596 cellular organisms;Eukaryota;Opisthokonta;Metazoa;Eumetazoa;Bilateria;Protostomia;Spiralia;Lophotrochozoa;Mollusca;Bivalvia;Autobranchia;Heteroconchia;Euheterodonta;Imparidentia;Venerida;Veneroidea;Veneridae;Mercenaria;Mercenaria mercenaria
UdeM00000033358 6596 103.2 UdeM00000033358 XP_045157166.1 52.1 96 45 1 18 113 31 125 2.30E-19 103.2 6596 cellular organisms;Eukaryota;Opisthokonta;Metazoa;Eumetazoa;Bilateria;Protostomia;Spiralia;Lophotrochozoa;Mollusca;Bivalvia;Autobranchia;Heteroconchia;Euheterodonta;Imparidentia;Venerida;Veneroidea;Veneridae;Mercenaria;Mercenaria mercenaria
UdeM00000033359 6596 78.6 UdeM00000033359 XP_045157188.1 35.2 122 78 1 1 121 2 123 6.20E-12 78.6 6596 cellular organisms;Eukaryota;Opisthokonta;Metazoa;Eumetazoa;Bilateria;Protostomia;Spiralia;Lophotrochozoa;Mollusca;Bivalvia;Autobranchia;Heteroconchia;Euheterodonta;Imparidentia;Venerida;Veneroidea;Veneridae;Mercenaria;Mercenaria mercenaria
UdeM00000033360 6596 72.8 UdeM00000033360 XP_045157166.1 30.2 126 77 2 1 117 2 125 3.40E-10 72.8 6596 cellular organisms;Eukaryota;Opisthokonta;Metazoa;Eumetazoa;Bilateria;Protostomia;Spiralia;Lophotrochozoa;Mollusca;Bivalvia;Autobranchia;Heteroconchia;Euheterodonta;Imparidentia;Venerida;Veneroidea;Veneridae;Mercenaria;Mercenaria mercenaria
UdeM00000033361 6596 67 UdeM00000033361 XP_045157166.1 41.6 77 44 1 47 123 50 125 2.00E-08 67 6596 cellular organisms;Eukaryota;Opisthokonta;Metazoa;Eumetazoa;Bilateria;Protostomia;Spiralia;Lophotrochozoa;Mollusca;Bivalvia;Autobranchia;Heteroconchia;Euheterodonta;Imparidentia;Venerida;Veneroidea;Veneridae;Mercenaria;Mercenaria mercenaria
UdeM00000033362 6596 63.2 UdeM00000033362 XP_045157188.1 42.9 84 46 1 1 82 2 85 2.20E-07 63.2 6596 cellular organisms;Eukaryota;Opisthokonta;Metazoa;Eumetazoa;Bilateria;Protostomia;Spiralia;Lophotrochozoa;Mollusca;Bivalvia;Autobranchia;Heteroconchia;Euheterodonta;Imparidentia;Venerida;Veneroidea;Veneridae;Mercenaria;Mercenaria mercenaria
UdeM00000033363 7741 93.2 UdeM00000033363 XP_019647702.1 41.5 130 65 5 13 138 199 321 3.00E-16 93.2 7741 cellular organisms;Eukaryota;Opisthokonta;Metazoa;Eumetazoa;Bilateria;Deuterostomia;Chordata;Cephalochordata;Leptocardii;Amphioxiformes;Branchiostomidae;Branchiostoma;Branchiostoma belcheri
UdeM00000033364 6596 75.1 UdeM00000033364 XP_045157188.1 39.8 108 63 1 1 106 13 120 6.20E-11 75.1 6596 cellular organisms;Eukaryota;Opisthokonta;Metazoa;Eumetazoa;Bilateria;Protostomia;Spiralia;Lophotrochozoa;Mollusca;Bivalvia;Autobranchia;Heteroconchia;Euheterodonta;Imparidentia;Venerida;Veneroidea;Veneridae;Mercenaria;Mercenaria mercenaria
UdeM00000033365 6596 77.4 UdeM00000033365 XP_045157188.1 38.7 119 71 1 1 117 2 120 1.40E-11 77.4 6596 cellular organisms;Eukaryota;Opisthokonta;Metazoa;Eumetazoa;Bilateria;Protostomia;

Spiralia;Lophotrochozoa;Mollusca;Bivalvia;Autobranchia;Heteroconchia;Euheterodonta;Imparidentia;Venerida;Veneroidea;Veneridae;Mercenaria;Mercenaria mercenaria
UdeM00000033366 6596 62.8 UdeM00000033366 XP_045157187.1 37.8 82 51 0 67 148 43 124 4.40E-07 62.8 6596 cellular organisms;Eukaryota;Opisthokonta;Metazoa;Eumetazoa;Bilateria;Protostomia;Spiralia;Lophotrochozoa;Mollusca;Bivalvia;Autobranchia;Heteroconchia;Euheterodonta;Imparidentia;Venerida;Veneroidea;Veneridae;Mercenaria;Mercenaria mercenaria
UdeM00000033367 6596 84.3 UdeM00000033367 XP_045157166.1 42.6 122 63 4 6 121 5 125 1.20E-13 84.3 6596 cellular organisms;Eukaryota;Opisthokonta;Metazoa;Eumetazoa;Bilateria;Protostomia;Spiralia;Lophotrochozoa;Mollusca;Bivalvia;Autobranchia;Heteroconchia;Euheterodonta;Imparidentia;Venerida;Veneroidea;Veneridae;Mercenaria;Mercenaria mercenaria
UdeM00000033368 6596 69.3 UdeM00000033368 XP_045157189.1 43.6 78 44 0 37 114 45 122 3.80E-09 69.3 6596 cellular organisms;Eukaryota;Opisthokonta;Metazoa;Eumetazoa;Bilateria;Protostomia;Spiralia;Lophotrochozoa;Mollusca;Bivalvia;Autobranchia;Heteroconchia;Euheterodonta;Imparidentia;Venerida;Veneroidea;Veneridae;Mercenaria;Mercenaria mercenaria
UdeM00000033369 6579 438.3 UdeM00000033369 XP_033728856.1 79.6 270 47 2 1 268 1 264 1.10E-119 438.3 6579 cellular organisms;Eukaryota;Opisthokonta;Metazoa;Eumetazoa;Bilateria;Protostomia;Spiralia;Lophotrochozoa;Mollusca;Bivalvia;Autobranchia;Pteriomorphia;Pectinida;Pectinoidea;Pectinidae;Pecten;Pecten maximus
UdeM00000033370 6596 658.3 UdeM00000033370 XP_045177717.1 53.3 732 249 19 37 696 59 769 1.20E-185 658.3 6596 cellular organisms;Eukaryota;Opisthokonta;Metazoa;Eumetazoa;Bilateria;Protostomia;Spiralia;Lophotrochozoa;Mollusca;Bivalvia;Autobranchia;Heteroconchia;Euheterodonta;Imparidentia;Venerida;Veneroidea;Veneridae;Mercenaria;Mercenaria mercenaria
UdeM00000033371 6573 265.4 UdeM00000033371 XP_021342561.1 57.9 233 93 2 1 233 1 228 6.90E-68 265.4 6573 cellular organisms;Eukaryota;Opisthokonta;Metazoa;Eumetazoa;Bilateria;Protostomia;Spiralia;Lophotrochozoa;Mollusca;Bivalvia;Autobranchia;Pteriomorphia;Pectinida;Pectinoidea;Pectinidae;Mizuhopecten;Mizuhopecten yessoensis
[truncated: 574,143 more chars]
